# EZH1/2 plays critical roles in oocyte meiosis prophase I in mice

**DOI:** 10.1186/s40659-024-00564-4

**Published:** 2024-11-08

**Authors:** Ting Jiang, Chengxiu Zhang, Xinjing Cao, Yingpu Tian, Han Cai, Shuangbo Kong, Jinhua Lu, Haibin Wang, Zhongxian Lu

**Affiliations:** 1 School of Pharmaceutical Sciences, Fujian Provincial Key Laboratory of Innovative Drug Target Research, Xiamen University, Xiamen, Fujian 361005 China; 2 Fujian Provincial Key Laboratory of Reproductive Health Research, Department of Obstetrics and Gynecology, School of Medicine, The First Affiliated Hospital of Xiamen University, Xiamen University, Xiamen, Fujian 361102 China; 3 School of Pharmaceutical Sciences, Xiamen University, Zhuangjin Hall, Room 363, Xiamen, Fujian, 361102 China; 4 Medical College of Xiamen University, Xiamen, Fujian 361102 China

**Keywords:** EZH1, EZH2, Oocyte meiosis, DSBs repair, Embryonic ovary

## Abstract

**Backgroud:**

abnormalities or defects in oocyte meiosis can result in decreased oocyte quality, reduced ovarian reserve, and female diseases. However, the mechanisms of oocyte meiosis remain largely unknown, especially epigenetic regulation. Here, we explored the role of EZH1/2 (histone methyltransferase of H3K27) in mouse oocyte meiosis by inhibiting its activity and deleting its gene.

**Results:**

with embryonic ovary cultured in vitro, EZH1/2 was demonstrated to be essential for oocyte development during meiosis prophase I in mice. Activity inhibition or gene knockout of EZH1/2 resulted in cell apoptosis and a reduction in oocyte numbers within embryonic ovaries. By observing the expression of some meiotic marker protein (γ-H2AX, diplotene stage marker MSY2 and synapsis complex protein SCP1), we found that function deficiency of EZH1/2 resulted in failure of DNA double-strand breaks (DSBs) repair and break of meiotic progression in fetal mouse ovaries. Moreover, *Ezh1/2* deficiency led to the suppression of ATM (Ataxia Telangiectasia Mutated kinase) phosphorylation and a decrease in the expression of key DNA repair proteins Hormad1, Mre11, Rad50, and Nbs1 in fetal mouse ovaries, underscoring the enzyme’s pivotal role in initiating DNA repair. RNA-seq analysis revealed that *Ezh1/2*-deletion induced abnormal expression of multiple genes involved into several function of oocyte development in embryonic ovaries. Knockout of *Ezh1/2* in ovaries also affected the levels of H3K9me3 and H4K20me2, as well as the expression of their target genes *L3mbtl4* and *Fbxo44*.

**Conclusions:**

our study demonstrated that EZH1/2 plays a role in the DSBs repair in oocyte meiosis prophase I *via* multiple mechanisms and offers new insights into the physiological regulatory role of histone modification in fetal oocyte guardianship and female fertility.

**Supplementary Information:**

The online version contains supplementary material available at 10.1186/s40659-024-00564-4.

## Background

In female mammals, the development of germ cells begins with the emergence of primordial germ cells (PGCs), which undergo extensive proliferation, germline cyst formation, and arrest in meiotic prophase I [1–4]. In fetal mouse ovaries, PGCs enter the meiotic phase at 13.5 days post coitum (dpc), differentiating into oocytes. These oocytes sequentially progress through the leptotene, zygotene, and pachytene stages before becoming arrested in the diplotene (dictyate) stage [5–7]. During meiotic prophase I, key biological events such as DNA double-strand breaks (DSBs), their repair, and the synapsis of homologous chromosomes occur. The successful progression of these events is vital for healthy oogenesis; disruptions can lead to a reduced oocyte count or quality, potentially causing infertility and female reproductive disorders [8]. Therefore, studying the regulatory mechanisms of meiotic prophase I in early embryonic ovarian is imperative for advancements in reproductive disease.

Recently, epigenetic regulation, particularly histone modification, has garnered significant attention in oocyte meiosis mechanisms [9, 10]. Some reports showed that histone modification play crucial roles in initiation of meiosis, DNA double-strand breaks and repair, association and arrangement of chromosomes [11, 12]. Prdm9-mediated H3K4me3 at DSB hotspots, along with H3K27ac and H3K36me3, is closely associated with DSB fate [13]. Maternal deletion of *Sall4* leads to abnormal H3K4me3 and H3K27me3 levels, inhibiting oocyte meiotic resumption in mice [14]. Similarly, a mutation in KMT5A, an H4K20 methyltransferase, results in incomplete DSB repair and embryonic lethality [11, 15]. Despite these insights, our understanding of epigenetic regulation in oocyte meiosis remains incomplete, necessitating further research.

EZH1 and EZH2, core components of the PRC2 complex within the PcG family, function as histone methyltransferases for H3K27. Their activity is implicated in cancers, developmental disorders, and reproductive development [16]. EZH1/2 has been shown to influence gamete development, maternal endometrial changes, embryonic development, and embryonic stem cells [17–20]. Our previous work indicated that the knockout of *Ezh1* and *Ezh2* leads to H3K27me3 abnormalities and impairs pluripotent ectodermal cells in late blastocysts, affecting subsequent embryonic development [19]. The PRC2 complex also plays a pivotal role in the DSB repair pathway; for example, the PARP-dependent recruitment of EZH2 to DSBs facilitates H3K27 trimethylation post-DNA damage [21]. EZH2 interacts with BubR1 and stabilizes spindle assembly checkpoint proteins, which subsequently maintain meiosis anaphase maturation in mouse oocytes [22]. However, the specific function of EZH1/2 in meiotic prophase I of oocytes requires further investigation.

This study primarily aimed to clarify the essential role of EZH1/2 in the progression of meiotic prophase I in the oocytes of the fetal mouse ovary. We cultured mouse embryonic ovaries at 14.5 dpc for 3 to 4 days and mimic this process in vitro. This process facilitates a deeper understanding of meiotic regulation and may provide new insights on the treatment of female infertility.

## Materials and methods

### Animal strains and cell line

C57BL/6 mice (7–8 weeks) were purchased from Xiamen University Laboratory Animal Center. *Ezh1*^*−/−*^ mice were obtained from Thomas Jenuwein’s laboratory, while *Ezh2*^*flox/flox*^ mice were generously provided by Professor Alexander Tarakhovsky of Rockefeller University, New York, USA. The mice were housed at 21–25 °C with a 12-hour light/dark cycle in the Laboratory Animal Center of Xiamen University. All experiments were conducted according to the guidelines of the Animal Care Committee of the Xiamen University (Approval NO. XMU20220140). Mouse genotypes were determined from tail samples, with relevant primers detailed in Supplementary Table S1. 293T cells were grown in DMEM (#SH30081.01, Hyclone, Utah, USA) supplemented with 10% FBS (Hyclone) at 37 °C in 5% CO_2_ using 100 mm culture dish.

## Ovary Culture and inhibitor treatment

Mating of male and female mice was conducted under standard conditions, with vaginal plugs examined the following morning (designated as 0.5 days post-coitum, dpc). Embryos at 14.5dpc were collected for females killed by cervical dislocation. Mouse embryonic ovaries were isolated under a stereoscopic microscope (SZX10, Olympus, Japan) using experiment-specific scissors and forceps, and then immediately placed in sterile pre-cooled PBS (10mM, pH7.4). The isolated mouse ovaries were placed into DMEM/F12 medium (#21331020, Gibco, NY, USA) with pre-balanced in 6-well plates and incubated at 37 °C in an incubator with 95% humidity and 5%CO2. To mimic oocyte meiosis prophase I, we cultured mouse embryonic ovary at 14.5 dpc for 3 ~ 4 days in vitro.

For inhibitors and treatment, ovaries were incubated in DMEM/F12 medium with 1µM UNC1999, GSK343 or RSL3 (HY-15646; HY-13500; HY-100218 A; MCE, Shanghai, China) or vehicle control (DMSO) for a defined period. During the culture, the inhibitor-containing or DMSO medium was changed in half volume every 24 h for 3 days.

## Histological sections and immunofluorescence (IF)

Mouse embryonic ovaries were washed with PBS (pH7.4), fixed in 4% paraformaldehyde (#P6148-500G, Sigma–Aldrich, Missouri, USA) for 48 h at 4 °C, dehydrated through a graded alcohol series and xylene, and embedded in paraffin. Tissue sections of 0.3 μm thickness were prepared using a paraffin microtome (Zeiss RM2255, Oberkochen, Germany). After dewaxing and rehydration, tissues were subjected to antigen retrieval in citrate buffer via high-pressure treatment for 15 min. Sections were blocked in 5%BSA/PBS for 1 h and incubator in the primary antibodies diluted in 5%BSA/PBS overnight at 4 °C. Following this, fluorescent secondary antibodies were applied at a 1:200 dilution in 5% BSA/PBS and incubated for 1 h at room temperature. After washing 3 times in PBS for 5 min each, slides were mounted with DAPI-containing antifade mounting medium (Vector H-1200, CA, USA) and examined using a confocal laser scanning microscope (ZEISS LSM880, Oberkochen, Germany).

Mouse ovaries were continuously sliced, and the slice with the largest section was selected for IF staining. The number of images assessed in a single experiment was at least 3, and the experiment was repeated for 3 times. We conducted a quantitative analysis of the fluorescence images utilizing ImageJ to determine the Integrated Density (IntDen) for each image, with the final data representing the average from three replicate experiments. Antibodies information was listed in Supplementary Table S2.

## TUNEL assays

Apoptosis was assessed using the TUNEL FITC apoptosis detection kit (# A111, Vazyme, Jiangsu, China). Ovarian tissue sections were dewaxed and hydrated before incubation with 2 mg/ml proteinase K solution for 20 min. Equilibration Buffer was added and incubated at room temperature for 10–30 min. After equilibration, the buffer was removed, and the TdT (terminal deoxynucleotidyl transferase) reaction mixture (include ddH_2_O, 5×Equilibration Buffer, FITC-12-dUTP Labeling Mix, Recombinant TdT Enzyme) was applied. Slides were then stained with DAPI-containing antifade mounting medium and examined using confocal microscopy.

## Chromosome spreading

After isolation and culture treatment, ovaries were digested with 0.25% trypsin (#T1300, Solarbio, Beijing, China) for 5 min, and centrifuged after terminating reaction with FBS (#10099-141, Gibco, Carlsbad, CA, USA). Oocytes were resuspended in 1%PFA, washed with 1×PBS for 5 min, blocked in 5%BSA/PBS for 1 h, and incubated with Primary antibodies overnight at 4 °C. Secondary antibodies were applied at a 1:100 dilution for 1 h at room temperature. The slides were sealed after adding the DAPI solution. Photos were taken with confocal microscopy.

ImageJ (MD, USA) was employed for the quantitative analysis of γ-H2AX foci. Initially, the image channels were separated, and an optimal threshold value was selected to ensure that each nucleus is represented by a distinct highlighted region; we established a size discrimination criterion of > 50 px to exclude smaller artifacts that do not correspond to nuclei. Ultimately, a total count is generated, representing the overall number of foci. Additionally, we assessed the overlap between γ-H2AX foci and oocyte chromosomes (SCP3, green), which constituted our result. All experiments were conducted under consistent background conditions, potentially in the presence of impurities. Antibodies information was listed in Supplementary Table S2.

### RNA purification and real-time qPCR

The embryonic ovaries were lysed by TRIzol (#15596-026, Invitrogen, CA, USA), and total RNA was extracted. The cDNA was synthesized according to the Reverse transcription and premix type kit (#AG11728, Accurate biology, Hunan, China). RT-qPCR were performed using AriaMx real-time qPCR instrument (Agilent, CA, USA) with SYBR green premix (#AG11718, Accurate biology, Hunan, China). Transcript levels were normalized to GAPDH expression using the 2-∆∆Ct method. The relative amount of target gene expression for each sample was calculated and plotted as the mean ± s.d. All primers were listed in Supplementary Table S3.

## Protein extraction and western blotting

Ovaries were collected and lysed using cell lysis buffer (#P0013, Beyotime, Shanghai, China) containing PMSF (#ST506, Beyotime, Shanghai, China). Protein lysate was mixed with 5×SDS loading buffer (#P0283, Beyotime, Shanghai, China), boiled at 100 °C for 5 min, and stored at -20 °C. Western blots were performed as previously described [23]. Proteins were separated on SDS-PAGE gels and transferred to PVDF membranes. Then, membrane was blocked in 5% skimmed milk, and incubated with primary antibodies overnight at 4 °C. After 1 h incubation with secondary antibodies at room temperature, proteins were detected using the ChemiDoc system (Bio-Rad, CA, USA) with Super ECL (#A38556, Thermo Fisher Scientific, CA, USA). Antibodies information was listed in Supplementary Table S2.

## Plasmid construction and ovarian injection

*Ezh1*^*−/−*^ and *Ezh2* double knockout (dKO) in oocytes in embryonic ovaries was achieved by transfecting plasmids expressing Cre recombinase into cells with loxP sites. The pLV-mAlpl-promoter-NLS-EGFP-Cre plasmid was constructed under the control of the Alpl promoter and purchased from Youbao Company (Hunan, China). Lentivirus was produced by co-transfecting 293T cells with pLV-mAlpl-promoter-NLS-EGFP-Cre, pMD2.G, and psPAX2. Lentiviral particles were harvested and concentrated after 48 h. Embryonic ovaries from *Ezh1*^*−/−*^, *Ezh2*^*flox/flox*^ mice were infected with lentivirus mixed with Trypan blue (#C0011, Beyotime Biotechnology, Shanghai, China) and cultured in vitro.

### RNA-seq analysis

Total RNA was extracted from 60 embryonic ovaries in each group, and total RNA was treated with the mRNA enrichment method. Library preparation is performed using Optimal Dual-mode mRNA Library Prep Kit (BGI-Shenzhen, China). Then First-strand cDNA is generated using random hexamer-primed reverse transcription, followed by a second-strand cDNA synthesis. After cDNA end repairment, a single ‘A’ nucleotide is added to the 3’ ends of the blunt fragments through A tailing reaction. Finally, the library products are amplified through PCR reaction and subjected to quality control. Finally, PE100/PE150 sequencing was carried out on a G400/T7/T10 platform (BGI-Shenzhen, China) by Combinatorial Probe-Anchor Synthesis (cPAS) technology. Gene expression data were analyzed by using DAVID (the Database for Annotation, Visualization, and Integrated Discovery, [https://david.ncifcrf.gov]). Differentially expressed genes (DEGs) were identified based on |log2(fold change)| > 1 and a p-adjusted value (padj) < 0.05.

### Statistical analysis

Data were analyzed using GraphPad Prism 6. Data are presented as mean ± S.D. Each experiment was repeated at least three times. Statistical comparisons between two groups were performed using two-tailed unpaired t-tests. Significance is indicated as follows: *P < 0.05; **P < 0.01.

## Results

### EZH1/2 functional deletion induces cell apoptosis and oocyte loss in fetal mouse ovaries

**Fig. 1:**
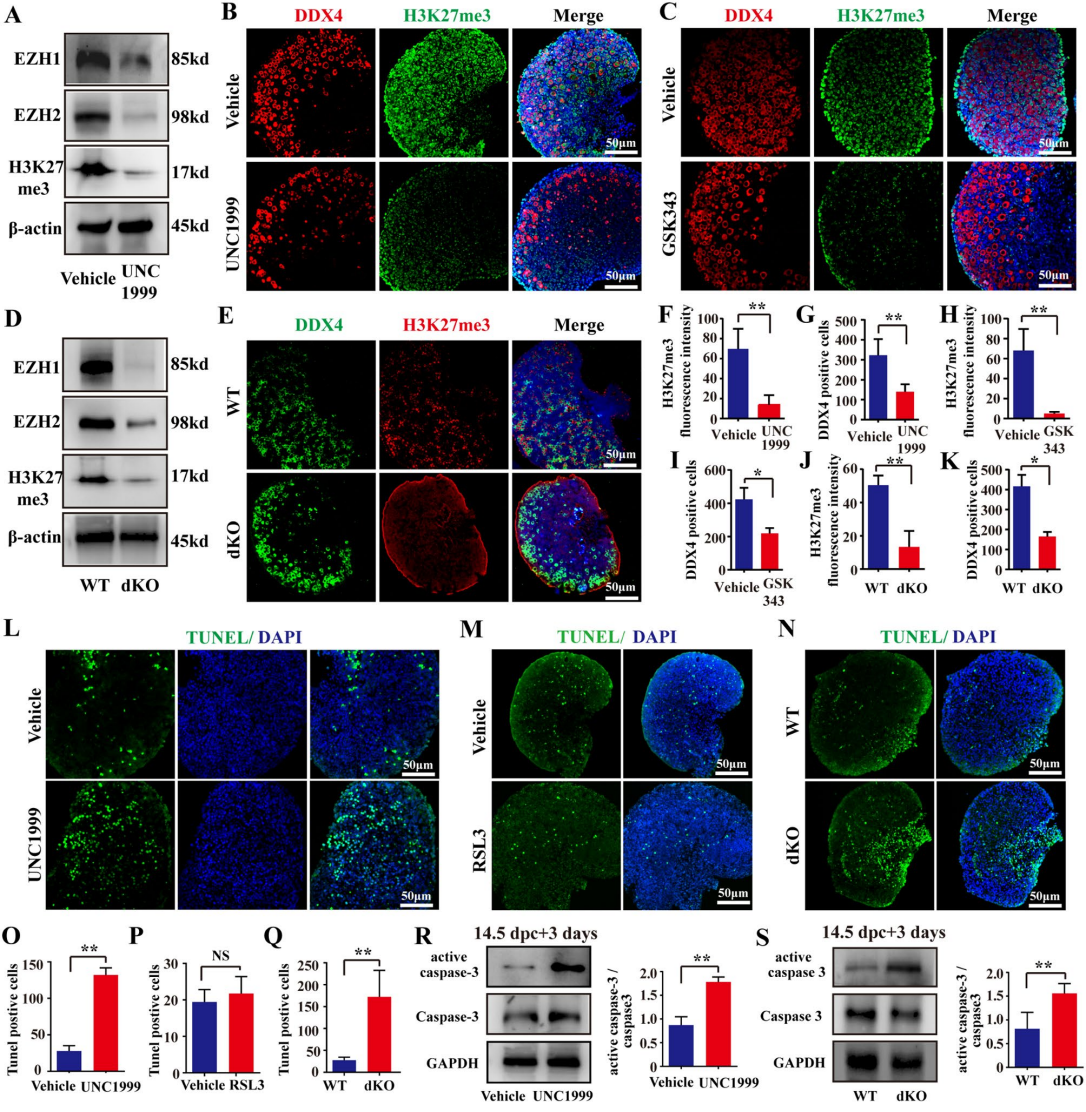
Inhibition of enzyme activation or gene knockout of *Ezh1/2*impairs the oocyte development in fetal mouse ovaries. (**A**) Protein levels of EZH1, EZH2, and H3K27me3 in whole fetal ovaries cultured with Ezh1/2 inhibitor UNC1999 checked by western blotting (WB). DMSO is a negative control of the inhibitor treatment (vehicle group). β-actin was used as an internal control. (**B**, **C**) Representative images of immunofluorescence (IF) staining of DDX4 and H3K27me3 in fetal ovaries treated with EZH1/2 inhibitor UNC1999 or GSK343 (green color). DDX4 (red color) is an oocyte marker. The nucleus was stained with DAPI (blue). Scale bar:50 μm; (**D**) Protein levels of EZH1, EZH2, and H3K27me3 in wildtype (WT) or *Ezh1/2* double knockout (dKO) embryonic ovaries checked by WB. β-actin was used as an internal control. (**E**) The levels of H3K27me3 and DDX4 stained by IF in fetal mouse ovaries after knockout *Ezh1/2*. The red color indicates H3K27me3 and the green color is DDX4. The nucleus was stained with DAPI (blue). Scale bar: 50 μm; (**F-K**) Quantification of H3K27me3 fluorescence intensity or DDX4 positive cells in B, C, and E. (**L-N**) TUNEL assays on fetal ovaries with treatment of EZH1/2 inhibitors or dKO ovaries. Green is a positive color. The nucleus was stained with DAPI (blue). Scale bar: 50 μm; (**O-R**) Quantitative analysis of TUNEL positive cells in L-N. (**R**, **S**) The protein level of active caspase3 and caspase3 checked by WB in fetal ovaries treated with UNC1999 or deleted *Ezh1/2* gene. GAPDH was used as an internal control. Vehicle: an equivalent volume of DMSO. WT: wild type; dKO: double knockout of both *Ezh1* and *Ezh2* in germ cells. Each group had 20 ovaries. The number of images assessed in a single experiment was at least 3.The experiments were repeated at least 3 times (*N* = 3). The data were presented as mean ± S.D. The asterisk (*) denotes a statistically significant difference between the vehicle (or WT) and treatment groups. NS: no significant, *P < 0.05, **P < 0.01 (*t*-test)

To explore the function of EZH1/2 in oocyte meiosis prophase I, we cultured mouse embryonic ovaries at 14.5 post-coitum (dpc) for 3 ~ 4 days to mimic this process in vitro. Initially, EZH1/2 function in embryonic ovaries was suppressed by EZH1/2 activity inhibitors. UNC1999 inhibits EZH2 and EZH1 histone-lysine N-methyltransferase activity through competitive inhibition of the cofactor S-Adenosyl-l-methionine (SAM). After treatment of UNC1999 for 3 days, H3K27me3 level in whole fetal ovaries was remarkably decreased compared to the vehicle control group (treated by an equivalent volume of DMSO) (Fig. 1A). The fluorescence intensity of H3K27me3 also showed a marked decrease in UNC1999 group **(**Fig. 1B, F**)**. Similarly, EZH1 and EZH2 protein levels were reduced after UNC1999 treatment (Fig. 1A). These results indicate that EZH1/2 function was effectively suppressed by inhibitor UNC1999. DDX4 is a marker of germ cells and the number of DDX4-positive cells was clearly reduced in embryonic ovary after UNC1999 treatment (Fig. 1B, G). GSK343 is a highly potent and selective EZH2 inhibitor with an IC50 of 4 nM. GSK343 also suppressed the H3K27me3 and reduced the DDX4-positive cells in embryonic ovaries cultured for 3 days in vitro (Fig. 1C, H, I**)**. These observations suggest that the inhibition of EZH1/2 leads to germ cell loss in embryonic ovary during meiosis prophase I.

Secondly, the *Ezh1/2* gene was specifically deleted in oocytes at the initiation of meiosis using a pLV-mAlpl-promoter-NLS-EGFP-Cre. The embryonic ovary at 14.5 dpc isolated from wildtype or *Ezh1*^*−/−*^, *Ezh2*^*flox/flox*^ mice was infected with lentivirus expressed pLV-mAlpl-promoter-NLS-EGFP-Cre and cultured for 3 days. The protein levels of H3K27me3, EZH1 and EZH2 was significantly reduced in the double knockout (dKO) ovaries (Fig. 1D, E, J). The number of DDX4-positive cells was also significantly reduced in dKO ovaries (Fig. 1K), consistent with the observations following inhibitor treatment.

Then, cell apoptosis in fetal mouse ovaries was assessed using TUNEL assay. After treatment with UNC1999, cell apoptosis (TUNEL-positive cells) was increased in embryonic ovaries (Fig. 1L, O). But, the negative control chemical RSL3 (an inhibitor of glutathione peroxidase 4 (GPX4)) did not enhance cell apoptosis (Fig. 1M, P). The number of TUNEL-positive cells was also raised in dKO ovaries (Fig. 1N, Q). Active caspase 3 levels, a marker of apoptosis, were elevated in both UNC1999-treated and dKO ovaries (Fig. 1R, S).

These results demonstrate that EZH1/2inhibition or gene knockout enhances cell apoptosis and leads to loss of oocytes in embryonic ovaries during oocyte meiosis prophase I, suggesting that EZH1/2 plays an important role in this developmental stage of the embryonic mouse ovary.

### EZH1/2-deficiency impedes meiotic progression in fetal mouse ovaries

**Fig. 2:**
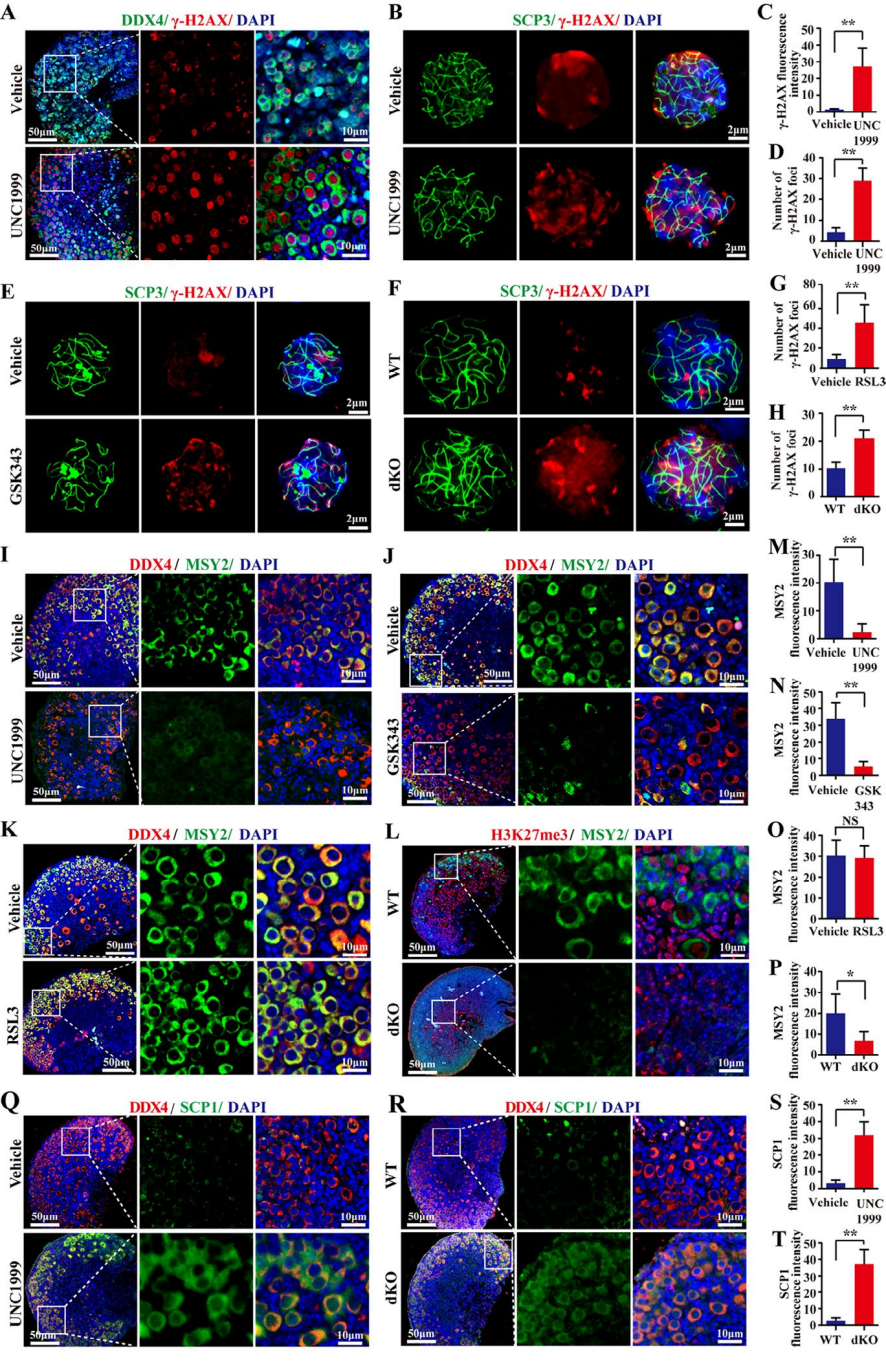
Defective *Ezh1/2* function leads to failure of DNA break repair and disrupts oocyte meiosis progression in embryonic mouse ovaries. (**A**) Representative images of γ-H2AX with IF staining in fetal mouse ovaries treated with EZH1/2 inhibitor UNC1999. Red is a positive color. DDX4 is green. The nucleus was stained with DAPI (blue). Scale bar: 50 μm, enlarged scale bar: 10 μm; (**C**) Quantitative analysis of γ-H2AX fluorescence intensity (**A**). (**B**,** E**, **F**) Representative images γ-H2AX (red), SCP3 (red), and DAPI (blue) by IF staining in pachytene stage oocytes with chromosome spread. SCP3, the major cytoskeletal protein of the synapsis complex, is continuously expressed throughout premeiotic phase I and is a chromosomal linkage site. Here SCP3 staining is present in the displayed chromosome. Oocytes were isolated from fetal ovaries treated with EZH1/2 inhibitors (UNC1999 (**B**) and GSK343(**E**)) or dKO ovaries (**F**). Scale bar:2 μm; (**D**, **G**,** H**) Quantitative analysis of γ-H2AX foci in B, E, F. (**I-L**) Representative images of IF staining of MSY2 (a marker of diplotene phase of meiosis) in fetal ovaries with the treatment of EZH1/2 inhibitor (UNC1999 (**I**) or GSK343 (**J**)), negative control chemical RLS3 (**K**), and lentivirus (dKO, **L**). Green is a positive color. DDX4 staining indicated oocytes. H3K27me3 was stained as red color. The nucleus was stained with DAPI (blue). Scale bar: 50 μm, enlarged scale bar: 10 μm; (**M-P**) Quantitative analysis of MSY2 fluorescence intensity in I-L. (**Q**,** R**) Representative images of SCP1 IF staining in fetal ovaries treated with UNC1999 or dKO ovaries. SCP1 was stained as green color. DDX4 is red. The nucleus was stained with DAPI (blue). Scale bar: 50 μm, enlarged scale bar:10 μm; (**S**,** T**) Quantitative analysis of SCP1 fluorescence intensity (**Q** and **R**). The number of images assessed in a single experiment was at least 3. The experiments were repeated at least 3 times (*N* = 3). The data were presented as mean ± S.D. *P < 0.05, **P < 0.01 (*t*-test)

We further investigated the impact of EZH1/2 on meiotic prophase I using meiosis-specific markers. Phosphorylated histone H2AX (γ-H2AX) marks double-strand breaks (DSBs) and is present from the leptotene to pachytene stages [24, 25]. Here, the γ-H2AX signal weaken in fetal ovary cultured with normal medium for 3 days (equaling 17.5 dpc), but remained strong in UNC1999-treated ovaries (Fig. 2A, C). To exactly observe the γ-H2AX in chromosome, nuclear spreading of fetal oocytes was performed. Results showed that few γ-H2AX foci in the vehicle group, but a substantial number of foci in oocytes from fetal ovary with UNC1999 or GSK343-treatment (Fig. 2B, E). In dKO ovaries, γ-H2AX foci also remained at a high level compared to wildtype ovaries (Fig. 2F). Statistical analysis showed that the number of γ-H2AX foci in oocytes at the late pachytene stage was maintained in fetal ovary with UNC1999 or GSK343-treatment or dKO ovaries (Fig. 2D, G, H). These results suggested that DNA damage repair was not completed in *Ezh1/2*-deficient oocyte.

Abnormal DNA damage repair may interfere the meiotic prophase progression. Therefore, the effect of the *Ezh1/2*-deficient on meiotic prophase progression in oocytes was further explored. MSY2 is a marker of the diplotene stage [26]. When mouse fetal ovaries at 15.5 dpc were cultured for 3 days (equaling 18.5 dpc), MSY2 was expressed in oocytes from vehicle ovaries. However, MSY2 protein was largely absent in oocytes in ovaries with UNC1999 or GSK343 treatment (Fig. 2I, J). Statistical analysis confirmed that the MSY2 fluorescence intensity was significantly reduced in the UNC1999 or GSK343 ovaries (Fig. 2M, N). However, the negative control chemical RSL3 did not affect the expression of MSY2 in embryonic ovaries (Fig. 2K, O). After ovaries (15.5 dpc) were infected with lentivirus for 3 days in vitro, few oocytes in the dKO ovaries expressed MSY2 (Fig. 2L, P). These results imply that oocytes with *Ezh1/2* deficiency are impeded in reaching the diplotene stage.

SCP1 is the major component of the synaptonemal complex (SC) transverse filament and disappears at the diplotene stage [27]. Here, we performed SCP1 immunofluorescence analysis in fetal mouse ovaries and found that SCP1 was lost in oocytes in vehicle or WT ovaries when 15.5 dpc ovaries were cultured for 3 days in vitro, while SCP1 expression persisted in the UNC1999-treated or dKO ovaries (Fig. 2I, J, K, L). The observation of SCP1 confirmed that oocytes did not enter the diplotene stage when EZH1/2 function was suppressed.

The above results show that most oocytes in EZH1/2-deficient ovaries fail to repair DNA damage, and thereby do not enter the diplotene stage, suggesting that EZH1/2 is required to ensure the normal process of meiotic prophase I in fetal oocytes.

### *Ezh1/2* deficiency causes abnormal expression of genes associated with DSBs repair initiation during meiosis prophase I in embryonic ovaries

**Fig. 3:**
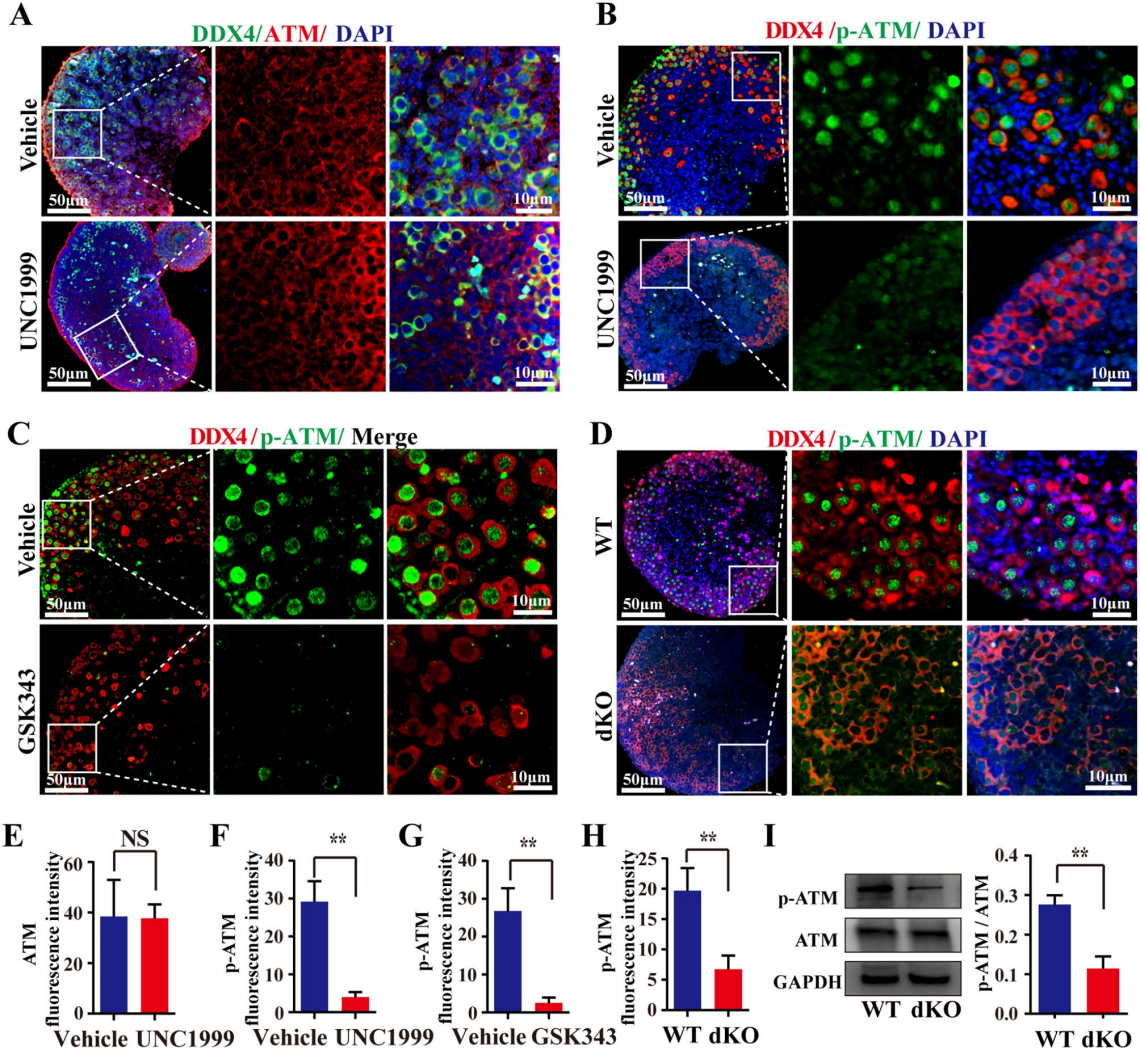
*Ezh1/2* function deficiency suppresses the phosphorylation of ATM in mouse fetal mice. **(A)** Representative images of IF staining of ATM (red) and DDX4 (green) in fetal ovaries treated with EZH1/2 inhibitor (UNC1999). The nucleus was stained with DAPI (blue). Scale bar: 50 μm, enlarged scale bar: 10 μm; **(B-D)** The level of p-ATM in fetal ovaries treated with UNC1999 **(B)**, GSK343**(C)**, and lentivirus (D, dKO). Green is the positive color for p-ATM. DDX4 is stained red. The nucleus was stained with DAPI (blue). Scale bar: 50 μm, enlarged scale bar: 10 μm; **(E)** Quantitative analysis of ATM fluorescence intensity in A. **(F-H)** Quantitative analysis of p-ATM fluorescence intensity in B-D. **(I)** The level of p-ATM in WT or dKO ovaries checked by WB. GAPDH was used as an internal control. The ratio of p-ATM to ATM protein is shown in the right figure. Every group had 20 ovaries. The number of images assessed in a single experiment was at least 3. The experiments were repeated at least 3 times (*N* = 3). The data were presented as mean ± S.D. *P < 0.05, **P < 0.01 (*t*-test)

**Fig. 4:**
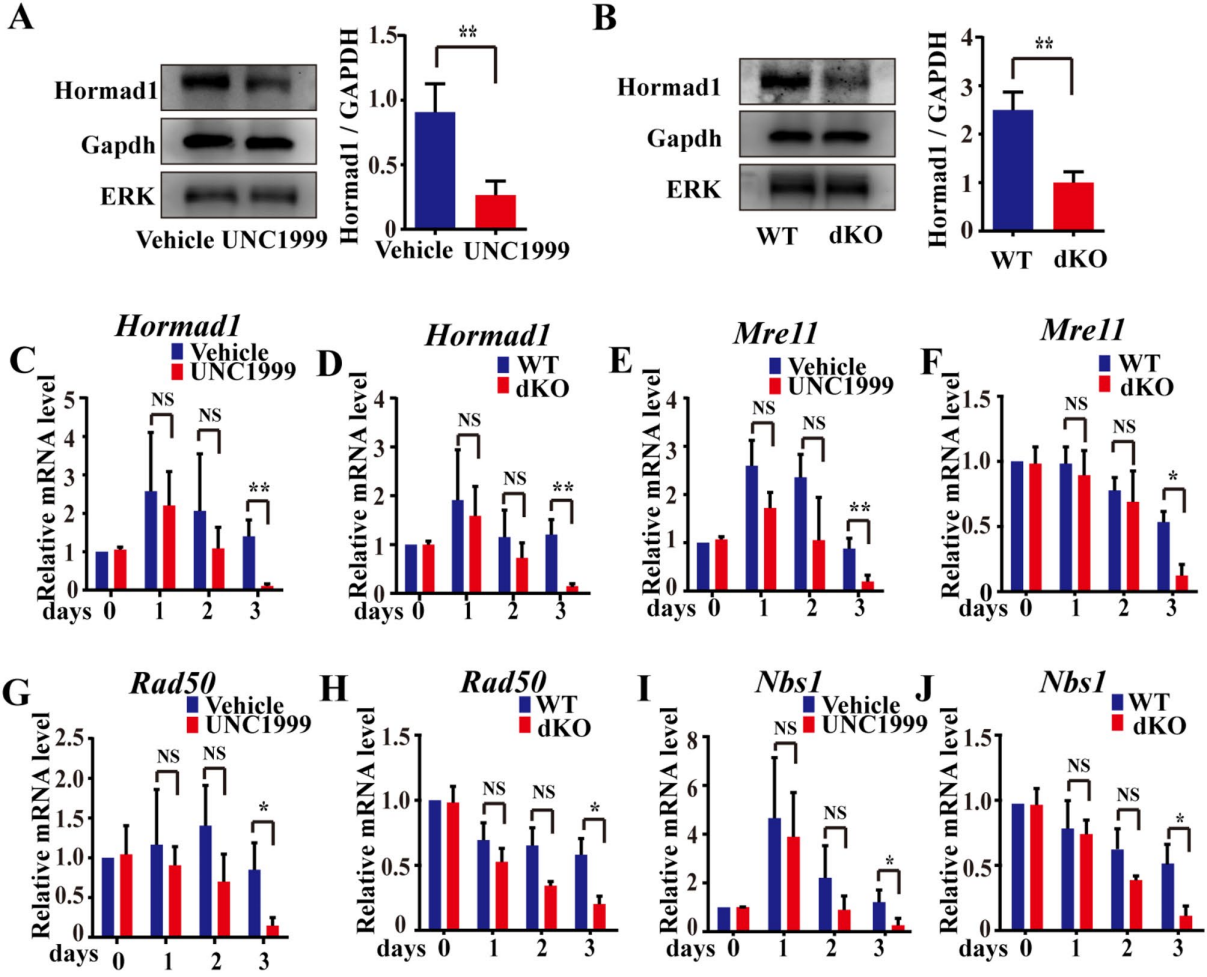
*Ezh1/2* function deficiency results in abnormal expression of genes involved in the initiation of DSBs. **(A-B)** The protein level of hormad1 in fetal ovaries treated with UNC1999 or lentivirus. GAPDH and ERK were used as an internal control. Quantitative analysis of Hormad1 protein level normalized by GAPDH protein. Every group had 20 ovaries. **(C-J)** The relative expression levels of *Hormad1*, *Mre11*, *Rad50*, and *Nbs1* mRNA in fetal ovaries treated with UNC1999 or lentivirus (dKO) by RT-qPCR. *Gapdh* is a reference gene. The data were normalized with that WT at 14.5 dpc + 0 days. The experiments were repeated at least 3 times (*N* = 3). Data were presented as mean ± S.D. The asterisk (*) denotes a statistically significant difference between the vehicle (or WT) and treatment groups. *P < 0.05, **P < 0.01 (*t*-test)

To discover the mechanism by which Ezh*1/2* deficiency impairs DSB repair, we assessed the function of proteins associated initiation of DSBs repair in meiotic prophase I. Ataxia-telangiectasia mutated proteins (ATM) is a serine/threonine protein kinase and play a key role in the initiation of DSBs repair. Immunofluorescence assays were performed to assess the levels of ATM and phospho-ATM (Ser1981) in mouse embryonic ovaries treated with UNC1999 for 3 days in vitro. The fluorescence intensity of ATM did not significantly change after the UNC1999 treatment (Fig. 3A, E). However, the fluorescence intensity of p-ATM was significantly decreased after treatment with UNC1999 or GSK343 (Fig. 3B, C, F, G). In dKO ovaries, the fluorescence intensity of p-ATM was also remarkably reduced (Fig. 3D, H). Meanwhile, p-ATM protein levels were dropped in dKO ovaries (Fig. 3I), and the ratio of p-ATM to ATM was clearly reduced (Fig. 3I). Hormad1, a meiosis-specific protein that promotes synapsis and recombination of homologous chromosomes in meiotic prophase, may affect the downstream phospho-ATM [28–31]. Western blotting and RT-qPCR analysis results showed that the protein and mRNA of Hormad1 was decreased in the UNC1999-treated or dKO ovaries (Fig. 4A, B, C, D).

ATM protein kinase is activated by DSBs through the Mre11-Rad50-Nbs1 (MRN) DNA repair complex and orchestrates signaling cascades that initiate the DSBs repair mechanism in meiosis [32]. When 14.5 dpc ovaries were cultured with UNC1999 or lentivirus for 1 or 2 days, the relative expression levels of *Mre11*, *Rad50*, and *Nbs1* were not significantly altered (Fig. 4E, F, G, H, I, J, *P* > 0.05). However, after 3 days of culture in vitro, the expression of *Mre11*, *Rad50*, and *Nbs1* was significantly decreased in UNC1999-treated or dKO ovaries.

These results suggest that EZH1/2 governs the initiation of DSBs repair by ATM-MRN complex.

### Abnormal gene expression in fetal mouse ovaries following *Ezh1/2* Depletion

**Fig. 5:**
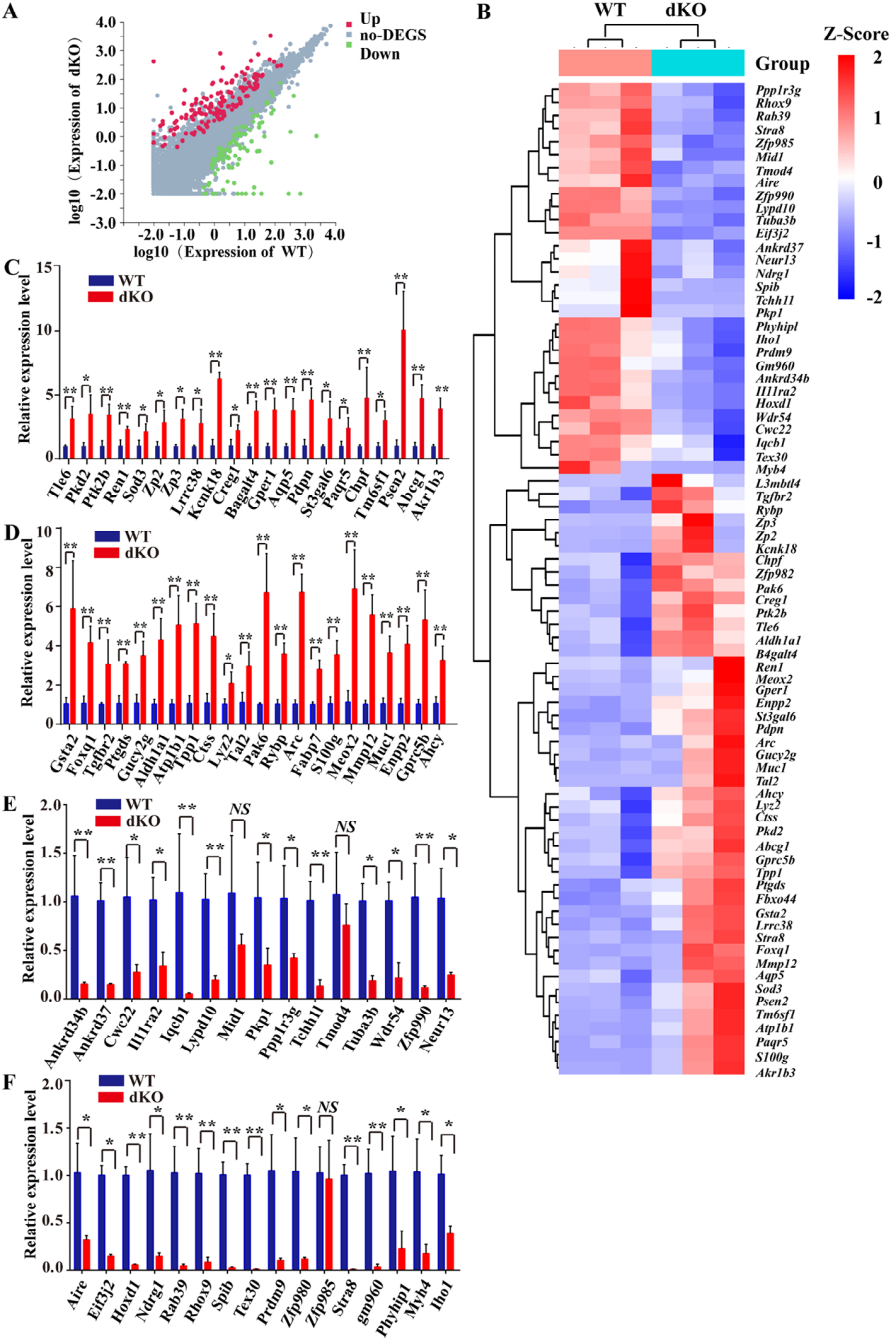
Knockout of *Ezh1/2* interferes with the expression of many functional genes in fetal mouse ovaries. Gene transcription in embryo ovaries were sequenced on an Illumina HiSeq 2500 with 125 bp single end sequencing and gene expression data were analyzed by using DAVID (the Database for Annotation, Visualization, and Integrated Discovery, [https://david.ncifcrf.gov]). **(A)** Volcano plot of differentially expressed genes in WT and dKO ovary tissues on 14.5dpc + 3 days as determined by RNA-seq. Blue or red dots represent genes that are differentially expressed (|log2FC|>=1, Qvalue < = 0.05) between WT and dKO group. Red dots indicate upregulated genes, and green dots indicate down-regulated genes. Genes not significantly differentially expression (no DEGs) are shown in gray. The FC in log2FC is fold change, which represents the ratio of the expression between the two samples (groups). Log10 (Expression of WT/dKO): the normalized expression value is taken as the log, usually using log10. **(B)** Heatmap of physiological function associated genes differentially regulated between WT and dKO ovary tissues on 14.5dpc + 3 days. Heatmap of the clustering of the different expressed genes. Green indicates lower expression, red indicates higher. FPKM of each gene from different samples was normalized to Z-score. **(C-F)** The relative expression level of genes in fetal ovaries treated with lentivirus by RT-qPCR. *Gapdh* is a reference gene. The values in each group were averaged and normalized with that of WT group. The experiments were repeated at least 3 times (*N* = 3). Data were presented as the mean ± S.D; The asterisk (*) denotes a statistically significant difference between the WT and dKO groups. *P < 0.05, **P < 0.01 (*t*-test)

To investigate the molecular consequences of *Ezh1/2* deficiency in fetal mouse ovaries, we extracted mRNA from WT and dKO ovaries at 14.5 dpc + 3 days and conducted RNA sequencing. A total 19,070 genes (S1 Data) were analyzed, revealing 244 differentially expressed genes (DEGs) based on a standard |log2 (fold change) | > 0 and Q value (padj) < 0.05 (S2 Data). These DEGs included 148 up-regulated genes and 96 down-regulated genes (Fig. 5A). The expression patterns of these DEGs were visualized in a heat map (Fig. 5B, Supplemental file 1). These genes were categorized into several groups, including those involved in embryonic development, DNA methylation, histone modification, and DNA damage repair, etc. The relative expression levels of these genes were further validated by RT-qPCR. As depicted in Fig. 5C **and D**, we confirmed the up-regulation of genes associated embryonic development (*Tle6*, *Pkd2*, *Ptk2b*, *Ren1*, *Sod3*, *Zp2*, *Zp3*, *Lrrc38*, *Kcnk18*, *Creg1*, *B4galt4*, *Gper1*), DNA methylation (*Aqp5*, *Pdpn*, *St3ga16*, *Paqr5*, *Chpf*, *Tm6sf1*, *Psen2*, *Abcg1*), histone modification (*Arc*, *Fabp7*, *S100g*, *Meox2*, *Mmp12*, *Muc1*, *Enpp2*, *Gprc5b*, *Ahcy*), and DNA damage or DSBs repair (*Gsta2*, *Foxq1*, *Tgfbr2*, *Ptgds*, *Gucy2g*, *Aldh1b1*, *Tpp1*, *Ctss*, *Lyz2*, *Tal2*, *Pak6*, *Rybp*). Conversely, in Fig. 5E **and F**, we verified the downregulation of genes associated with embryonic development (*Cwc22*, *I111ra2*, *Iqcb1*, *Lypd10*, *Tchh11*, *Tuba3b*, *Wdr54*, *Zfp990*, *Neur13*, *Spib*, *Myh4*), DNA methylation (*Ankrd34b*, *Ankrd37*, *Ppp1r3g*, *Pkp1*, *Aire*, *Phyhipl*), histone modification (*Hoxd1*, *Ndrg1*, *Rhox9*, *Prdm9*, *Stra8*), and DNA repair or DSBs (*Tex30*, *Prdm9*, *Stra8*, *Gm960*, *Iho1*).

These findings show that *Ezh1/2* deficiency impacts gene expression in various processes during embryonic ovarian development, including germ cell development and oocyte meiosis in fetal mouse ovaries.

### Impact of EZH1/2 Knockout on Histone Methylation in Fetal Mouse Ovaries

**Fig. 6:**
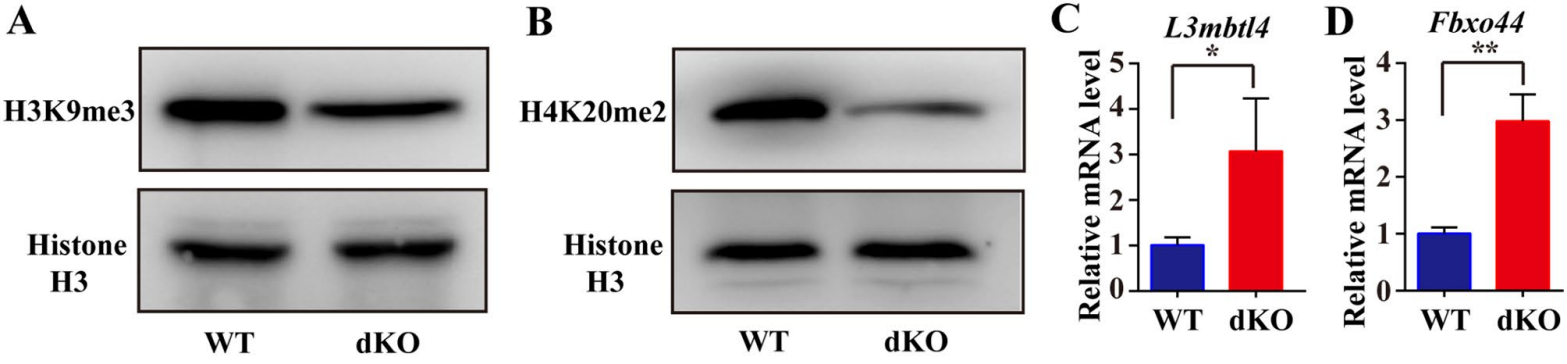
Deletion of *Ezh1/2* affects other histone proteins function in mouse embryonic ovaries. (**A**,** B**) The protein levels of H3K9me3 and H4K20me2 in embryonic ovaries checked by WB. Histone H3 was used as an internal control. Every group had 20 ovaries. (**C**,** D**) The relative expression levels of *L3mbtl4* and *Fbxo44* mRNA in fetal ovaries measured by RT-qPCR. *Gapdh* is a reference gene. The experiments were repeated at least 3 times (*N* = 3). Data were presented as the mean ± S.D. The asterisk (*) denotes a statistically significant difference between the WT and dKO groups. *P < 0.05, **P < 0.01 (*t*- test)

Dynamic changes in histone modifications are essential for the regulation of double-strand break repair during meiotic prophase I in oocytes. Histones such as H3K4me3, H3K79me2/3, H3K9me3, and H4K20me2 play significant roles in the meiosis of oocytes. These histones are involved in the selection of the homologous recombination (HR) repair pathway by facilitating chromatin opening through methylation of H3K9 and H4K20 and recruitment of repair proteins. After infecting 14.5 dpc ovaries with lentivirus in vitro for 3 days, we observed decreased methylation levels of H3K9me3 and H4K20me2 in dKO ovaries (Fig. 6A, B). mRNA level of their target genes *L3mbtl4* and *Fbxo44* was also increased in dKO ovaries (Fig. 6C, D). These findings suggest that the knockout of *Ezh1/2* affects the activity of other histones modification.

## Discussion

Epigenetic modifications, particularly those involving histones, are pivotal for the regulation of meiosis in germ cells. Histone modifications play a crucial role in facilitating DSBs repair in meiosis. Modifications, such as H3K9me3, H3K4me3, H3K36me3, H4K20me2 are key to this process [15, 33, 34]. ZCWPW1 specifically recognizes H3K4me3 and H3K36me3 near the hotspots of recombination catalyzed by PRDM9 and is integral to meiotic DSBs repair [35]. In mice, the deletion of Zcwpw1 leads to male infertility, impedes the meiotic progression of spermatocytes, and results in defects in double-strand break (DSB) repair and homologous recombination [35]. The meiotic prophase of *G9a*-deficient germ cells in mice exhibit a lack of H3K9 methylation and abnormality of synapsis, indicating that G9a-mediated H3K9 methylation was essential for meiotic prophase progression [36]. Ezh1 and Ezh2 are the major complex components of PRC2 in the PcG family of proteins that catalyze the methylation of histone H3 at lysine 27 (H3K27me3). Our experiments demonstrated that EZH1 and EZH2 play key role in early meiotic progress in mouse oocytes, complementing the histone modifications that occur during oocyte meiosis.

Researchers have established an in vitro culture model of mouse embryonic ovaries to study meiotic prophase I. Mouse oocyte meiosis commences at 13.5 days post coitum (dpc), with prophase I concluding approximately at 18.5 dpc. Therefore, oocytes from 13.5 dpc embryonic ovaries were cultured for 3–5 days and reach the pachytene or early diplotene stage of meiosis [37, 38]. Markers like γ-H2AX (for DSBs), MSY2 (for diplotene entry), and SCP1 (for synapsis complex) are used to track meiotic progression. In our experiments, 14.5 dpc or 15.5 dpc embryonic ovaries cultured for 3 days (equivalent to 17.5 or 17.5 dpc) showed expression of MSY2 but a near disappearance of γ-H2AX and SCP1 in oocytes, indicating the oocytes were at the early diplotene stage of meiosis. However, EZH1/2 functional deficiency results in an opposite expression pattern of these marker in oocytes, suggesting a disruption in DSB repair and a blockage in the progression of oocyte meiotic prophase I. This culture system, while valuable, also has limitations, such as the absence of long-term assessment of mouse reproductive capacity, including the female reproductive cycle, egg quality, fertilization rate, and offspring survival rate.

The mechanism of PRC2 in the repair of DSBs in mouse involves the selection of DSBs repair pathways, regulation of chromatin structure, and transcriptional regulation [16, 39]. DSBs repair efficiency is reduced following *Ezh2* knockdown in U2OS cells [39]. There are two primary pathways in DSBs repair: homologous recombination (HR) and non-homologous end joining (NHEJ). In the first meiotic prophase of mouse oocytes, DSBs repair relies on HR, and PRC2 may influence this pathway through ATM signaling to regulate the cellular repair machinery [39]. The PRC2 complex, along with Mre11-Rad50-Nbs1 complex and RNF168, is required for transcriptional repression and DNA repair through ATM-mediated phosphorylation of BRD7 recruited to the active transcription site of the DSBs [40].

ATM kinase promotes meiotic homologous recombination by targeting the axial element proteins in mammals [41]. Hormad1, which is upstream of ATM phosphorylation, is likely involved in activation of ATM kinase. Knockout of *Hormad1* gene leads to the loss of phosphorylation of ATM [31]. Abnormal Hormad1 expression can reduce in the efficiency of homologous recombination repair and an increase in genomic instability. The Mre11-Rad50-Nbs1 complex acts as a DSB sensor for ATM recruiting ATM to the broken DNA molecules [42]. It has been demonstrated that loss of function of the MRN complex causes severe chromosomal aberrations [43, 44]. Conditional deletion of Nbs1 in germ cells can severely disrupt chromosome synapsis, leading to abnormal chromosomal structures and eventual meiotic arrest and male infertility and male infertility [44]. *Nbs1* depletion does not affect the expression level of Mre11 or Rad50 but impairs the nuclear localization of Mre11 and the recruitment of DSBs sites [45]. We hypothesize that a decrease in MRN complex levels may affect phospho-ATM activity, highlighting the crucial correlation between ATM and the MRN complex. Abnormal levels of phospho-ATM and the MRN complex were closely associated with the abnormal accumulation of DSBs.

PRC2 also plays a significant role in regulating the expression of certain genes in the genome. We identified a set of DNA damage or DSBs repair-related genes (*Gsta2*, *Foxq1*, *Tgfbbr2*, *Ptgds*, *Gucy2g*, *Aldh1a1*, *Atp1b1*, *Tpp1*, *Ctss*, *Lyz2*, *Tal2*, *Pak6*, *RYBP*, *Arc*,* Fabp7*, *S100g*, *Meox2*, *Mmp12*, *muc1*, *Enpp2*, *Gprc5b*, *Ahcy*, *Ndrg1*, *Tex30*, *Stra8*, *Iho1*, *Prdm9*, *Gm960*, *Rhox9*, and *Hoxd1*). The expression of these genes was significantly altered in *Ezh1/2* knockout mouse embryonic ovaries. PAK6 activates the ATR/CHK1 signaling pathway, facilitating RAD51 recruitment to the nucleus to enhance HR repair [46, 47]. TPP1 primarily recruit telomerase to telomeres by inhibiting ATR-dependent DNA damage aiding DNA replication and telomere stabilization [48]. Overexpression or knockout of *Tpp1* in EC cells affects the expression of ATM/ATR pathway proteins in DNA damage [49]. High expression of RYBP inhibits the recruitment of BRCA1 repair complexes to DSBs, further reducing DNA end resection and HR repair [50]. These findings suggest that Ezh1/2 influences the expression of multiple genes, impacting oocyte development.

Our experiments revealed reduced level of H3K27me3, H3K9me3, and H4K20me2 methylation in *Ezh1/2*-deficient ovaries, indicating a synergistic role of multiple histone modifications in DSBs repair during meiosis. Other studies have shown interactions between different histone modifications, affecting chromatin structure and repair mechanisms [51]. The H4K20me2-binding factor L3mbtl1 indirectly promotes the recruitment of 53BP1 and triggers L3mbtl1 dissociation from chromatin, thereby facilitating H4K20me2 site recognition that facilitates DSBs repair [52]. 53BP1 was initially shown to bind to the methylation of H3K79, and further studies demonstrated that 53BP1 could also recognize DSB lesions through interaction with H4K20me2 and H2AK15ub [4]. In *Suv39h* double null cells, loss of H3K9me3 in pericentric heterochromatin is associated with a novel domain of H3K27me3, similar to the typical pattern of H3K9me3. When H3K9me3 is eliminated, H3K27me3 is reduced or lost at most of its normal locations and redistributed to constitutive heterochromatin [53]. Fbxo44 is a key regulator in H3K9me3-mediated cancer cells and may induce replication stress and DNA double-strand breaks in cancer cells by stimulating the antiviral pathway and the interferon (IFN) signaling pathways [54].

## Conclusion

**Fig. 7:**
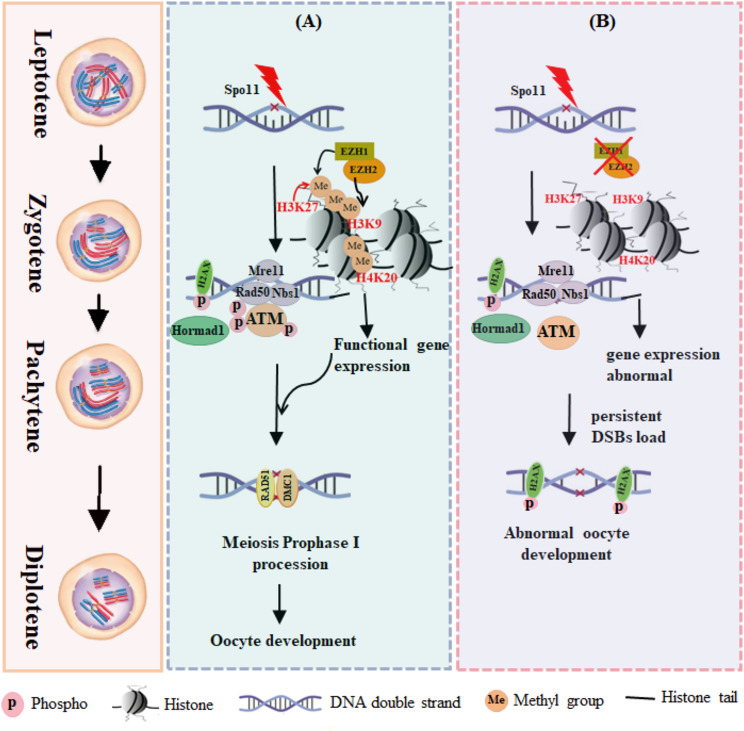
Schematic model of EZH1/2 functioning in oocytes during the first meiotic prophase. (**A**) At the onset of oocyte meiosis, SPO11-mediated DNA double-strand breaks swiftly recruit and activate elements involved in DNA damage repair, including γ-H2AX, Hormad1, the MRN complex, and ATM kinase. EZH1/2, along with other histone modification factors, directly regulate the activation of these DNA damage repair components through chromatin structural modifications and indirectly mediate the transcription of genes associated with meiosis and oocyte development. (**B**) Following the inhibition or knockout of EZH1/2, the activation of DNA damage repair elements—including the expression of Hormad1 and the MRN complex, as well as ATM phosphorylation— and the expression of multiple genes was disturbed. Consequently, DNA damage repair was impeded, adversely affecting the progression of meiotic prophase I

Our findings underscore the importance of EZH1/2 in DSBs repair and meiotic progression by modulating DSB repair pathways, gene expression, and histone modifications in mouse oocytes (Fig. 7). This study sheds new light on the mechanisms underlying mammalian ovarian physiology and pathological conditions related to infertility.

## Electronic supplementary material

Below is the link to the electronic supplementary material.

Supplementary Material 1

Supplementary Material 2

Supplementary Material 3

## Data Availability

All data generated or analyzed during this study are included in this published article and its supplementary information files.

## Abbreviations

ATM: Ataxia Telangiectasia Mutated kinase
ATR: Ataxia-Telangiectasia mutated- and Rad3-related
BSA: Bovine Serum Albumin
BRCA1: Breast cancer susceptibility gene 1
BRD7: Bromodomain Containing 7
CHK1: Checkpoint Kinase 1
CRE: Cyclization Recombinase
DDX4: DEAD-box helicase 4
DEGs: Differential gene expressions
DSBs: DNA double-strand breaks
DMSO: Dimethyl Sulfoxide
ERK: Extracellular regulated kinase
EZH1: Enhancer of Zeste Homolog 1
EZH2: Enhancer of Zeste Homolog 2
Fbox44: F-box protein 44
FBS: Fetal Bovine Serum
GAPDH: Glyceraldehyde-3-phosphate dehydrogenase
GSK343: GSK343 is a highly potent and selective EZH2 inhibitor
H3K27me3: Trimethylated histone H3 at lysine 27
HORMAD1: HORMA domain containing 1
HR: Homologous Recombination
IFN: Interferon
KMT5A: Lysine Methyltransferase 5 A
L3mbtl4: Histone Methyl-Lysine Binding Protein 4
MRE11: Meiotic recombination 11
MRN: MRE11, RAD50, NBS1
MSY2: Mouse Y-box proteins 2
NBS1: Nijmegen breakage syndrome 1
NHEJ: Non-homologous end joining
p-ATM: Phospho-Ataxia telangiectasia mutated kinase
PAK6: P21 (RAC1) Activated Kinase 6
PBS: Phosphate Buffered Saline
PGCs: Primordial germ cells
PRC2: Polycomb Repressive Complex 2
PFA: Paraformaldehyde
PMSF: phenylmethanesulfonylfluoride or phenylmethylsulfonyl fluorid
PRDM9: PR/SET Domain 9
PVDF: PolyVinylidene Fluoride
RAD50: RAD50 double strand break repair protein
RAD51: MRE11, RAD50, NBS1
MSY2: RING1 And YY1 Binding Protein
γ-H2AX: gamma-H2A histone family member X
TPP1: RAS-selective lethal 3
SCP1: Synaptonemal Complex Protein 1
SCP3: Synaptonemal Complex Protein 3
SDS-page: Sodium dodecyl sulfate - polyacrylamide gel electrophoresis
Spo11: Spo11 Initiator of Meiotic Double Strand Breaks
UNC1999: UNC1999 is a SAM-competitive, potent and selective inhibitor of EZH1/2
ZCWPW1: Zinc Finger CW-Type and PWWP Domain Containing 1
53BP1: p53-binding protein 1
