# Supplementary material for: EZH1/2 plays critical roles in oocyte meiosis prophase I in mice
Source: Biol Res. 2024 Nov 8;57:83. doi: 10.1186/s40659-024-00564-4 (PMC11545252; doi:10.1186/s40659-024-00564-4)
Supplement: Supplementary file 1 — Supplementary Material 1 [file 40659_2024_564_MOESM1_ESM.docx]

**Supplementary table S1. Primer sequences for mouse genotype identification**

| Gene | Forward (5’to 3’) |
| --- | --- |
| *Ezh1-F* | *GATGCCCTCAACCAGTACTC* |
| *Ezh1-R* | *TTTATATCACGCACCCACAC* |
| *Ezh1-R (LacZ)* | *TAAAGCGAGTGGCAACATGG* |
| *Ezh2-F* | *GCTAGAAGCATTCCCCACAC* |
| *Ezh2-R* | *CTGGCTCTGTGGAACCAAAC* |
| *Ezh2-R (LacZ)* | *GATGCCCTCAACCAGTACTC* |

**Supplementary table S2. Antibody information.**

| Antibody | Catalog | Manufacturer | Dilution |
| --- | --- | --- | --- |
| DDX4 | #12456P | Cell Signaling Technology | IF, 1:200 WB, 1:1000 |
| DDX4 | #ab27591 | Abcam | IF, 1:200 WB, 1:1000 |
| cleaved-Caspase-3 | #AC033 | Beyotime Biotechnology | IF, 1:200 WB, 1:1000 |
| Caspase-3 | #9662 | Cell Signaling Technology | WB, 1:1000 |
| MSY2 | #sc-21316 | Santa Cruz Biotechnology | IF, 1:200 |
| Ezh1 | #ab263961 | Abcam | IF, 1:200 WB, 1:1000 |
| Ezh2 | #5246 | Cell Signaling Technology | IF, 1:200 WB, 1:1000 |
| H3k27me3 | #9733 | Cell Signaling Technology | IF, 1:200 WB, 1:1000 |
| SCP3 | #sc-20845 | Santa Cruz Biotechnology | IF, 1:200 |
| SCP3 | #ab181746 | Abcam | IF, 1:200 |
| ATM | #ab32420 | Cell Signaling Technology | IF, 1:200 WB, 1:1000 |
| p-ATM | #AA866-1 | Beyotime Biotechnology | IF, 1:200 WB, 1:1000 |
| H4K20me2 | #A22269 | Abclonal | WB, 1:1000 |
| H3K9me3 | #13969 | Cell Signaling Technology | WB, 1:1000 |
| Hormad1 | #ab307424 | Abcam | WB, 1:1000 |
| γ-H2AX | #ab81299 | Abcam | IF, 1:200 |
| Histone H3 | #sc-10809 | Santa Cruz Biotechnology | WB, 1:1000 |
| GAPDH | #ab9485 | Abcam | WB, 1:1000 |

**Supplementary table S3. qRT-PCR Primers**

| *Gene* | *Forward (5’to 3’)* | *Reverse (5’to 3’)* |
| --- | --- | --- |
| *Ezh1* | *AGCGATGCTGTGTTTCTGGA* | *GGCGCTTCCGTTTTCTTGTT* |
| *Ezh2* | *TGACCCTGACCTCTGTCTCACG* | *TCAGACGGTGCCAGCAGTAAGT* |
| *GAPDH* | *AGGTCGGTGTGAACGGATTTG* | *TGTAGACCATGTAGTTGAGGTCA* |
| *Mre11* | *AGAAGGCGACATTTCCTGGG* | *GCAAGAGATTCCTCCTTTGCT* |
| *Rad50* | *GGCGGAAACCTTCTGTCTGA* | *GATCGCCCTAAGAGCTCCAC* |
| *Nbs1* | *AGAAGGCGACATTTCCTGGG* | *GCAAGAGATTCCTCCTTTGCT* |
| *Tm6sf1* | *TCCTAAGATTCAGATGCTGGC* | *ATGAAGGGACGCACCAATGT* |
| *Abcg1* | *TGGGAACGAAGCCAAGAAGG* | *CCAGTAGTTCAGGTGCTCCC* |
| *Tle6* | *AGTTCTCCCCAAATGGCAAGT* | *TTGATGTGGTACACCGAGGC* |
| *Aldh1a1* | *AACACAGGTTGGCAAGTTAATCA* | *TGCGACACAACATTGGCCTT* |
| *Akr1b1* | *ACTCAACAACGGCACCAAGA* | *CCACTCCCACCTCCTTCTCA* |
| *Alox12b* | *GTACGCGATGGAGAGGTACG* | *ATAGTTCGCAAGCAGGTGGG* |
| *Aqp5* | *TAACCTGGCCGTCAATGCG* | *GCCGGTGAAGTAGATCCCCAC* |
| *Arc* | *AGGGGTATGGAAGTGCAGGA* | *GACTCGCTGGTAAGAGCAGG* |
| *Atp1b1* | *GAAGAAGGAGTTTTTGGGCAGG* | *TCTGTGTCAATCCTGGCGG* |
| *Fabp7* | *TGGGAAACGTGACCAAACCA* | *AGCTTGTCTCCATCCAACCG* |
| *S100g* | *GGAGCTGGATAAGAATGGCGA* | *AGAGCGTGCGTTCAATCAGT* |
| *Tpp1* | *CTACTGGGTGGTCAGCAACA* | *CAGCCGTGGGTTACATCAAAG* |
| *Ctss* | *CCACGCTGCCATCAGAAGA* | *TTTTCCCAGATGAGACGCCG* |
| *Pdpn* | *GACAAGAAAACAAGTCACCCCA* | *TTTAGCTCTTTAGGGCGAGAACCT* |
| *Gsta2* | *TGAAGGAGAGAGCCCTGATT* | *GTTGCCCACAAGGTAGTCTT* |
| *Foxq1* | *TGACACTGGCCGAGATCAAC* | *AGGTGTATTCGCTGTTGGGG* |
| *Lyz2* | *AAGAGCTGTGAATGCCTGTGG* | *GCCATGCCACCCATGC* |
| *Meox2* | *AAAGGGAACGCTTCTTCCGT* | *GGCTGTCCTCGTTTGCTAGT* |
| *Paqr5* | *ATTCTCAGCACTGGCCTCTC* | *TGGGAACAGGAAGAGCCTG* |
| *Mmp12* | *AGTACCAGAGCCACACTATCCC* | *CTCCTGCCTCACATCATACCTC* |
| *Muc1* | *TACCACACTCACGGACGCTA* | *AGACTGCTACTGCCATTACCTG* |
| *Enpp2* | *CTGTCGGTGTGACAACCTATGTA* | *TTGGTAGTTGGTACAGCAGTCTC* |
| *Pkd2* | *GCCCAGCAGAAAGCAGAAATG* | *TCTGTATGGCCCTTCCCTTT* |
| *Chpf* | *TCTGCCCACGCATTGAAGTA* | *CAGTCGGTGTCCCAGAGTTC* |
| *Psen2* | *AGAACGGGCAGCTCATCTAC* | *AGGAGCATCAGGGAGGACA* |
| *Ptgds* | *GCCTCAATCTCACCTCTACCTTC* | *CCTTGGTGCCTCTGCTGAATA* |
| *Ptk2b* | *GCTTCACAACCGCCAGT* | *CTTTCTCCAGCACTCCGAT* |
| *Ren1* | *GGGAGCCAAGGAGAAGAGAATAG* | *TGTCTCTCCTGTTGGGATACTGTAG* |
| *Sod3* | *GAGAAGATAGGCGACACGCA* | *GAAGAGAACCAAGCCGGTGA* |
| *Tal2* | *ACAGCTACCTTGACTGCGCC* | *AAAGGCATTGTTGACACTCTGC* |
| *Pak6* | *GAGCTGTGGGTGTTGATGGA* | *ACAGTGGCAATCTGCTCCTC* |
| *Tgfbr2* | *ACGTTCCCAAGTCGGATGTG* | *TTCTGGTTGTCGCAAGTGGA* |
| *Zp2* | *GCCAGCCAATGCTACTGGAA* | *AGCGTGTGATGAGAAGACGA* |
| *Zp3* | *CCGAACTCCTCCCCCTATCA* | *GGCAGGTGATGTAGAGCGTA* |
| *Fbxo44* | *TCGAGGTCAAGGACTGGTTT* | *GACGCCGGGAGGATAGTTG* |
| *Lrrc38* | *GTTTCAGCGAGTGTAAGTTCAGC* | *ACAGCCACCCCAGAGAAGAT* |
| *Ahcy* | *GGTGCTGAGGTGCGGT* | *CATGTTGAGGGGTCCGTCC* |
| *Kcnk18* | *AGTGCTCCTGGTTTTGCCAT* | *TTGAAGGGCGAAGCTGTCAA* |
| *Creg1* | *TGTCGGGAACTGTGACCAAG* | *TTATGGCTGGAAGGCCAGTG* |
| *St3gal6* | *ACGAGTTTGACAGAGTGCCAT* | *GCCTAAGACAGGACCGTTGT* |
| *B4galt4* | *ACAACTACTGGGGATGGGGA* | *CAACAGCTTCATTCGGCCC* |
| *L3mbtl4* | *ACCTGAGAGAAGGGAACGCT* | *GTAGCCACACACCTCTGCTA* |
| *Gper1* | *GAAAGCCCTGAGGATGATCTTC* | *GTAGGTGGACACTGATGAAGAC* |
| *Rybp* | *GCACAGCAGTTGGCAGTAAC* | *TGGAGGAGGAGCGAGTCTTT* |
| *Gprc5b* | *CGATCAGCAGTGGGCTTTTC* | *AGCCATTTCAGTCCCTCCATT* |
| *Gucy2g* | *GCTGGGAGTTCACTTACACCAA* | *GAGGCCAATAAACCAATAACCTCTG* |
| *Zfp980* | *TGTACATGGATGTGATGTTGGAGA* | *CCCTCATTTGAGGTCTCTAAGTATT* |
| *Eif3j2* | *CCAACTCATTGACTGTGCTCTG* | *GCCTCCTTCATATCCACCATAATCT* |
| *Rhox9* | *ACGATGGATGGGTGTGGATG* | *TCGGTGGCAGTTCGCAGA* |
| *Rhox9* | *ACGATGGATGGGTGTGGATG* | *TCGGTGGCAGTTCGCAGA* |
| *Zfp990* | *CGTGGAAAGAATGGGAAGGTAT* | *CGAGTATTGAAGAGAGTCAGAGC* |
| *Aire* | *GAAGAACGAGGATGAGTGTGC* | *TGGAGGCAGCAGGAGCAT* |
| *Hoxd1* | *CCCACAGCACTTTCGAGTGG* | *GATGCGTCGGGCTCTAGTTA* |
| *Il11ra2* | *ATACCGACCAGCACAGCATC* | *TGCCAGCATCCAGAAAGTCC* |
| *Mid1* | *TGGCTAAACTCATCCAAACTTGTC* | *GCTAACTTGCGGAGCCTGAT* |
| *Myh4* | *TCATGTCCAATAAGAAACCAGAGC* | *AGCGCCTGTGAGCTTGTAAA* |
| *Ndrg1* | *CTGCCTGACATGGTGGTGTC* | *GTGTAGGTTGCTCGGGTTCA* |
| *Pkp1* | *ATGACCTCACAGCCTCAGAT* | *TCCCATCATATTCCTATCGAAACCT* |
| *Stra8* | *CAAGAGTGAGGCCCAGCATA* | *GGGCTCTGGTTCCTGGTTTAAT* |
| *Prdm9* | *CTGGACAGCACTCTGGGAAA* | *GACCTCCTCATAGGCAAGGC* |
| *Ankrd34b* | *GTGTTGGAAAGGCGTGGTTC* | *CATTCAAGGGCGGCAGAAAG* |
| *Tuba3b* | *CAAGCGCACCATCCAGTTTG* | *ATGGCCGTGGTATTGCTCA* |
| *Lypd10* | *TTCCTTGGGAGTGCATGGAG* | *GTCAGTAAGATGCTCGGGACA* |
| *Rab39* | *GTCGGCGTGGACTTCTTCTC* | *AGTTGCGGTAATAGGATCGAGT* |
| *Spib* | *TGGAGGTCTCGGACAGTGAG* | *TACAGGCGCAGCTTCTTGC* |
| *Iqcb1* | *TGTTCAGATAGGAGCTTCAGTCAT* | *AGCAGGAGCTTTGTAGCAGT* |
| *Gm960* | *GCAGCAGAATCCACCCTGT* | *TAGCAGAGGCAGACCCAAAG* |
| *Zfp985* | *GGCCTTGGACTTCTCACTGG* | *CAATGTACTGGCTGTCTTGATCC* |
| *Iho1* | *AGTGAGAGACAAAGGCAGCA* | *TTCAAGGTTCTTCAGGGTGGA* |
| *Tmod4* | *CAGCACAGGAACAGATTACGC* | *TGTACCACACTGCTAATGCCT* |
| *Ankrd37* | *CATCAGGTGAGCAACAGGAG* | *ACTCCACTCTCCACAGTCCT* |
| *Phyhipl* | *CAAGTCGAAGGACCGCATCA* | *CCAGTGTCCACGAACAGTCA* |
| *Tchhl1* | *CAACACTGACCCGCACAGA* | *TCTCTCCACAGCATGAAAGACA* |
| *Tex30* | *TCCCACCTTGCATCTCATGG* | *TGGCTCAGTATGACACATCACA* |
| *Wdr54* | *GCGTCCACTTCATCTGTGTG* | *GTCACCATGTCAGCAACACC* |
| *Ppp1r3g* | *GTCCAGCTCTACGTCTTCCG* | *AGCGGCGATATTCCTGTAGC* |
| *Cwc22* | *AGGATACTCTTACGATGACAGCA* | *TCCATGTCGGTTCCTCCTGA* |
| *Neur* | *CCAGCATGTGGTGCCTAAGT* | *GGTGTGGCCGGAAAAAGAAA* |
| *Hormad1* | *ACAACATCTGCTGACACCAAGAA* | *TACTCCTTCACAGTCACCATCCT* |
