# Supplementary material for: EZH1/2 plays critical roles in oocyte meiosis prophase I in mice
Source: Biol Res. 2024 Nov 8;57:83. doi: 10.1186/s40659-024-00564-4 (PMC11545252; doi:10.1186/s40659-024-00564-4)
Supplement: Supplementary file 2 — Supplementary Material 2 [file 40659_2024_564_MOESM2_ESM.pdf]

Gene ID	Gene Symbol	WT1	WT2	WT3	dKO1	dKO2	dKO3	WT Average	dKO Average	log2 (dF / dC)	Pvalue(dF / dC)	Qvalue (dF / dC
100009600	'Zglp1'	27	29	23	2	31	52	26.33333333	28.33333333	-0.020497768	0.98187102	0.999493374
100017	'Ldlrap1'	574	537	954	2142	570	1058	688.3333333	1256.666667	1.056689707	0.143728169	0.750606785
100019	'Mdn1'	8362	7700	1180	1879	4929	5173	5747.333333	3993.666667	-0.474342792	0.478812982	0.972790461
100033459	'Ifi208'	3	4	4.66	1	0	1	3.886666667	0.666666667	-2.377646627	0.166259778	0.780893009
100034251	'Wfdc17'	1	0	1	2	3	6	0.666666667	3.666666667	2.417042969	0.167096565	0.781318221
100034361	'Mfap1b'	1174	1247.3	1487	1081.4	1051.2	1338.43	1302.74	1157.016667	-0.121202873	0.746825909	0.972790461
100034363	'Tmsb15b2'	40.29	31.27	16.6	6.13	50.18	21.08	29.38666667	25.79666667	-0.203720491	0.79439774	0.972790461
100034684	'Cstdc5'	0	21	3	2	59	0	8	20.33333333	1.32489283	0.509834035	0.972790461
100034748	'A930017K11Rik'	15	14	6	10	2	12	11.66666667	8	-0.388388511	0.688436661	0.972790461
100036518	'Vmn1r223'	0	2	0	0	0	0	0.666666667	0	-1.695595436	0.675304306	0.972790461
100036521	'Umad1'	141	135	155	130	96	136	143.6666667	120.6666667	-0.167039459	0.707983005	0.972790461
100036568	'Gm3373'	12.75	9.13	7.98	0	3.12	0	9.953333333	1.04	-3.236672684	0.031698256	0.432625437
100037258	'Dnajc3'	1229	1120	2772	1181	1219	2937	1707	1779	-0.017066211	0.977420688	0.999493374
100037278	'Niban3'	73	77.72	71.01	28	73	50	73.91	50.33333333	-0.553654689	0.203821584	0.832079769
100037282	'Rsph3b'	509.36	609.07	288.66	89.21	367.27	600.99	469.03	352.49	-0.471934638	0.422989517	0.959880246
100037283	'Rnaset2a'	2200.7	2584.3	1614.6	690.48	2602.9	2562.16	2133.18	1951.836667	-0.171060832	0.705955655	0.972790461
100038347	'Fam174b'	81	79	665	95	115	154	275	121.3333333	-1.305294943	0.156574761	0.768046725
100038489	'Apela'	2	2	48	87	10	39	17.33333333	45.33333333	1.473495977	0.285799403	0.893820325
100038514	'Gm11837'	81	99.46	26.87	9.99	70.03	115.66	69.11	65.22666667	-0.144111311	0.85948899	0.976863639
100038538	'Gm10767'	19	21	6.96	11	29	21	15.65333333	20.33333333	0.454777707	0.516120731	0.972790461
100038570	'Prcd'	253	381	41	11	514	368	225	297.6666667	0.354474171	0.739079234	0.972790461
100038657	'Myocos'	1	0	0	14	0	3	0.333333333	5.666666667	4.357024185	0.072334036	0.595548709
100038725	'Cep85l'	654	691	541	763	548	532	628.6666667	614.3333333	0.119803497	0.790167739	0.972790461
100038847	'Gm10406'	144.97	168.75	99.25	7	97.66	23.67	137.6566667	42.77666667	-1.752795842	0.029859537	0.421134658
100038854	'Cstdc6'	0	6	0	0	0	0	2	0	-3.26985255	0.408345332	0.957157474
100038862	'Btnl1'	10	8	0	0	2	0	6	0.666666667	-3.101734209	0.182252682	0.803009965
100038882	'Isg15'	50	52	355	44	12	399	152.3333333	151.6666667	-0.270204811	0.823093734	0.972790461
100038909	'Gm14548'	0	2.82	0	1	1.43	2.13	0.94	1.52	1.106361402	0.654069527	0.972790461
100038947	'Sirpb1c'	2	1.23	0	3	2	2.51	1.076666667	2.503333333	1.414787056	0.428372937	0.962191208
100038949	'Gm1979'	18.14	33.83	19.5	119.11	37.05	81.55	23.82333333	79.23666667	1.965201302	0.005931065	0.181288429
100039008	'Mup10'	0	0	0	0	2	0	0	0.666666667	1.801491674	0.656112935	0.972790461
100039028	'Mup11'	0	0	0	0	0	2.5	0	0.833333333	1.749449488	0.66566965	0.972790461
100039045	'Gm10471'	73.1	77.71	11.33	212.34	109.82	100.94	54.04666667	141.0333333	1.671728479	0.035146559	0.449667823
100039060	'Gm2026'	959.69	1136.2	606.91	279.05	755	716.63	900.9266667	583.56	-0.626507763	0.102468503	0.671776916
100039087	'Gm2042'	0	0	1.43	0	0	0	0.476666667	0	-0.903279821	0.824807108	0.972790461
100039123	'Gm14295'	1051.6	1063.7	487.86	397.19	1049.1	834.8	867.71	760.3566667	-0.164908146	0.6896278	0.972790461
100039139	'Ccdc152'	188.52	204.64	37	4.06	111.2	154.29	143.3866667	89.85	-0.741136259	0.458664932	0.972790461
100039177	'Mup16'	0	0	0	0	0	2.64	0	0.88	1.749449488	0.66566965	0.972790461
100039210	'Gm2102'	15	12	9	0	15	26	12	13.66666667	0.059302486	0.959855165	0.999493374

100039239	'Gm2115'	2	6	13	3	5	0	7	2.666666667	-1.369114412	0.35650489	0.932066196
100039252	'Gm12693'	1.58	2.39	0	6.45	7.23	4.33	1.323333333	6.003333333	2.656773592	0.072505145	0.595707787
100039269	'Gm2128'	1	0	0	0	9	14	0.333333333	7.666666667	4.26753152	0.059044522	0.552865869
100039284	'Gm2137'	13.97	15.82	7.22	6.76	20.54	7.45	12.33666667	11.58333333	-0.046328027	0.954757305	0.999493374
100039315	'Pramel51'	1	1	0	0	0	0	0.666666667	0	-1.703500596	0.673820112	0.972790461
100039350	'Gm16500'	3	5	4.94	0	0	2	4.313333333	0.666666667	-2.647429659	0.160427532	0.771114735
100039441	'Gm2237'	139.01	112.99	101.1	29.42	175.18	83.45	117.7	96.01666667	-0.347385288	0.559764045	0.972790461
100039532	'Gm10029'	669.62	519.21	974.56	0	0	0	721.13	0	-11.92641496	7.70E-20	2.93E-16
100039596	'Tcf24'	436	404	57	482	191	317	299	330	0.418094253	0.595463851	0.972790461
100039672	'Msmp'	3.58	1.26	0	0	1.21	1.19	1.613333333	0.8	-0.974471758	0.701610197	0.972790461
100039674	'Gm10634'	64.71	57.32	18.01	0	14.58	9.58	46.68	8.053333333	-2.641612494	0.00757925	0.209755373
100039681	'Zcwpw2'	492.5	429.15	213.04	3.52	115.2	50.28	378.23	56.33333333	-2.833576357	0.001556362	0.084694293
100039707	'Mthfsl'	324.96	371.91	621.8	220.11	733.46	587.13	439.5566667	513.5666667	0.131479061	0.804484945	0.972790461
100039781	'Hrct1'	0	1	1	1	5	9	0.666666667	5	2.792926903	0.111486076	0.695565384
100039786	'Gm2423'	1786.6	1964.7	1948.9	1047.6	1822	1673.52	1900.043333	1514.363333	-0.317440358	0.267367176	0.883600995
100039789	'Gm12407'	2.6	0	4.47	2	3	2	2.356666667	2.333333333	0.186676628	0.910978601	0.989640031
100039794	'Gm2427'	0	0	2.76	0	0	0	0.92	0	-1.995285526	0.619817042	0.972790461
100039795	'Ildr2'	2253	2026	512	2185	2448	3345	1597	2659.333333	0.854083817	0.115312614	0.702526958
100039796	'Tgtp2'	6.12	22.97	50.51	0	3.17	31.13	26.53333333	11.43333333	-1.475973691	0.303590506	0.906520549
100039815	'Gm2436'	11.27	11.24	26.84	17.86	19.7	28.74	16.45	22.1	0.388458601	0.591495474	0.972790461
100039830	'Gm2446'	11.27	11.24	26.84	17.86	19.7	28.74	16.45	22.1	0.388458601	0.591495474	0.972790461
100039863	'Gm13306'	127.24	69.35	40.27	91.18	165.01	77.06	78.95333333	111.0833333	0.599397641	0.292942404	0.900145058
100039890	'Gm15093'	0	0	0	0	0	9.29	0	3.096666667	3.842052349	0.329786588	0.921648675
100039899	'Tex13d'	215	229	31	0	118	187	158.3333333	101.6666667	-0.715306629	0.589230128	0.972790461
100039913	'Rhox2b'	52.28	71.55	17.48	1	20.9	39.69	47.10333333	20.53	-1.294322428	0.188813632	0.811237237
100039934	'Gm15085'	0	5.06	0	0	1.66	0	1.686666667	0.553333333	-2.046244302	0.605319211	0.972790461
100039946	'Siglecl1'	0	0	1	0	0	0	0.333333333	0	-0.903279821	0.824807108	0.972790461
100039948	'Rhox2c'	39.1	24.19	5.22	0	8.09	32.68	22.83666667	13.59	-0.849095415	0.516812235	0.972790461
100039953	'Gfy'	78	96	14	0	42	8	62.66666667	16.66666667	-1.945926055	0.121835578	0.712603747
100039968	'Tmem35b'	169	164	66	38	270	251	133	186.3333333	0.437844063	0.510721981	0.972790461
100040016	'Rhox2e'	21.47	23.25	8.42	1	9.69	17.33	17.71333333	9.34	-0.998050344	0.295055023	0.90029104
100040048	'Ccl27b'	371.41	462.98	110.92	89.43	214.77	130.47	315.1033333	144.89	-1.045336677	0.064562696	0.570648987
100040049	'Smim38'	3	4	1	12	10	3	2.666666667	8.333333333	1.864151135	0.115760678	0.702825844
100040260	'Atf1-ps'	0	0	0	11.24	1.01	1.97	0	4.74	4.94486053	0.046690569	0.503220574
100040268	'Olfr157'	1	0	0	0	0	0	0.333333333	0	-0.903279821	0.824807108	0.972790461
100040298	'Gm15501'	183.3	230.53	207.52	209.2	106.02	130	207.1166667	148.4066667	-0.309947154	0.574761159	0.972790461
100040416	'Rpl13-ps6'	0	3.18	15.51	1.32	7.83	3.2	6.23	4.116666667	-0.920322345	0.577579329	0.972790461
100040462	'Mndal'	29	20	88.32	10	50	62	45.77333333	40.66666667	-0.362400236	0.67425085	0.972790461
100040500	'Gm2808'	173.02	226.83	30.56	10.38	21.35	19.48	143.47	17.07	-3.011873717	3.84E-05	0.007055669
100040531	'Dynlt1f'	338.67	358.64	351.92	271.3	429.52	435.46	349.7433333	378.76	0.133989081	0.63826624	0.972790461

100040545	'Gm2832'	5.96	1.05	1	0	2	0	2.67	0.666666667	-1.795029274	0.437753328	0.96622803
100040563	'Dynlt1c'	756.55	969.76	439.74	402.89	499.14	618.68	722.0166667	506.9033333	-0.431686145	0.222257581	0.84952978
100040591	'Kcnj13'	2	7	0	20	3	14	3	12.33333333	2.33540196	0.092825391	0.654355334
100040599	'Gm15319'	11.13	12.24	2.04	1.69	10.01	6.65	8.47	6.116666667	-0.555195293	0.624797944	0.972790461
100040608	'Fancf'	118.91	105.83	132.41	154.73	112.35	156.13	119.05	141.07	0.33505022	0.468584662	0.972790461
100040635	'Gm15023'	389.24	371.63	28.5	3	316.46	483.13	263.1233333	267.53	-0.037593914	0.975713657	0.999493374
100040657	'Gm2888'	2	1	1.01	0	0	0	1.336666667	0	-2.782259126	0.384965334	0.94674509
100040671	'Gm2897'	81.55	114.43	61.03	7.65	46.24	11.6	85.67	21.83	-2.012697119	0.005661475	0.177617092
100040682	'Esd-ps'	1	2	0	0	0	0	1	0	-2.28103714	0.568641838	0.972790461
100040697	'Gm10340'	0	0	0	0.28	1.45	0	0	0.576666667	1.020273531	0.802557913	0.972790461
100040766	'Mroh2a'	1.02	3.02	0	1	1.01	2	1.346666667	1.336666667	0.109119676	0.956209464	0.999493374
100040807	'Gm2977'	2.13	0.31	7.83	0	0	0	3.423333333	0	-4.118319299	0.204655271	0.833294914
100040843	'Cyp4a32'	0	0	0	0	1	1	0	0.666666667	1.775692139	0.660844521	0.972790461
100040870	'Gm3005'	20.68	42.04	34.79	0	6.44	0	32.50333333	2.146666667	-4.061814415	0.001055325	0.068012985
100040935	'Gm3050'	2.13	0.31	7.83	0	0	0	3.423333333	0	-4.118319299	0.204655271	0.833294914
100040937	'Cldn34b1'	11	9	0	0	2	7	6.666666667	3	-1.190328021	0.512961033	0.972790461
100040944	'Gm3055'	28.51	0	11.11	57.15	161.76	240.89	13.20666667	153.2666667	3.493437403	9.96E-04	0.065718218
100040961	'Gm10145'	1.06	0	2.25	1.12	0	0	1.103333333	0.373333333	-1.526634549	0.634193714	0.972790461
100040972	'Tceal7'	17	9	8	0	4	7	11.33333333	3.666666667	-1.706941973	0.132450783	0.730178517
100041012	'Gm3095'	31.37	30.19	20.4	0	17.61	3.04	27.32	6.883333333	-2.092990952	0.063358715	0.567231213
100041057	'LOC100041057'	0	0	0	0	0	5	0	1.666666667	3.001401729	0.449669959	0.97049895
100041106	'Gm3141'	0	0	0	0	1.75	0	0	0.583333333	1.020273531	0.802557913	0.972790461
100041121	'Gm10144'	4	5	0	2	10	5	3	5.666666667	0.964158961	0.489635069	0.972790461
100041146	'Gm15448'	0	0	0	0	0	1	0	0.333333333	1.020273531	0.802557913	0.972790461
100041151	'Gm3636'	516.41	534.75	200.28	54.92	463.31	151.95	417.1466667	223.3933333	-0.924932256	0.195408134	0.820291331
100041194	'Ahnak2'	215	146	883	684	299	644	414.6666667	542.3333333	0.397639934	0.638521338	0.972790461
100041230	'H4c17'	0	0	2.61	2.52	4.5	4.01	0.87	3.676666667	2.196316068	0.26789015	0.883600995
100041253	'Gm16513'	0	0	0	0	0	2.18	0	0.726666667	1.749449488	0.66566965	0.972790461
100041262	'Gm3239'	67.66	63.51	21.94	2	36.27	14.14	51.03666667	17.47	-1.571280195	0.078463517	0.615492265
100041273	'Ndufb4c'	319.52	307.07	220.27	372.99	164.06	281.15	282.2866667	272.7333333	0.132904872	0.806009749	0.972790461
100041279	'Gm3248'	0	0	0	1	1	0	0	0.666666667	2.050872943	0.610671515	0.972790461
100041283	'Gm3252'	46.8	43.37	19.56	2	21.91	5.71	36.57666667	9.873333333	-1.956095919	0.026027107	0.397137149
100041294	'Supt4b'	0	0	0	0	1.65	0	0	0.55	1.020273531	0.802557913	0.972790461
100041306	'Gm3264'	114.01	110.98	113.43	3	42.35	12.42	112.8066667	19.25666667	-2.648168666	6.80E-04	0.052033928
100041352	'Tcp10c'	2	4.08	2.71	1	3	0	2.93	1.333333333	-0.929818686	0.589967571	0.972790461
100041354	'Gm3286'	0	0	0	0	1	0	0	0.333333333	1.020273531	0.802557913	0.972790461
100041379	'Zfp980'	86.77	98.01	38.39	1.19	19.43	6.88	74.39	9.166666667	-3.116222355	2.09E-04	0.023202252
100041420	'Gm3325'	623.54	634.23	189.23	148.16	983.61	751.43	482.3333333	627.7333333	0.354493247	0.592906084	0.972790461
100041433	'Zfp981'	63.55	72.33	20.9	7	88	56	52.26	50.33333333	-0.079453413	0.923567016	0.991932584
100041449	'Cyp3a59'	0	1	0	0	0	0	0.333333333	0	-0.903279821	0.824807108	0.972790461

100041515	'Gm3383'	57.62	45.62	53.55	2.3	63.38	37.56	52.26333333	34.41333333	-0.735143589	0.415878402	0.95722888
100041546	'Ly6c2'	0	0	0	0	1	0	0	0.333333333	1.020273531	0.802557913	0.972790461
100041574	'9030025P20Rik'	291.72	246.52	90.95	152.77	255.73	211.17	209.73	206.5566667	0.068675691	0.884542854	0.98386821
100041578	'Gm3415'	0	1	0	0	0	0	0.333333333	0	-0.903279821	0.824807108	0.972790461
100041579	'Gm20878'	0.26	1.5	0.72	1	0	0.95	0.826666667	0.65	0.058500858	0.988561293	0.999493374
100041581	'Zkscan16'	291	299	12	72	521	248	200.6666667	280.3333333	0.509882312	0.604515711	0.972790461
100041585	'Amd2'	103.4	98.11	47.37	51.9	56.5	64.01	82.96	57.47	-0.436839805	0.323373636	0.918976057
100041586	'Gm3417'	2	0	3.59	0.89	18.82	16.98	1.863333333	12.23	2.547723728	0.146574082	0.753611197
100041596	'Gal3st2b'	1.24	0	0	0	0	0	0.413333333	0	-0.903279821	0.824807108	0.972790461
100041598	'Gm3424'	0	0	1	0	0	0	0.333333333	0	-0.903279821	0.824807108	0.972790461
100041621	'Gm3435'	48.01	42.11	3.53	47.94	160.3	68.86	31.21666667	92.36666667	1.646073603	0.060356537	0.557850498
100041639	'Gm3448'	3.6	23.62	10.7	3.81	18.82	19.02	12.64	13.88333333	0.08584306	0.932524671	0.993954086
100041658	'Mup7'	0	0	0	3	1	0	0	1.333333333	3.197118853	0.416786276	0.95722888
100041677	'Zfp984'	378.19	399.57	565.3	191.87	562.38	482.04	447.6866667	412.0966667	-0.190011223	0.679631917	0.972790461
100041678	'Gm3500'	9.91	6.15	12.36	1	9.61	6.68	9.473333333	5.763333333	-0.854462457	0.402919228	0.955996639
100041702	'Gm3476'	4.99	3.56	2	1	0.83	0	3.516666667	0.61	-2.98153801	0.149906356	0.759066532
100041708	'LOC100041708'	1	0	0	0	0	0	0.333333333	0	-0.903279821	0.824807108	0.972790461
100041722	'1700049E17Rik2'	7.6	12.82	1	0	3.02	8.19	7.14	3.736666667	-0.907551194	0.543452098	0.972790461
100041734	'4930522L14Rik'	282.63	305	150.01	273	416.14	268.88	245.88	319.34	0.475956585	0.238779733	0.864053647
100041735	'Gm3488'	47.18	47.05	19.85	13	48.54	35.88	38.02666667	32.47333333	-0.224626949	0.710299517	0.972790461
100041749	'Speer4f2'	0	0	0	0	1	3	0	1.333333333	2.701386663	0.497818139	0.972790461
100041774	'Gm10413'	0	0	2.13	0	2	1	0.71	1	0.294408378	0.92562677	0.992381554
100041840	'Gm10408'	2.88	8	1.44	1	2.48	0	4.106666667	1.16	-1.721257522	0.341258135	0.925308103
100041874	'Gm3558'	71.6	64.74	40.3	2.13	15.14	1.37	58.88	6.213333333	-3.272399366	3.61E-04	0.034287299
100041903	'LOC100041903'	1	0	0	0	0	0	0.333333333	0	-0.903279821	0.824807108	0.972790461
100041953	'Sap18b'	2034.8	2200.6	4144.8	1343.5	1621.3	2914.26	2793.426667	1959.673333	-0.56346895	0.273088888	0.884738928
100041958	'Lamtor3-ps'	4.47	6	3.25	1	1.58	1.06	4.573333333	1.213333333	-2.035726872	0.158560253	0.768890221
100041964	'Gm3594'	1	0	0	0	1	0	0.333333333	0.333333333	0.058500858	0.988561293	0.999493374
100041979	'Gm3604'	249.76	335.51	150.06	40	252.78	166.73	245.11	153.17	-0.719811191	0.229265138	0.855596006
100042024	'Gm3629'	2.79	0	2.34	0	1	1	1.71	0.666666667	-1.114239787	0.652737689	0.972790461
100042065	'Gm3646'	2	0	0	0	0	0	0.666666667	0	-1.711373851	0.67234292	0.972790461
100042074	'Gm3650'	83.65	88.6	71.56	4.26	1.89	3.83	81.27	3.326666667	-4.834371984	2.33E-11	2.95E-08
100042100	'Gm3667'	30.95	42.98	38.27	0	18.13	6.88	37.4	8.336666667	-2.309142622	0.0233442	0.379052873
100042149	'Gm3696'	74.34	53.93	82.42	5.75	40.97	9.7	70.23	18.80666667	-2.028527106	0.010277311	0.250824438
100042150	'Nrg2'	68	46	165	31	54	32	93	39	-1.297433028	0.065051249	0.572836659
100042165	'Thoc2l'	918	1008	386	1381	1340	1062	770.6666667	1261	0.876189205	0.067025919	0.578437029
100042235	'Gm3739'	76.6	87.83	101.2	1	30.66	6.81	88.54333333	12.82333333	-2.94553764	0.001356497	0.078867628
100042295	'Gm3776'	0	0	3	3	0	2.73	1	1.91	0.777899743	0.779238351	0.972790461
100042304	'Gm38418'	2	3.76	1	0	1	1	2.253333333	0.666666667	-1.586116296	0.426292054	0.960958977
100042335	'Rps15a-ps5'	668.35	788.41	1042.6	983.66	576	753.03	833.11	770.8966667	0.002307405	0.996521991	0.999616639

100042450	'Smim17'	2	4	0	2	5	7	2	4.666666667	1.265954555	0.384419735	0.94670829
100042480	'Nhsl2'	636.2	558.01	405.78	285.7	601.89	366.98	533.33	418.19	-0.309829855	0.38826928	0.950023663
100042493	'Ccl21b'	26.39	21.98	13.39	50.81	33.23	57.81	20.58666667	47.28333333	1.34367256	0.019391833	0.347725165
100042499	'Vmn2r55'	0	0	0	0	0	1	0	0.333333333	1.020273531	0.802557913	0.972790461
100042503	'Ndufb4b'	0	3.67	0	0	0	3.12	1.223333333	1.04	-0.059575695	0.987908582	0.999493374
100042514	'Sprr2a3'	0	0	3.36	0	1	0	1.12	0.333333333	-1.626789964	0.682990322	0.972790461
100042533	'Gm11578'	0	7.02	2.21	7.47	38.54	3.05	3.076666667	16.35333333	2.432397255	0.092937825	0.654455509
100042555	'Gm13305'	59.41	54.24	21.49	0	0.78	0	45.04666667	0.26	-7.814564568	2.90E-08	1.78E-05
100042625	'Gstp-ps'	19.08	29.41	30.01	61.79	11.57	42.05	26.16666667	38.47	0.731081219	0.38158496	0.94429001
100042698	'Gm3973'	80.66	53.27	28.72	55.16	53.85	26.15	54.21666667	45.05333333	-0.080363681	0.900920419	0.98672375
100042715	'Gm3985'	0	3.23	0	0	2.32	0	1.076666667	0.773333333	-0.532516362	0.893197773	0.985171
100042773	'Tmed10-ps'	11.86	11.8	7.6	2.26	8.2	3.08	10.42	4.513333333	-1.139212811	0.230365277	0.857148178
100042776	'Gm4027'	2	2.21	0	0	1	0	1.403333333	0.333333333	-1.740097262	0.578803281	0.972790461
100042781	'Vmn2r40'	0	0	0	0	0	1	0	0.333333333	1.020273531	0.802557913	0.972790461
100042782	'Fth117d'	0	0	1	0	0	0	0.333333333	0	-0.903279821	0.824807108	0.972790461
100042784	'Prdm11'	1701	1689	504	259	1974	1242	1298	1158.333333	-0.189683478	0.781113017	0.972790461
100042785	'Cldn25'	1	0	0	0	0	0	0.333333333	0	-0.903279821	0.824807108	0.972790461
100042807	'Eif3j2'	1582	1529.5	1709.7	51.33	19.46	30.19	1607.06	33.66	-5.422327747	2.89E-20	1.38E-16
100042848	'Vmn2r41'	0	0	0	0	0	1	0	0.333333333	1.020273531	0.802557913	0.972790461
100042856	'Gvin2'	53.96	58.43	39.57	45.21	47.49	67.52	50.65333333	53.40666667	0.148205439	0.721564336	0.972790461
100042894	'Vmn2r46'	1.78	1.58	1.46	0	0	1.44	1.606666667	0.48	-1.427506251	0.602905389	0.972790461
100042922	'H2al1a'	1	0	0	0	0	0	0.333333333	0	-0.903279821	0.824807108	0.972790461
100042945	'Gm4120'	22.28	24.32	23.06	14.01	25.57	14.15	23.22	17.91	-0.340127248	0.563263321	0.972790461
100042960	'Gm4131'	0	0	0	1	0	1	0	0.666666667	2.022653929	0.615796212	0.972790461
100043002	'Llph-ps2'	269.08	331.56	679.8	69.6	80.67	158.01	426.8133333	102.76	-2.125136572	3.00E-04	0.030177047
100043034	'Rex2'	161.55	135.04	60.93	4.06	18.23	4.68	119.1733333	8.99	-3.728888669	2.37E-07	1.25E-04
100043059	'Gm4199'	6.21	4.06	18.82	0	0	1.02	9.696666667	0.34	-4.74812762	0.007640532	0.21034504
100043125	'Cd300ld5'	2	0	0	0	0	0	0.666666667	0	-1.711373851	0.67234292	0.972790461
100043133	'9130023H24Rik'	432.8	435.03	92.72	80.26	331.8	271.17	320.1833333	227.7433333	-0.475810154	0.466506006	0.972790461
100043188	'Spopfm2'	1	1	0	0	1	1	0.666666667	0.666666667	0.015019958	0.995726078	0.999562152
100043200	'Olfr819'	7.04	12.32	1	0	10.5	5.52	6.786666667	5.34	-0.443373937	0.762645993	0.972790461
100043224	'Gm4301'	0	0	1	0	0	0	0.333333333	0	-0.903279821	0.824807108	0.972790461
100043227	'Gm4302'	0	0	0	0	1	0	0	0.333333333	1.020273531	0.802557913	0.972790461
100043247	'Gm4312'	0	0	1	0	0	0	0.333333333	0	-0.903279821	0.824807108	0.972790461
100043254	'Nps'	1	0	0	0	2	0	0.333333333	0.666666667	0.839717162	0.835573887	0.974723675
100043257	'Rbm3-ps'	21.51	18.69	57.56	5.99	30.65	23	32.58666667	19.88	-0.886336816	0.297781998	0.900843503
100043272	'Inafm2'	303	311	415	452	351	367	343	390	0.279381422	0.556123162	0.972790461
100043314	'Tigit'	0	0	0	1	1	0	0	0.666666667	2.050872943	0.610671515	0.972790461
100043324	'Bnip3l-ps'	0	0	1.77	0	0	0	0.59	0	-0.903279821	0.824807108	0.972790461
100043332	'Ankrd66'	0	0	0	2	0	1	0	1	2.714766126	0.495614689	0.972790461

100043381	'Gm14308'	110.92	117.39	16.19	15.19	171.47	36.95	81.5	74.53666667	-0.121835763	0.898153289	0.985837442
100043387	'Gm14305'	101.18	110.37	12.43	58.41	204.79	102.33	74.66	121.8433333	0.774611729	0.320862912	0.917442833
100043403	'Gm14410'	1319	1307.5	431.56	218.48	612.13	412.73	1019.37	414.4466667	-1.254191063	0.013027575	0.285349599
100043456	'Gm10681'	1	0	0	0	0	0	0.333333333	0	-0.903279821	0.824807108	0.972790461
100043461	'Gm4450'	0	2	1	3	0	0	1	1	0.431352611	0.885187127	0.984047342
100043467	'Gm4454'	0	0	0	0	1	0	0	0.333333333	1.020273531	0.802557913	0.972790461
100043468	'Zfp955b'	584.93	598.43	529.6	392.76	589.8	663.43	570.9866667	548.6633333	-0.033223201	0.895122338	0.985469101
100043474	'Olfr1564'	0	1	3	0	0	0	1.333333333	0	-2.933515612	0.459648379	0.972790461
100043508	'Ptges3-ps'	150.66	118.05	213.31	76.44	57.55	86.28	160.6733333	73.42333333	-1.088016993	0.037709241	0.459930654
100043597	'Srcap'	6557	6169	2639	2135	4646	3350	5121.666667	3377	-0.542715519	0.190889277	0.813536636
100043604	'Vmn1r132'	0	0	0	2	0	0	0	0.666666667	2.299096387	0.566358544	0.972790461
100043617	'Gm4553'	0	0	0	2	1	0	0	1	2.732929573	0.492631579	0.972790461
100043627	'Gm4559'	1	2	0	0	0	1	1	0.333333333	-1.323989397	0.695217833	0.972790461
100043629	'Gm10013'	0	0	0	0	1	1	0	0.666666667	1.775692139	0.660844521	0.972790461
100043638	'Gm4565'	0	0	0	0	0.5	1	0	0.5	1.020273531	0.802557913	0.972790461
100043665	'Gm10662'	4	2	0	0	0	0	2	0	-3.282198642	0.379254284	0.942621344
100043695	'Gm15483'	15.42	12.6	1.04	3	9.01	5	9.686666667	5.67	-0.637284424	0.57131045	0.972790461
100043721	'Gm4606'	0	1	0	0	1	0	0.333333333	0.333333333	0.058500858	0.988561293	0.999493374
100043757	'Zfp831'	34	41	2	1	14	15	25.66666667	10	-1.371577455	0.241025265	0.86449675
100043772	'Zfp850'	504	487	224	138	250	268	405	218.6666667	-0.840800381	0.027954619	0.40918746
100043805	'Gm10224'	2.78	2.56	4.01	0	2	1	3.116666667	1	-1.526260972	0.386060296	0.947679581
100043813	'Rps27rt'	0	3.19	10.42	0	0	2.66	4.536666667	0.886666667	-2.925752629	0.238355731	0.864053647
100043899	'R3hdml'	0	0	0	0	1	9	0	3.333333333	4.004067982	0.238056436	0.864053647
100043914	'Zfp968'	5.3	4.13	0	0	5.06	4.77	3.143333333	3.276666667	-0.024978686	0.989350401	0.999493374
100043915	'Gm4724'	2208.1	2217	554.62	1049.4	2619.3	1533.08	1659.883333	1733.926667	0.139663395	0.803435323	0.972790461
100043920	'Fam205a4'	97.8	108.64	31.7	49.23	102.96	57.7	79.38	69.96333333	-0.091319874	0.872409333	0.980171118
100044193	'Gm20939'	200.38	225.65	129.04	131.6	236.42	177.38	185.0233333	181.8	0.030252617	0.930187916	0.99325468
100044509	'Tgfbr3l'	320	306	36	9	487	362	220.6666667	286	0.323168963	0.766714059	0.972790461
100045125	'Gm17768'	1186.7	1216.8	282.25	377.52	1100.6	634.36	895.2633333	704.16	-0.284084229	0.630338239	0.972790461
100045778	'Rnf223'	13	12	13	28	11	7	12.66666667	15.33333333	0.51276435	0.57085432	0.972790461
100045792	'Ect2l'	2	4	0	1	4	10	2	5	1.310038958	0.404349674	0.956545617
100047671	'Gm17783'	0	0	0	0	1	1	0	0.666666667	1.775692139	0.660844521	0.972790461
100048534	'Cfap43'	52	66	25	17	57	54	47.66666667	42.66666667	-0.157078598	0.782276987	0.972790461
100048644	'Pet117'	6.28	6.56	2.15	3.05	0	5.63	4.996666667	2.893333333	-0.676866738	0.641995413	0.972790461
100048658	'Ddx43'	218	209	126	35	74	27	184.3333333	45.33333333	-1.947780968	1.61E-04	0.019471673
100048884	'Mup18'	0	0	0	0	0	3.85	0	1.283333333	2.274429265	0.570697589	0.972790461
100061	'Lrrc19'	10	10	7	6	16	9	9	10.33333333	0.22294405	0.772738451	0.972790461
100072	'Camta1'	1073	1060	2241	523	748	1186	1458	819	-0.899987269	0.087792669	0.642214018
100073351	'Yy2'	608	575	153	40	371	274	445.3333333	228.3333333	-0.997757829	0.180558917	0.801479605
100087	'Kti12'	281	266	287	190	318	349	278	285.6666667	0.041145434	0.893451103	0.985171

100088	'Rcc1'	422	437	405	234	428	377	421.3333333	346.3333333	-0.267921139	0.347830996	0.928444937
100090	'Zbtb48'	293	279	194	270	257	291	255.3333333	272.6666667	0.199242588	0.57395623	0.972790461
100101806	'Srp54c'	1405.1	1276.5	4070.3	804.64	1611.9	2213.91	2250.616667	1543.483333	-0.669850527	0.296584405	0.90029104
100101807	'Fam177a2'	155.14	151.15	308.15	190.99	203.56	304.88	204.8133333	233.1433333	0.163543772	0.755145792	0.972790461
100101919	'Dnah7c'	79.99	79.95	7.1	7.37	60.19	54.28	55.68	40.61333333	-0.455966102	0.632295701	0.972790461
100102	'Pcsk9'	13	13	5	5	19	2	10.33333333	8.666666667	-0.189379475	0.856728551	0.976055348
100113365	'Nlgn4l'	144	190	99	564	349	249	144.3333333	387.3333333	1.646225083	0.004783795	0.163872981
100113398	'Adat3'	63	52	60	60	47	51	58.33333333	52.66666667	-0.045998741	0.925862187	0.992381554
100121	'Tdrd7'	1265	1398	549	358	1039	1194	1070.666667	863.6666667	-0.310844989	0.513832663	0.972790461
100125586	'Vmn2r2'	0	2.09	0	0	1.02	0	0.696666667	0.34	-0.733814424	0.856146772	0.975734242
100126226	'Krt83'	0	1	0	0	1	4	0.333333333	1.666666667	2.065845676	0.501717296	0.972790461
100126824	'Sco2'	34.97	35.67	204.72	16.25	69.01	80.26	91.78666667	55.17333333	-0.947010096	0.305314793	0.907188578
100129	'Gpr153'	546	466	437	444	514	367	483	441.6666667	-0.03163346	0.933994855	0.994249017
100135654	'Rhox3c'	4.96	0	0	0	0	0	1.653333333	0	-2.70473077	0.496508955	0.972790461
100135657	'Rhox3e'	9.01	5.42	0	0	0	0	4.81	0	-4.502527228	0.126484231	0.721271206
100141474	'4933428G20Rik'	118	132	42	185.01	141	47.03	97.33333333	124.3466667	0.614262405	0.407021485	0.957157474
100151772	'Srsx'	1	1	0	0	0	0	0.666666667	0	-1.703500596	0.673820112	0.972790461
100155	'Shoc1'	1000	1058	211	2	444	306	756.3333333	250.6666667	-1.662511898	0.155262012	0.766453805
100163	'Pafah2'	293	319	265	486	443	414	292.3333333	447.6666667	0.727883864	0.061345075	0.562479029
100169	'Phactr4'	995	1054	1055	1080	1032	1056	1034.666667	1056	0.115282893	0.752223106	0.972790461
100169864	'Gm44504'	51.47	96.83	43.11	6.16	27.47	134.64	63.80333333	56.09	-0.300814174	0.744645856	0.972790461
100169868	'Gm3173'	138.29	188.99	176.84	0	7.39	2.7	168.04	3.363333333	-5.842829962	2.44E-14	4.82E-11
100170401	'EU599041'	28.35	17.98	1	0	8	17.09	15.77666667	8.363333333	-0.932350068	0.518694352	0.972790461
100177	'Zmym6'	2826	2774	772	503	2038	1204	2124	1248.333333	-0.743148758	0.209021736	0.836616817
100182	'Akna'	259	243	617	119	263	283	373	221.6666667	-0.849897893	0.14555845	0.753611197
100188919	'Gm45915'	4	3	4	0	2	2	3.666666667	1.333333333	-1.542036152	0.311743683	0.911537035
100189605	'Mup19'	2	0	0	0	0	0	0.666666667	0	-1.711373851	0.67234292	0.972790461
100191037	'Krtap10-4'	0	0	0	2	0	0	0	0.666666667	2.299096387	0.566358544	0.972790461
100198	'H6pd'	908	920	389	4948	1141	2739	739	2942.666667	2.275108022	0.00140048	0.080440873
100201	'Tmem64'	1531	1498	2641	1029	1574	2270	1890	1624.333333	-0.271079132	0.557438551	0.972790461
100206	'Adprhl2'	315	318	474	297	409	486	369	397.3333333	0.097810472	0.809268567	0.972790461
100210	'Gpn2'	122	120	148	303	150	246	130	233	0.970776315	0.075331335	0.60481391
100213	'Rusc2'	345	338	572	525	412	570	418.3333333	502.3333333	0.312525941	0.529086102	0.972790461
100216455	'Gm14124'	30.31	20.3	3.88	13	16	15.01	18.16333333	14.67	-0.128327503	0.882479711	0.982819066
100216474	'Ttll2'	13	9	14	3	1	6	12	3.333333333	-1.830678789	0.068382636	0.583668637
100226	'Stx12'	1942	1905	2435	1235	1674	1906	2094	1605	-0.374032358	0.27194863	0.884539076
100233175	'Gon7'	118.34	151.04	256.54	191.81	118.81	165.71	175.3066667	158.7766667	-0.07331818	0.9013716	0.98672375
100233207	'Gm17359'	0	0	0	1	1	0	0	0.666666667	2.050872943	0.610671515	0.972790461
100233208	'Gm10778'	186.46	178.58	172.23	127.43	170.55	178.05	179.09	158.6766667	-0.133905238	0.660421833	0.972790461
100270744	'Btbd18'	806	778	102	10	310	309	562	209.6666667	-1.475952068	0.145874503	0.753611197

100271704	'Gm17660'	12	14	5	0	22	23	10.33333333	15	0.444875989	0.716448704	0.972790461
100271882	'Gm14139'	18.27	38.37	15.15	4	28	20.99	23.93	17.66333333	-0.485346185	0.535252023	0.972790461
100273	'Osbpl9'	1381.2	1412.3	1844.4	4589.2	1692.8	2109.34	1545.936667	2797.103333	1.054935644	0.102122533	0.671776916
100294583	'Alkal2'	1	0	1	1	0	2.01	0.666666667	1.003333333	0.596872118	0.818185086	0.972790461
100302688	'Gm17455'	0	0	0	1	0	0	0	0.333333333	1.020273531	0.802557913	0.972790461
100303732	'Zfp967'	14.6	8.44	0	2.83	11.69	11.16	7.68	8.56	0.146866043	0.913092528	0.989773583
100303744	'Sprr2a2'	1	4	7.32	0.5	5	1	4.106666667	2.166666667	-1.160422221	0.493556547	0.972790461
100310809	'Gm10509'	166	185	87	133	215	173	146	173.6666667	0.326888658	0.391230792	0.952371379
100310872	'Dynlt1a'	362.45	397.15	317.56	516.41	470.07	365.52	359.0533333	450.6666667	0.45932932	0.280578315	0.889355605
100312471	'Vmn1r3'	1	0	0	0	0	0	0.333333333	0	-0.903279821	0.824807108	0.972790461
100312473	'Vmn1r86'	1	0	0	0	0	0	0.333333333	0	-0.903279821	0.824807108	0.972790461
100312956	'Pate3'	0	0	0	2	1	0	0	1	2.732929573	0.492631579	0.972790461
100317	'AU040320'	805	827.38	312	661.36	937	776	648.1266667	791.4533333	0.396275954	0.352447455	0.930011243
100322896	'Dthd1'	0	0	4	0	0	0	1.333333333	0	-3.00798671	0.447987178	0.970198456
100328588	'Il4i1b'	65.5	74	15	0	30.79	49	51.5	26.59666667	-1.04432486	0.378756901	0.94215965
100336	'Ppp1r8'	1209	1274	921	300	1026	1053	1134.666667	793	-0.56024566	0.188935402	0.811393689
100340	'Smpdl3b'	575	549	63	155	763	768	395.6666667	562	0.514174274	0.516437816	0.972790461
100342	'Tent5b'	87	78	60	213	42	104	75	119.6666667	0.93900653	0.215091969	0.842760284
100383	'Bsdc1'	1086	1071	2196	2989	1167	1622	1451	1926	0.552515723	0.417837139	0.957720764
100384868	'Gm37013'	11.58	11.14	0	0	0	0	7.573333333	0	-5.15125104	0.052053626	0.529628644
100415785	'Gm11559'	0	0	0	0	2.68	2	0	1.56	2.719229454	0.494880768	0.972790461
100416706	'Zfp729b'	645.69	626.63	598.72	250	474	540.36	623.68	421.4533333	-0.572201232	0.053437332	0.533767484
100417514	'Adh6b'	1	2	1	1	1	0	1.333333333	0.666666667	-0.840561797	0.695283857	0.972790461
100434	'Slc44a1'	1368	1354	1323	3845	1800	2498	1348.333333	2714.333333	1.184516603	0.027204214	0.40421521
100463512	'Gm20594'	132	151	193	287	135	204	158.6666667	208.6666667	0.53245038	0.359219727	0.933706718
100465	'Mob3c'	291	301	335	191	351	270	309	270.6666667	-0.181448563	0.60108422	0.972790461
100494	'Zfand2a'	475	553	925	436	492	568	651	498.6666667	-0.379040553	0.424187039	0.960285372
100502590	'Tex50'	0	0	0	1	1	1	0	1	2.526085132	0.463240869	0.972790461
100502680	'Gm10015'	4.16	1.01	0	3	3	2	1.723333333	2.666666667	0.857710689	0.611529886	0.972790461
100502698	'Rubcn'	1164.5	1087.7	1852.2	958.09	1065.2	1177.12	1368.13	1066.803333	-0.340137717	0.44341597	0.969836424
100502723	'Tsga8'	2	6	1	0	60	74	3	44.66666667	3.799690818	0.008982748	0.23172322
100502736	'Gm19345'	2	1	0	3.4	1	4	1	2.8	1.572444391	0.382465241	0.945077873
100502766	'Kifc1'	284.52	324.84	115.42	130.71	313.39	357.05	241.5933333	267.05	0.16799019	0.722033191	0.972790461
100502825	'Rpl37rt'	34.14	6.33	32.87	15.01	34.55	133.36	24.44666667	60.97333333	1.185453076	0.207073476	0.83479753
100502831	'Gm19402'	0	1	0	0	0	0	0.333333333	0	-0.903279821	0.824807108	0.972790461
100502841	'Epg5'	1367	1289	1244	1168	1055	1181	1300	1134.666667	-0.103008789	0.772829964	0.972790461
100502846	'Gm19410'	19	33	4	2	65	11	18.66666667	26	0.458346874	0.698664554	0.972790461
100502861	'Ccdc13'	48	43	11	6	33	34	34	24.33333333	-0.493542602	0.528169592	0.972790461
100502876	'Kcnmb3'	7.29	13.45	10.86	55.05	9.48	13.67	10.53333333	26.06666667	1.671247706	0.100674502	0.671759705
100502880	'Gm19428'	8.77	15.9	0	0	4.33	28.43	8.223333333	10.92	0.409974363	0.821363496	0.972790461

100502936	'E330021D16Rik'	0	0	0	3	0	0	0	1	2.899270347	0.465759809	0.972790461
100502940	'Colca2'	57	67	31	3	58	45	51.66666667	35.33333333	-0.628007185	0.461435419	0.972790461
100502941	'Gm17353'	8.27	13.62	0	0	0	3.11	7.296666667	1.036666667	-2.826040156	0.211215535	0.840335617
100502950	'Gm3336'	0	1	0	2	0	0	0.333333333	0.666666667	1.337350669	0.73871782	0.972790461
100502955	'Gm19470'	2	2	0	0	13	33	1.333333333	15.33333333	3.437031585	0.048029346	0.508993273
100502967	'Speer4c'	61	56.98	26	182.97	116.01	99.02	47.99333333	132.6666667	1.68661173	0.004717003	0.162757993
100503014	'Gm19505'	0	0	0	0	1	0	0	0.333333333	1.020273531	0.802557913	0.972790461
100503041	'Pdzd7'	45	77	19	164.08	112.03	50	47	108.7033333	1.475517733	0.050215898	0.518969204
100503043	'Armcx4'	1471	1603	881	1156	1739	1041	1318.333333	1312	0.093623831	0.810017097	0.972790461
100503068	'Smim43'	11.08	6.03	2	3.39	25.01	6.01	6.37	11.47	0.845038321	0.448224414	0.970242806
100503085	'Klhl3'	239	277	225	31	353	234	247	206	-0.366183445	0.599083882	0.972790461
100503185	'Btbd8'	644	653	137	52	537	395	478	328	-0.575059433	0.466081509	0.972790461
100503240	'Trpc5os'	3	8	6	2	0	5	5.666666667	2.333333333	-1.259342707	0.375330702	0.940406075
100503280	'Gm38425'	123	111	21	3	113	269	85	128.3333333	0.510227586	0.646182142	0.972790461
100503311	'Pifo'	4	1	0	1	1	1	1.666666667	1	-0.584520201	0.775978551	0.972790461
100503353	'Gm14440'	249.44	279.05	87.81	181.57	477.65	315.26	205.4333333	324.8266667	0.714140569	0.159610792	0.771114735
100503355	'Zfp648'	1	5	1	0	2	4	2.333333333	2	-0.284509195	0.873250404	0.980171118
100503361	'Tmem95'	4	3	47	12	4	8	18	8	-1.165792997	0.367904824	0.937190492
100503368	'Gm20599'	0	0	0	0	1	0	0	0.333333333	1.020273531	0.802557913	0.972790461
100503386	'Tpbgl'	88	67	66	128	55	28	73.66666667	70.33333333	0.200465651	0.794107878	0.972790461
100503545	'Nuggc'	33	31	7	1	9	6	23.66666667	5.333333333	-2.138608195	0.024799714	0.389862092
100503549	'Gm10354'	0	0	1	209	0	58.86	0.333333333	89.28666667	8.321973915	0.007871489	0.214208031
100503572	'Bbip1'	233.28	265.23	264.37	386	244.33	276.33	254.2933333	302.22	0.388199595	0.4294693	0.962386546
100503583	'Fsbp'	128.51	140.09	12.09	48	91.27	76.04	93.56333333	71.77	-0.270117136	0.730822344	0.972790461
100503584	'Zfp534'	103.27	101.15	60.49	0	46.35	15.21	88.30333333	20.52	-2.208832833	0.039693155	0.464973418
100503605	'Hbb-bs'	2646.8	2649.9	10485	684.35	8671.3	1668.83	5260.613333	3674.816667	-0.719967015	0.448006829	0.970198456
100503609	'Lnp1'	1	2	0	0	1	0	1	0.333333333	-1.324111362	0.694133627	0.972790461
100503659	'Cbarp'	384.01	418.05	1033.3	528.02	513	746.01	611.7933333	595.6766667	-0.062107534	0.916389689	0.990526893
100503670	'Rpl5'	14949	14586	34297	27990	14019	17925.52	21277.42333	19978.19	0.010567213	0.9872071	0.999493374
100503710	'Gm5741'	0	0	0	0	1	0	0	0.333333333	1.020273531	0.802557913	0.972790461
100503884	'Ccdc149'	76	85	242	31	163	148	134.3333333	114	-0.412816678	0.582691756	0.972790461
100503890	'Pet100'	102	122	60	29	68	97	94.66666667	64.66666667	-0.553557258	0.244343048	0.866091613
100503915	'Smpd5'	51	68	32	9	73	65	50.33333333	49	-0.100582599	0.886258766	0.984348372
100503924	'Fcor'	19	17	15	4	8	9	17	7	-1.269653307	0.069538166	0.587499452
100503949	'Zfp965'	1004.5	1009.3	354.08	176.36	924.27	518.41	789.2933333	539.68	-0.553363286	0.353724865	0.930465111
100503964	'Gm17266'	297	306	61	1	108	31	221.3333333	46.66666667	-2.292846212	0.037441475	0.459930654
100503991	'Acp4'	4.05	3.1	2.04	2.06	1.02	0	3.063333333	1.026666667	-1.357204378	0.429486768	0.962386546
100503992	'Gm6040'	0	0	0	1	0	0	0	0.333333333	1.020273531	0.802557913	0.972790461
100504014	'Gm15056'	1	0	1	0	0	0	0.666666667	0	-1.858475606	0.644939423	0.972790461
100504089	'Gm20056'	6.69	8.16	8.76	18	3	7	7.87	9.333333333	0.587478517	0.580446896	0.972790461

100504112	'Ccer2'	0	1	1	1	0	1	0.666666667	0.666666667	0.098639155	0.971302795	0.999493374
100504116	'Brd3os'	125	119	211	104	144	165	151.6666667	137.6666667	-0.157189211	0.738296541	0.972790461
100504180	'LOC100504180'	6	5.8	0	0	4.54	0	3.933333333	1.513333333	-1.441785643	0.560219367	0.972790461
100504195	'Micalcl'	44	32	63	57	23	46	46.33333333	42	-0.032297181	0.962727544	0.999493374
100504200	'Gm8050'	0	0	0	0	1	0	0	0.333333333	1.020273531	0.802557913	0.972790461
100504221	'Efcab8'	24.95	26.96	10	52.07	29.01	11.59	20.63666667	30.89	0.898365791	0.295065269	0.90029104
100504234	'Ccdc170'	187	171	33	22	222.03	117	130.3333333	120.3433333	-0.130699145	0.873968721	0.980171118
100504263	'2210418O10Rik'	103.62	1.89	100.28	82.51	441.56	414.51	68.59666667	312.86	2.049124662	0.073308394	0.599174173
100504285	'Abhd12b'	4.98	1.65	1	0	4.98	0	2.543333333	1.66	-0.623858212	0.783814928	0.972790461
100504309	'Smim27'	20	29	34	6	26	23	27.66666667	18.33333333	-0.671488154	0.32263191	0.918775094
100504323	'Gm20172'	0	0	0	0	1	1	0	0.666666667	1.775692139	0.660844521	0.972790461
100504429	'Spin2d'	2	0	0	0	2	0	0.666666667	0.666666667	0.031622541	0.993710128	0.999493374
100504446	'Prr22'	4	6	4	4	14	5	4.666666667	7.666666667	0.726749506	0.464878664	0.972790461
100504491	'Dcdc2b'	16	18	10	16	39	16.14	14.66666667	23.71333333	0.74265783	0.264727325	0.88128484
100504518	'3425401B19Rik'	1	2	1	0	0	2	1.333333333	0.666666667	-1.061758588	0.669168024	0.972790461
100504608	'Eef1akmt3'	3	3	32	2	0	1	12.66666667	1	-3.641745528	0.028503245	0.410671038
100504663	'Atg14'	1269	1242	1059	555	1141	1225	1190	973.6666667	-0.295128177	0.30410127	0.906771226
100504689	'Plscr5'	0	1	0	0	1	0	0.333333333	0.333333333	0.058500858	0.988561293	0.999493374
100504715	'Tmppe'	409	379.99	160	204	413	448.49	316.33	355.1633333	0.207367562	0.622210183	0.972790461
100505397	'Gm20385'	12.15	4.95	3.37	7.49	40.22	30.77	6.823333333	26.16	1.988287384	0.027213882	0.40421521
100515	'Zfp518b'	731	808	417	318	780	567	652	555	-0.202592212	0.589517992	0.972790461
100529082	'Gm11127'	10.69	14.62	9.47	11.57	9.06	4.01	11.59333333	8.213333333	-0.300241326	0.729081343	0.972790461
100532	'Rell1'	398	339	798	1279	499	752	511.6666667	843.3333333	0.85134225	0.21824969	0.844387594
100534273	'Fer1l5'	71.93	91.68	18.74	0	41.31	0	60.78333333	13.77	-2.169862727	0.185261599	0.805625233
100534287	'Dchs2'	85	57	50	61	35	20	64	38.66666667	-0.503555347	0.466992597	0.972790461
100534296	'Gm9805'	33	36	18	5	59	24	29	29.33333333	-0.033697718	0.967146976	0.999493374
100535	'Oas1d'	0	0	0	1	2	32	0	11.66666667	5.829309843	0.008960002	0.231450478
100561	'Slc15a4'	521	520	371	703	586	723	470.6666667	670.6666667	0.62645136	0.090401437	0.649074572
100604	'Lrrc8c'	398	340	345	376	625	692	361	564.3333333	0.65425149	0.019898817	0.349283865
100608	'Noc4l'	788	863	451	259	559	887	700.6666667	568.3333333	-0.312468454	0.465633544	0.972790461
100609	'Nsun5'	292.31	324.07	239.14	191.59	286.45	247.17	285.1733333	241.7366667	-0.185487373	0.518852884	0.972790461
100637	'N4bp2l1'	105.81	113.21	254.74	180.67	207.7	181.6	157.92	189.99	0.272681259	0.637409392	0.972790461
100647	'Upk3b'	1922	1769	136	67	1914	2201	1275.666667	1394	0.07973807	0.940680038	0.996019386
100678	'Psph'	295	341	378	147	293	358	338	266	-0.381262413	0.31238355	0.911537035
100683	'Trrap'	7216	6840	2495	4305	8970	6303	5517	6526	0.314955199	0.48657295	0.972790461
100689	'Spon2'	79	62	8	87	98	91	49.66666667	92	1.053992466	0.156668094	0.768046725
100702	'Gbp6'	423.69	355.19	1199.4	51	278.59	558.14	659.43	295.91	-1.377507946	0.094888721	0.661299253
100705	'Acacb'	389	328	92	448	529	830	269.6666667	602.3333333	1.253702156	0.019852466	0.349283865
100710	'Pds5b'	2418	2423	1590	1245	2957	2511	2143.666667	2237.666667	0.072968657	0.819446928	0.972790461
100715	'Tent2'	1039	1085	1623	719	1223	1339	1249	1093.666667	-0.223310244	0.581391759	0.972790461

100727	'Ugt2b34'	0	2	0	0	2	0	0.666666667	0.666666667	0.047401214	0.990575369	0.999493374
100732	'Mapre3'	469	432	365	694	352	418	422	488	0.399085968	0.454460135	0.970649024
100737	'Dcun1d4'	1235	1161	682	1138	1334	1537	1026	1336.333333	0.467123525	0.138609002	0.739818738
100756	'Usp30'	926	956	816	512	945	1235	899.3333333	897.3333333	-0.013308086	0.965141224	0.999493374
100763	'Ube3c'	1725	1755	1417	791	1769	1798	1632.333333	1452.666667	-0.177022391	0.555078505	0.972790461
100764	'Rita1'	142	131	271	568	154	245	181.3333333	322.3333333	1.018480562	0.182909166	0.804092742
100855	'Tbc1d14'	1503	1507	1111	1000	1641	1529	1373.666667	1390	0.058076401	0.80868823	0.972790461
100859931	'Gm20604'	630.59	666.01	426.82	487.03	505.18	473.97	574.4733333	488.7266667	-0.121238416	0.72973942	0.972790461
100861615	'Gm3411'	232.95	233.43	143.29	0	23.04	7.82	203.2233333	10.28666667	-4.41526861	7.44E-07	2.95E-04
100861623	'Rhox3a2'	11.2	13.78	1	0	8.92	20.92	8.66	9.946666667	0.099194303	0.945489561	0.996592403
100861640	'Rhox4a2'	0	1.3	2.71	0	1	4	1.336666667	1.666666667	0.512300119	0.828434589	0.973315932
100861651	'Gm9595'	106.21	130.99	21.15	0	33.82	21.95	86.11666667	18.59	-2.303014786	0.040949767	0.474812891
100861663	'Gm6605'	0	1	0	1	0	2	0.333333333	1	1.543618898	0.640011393	0.972790461
100861668	'Gm21119'	11804	11508	1898.8	1455.9	15828	9018.04	8403.78	8767.286667	0.041911829	0.95933266	0.999493374
100861686	'Gm21136'	0	0	0	0	2	0	0	0.666666667	1.801491674	0.656112935	0.972790461
100861702	'Gm21149'	0	2.17	4.98	137.73	1	19.91	2.383333333	52.88	5.035914572	0.00207549	0.100405117
100861708	'Gm21154'	0	2	0	0	0	1	0.666666667	0.333333333	-0.733814424	0.856146772	0.975734242
100861744	'Gm21181'	2.87	0	1.59	0	0	0.87	1.486666667	0.29	-2.396486725	0.548517172	0.972790461
100861753	'Gm21188'	1	0	0	0	0	0	0.333333333	0	-0.903279821	0.824807108	0.972790461
100861755	'Gm21190'	7	9.99	12.02	39.72	10.48	20.12	9.67	23.44	1.491720048	0.096154553	0.662767142
100861814	'Gm3371'	1	1	1	0	1	0.98	1	0.66	-1.427484097	0.601914152	0.972790461
100862066	'Cfap99'	26.05	23.95	4	4.9	8	7	18	6.633333333	-1.392740598	0.124402972	0.717146542
100862067	'Btbd35f29'	0	0	0	1	0	0	0	0.333333333	1.020273531	0.802557913	0.972790461
100862072	'Gm21451'	217.04	99.39	27.21	732.11	189.04	374.19	114.5466667	431.78	2.232536107	0.008833564	0.230691912
100862085	'Gm16867'	29.73	22.85	25.63	188.76	56.14	98.64	26.07	114.5133333	2.388692501	8.37E-04	0.059826746
100862177	'Ccl21d'	5.59	5.71	0.55	6.19	7.06	10.41	3.95	7.886666667	1.317555769	0.274836798	0.885974997
100862203	'Gm6712'	131	95.98	78.56	94	124	100	101.8466667	106	0.145853094	0.707317974	0.972790461
100862247	'Gm21586'	4.26	5.5	2.61	3	3	4.05	4.123333333	3.35	-0.051222905	0.964572496	0.999493374
100862261	'Fam205a3'	88.52	75.04	31.7	49.23	102.96	57.7	65.08666667	69.96333333	0.179757499	0.734098877	0.972790461
100862314	'Gm21637'	0	0	0	0	0	1	0	0.333333333	1.020273531	0.802557913	0.972790461
100862349	'Gm21663'	2	8.67	13.78	796.2	16.62	229.1	8.15	347.3066667	5.78087972	3.18E-06	9.75E-04
100862359	'Gm21671'	0	1.85	1.14	5.3	6.23	2.87	0.996666667	4.8	2.797248248	0.102183427	0.671776916
100862368	'Gm21680'	70.07	91.91	36.91	292.54	235	106.65	66.29666667	211.3966667	1.908407352	0.00300334	0.122531121
100862375	'Entpd4b'	803.68	758.23	494.63	724.28	1036	1246.07	685.5133333	1002.116667	0.596979467	0.029685643	0.420102506
100862388	'Gm21698'	26.18	18.55	4.02	177.15	37.61	81.92	16.25	98.89333333	2.946587891	0.001154563	0.071037376
100900	'Hscb'	179	203	255	103	176	263	212.3333333	180.6666667	-0.275850373	0.516939118	0.972790461
100910	'Chpf2'	741.17	744.83	345.84	860.54	893.04	948.8	610.6133333	900.7933333	0.684143985	0.082058146	0.622020945
100929	'Tyw1'	618	612	337	305	649	829	522.3333333	594.3333333	0.192614944	0.608100351	0.972790461
100952	'Emilin1'	1725	1692	859	917	1678	1242	1425.333333	1279	-0.088790217	0.803765948	0.972790461
100972	'Rab28'	804	777	767	496	717	791	782.6666667	668	-0.203841864	0.441448562	0.968551476

100978	'Nfxl1'	2445	2344	1849	672	1445	1587	2212.666667	1234.666667	-0.847935583	0.004746945	0.163012598
100986	'Akap9'	2818	2935	1711.2	1214	3796	2846	2488.063333	2618.666667	0.070348421	0.860354621	0.976863639
101023	'Zfp513'	517	562	430	362	538	466	503	455.3333333	-0.089704348	0.741420255	0.972790461
101055670	'Heatr4'	15	22	1	2	24	15	12.66666667	13.66666667	0.108919354	0.925942632	0.992381554
101055671	'Lipo2'	36.77	43.64	7.02	0	27.87	8.6	29.14333333	12.15666667	-1.335817615	0.282658353	0.890750971
101055672	'LOC101055672'	0	1	1.01	0	0	3.34	0.67	1.113333333	0.382584832	0.905851195	0.988794924
101055676	'LOC101055676'	289.94	227.7	46.61	20.42	62.28	31.35	188.0833333	38.01666667	-2.24135638	0.00150293	0.084039761
101055745	'Gm29721'	3.89	3.93	0	0	0	2	2.606666667	0.666666667	-1.576822352	0.572790171	0.972790461
101055754	'Gm2974'	7.1	2.82	9.68	1	2.11	2.14	6.533333333	1.75	-1.891199893	0.149557258	0.758505335
101055758	'Gm7592'	1	2	1	0	1	2	1.333333333	1	-0.487342322	0.81139465	0.972790461
101055806	'Gm8032'	0	0	0	1	1	0	0	0.666666667	2.050872943	0.610671515	0.972790461
101055864	'Gm4513'	0	0	0	0	0.5	1	0	0.5	1.020273531	0.802557913	0.972790461
101055907	'Gm15246'	2657	2674	651	133	1713	1420	1994	1088.666667	-0.921994056	0.251367226	0.871230154
101055909	'Gm10320'	30.73	43.05	305.53	0	0	0	126.4366667	0	-9.524053958	1.03E-08	7.83E-06
101055915	'Gm14438'	0	0	0	1.13	0	0	0	0.376666667	1.020273531	0.802557913	0.972790461
101055939	'Kif19b'	3	2	2	1	0	1	2.333333333	0.666666667	-1.694732338	0.369726509	0.937712122
101055956	'Gm29753'	5	3	9	5	1	1	5.666666667	2.333333333	-1.094572004	0.42306489	0.95993671
101055983	'Gm14569'	8	2	0	0	10	13	3.333333333	7.666666667	1.147221662	0.52621499	0.972790461
101055987	'Gm29758'	31.45	36.48	31.23	1.45	16.15	12.61	33.05333333	10.07	-1.840660677	0.021188604	0.361289452
101056017	'Gm28802'	1	1	11	0	4	0	4.333333333	1.333333333	-1.9407087	0.369617096	0.937712122
101056029	'Gm7206'	0	1.13	3.02	1.55	0	1.07	1.383333333	0.873333333	-0.982765013	0.69527929	0.972790461
101056047	'Gm28455'	40.59	23.89	12.56	6	39	19	25.68	21.33333333	-0.239562664	0.761348514	0.972790461
101056061	'Gm29770'	0	0	1	0	0	0	0.333333333	0	-0.903279821	0.824807108	0.972790461
101056073	'Zfp990'	606.98	662.16	351.33	1.96	33.88	29.01	540.1566667	21.61666667	-4.76816373	7.25E-10	7.26E-07
101056084	'Gm29776'	11	11	0	0	13	3	7.333333333	5.333333333	-0.469445638	0.794431844	0.972790461
101056136	'Gm17026'	2.58	3.17	0	0	0	0	1.916666667	0	-3.014977875	0.444538231	0.969888068
101056159	'Gm8104'	5.07	3.59	0	0	0	0	2.886666667	0	-3.69598494	0.279393706	0.889355605
101056205	'Gm29797'	6	10	4	13	9	4	6.666666667	8.666666667	0.588689518	0.549940858	0.972790461
101056240	'Gm13430'	9.41	10.43	23.28	11.26	14.29	12.32	14.37333333	12.62333333	-0.188491528	0.812448379	0.972790461
101056241	'Gm29808'	1	1	0	2	1	3	0.666666667	2	1.713163839	0.388087485	0.950023663
101095	'Zfp282'	867	922	758	206	946	877	849	676.3333333	-0.397882973	0.421946446	0.959445304
101100	'Ttll3'	110.46	88.29	49.23	34.18	94.14	54.08	82.66	60.8	-0.405197687	0.416058918	0.95722888
101113	'Snx21'	359.8	332.62	939.34	1009.7	378	574.4	543.92	654.0466667	0.384443448	0.605422175	0.972790461
101118	'Tmem168'	1007	1023	586	857	1328	1496	872	1227	0.542185054	0.061948304	0.564873458
101122	'Rpusd3'	43	47	31	20	88	91	40.33333333	66.33333333	0.666777569	0.241356529	0.86449675
101142	'Itfg2'	471.34	427.15	548.2	191.64	356.91	290.6	482.23	279.7166667	-0.791310026	0.027677105	0.407338487
101148	'Bmt2'	1365	1273	2655	782	995	1172	1764.333333	983	-0.866955776	0.080182413	0.617305615
101185	'Pot1a'	557	589	591	214	558	551	579	441	-0.431956122	0.242461495	0.86449675
101187	'Parp11'	432.04	475.53	196.23	135.82	567	477.47	367.9333333	393.43	0.08020954	0.878570539	0.981388029
101197	'Zfp956'	331	350	162	107	485	308	281	300	0.081059832	0.878007308	0.981341203

101202	'Hepacam2'	42	37	11	0	29	14	30	14.33333333	-1.127958817	0.329588593	0.921648675
101206	'Tada3'	560	648	746	264	577	687	651.3333333	509.3333333	-0.402949408	0.303062595	0.906189263
101214	'Tra2a'	2608	2650	3596	1143	3038	1567	2951.333333	1916	-0.650928577	0.156591021	0.768046725
101240	'Wdr91'	358	315	339	706	422	448	337.3333333	525.3333333	0.796552631	0.117794318	0.706468633
101314	'Brk1'	1439	1585	1975	1324	1281	1576	1666.333333	1393.666667	-0.209153608	0.595035979	0.972790461
101320	'Dyrk4'	1	1	2	0	2	0	1.333333333	0.666666667	-1.088957393	0.659967391	0.972790461
101351	'Eogt'	234	282	128	70	473	288	214.6666667	277	0.327124626	0.593127509	0.972790461
101358	'Fbxl14'	602	553	754	803	579	1022	636.3333333	801.3333333	0.389112133	0.387651382	0.949501169
101359	'Prrt4'	95	81	13	12	227	154	63	131	1.016850158	0.285584897	0.893820325
101401	'Adamts9'	1408	1340	2508	3717	1905	2221	1752	2614.333333	0.703974388	0.25218002	0.871252558
101434	'Ceacam15'	74	37	59	0	4	0	56.66666667	1.333333333	-5.438280198	5.75E-07	2.38E-04
101437	'Dhx32'	673.02	656	816.8	497.27	757	942.54	715.2733333	732.27	0.023812356	0.944395019	0.996592403
101471	'Phrf1'	2022.7	2014.1	1873	1678	1894	1877.89	1969.933333	1816.63	-0.045459744	0.881299985	0.982528614
101476	'Plekha1'	1168	1252	965	1128	1181	975	1128.333333	1094.666667	0.065058085	0.856717658	0.976055348
101488	'Slco2b1'	18	18	0	3	15	23	12	13.66666667	0.196058319	0.879527357	0.981807495
101488143	'Hbb-bt'	490.18	515.07	1647.9	242.65	2161.7	454.17	884.3866667	952.85	-0.064443157	0.941128837	0.996040262
101489	'Ric8a'	732	708	1641	636	835	980	1027	817	-0.36716194	0.49222417	0.972790461
101490	'Inpp5f'	1504	1618	528	880	1235	1045	1216.666667	1053.333333	-0.093917417	0.836584751	0.974723675
101497	'Plekhg2'	1537	1582	1062	1664	1728	1159	1393.666667	1517	0.259122741	0.535172561	0.972790461
101502	'Hsd3b7'	241	278	316	71	335	418	278.3333333	274.6666667	-0.131810588	0.8186603	0.972790461
101513	'Mob2'	478	412	319	386	396	501	403	427.6666667	0.166179669	0.60256869	0.972790461
101533	'Klk9'	3	4	1	0	0	0	2.666666667	0	-3.738974899	0.151214634	0.759950471
101540	'Prkd2'	344	313	407	383	402	347	354.6666667	377.3333333	0.159933343	0.698976507	0.972790461
101543	'Wtip'	672	689	958	473	584	718	773	591.6666667	-0.378294061	0.327108474	0.920734892
101544	'Zfp575'	42.41	39.64	25.34	21.49	27.04	21.01	35.79666667	23.18	-0.533631671	0.294467657	0.90029104
101565	'Ccp110'	1955	1946	438	409	1183	940	1446.333333	844	-0.730152488	0.212318762	0.84083615
101568	'Vrk3'	669	685	572	357	635	873	642	621.6666667	-0.054142421	0.861974774	0.976863639
101592	'Efl1'	638	585	972	751	679	835	731.6666667	755	0.087683916	0.849151383	0.975734242
101602	'AI467606'	80	61	34	162	82	76	58.33333333	106.6666667	1.109176001	0.085439163	0.634617076
101604	'E430018J23Rik'	235	206	207	119	221	281	216	207	-0.073803744	0.826494011	0.972859382
101612	'Grwd1'	542	498	460	187	465	547	500	399.6666667	-0.357783016	0.331097126	0.921648675
101613	'Nlrp6'	0	0	0	1	1	2	0	1.333333333	2.883196101	0.363428541	0.935022643
101631	'Pwwp2b'	241	277	258	221	340	352	258.6666667	304.3333333	0.255459946	0.382918776	0.945352503
101685	'Spty2d1'	979	950	2966	1629	957	1215	1631.666667	1267	-0.311171929	0.656251366	0.972790461
101700	'Trim68'	245	240	150	53	353	340	211.6666667	248.6666667	0.160759052	0.791086393	0.972790461
101706	'Numa1'	5399	5010	3522	2188	5444	4613	4643.666667	4081.666667	-0.182284184	0.578586317	0.972790461
101739	'Psip1'	3834.6	4003.1	3413.1	2469.1	3916.8	4928.38	3750.253333	3771.43	0.018072881	0.944037583	0.996592403
101744	'Acp7'	8	5	1	1	7	3	4.666666667	3.666666667	-0.323509535	0.804088683	0.972790461
101772	'Ano1'	1991	1927	215	474	2734	2991	1377.666667	2066.333333	0.579704069	0.477303723	0.972790461
101809	'Spred3'	185	196	311	343	220	196	230.6666667	253	0.256096326	0.662362571	0.972790461

101831	'Faap24'	118	140	88	43	85	144	115.3333333	90.66666667	-0.362940694	0.414151436	0.95722888
101835	'AW146154'	205.22	253.13	117.19	122.3	278.77	142.3	191.8466667	181.1233333	-0.02380939	0.958280987	0.999493374
101861	'Ints4'	1580	1592	1126	878	1773	1772	1432.666667	1474.333333	0.054674134	0.843072938	0.975182082
101867	'Rrp8'	522	499	770	154	572	644	597	456.6666667	-0.496388833	0.352201315	0.929748874
101869	'Unc45a'	648	618	606	1259	780	830	624	956.3333333	0.772070675	0.113496128	0.698948431
101883	'Igflr1'	29	40	30	21	44	15	33	26.66666667	-0.252477638	0.684604724	0.972790461
101943	'Sf3b3'	6604	6391	4643	3515	5191	5116	5879.333333	4607.333333	-0.298941944	0.205881431	0.833294914
101966	'D8Ertd738e'	655.69	722.15	1087.3	820.3	684.51	898.72	821.7233333	801.1766667	0.012615456	0.978567834	0.999493374
101985	'Usb1'	416.56	433.7	610.49	285.1	400.35	406.08	486.9166667	363.8433333	-0.414743775	0.289359627	0.896416038
101994	'Champ1'	1746	1760	1272	572	1616	1708	1592.666667	1298.666667	-0.321552487	0.392211703	0.952648938
102032	'Smim19'	408	415	305	20	345	437	376	267.3333333	-0.617765112	0.447380821	0.970198456
102058	'Exoc8'	614	614	273	455	533	566	500.3333333	518	0.156761074	0.685660086	0.972790461
102060	'Gadd45gip1'	338.73	317.35	429.05	276.91	417.43	530.29	361.71	408.21	0.158591427	0.668345876	0.972790461
102075	'Plekhg4'	362	343	20	28	161	250	241.6666667	146.3333333	-0.734607529	0.443639206	0.969836424
102093	'Phkb'	1457	1530	707	416	1803	1419	1231.333333	1212.666667	-0.042709235	0.933021578	0.994031155
102098	'Arhgef18'	1113	1174	815	2381	1416	1371	1034	1722.666667	0.929370448	0.068429434	0.583668637
102103	'Mtus1'	520	466	755	1141	926	1324	580.3333333	1130.333333	1.009304979	0.029799236	0.42090867
102115	'Dohh'	382	444	620	381	425	478	482	428	-0.146842151	0.728659634	0.972790461
102122	'Psme3ip1'	964	933	1211	652	865	1049	1036	855.3333333	-0.269865559	0.439422741	0.967427574
102124	'Enkd1'	250	231	151	115	168	213	210.6666667	165.3333333	-0.307912814	0.331314035	0.921648675
102141	'Snx25'	882	860	800	632	618	820	847.3333333	690	-0.229729222	0.473068489	0.972790461
102162	'Taf5l'	837	805	1045	437	797	1213	895.6666667	815.6666667	-0.185837261	0.647501585	0.972790461
102182	'Prmt9'	1086	1101	452	277	878	784	879.6666667	646.3333333	-0.438476251	0.352366414	0.930011243
102193	'Zdhhc7'	489	515	306	478	519	566	436.6666667	521	0.35158987	0.283204829	0.891393415
102209	'Snapc2'	693	756	925	445	695	794	791.3333333	644.6666667	-0.304916059	0.382091003	0.94434907
102216272	'Ak6'	212.49	191.28	759.35	197.56	177.18	230	387.7066667	201.58	-0.975101645	0.172479564	0.790153935
102247	'Gpat4'	1728	1663	2092	1213	1960	1943	1827.666667	1705.333333	-0.100253458	0.761525212	0.972790461
102278	'Cpne7'	6	7	4	87	3	9	5.666666667	33	2.980170171	0.021372673	0.362154416
102294	'Cyp4v3'	202	193	114	61	259	248	169.6666667	189.3333333	0.119746807	0.814471662	0.972790461
102323	'Dcun1d2'	422.47	457	281	524	513	431.72	386.8233333	489.5733333	0.471320138	0.233606874	0.860393388
102334	'Ankrd10'	2218.4	2234	1510	3227	2931	1714	1987.453333	2624	0.568633134	0.242357213	0.86449675
102339	'Cog4'	1888	1817	1281	1212	1523	1756	1662	1497	-0.087072921	0.737495624	0.972790461
102371	'Myzap'	164	158	81	179	252	289	134.3333333	240	0.903309235	0.01087513	0.257161661
102414	'Clk3'	1039.9	1012	1905.1	1233.4	1147.7	1093.77	1319.016667	1158.29	-0.139601336	0.786526301	0.972790461
102423	'Hinfp'	1050	1032	929	958	813	717	1003.666667	829.3333333	-0.147849598	0.721706072	0.972790461
102436	'Lars2'	495	480	261	232	521	533	412	428.6666667	0.075026385	0.836347625	0.974723675
102442	'Dennd4a'	898	860	1346	1235	695	704	1034.666667	878	-0.098274555	0.865207028	0.978040999
102443350	'Xndc1'	667	666	226	138	570.19	449	519.6666667	385.73	-0.430147038	0.441037855	0.968551476
102443351	'Xntrpc'	0	0	0	0	24.23	0	0	8.076666667	5.327410298	0.142845075	0.749054337
102448	'Xylb'	146	139	280	260	159	213	188.3333333	210.6666667	0.239142278	0.687929453	0.972790461

102462	'Imp3'	344	349	1196	862	363	570	629.6666667	598.3333333	0.003376122	0.996510192	0.999616639
102502	'Pls1'	209	206	54	44	122	293	156.3333333	153	-0.054838757	0.937793503	0.994698142
102545	'Cmtm7'	209	212	286	206	296	351	235.6666667	284.3333333	0.265225253	0.484992885	0.972790461
102566	'Ano10'	474	506	305	312	661	790	428.3333333	587.6666667	0.460396073	0.178017975	0.797422443
102570	'Slc22a13'	7	6	4	9	10	16	5.666666667	11.66666667	1.092221727	0.172632312	0.790483026
102577427	'Esp5'	0	1	0	0	0	2	0.333333333	0.666666667	0.78767502	0.845746136	0.975182082
102580	'Alg9'	372	387	584	146	522	550	447.6666667	406	-0.245137063	0.639959717	0.972790461
102595	'Plekho2'	375	387	652	772	511	624	471.3333333	635.6666667	0.51767188	0.339828812	0.92508569
102607	'Snx19'	1034	1017	276	317	1313	1093	775.6666667	907.6666667	0.232624959	0.697918167	0.972790461
102614	'Rpp25'	461	490	605	208	660	585	518.6666667	484.3333333	-0.167212538	0.707622066	0.972790461
102626	'Mapkapk3'	66	63	150	333	145	254	93	244	1.483383577	0.030878223	0.426950382
102631503	'Gm29825'	1	1	1	0	6	2.41	1	2.803333333	1.311858152	0.492437628	0.972790461
102631705	'Gm29975'	3	4	5	12	5	3	4	6.666666667	0.940925263	0.411692288	0.95722888
102631730	'4930512M02Rik'	15.2	16.82	3	4.04	12.19	12.01	11.67333333	9.413333333	-0.241979713	0.787658225	0.972790461
102631805	'Gm272'	21	19	2	2	3	11	14	5.333333333	-1.359872526	0.229264067	0.855596006
102631912	'Ndufb1'	179	193	323	214	146	246	231.6666667	202	-0.151236326	0.777680166	0.972790461
102631940	'Gm13149'	1	1	1.46	0	4.61	0	1.153333333	1.536666667	0.327675531	0.894927201	0.985422597
102631952	'Gm5928'	3	3	0	7	0	1	2	2.666666667	0.889824398	0.676677839	0.972790461
102631956	'Gm30155'	0	1	0	0	0	0	0.333333333	0	-0.903279821	0.824807108	0.972790461
102632	'Acad11'	843	874	393	369	690	733	703.3333333	597.3333333	-0.19113216	0.606349543	0.972790461
102632113	'Gm3727'	1	0	0	0	0	0	0.333333333	0	-0.903279821	0.824807108	0.972790461
102632142	'Gm18905'	4	4	0	1	2	0	2.666666667	1	-1.228597515	0.56688963	0.972790461
102632152	'Gm30302'	38	6	1	0	9	48	15	19	0.259187699	0.872515479	0.980171118
102632224	'Gm8062'	1	2.25	0	1	0	0.97	1.083333333	0.656666667	-1.32519453	0.684707949	0.972790461
102632353	'Gm30447'	2	2	0	2	0	1	1.333333333	1	-0.094645024	0.967892239	0.999493374
102632403	'Rpl17-ps8'	3.02	2.2	6.26	5.49	4.24	10.5	3.826666667	6.743333333	0.764618106	0.487264	0.972790461
102632427	'Gm30502'	5.03	6.05	7.06	8	9	4	6.046666667	7	0.311931282	0.741482698	0.972790461
102632483	'Gm17330'	489.36	499.94	493.61	131.75	426.41	383.42	494.3033333	313.86	-0.707264942	0.089074137	0.645388896
102632554	'Gm30599'	0	1	0	0	1	0	0.333333333	0.333333333	0.058500858	0.988561293	0.999493374
102632660	'Gm30679'	2	8.5	4	0	1.06	1.03	4.833333333	0.696666667	-2.826633239	0.09917571	0.668798985
102632693	'Gm30698'	11.08	11	6.04	6	9.04	12	9.373333333	9.013333333	-0.011023791	0.988480898	0.999493374
102632737	'Gm30732'	2	1	4	38	6	2	2.333333333	15.33333333	3.034210179	0.033683215	0.442866718
102632738	'Gm30733'	1	0	0	0	1	1	0.333333333	0.666666667	0.817432597	0.823901819	0.972790461
102632837	'Rnf212b'	381.42	388.92	70.91	1	153	223	280.4166667	125.6666667	-1.233258836	0.292060301	0.89958198
102633050	'Gm6293'	0	0	1	0	0	2	0.333333333	0.666666667	0.78767502	0.845746136	0.975182082
102633075	'Gm30990'	24.54	28.17	5.12	39.89	15.21	14.29	19.27666667	23.13	0.559982276	0.54094457	0.972790461
102633131	'Gm31035'	1	0	1	0	0	0	0.666666667	0	-1.858475606	0.644939423	0.972790461
102633156	'Gm4631'	68.33	14.08	0	43.61	69.72	40.71	27.47	51.34666667	1.055020402	0.396875357	0.954358954
102633230	'Gm11633'	2	3	2	3	6	6	2.333333333	5	1.114678953	0.332940126	0.921648675
102633301	'Gm31160'	7	10	4	0	1	0	7	0.333333333	-4.191313514	0.01605173	0.315916649

102633424	'Gm31255'	29.04	26.73	25.53	156.51	317.77	171.22	27.1	215.1666667	3.045548973	1.45E-11	1.97E-08
102633458	'Gm11100'	1.01	0	6.05	2.07	1.01	2.01	2.353333333	1.696666667	-0.530453367	0.777621524	0.972790461
102633525	'Gm31332'	6	9	5	7	5	6	6.666666667	6	-0.035506614	0.968973644	0.999493374
102633750	'Gm10130'	234.01	241.24	188.76	115.11	210.78	219.59	221.3366667	181.8266667	-0.268702502	0.350784082	0.92886351
102633763	'Gm31513'	41.65	49.22	51.15	58.95	27.15	23.8	47.34	36.63333333	-0.18968951	0.785672653	0.972790461
102633782	'Gm31526'	75	64.94	21.96	18	44.58	42.01	53.96666667	34.86333333	-0.578297609	0.328384148	0.921397778
102633809	'Gm3785'	9	4	16	1	5	6	9.666666667	4	-1.398827141	0.203979244	0.832200296
102633888	'Gm31606'	16.71	21.74	7.53	11.82	9.21	7.04	15.32666667	9.356666667	-0.542759069	0.495701529	0.972790461
102633951	'Gm31649'	20.05	11.59	2.02	2.02	9.3	8.24	11.22	6.52	-0.768718997	0.47208274	0.972790461
102633968	'Gm31665'	1	0	0	0	0	1	0.333333333	0.333333333	0.058500858	0.988561293	0.999493374
102634030	'Gm31714'	11.88	9.97	4.62	0	10.79	37.96	8.823333333	16.25	0.850526334	0.523022911	0.972790461
102634078	'LOC102634078'	86.42	82.97	24.53	110.11	116.82	57.23	64.64	94.72	0.75374834	0.244349359	0.866091613
102634296	'Gm4779'	1418.8	1373.5	314.74	11	728	1223.63	1035.66	654.21	-0.749785572	0.492902412	0.972790461
102634304	'Gm16506'	0.94	1	1.94	0	1	1	1.293333333	0.666666667	-0.106831332	0.969373652	0.999493374
102634333	'1110002E22Rik'	9	3	16	60	0	7	9.333333333	22.33333333	1.596990416	0.315568185	0.914723408
102634429	'Gm6569'	0	0	0	0	0	1	0	0.333333333	1.020273531	0.802557913	0.972790461
102634451	'Ndufab1-ps'	0	0	0	0	0	1.03	0	0.343333333	1.020273531	0.802557913	0.972790461
102634532	'Gm3642'	7.57	10.06	1.07	0	0	0	6.233333333	0	-4.883340624	0.025896165	0.397046689
102634715	'Gm32234'	86	77.87	8.26	31	48	7	57.37666667	28.66666667	-0.779508415	0.429638187	0.962386546
102634841	'Gm38469'	21	26	3	8	14	18	16.66666667	13.33333333	-0.24282088	0.779486927	0.972790461
102634987	'Gm32436'	0	0	1	0	0	0	0.333333333	0	-0.903279821	0.824807108	0.972790461
102635067	'Gm7954'	0.92	2.06	0	0	0	0	0.993333333	0	-1.695595436	0.675304306	0.972790461
102635068	'Gm24392'	11.85	12.04	5.16	0	4.12	1.06	9.683333333	1.726666667	-2.496068243	0.055651551	0.541754883
102635181	'Gm32584'	3	6	0	6	6	10	3	7.333333333	1.412713271	0.260992398	0.878842185
102635315	'Gm32687'	170.66	96.95	65.7	74.45	234.34	210.37	111.1033333	173.0533333	0.641960613	0.208459997	0.836479837
102635357	'Gm32717'	0	0	0	0.98	2.64	2.98	0	2.2	2.719229454	0.494880768	0.972790461
102635360	'Gm32719'	214.28	273.16	270.54	79	161.39	151.56	252.66	130.65	-0.965237501	0.007896466	0.214208031
102635385	'Gm32742'	6	5	0	0	13	27	3.666666667	13.33333333	1.798556809	0.287459158	0.894756633
102635496	'Malrd1'	26	22	5	0	16	16	17.66666667	10.66666667	-0.790167624	0.510182907	0.972790461
102635552	'Gm32856'	118.53	115.35	80.96	108.4	165.6	99.62	104.9466667	124.54	0.331999446	0.414771365	0.95722888
102635566	'Gm17434'	24	21	4.67	0	5	7	16.55666667	4	-2.061468942	0.081221624	0.619657106
102635595	'Gm32886'	0	1	0	0	0	0	0.333333333	0	-0.903279821	0.824807108	0.972790461
102635744	'Gm28729'	1	0	0	0	2	3	0.333333333	1.666666667	2.081200529	0.486776433	0.972790461
102635802	'Gm33049'	11.31	5.15	1.02	1.05	1.02	4.1	5.826666667	2.056666667	-1.448253762	0.308120821	0.907963267
102635873	'Gm33099'	0	0	0	1	0	0	0	0.333333333	1.020273531	0.802557913	0.972790461
102635879	'4933407O12Rik'	10	3	5	0	6	1	6	2.333333333	-1.432763189	0.337917657	0.924020038
102635944	'Gm33153'	10.07	4.1	34.28	18.36	7.04	9.11	16.15	11.50333333	-0.421860401	0.698998104	0.972790461
102635990	'LOC102635990'	11	9	0	3	7	6	6.666666667	5.333333333	-0.228417044	0.862480697	0.977161807
102636051	'Tmem274'	0	1	0	0	0	0	0.333333333	0	-0.903279821	0.824807108	0.972790461
102636082	'Gm8251'	6	10	1	120	28	10	5.666666667	52.66666667	3.648215972	0.002035341	0.099648902

102636110	'Zfp640'	14.41	11	2	0	16.54	48.7	9.136666667	21.74666667	1.155209652	0.406390241	0.957157474
102636203	'4930519L02Rik'	5.97	2.06	0	1	4	2.04	2.676666667	2.346666667	0.065787658	0.969099929	0.999493374
102636475	'Gm33532'	0	0	0	0	1	0	0	0.333333333	1.020273531	0.802557913	0.972790461
102636661	'Gm33666'	0	0	2	0	0	0	0.666666667	0	-1.995285526	0.619817042	0.972790461
102636701	'Gm33700'	0	0	0	1	0	0	0	0.333333333	1.020273531	0.802557913	0.972790461
102636878	'Gm33828'	0	1	0	1	0	0	0.333333333	0.333333333	0.058500858	0.988561293	0.999493374
102636907	'Gm4275'	12	8	3	1	8	1	7.666666667	3.333333333	-1.173530363	0.353440657	0.930050349
102636909	'Gm33851'	7.43	16.22	6.5	13.31	5.22	6.02	10.05	8.183333333	-0.049290834	0.959165831	0.999493374
102636931	'Gm33869'	126	149	15	98	124	26	96.66666667	82.66666667	0.009458373	0.991637874	0.999493374
102637020	'Gm33933'	53.79	51.23	42.09	0	19.4	4.19	49.03666667	7.863333333	-2.755386589	0.007326342	0.204235208
102637087	'Gm33989'	1428	1472	590	1391	2423	1391	1163.333333	1735	0.678570242	0.131699076	0.72897159
102637129	'Gm12854'	55.38	59.97	36.1	84.9	54.07	36.3	50.48333333	58.42333333	0.421148645	0.500797895	0.972790461
102637189	'Gm34066'	0	0	1	1	0	0	0.333333333	0.333333333	0.058500858	0.988561293	0.999493374
102637366	'Gm10037'	18	21	1	25	62	23	13.33333333	36.66666667	1.579749739	0.094996694	0.661529207
102637505	'Gm34296'	7	5	0	0	9	7	4	5.333333333	0.381455246	0.82914053	0.973678583
102637507	'Gm34298'	3	1	0	0	0	0	1.333333333	0	-2.70051453	0.497202575	0.972790461
102637572	'Gm38499'	17	37	11	8	18	21	21.66666667	15.66666667	-0.437253204	0.533991169	0.972790461
102637593	'Gm34362'	10	17	6	6	8	5	11	6.333333333	-0.689910722	0.417957563	0.957720764
102637705	'Ly6l'	0	0	0	1	0	0	0	0.333333333	1.020273531	0.802557913	0.972790461
102637808	'Gm21976'	1	0	1	1	1	1	0.666666667	1	0.617211605	0.784141323	0.972790461
102637817	'Gm34531'	0	4	0	1	0	0	1.333333333	0.333333333	-1.726022703	0.664399597	0.972790461
102637839	'Gm10476'	39.32	41.31	23.28	4.6	20.27	12.37	34.63666667	12.41333333	-1.522673049	0.021606573	0.364161986
102637897	'Gm34593'	0	0	0	1	0	0	0	0.333333333	1.020273531	0.802557913	0.972790461
102637899	'Gm34595'	1	3.13	0	9.78	3	2	1.376666667	4.926666667	2.143423315	0.192974091	0.816019443
102637973	'Gm34653'	2	5	0	0	2	0	2.333333333	0.666666667	-1.746909763	0.525848022	0.972790461
102638047	'LOC102638047'	1.25	0	1.34	20.73	3	14.99	0.863333333	12.90666667	4.391732671	0.005070441	0.16781708
102638083	'Ccdc188'	0	0	0	1	0	0	0	0.333333333	1.020273531	0.802557913	0.972790461
102638268	'1700014D04Rik'	3	2	4	4	2	1	3	2.333333333	-0.210329915	0.879686228	0.981807495
102638382	'Gm34962'	30.83	30	16	12	21	12	25.61	15	-0.679734702	0.263115851	0.88013169
102638498	'Gm15732'	1	0	0	0	2	0	0.333333333	0.666666667	0.839717162	0.835573887	0.974723675
102638514	'Gm35060'	3	1	0	9	0	0	1.333333333	3	1.725883101	0.517300837	0.972790461
102638541	'Gm35078'	0	1	2	0	0	0	1	0	-2.490290069	0.532417892	0.972790461
102638546	'Gm35083'	0	0	2	5	0	0	0.666666667	1.666666667	1.596035935	0.656413514	0.972790461
102638555	'Gm38510'	0	0	0	0	0	2	0	0.666666667	1.749449488	0.66566965	0.972790461
102638674	'Gm14428'	1	0	0	2	3	1	0.333333333	2	2.571442575	0.267540412	0.883600995
102638837	'Tle7'	7	1	5	0	0	3	4.333333333	1	-2.251276245	0.253601539	0.872664697
102638847	'Gm35315'	85.04	86.94	34.81	22	61.02	88.98	68.93	57.33333333	-0.266763803	0.637448245	0.972790461
102638882	'Gm35339'	54	45	32	75	64	29	43.66666667	56	0.54463315	0.392895031	0.952648938
102638888	'Gm20346'	21	22	37.96	61	42.19	64	26.98666667	55.73	1.120991574	0.063370455	0.567231213
102638918	'Gm35364'	4.23	5	1	0	5.97	3	3.41	2.99	-0.358068816	0.818347039	0.972790461

102639021	'Gm3993'	38.08	37.57	17.54	7.59	19.32	24.13	31.06333333	17.01333333	-0.871865191	0.160250535	0.771114735
102639170	'Cdv3-ps'	0	0	0	0	1.05	1.14	0	0.73	1.775692139	0.660844521	0.972790461
102639178	'Gm35549'	0	0	1.05	1.56	0	0	0.35	0.52	0.058500858	0.988561293	0.999493374
102639229	'Gm35588'	84	49	8	1	42	38	47	27	-0.848493879	0.44726319	0.970198456
102639543	'Ifi206'	0	2	2	0	0	0	1.333333333	0	-2.855525825	0.472040045	0.972790461
102639577	'Gm35857'	4	1	0	4	0	1	1.666666667	1.666666667	0.414498339	0.855930842	0.975734242
102639598	'Gm14296'	324.74	456.28	70.15	82.51	0	3.65	283.7233333	28.72	-2.776970457	0.06590605	0.574593252
102639650	'Vamp9'	57	63	9	1	33	11	43	15	-1.539796618	0.152910406	0.764029604
102639653	'Gm2007'	482.56	478.26	173.1	147.99	451.67	344.72	377.9733333	314.7933333	-0.241304764	0.628036714	0.972790461
102639700	'Gm21297'	223.48	228.49	217.89	62.06	208.76	159.67	223.2866667	143.4966667	-0.678795412	0.112749018	0.698666814
102639702	'Gm35953'	55	44	55	0	7	1	51.33333333	2.666666667	-4.325827273	6.28E-06	0.001706877
102639713	'Gm14421'	131.38	139.69	36.31	65.85	156.81	109.28	102.46	110.6466667	0.176143449	0.755094568	0.972790461
102639802	'Gm36028'	20	19	4	7	11	0	14.33333333	6	-1.051428732	0.416031323	0.95722888
102639828	'Gm36049'	88	80	23	0	33	20	63.66666667	17.66666667	-1.915297036	0.079672345	0.617305615
102639870	'Gm38523'	0	1	0	1	1	0	0.333333333	0.666666667	1.081692101	0.760081965	0.972790461
102639919	'Gm36118'	0	0	0	2	0	0	0	0.666666667	2.299096387	0.566358544	0.972790461
102639922	'Gm10157'	6.44	1.18	0	2	1.88	0	2.54	1.293333333	-0.881615081	0.702966888	0.972790461
102640003	'Gm36182'	19	11	6.33	0	10	9	12.11	6.333333333	-0.993575878	0.391089681	0.952371379
102640040	'Gm11971'	1	1	0	0	1	1	0.666666667	0.666666667	0.015019958	0.995726078	0.999562152
102640043	'Gm36210'	48	40	11	19	24	47	33	30	-0.073918755	0.912650189	0.989773583
102640165	'Gm36298'	111.18	92.75	60.33	49.85	102.09	69.28	88.08666667	73.74	-0.211159827	0.611988569	0.972790461
102640171	'Gm38525'	0	0	0	0	1	0	0	0.333333333	1.020273531	0.802557913	0.972790461
102640263	'Gm36375'	3	6	1	7	2	5	3.333333333	4.666666667	0.705609842	0.577953226	0.972790461
102640268	'Gm2956'	100.57	83.01	41.31	3.45	29.84	10.07	74.96333333	14.45333333	-2.431892718	0.001379489	0.079475261
102640376	'Gm36448'	0	2	0	3	0	0	0.666666667	1	1.14429675	0.769651733	0.972790461
102640594	'Gm28710'	0	1	1	1	1	2	0.666666667	1.333333333	0.993345132	0.643020894	0.972790461
102640673	'Gm5165'	213.42	205.6	209.63	87.3	219.2	199	209.55	168.5	-0.337595324	0.359578105	0.933706718
102640707	'Gm15793'	1.62	0.77	2.28	13.68	3.15	6	1.556666667	7.61	3.043693453	0.04877907	0.513405877
102640710	'Gm36712'	1	0	2	6	2	1	1	3	1.775457984	0.33599914	0.922539021
102640717	'Gm36718'	3	2	0	2	0	0	1.666666667	0.666666667	-0.82444629	0.773940896	0.972790461
102640722	'Gm36722'	32	22	8	25	39	7	20.66666667	23.66666667	0.364789307	0.67642156	0.972790461
102640804	'Gm21818'	0	0	0	1	2	1.31	0	1.436666667	2.898671682	0.359820271	0.933706718
102640809	'Gm36789'	20	15	1	2	26	8	12	12	0.011264998	0.992605162	0.999493374
102640913	'Gm36864'	70	74	3	1	20	3	49	8	-2.59078414	0.038899783	0.461389364
102640920	'Pvrig'	2	8	0	0	0	1	3.333333333	0.333333333	-3.051369882	0.253869975	0.873052317
102641157	'Gm38538'	17.57	15.01	13.47	3.09	9.49	14.93	15.35	9.17	-0.82527359	0.274753409	0.885958584
102641752	'Gm38574'	1.04	1.07	1.02	1	1	0	1.043333333	0.666666667	-0.443373515	0.844041211	0.975182082
102642386	'Gm6871'	116	107	15	0	52	110	79.33333333	54	-0.63441564	0.625665702	0.972790461
102643076	'Percc1'	1	2	0	1	3	1	1	1.666666667	0.82926288	0.675692832	0.972790461
102643210	'Gm38664'	11	13	2	4	7.01	5	8.666666667	5.336666667	-0.595453482	0.554668155	0.972790461

102644	'Oaf'	279	264	187	1154	367	412	243.3333333	644.3333333	1.683793717	0.014247149	0.297003065
102657	'Cd276'	746	715	1311	641	856	1070	924	855.6666667	-0.135833504	0.767582623	0.972790461
102680	'Slc6a20a'	2	0	0	0	5	9.5	0.666666667	4.833333333	2.725532477	0.259442024	0.877955237
102693	'Phldb1'	1155	1195	1387	974	1422	1230	1245.666667	1208.666667	-0.016126828	0.961395131	0.999493374
102747	'Lrrc49'	498	517	284	31	379	354	433	254.6666667	-0.854759892	0.240885534	0.86449675
102774	'Bbs4'	406	409	413	159	442	463	409.3333333	354.6666667	-0.254305537	0.517402925	0.972790461
102791	'Tcta'	591	646	401	159	442	501	546	367.3333333	-0.592767053	0.136138082	0.736689633
102857	'Slc6a8'	732	683	1081	1374	698	949	832	1007	0.402773398	0.480641717	0.972790461
102866	'Pls3'	1991	1975	1688	3054	2303	2731	1884.666667	2696	0.640202527	0.107976083	0.684280429
102871	'Radx'	20	18	3	17	19	11	13.66666667	15.66666667	0.374659463	0.663702371	0.972790461
102902673	'Gm21992'	9.22	18.79	10.15	6.12	11.46	19.18	12.72	12.25333333	-0.045244668	0.951842901	0.998640168
102920	'Cenpi'	253	247	81.03	169.85	196	139	193.6766667	168.2833333	-0.051669281	0.921758841	0.991121001
102954	'Nudt10'	139.91	142.2	181.38	14.28	141.34	125.54	154.4966667	93.72	-0.859025309	0.22635181	0.853679947
102991	'Ezhip'	233	216	52	0	96	217	167	104.3333333	-0.775622884	0.548888006	0.972790461
103080	'Septin10'	963	1078	884	999	1145	1179	975	1107.666667	0.255222539	0.382773528	0.945348183
103098	'Slc6a15'	10	9	223	174	38	33	80.66666667	81.66666667	0.148358056	0.91098728	0.989640031
103135	'Pan2'	1234	1252	845	622	1186	912	1110.333333	906.6666667	-0.251102665	0.389482196	0.951231321
103136	'Pwp1'	850	889	976	426	860	1047	905	777.6666667	-0.253029306	0.470823575	0.972790461
103140	'Gstt3'	221	242	113	370	277	360	192	335.6666667	0.955494204	0.034945021	0.447993761
103142	'Rdh9'	31.02	21	27	191.49	21	41	26.34	84.49666667	2.016413033	0.038045056	0.459930654
103149	'Upb1'	1	4	1	1	6	3	2	3.333333333	0.735069565	0.617409982	0.972790461
103161	'Apof'	5	2	2	4	5	4	3	4.333333333	0.614561071	0.595925202	0.972790461
103172	'Chchd10'	601	649	3609	2784	1283	2331	1619.666667	2132.666667	0.408541313	0.638768826	0.972790461
103199	'Fig4'	899	991	521	354	837	1001	803.6666667	730.6666667	-0.137238438	0.721255226	0.972790461
103213	'Traf3ip2'	549	621	570	96	460	373	580	309.6666667	-0.98060384	0.052829928	0.532526438
103220	'Ttc41'	278.65	296.98	195.03	22.12	193.67	84.23	256.8866667	100.0066667	-1.423021605	0.030053676	0.421372044
103236	'Csnk1g2'	2636	2699	2794	2224	2441	3221	2709.666667	2628.666667	-0.0027214	0.993037509	0.999493374
103266	'Tmem263'	1777	1674.4	5637	1161.1	1320	1876.5	3029.446667	1452.533333	-1.123729946	0.08366031	0.627931232
103268	'Cep57l1'	248	273	202	184	296	292	241	257.3333333	0.130801394	0.626401086	0.972790461
103284	'Zc3h10'	377	395	437	330	273	428	403	343.6666667	-0.170728045	0.668029531	0.972790461
103406	'Zfr2'	243	226	95	68	322	330	188	240	0.321043078	0.581585378	0.972790461
103425	'Ncln'	927	886	508	627	1057	1102	773.6666667	928.6666667	0.309991693	0.305994387	0.907188578
103466	'Nt5dc3'	429	345	658	884	384	486	477.3333333	584.6666667	0.442886995	0.490900006	0.972790461
103468	'Nup107'	2025	2241	756	714	1464	1646	1674	1274.666667	-0.350361666	0.435915441	0.96581102
103511	'Calhm5'	5	14	10	7	8	4	9.666666667	6.333333333	-0.518518099	0.573781352	0.972790461
103534	'Mgat4b'	984	1050	1789	1573	1555	1990	1274.333333	1706	0.438101623	0.353321595	0.930050349
103537	'Mbtd1'	3247	3170	3444	2094	2994	2632	3287	2573.333333	-0.316837961	0.297508472	0.900750988
103551	'Epop'	127	96	246	188	70	104	156.3333333	120.6666667	-0.238448972	0.744003856	0.972790461
103554	'Psme4'	3372	3325.9	2095	3288	3161	3065	2930.966667	3171.333333	0.239246238	0.51183828	0.972790461
103573	'Xpo1'	6456	6462	6010	3877	5988	5533	6309.333333	5132.666667	-0.26477055	0.292295949	0.899940175

103583	'Fbxw11'	2565	2456	3101	3879	2467	3463	2707.333333	3269.666667	0.377693101	0.422192269	0.959445304
103655	'Sec14l4'	20	15	2	4	9	19	12.33333333	10.66666667	-0.184204178	0.854173753	0.975734242
103677	'Smg6'	2363.3	2310.9	1360.4	830.66	2012.4	1669.56	2011.52	1504.216667	-0.401420997	0.244819803	0.866091613
103694	'Tmed4'	941	950	733	1199	1349	1791	874.6666667	1446.333333	0.78459138	0.004524335	0.158153575
103710	'Slc35e4'	73	33	120	295	73	124	75.33333333	164	1.306112147	0.122598101	0.714169238
103711	'Pnpo'	315	345	573	397	341	519	411	419	0.050194377	0.918927564	0.990815958
103712	'6330403K07Rik'	649	707	353	60	560	329	569.6666667	316.3333333	-0.907910375	0.171154963	0.787010498
103724	'Tbc1d10a'	361	335	511	338	379	527	402.3333333	414.6666667	0.049783745	0.904678552	0.988191689
103733	'Tubg1'	519.47	539.97	338.2	948	451.49	550.91	465.88	650.1333333	0.695532463	0.209009177	0.836616817
103737	'Pex12'	619.53	560.64	411.96	186	483.82	470.96	530.71	380.26	-0.49117117	0.16624142	0.780893009
103742	'Mien1'	390	424	493	369	292	552	435.6666667	404.3333333	-0.066475265	0.876655093	0.981341203
103743	'Tmem98'	851	924	459	445	1228	1134	744.6666667	935.6666667	0.334578594	0.404531931	0.956587198
103765	'Tmem17'	91	79	43	52	129	100	71	93.66666667	0.427698363	0.332702236	0.921648675
103768	'Tubg2'	67.53	67.03	50.8	14	43.51	38.09	61.78666667	31.86666667	-0.965320496	0.041741426	0.477375478
103775	'Slc25a41'	6	3	1	2	3	4	3.333333333	3	-0.082980458	0.948599015	0.997521806
103784	'Wdr92'	450	482	1776.1	2870.1	473	621	902.6866667	1321.373333	0.770498199	0.4339093	0.965077633
103806	'Maml1'	1197	1160	2857	1335	1177	1226	1738	1246	-0.453336075	0.441524664	0.968551476
103836	'Zfp692'	450	449	166	259	602	314	355	391.6666667	0.216287638	0.662393499	0.972790461
103841	'Cuedc1'	301	319	383	173	358	317	334.3333333	282.6666667	-0.262782616	0.47422741	0.972790461
103844	'Inca1'	471	494	276	8	155	144	413.6666667	102.3333333	-2.114301532	0.008266804	0.220432644
103850	'Nt5m'	253	263	243	50	224	203	253	159	-0.741293709	0.139413392	0.741642687
103889	'Hoxb2'	20	17	54	31	35	21	30.33333333	29	-0.047469475	0.95076612	0.998065566
103963	'Rpn1'	2872	2785	2970	2921	3469	6626	2875.666667	4338.666667	0.588151516	0.113671591	0.698948431
103964	'Try5'	0	0	0	0	1	0	0	0.333333333	1.020273531	0.802557913	0.972790461
103967	'Dnm3'	79	52	19	524	72	81	50	225.6666667	2.599994724	0.008436737	0.222807422
103968	'Plin1'	47	53	2	5	41	13	34	19.66666667	-0.752846526	0.499436477	0.972790461
103978	'Gpc5'	0	0	0	1	2	1	0	1.333333333	2.898671682	0.359820271	0.933706718
103988	'Gck'	38	39	92	3	21	20	56.33333333	14.66666667	-2.095432386	0.008889443	0.231288612
104001	'Rtn1'	247	262	583	508	141	307	364	318.6666667	-0.05226228	0.944624101	0.996592403
104009	'Qsox1'	1868	2042	845	1971	4834	3434	1585	3413	1.150003543	0.006034699	0.183570701
104010	'Cdh22'	18	16	15	3	7	6	16.33333333	5.333333333	-1.604852369	0.032395677	0.434162511
104015	'Synj1'	2081	2072	437	754	1003	928	1530	895	-0.641049365	0.260950121	0.878842185
104027	'Synpo'	865	834	1544	1081	597	744	1081	807.3333333	-0.313093219	0.59665623	0.972790461
104069	'Sncb'	23	13	347	0	5	3	127.6666667	2.666666667	-5.868417501	3.21E-05	0.006112081
104079	'Nxph3'	95	100	26	129	67	90	73.66666667	95.33333333	0.600706617	0.366941062	0.936808167
104080	'Nxph4'	56	42	396	72	17	78	164.6666667	55.66666667	-1.582538297	0.129969486	0.727578716
104082	'Wdr7'	1081	969	556	961	900	1038	868.6666667	966.3333333	0.275235117	0.466033047	0.972790461
104086	'Cyp27a1'	47	51	83	57	37	45	60.33333333	46.33333333	-0.296094961	0.624699947	0.972790461
104099	'Itga9'	1242	1145	678	261	1903	1660	1021.666667	1274.666667	0.250287533	0.683350439	0.972790461
104110	'Adcy4'	90	81	97	8	126	110	89.33333333	81.33333333	-0.275259988	0.729125632	0.972790461

104111	'Adcy3'	780	808	268	252	1044	842.96	618.6666667	712.9866667	0.202535578	0.71643243	0.972790461
104112	'Acly'	3757	3680	2105	3094	3386	3422	3180.666667	3300.666667	0.16168419	0.63342733	0.972790461
104130	'Ndufb11'	1114.6	1212.5	1376.9	952	980.92	1461.82	1234.67	1131.58	-0.096549775	0.789154069	0.972790461
104156	'Etv5'	337	345	827	862	322	561	503	581.6666667	0.322480973	0.650442298	0.972790461
104158	'Ces1d'	2	2	0	21	7	12	1.333333333	13.33333333	3.575498646	0.005254313	0.171346471
104174	'Gldc'	78	65	36	12	26	29	59.66666667	22.33333333	-1.390298379	0.005942667	0.1813515
104175	'Sbk1'	1979	2058	1030	1322	2526	1941	1689	1929.666667	0.25105887	0.471431082	0.972790461
104183	'Chil4'	1	0	0	48	0	2	0.333333333	16.66666667	6.000563263	0.010580428	0.254415968
104184	'Blmh'	1899	1961	1696	951	1953	1881	1852	1595	-0.216477095	0.433627333	0.964992995
104215	'Rhoq'	1350.2	1239	4706.8	1533	1380.8	1684.73	2432.016667	1532.856667	-0.688910562	0.320609619	0.917433787
104245	'Slc6a5'	3	5	2	1	0	0	3.333333333	0.333333333	-3.124329604	0.125474115	0.72022881
104248	'Cabin1'	2533	2393	974	1217	2424	2215	1966.666667	1952	0.044412125	0.913540462	0.989868418
104252	'Cdc42ep2'	294	225	1609	357	256	256	709.3333333	289.6666667	-1.296393499	0.137530911	0.738626463
104263	'Kdm3a'	6775	6141	12240	2754	5735	4640	8385.333333	4376.333333	-0.991276695	0.040785607	0.473970635
104271	'Tex15'	7554	7592	953	301	5429	7876	5366.333333	4535.333333	-0.298762909	0.758711878	0.972790461
104303	'Arl1'	2484	2368	4756	2533	2400	3436	3202.666667	2789.666667	-0.19587145	0.699323704	0.972790461
104318	'Csnk1d'	4747.1	4742.1	5880.4	4583.2	4647	5494.09	5123.18	4908.086667	-0.01239481	0.973222985	0.999493374
104346	'Gas8'	768	765	438	391	628	534	657	517.6666667	-0.277147011	0.374909719	0.940406075
104348	'Zfp120'	316	316	324	186	297	331	318.6666667	271.3333333	-0.223801976	0.450764936	0.970590732
104349	'Zfp119a'	150	165.65	112	58.04	153	115	142.55	108.68	-0.383297611	0.318425229	0.916136994
104360	'Isl2'	13	17	3	9	7	5	11	7	-0.454076379	0.63617953	0.972790461
104362	'Meig1'	6	6	4	0	6	14	5.333333333	6.666666667	0.201768555	0.877457542	0.981341203
104382	'Barhl2'	0	0	1	0	1	1	0.333333333	0.666666667	0.818407084	0.819542986	0.972790461
104383	'Rcor2'	609	589	1488	267	396	534	895.3333333	399	-1.237978139	0.029828417	0.421008072
104384	'Rhox9'	39	37	58.89	4	12	13	44.96333333	9.666666667	-2.268339086	4.71E-04	0.042077778
104394	'E2f4'	586	626	674	598	623	668	628.6666667	629.6666667	0.064832963	0.854510932	0.975734242
104401	'Pcnx3'	2375	2134.9	649	1480.1	2865.3	2322.65	1719.636667	2222.673333	0.451497653	0.361865969	0.93373993
104416	'Bap1'	2111	2033	1695	1346	1800	2337	1946.333333	1827.666667	-0.057175038	0.819699711	0.972790461
104418	'Dgkz'	1976	2027	801.95	1558	2786	2155	1601.65	2166.333333	0.51587792	0.207260004	0.83479753
104443	'Npffr2'	4	3	1	0	0	0	2.666666667	0	-3.740992684	0.150928413	0.759755159
104444	'Rexo2'	354	385	448	244	421	768	395.6666667	477.6666667	0.217095856	0.619835557	0.972790461
104445	'Cdc42ep1'	671	633	449	1144	787	1026	584.3333333	985.6666667	0.897390114	0.034616221	0.446185491
104457	'0610010K14Rik'	334	417	321	157	309	419	357.3333333	295	-0.29350107	0.403539803	0.956143911
104458	'Rars'	1856	1906	2419	901	1712	2149	2060.333333	1587.333333	-0.41770094	0.266184989	0.882887128
104479	'Ccdc117'	1474	1403	1207	1296	1109	1458	1361.333333	1287.666667	0.014244857	0.967453011	0.999493374
104570	'Ppp4r3b'	2285.2	2501.1	2584.6	1643.6	2556.5	2226.18	2456.98	2142.076667	-0.172582323	0.567005788	0.972790461
104582	'Rprml'	3	5	4	2	3	2	4	2.333333333	-0.730978258	0.546525771	0.972790461
104601	'Mycbpap'	68	69	42	104	89	45	59.66666667	79.33333333	0.595855704	0.315988832	0.914806078
104625	'Cnot6'	3372	3623	3204	2340	3861	3025	3399.666667	3075.333333	-0.106910207	0.704035757	0.972790461
104662	'Tsr1'	1385.8	1308.7	1544.2	485.99	1240	1454.43	1412.893333	1060.136667	-0.473352836	0.237004235	0.862272407

104681	'Slc16a6'	120.88	129.9	279.98	24.67	113.72	158	176.92	98.79666667	-1.005755842	0.152549144	0.763026656
104709	'Pik3r6'	21	15	2	0	38	14	12.66666667	17.33333333	0.408741986	0.769990165	0.972790461
104718	'Ttc7b'	603	552	812	482	604	718	655.6666667	601.3333333	-0.116576587	0.762823294	0.972790461
104721	'Ddx1'	4217	4175	3197	1466	3046	3861	3863	2791	-0.481324788	0.131860577	0.72897159
104725	'Sptssa'	1063	1210	1322	796	1318	1291	1198.333333	1135	-0.075548746	0.81198586	0.972790461
104732	'Tedc1'	261	308	65	147	166	187	211.3333333	166.6666667	-0.207345431	0.716965716	0.972790461
104759	'Pld4'	15	14	2	31	24	46	10.33333333	33.66666667	1.836584248	0.019757327	0.349283865
104771	'Jkamp'	677	662	381	411	656	764	573.3333333	610.3333333	0.134033909	0.660207179	0.972790461
104776	'Aldh6a1'	1424.9	1251	1274	1264.1	1560.9	1890.98	1316.626667	1572.02	0.295329029	0.290313157	0.898321851
104799	'Vipas39'	1032	1043	836	412	1045	927	970.3333333	794.6666667	-0.300229993	0.360365761	0.933706718
104806	'Fancm'	616	665	215	296	552	419	498.6666667	422.3333333	-0.155817053	0.737582928	0.972790461
104816	'Aspg'	6	5	9	26	4	12	6.666666667	14	1.280622675	0.224718992	0.850759465
104831	'Ptpn23'	1950	1968	2883	2694	2054	2040	2267	2262.666667	0.09264008	0.851237162	0.975734242
104836	'Cbll1'	903	947	650	450	653	927	833.3333333	676.6666667	-0.274115541	0.34345219	0.925308103
104859	'Tecpr2'	757	771	435	417	646	414	654.3333333	492.3333333	-0.318874716	0.393888883	0.953178011
104871	'Spata7'	300	255	153	182	237	266	236	228.3333333	0.025597388	0.939762295	0.995607401
104884	'Tdp1'	422	507	296	331	410	457	408.3333333	399.3333333	0.041433605	0.890813444	0.985046501
104885	'Tmem179'	29	17	13	4	17	15	19.66666667	12	-0.728863606	0.317949735	0.916136994
104886	'Rab15'	178	171	450	252	119	206	266.3333333	192.3333333	-0.400098358	0.562217219	0.972790461
104910	'Slc25a47'	22	19	18	17	43	35	19.66666667	31.66666667	0.683624021	0.200316336	0.82759347
104923	'Adi1'	458.01	495.95	526.97	437	531	810	493.6433333	592.6666667	0.275683321	0.418853344	0.958308933
104943	'Fam110c'	273	237	387	355	417	551	299	441	0.562616128	0.183866233	0.804729813
105000	'Dnal1'	737	770	308	687	747	496	605	643.3333333	0.248470849	0.608036557	0.972790461
105005	'Lratd1'	129	98	64	96	213	167	97	158.6666667	0.742184414	0.061753729	0.563910613
105014	'Rdh14'	319	316	149	242	380	371	261.3333333	331	0.410862067	0.256438507	0.876779958
105083	'Pelo'	241	240	428	349	237	348	303	311.3333333	0.098981906	0.854471534	0.975734242
105148	'Iars'	2738	2577	6701	1995	2394	3594	4005.333333	2661	-0.645535345	0.259662391	0.878316184
105171	'Arrdc3'	2978	2884	9631	4291	1856	2264	5164.333333	2803.666667	-0.779362491	0.310843849	0.910971113
105180375	'Tmem265'	184	185	95	31	146	87	154.6666667	88	-0.828016948	0.128340262	0.724799196
105193	'Nhlrc1'	110	109	57	51	134	173	92	119.3333333	0.365379336	0.435060884	0.965608974
105203	'Tasor2'	3773.1	3989.1	1480.1	1467	2951.1	2611.01	3080.773333	2343.026667	-0.336349492	0.422076879	0.959445304
105239	'Rnf44'	2678	2626	3636	3622	2681	2874	2980	3059	0.136756883	0.774264538	0.972790461
105242399	'Gm21083'	14	6.86	3	39.16	11.51	18.07	7.953333333	22.91333333	1.843945006	0.057059338	0.547413448
105242401	'Gm21115'	2	0	0	0	0	0	0.666666667	0	-1.711373851	0.67234292	0.972790461
105242430	'Gm38699'	133.27	133.91	102.82	56.35	128.71	103.31	123.3333333	96.12333333	-0.346438934	0.331283124	0.921648675
105242433	'Gm38702'	434.12	418.4	244.66	108.11	369.95	296.65	365.7266667	258.2366667	-0.515209228	0.239273967	0.86449675
105242435	'Gm38704'	21.49	30.1	5.2	1	16.42	12	18.93	9.806666667	-0.972306836	0.344315053	0.925308103
105242449	'Gm21103'	3.95	9	12	9	6	3	8.316666667	6	-0.29487007	0.784428025	0.972790461
105242472	'Gm11639'	42	43	4	0	56	2	29.66666667	19.33333333	-0.639442487	0.68251654	0.972790461
105242668	'Gm38821'	1	2	1	1	0	0	1.333333333	0.333333333	-1.817755807	0.471982145	0.972790461

105242736	'Gm6749'	2	3	1	0	0	0	2	0	-3.337472277	0.23673828	0.862272407
105242927	'Gm38999'	1	1	0	0	0	0	0.666666667	0	-1.703500596	0.673820112	0.972790461
105242930	'Gm39002'	0	1	0	6	0	1	0.333333333	2.333333333	3.084516381	0.270871292	0.884086697
105242931	'Gm39003'	29	26	7	15	15	8	20.66666667	12.66666667	-0.52430497	0.513576314	0.972790461
105243	'Slc9a3'	8	6	1	192	11	10	5	71	4.333963701	0.001197194	0.072517381
105243089	'Gm39115'	0	0	0	1	1	0	0	0.666666667	2.050872943	0.610671515	0.972790461
105243147	'Gm39147'	2	3	4	2	1	2	3	1.666666667	-0.791220305	0.575683523	0.972790461
105243585	'Gm39469'	375	398	209.99	317	453	326	327.6633333	365.3333333	0.255824007	0.493310594	0.972790461
105243794	'Gm39566'	5	1	1	9	3	2	2.333333333	4.666666667	1.293956996	0.368751345	0.937530612
105244006	'Gm39701'	182.64	167.12	224.13	366.16	610.83	574.55	191.2966667	517.18	1.433987656	6.36E-05	0.009744897
105244034	'Gm33887'	454.99	432.71	188.34	106.72	370.17	188.53	358.68	221.8066667	-0.668194868	0.195082488	0.819942118
105244158	'1700024J04Rik'	12	17	6	0	9	9	11.66666667	6	-1.028502218	0.367551639	0.937190492
105244392	'Gm40011'	0	0	0	0	0	1	0	0.333333333	1.020273531	0.802557913	0.972790461
105244402	'Gm14444'	37.58	23.8	6.45	7.02	12.92	15.42	22.61	11.78666667	-0.883989012	0.272230932	0.884738928
105244426	'Gm40035'	2	3	0	1	4	1	1.666666667	2	0.348427851	0.848339548	0.975686757
105244498	'Gm40095'	8	6	6	4	4	3	6.666666667	3.666666667	-0.776576695	0.432266425	0.963974389
105244600	'Gm40190'	7	10	4	2	7	8	7	5.666666667	-0.306787953	0.749683889	0.972790461
105244717	'Gm40275'	3	8	3	0	2	0	4.666666667	0.666666667	-2.796330469	0.136383971	0.736837756
105244808	'Gm40353'	2	1	0	0	2	0	1	0.666666667	-0.54242627	0.865172559	0.978040999
105244828	'Gm40364'	41.69	0	0	7.35	0	23.78	13.89666667	10.37666667	-0.353996552	0.879533419	0.981807495
105244829	'Gm40365'	0	9.6	0	0	19.33	0	3.2	6.443333333	1.078943729	0.724974382	0.972790461
105244831	'Gm40367'	88.69	41.06	41.07	0	7.08	41	56.94	16.02666667	-1.969370272	0.091110376	0.649965349
105244833	'Gm40369'	134.12	147.37	108.07	4.65	84.74	81.53	129.8533333	56.97333333	-1.320168718	0.107368943	0.683046488
105244844	'Gm40378'	7.55	6.67	5.73	1.63	3.02	0	6.65	1.55	-2.112751679	0.137500226	0.738626463
105244925	'Gm40447'	2	6	11	9	2	6	6.333333333	5.666666667	-0.053428614	0.964424995	0.999493374
105244931	'Gm40453'	15.56	22.55	11.18	3.47	4.41	1.96	16.43	3.28	-2.475906771	0.009551776	0.240209487
105244947	'Gm40469'	129	138	104	18	54	66	123.6666667	46	-1.459371683	0.001379187	0.079475261
105245	'Txndc5'	2872	2804	2066	2680	4026	5458	2580.666667	4054.666667	0.67487253	0.012138266	0.272458927
105245043	'Gm2396'	19.9	30.51	1.99	0	21.77	17	17.46666667	12.92333333	-0.433589394	0.763436307	0.972790461
105245097	'Gm40595'	86.12	76.42	15.56	38.89	68.22	28.18	59.36666667	45.09666667	-0.2604834	0.720353718	0.972790461
105245236	'Gm19802'	3.6	0	0	0	7.62	0	1.2	2.54	1.204996899	0.733196883	0.972790461
105245338	'Gm40811'	3	2	3	5	2	2	2.666666667	3	0.336690793	0.799232656	0.972790461
105245342	'Gm40814'	25	12.38	1.17	0	4.32	8.56	12.85	4.293333333	-1.698502239	0.240551694	0.86449675
105245381	'Gm40847'	165.59	172.79	90.76	55.28	124.89	105.86	143.0466667	95.34333333	-0.55643125	0.156703832	0.768046725
105245382	'Gm9241'	56.15	62.71	56.62	16.98	55.44	30.78	58.49333333	34.4	-0.802362821	0.126463391	0.721271206
105245383	'Zfp996'	130.51	133.11	44.34	10.8	48.54	46.84	102.6533333	35.39333333	-1.571197255	0.013970539	0.293124205
105245389	'Gm40853'	24.51	26.96	33.45	17.68	31.2	30.34	28.30666667	26.40666667	-0.097934652	0.854054514	0.975734242
105245393	'Gm40857'	15.38	9.29	8.7	4.58	8.79	8.32	11.12333333	7.23	-0.653312527	0.415940478	0.95722888
105245406	'Gm5954'	33.49	22.32	19.58	5.72	17.63	12.56	25.13	11.97	-1.12206194	0.087464841	0.642039209
105245424	'Gm40881'	109.98	95.08	25.74	4.98	51.24	33.29	76.93333333	29.83666667	-1.410830364	0.094107653	0.658197981

105245436	'Gm40892'	0	2	0	0	1	0	0.666666667	0.333333333	-0.733814424	0.856146772	0.975734242
105245545	'Gm10323'	10.12	8.59	0	0	5	24.01	6.236666667	9.67	0.621224772	0.727049481	0.972790461
105245577	'Gm10772'	5.04	6.26	2	0	0	3	4.433333333	1	-2.167138785	0.243522233	0.865085867
105245580	'A930018O16Rik'	3.1	5	0	4	6	3	2.7	4.333333333	0.85685285	0.54535903	0.972790461
105245675	'Gm21560'	2.53	2.01	2.92	0	0	0	2.486666667	0	-3.391022779	0.216039438	0.843391934
105245682	'Gm41099'	2	0	0	3	3	2	0.666666667	2.666666667	2.168219758	0.294123896	0.90029104
105245684	'Gm3012'	2.12	7.38	0	0	0	0	3.166666667	0	-3.858461691	0.250650254	0.871167957
105245737	'Gm8138'	0	1.69	2.06	0	2	0	1.25	0.666666667	-0.733221529	0.816021492	0.972790461
105245911	'Gm41291'	34.31	32.55	11.59	22.78	35.59	24.27	26.15	27.54666667	0.173441018	0.776649799	0.972790461
105246	'Brd9'	905	903	429	652	1130	806	745.6666667	862.6666667	0.290366065	0.444630916	0.969888068
105246049	'Gm41408'	61.71	60.57	27.74	30.78	39.18	34.67	50.00666667	34.87666667	-0.425160116	0.390859341	0.952178913
105246303	'Gm41607'	68.61	74.07	24.68	48.06	89.93	62.53	55.78666667	66.84	0.346994498	0.509648557	0.972790461
105246320	'Gm26637'	2	8	2	6	33	17	4	18.66666667	2.210637479	0.027713642	0.407338487
105246572	'Gm41844'	91.68	74.69	42.07	3.33	4.09	4.3	69.48	3.906666667	-4.164188781	6.43E-10	6.85E-07
105246961	'AB010352'	388.36	406	103	215	419.86	305	299.12	313.2866667	0.157177791	0.769374705	0.972790461
105247050	'Gm42226'	67.89	130.23	82.77	827.65	595	459.39	93.63	627.3466667	2.924771371	1.04E-07	5.99E-05
105247180	'Gm42323'	20	24	5	1	20	7	16.33333333	9.333333333	-0.825142401	0.441881763	0.969048871
105247198	'Gm42337'	1	1	0	4	0	1	0.666666667	1.666666667	1.7243165	0.479629174	0.972790461
105247207	'Gm42346'	74.34	70.08	44.76	1	20.13	9.82	63.06	10.31666667	-2.699529636	6.04E-04	0.048169622
105247240	'Gm42372'	96	87	32	60	86	47	71.66666667	64.33333333	-0.023523685	0.966587675	0.999493374
105278	'Cdk20'	142	111	143	91	100	131	132	107.3333333	-0.264196144	0.497384249	0.972790461
105298	'Epdr1'	366	347	152	61	734	727	288.3333333	507.3333333	0.73842777	0.326580225	0.920734892
105348	'Golm1'	841	877	459	534	898	1133	725.6666667	855	0.271686461	0.416438635	0.95722888
105349	'Akr1c18'	22	23	71	3	116	33	38.66666667	50.66666667	0.176207222	0.871387034	0.980171118
105351	'AW209491'	244	253	331	64	266	419	276	249.6666667	-0.269109242	0.653016874	0.972790461
105352	'Dusp22'	404	436	760	102	365	503	533.3333333	323.3333333	-0.855158039	0.148373676	0.756674202
105355	'Slc17a3'	1	1	0	0	2	10	0.666666667	4	2.497227322	0.256697643	0.877127174
105372	'Utp15'	799	771	1123	578	738	810	897.6666667	708.6666667	-0.330023455	0.39505834	0.954014376
105377	'Slf1'	741	714	264	112	598	472	573	394	-0.562017149	0.345172944	0.926100052
105387	'Akr1c14'	1104	1286	102	296	3330	1646	830.6666667	1757.333333	1.078224238	0.241464777	0.86449675
105418	'E330034G19Rik'	3	1	3	1	1	6	2.333333333	2.666666667	0.110980757	0.94161469	0.996210609
105428	'Fam149b'	927	886.51	393	193.15	783	747	735.5033333	574.3833333	-0.377149189	0.468411475	0.972790461
105439	'Slain1'	163	186	84	39	145	164	144.3333333	116	-0.340285025	0.518756716	0.972790461
105440	'Kctd9'	633	677	882	526	581	498	730.6666667	535	-0.392392178	0.346615809	0.928149262
105445	'Dock9'	2329	2313	945	1044	2979	2208	1862.333333	2077	0.184886275	0.681788133	0.972790461
105446	'Gmpr2'	366	358.48	377.01	246.99	355.52	348	367.1633333	316.8366667	-0.185293446	0.535649317	0.972790461
105450	'Mmrn2'	81	75	18	2	155	197	58	118	0.942014461	0.396525493	0.954271224
105501	'Abhd4'	2071	2017	3151	2856	2483	3039	2413	2792.666667	0.262439948	0.564063237	0.972790461
105504	'Exoc5'	1146	1270.8	1787	1391.9	1503	1398	1401.25	1430.976667	0.076950639	0.858336205	0.976475731
105511	'Fam170b'	1	0	1	0	0	1	0.666666667	0.333333333	-0.894127833	0.804654786	0.972790461

105513	'Chmp7'	1238	1172	1453	1536	1314	1698	1287.666667	1516	0.304268113	0.444868669	0.969888068
105518	'A630023A22Rik'	0	0	0	0	0	2	0	0.666666667	1.749449488	0.66566965	0.972790461
105522	'Ankrd28'	1671	1812	1893	1795	2002	1925	1792	1907.333333	0.153994604	0.652431121	0.972790461
105559	'Mbnl2'	2263	2184	10520	12942	2853	4659	4989	6818	0.601007391	0.524441727	0.972790461
105590	'Zfp957'	2	1	1	1	3	3	1.333333333	2.333333333	0.800035907	0.610003708	0.972790461
105594	'Cphx1'	0	1	7	7	4	29	2.666666667	13.33333333	2.137632647	0.150683188	0.759755159
105638	'Dph3'	456.26	534.08	487.35	323.96	465.67	546.93	492.5633333	445.52	-0.12704736	0.656044426	0.972790461
105651	'Ppp1r3e'	85	80	56	75	156	229	73.66666667	153.3333333	1.044415515	0.012327515	0.274438769
105653	'Phyhip'	46	47	23	95	32	36	38.66666667	54.33333333	0.770854498	0.305114886	0.907188578
105663	'Thtpa'	395	470	161	204	444	432	342	360	0.118228299	0.79183167	0.972790461
105670	'Rcbtb2'	1308	1419	1225	77	1449	1047	1317.333333	857.6666667	-0.745435897	0.341865276	0.925308103
105675	'Ppif'	339	305	488	327	296	418	377.3333333	347	-0.088274879	0.844850576	0.975182082
105689	'Mycbp2'	6873	6838	1665	5688	5847	4753	5125.333333	5429.333333	0.259665944	0.647813919	0.972790461
105704528	'Gm13090'	2	3	0	0	1	0	1.666666667	0.333333333	-2.058978643	0.491488033	0.972790461
105722	'Ano6'	1933	1828	1181	4090	2900	3092	1647.333333	3360.666667	1.19290404	0.007245727	0.202308598
105727	'Slc38a1'	1187	1050	1022	2664	1676	1664	1086.333333	2001.333333	1.0450456	0.033264166	0.440096253
105732	'Fam83h'	263	263	228	159	239	231	251.3333333	209.6666667	-0.223436144	0.417668774	0.957720764
105734	'Tigd5'	105	117	123	182	139	149	115	156.6666667	0.554680359	0.231698759	0.858905111
105734727	'Gm27021'	21.61	10.59	13.45	16.86	6.32	18.86	15.21666667	14.01333333	-0.024196427	0.976118565	0.999493374
105782	'Scrib'	1514	1484	682	1746	1900	2003	1226.666667	1883	0.736069455	0.057468802	0.548760163
105785	'Kdelr3'	316	300	1154	516	345	520	590	460.3333333	-0.357540563	0.62451666	0.972790461
105787	'Prkaa1'	965	962	1472	1285	1099	1128	1133	1170.666667	0.118294837	0.802708221	0.972790461
105827	'Amigo2'	458	457.79	269	509	571	471	394.93	517	0.506939432	0.173376329	0.792172738
105833	'Ccdc65'	33	43	17	18	40	55	31	37.66666667	0.292934943	0.601557574	0.972790461
105835	'Sgsm3'	742	722	300	743	438	550	588	577	0.174691024	0.744446016	0.972790461
105837	'Mtbp'	492	547	428	271	404	319	489	331.3333333	-0.50381016	0.091139927	0.649965349
105841	'Dennd3'	213	242	140	454	329	283	198.3333333	355.3333333	1.020304348	0.038122377	0.459930654
105844	'Card10'	246	224	115	814	277	287	195	459.3333333	1.531920047	0.02861963	0.411897356
105847	'Lmf2'	605	686	781	225	851	864	690.6666667	646.6666667	-0.182294874	0.708630883	0.972790461
105853	'Mal2'	10	11	16	55	12	18	12.33333333	28.33333333	1.440540064	0.121043156	0.712316789
105855	'Nckap1l'	70	63	21	107	103	125	51.33333333	111.6666667	1.25411917	0.017599071	0.331280729
105859	'Csdc2'	529	492	140	985	544	720	387	749.6666667	1.17910818	0.055303525	0.539316735
105886298	'Cmc4'	132.85	143.35	77.21	22.33	129.78	127.8	117.8033333	93.30333333	-0.396079632	0.509920303	0.972790461
105886299	'Gm44805'	5.92	0	0	0	1.52	2.77	1.973333333	1.43	-0.773952548	0.79138606	0.972790461
105940408	'Gm20498'	106.42	109.98	154.7	33.36	67.57	112.97	123.7	71.3	-0.871087647	0.087780103	0.642214018
105943584	'Gm45927'	362.76	463.08	422.67	209.46	444.35	370.44	416.17	341.4166667	-0.290320403	0.391294374	0.952371379
105980076	'Gm45929'	0	2.97	9.18	0	3.01	0	4.05	1.003333333	-2.108541084	0.407882569	0.957157474
105988	'Espl1'	1725	1576	132	36	977	1575	1144.333333	862.6666667	-0.464804508	0.668266352	0.972790461
106014	'Tafa5'	710	750	431	77	637	595	630.3333333	436.3333333	-0.609196208	0.345126514	0.926100052
106021	'Topors'	1441	1485	1651	1328	1527	1803	1525.666667	1552.666667	0.064015013	0.843303838	0.975182082

106025	'Sharpin'	465	476	324	320	420	411	421.6666667	383.6666667	-0.065659771	0.81541953	0.972790461
106029237	'Gm21761'	0	0	0	0	0	2.69	0	0.896666667	1.749449488	0.66566965	0.972790461
106039	'Gga1'	885	894	940	725	980	1140	906.3333333	948.3333333	0.088138803	0.760205919	0.972790461
106042	'Prickle1'	431	401	523	835	500	667	451.6666667	667.3333333	0.68153814	0.173540664	0.79261444
106052	'Fbxo4'	176	161	266	61	205	182	201	149.3333333	-0.517479934	0.319117711	0.916136994
106064	'AW549877'	2815	2849	2489	1889	2908	2408	2717.666667	2401.666667	-0.132405563	0.6205903	0.972790461
106068	'Slc45a4'	1350	1550	640	3582	1907	2473	1180	2654	1.382445324	0.011230473	0.262624548
106073	'Mfsd5'	354	360	395	394	448	546	369.6666667	462.6666667	0.362772578	0.272066348	0.884539076
106143	'Cggbp1'	4155	4271	4303	3456	3480	4409	4243	3781.666667	-0.106683246	0.741679137	0.972790461
106200	'Txndc11'	423	380	695	239	440	613	499.3333333	430.6666667	-0.283288669	0.555012589	0.972790461
106205	'Zc3h7a'	1115	1099	2628	1094	1334	1024	1614	1150.666667	-0.483747372	0.39674488	0.954358954
106248	'Qtrt2'	763	651	606	160	630	673	673.3333333	487.6666667	-0.532528966	0.263454627	0.88013169
106264	'0610012G03Rik'	284	297	627	165	340	493	402.6666667	332.6666667	-0.37838916	0.499938024	0.972790461
106298	'Rrn3'	1495	1526	1433	788	1315	1585	1484.666667	1229.333333	-0.27106472	0.321546423	0.91776619
106326	'Osbpl11'	1075	1135	887	1318	1356	1513	1032.333333	1395.666667	0.520502327	0.089610036	0.646660839
106338	'Nsun3'	581	580	236	25	369	366	465.6666667	253.3333333	-0.957088362	0.225568535	0.852751449
106344	'Rfc4'	1015	1000	439	163	552	727	818	480.6666667	-0.791948355	0.127810646	0.723917691
106347	'Ildr1'	7	11	8	0	2	3	8.666666667	1.666666667	-2.437018526	0.046629113	0.503156685
106369	'Ypel1'	420.21	433.11	147	44.27	402.49	324.32	333.44	257.0266667	-0.425130598	0.549577502	0.972790461
106389	'Eaf2'	109.58	110.41	120.02	18	73.02	31.13	113.3366667	40.71666667	-1.510025648	0.006754077	0.194853582
106393	'Srl'	993	1022	243	452	761	602	752.6666667	605	-0.207628451	0.701188024	0.972790461
106407	'Slc51a'	2	0	2	1	1	1	1.333333333	1	-0.39429516	0.846681266	0.975209338
106489	'Sft2d1'	182	224	186	306	230	321	197.3333333	285.6666667	0.635203283	0.123099552	0.714726459
106504	'Stk38'	1946	2001	1420	1405	1972	1495	1789	1624	-0.060300085	0.844390353	0.975182082
106512	'Gpsm3'	53	47	39	37	66	76	46.33333333	59.66666667	0.379578594	0.337791732	0.924020038
106522	'Pkdcc'	560	527	535	127	752	665	540.6666667	514.6666667	-0.166308732	0.767625953	0.972790461
106529	'Tecr'	1532	1697	802	1534	2241	2193	1343.666667	1989.333333	0.643894087	0.054484724	0.535883893
106557	'Ldhal6b'	16	14	1	10	23	7	10.33333333	13.33333333	0.499708131	0.634263204	0.972790461
106564	'Ppcs'	195	248	137	98	258	258	193.3333333	204.6666667	0.07861537	0.842702862	0.975182082
106565	'Dlk2'	10	9	19	8	1	7	12.66666667	5.333333333	-1.151669748	0.280874128	0.889693736
106572	'Rab31'	1304	1348	1657	1932	1498	1899	1436.333333	1776.333333	0.389829467	0.364640695	0.935515019
106581	'Fam234a'	1894	1920	1552	5575	2513	4203	1788.666667	4097	1.370446859	0.008673641	0.227139468
106582	'Nrm'	121	124	67	149	110	142	104	133.6666667	0.504222937	0.271751565	0.884539076
106583	'Scaf8'	1808	1845	1582	1057	1807	1795	1745	1553	-0.148273305	0.53529239	0.972790461
106585	'Ankrd12'	1021	1089	650	556	973.01	887.01	920.0066667	805.34	-0.145527049	0.607936533	0.972790461
106618	'Wdr90'	894	923	360	239	764	598	725.6666667	533.6666667	-0.428197119	0.371983759	0.938527219
106628	'Trip10'	752	706	2486	1293	830	1150	1314.666667	1091	-0.246315086	0.72828803	0.972790461
106633	'Ift140'	1249	1157	365	763	1145	953	923.6666667	953.6666667	0.156188985	0.746038184	0.972790461
106639	'Vmac'	527	515	427	165	602	576	489.6666667	447.6666667	-0.180563817	0.681583424	0.972790461
106648	'Cyp4f15'	37	45	1	0	43	17	27.66666667	20	-0.495303613	0.740290309	0.972790461

106672	'AI413582'	64	69	411	271	77	127	181.3333333	158.3333333	-0.100962772	0.917547141	0.990547571
106707	'Rpusd1'	516.16	683.39	308.02	143	915.22	594	502.5233333	550.74	0.090327196	0.878739385	0.981412981
106722	'Mpig6b'	4	5	1	0	3	3	3.333333333	2	-0.769275742	0.627010255	0.972790461
106757	'Catsperd'	56	68	80	11	44	44	68	33	-1.122451642	0.049934807	0.518209912
106759	'Ticam1'	280	315	1929	488	339	506	841.3333333	444.3333333	-0.961123621	0.268436976	0.883600995
106763	'Ttbk1'	28.78	16	4.31	4	5	7.99	16.36333333	5.663333333	-1.486695417	0.114845064	0.70178885
106766	'Stap2'	84	78	142.96	52.22	97.06	92.38	101.6533333	80.55333333	-0.374381266	0.452154597	0.970649024
106794	'Dhx57'	971	950	1182	582	1107	973	1034.333333	887.3333333	-0.232551716	0.500209326	0.972790461
106795	'Tcf19'	230	243	109	161	227	176	194	188	0.056318503	0.890027394	0.984980969
106821	'Oard1'	479	501	242	166	403	381	407.3333333	316.6666667	-0.343223806	0.381669973	0.94429001
106840	'Unc119b'	1319	1349	1303	996	1124	1267	1323.666667	1129	-0.173038505	0.560926313	0.972790461
106861	'Abhd3'	328	365	91	225	541	428	261.3333333	398	0.667722923	0.216013641	0.843391934
106869	'Tnfaip8'	438	410	890	560	475	582	579.3333333	539	-0.074594234	0.891965787	0.985171
106877	'Afap1l1'	691	709	303	568	1044	762	567.6666667	791.3333333	0.555092568	0.165001015	0.778863111
106878	'Smim3'	172	196	499	279	191	220	289	230	-0.281168705	0.671220076	0.972790461
106894	'Hmgxb3'	1055	995.26	1141.6	1317.2	959.24	1105.31	1063.956667	1127.253333	0.194636463	0.658079699	0.972790461
106931	'Kctd1'	250	280	290	108	200	274	273.3333333	194	-0.523544254	0.158671412	0.768918948
106947	'Slc39a3'	574	571	453	153	551	461	532.6666667	388.3333333	-0.495882125	0.244527861	0.866091613
106952	'Arap3'	619	550	408	132	746	412	525.6666667	430	-0.337660437	0.535186611	0.972790461
106957	'Slc39a6'	1469	1275	943	1001	1409	1779	1229	1396.333333	0.225049585	0.403046174	0.955996639
107022	'Gramd3'	225	208	375	426	292	383	269.3333333	367	0.518583065	0.334971081	0.922183948
107029	'Me2'	5106	5286	12188	2116	5892	8222	7526.666667	5410	-0.613946379	0.31067071	0.910971113
107035	'Fbxo38'	2170	2160	1608	1583	1840	2462	1979.333333	1961.666667	0.043819008	0.871059831	0.980126495
107045	'Lars'	2159	2162	2677	852	1694	1728	2332.666667	1424.666667	-0.739479028	0.034438974	0.445410735
107047	'Psmg2'	534	658	574	221	458	574	588.6666667	417.6666667	-0.521197865	0.134538627	0.734591725
107065	'Lrrtm2'	5	1	1	8	0	2	2.333333333	3.333333333	0.895345365	0.621200267	0.972790461
107071	'Wdr74'	427	439	727	278	439	645	531	454	-0.27980111	0.544504867	0.972790461
107094	'Rrp12'	1193	1053	418	420	661	870	888	650.3333333	-0.391013391	0.365834317	0.936406787
107173	'Gpr137'	361.76	392.55	190	344.61	471	410.11	314.77	408.5733333	0.470858772	0.190670333	0.813136091
107182	'Btaf1'	3249	3239	1386	1438	2467	2448	2624.666667	2117.666667	-0.245891005	0.512952231	0.972790461
107197	'Uqcc3'	266	311	307	147	303	320	294.6666667	256.6666667	-0.218886351	0.523084826	0.972790461
107221	'Ffar4'	0	1	10	1	0	1	3.666666667	0.666666667	-2.508634263	0.278644116	0.889355605
107227	'Macrod1'	101	91	91	117	96	344	94.33333333	185.6666667	0.963351192	0.080426363	0.617305615
107239	'Carns1'	290	314	66	45	234	116	223.3333333	131.6666667	-0.741398936	0.287272522	0.894554788
107242	'AI837181'	741	786	1010	507	736	881	845.6666667	708	-0.263031169	0.464579113	0.972790461
107250	'Kazald1'	60	72	7	88	262	258	46.33333333	202.6666667	2.175424303	0.003736605	0.140118993
107260	'Otub1'	1437	1457	1799	980	1459	1716	1564.333333	1385	-0.179488025	0.592149945	0.972790461
107271	'Yars'	1133	1167	3613	1633	1221	1936	1971	1596.666667	-0.312951214	0.638188855	0.972790461
107272	'Psat1'	833	848	2212	3285	853	1358	1297.666667	1832	0.670043657	0.406670747	0.957157474
107303348	'Gm45935'	15.72	13.97	35.1	18.22	9.77	78.6	21.59666667	35.53	0.617167009	0.510227293	0.972790461

107305	'Vps37c'	639	642	1497	984	534	735	926	751	-0.221918343	0.729989229	0.972790461
107321	'Lpxn'	110	137	29	19	74	22	92	38.33333333	-1.200076085	0.10618315	0.679124905
107328	'Trpt1'	64.26	71.71	96.27	30.33	83.34	67.24	77.41333333	60.30333333	-0.410721779	0.409087781	0.957157474
107338	'Gbf1'	2483	2238	1268	1514	1875	1784	1996.333333	1724.333333	-0.113420895	0.736842339	0.972790461
107351	'Kank1'	750	831	796	1468	1347	1323	792.3333333	1379.333333	0.897534954	0.018844812	0.341867915
107358	'Tm9sf3'	4054	3816	2955	3919	5307	6817	3608.333333	5347.666667	0.606376156	0.014618107	0.300778621
107368	'Pdzd8'	1415	1328	843	1152	1783	1847	1195.333333	1594	0.467447729	0.084963382	0.633939424
107371	'Exoc6'	191	173	204	153	241	351	189.3333333	248.3333333	0.373090058	0.309861448	0.910804861
107373	'Fam111a'	262	301	169	451	330	204	244	328.3333333	0.632603261	0.254464876	0.873895634
107375	'Slc25a45'	76	97	41	86	150	100	71.33333333	112	0.731549165	0.101333782	0.671759705
107392	'Brms1'	331	334	326	175	331	423	330.3333333	309.6666667	-0.11186257	0.73606187	0.972790461
107435	'Hat1'	2471	2490	1837	530	1676	2357	2266	1521	-0.631619628	0.170013127	0.785454025
107448	'Unc5a'	89	100	40	25	88	102	76.33333333	71.66666667	-0.105485456	0.85059387	0.975734242
107449	'Unc5b'	869	807	325	713	1206	1425	667	1114.666667	0.797087044	0.05356811	0.533767484
107476	'Acaca'	3498	3424	961	728	2749	1801	2627.666667	1759.333333	-0.554280274	0.335703499	0.922539021
107477	'Guca1b'	22	33	23	5	15	8	26	9.333333333	-1.467108408	0.028016864	0.40918746
107503	'Atf5'	670	580	7682	342	553	1467	2977.333333	787.3333333	-2.156310912	0.037952498	0.459930654
107508	'Eprs'	5741	5537	10886	3301	4096	7129	7388	4842	-0.661466043	0.190390626	0.813136091
107513	'Ssr1'	3470	3425	4831	2201	4021	5176	3908.666667	3799.333333	-0.087229311	0.825886744	0.972844676
107515	'Lgr4'	3508	3335	1083	5552	4630	4100	2642	4760.666667	1.041134964	0.052910922	0.532526438
107522	'Ece2'	24.64	32	15	1	27	16	23.88	14.66666667	-0.763012993	0.415076581	0.95722888
107526	'Gimap4'	192	162	18	1	182	200	124	127.6666667	-0.020359594	0.986891231	0.999493374
107527	'Il1rl2'	230	198	335	186	284	270	254.3333333	246.6666667	-0.052435873	0.902882386	0.987381169
107528	'Magee1'	380	346	435	515	437	419	387	457	0.337099494	0.445100166	0.969888068
107566	'Arl2bp'	1149	1173	817	208	1312	1135	1046.333333	885	-0.31565967	0.578494236	0.972790461
107568	'Wwp1'	1389	1460	1431	1025	1287	1440	1426.666667	1250.666667	-0.149942938	0.595140459	0.972790461
107569	'Nt5c3'	755	818	983	835	1048	1414	852	1099	0.374059545	0.288020213	0.895128365
107581	'Col16a1'	2115	1943	607	287	1376	1046	1555	903	-0.788973862	0.189914464	0.813027197
107585	'Dio3'	99	111	6	1	50	72	72	41	-0.86303539	0.476560594	0.972790461
107586	'Ovol2'	3	3	4	13	2	2	3.333333333	5.666666667	1.062211137	0.428410928	0.962191208
107587	'Osr2'	39	42	52	15	86	53	44.33333333	51.33333333	0.123526507	0.846365293	0.975201539
107589	'Mylk'	1928	1718	570	2225	1843	1291	1405.333333	1786.333333	0.557315505	0.334249743	0.921648675
107605	'Rdh1'	25	23	2	94.44	21	46	16.66666667	53.81333333	2.019256297	0.04314325	0.484697385
107607	'Nod1'	384	392	320	967	490	497	365.3333333	651.3333333	1.037695081	0.062422494	0.566066012
107626	'Asmt'	1	0	0	0	1	0	0.333333333	0.333333333	0.058500858	0.988561293	0.999493374
107650	'Pi4kb'	1426.8	1455.7	1200.9	916.67	1472.4	1544.54	1361.1	1311.193333	-0.027302736	0.905853015	0.988794924
107652	'Uap1'	924	947	2863	1697	684	986	1578	1122.333333	-0.389396712	0.606121498	0.972790461
107656	'Krt9'	6	6	6	3	2	0	6	1.666666667	-1.668430405	0.228155864	0.854550688
107684	'Coro2a'	52	46	43	37	55	52	47	48	0.069851563	0.86154116	0.976863639
107686	'Snrpd2'	982	1007	1066	659	679	1001	1018.333333	779.6666667	-0.347636624	0.303618092	0.906520549

107701	'Sf3b4'	857	898	959	697	818	1136	904.6666667	883.6666667	-0.010116861	0.974939753	0.999493374
107702	'Rnh1'	1268	1333	3241	1211	1597	1857	1947.333333	1555	-0.368829817	0.510286683	0.972790461
107723	'Slc12a6'	2387	2291	687	1665	2758	2578	1788.333333	2333.666667	0.473899666	0.323381478	0.918976057
107732	'Mrpl10'	793	821	844	712	675	869	819.3333333	752	-0.059132277	0.864963921	0.978035808
107733	'Mrpl41'	270	268	206	180	319	345	248	281.3333333	0.20077111	0.468241946	0.972790461
107734	'Mrpl30'	1002	1072	1231	908	736	1158	1101.666667	934	-0.179556312	0.656523688	0.972790461
107746	'Rapgef1'	2320	2299	2142	1999	2300	2374	2253.666667	2224.333333	0.046396408	0.87365734	0.980171118
107747	'Aldh1l1'	150	144	108	114	128	229	134	157	0.263115625	0.477876771	0.972790461
107751	'Prrx11'	6	8	2	1	4	2	5.333333333	2.333333333	-1.146709375	0.357572213	0.932505998
107753	'Lgals2'	24	28	1	1	11	9	17.66666667	7	-1.327321015	0.279946272	0.889355605
107765	'Ankrd1'	7	15	114	9	18	14	45.33333333	13.66666667	-1.882174265	0.087206806	0.640887434
107766	'Haao'	22	31	1	1	18	6	18	8.333333333	-1.098487712	0.392558314	0.952648938
107767	'Scamp1'	923	867	865	1518	1123	1740	885	1460.333333	0.812672023	0.039483323	0.463654682
107769	'Tm6sf1'	90.84	93.04	67.12	2199.1	135.04	750.98	83.66666667	1028.37	3.964679779	2.65E-05	0.005255191
107770	'Tm6sf2'	17	20	3	5	11	7	13.33333333	7.666666667	-0.705690872	0.443104868	0.96975311
107771	'Bmyc'	1029	1189	470	117	1745	1315	896	1059	0.167410022	0.828070983	0.973238667
107815	'Scml2'	1665	1596	572	16	975	893	1277.666667	628	-1.115912468	0.26673705	0.883600995
107817	'Jmjd6'	870	760	5626	988	592	952	2418.666667	844	-1.547031918	0.082460164	0.62310518
107823	'Nsd2'	3667	3752	1586	1079	2761	2548	3001.666667	2129.333333	-0.472351195	0.262662029	0.88013169
107829	'Thoc5'	865	900	554	308	769	804	773	627	-0.306148109	0.390832803	0.952178913
107831	'Adgrb1'	114	103	12	11	132	87	76.33333333	76.66666667	-0.010995132	0.990609631	0.999493374
107869	'Cth'	124	144	304	77	110	124	190.6666667	103.6666667	-0.922039325	0.099171055	0.668798985
107885	'Mthfs'	110.19	127	193.32	58.67	94.42	77.78	143.5033333	76.95666667	-0.916426198	0.05415165	0.534539595
107889	'Gcm2'	2	1	0	0	3	4	1	2.333333333	1.173261034	0.589376337	0.972790461
107895	'Mgat5'	354	349	51	398	701	607	251.3333333	568.6666667	1.286971549	0.048941035	0.514354314
107932	'Chd4'	11425	11214	5162	12113	11996	12420	9267	12176.33333	0.527807236	0.192133489	0.813837363
107934	'Celsr3'	83	76	42	20	31	10	67	20.33333333	-1.600101541	0.010095021	0.248609512
107939	'Pom121'	1453.7	1380.9	924.86	1209.4	1451.6	1497.83	1253.16	1386.263333	0.229452369	0.429095886	0.962386546
107951	'Cdk9'	1478	1583	1593	1707	1628	1707	1551.333333	1680.666667	0.19892097	0.582952679	0.972790461
107970	'H1f6'	1	2	1	1	0	0	1.333333333	0.333333333	-1.817755807	0.471982145	0.972790461
107971	'Frs3'	181	195	131	250	163	156	169	189.6666667	0.343316025	0.495246397	0.972790461
107975	'Pacs1'	2612	2375	2595	435	2040	2672	2527.333333	1715.666667	-0.6643168	0.232469878	0.859679981
107976	'Babam2'	431	442	474	354	443	569	449	455.3333333	0.04291153	0.89163789	0.985171
107986	'Ddb2'	571.09	575.89	367.34	108.92	367.41	312.32	504.7733333	262.8833333	-0.963691942	0.022511036	0.372480251
107993	'Bfsp2'	2	1	1	1	1	5	1.333333333	2.333333333	0.781826564	0.637404952	0.972790461
107995	'Cdc20'	530	587	202	142	446	351	439.6666667	313	-0.468808886	0.349280097	0.928823768
107999	'Gtpbp6'	231.83	247.79	740.81	327.83	315.84	382.64	406.81	342.1033333	-0.270080871	0.67805246	0.972790461
108000	'Cenpf'	3532.8	3885	496	88	1748	1355	2637.946667	1063.666667	-1.351019933	0.14961051	0.758505335
108011	'Ap4e1'	1355	1304	965	729	1188	734	1208	883.6666667	-0.379556902	0.272388589	0.884738928
108012	'Ap1s2'	907	991	688	1012	812	534	862	786	0.041073346	0.933940414	0.994249017

108013	'Celf4'	136	113	87	81	92	94	112	89	-0.250224073	0.492339492	0.972790461
108014	'Srsf9'	1052	1060	1200	976	873	1386	1104	1078.333333	0.012392438	0.973720843	0.999493374
108015	'Chrnb4'	2	1	1	2	4	6	1.333333333	4	1.58106555	0.25879801	0.877564912
108017	'Fxyd4'	4	4	1	19	0	5	3	8	1.823914093	0.260356862	0.878537553
108030	'Lin7a'	40	29	34	9	14	28	34.33333333	17	-1.035171186	0.079838826	0.617305615
108037	'Shmt2'	1032	894	3681	1246	1081	1528	1869	1285	-0.575590223	0.417302042	0.95771927
108043	'Chrnb3'	0	0	0	3	0	0	0	1	2.899270347	0.465759809	0.972790461
108052	'Slc14a1'	37	56	1	71	18	30	31.33333333	39.66666667	0.699287485	0.544188389	0.972790461
108058	'Camk2d'	856	925	2360	759	869	1228	1380.333333	952	-0.583084676	0.3217651	0.917918531
108062	'Cstf2'	3669.9	3649.6	2904.1	558.77	2516.1	2539.8	3407.856667	1871.563333	-0.933940353	0.059367612	0.553094905
108067	'Eif2b3'	962	944	583	277	646	654	829.6666667	525.6666667	-0.651651521	0.055310026	0.539316735
108068	'Grm2'	2	1	0	1	0	0	1	0.333333333	-1.330404752	0.683404215	0.972790461
108069	'Grm3'	3	2	1	0	0	1	2	0.333333333	-2.380782061	0.310154651	0.910971113
108071	'Grm5'	7	10	2	0	7	6	6.333333333	4.333333333	-0.594347317	0.657917807	0.972790461
108072	'Grm6'	6	4	2	0	5	1	4	2	-1.03451346	0.513234397	0.972790461
108073	'Grm7'	7	3	10	1	3	6	6.666666667	3.333333333	-1.103215346	0.344938508	0.926100052
108075	'Ltbp4'	2455	2201	525	460	2803	2698	1727	1987	0.181515401	0.795439949	0.972790461
108077	'Skiv2l'	1238	1272	699	799	1276	1272	1069.666667	1115.666667	0.118256623	0.692565461	0.972790461
108078	'Olr1'	1	3	192	287	2	8	65.33333333	99	0.854182892	0.719564279	0.972790461
108079	'Prkaa2'	150	146	234	255	167	216	176.6666667	212.6666667	0.355349196	0.506845122	0.972790461
108083	'Pip4k2b'	1229	1244	913	932	1113	1181	1128.666667	1075.333333	0.003719606	0.989055094	0.999493374
108086	'Rnf216'	3620	3489	1804	1550	2215	3099	2971	2288	-0.327507777	0.342683968	0.925308103
108089	'Rnf144a'	1300	1209	1867	1136	1616	1722	1458.666667	1491.333333	0.027319545	0.944615196	0.996592403
108096	'Slco1a5'	2	4	1	1	7	4	2.333333333	4	0.76870813	0.572275866	0.972790461
108097	'Prkab2'	1031	920	1420	515	708	1066	1123.666667	763	-0.586383933	0.155598232	0.766779052
108098	'Med21'	776	888	1044	218	519	650	902.6666667	462.3333333	-1.027116238	0.014042274	0.293375506
108099	'Prkag2'	719	772	517	140	1071	978	669.3333333	729.6666667	0.039533181	0.949108094	0.997742943
108100	'Baiap2'	1087	1011	476	578	734	418	858	576.6666667	-0.432300811	0.356118614	0.931977458
108101	'Fermt3'	40.74	49.29	10.73	19.67	49.66	72.76	33.58666667	47.36333333	0.518857057	0.4544242	0.970649024
108105	'B3gnt5'	209	200	307	54	182	114	238.6666667	116.6666667	-1.098730849	0.031765846	0.432926355
108112	'Eif4ebp3'	127	110	168	67	74	53	135	64.66666667	-0.999900129	0.044680123	0.492557243
108114	'Slc22a7'	1	2	1	0	2	0	1.333333333	0.666666667	-1.017906319	0.681666644	0.972790461
108115	'Slco4a1'	0	2	33	1265	14	381	11.66666667	553.3333333	5.720614607	0.01051881	0.254108784
108116	'Slco3a1'	127	171	165	1224	193	294	154.3333333	570.3333333	2.200738928	0.009932159	0.245872663
108121	'U2af1'	1178	1252	1927	760	1090	1287	1452.333333	1045.666667	-0.493147346	0.235294416	0.861636582
108123	'Napg'	718	758	768	1603	842	932	748	1125.666667	0.762771064	0.156288432	0.767792163
108124	'Napa'	1246	1315	2646	1007	1410	1908	1735.666667	1441.666667	-0.317660216	0.530384383	0.972790461
108138	'Xrcc4'	311	310	254	52	272	277	291.6666667	200.3333333	-0.620072166	0.254996811	0.874595535
108143	'Taf9'	1543.5	1593.7	1942.7	716.44	1074.8	1310	1693.293333	1033.753333	-0.719433585	0.033127638	0.438703227
108147	'Atic'	1731	1677	1819	482	1542	1814	1742.333333	1279.333333	-0.519074276	0.243288044	0.865044378

108148	'Galnt2'	1747	1763	1054	2054	2470	2810	1521.333333	2444.666667	0.764822063	0.009496296	0.240190842
108150	'Galnt7'	833	924	287	1295	1480	1303	681.3333333	1359.333333	1.138583132	0.018009076	0.335354139
108151	'Sema3d'	108	120	177	163	272	216	135	217	0.680445334	0.123461822	0.715310266
108153	'Adamts7'	396	391	614	1364	480	400	467	748	0.904789591	0.219352457	0.846595396
108154	'Adamts6'	197	214	99	107	178	105	170	130	-0.2921842	0.507596709	0.972790461
108155	'Ogt'	10627	10183	3452	6003	14749	6648	8087.333333	9133.333333	0.256108911	0.629563089	0.972790461
108156	'Mthfd1'	656	679	427	384	755	836	587.3333333	658.3333333	0.180764785	0.552028678	0.972790461
108159	'Ubxn8'	492.41	469.39	166.25	88.1	494.25	456.08	376.0166667	346.1433333	-0.148378	0.811953564	0.972790461
108160	'Fam50a'	652	699	1746	886	725	1236	1032.333333	949	-0.136423311	0.821891963	0.972790461
108161	'Fam50b'	1	4	0	1	0	1	1.666666667	0.666666667	-1.089648986	0.659232279	0.972790461
108167321	'Gm10479'	8.36	6.25	18.95	6.28	20.92	14.84	11.18666667	14.01333333	0.228801482	0.791621816	0.972790461
108167370	'Gm45965'	0	1.5	0	0	1	3	0.5	1.333333333	1.750813674	0.584138459	0.972790461
108167412	'Gm45975'	1	1	0	1	0	3	0.666666667	1.333333333	1.086821783	0.666577752	0.972790461
108167415	'Gm45978'	0	0	0	1.38	1.39	0	0	0.923333333	2.050872943	0.610671515	0.972790461
108167434	'Gm45988'	36.82	20.14	0	0	0	0	18.98666667	0	-6.499968603	0.004840079	0.164614637
108167437	'Gm44812'	1	2	0	3	3	4	1	3.333333333	1.857782572	0.256457676	0.876779958
108167466	'Gm45618'	1	0	1	0	0	0	0.666666667	0	-1.858475606	0.644939423	0.972790461
108167506	'Gm46022'	3	0	0	2	1	0	1	1	0.35933736	0.905151243	0.988451813
108167548	'Gm45855'	920.22	722.61	1652	2096.8	1128.1	15.61	1098.27	1080.156667	0.212315319	0.860878377	0.976863639
108167553	'LOC108167553'	247.9	236.31	59.73	241.93	179.37	51.61	181.3133333	157.6366667	0.082800414	0.917528427	0.990547571
108167560	'Gm46058'	27	20	4	13	31	5	17	16.33333333	0.075846527	0.938152221	0.994698142
108167565	'Gm17149'	8.79	9.37	1	7.98	3	9.05	6.386666667	6.676666667	0.242553411	0.829841157	0.973764972
108167594	'LOC108167594'	1	0	0	3	0	0	0.333333333	1	1.937497013	0.625951132	0.972790461
108167670	'Gm17415'	4	8	6	7	4	2	6	4.333333333	-0.293329947	0.790336187	0.972790461
108167700	'Gm46139'	77.54	63.05	9.96	8.74	66.11	29.14	50.18333333	34.66333333	-0.521782116	0.565278584	0.972790461
108167775	'Gm8112'	1.52	0	0	0	0	0	0.506666667	0	-0.903279821	0.824807108	0.972790461
108167789	'Gm10310'	12.96	16.68	0	0	16.5	15.48	9.88	10.66	0.110229939	0.947844613	0.997201139
108167848	'Gm12258'	391	423	118	104	386	287	310.6666667	259	-0.243598158	0.671862971	0.972790461
108167860	'Efcab13'	10	10	2	0	3	0	7.333333333	1	-2.849613441	0.09929154	0.668798985
108167871	'Gm11703'	674.91	838.2	1750.2	4.5	3.01	3.62	1087.76	3.71	-8.349921202	6.07E-28	5.77E-24
108167874	'Gm12586'	27	69.06	11	0	6	2	35.68666667	2.666666667	-3.732754761	9.04E-04	0.062744556
108167910	'Gm12712'	5.64	4.48	0	5.96	5.02	2	3.373333333	4.326666667	0.634595413	0.664272452	0.972790461
108167918	'Gm46290'	166.2	172.11	90.49	120.65	192.58	89.83	142.9333333	134.3533333	0.014255738	0.976474515	0.999493374
108167925	'Gm46294'	5	4	2	4	4.03	2	3.666666667	3.343333333	0.004373384	0.997071628	0.9996806
108167945	'Gm46305'	35.71	21.73	36.9	9.23	24.83	29.77	31.44666667	21.27666667	-0.624874552	0.312821415	0.911912367
108167961	'Gm46319'	2	0	1	0	0	1	1	0.333333333	-1.433748416	0.667550462	0.972790461
108167995	'Gm46345'	1	2	0	3	0	0	1	1	0.528096191	0.860012397	0.976863639
108168003	'Gm46353'	0	0	3.31	0	0	0	1.103333333	0	-2.588571306	0.51580235	0.972790461
108168034	'Gm46382'	53	49	13	4	66	21	38.33333333	30.33333333	-0.364411299	0.697901103	0.972790461
108168061	'Gm46399'	1.13	2	2.41	2	0	1.13	1.846666667	1.043333333	-0.551734175	0.776763929	0.972790461

108168084 'Gm46415'	4	3	1	10	4	0	2.666666667	4.666666667	1.167864455	0.475517129	0.972790461
108168096 'Gm46425'	0	1	0	0	0	9.88	0.333333333	3.293333333	2.882818727	0.416771715	0.95722888
108168098 'Gm46427'	9.02	8	0	0	0	7	5.673333333	2.333333333	-1.338691219	0.566893927	0.972790461
108168101 'Gm46430'	1041.2	1172.9	638.91	306.15	1358	722.93	950.9933333	795.6766667	-0.272942323	0.587689194	0.972790461
108168114 'Gm5451'	9.35	11.6	9.52	15.24	2.33	1.14	10.15666667	6.236666667	-0.330422306	0.794932652	0.972790461
108168140 'Gm46442'	1	0	0	1	0	0	0.333333333	0.333333333	0.058500858	0.988561293	0.999493374
108168145 'Gm5459'	2.72	2.96	0	8.38	2.11	5	1.893333333	5.163333333	2.164973419	0.158823096	0.768918948
108168148 'Gm5798'	1	3.12	0	0	0	1	1.373333333	0.333333333	-1.735451621	0.587572465	0.972790461
108168152 'Gm8126'	0	4.59	0	1	4	0	1.53	1.666666667	0.452660191	0.872986876	0.980171118
108168155 'Gm5799'	3.37	0.44	0	0	0	1	1.27	0.333333333	-1.33025371	0.739510174	0.972790461
108168176 'Gm10338'	0	1	0	0	0	0	0.333333333	0	-0.903279821	0.824807108	0.972790461
108168208 'Gm45521'	0	1	0	0	0	0	0.333333333	0	-0.903279821	0.824807108	0.972790461
108168218 'Gm5218'	3	0	0	1	0	3	1	1.333333333	0.509974864	0.862772307	0.977220339
108168301 'Gm46562'	0	2	0	0	0	0	0.666666667	0	-1.695595436	0.675304306	0.972790461
108168308 'Smim34'	0	0	0	2	2	1	0	1.666666667	3.309657169	0.252628325	0.871252558
108168333 'Gm15946'	3	1	0	1	0	0	1.333333333	0.333333333	-1.744289904	0.575380654	0.972790461
108168336 'Gm46592'	0	1.09	1.13	12.88	1.24	0	0.74	4.706666667	3.08940837	0.17497738	0.793765197
108168358 'Gm46608'	76	72	44	42	69	74	64	61.66666667	-0.010529801	0.978234503	0.999493374
108168363 'Gm17669'	3.19	0	2.68	1	1	2	1.956666667	1.333333333	-0.322148131	0.865198148	0.978040999
108168367 'LOC108168367'	0	1.74	1.39	1.79	3.76	1.69	1.043333333	2.413333333	1.307704765	0.530467353	0.972790461
108168373 'Gm46617'	25	15	4	8	18	12	14.66666667	12.66666667	-0.131979514	0.874474114	0.980175262
108168387 'Gm46629'	4	5	0	12	4	3	3	6.333333333	1.423234874	0.323815714	0.919383393
108168395 'Gm45871'	314.32	328	298.48	217.01	452.98	326.02	313.6	332.0033333	0.095625474	0.769596169	0.972790461
108168411 'Gm46650'	0	0	0	1	1	0	0	0.666666667	2.050872943	0.610671515	0.972790461
108168446 'Mageb17-ps'	6	14	0	0	0	8	6.666666667	2.666666667	-1.379105787	0.553164081	0.972790461
108168448 'Gm9009'	1	0	1	0	0	0	0.666666667	0	-1.858475606	0.644939423	0.972790461
108168453 'Gm15262'	93	79	30	1	28	24	67.33333333	17.66666667	-1.992083893	0.024996075	0.391454179
108168681 'LOC108168681'	0	0	1	0	0	0	0.333333333	0	-0.903279821	0.824807108	0.972790461
108168684 'LOC108168684'	0	1	0	0	0	0	0.333333333	0	-0.903279821	0.824807108	0.972790461
108168685 'LOC108168685'	0	0	0	1	0	0	0	0.333333333	1.020273531	0.802557913	0.972790461
108168727 'Gm46725'	2	1	0	0	3	2	1	1.666666667	0.707887742	0.757064231	0.972790461
108168740 'Gm46731'	63.16	38.58	17.84	48.84	51.09	16.18	39.86	38.70333333	0.165884536	0.827397082	0.973179493
108168770 'Gm13653'	55.23	52.4	147.61	0	0	0	85.08	0	-8.87713825	8.98E-10	8.13E-07
108168771 'Gm50595'	2	2	0	2	1	0	1.333333333	1	-0.077696988	0.973639762	0.999493374
108168799 'Gm11686'	1	0	0	0	0	0	0.333333333	0	-0.903279821	0.824807108	0.972790461
108168809 'Gm14279'	382.88	274.68	611.15	1	0	0	422.9033333	0.333333333	-10.20418765	1.52E-14	3.62E-11
108168824 'Gm14439'	2.02	4	4	3	6	1	3.34	3.333333333	0.051268783	0.968506788	0.999493374
108168832 'Gm13981'	0	0.56	0.98	1	3	0	0.513333333	1.333333333	2.915122243	0.463241805	0.972790461
108168884 'Gm37500'	15	21	4	1	17	4	13.33333333	7.333333333	-0.866635119	0.445923646	0.969915971
108168963 'Zfp978'	59.12	65.52	24.53	1.03	20.72	6.24	49.72333333	9.33	-2.479170497	0.005717025	0.178183733

108169021	'Gm46900'	2	3	2	1	3	1	2.333333333	1.666666667	-0.45688159	0.760866558	0.972790461
108169040	'Gm42935'	1	1	0	0	1	2	0.666666667	1	0.547742134	0.835241607	0.974723675
108169043	'Gm46911'	116.21	103.7	57.73	192.17	256.17	111.56	92.54666667	186.6333333	1.157332981	0.028978372	0.415174688
108169053	'Gm15682'	261.27	444.96	514.1	0	1.62	0	406.7766667	0.54	-10.12622939	8.53E-15	2.32E-11
108169054	'Gm46915'	1	4	0	0	0	1	1.666666667	0.333333333	-2.054947434	0.504125723	0.972790461
108169060	'Gm50598'	15	17	2	6	20	11	11.33333333	12.33333333	0.185792565	0.843556197	0.975182082
108169063	'Gm43247'	1.49	6.19	17.21	1.34	0	0	8.296666667	0.446666667	-4.545051935	0.02154527	0.363782119
108169072	'Gm6139'	3644.7	3160	7155.2	2452.9	1343.4	80.38	4653.326667	1292.216667	-1.62536517	0.119636216	0.710007992
108169096	'Gm46933'	6.01	3.01	1	1	3	5	3.34	3	-0.141986636	0.916072454	0.990491125
108169097	'Pramel44'	0	0	0	0	0	1	0	0.333333333	1.020273531	0.802557913	0.972790461
108169101	'Gm46935'	0	2	0	0	0	0	0.666666667	0	-1.695595436	0.675304306	0.972790461
108169152	'Gm46965'	70	51	26	28	42	21	49	30.33333333	-0.575372223	0.327785965	0.921056277
108169197	'Gm10420'	422.44	324.29	871.85	126.37	104.37	2.28	539.5266667	77.67333333	-2.640060147	0.018674079	0.340229851
108645	'Mat2b'	1245	1345	1654	1257	1318	2435	1414.666667	1670	0.238740743	0.555118849	0.972790461
108652	'Slc35b3'	358	349	210	181	343	312	305.6666667	278.6666667	-0.09445594	0.764600871	0.972790461
108653	'Rimklb'	1953	2018	718	413.01	1499	1330	1563	1080.67	-0.53136309	0.307609256	0.907963267
108654	'Fam210a'	1361.9	1380	2419.7	1336.1	1253.5	1849.31	1720.523333	1479.646667	-0.206490802	0.666435565	0.972790461
108655	'Foxp1'	1092	1082.9	2000.4	292.63	1098.1	958.11	1391.753333	782.95	-0.943717345	0.088748667	0.64452174
108657	'Rnpepl1'	840	888	796	136	1017	988	841.3333333	713.6666667	-0.344031116	0.578459869	0.972790461
108660	'Rnf187'	2560	2808	2134	1276	2656	3115	2500.666667	2349	-0.099717132	0.742574037	0.972790461
108664	'Atp6v1h'	2647	2579	2481	1987	1516	2615	2569	2039.333333	-0.257640097	0.50171426	0.972790461
108670	'Epsti1'	10	11	16	0	12	12	12.33333333	8	-0.785706374	0.489559085	0.972790461
108671	'Dnajc9'	783	778	429	351	676	670	663.3333333	565.6666667	-0.194057226	0.548741934	0.972790461
108672	'Zdhhc15'	279	298	126	37	308	377	234.3333333	240.6666667	-0.032827639	0.962922161	0.999493374
108673	'Ccdc86'	511.97	432.94	393	248.56	399	554.97	445.97	400.8433333	-0.149796118	0.628593204	0.972790461
108679	'Cops8'	1804	1853	1314	723	1466	1907	1657	1365.333333	-0.28659275	0.377177266	0.941504733
108682	'Gpt2'	799	829	956	1024	829	1153	861.3333333	1002	0.290458456	0.471606059	0.972790461
108686	'Ccdc88a'	1832	1900	858	477	2001	1368	1530	1282	-0.264810107	0.599173891	0.972790461
108687	'Edem2'	895	965	543	476	840	1192	801	836	0.076683136	0.825258929	0.972790461
108689	'Stn1'	343	397	195	115	366	320	311.6666667	267	-0.227843707	0.604197937	0.972790461
108699	'Chn1'	619	554	426	36	374	310	533	240	-1.247480224	0.065743483	0.574593252
108705	'Pttg1ip'	2380	2268	2364	3399	3185	3851	2337.333333	3478.333333	0.648222942	0.060808741	0.559852676
108707	'Fam207a'	595	628	693	465	577	722	638.6666667	588	-0.096504746	0.764624537	0.972790461
108723	'Card11'	7	2	1	3	13	6	3.333333333	7.333333333	1.163113854	0.3319203	0.921648675
108735	'Sft2d2'	2020	1970	1363	2873	2768	3867	1784.333333	3169.333333	0.914155966	0.004160745	0.149677594
108737	'Oxsr1'	2063	1958	2345	1592	1891	2239	2122	1907.333333	-0.122857558	0.70809892	0.972790461
108755	'Lyrm2'	450	446	187	35	493	347	361	291.6666667	-0.376418097	0.622202686	0.972790461
108760	'Galnt16'	94	94	67	20	89	89	85	66	-0.416122449	0.436815461	0.96622803
108767	'Pnrc1'	1885	1849	12233	4625	1622	2673	5322.333333	2973.333333	-0.780850405	0.404224302	0.956545617
108797	'Mex3b'	770	833	1178	193	906	527	927	542	-0.867133286	0.115750515	0.702825844

108800	'Ston2'	546	490	62	735	400	564.88	366	566.6266667	0.874449043	0.252223939	0.871252558
108802	'Calr4'	3	7	5	107	7	41	5	51.66666667	3.67212001	9.25E-04	0.063607704
108803	'Spem2'	2	0	0	2	0	0	0.666666667	0.666666667	0.529257145	0.894071384	0.985171
108811	'Ccdc122'	257	293	99	44	187	213	216.3333333	148	-0.569242448	0.336373744	0.92281928
108812	'Flacc1'	4	4	1	1	4	0	3	1.666666667	-0.760672873	0.6596888	0.972790461
108829	'Jmjd1c'	3564.7	3684.7	2985.3	2350.3	3148	2906.29	3411.563333	2801.523333	-0.222505589	0.391865708	0.952648938
108832	'Tmem74b'	338.16	253.02	397.08	11	97.05	110.19	329.42	72.74666667	-2.323324246	8.82E-04	0.061899009
108837	'Ibtk'	2656	2765	1998	931	2063	2279	2473	1757.666667	-0.499287142	0.107900234	0.684280429
108841	'Rdh13'	425	340	455	459	412	399	406.6666667	423.3333333	0.145710679	0.735073301	0.972790461
108853	'Mtrf1l'	241	239	221	215	263	366	233.6666667	281.3333333	0.298886469	0.326915073	0.920734892
108857	'Ankhd1'	3649	3652	2555	2831	2466	2095	3285.333333	2464	-0.270715587	0.510668507	0.972790461
108888	'Atad3a'	945	956	1043	433	870	962	981.3333333	755	-0.403149051	0.221345595	0.849403976
108897	'Aif1l'	160	148	437	113	142	131	248.3333333	128.6666667	-0.977218655	0.119914798	0.710102513
108899	'2700081O15Rik'	2606	2661	1580	939	2803	1613	2282.333333	1785	-0.339683803	0.408940959	0.957157474
108900	'Fam72a'	88	94	66	28	36	42	82.66666667	35.33333333	-1.174286332	0.002246114	0.106226643
108902	'B4gat1'	419	434	158	319	656	734	337	569.6666667	0.797389258	0.071924113	0.593728759
108903	'Tbcd'	1254.3	1301	425.23	828.72	1251.3	1601.68	993.51	1227.246667	0.375846679	0.406220171	0.957157474
108907	'Nusap1'	410	486	154.38	50	434	355	350.1266667	279.6666667	-0.369545428	0.599098333	0.972790461
108909	'Aida'	1380.5	1396.2	1591.5	503.83	1222.7	1370.71	1456.066667	1032.42	-0.546783138	0.150766733	0.759755159
108911	'Rcc2'	2818	2940	3021	2109	3285	4697	2926.333333	3363.666667	0.188391691	0.555766466	0.972790461
108912	'Cdca2'	601	602	110	39	397	422	437.6666667	286	-0.653484474	0.429263572	0.962386546
108927	'Lhfp'	366	337	537	408	459	507	413.3333333	458	0.171195696	0.688208905	0.972790461
108934	'Smim13'	611	604	562	1075	633	629	592.3333333	779	0.568145757	0.26852084	0.883600995
108937	'Rnf169'	1404.1	1283.2	1069	988	1231.9	1169	1252.066667	1129.62	-0.077445921	0.781741791	0.972790461
108943	'Trmt10a'	1795	1957	621	637	738	499	1457.666667	624.6666667	-1.063057746	0.037780361	0.459930654
108946	'Zzz3'	2457	2494	2169	1723	2627	2617	2373.333333	2322.333333	0.002073442	0.992919943	0.999493374
108954	'Ppp1r15b'	1818	1788	1773	1988	1909	2403	1793	2100	0.297777634	0.368678935	0.937530612
108956	'Apol7c'	1	0	1	0	1	1	0.666666667	0.666666667	-0.113177234	0.967544413	0.999493374
108958	'Miga2'	380	392	217	489	569	492	329.6666667	516.6666667	0.759167318	0.036517435	0.457518227
108960	'Irak2'	169	169	372	235	220	248	236.6666667	234.3333333	0.004257692	0.993961108	0.999562152
108961	'E2f8'	164	166	34	92	111	87	121.3333333	96.66666667	-0.171761766	0.782515375	0.972790461
108978	'4930555G01Rik'	9.65	5.49	2.1	1	2.35	2.06	5.746666667	1.803333333	-1.614396385	0.216969426	0.843391934
108989	'Tpr'	4972.1	4963.1	4852.8	3499	4765.2	5119	4929.326667	4461.05	-0.109770993	0.673160063	0.972790461
108995	'Tbc1d10c'	42	54	12	7	35	22	36	21.33333333	-0.738102286	0.330592709	0.921648675
109006	'Ciapin1'	754	830	609	313	504	685	731	500.6666667	-0.534265966	0.065140337	0.573355596
109019	'Nabp1'	1638	1650	706	345	654	882	1331.333333	627	-1.057938925	0.010639859	0.255084633
109032	'Sp110'	7.75	8	11.51	30.25	11	38.43	9.086666667	26.56	1.679619633	0.037433697	0.459930654
109042	'Cavin3'	485	447	2185	498	501	722	1039	573.6666667	-0.930019276	0.221178922	0.849153089
109050	'Inka2'	42	44	37	90	77	66	41	77.66666667	1.046494035	0.03359094	0.442492678
109052	'Krt75'	2	2	1	0	0	0	1.666666667	0	-3.08654517	0.29806771	0.901330779

109054	'Pfdn4'	172	212	460	394	208	319	281.3333333	307	0.197648391	0.763958666	0.972790461
109065	'Dnaaf2'	458	528	468	237	393	511	484.6666667	380.3333333	-0.352498959	0.249551857	0.869928149
109075	'Exosc4'	346.95	393.9	493.85	387.9	374.96	478.97	411.5666667	413.9433333	0.051607455	0.899112843	0.986106073
109077	'Ints5'	863	857	760	683	719	952	826.6666667	784.6666667	-0.016152084	0.956763597	0.999493374
109079	'Sephs1'	1519	1495	549	686	1477	1207	1187.666667	1123.333333	-0.022619384	0.959076186	0.999493374
109082	'Fbxw17'	394	415	175	198	509	429	328	378.6666667	0.235108231	0.583764797	0.972790461
109093	'Rars2'	741	798	342	132	632	708	627	490.6666667	-0.394902721	0.488785507	0.972790461
109095	'Rbm15b'	1391	1429	1139	1262	1724	1671	1319.666667	1552.333333	0.290431263	0.241587596	0.86449675
109108	'Slc30a9'	1934	1932	1843	1915	1970	2186	1903	2023.666667	0.162113995	0.610274668	0.972790461
109113	'Uhrf2'	1767	1788	3637	1676	1683	1709	2397.333333	1689.333333	-0.481889549	0.358074644	0.933074991
109115	'Supt3'	795	694	751	106	360	489	746.6666667	318.3333333	-1.310902017	0.007071611	0.198883838
109129	'Mmadhc'	1314	1337	2156	1322	1179	1452	1602.333333	1317.666667	-0.239530604	0.606922535	0.972790461
109135	'Plekha5'	2026	2012	1450	305	1556	1634	1829.333333	1165	-0.722984078	0.175598013	0.794684462
109136	'Mmaa'	1066	1023	341	334	780	434	810	516	-0.574251259	0.259207429	0.877955237
109145	'Gins4'	526	544	492	353	448	653	520.6666667	484.6666667	-0.081185614	0.78499954	0.972790461
109151	'Chd9'	2112.7	2213.7	1232	829.19	1764	1623.8	1852.8	1405.663333	-0.371120625	0.251297958	0.871230154
109154	'Mlec'	6292	6241	3201	5953	6403	9772	5244.666667	7376	0.571219807	0.111390836	0.695488533
109161	'Ube2q2'	1445	1473	1134	1328	1270	1997	1350.666667	1531.666667	0.247379194	0.441221407	0.968551476
109168	'Atl3'	3916	4176	1469	1537	4011	4084	3187	3210.666667	0.033462412	0.943000648	0.996301887
109169	'Igip'	60.99	62.08	175.79	1.01	42.05	29.2	99.62	24.08666667	-2.262693264	0.023581257	0.380103384
109181	'Trip11'	1576	1627	1107	1026	1620	1422	1436.666667	1356	-0.028380122	0.912969674	0.989773583
109205	'Sobp'	558	494	343	358	717	720	465	598.3333333	0.383043043	0.204746674	0.833294914
109212	'Pimreg'	122	124	31	24	118	70	92.33333333	70.66666667	-0.372077116	0.583831837	0.972790461
109218	'Tmem139'	12	20	6	50	17	10	12.66666667	25.66666667	1.349883554	0.160570458	0.771114735
109222	'Rarres1'	19	11	4	22	23	43	11.33333333	29.33333333	1.449802928	0.044615414	0.492557243
109225	'Ms4a7'	17	14	4	5	19	65	11.66666667	29.66666667	1.286486301	0.184387399	0.804780995
109229	'Fam118b'	426	410	185	334	403	374	340.3333333	370.3333333	0.235039554	0.557767451	0.972790461
109232	'Sccpdh'	804	862	1156	182	791	884	940.6666667	619	-0.71789079	0.191207983	0.813837363
109241	'Mbd5'	975	982	623	665	704	709	860	692.6666667	-0.207044783	0.533591315	0.972790461
109242	'Kif24'	675.02	646	98	98	257	271.02	473.0066667	208.6733333	-1.129594646	0.096350564	0.662981155
109245	'Lrrc39'	34.25	46.78	13.17	32.54	46.15	20.37	31.4	33.02	0.220167225	0.744198459	0.972790461
109246	'Tspan9'	1240	1225	1070	1624	1461	1679	1178.333333	1588	0.527845581	0.128691638	0.72577634
109254	'Adtrp'	14	11	6	53	12	12	10.33333333	25.66666667	1.649430901	0.092469152	0.653540343
109263	'Rlf'	3172	3060	4638	1547	2227	2237	3623.333333	2003.666667	-0.856220205	0.029015594	0.41528417
109264	'Me3'	108.61	148.68	74.53	65.92	125.3	48.14	110.6066667	79.78666667	-0.382957426	0.469205561	0.972790461
109267	'Ssc4d'	147	145	39	220	176	278	110.3333333	224.6666667	1.172391336	0.037337931	0.459930654
109270	'Prr5'	511	498	586	1242	420	618	531.6666667	760	0.722152045	0.260553636	0.878537553
109272	'Mybpc1'	60	40	3	1	21	21	34.33333333	14.33333333	-1.28631041	0.268541237	0.883600995
109275	'Actr5'	184	179	106	102	232	201	156.3333333	178.3333333	0.212848699	0.557332615	0.972790461
109284	'R3hdm4'	1051	1081	1022	979	1225	1181	1051.333333	1128.333333	0.158346524	0.585272979	0.972790461

109294	'Prex2'	161	216	59	11	353	123	145.3333333	162.3333333	0.10318063	0.91308904	0.989773583
109299	'Tmem250-ps'	815	843	585	301	825	979	747.6666667	701.6666667	-0.120926119	0.757443214	0.972790461
109305	'Orai1'	103	139	88	234	187	200	110	207	1.044632813	0.017206793	0.327921282
109314	'Prr9'	0	0	1	1	0	0	0.333333333	0.333333333	0.058500858	0.988561293	0.999493374
109323	'C1qtnf7'	580	604	122	47	963	760	435.3333333	590	0.385144385	0.66800887	0.972790461
109331	'Rnf20'	2528	2446	1696	1855	2201	2008	2223.333333	2021.333333	-0.044676581	0.883865928	0.98358443
109332	'Cdcp1'	148	153	121	66	119	185	140.6666667	123.3333333	-0.20165689	0.598596943	0.972790461
109333	'Pkn2'	2149	2252	3411	2543	2483	2773	2604	2599.666667	0.039701648	0.928363193	0.993096978
109342	'Slc5a10'	24.09	33	8	0	20	10.03	21.69666667	10.01	-1.171092725	0.312507805	0.911537035
109346	'Ankrd39'	133	129	64	111	172	140	108.6666667	141	0.456444083	0.244807598	0.866091613
109349	'Fam163b'	15	11	5	0	7	2	10.33333333	3	-1.816111695	0.147607224	0.754705732
109359	'Abraxas2'	1012	1101	1129	865	1010	1010	1080.666667	961.6666667	-0.113202131	0.727067607	0.972790461
109552	'Sri'	871	912	1249	103	1204	1197	1010.666667	834.6666667	-0.434228881	0.55740552	0.972790461
109575	'Tbx10'	1	0	0	1	0	0	0.333333333	0.333333333	0.058500858	0.988561293	0.999493374
109593	'Lmo3'	12	10	0	1	29	30	7.333333333	20	1.411968346	0.316093113	0.914806078
109594	'Lmo1'	90	110	104	41	32	77	101.3333333	50	-0.990745299	0.04307522	0.484583479
109620	'Dsp'	334	272	347	1782	381	739	317.6666667	967.3333333	1.855746089	0.01224169	0.273764981
109624	'Cald1'	5026	5020	7125	6058	4779	5666	5723.666667	5501	0.019521822	0.96589574	0.999493374
109637	'Upk1a'	9	9	24	1	11	12	14	8	-0.97971733	0.340931395	0.925308103
109648	'Npy'	0	0	2	2	0	0	0.666666667	0.666666667	0.245345372	0.950470028	0.998065566
109652	'Acy1'	220	238	171	51	211	213	209.6666667	158.3333333	-0.45934939	0.349426733	0.92886351
109658	'Txlna'	2434	2433	1864	1876	2716	2883	2243.666667	2491.666667	0.19598187	0.385306955	0.946930692
109660	'Ctrl'	9	13	3	1	15	5	8.333333333	7	-0.266092401	0.815317113	0.972790461
109663	'Hoxc11'	0	0	1	0	0	0	0.333333333	0	-0.903279821	0.824807108	0.972790461
109672	'Cyb5a'	823	936	1101	998	989	1381	953.3333333	1122.666667	0.272544287	0.472556227	0.972790461
109674	'Ampd2'	1000.8	898.52	1467.5	3282.6	1185.8	1209.56	1122.27	1892.65	0.961065007	0.168160727	0.783596014
109676	'Ank2'	348	322	645	16	366	241	438.3333333	207.6666667	-1.26602033	0.14607202	0.753611197
109685	'Hyal3'	12	17	6	20	30	13	11.66666667	21	0.96983352	0.1874307	0.808617437
109689	'Arrb1'	750	643	652	3435	899	1531	681.6666667	1955	1.768254959	0.009896845	0.24563814
109697	'Cpa1'	36	41	6	1	23	3	27.66666667	9	-1.617953283	0.166888684	0.781229856
109700	'Itga1'	124	115	61	228	131	112	100	157	0.875379206	0.140204768	0.74306325
109711	'Actn1'	1612	1512	4261	2011	1623	1877	2461.666667	1837	-0.400831534	0.52231428	0.972790461
109729085	'Htd2'	13.52	0	24.35	12.31	12.88	31.56	12.62333333	18.91666667	0.464081319	0.714576206	0.972790461
109731	'Maob'	71	98	148	99	104	142	105.6666667	115	0.126615443	0.804094817	0.972790461
109754	'Cyb5r3'	2030	2244	3004	1866	1924	2689	2426	2159.666667	-0.146529873	0.715410476	0.972790461
109778	'Blvra'	208	247	150	53	234	336	201.6666667	207.6666667	-0.027861221	0.960983804	0.999493374
109785	'Pgm3'	403.52	370.79	796.19	386.12	333.99	320.79	523.5	346.9666667	-0.544538885	0.332390233	0.921648675
109791	'Clps'	1	1	0	0	0	0	0.666666667	0	-1.703500596	0.673820112	0.972790461
109801	'Glo1'	3875	4123	3461	1049	2006	2272	3819.666667	1775.666667	-1.108466685	5.63E-05	0.008853638
109815	'Selenos'	399	416	945	408	476	959	586.6666667	614.3333333	-0.005851423	0.991957037	0.999493374

109820	'Pgc'	2	4	0	182	5	33	2	73.33333333	5.666602615	8.96E-05	0.012810696
109821	'F11'	0	0	0	0	1	0	0	0.333333333	1.020273531	0.802557913	0.972790461
109828	'C7'	16	25	2	0	4	0	14.33333333	1.333333333	-3.390290599	0.038638647	0.460851913
109857	'Cbr3'	98	124	304	359	163	631	175.3333333	384.3333333	1.106518501	0.128915774	0.72577634
109880	'Braf'	1373	1432	1527	1435	1268	1343	1444	1348.666667	-0.007942385	0.98375031	0.999493374
109889	'Mzf1'	112	104	19	23	120	41	78.33333333	61.33333333	-0.313484948	0.692369403	0.972790461
109900	'Asl'	486	558	1223	532	607	719	755.6666667	619.3333333	-0.307230041	0.578065072	0.972790461
109901	'Cela1'	16	18	15	8	203	113	16.33333333	108	2.604527703	0.004081203	0.147793951
109904	'Mcf2'	22	31	1	2	65	42	18	36.33333333	0.985806679	0.424749846	0.960285372
109905	'Rap1a'	1159	1276	2292	1662	1339	1773	1575.666667	1591.333333	0.053651386	0.917707201	0.990547571
109910	'Zfp91'	3296	3319	2715	2695	2789	3114	3110	2866	-0.034793256	0.908020656	0.988823459
109929	'Zbtb25'	547.5	504	330.09	207	369.03	313.67	460.53	296.5666667	-0.587866267	0.051646691	0.527906934
109934	'Abr'	1847	1921	1321	1230	1611	1328	1696.333333	1389.666667	-0.203076058	0.501740041	0.972790461
109959	'Amy2a5'	1	1	0	0	0	0	0.666666667	0	-1.703500596	0.673820112	0.972790461
109978	'Art4'	16	16	0	4	44	44	10.66666667	30.66666667	1.50975509	0.227131381	0.854001521
109979	'Art3'	6	6	11	6	4	13	7.666666667	7.666666667	-0.016822058	0.985730756	0.999493374
110006	'Gusb'	589.67	676.28	292.04	439.96	1064.8	884.34	519.33	796.35	0.648093278	0.106430769	0.679124905
110012	'Tpgs1'	300	323	646	240	447	458	423	381.6666667	-0.210663034	0.681637233	0.972790461
110033	'Kif22'	579	599	169	259	479	448	449	395.3333333	-0.106843712	0.829540745	0.973678583
110052	'Dek'	2499.7	2727.6	2179.4	1254.3	2678.9	2347.2	2468.9	2093.473333	-0.233081332	0.408463972	0.957157474
110058	'Syt17'	42	24	218	149	13	47	94.66666667	69.66666667	-0.269801384	0.815990852	0.972790461
110074	'Dut'	528	468	557	133	472	555	517.6666667	386.6666667	-0.50513665	0.296655537	0.90029104
110075	'Bmp3'	98	135	41	39	175	107	91.33333333	107	0.234242624	0.699852743	0.972790461
110078	'Pygb'	4078	4111	1861	2973	4609	4316	3350	3966	0.321623076	0.368322434	0.937530612
110082	'Dnah5'	138	142	19	2	98	54	99.66666667	51.33333333	-1.000043674	0.35120785	0.928932061
110083	'Dnah12'	461	463	45	6	202	93	323	100.3333333	-1.716818076	0.10127554	0.671759705
110084	'Dnah1'	232	262	77	27	268	122	190.3333333	139	-0.48091769	0.520762997	0.972790461
110094	'Phka2'	11552	11089	2025.9	1550.3	14383	8957.98	8222.126667	8297.236667	-0.004635856	0.995289143	0.999562152
110095	'Pygl'	1168	1087	2925	468	1102	1426	1726.666667	998.6666667	-0.914653156	0.131850081	0.72897159
110109	'Nop2'	1001	1034	963	611	860	1005	999.3333333	825.3333333	-0.25053185	0.334238814	0.921648675
110115	'Cyp11b1'	1	0	0	6	0	0	0.333333333	2	2.949224807	0.406616877	0.957157474
110119	'Mpi'	324.44	413	173.4	674.36	385.35	420.97	303.6133333	493.56	0.914441638	0.096847899	0.663693139
110135	'Fgb'	0	4	2	2	1	2	2	1.666666667	-0.160977071	0.928411707	0.993096978
110147	'Ehmt2'	3578	3743	2738	2881	3988	4695	3353	3854.666667	0.245109917	0.293255475	0.90029104
110157	'Raf1'	1364	1449.2	1250.3	586.78	1565.6	1287.06	1354.49	1146.466667	-0.261031913	0.448144712	0.970198456
110168	'Gpr18'	1	4	1	0	0	0	2	0	-3.334496305	0.250892177	0.871167957
110172	'Slc35b1'	324	352	513	567	469	775	396.3333333	603.6666667	0.637987069	0.167784385	0.782225778
110173	'Manba'	411	439	268	1154	950	1303	372.6666667	1135.666667	1.723372111	4.63E-06	0.001354787
110175	'Ggct'	110	123	74	191	174	293	102.3333333	219.3333333	1.177209196	0.002441348	0.110775422
110196	'Fdps'	578	730	1050	925	704	930	786	853	0.180001887	0.716544117	0.972790461

110197	'Dgkg'	6	10	6	5	7	3	7.333333333	5	-0.461378991	0.628174643	0.972790461
110198	'Akr7a5'	770	861	572	401	949	1065	734.3333333	805	0.121857626	0.72223604	0.972790461
110208	'Pgd'	2321	2395	3037	2536	2275	4271	2584.333333	3027.333333	0.246946803	0.552609041	0.972790461
110213	'Tmbim6'	3183	3100	2701	4532	4880	5595	2994.666667	5002.333333	0.811701552	0.004892888	0.165356847
110253	'Triobp'	1574.6	1391	3738	805	1220	1352	2234.516667	1125.666667	-1.049149872	0.062853216	0.566066012
110257	'Hba-a2'	357.5	378.6	2287.9	120.84	3091.3	591.36	1007.993333	1267.816667	0.076853089	0.944494958	0.996592403
110265	'Msra'	211	218	206	84	177	158	211.6666667	139.6666667	-0.601536903	0.067948053	0.582429387
110279	'Bcr'	1797	1681	1373	2249	1770	1652	1617	1890.333333	0.369125401	0.384006794	0.945936404
110304	'Glra3'	0	0	4	6	0	0	1.333333333	2	0.847956029	0.803130028	0.972790461
110308	'Krt5'	0	0	1	2	4	4	0.333333333	3.333333333	3.221013991	0.10960074	0.689773582
110310	'Krt7'	628	782	1121	591	768	945	843.6666667	768	-0.14482446	0.734991311	0.972790461
110312	'Pmch'	3.15	1.04	0	0	0	0	1.396666667	0	-2.70051453	0.497202575	0.972790461
110323	'Cox6b1'	858	989	1208	1204	674	953	1018.333333	943.6666667	0.010867919	0.983241182	0.999493374
110326	'Tas1r1'	43	33	17	26	24	17	31	22.33333333	-0.318734883	0.612417185	0.972790461
110332	'Pp2d1'	39.07	52.13	16.06	7	25.06	6.01	35.75333333	12.69	-1.430244623	0.071214874	0.591632421
110350	'Dync2h1'	1579	1690	349	397.96	1454	1283	1205.986667	1044.986667	-0.185227413	0.767550859	0.972790461
110351	'Rap1gap'	739	698	276	980	924	897	571	933.6666667	0.864143413	0.063625186	0.567640559
110355	'Grk2'	948	911	1186	677	1227	1124	1015	1009.333333	-0.019482481	0.955680525	0.999493374
110379	'Sec13'	812	942	835	1177	1027	1212	863	1138.666667	0.493023655	0.178814276	0.798594556
110380	'Shroom2'	1124	1020	584	640	1147	1475	909.3333333	1087.333333	0.282404486	0.411205706	0.95722888
110382	'C8b'	8	6	5	1	11	20	6.333333333	10.66666667	0.650687325	0.54065081	0.972790461
110385	'Pde4c'	6	4	4	5	3	3	4.666666667	3.666666667	-0.213935872	0.845395677	0.975182082
110391	'Qdpr'	1134	1155	1529	455	1132	1677	1272.666667	1088	-0.310344826	0.506356699	0.972790461
110417	'Pigh'	459.98	429.68	202.3	124.7	453.37	511	363.9866667	363.0233333	-0.026690478	0.958484127	0.999493374
110446	'Acat1'	1782	1956	1633	1139	2379	4352	1790.333333	2623.333333	0.501011521	0.2448237	0.866091613
110454	'Ly6a'	4	1	11	38	1	4	5.333333333	14.33333333	1.724331174	0.26049022	0.878537553
110460	'Acat2'	729.82	747.43	521.7	1682.9	494.13	660.69	666.3166667	945.91	0.780533208	0.251371404	0.871230154
110521	'Hivep1'	2401	2150	1923	3991	1437	1938	2158	2455.333333	0.416138441	0.499181348	0.972790461
110524	'Dgkq'	449.97	472.7	274.82	443.15	628.15	376.03	399.1633333	482.4433333	0.381732299	0.33950307	0.924888762
110532	'Adarb1'	729	683	218	146	330	301	543.3333333	259	-1.01642448	0.040001333	0.467715468
110542	'Amhr2'	2526	2929	561	374	5370	4032	2005.333333	3258.666667	0.6573568	0.432885983	0.964494117
110557	'H2-Q6'	72.65	60.1	176.49	55.87	59.35	39.45	103.08	51.55666667	-0.986843975	0.148332526	0.756674202
110558	'H2-Q9'	32.17	54.1	84.86	37.26	21.87	17.47	57.04333333	25.53333333	-1.056837037	0.156943633	0.768337387
110593	'Prdm2'	2666	2442	1465	2484	2252	2259	2191	2331.666667	0.226188532	0.562544199	0.972790461
110595	'Timp4'	40	23	3	5	36	19	22	20	-0.121234211	0.903518287	0.987564084
110596	'Arhgef28'	826	805	492	265	998	902	707.6666667	721.6666667	-0.002629174	0.995386058	0.999562152
110599566	'Eef1akmt4'	147.22	131	182	81	112	171	153.4066667	121.3333333	-0.355985678	0.393606352	0.953178011
110599584	'Eef1akmt4-ece2	7.15	0	0	0	0	0	2.383333333	0	-3.508862209	0.374065944	0.939960576
110599589	'Asdurf'	0.06	13.62	0	12.8	22.12	22.41	4.56	19.11	2.219576568	0.146092518	0.753611197
110606	'Fntb'	469	489	1201	160	314	331	719.6666667	268.3333333	-1.509840824	0.008770155	0.229351006

110611	'Hdlbp'	10831	10217	29477	11044	9136	12817	16841.66667	10999	-0.617970231	0.321153573	0.917442833
110616	'Atxn3'	741.46	746.45	563.8	575.78	625.86	595.93	683.9033333	599.19	-0.09748698	0.761007031	0.972790461
110637	'Grik4'	16	20	31	9	6	10	22.33333333	8.333333333	-1.384985432	0.065881193	0.574593252
110639	'Prps2'	611	667	605	511	671	818	627.6666667	666.6666667	0.11820541	0.664746424	0.972790461
110648	'Lmx1a'	3	0	2	2	3	4	1.666666667	3	0.839546138	0.597025693	0.972790461
110651	'Rps6ka3'	4899	5217	3392	4611	4499	5269	4502.666667	4793	0.192004471	0.553543477	0.972790461
110695	'Aldh7a1'	1272	1358	968	881	1597	2069	1199.333333	1515.666667	0.341660579	0.247086631	0.868801743
110749	'Chaf1b'	319	325	283	302	283	280	309	288.3333333	0.00173599	0.996270945	0.999616639
110750	'Cse1l'	2628	2672	1392	1250	2390	2477	2230.666667	2039	-0.093683098	0.775987335	0.972790461
110751	'Adam33'	119	113	14	42	116	62	82	73.33333333	-0.072956086	0.923841572	0.991932584
110784	'Nr3c2'	159	161	29	92	435	361	116.3333333	296	1.355739388	0.054069528	0.534315058
110789	'Adgrv1'	66	64	102	81	57	45	77.33333333	61	-0.226181671	0.710828201	0.972790461
110794	'Cebpe'	14	20	1	2	18	4	11.66666667	8	-0.504755286	0.684918809	0.972790461
110796	'Tshz1'	1984	1922	1627	1378	2061	1780	1844.333333	1739.666667	-0.033878832	0.895608981	0.985505807
110805	'Foxe1'	3	3	4	0	1	1	3.333333333	0.666666667	-2.37738374	0.174790453	0.793295795
110809	'Srsf1'	5167	5246	2993	2082	5903	3885	4468.666667	3956.666667	-0.159892805	0.682431194	0.972790461
110816	'Pwp2'	587	578	644	326	630	667	603	541	-0.17397441	0.590785587	0.972790461
110821	'Pcca'	366	429	114	234	503	661	303	466	0.656186252	0.215478051	0.843391934
110826	'Etfb'	922	1113	765	375	1062	1113	933.3333333	850	-0.163341278	0.669036768	0.972790461
110829	'Lims1'	2813	3076	3465	2185	2969	3409	3118	2854.333333	-0.112733115	0.722307481	0.972790461
110834	'Chrna3'	3.3	1.08	1	0	3.59	1.18	1.793333333	1.59	-0.3730735	0.847533968	0.975439569
110835	'Chrna5'	31.7	32.92	6	7	27.41	46.82	23.54	27.07666667	0.201928579	0.810109231	0.972790461
110842	'Etfa'	2179.6	2332	1963	2107	2058	2807	2158.206667	2324	0.17585336	0.571511308	0.972790461
110854	'Ptpa'	2241	2449	1452	829	2664	2716	2047.333333	2069.666667	-0.010513237	0.979990564	0.999493374
110855	'Pde6c'	0	0	0	0	0	1	0	0.333333333	1.020273531	0.802557913	0.972790461
110862	'Kcnq3'	40	30	118	0	19	10	62.66666667	9.666666667	-2.919996108	0.008572688	0.22511595
110876	'Scn2a'	51	41	14.98	6	36	25	35.66	22.33333333	-0.668343254	0.372405078	0.938816563
110877	'Slc18a1'	71	74	20	20	80	46	55	48.66666667	-0.148394606	0.821905064	0.972790461
110880	'Scn4a'	7	9	0	7	3	4	5.333333333	4.666666667	0.077409971	0.956027381	0.999493374
110886	'Gabra5'	48	45	8	0	13	6	33.66666667	6.333333333	-2.436400455	0.030727055	0.425931054
110891	'Slc8a2'	131	118	34	83	218	173	94.33333333	158	0.792032531	0.154682324	0.766453805
110893	'Slc8a3'	29	34	7	6	23	13	23.33333333	14	-0.693040979	0.388302402	0.950023663
110895	'Slc9a4'	9	8	0	5	8	20	5.666666667	11	1.006270813	0.411045745	0.95722888
110902	'Chrna2'	1	0	0	2	1	2	0.333333333	1.666666667	2.329090277	0.33135092	0.921648675
110911	'Cds2'	1791	1664	955	2160	2245	2682	1470	2362.333333	0.786514952	0.02162566	0.364161986
110920	'Hspa13'	1232	1235	1096	2330	1660	2459	1187.666667	2149.666667	0.96327039	0.015562943	0.309500711
110935	'Atp6v1b1'	5	6	2	0	4	1	4.333333333	1.666666667	-1.399640219	0.368250669	0.937490857
110948	'Hlcs'	471	490	120	243	459	548	360.3333333	416.6666667	0.270889802	0.615508207	0.972790461
110954	'Rpl10'	15743	15867	36273	18171	12230	16433	22627.68667	15611.33333	-0.485711105	0.415880798	0.95722888
110956	'D17H6S56E-5'	495	484	164	244	568	372	381	394.6666667	0.116804501	0.811248516	0.972790461

110957	'D1Pas1'	1120	1076	153	6	508	529	783	347.6666667	-1.236232315	0.271222885	0.884312405
110958	'M1ap'	1197	960	811	2	222	168	989.3333333	130.6666667	-3.05359753	0.002699442	0.116112669
110959	'Nudt19'	242	294	443	62	421	500	326.3333333	327.6666667	-0.149950857	0.826263237	0.972844676
110960	'Tars'	1305	1269	2788	1333	1500	2020	1787.333333	1617.666667	-0.167830306	0.748997794	0.972790461
110962	'Mbd6'	1370	1378	1749	639	1553	997	1499	1063	-0.519106511	0.20051374	0.827996074
111173	'Erc1'	1674	1621	1224	1245	1700	1521	1506.333333	1488.67	0.051180472	0.848612216	0.975686757
111175	'Pecr'	226	252	315	80	185	243	264.3333333	169.3333333	-0.706148978	0.112120715	0.69684179
111241	'Hmga1b'	206.02	168.94	837.44	2521.4	524.04	748.12	404.1333333	1264.513333	1.821157837	0.058186741	0.551665011
112403	'Dxo'	567	499	430	305	451	628	498.6666667	461.3333333	-0.09630682	0.741558468	0.972790461
112405	'Egln1'	3047	2334	17271	754	1850	2956	7550.666667	1853.333333	-2.233985642	0.01022094	0.250736132
112406	'Egln2'	1691	1779	996	755	1462	2040	1488.666667	1419	-0.062322079	0.862854925	0.977220339
112407	'Egln3'	674	518	11335	1055	312	1015	4175.666667	794	-2.432754761	0.036311775	0.456985794
112415	'Zfp607b'	336	332	206	57	194	213	291.3333333	154.6666667	-0.944879254	0.038830036	0.461389364
112418	'1700102P08Rik'	21	20	12	9	11	9	17.66666667	9.666666667	-0.783701143	0.238656194	0.864053647
112419	'Ifit1bl2'	2	1	2.98	5	11	17	1.993333333	11	2.67972878	0.015586892	0.309653066
112422	'Zfp979'	237.26	234.82	694.29	23.83	178.67	123.2	388.79	108.5666667	-2.032568527	0.009008998	0.231771405
112694759	'Gm20517'	4.06	8.05	8.91	7.39	4.18	4.65	7.006666667	5.406666667	-0.297306625	0.76881765	0.972790461
11287	'Pzp'	2	3	4	2	1	1	3	1.333333333	-1.074420459	0.470480102	0.972790461
11298	'Aanat'	1	0	2	1	2	0	1	1	8.55E-04	0.999723172	0.999900097
113002583	'Shld3'	48.15	45.17	56.77	43.05	48.08	52.8	50.03	47.97666667	-0.016094583	0.971584615	0.999493374
11302	'Aatk'	18	11	6	2	15	24	11.66666667	13.66666667	0.167118338	0.858857093	0.976765631
11303	'Abca1'	3403	3401	1097	8054	5133	4344	2633.666667	5843.666667	1.38750345	0.020555563	0.356011446
11304	'Abca4'	323	282	39	64	254	122	214.6666667	146.6666667	-0.492944621	0.517730553	0.972790461
11305	'Abca2'	1213	1193	261	669	1444	1879	889	1330.666667	0.626926219	0.283157642	0.891393415
11306	'Abcb7'	774	780	337	303	602	590	630.3333333	498.3333333	-0.292903542	0.45263502	0.970649024
11307	'Abcg1'	164	185	76	1079	401	475	141.6666667	651.6666667	2.479764746	2.00E-04	0.02246214
11308	'Abi1'	1592	1647	3651	2986	1775	2170	2296.666667	2310.333333	0.089199836	0.886140479	0.984348372
11350	'Abl1'	3798	3746	2467.2	2161	4088	3494	3337.07	3247.666667	-0.001454007	0.995802165	0.999562152
11352	'Abl2'	2848	2802	1199	2043	1903	2009	2283	1985	-0.056763461	0.895725875	0.985505807
113523645	'Lrrc70'	16	23	9	4	20	9	16	11	-0.532358309	0.509745347	0.972790461
11363	'Acadl'	968	1058	2065	2972	2001	3369	1363.666667	2780.666667	1.06399725	0.058207721	0.551665011
11364	'Acadm'	924	1060	1051	1368	1256	1499	1011.666667	1374.333333	0.518188818	0.155072825	0.766453805
11370	'Acadvl'	1273	1406	812	2154	1648	2052	1163.666667	1951.333333	0.885769459	0.02888294	0.414174039
113845	'Vmn1r48'	0	0	2	0	1.34	1.34	0.666666667	0.893333333	-0.259807793	0.938193093	0.994698142
113846	'Vmn1r47'	1	2.99	0	0	2.66	2.66	1.33	1.773333333	0.390967697	0.86840498	0.979229375
113849	'Vmn1r52'	1	0	0	0	0	0	0.333333333	0	-0.903279821	0.824807108	0.972790461
113850	'V1ra8'	0	1	0	0	0	0	0.333333333	0	-0.903279821	0.824807108	0.972790461
113853	'Vmn1r53'	3	6	1	0	0	0	3.333333333	0	-4.050681912	0.103223715	0.673650969
113855	'Vmn1r40'	0	0	1	0	0	0	0.333333333	0	-0.903279821	0.824807108	0.972790461
113868	'Acaa1a'	758.81	901.91	948.57	1387.9	1281.9	1444.13	869.7633333	1371.3	0.732255468	0.05699287	0.547413448

11409	'Acads'	648	687	516	535	778	1034	617	782.3333333	0.367688659	0.168554003	0.784384366
114128	'Laptm4b'	3742	3904	2015	4655	5215	4097	3220.333333	4655.666667	0.661681272	0.091206537	0.649965349
114141	'Cldn16'	4	0	0	0	3	4	1.333333333	2.333333333	0.7548377	0.775139419	0.972790461
114142	'Foxp2'	45	42	39	138	38	20	42	65.33333333	0.959032277	0.282904757	0.890936762
114143	'Atp6v0b'	1140	1196	1499	2967	1232	2191	1278.333333	2130	0.882876803	0.123087875	0.714726459
11416	'Slc33a1'	472.52	430.6	506.03	592.46	512.08	727.52	469.7166667	610.6866667	0.442181425	0.250376163	0.871106156
11418	'Asic2'	188	193	164	81	116	87	181.6666667	94.66666667	-0.879535266	0.011475612	0.265418906
11419	'Asic1'	114	98	35	82	92	68	82.33333333	80.66666667	0.120689393	0.825786397	0.972817749
11421	'Ace'	232	208	530	653	129	636	323.3333333	472.6666667	0.632845254	0.42919588	0.962386546
114229	'Kiss1r'	66	58	16	84	50	54	46.66666667	62.66666667	0.650057922	0.342029621	0.925308103
11423	'Ache'	42	27	24	121	12	15	31	49.33333333	1.069124399	0.312721146	0.911912367
114230	'Aipl1'	3	5	6	4	7	2	4.666666667	4.333333333	-0.063272212	0.955020254	0.999493374
114249	'Npnt'	531	408	93	61	796	503	344	453.3333333	0.363109945	0.663999377	0.972790461
11425	'Apoc4'	0	1	0	0	4	2	0.333333333	2	2.360230172	0.41369376	0.95722888
114255	'Dok4'	247.65	258.24	238.46	346.7	369.75	303.52	248.1166667	339.99	0.547349023	0.147425415	0.754468905
11426	'Macf1'	9052	8825	5325	7137	8822	8298	7734	8085.666667	0.157557821	0.611610113	0.972790461
11428	'Aco1'	1325	1281	1042	1170	1579	1663	1216	1470.666667	0.325938555	0.174371517	0.793128181
11429	'Aco2'	3678	3653	2998	2494	3777	3852	3443	3374.333333	0.008295596	0.970346453	0.999493374
11430	'Acox1'	1747	1649	3439	1675	1724	2103	2278.333333	1834	-0.30935936	0.544336675	0.972790461
114301	'Palmd'	144	173	126	22	199	169	147.6666667	130	-0.278914335	0.675431816	0.972790461
114304	'Slc28a3'	0	0	5	0	1	3	1.666666667	1.333333333	-0.664014091	0.81437248	0.972790461
11431	'Acp1'	1273	1321	1645	443	1168	1412.99	1413	1007.996667	-0.559065836	0.194805848	0.819935527
11432	'Acp2'	1018.9	1031.1	377.66	179.08	1412.6	1235.68	809.2266667	942.45	0.171288881	0.798892564	0.972790461
11433	'Acp5'	162	158	136	16	94	104	152	71.33333333	-1.177220817	0.046289541	0.501171272
114332	'Lyve1'	6	3	0	1	2	6	3	3	0.02697894	0.986782482	0.999493374
11434	'Acr'	7	8	2	1	7	1	5.666666667	3	-0.879999175	0.505479175	0.972790461
11435	'Chrna1'	3.82	3.1	5	5	1	1	3.973333333	2.333333333	-0.437512828	0.758085717	0.972790461
11438	'Chrna4'	87	104	27	5	164	92	72.66666667	87	0.198100862	0.836284413	0.974723675
11441	'Chrna7'	50	39	2	12	59	25	30.33333333	32	0.133180431	0.895681893	0.985505807
11443	'Chrnb1'	103	85	205	232	141	144	131	172.3333333	0.491174483	0.452039985	0.970649024
11444	'Chrnb2'	31	33	17	33	35	14	27	27.33333333	0.182552392	0.787654283	0.972790461
114479	'Slc5a5'	15	23	5	21	9	6	14.33333333	12	0.0359963	0.970273065	0.999493374
11448	'Chrne'	0	1	0	0	2	3	0.333333333	1.666666667	2.081165771	0.487041593	0.972790461
11449	'Chrng'	0	1	0	0	0	0	0.333333333	0	-0.903279821	0.824807108	0.972790461
11450	'Adipoq'	2	3	0	0	1	2	1.666666667	1	-0.75018494	0.743459437	0.972790461
114564	'Csprs'	0	0	0	0	0	1	0	0.333333333	1.020273531	0.802557913	0.972790461
114565	'Zbtb21'	1108	1091	1507	605	843	866	1235.333333	771.3333333	-0.671460157	0.068895999	0.585534444
114570	'Crip3'	308.35	323.24	42.42	50.43	274.6	106.96	224.67	143.9966667	-0.607832206	0.447694644	0.970198456
114584	'Clic1'	2009	2010	4745	1935	2681	3137	2921.333333	2584.333333	-0.224052971	0.678997859	0.972790461
114585	'D17H6S53E'	126	143	138	45	164	168	135.6666667	125.6666667	-0.177190979	0.713530143	0.972790461

11459	'Acta1'	2	2	2	2	0	1	2	1	-0.806326203	0.662867234	0.972790461
114601	'Ehbp1l1'	322.3	375	260.05	565	486	484	319.1166667	511.6666667	0.809039108	0.04100014	0.475011978
114602	'Zmynd10'	3	9	4	19	12	2	5.333333333	11	1.302069445	0.263058901	0.88013169
114604	'Prdm15'	517	500	276	930	544	393	431	622.3333333	0.765425937	0.193321685	0.816702866
114606	'Tle6'	731	784	469	1115	1236	2035	661.3333333	1462	1.200381509	3.41E-04	0.033218136
11461	'Actb'	13856	14907	21571	7156	15174	15923	16777.95	12751	-0.449369907	0.278833166	0.889355605
114615	'Elac1'	791	839	357	238	645	486	662.3333333	456.3333333	-0.508681057	0.239659985	0.86449675
11464	'Actc1'	25	18	29	0	24	6	24	10	-1.405200602	0.227335456	0.854001521
114640	'Pth2'	35	30	1	0	15	1	22	5.333333333	-2.043926208	0.201196968	0.828853034
114641	'Rpl31'	9204.8	9836	17465	21483	7047.8	10022.86	12168.78	12851.18667	0.25664389	0.714921331	0.972790461
114642	'Brdt'	638	635	158	78	331	312	477	240.3333333	-0.98708969	0.117406275	0.705476642
114643	'Oas1c'	26	27	13	9	38	104	22	50.33333333	1.113794311	0.168844057	0.784384366
114644	'Slc13a3'	112	112	3	10	44	9	75.66666667	21	-1.75450428	0.1162435	0.703687844
11465	'Actg1'	27353	27471	53378	11745	28747	34725	36067.33667	25072.33333	-0.625125184	0.230177349	0.856721174
114652	'Ly6g5c'	0	0	0	0	1	0	0	0.333333333	1.020273531	0.802557913	0.972790461
114654	'Ly6g6d'	2	6	0	1	4	1	2.666666667	2	-0.318469838	0.858339043	0.976475731
114661	'Prss28'	0	0	0	1	0	0	0	0.333333333	1.020273531	0.802557913	0.972790461
114663	'Impa2'	217	206	162	87	176	269	195	177.3333333	-0.157733988	0.687130222	0.972790461
114664	'Hsd17b11'	872	890	535	899	1436	1331	765.6666667	1222	0.73200612	0.009188783	0.234872395
114671	'4930444G20Rik'	20.76	23.61	6.19	1	12.63	2.1	16.85333333	5.243333333	-1.694698017	0.124402502	0.717146542
114674	'Gtf2ird2'	321	358	187	119	374	283	288.6666667	258.6666667	-0.156433928	0.714606132	0.972790461
114679	'Selenom'	214	191	262	256	207	238	222.3333333	233.6666667	0.153315928	0.735402785	0.972790461
11468	'Actg2'	11	5	14	0	23	5	10	9.333333333	-0.263605743	0.845344574	0.975182082
11470	'Actl7a'	0	0	1	2	0	0	0.333333333	0.666666667	1.337350669	0.73871782	0.972790461
11471	'Actl7b'	0	1	0	1	1	0	0.333333333	0.666666667	1.081692101	0.760081965	0.972790461
114713	'Rasa2'	802	887	601	298	890	787	763.3333333	658.3333333	-0.232784303	0.539676592	0.972790461
114714	'Rad51c'	311	290	111	82	205	210	237.3333333	165.6666667	-0.491983057	0.304074113	0.906771226
114715	'Spred1'	3344	3210	5825	6683	2800	4004	4126.333333	4495.666667	0.264797826	0.677625899	0.972790461
114716	'Spred2'	635.93	576	1792	1953	537	1111.01	1001.31	1200.336667	0.388807514	0.626271335	0.972790461
11472	'Actn2'	21	10	5	20	9	8	12	12.33333333	0.292537534	0.754219309	0.972790461
11474	'Actn3'	42	61	34	13	76	129	45.66666667	72.66666667	0.581168413	0.412402541	0.95722888
114741	'Supt16'	7191.8	6825.2	2443.8	1592.5	4357.9	5402.33	5486.936667	3784.226667	-0.529313893	0.294466457	0.90029104
11475	'Acta2'	923	931	856	274	1318	717	903.3333333	769.6666667	-0.28644709	0.571498653	0.972790461
11477	'Acvr1'	335	354	328	672	493	517	339	560.6666667	0.856377444	0.051859142	0.529072762
114774	'Pawr'	587	621	1351	964	427	862	853	751	-0.108749087	0.867668759	0.979056232
11479	'Acvr1b'	752	808	703	782	863	946	754.3333333	863.6666667	0.263410291	0.376906905	0.941504733
11480	'Acvr2a'	894.56	862.17	496.47	692.68	1227	1145.19	751.0666667	1021.636667	0.492284765	0.105930707	0.679124905
11481	'Acvr2b'	1375	1383	499	542	1174	889	1085.666667	868.3333333	-0.259860821	0.561496477	0.972790461
11482	'Acvrl1'	372	350	294	104	512	466	338.6666667	360.6666667	0.020513624	0.96837508	0.999493374
11484	'Aspa'	206	191	15	5	104	59	137.3333333	56	-1.315123757	0.205926509	0.833294914

114841036	'LOC114841036'	63	55.36	159.85	37.95	60.38	67.35	92.73666667	55.22666667	-0.826632328	0.195877549	0.82153628
11486	'Ada'	81	83	27	93	141	133	63.66666667	122.3333333	1.032987344	0.0331358	0.438703227
114863	'Plpbp'	477	475	1068	540	590	630	673.3333333	586.6666667	-0.200220982	0.712837559	0.972790461
11487	'Adam10'	4321	4577	1543	11514	6321	8432	3480.333333	8755.666667	1.548159018	0.006492431	0.190779128
114871	'Psg28'	0	3.15	0	0	0	0	1.05	0	-2.275512037	0.569613276	0.972790461
114872	'Psg29'	0	0	1	0	0	0	0.333333333	0	-0.903279821	0.824807108	0.972790461
114873	'Dscaml1'	24	21	16	1	34	14	20.33333333	16.33333333	-0.406826968	0.67912482	0.972790461
114874	'Ddhd1'	1104	1180	579	552	931	1044	954.3333333	842.3333333	-0.132865992	0.691063809	0.972790461
11488	'Adam11'	99	110	49	42	110	49	86	67	-0.303904343	0.565904087	0.972790461
114886	'Cygb'	768	798	470	179	746	828	678.6666667	584.3333333	-0.264853914	0.598943055	0.972790461
114889	'Vsx1'	5	9	3	0	1	2	5.666666667	1	-2.517837456	0.095808121	0.662411648
11489	'Adam12'	703	662	444	37	910	1040	603	662.3333333	0.013857994	0.987189373	0.999493374
114893	'Dcun1d1'	1506	1539.3	1385.1	836.27	1628.7	1914.89	1476.836667	1459.963333	-0.028975516	0.921554966	0.991066749
114896	'Afg3l1'	1245	1193	900	538	1238	1321	1112.666667	1032.333333	-0.116476893	0.715223043	0.972790461
11490	'Adam15'	538	450	346	1079	736	943	444.6666667	919.3333333	1.192462289	0.006062311	0.18382242
11491	'Adam17'	1310	1214	1058	2623	1469	1769	1194	1953.666667	0.881224243	0.074580404	0.603900623
11492	'Adam19'	568	597	268	605	926	596	477.6666667	709	0.675209119	0.104284056	0.676639601
11495	'Adam2'	0	0	0	2	0	1	0	1	2.714766126	0.495614689	0.972790461
11496	'Adam22'	311	361	71	9	380	391	247.6666667	260	-1.49E-04	0.999882938	0.999935533
11497	'Adam3'	2	2	0	0	4	5	1.333333333	3	1.124025962	0.575766789	0.972790461
11498	'Adam4'	7	8	4	9	12	5	6.333333333	8.666666667	0.571820678	0.524833652	0.972790461
11499	'Adam5'	15	17	0	1	16	5	10.66666667	7.333333333	-0.521856225	0.724986549	0.972790461
11500	'Adam7'	1	1	1	0	0	0	1	0	-2.390783043	0.494152846	0.972790461
11501	'Adam8'	195	156	73	220	246	183	141.3333333	216.3333333	0.756741386	0.114196417	0.699220241
11502	'Adam9'	802	755	898	2727	972	1712	818.3333333	1803.666667	1.320581223	0.0290297	0.41528417
11504	'Adamts1'	848	740	1182	2380	997	1187	923.3333333	1521.333333	0.893805044	0.158201028	0.768890221
11512	'Adcy6'	1845	1853	699	3118	2755	2228	1465.666667	2700.333333	1.063418879	0.034843871	0.447758353
11513	'Adcy7'	83	67	10	215	85	81	53.33333333	127	1.575621199	0.067090973	0.578735744
11514	'Adcy8'	12	8	24	1	3	2	14.66666667	2	-2.940611783	0.007011524	0.197779062
11515	'Adcy9'	490	528	159	523	612	579	392.3333333	571.3333333	0.676139099	0.164561994	0.777691433
11516	'Adcyap1'	11	16	10	51	5	2	12.33333333	19.33333333	1.074524885	0.398029127	0.954563638
11517	'Adcyap1r1'	275	279	112	889	284	155	222	442.6666667	1.352607568	0.100817098	0.671759705
11518	'Add1'	4914	5180	4545	2864	5386	5547	4879.666667	4599	-0.08296926	0.750333954	0.972790461
11519	'Add2'	97	103	37	13	192	378	79	194.3333333	1.203155488	0.181841943	0.802193703
11520	'Plin2'	1769	2102	3523	9313	4118	5424	2464.666667	6285	1.48978428	0.019539424	0.348059072
11522	'Adh1'	60	60	350	773	73	481	156.6666667	442.3333333	1.5982789	0.13158345	0.72897159
11529	'Adh7'	0	1	41	66	0	4	14	23.33333333	0.979225074	0.650967021	0.972790461
11532	'Adh5'	1891	1948	2919	1543	2013	2637	2252.666667	2064.333333	-0.138220102	0.733394854	0.972790461
11534	'Adk'	1297	1359	900	850	1447	1723	1185.333333	1340	0.200429561	0.4594035	0.972790461
11535	'Adm'	377	298	1689	155	198	469	788	274	-1.68059415	0.038934359	0.461483815

11536	'Gpr182'	35	39	23	348	48	69	32.33333333	155	2.633469228	0.004348287	0.153661044
11538	'Adnp'	6157	6354	3807	3056	5988	5298	5439.333333	4780.666667	-0.150745419	0.606316569	0.972790461
11539	'Adora1'	953.82	1104	1032.3	2491	935	1628.44	1030.046667	1684.813333	0.894998515	0.124288263	0.716961086
11540	'Adora2a'	179	151	72	37	154	71	134	87.33333333	-0.604417116	0.292013694	0.89958198
11541	'Adora2b'	19	19	224	19	4	43	87.33333333	22	-2.126047963	0.075616061	0.605310548
11542	'Adora3'	0	0	0	0	5	0	0	1.666666667	3.073512668	0.438501572	0.966976432
11544	'Adprh'	811	796	774	1409	1033	1341	793.6666667	1261	0.777837384	0.056195398	0.545652152
11545	'Parp1'	6048	5733	2984	5175	5494	7096	4921.666667	5921.666667	0.364153016	0.303874995	0.906771226
11546	'Parp2'	689.95	698	612	214	779	754	666.65	582.3333333	-0.251710922	0.567608285	0.972790461
11548	'Adra1b'	5	9	68	4	4	3	27.33333333	3.666666667	-2.968289836	0.015065974	0.306019557
115486031	'Gm51425'	185.63	173.77	116.41	52.7	151.8	101.34	158.6033333	101.9466667	-0.632679539	0.129111878	0.726020415
115486427	'LOC115486427'	0	0	1	0	0	0	0.333333333	0	-0.903279821	0.824807108	0.972790461
115486435	'LOC115486435'	1	0	0	0	0	0	0.333333333	0	-0.903279821	0.824807108	0.972790461
115486479	'Gm51460'	4.31	3.89	1	2	0	2.11	3.066666667	1.37	-0.815083424	0.637596303	0.972790461
115486481	'Gm45337'	1	0	0	1	0	0	0.333333333	0.333333333	0.058500858	0.988561293	0.999493374
115486489	'Gm51464'	0	1	1	2	0	6	0.666666667	2.666666667	1.973741232	0.38479424	0.94674509
115486510	'Gm45095'	107.07	108.16	76.03	43	73	53	97.08666667	56.33333333	-0.729559932	0.054305473	0.534951117
115486519	'LOC115486519'	14	7	6	0	19.12	7	9	8.706666667	-0.144092991	0.909885098	0.989364653
115486880	'LOC115486880'	2.7	9.59	0	0	2.4	3.64	4.096666667	2.013333333	-1.148276599	0.570966084	0.972790461
115486883	'Gm8940'	0	1	0	4	0	0	0.333333333	1.333333333	2.358713038	0.538795805	0.972790461
115486932	'Gm51573'	0	0	9.06	2	0.62	4.01	3.02	2.21	-0.729080715	0.778783024	0.972790461
115486940	'Gm51579'	72.16	52.06	5	49	44	54.06	43.07333333	49.02	0.366560123	0.662105208	0.972790461
115486964	'Gm51590'	0	0	6.02	0	0	0	2.006666667	0	-3.597486081	0.361825946	0.93373993
115486965	'Gm51591'	1	0	5.27	0	0	1	2.09	0.333333333	-2.58051353	0.377056213	0.941504733
115486966	'Gm51592'	0	0	11.15	0	0	0	3.716666667	0	-4.476211903	0.254425762	0.873895634
115486967	'Gm51593'	0	1	5.17	2	0	0	2.056666667	0.666666667	-1.340221338	0.638187283	0.972790461
115486969	'Gm51595'	0	0	1.75	0	0	0	0.583333333	0	-0.903279821	0.824807108	0.972790461
115486972	'Gm51598'	0	2	1.16	0	0	0	1.053333333	0	-2.386119369	0.550310572	0.972790461
115487111	'Gm29667'	31.78	22.1	209.21	28.89	26	21.08	87.69666667	25.32333333	-1.847536125	0.060599022	0.559004658
115487184	'Tma7-ps'	245.5	185.87	219.64	5.48	11.95	25.61	217.0033333	14.34666667	-4.03359071	2.35E-13	4.07E-10
115487193	'Gm51691'	40.03	34.62	16.01	21	26	12	30.22	19.66666667	-0.467390594	0.480139205	0.972790461
115487351	'Gm39743'	9.17	11.84	5.26	0	1.33	0	8.756666667	0.443333333	-4.446234009	0.007855658	0.214208031
115487375	'Gm48826'	2	1	1	2	2	8	1.333333333	4	1.569916342	0.286449598	0.894199485
115487414	'Gm51777'	1.81	4.88	0	0	0	0	2.23	0	-3.011457993	0.447447746	0.970198456
115487436	'Gm51792'	21.3	23.05	8.41	13	37.38	28	17.58666667	26.12666667	0.616143162	0.339995464	0.925145808
115487437	'Gm51793'	6.1	0	9.59	0	0	0	5.23	0	-4.80321624	0.090839176	0.649965349
115487466	'Gm48175'	3	2	0	0	0	0	1.666666667	0	-3.018420173	0.443727506	0.969836424
115487746	'Gm51877'	27.3	25.44	10.76	13.61	12.76	7.81	21.16666667	11.39333333	-0.789255244	0.294366661	0.90029104
115487818	'Gm10840'	2	1.04	1	2	2.18	1	1.346666667	1.726666667	0.44198908	0.790522696	0.972790461
115487959	'Gm51965'	7.05	19	5.05	5	15.06	1	10.36666667	7.02	-0.4658564	0.687288736	0.972790461

115488002	'LOC115488002'	206.95	189.76	365.38	50.93	168.15	109.93	254.03	109.67	-1.301978331	0.01984419	0.349283865
115488008	'Gm51999'	4.18	5.17	7.09	3.07	1.04	1.01	5.48	1.706666667	-1.544859871	0.239966273	0.86449675
115488020	'Gm52009'	15.74	24.52	11.1	10.45	10.22	13	17.12	11.22333333	-0.515661802	0.447310073	0.970198456
115488029	'LOC115488029'	375.69	325.7	94.84	61.28	139.94	64.33	265.41	88.51666667	-1.492371815	0.012313767	0.274438769
115488119	'Gm47655'	3.01	0	2	0	1	0	1.67	0.333333333	-2.19112013	0.458761794	0.972790461
115488129	'LOC115488129'	1	0.74	0	0	1	4	0.58	1.666666667	2.065879757	0.50146354	0.972790461
115488130	'LOC115488130'	555.72	577.52	225.28	195.05	659.4	310.72	452.84	388.39	-0.186112015	0.723811825	0.972790461
115488140	'Gm53057'	7.14	5.33	0	0	3.16	26.72	4.156666667	9.96	1.196655873	0.519446126	0.972790461
115488157	'Gm52042'	1024.6	1247.7	406.64	15.98	166.39	50.37	892.98	77.58	-3.559569404	2.84E-06	9.17E-04
115488169	'Gm52051'	8	6	3	2	1	6	5.666666667	3	-0.875199095	0.466130585	0.972790461
115488170	'Rpl27a-ps4'	11.14	12.23	7.89	24.18	12.01	19.17	10.42	18.45333333	1.033979631	0.167543697	0.781678718
115488190	'Gm48350'	14	19	3	2	24	8	12	11.33333333	-0.083668131	0.937868135	0.994698142
115488195	'Gm20379'	24	31.89	22.63	5	8	1	26.17333333	4.666666667	-2.360068691	0.006369852	0.189224425
115488203	'LOC115488203'	2	2	0	0	8	4.95	1.333333333	4.316666667	1.54742227	0.429615994	0.962386546
115488204	'Gm45623'	16	17	6	5	10	2	13	5.666666667	-1.082186048	0.257704264	0.877405867
115488282	'Gm3512'	73.66	81.63	28.79	3	21	11.99	61.36	11.99666667	-2.375636309	0.001087511	0.069381767
115488283	'LOC115488283'	3.93	6.1	3.2	0	2	0	4.41	0.666666667	-2.586963161	0.175130821	0.793892984
115488284	'Gm3752'	36.84	16.47	5.11	2.89	29.41	14.89	19.47333333	15.73	-0.362906037	0.726531827	0.972790461
115488350	'LOC115488350'	1	1	1	0	0	0	1	0	-2.390783043	0.494152846	0.972790461
115488359	'Gm52140'	4.01	1	4.02	1	2.01	2	3.01	1.67	-0.873258724	0.550647779	0.972790461
115488379	'Gm6526'	1	3	1	0	4	2	1.666666667	2	0.201134424	0.91196734	0.989773583
115488470	'LOC115488470'	109.63	107.02	24	76	54	33	80.21666667	54.33333333	-0.309202117	0.673639647	0.972790461
115488490	'LOC115488490'	23.04	15	29.88	28	6	7	22.64	13.66666667	-0.463099919	0.632469946	0.972790461
115488529	'LOC115488529'	11.29	14.3	2.08	4.25	10.09	4.04	9.223333333	6.126666667	-0.486211068	0.639088655	0.972790461
115488536	'Gm52217'	100.16	75.1	17.03	0	41	8.32	64.09666667	16.44	-2.012062708	0.102409785	0.671776916
115488611	'Gm52229'	162.81	168.75	48.05	66.28	113.17	68.5	126.5366667	82.65	-0.500612917	0.367616796	0.937190492
115488633	'Gm52241'	5	2	0	2	4	1	2.333333333	2.333333333	0.148191256	0.930919035	0.99357956
115488637	'LOC115488637'	0	2	0	0	0	1	0.666666667	0.333333333	-0.733814424	0.856146772	0.975734242
115488671	'LOC115488671'	22	25	4	37	8	15	17	20	0.565370374	0.572004344	0.972790461
115488765	'LOC115488765'	2.04	2.15	0	0	3.08	4.07	1.396666667	2.383333333	0.766924439	0.712109315	0.972790461
115488789	'LOC115488789'	56	67	17	28	47	17	46.66666667	30.66666667	-0.464879119	0.507107036	0.972790461
115488807	'Gm52310'	4.2	4.06	0	5.15	1.04	2.04	2.753333333	2.743333333	0.320380194	0.847880947	0.975464187
115488928	'Gm52351'	4.5	8.02	0	0	0	1	4.173333333	0.333333333	-3.316298754	0.190303146	0.813136091
115489041	'9430078G10Rik'	101.27	81.02	22.64	27.48	47.94	35.81	68.31	37.07666667	-0.80220947	0.196232674	0.822481393
115489044	'LOC115489044'	9.62	6.09	9.74	115.75	9.91	3.85	8.483333333	43.17	2.817032414	0.032213503	0.43404899
115489115	'LOC115489115'	1	1	0	1	1	1	0.666666667	1	0.724224998	0.748891473	0.972790461
115489118	'Gm52429'	586	595	286	5	191	168	489	121.3333333	-2.110592469	0.020311251	0.353948215
115489150	'Gm14681'	19.59	20.24	9.46	1.63	16.37	1.01	16.43	6.336666667	-1.432057801	0.2265768	0.854001521
115489304	'Gm52481'	14	11	53	2	2	4	26	2.666666667	-3.37671989	0.001738547	0.08981863
115489397	'LOC115489397'	0	0	0.77	0	1.19	3	0.256666667	1.396666667	2.701386663	0.497818139	0.972790461

115489417	'LOC115489417'	31972	33048	7506.8	3377.4	55226	67091.38	24175.50667	41898.28333	0.728153973	0.40441467	0.956545617
115489434	'Gm13194'	1	1	0	0	0	0	0.666666667	0	-1.703500596	0.673820112	0.972790461
115489454	'Gm52512'	228.55	236.25	90.01	72.05	240.43	80.5	184.9366667	130.9933333	-0.447043627	0.453877418	0.970649024
115489469	'Gm52523'	6	6	2	4	6	7	4.666666667	5.666666667	0.345933355	0.729642505	0.972790461
115489487	'Gm14200'	5.01	4	4	4	8	2	4.336666667	4.666666667	0.171018527	0.877076516	0.981341203
115489545	'LOC115489545'	11.31	25.86	8.56	14.58	21.15	8.77	15.24333333	14.83333333	0.093923366	0.907003267	0.988823459
115489748	'LOC115489748'	10.95	10.44	1.78	0	9.42	1.56	7.723333333	3.66	-1.077556172	0.505208208	0.972790461
115489888	'Gm52666'	145.32	145.54	40.61	67.87	106.86	49.22	110.49	74.65	-0.431284468	0.481867223	0.972790461
115489931	'LOC115489931'	0	2	0	0	0	0	0.666666667	0	-1.695595436	0.675304306	0.972790461
115489946	'Gm30191'	0	0	1	0	0	0	0.333333333	0	-0.903279821	0.824807108	0.972790461
115489950	'Zfp988'	46.21	48.61	33.71	2.33	16.13	17.74	42.84333333	12.06666667	-1.914745772	0.005839663	0.180170079
115489970	'Gm12790'	0	0	0	0	0	1	0	0.333333333	1.020273531	0.802557913	0.972790461
115489972	'Gm52720'	1864	2374.6	968.97	86.07	869.12	332.87	1735.843333	429.3533333	-2.058550208	0.003529477	0.137037276
115489981	'LOC115489981'	483.55	506.36	180.27	211.79	389.77	206.65	390.06	269.4033333	-0.432766556	0.383503647	0.945630828
11549	'Adra1a'	78.01	88.92	22.02	151	63	24	62.98333333	79.33333333	0.6915796	0.437200387	0.96622803
115490127	'Gm10461'	111.92	104.63	69.82	25.87	91.18	58.58	95.45666667	58.54333333	-0.713989474	0.142897299	0.749054337
115490131	'Gm42517'	44	52	9	0	28	27	35	18.33333333	-0.996977106	0.40138436	0.955860942
115490161	'Gm52800'	316	354.04	100.04	148.93	246.43	156.9	256.6933333	184.0866667	-0.37047797	0.480325345	0.972790461
115490169	'Gm52806'	3	2	0	7	6	4	1.666666667	5.666666667	1.96206083	0.156383063	0.767860225
115490184	'Gm42427'	241.86	189.27	306.44	312.99	528.28	528.7	245.8566667	456.6566667	0.876845879	0.0283559	0.410275773
115490191	'Gm52817'	1	5	0	4	1	2	2	2.333333333	0.517121931	0.775132613	0.972790461
115490200	'LOC115490200'	143.87	155.13	43.04	125.29	328.75	214.3	114.0133333	222.78	1.025892861	0.059355917	0.553094905
115490335	'Gm44180'	26	16.18	33.37	7	22.06	16	25.18333333	15.02	-0.793183953	0.250547349	0.871142318
115490369	'Gm52875'	0	1.1	0	2	0	0	0.366666667	0.666666667	1.337350669	0.73871782	0.972790461
115490387	'Gm10209'	15.17	18.59	2.06	1.02	10.15	4.11	11.94	5.093333333	-1.199339034	0.304617224	0.907164095
11550	'Adra1d'	14	16	19	35	15	19	16.33333333	23	0.657798124	0.38387966	0.945936404
11551	'Adra2a'	19	22	28	45	33	16	23	31.33333333	0.597833039	0.415666414	0.95722888
11552	'Adra2b'	3	2	0	3	2	2	1.666666667	2.333333333	0.687752361	0.680055684	0.972790461
11553	'Adra2c'	2	4	2	0	4	21	2.666666667	8.333333333	1.502193963	0.331690513	0.921648675
11554	'Adrb1'	5	1	29	31	4	1	11.66666667	12	0.279473098	0.859232398	0.976863639
11555	'Adrb2'	15	10	5	45	33	29	10	35.66666667	2.012808434	0.004487724	0.157418103
11556	'Adrb3'	4	3	2	1	0	8	3	3	-0.050190421	0.975868996	0.999493374
11564	'Adsl'	867	783	443	183	903	1021	697.6666667	702.3333333	-0.04544922	0.935032203	0.994413066
11565	'Adssl1'	82	84	274	113	54	109	146.6666667	92	-0.642421701	0.396408653	0.954259077
11566	'Adss'	2935	3021	2967	1121	2496	3814	2974.333333	2477	-0.316910767	0.429909881	0.962548446
11567	'Avil'	16	12	86	23	13	77	38	37.66666667	-0.173139567	0.866378208	0.978294615
11568	'Aebp1'	1940	1693	2226	1756	2213	2510	1953	2159.666667	0.167459464	0.622820476	0.972790461
11569	'Aebp2'	1690	1689	3147	2396	1728	2200	2175.333333	2108	0.015998298	0.976208024	0.999493374
11571	'Crisp1'	2	1	0	0	1	0	1	0.333333333	-1.329381507	0.692813154	0.972790461
11572	'Crisp3'	0	0	0	0	1	3	0	1.333333333	2.701386663	0.497818139	0.972790461

11576	'Afp'	3	4	1	1	1	1	2.666666667	1	-1.30273919	0.424657701	0.960285372
11593	'Aga'	368	388	206	133	514	416	320.6666667	354.3333333	0.123972965	0.793227756	0.972790461
11595	'Acan'	100	143	78	557	197	206	107	320	1.854181075	0.006961123	0.197529663
11596	'Ager'	24	22	21	21	36	14	22.33333333	23.66666667	0.152295129	0.809126045	0.972790461
11600	'Angpt1'	101	122	63	5	228	136	95.33333333	123	0.265667378	0.779514914	0.972790461
11601	'Angpt2'	333	369	12	83	474	545	238	367.3333333	0.632923068	0.52429695	0.972790461
11602	'Angpt4'	16	8	6	2	15	10	10	9	-0.184750282	0.843549048	0.975182082
11603	'Agrn'	5120	4685	2090	4172	5030	4525	3965	4575.666667	0.326254174	0.419136803	0.958308933
11604	'Agrp'	2	0	0	14	2	3	0.666666667	6.333333333	3.625917574	0.060845289	0.559918019
11605	'Gla'	292	282	633	333	264	368	402.3333333	321.6666667	-0.299646254	0.599947281	0.972790461
11606	'Agt'	18	24	56	185	48	95	32.66666667	109.3333333	1.894521426	0.024710903	0.389232557
11607	'Agtr1a'	137	164	21	25	88	59	107.3333333	57.33333333	-0.851975871	0.25494351	0.874595535
11608	'Agtr1b'	0	3	0	0	2	1	1	1	-0.007595086	0.998030128	0.999900097
11609	'Agtr2'	353	325	5	99	621	532	227.6666667	417.3333333	0.891602514	0.413355621	0.95722888
11610	'Agtrap'	537	537	255	377	658	945	443	660	0.601925118	0.125965823	0.72022881
11614	'Nr0b1'	320	336	154	51	297	186	270	178	-0.629821511	0.280738558	0.889418676
11615	'Gm4737'	1308.9	2123.7	1283.5	193.99	687.32	359.24	1572.033333	413.5166667	-1.938223555	4.24E-05	0.00752867
11622	'Ahr'	425	453	718	711	296	411	532	472.6666667	-0.019124633	0.975938028	0.999493374
11624	'Ahrr'	82	74	45	138	123	223	67	161.3333333	1.345026093	0.001605504	0.085771147
11625	'Ahsg'	3	7	0	0	2	0	3.333333333	0.666666667	-2.256980875	0.382120629	0.94434907
11628	'Aicda'	2	0	0	0	1	1	0.666666667	0.666666667	0.006630366	0.998451383	0.999900097
11629	'Aif1'	27	23	23	0	15	21	24.33333333	12	-1.157970018	0.267647679	0.883600995
11630	'Crybg1'	90	70	85	173	45	109	81.66666667	109	0.613077534	0.379199833	0.942621344
11632	'Aip'	778	873	1046	851	835	1035	899	907	0.061106755	0.874053515	0.980171118
11634	'Aire'	55	52	249	8	7	13	118.6666667	9.333333333	-3.749846127	1.63E-05	0.003636443
11636	'Ak1'	84	73	85	644	78	176	80.66666667	299.3333333	2.206457985	0.012485733	0.277311632
11637	'Ak2'	1443	1710	1837	3795	1490	2152	1663.333333	2479	0.761353107	0.203881586	0.832079769
11639	'Ak4'	1700.8	1506	5739.2	1032.4	1002.9	2546.78	2981.98	1527.383333	-1.072647121	0.135341658	0.736012522
11640	'Akap1'	845	857	584	2408	1022	1460	762	1630	1.309802763	0.019743138	0.349283865
11642	'Akap3'	168	174	31	1	118	43	124.3333333	54	-1.252310965	0.267923466	0.883600995
11643	'Akap4'	1	1	0	0	0	0	0.666666667	0	-1.703500596	0.673820112	0.972790461
11647	'Alpl'	48	51	69	46	45	75	56	55.33333333	-0.003181433	0.994840215	0.999562152
11648	'Akp3'	3	0	0	2	0	0	1	0.666666667	-0.051430757	0.989553961	0.999493374
11650	'Alppl2'	1	4	0	8	0	1	1.666666667	3	1.339494656	0.545306149	0.972790461
11651	'Akt1'	4086	4078	2933	3377	5170	5403	3699	4650	0.373144921	0.102870463	0.672865573
11652	'Akt2'	4099	4357	4106	2329	4253	5251	4187.333333	3944.333333	-0.099775746	0.737235531	0.972790461
11655	'Alas1'	807	820	692	3720	746	1133	773	1866.333333	1.576078031	0.040635194	0.473670329
11656	'Alas2'	84	82.98	153.99	5	395.99	70.98	106.99	157.3233333	0.381196114	0.729303456	0.972790461
11657	'Alb'	0	2	0	8	6	11	0.666666667	8.333333333	3.764028335	0.018436981	0.33834352
11658	'Alcam'	2416	2291	1011	3234	2773	2658	1906	2888.333333	0.76545815	0.097179703	0.663789939

116621581	'Derpc'	1537.4	1572	821.94	941.39	1377.4	1595.35	1310.433333	1304.7	0.053805201	0.864329688	0.977779046
11666	'Abcd1'	1367	1252	317	331	770	877	978.6666667	659.3333333	-0.526729221	0.346243139	0.927412588
11668	'Aldh1a1'	2959	3644	1483	5890	12464	16358	2695.333333	11570.66667	2.118232539	4.14E-07	1.79E-04
11669	'Aldh2'	3377	3793	2639	4733	4656	6315	3269.666667	5234.666667	0.759597313	0.013869786	0.292954545
11670	'Aldh3a1'	0	0	79	0	0	2	26.33333333	0.666666667	-5.634121185	0.146114009	0.753611197
116701	'Fgfrl1'	489	467	377	657	665	762	444.3333333	694.6666667	0.730535666	0.020597831	0.356011446
11671	'Aldh3a2'	1055	1152	1839	498	1512	1315	1348.666667	1108.333333	-0.369684606	0.454101662	0.970649024
116731	'Pcdha1'	0	0	0	0	0	2.44	0	0.813333333	1.749449488	0.66566965	0.972790461
116732	'Tsga13'	15	15	5	0	8	2	11.66666667	3.333333333	-1.83486723	0.142857381	0.749054337
116733	'Vps4a'	1383	1374	1185	785	1152	1451	1314	1129.333333	-0.197721192	0.433100045	0.964631919
11674	'Aldoa'	14081	12196	90988	24859	11282	23451	39088.33333	19864	-0.988784498	0.277680023	0.889355605
116748	'Lsm10'	171	205	374	140	187	272	250	199.6666667	-0.369578142	0.476260587	0.972790461
11676	'Aldoc'	492	461	2330	1137	230	726	1094.333333	697.6666667	-0.560738747	0.546877462	0.972790461
11677	'Akr1b3'	1270	1342	1388	136794	2150	29365	1333.333333	56103	5.765923303	4.12E-07	1.79E-04
116810	'Foxn4'	2	0	0	0	0	0	0.666666667	0	-1.711373851	0.67234292	0.972790461
11682	'Alk'	1	4	5	4	4	1	3.333333333	3	-0.05825071	0.966343152	0.999493374
116837	'Rims1'	32	38	16	3	41	66	28.66666667	36.66666667	0.264536042	0.768031422	0.972790461
116838	'Rims2'	394	411	202	19	296	228	335.6666667	181	-0.974023579	0.206141154	0.833294914
11684	'Alox12'	8	7	6	7	9	9	7	8.333333333	0.30628477	0.702300493	0.972790461
116847	'Prelp'	106	81	10	7	67	113	65.66666667	62.33333333	-0.116380981	0.905544962	0.988585749
116848	'Baz2a'	4719	4277	2277	3216	3253	3782	3757.666667	3417	-0.023132032	0.950022326	0.998065566
11685	'Alox12e'	9	2	0	20	0	1	3.666666667	7	1.476498976	0.482657431	0.972790461
11686	'Alox12b'	0	1	0	46	4	10	0.333333333	20	6.151903241	5.81E-04	0.047144852
11687	'Alox15'	197	198	41	199	136	115	145.3333333	150	0.284284585	0.677749512	0.972790461
116870	'Mta1'	2155	2059	3681	1478	1787	2675	2631.666667	1980	-0.43638502	0.343547999	0.925308103
116871	'Mta3'	1363	1421	965	1005	1590	1881	1249.666667	1492	0.287144263	0.258241254	0.877405867
116872	'Serpinb7'	1	1	1	1	0	0	1	0.333333333	-1.427293836	0.593581412	0.972790461
116873	'Stim2'	590	553	272	576	717	676	471.6666667	656.3333333	0.580710707	0.118559304	0.707707843
11688	'Alox8'	3	1	0	3	2	0	1.333333333	1.666666667	0.641728899	0.774203399	0.972790461
11689	'Alox5'	29	35	5	0	31	11	23	14	-0.758246833	0.554588335	0.972790461
116891	'Derl2'	824.03	793.09	906.03	461	947.07	1176.02	841.05	861.3633333	-0.003330226	0.992575557	0.999493374
11690	'Alox5ap'	10	7	1	23	27	44	6	31.33333333	2.469692906	0.002054004	0.100130046
116903	'Calcb'	0	3	4	17	7	4	2.333333333	9.333333333	2.192478034	0.115788284	0.702825844
116904	'Alpk3'	88	73	216	66	42	35	125.6666667	47.66666667	-1.315415668	0.072490345	0.595707787
116905	'Dph1'	362.59	339.17	243.11	202.97	391.09	412.45	314.9566667	335.5033333	0.106989307	0.716940581	0.972790461
116914	'Slc19a2'	396	380	701	903	588	844	492.3333333	778.3333333	0.728799524	0.182025227	0.802379693
11692	'Gfer'	253.07	252.27	463.31	139.07	249.6	438.46	322.8833333	275.71	-0.31826641	0.550181164	0.972790461
116939	'Pnpla3'	22	25	12	88	23	18	19.66666667	43	1.46756209	0.104805919	0.67684333
11694	'Alx3'	3	4	2	0	2	1	3	1	-1.615178348	0.341046156	0.925308103
116940	'Tgs1'	1173	1146	737	664	1181	972	1018.666667	939	-0.067980144	0.813615408	0.972790461

11695	'Alx4'	18	12	3	7	28	16	11	17	0.66109749	0.446488658	0.970198456
116972	'Tlcd3a'	190	169	415	44	171	149	258	121.3333333	-1.220582098	0.052425054	0.531897727
11698	'Ambn'	0	2	2	0	0	0	1.333333333	0	-2.855525825	0.472040045	0.972790461
11702	'Amd1'	2475.6	2539.9	1833.6	3231.1	1959.5	2404.99	2283.04	2531.863333	0.315609077	0.497030755	0.972790461
11705	'Amh'	20	22	10	7	45	18	17.33333333	23.33333333	0.414384188	0.594960058	0.972790461
117109	'Pop5'	210	171	200	331	192	346	193.6666667	289.6666667	0.685253614	0.155477467	0.766717687
117146	'Ube3b'	2412	2439	1575	1113	2348	2189	2142	1883.333333	-0.166675951	0.571479685	0.972790461
117147	'Acsm1'	0	0	0	0	1	0	0	0.333333333	1.020273531	0.802557913	0.972790461
117148	'Necab2'	67	81	93	8	49	59	80.33333333	38.66666667	-1.170191051	0.076038966	0.607417148
117149	'Tirap'	260	292	313	75	273	241	288.3333333	196.3333333	-0.623016252	0.181936971	0.802193703
117150	'Pip4k2c'	1290	1316	1392	1577	1159	1517	1332.666667	1417.666667	0.19006913	0.647716999	0.972790461
117160	'Ttyh2'	1248	1293	2132	1151	2537	1933	1557.666667	1873.666667	0.221267692	0.626550021	0.972790461
117167	'Steap4'	187	186	794	316	86	122	389	174.6666667	-1.016549032	0.263444539	0.88013169
11717	'Ampd3'	208	229	463.96	6362.8	186	1635.98	300.32	2728.266667	3.478813383	0.001507341	0.084039761
117171	'1110038F14Rik'	388	398	366	100	394	380	384	291.3333333	-0.464454819	0.321322982	0.917541683
117197	'Bloc1s4'	205	216	349	250	227	328	256.6666667	268.3333333	0.087258148	0.855113185	0.975734242
117198	'Ivns1abp'	6876	7238	4945	3522	7431	7727	6353	6226.666667	-0.022782242	0.937362969	0.994594529
11720	'Mat1a'	26	19	4	2	39	129	16.33333333	56.66666667	1.707067255	0.138786842	0.740560047
11722	'Amy1'	275	315	66	10	212	78	218.6666667	100	-1.164088576	0.200862156	0.828371216
117229	'Stk33'	327	320	94	7	143	113	247	87.66666667	-1.556379833	0.071372979	0.591776309
11727	'Ang'	6	8	5	25.11	24	21	6.333333333	23.37	1.991837548	0.004701267	0.16272038
11732	'Ank'	272	256	258	511	524	390	262	475	0.962673689	0.019570542	0.348059072
11733	'Ank1'	149	169	140	57	125	97	152.6666667	93	-0.705350784	0.046191052	0.50074283
11735	'Ank3'	770.02	754.04	1010	485	712.01	550.37	844.6866667	582.46	-0.511137251	0.185466174	0.805706284
11736	'Ankfy1'	3095	2927.3	1731.1	1569	2623.4	2418	2584.45	2203.473333	-0.174548526	0.552185433	0.972790461
11737	'Anp32a'	2620	2625	6962	2034	3077.8	3742	4069.013333	2951.27	-0.535015577	0.355461779	0.931809687
11739	'Slc25a4'	3386	3496	4569	2854	3961	4069	3817	3628	-0.0620663	0.86107988	0.976863639
11740	'Slc25a5'	1644	1923	3159	4517	1948	2589	2242	3018	0.573342787	0.369257683	0.937712122
11744	'Anxa11'	533	562	455	697	757	930	516.6666667	794.6666667	0.687169229	0.017591491	0.331280729
11745	'Anxa3'	305	270	890	365	427	340	488.3333333	377.3333333	-0.385437695	0.556385687	0.972790461
11746	'Anxa4'	982	1018	3172	4993	1159	1885	1724	2679	0.808197752	0.348320311	0.928444937
11747	'Anxa5'	2283	2392	9776	7881	2780	4791	4817	5150.666667	0.178529496	0.83045696	0.974233546
11749	'Anxa6'	4379	4487	4797	8994	5910	6391	4554.333333	7098.333333	0.77675321	0.100060326	0.671297438
11750	'Anxa7'	1024	951	3094	954	1602	3619	1689.666667	2058.333333	0.135808514	0.845004527	0.975182082
11752	'Anxa8'	1	3	8	5	3	4	4	4	0.026758898	0.983395968	0.999493374
11754	'Aoc3'	30	32	7	8	47	33	23	29.33333333	0.350112501	0.661361909	0.972790461
11757	'Prdx3'	2242	2541	1739	1162	2723	3440	2174	2441.666667	0.146515873	0.682080775	0.972790461
11758	'Prdx6'	1522	1560	3753	2305	1786	2244	2278.333333	2111.666667	-0.080369445	0.892763561	0.985171
117586	'A1bg'	33	38	6	69	33	11	25.66666667	37.66666667	0.899176894	0.358481041	0.933467324
117589	'Asb7'	643	683	564	412	587	622	630	540.3333333	-0.181933524	0.459815421	0.972790461

117590	'Asb10'	12	13	0	0	13	2	8.333333333	5	-0.741463158	0.686481927	0.972790461
117591	'Slc2a9'	39	27	28	12	56	52	31.33333333	40	0.292165351	0.633302034	0.972790461
117592	'B3galt6'	244	236	97	183	307	334	192.3333333	274.6666667	0.574923729	0.160141854	0.771114735
117599	'Helb'	398	361	514	460	388	377	424.3333333	408.3333333	0.03166573	0.94562757	0.996592403
117600	'Srgap1'	1070	1165	1568	594	1141	1143	1267.666667	959.3333333	-0.434570589	0.259352246	0.877955237
117606	'Boc'	1209	1241	297	477	2061	1841	915.6666667	1459.666667	0.675673368	0.281192407	0.889731741
11761	'Aox1'	84	78	107	124	139	508	89.66666667	257	1.442966056	0.017265369	0.327921282
11764	'Ap1b1'	1824	1696	1029	1283	1646	2223.04	1516.333333	1717.346667	0.237427191	0.437238712	0.96622803
11765	'Ap1g1'	4263	4184	2891	2464	3404	3586	3779.333333	3151.333333	-0.202855614	0.409538485	0.957157474
11766	'Ap1g2'	152	172	175	136	229	179	166.3333333	181.3333333	0.148309125	0.671990064	0.972790461
11767	'Ap1m1'	1705	1635	1144	585	1300	1310	1494.666667	1065	-0.484594414	0.11504973	0.702296207
11768	'Ap1m2'	201	201	34	21	122	256	145.3333333	133	-0.17051468	0.841434535	0.975182082
11769	'Ap1s1'	871	962	891	881	846	1211	908	979.3333333	0.167517154	0.614956085	0.972790461
11770	'Fabp4'	267	180	476	639	120	113	307.6666667	290.6666667	0.188964885	0.838129896	0.974723675
11771	'Ap2a1'	1550.1	1620.5	998.77	1628.5	1616.8	1842.48	1389.813333	1695.903333	0.392587793	0.237795046	0.863437626
11772	'Ap2a2'	2430	2462.9	3981.9	1765.9	1934	2426.97	2958.28	2042.29	-0.524688455	0.229007245	0.855540033
11773	'Ap2m1'	3827	3978	7206	3640	4368	6057	5003.666667	4688.333333	-0.116214203	0.805327611	0.972790461
11774	'Ap3b1'	2493	2564	2656	1513.1	2504	3268	2571	2428.37	-0.094688966	0.758626533	0.972790461
11775	'Ap3b2'	78	70	26	2	54	129	58	61.66666667	-0.014488038	0.988751116	0.999493374
11776	'Ap3d1'	4167	3921	3355	1391	3661	3615	3814.333333	2889	-0.426709159	0.214989152	0.842760284
11777	'Ap3s1'	924	957	2059	1774	946	1272	1313.333333	1330.666667	0.110016262	0.861695171	0.976863639
11778	'Ap3s2'	1539	1510	989	780	1121	1368	1346	1089.666667	-0.25954041	0.329688589	0.921648675
11781	'Ap4m1'	730.62	798.89	670.36	243.2	692	494.42	733.29	476.54	-0.637739361	0.084316447	0.631360496
11782	'Ap4s1'	178	213.01	102	134	175	136	164.3366667	148.3333333	-0.043075412	0.914499626	0.989964366
11783	'Apaf1'	1006	934	1239	1223	996	1007	1059.666667	1075.333333	0.11568377	0.796824639	0.972790461
11784	'Apba2'	17	23	9	0	13	26	16.33333333	13	-0.434902543	0.708946217	0.972790461
11785	'Apbb1'	618	576	777	319	505	887	657	570.3333333	-0.252656504	0.553939162	0.972790461
11787	'Apbb2'	727.76	767.24	667.13	2827.2	921.32	1161.31	720.71	1636.623333	1.431673373	0.029270692	0.417580391
11789	'Apc'	3386	3490	1993	3452	2819	2322	2956.333333	2864.333333	0.123806856	0.784816187	0.972790461
11790	'Speg'	835	869	858	594	1153	833	854	860	0.025359662	0.936905118	0.994413066
117903916	'Gm16286'	630.51	554.43	855.8	1004.3	620.68	724.86	680.2466667	783.2866667	0.318735131	0.547134732	0.972790461
11792	'Apex1'	1293	1475	1725	327	1316	1554	1497.666667	1065.666667	-0.594264552	0.255912736	0.876684814
11793	'Atg5'	656	625	529	350	591	813	603.3333333	584.6666667	-0.045677862	0.8795856	0.981807495
117934532	'Gm48552'	30	26	29	23	24	23	28.33333333	23.33333333	-0.21283689	0.68847145	0.972790461
11796	'Birc3'	283	275	1837	328	316	320	798.3333333	321.3333333	-1.367729586	0.111608239	0.695703551
11797	'Birc2'	2204	2290	1680	3610	2326	2104	2058	2680	0.56378668	0.253568184	0.872664697
11798	'Xiap'	4361	4519.9	2604	3708	3816	4357	3828.313333	3960.333333	0.153713617	0.643954169	0.972790461
11799	'Birc5'	691	734	183	152	461	730	536	447.6666667	-0.264106667	0.672123845	0.972790461
11800	'Api5'	3321	3335	2329	3188	2992	3532	2995	3237.333333	0.216120515	0.513580513	0.972790461
11801	'Cd5l'	0	0	0	6	4	102	0	37.33333333	7.522284413	7.68E-05	0.011410709

11803	'Aplp1'	379	283	359	978	292	522	340.3333333	597.3333333	1.022250907	0.121378333	0.712603747
11804	'Aplp2'	6614	6305	2285	8883	9389	9512	5068	9261.333333	1.008372594	0.027078142	0.403710299
11806	'Apoa1'	6	12	4	0	8	3	7.333333333	3.666666667	-1.050359526	0.424023541	0.960285372
11807	'Apoa2'	4	6	0	3	5	10	3.333333333	6	0.908297344	0.496328896	0.972790461
11808	'Apoa4'	0	2	1	0	2	0	1	0.666666667	-0.635245148	0.84132292	0.975182082
11810	'Apobec1'	19	26	19	23	39	55	21.33333333	39	0.873106075	0.085236438	0.634259952
11811	'Apobec2'	3	2	0	3	4	1	1.666666667	2.666666667	0.867333526	0.607690678	0.972790461
11812	'Apoc1'	140	145	28	102	128	172	104.3333333	134	0.46926	0.440507269	0.968551476
11813	'Apoc2'	32.08	39	5	7	29.48	131.23	25.36	55.90333333	1.078471779	0.296754714	0.90029104
11814	'Apoc3'	0	0	0	0	0	1	0	0.333333333	1.020273531	0.802557913	0.972790461
11815	'Apod'	11	0	0	0	0	1	3.666666667	0.333333333	-3.198625853	0.347956119	0.928444937
11816	'Apoe'	2049	2176	1834	7862	3135	6821	2019.666667	5939.333333	1.716260063	0.001513175	0.084118359
11818	'Apoh'	0	1	0	0	3	2	0.333333333	1.666666667	2.094832999	0.483632295	0.972790461
11819	'Nr2f2'	1166	1133	1026	669	1140	623	1108.333333	810.6666667	-0.392882375	0.29896274	0.902919716
11820	'App'	4846	4779	3174	6127	6869	8213	4266.333333	7069.666667	0.808963854	0.004750104	0.163012598
11821	'Aprt'	657	711	1002	370	672	729	790	590.3333333	-0.455298689	0.254526168	0.873948259
11826	'Aqp1'	75	88	11	230	95	38	58	121	1.433267795	0.132715226	0.731080419
11827	'Aqp2'	7	4	1	269	1	31	4	100.3333333	5.143693795	5.84E-04	0.047144852
11828	'Aqp3'	7	7	19	95	4	8	11	35.66666667	2.035138286	0.117905789	0.706573583
11829	'Aqp4'	6	8	0	0	9	5	4.666666667	4.666666667	-0.02142474	0.99040013	0.999493374
11830	'Aqp5'	46	62	5	806	42	620	37.66666667	489.3333333	4.013592845	2.27E-04	0.024847753
11831	'Aqp6'	0	0	0	1	0	0	0	0.333333333	1.020273531	0.802557913	0.972790461
11832	'Aqp7'	3	2	3	1	0	1	2.666666667	0.666666667	-1.914585927	0.297706204	0.900843503
11833	'Aqp8'	2	2	1	1	0	1	1.666666667	0.666666667	-1.177726546	0.56431884	0.972790461
11834	'Aqr'	1712	1566	1042	970	1861	2314	1440	1715	0.261951305	0.408885969	0.957157474
11835	'Ar'	187	200	108	264	226	181	165	223.6666667	0.601658002	0.201580735	0.82952222
11836	'Araf'	1610	1615	1592	1590	1755	1577	1605.666667	1640.666667	0.109412429	0.746821751	0.972790461
11837	'Rplp0'	17310	18001	38616	30793	18219	21247	24642.33333	23419.66667	0.015119411	0.980525946	0.999493374
11838	'Arc'	17	11	36	2684	6	213	21.33333333	967.6666667	5.86709853	5.22E-05	0.00848495
11839	'Areg'	3	6	24	62	4	5	11	23.66666667	1.38755028	0.31535781	0.914723408
11840	'Arf1'	5171	5261	12520	8392	5438	7331	7650.666667	7053.666667	-0.065358622	0.915350405	0.990364946
11841	'Arf2'	1041	1055	880	705	978	1145	992	942.6666667	-0.037747118	0.875193398	0.980678781
11842	'Arf3'	4978	4887	5862	3464	4334	5588	5242.333333	4462	-0.217755458	0.507145325	0.972790461
11843	'Arf4'	3657	3610	11392	3351	3442	4760	6219.666667	3851	-0.734732136	0.250477218	0.871106156
11844	'Arf5'	1516	1661	1787	973	1542	2246	1654.666667	1587	-0.081562924	0.813214051	0.972790461
118445	'Klf16'	376	347	923	392	307	402	548.6666667	367	-0.56092373	0.360676892	0.933706718
118446	'Gjc3'	5.93	5.31	2.01	2.87	2.96	1	4.416666667	2.276666667	-1.128634157	0.401000163	0.955860942
118449	'Synpo2'	129	148	45	38	106	78	107.3333333	74	-0.493914444	0.363635643	0.935022643
11845	'Arf6'	1716	1656	4469	3605	1830	2985	2613.666667	2806.666667	0.165913344	0.805012807	0.972790461
118451	'Mrps2'	615	688	860	318	637	837	721	597.3333333	-0.323337163	0.424734488	0.960285372

118452	'Baalc'	6	7	16	11.01	5	4	9.666666667	6.67	-0.409344678	0.692011109	0.972790461
118453	'Mmp28'	81.82	67.71	18.01	33	71.66	38.02	55.84666667	47.56	-0.129718564	0.840127002	0.974780034
118454	'Gjc2'	29	29	27	3	42	27	28.33333333	24	-0.339928215	0.675922358	0.972790461
11846	'Arg1'	61	62	3	210	48	92	42	116.6666667	1.831494757	0.075049087	0.603900623
11847	'Arg2'	12.01	12	75.18	92.39	6	12	33.06333333	36.79666667	0.400216498	0.754829966	0.972790461
11848	'Rhoa'	5951	5889	13295	5748	5897	7503	8378.333333	6382.666667	-0.400814137	0.457318603	0.972326244
11852	'Rhob'	1646	1730	9083	3878	1756	2280	4153	2638	-0.60037872	0.489853404	0.972790461
11853	'Rhoc'	1751	1895	4536	1925	1807	2194	2727.333333	1975.333333	-0.464230638	0.423988084	0.960285372
11854	'Rhod'	188	189	222	300	182	211	199.6666667	231	0.344152631	0.50352608	0.972790461
11855	'Arhgap5'	4719	4760	4948	3845	3705	3922	4809	3824	-0.249916429	0.482377749	0.972790461
11856	'Arhgap6'	158	142	574	260	147	158	291.3333333	188.3333333	-0.570833848	0.466718392	0.972790461
118567336	'LOC118567336'	510.73	418	342.94	3306	1148	596.91	423.89	1683.636667	2.304340389	0.003039521	0.123214035
118567343	'LOC118567343'	0	0	0	3	2	0	0	1.666666667	3.440499981	0.350686093	0.92886351
118567355	'LOC118567355'	55.2	48.56	28.25	12.91	30.2	26.38	44.00333333	23.16333333	-0.922043124	0.074406227	0.603245712
118567384	'LOC118567384'	57.59	79.66	23.08	42.6	65.01	72.97	53.44333333	60.19333333	0.247002668	0.636223	0.972790461
118567385	'LOC118567385'	0	1	0	0	0	0	0.333333333	0	-0.903279821	0.824807108	0.972790461
118567399	'LOC118567399'	2	3	0	1	4	0	1.666666667	1.666666667	0.120147207	0.956613898	0.999493374
118567439	'LOC118567439'	272.87	294.01	253.04	0	195.23	136.69	273.3066667	110.64	-1.454573466	0.231937831	0.858905111
118567440	'LOC118567440'	4.63	3.96	2.83	0	4.62	0	3.806666667	1.54	-1.213173562	0.541434157	0.972790461
118567464	'LOC118567464'	13.14	13.21	1.77	0	18	11.98	9.373333333	9.993333333	0.063109116	0.964821807	0.999493374
118567466	'LOC118567466'	2.9	1.59	0	0	0	3.28	1.496666667	1.093333333	-0.061186102	0.984156508	0.999493374
118567479	'LOC118567479'	963.87	1161	359.54	181.79	1990.8	808.88	828.1233333	993.8233333	0.229196131	0.758714529	0.972790461
118567481	'LOC118567481'	147.21	118.18	33.71	26.61	24.07	15.54	99.7	22.07333333	-2.003296666	0.003509485	0.136726071
118567495	'LOC118567495'	0	1	0	0	1	0	0.333333333	0.333333333	0.058500858	0.988561293	0.999493374
118567505	'LOC118567505'	28	34.42	5	1	16	5	22.47333333	7.333333333	-1.601491389	0.138511145	0.739741822
118567506	'LOC118567506'	1	0	0	0	1	0	0.333333333	0.333333333	0.058500858	0.988561293	0.999493374
118567551	'LOC118567551'	421.5	512.9	54.44	34.76	260.81	134.67	329.6133333	143.4133333	-1.194440716	0.157028555	0.768337387
118567557	'LOC118567557'	100.1	76.63	23.63	81.1	64.52	56.32	66.78666667	67.31333333	0.213162442	0.74107266	0.972790461
118567572	'LOC118567572'	1	1	1.7	0	0	0.99	1.233333333	0.33	-2.390783043	0.494152846	0.972790461
118567575	'LOC118567575'	0	0	0	0	0	1	0	0.333333333	1.020273531	0.802557913	0.972790461
118567621	'LOC118567621'	282.1	268.29	173.05	91.26	173.13	105.42	241.1466667	123.27	-0.909934036	0.017030387	0.327052234
118567625	'LOC118567625'	1.66	3.08	3.94	1.06	12.37	3.02	2.893333333	5.483333333	1.104388157	0.429492338	0.962386546
118567631	'LOC118567631'	503.95	504.87	72.08	20.25	254.38	108.71	360.3	127.78	-1.509603495	0.08707029	0.640379245
118567641	'LOC118567641'	675	564	733	1646	408	236	657.3333333	763.3333333	0.529801285	0.537447822	0.972790461
118567643	'LOC118567643'	36	33	4	10	21	11	24.33333333	14	-0.681008187	0.438424953	0.966976432
118567664	'LOC118567664'	162.99	155.98	39.07	475	146	97.03	119.3466667	239.3433333	1.383165538	0.108873674	0.68767651
118567759	'LOC118567759'	0	0.16	1.74	14.06	0	1.45	0.633333333	5.17	4.238142119	0.099832472	0.670676666
118567760	'LOC118567760'	0	0	0	1	0	0	0	0.333333333	1.020273531	0.802557913	0.972790461
118567769	'LOC118567769'	4725.6	5665.4	3862.8	181.7	3336.5	1408.79	4751.283333	1642.323333	-1.631369007	0.036943974	0.459648634
118567777	'LOC118567777'	0	0	1	0	0	1	0.333333333	0.333333333	0.058500858	0.988561293	0.999493374

118567781	'LOC118567781'	1	0	3.39	0	2.38	1	1.463333333	1.126666667	-0.626537636	0.794479535	0.972790461
118567800	'LOC118567800'	0	0	0	0	0	1	0	0.333333333	1.020273531	0.802557913	0.972790461
118567804	'LOC118567804'	0	1.26	0	0	2	0	0.42	0.666666667	0.839717162	0.835573887	0.974723675
118567805	'LOC118567805'	2.91	0	0	0	0	0	0.97	0	-1.711373851	0.67234292	0.972790461
118567814	'LOC118567814'	61.22	77.21	22.03	13.11	29.42	19.4	53.48666667	20.64333333	-1.317597069	0.03197017	0.433742328
118567837	'LOC118567837'	7.33	14.27	0.55	11.66	9.24	1.79	7.383333333	7.563333333	0.31575471	0.835436369	0.974723675
118567843	'LOC118567843'	26.5	18.28	3.3	4.7	12.82	10.95	16.02666667	9.49	-0.797741608	0.395417634	0.954014376
118567871	'LOC118567871'	433.08	508.82	103.25	352.72	477.25	290.68	348.3833333	373.55	0.258972118	0.668474985	0.972790461
118567886	'LOC118567886'	16.5	19	1	4	5.29	2	12.16666667	3.763333333	-1.519955737	0.208761973	0.836616817
118567893	'LOC118567893'	94.43	93.28	38.31	14.2	53.54	14.98	75.34	27.57333333	-1.426841955	0.03794494	0.459930654
118567918	'LOC118567918'	104	80.52	34.34	77.76	180.06	90.21	72.95333333	116.01	0.743473112	0.170530336	0.785702308
118567927	'LOC118567927'	106.29	125.32	27.75	14.31	53.53	40.39	86.45333333	36.07666667	-1.2401116	0.065581721	0.574593252
118567987	'LOC118567987'	87.17	111.95	104.32	11.83	53.16	40.24	101.1466667	35.07666667	-1.601420271	0.00372565	0.140118993
118567992	'LOC118567992'	83.29	78.13	46.51	40.13	87.63	88.7	69.31	72.15333333	0.075034958	0.854590809	0.975734242
118568006	'LOC118568006'	14	15	11	11	7	5	13.33333333	7.666666667	-0.639059298	0.434065378	0.965086068
118568020	'LOC118568020'	55	75	14	30	75	32	48	45.66666667	0.023265171	0.973680025	0.999493374
118568028	'LOC118568028'	10.74	12.36	2.94	4	10	5.87	8.68	6.623333333	-0.251721328	0.802530989	0.972790461
118568032	'LOC118568032'	371.58	343.56	119.17	128.49	192.53	98.73	278.1033333	139.9166667	-0.858287904	0.113505255	0.698948431
118568047	'LOC118568047'	15	21	3	1	8	12	13	7	-0.910027391	0.398535503	0.954563638
118568050	'LOC118568050'	106.14	109.17	17.04	41	130	67	77.45	79.33333333	0.106655771	0.882689624	0.982992879
118568052	'LOC118568052'	4	11	1	0	6	4	5.333333333	3.333333333	-0.704484236	0.64328977	0.972790461
118568053	'LOC118568053'	18.91	20.39	2	0	8.71	17.47	13.76666667	8.726666667	-0.736299123	0.583156353	0.972790461
118568056	'LOC118568056'	0	0	2	0	1	1	0.666666667	0.666666667	-0.259807793	0.938193093	0.994698142
118568062	'LOC118568062'	2540.2	3421.5	770.87	175.01	2012.8	829.44	2244.176667	1005.746667	-1.183076732	0.131345394	0.72897159
118568072	'LOC118568072'	166.94	212.15	68.9	65.33	193.7	114.73	149.33	124.5866667	-0.218538111	0.681020383	0.972790461
118568080	'LOC118568080'	140.74	179.98	92.76	81	83.04	81.16	137.8266667	81.73333333	-0.633508187	0.125263446	0.720137383
118568094	'LOC118568094'	483	449	168	292	345	193	366.6666667	276.6666667	-0.242890508	0.6482406	0.972790461
118568135	'LOC118568135'	0	3.46	11.53	3.75	2.15	4.03	4.996666667	3.31	-0.701247394	0.659466196	0.972790461
118568141	'LOC118568141'	5.18	0.75	0	0	0	0	1.976666667	0	-3.025187063	0.445317893	0.969888068
118568150	'LOC118568150'	2.87	3	0	0	0	0	1.956666667	0	-3.014977875	0.444538231	0.969888068
118568152	'LOC118568152'	110.8	141.9	85.65	147.16	189.63	237.18	112.7833333	191.3233333	0.823898014	0.012215313	0.273764981
118568183	'LOC118568183'	5	6	0	0	3	4	3.666666667	2.333333333	-0.673704114	0.719450579	0.972790461
118568219	'LOC118568219'	6	5	1	6	4	2	4	4	0.229199692	0.85483913	0.975734242
118568222	'LOC118568222'	1	0	1	1	0	0	0.666666667	0.333333333	-0.892941112	0.796499214	0.972790461
118568229	'LOC118568229'	0	5	0	0	3	0	1.666666667	1	-0.721785035	0.845558471	0.975182082
118568241	'LOC118568241'	133.24	132.68	134.27	52	69.61	65.25	133.3966667	62.28666667	-1.058643535	0.003272874	0.130175496
118568243	'LOC118568243'	97	80	20	16	59	24	65.66666667	33	-0.935896547	0.196783882	0.822696823
118568255	'LOC118568255'	0	0	1	4	0	3	0.333333333	2.333333333	2.917675885	0.278865072	0.889355605
118568281	'LOC118568281'	22.1	16.02	9.54	28.01	17	11	15.88666667	18.67	0.471833707	0.550054445	0.972790461
118568284	'LOC118568284'	42.87	57.12	11.78	35.25	51.42	26.3	37.25666667	37.65666667	0.176238416	0.80159873	0.972790461

118568297	'LOC118568297'	171.7	164.65	37.67	73.08	163.17	65.41	124.6733333	100.5533333	-0.193735084	0.769020462	0.972790461
118568299	'LOC118568299'	45.65	43.88	5.13	27.85	91.13	33.53	31.55333333	50.83666667	0.781878098	0.353203251	0.930050349
118568300	'LOC118568300'	109.28	99.36	24.08	49.44	67.38	25.1	77.57333333	47.30666667	-0.541896533	0.445795187	0.969915971
118568301	'LOC118568301'	92.18	89.06	18.24	25.4	26.56	22.28	66.49333333	24.74666667	-1.281521359	0.064274217	0.569422842
118568304	'LOC118568304'	26.85	35.83	5.37	7.01	17.44	3.01	22.68333333	9.153333333	-1.157174581	0.241961999	0.86449675
118568306	'LOC118568306'	158.54	152.26	44.43	143.96	157.7	87.25	118.41	129.6366667	0.311571164	0.607968229	0.972790461
118568307	'LOC118568307'	238.32	201.94	74.1	55.73	161.31	91.76	171.4533333	102.9333333	-0.691935518	0.205047519	0.833294914
118568308	'LOC118568308'	106.35	93.02	26.83	76.52	56.65	40.4	75.4	57.85666667	-0.161903689	0.811896633	0.972790461
118568310	'LOC118568310'	37.14	46.3	3.18	16.76	31.82	6.95	28.87333333	18.51	-0.522140438	0.613090442	0.972790461
118568311	'LOC118568311'	80.36	55.57	15.77	13.8	43.72	27.78	50.56666667	28.43333333	-0.805561709	0.257960216	0.877405867
118568312	'LOC118568312'	0	1	0	0	0	0	0.333333333	0	-0.903279821	0.824807108	0.972790461
118568322	'LOC118568322'	4	1	0	5	1	0	1.666666667	2	0.710945127	0.753109109	0.972790461
118568335	'LOC118568335'	37	40	32	62.07	42.83	20	36.33333333	41.63333333	0.386631371	0.57657152	0.972790461
118568337	'LOC118568337'	178.33	137.56	20.63	67.42	103.88	44.69	112.1733333	71.99666667	-0.475030074	0.540658677	0.972790461
118568338	'LOC118568338'	5	10	1	3	9	4	5.333333333	5.333333333	0.079847791	0.945516682	0.996592403
118568339	'LOC118568339'	27.28	34.24	12.98	31.48	16.28	10.83	24.83333333	19.53	-0.09846796	0.903451922	0.987564084
118568340	'LOC118568340'	49.17	57.58	12.65	59.21	69.25	30.71	39.8	53.05666667	0.612409521	0.395577213	0.954014376
118568343	'LOC118568343'	64.03	99.46	27.31	77.32	100.68	63.26	63.6	80.42	0.478968381	0.403380641	0.955996639
118568345	'LOC118568345'	73.19	68.04	21.97	49.91	85.41	30.79	54.4	55.37	0.153663498	0.816950664	0.972790461
118568348	'LOC118568348'	116.04	104.42	17.77	42.98	89.23	42.04	79.41	58.08333333	-0.335088797	0.645440255	0.972790461
118568354	'LOC118568354'	1.01	1.03	0	0	4	2	0.68	2	1.547991444	0.509349102	0.972790461
118568407	'C19H9orf57'	0	0	0	0	1	0	0	0.333333333	1.020273531	0.802557913	0.972790461
118568416	'LOC118568416'	2428	3152.3	1932.2	108.54	1623.9	626.03	2504.17	786.17	-1.756900966	0.019763344	0.349283865
118568424	'Akap17a'	324.14	310.04	422.12	399.37	525.06	551.83	352.1	492.0866667	0.499160531	0.176847017	0.796544771
118568432	'LOC118568432'	12.27	10.78	2.38	4.53	10.55	11.18	8.476666667	8.753333333	0.103654848	0.913064442	0.989773583
118568452	'Rpa4'	1	1	0	1	2	0	0.666666667	1	0.757462573	0.771089134	0.972790461
118568468	'LOC118568468'	0	0	0	1	0	0	0	0.333333333	1.020273531	0.802557913	0.972790461
118568482	'LOC118568482'	3	4	1	0	1	0	2.666666667	0.333333333	-2.780154366	0.20902219	0.836616817
118568567	'LOC118568567'	37.01	28	5.13	17	21	8	23.38	15.33333333	-0.414473082	0.639904378	0.972790461
118568585	'LOC118568585'	1	5.1	5.14	5	4.26	5	3.746666667	4.753333333	0.403158286	0.731719849	0.972790461
118568593	'LOC118568593'	838.45	755.95	232.59	225.11	907.39	426.48	608.9966667	519.66	-0.196410401	0.744378616	0.972790461
118568594	'LOC118568594'	3	1	0	0	2	1.28	1.333333333	1.093333333	-0.420603145	0.861776583	0.976863639
118568607	'LOC118568607'	85.05	140.49	121.2	0	0	0	115.58	0	-9.246153146	1.63E-12	2.38E-09
118568612	'LOC118568612'	3	2	0	0	1	3	1.666666667	1.333333333	-0.354282618	0.874222642	0.980171118
118568627	'LOC118568627'	0	3.02	1	3	3	1	1.34	2.333333333	0.966019457	0.592687302	0.972790461
118568632	'LOC118568632'	17.37	33.81	1.35	15.68	22.5	9.28	17.51	15.82	0.040709783	0.969777526	0.999493374
118568634	'LOC118568634'	314.13	282.99	48.72	98.58	253.77	120.26	215.28	157.5366667	-0.355355748	0.607964316	0.972790461
118568637	'LOC118568637'	0	0	0	0	2	1	0	1	2.320886	0.562538074	0.972790461
118568644	'LOC118568644'	194.63	154.43	39.14	25.94	172.01	96.38	129.4	98.11	-0.405094479	0.585400355	0.972790461
118568645	'LOC118568645'	1090.1	965.82	182.76	79.92	526.72	321.65	746.21	309.43	-1.271380435	0.088344107	0.643524199

118568650	'LOC118568650'	47	34	14.41	14	37	22	31.80333333	24.33333333	-0.325843302	0.61074929	0.972790461
118568652	'LOC118568652'	0	0	0	0	1	1	0	0.666666667	1.775692139	0.660844521	0.972790461
118568653	'LOC118568653'	0	1	0	0	0	0	0.333333333	0	-0.903279821	0.824807108	0.972790461
118568662	'LOC118568662'	0	0	0	0	0	1	0	0.333333333	1.020273531	0.802557913	0.972790461
118568683	'LOC118568683'	1654.5	1659.6	990.45	2159.6	3625.4	3117.43	1434.86	2967.46	1.106436376	1.35E-04	0.017243116
118568702	'LOC118568702'	7.07	11	6	1.01	12	6.05	8.023333333	6.353333333	-0.385746365	0.708940205	0.972790461
118568705	'LOC118568705'	692.79	731.86	1500.4	2866.1	4644.5	4553.97	975.03	4021.53	2.001263867	7.30E-05	0.01092959
118568709	'LOC118568709'	0	0	0	1	0	0	0	0.333333333	1.020273531	0.802557913	0.972790461
118568716	'LOC118568716'	0	1	0	0	0	0	0.333333333	0	-0.903279821	0.824807108	0.972790461
118568718	'LOC118568718'	1573	1822	858	179	2761	2939	1417.666667	1959.666667	0.372211116	0.635768961	0.972790461
118568721	'LOC118568721'	0	0	1	0	0	0	0.333333333	0	-0.903279821	0.824807108	0.972790461
118568751	'LOC118568751'	0	2	1	3	0	1	1	1.333333333	0.706510274	0.767677177	0.972790461
118568753	'LOC118568753'	6	9	5	2	6	1	6.666666667	3	-1.099747422	0.338840645	0.924650256
118568783	'LOC118568783'	3700.5	3745	1073.1	2258.9	3892.6	1912.52	2839.54	2688.003333	0.048869112	0.929104897	0.993096978
118568792	'LOC118568792'	234.69	199.95	56.34	53.92	106.55	54.04	163.66	71.50333333	-1.091471077	0.069424502	0.58740482
118568793	'LOC118568793'	2.51	5.1	0	4	210.17	45.08	2.536666667	86.41666667	5.173359364	1.36E-04	0.017287254
11857	'Arhgdib'	112	123	41	48	137	167	92	117.3333333	0.356683643	0.513958804	0.972790461
11858	'Rnd2'	970	1084	251	309	1701	1767	768.3333333	1259	0.692088483	0.30677483	0.907830798
11859	'Phox2a'	4	9	33	32	2	4	15.33333333	12.66666667	-0.018978842	0.988922405	0.999493374
11861	'Arl4a'	605	607	763	127	494	516	658.3333333	379	-0.890450799	0.076552838	0.609792246
11863	'Arnt'	1915	1881	3117	3041	2080	2116	2304.333333	2412.333333	0.167560729	0.756349819	0.972790461
11864	'Arnt2'	831	833	656	159	823	581	773.3333333	521	-0.629927438	0.218572933	0.844891257
11865	'Arntl'	333	313	943	1495	323	481	529.6666667	766.3333333	0.729316628	0.404619181	0.956674527
11867	'Arpc1b'	1767.7	1719	3359	2233	2082	3319	2281.9	2544.663333	0.153678706	0.761620857	0.972790461
11870	'Art1'	7	11	0	2	10	6	6	6	0.051898165	0.969796662	0.999493374
11872	'Art2b'	0	3	0	0	0	1	1	0.333333333	-1.313730691	0.742696629	0.972790461
11875	'Art5'	23	22	4	12	24	4	16.33333333	13.33333333	-0.139906087	0.886538631	0.984398578
11876	'Artn'	8	6	46	20	2	18	20	13.33333333	-0.551302506	0.650697267	0.972790461
11877	'Arvcf'	516	485	404	156	473	525	468.3333333	384.6666667	-0.326715703	0.424886906	0.960285372
11878	'Arx'	374	341	173	400	422	309	296	377	0.495619944	0.269106135	0.883851074
11881	'Arsb'	274	279	72	412	481	506	208.3333333	466.3333333	1.294063513	0.013926145	0.29304295
11883	'Arsa'	597	557	453	610	670	786	535.6666667	688.6666667	0.434482852	0.134029125	0.733301819
11886	'Asah1'	1185.9	1122.9	1321	3410.7	2108.8	2738.7	1209.923333	2752.74	1.310210915	0.005912245	0.181288429
11889	'Asgr1'	5	16	6	2	10	7	9	6.333333333	-0.518963543	0.599178546	0.972790461
11890	'Asgr2'	0	0	0	0	3	10	0	4.333333333	4.39216886	0.149928081	0.759066532
11891	'Rab27a'	51	87	107	38	64	65	81.66666667	55.66666667	-0.573491281	0.261335834	0.879383517
11898	'Ass1'	343	315	909	148	194	373	522.3333333	238.3333333	-1.228402891	0.0524288	0.531897727
11899	'Astn1'	32	64	7	3	81	49	34.33333333	44.33333333	0.331514685	0.75369722	0.972790461
11905	'Serpinc1'	1	2	2	1	0	1	1.666666667	0.666666667	-1.228731204	0.547662654	0.972790461
11906	'Zfhx3'	681	699	297	228	609	681	559	506	-0.137360539	0.760297264	0.972790461

11907	'Ate1'	1461.8	1407.8	1133	2226.2	1349.7	1526.51	1334.2	1700.793333	0.518770287	0.276471351	0.888034012
11908	'Atf1'	1218	1097	1747	1057.8	907.99	1202.03	1354	1055.926667	-0.312185553	0.489355278	0.972790461
11909	'Atf2'	2537	2633	2798	1297	2291	2250	2656	1946	-0.449041912	0.125847564	0.72022881
11910	'Atf3'	175	147	2491	391	131	242	937.6666667	254.6666667	-1.863955274	0.09741301	0.663789939
11911	'Atf4'	1778	1700	24927	3819	1925	3979	9468.333333	3241	-1.606715164	0.13146022	0.72897159
11920	'Atm'	2409	2426	852	930	2008	1572	1895.666667	1503.333333	-0.272635119	0.544775091	0.972790461
11921	'Atoh1'	0	0	0	3	0	0	0	1	2.899270347	0.465759809	0.972790461
11924	'Neurog2'	3	4	3	0	1	1	3.333333333	0.666666667	-2.34863448	0.179038027	0.798783498
11927	'Atox1'	547	504	419	609	429	568	490	535.3333333	0.25462066	0.539147852	0.972790461
11928	'Atp1a1'	6537	6550	3295	32214	8286	13971	5460.666667	18157	2.027357003	0.003831201	0.141968654
11931	'Atp1b1'	2596.5	2494.6	2689	34731	3977.9	13789.55	2593.363333	17499.43333	3.037686024	2.64E-04	0.027583361
11932	'Atp1b2'	1903	1975	344	3298	3924	2483	1407.333333	3235	1.383426999	0.03210941	0.433766761
11933	'Atp1b3'	1617	1712	978	2810	2240	2662	1435.666667	2570.666667	0.980377107	0.014386919	0.298933457
11936	'Fxyd2'	13	18	18	689	16	118	16.33333333	274.3333333	4.449926482	1.28E-04	0.01664332
11937	'Atp2a1'	8	7	5	8	6	4	6.666666667	6	-0.003062122	0.997405846	0.999723781
11938	'Atp2a2'	5063	4945	4479	11498	5205	8394	4829	8365.666667	0.963828437	0.066529631	0.576509273
11941	'Atp2b2'	11	17	4	1	10	9	10.66666667	6.666666667	-0.699064241	0.496677855	0.972790461
11944	'Atp4a'	30	49	117	21	46	56	65.33333333	41	-0.786644253	0.273656178	0.885016511
11945	'Atp4b'	0	0	0	0	1	0	0	0.333333333	1.020273531	0.802557913	0.972790461
11946	'Atp5a1'	10310	10988	10106	9665	10082	13044	10468	10930.33333	0.120357214	0.690123584	0.972790461
11947	'Atp5b'	11584	11893	16017	11530	12135	15412	13164.66667	13025.66667	0.014257509	0.970567935	0.999493374
11949	'Atp5c1'	2473	2633	3500	2157	2688	3084	2868.666667	2643	-0.10393823	0.779483076	0.972790461
11950	'Atp5pb'	6068	6258	5272	2017	3615	4917	5866	3516.333333	-0.747921423	0.013146887	0.286638321
11951	'Atp5g1'	1028	1088	1215	582	896	1199	1110.333333	892.3333333	-0.327459346	0.323029118	0.918843465
11957	'Atp5j'	1751	1864	1830	1785	1634	2023	1815	1814	0.075704368	0.828912154	0.973678583
11958	'Atp5k'	234	326	262	135	173	254	274	187.3333333	-0.528410692	0.126807316	0.721814579
11964	'Atp6v1a'	2191	2226	4729	5214	2019	3181	3048.666667	3471.333333	0.315669329	0.642978777	0.972790461
11966	'Atp6v1b2'	2117	2187	3733	2946	2428	3379	2679	2917.666667	0.160736556	0.742927664	0.972790461
11972	'Atp6v0d1'	1272	1306	2879	921	1176	1760	1819	1285.666667	-0.554009825	0.301723543	0.904789907
11973	'Atp6v1e1'	885	943	1661	1669	1064	1550	1163	1427.666667	0.365377031	0.504809989	0.972790461
11974	'Atp6v0e'	543	554	1057	1019	648	1120	718	929	0.418374077	0.453935556	0.970649024
11975	'Atp6v0a1'	923	1001	549	627	1340	1139	824.3333333	1035.333333	0.360870774	0.27258975	0.884738928
11977	'Atp7a'	1596	1485	1067	2169	2003	2244	1382.666667	2138.666667	0.740716885	0.032447347	0.434428844
11979	'Atp7b'	162	122	208	126	75	124	164	108.3333333	-0.517104643	0.341491869	0.925308103
11980	'Atp8a1'	808	820	261	927	1595	922	629.6666667	1148	0.98046818	0.051722306	0.528395745
11981	'Atp9a'	1146	1058	433	223	1656	2465	879	1448	0.648758116	0.362030628	0.934038037
11982	'Atp10a'	76	75	104	30	119	82	85	77	-0.217049986	0.689825342	0.972790461
11983	'Atpif1'	1436	1645	1899	1547	1748	2092	1660	1795.666667	0.146605869	0.678592753	0.972790461
11984	'Atp6v0c'	2775	2881	4326	3448	3051	4074	3327.333333	3524.333333	0.124273727	0.780281309	0.972790461
11987	'Slc7a1'	939	892	1781	4072	997	2019	1204	2362.666667	1.154085963	0.124295379	0.716961086

11988	'Slc7a2'	2233	1512	2996	1529	4322	3196	2247	3015.666667	0.356486194	0.466160516	0.972790461
11989	'Slc7a3'	35	38	221	76	54	77	98	69	-0.540913124	0.541929696	0.972790461
11990	'Atrn'	2072	1949	554	2808	2402	2379	1525	2529.666667	0.913547736	0.097102622	0.663789939
11991	'Hnrnpd'	4002	4045	3676	1926	3392	3275	3907.666667	2864.333333	-0.433918576	0.08143401	0.620530417
11992	'Auh'	307	310	539	494	302	357	385.3333333	384.3333333	0.096066029	0.866349935	0.978294615
11993	'Aup1'	766	791	670	511	857	860	742.3333333	742.6666667	0.022940543	0.925204054	0.992381554
11994	'Pcdh15'	163	126	327	22	51	131	205.3333333	68	-1.74907079	0.013724256	0.292512191
11997	'Akr1b7'	7	10	81	10186	22	665	32.66666667	3624.333333	7.08022401	4.27E-04	0.039568533
12000	'Avpr2'	8	3	0	4	4	2	3.666666667	3.333333333	0.072634086	0.961915224	0.999493374
12005	'Axin1'	2128	2203	1688	1756	1791	2392	2006.333333	1979.666667	0.053034387	0.857628272	0.976399524
12006	'Axin2'	1621	1664	413	1445	1647	2051	1232.666667	1714.333333	0.599026391	0.256027466	0.876684814
12007	'Azgp1'	0	0	0	1	0	0	0	0.333333333	1.020273531	0.802557913	0.972790461
12009	'Cep131'	937	864	449	371	812	767	750	650	-0.176646955	0.629886663	0.972790461
12010	'B2m'	1176	1261	3642	2142	1265	2138	2026.333333	1848.333333	-0.107312088	0.874337694	0.980175262
12012	'Baat'	5	4	11	16	3	4	6.666666667	7.666666667	0.418145562	0.724736428	0.972790461
12013	'Bach1'	1345	1280	3826	2654	1204	1754	2150.333333	1870.666667	-0.115156655	0.873092596	0.980171118
12014	'Bach2'	534	549	652	88	284	394	578.3333333	255.3333333	-1.270779457	0.010731276	0.255940608
12015	'Bad'	362.24	388.45	372	209.39	421	398.89	374.23	343.0933333	-0.131022008	0.670367338	0.972790461
12017	'Bag1'	2446	2517	3814	2080	2365	2856	2925.666667	2433.666667	-0.252442344	0.54287118	0.972790461
12018	'Bak1'	510	486	695	160	519	509	563.6666667	396	-0.593281978	0.212930534	0.841028891
12021	'Bard1'	273	270	56	19	177	150	199.6666667	115.3333333	-0.821893267	0.300355577	0.904081584
12022	'Barx1'	59	34	23	11	15	19	38.66666667	15	-1.305871591	0.026487686	0.399036357
12023	'Barx2'	12	20	116	25	3	9	49.33333333	12.33333333	-1.867684852	0.118332873	0.707231175
12028	'Bax'	554	603	1456	1062	639	1184	871	961.6666667	0.168717862	0.789952342	0.972790461
12029	'Bcl6b'	634	656	122	68	909	302	470.6666667	426.3333333	-0.158991214	0.85320293	0.975734242
12032	'Bcan'	14	13	2	10	26	45	9.666666667	27	1.49238673	0.081549452	0.620912368
12033	'Bcap29'	608	665	337	55	721	826	536.6666667	534	-0.102534602	0.893166618	0.985171
12034	'Phb2'	2212	2166	2959	1594	1897	2850	2445.666667	2113.666667	-0.213263799	0.578803176	0.972790461
12035	'Bcat1'	260	230	889	154	605	412	459.6666667	390.3333333	-0.3947568	0.591804219	0.972790461
12036	'Bcat2'	909	950	1739	884	1109	1698	1199.333333	1230.333333	-0.001444448	0.997622663	0.999778718
12038	'Bche'	173	226	53	7	630	255	150.6666667	297.3333333	0.916072407	0.395862731	0.954181778
12039	'Bckdha'	1017	1055	1592	834	1127	1439	1221.333333	1133.333333	-0.123283206	0.763091025	0.972790461
12040	'Bckdhb'	280	336	179	135	422	412	265	323	0.270008558	0.531382651	0.972790461
12041	'Bckdk'	830	812	511	590	1107	1393	717.6666667	1030	0.534322894	0.09414091	0.658197981
12042	'Bcl10'	521	549	2805	760	579	728	1291.666667	689	-0.931291081	0.248898969	0.869594312
12043	'Bcl2'	822	808	775	125	964	688	801.6666667	592.3333333	-0.535034652	0.379487321	0.942989536
12044	'Bcl2a1a'	5.57	4.37	9.33	0	3	0	6.423333333	1	-2.689055108	0.118277804	0.707231175
12045	'Bcl2a1b'	12.14	12.33	9.58	6	15.87	33	11.35	18.29	0.659511859	0.390206931	0.951958703
12046	'Bcl2a1c'	2	0	3	0	0	0	1.666666667	0	-3.215996145	0.397756436	0.954563638
12047	'Bcl2a1d'	2.3	6.3	21.09	1	1.13	0	9.896666667	0.71	-3.870434911	0.02206095	0.36727039

12048	'Bcl2l1'	959	947	482	1321	951	1185	796	1152.333333	0.693521269	0.121717876	0.712603747
12049	'Bcl2l10'	0	0	0	1	2	9	0	4	4.343801036	0.076490331	0.609792246
12050	'Bcl2l2'	1741	1870.8	1323.9	2052	2605.7	1901.62	1645.24	2186.453333	0.506091994	0.13598203	0.736689633
12051	'Bcl3'	147	129	849	457	101	224	375	260.6666667	-0.422723413	0.668451965	0.972790461
12053	'Bcl6'	188	166	1353	4243	217	607	569	1689	1.810265906	0.144373223	0.751183282
12054	'Bcl7b'	345	309	501	266	310	336	385	304	-0.321603875	0.452965199	0.970649024
12055	'Bcl7c'	897	876	902	214	924	963	891.6666667	700.3333333	-0.43395218	0.386539101	0.94848753
12057	'Opn1sw'	7	13	0	6	17	10	6.666666667	11	0.815660806	0.500409188	0.972790461
12061	'Bdkrb1'	1	0	2	5	1	2	1	2.666666667	1.573251662	0.396281803	0.954259077
12062	'Bdkrb2'	10	5	98	153	4	28	37.66666667	61.66666667	0.920975837	0.520087054	0.972790461
12064	'Bdnf'	3	2	30	19	7	21	11.66666667	15.66666667	0.382713504	0.756948426	0.972790461
12068	'Bet1'	611	621	935	345	524	710	722.3333333	526.3333333	-0.490781934	0.239163607	0.86449675
12069	'Bex2'	580.44	591.72	454.69	603	464	376	542.2833333	481	-0.011951835	0.979462205	0.999493374
12070	'Bex3'	1110.8	1150	1626.9	1493	1042	1261	1295.88	1265.333333	0.059451403	0.903354234	0.987555097
12075	'Bfsp1'	50	58	16	14	61	38	41.33333333	37.66666667	-0.117602438	0.861930571	0.976863639
12091	'Glb1'	447	388	353	485	589	595	396	556.3333333	0.555473885	0.056996873	0.547413448
12095	'Bglap3'	1	3	1	0	3	3	1.666666667	2	0.192319148	0.914745685	0.989989467
121021	'Cspg4'	271	285	108	188	519	398	221.3333333	368.3333333	0.765901399	0.101246626	0.671759705
121022	'Mrps6'	289	323	343	1353	248	583	318.3333333	728	1.4474362	0.056832292	0.547413448
12111	'Bgn'	1879	1676	3439	6424	2704	3663	2331.333333	4263.666667	1.009592868	0.124606769	0.717886026
12116	'Bhmt'	46	37	2	7	7	1	28.33333333	5	-2.24027703	0.065976071	0.574593252
12121	'Bicd1'	305	274	437	179	388	377	338.6666667	314.6666667	-0.155289536	0.718907384	0.972790461
12122	'Bid'	182	208	372	152	265	391	254	269.3333333	0.009579519	0.985216499	0.999493374
12123	'Hrk'	11	15	212	4	12	24	79.33333333	13.33333333	-2.838403666	0.018549019	0.339743696
12124	'Bik'	13	13	26	23	20	17	17.33333333	20	0.265833118	0.713630884	0.972790461
12125	'Bcl2l11'	644.93	606.95	852.97	1511	580	514	701.6166667	868.33	0.527766237	0.443101915	0.96975311
12140	'Fabp7'	38	51	35	1182	45	1616	41.33333333	947.6666667	4.659128624	2.96E-06	9.39E-04
12142	'Prdm1'	146	139	48	10	291	140	111	147	0.339765119	0.710532213	0.972790461
12143	'Blk'	1	5	0	2	2	0	2	1.333333333	-0.305544176	0.891530722	0.985171
12144	'Blm'	960	997	385	191	630	637	780.6666667	486	-0.68553753	0.168962188	0.784638279
12145	'Cxcr5'	2	2	1	0	1	1	1.666666667	0.666666667	-1.335893275	0.514852727	0.972790461
12151	'Bmi1'	1233	1339	1193	454	1153	1190	1255	932.3333333	-0.461044729	0.186927506	0.808483975
12153	'Bmp1'	2577	2500	1881	3253	3901	1998	2319.333333	3050.666667	0.522385749	0.243527223	0.865085867
12155	'Bmp15'	9	7	2	0	7	6	6	4.333333333	-0.520635902	0.698425527	0.972790461
12156	'Bmp2'	782	805	1262	180	799	738	949.6666667	572.3333333	-0.84771102	0.129447027	0.727256061
12159	'Bmp4'	102	50	115	67	86	116	89	89.66666667	-5.07E-04	0.9992256	0.999900097
12160	'Bmp5'	29	42	13	97	41	41	28	59.66666667	1.363046134	0.069330719	0.587268017
12161	'Bmp6'	95	98	358	1031	141	256	183.6666667	476	1.59439876	0.109361107	0.689609738
12162	'Bmp7'	299	354	223	199	280	393	292	290.6666667	0.026788934	0.931316309	0.993601701
12163	'Bmp8a'	4	2	2	15	3	3	2.666666667	7	1.704547977	0.192326392	0.814004755

12164	'Bmp8b'	3	2	2	35	0	6	2.333333333	13.66666667	2.951154309	0.06922433	0.586755664
12165	'Gdf2'	1	0	0	3	0	1	0.333333333	1.333333333	2.20694279	0.470369097	0.972790461
12166	'Bmpr1a'	3168	2906	2467	3015	4250	5028	2847	4097.666667	0.560306865	0.017589963	0.331280729
12167	'Bmpr1b'	182.12	169.43	24	10.05	409.1	327.26	125.1833333	248.8033333	0.938422263	0.370130049	0.937712122
12168	'Bmpr2'	2545	2563	3016	4276	3619	4306	2708	4067	0.667066119	0.095787859	0.662411648
12169	'Bmx'	0	2	1	0	1	4	1	1.666666667	0.600348378	0.799544839	0.972790461
12173	'Bnc1'	939	885	229	436	542	862	684.3333333	613.3333333	-0.069805914	0.89727193	0.985794067
12175	'Bnip2'	1594	1603	1610	2559	2065	2137	1602.333333	2253.666667	0.605154811	0.139164652	0.741642687
12176	'Bnip3'	2262	1814	24621	1277	948	3358	9565.666667	1861	-2.537790212	0.014438554	0.299352014
12177	'Bnip3l'	4841	4503	16836	3709	3715	5918	8726.743333	4447.333333	-1.036430081	0.129711342	0.727578716
12180	'Smyd1'	62	88	24	6	56	54	58	38.66666667	-0.628724453	0.425108905	0.960285372
12181	'Bop1'	623	604	271	611	590	769	499.3333333	656.6666667	0.512042352	0.210582596	0.839637916
12182	'Bst1'	6	16	12	31	10	18	11.33333333	19.66666667	0.981951437	0.260464799	0.878537553
12183	'Bpgm'	197	207	167	1501	480	1040	190.3333333	1007	2.603234401	1.88E-05	0.004028556
12189	'Brca1'	1193	1239	207	43	682	689	879.6666667	471.3333333	-0.949276779	0.287866369	0.894952226
12190	'Brca2'	3860	3972	576	124	1772	1413	2802.666667	1103	-1.38125684	0.116914408	0.705163127
12192	'Zfp36l1'	4103	4083	9438	24997	4915	6148	5874.666667	12020	1.274952187	0.144252294	0.750780289
12193	'Zfp36l2'	2100	1979	6032	1780	2184	2673	3370.333333	2212.333333	-0.660010538	0.282807422	0.890925385
12209	'Brs3'	8	6	3	0	0	0	5.666666667	0	-4.844784976	0.016660884	0.323882143
12211	'Birc6'	6268	6224	2174	4013	4805	5107	4888.666667	4641.666667	0.042138233	0.924024485	0.991932584
12212	'Chic1'	405.07	372.09	302	386.05	415	357	359.72	386.0166667	0.203338083	0.565833639	0.972790461
12215	'Bsg'	6724	5632	16115	6464	5943	11672	9490.333333	8026.333333	-0.290013241	0.633441235	0.972790461
12217	'Bsn'	226	184	316	38	207	106	242	117	-1.148482989	0.060451049	0.558329328
12223	'Btc'	57	46	90	46	94	170	64.33333333	103.3333333	0.587806391	0.312305258	0.911537035
12224	'Klf5'	42	61	361	722	33	88	154.6666667	281	1.11761314	0.377575003	0.941536807
12226	'Btg1'	1939	2027	10443	10417	1937	2846	4803	5066.666667	0.252724836	0.801195142	0.972790461
12227	'Btg2'	570	680	3000	1988	714	1086	1416.666667	1262.666667	-0.083132547	0.924022211	0.991932584
12228	'Btg3'	540	468	1867	2427	438	1193	958.3333333	1352.666667	0.640693162	0.478065375	0.972790461
12229	'Btk'	17	34	2	1	24	37	17.66666667	20.66666667	0.180774801	0.881716185	0.982528614
12231	'Btn1a1'	16	15	22	6	10	2	17.66666667	6	-1.504834619	0.088988948	0.645388896
12234	'Btrc'	1882	1792	1077	910	1589	1662	1583.666667	1387	-0.151934919	0.601797155	0.972790461
12235	'Bub1'	2294	2203	819	35	1074	1087	1772	732	-1.364298038	0.134997555	0.735841445
12236	'Bub1b'	746	762	192	1029	684	557	566.6666667	756.6666667	0.659848823	0.302274203	0.905122538
12237	'Bub3'	1035	1063	767	1153	1391	1392	955	1312	0.536061625	0.05407654	0.534315058
12238	'Commd3'	660	725	758	293	820	1049	714.3333333	720.6666667	-0.055423325	0.898279899	0.985862578
12257	'Tspo'	344	388	928	438	440	833	553.3333333	570.3333333	-0.00988019	0.986861431	0.999493374
12258	'Serping1'	562	555	613	713	496	560	576.6666667	589.6666667	0.153301366	0.73722054	0.972790461
12259	'C1qa'	20	29	2	6	54	136	17	65.33333333	1.890297208	0.081826833	0.621457906
12260	'C1qb'	39	47	7	17	86	228	31	110.3333333	1.782953205	0.04912039	0.515210762
12261	'C1qbp'	947	1029	2631	972	1080	1872	1535.666667	1308	-0.29441598	0.624145421	0.972790461

12262	'C1qc'	33	25	2	13	72	194	20	93	2.17592227	0.034800129	0.447645508
12263	'C2'	253	222	196	136	422	748	223.6666667	435.3333333	0.884487687	0.092656512	0.654099957
12265	'Ciita'	47.28	27.26	13.6	23.87	45.44	26.7	29.38	32.00333333	0.196016948	0.757927557	0.972790461
12266	'C3'	1050	797	12269	3561	477	1162	4705.333333	1733.333333	-1.312684616	0.270035912	0.883851074
12267	'C3ar1'	47	64	28	79	102	219	46.33333333	133.3333333	1.547689115	0.002069818	0.100386172
12268	'C4b'	79	69	43	219	53	117	63.66666667	129.6666667	1.286748147	0.075278462	0.60481391
12269	'C4bp'	0	0	0	1	1	1	0	1	2.526085132	0.463240869	0.972790461
12273	'C5ar1'	20	18	0	9	21	27	12.66666667	19	0.653530779	0.571383779	0.972790461
12274	'C6'	3	4	3	1	8	5	3.333333333	4.666666667	0.432358646	0.717498647	0.972790461
12279	'C9'	0	1	0	0	0	1	0.333333333	0.333333333	0.058500858	0.988561293	0.999493374
12282	'Hyou1'	2854	2434	5319	6554	2776	6925	3535.666667	5418.333333	0.681148781	0.282379625	0.890463
12283	'Cab39'	2657.7	2477	2770	3310	2532	3202.45	2634.906667	3014.806667	0.291376146	0.475728753	0.972790461
12286	'Cacna1a'	137	108	34	10	80	66	93	52	-0.868627411	0.247454451	0.868994456
12287	'Cacna1b'	135	121	33	0	76	23	96.33333333	33	-1.606685656	0.183794673	0.804729813
12288	'Cacna1c'	266	238	179	344	175	137	227.6666667	218.6666667	0.173707846	0.776156382	0.972790461
12289	'Cacna1d'	664.27	717.44	47.78	21.11	890.57	520.46	476.4966667	477.38	-0.031956956	0.97694662	0.999493374
12290	'Cacna1e'	23	14	3	7	10	2	13.33333333	6.333333333	-0.889905358	0.407510675	0.957157474
12291	'Cacna1g'	910	798	167	2513	755	411	625	1226.333333	1.366537354	0.129994193	0.727578716
12292	'Cacna1s'	2	2	2	0	2	0	2	0.666666667	-1.622445843	0.464525291	0.972790461
12293	'Cacna2d1'	12876	12264	5203	5699.8	11345	5717.7	10114.40667	7587.366667	-0.324019154	0.485876247	0.972790461
12294	'Cacna2d3'	42	54	11	46	48	66	35.66666667	53.33333333	0.70190123	0.263463603	0.88013169
12295	'Cacnb1'	335.09	320.58	678.68	318.96	463.26	303.77	444.7833333	361.9966667	-0.296873107	0.583447775	0.972790461
12296	'Cacnb2'	581	613	399	1066	1000	1015	531	1027	1.071766197	0.003132388	0.126171513
12297	'Cacnb3'	1015	993	1584	2166	1098	1377	1197.333333	1547	0.503005312	0.388843188	0.950889716
12298	'Cacnb4'	163	181	24	49	167	79	122.6666667	98.33333333	-0.248424597	0.739590064	0.972790461
12299	'Cacng1'	0	4	1	0	1	0	1.666666667	0.333333333	-2.11821893	0.487545988	0.972790461
12300	'Cacng2'	0	0	0	1	1	0	0	0.666666667	2.050872943	0.610671515	0.972790461
12301	'Cacybp'	2302	2295	1485	1386	2139	2809	2027.32	2111.333333	0.089216162	0.752357722	0.972790461
12304	'Pdia4'	2829	2368	8807	2880	2834	8443	4668	4719	-0.105527596	0.88414125	0.983632362
12305	'Ddr1'	1696	1541.9	1602	10719	2541.2	4330.01	1613.303333	5863.386667	2.120969432	0.002893053	0.120071993
12306	'Anxa2'	7861	8178	26528	6857	11271	14094	14189	10740.66667	-0.503210085	0.437262101	0.96622803
12307	'Calb1'	522	694	73	0	760	451	429.6666667	403.6666667	-0.144990909	0.920849559	0.991047523
12308	'Calb2'	3	7	2	0	5	3	4	2.666666667	-0.634158868	0.667896212	0.972790461
12309	'S100g'	0	0	0	2830	3	327	0	1053.333333	12.91478734	5.22E-05	0.00848495
12310	'Calca'	3	2	0	2	0	1	1.666666667	1	-0.416964608	0.855638784	0.975734242
12311	'Calcr'	1	1	0	0	1	1	0.666666667	0.666666667	0.015019958	0.995726078	0.999562152
12313	'Calm1'	5567	5671	4658	2219	5731	7313	5298.666667	5087.666667	-0.099534435	0.794294951	0.972790461
12314	'Calm2'	5087	5583	4673	4212	6113	6724	5114.333333	5683	0.184319737	0.43059188	0.962994098
12315	'Calm3'	3901	4299	3244	3209	4269	4602	3814.666667	4026.666667	0.131023298	0.582177625	0.972790461
12316	'Aspm'	940	1030	119	131	720	555	696.3333333	468.6666667	-0.558482911	0.475783926	0.972790461

12317	'Calr'	11262	10689	20437	9062	10893	24477	14129.33333	14810.66667	-0.001383418	0.997931641	0.999900097
12319	'Car8'	47	55	148	479	95	144	83.33333333	239.3333333	1.729840775	0.057034711	0.547413448
12321	'Calu'	5999	5875	5246	19509	7857	10556	5706.666667	12640.66667	1.357192341	0.019467427	0.347725165
12322	'Camk2a'	42	32	29	24	22	18	34.33333333	21.33333333	-0.575993853	0.302015409	0.904841461
12323	'Camk2b'	33	42	34	25	27	60	36.33333333	37.33333333	0.047725836	0.927562536	0.992951516
12325	'Camk2g'	1137	1079	744	1198	1088	979	986.6666667	1088.333333	0.276559908	0.481656783	0.972790461
12326	'Camk4'	140	84	40	30	60	13	88	34.33333333	-1.231263826	0.092164878	0.652602851
12328	'Caml'	433	446	399	204	550	577	426	443.6666667	0.02191236	0.953083494	0.999032683
12330	'Canx'	7336	6672	11330	6520	7374	14558	8446	9484	0.129823864	0.783858434	0.972790461
12331	'Cap1'	3398	3531	4695	5416	3758	4433	3874.666667	4535.666667	0.328730283	0.49316146	0.972790461
12332	'Capg'	266	293	559	773	508	637	372.6666667	639.3333333	0.850826601	0.141322446	0.746115932
12333	'Capn1'	589	654	709	337	859	869	650.6666667	688.3333333	0.034291023	0.928754484	0.993096978
12334	'Capn2'	1751	1758	3175	1137	2163	2376	2228	1892	-0.295730027	0.52505712	0.972790461
12335	'Capn3'	14	7	1	1	1	2	7.333333333	1.333333333	-2.353512133	0.101347711	0.671759705
12336	'Capns1'	3103	3376	5238	1864	3584	4331	3905.666667	3259.666667	-0.319749301	0.465827228	0.972790461
12337	'Capn5'	336	368	376	709	401	533	360	547.6666667	0.746301017	0.134778803	0.735480653
12338	'Capn6'	604	644	323	896	446	585	523.6666667	642.3333333	0.507347939	0.353745099	0.930465111
12339	'Capn7'	1561	1644	1467	643	1668	1399	1557.333333	1236.666667	-0.353121181	0.296813814	0.90029104
12340	'Capza1'	3593	3840	4251	2713	3628	3794	3894.666667	3378.333333	-0.178244818	0.565712772	0.972790461
12343	'Capza2'	2134	2308	3288	2337	2073	2728	2576.666667	2379.333333	-0.069836127	0.873252779	0.980171118
12345	'Capzb'	2771	2745	3872	4158	2801	4323	3129.333333	3760.666667	0.341122748	0.471829711	0.972790461
12346	'Car1'	2	2	0	0	0	0	1.333333333	0	-2.696289772	0.497898105	0.972790461
12348	'Car11'	59	54	27	33	68	50	46.66666667	50.33333333	0.162333777	0.731962172	0.972790461
12349	'Car2'	171	173	112	253	302	109	152	221.3333333	0.695357034	0.216560153	0.843391934
12350	'Car3'	7	9	3	2	12	1	6.333333333	5	-0.306619713	0.805348063	0.972790461
12351	'Car4'	3	2	0	2	10	2	1.666666667	4.666666667	1.545987725	0.329436263	0.921648675
12352	'Car5a'	0	2	1	0	0	1	1	0.333333333	-1.42378721	0.670046855	0.972790461
12353	'Car6'	1	1	499	1	0	29	167	10	-4.445284279	0.112804084	0.698666814
12354	'Car7'	34	36	10	4	26	36	26.66666667	22	-0.313626844	0.705260274	0.972790461
12355	'Nr1i3'	2.28	2.12	0	8.44	3.38	3.42	1.466666667	5.08	2.090544492	0.175859294	0.795298979
12359	'Cat'	1045	1158	2373	2213	1435	2463	1525.333333	2037	0.452592919	0.436654019	0.966208823
12361	'Cask'	1075	1066	1766	812	1062	1018	1302.333333	964	-0.426882228	0.327494539	0.920734892
12362	'Casp1'	10	16	4	10	9	10	10	9.666666667	0.085382591	0.918381636	0.990727847
12363	'Casp4'	38	35	427	242	23	83	166.6666667	116	-0.385985535	0.751441194	0.972790461
12364	'Casp12'	163	185	360	128	191	193	236	170.6666667	-0.500174107	0.336140496	0.922539021
12365	'Casp14'	1	0	0	1	0	0	0.333333333	0.333333333	0.058500858	0.988561293	0.999493374
12366	'Casp2'	1206	1347	1284	711	1252	1299	1279	1087.333333	-0.232077187	0.408535592	0.957157474
12367	'Casp3'	922	1052	1300	1241	1032	1051	1091.333333	1108	0.110498106	0.806006565	0.972790461
12368	'Casp6'	966	1132	1106	681	1912	1778	1068	1457	0.402567671	0.296032743	0.90029104
12369	'Casp7'	393	393	328	52	469	349	371.3333333	290	-0.453289362	0.482432685	0.972790461

12370	'Casp8'	458	509	964	97	606	639	643.6666667	447.3333333	-0.68807674	0.311523694	0.911537035
12371	'Casp9'	906	868	617	439	919	921	797	759.6666667	-0.058658072	0.841497775	0.975182082
12372	'Casq1'	7	5	6	6	9	2	6	5.666666667	0.009573815	0.992635935	0.999493374
12373	'Casq2'	4	1	1	6	3	2	2	3.666666667	1.0999038	0.442194718	0.969177389
12374	'Casr'	13	27	2	0	26	10	14	12	-0.256232265	0.854785128	0.975734242
12380	'Cast'	1046.7	1024.1	2226.1	771.83	1498.6	2136.05	1432.336667	1468.816667	-0.060441808	0.911375382	0.989773583
12385	'Ctnna1'	4938	5156	7477	6287	5472	6477	5857	6078.666667	0.111690233	0.802190148	0.972790461
12386	'Ctnna2'	174	195	98	13	315	162	155.6666667	163.3333333	-0.016711455	0.984300415	0.999493374
12387	'Ctnnb1'	15277	15348	11441	18806	17632	21050	14022	19162.66667	0.549340925	0.090267237	0.648589738
12388	'Ctnnd1'	9938	10601	11286	7682	12781	11411	10608.33333	10624.66667	0.015345785	0.958937596	0.999493374
12389	'Cav1'	5209	4956	2544	1368	5337	4289	4236.333333	3664.666667	-0.22622293	0.634128259	0.972790461
12390	'Cav2'	253	230	173	444	470	542	218.6666667	485.3333333	1.234662384	1.02E-04	0.013994201
12391	'Cav3'	2	1	1	5	1	2	1.333333333	2.666666667	1.236125699	0.441443477	0.968551476
12393	'Runx2'	55	41	392	187	45	98	162.6666667	110	-0.489322139	0.641253947	0.972790461
12394	'Runx1'	1211	1290	2006	1068	1551	1318	1502.333333	1312.333333	-0.190641263	0.65661753	0.972790461
12395	'Runx1t1'	163	154	61	6	158	120	126	94.66666667	-0.491421282	0.580124506	0.972790461
12396	'Cbfa2t2'	1745	1676	1438	1085	1278	1457	1619.666667	1273.333333	-0.286884913	0.280918972	0.889693736
12398	'Cbfa2t3'	201	216	134	100	163	160	183.6666667	141	-0.334732461	0.2769394	0.88878661
12399	'Runx3'	25	14	124	8	10	7	54.33333333	8.333333333	-2.773951552	0.005639268	0.177386075
12400	'Cbfb'	2108	2227	2254	1385	2427	2082	2196.333333	1964.666667	-0.147020653	0.613281241	0.972790461
12401	'Serpina6'	10	9	0	0	0	3	6.333333333	1	-2.68557031	0.239383126	0.86449675
12402	'Cbl'	2190	2156	1366	2066	2050	2028	1904	2048	0.223294551	0.525888595	0.972790461
12404	'Cbln1'	44	35	13	47	22	57	30.66666667	42	0.610457636	0.374594354	0.940406075
12405	'Cbln2'	3	7	4	1	7	6	4.666666667	4.666666667	-0.050710641	0.964487314	0.999493374
12406	'Serpinh1'	6055	6132	9836	4336	11214	16145	7341	10565	0.42313711	0.403357847	0.955996639
12408	'Cbr1'	423	441	534	718	413	527	466	552.6666667	0.376795658	0.463573056	0.972790461
12409	'Cbr2'	1	0	2	19	2	9	1	10	3.517482937	0.025100395	0.392118909
12411	'Cbs'	43	46	44	60	27	20	44.33333333	35.66666667	-0.093571352	0.896974345	0.985794067
12412	'Cbx1'	2397	2358	2092	1736	1879	2116	2282.333333	1910.333333	-0.186250437	0.523389959	0.972790461
12416	'Cbx2'	748	691	812	347	558	532	750.3333333	479	-0.636888214	0.041956311	0.477648737
12417	'Cbx3'	3323	3403	4963	2527	2980	3800	3896.333333	3102.333333	-0.322332829	0.419105036	0.958308933
12418	'Cbx4'	713	629	1863	967	771	972	1068.333333	903.3333333	-0.2284394	0.717765965	0.972790461
12419	'Cbx5'	6386	6264	5012	2813	5221	4933	5887.333333	4322.333333	-0.426288006	0.080290698	0.617305615
12421	'Rb1cc1'	2800	2912	2199	1697	2291	2252	2637	2080	-0.282150562	0.251058765	0.871167957
12424	'Cck'	5	1	31	12752	2	944	12.33333333	4566	8.812516118	1.40E-04	0.017654732
12425	'Cckar'	0	1	0	0	1	0	0.333333333	0.333333333	0.058500858	0.988561293	0.999493374
12426	'Cckbr'	0	0	1	3	1	0	0.333333333	1.333333333	2.219215901	0.463241285	0.972790461
12427	'Ccna1'	13.45	15.22	0	0	3	3.81	9.556666667	2.27	-2.222408305	0.201543164	0.82952222
12428	'Ccna2'	698.95	741.15	242	86.87	886.91	584.77	560.7	519.5166667	-0.15721781	0.828302269	0.973238667
12442	'Ccnb2'	696	705	100	53	497	685	500.3333333	411.6666667	-0.321826201	0.711466478	0.972790461

12443	'Ccnd1'	557.88	553.4	1064.8	85.8	549.32	894.2	725.36	509.7733333	-0.68966406	0.346468946	0.927886688
12444	'Ccnd2'	6286	6528	27703	1895	6635	9385	13505.66667	5971.666667	-1.380944531	0.080297746	0.617305615
12445	'Ccnd3'	2481.9	2530.5	3334.2	2135	2726.9	2862.94	2782.206667	2574.95	-0.090975572	0.801488536	0.972790461
12447	'Ccne1'	225	234	98	157	175	161	185.6666667	164.3333333	-0.05061692	0.907588664	0.988823459
12448	'Ccne2'	1019.2	1009.6	607.54	46.66	538.51	294.52	878.7533333	293.23	-1.660582338	0.017166403	0.327713554
12449	'Ccnf'	274	312	132	138	339	317	239.3333333	264.6666667	0.169573412	0.687648066	0.972790461
12450	'Ccng1'	1223	1179	2765	696	1630	1995	1722.333333	1440.333333	-0.368505248	0.515522638	0.972790461
12452	'Ccng2'	2341	2089	7201	1156	1427	1240	3877	1274.333333	-1.637324029	0.012089571	0.271772812
12453	'Ccni'	5559	5397	10782	6114	4565	7049	7246	5909.333333	-0.262579512	0.624480951	0.972790461
12454	'Ccnk'	1620.4	1533.6	1715.1	842.44	933.7	1249.23	1623.013333	1008.456667	-0.651810577	0.04393187	0.488595692
12455	'Ccnt1'	1915	1918	1957	5139	1749	2142	1930	3010	0.874931405	0.178648564	0.798304697
12457	'Noct'	307	242	1348	256	237	428	632.3333333	307	-1.129318373	0.151714001	0.76105187
12458	'Ccr6'	0	0	0	0	2	0	0	0.666666667	1.801491674	0.656112935	0.972790461
12460	'Ccs'	318	355	337	131	343	441	336.6666667	305	-0.196286311	0.635965794	0.972790461
12461	'Cct2'	6912	6600	8707	5741	5318	8523	7406.333333	6527.333333	-0.152182108	0.70117417	0.972790461
12462	'Cct3'	5799	5942	4830	2247	5051	6215	5523.666667	4504.333333	-0.318321172	0.340235418	0.925269028
12464	'Cct4'	4857	4732	9624	3458	4148	5435	6404.333333	4347	-0.583202581	0.2400221	0.86449675
12465	'Cct5'	5516	5546	8231	4383	5287	6345	6431	5338.333333	-0.260709288	0.512825678	0.972790461
12466	'Cct6a'	5175	5174	7066	2105	4972	6717	5805	4598	-0.411499267	0.350041685	0.92886351
12467	'Cct6b'	18	19	4	16	12	21	13.66666667	16.33333333	0.393281359	0.61820136	0.972790461
12468	'Cct7'	5623	5850	6476	4849	4297	6037.99	5983	5061.33	-0.180683264	0.625614536	0.972790461
12469	'Cct8'	6484.8	6463.8	7568.9	4031	5297	6644.71	6839.14	5324.236667	-0.349267262	0.272454496	0.884738928
12475	'Cd14'	24	26	190	312	12	23	80	115.6666667	0.807175166	0.549270178	0.972790461
12476	'Cd151'	1619	1724	1530	1182	2882	4231	1624.333333	2765	0.715226882	0.078933151	0.616000706
12477	'Ctla4'	2	3	1	0	0	0	2	0	-3.337472277	0.23673828	0.862272407
12478	'Cd19'	1	0	0	0	0	0	0.333333333	0	-0.903279821	0.824807108	0.972790461
12479	'Cd1d1'	182	188	43	14	309	140	137.6666667	154.3333333	0.120880308	0.89423135	0.985229858
12482	'Ms4a1'	0	1	0	0	7	23	0.333333333	10	4.636510165	0.03798487	0.459930654
12483	'Cd22'	2	1	2	63	3	14	1.666666667	26.66666667	4.342822393	0.001563628	0.084694293
12484	'Cd24a'	790	859	1297	3517	1063	780	982	1786.666667	1.117973897	0.158065126	0.768890221
12487	'Cd28'	1	3	2	4	3	30	2	12.33333333	2.538355892	0.052632845	0.532526438
12488	'Cd2ap'	4332	4694	4878	13732	4291	5751	4634.666667	7924.666667	1.008279734	0.129961359	0.727578716
12489	'Cd33'	3	2	1	16	7	8	2	10.33333333	2.586123949	0.023668097	0.380370122
12490	'Cd34'	636	665	78	534	1428	1097	459.6666667	1019.666667	1.222497374	0.084758584	0.633355364
12491	'Cd36'	174	178	15	447	337	1156	122.3333333	646.6666667	2.502336696	0.002378983	0.109249332
12492	'Scarb2'	934	797	860	2085	1028	1464	863.6666667	1525.666667	0.982664053	0.060481235	0.558329328
12493	'Cd37'	6	6	5	10	16	25	5.666666667	17	1.587805144	0.033319606	0.440523189
12494	'Cd38'	77	61	28	16	126	151	55.33333333	97.66666667	0.755484004	0.30497788	0.907188578
12495	'Entpd1'	86	79	10	32	121	126	58.33333333	93	0.695429779	0.373953701	0.939802744
12496	'Entpd2'	48	51	35	20	36	89	44.66666667	48.33333333	0.077461002	0.894013529	0.985171

12497	'Entpd6'	340	354	121	100	489	738	271.6666667	442.3333333	0.657404078	0.31553626	0.914723408
12499	'Entpd5'	441	458	324	789	536	574	407.6666667	633	0.795557191	0.080600121	0.617641878
12500	'Cd3d'	0	0	1	0	0	0	0.333333333	0	-0.903279821	0.824807108	0.972790461
12501	'Cd3e'	1	2	3	0	1	6	2	2.333333333	0.04060056	0.982107456	0.999493374
12502	'Cd3g'	1	0	0	0	1	0	0.333333333	0.333333333	0.058500858	0.988561293	0.999493374
12503	'Cd247'	66.46	58	18	25	67	39	47.48666667	43.66666667	-0.055346431	0.928427921	0.993096978
12504	'Cd4'	4	3	4	0	12	21	3.666666667	11	1.423500365	0.295157275	0.90029104
12505	'Cd44'	951	967	6043	229	1359	1278	2653.666667	955.3333333	-1.714485242	0.0597696	0.554643744
12506	'Cd48'	2	9	1	3	8	27	4	12.66666667	1.632220578	0.180773103	0.801479605
12507	'Cd5'	1	0	0	0	0	2	0.333333333	0.666666667	0.78767502	0.845746136	0.975182082
12508	'Cd53'	33	28	11	83	58	123	24	88	1.989265205	6.42E-04	0.049996188
12509	'Cd59a'	260.83	262.81	76	149.95	273.88	348.91	199.88	257.58	0.418156963	0.413320017	0.95722888
12511	'Cd6'	2	3	1	2	1	0	2	1	-0.740150203	0.696252611	0.972790461
12512	'Cd63'	2471	2579	4247	6118	3610	5822	3099	5183.333333	0.819751896	0.128210687	0.724799196
12514	'Cd68'	53	44	236	148	87	270	111	168.3333333	0.521687326	0.53728695	0.972790461
12515	'Cd69'	1	0	4	0	1	0	1.666666667	0.333333333	-2.306357808	0.442339592	0.969201374
12516	'Cd7'	1	0	6	0	0	2	2.333333333	0.666666667	-2.065383171	0.460912889	0.972790461
12517	'Cd72'	14.25	14.3	7.22	17.22	13.1	8.18	11.92333333	12.83333333	0.296993665	0.708418613	0.972790461
12518	'Cd79a'	1	3	4	3	1	5	2.666666667	3	0.189003525	0.892720902	0.985171
12519	'Cd80'	5	9	0	29	12	11	4.666666667	17.33333333	2.210436684	0.065708311	0.574593252
12520	'Cd81'	3004.3	3013.5	2788.2	6212.8	4222.5	5091.19	2935.316667	5175.49	0.950769988	0.028112655	0.409247928
12521	'Cd82'	665	677	949	720	991	1276	763.6666667	995.6666667	0.371216804	0.333724831	0.921648675
12522	'Cd83'	130	115	303	32	147	100	182.6666667	93	-1.111596826	0.10125358	0.671759705
12523	'Cd84'	100	91	75	51	100	157	88.66666667	102.6666667	0.191971194	0.648164063	0.972790461
12524	'Cd86'	5	8	5	2	8	12	6	7.333333333	0.240076457	0.804518096	0.972790461
12525	'Cd8a'	8	11	10	0	9	7	9.666666667	5.333333333	-0.973148489	0.39368166	0.953178011
12527	'Cd9'	341	358	298	4108	698	1486	332.3333333	2097.333333	2.953824799	1.45E-04	0.018115704
12530	'Cdc25a'	1092	1062	912	301	940	991	1022	744	-0.504654124	0.212810006	0.841028891
12531	'Cdc25b'	249	269	114	288	364	229	210.6666667	293.6666667	0.614741279	0.185670787	0.80592991
12532	'Cdc25c'	65	64	19	18	53	30	49.33333333	33.66666667	-0.496973759	0.431705974	0.963677723
12534	'Cdk1'	626	642	284	355	752	780	517.3333333	629	0.314922061	0.42170145	0.959384282
12537	'Cdk11b'	3324	3225	2338	2076	2141	2711	2962.333333	2309.333333	-0.277611931	0.350473159	0.92886351
12539	'Cdc37'	2528	2664	3345	3370	2756	3496	2845.666667	3207.333333	0.245170868	0.561390868	0.972790461
12540	'Cdc42'	8044	8151	11876	9376	7696	9971	9357	9014.333333	0.005435817	0.990339508	0.999493374
12544	'Cdc45'	340	358	164	54	239	202	287.3333333	165	-0.822870117	0.11985191	0.710102513
12545	'Cdc7'	1018	995	268	195	688	481	760.3333333	454.6666667	-0.71262942	0.216969806	0.843391934
12549	'Arhgap31'	856	824	662	1645	1174	1252	780.6666667	1357	0.94520811	0.029409785	0.418863909
12550	'Cdh1'	161	147	38	518	104	195	115.3333333	272.3333333	1.588983614	0.059100756	0.552865869
12552	'Cdh11'	2690	2429	1823	1182	2831	2269	2314	2094	-0.135993745	0.669492623	0.972790461
12554	'Cdh13'	30	20	2	15	66	82	17.33333333	54.33333333	1.651915493	0.069572362	0.587499452

12555	'Cdh15'	3	3	4	1	7	2	3.333333333	3.333333333	-0.052705867	0.967660979	0.999493374
12556	'Cdh16'	10	5	0	139	33	15	5	62.33333333	4.077266026	0.001273649	0.075434925
12557	'Cdh17'	19	19	9	5	19	39	15.66666667	21	0.372041248	0.639813318	0.972790461
12558	'Cdh2'	1739	1773	2264	840	1932	2705	1925.333333	1825.666667	-0.146773461	0.733387517	0.972790461
12560	'Cdh3'	711	593	339	642	568	748	547.6666667	652.6666667	0.372524243	0.348112079	0.928444937
12561	'Cdh4'	33	26	8	6	33	26	22.33333333	21.66666667	-0.048743773	0.950586389	0.998065566
12562	'Cdh5'	747	682	167	7	1294	1087	532	796	0.502983905	0.664784415	0.972790461
12563	'Cdh6'	152	125	102	8	94	39	126.3333333	47	-1.500549311	0.0388683	0.461389364
12564	'Cdh8'	4	2	1	0	8	5	2.333333333	4.333333333	0.8224292	0.602629342	0.972790461
12565	'Cdh9'	7	6	4	2	1	3	5.666666667	2	-1.425661099	0.231826339	0.858905111
12566	'Cdk2'	1471	1505	1052	659	1351	1173	1342.666667	1061	-0.317869063	0.256014931	0.876684814
12567	'Cdk4'	4058	4753	6617	1907	4971	4681	5142.666667	3853	-0.484584279	0.275119267	0.886386629
12568	'Cdk5'	674	869	310	556	1076	890	617.6666667	840.6666667	0.510354242	0.222468841	0.84974371
12569	'Cdk5r1'	122	125	70	65	76	45	105.6666667	62	-0.639268742	0.186107953	0.807018167
12570	'Cdk5r2'	10	10	5	3	4	4	8.333333333	3.666666667	-1.112539378	0.23823092	0.864053647
12571	'Cdk6'	1347	1390	1785	281	1660	1536	1507.333333	1159	-0.499101458	0.395193459	0.954014376
12572	'Cdk7'	1692.8	1692	1397.7	670	1700	1766	1594.15	1378.666667	-0.233670301	0.492266212	0.972790461
12575	'Cdkn1a'	641	573	8531	2095	738	1734	3248.333333	1522.333333	-1.109553149	0.308122307	0.907963267
12576	'Cdkn1b'	3338	3723	6808	2389	6327	5334	4623	4683.333333	-0.064998697	0.898790384	0.986081296
12577	'Cdkn1c'	8076	8030	6911	2712	6868	4858	7672.333333	4812.666667	-0.676225251	0.043221413	0.484697385
12578	'Cdkn2a'	63	54	139	18	28	180	85.33333333	75.33333333	-0.371597124	0.676781549	0.972790461
12579	'Cdkn2b'	34	18	227	563	20	119	93	234	1.543772142	0.232650309	0.859679981
12580	'Cdkn2c'	111	105	35	35	95	62	83.66666667	64	-0.33578747	0.54536987	0.972790461
12581	'Cdkn2d'	232	220	538	514	210	339	330	354.3333333	0.210398454	0.76338072	0.972790461
12583	'Cdo1'	58	76	64	30	84	57	66	57	-0.223040756	0.627256427	0.972790461
12585	'Cdr2'	307	267	315	434	395	239	296.3333333	356	0.392986534	0.436399357	0.965982602
12587	'Mia'	70	57	6	7	103	70	44.33333333	60	0.413656775	0.679290253	0.972790461
12589	'Ift81'	1073	1009	405	296	936	781	829	671	-0.292072883	0.54431746	0.972790461
12590	'Cdx1'	39.5	38.98	8	2	13	16	28.82666667	10.33333333	-1.464156226	0.100227048	0.671535889
12591	'Cdx2'	0	1	3	0	0	0	1.333333333	0	-2.933515612	0.459648379	0.972790461
12593	'Cdyl'	882	808	949	949	739	1209	879.6666667	965.6666667	0.199288045	0.622042222	0.972790461
12606	'Cebpa'	155	139	294	144	199	306	196	216.3333333	0.087382064	0.867861362	0.979099229
12607	'Cebpz'	1759	1718	1755	628	1472	1453	1744	1184.333333	-0.586201835	0.077967517	0.61481478
12608	'Cebpb'	225	239	2666	3502	276	1410	1043.333333	1729.333333	0.85450207	0.476659459	0.972790461
12609	'Cebpd'	142.75	123.76	2430.4	268.22	110.47	176.1	898.9566667	184.93	-2.287977225	0.045587193	0.498244985
12611	'Cebpg'	945.93	902.84	3131	918	841	1243	1659.913333	1000.666667	-0.768087659	0.252723466	0.871252558
12613	'Cel'	1	2	0	0	3	1	1	1.333333333	0.405502065	0.866582138	0.978294615
12614	'Celsr1'	755	660	184	748	1134	1011	533	964.3333333	0.964507975	0.062768088	0.566066012
12615	'Cenpa'	205	221	159	140	247	187	195	191.3333333	0.018048559	0.954445356	0.999493374
12616	'Cenpb'	1599	1769	1509	966	2073	1998	1625.666667	1679	0.039270366	0.893254468	0.985171

12617	'Cenpc1'	960.92	913.58	502.21	518.72	886.99	812.98	792.2366667	739.5633333	-0.042931377	0.893074708	0.985171
12622	'Cer1'	0	0	0	0	0	1	0	0.333333333	1.020273531	0.802557913	0.972790461
12623	'Ces1g'	4	4	0	1	5	2	2.666666667	2.666666667	0.061846647	0.969715839	0.999493374
12626	'Cetn3'	1194	1415	1502	460	1392	1365	1370.333333	1072.333333	-0.416501387	0.323950576	0.919383393
12627	'Cfc1'	0	0	0	0	2	4	0	2	3.287939522	0.381618281	0.94429001
12628	'Cfh'	1785.6	1777.9	1056.2	311	3031	2425.39	1539.87	1922.463333	0.23937917	0.719447375	0.972790461
12630	'Cfi'	19	20	5	11	28	21	14.66666667	20	0.501092047	0.489480955	0.972790461
12631	'Cfl1'	5788	6504	9286	4543	6431	7797	7192.666667	6257	-0.216857056	0.586741031	0.972790461
12632	'Cfl2'	1919	1844	3996	1260	1979	2540	2586.333333	1926.333333	-0.487582701	0.342645847	0.925308103
12633	'Cflar'	1476.2	1449.9	2628.7	2084.8	1584	1765.92	1851.613333	1811.576667	0.037244174	0.94377325	0.996592403
12638	'Cftr'	199	211	102	16	204	142	170.6666667	120.6666667	-0.57238789	0.439601517	0.96756958
12640	'Cga'	20	21	4	1	19	3	15	7.666666667	-0.969118472	0.412061777	0.95722888
12642	'Ch25h'	9	5	67	15	2	7	27	8	-1.670533856	0.194057319	0.818052716
12643	'Chad'	17	24	3	39	9	13	14.66666667	20.33333333	0.82168906	0.42831845	0.962191208
12647	'Chat'	1	0	0	1	1	1	0.333333333	1	1.558384074	0.565724181	0.972790461
12648	'Chd1'	1614	1610	1584	2094	1737	1691	1602.666667	1840.666667	0.315666036	0.439724152	0.967599153
12649	'Chek1'	987	1016	318	107	669	752	773.6666667	509.3333333	-0.643908934	0.33582136	0.922539021
12651	'Chkb'	334	366	507	434	502	427	402.3333333	454.3333333	0.220822647	0.609496872	0.972790461
12652	'Chga'	59	53	19	0	14	17	43.66666667	10.33333333	-2.151217749	0.032044641	0.433742328
12653	'Chgb'	11	10	7	2	9	5	9.333333333	5.333333333	-0.810718538	0.371368619	0.938527219
12654	'Chil1'	13	8	300	70	17	20	107	35.66666667	-1.495874368	0.265556222	0.881878584
12655	'Chil3'	5	4	2	0	23	1	3.666666667	8	1.056792272	0.529371708	0.972790461
12659	'Ovgp1'	118	135	42	323	80	45	98.33333333	149.3333333	0.996708641	0.276617937	0.888054748
12660	'Chka'	2019	2231	1996.1	9028	3313	2209	2082.016667	4850	1.495316305	0.037417635	0.459930654
12661	'Chl1'	16	22	9	1	28	12	15.66666667	13.66666667	-0.259470036	0.797913349	0.972790461
12662	'Chm'	914	975	1327	493	923	991	1072	802.3333333	-0.452646585	0.241579305	0.86449675
12663	'Chml'	546	554	393	124	544	428	497.6666667	365.3333333	-0.491638427	0.30329732	0.906364139
12667	'Chrd'	148	99	52	67	79	69	99.66666667	71.66666667	-0.357934077	0.464689533	0.972790461
12669	'Chrm1'	12	5	0	8	5	8	5.666666667	7	0.504191785	0.700562234	0.972790461
12671	'Chrm3'	8	1	26	15	16	23	11.66666667	18	0.541559693	0.624358738	0.972790461
12672	'Chrm4'	1497	1635	176	116	1147	831	1102.666667	698	-0.673841345	0.436987232	0.96622803
12675	'Chuk'	1610	1699	1015	1339	1674	1267	1441.333333	1426.666667	0.091912068	0.79401929	0.972790461
12677	'Vsx2'	0	0	0	1	1	4	0	2	3.406172247	0.238166183	0.864053647
12683	'Cidea'	0	0	3	5	0	1	1	2	1.178652789	0.67141348	0.972790461
12684	'Cideb'	7.49	10.49	1.07	0	10.29	8.26	6.35	6.183333333	-0.042942351	0.976183657	0.999493374
12686	'Elovl3'	1	1	0	23	0	0	0.666666667	7.666666667	4.080542132	0.110429628	0.691390221
12695	'Patj'	964	929	610	143	1095	951	834.3333333	729.6666667	-0.27192841	0.65967905	0.972790461
12696	'Cirbp'	2249	2231	715	177	1641	1867	1731.666667	1228.333333	-0.554774933	0.453029824	0.970649024
12700	'Cish'	123	120	216	72	174	161	153	135.6666667	-0.242427909	0.631497307	0.972790461
12702	'Socs3'	250	207	559	1124	283	314	338.6666667	573.6666667	0.973730833	0.250596095	0.871152488

12703	'Socs1'	60	46	145	59	78	68	83.66666667	68.33333333	-0.319104841	0.623348547	0.972790461
12704	'Cit'	672	603	76	41	376	279	450.3333333	232	-0.969881525	0.257327898	0.877405867
12705	'Cited1'	206	172	343	27	97	145	240.3333333	89.66666667	-1.556776306	0.012264538	0.273764981
12709	'Ckb'	384	359	131	873	479	1821	291.3333333	1057.666667	1.952983832	0.001269049	0.07539735
12715	'Ckm'	0	0	0	1	1	1	0	1	2.526085132	0.463240869	0.972790461
12716	'Ckmt1'	11	12	5	6	10	6	9.333333333	7.333333333	-0.26272759	0.752107433	0.972790461
12721	'Coro1a'	44	49	6	28	86	53	33	55.66666667	0.820642804	0.285454687	0.893820325
12722	'Clca3a1'	1204.3	1418.2	803.74	1568	2431.5	1509.94	1142.073333	1836.466667	0.775216303	0.036132856	0.456753899
12723	'Clcn1'	78.83	62.46	12.3	45.01	47	26.72	51.19666667	39.57666667	-0.164346911	0.828075275	0.973238667
12724	'Clcn2'	984.42	987.87	230.3	504.26	1661.7	981.75	734.1966667	1049.25	0.563994208	0.347685284	0.928444937
12725	'Clcn3'	1524	1429	1753	1743	1757	2026	1568.666667	1842	0.287582691	0.430190098	0.962548446
12727	'Clcn4'	744	799	707	787	1117	855	750	919.6666667	0.354028832	0.257031347	0.8773831
12728	'Clcn5'	1191	1188	1503	2366	1258	1481	1294	1701.666667	0.545127167	0.31636549	0.915207044
12729	'Clns1a'	1249	1293	1228	495	1164	1445	1256.666667	1034.666667	-0.320870309	0.377354622	0.941504733
12733	'Clcnka'	4	7	5	9	10	24	5.333333333	14.33333333	1.428505698	0.088211111	0.643048174
12737	'Cldn1'	21	29	60	32	28	34	36.66666667	31.33333333	-0.211422237	0.757469719	0.972790461
12738	'Cldn2'	29	31	0	14	21	7	20	14	-0.309773181	0.808010863	0.972790461
12739	'Cldn3'	9	5	23	51	10	22	12.33333333	27.66666667	1.328903896	0.197413177	0.822893955
12740	'Cldn4'	22	14	700	2610	56	243	245.3333333	969.6666667	2.207945463	0.268507652	0.883600995
12741	'Cldn5'	295	276	10	4	596	310	193.6666667	303.3333333	0.611308296	0.637995049	0.972790461
12745	'Clgn'	27	24	6	0	23	6	19	9.666666667	-1.019935215	0.417921074	0.957720764
12747	'Clk1'	2608	2745	3217	4425	3385	2131	2856.666667	3313.666667	0.36174432	0.501997756	0.972790461
12748	'Clk2'	761.04	761.43	807.74	366.72	954.57	696.1	776.7366667	672.4633333	-0.231703101	0.529487335	0.972790461
12750	'Clk4'	1193	1165	2025	1258	1457	872	1461	1195.666667	-0.232554418	0.657357066	0.972790461
12751	'Tpp1'	1037	1017	890	2224	1971	2208	981.3333333	2134.333333	1.223295316	5.84E-04	0.047144852
12752	'Cln3'	460	493	235	441	809	808	396	686	0.839593323	0.015745609	0.31182866
12753	'Clock'	1911	1897	885	1323	1643	1466	1564.333333	1477.333333	0.028684846	0.939657551	0.995607401
12757	'Clta'	2007	1871	3708	3405	2232	3089	2528.666667	2908.666667	0.267900318	0.62999662	0.972790461
12759	'Clu'	2703	3142	7699	22734	12233	16481	4514.666667	17149.33333	2.000179752	0.00288755	0.120071993
12763	'Cmah'	558.62	501.97	161.26	160	357.05	226.99	407.2833333	248.0133333	-0.638479389	0.21687352	0.843391934
12764	'Cmas'	765	786	1691	796	848	1341	1080.666667	995	-0.151022262	0.778543801	0.972790461
12765	'Cxcr2'	2.96	6	2.34	3.03	6	3.45	3.766666667	4.16	0.333672114	0.780987368	0.972790461
12766	'Cxcr3'	0	0	0	0	1	0	0	0.333333333	1.020273531	0.802557913	0.972790461
12767	'Cxcr4'	227	217	92	140	765	672	178.6666667	525.6666667	1.521388706	0.010177534	0.249993887
12768	'Ccr1'	6	4	1	34	7	17	3.666666667	19.33333333	2.689344555	0.013765593	0.292512191
12769	'Ccr9'	5	5	0	5	4	0	3.333333333	3	0.16865308	0.929329612	0.993098341
12770	'Ccr1l1'	2	1	1	0	0	0	1.333333333	0	-2.782259126	0.384965334	0.94674509
12771	'Ccr3'	2	2	0	0	0	0	1.333333333	0	-2.696289772	0.497898105	0.972790461
12772	'Ccr2'	0	0	0	0	3	2	0	1.666666667	3.04513816	0.441518888	0.968551476
12773	'Ccr4'	0	2	0	0	0	3	0.666666667	1	0.52034112	0.895931272	0.985560686

12774	'Ccr5'	3	5	1	6	7	3	3	5.333333333	0.988371967	0.405792004	0.957157474
12775	'Ccr7'	1	5	3	5	4	3	3	4	0.535257566	0.668286388	0.972790461
12776	'Ccr8'	1	2	0	0	0	1	1	0.333333333	-1.323989397	0.695217833	0.972790461
12777	'Ccr10'	22	21	8	10	20	20	17	16.66666667	0.020128327	0.975489792	0.999493374
12778	'Ackr3'	100	91	32	19	276	250	74.33333333	181.6666667	1.221654961	0.137391192	0.738626463
12780	'Abcc2'	21	16	6	1	58	3	14.33333333	20.66666667	0.481675213	0.715340065	0.972790461
12785	'Cnbp'	5900	6191	9230	5757	6109	7938	7107	6601.333333	-0.089468273	0.830939875	0.974324192
12788	'Cnga1'	3	5	0	0	1	2	2.666666667	1	-1.417436104	0.509301815	0.972790461
12789	'Cnga2'	1	4	1	5	8	3	2	5.333333333	1.528430022	0.238937951	0.864286212
12790	'Cnga3'	2	1	0	0	0	2	1	0.666666667	-0.591584204	0.853544845	0.975734242
12793	'Cnih1'	836	874	1414	833	996	1502	1041.333333	1110.333333	0.072468607	0.871099368	0.980126495
12794	'Cnih2'	63	69	21	16	63	39	51	39.33333333	-0.352538334	0.58086334	0.972790461
12795	'Plk3'	64	73	141	589	75	152	92.66666667	272	1.819975386	0.050434581	0.519327729
12796	'Camp'	2	8	40	4	38	2	16.66666667	14.66666667	-0.387530825	0.781681476	0.972790461
12797	'Cnn1'	19	23	17	15	19	10	19.66666667	14.66666667	-0.329234299	0.611017141	0.972790461
12798	'Cnn2'	830	817	1271	1342	829	827	972.6666667	999.3333333	0.163530284	0.769431783	0.972790461
12799	'Cnp'	316	300	181	294	399	461	265.6666667	384.6666667	0.596286198	0.050399053	0.519327729
12801	'Cnr1'	548	615	270	15	622	406	477.6666667	347.6666667	-0.551615756	0.551828481	0.972790461
12802	'Cnr2'	3	10	1	6	15	7	4.666666667	9.333333333	1.088413662	0.321889483	0.917918531
12803	'Cntf'	8	11	40	36	10	10	19.66666667	18.66666667	0.083968443	0.937311043	0.994594529
12804	'Cntfr'	130.02	101.01	181.95	121	117	146	137.66	128	-0.070080677	0.883933008	0.98358443
12805	'Cntn1'	34	47	15	20	41	21	32	27.33333333	-0.141407113	0.820595531	0.972790461
12807	'Hps3'	878	916	302	683	1111	665	698.6666667	819.6666667	0.347906538	0.47913948	0.972790461
12808	'Cobl'	200	168	115	70	154	157	161	127	-0.329478392	0.366219253	0.936406787
12810	'Coch'	66	94	48	3	86	34	69.33333333	41	-0.842103379	0.349647729	0.92886351
12812	'Coil'	378	376	266	105	362	335	340	267.3333333	-0.380000623	0.375067074	0.940406075
12813	'Col10a1'	0	1	0	2	1	3	0.333333333	2	2.552084926	0.271508148	0.884495016
12814	'Col11a1'	256	261	23	350	355	186	180	297	0.954661622	0.243033552	0.864948315
12815	'Col11a2'	126	132	137	169	107	52	131.6666667	109.3333333	-0.069360189	0.91596285	0.990491125
12816	'Col12a1'	3180	2960	344	210	3175	2092	2161.333333	1825.666667	-0.270219234	0.768865218	0.972790461
12817	'Col13a1'	179	124	19	236	76	49	107.3333333	120.3333333	0.547387711	0.565454361	0.972790461
12818	'Col14a1'	316	261	40	41	350	197	205.6666667	196	-0.073338744	0.929746103	0.993164002
12819	'Col15a1'	346	314	674	995	711	768	444.6666667	824.6666667	0.961983403	0.09686245	0.663693139
12821	'Col17a1'	41	44	6	3	33	35	30.33333333	23.66666667	-0.386071642	0.690226808	0.972790461
12822	'Col18a1'	2634	2263	370	7069	4188	4510	1755.666667	5255.666667	1.834848507	0.011065857	0.259994952
12823	'Col19a1'	55	45	8	5	25	13	36	14.33333333	-1.291325579	0.133633521	0.733301819
12824	'Col2a1'	57	45	9	9	30	18	37	19	-0.904247596	0.248150114	0.869491261
12825	'Col3a1'	19583	18535	5478	3805	26921	17423	14532	16049.66667	0.121797654	0.856149069	0.975734242
12826	'Col4a1'	7646	6962	6419	28937	9216	11351	7009	16501.33333	1.489818679	0.02476391	0.38974458
12827	'Col4a2'	4263	4030	3277	11419	5869	5836	3856.666667	7708	1.205866675	0.027806631	0.407916408

12828	'Col4a3'	40	37	16	227	14	14	31	85	1.929015842	0.101585131	0.671759705
12829	'Col4a4'	133	99	27	488	37	52	86.33333333	192.3333333	1.63065073	0.139457848	0.741642687
12830	'Col4a5'	2487	2624	532	5815	4258	4187	1881	4753.333333	1.556568323	0.014031787	0.293375506
12831	'Col5a1'	5140	4613	1743	7899	6339	4486	3832	6241.333333	0.913653649	0.102268502	0.671776916
12832	'Col5a2'	2677	2385	1046	1032	4098	2289	2036	2473	0.289897787	0.577260734	0.972790461
12833	'Col6a1'	6857	6671	1897	4454	9138	5581	5141.666667	6391	0.407233927	0.435751127	0.96581102
12834	'Col6a2'	7047	6386	2026	2268	8755	5740	5153	5587.666667	0.135609527	0.809575669	0.972790461
12835	'Col6a3'	2447	2177	682	1748	2650	2267	1768.666667	2221.666667	0.436888721	0.368584762	0.937530612
12836	'Col7a1'	222	228	31	140	170	65	160.3333333	125	-0.14645317	0.850228629	0.975734242
12837	'Col8a1'	88	128	8	19	463	359	74.66666667	280.3333333	1.874019158	0.075688619	0.605636377
12839	'Col9a1'	6	5	0	1	4	0	3.666666667	1.666666667	-1.01078265	0.617446241	0.972790461
12840	'Col9a2'	1105	1173	101	546	1571	631	793	916	0.312411492	0.696978495	0.972790461
12841	'Col9a3'	34	47.18	12	32	115	104	31.06	83.66666667	1.440408143	0.021798688	0.365836629
12842	'Col1a1'	6948	6175	1140	5647	9573	7099	4754.333333	7439.666667	0.767091431	0.214002588	0.842446242
12843	'Col1a2'	7925	7176	1268	5498	9925	6691	5456.333333	7371.333333	0.554151134	0.379116477	0.942578399
12845	'Comp'	12	11	2	0	6	8	8.333333333	4.666666667	-0.883655277	0.502676022	0.972790461
12846	'Comt'	473	439.06	649.04	187	648	737	520.3666667	524	-0.090144618	0.859672345	0.976863639
12847	'Copa'	10817	9998	11599	5835	8154	11454	10804.66667	8481	-0.351985993	0.273218829	0.884738928
12848	'Cops2'	3392	3471	6941	2268	3030	3545	4601.333333	2947.666667	-0.671985217	0.172885216	0.790917985
12850	'Coq7'	293	350	325	103	223	275	322.6666667	200.3333333	-0.719072732	0.05125869	0.525036139
12854	'Cort'	0	2	1	0	1	0	1	0.333333333	-1.423774587	0.66900121	0.972790461
12856	'Cox17'	323	308	423	604	217	399	351.3333333	406.6666667	0.372399601	0.54880683	0.972790461
12857	'Cox4i1'	4459	4760	4720	5625	3552	5449	4646.333333	4875.333333	0.181824284	0.679885532	0.972790461
12858	'Cox5a'	1175	1252	1183	873	862	1338	1203.333333	1024.333333	-0.186211737	0.574310334	0.972790461
12859	'Cox5b'	1086.2	1242	1446	491	1057	1381	1258.056667	976.3333333	-0.42031576	0.293900697	0.90029104
12861	'Cox6a1'	1175	1235	1346	4172	1319	2263	1252	2584.666667	1.251010302	0.049621345	0.516509085
12862	'Cox6a2'	4	1	108	0	0	30	37.66666667	10	-2.307179898	0.267247694	0.883600995
12864	'Cox6c'	1959	2518	1290	1551	1733	2308	1922.333333	1864	0.035731385	0.914455083	0.989964366
12865	'Cox7a1'	27	33	31	19	28	29	30.33333333	25.33333333	-0.234593802	0.632737191	0.972790461
12866	'Cox7a2'	1071	1088	1026	902	560	945	1061.666667	802.3333333	-0.294331383	0.501127078	0.972790461
12867	'Cox7c'	2721	3010	3808	1851	1696	2316	3179.666667	1954.333333	-0.656754135	0.103857306	0.675747811
12868	'Cox8a'	816	905	1637	1661	1029	1362	1119.333333	1350.666667	0.351302937	0.538371607	0.972790461
12870	'Cp'	57	45	465	198	58	205	189	153.6666667	-0.320010997	0.757123087	0.972790461
12873	'Cpa3'	25	34	1	0	192	41	20	77.66666667	1.9305819	0.216473531	0.843391934
12874	'Cpd'	2922	2945	924	6470	3734	4081	2263.666667	4761.666667	1.305180053	0.02796542	0.40918746
12876	'Cpe'	5058	5471	4441	6426	9836	11140	4990	9134	0.897757903	9.40E-05	0.013244807
12877	'Cpeb1'	2686	2589	1106	29	1292	2615	2127	1312	-0.812763271	0.428267907	0.962191208
12879	'Cys1'	10	7	9	5	6	6	8.666666667	5.666666667	-0.569746164	0.501498517	0.972790461
12889	'Cplx1'	5	10	6	1	4	1	7	2	-1.787775339	0.138519122	0.739741822
12890	'Cplx2'	713	689	379	262	770	600	593.6666667	544	-0.119514173	0.768463388	0.972790461

12891	'Cpne6'	4	2	25	0	1	0	10.33333333	0.333333333	-4.947264159	0.010536237	0.254206775
12892	'Cpox'	572	570	2958	1754	685	1444	1366.666667	1294.333333	-0.050427947	0.953327833	0.99904469
12894	'Cpt1a'	1872	1845	1086	2722	3181	3176	1601	3026.333333	1.016160898	0.00146345	0.082561154
12895	'Cpt1b'	47	40	126	99	73	79	71	83.66666667	0.274139059	0.696788187	0.972790461
12896	'Cpt2'	850	894	432	358	801	1023	725.3333333	727.3333333	0.011014693	0.978225608	0.999493374
12902	'Cr2'	1	3	1	0	1	4	1.666666667	1.666666667	-0.085620964	0.964304406	0.999493374
12903	'Crabp1'	34	53	29	5	56	125	38.66666667	62	0.560348805	0.528338574	0.972790461
12904	'Crabp2'	42	24	22	39	32	29	29.33333333	33.33333333	0.319516813	0.586729072	0.972790461
12905	'Cradd'	106.03	123.64	94.01	55.33	128.87	145.17	107.8933333	109.79	0.006946449	0.985473855	0.999493374
12908	'Crat'	888	885	508	290	755	796	760.3333333	613.6666667	-0.312586159	0.413186413	0.95722888
12909	'Crcp'	394	407	330	212	296	366	377	291.3333333	-0.339907842	0.200688507	0.828336309
12912	'Creb1'	1592	1635	1935	1446	1746	1543	1720.666667	1578.333333	-0.073276314	0.839006042	0.974723675
12913	'Creb3'	602	638	1440	1140	1021	1470	893.3333333	1210.333333	0.435670001	0.44012367	0.968139674
12914	'Crebbp'	2429	2399	2087	1536	2171	1857	2305	1854.666667	-0.257318184	0.343684183	0.925308103
12915	'Atf6b'	1049	976	379	991	1499	1811	801.3333333	1433.666667	0.909384806	0.029436994	0.418904292
12916	'Crem'	312	319	497	980	321	348	376	549.6666667	0.762349861	0.291236335	0.899151786
12919	'Crhbp'	5	2	1	5	4	6	2.666666667	5	1.02266422	0.394622962	0.953312802
12921	'Crhr1'	0	1	0	0	4	4	0.333333333	2.666666667	2.76122033	0.306518136	0.907713832
12922	'Crhr2'	3	2	0	21	4	1	1.666666667	8.666666667	2.825620093	0.088908246	0.645161668
12925	'Crip1'	20	22	24	68	35	75	22	59.33333333	1.528361546	0.011058616	0.259994952
12927	'Bcar1'	1195	1220	4255	2521	1143	1717	2223.333333	1793.666667	-0.2414924	0.751549784	0.972790461
12928	'Crk'	3639	3690	5385	5219	3955	5019	4238	4731	0.232401905	0.619681474	0.972790461
12929	'Crkl'	1825	1774	2010	1251	1726	1615	1869.666667	1530.666667	-0.255387754	0.412742986	0.95722888
12931	'Crlf1'	38	25	13	86	40	192	25.33333333	106	2.139495904	0.002411245	0.110099123
12933	'Crmp1'	140	134	113	4	276	84	129	121.3333333	-0.207875358	0.836932502	0.974723675
12934	'Dpysl2'	1000	1119	1404	576	1103.8	1166	1174.333333	948.6133333	-0.339638744	0.359946142	0.933706718
12936	'Pcdha4'	0	0	0	0	32.19	11.94	0	14.71	6.151542467	0.01026491	0.250824438
12939	'Pcdha7'	0	0	0	0	4.58	0	0	1.526666667	2.75434637	0.489126355	0.972790461
12941	'Pcdha5'	6.34	6.08	0	2.09	4.54	4.7	4.14	3.776666667	-0.167165407	0.907439094	0.988823459
12942	'Pcdha11'	0	0	0	0	2.34	0	0	0.78	1.801491674	0.656112935	0.972790461
12943	'Pcdha10'	6.53	12.52	4.8	0	9.4	7.33	7.95	5.576666667	-0.530212115	0.673567027	0.972790461
12944	'Crp'	3	1	1	0	3	3	1.666666667	2	0.186977952	0.917129938	0.99053993
12945	'Dmbt1'	2	0	5	1	0	3	2.333333333	1.333333333	-0.915187436	0.674934443	0.972790461
12946	'Cr1l'	559	620	610	289	863	851	596.3333333	667.6666667	0.110037633	0.785565286	0.972790461
12950	'Hapln1'	145	154	55	6	168	118	118	97.33333333	-0.353459005	0.694737811	0.972790461
12951	'Crx'	0	0	0	10	0	0	0	3.333333333	4.658927321	0.235477394	0.861636582
12952	'Cry1'	1375	1482	2584	1159	861	1169	1813.666667	1063	-0.718094224	0.168420175	0.784384366
12953	'Cry2'	523	498	675	1641	539	653	565.3333333	944.3333333	0.959313614	0.161425149	0.771435531
12954	'Cryaa'	5	1	8	0	1	0	4.666666667	0.333333333	-3.726123235	0.068530984	0.583886121
12955	'Cryab'	91	81	105	4066	125	895	92.33333333	1695.333333	4.551084218	2.21E-05	0.0044251

12959	'Cryba4'	1	1	0	0	2	3	0.666666667	1.666666667	1.273050587	0.596162899	0.972790461
12960	'Crybb1'	2	5	0	0	4	4	2.333333333	2.666666667	0.166690415	0.932496091	0.993954086
12962	'Crybb3'	9	9	1	17	6	5	6.333333333	9.333333333	0.879602614	0.448095898	0.970198456
12964	'Cryga'	1	2	0	1	1	0	1	0.666666667	-0.334057484	0.89800003	0.985794067
12965	'Crygb'	26	33	1	63	59	123.86	20	81.95333333	2.156792831	0.017751584	0.333162012
12966	'Crygc'	1	4	0	11	12	9.14	1.666666667	10.71333333	2.836701553	0.023090108	0.377626024
12967	'Crygd'	1.34	1.92	0	0	0	0	1.086666667	0	-1.703500596	0.673820112	0.972790461
12968	'Cryge'	26.66	27.08	15	51	10	16	22.91333333	25.66666667	0.491152741	0.584120231	0.972790461
12970	'Crygs'	4	2	0	0	1	3	2	1.333333333	-0.614962631	0.779756087	0.972790461
12971	'Crym'	2	3	4	6	5	1	3	4	0.557832898	0.670373758	0.972790461
12972	'Cryz'	573.59	558.01	854.85	112.61	552.77	685.53	662.15	450.3033333	-0.691642476	0.25249625	0.871252558
12974	'Cs'	4193	3908	4908	5342	3164	3709	4336.333333	4071.666667	0.045493319	0.928481285	0.993096978
12977	'Csf1'	294	287	1944	696	240	357	841.6666667	431	-0.894722202	0.348283608	0.928444937
12978	'Csf1r'	23	19	243	158	51	100	95	103	0.14312212	0.896124838	0.985642009
12981	'Csf2'	0	0	4	1	0	1	1.333333333	0.666666667	-1.047534352	0.7316437	0.972790461
12982	'Csf2ra'	433.42	431.95	257	260	568.77	296.78	374.1233333	375.1833333	0.059987063	0.883371717	0.983244267
12983	'Csf2rb'	4	11	45.64	39.01	34	70.04	20.21333333	47.68333333	1.159345924	0.226881095	0.854001521
12984	'Csf2rb2'	6	4	24.36	10.99	22	63.96	11.45333333	32.31666667	1.282965919	0.202591847	0.830701895
12985	'Csf3'	4	0	62	3	3	2	22	2.666666667	-3.138115299	0.060290563	0.557511761
12986	'Csf3r'	7	7	6	4	14	18	6.666666667	12	0.802001934	0.336668494	0.923078936
12988	'Csk'	1168	1112	965	887	1158	1352	1081.666667	1132.333333	0.109441308	0.658610298	0.972790461
12994	'Csn3'	1	0	104	5	2	3	35	3.333333333	-3.43151254	0.05943472	0.553094905
12995	'Csnk2a1'	3411	3480	5207	3662	3430	3684	4032.666667	3592	-0.112931248	0.799587512	0.972790461
13000	'Csnk2a2'	1883	1743	2113	1442	1715	2076	1913	1744.333333	-0.104289675	0.751220568	0.972790461
13001	'Csnk2b'	1178.4	1183.2	1614.9	996.89	945.97	1332.99	1325.496667	1091.95	-0.245759162	0.544962424	0.972790461
13002	'Dnajc5'	3316	3268	8969	1766	3038	3455	5184.333333	2753	-0.997512062	0.08982403	0.647605029
13003	'Vcan'	791	768	987	718	773	495	848.6666667	662	-0.270522278	0.558810106	0.972790461
13004	'Ncan'	18	14	5	2	3	5	12.33333333	3.333333333	-1.829160717	0.059025091	0.552865869
13006	'Smc3'	3643	3681	3835	3140	3106	3077	3719.666667	3107.666667	-0.177040269	0.622189101	0.972790461
13007	'Csrp1'	1429	1559	4244	2663	1577	1643	2410.666667	1961	-0.220176921	0.750704442	0.972790461
13008	'Csrp2'	208	168	584	291	164	146	320	200.3333333	-0.585029808	0.437317903	0.96622803
13009	'Csrp3'	0	2	0	2	0	0	0.666666667	0.666666667	0.545035566	0.890974668	0.985046501
13010	'Cst3'	2153	2399	1419	4728	2116	3306	1990.333333	3383.333333	0.967147233	0.072812798	0.596837647
13011	'Cst7'	1	0	2	0	4	0	1	1.333333333	0.219124763	0.941179226	0.996040262
13012	'Cst8'	165	224	27	38	273	180	138.6666667	163.6666667	0.240484611	0.767950038	0.972790461
13013	'Cst9'	6	5	0	2	20	5	3.666666667	9	1.319774109	0.350936876	0.92886351
13014	'Cstb'	412	446	1786	896	487	804	881.3333333	729	-0.257115719	0.740620402	0.972790461
13016	'Ctbp1'	2775	2774	2729	2032	3048	3487	2759.333333	2855.666667	0.065262325	0.801859851	0.972790461
13017	'Ctbp2'	657.78	669	1600	473	778.03	872	975.5933333	707.6766667	-0.53018406	0.337318017	0.92380462
13018	'Ctcf'	2767	2852	1425	1244	2909	2192	2348	2114.996667	-0.114734061	0.757376909	0.972790461

13019	'Ctf1'	83	114	35	67	115	129	77.33333333	103.6666667	0.484620543	0.319370151	0.916507972
13024	'Ctla2a'	79.67	98.75	35.18	13.89	131.03	154.64	71.2	99.85333333	0.421733622	0.585929268	0.972790461
13025	'Ctla2b'	14	11	4	2	29	29	9.666666667	20	0.986615755	0.320186319	0.917050664
13026	'Pcyt1a'	1646	1815	4259	1586	1472	1804	2573.333333	1620.666667	-0.664145405	0.251238906	0.871230154
13030	'Ctsb'	4127.8	4289.8	4504.4	14563	6396.9	10867.7	4307.323333	10609.35	1.45744142	0.006957759	0.197529663
13032	'Ctsc'	369	343	1103	940	492	740	605	724	0.313573448	0.660084564	0.972790461
13033	'Ctsd'	3344	3359	2910	11693	5246	11195	3204.333333	9378	1.692163543	9.08E-04	0.062759781
13034	'Ctse'	26	23	5	5	28	8	18	13.66666667	-0.359660296	0.699893104	0.972790461
13035	'Ctsg'	0	0	0	0	0	1	0	0.333333333	1.020273531	0.802557913	0.972790461
13036	'Ctsh'	556	588	606	4468	840	3003	583.3333333	2770.333333	2.452825042	6.05E-04	0.048169622
13038	'Ctsk'	47	43	28	129	91	63	39.33333333	94.33333333	1.455195228	0.013698244	0.292512191
13039	'Ctsl'	3356	3356	4465	18913	3874	9528	3725.666667	10771.66667	1.750626504	0.016745703	0.323948168
13040	'Ctss'	31	40	5	412	76	243	25.33333333	243.6666667	3.58175299	6.75E-05	0.01026066
13041	'Ctsw'	1	0	3	1	1	0	1.333333333	0.666666667	-0.961298317	0.701514401	0.972790461
13043	'Cttn'	3980	4159	4505	5390	4788	5215	4214.666667	5131	0.370084475	0.336634394	0.923078936
13046	'Celf1'	3993	3936	3078	3027	3322	3826	3669	3391.666667	-0.037832127	0.892233812	0.985171
13047	'Cux1'	2558	2572	2950	3150	2189	2351	2693.333333	2563.333333	0.051753365	0.911335488	0.989773583
13048	'Cux2'	127	91	18	27	149	125	78.66666667	100.3333333	0.351308862	0.64921814	0.972790461
13051	'Cx3cr1'	16	22	2	1	22	33	13.33333333	18.66666667	0.435924295	0.711529879	0.972790461
13052	'Cxadr'	625	623	1797	3453	1104	2382	1015	2313	1.288567751	0.090128899	0.648437606
13056	'Cyb561'	299	283	366	127	330	608	316	355	0.071267399	0.892100492	0.985171
13057	'Cyba'	510	590	809	330	716	914	636.3333333	653.3333333	-0.029103558	0.947583154	0.997146783
13058	'Cybb'	30	33	21	220	55	97	28	124	2.417903011	0.001287956	0.075575987
13063	'Cycs'	1016	1094	1046	1445	1000	1163	1052	1202.666667	0.320881198	0.466398714	0.972790461
13067	'Cyct'	32	28	36	0	23	40	32	21	-0.782484242	0.482581975	0.972790461
13070	'Cyp11a1'	25	36	437	1323	51	455	166	609.6666667	2.023301694	0.124047992	0.716621217
13074	'Cyp17a1'	35	49	1	1	83	24	28.33333333	36	0.327468511	0.811504993	0.972790461
13075	'Cyp19a1'	10	9	29	0	12	2	16	4.666666667	-1.96588228	0.136940825	0.73816812
13076	'Cyp1a1'	2	4.12	5	9	2	96	3.706666667	35.66666667	3.127821708	0.016755676	0.323948168
13077	'Cyp1a2'	4	0.88	0	0	1	1	1.626666667	0.666666667	-0.983419122	0.753934186	0.972790461
13078	'Cyp1b1'	1160	1172	1750	1940	1040	1823	1360.666667	1601	0.327915158	0.539764302	0.972790461
13079	'Cyp21a1'	0	2	0	4	0	1	0.666666667	1.666666667	1.755643056	0.542129974	0.972790461
13081	'Cyp24a1'	1	4	0	0	5	2	1.666666667	2.333333333	0.47112896	0.826396041	0.972844676
13082	'Cyp26a1'	0	1	8	0	1	2	3	1	-1.862054601	0.410072418	0.957157474
13088	'Cyp2b10'	0	1	0	0	0	0	0.333333333	0	-0.903279821	0.824807108	0.972790461
13090	'Cyp2b19'	0	1	0	0	0	0	0.333333333	0	-0.903279821	0.824807108	0.972790461
13095	'Cyp2c29'	0	0	0	0	3	2	0	1.666666667	3.04513816	0.441518888	0.968551476
13101	'Cyp2d10'	2	0	0	5	0	2	0.666666667	2.333333333	2.180859721	0.416653761	0.95722888
13105	'Cyp2d9'	0	0	0	29	0	4	0	11	6.31620187	0.013336327	0.288185595
13106	'Cyp2e1'	0	2	1	0	2	1	1	1	-0.092627511	0.970247576	0.999493374

13107	'Cyp2f2'	15	12	1	22	31	29	9.333333333	27.33333333	1.673821631	0.049211109	0.515210762
13110	'Cyp2j6'	184	157	79	63	256	235	140	184.6666667	0.377977004	0.475245304	0.972790461
13113	'Cyp3a13'	167.55	184.46	54.32	242.43	338.6	201.09	135.4433333	260.7066667	1.088577149	0.043125601	0.484697385
13115	'Cyp27b1'	100	110	122	54	88	118	110.6666667	86.66666667	-0.36763944	0.350820247	0.92886351
13116	'Cyp46a1'	253	196	43	65	149	134	164	116	-0.432249423	0.492797699	0.972790461
13117	'Cyp4a10'	0	1	0	3	1	2	0.333333333	2	2.650065168	0.25227192	0.871252558
13118	'Cyp4a12b'	0	0	0	0	0	1	0	0.333333333	1.020273531	0.802557913	0.972790461
13119	'Cyp4a14'	0	0	0	0	1	0	0	0.333333333	1.020273531	0.802557913	0.972790461
13120	'Cyp4b1'	2	0	1	10	5	7	1	7.333333333	3.023037555	0.035456066	0.452318994
13121	'Cyp51'	828	898	1084	1221	991	919	936.6666667	1043.666667	0.260400243	0.571596469	0.972790461
13122	'Cyp7a1'	0	0	0	0	0	1	0	0.333333333	1.020273531	0.802557913	0.972790461
13123	'Cyp7b1'	63	79	109	22	108	81	83.66666667	70.33333333	-0.353305031	0.561848588	0.972790461
13124	'Cyp8b1'	0	2	0	0	0	1	0.666666667	0.333333333	-0.733814424	0.856146772	0.975734242
13131	'Dab1'	398	355	306	76	427	317	353	273.3333333	-0.43817653	0.417099918	0.957382938
13132	'Dab2'	1088	1124	1698	851	1110	1074	1303.333333	1011.666667	-0.352671799	0.392885157	0.952648938
13134	'Dach1'	712	688	380	46	815	711	593.3333333	524	-0.275627924	0.72828423	0.972790461
13135	'Dad1'	879	841	1428	1277	832	1339	1049.333333	1149.333333	0.194075564	0.708566245	0.972790461
13136	'Cd55'	797.95	807.96	292.88	6669	1264	2236	632.93	3389.66	2.772882586	5.62E-04	0.046680135
13137	'Cd55b'	12.05	8.09	2.12	2	29.02	73	7.42	34.67333333	2.157620478	0.057252933	0.547807126
13138	'Dag1'	6316	6318	2289	10372	9155	11171	4974.333333	10232.66667	1.188733588	0.010775749	0.256406185
13139	'Dgka'	317	377	277	457	410	294	323.6666667	387	0.400541859	0.375515669	0.940406075
13142	'Dao'	5	3	0	0	1	3	2.666666667	1.333333333	-1.02339221	0.627620093	0.972790461
13143	'Dapk2'	128	165	10	286	139	180	101	201.6666667	1.277401403	0.149140258	0.757738857
13144	'Dapk3'	414	384	1269	660	573	801	689	678	-0.043331287	0.947662743	0.997175341
13162	'Slc6a3'	2	0	1	5	1	2	1	2.666666667	1.643227949	0.374205531	0.940062838
13163	'Daxx'	672	633	871	938	658	1112	725.3333333	902.6666667	0.379283705	0.40781289	0.957157474
13164	'Dazl'	4671	4557	2937	33	2535	3742	4055	2103.333333	-1.08323373	0.2999893	0.903723114
13166	'Dbh'	4	3	0	0	1	0	2.333333333	0.333333333	-2.54542561	0.360550828	0.933706718
13167	'Dbi'	567	693	399	1103	433	655	553	730.3333333	0.626041087	0.286539773	0.894199485
13168	'Dbil5'	20	19	12	0	11	14	17	8.333333333	-1.128922733	0.284916822	0.893711786
13169	'Dbnl'	1314	1356	1244	919	1345	1600	1304.666667	1288	0.00149298	0.995322142	0.999562152
13170	'Dbp'	259.59	240.42	292.47	77.54	312.41	294.64	264.16	228.1966667	-0.295957669	0.554612765	0.972790461
13171	'Dbt'	623	648	276	760	798	1036	515.6666667	864.6666667	0.851968278	0.03276798	0.436246529
13175	'Dclk1'	721	630	745	1099	2305	1176	698.6666667	1526.666667	1.146392623	0.005762753	0.178438859
13176	'Dcc'	16	18	10	1	24	4	14.66666667	9.666666667	-0.655665334	0.548558655	0.972790461
13177	'Eci1'	377	400	442	279	564	715	406.3333333	519.3333333	0.316268997	0.38886848	0.950889716
13178	'Dck'	521	525	1021	304	371	307	689	327.3333333	-1.059332346	0.038532978	0.460411379
13179	'Dcn'	1328	1160	775	1589	1509	1132	1087.666667	1410	0.522031965	0.221947664	0.849517424
13180	'Pcbd1'	105	150	159	32	147	134	138	104.3333333	-0.496001541	0.376939463	0.941504733
13184	'Dcpp1'	1	0	0	0	2	0	0.333333333	0.666666667	0.839717162	0.835573887	0.974723675

13185	'Vps26c'	669	749	1183	1087	770	1014	867	957	0.212666588	0.679329915	0.972790461
13190	'Dct'	17	13	1	0	11	8	10.33333333	6.333333333	-0.739329034	0.603238412	0.972790461
13191	'Dctn1'	2900	2752	2429	1560	3139	3201	2693.666667	2633.333333	-0.034166023	0.900513373	0.986605212
13193	'Dcx'	11	8	4	29	6	7	7.666666667	14	1.202464978	0.25885849	0.877570902
13194	'Ddb1'	12212	12032	10971	11396	10309	11573	11738.33333	11092.66667	0.014406229	0.966990596	0.999493374
13195	'Ddc'	18	25	42	14	19	32	28.33333333	21.66666667	-0.435389473	0.512659891	0.972790461
13196	'Asap1'	2513	2411	3792	1461	2125	2465	2905.333333	2017	-0.54400793	0.177363677	0.79704932
13197	'Gadd45a'	213	199	3927	1716	215	446	1446.333333	792.3333333	-0.72844665	0.566390711	0.972790461
13198	'Ddit3'	365	300	17443	460	248	1291	6036	666.3333333	-3.38929527	0.011351308	0.264311324
13199	'Ddn'	4	2	22	193	0	4	9.333333333	65.66666667	3.139668651	0.083462745	0.627571099
13200	'Ddost'	3077	2808	2739	2004	3310	5243	2874.666667	3519	0.272422709	0.42469448	0.960285372
13202	'Ddt'	134	141	208	242	161	265	161	222.6666667	0.532479867	0.297156492	0.900647119
13204	'Dhx15'	3474	3499	4573	2994	3426	3723	3848.666667	3381	-0.15228614	0.679929827	0.972790461
13205	'Ddx3x'	9908	9765	14032	14908	10194	12653	11235	12585	0.258300189	0.596257386	0.972790461
13206	'Ddx4'	3024	3011	755	83	1589	2189	2263.333333	1287	-0.889006848	0.324666677	0.91982931
13207	'Ddx5'	19908	20832	13869	11523	19927	16723.77	18202.7	16057.86	-0.133228856	0.612978963	0.972790461
13209	'Ddx6'	6887	7030	7004	4592	6741	7849	6973.666667	6394	-0.109894723	0.679103355	0.972790461
13211	'Dhx9'	5838	6332	3206	2977	5699	6809	5125.333333	5161.666667	0.035293822	0.918201162	0.990727847
13244	'Degs1'	750	790	848	1222	1318	1893	796	1477.666667	0.92441253	0.005758666	0.178438859
13340	'Slc29a2'	159	136	313	76	168	188	202.6666667	144	-0.581422539	0.294357052	0.90029104
13345	'Twist2'	33	27	76	286	28	45	45.33333333	119.6666667	1.692924769	0.109889427	0.689773582
13346	'Des'	195	188	222	294	175	102	201.6666667	190.3333333	0.108862767	0.863962646	0.977632919
13347	'Dffa'	298	279	189	280	299	332	255.3333333	303.6666667	0.341699283	0.299232044	0.903198439
13349	'Ackr1'	0	0.56	4	7.35	2.09	1.8	1.52	3.746666667	1.44400486	0.497541826	0.972790461
13350	'Dgat1'	744	795	252	211	646	446	597	434.3333333	-0.423718883	0.412612265	0.95722888
13353	'Dgcr6'	460	482	307	153	501	550	416.3333333	401.3333333	-0.08717822	0.841837168	0.975182082
13356	'Dgcr2'	2765	2748	950	1252	3605	3351	2154.333333	2736	0.365836543	0.455764222	0.971239019
13358	'Slc25a1'	932	931	1809	878	954	1522	1224	1118	-0.156625578	0.75493082	0.972790461
13360	'Dhcr7'	492.18	554	286	800	574	520	444.06	631.3333333	0.69260374	0.156267839	0.767792163
13361	'Dhfr'	559.48	643.44	316	347	425.63	426.39	506.3066667	399.6733333	-0.245625938	0.480806163	0.972790461
13363	'Dhh'	7	7	5	53	16	26	6.333333333	31.66666667	2.55742341	0.003000684	0.122531121
13367	'Diaph1'	1046	1071	911	1449	1322	1564	1009.333333	1445	0.610883404	0.071971918	0.593728759
13368	'Dffb'	249	233	114	26	249	173	198.6666667	149.3333333	-0.471430153	0.493374844	0.972790461
13370	'Dio1'	1	5	4	8	1	1	3.333333333	3.333333333	0.305137569	0.842649539	0.975182082
13371	'Dio2'	20	37	5	98	30	14	20.66666667	47.33333333	1.588233099	0.12384203	0.71638709
13380	'Dkk1'	3	5	0	0	1	0	2.666666667	0.333333333	-2.734058325	0.314577949	0.914348871
13382	'Dld'	3776	4052	2995	2437	3786	4559	3607.666667	3594	0.020126174	0.933325528	0.994078204
13383	'Dlg1'	2236	2154.7	6237	2745	2063	2441	3542.56	2416.333333	-0.524540579	0.416180574	0.95722888
13384	'Mpp3'	154	157	68	64	239	109	126.3333333	137.3333333	0.142821282	0.795286384	0.972790461
13385	'Dlg4'	689	670	1872	1129	632	696	1077	819	-0.311368534	0.653201694	0.972790461

13386	'Dlk1'	3261	2696	1083	845	4078	4719	2346.666667	3214	0.418602985	0.486875974	0.972790461
13388	'Dll1'	127	131	20	22	110	55	92.66666667	62.33333333	-0.538554322	0.487556422	0.972790461
13389	'Dll3'	18	19	17	4	7	14	18	8.333333333	-1.129335679	0.115297488	0.702526958
13390	'Dlx1'	7	5	0	0	9	7	4	5.333333333	0.381455246	0.82914053	0.973678583
13392	'Dlx2'	5	3	3	2	0	0	3.666666667	0.666666667	-2.139718632	0.274212115	0.885423597
13393	'Dlx3'	4	4	46	1	3	0	18	1.333333333	-3.876658202	0.014346146	0.298412386
13394	'Dlx4'	1	4	4	9	1	0	3	3.333333333	0.510799375	0.776012905	0.972790461
13395	'Dlx5'	1	1	27	1	2	1	9.666666667	1.333333333	-3.010932677	0.076817401	0.609792246
13396	'Dlx6'	1	1	5	0	3	1	2.333333333	1.333333333	-0.997091461	0.600539707	0.972790461
13400	'Dmpk'	337	411	260	401	292	254	336	315.6666667	0.079507498	0.868327023	0.979229375
13401	'Dmwd'	960	995	1716	1461	1217	1311	1223.666667	1329.666667	0.179343848	0.724202395	0.972790461
13404	'Dmc1'	1016	1023	335	68	366	188	791.3333333	207.3333333	-1.930870351	0.001766497	0.09046942
13405	'Dmd'	870	1004	591	1374	889	958	821.6666667	1073.666667	0.563736075	0.235672451	0.861636582
13406	'Dmp1'	4	4	2	2	10	6	3.333333333	6	0.838231049	0.450724161	0.970590732
13409	'Tmc1'	38	31	1	30	42	13	23.33333333	28.33333333	0.492051142	0.646080283	0.972790461
13411	'Dnah11'	78.82	90.55	2.25	3.21	22.3	36.95	57.20666667	20.82	-1.488413391	0.200140905	0.82759347
13417	'Dnah8'	1989	1827	175	76	183	103	1330.333333	120.6666667	-3.35497599	1.46E-05	0.003424174
13418	'Dnajc1'	860.48	927.71	653.78	887.77	918.43	995	813.99	933.7333333	0.291964673	0.352130023	0.929748874
13419	'Dnase1'	5	1	1	4	3	4	2.333333333	3.666666667	0.780501503	0.565118313	0.972790461
13421	'Dnase1l3'	0	1	1	0	1	2	0.666666667	1	0.429670739	0.869759782	0.979794316
13423	'Dnase2a'	65	69	39	137	97	154	57.66666667	129.3333333	1.288917756	0.005687627	0.178006489
13424	'Dync1h1'	13808	12641	5709	8992	12751	10781	10719.33333	10841.33333	0.116975811	0.763065054	0.972790461
13426	'Dync1i1'	164	154	455	141	672	754	257.6666667	522.3333333	0.839684272	0.242965003	0.864948315
13427	'Dync1i2'	2564	2560	3049	2322	2663	2721	2724.333333	2568.666667	-0.037463478	0.914114029	0.989964366
13429	'Dnm1'	562	557	421	655	555	419	513.3333333	543	0.232950874	0.604779331	0.972790461
13430	'Dnm2'	1745.1	1774.3	1800.8	2699.6	1821	2076.25	1773.403333	2198.95	0.441889224	0.328319431	0.921397778
13433	'Dnmt1'	2990	2903	926	2983	2528	2952	2273	2821	0.476287324	0.349757702	0.92886351
13434	'Trdmt1'	186	207	94	92	151	125	162.3333333	122.6666667	-0.329499063	0.400410878	0.955860942
13435	'Dnmt3a'	6549	6428	4123	2581	6603	5960	5700	5048	-0.174142819	0.610982627	0.972790461
13436	'Dnmt3b'	262	310	120	176	285	246	230.6666667	235.6666667	0.112464801	0.783186811	0.972790461
13437	'Dnpep'	1250	1363	1296	1025	1405	1509	1303	1313	0.043564589	0.871995271	0.980171118
13445	'Cdk2ap1'	671	700	762	940	1150	1142	711	1077.333333	0.645604147	0.046891014	0.50423753
13446	'Doc2a'	77.79	61.83	12.8	2.88	30.53	78.87	50.80666667	37.42666667	-0.518549599	0.62161662	0.972790461
13447	'Doc2b'	2693	2705	2301	1084	3544	3655	2566.333333	2761	0.054885622	0.894074292	0.985171
13448	'Dok1'	125	126	248	105	127	181	166.3333333	137.6666667	-0.302620699	0.561332843	0.972790461
13449	'Dok2'	228	217	45	7	334	69	163.3333333	136.6666667	-0.29689898	0.779906249	0.972790461
13476	'Reep5'	1077	1131	1013	1658	2046	2687	1073.666667	2130.333333	1.02388113	1.59E-04	0.01938235
13478	'Dpagt1'	622.51	630.71	1065.6	272.46	700.89	850.75	772.94	608.0333333	-0.439987254	0.377892644	0.941757196
13479	'Dpep1'	17	15	2	35	34	20	11.33333333	29.66666667	1.592254747	0.061290855	0.562479029
13480	'Dpm1'	708	787	460	510	617	957	651.6666667	694.6666667	0.142454809	0.661921038	0.972790461

13481	'Dpm2'	256	307	271	202	301	335	278	279.3333333	0.028742978	0.921515618	0.991066749
13482	'Dpp4'	82	61	6	25	92	58	49.66666667	58.33333333	0.288448227	0.735987709	0.972790461
13483	'Dpp6'	255	272	145	27	373	542	224	314	0.381134646	0.636180599	0.972790461
13486	'Dr1'	1916	1883	2136	1143	1568	2063	1978.333333	1591.333333	-0.308881625	0.324744073	0.91982931
13487	'Slc26a3'	0	1	0	1	1	0	0.333333333	0.666666667	1.081692101	0.760081965	0.972790461
13488	'Drd1'	2	1	2	1	5	1	1.666666667	2.333333333	0.45848058	0.773236974	0.972790461
13489	'Drd2'	12	16	4	0	7	0	10.66666667	2.333333333	-2.206282594	0.173697415	0.79261444
13490	'Drd3'	0	0	0	0	1	0	0	0.333333333	1.020273531	0.802557913	0.972790461
13491	'Drd4'	1	0	0	0	2	9	0.333333333	3.666666667	3.193247385	0.225007142	0.851475917
13494	'Drg1'	1723	1777	2656	1209	1634	2489	2052	1777.333333	-0.237497638	0.572607369	0.972790461
13495	'Drg2'	646	673	1353	427	786	889	890.6666667	700.6666667	-0.414348274	0.414133967	0.95722888
13496	'Arid3a'	462	453	1008	574	425	433	641	477.3333333	-0.36147846	0.545088107	0.972790461
13497	'Drp2'	1054	1165	160	27	1430	742	793	733	-0.161073737	0.878325835	0.981341203
13498	'Atn1'	2352	2138	2590	1248	2461	1935	2360	1881.333333	-0.33057095	0.327959323	0.92113012
13505	'Dsc1'	0	1	2	0	2	0	1	0.666666667	-0.733221529	0.816021492	0.972790461
13506	'Dsc2'	570	606	199	140	591	318	458.3333333	349.6666667	-0.373912351	0.514250968	0.972790461
13507	'Dsc3'	5	7	12	1	8	2	8	3.666666667	-1.213274248	0.297527064	0.900750988
13508	'Dscam'	54	35	9	0	19	46	32.66666667	21.66666667	-0.679854683	0.58358945	0.972790461
13510	'Dsg1a'	12	10	0	1	5.16	2	7.333333333	2.72	-1.38457515	0.366580592	0.936625482
13511	'Dsg2'	314	325	388	315	477	453	342.3333333	415	0.291430252	0.390409398	0.95208613
13516	'Epyc'	1	1	0	0	0	2	0.666666667	0.666666667	-0.01112152	0.997410093	0.999723781
13518	'Dst'	5733.7	6745.5	4360.6	1691.5	4412.1	2971.42	5613.263333	3025.003333	-0.880667099	0.013051994	0.285349599
13521	'Slc26a2'	191	166	50	301	194	220	135.6666667	238.3333333	1.024580664	0.091180336	0.649965349
13522	'Adam28'	0	0	0	9	2	0	0	3.666666667	4.698996723	0.120943175	0.712100232
13524	'Adam18'	1	0	0	0	0	0	0.333333333	0	-0.903279821	0.824807108	0.972790461
13527	'Dtna'	73	85	550	98	148	168	236	138	-0.904601756	0.301096334	0.904375823
13528	'Dtnb'	1615	1548	943	1296	1344	1416	1368.666667	1352	0.091277794	0.788284187	0.972790461
13531	'Usp17la'	2	5.19	2	1	0	2	3.063333333	1	-1.507439177	0.385655195	0.947213579
13532	'Usp17lc'	0	1.67	0	0	0	1.97	0.556666667	0.656666667	0.058500858	0.988561293	0.999493374
13537	'Dusp2'	60	57	36	85	71	35	51	63.66666667	0.508274635	0.408925579	0.957157474
13542	'Dvl1'	1297	1271	1133	3561	1243	1265	1233.666667	2023	0.970198504	0.144870902	0.752280685
13543	'Dvl2'	989	1007	1209	673	1015	989	1068.333333	892.3333333	-0.247918845	0.449539488	0.97049895
13544	'Dvl3'	1481	1443	1197	1196	1664	1815	1373.666667	1558.333333	0.224985815	0.333971139	0.921648675
13548	'Dyrk1a'	2391	2447	2195	1651	1801	1776	2344.333333	1742.666667	-0.349222727	0.263497706	0.88013169
13549	'Dyrk1b'	3254	3139	1104	774	2824	1529	2499	1709	-0.520642643	0.334446864	0.921767653
13555	'E2f1'	472	461	202	161	327	438	378.3333333	308.6666667	-0.272513343	0.526495109	0.972790461
13557	'E2f3'	774.01	842.43	766.87	529.9	725.94	807.46	794.4366667	687.7666667	-0.17584793	0.50543569	0.972790461
13559	'E2f5'	769	759	687	261	620	1166	738.3333333	682.3333333	-0.179241696	0.704276674	0.972790461
13560	'E4f1'	455	424	712	342	492	429	530.3333333	421	-0.330210551	0.450111226	0.970590732
13586	'Ear1'	0	2	1	0	2	1	1	1	-0.092627511	0.970247576	0.999493374

13587	'Ear2'	0	0	0	0	2	2	0	1.333333333	2.719229454	0.494880768	0.972790461
13589	'Mapre1'	3941	4210	5158	2633	3859	4292	4436.333333	3594.666667	-0.302190806	0.36910867	0.937712122
13590	'Lefty1'	3	2	26	17	3	6	10.33333333	8.666666667	-0.143424672	0.916094885	0.990491125
13591	'Ebf1'	69	97	18	7	97	76	61.33333333	60	-0.072095186	0.934877324	0.994413066
13592	'Ebf2'	19	26	2	15	13	5	15.66666667	11	-0.258743847	0.804981338	0.972790461
13593	'Ebf3'	157	179	30	36	114	78	122	76	-0.628257937	0.356301058	0.931977458
13595	'Ebp'	92	110	161	105	175	181	121	153.6666667	0.320121401	0.475650797	0.972790461
13599	'Ecel1'	31	37	385	32	19	14	151	21.66666667	-2.786311902	0.010653124	0.255084633
13601	'Ecm1'	148	148	97	290	146	177	131	204.3333333	0.842361192	0.127691939	0.723917691
13602	'Sparcl1'	3422	3339	876	747	2932	2387	2545.666667	2022	-0.319575398	0.590926089	0.972790461
13603	'Opn3'	145	118	121	55	120	108	128	94.33333333	-0.442680622	0.222544874	0.849773677
13605	'Ect2'	1325	1288	351	333	897	1489	988	906.3333333	-0.124629064	0.835763511	0.974723675
13607	'Eda'	367	323	115	230	250	267	268.3333333	249	0.021840462	0.963754154	0.999493374
13608	'Edar'	80	66	11	10	47	59	52.33333333	38.66666667	-0.438999872	0.593158063	0.972790461
13609	'S1pr1'	826	866	146	173	821	682	612.6666667	558.6666667	-0.122500703	0.861792903	0.976863639
13610	'S1pr3'	213	178	255	698	319	342	215.3333333	453	1.252286377	0.042298117	0.479588013
13611	'S1pr4'	0	1	0	2	4	2	0.333333333	2.666666667	2.936398029	0.170423496	0.785667192
13612	'Edil3'	125	115	75	14	201	132	105	115.6666667	0.051177928	0.946250986	0.996592403
13614	'Edn1'	6	9	24	62	6	8	13	25.33333333	1.245642224	0.304588168	0.907164095
13615	'Edn2'	18	34	72	7	40	44	41.33333333	30.33333333	-0.618707635	0.460981176	0.972790461
13616	'Edn3'	143	155	13	10	213	185	103.6666667	136	0.354666504	0.73028945	0.972790461
13617	'Ednra'	677	625	405	224	844	1127	569	731.6666667	0.307843212	0.544533121	0.972790461
13618	'Ednrb'	139	155	20	200	124	116	104.6666667	146.6666667	0.744118189	0.332223235	0.921648675
13619	'Phc1'	2330	2249	1075	726	1592	1933	1884.666667	1417	-0.396186441	0.320947212	0.917442833
13626	'Eed'	671	687	990	523	579	612	782.6666667	571.3333333	-0.421021152	0.308034445	0.907963267
13627	'Eef1a1'	136082	132406	256961	244669	122074	143911.5	175149.6167	170218.19	0.084772688	0.892504428	0.985171
13628	'Eef1a2'	1	0	1	1	0	1	0.666666667	0.666666667	0.092317552	0.973134126	0.999493374
13629	'Eef2'	48135	44242	100235	101788	39541	60707	64204	67345.33333	0.198243606	0.772083852	0.972790461
13631	'Eef2k'	663	642	1153	108	705	745	819.3333333	519.3333333	-0.813670291	0.221447455	0.849403976
13636	'Efna1'	652	658	786	650	770	1037	698.6666667	819	0.247192197	0.472591181	0.972790461
13637	'Efna2'	304	266	192	106	324	296	254	242	-0.088737892	0.827136478	0.973067256
13638	'Efna3'	114	100	172	444	99	112	128.6666667	218.3333333	1.024636623	0.21905939	0.845979511
13639	'Efna4'	180	182	121	233	216	239	161	229.3333333	0.621407879	0.096334589	0.662981155
13640	'Efna5'	170	164	384	206	185	187	239.3333333	192.6666667	-0.282333702	0.631877034	0.972790461
13641	'Efnb1'	1619	1682	555	709	2205	1931	1285.333333	1615	0.347964255	0.492710157	0.972790461
13642	'Efnb2'	1367	1354	2562	1125	1780	1338	1761	1414.333333	-0.328208594	0.498827792	0.972790461
13643	'Efnb3'	438	413	104	75	503	273	318.3333333	283.6666667	-0.171257297	0.807478288	0.972790461
13644	'Efs'	621.95	680.18	772.51	106.19	1068.5	861.41	691.5466667	678.71	-0.158635158	0.816686007	0.972790461
13645	'Egf'	101	90	37	19	55	33	76	35.66666667	-1.048349985	0.059182575	0.552865869
13649	'Egfr'	722	700	794	2072	1015	598	738.6666667	1228.333333	0.960528628	0.151415987	0.75995796

13650	'Rhbdf1'	529	504	603	701	669	627	545.3333333	665.6666667	0.371098806	0.359359248	0.933706718
13653	'Egr1'	827	805	6000	4257	1304	1693	2544	2418	0.024004984	0.980752099	0.999493374
13654	'Egr2'	48.94	55.65	306.67	167.38	41.86	75.58	137.0866667	94.94	-0.42360533	0.669883575	0.972790461
13655	'Egr3'	22	19	107	126	22	19	49.33333333	55.66666667	0.389940456	0.728767157	0.972790461
13656	'Egr4'	11	3	9	0	2	0	7.666666667	0.666666667	-3.566904417	0.03770085	0.459930654
13660	'Ehd1'	924	933	1587	1243	905	1219	1148	1122.333333	0.028721028	0.955215334	0.999493374
13661	'Ehf'	23	28	233	1012	37	227	94.66666667	425.3333333	2.373952187	0.063157184	0.566852792
13663	'Ei24'	1603	1674	904	1057	1659	1705	1393.666667	1473.666667	0.138606028	0.643723447	0.972790461
13664	'Eif1a'	1724	1701	6749	2032	1535	2488	3391.333333	2018.333333	-0.778668663	0.283418604	0.891816369
13665	'Eif2s1'	1959	2027	2954	1271	1881	2318	2313.333333	1823.333333	-0.36481944	0.354482203	0.931809687
13666	'Eif2ak3'	361	367	464	945	453	739	397.3333333	712.3333333	0.974763677	0.073687653	0.599721598
13667	'Eif2b4'	599	606	444	407	549	704	549.6666667	553.3333333	0.05037995	0.847731958	0.975439569
13669	'Eif3a'	8387	8241	15213	8062	8978	11256	10613.66667	9432	-0.172180762	0.713970187	0.972790461
13680	'Ddx19a'	1206.6	1201.7	2017.3	1246.3	1176.7	1727.14	1475.183333	1383.373333	-0.078022201	0.86633112	0.978294615
13681	'Eif4a1'	9377	9738	22691	4731	9973	13229	13935.33333	9311	-0.689991076	0.225612234	0.852751449
13682	'Eif4a2'	16848	17468	20052	13412	14844	16622	18122.66667	14959.33333	-0.230491821	0.497193677	0.972790461
13684	'Eif4e'	3202	3283	3442	2351	2645	3332	3309	2776	-0.212631184	0.491879387	0.972790461
13685	'Eif4ebp1'	572	532	5214	919	597	1092	2106	869.3333333	-1.347141795	0.160130699	0.771114735
13688	'Eif4ebp2'	775	708	673	827	725	807	718.6666667	786.3333333	0.230184512	0.531696977	0.972790461
13690	'Eif4g2'	19229	19531	22186	9758	19459	20021	20315.33333	16412.66667	-0.330899227	0.30920549	0.910002286
13706	'Cela2a'	0	1	0	0	0	1	0.333333333	0.333333333	0.058500858	0.988561293	0.999493374
13709	'Elf1'	688	747	959	2830	1142.6	1497.42	798	1823.34	1.371478041	0.026171194	0.397137149
13710	'Elf3'	59	56	1211	288	28	83	442	133	-1.597645827	0.223104146	0.850372098
13711	'Elf5'	0	0	1	3	1	1	0.333333333	1.666666667	2.443463398	0.313172719	0.912356687
13712	'Elk1'	713	699	713	450	736	648	708.3333333	611.3333333	-0.189394546	0.509069865	0.972790461
13713	'Elk3'	674	694	534	2308	841	1013	634	1387.333333	1.374266899	0.028812212	0.413729443
13714	'Elk4'	364.69	366.76	140.7	288.26	261.79	298.64	290.7166667	282.8966667	0.106237742	0.81984544	0.972790461
13716	'Ell'	884	865	1259	1071	745	903	1002.666667	906.3333333	-0.05274527	0.914520993	0.989964366
13717	'Eln'	4003	3898	244	129	4128	4077	2715	2778	-0.010457427	0.992365341	0.999493374
13722	'Aimp1'	964	913	1648	732	829	1065	1175	875.3333333	-0.427823188	0.352637574	0.930050349
13723	'Emb'	44	47	41	535	70	121	44	242	2.800778914	0.001817098	0.091879425
13726	'Emd'	908.11	896.03	1636	316.02	687.04	989.05	1146.703333	664.0366667	-0.882114679	0.084868207	0.633597527
13728	'Mark2'	1489	1542	1371	1394	1553	1790	1467.333333	1579	0.168049065	0.556818543	0.972790461
13730	'Emp1'	2277	2230	3271	1492	2762	3316	2592.666667	2523.333333	-0.084317427	0.833320047	0.974723675
13731	'Emp2'	566	530	659	1152	802	1408	585	1120.666667	1.007185401	0.022300744	0.370441983
13732	'Emp3'	458	468	647	292	695	779	524.3333333	588.6666667	0.103675862	0.808088276	0.972790461
13733	'Adgre1'	169	176	12	39	79	115	119	77.66666667	-0.548117292	0.515261136	0.972790461
13796	'Emx1'	4	3	1	0	1	0	2.666666667	0.333333333	-2.782071021	0.208703392	0.836616817
13797	'Emx2'	1341	1437	459	527	1944	1918	1079	1463	0.437061247	0.425146096	0.960285372
13798	'En1'	0	0	9	2	1	0	3	1	-1.526543217	0.576872992	0.972790461

13799	'En2'	83	74	10	5	20	30	55.66666667	18.33333333	-1.593032553	0.066804562	0.578101196
13800	'Enah'	3242	3269	3420	4570	3040	3511	3310.333333	3707	0.29381015	0.519698721	0.972790461
13801	'Enam'	0	3	0	0	2	0	1	0.666666667	-0.532516362	0.893197773	0.985171
13803	'Enc1'	378	341	333	2210	615	1213	350.6666667	1346	2.164635724	9.36E-04	0.064031074
13804	'Endog'	51.74	44.16	143.35	21.73	52.93	90.82	79.75	55.16	-0.699766055	0.344730622	0.926100052
13805	'Eng'	309	224	64	1130	424	330	199	628	1.992717306	0.011901921	0.269701225
13806	'Eno1'	15998	13566	80576	27204	9725.8	26628	36713.43	21185.93667	-0.786251158	0.37106554	0.938513534
13807	'Eno2'	853	451	3768	846	92	162	1690.666667	366.6666667	-1.988210294	0.074964425	0.603900623
13808	'Eno3'	670.46	767.8	286.2	861.46	642.89	672.04	574.82	725.4633333	0.519788028	0.301852565	0.904841461
13809	'Enpep'	104	123	129	967	162	119	118.6666667	416	2.158747623	0.02057361	0.356011446
13813	'Eomes'	4	2	20	40	1	43	8.666666667	28	1.719065323	0.209501732	0.837080112
13819	'Epas1'	236	225	93	659	393	466	184.6666667	506	1.659661939	0.002426657	0.110372261
13821	'Epb41l1'	2089	2215	2271	3174	2015	2212	2191.666667	2467	0.313266747	0.513403168	0.972790461
13822	'Epb41l2'	5466	5532	4906	3837	5696	6797	5301.333333	5443.333333	0.059047775	0.806676763	0.972790461
13823	'Epb41l3'	959	943	1222	491	1048	1508	1041.333333	1015.666667	-0.100031725	0.813129099	0.972790461
13824	'Epb41l4a'	187	184	158	319	169	136	176.3333333	208	0.44922466	0.450230399	0.970590732
13828	'Epb42'	13	21	3	2	55	13	12.33333333	23.33333333	0.894223115	0.437780783	0.96622803
13829	'Dmtn'	47	53	40	56	113	86	46.66666667	85	0.890874795	0.023080956	0.377626024
13830	'Stom'	515	676	189	2353	1623	1800	460	1925.333333	2.267726293	4.99E-05	0.008322658
13831	'Epc1'	1072	1100	1346	248	1192	818	1172.666667	752.6666667	-0.728113829	0.164319035	0.777509578
13835	'Epha1'	198	148	14	182	164	133	120	159.6666667	0.630592769	0.44222614	0.969177389
13836	'Epha2'	245	231	599	4280	292	982	358.3333333	1851.333333	2.646437549	0.009734107	0.242548952
13837	'Epha3'	106	119	12	12	172	105	79	96.33333333	0.266120801	0.780821406	0.972790461
13838	'Epha4'	328	320	137	163	342	288	261.6666667	264.3333333	0.065661005	0.873614681	0.980171118
13839	'Epha5'	68	30	26	3	44	21	41.33333333	22.66666667	-0.929039181	0.281782737	0.889761402
13840	'Epha6'	23	18	1	6	13	4	14	7.666666667	-0.72466887	0.523176156	0.972790461
13841	'Epha7'	691	615	176	237	1194	693	494	708	0.52654187	0.406479059	0.957157474
13842	'Epha8'	15	10	2	3	7	11	9	7	-0.326751098	0.746859291	0.972790461
13844	'Ephb2'	804	691	196	503	1258	1199	563.6666667	986.6666667	0.851781214	0.11507387	0.702296207
13845	'Ephb3'	130	120	53	160	174	151	101	161.6666667	0.811292156	0.076476313	0.609792246
13846	'Ephb4'	1947	1856	810	2027	3063	2651	1537.666667	2580.333333	0.837460291	0.029588461	0.419705596
13848	'Ephb6'	730	716	273	233	942	1131	573	768.6666667	0.397647303	0.482412367	0.972790461
13849	'Ephx1'	101	98	185	1092	122	870	128	694.6666667	2.588151507	0.002506794	0.112403703
13850	'Ephx2'	387	410	695	132	652	623	497.3333333	469	-0.218059347	0.717831939	0.972790461
13852	'Stx2'	595	590	287	623	561	580	490.6666667	588	0.404814948	0.337383393	0.92380462
13853	'Epm2a'	44	55	92	2	42	43	63.66666667	29	-1.316217332	0.142727264	0.749054337
13854	'Epn1'	1483	1394	1550	1814	1736	2099	1475.666667	1883	0.418575125	0.234552908	0.861206181
13855	'Epn2'	1457	1358	663	1090	1200	1262	1159.333333	1184	0.144390338	0.703477632	0.972790461
13856	'Epo'	1	1	2	0	0	1	1.333333333	0.333333333	-1.889208538	0.461766352	0.972790461
13857	'Epor'	46	34	112	20	39	44	64	34.33333333	-0.991598634	0.150392591	0.759693458

13858	'Eps15'	3445	3527	2979	2362	2514	2836	3317	2570.666667	-0.292640953	0.318124811	0.916136994
13859	'Eps15l1'	1561	1528	1117	768	1662	1241	1402	1223.666667	-0.172684924	0.569298732	0.972790461
13860	'Eps8'	260	317	933	2128	807	1132	503.3333333	1355.666667	1.540477611	0.049359679	0.515210762
13861	'Epx'	7	7	1	0	8	4	5	4	-0.354086759	0.811348318	0.972790461
13864	'Nr2f6'	835	909	615	856	1130	1317	786.3333333	1101	0.540588493	0.033807706	0.443127738
13865	'Nr2f1'	105	119	53	25	104	73	92.33333333	67.33333333	-0.461895148	0.395824925	0.954181778
13866	'Erbb2'	844	879	216	415	1312	900	646.3333333	875.6666667	0.480636406	0.40234999	0.955996639
13867	'Erbb3'	117	114	68	291	108	120	99.66666667	173	1.059195412	0.112928841	0.698666814
13869	'Erbb4'	18	21	18	90	42	54	19	62	1.886650381	0.003840455	0.141968654
13870	'Ercc1'	263	280	877	116	263	223	473.3333333	200.6666667	-1.345739612	0.04181495	0.477375478
13871	'Ercc2'	656.16	666.57	493.19	416.28	706.64	624.66	605.3066667	582.5266667	-0.016021027	0.950323365	0.998065566
13872	'Ercc3'	928	979	447	739	973	1195	784.6666667	969	0.380193353	0.285813715	0.893820325
13874	'Ereg'	45	23	352	1892	116	403	140	803.6666667	2.717396461	0.030662789	0.425829766
13875	'Erf'	962	1027	1568	1278	1052	1226	1185.666667	1185.333333	0.062581443	0.895643056	0.985505807
13876	'Erg'	186	178	32	88	253	231	132	190.6666667	0.57608383	0.379289569	0.942621344
13877	'Erh'	1957	2119	2361	1055	1708	2023.99	2145.663333	1595.663333	-0.4365541	0.170389963	0.785667192
13884	'Ces1c'	0	1	1	1	11	8	0.666666667	6.666666667	3.208620967	0.058982322	0.552865869
13885	'Esd'	1562	1625	5759	6052	1785	3015	2982	3617.333333	0.401459177	0.630872224	0.972790461
13897	'Ces1e'	0	1	0	0	6	3	0.333333333	3	2.941299941	0.268277725	0.883600995
13909	'Ces3b'	0	1	0	0	1	0	0.333333333	0.333333333	0.058500858	0.988561293	0.999493374
13929	'Amz2'	1224	1255	1228	674	1013	1244	1235.666667	977	-0.329419896	0.231938714	0.858905111
13972	'Gnb1l'	132	142	103	75	126	162	125.6666667	121	-0.039862285	0.904146532	0.987963813
13982	'Esr1'	819	834	176	43	977	1081	609.6666667	700.3333333	0.134317619	0.884837256	0.98388852
13983	'Esr2'	59	69	23	9	73	37	50.33333333	39.66666667	-0.365727364	0.623104472	0.972790461
13984	'Esx1'	41	29	6	0	13	36	25.33333333	16.33333333	-0.715304937	0.572092269	0.972790461
13990	'Smarcad1'	1589	1713	1098	342	1771	1370	1466.666667	1161	-0.390312735	0.450017933	0.970590732
13992	'Khdrbs3'	1011	999	507	388	1291	1124	839	934.3333333	0.147493563	0.739753553	0.972790461
13998	'Fgd6'	723	694	641	1004	489	719	686	737.3333333	0.271388428	0.600270256	0.972790461
13999	'Gm14288'	72.74	72.17	44.11	58.76	110.39	90.35	63.00666667	86.5	0.499743505	0.190689611	0.813136091
14000	'Drosha'	3257	3299	1470	665	2435	3172	2675.333333	2090.666667	-0.388201018	0.461254075	0.972790461
14004	'Chchd2'	2737.9	2782.1	3808.6	2596.9	2841.2	4522.95	3109.503333	3320.343333	0.096163713	0.809571917	0.972790461
14007	'Celf2'	1764	1720.5	1863	402.45	1435.7	1518.48	1782.483333	1118.87	-0.748401834	0.101854772	0.671776916
14008	'Etv2'	0	0	1	1	0	0	0.333333333	0.333333333	0.058500858	0.988561293	0.999493374
14009	'Etv1'	183	170	226	813.74	273	309	193	465.2466667	1.492583805	0.031156067	0.42861408
14011	'Etv6'	1052	1050	1743	2020	1278	1517	1281.666667	1605	0.423387615	0.43653149	0.966050133
14012	'Mpzl2'	284	213	246	212	156	247	247.6666667	205	-0.188550252	0.659401956	0.972790461
14013	'Mecom'	303	293	21	22	597	212	205.6666667	277	0.410829544	0.700628028	0.972790461
14017	'Evi2a'	11	7	0	10	21	37	6	22.66666667	1.960725879	0.068068231	0.583196579
14020	'Evi5'	2261	2471	1449	1554	2617	2538	2060.333333	2236.333333	0.16556779	0.546702906	0.972790461
14025	'Bcl11a'	139	104	403	102	113	88	215.3333333	101	-1.10203195	0.116757252	0.704694883

14026	'Evl'	2322	2309.1	1387	498.55	1679.5	1768.21	2006.04	1315.41	-0.63922627	0.145043185	0.752609452
14027	'Evpl'	234	225	83	66	282	218	180.6666667	188.6666667	0.058366257	0.917782987	0.990569977
14028	'Evx1'	22	21	6	0	10	3	16.33333333	4.333333333	-1.942453602	0.102762479	0.672865573
14029	'Evx2'	2	0	0	1	0	1	0.666666667	0.666666667	0.237205023	0.94341932	0.996436989
14030	'Ewsr1'	6017.2	6473	3976	4365	6380	3697	5488.746667	4814	-0.088762164	0.817884953	0.972790461
14038	'Wfdc18'	2	0	0	0	0	0	0.666666667	0	-1.711373851	0.67234292	0.972790461
14042	'Ext1'	1403	1496	2694	1561	1782	2051	1864.333333	1798	-0.051049113	0.914537084	0.989964366
14043	'Ext2'	1229	1282	555	1439	1791	1849	1022	1693	0.829939937	0.027728444	0.407338487
140474	'Muc4'	12	10	0	7	2	6	7.333333333	5	-0.301740603	0.831208896	0.974324192
140475	'Bsnd'	6	4	2	0	1	3	4	1.333333333	-1.629247949	0.30418639	0.906882806
140476	'Strc'	4	3	1	0	3	0	2.666666667	1	-1.422350783	0.501422491	0.972790461
140477	'Dmbx1'	5	5	2	1	0	0	4	0.333333333	-3.378440087	0.085237438	0.634259952
14048	'Eya1'	18	11	28	4	13	18	19	11.66666667	-0.808258288	0.316089529	0.914806078
140481	'Man2a2'	2608	2786	936	2563	2826	2468	2110	2619	0.457532827	0.330718962	0.921648675
140482	'Zfp358'	469	508	687	278	606	582	554.6666667	488.6666667	-0.226292629	0.575907896	0.972790461
140483	'Hnmt'	53	46	23	3	94	75	40.66666667	57.33333333	0.404210159	0.666000947	0.972790461
140484	'Pofut1'	1503	1506	554	808	1650	1284	1187.666667	1247.333333	0.13751308	0.752664062	0.972790461
140486	'Igf2bp1'	817	831	934	442	353	371	860.6666667	388.6666667	-1.044316372	0.015141225	0.306224446
140488	'Igf2bp3'	1698	1723	1771	585	1078	1448	1730.666667	1037	-0.764624885	0.021615139	0.364161986
140489	'Bhlhe23'	0	1	0	0	1	0	0.333333333	0.333333333	0.058500858	0.988561293	0.999493374
14049	'Eya2'	0	3	8	6	5	3	3.666666667	4.666666667	0.376129631	0.800106051	0.972790461
140491	'Ppp1r3a'	1	0	2	0	3	0	1	1	-0.190715569	0.950034966	0.998065566
140492	'Kcnn2'	145	199	38	93	254	180	127.3333333	175.6666667	0.524407123	0.391844104	0.952648938
140493	'Kcnn3'	44	24	2	123	39	35	23.33333333	65.66666667	1.860552741	0.075752528	0.605637955
140494	'Atp6v0a4'	1.01	2	0	16	3	0	1.003333333	6.333333333	3.132151764	0.125300571	0.720137383
140497	'Cd300c2'	10	6	1	19	15	20	5.666666667	18	1.82379369	0.043641248	0.487049111
140498	'Rxfp2'	5	13	6	3	6	11	8	6.666666667	-0.270289726	0.772859997	0.972790461
140499	'Ube2j2'	1097	1029	1359	902	870	1137	1161.666667	969.6666667	-0.215966419	0.577344694	0.972790461
14050	'Eya3'	1358	1307	1133	922	1303	1338	1266	1187.666667	-0.049109075	0.837563076	0.974723675
140500	'Acap3'	389	406	382	600	383	488	392.3333333	490.3333333	0.455026576	0.319756018	0.916646777
14051	'Eya4'	6	8	6	7	17	12	6.666666667	12	0.859508086	0.269475089	0.883851074
140546	'Eri3'	1046	1164	1006	886	1342	1321	1072	1183	0.174856255	0.486655921	0.972790461
14055	'Ezh1'	1485	1439	761	272	128	215	1228.333333	205	-2.389915898	8.07E-06	0.002131999
140557	'Smc1b'	8200	8339	2899	27	4600	5097	6479.333333	3241.333333	-1.098941944	0.33447962	0.921767653
140559	'Igsf8'	404	405	268	569	600	737	359	635.3333333	0.908664311	0.003656599	0.139157376
14056	'Ezh2'	1218	1229	1117	300	185	108	1188	197.666667	-2.554066681	1.05406E-11	0.026431132
14057	'Sfxn1'	597	616	966	483	637	863	726.3333333	661	-0.156118771	0.71487581	0.972790461
140570	'Plxnb2'	4442	4269	1831	5747	7264	7340	3514	6783.666667	1.053082715	0.006723064	0.194707262
140571	'Plxnb3'	1	1	1	5	2	4	1	3.666666667	2.010769102	0.185051414	0.805079516
140577	'Ankrd6'	279	289	83	71	310	355	217	245.3333333	0.160312601	0.798434813	0.972790461

140579	'Elmo2'	1049	1126	550	2411	1278	1110	908.3333333	1599.666667	1.058546753	0.071293387	0.591632421
14058	'F10'	0	1	0	0	2	3	0.333333333	1.666666667	2.081165771	0.487041593	0.972790461
140580	'Elmo1'	214	228	164	116	278	266	202	220	0.120001739	0.72521982	0.972790461
14060	'F13b'	1	0	1	3	0	0	0.666666667	1	0.96683075	0.75605331	0.972790461
14061	'F2'	6	13	1	5	2	9	6.666666667	5.333333333	-0.184868617	0.879292549	0.981800078
14062	'F2r'	756	651	891	1649	924	1125	766	1232.666667	0.824052208	0.119487441	0.710007992
140629	'Ubox5'	576.57	598.18	288	170.3	356	413.15	487.5833333	313.15	-0.616573721	0.108165075	0.684880244
14063	'F2rl1'	14	27	54	594	70	173	31.66666667	279	3.381595522	5.74E-04	0.047144852
140630	'Ube4a'	2662	2404	1001	1527	2515	2186	2022.333333	2076	0.120280974	0.766334807	0.972790461
14064	'F2rl2'	3	3	4	1	0	4	3.333333333	1.666666667	-1.036876432	0.519579278	0.972790461
14065	'F2rl3'	23	32	2	0	50	37	19	29	0.561421464	0.687493647	0.972790461
14066	'F3'	113	117	811	890	149	658	347	565.6666667	0.758658055	0.458916961	0.972790461
14067	'F5'	10	8	3	28	32	13	7	24.33333333	1.964877484	0.016779457	0.323948168
14068	'F7'	0	1	0	0	2	0	0.333333333	0.666666667	0.839717162	0.835573887	0.974723675
14069	'F8'	290	287	63	66	376	242	213.3333333	228	0.098661356	0.887374227	0.984534762
14070	'F8a'	120	119	119	82	122	177	119.3333333	127	0.089295941	0.802628155	0.972790461
140703	'Emid1'	131	136	5	19	190	138	90.66666667	115.6666667	0.349551918	0.737158884	0.972790461
140709	'Col26a1'	580	611	79	198	760	400	423.3333333	452.6666667	0.152095185	0.836009392	0.974723675
140721	'Caskin2'	1032	983	614	911	1191	906	876.3333333	1002.666667	0.293860391	0.394308997	0.953178011
140723	'Cacng5'	82	98	1	0	23	25	60.33333333	16	-1.953612598	0.184155456	0.804780995
14073	'Faah'	88	62	76	7	98	75	75.33333333	60	-0.450879613	0.5604631	0.972790461
140740	'Sec63'	2976	2868	3581	3575	3045	3620	3141.666667	3413.333333	0.195940607	0.630063175	0.972790461
140742	'Sesn1'	1413	1549	732	1585	1601	1290	1231.333333	1492	0.424233424	0.319064213	0.916136994
140743	'Rem2'	17	9	33	151	11	24	19.66666667	62	1.956360002	0.086521118	0.637937705
140765	'Tmprss3'	3	0	0	0	5	0	1	1.666666667	0.72323488	0.845053444	0.975182082
14077	'Fabp3'	84.35	103.02	533.97	793.88	129.33	265.77	240.4466667	396.3266667	0.880368606	0.39183084	0.952648938
140780	'Bmp2k'	1345.6	1402.6	2290.9	2028.4	1504.7	1517.39	1679.686667	1683.516667	0.091557414	0.861903216	0.976863639
140781	'Myh7'	14.1	15.07	5	7	86.26	36.03	11.39	43.09666667	1.882310687	0.035927739	0.455989547
140792	'Colec12'	2854	2775.4	1903.5	1421.4	3367.2	3204.25	2510.953333	2664.286667	0.087508962	0.783534292	0.972790461
140795	'P2ry14'	5	3	5	226	3	92	4.333333333	107	4.921692859	8.82E-05	0.012798273
14080	'Fabp1'	0	0	0	0	1	0	0	0.333333333	1.020273531	0.802557913	0.972790461
140806	'Il25'	4	5	2	0	2	0	3.666666667	0.666666667	-2.443465085	0.21104927	0.840335617
14081	'Acsl1'	805	777	271	250	429	638	617.6666667	439	-0.45022476	0.343917013	0.925308103
140810	'Ttbk2'	1680	1684	731	1359	1283	1077	1365	1239.666667	0.022145129	0.961408332	0.999493374
14082	'Fadd'	309	329	246	46	347	355	294.6666667	249.3333333	-0.335511341	0.594652309	0.972790461
14083	'Ptk2'	2497	2547	1891	1599	2010	2139	2311.666667	1916	-0.205514508	0.415815546	0.95722888
14084	'Faf1'	1347	1174	1430	570	1137	1844	1317	1183.666667	-0.210671738	0.613185954	0.972790461
14085	'Fah'	339	399	324	89	279	447	354	271.6666667	-0.455209564	0.364685079	0.935515019
140858	'Wdr5'	2186	2071	1919	878	1520	2100	2058.666667	1499.333333	-0.468092748	0.126673936	0.721703589
140859	'Nek8'	262	247	90	83	387	257	199.6666667	242.3333333	0.276192372	0.635203899	0.972790461

14086	'Fscn1'	2248	2134	5233	2786	2853	3134	3205	2924.333333	-0.131860609	0.81477453	0.972790461
14087	'Fanca'	377.43	349.69	63.03	140.97	273.89	173.28	263.3833333	196.0466667	-0.316936968	0.621941615	0.972790461
14088	'Fancc'	314	344	155	48	276	237	271	187	-0.576595253	0.326139341	0.920269559
140887	'Lnx2'	437	427	442	181	411	460	435.3333333	350.6666667	-0.344412722	0.328440603	0.921397778
14089	'Fap'	59	80	16	0	91	37	51.66666667	42.66666667	-0.338379742	0.78913216	0.972790461
140904	'Caln1'	125.59	99	3	18	44	18	75.86333333	26.66666667	-1.373707039	0.186628006	0.808423707
140917	'Dclre1b'	288.56	306.82	197	761.22	280.76	334.72	264.1266667	458.9	1.051671779	0.099027457	0.668585731
140919	'Slc17a6'	0	0	0	10	0	2	0	4	4.824368924	0.105976816	0.679124905
14102	'Fas'	25	26	499	62	38	51	183.3333333	50.33333333	-1.919624604	0.097376185	0.663789939
14103	'Fasl'	1	1	0	0	0	1	0.666666667	0.333333333	-0.744986434	0.839556299	0.974723675
14104	'Fasn'	5372	5197	1677	1649	5190	3688	4082	3509	-0.18662549	0.719452985	0.972790461
14105	'Srsf10'	3447	3688	3120	2272	3714	3121	3418.333333	3035.666667	-0.13391373	0.605248309	0.972790461
14106	'Foxh1'	41.29	31.75	14.03	0	21.9	7.15	29.02333333	9.683333333	-1.680929232	0.131345904	0.72897159
14107	'Fat1'	2530	2255	654	3100	3435	3159	1813	3231.333333	0.983923216	0.059805369	0.554643744
14109	'Fau'	5310	5352	15897	8650	3936	6232	8853	6272.666667	-0.422157913	0.557056244	0.972790461
14113	'Fbl'	1760	1841	2614	827	1292	1989	2071.666667	1369.333333	-0.642528359	0.125757266	0.72022881
14114	'Fbln1'	1168	1023	160	929	1370	987	783.6666667	1095.333333	0.629228788	0.342693233	0.925308103
14115	'Fbln2'	370	358	120	1592	791	697	282.6666667	1026.666667	2.132601583	0.00116267	0.071305454
14118	'Fbn1'	2118	1900	1339	788	2412	1925	1785.666667	1708.333333	-0.07650498	0.842616705	0.975182082
14119	'Fbn2'	5516	5556	1291	838	6469	4822	4121	4043	-0.051036833	0.943501929	0.996436989
14120	'Fbp2'	0	0	0	3	2	4	0	3	4.105951009	0.086310827	0.63725881
14121	'Fbp1'	6	13	0	6	3	5	6.333333333	4.666666667	-0.208002475	0.882092278	0.982560253
14123	'Fbrs'	1595	1484	1537	1134	1721	1251	1538.666667	1368.666667	-0.121496421	0.708935768	0.972790461
14126	'Ms4a2'	1	0	0	2	1	2	0.333333333	1.666666667	2.329090277	0.33135092	0.921648675
14127	'Fcer1g'	25	42	11	41	43	57	26	47	0.962978185	0.103811464	0.675747811
14128	'Fcer2a'	16.01	15.75	20.43	7.44	16.64	12.66	17.39666667	12.24666667	-0.554865014	0.407306827	0.957157474
14129	'Fcgr1'	10	8	0	8	7	20	6	11.66666667	1.059013699	0.377080029	0.941504733
14130	'Fcgr2b'	40	44.79	7.05	4	73	82	30.61333333	53	0.752245579	0.449015514	0.97049895
14131	'Fcgr3'	15	16.21	39.95	50	50	119	23.72	73	1.580988735	0.021454691	0.362574735
14132	'Fcgrt'	654	661	618	893	1042	1266	644.3333333	1067	0.775768953	0.006115389	0.184842241
14133	'Fcna'	3	0	0	3	2	11	1	5.333333333	2.455826231	0.190015347	0.813092906
14134	'Fcnb'	0	0	0	0	2	0	0	0.666666667	1.801491674	0.656112935	0.972790461
14137	'Fdft1'	1033.2	1112.2	1508.6	972.52	1044.1	1626.3	1218.01	1214.316667	6.55E-04	0.998712916	0.999900097
14148	'Fdx1'	311	324	1653	3268	350	847	762.6666667	1488.333333	1.166681779	0.27320867	0.884738928
14149	'Fdxr'	806	953	619	1602	1358	1300	792.6666667	1420	0.979431615	0.015156596	0.306224446
14151	'Fech'	983	1007	624	561	1130	1333	871.3333333	1008	0.219952243	0.491574269	0.972790461
14154	'Fem1a'	611	618	404	1037	567	693	544.3333333	765.6666667	0.681849026	0.179054893	0.798783498
14155	'Fem1b'	2412	2422	2507	4419	1846	2360	2447	2875	0.429134164	0.462757227	0.972790461
14156	'Fen1'	690	755	338	180	601	818	594.3333333	533	-0.18653035	0.716670164	0.972790461
14158	'Fer'	990	1097	1753	809	1090	1093	1280	997.3333333	-0.361934062	0.40897763	0.957157474

14159	'Fes'	147	130	69	20	179	94	115.3333333	97.66666667	-0.289038037	0.673583923	0.972790461
14160	'Lgr5'	1402	1412	39	103	2587	1816	951	1502	0.634247169	0.578943705	0.972790461
14161	'Fga'	0	0	0	0	2	1	0	1	2.320886	0.562538074	0.972790461
14163	'Fgd1'	1002.2	977.21	757.11	507.87	1015.3	1032.47	912.1766667	851.8666667	-0.091082952	0.73948305	0.972790461
14164	'Fgf1'	79	82	13	39	44	28	58	37	-0.465349327	0.528299992	0.972790461
14165	'Fgf10'	7	7	6	31	15	13	6.666666667	19.66666667	1.763902241	0.038208274	0.459930654
14166	'Fgf11'	1894	1422	2375	339	1126	722	1897	729	-1.446159749	0.003017787	0.122594353
14167	'Fgf12'	29	31	58	6	16	24	39.33333333	15.33333333	-1.457248345	0.036752548	0.457889546
14168	'Fgf13'	133	138	153	7	79	134	141.3333333	73.33333333	-1.097710533	0.167361655	0.781391371
14169	'Fgf14'	374.57	417.81	107.79	237.67	468.49	273.59	300.0566667	326.5833333	0.225040381	0.681149191	0.972790461
14170	'Fgf15'	0	0	0	5	0	0	0	1.666666667	3.648996947	0.355284993	0.931809687
14171	'Fgf17'	15	13	3	2	16	3	10.33333333	7	-0.535677459	0.633365088	0.972790461
14172	'Fgf18'	40	39	15	61	40	51	31.33333333	50.66666667	0.866899007	0.150722149	0.759755159
14173	'Fgf2'	611.61	544.77	169.44	1014.2	484.56	567.54	441.94	688.7633333	0.89919556	0.166358524	0.780893009
14174	'Fgf3'	6	2	0	1	6	2	2.666666667	3	0.220842059	0.895239468	0.985505807
14175	'Fgf4'	0	0	0	0	0	1	0	0.333333333	1.020273531	0.802557913	0.972790461
14176	'Fgf5'	3	0	3	0	0	0	2	0	-3.449098221	0.339402053	0.924888762
14177	'Fgf6'	0	1	0	0	0	0	0.333333333	0	-0.903279821	0.824807108	0.972790461
14178	'Fgf7'	30	20	92	94	14	7	47.33333333	38.33333333	-0.031279353	0.978737447	0.999493374
14179	'Fgf8'	1	3	2	0	0	4	2	1.333333333	-0.72446018	0.741879883	0.972790461
14180	'Fgf9'	118	97	76	89	60	72	97	73.66666667	-0.253580744	0.602233904	0.972790461
14181	'Fgfbp1'	0	0	1	1	1	1	0.333333333	1	1.557984566	0.561051704	0.972790461
14182	'Fgfr1'	1061	987	1104	3315	1222	1996	1050.666667	2177.666667	1.239942537	0.037783939	0.459930654
14183	'Fgfr2'	3138	3131	906	2081	3883	3235	2391.666667	3066.333333	0.442458624	0.366062441	0.936406787
14184	'Fgfr3'	204	206	75	141	141	147	161.6666667	143	-0.03868351	0.935768907	0.994413066
14186	'Fgfr4'	36	24	17	158	29	29	25.66666667	72	1.852271172	0.047415409	0.50504201
14187	'Akr1b8'	486	489	1285	11060	982	3079	753.3333333	5040.333333	2.992887713	0.002222878	0.105653383
14190	'Fgl2'	31	41	4	22	29	41	25.33333333	30.66666667	0.382462643	0.633114938	0.972790461
14191	'Fgr'	3	9	6	0	3	6	6	3	-1.112367237	0.409039111	0.957157474
14194	'Fh1'	1442	1478	1189	734	1443	1797	1369.666667	1324.666667	-0.057443814	0.849309826	0.975734242
14198	'Fhit'	45	56	59	13	49	30	53.33333333	30.66666667	-0.84266599	0.133428812	0.733301819
14199	'Fhl1'	483	395	560	96	789	710	479.3333333	531.6666667	0.015802412	0.980910476	0.999493374
14200	'Fhl2'	100	105	211	768	111	143	138.6666667	340.6666667	1.581723557	0.093674964	0.657826859
14201	'Fhl3'	198	157	282	816	175	294	212.3333333	428.3333333	1.248150598	0.113509779	0.698948431
14202	'Fhl4'	463	494	70	5	211	191	342.3333333	135.6666667	-1.391664635	0.179208568	0.798783498
14204	'Il4i1'	7.5	0	0	23	4.21	0	2.5	9.07	2.442645925	0.292118402	0.89958198
14205	'Vegfd'	21	12	7	69	45	38	13.33333333	50.66666667	2.128247108	0.002493886	0.112089265
14208	'Ppm1g'	1664	1785	1199	2691	1860	2083	1549.333333	2211.333333	0.67065597	0.125893563	0.72022881
14211	'Smc2'	1666	1789	494	288	1516	1630	1316.333333	1144.666667	-0.226842197	0.725493525	0.972790461
14218	'Sh3pxd2a'	3322	3061	2677	2193	3544	3209	3020	2982	0.017694386	0.942036983	0.996253096

14219	'Ccn2'	112	98	643	299	239	255	284.3333333	264.3333333	-0.131221339	0.878665423	0.981388029
14221	'Fjx1'	107	102	178	142	125	165	129	144	0.192836349	0.702251147	0.972790461
14225	'Fkbp1a'	2900	2952	5483	2682	3237	4436	3778.333333	3451.666667	-0.153719476	0.746558454	0.972790461
14226	'Fkbp1b'	26	43	150	13	43	56	73	37.33333333	-1.152048779	0.179628381	0.799788005
14227	'Fkbp2'	242	267.64	358.15	214.05	216.62	399.65	289.2633333	276.7733333	-0.06721169	0.879372192	0.981807495
14228	'Fkbp4'	3791	3900	3037	2229	4234	5661	3576	4041.333333	0.167368024	0.588079702	0.972790461
14229	'Fkbp5'	4058	4335	2727	1544	5135	4780	3706.666667	3819.666667	0.016149961	0.968817628	0.999493374
14230	'Fkbp10'	1208	1217.9	936	760	2060	2082	1120.63	1633.99	0.519890974	0.144623782	0.752114557
14231	'Fkbp7'	148	167	96	123	208	179	137	170	0.365665382	0.270070184	0.883851074
14232	'Fkbp8'	2535	2631	2582	2591	2702	3436	2582.666667	2909.666667	0.227541094	0.46597515	0.972790461
14234	'Foxc2'	20	20	29	57	14	14	23	28.33333333	0.557424252	0.539308187	0.972790461
14235	'Foxm1'	894	875	191	89	640	541	653.3333333	423.3333333	-0.647726515	0.375329739	0.940406075
14236	'Foxn2'	1147	1019	979	865	868	1155	1048.333333	962.6666667	-0.059296942	0.850605979	0.975734242
14237	'Foxd4'	1	1	2	1	0	0	1.333333333	0.333333333	-1.888603506	0.453455542	0.970649024
14238	'Foxf2'	3	2	10	128	2	151	5	93.66666667	4.282352726	9.62E-04	0.06437552
14239	'Foxs1'	72	69	481	112	77	111	207.3333333	100	-1.090117148	0.226504393	0.854001521
14241	'Foxl1'	3	4	0	4	4	3	2.333333333	3.666666667	0.830969784	0.568001967	0.972790461
14245	'Lpin1'	619	541	815	619	401	585	658.3333333	535	-0.212156265	0.667504942	0.972790461
14246	'Flg'	7	10	3	2	3	6	6.666666667	3.666666667	-0.822611358	0.439258592	0.967427574
14247	'Fli1'	210	194	78	46	276	158	160.6666667	160	-0.024022548	0.969356685	0.999493374
14248	'Flii'	2156	2139	2586	1314	2299	2421	2293.666667	2011.333333	-0.202485727	0.535323368	0.972790461
14251	'Flot1'	485	486	868	3209	692	1236	613	1712.333333	1.702413389	0.029651953	0.420076694
14252	'Flot2'	1353	1281	1370	891	1312	1804	1334.666667	1335.666667	-5.03E-04	0.998691152	0.999900097
14254	'Flt1'	257	186	146	37	148	88	196.3333333	91	-1.126298061	0.023690122	0.380402529
14255	'Flt3'	1	3	1	0	4	3	1.666666667	2.333333333	0.413996252	0.815015673	0.972790461
14256	'Flt3l'	18	20	28	41	28	41	22	36.66666667	0.810540493	0.183345077	0.804652954
14257	'Flt4'	154	134	71	111	166	153	119.6666667	143.3333333	0.335996914	0.37861859	0.94215965
14260	'Fmn1'	66	70	49	278	59	66	61.66666667	134.3333333	1.452116736	0.080332392	0.617305615
14261	'Fmo1'	69.04	103.21	26.15	11.35	84.34	44.42	66.13333333	46.70333333	-0.52324249	0.486155869	0.972790461
14262	'Fmo3'	0	0	1	0	0	0	0.333333333	0	-0.903279821	0.824807108	0.972790461
14263	'Fmo5'	140	161	49	69	170	153	116.6666667	130.6666667	0.205213867	0.688818433	0.972790461
14264	'Fmod'	150	137	3	20	161	89	96.66666667	90	-0.082813628	0.93953668	0.995607401
14265	'Fmr1'	10065	10191	3421	1110	5188	3466	7892.333333	3254.666667	-1.280029058	0.025372607	0.39436194
14266	'Aff2'	769	824	695	391	1315	1393	762.6666667	1033	0.382171394	0.372434459	0.938816563
14268	'Fn1'	5088	4108	7067	7589	5479	5380	5421	6149.333333	0.282990177	0.587772803	0.972790461
142681	'Slc34a3'	1	0	0	0	0	0	0.333333333	0	-0.903279821	0.824807108	0.972790461
142682	'Zcchc14'	1865	1882	1537	7577	1946	2438	1761.333333	3987	1.468124757	0.042382676	0.479588013
142687	'Asb14'	7	7	6	11	11	0	6.666666667	7.333333333	0.338582033	0.79930674	0.972790461
142688	'Asb13'	139	144	78	76	216	132	120.3333333	141.3333333	0.255098497	0.563906632	0.972790461
14269	'Fnbp1'	1452	1389	1123	1377	1395	1192	1321.333333	1321.333333	0.111434925	0.757988724	0.972790461

14270	'Srgap2'	2325	2145	1701	1916	1696	1389	2057	1667	-0.164697037	0.690511928	0.972790461
14272	'Fnta'	1342	1386	1559	1069	1493	1761	1429	1441	0.023155098	0.940211067	0.995805886
14275	'Folr1'	278	297	291	3220	434	1145	288.6666667	1599.666667	2.763552569	7.55E-04	0.055009373
14276	'Folr2'	17	6	6	9	14	17	9.666666667	13.33333333	0.506575195	0.51899377	0.972790461
14281	'Fos'	90	93	1471	882	161	197	551.3333333	413.3333333	-0.275441815	0.822470352	0.972790461
14282	'Fosb'	56	53	183	204	27	13	97.33333333	81.33333333	0.034153952	0.976289319	0.999493374
14283	'Fosl1'	8.73	10.72	81.36	187.29	4.7	21.15	33.60333333	71.04666667	1.356044081	0.338596897	0.924608313
14284	'Fosl2'	1628	1538	9510	20780	1730	3906	4225.333333	8805.333333	1.284621143	0.257725977	0.877405867
14287	'Fpgs'	316	285	335	594	366	473	312	477.6666667	0.740514563	0.125857253	0.72022881
14289	'Fpr2'	2	2	1	21	9	3	1.666666667	11	3.016187392	0.02043838	0.355230659
14293	'Fpr1'	5	11	0	40	9	7	5.333333333	18.66666667	2.227315923	0.10017277	0.671535889
14296	'Frat1'	51	73	54	193	72	93	59.33333333	119.3333333	1.231104647	0.063104479	0.566852792
14297	'Fxn'	203.41	180	120	26	211	137	167.8033333	124.6666667	-0.492934222	0.439832989	0.967612218
142980	'Tlr3'	55	66	29	31	123	105	50	86.33333333	0.772442272	0.160630062	0.771114735
14299	'Ncs1'	348	357	640	187	347	289	448.3333333	274.3333333	-0.744657543	0.117573917	0.705814751
14300	'Frg1'	732.09	822.14	1039	454.31	715.17	865.3	864.41	678.26	-0.36893072	0.31693061	0.916136994
14302	'Frk'	145	184	79	47	172	140	136	119.6666667	-0.190559221	0.705336869	0.972790461
14309	'Fshr'	0	0	0	0	2	1	0	1	2.320886	0.562538074	0.972790461
14311	'Cidec'	1	0	0	1	0	0	0.333333333	0.333333333	0.058500858	0.988561293	0.999493374
14312	'Brd2'	7464	7671	8835	6864	5444	7819	7989.98	6708.986667	-0.179195474	0.655499512	0.972790461
14313	'Fst'	2048	1901	305	27	3174	1889	1418	1696.666667	0.200823589	0.859639224	0.976863639
14314	'Fstl1'	5066	4830	6246	9229	6607	7021	5380.666667	7619	0.613716979	0.19306839	0.816149625
14317	'Ftcd'	6	1	0	1	2	1	2.333333333	1.333333333	-0.682740626	0.724156725	0.972790461
14319	'Fth1'	9348	9601	20385	23741	9564	17670	13111.33333	16991.66667	0.47818566	0.46996269	0.972790461
14325	'Ftl1'	11785	11601	49571	28760	14703	40737	24318.99667	28066.66667	0.170835412	0.827809623	0.973238667
14339	'Aktip'	910.69	825.25	899.98	958.09	898.7	1312.58	878.64	1056.456667	0.320942163	0.358716194	0.933467324
14343	'Fut1'	4	2	12	7	0	12	6	6.333333333	0.057717358	0.969326505	0.999493374
14344	'Fut2'	21.95	16.04	8	30	10	8	15.33	16	0.390248231	0.672556912	0.972790461
14345	'Fut4'	142	150	59	22	132	114	117	89.33333333	-0.423429167	0.503378857	0.972790461
14348	'Fut9'	4	3	17	60	5	7	8	24	1.853224758	0.15971851	0.771114735
14349	'Fv1'	69	56	17	91	60	51	47.33333333	67.33333333	0.735831944	0.280120377	0.889355605
14356	'Timm10b'	197	216	387	211	238	351	266.6666667	266.6666667	-0.019551646	0.96864742	0.999493374
14357	'Dtx1'	609	602	473	431	844	1103	561.3333333	792.6666667	0.488550933	0.125714248	0.72022881
14359	'Fxr1'	4308	4023	6205	5095	3511	4652	4845.333333	4419.333333	-0.049658962	0.919334041	0.990815958
14360	'Fyn'	1897	1906	1818	1450	2283	2411	1873.666667	2048	0.14653245	0.559815471	0.972790461
14362	'Fzd1'	609	523	2336	2991	537	1333	1156	1620.333333	0.634023867	0.495075901	0.972790461
14365	'Fzd3'	1048	1133	518	542	1377	1236	899.6666667	1051.666667	0.245317882	0.53902632	0.972790461
14366	'Fzd4'	338.37	348.68	95	262.65	457.22	346.63	260.6833333	355.5	0.547274745	0.288305647	0.895485536
14367	'Fzd5'	283	309	426	1332	340	531	339.3333333	734.3333333	1.340429535	0.067715846	0.580700792
14368	'Fzd6'	251	232	159	162	471	407	214	346.6666667	0.68566213	0.078730707	0.615899891

14369	'Fzd7'	811	770	684	886	610	731	755	742.3333333	0.110674566	0.797559823	0.972790461
14370	'Fzd8'	260	244	137	306	399	333	213.6666667	346	0.793454928	0.028260221	0.409827099
14371	'Fzd9'	8	10	8	110	7	25	8.666666667	47.33333333	2.807414284	0.010691924	0.255370433
14373	'G0s2'	163	177	105	160	207	190	148.3333333	185.6666667	0.406787333	0.227176396	0.854001521
14375	'Xrcc6'	1040	1058	743	458	1045	1555	947	1019.333333	0.077937003	0.843134336	0.975182082
14376	'Ganab'	3808	4026	1727	2673	5268	5125	3187	4355.333333	0.497407807	0.187032488	0.808483975
14378	'G6pc2'	6	1	5	0	1	0	4	0.333333333	-3.457562072	0.095618059	0.662411648
14381	'G6pdx'	781	823	1517	1496	853	1708	1040.333333	1352.333333	0.427483491	0.453630939	0.970649024
14385	'Slc37a4'	168	148	483	85	210	224	266.3333333	173	-0.749659677	0.257409899	0.877405867
14387	'Gaa'	1744	1653	1521	4818	2649	4041	1639.333333	3836	1.371685198	0.003765311	0.140640662
14388	'Gab1'	2176	2259.9	1953.1	1650.6	2595.2	2767.99	2129.656667	2337.93	0.158414727	0.497976191	0.972790461
14389	'Gab2'	1651	1668	1599	773	1389	1538	1639.333333	1233.333333	-0.412842631	0.133849207	0.733301819
14390	'Gabpa'	2299	2317	2494	1930	2190	2172	2370	2097.333333	-0.117222809	0.722526129	0.972790461
14391	'Gabpb1'	1359	1322	1338	905	1190	1104	1339.666667	1066.333333	-0.280009166	0.341181317	0.925308103
14394	'Gabra1'	13	12	4	0	24	13	9.666666667	12.33333333	0.278863649	0.825513159	0.972790461
14395	'Gabra2'	13	10	10	1	11	7	11	6.333333333	-0.860275561	0.361486842	0.933706718
14396	'Gabra3'	95	86	34	15	300	482	71.66666667	265.6666667	1.7934456	0.050141984	0.518969204
14397	'Gabra4'	924	950	245	331	872.99	543	706.3333333	582.33	-0.213939455	0.699630396	0.972790461
14400	'Gabrb1'	317	357	57	104	364.01	179	243.6666667	215.67	-0.114394086	0.869192268	0.979633196
14401	'Gabrb2'	26	20	18	3	23	19	21.33333333	15	-0.573136722	0.448322858	0.97034542
14402	'Gabrb3'	119	120	81	8	123	58	106.6666667	63	-0.843080724	0.27487981	0.885974997
14403	'Gabrd'	2	3	0	6	3	7	1.666666667	5.333333333	1.846774585	0.192158422	0.813837363
14404	'Gabre'	52	47	96	126	29	29	65	61.33333333	0.163049187	0.85497766	0.975734242
14405	'Gabrg1'	2	2	0	0	5	0	1.333333333	1.666666667	0.321999298	0.908542587	0.988823459
14406	'Gabrg2'	0	0	0	0	3	0	0	1	2.343657704	0.558557988	0.972790461
14407	'Gabrg3'	88	94	5	2	101	69	62.33333333	57.33333333	-0.156469596	0.895453864	0.985505807
14408	'Gabrr1'	33	15	9	0	9	7	19	5.333333333	-1.889205655	0.084397944	0.631721936
14409	'Gabrr2'	1	2	8	0	1	2	3.666666667	1	-2.075493348	0.254034872	0.873052317
14411	'Slc6a12'	2	5	5	3188	5	827	4	1340	8.731637333	1.64E-09	1.36E-06
14412	'Slc6a13'	32	39	2	17	46	21	24.33333333	28	0.308971962	0.745253662	0.972790461
14415	'Gad1'	5	5	106	15	3	17	38.66666667	11.66666667	-1.786521285	0.183183273	0.804128465
14417	'Gad2'	2	1	1	0	0	0	1.333333333	0	-2.782259126	0.384965334	0.94674509
14419	'Gal'	0	0	5	5	1	3	1.666666667	3	0.870852372	0.679580047	0.972790461
14420	'Galc'	676.54	760.92	603.5	398.8	1362	1054.24	680.32	938.3366667	0.428016884	0.296736667	0.90029104
14421	'B4galnt1'	35.58	38	152.54	50.48	33.68	36	75.37333333	40.05333333	-0.894742707	0.28010645	0.889355605
14422	'B4galnt2'	17	17	18	20	17	38	17.33333333	25	0.55426718	0.362579063	0.934677306
14423	'Galnt1'	3154	3197	1607	2338	4271	5143	2652.666667	3917.333333	0.594421262	0.086459378	0.637860184
14425	'Galnt3'	75	60	13	23	39	42	49.33333333	34.66666667	-0.422309335	0.529451979	0.972790461
14426	'Galnt4'	319	352	179	174	396	351	283.3333333	307	0.142359893	0.697980581	0.972790461
14427	'Galr1'	3	3	1	0	0	0	2.333333333	0	-3.553368527	0.187188305	0.808483975

14428	'Galr2'	12	16	17	43	13	10	15	22	0.81074436	0.378148082	0.9419364
14429	'Galr3'	1.04	2	1	3	2	2	1.346666667	2.333333333	0.942838558	0.54154128	0.972790461
14430	'Galt'	231	216	232	146	289	310	226.3333333	248.3333333	0.119235953	0.719046394	0.972790461
14431	'Gamt'	256	315	119	44	346	297	230	229	-0.058322332	0.930585554	0.993474751
14432	'Gap43'	153	138	36	1	151	111	109	87.66666667	-0.389137533	0.729883405	0.972790461
14433	'Gapdh'	34340	29353	237929	32839	19494	38175.92	100540.6367	30169.67667	-1.7893733	0.048600815	0.512194398
14447	'Gapdhs'	1	0	2	0	0	2	1	0.666666667	-0.786979378	0.803234187	0.972790461
14450	'Gart'	1787	1946	974	1087	2237	2196	1569	1840	0.261712117	0.443137507	0.96975311
14451	'Gas1'	1400	1315	2429	3095	1501	1621	1714.666667	2072.333333	0.417354988	0.510331644	0.972790461
14453	'Gas2'	540.09	556.17	170.59	157.27	547.65	515.87	422.2833333	406.93	-0.046379577	0.933172105	0.994031155
14456	'Gas6'	6437	7088	863	6071	15374	17668	4796	13037.66667	1.494879705	0.03236881	0.434162511
14457	'Gas7'	1790	2092	1852	3111	2501	2462	1911.333333	2691.333333	0.616446615	0.138920497	0.740857359
14460	'Gata1'	56	63	3	162	74	119	40.66666667	118.3333333	1.807698725	0.051092582	0.524065182
14461	'Gata2'	205	221	68	93	130	111	164.6666667	111.3333333	-0.451340931	0.363903015	0.93523525
14462	'Gata3'	114	89	401	301	74	139	201.3333333	171.3333333	-0.101057533	0.910671497	0.989608384
14463	'Gata4'	5147	5631	5523	3929	6805	6349	5433.666667	5694.333333	0.077847077	0.779267969	0.972790461
14464	'Gata5'	21	15	2	13	22	16	12.66666667	17	0.541475192	0.535959305	0.972790461
14465	'Gata6'	2262	2261	3396	1014	2686	2521	2639.666667	2073.666667	-0.417818233	0.346973713	0.928289998
14466	'Gba'	420	400	538	577	471	658	452.6666667	568.6666667	0.390469578	0.362720869	0.934677306
14467	'Nipsnap2'	1121	1161	1350	493	1425	2212	1210.666667	1376.666667	0.094831628	0.846388753	0.975201539
14468	'Gbp2b'	37	51	38	0	10	2	42	4	-3.455463727	3.90E-04	0.036760231
14469	'Gbp2'	721	737	1660	78	544	548	1039.333333	390	-1.592069608	0.026456543	0.398883266
14470	'Rabac1'	762	699	1171	1478	1016	1791	877.3333333	1428.333333	0.750520967	0.13444208	0.734275447
14472	'Gbx2'	17	11	4	0	4	2	10.66666667	2	-2.422284695	0.054421999	0.535821363
14473	'Gc'	13	11	0	1	21	31	8	17.66666667	1.104839211	0.435823142	0.96581102
14479	'Usp15'	1908	2010	1126	1354	1709	1874	1681.333333	1645.666667	0.048754041	0.871143358	0.980126495
14489	'Mtpn'	4430	4340	6980	3381	4215	5330	5250	4308.666667	-0.292023316	0.485958859	0.972790461
14525	'Gcsam'	2	1	0	0	2	0	1	0.666666667	-0.54242627	0.865172559	0.978040999
14526	'Gcg'	0	1	0	0	0	2	0.333333333	0.666666667	0.78767502	0.845746136	0.975182082
14527	'Gcgr'	0	3	1	9	1	2	1.333333333	4	1.922456353	0.285376047	0.893820325
14528	'Gch1'	75	92	818	878	108	333	328.3333333	439.6666667	0.549104269	0.631083108	0.972790461
14533	'Bloc1s1'	406	505	180	156	402	522	363.6666667	360	-0.009709777	0.984198605	0.999493374
14534	'Kat2a'	1767	1763	753	740	1801	1659	1427.666667	1400	1.84E-04	0.999647034	0.999900097
14536	'Nr6a1'	1299	1308	373	433	632	769	993.33	611.3333333	-0.613468565	0.211347885	0.840335617
14537	'Gcnt1'	147	100	35	166	85	95	94	115.3333333	0.541375474	0.426885887	0.961605981
14538	'Gcnt2'	49	57	42	205	85	87	49.33333333	125.6666667	1.582476583	0.016357412	0.319945603
14544	'Gda'	11	7	136	520	17	146	51.33333333	227.6666667	2.317238202	0.089557624	0.646660839
14545	'Gdap1'	42	34	9	0	20	14	28.33333333	11.33333333	-1.380134793	0.224218641	0.850759465
14547	'Gdap2'	552	549	654	507	415	492	585	471.3333333	-0.227302215	0.586428931	0.972790461
14548	'Mrps33'	367	402	548	238	347	408	439	331	-0.418712243	0.283811568	0.892170588

14555	'Gpd1'	19	13	6	30	22	22	12.66666667	24.66666667	1.130231628	0.11289373	0.698666814
14559	'Gdf1'	0	0.01	1.19	0	0	102	0.4	34	6.347580088	0.018257793	0.33668007
14560	'Gdf10'	114	120	2	477	126	229	78.66666667	277.3333333	2.168365278	0.04998306	0.518369283
14561	'Gdf11'	540	567	199	1054	649	670	435.3333333	791	1.081854582	0.05698069	0.547413448
14562	'Gdf3'	0	3	0	0	2	6	1	2.666666667	1.357904296	0.609703893	0.972790461
14563	'Gdf5'	11	13	15	10	3	3	13	5.333333333	-1.087676694	0.270703326	0.883910323
14566	'Gdf9'	6	11	19	4	34	137	12	58.33333333	2.060626556	0.056411821	0.545987502
14567	'Gdi1'	2438	2581	2673	2036	2867	2337	2564	2413.333333	-0.042381396	0.894417916	0.985259027
14569	'Gdi2'	7161.9	6996.9	11868	5654	6675	9406.99	8675.56	7245.306667	-0.27361347	0.536361868	0.972790461
14570	'Arhgdig'	133	149	418	14	110	213	233.3333333	112.3333333	-1.28201144	0.142666699	0.749054337
14571	'Gpd2'	561	557	728	1104	550	664	615.3333333	772.6666667	0.483778051	0.393696571	0.953178011
14573	'Gdnf'	19	16	151	12	33	31	62	25.33333333	-1.481705664	0.142113958	0.74797073
14579	'Gem'	218	200	1010	10339	251	892	476	3827.333333	3.309919721	0.009920923	0.245872663
14580	'Gfap'	63	81	13	0	118	58	52.33333333	58.66666667	0.105216362	0.935728765	0.994413066
14581	'Gfi1'	5	6	8	29	7	6	6.333333333	14	1.410032136	0.190084798	0.813136091
14582	'Gfi1b'	12	12	1	0	4	2	8.333333333	2	-2.052549873	0.166268069	0.780893009
14583	'Gfpt1'	2709	2436	8736	2290	1993	3408	4627	2563.666667	-0.898147818	0.184374935	0.804780995
14584	'Gfpt2'	68	46	848	8657	49	1295	320.6666667	3333.666667	3.605550334	0.016476286	0.321279678
14585	'Gfra1'	35	13	11	10	29	31	19.66666667	23.33333333	0.246730316	0.730864173	0.972790461
14586	'Gfra2'	105	114	25	34	144	83	81.33333333	87	0.125844355	0.852203165	0.975734242
14587	'Gfra3'	23	25	1	3	4	1	16.33333333	2.666666667	-2.414725485	0.06071518	0.559627259
14588	'Gfra4'	101	79	35	1	60	128	71.66666667	63	-0.297090176	0.783521183	0.972790461
14590	'Ggh'	202	261	156	184	454	430	206.3333333	356	0.786817745	0.030132867	0.422171018
14593	'Ggps1'	798	773	1251	2093	1088	1159	940.6666667	1446.666667	0.764872706	0.201520784	0.82952222
14594	'Ggta1'	262	264	156	338	344	344	227.3333333	342	0.703703041	0.055913602	0.543749055
14595	'B4galt1'	1381	1286	1908	4634	1801	2574	1525	3003	1.148741573	0.065792121	0.574593252
14598	'Ggt1'	2	3	2	27	1	2	2.333333333	10	2.522460349	0.100066113	0.671297438
14600	'Ghr'	1026	937	1175	776	1372	1126	1046	1091.333333	0.065709321	0.846827278	0.975209338
14601	'Ghrh'	1	2	14	0	0	0	5.666666667	0	-5.046199118	0.032075271	0.433742328
14605	'Tsc22d3'	2292	2195	900	600	1213	1764	1795.666667	1192.333333	-0.5720699	0.204466325	0.833273525
14608	'Gpr83'	8	15	4	3	2	1	9	2	-1.987860664	0.091056899	0.649965349
14609	'Gja1'	3553	3459	5416	1734	4648	3880	4142.666667	3420.666667	-0.342355202	0.447087294	0.970198456
14610	'Gja10'	47	39	7	0	25	5	31	10	-1.664033861	0.191621748	0.813837363
14611	'Gja3'	4	1	2	0	1	1	2.333333333	0.666666667	-1.844075161	0.346791402	0.928289998
14612	'Gja4'	244	256	23	23	417	334	174.3333333	258	0.5326876	0.589498788	0.972790461
14613	'Gja5'	5	7	3	33	1	18	5	17.33333333	2.09017705	0.093318858	0.656130962
14615	'Gjc1'	946	906	683	185	756	646	845	529	-0.722905029	0.115438412	0.702825844
14616	'Gja8'	14	22	11	0	13	8	15.66666667	7	-1.245509893	0.251715969	0.871252558
14617	'Gjd2'	0	2	0	0	0	0	0.666666667	0	-1.695595436	0.675304306	0.972790461
14619	'Gjb2'	6	4	23	1	5	10	11	5.333333333	-1.247904037	0.296257218	0.90029104

14620	'Gjb3'	30	20	7	32	45	76	19	51	1.478006248	0.019324633	0.347258906
14621	'Gjb4'	3	2	0	12	15	10	1.666666667	12.33333333	3.024841986	0.00842974	0.222807422
14622	'Gjb5'	8	5	3	16	20	21	5.333333333	19	1.910033357	0.010569934	0.254415968
14626	'Gk2'	6	3	1	0	3	0	3.333333333	1	-1.734882932	0.400106081	0.955860942
14628	'Ostm1'	309	293	299	984	449	705	300.3333333	712.6666667	1.410703397	0.008909434	0.231288612
14629	'Gclc'	898	903	611	1515	717	1345	804	1192.333333	0.734517967	0.146907428	0.753611197
14630	'Gclm'	1006	962	597	514	848	1786	855	1049.333333	0.282360943	0.522931463	0.972790461
14632	'Gli1'	166	165	39	38	141	29	123.3333333	69.33333333	-0.754882992	0.326004972	0.920269559
14633	'Gli2'	376	396	279	157	318	161	350.3333333	212	-0.670086822	0.095442055	0.662411648
14634	'Gli3'	1184	1120	802	707	936	699	1035.333333	780.6666667	-0.317043083	0.337413175	0.92380462
14635	'Galk1'	1236	1192	2709	565	978	1586	1712.333333	1043	-0.81250461	0.146837224	0.753611197
14645	'Glul'	1663	1710	1221	2175	1623	1964	1531.333333	1920.666667	0.461632081	0.249017116	0.86963876
14651	'Hagh'	231.15	215.78	398.56	429	205.16	517.06	281.83	383.74	0.501944236	0.402129328	0.955996639
14652	'Glp1r'	2	0	7	8	0	0	3	2.666666667	0.145688363	0.954776715	0.999493374
14657	'Glra4'	10	12	1	6	12	2	7.666666667	6.666666667	-0.041259117	0.972530686	0.999493374
14658	'Glrb'	119	120	41	5	73	90	93.33333333	56	-0.809124828	0.338510748	0.924608313
14659	'Glrp1'	0	0	0	2	0	1	0	1	2.714766126	0.495614689	0.972790461
14660	'Gls'	4425	4404	2630	8015	4378	2787	3819.666667	5060	0.656293435	0.288099708	0.895138365
14661	'Glud1'	2616	2447	2340	1631	2730	3422	2467.666667	2594.333333	0.071313019	0.797051742	0.972790461
14664	'Slc6a9'	362.85	320.15	597.46	776.27	704.78	1289.15	426.82	923.4	1.108918459	0.025502951	0.395185754
14667	'Gm2a'	1462	1414	1581	2716	1773	2048	1485.666667	2179	0.682345561	0.145412722	0.753611197
14670	'Gnl1'	1632.2	1653.7	2453.2	1744.7	1325.9	2168.1	1913.066667	1746.236667	-0.085987537	0.853179567	0.975734242
14672	'Gna11'	1709	1727	1376	1265	1658	2129	1604	1684	0.109062843	0.661127527	0.972790461
14673	'Gna12'	1172	1137	967	1335	1200	1396	1092	1310.333333	0.361759606	0.294876648	0.90029104
14674	'Gna13'	1590	1534	6643	6335	1491	2672	3255.666667	3499.333333	0.243328349	0.788099205	0.972790461
14675	'Gna14'	147	147	35	127	135	179	109.6666667	147	0.548362483	0.334635029	0.921773568
14676	'Gna15'	1	2	0	3	4	10	1	5.666666667	2.539243302	0.095495987	0.662411648
14677	'Gnai1'	577	512	1515	441	630	1168	868	746.3333333	-0.322511653	0.607952672	0.972790461
14678	'Gnai2'	3777	3609	4874	2576	4623	4684	4086.666667	3961	-0.065921998	0.853078274	0.975734242
14679	'Gnai3'	5551	5666	11595	4802	5561	5907	7604	5423.333333	-0.490436465	0.334481837	0.921767653
14680	'Gnal'	97.88	61.04	33.68	58.01	65.57	35.89	64.2	53.15666667	-0.115967781	0.846180244	0.975182082
14681	'Gnao1'	227	204	177	166	276	182	202.6666667	208	0.092383122	0.791896776	0.972790461
14682	'Gnaq'	1926	1958	1944	1055	1777	1895	1942.666667	1575.666667	-0.296613314	0.271424447	0.884495016
14683	'Gnas'	12252	12244	21931	16083	15263	17562	15475.66667	16302.66667	0.106701701	0.825391397	0.972790461
14685	'Gnat1'	0	1	1	2	0	0	0.666666667	0.666666667	0.369435326	0.909857284	0.989364653
14686	'Gnat2'	6	11	14	12	5	5	10.33333333	7.333333333	-0.342798778	0.723444002	0.972790461
14687	'Gnaz'	320	323	122	86	211	332	255	209.6666667	-0.284228945	0.587587608	0.972790461
14688	'Gnb1'	7484	7682	11500	6335	6799	8819	8888.666667	7317.666667	-0.264550863	0.524339496	0.972790461
14693	'Gnb2'	2554	2695	4624	3277	3464	3722	3291	3487.666667	0.106746993	0.820026897	0.972790461
14694	'Rack1'	14270	14454	28619	31642	13772	18639	19114.33333	21351	0.290851357	0.656072495	0.972790461

14695	'Gnb3'	8	8	12	2	10	8	9.333333333	6.666666667	-0.560021839	0.53801413	0.972790461
14696	'Gnb4'	945.47	961.55	978.93	284.27	1218.8	1377.31	961.9833333	960.13	-0.092669353	0.855940696	0.975734242
14697	'Gnb5'	248	301	541	481	402	440	363.3333333	441	0.327703249	0.548887222	0.972790461
14699	'Gngt1'	0	0	0	1	0	1	0	0.666666667	2.022653929	0.615796212	0.972790461
14700	'Gng10'	404	373	610	69	413	361	462.3333333	281	-0.850314177	0.169219867	0.784876338
14701	'Gng12'	2042	2082	5607	5539	2122	3322	3243.666667	3661	0.285910169	0.696074069	0.972790461
14702	'Gng2'	1086	1072	1986	196	732	850	1381.333333	592.6666667	-1.349918933	0.019083414	0.344064642
14704	'Gng3'	12.48	13.77	5.19	5.39	8.23	5.06	10.48	6.226666667	-0.646492436	0.451827976	0.970649024
14705	'Bscl2'	293.52	328.23	208.81	262.61	477.77	422.94	276.8533333	387.7733333	0.524259759	0.071003887	0.591553852
14706	'Gng4'	5	9	6	1	4	3	6.666666667	2.666666667	-1.333275701	0.225095622	0.851475917
14707	'Gng5'	1213	1265	2504	656	1168	1561	1660.666667	1128.333333	-0.633978386	0.214022836	0.842446242
14708	'Gng7'	144	147	36	92	140	156	109	129.3333333	0.338040231	0.538298117	0.972790461
14709	'Gng8'	20	25	10	15	19	19	18.33333333	17.66666667	0.034217384	0.955577471	0.999493374
14710	'Gngt2'	25	21	0	25	32	30	15.33333333	29	1.080773361	0.325417788	0.920269559
14711	'Gnmt'	5	3	3	5	0	2	3.666666667	2.333333333	-0.382616238	0.806203581	0.972790461
14712	'Gnpat'	2157	2062	1091	828	1757	1929	1770	1504.666667	-0.21372216	0.550219084	0.972790461
14714	'Gnrh1'	2	0	0	3	1	1	0.666666667	1.666666667	1.615262839	0.496671587	0.972790461
14715	'Gnrhr'	4	0	0	0	3	2	1.333333333	1.666666667	0.287578281	0.917451156	0.990547571
14718	'Got1'	775	784	1690	3096	806	1440	1083	1780.666667	0.893844394	0.242211329	0.86449675
14719	'Got2'	2369	2469	2693	2919	2334	3069	2510.333333	2774	0.228278616	0.5652148	0.972790461
14723	'Gp1ba'	167	152	49	79	93	91	122.6666667	87.66666667	-0.358326367	0.490589659	0.972790461
14724	'Gp1bb'	2.34	3.42	9.32	19.91	5.6	4.34	5.026666667	9.95	1.196189396	0.336358058	0.92281928
14725	'Lrp2'	131	110	16	314	100	41	85.66666667	151.6666667	1.229661339	0.216455374	0.843391934
14726	'Pdpn'	523	561	494	5230	1163	1964	526	2785.666667	2.680638716	2.40E-04	0.025741813
14727	'Lilr4b'	10.47	14.94	29.54	46.49	25.78	45.32	18.31666667	39.19666667	1.183789592	0.11563362	0.702825844
14728	'Lilrb4a'	39.53	37.06	39.46	91.51	43.22	96.68	38.68333333	77.13666667	1.112211693	0.053944306	0.534315058
14729	'Gp5'	4	3	4	27	9	10	3.666666667	15.33333333	2.287186972	0.024829589	0.389862092
14731	'Gpaa1'	640	630	231	748	872	873	500.3333333	831	0.854779769	0.052906379	0.532526438
14732	'Gpam'	2627	2625	751	2482	3265	2433	2001	2726.666667	0.586349838	0.253148847	0.871990757
14733	'Gpc1'	1093.1	964.26	754.79	3711.4	1878.3	1674.27	937.3666667	2421.346667	1.59282738	0.005644781	0.177386075
14734	'Gpc3'	2032	1882	687	1768	2465	2437	1533.666667	2223.333333	0.63639597	0.142625462	0.749054337
14735	'Gpc4'	1709	1793	688	1967	3151	2675	1396.666667	2597.666667	0.983881669	0.015423491	0.308989889
14738	'Gpr12'	0	2	1	0	1	1	1	0.666666667	-0.64771931	0.805025839	0.972790461
14739	'S1pr2'	503	515	622	669	533	717	546.6666667	639.6666667	0.301312426	0.472444768	0.972790461
14744	'Gpr65'	4	6	0	0	8	5	3.333333333	4.333333333	0.353069488	0.846067578	0.975182082
14745	'Lpar1'	284	321	228	215	262	319	277.6666667	265.3333333	-0.008920191	0.975155618	0.999493374
14747	'Cmklr1'	74	84	50	11	46	50	69.33333333	35.66666667	-0.995918143	0.07170169	0.592919704
14748	'Gpr3'	3	3	14	87	8	15	6.666666667	36.66666667	2.712951561	0.028751311	0.413166984
14751	'Gpi1'	7465	5851	49505	6551	4612	9854	20940.33	7005.663333	-1.668925837	0.063588797	0.567640559
14755	'Pigq'	1208	1290	557	515	1316	1239	1018.333333	1023.333333	0.026926567	0.948368169	0.997386511

14756	'Gpld1'	185.26	185.35	20.38	5.34	250.14	147.67	130.33	134.3833333	5.63E-04	0.999589336	0.999900097
14758	'Gpm6b'	623.12	614.07	418.42	1345.6	877.21	587	551.87	936.6	0.973704336	0.07971121	0.617305615
14760	'Gpr19'	239	207	104	27	188	145	183.3333333	120	-0.658140671	0.297533989	0.900750988
14761	'Gpr27'	154.3	150.98	123.52	1	131.53	206.79	142.9333333	113.1066667	-0.490192026	0.65379514	0.972790461
14762	'Gpr33'	0	0	0	0	1	0	0	0.333333333	1.020273531	0.802557913	0.972790461
14763	'Gpr37'	370	344	54	86	550	396	256	344	0.426766707	0.579683003	0.972790461
14764	'Ptgdr2'	3.03	4.06	0	6.44	0	2.03	2.363333333	2.823333333	0.597263122	0.768110351	0.972790461
14765	'Gpr50'	10	7	6	3	8	18	7.666666667	9.666666667	0.286347932	0.751518714	0.972790461
14766	'Adgrg1'	1808	1838	884	1214	3724	3745	1510	2894.333333	0.931710636	0.034047456	0.443971353
14767	'Nmur1'	0	1	0	1	0	1	0.333333333	0.666666667	1.054274597	0.766617507	0.972790461
14768	'Lancl1'	1170	1259	691	926	1129	1408	1040	1154.333333	0.22430315	0.468969019	0.972790461
14772	'Grk4'	716.17	745.1	175.71	86.01	376.63	253.28	545.66	238.64	-1.176564174	0.063252842	0.566979274
14773	'Grk5'	894	881	341	1525	815	678	705.3333333	1006	0.770500597	0.216727215	0.843391934
14775	'Gpx1'	904	1046	1328	716	1310	1088	1092.666667	1038	-0.08460518	0.82372171	0.972790461
14776	'Gpx2'	0	1	1	8	1	1	0.666666667	3.333333333	2.632479689	0.187982714	0.809755101
14778	'Gpx3'	280	241	582	595	130	162	367.6666667	295.6666667	-0.084428274	0.921467417	0.991066749
14782	'Gsr'	593	601	1114	678	893	1948	769.3333333	1173	0.531641618	0.323174569	0.918976057
14783	'Grb10'	5972	5963	14169	4885	4578	5512	8701.333333	4991.666667	-0.795849938	0.159523297	0.771073131
14784	'Grb2'	1205	1263	2176	2301	1362	2097	1548	1920	0.388857408	0.481273476	0.972790461
14786	'Grb7'	14	4	7	54	14	23	8.333333333	30.33333333	2.115969194	0.024592322	0.388329921
14787	'Rhpn1'	74	74	18	39	49	35	55.33333333	41	-0.286270233	0.650407193	0.972790461
14788	'Gpr162'	323	337	105	47	333	163	255	181	-0.508130957	0.455390091	0.971158318
14789	'P3h3'	1099	1020	481	200	1404	1087	866.6666667	897	5.10E-04	0.999342177	0.999900097
14790	'Grcc10'	664	785	548	316	893	921	665.6666667	710	0.065524393	0.862974219	0.977227788
14791	'Emg1'	835.89	827.67	1437.7	676.8	680.53	841.85	1033.766667	733.06	-0.47733726	0.307197534	0.907830798
14792	'Lpcat3'	654.11	672.33	378.26	637.2	1003.5	1060.15	568.2333333	900.2733333	0.717107903	0.014957027	0.305110509
14793	'Cdca3'	200	239	125	49	260	156	188	155	-0.309393694	0.576640311	0.972790461
14794	'Spsb2'	114	114	286	113	121	199	171.3333333	144.3333333	-0.293923454	0.625600028	0.972790461
14797	'Tle5'	4692	4797	5338	5327	6110	7085	4942.333333	6174	0.36196497	0.259855581	0.878537553
14799	'Gria1'	68	50	5	7	22	16	41	15	-1.382339576	0.129248515	0.726372284
14800	'Gria2'	72	60	8	3	86	34	46.66666667	41	-0.214330809	0.840852093	0.975182082
14802	'Gria4'	150	128	50	16	114	55	109.3333333	61.66666667	-0.842312987	0.223031918	0.85026726
14803	'Grid1'	125	99	33	0	223	135	85.66666667	119.3333333	0.395318202	0.76165049	0.972790461
14804	'Grid2'	52	50	28	3	104	228	43.33333333	111.6666667	1.237036824	0.228734181	0.855540033
14805	'Grik1'	37	22	15	13	18	30	24.66666667	20.33333333	-0.241915857	0.692202727	0.972790461
14806	'Grik2'	13	12	3	16	14	7	9.333333333	12.33333333	0.605660321	0.503963836	0.972790461
14807	'Grik3'	1233	1376	100	6	613	437	903	352	-1.405941829	0.235284581	0.861636582
14809	'Grik5'	1804	1776	996	916	2247	1432	1525.333333	1531.666667	0.041328634	0.913268801	0.989773583
14810	'Grin1'	16	8	16	1	7	0	13.33333333	2.666666667	-2.35806641	0.067628943	0.580700792
14811	'Grin2a'	48	30	39	10	22	10	39	14	-1.452117394	0.018097474	0.335677246

14812	'Grin2b'	64	53	20	2	20	8	45.66666667	10	-2.19641827	0.007796168	0.213267264
14813	'Grin2c'	28	38	19	6	24	4	28.33333333	11.33333333	-1.285167415	0.121154921	0.712451374
14814	'Grin2d'	91	108	50	15	88	43	83	48.66666667	-0.788280278	0.209282173	0.836952601
14815	'Nr3c1'	1686	1557	2519	1506	1361	1909	1920.666667	1592	-0.239113564	0.593377799	0.972790461
14816	'Grm1'	5	4	0	13	0	0	3	4.333333333	1.10244365	0.654329465	0.972790461
14823	'Grm8'	3	2	4	1	4	1	3	2	-0.612318165	0.668396661	0.972790461
14824	'Grn'	1130	1151	1027	1599	1789	2209	1102.666667	1865.666667	0.814535809	0.003835434	0.141968654
14825	'Cxcl1'	79	96	5489	2022	32	181	1888	745	-1.117917753	0.594901794	0.972790461
14827	'Pdia3'	8663	7977.9	14003	5263	9092	22437.98	10214.62667	12264.31	0.155354811	0.784341336	0.972790461
14828	'Hspa5'	15973	15152	36585	14596	14396	35041.38	22569.82	21344.42667	-0.152200604	0.799701715	0.972790461
14829	'Grpr'	1	0	1	5	1	3	0.666666667	3	2.334195117	0.215621114	0.843391934
14836	'Gsc'	0	0	1	0	0	1	0.333333333	0.333333333	0.058500858	0.988561293	0.999493374
14840	'Gsg1'	0	0	0	1	1	1	0	1	2.526085132	0.463240869	0.972790461
14841	'Haspin'	409	371	62	74	160	236	280.6666667	156.6666667	-0.796222491	0.240644305	0.86449675
14843	'Gsx2'	0	0	0	1	0	0	0	0.333333333	1.020273531	0.802557913	0.972790461
14852	'Gspt1'	3219	3221	2471	3978	3398	4671	2970.333333	4015.666667	0.532321912	0.121054889	0.712316789
14853	'Gspt2'	198	202	150	40	257	315	183.3333333	204	0.061900259	0.920897951	0.991047523
14854	'Gss'	285	311	658	371	400	452	418	407.6666667	-0.038927751	0.9427494	0.996301887
14857	'Gsta1'	0	0	0	0	0	9.6	0	3.2	3.842052349	0.329786588	0.921648675
14858	'Gsta2'	2	1	3	127	7	103.67	2	79.22333333	5.476533448	5.35E-06	0.001517766
14859	'Gsta3'	52	68	546	1229	73	806	222	702.6666667	1.738843143	0.140894186	0.744698431
14860	'Gsta4'	553	650	414	895	606	2009	539	1170	1.160827748	0.020411734	0.355230659
14862	'Gstm1'	2859	3153	4316	4899	6737	10180	3442.666667	7272	1.051766867	0.009162963	0.234872395
14863	'Gstm2'	5724	6446	2796	2660	11959	12477	4988.666667	9032.023333	0.820312582	0.128028028	0.724425259
14864	'Gstm3'	1	0	3	11	2	5	1.333333333	6	2.316480149	0.148777295	0.757633614
14865	'Gstm4'	175	207	121	74	246.93	273	167.6666667	197.9766667	0.204904631	0.656969397	0.972790461
14866	'Gstm5'	602	673	698	388	807	770	657.6666667	655	-0.023479388	0.94307847	0.996301887
14867	'Gstm6'	458	657	342	211	1865	1432	485.6666667	1169.333333	1.191204495	0.068578672	0.583886121
14869	'Gstp2'	10.22	7.81	15.55	26.41	7.42	40.46	11.19333333	24.76333333	1.231720116	0.166712805	0.781229856
14870	'Gstp1'	1500.7	1777.8	1780.4	3260.8	1157	2136.49	1686.303333	2184.766667	0.555113528	0.352920149	0.930050349
14871	'Gstt1'	183	207	43	229	315	438	144.3333333	327.3333333	1.272018582	0.025297053	0.393896457
14872	'Gstt2'	146	178	141	45	176	214	155	145	-0.16648142	0.745679438	0.972790461
14873	'Gsto1'	487	558	904	698	475	636	649.6666667	603	-0.035808031	0.946403721	0.996592403
14874	'Gstz1'	550.62	597.86	376.21	134.3	620.89	480.35	508.23	411.8466667	-0.346810449	0.483505978	0.972790461
14884	'Gtf2h1'	1679	1588	2764	923	1174	1471	2010.333333	1189.333333	-0.77126033	0.079789524	0.617305615
14885	'Gtf2h4'	432	495	255	118	387	538	394	347.6666667	-0.218230845	0.661250026	0.972790461
14886	'Gtf2i'	6712	6757	3977	2018	8684	7118	5815.333333	5940	-0.005026705	0.991633605	0.999493374
14894	'Cfap20'	941	993	1014	477	911	929	982.6666667	772.3333333	-0.356920737	0.237077339	0.862313827
14897	'Trip12'	14814	14202	7929	8150	10419	11314	12315	9961	-0.224176894	0.469433495	0.972790461
14904	'Gtpbp1'	1085	1112	534	729	996	1030	910.3333333	918.3333333	0.095751695	0.780323244	0.972790461

14911	'Thumpd3'	763	793	666	288	736	840	740.6666667	621.3333333	-0.286757727	0.429363549	0.962386546
14912	'Nkx6-2'	22.01	18.01	6.03	2	9.03	3.01	15.35	4.68	-1.66880323	0.082343572	0.622509308
14913	'Guca1a'	9	13	58	9	6	12	26.66666667	9	-1.622189987	0.105279247	0.678099427
14917	'Gucy2c'	1	2	0	2	2	3	1	2.333333333	1.338523244	0.451729408	0.970649024
14918	'Gucy2d'	0	1	0	0	0	0	0.333333333	0	-0.903279821	0.824807108	0.972790461
14919	'Gucy2e'	17	17	3	8	11	31	12.33333333	16.66666667	0.467413401	0.599564859	0.972790461
14923	'Guk1'	357	404	498	230	498	603	419.6666667	443.6666667	0.027454731	0.946313203	0.996592403
14924	'Magi1'	2473	2526	1473	2621	2939	2489	2157.333333	2683	0.431455677	0.226997751	0.854001521
14933	'Gk'	220	254	243	281	185	207	239	224.3333333	0.043330752	0.928085916	0.993096978
14934	'Gypa'	18	39	7	2	122	9	21.33333333	44.33333333	1.018032482	0.420269403	0.958308933
14936	'Gys1'	1544.6	1380.6	10890	383.94	990.3	1292.15	4605.023333	888.7966667	-2.579950161	0.003940263	0.144339639
14939	'Gzmb'	8	1	0	0	1	0	3	0.333333333	-2.910927952	0.299387574	0.903198439
14940	'Gzmc'	4	1	0	0	1	1	1.666666667	0.666666667	-1.293320005	0.602288328	0.972790461
14945	'Gzmk'	0	3	0	0	0	0	1	0	-2.275512037	0.569613276	0.972790461
14950	'H13'	1271	1268	2773	973	1729	2156	1770.666667	1619.333333	-0.20619328	0.69575559	0.972790461
14957	'H1f3'	4	3	12	1	1	5	6.333333333	2.333333333	-1.567406549	0.242497431	0.86449675
14958	'H1f0'	6546	6339	11455	6029	4988	6652	8113.333333	5889.666667	-0.423057805	0.39448548	0.953222922
14960	'H2-Aa'	5	9	23	2	17	19	12.33333333	12.66666667	-0.151439255	0.88472481	0.98386821
14961	'H2-Ab1'	46	54	47	161	43	77	49	93.66666667	1.170244686	0.105747764	0.679124905
14962	'Cfb'	15	13	105	50	35	47	44.33333333	44	-0.047672182	0.960909028	0.999493374
14964	'H2-D1'	1453.8	1500.4	1977.5	1628.8	1202.4	1512.56	1643.87	1447.916667	-0.097131744	0.831617102	0.974405544
14969	'H2-Eb1'	34	28	77	2	13	27	46.33333333	14	-1.912721902	0.032621873	0.435538654
14972	'H2-K1'	1386.4	1297.1	2344.1	1191	1072.6	1656.89	1675.85	1306.846667	-0.346333344	0.47287161	0.972790461
14976	'Pfdn6'	780	815	776	454	478	831	790.3333333	587.6666667	-0.40133395	0.244280015	0.866091613
14977	'Slc39a7'	1834	1804	1985	2523	2069	2886	1874.333333	2492.666667	0.48712305	0.204728671	0.833294914
14979	'H2-Ke6'	321	343	216	379	396	422	293.3333333	399	0.543670707	0.105293034	0.678099427
14990	'H2-M2'	89	122	112	0	32	13	107.6666667	15	-2.974151734	0.002478574	0.111665047
14991	'H2-M3'	31	28	54	50	34	88	37.66666667	57.33333333	0.604382384	0.338776642	0.924608313
14998	'H2-DMa'	177	146.01	102.05	192.04	91	109.94	141.6866667	130.9933333	0.095320401	0.869916973	0.979794316
14999	'H2-DMb1'	31	37.77	21.69	14.89	48.84	50.01	30.15333333	37.91333333	0.309131377	0.582342875	0.972790461
15000	'H2-DMb2'	3	6.23	2.31	2.11	11.16	3.99	3.846666667	5.753333333	0.553800659	0.646183876	0.972790461
15001	'H2-Oa'	4	12	5	4	14	9	7	9	0.366247856	0.692318935	0.972790461
15002	'H2-Ob'	3	9	1	0	0	0	4.333333333	0	-4.419234069	0.063510371	0.567415027
15006	'H2-Q1'	37.09	36.66	87.67	2.19	17.56	7	53.80666667	8.916666667	-2.750317556	0.001280049	0.075554997
15007	'H2-Q10'	26.04	26.35	79.66	36	8	8	44.01666667	17.33333333	-1.129611744	0.262477408	0.88013169
15013	'H2-Q2'	30.15	38	108.72	2	10	17	58.95666667	9.666666667	-2.781017943	0.001774823	0.09046942
15015	'H2-Q4'	417.47	373.28	568.05	201.12	307.02	402.81	452.9333333	303.65	-0.604722205	0.135474677	0.736012522
15018	'H2-Q7'	10.54	0	6.37	3.74	7.28	15.6	5.636666667	8.873333333	0.559189522	0.67756316	0.972790461
15039	'H2-T22'	298.12	286.87	662.02	840.07	308.99	657.03	415.67	602.03	0.632981641	0.36002547	0.933706718
15040	'H2-T23'	75.27	60.08	226.11	374.1	74.36	229.82	120.4866667	226.0933333	1.028733415	0.242799505	0.864924901

15042	'H2-T24'	9	4	8	47	19	43	7	36.33333333	2.500774059	0.001656292	0.08820568
15043	'H2-T3'	1	0	0	0	0	0	0.333333333	0	-0.903279821	0.824807108	0.972790461
15061	'Ifi44l'	0	0	1	0	1	0	0.333333333	0.333333333	0.058500858	0.988561293	0.999493374
15064	'Mr1'	273	228	107	786	329	415	202.6666667	510	1.584876698	0.011841656	0.26929851
15077	'H3c14'	0	0	0	0	8.01	0	0	2.67	3.747197207	0.342161114	0.925308103
15078	'H3f3a'	6567.1	7164.1	7768.7	4170.5	6922.2	6765.22	7166.616667	5952.633333	-0.263400752	0.383404317	0.945630828
15081	'H3f3b'	14402	14556	41361	7651	16145	19282	23439.61333	14359.33333	-0.82250016	0.181238742	0.801887586
15107	'Hadh'	1598	1704	1325	1971	2282	2525	1542.333333	2259.333333	0.619907551	0.021840131	0.365836629
15108	'Hsd17b10'	940	1056	1725	830	1331	1588	1240.333333	1249.666667	-0.030561965	0.945537168	0.996592403
15109	'Hal'	4	11	4	40	11	11	6.333333333	20.66666667	2.012451595	0.049119202	0.515210762
15110	'Hand1'	0	0	4	0	0	0	1.333333333	0	-3.00798671	0.447987178	0.970198456
15111	'Hand2'	41	32	53	39	24	9	42	24	-0.627077979	0.434317138	0.965118278
15112	'Hao1'	5	11	1	2	17	15	5.666666667	11.33333333	0.986127283	0.38940946	0.951231321
15114	'Hap1'	77	75	33	808	285	336	61.66666667	476.3333333	3.234271009	2.82E-06	9.17E-04
15115	'Hars'	1122	1240	1256	547	1111	1561	1206	1073	-0.210259087	0.571523998	0.972790461
15116	'Has1'	3	5	2	76	0	7	3.333333333	27.66666667	3.523964309	0.030155709	0.422179923
15117	'Has2'	38	25	234	109	35	48	99	64	-0.547269799	0.596873428	0.972790461
15118	'Has3'	155	149	28	54	137	64	110.6666667	85	-0.286052261	0.675600421	0.972790461
15122	'Hba-a1'	3031.5	3030.4	9854.1	154.16	5496.8	998.64	5305.34	2216.516667	-1.476042039	0.156749099	0.768070584
15126	'Hba-x'	32	29	156	0	30	4	72.33333333	11.33333333	-2.920874447	0.026660831	0.401009275
15132	'Hbb-bh1'	3	3	2	0	0	0	2.666666667	0	-3.780202362	0.131381076	0.72897159
15135	'Hbb-y'	102	165	384	3	36	3	217	14	-4.105317174	4.83E-05	0.008204297
15139	'Hc'	11	4	3	0	3	5	6	2.666666667	-1.23187533	0.371702476	0.938527219
15159	'Hccs'	1225	1255	1144	1308	784	1087	1208	1059.666667	-0.050327431	0.912628488	0.989773583
15160	'Serpind1'	0	2	0	1	1	2	0.666666667	1.333333333	1.106361402	0.654069527	0.972790461
15161	'Hcfc1'	6449	6008	2532	1567	4772	3401	4996.333333	3246.666667	-0.598887142	0.19575734	0.821394515
15162	'Hck'	20	13	11	17	17	25	14.66666667	19.66666667	0.48927275	0.427217914	0.961670256
15163	'Hcls1'	89	104	16	68	121	92	69.66666667	93.66666667	0.538404922	0.41553063	0.95722888
15165	'Hcn1'	10	17	20	3	8	9	15.66666667	6.666666667	-1.281402183	0.111937724	0.69684179
15166	'Hcn2'	36	31	32	4	29	56	33	29.66666667	-0.274444155	0.730779708	0.972790461
15168	'Hcn3'	109	97	85	17	163	80	97	86.66666667	-0.243880965	0.722324925	0.972790461
15170	'Ptpn6'	51	58	33	31	64	60	47.33333333	51.66666667	0.154193835	0.717079001	0.972790461
15171	'Hcrt'	0	0	0	0	0	1	0	0.333333333	1.020273531	0.802557913	0.972790461
15182	'Hdac2'	3523	3510	3190	2372	3593	4914	3407.666667	3626.333333	0.095341835	0.731311715	0.972790461
15183	'Hdac3'	1590	1813	1708	1027	1730	1753	1703.666667	1503.333333	-0.17098905	0.53365544	0.972790461
15184	'Hdac5'	1399	1479	3531	2162	1639	1597	2136.333333	1799.333333	-0.191932451	0.756883037	0.972790461
15185	'Hdac6'	1159	1118	579	1077	1637	1973	952	1562.333333	0.768444644	0.020003379	0.349865912
15186	'Hdc'	160	187	58	7145	165	1574	135	2961.333333	4.893423833	1.28E-05	0.003158437
15191	'Hdgf'	2881	2985	5184	3609	3442	5211	3683.333333	4087.333333	0.155781169	0.743877339	0.972790461
15192	'Hdgfl1'	51	50	3	2	23	46	34.66666667	23.66666667	-0.588865301	0.605258059	0.972790461

15193	'Hdgfl2'	2124	2121	1198	1310	1564	1686	1814.333333	1520	-0.165420625	0.599684091	0.972790461
15194	'Htt'	3484	3299	909	1343	2519	1868	2564	1910	-0.330766635	0.521755284	0.972790461
15199	'Hebp1'	332	304	508	109	297	363	381.3333333	256.3333333	-0.665434771	0.184964341	0.804884883
15200	'Hbegf'	44	49	104	120	40	55	65.66666667	71.66666667	0.28489872	0.716348524	0.972790461
15201	'Hells'	4278	4326	1833	299	2410	3003	3479	1904	-0.940858856	0.173725639	0.79261444
15202	'Gml2'	17	18	0	0	6	17.42	11.66666667	7.806666667	-0.65691906	0.702061183	0.972790461
15203	'Heph'	788	773	104	890	1086	953	555	976.3333333	0.975808199	0.150251348	0.759693458
15204	'Herc2'	5723	5738	1272	5076	5304	3993	4244.333333	4791	0.357867085	0.546858165	0.972790461
15205	'Hes1'	515	583	846	275	788	915	648	659.3333333	-0.066747167	0.891654925	0.985171
15206	'Hes2'	2	1	2	2	4	1	1.666666667	2.333333333	0.527854033	0.729687502	0.972790461
15207	'Hes3'	8	11	4	0	6	1	7.666666667	2.333333333	-1.744088501	0.210253909	0.838721635
15208	'Hes5'	9	12	5	1	4	8	8.666666667	4.333333333	-1.02476587	0.31855499	0.916136994
15209	'Hesx1'	0	1	3	0	1	0	1.333333333	0.333333333	-1.964832992	0.528894878	0.972790461
15211	'Hexa'	958.99	1036	956	1178	1530	2041	983.6633333	1582.996667	0.710511193	0.011162604	0.261358894
15212	'Hexb'	375	362	875.98	455	566.92	862.96	537.66	628.2933333	0.168016834	0.764363793	0.972790461
15213	'Hey1'	361	362	344	77	383	363	355.6666667	274.3333333	-0.458073951	0.385279555	0.946930692
15214	'Hey2'	52	31	457	6	38	58	180	34	-2.690259112	0.015674928	0.311076958
15216	'Hfe'	77.29	72.17	42.02	48.71	169.98	107.24	63.82666667	108.6433333	0.761612412	0.123555005	0.715310266
15218	'Foxn1'	0	0	1	1	0	0	0.333333333	0.333333333	0.058500858	0.988561293	0.999493374
15220	'Foxq1'	21	35	14	1467	18	925	23.33333333	803.3333333	5.402579974	2.52E-06	8.70E-04
15221	'Foxd3'	3	8	1	1	5	4	4	3.333333333	-0.240181782	0.856433445	0.975935786
15223	'Foxj1'	20	23	21	12	26	21	21.33333333	19.66666667	-0.110481304	0.842814506	0.975182082
15227	'Foxf1'	12	10	15	20	7	12	12.33333333	13	0.228139594	0.78756442	0.972790461
15228	'Foxg1'	7	15	0	24	3	2	7.333333333	9.666666667	0.884053646	0.578857164	0.972790461
15229	'Foxd1'	7	6	16	15	1	0	9.666666667	5.333333333	-0.511483771	0.749715865	0.972790461
15233	'Hgd'	0	1	3	0	0	1	1.333333333	0.333333333	-1.964920319	0.529831773	0.972790461
15234	'Hgf'	50	31	19	53	32	50	33.33333333	45	0.587829925	0.332423538	0.921648675
15235	'Mst1'	9	13	0	0	0	0	7.333333333	0	-5.149623498	0.052670981	0.532526438
15239	'Hgs'	1632	1538	2614	1279	1201	1646	1928	1375.333333	-0.464081122	0.312122578	0.911537035
15242	'Hhex'	62	57	23	22	39	44	47.33333333	35	-0.382021888	0.462450354	0.972790461
15245	'Hhip'	50	66	5	0	58	85	40.33333333	47.66666667	0.176781025	0.896610941	0.985710789
15247	'Mfsd14a'	1292	1270	978	878	1341	1695	1180	1304.666667	0.168661372	0.506286973	0.972790461
15248	'Hic1'	659.74	608.5	1182.4	241.34	564.57	356.44	816.8733333	387.45	-1.126186939	0.027246854	0.404239957
15251	'Hif1a'	4169	4449	6287	42802	6715	14997	4968.333333	21504.66667	2.373942406	0.003583962	0.137931741
15254	'Hint1'	1814	1892	2606	924	1437	2079	2104	1480	-0.544308803	0.173199879	0.791556755
15257	'Hipk1'	2330	2219	2422	7686	2436	3101	2323.666667	4407.666667	1.161343379	0.082167768	0.622135682
15258	'Hipk2'	1850.7	1875.8	1093.9	2597	1726.8	1840.99	1606.806667	2054.91	0.533960189	0.256324128	0.876684814
15259	'Hipk3'	2283	2169	3111	2669	2458	2716	2521	2614.333333	0.112749693	0.791495264	0.972790461
15260	'Hira'	1117.4	1090.6	898.84	833.89	1040.5	1171.75	1035.583333	1015.383333	0.02675047	0.915877921	0.990491125
15267	'H2ac18'	62.87	82.7	125.86	20	50.1	72.13	90.47666667	47.41	-1.015038694	0.082087658	0.622020945

15270	'H2ax'	751.49	725.29	391.4	254.54	687.11	700.25	622.7266667	547.3	-0.186528467	0.642769432	0.972790461
15273	'Hivep2'	415	372.51	1633	988	321	371	806.8366667	560	-0.39309014	0.655533759	0.972790461
15275	'Hk1'	1761	1545	9887	3029	1409	2118	4397.666667	2185.333333	-0.980465078	0.267178768	0.883600995
15277	'Hk2'	2102	1505	15161	2394	911	1930	6256	1745	-1.837711312	0.059888829	0.555146963
15278	'Tfb2m'	613	653	894	480	598	549	720	542.3333333	-0.378398611	0.345360555	0.926100052
15284	'Hlx'	89	79	24	8	237	94	64	113	0.764854804	0.421882865	0.959445304
15285	'Mnx1'	0	0	0	1	0	0	0	0.333333333	1.020273531	0.802557913	0.972790461
15288	'Hmbs'	482	540	432	240	716	634	484.6666667	530	0.098083206	0.796795401	0.972790461
15289	'Hmgb1'	8422	8783.9	6820	2809	8465.8	7300.79	8008.623333	6191.846667	-0.398482092	0.282037915	0.890082399
15312	'Hmgn1'	4636	5047	9778	3891	4487	5309	6487	4562.333333	-0.517452552	0.299948959	0.903723114
15331	'Hmgn2'	5162.6	5430.3	5560	381.2	6772.4	5343.13	5384.293333	4165.57	-0.510130694	0.513491315	0.972790461
15353	'Hmg20b'	452	447	310	880	561	497	403	646	0.871315117	0.087575462	0.642214018
15354	'Hmgb3'	2612	2716	2120	322	2831	2415	2482.666667	1856	-0.518455904	0.417736479	0.957720764
15356	'Hmgcl'	560	599	1195	1061	594	796	784.6666667	817	0.146728989	0.810055217	0.972790461
15357	'Hmgcr'	1337	1324	749	1018	1561	1289	1136.666667	1289.333333	0.258162172	0.416315533	0.95722888
15360	'Hmgcs2'	4013	4634	54	1737	9310	6302	2900.333333	5783	1.034530947	0.343891522	0.925308103
15361	'Hmga1'	1395	1400.1	11073	3241.6	636.96	3773.88	4622.533333	2550.82	-0.879306509	0.390837104	0.952178913
15364	'Hmga2'	690	673	1302	574	603	560	888.3333333	579	-0.588190861	0.248477243	0.869491261
15366	'Hmmr'	576.25	581.99	197.33	47	428	454	451.8566667	309.6666667	-0.601258654	0.408517577	0.957157474
15368	'Hmox1'	1440	1398	5100	3751	1662	3426	2646	2946.333333	0.190285876	0.801850754	0.972790461
15369	'Hmox2'	1137.8	1193.2	1217.8	741.2	958.7	1378	1182.923333	1025.966667	-0.193968301	0.532811677	0.972790461
15370	'Nr4a1'	173	202	1069	5922	137	362	481.3333333	2140.333333	2.457370052	0.065707618	0.574593252
15371	'Hmx1'	26	39	9	0	10	14	24.66666667	8	-1.687106383	0.123118512	0.714726459
15372	'Hmx2'	0	0	0	0	1	0	0	0.333333333	1.020273531	0.802557913	0.972790461
15373	'Hmx3'	0	3	4	0	3	0	2.333333333	1	-1.379682663	0.602978873	0.972790461
15374	'Jpt1'	1291	1405	1443	1069	1496	1847	1379.666667	1470.666667	0.104412545	0.722956346	0.972790461
15375	'Foxa1'	28	23	2	3	10	5	17.66666667	6	-1.475133449	0.165767687	0.780440621
15376	'Foxa2'	2	1	1	1	1	0	1.333333333	0.666666667	-0.843860865	0.694141352	0.972790461
15377	'Foxa3'	9	11	26	57	8	33	15.33333333	32.66666667	1.249465962	0.206531015	0.834040055
15378	'Hnf4a'	2	2	0	1	0	0	1.333333333	0.333333333	-1.740850292	0.569351619	0.972790461
15379	'Onecut1'	5	5	6	5	7	6	5.333333333	6	0.203832373	0.82511313	0.972790461
15381	'Hnrnpc'	6263	6509	9872	5581	6747	9020	7548	7116	-0.090198328	0.827199294	0.973067256
15382	'Hnrnpa1'	16141	16201	33281	5208	13826	12630	21874.33	10554.66667	-1.146071731	0.030405985	0.4240152
15384	'Hnrnpab'	7038.1	7631.3	7117.5	3378.8	7536.7	8130.92	7262.3	6348.82	-0.220041925	0.493908528	0.972790461
15387	'Hnrnpk'	12769	13285	18272	7301	13690	15387	14775.33333	12126	-0.324009082	0.39856527	0.954563638
15388	'Hnrnpl'	4509	4791	5256	2566	4694	4260	4852	3840	-0.339812065	0.273684022	0.885016511
15394	'Hoxa1'	5	7	10	10	4	3	7.333333333	5.666666667	-0.19934807	0.852106391	0.975734242
15395	'Hoxa10'	21	29	10	63	25	8	20	32	1.017248629	0.290630329	0.898595513
15396	'Hoxa11'	1	4	2	4	2	1	2.333333333	2.333333333	0.203587663	0.891757186	0.985171
15398	'Hoxa13'	13	12	0	0	3	0	8.333333333	1	-3.020853922	0.170833143	0.786030911

15399	'Hoxa2'	16	21	7	18	16	11	14.66666667	15	0.198702127	0.790457802	0.972790461
15400	'Hoxa3'	98	97	47	93	60	39	80.66666667	64	-0.10871711	0.863537704	0.977452409
15401	'Hoxa4'	47	42	17	19	20	26	35.33333333	21.66666667	-0.605944936	0.293465145	0.90029104
15402	'Hoxa5'	25	21	32	83	28	16	26	42.33333333	0.959580004	0.276176091	0.88793704
15403	'Hoxa6'	26	30	22	27	22	11	26	20	-0.215327487	0.753472371	0.972790461
15404	'Hoxa7'	18	14	8	12	21	17	13.33333333	16.66666667	0.38082736	0.554492964	0.972790461
15405	'Hoxa9'	31	32	24	88	45	21	29	51.33333333	1.076641116	0.162961285	0.774492858
15408	'Hoxb13'	0	0	0	2	0	0	0	0.666666667	2.299096387	0.566358544	0.972790461
15410	'Hoxb3'	110	88	48	94	141	98	82	111	0.531833737	0.228703302	0.855540033
15412	'Hoxb4'	21	25	12	107	40	33	19.33333333	60	1.920427687	0.015111704	0.306224446
15413	'Hoxb5'	41	33	6	7	56	25	26.66666667	29.33333333	0.143142415	0.87377823	0.980171118
15414	'Hoxb6'	27	14	10	32	40	30	17	34	1.105860743	0.071224404	0.591632421
15415	'Hoxb7'	49	44	40	138	95	116	44.33333333	116.3333333	1.521331035	0.001855614	0.09357806
15416	'Hoxb8'	35	33	16	48	61	43	28	50.66666667	0.968226319	0.063601209	0.567640559
15417	'Hoxb9'	57	81	173	214	137	124	103.6666667	158.3333333	0.698862134	0.315298207	0.914723408
15421	'Hoxc12'	0	0	0	1	0	0	0	0.333333333	1.020273531	0.802557913	0.972790461
15422	'Hoxc13'	13	26	8	27	5	9	15.66666667	13.66666667	0.121502056	0.903252489	0.987531419
15423	'Hoxc4'	301	265	274	137	263	151	280	183.6666667	-0.575109371	0.137125315	0.738570697
15424	'Hoxc5'	211	242	68	44	169	88	173.6666667	100.3333333	-0.758661992	0.209539851	0.837080112
15425	'Hoxc6'	436	492	401	433	486	304	443	407.6666667	-0.007713884	0.985388107	0.999493374
15426	'Hoxc8'	199	188	270	133	199	269	219	200.3333333	-0.152066208	0.708417026	0.972790461
15427	'Hoxc9'	61	54	74	34	65	35	63	44.66666667	-0.479491801	0.345405785	0.926100052
15429	'Hoxd1'	59	39	32	6	12	10	43.33333333	9.333333333	-2.177861783	1.82E-04	0.021021471
15430	'Hoxd10'	143	139	83	206	167	115	121.6666667	162.6666667	0.597388226	0.249472831	0.869928149
15431	'Hoxd11'	34	41	30	106	38	22	35	55.33333333	0.950607732	0.246979185	0.868602029
15432	'Hoxd12'	0	0	0	0	1	1	0	0.666666667	1.775692139	0.660844521	0.972790461
15433	'Hoxd13'	5	7	0	2	0	1	4	1	-1.679363437	0.414508659	0.95722888
15434	'Hoxd3'	229	228	41	72	212	55	166	113	-0.452207785	0.549385618	0.972790461
15436	'Hoxd4'	370	404	77	78	413	170	283.6666667	220.3333333	-0.33598661	0.641059405	0.972790461
15437	'Hoxd8'	670	756	363	358	617	530	596.3333333	501.6666667	-0.185172886	0.590032819	0.972790461
15438	'Hoxd9'	609	591	366	349	541	415	522	435	-0.18858867	0.558691237	0.972790461
15439	'Hp'	7	3	134	439	18	35	48	164	2.002919469	0.187683397	0.808805329
15441	'Hp1bp3'	3833	3740	3608	2689	4730	3987	3727	3802	0.049702039	0.854679809	0.975734242
15442	'Hpse'	125	87	230	344	122	228	147.3333333	231.3333333	0.772545756	0.281461891	0.889731741
15444	'Hpca'	85	78	23	2	69	17	62	29.33333333	-1.122122469	0.267831115	0.883600995
15445	'Hpd'	1	0	0	1	0	0	0.333333333	0.333333333	0.058500858	0.988561293	0.999493374
15446	'Hpgd'	139	129	94	1487	221	179	120.6666667	629	2.773180525	0.003142436	0.126308648
15450	'Lipc'	1	0	1	1	3	4	0.666666667	2.666666667	1.92623313	0.307757045	0.907963267
15451	'Hpn'	22	20	10	34	19	27	17.33333333	26.66666667	0.790932852	0.238785003	0.864053647
15452	'Hprt'	1360	1310	2236	580	1046	1322	1635.333333	982.6666667	-0.792671264	0.077555784	0.612838969

15458	'Hpx'	17	21	26	18	17	13	21.33333333	16	-0.338314956	0.607811901	0.972790461
15460	'Hr'	28	24	31	120	16	23	27.66666667	53	1.263191435	0.194555542	0.819426224
15461	'Hras'	466	447	670	271	422	629	527.6666667	440.6666667	-0.302145458	0.476417315	0.972790461
15463	'Agfg1'	2127	1925	2533	3155	1763	2405	2195	2441	0.281344435	0.582466864	0.972790461
15464	'Hrc'	18	10	11	14	8	2	13	8	-0.468630312	0.641335328	0.972790461
15465	'Hrh1'	0	1	16	12	1	4	5.666666667	5.666666667	0.112004428	0.950696874	0.998065566
15466	'Hrh2'	0	1	0	1	1	2	0.333333333	1.333333333	1.921402722	0.452416357	0.970649024
15467	'Eif2ak1'	964	949	592	803	988	1227	835	1006	0.336053419	0.244918141	0.866091613
15468	'Prmt2'	670	724	1358	640	983	953	917.3333333	858.6666667	-0.124637761	0.796539661	0.972790461
15469	'Prmt1'	2731	3240	4351	1021	3039	3330	3440.666667	2463.333333	-0.569883454	0.229839581	0.856721174
15473	'Rida'	294	299	163	177	337	465	252	326.3333333	0.38544192	0.307272689	0.907830798
15476	'Hs3st1'	298	312	514	1279	381	744	374.6666667	801.3333333	1.263690293	0.067246291	0.579357341
15478	'Hs3st3a1'	601.16	613.73	406.06	106.25	455.96	650.55	540.3166667	404.2533333	-0.490715509	0.37091566	0.9384981
15481	'Hspa8'	28143	28396	25726	33767	30754	36296	27421.66667	33605.66667	0.383427927	0.259110261	0.877926692
15482	'Hspa1l'	51.03	58	22.12	49.04	62	30.07	43.71666667	47.03666667	0.254285786	0.671437116	0.972790461
15483	'Hsd11b1'	0	2	0	91	2	7	0.666666667	33.33333333	6.144142123	0.001145604	0.070943768
15484	'Hsd11b2'	61	75	2	33	167	83	46	94.33333333	1.087451441	0.280702138	0.889418676
15485	'Hsd17b1'	11	11	13	29	24	22	11.66666667	25	1.199850599	0.067247925	0.579357341
15486	'Hsd17b2'	43	65	22	2	33	47	43.33333333	27.33333333	-0.744695448	0.407563241	0.957157474
15487	'Hsd17b3'	3	8	0	1	2	1	3.666666667	1.333333333	-1.322391498	0.455943656	0.971249389
15488	'Hsd17b4'	1667	1610	972	1134	1684	2144	1416.333333	1654	0.266907248	0.363992646	0.93523525
15490	'Hsd17b7'	335	365	170	402	388	291	290	360.3333333	0.472153338	0.308130602	0.907963267
15492	'Hsd3b1'	832	1150	118	102	5593	4402	700	3365.666667	2.213422006	0.040666011	0.473739094
15493	'Hsd3b2'	1	1	0	1	0	0	0.666666667	0.333333333	-0.746871675	0.831393929	0.974324192
15494	'Hsd3b3'	6	2	1	0	1	0	3	0.333333333	-2.949227382	0.183635662	0.804729813
15496	'Hsd3b5'	0	0	0	0	1	2	0	1	2.297812331	0.566584047	0.972790461
15497	'Hsd3b6'	6	5	3	0	8	16	4.666666667	8	0.663704439	0.618641811	0.972790461
15499	'Hsf1'	657	673	547	568	656	578	625.6666667	600.6666667	0.027019425	0.932627711	0.993970337
15500	'Hsf2'	868	895	735	2117	780	1137	832.6666667	1344.666667	0.911224368	0.128754431	0.72577634
15502	'Dnaja1'	6369	6913	3834	6630	7575	8120	5705.333333	7441.666667	0.479414969	0.131302721	0.72897159
15504	'Dnajb3'	67	83	44	15	57	60	64.66666667	44	-0.582556382	0.281107095	0.889731741
15505	'Hsph1'	2305	2343	880	3048	2325	3834	1842.666667	3069	0.873990762	0.066192266	0.575421751
15507	'Hspb1'	409	463	892	883	473	945	588	767	0.436386643	0.468484237	0.972790461
15510	'Hspd1'	8407	8451	10821	4619	7144	10761	9226.333333	7508	-0.329802831	0.383534574	0.945630828
15511	'Hspa1b'	150.85	137.93	760.88	9957.2	232.32	3998.34	349.8866667	4729.283333	3.9396991	0.001009186	0.066185633
15512	'Hspa2'	300	316	437	501	255	360	351	372	0.212653866	0.704455626	0.972790461
15516	'Hsp90ab1'	43399	42540	81207	25746	40859	55861	55715.33333	40822	-0.50828866	0.290385926	0.898321851
15519	'Hsp90aa1'	24331	23451	19573	9270	16300	22539	22451.66667	16036.33333	-0.491461354	0.100995545	0.671759705
15525	'Hspa4'	5936	5943	4500	9466	5634	9367	5459.666667	8155.666667	0.714330989	0.104560957	0.67684333
15526	'Hspa9'	7717	7476	36919	9475	6383	13171	17370.66667	9676.333333	-0.897347284	0.257073245	0.8773831

15528	'Hspe1'	1419	1533	2448	1543	1246	1783	1800	1524	-0.198408186	0.680817955	0.972790461
15529	'Sdc2'	1284	1293	869	353	1722	1934	1148.666667	1336.333333	0.149980763	0.779121318	0.972790461
15530	'Hspg2'	6191	5349	5290	18416	7211	8311	5610	11312.66667	1.237455157	0.043697134	0.487255084
15531	'Ndst1'	2139	2048.1	789	1756.1	2631.2	2828	1658.703333	2405.106667	0.617001188	0.132105778	0.729631651
15547	'Trmt2a'	876.11	876.1	765.4	388.64	784.1	938.26	839.2033333	703.6666667	-0.268286534	0.382763072	0.945348183
15550	'Htr1a'	0	1	0	0	2	1	0.333333333	1	1.368205316	0.684632717	0.972790461
15551	'Htr1b'	33	33	75	39	104	77	47	73.33333333	0.560463357	0.365664202	0.936382025
15552	'Htr1d'	2	3	3	8.16	6	6	2.666666667	6.72	1.425394203	0.185207128	0.805572619
15557	'Htr1f'	3	3	21	13	2	17	9	10.66666667	0.222108089	0.860874526	0.976863639
15558	'Htr2a'	8	5	42	13	11	9	18.33333333	11	-0.755057969	0.478152777	0.972790461
15559	'Htr2b'	20	19	10	34	20	13	16.33333333	22.33333333	0.674026973	0.379869937	0.943232396
15561	'Htr3a'	0	0	0	1	1	0	0	0.666666667	2.050872943	0.610671515	0.972790461
15562	'Htr4'	7	4	0	2	14	9	3.666666667	8.333333333	1.199772934	0.373160532	0.93929675
15563	'Htr5a'	1	0	0	0	1	7	0.333333333	2.666666667	2.732017288	0.338318819	0.924608313
15564	'Htr5b'	5	1	11	0	1	1	5.666666667	0.666666667	-3.22518328	0.071705976	0.592919704
15565	'Htr6'	3	3	71	9	1	7	25.66666667	5.666666667	-2.184007517	0.137306019	0.738626463
15566	'Htr7'	34	22	5	1	13	12	20.33333333	8.666666667	-1.251262921	0.222087171	0.849517424
15567	'Slc6a4'	76	130	42	149	20	40	82.66666667	69.66666667	0.126034707	0.891260874	0.985096898
15568	'Elavl1'	2335	2350	2412	1759	2067	2010	2365.666667	1945.333333	-0.222881407	0.478426349	0.972790461
15569	'Elavl2'	1670	1711	303	38	749	1999	1228	928.6666667	-0.484561117	0.636565167	0.972790461
15571	'Elavl3'	117	111	20	7	37	5	82.66666667	16.33333333	-2.274628	0.014034946	0.293375506
15572	'Elavl4'	21	31	17	5	15	28	23	16	-0.563061211	0.424822103	0.960285372
15574	'Hus1'	669	676	488	183	575	506	611	421.3333333	-0.560920446	0.153096792	0.764400413
15586	'Hyal1'	101.96	130.33	38.58	156	137.24	111	90.29	134.7466667	0.770805556	0.175101116	0.793892984
15587	'Hyal2'	596	666	256	547	1077	922	506	848.6666667	0.806099905	0.047874875	0.507922499
15891	'Ibsp'	10	4	0	43	5	27	4.666666667	25	2.742119751	0.03473173	0.447068151
15893	'Ica1'	95	76	195	87	77	158	122	107.3333333	-0.219539191	0.719485413	0.972790461
15894	'Icam1'	226	248	1352	1118	206	407	608.6666667	577	0.068416248	0.945628349	0.996592403
15896	'Icam2'	155	161	29	2	280	193	115	158.3333333	0.396508598	0.730891849	0.972790461
15898	'Icam5'	6	4	4	21	3	6	4.666666667	10	1.398478905	0.228799497	0.855540033
15900	'Irf8'	162	135	102	606	96	99	133	267	1.377987408	0.12605797	0.72022881
15901	'Id1'	1012	1172	298	692	1388	2038	827.3333333	1372.666667	0.766597565	0.155689369	0.766830643
15902	'Id2'	540	555	862	402	882	824	652.3333333	702.6666667	0.054985291	0.899378741	0.986106073
15903	'Id3'	1175	1288	832	423	1768	2252	1098.333333	1481	0.36652066	0.478343818	0.972790461
15904	'Id4'	355	424	80	54	292	200	286.3333333	182	-0.646838	0.355556744	0.931809687
15925	'Ide'	2000	2048	1815	1283	2026	2855	1954.34	2054.666667	0.07128411	0.807425033	0.972790461
15926	'Idh1'	5907	6785	1774	26340	7045	12634	4822	15339.66667	1.980212413	0.007883416	0.214208031
15929	'Idh3g'	1241	1254	1738	341	1289	1571	1411	1067	-0.513085278	0.332314258	0.921648675
15930	'Ido1'	1	1	0	0	0	0	0.666666667	0	-1.703500596	0.673820112	0.972790461
15931	'Ids'	1072	1086	827	536	1130	1264	995	976.6666667	-0.031409549	0.916676431	0.990526893

15932	'Idua'	329	316	131	220	618	590.39	258.6666667	476.13	0.893549698	0.053026855	0.532847023
15936	'Ier2'	391	383	1335	577	521	624	703	574	-0.309777167	0.650012263	0.972790461
15937	'Ier3'	341	268	9857	12340	124	2425	3488.666667	4963	0.696516227	0.724008276	0.972790461
15939	'Ier5'	463	451	472	1515	451	384	462	783.3333333	1.042030186	0.163031011	0.774499146
15944	'Irgm1'	307	313	933	54	367	838	517.6666667	419.6666667	-0.536101978	0.538338971	0.972790461
15945	'Cxcl10'	82	94	2367	638	86	184	847.6666667	302.6666667	-1.365604482	0.300929899	0.904264146
15950	'Ifi203'	34	23	502.26	19	65	88	186.42	57.33333333	-1.963353774	0.086659571	0.638346283
15951	'Ifi204'	37.54	73	566.95	24.59	13	76.3	225.83	37.96333333	-2.76409464	0.011425693	0.264873743
15953	'Ifi47'	26	20	136	6	20	40	60.66666667	22	-1.690968493	0.08692735	0.640026928
15957	'Ifit1'	39	38	142.02	5	16	292	73.00666667	104.3333333	0.227497479	0.854417443	0.975734242
15958	'Ifit2'	284	263	231	49	228	226	259.3333333	167.6666667	-0.70015701	0.174440075	0.793220451
15959	'Ifit3'	44.58	43.86	229.65	7	101.43	277.91	106.03	128.78	-0.008961394	0.993551612	0.999493374
15965	'Ifna2'	0	0	0	1	0	0	0	0.333333333	1.020273531	0.802557913	0.972790461
15969	'Ifna6'	1	0	0	0	0	0	0.333333333	0	-0.903279821	0.824807108	0.972790461
15970	'Ifna7'	0	0	0	1	0	0	0	0.333333333	1.020273531	0.802557913	0.972790461
15974	'Ifnab'	1	0	0	0	0	0	0.333333333	0	-0.903279821	0.824807108	0.972790461
15975	'Ifnar1'	4224.1	4218	2334.4	2533.4	5018	4174	3592.18	3908.46	0.166109003	0.605182478	0.972790461
15976	'Ifnar2'	780	809	1553	1120	1052	1113	1047.333333	1095	0.09777766	0.85063213	0.975734242
15977	'Ifnb1'	0	0	1	0	0	0	0.333333333	0	-0.903279821	0.824807108	0.972790461
15979	'Ifngr1'	397	345	348	230	547	615	363.3333333	464	0.321079212	0.369955397	0.937712122
15980	'Ifngr2'	1283	1492	3046	1198	1598	1697	1940.333333	1497.666667	-0.401128072	0.44271199	0.969530743
15982	'Ifrd1'	843	759	5919	2740	758	1473	2507	1657	-0.525135414	0.595025645	0.972790461
15983	'Ifrd2'	323	297.85	400	172.77	281.99	335	340.2833333	263.2533333	-0.390645163	0.290628802	0.898595513
15985	'Cd79b'	2	3	0	5	5	8	1.666666667	6	1.95907399	0.143121357	0.749054337
16000	'Igf1'	2188	2202	83	515	2543	1346	1491	1468	0.027666559	0.976764575	0.999493374
16001	'Igf1r'	3319.7	2787.9	1261	3747.8	2847.1	3127	2456.2	3240.64	0.574328823	0.23572678	0.861636582
16002	'Igf2'	18262	17700	12954	4775	19924	12579	16305.33333	12426	-0.425600909	0.353348583	0.930050349
16004	'Igf2r'	5495	5108	1368	11733	5430	5877	3990.333333	7680	1.220964054	0.071882135	0.593667748
16005	'Igfals'	6	6	1	4	5	4	4.333333333	4.333333333	0.126213006	0.912190082	0.989773583
16006	'Igfbp1'	0	0	0	0	4	3	0	2.333333333	3.527222849	0.321191852	0.917442833
16007	'Ccn1'	220	232	1753	1263	299	652	735	738	0.087242803	0.93164414	0.993601701
16008	'Igfbp2'	2289	2130	761	3534	2575	2619	1726.666667	2909.333333	0.949787404	0.075307408	0.60481391
16009	'Igfbp3'	4026	3643	1482	98	4260	3391	3050.333333	2583	-0.330361766	0.727373269	0.972790461
16010	'Igfbp4'	4280	4310	2391	2072	4194	4332	3660.333333	3532.666667	-0.02519963	0.937746387	0.994698142
16011	'Igfbp5'	29056	28477	5684	222915	26552	46032	21072.33333	98499.66667	2.656551022	0.006155471	0.185170597
16012	'Igfbp6'	157	143	160	365	187	352	153.3333333	301.3333333	1.09349592	0.032575996	0.435537855
16068	'Il18bp'	148.21	151.83	85.29	54.13	212.01	114.17	128.4433333	126.77	-0.017882192	0.971871948	0.999493374
16069	'Jchain'	27	21	2	0	19	3	16.66666667	7.333333333	-1.202013846	0.406669787	0.957157474
16136	'Igll1'	0	1	0	0	1	0	0.333333333	0.333333333	0.058500858	0.988561293	0.999493374
16145	'Igtp'	135.51	130.02	111	93	179	363	125.51	211.6666667	0.711328642	0.130930766	0.728882802

16147	'Ihh'	7	10	5	1	5	4	7.333333333	3.333333333	-1.143838993	0.274506278	0.885762621
16149	'Cd74'	119	101	339	537	73	113	186.3333333	241	0.617114521	0.53344995	0.972790461
16150	'Ikbkb'	980	945	746	1497	1004	993	890.3333333	1164.666667	0.552936993	0.236410032	0.862272407
16151	'Ikbkg'	1298	1292	750	719	1178	1374	1113.333333	1090.333333	0.009467473	0.97459132	0.999493374
16153	'Il10'	4	0	1	14	2	1	1.666666667	5.666666667	2.160489621	0.22786237	0.854324922
16154	'Il10ra'	49	51	15	48	100	91	38.33333333	79.66666667	1.113229533	0.039266432	0.46244458
16155	'Il10rb'	622	655	939	431	996	1157	738.6666667	861.3333333	0.154265897	0.723985258	0.972790461
16156	'Il11'	7	7	18	2321	2	9	10.66666667	777.3333333	6.590666439	0.002797148	0.118703962
16157	'Il11ra1'	993.52	883.25	286.9	797.97	1801	1926.74	721.2233333	1508.563333	1.106138879	0.02580031	0.396536375
16158	'Il11ra2'	89.08	96.51	45.61	1.03	12.24	8.26	77.06666667	7.176666667	-3.467891614	8.54E-07	3.19E-04
16159	'Il12a'	1	3	0	2	1	1	1.333333333	1.333333333	0.239083901	0.904357927	0.988027405
16160	'Il12b'	0	1	6	0	0	1	2.333333333	0.333333333	-2.80898937	0.32535753	0.920269559
16161	'Il12rb1'	7	8	22	27	4	7	12.33333333	12.66666667	0.256071383	0.821021636	0.972790461
16162	'Il12rb2'	167	165.64	124.85	98.09	212.93	203.38	152.4966667	171.4666667	0.177450388	0.586773078	0.972790461
16163	'Il13'	0	2	0	0	0	0	0.666666667	0	-1.695595436	0.675304306	0.972790461
16164	'Il13ra1'	378	334	618	544	485	568	443.3333333	532.3333333	0.305776569	0.535794331	0.972790461
16165	'Il13ra2'	48	38	60	3	24	12	48.66666667	13	-1.99477738	0.006738764	0.194707262
16168	'Il15'	10	9	19	223	32	50	12.66666667	101.6666667	3.283800602	8.50E-04	0.060306819
16169	'Il15ra'	16	24	24	138	42	16	21.33333333	65.33333333	1.918672896	0.043805411	0.487890148
16170	'Il16'	252	253	25	48	216	330	176.6666667	198	0.159653486	0.850527572	0.975734242
16172	'Il17ra'	306	261	118	202	217	227	228.3333333	215.3333333	0.037180058	0.931775969	0.993601701
16173	'Il18'	270	332	61	13	139	111	221	87.66666667	-1.364781928	0.094318418	0.65877361
16174	'Il18rap'	1	1	2	69	10	6	1.333333333	28.33333333	4.755043163	6.34E-04	0.049568551
16175	'Il1a'	21	19	85	10	11	26	41.66666667	15.66666667	-1.529931624	0.083558125	0.627659848
16176	'Il1b'	10	15	11	201	6	8	12	71.66666667	3.03191897	0.02102491	0.360113137
16177	'Il1r1'	442	417	473	1947	664	744	444	1118.333333	1.567687472	0.0194307	0.347725165
16178	'Il1r2'	7	6	6	6	5	1	6.333333333	4	-0.499471117	0.657390948	0.972790461
16179	'Irak1'	1451	1471	1724	332	1886	1927	1548.656667	1381.666667	-0.278515896	0.62876331	0.972790461
16180	'Il1rap'	117	137	166	514	193	317	140	341.3333333	1.455216547	0.019930522	0.349283865
16181	'Il1rn'	17	20	235	245	9	60	90.66666667	104.6666667	0.390787956	0.771032742	0.972790461
16182	'Il18r1'	7	1	0	14	0	1	2.666666667	5	1.431528565	0.510356032	0.972790461
16183	'Il2'	3	1	0	0	3	1	1.333333333	1.333333333	-0.009804843	0.996674027	0.999616639
16184	'Il2ra'	0	2	1	5	3	0	1	2.666666667	1.675636896	0.435866819	0.96581102
16185	'Il2rb'	10	17	7	12	12	14	11.33333333	12.66666667	0.257017464	0.720698345	0.972790461
16186	'Il2rg'	31	31	20	4	41	24	27.33333333	23	-0.311112744	0.686511463	0.972790461
16188	'Il3ra'	85	79	100	111	102	171	88	128	0.572466418	0.185576889	0.805706284
16190	'Il4ra'	360	371	614	2785	742	1282	448.3333333	1603	2.03591512	0.005057659	0.16781708
16191	'Il5'	1	2	0	1	3	0	1	1.333333333	0.555475996	0.817228478	0.972790461
16192	'Il5ra'	1	0	0	2.99	0	0	0.333333333	0.996666667	1.337350669	0.73871782	0.972790461
16193	'Il6'	1	0	25	3	0	0	8.666666667	1	-2.907681023	0.244436095	0.866091613

16194	'Il6ra'	38	40	74	374	40	103	50.66666667	172.3333333	2.039331661	0.032069527	0.433742328
16195	'Il6st'	2401	2232	1739	4034	3240	3232	2124	3502	0.85987691	0.033730208	0.442872044
16196	'Il7'	1	1	15	1	3	2	5.666666667	2	-1.677051692	0.294838634	0.90029104
16197	'Il7r'	0	1	5	12	10	23	2	15	2.834215525	0.024222948	0.384734075
16199	'Il9r'	143	148	19	0	93	15	103.3333333	36	-1.557137897	0.244905329	0.866091613
16201	'Ilf3'	4781	4898	3221	1458	3977	3286	4300	2907	-0.567608543	0.106386996	0.679124905
16202	'Ilk'	2318	2364	2961	2705	2553	3485	2547.666667	2914.333333	0.238950027	0.534197172	0.972790461
16204	'Fabp6'	0	1	0	1	1	0	0.333333333	0.666666667	1.081692101	0.760081965	0.972790461
16205	'Gimap1'	33.04	61	4	1	86	70.01	32.68	52.33666667	0.632740444	0.607177019	0.972790461
16206	'Lrig1'	568	603	1104	937	627	831	758.3333333	798.3333333	0.142449449	0.797303395	0.972790461
16210	'Impact'	1082	1104	1786	1514	1196	1574	1324	1428	0.163756228	0.735762158	0.972790461
16211	'Kpnb1'	8229	8123	12316	6065	7455	8855	9556	7458.333333	-0.352403815	0.37904687	0.942578399
16319	'Incenp'	821	807	240	445	815	868	622.6666667	709.3333333	0.255200411	0.599851548	0.972790461
16322	'Inha'	1370	1417	469	6360	1281	2615	1085.333333	3418.666667	1.986480549	0.011009887	0.259702189
16323	'Inhba'	86	79	424	1357	142	119	196.3333333	539.3333333	1.724500779	0.139522696	0.741695575
16324	'Inhbb'	771	665	131	1187	1267	1492	522.3333333	1315.333333	1.479422199	0.015253386	0.307526371
16325	'Inhbc'	0	1	0	0	0	0	0.333333333	0	-0.903279821	0.824807108	0.972790461
16326	'Inhbe'	0	0	0	0	0	1	0	0.333333333	1.020273531	0.802557913	0.972790461
16328	'Cep250'	1459	1397	429	249	1787	988	1095	1008	-0.137406297	0.838282287	0.974723675
16329	'Inpp1'	354	394.95	475	439	465	409	407.9833333	437.6666667	0.168788472	0.682027593	0.972790461
16330	'Inpp5b'	551	585	470	670	713	818	535.3333333	733.6666667	0.530066662	0.076686989	0.609792246
16331	'Inpp5d'	106	94	15	19	107	175	71.66666667	100.3333333	0.457292766	0.587991488	0.972790461
16332	'Inppl1'	3855	3752	6793	4893	3293	4224	4800	4136.666667	-0.139424389	0.795716646	0.972790461
16334	'Ins2'	1	0	1	0	0	0	0.666666667	0	-1.858475606	0.644939423	0.972790461
16336	'Insl3'	0	2.82	0	0	0	0	0.94	0	-1.695595436	0.675304306	0.972790461
16337	'Insr'	1805	1656	1602	2597	1699	1836	1687.666667	2044	0.425053907	0.35916934	0.933706718
16341	'Eif3e'	5223	5280	8424	4051	4712	5052	6309	4605	-0.437386739	0.307414861	0.907830798
16348	'Invs'	873.9	872.45	457.65	389	907.13	815.91	734.6666667	704.0133333	-0.037290974	0.918176672	0.990727847
16351	'Ipp'	573.83	649.56	224.62	222.96	424.93	470.62	482.67	372.8366667	-0.323289019	0.463250065	0.972790461
16362	'Irf1'	404	418	1185	335	343	520	669	399.3333333	-0.789123063	0.205979786	0.833294914
16363	'Irf2'	265	278	581	597	324	369	374.6666667	430	0.304793478	0.636065437	0.972790461
16364	'Irf4'	0	2	2	0	2	2	1.333333333	1.333333333	-0.152536847	0.945767026	0.996592403
16365	'Acod1'	7	6	73	38	6	9	28.66666667	17.66666667	-0.550773345	0.66983256	0.972790461
16367	'Irs1'	1262	1249	585	797	1042	1029	1032	956	-0.01506615	0.966805054	0.999493374
16369	'Irs3'	3	7	12	35	1	68	7.333333333	34.66666667	2.244013928	0.07330516	0.599174173
16370	'Irs4'	95	83	14	6	31	15	64	17.33333333	-1.845810516	0.025887643	0.397046689
16371	'Irx1'	14	6	6	1	2	1	8.666666667	1.333333333	-2.643723894	0.031413563	0.430284336
16372	'Irx2'	120	121	75	24	53	54	105.3333333	43.66666667	-1.252200419	0.001744478	0.089880779
16373	'Irx3'	2339	2656	994	182	2089	2283	1996.333333	1518	-0.470029248	0.531806508	0.972790461
16391	'Irf9'	398	427	831	232	442	916	552	530	-0.176147766	0.764671505	0.972790461

16392	'Isl1'	12	12	2	5	16	6	8.666666667	9	0.129439069	0.896160728	0.985642009
16396	'Itch'	2771	2719	2623	2417	2766	2707	2704.333333	2630	0.028169586	0.92671419	0.992540506
16398	'Itga2'	212	223	211	288	225	147	215.3333333	220	0.186693611	0.720885734	0.972790461
16399	'Itga2b'	35	24	13	37	26	23	24	28.66666667	0.438141009	0.509867651	0.972790461
16400	'Itga3'	1174	1066	454	3112	1358	1816	898	2095.333333	1.468006227	0.015557463	0.309500711
16401	'Itga4'	49	47	8	65	79	65	34.66666667	69.66666667	1.1588667	0.087760406	0.642214018
16402	'Itga5'	1000	966	910	5025	1603	1687	958.6666667	2771.666667	1.795338656	0.009071715	0.233069516
16403	'Itga6'	7746	8299	949	3670	17220	13706	5664.666667	11532	1.04759469	0.169108288	0.784741705
16404	'Itga7'	32	29	117	516	41	86	59.33333333	214.3333333	2.117667777	0.056749706	0.547413448
16407	'Itgae'	26	24	3	2	8	6	17.66666667	5.333333333	-1.676054672	0.099137407	0.668798985
16408	'Itgal'	43	38	6	15	39	26	29	26.66666667	-0.044794353	0.953939445	0.999255249
16409	'Itgam'	35	48	5	22	32	60	29.33333333	38	0.445000597	0.577850692	0.972790461
16410	'Itgav'	1414	1562	1217	6367	2205	2949	1397.666667	3840.333333	1.698084208	0.006834354	0.195923816
16411	'Itgax'	11	15	25.99	3	17	26	17.33	15.33333333	-0.292594888	0.736644109	0.972790461
16412	'Itgb1'	6225	6141	6701	12447	8362	10870	6355.666667	10559.66667	0.847469148	0.055267086	0.539316735
16413	'Itgb1bp1'	355.63	333.12	427.83	260.62	325.43	422.1	372.1933333	336.05	-0.135710996	0.704442525	0.972790461
16414	'Itgb2'	17	31	15	19	40	73	21	44	1.046305201	0.088752257	0.64452174
16415	'Itgb2l'	0	2	0	0	4	2	0.666666667	2	1.55425938	0.580058705	0.972790461
16416	'Itgb3'	138	110	116	136	165	174	121.3333333	158.3333333	0.437278278	0.199084762	0.826058378
16418	'Eif6'	1220	1161	2711	853	941	1424	1697.333333	1072.666667	-0.698403868	0.200776818	0.828371216
16419	'Itgb5'	1888	2018	1034	3652	3168	3709	1646.666667	3509.666667	1.226390464	0.001876143	0.093866381
16420	'Itgb6'	0	2	5	7	0	1	2.333333333	2.666666667	0.459789003	0.831581227	0.974405544
16421	'Itgb7'	10	11	20	4	9	10	13.66666667	7.666666667	-0.890981685	0.274039762	0.88525018
16423	'Cd47'	457	384	1182	942	382	726	674.3333333	683.3333333	0.096743571	0.893630799	0.985171
16424	'Itih1'	2	2	0	1	0	0	1.333333333	0.333333333	-1.740850292	0.569351619	0.972790461
16425	'Itih2'	61	57	9	0	43	58	42.33333333	33.66666667	-0.402775568	0.749557875	0.972790461
16426	'Itih3'	1	0	1	0	1	3	0.666666667	1.333333333	0.825638239	0.744342789	0.972790461
16427	'Itih4'	9	2	5	56	4	15	5.333333333	25	2.561574993	0.034349954	0.445167916
16428	'Itk'	2	4	11	20	2	3	5.666666667	8.333333333	0.811832825	0.555255577	0.972790461
16429	'Itln1'	3	3	2	0	0	0	2.666666667	0	-3.780202362	0.131381076	0.72897159
16430	'Stt3a'	2560	2568	3336	2821	2826	5083	2821.333333	3576.666667	0.348789863	0.388140145	0.950023663
16431	'Itm2a'	1865	1778	437	3429	3246	2199	1360	2958	1.32426466	0.028886744	0.414174039
16432	'Itm2b'	3227	3221	2555	7282	5484	7953	3001	6906.333333	1.311719711	5.14E-04	0.044250111
16433	'Cuzd1'	1	0	1	3	1	0	0.666666667	1.333333333	1.260598586	0.612902094	0.972790461
16434	'Itpa'	754	841	529	400	798	931	708	709.6666667	0.011966892	0.969370681	0.999493374
16438	'Itpr1'	5052	4936	932	4279	6515	6515	3640	5769.666667	0.776139149	0.188206293	0.81021541
16439	'Itpr2'	1827	1659	416	872	2249	1602	1300.666667	1574.333333	0.338272183	0.548345063	0.972790461
16440	'Itpr3'	653	622	807	1254	806	899	694	986.3333333	0.631236757	0.20671142	0.834086989
16443	'Itsn1'	2613	2587	1516	3154	2831	2532	2238.666667	2839	0.489914761	0.231358092	0.858762213
16447	'Ivl'	1	0	3	13	0	3	1.333333333	5.333333333	2.248168115	0.274642294	0.885900797

16449	'Jag1'	278	241	282	476	289	308	267	357.6666667	0.570039029	0.267658455	0.883600995
16450	'Jag2'	909	786	433	184	914	1883	709.3333333	993.6666667	0.403892559	0.548737824	0.972790461
16451	'Jak1'	5021.7	5028.4	4243.5	4693.9	4492.5	4825.74	4764.52	4670.736667	0.067666855	0.83867254	0.974723675
16452	'Jak2'	1467	1464	8051	873	1389	1724	3660.666667	1328.666667	-1.59412281	0.044321757	0.490480355
16453	'Jak3'	172	144.18	390	129	147	156	235.3933333	144	-0.723741827	0.218872553	0.845429697
16456	'F11r'	1476	1599	2150	4302	2002	3027	1741.666667	3110.333333	0.975129913	0.085452989	0.634617076
16467	'Atcay'	1	1	3	1	2	1	1.666666667	1.333333333	-0.350987914	0.837210472	0.974723675
16468	'Jarid2'	2987	2801	2461	1968	1842	2221	2749.67	2010.333333	-0.363549504	0.267410004	0.883600995
16469	'Jrk'	353	376	94	94	310	322	274.3333333	242	-0.166083831	0.779697753	0.972790461
16470	'Ush1g'	8	2	1	6	3	15	3.666666667	8	1.198921042	0.329298651	0.921648675
16475	'Ajuba'	1223	1201	1579	521	1328	2275	1334.333333	1374.666667	-0.051621521	0.917701268	0.990547571
16476	'Jun'	1095	964	6337	6608	963	1562	2798.666667	3044.333333	0.309502535	0.771904951	0.972790461
16477	'Junb'	203	185	4657	2440	221	690	1681.666667	1117	-0.453459805	0.73258667	0.972790461
16478	'Jund'	1648	1571	6502	3856	2389	3418	3240.333333	3221	0.004172778	0.995584274	0.999562152
16480	'Jup'	682	659	1461	3394	1005	1299	934	1899.333333	1.211800199	0.114195571	0.699220241
16483	'Kap'	0	1	2	1	0	0	1	0.333333333	-1.521952424	0.635355647	0.972790461
16485	'Kcna1'	25	29	28	38	9	1	27.33333333	16	-0.420491927	0.719850543	0.972790461
16490	'Kcna2'	12	14	1	0	14	5	9	6.333333333	-0.53107901	0.717237401	0.972790461
16491	'Kcna3'	3	4	0	2	2	0	2.333333333	1.333333333	-0.536367484	0.798561449	0.972790461
16492	'Kcna4'	12	13	9	5	8	12	11.33333333	8.333333333	-0.426085939	0.566328389	0.972790461
16493	'Kcna5'	109	108	18	0	159	127	78.33333333	95.33333333	0.215336219	0.870606787	0.979986751
16494	'Kcna6'	138	173	71	2146	152	191	127.3333333	829.6666667	3.162609187	0.003122414	0.126036817
16495	'Kcna7'	10	18	17	31	7	11	15	16.33333333	0.362133682	0.69831665	0.972790461
16497	'Kcnab1'	265	287	34	80	299	300	195.3333333	226.3333333	0.239133211	0.750595428	0.972790461
16498	'Kcnab2'	55	61	19	295	64	92	45	150.3333333	2.089635074	0.013031779	0.285349599
16499	'Kcnab3'	54	77	50	269	109	68	60.33333333	148.6666667	1.578330047	0.033837671	0.443127738
16500	'Kcnb1'	527	402	715	128	396	301	548	275	-1.067998861	0.030037324	0.421372044
16502	'Kcnc1'	13	11	7	0	4	0	10.33333333	1.333333333	-2.973051086	0.044906519	0.493464388
16504	'Kcnc3'	171	114	88	177	64	51	124.3333333	97.33333333	-0.06591892	0.92879083	0.993096978
16506	'Kcnd1'	118	144	63	31	154	95	108.3333333	93.33333333	-0.23138619	0.68652107	0.972790461
16508	'Kcnd2'	695.4	691.79	260.99	170.44	830	631.21	549.3933333	543.8833333	-0.028923887	0.959586832	0.999493374
16509	'Kcne1'	1	0	1	3	0	5	0.666666667	2.666666667	2.048466294	0.358622422	0.933467324
16510	'Kcnh1'	314	315	60	31	723	739	229.6666667	497.6666667	1.05479688	0.26276722	0.88013169
16511	'Kcnh2'	871	893	308	1240	1577	1180	690.6666667	1332.333333	1.083509803	0.02136599	0.362154416
16512	'Kcnh3'	212	194	6	109	187	108	137.3333333	134.6666667	0.130381442	0.891939024	0.985171
16513	'Kcnj10'	21	24	0	57	10	10	15	25.66666667	1.220587836	0.377184691	0.941504733
16514	'Kcnj11'	10	8	54	14	7	12	24	11	-1.130108498	0.277769499	0.889355605
16515	'Kcnj12'	26	38	4	2	20	7	22.66666667	9.666666667	-1.209645022	0.25078413	0.871167957
16516	'Kcnj15'	1	2	1	6	3	2	1.333333333	3.666666667	1.667240863	0.257234561	0.877405867
16517	'Kcnj16'	1	1	6	102	0	14	2.666666667	38.66666667	4.153634083	0.01532662	0.307843112

16518	'Kcnj2'	194	152	62	534	208	160	136	300.6666667	1.45747749	0.050088278	0.518952771
16519	'Kcnj3'	90	99	21	14	175	55	70	81.33333333	0.200799158	0.818331421	0.972790461
16520	'Kcnj4'	13	13	52	34	9	11	26	18	-0.37980029	0.712561674	0.972790461
16521	'Kcnj5'	1	4	6	1	2	4	3.666666667	2.333333333	-0.739398144	0.596844629	0.972790461
16522	'Kcnj6'	11	8	1	2	6	4	6.666666667	4	-0.667189896	0.572435843	0.972790461
16523	'Kcnj8'	147	131	40	60	258	137	106	151.6666667	0.538054868	0.390694892	0.952178913
16524	'Kcnj9'	1	1	0	3	1	0	0.666666667	1.333333333	1.381334915	0.579914507	0.972790461
16525	'Kcnk1'	95	100	24	54	108	155	73	105.6666667	0.574131433	0.333702044	0.921648675
16526	'Kcnk2'	337	379	175	251	291	295	297	279	0.015899621	0.966509957	0.999493374
16527	'Kcnk3'	257	235	16	1659	393	241	169.3333333	764.3333333	2.612078497	0.013982513	0.293124205
16528	'Kcnk4'	10	5	8	10	1	3	7.666666667	4.666666667	-0.453990828	0.709084053	0.972790461
16529	'Kcnk5'	311	372	107	672	478	441	263.3333333	530.3333333	1.222988691	0.034438761	0.445410735
16530	'Kcnk7'	0	0	0	2	0	0	0	0.666666667	2.299096387	0.566358544	0.972790461
16531	'Kcnma1'	369	340	303	14	278	212	337.3333333	168	-1.129136966	0.156900594	0.768337387
16532	'Kcnu1'	12	12	7	6	18	9	10.33333333	11	0.11750538	0.878943921	0.981583754
16533	'Kcnmb1'	3	2	5	1	2	3	3.333333333	2	-0.79311064	0.559184596	0.972790461
16534	'Kcnn4'	11	6	9	8	16	7	8.666666667	10.33333333	0.289816895	0.724628177	0.972790461
16535	'Kcnq1'	180	169	54	117	328	337	134.3333333	260.6666667	0.977620694	0.066968295	0.57823282
16536	'Kcnq2'	77	66	8	0	17	10	50.33333333	9	-2.514646844	0.030857111	0.426950382
16538	'Kcns1'	23	23	1	2	33	38	15.66666667	24.33333333	0.604148826	0.612189366	0.972790461
16539	'Kcns2'	2	5	0	0	3	0	2.333333333	1	-1.199353354	0.657277539	0.972790461
16541	'Napsa'	0	1	2	0	2	2	1	1.333333333	0.218673443	0.926038439	0.992381554
16542	'Kdr'	1867	1644	161	275	2279	1406	1224	1320	0.110674377	0.898976783	0.986106073
16543	'Mdfic'	1620	1866	2598	2836	1516	1943	2028	2098.333333	0.176934134	0.751373024	0.972790461
16545	'Kera'	2	2	0	0	4	0	1.333333333	1.333333333	0.004485678	0.998745477	0.999900097
16548	'Khk'	256	242	188	223	386	420	228.6666667	343	0.606546288	0.027059969	0.403710299
16549	'Khsrp'	3157	3161	4322	2151	2523	2606	3546.666667	2426.666667	-0.5155717	0.177118788	0.79682499
16551	'Kif11'	1058	1111	295	99	931	761	821.3333333	597	-0.500962703	0.4982246	0.972790461
16552	'Kif12'	0	2	0	0	1	0	0.666666667	0.333333333	-0.733814424	0.856146772	0.975734242
16553	'Kif13a'	1013	1006	906	242	1107	927	975	758.6666667	-0.431019806	0.37714049	0.941504733
16554	'Kif13b'	1777	1670	606	2025	1392	1264	1351	1560.333333	0.423054453	0.449248454	0.97049895
16558	'Kif16b'	1571	1598	620	478	1818	2522	1263	1606	0.314879458	0.577643194	0.972790461
16559	'Kif17'	79	69	64	40	87	113	70.66666667	80	0.158170406	0.703338832	0.972790461
16560	'Kif1a'	839	746	906	2867	786	928	830.3333333	1527	1.138374255	0.117238598	0.70525123
16561	'Kif1b'	3814	3802	2624	4574	3954	4025	3413.333333	4184.333333	0.425019415	0.262719697	0.88013169
16562	'Kif1c'	2178	2164	2234	1609	2545	2879	2192	2344.333333	0.103358978	0.707078042	0.972790461
16563	'Kif2a'	2185.4	2236.6	1648.3	1012.6	1650.3	1714	2023.42	1458.97	-0.43881651	0.057525721	0.548760163
16564	'Kif21a'	330	303	409	560	277	455	347.3333333	430.6666667	0.435092769	0.421815676	0.959445304
16565	'Kif21b'	450	356	3227	880	277	505	1344.333333	554	-1.224506273	0.218175686	0.844387594
16568	'Kif3a'	2193	2090	1124	1181	2048	1851	1802.333333	1693.333333	-0.032229343	0.920247814	0.990979973

16569	'Kif3b'	1274	1246	765	1341	1187	1212	1095	1246.666667	0.321988552	0.405741048	0.957157474
16570	'Kif3c'	779	765	877	1195	771	718	807	894.6666667	0.297032083	0.561145559	0.972790461
16571	'Kif4'	569.03	667.03	211.02	85.36	544.83	964.79	482.36	531.66	0.074014653	0.91948738	0.990815958
16572	'Kif5a'	110	111	81	24	108	40	100.6666667	57.33333333	-0.825527859	0.153941321	0.765411885
16573	'Kif5b'	3815	3928	4307	5309	5593	6149	4016.666667	5683.666667	0.55989178	0.099904738	0.670873716
16574	'Kif5c'	318	254	459	948	340	493	343.6666667	593.6666667	0.960833502	0.151378335	0.75995796
16576	'Kif7'	352	428	304	244	473	371	361.3333333	362.6666667	0.034739981	0.908969329	0.989086818
16578	'Kif9'	280	305	53	38	179	285	212.6666667	167.3333333	-0.367351053	0.63045457	0.972790461
16579	'Kifap3'	1577	1374	1033	545	1447	1913	1328	1301.666667	-0.063001351	0.877026801	0.981341203
16580	'Kifc5b'	221.48	224.16	118.58	86.29	138.61	106.95	188.0733333	110.6166667	-0.694243065	0.065326657	0.574020335
16581	'Kifc2'	453.6	416.15	85.29	359	527.1	290.81	318.3466667	392.3033333	0.458044224	0.469448164	0.972790461
16582	'Kifc3'	545	561	237	2224	574	1264	447.6666667	1354	1.869946763	0.006918036	0.196928988
16588	'Kin'	265	299	463	342	369	300	342.3333333	337	0.026316809	0.956858128	0.999493374
16589	'Uhmk1'	2133	2168	1571	1906	1981	2136	1957.333333	2007.666667	0.1302588	0.672305652	0.972790461
16590	'Kit'	1630	1492	877	96	2467	4808	1333	2457	0.755114029	0.419567749	0.958308933
16591	'Kl'	7	4	7	12	17	14	6	14.33333333	1.291651551	0.095721752	0.662411648
16592	'Fabp5'	744	680	3716	1518	590	1183	1713.333333	1097	-0.609186859	0.481693021	0.972790461
16593	'Klc1'	1637	1587.5	1537.9	2452.2	1547	1608.46	1587.463333	1869.236667	0.391962721	0.416287605	0.95722888
16594	'Klc2'	1270	1333	402	705	966	1184	1001.666667	951.6666667	0.017177273	0.970828417	0.999493374
16596	'Klf1'	16	36	13	10	26	9	21.66666667	15	-0.465795985	0.540536259	0.972790461
16597	'Klf12'	343	352	649	293	480	533	448	435.3333333	-0.085600809	0.85699087	0.976149994
16598	'Klf2'	116	103	508	367	146	160	242.3333333	224.3333333	-0.011898532	0.989258571	0.999493374
16599	'Klf3'	1077	1072	1881	2399	907	1385	1343.333333	1563.666667	0.371174257	0.570936074	0.972790461
16600	'Klf4'	244	200	2908	910	131	420	1117.333333	487	-1.108551556	0.342528516	0.925308103
16601	'Klf9'	272	220	1947	1186	267	559	813	670.6666667	-0.185470925	0.857339039	0.976287979
16612	'Klk1'	17	45	1	4	28	34	21	22	0.068279811	0.953176984	0.999032683
16617	'Klk1b24'	0	1	0	0	0	0	0.333333333	0	-0.903279821	0.824807108	0.972790461
16621	'Klkb1'	13	8	10	4	12	8	10.33333333	8	-0.377596769	0.638896104	0.972790461
16625	'Serpina3c'	4	3	1	10	4	8	2.666666667	7.333333333	1.644964181	0.150998873	0.759755159
16633	'Klra2'	0	2	0	4	4	0	0.666666667	2.666666667	2.286960476	0.379619597	0.943151111
16635	'Klra4'	0	0	0	1	0	1	0	0.666666667	2.022653929	0.615796212	0.972790461
16643	'Klrd1'	0	0	0	1	2	0	0	1	2.548734582	0.523314857	0.972790461
16644	'Kng1'	0	1	1	7	2	4	0.666666667	4.333333333	2.869900023	0.098311552	0.666744224
16646	'Kpna1'	1488	1647	1415	1922	1631	1815	1516.666667	1789.333333	0.345457587	0.355519943	0.931809687
16647	'Kpna2'	1985.8	2005.5	671	960	1720	1978	1554.076667	1552.666667	0.057740323	0.898059624	0.985794067
16648	'Kpna3'	1922	1967	3363	915	1686	1691	2417.333333	1430.666667	-0.802160105	0.07198321	0.593728759
16649	'Kpna4'	2155	2204	1652	1643	2162	2522	2003.666667	2109	0.124407387	0.602157508	0.972790461
16650	'Kpna6'	2174	2270	1313	1474	2224	2626	1919	2108	0.183397423	0.518578677	0.972790461
16651	'Sspn'	572	530	137	74	1064	756	413	631.3333333	0.563820515	0.49145895	0.972790461
16653	'Kras'	1388.1	1419	1838.5	1674.3	1499.3	1663.36	1548.516667	1612.29	0.127803492	0.759336043	0.972790461

16656	'Hivep3'	110	113	101	131	78	69	108	92.66666667	-0.04442828	0.936759544	0.994413066
16658	'Mafb'	299	302	396	487	439	424	332.3333333	450	0.517388857	0.242299842	0.86449675
16660	'Krt31'	2	2	0	0	0	0	1.333333333	0	-2.696289772	0.497898105	0.972790461
16661	'Krt10'	157	163	106	48	120	77	142	81.66666667	-0.773413541	0.054040117	0.534315058
16663	'Krt13'	0	0	0	0	1	0	0	0.333333333	1.020273531	0.802557913	0.972790461
16664	'Krt14'	47	55	26	3	43	30	42.66666667	25.33333333	-0.81775452	0.309933808	0.910876728
16665	'Krt15'	25	20	7	0	7	14	17.33333333	7	-1.381697599	0.223949797	0.850759465
16666	'Krt16'	1	0	1	1	4	1	0.666666667	2	1.549849627	0.449226402	0.97049895
16667	'Krt17'	23	11	46	5	9	19	26.66666667	11	-1.396036511	0.107954658	0.684280429
16668	'Krt18'	780	705	3964	2747	813	2340	1816.333333	1966.666667	0.14784507	0.869067171	0.979633196
16669	'Krt19'	1465	1527	10442	1101	1066	2033	4478	1400	-1.797268639	0.038851249	0.461389364
16671	'Krt33b'	0	0	0	0	1	1	0	0.666666667	1.775692139	0.660844521	0.972790461
16673	'Krt36'	0	1	0	0	1	0	0.333333333	0.333333333	0.058500858	0.988561293	0.999493374
16675	'Krt27'	0	1	0	0	0	0	0.333333333	0	-0.903279821	0.824807108	0.972790461
16678	'Krt1'	16	15	12	0	31	19	14.33333333	16.66666667	0.09543021	0.934961339	0.994413066
16680	'Krt84'	0	1	0	0	0	1	0.333333333	0.333333333	0.058500858	0.988561293	0.999493374
16682	'Krt4'	0	0	2	0	0	1	0.666666667	0.333333333	-1.033504517	0.79720072	0.972790461
16687	'Krt6a'	0	3	0	0	0	1	1	0.333333333	-1.313730691	0.742696629	0.972790461
16688	'Krt6b'	0	0	2	0	2	0	0.666666667	0.666666667	-0.252288975	0.949539803	0.997836337
16691	'Krt8'	1397	1309	3912	1025	1543	3012	2206	1860	-0.364706062	0.5716354	0.972790461
16706	'Ksr1'	557	586	780	2942	538	593	641	1357.666667	1.385798934	0.105275243	0.678099427
16709	'Ktn1'	2611	2608	2363	3541	3245	3839	2527.333333	3541.666667	0.575846479	0.089292259	0.645730097
16716	'Ky'	33	57	51	27	91	129	47	82.33333333	0.724894703	0.218394085	0.84443936
16728	'L1cam'	21	24	6	11	18	10	17	13	-0.271562308	0.722413665	0.972790461
16763	'Lad1'	63	69	25	69	77	113	52.33333333	86.33333333	0.809604434	0.095849697	0.662411648
16764	'Aff3'	1398	1336	208	34	1344	1065	980.6666667	814.3333333	-0.323813866	0.749958566	0.972790461
16765	'Stmn1'	2516	2683	3256	950	2430	2845	2818.333333	2075	-0.507350802	0.22214653	0.849517424
16768	'Lag3'	32	19	4	14	31	28	18.33333333	24.33333333	0.477767323	0.545136265	0.972790461
16769	'Dsg4'	0	1	0	0	0	0	0.333333333	0	-0.903279821	0.824807108	0.972790461
16770	'Lalba'	0	0	0	0	0	1	0	0.333333333	1.020273531	0.802557913	0.972790461
16772	'Lama1'	2276	2140	869	456	3814	3151	1761.666667	2473.666667	0.436535192	0.515190462	0.972790461
16773	'Lama2'	636	628	49	189	604	739	437.6666667	510.6666667	0.257457133	0.753358955	0.972790461
16774	'Lama3'	157	155	79	144	633	535	130.3333333	437.3333333	1.717079737	9.61E-04	0.06437552
16775	'Lama4'	1193	1058	863	451	1218	1084	1038	917.6666667	-0.193627001	0.579849206	0.972790461
16776	'Lama5'	1265	1046	968	8243	1807	2330	1093	4126.666667	2.223360487	0.003733759	0.140118993
16777	'Lamb1'	5054	4443	2483	4337	6051	7263	3993.333333	5883.666667	0.622750983	0.053355619	0.533767484
16779	'Lamb2'	2456	2286	1105	3833	3377	2894	1949	3368	0.955743349	0.036707219	0.457624685
16780	'Lamb3'	20	20	16	68	15	14	18.66666667	32.33333333	1.111892672	0.230540504	0.857569177
16782	'Lamc2'	161	126	186	1307	189	375	157.6666667	623.6666667	2.274169391	0.007645089	0.21034504
16783	'Lamp1'	5979	5767	5279	5660	6975	10072	5675	7569	0.443577782	0.113357964	0.698948431

16784	'Lamp2'	6111	6139	6576	15410	7351	9682	6275.333333	10814.33333	0.955030961	0.078101356	0.614954936
16785	'Rpsa'	23680	23562	45139	22936	19639	24342	30793.66667	22305.66667	-0.430287241	0.402094008	0.955996639
16790	'Anpep'	993	1047	469	366	1473	1377	836.3333333	1072	0.335950432	0.506830088	0.972790461
16792	'Laptm5'	48	59	15	53	93	202	40.66666667	116	1.527663427	0.015502313	0.309500711
16795	'Large1'	855	776	580	598	1061	1002	737	887	0.29970545	0.249109555	0.869642097
16796	'Lasp1'	3125	3131	3934	3560	3868	4783	3396.666667	4070.333333	0.295030806	0.412032277	0.95722888
16797	'Lat'	3	4	2	2	12	1	3	5	0.748577579	0.58326952	0.972790461
16798	'Lats1'	2041	2067	2847	1796	2111	2064	2318.333333	1990.333333	-0.184846451	0.636424341	0.972790461
16800	'Arhgef2'	1782	1814	7236	1082	2014	2126	3610.666667	1740.666667	-1.170311652	0.097195543	0.663789939
16801	'Arhgef1'	1373	1430	987	1238	1678	1272	1263.333333	1396	0.229754817	0.469165368	0.972790461
16803	'Lbp'	116	101	307	645	69	117	174.6666667	277	0.937196974	0.358238176	0.933245301
16814	'Lbx1'	0	2	0	0	2	1	0.666666667	1	0.569767569	0.858446798	0.976475731
16815	'Lbx2'	186	213	42	13	260	204	147	159	0.061278002	0.946123362	0.996592403
16816	'Lcat'	28	19	37	22	16	17	28	18.33333333	-0.533208622	0.427733803	0.961947592
16818	'Lck'	63	82	204	17	59	68	116.3333333	48	-1.426530451	0.046032234	0.500666378
16819	'Lcn2'	1	17	1139	312	21	56	385.6666667	129.6666667	-1.410984661	0.522528861	0.972790461
16820	'Lcn3'	3	2	0	0	2	1	1.666666667	1	-0.734458786	0.748652517	0.972790461
16822	'Lcp2'	28	32	14	14	21	44	24.66666667	26.33333333	0.111284729	0.856911517	0.976149994
16825	'Ldb1'	2693	2690	2795	1383	3226	2719	2726	2442.666667	-0.177691821	0.589993598	0.972790461
16826	'Ldb2'	590	605	298	56	742	649	497.6666667	482.3333333	-0.128787297	0.863015704	0.977227788
16828	'Ldha'	12362	11030	64066	15534	7647	17399	29152.66667	13526.66667	-1.130470041	0.182179032	0.802871523
16832	'Ldhb'	4598	5123	2479	1216	5698	5777	4066.666667	4230.333333	0.013679303	0.979521244	0.999493374
16833	'Ldhc'	26	25	4	1	11	28	18.33333333	13.33333333	-0.5128652	0.643889423	0.972790461
16834	'Cog1'	400	503.37	272	207.11	636.43	502.94	391.79	448.8266667	0.19087413	0.642480794	0.972790461
16835	'Ldlr'	349	357	207	608	485	299	304.3333333	464	0.801001096	0.129845831	0.727578716
16840	'Cnmd'	6	6	0	16	14	14	4	14.66666667	2.057894328	0.053735812	0.533926597
16841	'Lect2'	0	0	0	1	0	0	0	0.333333333	1.020273531	0.802557913	0.972790461
16842	'Lef1'	381	358	100	34	229	208	279.6666667	157	-0.860691265	0.21775794	0.844165262
16847	'Lepr'	66	62	78	128	75.47	54	68.66666667	85.82333333	0.492747193	0.437341289	0.96622803
16848	'Lfng'	44	56	18	29	60	54	39.33333333	47.66666667	0.33154368	0.535094625	0.972790461
16852	'Lgals1'	2236	2684	5898	2747	2851	3739	3606	3112.333333	-0.230247654	0.681812579	0.972790461
16854	'Lgals3'	76	76	1399	493	247	919	517	553	-0.042042266	0.970498782	0.999493374
16855	'Lgals4'	82	85	39	21	134	62	68.66666667	72.33333333	0.053578665	0.934650873	0.994413066
16858	'Lgals7'	1160	1367	1458	302	1070	1319	1328.333333	897	-0.656284183	0.181417317	0.802050906
16859	'Lgals9'	308	332	334	480	222	722	324.6666667	474.6666667	0.619193714	0.25246972	0.871252558
16865	'Eif2d'	820.67	824.58	586.15	481.19	794.7	879.11	743.8	718.3333333	-0.020394245	0.93534022	0.994413066
16866	'Lhb'	8	14	4	4	5	2	8.666666667	3.666666667	-1.10277013	0.288386784	0.895591234
16867	'Lhcgr'	56	79	2	1	406	142	45.66666667	183	1.972642239	0.164316807	0.777509578
16869	'Lhx1'	15	11	5	2	9	1	10.33333333	4	-1.315905618	0.242325255	0.86449675
16870	'Lhx2'	30	36	9	0	9	7	25	5.333333333	-2.265226545	0.030690596	0.425904823

16871	'Lhx3'	4	0	2	0	1	0	2	0.333333333	-2.434571106	0.395615892	0.954014376
16872	'Lhx4'	13	21	2	2	1	7	12	3.333333333	-1.773385412	0.147808968	0.755212067
16873	'Lhx5'	4	0	2	4	0	0	2	1.333333333	-0.158419748	0.953233134	0.999032683
16874	'Lhx6'	49	44	24	13	21	14	39	16	-1.206804959	0.029571189	0.419705596
16875	'Lhx8'	254	218	15	0	316	1000	162.3333333	438.6666667	1.35671955	0.518875522	0.972790461
16876	'Lhx9'	3034	2980	3850	1579	2586	2421	3288	2195.333333	-0.5835091	0.089130802	0.645388896
16878	'Lif'	7	6	147	11	8	15	53.33333333	11.33333333	-2.355543167	0.061658547	0.56365808
16880	'Lifr'	614	564	295	2686	711	957	491	1451.333333	1.87392574	0.009748683	0.242594181
16881	'Lig1'	2102.5	2020.2	830.2	511	1445	1772.06	1650.936667	1242.686667	-0.411765479	0.393228184	0.953141889
16882	'Lig3'	2463.7	2246.5	1063.8	712.73	1777	1973.15	1924.66	1487.61	-0.361551495	0.391666746	0.952648938
16885	'Limk1'	915	849	473	831	660	883	745.6666667	791.3333333	0.219101338	0.592402	0.972790461
16886	'Limk2'	809	866	1444	916	886	819	1039.666667	873.6666667	-0.199802576	0.685437822	0.972790461
16889	'Lipa'	543	520	382	1002	759	1120	481.6666667	960.3333333	1.10791849	0.004000542	0.145912662
16890	'Lipe'	284	289	130	1635	260	534	234.3333333	809.6666667	2.136614189	0.009311514	0.236988628
16891	'Lipg'	195	215	40	247	218	139	150	201.3333333	0.647343539	0.342440276	0.925308103
16897	'Llgl1'	1880	1948	2091	819	2315	2005	1973	1713	-0.248965712	0.516312663	0.972790461
16898	'Rps2'	14198	15612	32189	21648	15301	25827.13	20666.23333	20925.31667	0.041238226	0.942174977	0.996253096
16904	'Gzmm'	5	12	3	21	4	13	6.666666667	12.66666667	1.182950732	0.263647717	0.88013169
16905	'Lmna'	835	855	2664	2728	1176	2058	1451.333333	1987.333333	0.52195396	0.479145067	0.972790461
16906	'Lmnb1'	1857	1961	1352	1697	1775	1741	1723.333333	1737.666667	0.115049932	0.723467781	0.972790461
16907	'Lmnb2'	624	711	336	300	722	753	557	591.6666667	0.100193485	0.797732414	0.972790461
16909	'Lmo2'	345	348	35	10	492	227	242.6666667	243	-0.035404217	0.974077135	0.999493374
16911	'Lmo4'	302	306	4928	1378	427	836	1845.333333	880.3333333	-1.046105078	0.356514276	0.932066196
16912	'Psmb9'	57	43	35	3	21	40	45	21.33333333	-1.168827107	0.128889325	0.72577634
16913	'Psmb8'	37	20	135	31	14	58	64	34.33333333	-0.973209125	0.296020208	0.90029104
16917	'Lmx1b'	157	145	18	10	50	26	106.6666667	28.66666667	-1.854525063	0.026045508	0.397137149
16918	'Mycl'	288	257	271	528	241	272	272	347	0.546859715	0.349816925	0.92886351
16922	'Phyh'	492	564	331	101	639	701	462.3333333	480.3333333	-0.020938952	0.97226418	0.999493374
16923	'Sh2b3'	807.36	778.23	912.38	632.79	834.02	945.29	832.6566667	804.0333333	-0.029114065	0.926760944	0.992540506
16924	'Lnx1'	100.84	106.29	41.02	71.4	96.35	57.1	82.71666667	74.95	-0.011051061	0.983049404	0.999493374
16939	'Lor'	6	14	33	36	52	31	17.66666667	39.66666667	1.149117529	0.158338856	0.768890221
16948	'Lox'	577	534	1227	275	554	737	779.3333333	522	-0.675105362	0.220780597	0.849076011
16949	'Loxl1'	682	637	415	834	903	905	578	880.6666667	0.710364532	0.032183648	0.433954272
16950	'Loxl3'	207.87	189.2	25.94	107.08	229.61	222.55	141.0033333	186.4133333	0.486419865	0.487997493	0.972790461
16952	'Anxa1'	398	465	1738	587	552	584	867	574.3333333	-0.617449672	0.396162674	0.954259077
16956	'Lpl'	1345	1119	561	2402	1833	3067	1008.333333	2434	1.397429518	0.001680453	0.088782789
16969	'Zbtb7a'	652	656	1147	1154	711	957	818.3333333	940.6666667	0.286923683	0.603806981	0.972790461
16970	'Lrmp'	70	69	36	119	29	27	58.33333333	58.33333333	0.339231328	0.688933485	0.972790461
16971	'Lrp1'	6168	5314	2275	11409	8542	7681	4585.666667	9210.666667	1.202969659	0.020571268	0.356011446
16973	'Lrp5'	1089	1090	1229	2299	1407	2341	1136	2015.666667	0.928147491	0.042906723	0.483384062

16974	'Lrp6'	2971	2812	1848	10181	3297	3964	2543.666667	5814	1.468933461	0.027279606	0.404239957
16975	'Lrp8'	55	51	54	77	98	81	53.33333333	85.33333333	0.737725894	0.070269194	0.589465353
16976	'Lrpap1'	1211	1202	602	725	1590	1756	1005	1357	0.451866958	0.226345738	0.853679947
16977	'Lrrc23'	3	11	12	1	8	11	8.666666667	6.666666667	-0.509875262	0.644441967	0.972790461
16978	'Lrrfip1'	608	689	2106	3244	1322	1883	1134.333333	2149.666667	1.01975172	0.183800693	0.804729813
16979	'Lrrn1'	3236	3154	380	717	5018	3489	2256.666667	3074.666667	0.448229724	0.579684	0.972790461
16980	'Lrrn2'	87	69	51	81	87	134	69	100.6666667	0.60417151	0.127407472	0.723768792
16981	'Lrrn3'	163	139	40	38	153	85	114	92	-0.280211747	0.660988687	0.972790461
16985	'Lsp1'	1101	1119	434	175	1170	568	884.6666667	637.6666667	-0.492839365	0.435168511	0.965731729
16987	'Lss'	683	732	705	942	603	566	706.6666667	703.6666667	0.152855964	0.760381291	0.972790461
16988	'Lst1'	9	8	3	0	8	6	6.666666667	4.666666667	-0.574158779	0.652942233	0.972790461
16992	'Lta'	3	8	5	6	3	1	5.333333333	3.333333333	-0.464906337	0.709398059	0.972790461
16993	'Lta4h'	1061	1116	1450	963	1245	1153	1209	1120.333333	-0.080574897	0.829446939	0.973678583
16994	'Ltb'	7	9	37	0	6	6	17.66666667	4	-2.372925913	0.060135545	0.556619755
16995	'Ltb4r1'	1	1	5	1	3	0	2.333333333	1.333333333	-0.86638622	0.650508724	0.972790461
16997	'Ltbp2'	351	257	1437	218	439	365	681.6666667	340.6666667	-1.121685069	0.143084364	0.749054337
16998	'Ltbp3'	2620	2601	1131	1710	5283	3241	2117.333333	3411.333333	0.714638138	0.121350829	0.712603747
17000	'Ltbr'	626	695	687	777	924	1044	669.3333333	915	0.495429718	0.104728718	0.67684333
17001	'Ltc4s'	0	0	0	3	0	0	0	1	2.899270347	0.465759809	0.972790461
17002	'Ltf'	0	32	0	220	106	45	10.66666667	123.6666667	3.913098846	0.012977098	0.284896741
17005	'Ltk'	51	40	16	2	34	28	35.66666667	21.33333333	-0.799012913	0.366705923	0.93669394
17022	'Lum'	446	412	11	75	712	505	289.6666667	430.6666667	0.574558539	0.588200847	0.972790461
17025	'Alad'	4199	4243	1295	1007	2543	3194	3245.666667	2248	-0.510585702	0.326952471	0.920734892
17035	'Lxn'	323	343	454	95	429	493	373.3333333	339	-0.254766871	0.65146659	0.972790461
170439	'Elovl6'	592	647	217	406	713	490	485.3333333	536.3333333	0.240440792	0.603757355	0.972790461
170441	'Slc2a10'	76	77	40	111	75	118	64.33333333	101.3333333	0.792562547	0.107158606	0.682129732
170442	'Bbox1'	0	0	0	1	0	0	0	0.333333333	1.020273531	0.802557913	0.972790461
170458	'Gpha2'	8	17	0	31	10	7	8.333333333	16	1.326147877	0.321136654	0.917442833
170459	'Stard4'	715	750	386	664	659	530	617	617.6666667	0.146906686	0.728183716	0.972790461
170460	'Stard5'	420	451	1108	382	377	544	659.6666667	434.3333333	-0.627487544	0.285697636	0.893820325
170461	'Stard6'	171	220	32	2	135	114	141	83.66666667	-0.812690407	0.454589675	0.970649024
170472	'Recql5'	788	795	215	203	713	614	599.3333333	510	-0.215234509	0.708306516	0.972790461
170483	'Grin3b'	16	21	6	136	21	48	14.33333333	68.33333333	2.60073171	0.005393307	0.173498385
170484	'Nphs2'	42	43	2	14	102	38	29	51.33333333	0.856136914	0.403831752	0.956478046
17057	'Klrb1a'	5	2	1	6	2	3	2.666666667	3.666666667	0.69000268	0.610762986	0.972790461
170571	'Cntnap4'	13	16	3	3	12	5	10.66666667	6.666666667	-0.623832813	0.528616636	0.972790461
170574	'Sp7'	17	22	1	3	3	4	13.33333333	3.333333333	-1.851711357	0.119350937	0.709905563
17059	'Klrb1c'	2	3	0	1	2	0	1.666666667	1	-0.551912615	0.809632572	0.972790461
17060	'Blnk'	10	14	43	34	20	16	22.33333333	23.33333333	0.143275459	0.874080652	0.980171118
170625	'Snx18'	1329	1276	2292	2624	1393	1845	1632.333333	1954	0.370891801	0.527247049	0.972790461

17063	'Muc13'	7	3	8	15	5	4	6	8	0.623376827	0.574592467	0.972790461
170638	'Hpcal4'	4	5	9	3	2	0	6	1.666666667	-1.711110749	0.235208498	0.861636582
170639	'Olfr78'	0	0	2	4	0	1	0.666666667	1.666666667	1.490836033	0.604437897	0.972790461
17064	'Cd93'	885	885	119	15	1781	1200	629.6666667	998.6666667	0.608461606	0.597207911	0.972790461
170643	'Kirrel'	1674	1545	907.01	856	1713	2176	1375.336667	1581.666667	0.212698649	0.545791072	0.972790461
170644	'Ubn1'	4626	4214	2366	1249	2951	3325	3735.333333	2508.333333	-0.569474752	0.13046913	0.728053743
170648	'Olfr138'	0	0	0	0	0	1	0	0.333333333	1.020273531	0.802557913	0.972790461
17067	'Ly6c1'	2	1	0	1	8	7	1	5.333333333	2.393543511	0.14247725	0.748929878
170676	'Peg10'	7648	7414	3327	1585	9242	4874	6129.666667	5233.666667	-0.249487554	0.668397989	0.972790461
170677	'Cdhr1'	4	5	27	0	0	0	12	0	-6.108324536	0.001601673	0.085771147
17068	'Ly6d'	67	75	5	79	43	53	49	58.33333333	0.512031368	0.568964838	0.972790461
17069	'Ly6e'	939	934	407	2533	1125	1667	760	1775	1.45270007	0.012590753	0.278020211
170706	'Tmem37'	26	32	43	67	38	77	33.66666667	60.66666667	0.918357863	0.125623358	0.72022881
170707	'Usp48'	4071	3650	2534	5244	4647	4022	3418.333333	4637.666667	0.584284673	0.152979995	0.764176477
17071	'Ly6f'	0	0	0	0	1	0	0	0.333333333	1.020273531	0.802557913	0.972790461
170711	'Otud7a'	87	92	129	32	46	38	102.6666667	38.66666667	-1.393570805	0.00319964	0.12819988
170716	'Cyp4f13'	329	310	231	145	462	278	290	295	0.019418833	0.96295781	0.999493374
170718	'Idh3b'	1396	1424	1771	1065	1482	1746	1530.333333	1431	-0.092760228	0.783961546	0.972790461
170719	'Oxr1'	1671	1708	1589	2477	1699	2460	1656	2212	0.529136868	0.201073474	0.828703423
170720	'Card14'	10.53	7.4	152.44	1.22	8.39	1	56.79	3.536666667	-4.300261936	0.002202593	0.104951604
170721	'Papln'	1524.4	1479.3	68.9	2380.3	1858.8	1106.78	1024.17	1781.956667	1.073509247	0.24751079	0.868994456
170722	'Nxf7'	0	0	0	0	0	2	0	0.666666667	1.749449488	0.66566965	0.972790461
170725	'Capn8'	4	4	1	2	1	7	3	3.333333333	0.194421099	0.888646841	0.984710124
170728	'Rtn4ip1'	373	377	91	68	327	294	280.3333333	229.6666667	-0.291619224	0.656741387	0.972790461
170729	'Scrt1'	16	11	12	9	3	2	13	4.666666667	-1.252533397	0.216741407	0.843391934
170731	'Mfn2'	1781	1749	870	1063	1726	2017	1466.666667	1602	0.177307543	0.601265652	0.972790461
170732	'Trhr2'	1	1	1	0	3	0	1	1	-0.079660654	0.974922617	0.999493374
170734	'Zscan5b'	0	1	0	0	0	3	0.333333333	1	1.312655931	0.743478444	0.972790461
170735	'Arr3'	0	1	0	0	1	2	0.333333333	1	1.346096926	0.690173371	0.972790461
170736	'Parvb'	1463	1560	480	1888	2030	2451	1167.666667	2123	0.987746047	0.037038802	0.459648634
170737	'Znrf1'	1367	1419	1999	1854	1738	1524	1595	1705.333333	0.172178922	0.707163923	0.972790461
170738	'Kcnh7'	75.12	48.33	18.76	254.46	63.63	39.39	47.40333333	119.16	1.726910962	0.065476024	0.574448624
170740	'Zfp287'	292	282	177	46	245	156	250.3333333	149	-0.78725798	0.144750009	0.752114557
170741	'Pilrb1'	1	1	0	0	1	1	0.666666667	0.666666667	0.015019958	0.995726078	0.999562152
170742	'Sertad3'	284	227	277	206	271	350	262.6666667	275.6666667	0.085163941	0.800603778	0.972790461
170743	'Tlr7'	110.08	102.76	42.71	77.36	95.02	83.22	85.18333333	85.2	0.121009182	0.797291972	0.972790461
170744	'Tlr8'	2	4	0	11	8	21	2	13.33333333	2.841253734	0.014783597	0.303199291
170745	'Xpnpep2'	4	7	21	2	2	12	10.66666667	5.333333333	-1.162744467	0.331725012	0.921648675
170748	'Smco4'	303	321	365	88	258	342	329.6666667	229.3333333	-0.601101452	0.19946875	0.826749484
170749	'Mtmr4'	1604	1591	2364	812	1366	1965	1853	1381	-0.474058889	0.260281903	0.878537553

17075	'Epcam'	343	312	270	2211	368	698	308.3333333	1092.333333	2.136232599	0.007403479	0.206083364
170750	'Xpnpep1'	1213.1	1272.4	2084.7	1424.9	1221.2	1299.24	1523.383333	1315.11	-0.152362801	0.756803785	0.972790461
170752	'Bco2'	77	66	12	2	32	13	51.66666667	15.66666667	-1.731964289	0.072794505	0.596837647
170753	'Zfp704'	2423	2566	1076	1082	2293	2334	2021.666667	1903	-0.050862039	0.897545468	0.985794067
170755	'Sgk3'	301	313	98	168	384	443	237.3333333	331.6666667	0.516131189	0.302711241	0.905839913
170756	'Slc8b1'	103	103	70	1470	158	405	92	677.6666667	3.233277251	2.63E-04	0.027583361
170757	'Adgrl4'	50	38	17	0	100	95	35	65	0.790786177	0.526549407	0.972790461
170758	'Rac3'	117	99	207	28	115	85	141	76	-1.001585063	0.100354817	0.671759705
170759	'Atp13a1'	1021	1039	524	943	1413	1280	861.3333333	1212	0.571234905	0.086536995	0.637937705
17076	'Ly75'	119	94	16	134	131	43	76.33333333	102.6666667	0.676076588	0.411203686	0.95722888
170760	'Acbd3'	2012	1977	3567	2149	1433	2057	2518.666667	1879.666667	-0.357173064	0.504192626	0.972790461
170761	'Pdzd3'	2	2	0	0	4	0	1.333333333	1.333333333	0.004485678	0.998745477	0.999900097
170762	'Nup155'	1889	1872	722	939	1816	1523	1494.333333	1426	-0.001168354	0.997767247	0.999870909
170763	'Zfp87'	342	326	319	246	332	359	329	312.3333333	-0.038585821	0.891158256	0.985096898
170765	'Ripply3'	5	4	3	13	12	8	4	11	1.591365528	0.080481717	0.617481196
170767	'Rfxap'	468	469	1027	239	370	560	654.6666667	389.6666667	-0.824953914	0.128960758	0.725814662
170768	'Pfkfb3'	1120	836	9880	2649	433	985	3945.333333	1355.666667	-1.422544487	0.203028756	0.83089164
170770	'Bbc3'	110	91	1600	127	140	279	600.3333333	182	-1.89780997	0.077730006	0.613705515
170771	'Khdrbs2'	514.08	457.9	132.05	389.68	591.74	751.92	368.01	577.78	0.728755578	0.153440895	0.765299624
170772	'Glcci1'	834	832	450	458	696.39	561	705.3333333	571.7966667	-0.221719792	0.510746879	0.972790461
170776	'Cd209c'	232.39	234.12	57.93	142	185.11	96.25	174.8133333	141.12	-0.137809749	0.823782459	0.972790461
170779	'Cd209d'	1	0	0	0	0	0	0.333333333	0	-0.903279821	0.824807108	0.972790461
170780	'Cd209e'	1	0	0	0	0	0	0.333333333	0	-0.903279821	0.824807108	0.972790461
170787	'Hdac10'	124	125	133	39	223	122	127.3333333	128	-0.067885168	0.906355831	0.988823459
170788	'Crb1'	30.5	38.51	27.74	29.37	30.84	64.96	32.25	41.72333333	0.397576613	0.441194617	0.968551476
170789	'Acot8'	108.2	119.38	109.66	62.26	114	117.6	112.4133333	97.95333333	-0.192164327	0.571517233	0.972790461
17079	'Cd180'	14	9	3	0	17	50	8.666666667	22.33333333	1.261299766	0.350947236	0.92886351
170790	'Mlc1'	181	180	24	1	93	84	128.3333333	59.33333333	-1.170949321	0.30153428	0.90469437
170791	'Rbm39'	10233	10293	12037	6443	12880	8052	10854.33333	9125	-0.242110088	0.51764156	0.972790461
170799	'Rtkn2'	66	58	24	110	60	98	49.33333333	89.33333333	1.038427521	0.079952833	0.617305615
170812	'Ahsp'	25	54	8	6	55	1	29	20.66666667	-0.454461356	0.716925423	0.972790461
170813	'Ms4a3'	0	0	0	0	1	0	0	0.333333333	1.020273531	0.802557913	0.972790461
17082	'Il1rl1'	5	8	1	3	42	23	4.666666667	22.66666667	2.247433866	0.041285331	0.475995577
170822	'Usp33'	1706	1671	1969	1514	2158	2153	1782	1941.666667	0.144150265	0.6466543	0.972790461
170823	'Glmn'	393	433	420	128	426	303	415.3333333	285.6666667	-0.581761407	0.169720552	0.785344769
170826	'Ppargc1b'	194	147	126	277	126	119.66	155.6666667	174.22	0.39119736	0.530178175	0.972790461
170828	'Vgll1'	0	1	0	0	3	4	0.333333333	2.333333333	2.564885674	0.356329905	0.931977458
170829	'Tram2'	93	98	105	113	83	116	98.66666667	104	0.164421802	0.717918327	0.972790461
17083	'Tmed1'	407.88	460.7	238.21	209.41	651.99	557.75	368.93	473.05	0.353971154	0.402111758	0.955996639
170833	'Hook2'	277	273	396	588	368	452	315.3333333	469.3333333	0.682241574	0.191834411	0.813837363

170835	'Inpp5j'	13	17	18	69	7	24	16	33.33333333	1.341570586	0.176729212	0.796450095
17084	'Ly86'	7	10	2	0	15	19	6.333333333	11.33333333	0.762051008	0.56926104	0.972790461
17085	'Ly9'	7	7	11	14	20	37	8.333333333	23.66666667	1.470607669	0.038246805	0.459930654
17087	'Ly96'	20	33	56	26	26	49	36.33333333	33.66666667	-0.141213281	0.831501657	0.974390379
17089	'Lyar'	554	601	620	737	506	772	591.6666667	671.6666667	0.278339391	0.517338838	0.972790461
170930	'Sumo2'	3712.2	4026.5	4172.8	2422	3475.6	3545.01	3970.506667	3147.553333	-0.31097377	0.280531199	0.889355605
170935	'Grid2ip'	4	1	5	3	3	0	3.333333333	2	-0.620272286	0.706872829	0.972790461
170936	'Zfp369'	1832.7	1890	733.05	698.57	1884.8	1136.62	1485.223333	1239.996667	-0.213725158	0.646504786	0.972790461
170938	'Zfp617'	812	833	592	319	733	547	745.6666667	533	-0.466843746	0.143246275	0.749215454
170947	'Myoz3'	5	3	3	3	17	101	3.666666667	40.33333333	3.313942847	0.004707354	0.16272038
17095	'Lyl1'	10.39	12.77	3	1.14	18.39	2	8.72	7.176666667	-0.260497276	0.837031414	0.974723675
170952	'Prima1'	9	10	4	5	5	4	7.666666667	4.666666667	-0.594950537	0.528535777	0.972790461
17096	'Lyn'	152	191	148	119	282	229	163.6666667	210	0.358055643	0.311195265	0.911205466
17101	'Lyst'	1782	1894	506	862	1252	1073	1394	1062.333333	-0.27502058	0.585848409	0.972790461
17105	'Lyz2'	186	263	8	6540	443	1505	152.3333333	2829.333333	4.682195429	8.95E-05	0.012810696
171095	'Il17rc'	67	93	53	31	114	159	71	101.3333333	0.459333029	0.409282601	0.957157474
17110	'Lyz1'	6	9	0	310	25	69	5	134.6666667	5.202465421	2.96E-05	0.005737356
17112	'Tm4sf1'	87	103	97	550	202	178	95.66666667	310	1.944847938	0.005378587	0.17331813
17113	'M6pr'	2187	2062	2197	2948	2564	3646	2148.666667	3052.666667	0.575606543	0.11052954	0.691700995
17116	'Mab21l1'	11	13	0	9	5	3	8	5.666666667	-0.196344099	0.887698466	0.984534762
171166	'Mcoln3'	7	10	37	38	9	14	18	20.33333333	0.321118763	0.763067049	0.972790461
171167	'Fut10'	243	240	89	307	537	542	190.6666667	462	1.342558511	0.001690478	0.088782789
171168	'Acer1'	1	3	1	0	8	8	1.666666667	5.333333333	1.581130041	0.331995233	0.921648675
17117	'Amacr'	284	268	221	65	259	285	257.6666667	203	-0.406260642	0.405419953	0.957157474
171170	'Mbnl3'	2619	3270	561	264	2853	1987	2150	1701.333333	-0.366139663	0.648911127	0.972790461
171171	'Ntng2'	32	13	35	49	14	18	26.66666667	27	0.226373501	0.797361673	0.972790461
17118	'Marcks'	4065	3910	8875	3000	5415	3826	5616.666667	4080.333333	-0.50036998	0.351766684	0.929586489
171180	'Syt12'	62	86	77	63	54	58	75	58.33333333	-0.271651888	0.562710149	0.972790461
171183	'Vmn1r37'	0	1	0	0	0	0	0.333333333	0	-0.903279821	0.824807108	0.972790461
171184	'Vmn1r36'	1	0	0	0	0	0	0.333333333	0	-0.903279821	0.824807108	0.972790461
171185	'Vmn1r35'	1	4	0	1	2	1	1.666666667	1.333333333	-0.193899554	0.921242854	0.991066749
171188	'Vmn1r32'	22	22	0	1	12	2	14.66666667	5	-1.50806225	0.327526303	0.920734892
17119	'Mxd1'	457	483	2590	830	540	464	1176.666667	611.3333333	-0.912839162	0.287319528	0.894554788
171192	'Vmn1r5'	1	1	0	0	1	0	0.666666667	0.333333333	-0.74517548	0.838722495	0.974723675
171193	'Vmn1r6'	0	0	0	1	0	0	0	0.333333333	1.020273531	0.802557913	0.972790461
171194	'Vmn1r4'	1	2	0	0	2	0	1	0.666666667	-0.537241284	0.8664971	0.978294615
17120	'Mad1l1'	765.01	810	229	545	666	746	601.3366667	652.3333333	0.234158354	0.63352272	0.972790461
171201	'Vmn1r21'	0	1	0	0	0	0	0.333333333	0	-0.903279821	0.824807108	0.972790461
171207	'Arhgap4'	167	202	39	43	231	84	136	119.3333333	-0.158230699	0.830496691	0.974233546
171209	'Asic3'	13	9	12	15	14	3	11.33333333	10.66666667	0.068088834	0.942089527	0.996253096

17121	'Mxd3'	47	49	15	6	60	57	37	41	0.1000951	0.902131014	0.987328425
171210	'Acot2'	278.19	287.18	1245.8	67.52	339.04	434.55	603.7366667	280.37	-1.329618106	0.112070539	0.69684179
171211	'Edaradd'	84	89	17	2	207	98	63.33333333	102.3333333	0.634201784	0.574565437	0.972790461
171212	'Galnt10'	232	245	55	266	337	306	177.3333333	303	0.908983355	0.10539574	0.678099427
17122	'Mxd4'	743	853	429	325	1041	966	675	777.3333333	0.192546623	0.656185081	0.972790461
17123	'Madcam1'	181	184	1259	3	65	29	541.3333333	32.33333333	-4.329170115	5.18E-05	0.00848495
171233	'Vmn1r235'	1	0	0	0	0	0	0.333333333	0	-0.903279821	0.824807108	0.972790461
171234	'Vmn1r237'	0	1	0	0	0	0	0.333333333	0	-0.903279821	0.824807108	0.972790461
171245	'Vmn1r-ps103'	4	0	0	0	2	0	1.333333333	0.666666667	-0.961736678	0.807247815	0.972790461
17125	'Smad1'	571	533	820	195	712	632	641.3333333	513	-0.416230017	0.410696407	0.95722888
17126	'Smad2'	2638	2644	2123	1076	2289	2665	2468.333333	2010	-0.308624327	0.314031655	0.913878742
17127	'Smad3'	3414	3355	5373	2719	4698	4472	4047.333333	3963	-0.057000317	0.890374414	0.984980969
17128	'Smad4'	3382	3192	4519	3157	3379	4222	3697.666667	3586	-0.016428864	0.966243413	0.999493374
171281	'Acot3'	9	2	5.01	2	7.01	4	5.336666667	4.336666667	-0.313101676	0.782964877	0.972790461
171282	'Acot4'	28	38	23.71	20	30.26	32.96	29.90333333	27.74	-0.07342354	0.879656204	0.981807495
171283	'Havcr1'	11	18	2	0	7	5	10.33333333	4	-1.393171268	0.293647525	0.90029104
171284	'Timd2'	11	13	4	0	0	0	9.333333333	0	-5.55167385	0.002096577	0.101167841
171285	'Havcr2'	4	5	2	19	11	23	3.666666667	17.66666667	2.388836506	0.006353611	0.189092275
171286	'Slc12a8'	29.46	20.15	21	55	15	23	23.53666667	31	0.653545558	0.416748943	0.95722888
17129	'Smad5'	5858	5983	2440	3853	5317	4333	4760.333333	4501	0.029177932	0.942730084	0.996301887
17130	'Smad6'	263	281	263	260	276	410	269	315.3333333	0.269916153	0.414645849	0.95722888
17131	'Smad7'	500	464	1858	617	497	636	940.6666667	583.3333333	-0.703740246	0.329342567	0.921648675
17132	'Maf'	658	680	883	611	774	771	740.3333333	718.6666667	-0.015823437	0.965801679	0.999493374
17133	'Maff'	231	175	4038	1165	129	432	1481.333333	575.3333333	-1.255583919	0.323233148	0.918976057
17134	'Mafg'	2634	2523	2907	2063	2521	2322	2688	2302	-0.172676015	0.607104795	0.972790461
17135	'Mafk'	365	346	1183	1894	370	670	631.3333333	978	0.814086394	0.365175107	0.935765447
17136	'Mag'	13	8	8	47	5	8	9.666666667	20	1.41236864	0.207792956	0.835213465
17137	'Magea1'	0	0	0	0	0	1.36	0	0.453333333	1.020273531	0.802557913	0.972790461
17138	'Magea2'	23.67	23.99	0	0	5	22.93	15.88666667	9.31	-0.827225568	0.634260411	0.972790461
171388	'Bnip1'	6.69	9	2	2	4.33	10.78	5.896666667	5.703333333	-0.078347843	0.943497972	0.996436989
17139	'Magea3'	55.99	47.57	4	0	7	35	35.85333333	14	-1.408245983	0.301424083	0.90469437
171395	'Pkd1l1'	613	449	16	0	57	80	359.3333333	45.66666667	-3.030452669	0.02774581	0.407338487
17140	'Magea4'	35	39	7	0	12	29	27	13.66666667	-1.059184776	0.383167203	0.945460723
17141	'Magea5'	48.36	39.71	12	0	19	58.28	33.35666667	25.76	-0.464635671	0.706497674	0.972790461
17142	'Magea6'	52.73	59.54	7	0	7	28	39.75666667	11.66666667	-1.824438105	0.146081519	0.753611197
171429	'Slc26a6'	110	118	68	196	153	168	98.66666667	172.3333333	0.949131099	0.034293371	0.445041341
17144	'Magea8'	57.26	71.2	11	0	11	48.43	46.48666667	19.81	-1.323135209	0.293994769	0.90029104
17146	'Mageb2'	0	0	0	0	0	1	0	0.333333333	1.020273531	0.802557913	0.972790461
171463	'Il17rd'	708	680	614	1159	1021	1298	667.3333333	1159.333333	0.886306823	0.010831074	0.256758571
171469	'Gpr37l1'	0	1	1	1	0	0	0.666666667	0.333333333	-0.885963919	0.798167427	0.972790461

17147	'Mageb3'	12.36	14.52	4.4	0	10	3	10.42666667	4.333333333	-1.243767477	0.331272384	0.921648675
171486	'Cd99l2'	1068	1089	822	956	1523	1664	993	1381	0.506300797	0.029963853	0.421360177
17149	'Magoh'	615	686	1061	367	572	724	787.3333333	554.3333333	-0.543708005	0.211542513	0.840335617
17150	'Mfap2'	1369	1392	279	198	1436	825	1013.333333	819.6666667	-0.311529079	0.671291913	0.972790461
171504	'Apobr'	35	31	232	24	30	80	99.33333333	44.66666667	-1.3344047	0.161804255	0.771541907
171506	'H1f8'	1	0	1	1	5	35	0.666666667	13.66666667	4.176323478	0.016957677	0.326315141
171508	'Creld1'	299	311	740	412	343	795	450	516.6666667	0.154174588	0.800899974	0.972790461
17151	'Ccndbp1'	460	503	578	452	512	537	513.6666667	500.3333333	0.00832188	0.981511177	0.999493374
17152	'Mak'	33	45	6	24	18	7	28	16.33333333	-0.510947129	0.582844135	0.972790461
17153	'Mal'	7	4	5	35	8	18	5.333333333	20.33333333	2.156986036	0.025568259	0.395206288
171530	'Ucn2'	6	0	0	0	2	0	2	0.666666667	-1.543354976	0.676514821	0.972790461
171531	'Mlph'	64	47	69	27	15	25	60	22.33333333	-1.334902631	0.024389871	0.386416857
171543	'Bmf'	624	653	284	2797	791	875	520.3333333	1487.666667	1.839906857	0.012948049	0.284587648
17155	'Man1a'	1668	1666	691	1587	1954	2063	1341.666667	1868	0.582195716	0.13944047	0.741642687
17156	'Man1a2'	1737	1752	851	899	2018	1595	1446.666667	1504	0.096198346	0.796233424	0.972790461
171567	'Nme7'	384.55	361.36	281	191.11	443.14	385.45	342.3033333	339.9	-0.005737403	0.985648626	0.999493374
17158	'Man2a1'	1449	1391	719	4755	1847	1969	1186.333333	2857	1.541280236	0.016740367	0.323948168
171580	'Mical1'	342	390	440	439	513	592	390.6666667	514.6666667	0.431591823	0.219329035	0.846595396
17159	'Man2b1'	627	674	440	628	1169	1050	580.3333333	949	0.743404643	0.006593576	0.192446523
17160	'Man2b2'	488	516	252	302	696	828	418.6666667	608.6666667	0.548062762	0.168633839	0.784384366
17161	'Maoa'	3135.4	3288	5263	1342	1723.4	1960.9	3895.463333	1675.446667	-1.22162036	0.004198979	0.149966291
17164	'Mapkapk2'	2576	2524	4790	7076	2593	3893	3296.666667	4520.666667	0.608549333	0.366765145	0.936719363
17165	'Mapkapk5'	517	553	647	241	515	480	572.3333333	412	-0.50091755	0.163544258	0.77577431
17167	'Marco'	186	188	39	6	68	304	137.6666667	126	-0.217038637	0.836399023	0.974723675
17168	'Nprl3'	489.02	474.17	175.8	99.63	527.31	421.96	379.6633333	349.6333333	-0.142318529	0.811775333	0.972790461
17169	'Mark3'	2951	2957	1557	2072	2661	2719	2488.333333	2484	0.085908154	0.791092483	0.972790461
17171	'Mas1'	22	21	4	80	19	9	15.66666667	36	1.6148602	0.13804711	0.739518639
17172	'Ascl1'	0	0	1	1	1	1	0.333333333	1	1.557984566	0.561051704	0.972790461
17173	'Ascl2'	27	17	451	7	7	19	165	11	-4.104979491	6.97E-04	0.052467355
17174	'Masp1'	84	89	59	522	193	316	77.33333333	343.6666667	2.371135216	1.01E-04	0.013994201
17175	'Masp2'	19.93	28.35	57.98	1.32	4.31	0	35.42	1.876666667	-4.425254615	1.06E-04	0.014511205
17178	'Fxyd3'	4	1	0	1	0	1	1.666666667	0.666666667	-1.098882957	0.656449263	0.972790461
17179	'Matk'	50	43	125	17	65	100	72.66666667	60.66666667	-0.430164408	0.567165886	0.972790461
17181	'Matn2'	946	893	459	732	1377	1030	766	1046.333333	0.513276705	0.154938528	0.766453805
17182	'Matn3'	14	9	2	0	5	1	8.333333333	2	-2.057828287	0.16170581	0.771541907
17183	'Matn4'	4	5	5	14	11	8	4.666666667	11	1.361979352	0.129704235	0.727578716
17184	'Matr3'	9111	9389	9817	5905	9008	8750	9439	7887.666667	-0.238251866	0.395574812	0.954014376
17187	'Max'	917	921	2203	1217	932	1409	1347	1186	-0.169982605	0.773032048	0.972790461
17188	'Maz'	3396	3433	3795	2099	3923	3745	3541.333333	3255.666667	-0.128747674	0.674015264	0.972790461
17189	'Mb'	0	0	4	1	0	1	1.333333333	0.666666667	-1.047534352	0.7316437	0.972790461

17190	'Mbd1'	1985	2001	1614	2501	1928	1465	1866.666667	1964.666667	0.236239249	0.616335467	0.972790461
17191	'Mbd2'	776	785	1544	1083	927	1217	1035	1075.666667	0.082179114	0.875543712	0.980897882
17192	'Mbd3'	1464	1537	1661	659	1329	1483	1554	1157	-0.452554631	0.172322349	0.790015071
17193	'Mbd4'	298	350	128	171	258	217	258.6666667	215.3333333	-0.168745391	0.69394369	0.972790461
17194	'Mbl1'	2	1	0	1	1	0	1	0.666666667	-0.338798923	0.89654217	0.985710789
17195	'Mbl2'	8	8	2	0	29	82	6	37	2.517545828	0.069047076	0.58603706
17196	'Mbp'	234	258	487	328	240	423	326.3333333	330.3333333	0.03807974	0.945145291	0.996592403
17199	'Mc1r'	5	6	2	8	3	3	4.333333333	4.666666667	0.349935296	0.765102568	0.972790461
17200	'Mc2r'	9	14	1	21	11	11	8	14.33333333	1.10168886	0.285711866	0.893820325
17201	'Mc3r'	0	0	9	0	0	1	3	0.333333333	-3.221439683	0.345829398	0.926957353
17202	'Mc4r'	37	30	11	25	37	26	26	29.33333333	0.282138226	0.645115679	0.972790461
17203	'Mc5r'	1	2	4	11	6	4	2.333333333	7	1.722134964	0.163756754	0.776394866
17207	'Mcf2l'	153	161	133	72	220	156	149	149.3333333	-0.014916142	0.970923542	0.999493374
17210	'Mcl1'	2872	2921	3884	4571	3206	3683	3225.666667	3820	0.347064972	0.467809325	0.972790461
17215	'Mcm3'	1829	1952	870	363	1428	1650	1550.333333	1147	-0.466628962	0.371788361	0.938527219
17216	'Mcm2'	1350	1421	704	736	1337	1569	1158.333333	1214	0.102328101	0.76665572	0.972790461
17217	'Mcm4'	1747	1773	841	586	1426	1417	1453.666667	1143	-0.329341922	0.40208278	0.955996639
17218	'Mcm5'	2193	2274	674	58	1477	1532	1713.666667	1022.333333	-0.823663462	0.361401275	0.933706718
17219	'Mcm6'	3302	3551	1724	1684	3087	3232	2859	2667.666667	-0.057057222	0.865225729	0.978040999
17220	'Mcm7'	1619	1786	2008	697	1484	1553	1804.333333	1244.666667	-0.569123916	0.105715624	0.679124905
17221	'Cd46'	21	23	9	13	53	13	17.66666667	26.33333333	0.613743751	0.438565787	0.966976432
17222	'Anapc1'	5804	5754	2179	4086	5299	4722	4579	4702.333333	0.154597391	0.716364649	0.972790461
17224	'Mcpt1'	2	4	0	0	7	16	2	7.666666667	1.87408888	0.299935628	0.903723114
17225	'Mcpt2'	5	2	0	0	25	29	2.333333333	18	2.881533117	0.086576103	0.637978633
17227	'Mcpt4'	4	4	0	0	91	47	2.666666667	46	4.057038515	0.011889994	0.269701225
17228	'Cma1'	22	22	3	1	175	46	15.66666667	74	2.194619968	0.085569761	0.634617076
17229	'Tpsb2'	20	20	0	0	179	72	13.33333333	83.66666667	2.617908629	0.121235278	0.712496171
17231	'Mcpt8'	0	0	0	0	2	0	0	0.666666667	1.801491674	0.656112935	0.972790461
17237	'Mgrn1'	1596	1545	1196	546	1824	1827	1445.666667	1399	-0.089827611	0.828812227	0.973639277
17240	'Mdfi'	592	658	1539	1028	881	1057	929.6666667	988.6666667	0.106008127	0.857662596	0.976399524
17242	'Mdk'	1495	1480	1409	468	1708	1382	1461.333333	1186	-0.355002735	0.408771923	0.957157474
17245	'Mdm1'	395	439	134	485	355	297	322.6666667	379	0.447144776	0.440860459	0.968551476
17246	'Mdm2'	2035.9	2025.1	2774	1925	1671	1756	2278.336667	1784.013333	-0.276790639	0.528933232	0.972790461
17248	'Mdm4'	4912	5106	2583	4895	5444	3487	4200.333333	4608.666667	0.281167772	0.523753607	0.972790461
17250	'Abcc1'	1590	1428	1270	3383	1716	2849	1429.333333	2649.333333	1.043715693	0.033357364	0.440560798
17252	'Rdh11'	482	474	542	443	667	757	499.3333333	622.3333333	0.323236902	0.298740624	0.902535634
17254	'Slc3a2'	1315.1	1201.9	3864.2	1435.8	1735	2855	2127.076667	2008.586667	-0.163441811	0.797815915	0.972790461
17256	'Mea1'	861	891	1103	479	788	1241	951.6666667	836	-0.228042564	0.564368276	0.972790461
17257	'Mecp2'	1914	1930	1324	1723	1580	1637	1722.666667	1646.666667	0.055031576	0.878163775	0.981341203
17258	'Mef2a'	3450	3707	4966	3369	4762	4034	4041	4055	0.02537076	0.947384282	0.997071032

17259	'Mef2b'	9	5.05	32.86	21	0	11	15.63666667	10.66666667	-0.374087044	0.796279996	0.972790461
17260	'Mef2c'	462.44	481.21	377.59	118.5	428.95	278.22	440.4133333	275.2233333	-0.70735377	0.102170588	0.671776916
17261	'Mef2d'	1883	1864	3114	3005	1725	2555	2287	2428.333333	0.178526691	0.744383562	0.972790461
17268	'Meis1'	820	849	624	661	850	493	764.3333333	668	-0.086945253	0.829274934	0.973678583
17274	'Rab8a'	1091	1207	1609	767	1056	1304	1302.333333	1042.333333	-0.32844881	0.385671171	0.947213579
17279	'Melk'	509	525	105	38	356	537	379.6666667	310.3333333	-0.343590199	0.681223736	0.972790461
17281	'Fyco1'	1629	1610	523	1365	1845	1580	1254	1596.666667	0.468773134	0.316130651	0.914806078
17283	'Men1'	1470	1437	1440	1339	1390	1642	1449	1457	0.071310191	0.822001701	0.972790461
17285	'Meox1'	82	70	109	20	58	176	87	84.66666667	-0.181081242	0.801844004	0.972790461
17286	'Meox2'	31	33	17	2243	56	92	27	797	5.373619304	1.92E-05	0.004057658
17287	'Mep1a'	5	4	1	0	1	0	3.333333333	0.333333333	-3.095172496	0.145915174	0.753611197
17288	'Mep1b'	2	2	0	0	0	2	1.333333333	0.666666667	-0.99885442	0.740500622	0.972790461
17289	'Mertk'	671.01	670.51	196.37	1098.8	1076.2	913.65	512.63	1029.56	1.17863725	0.027214153	0.40421521
17292	'Mesp1'	0	0	0	0	1	0	0	0.333333333	1.020273531	0.802557913	0.972790461
17293	'Mesp2'	18	14	18	6	12	19	16.66666667	12.33333333	-0.466846226	0.493710572	0.972790461
17294	'Mest'	9915.2	10057	2719.8	6952.6	11346	9406.98	7563.94	9235.186667	0.392154841	0.435667195	0.96581102
17295	'Met'	151	160	103	491	708	826	138	675	2.336198973	6.13E-17	1.94E-13
17299	'Mettl1'	244	256	336	43	180	292	278.6666667	171.6666667	-0.825000831	0.174077968	0.793056387
17300	'Foxc1'	65	62	46	12	43	23	57.66666667	26	-1.149993159	0.032325757	0.434162511
17301	'Foxd2'	16	19	41	13	17	11	25.33333333	13.66666667	-0.884661534	0.239691511	0.86449675
17304	'Mfge8'	1780	1836	1046	4927	3067	2772	1554	3588.666667	1.412743487	0.0064364	0.190013707
17305	'Mfng'	107	102	14	5	132	63	74.33333333	66.66666667	-0.189737096	0.851759625	0.975734242
17306	'Sypl2'	9	7	2	3	5	0	6	2.666666667	-0.995346394	0.488109041	0.972790461
17308	'Mgat1'	978	1006	656	798	1316	1221	880	1111.666667	0.385159677	0.140328372	0.743154041
17309	'Mgat3'	756	709	793	573	918	1216	752.6666667	902.3333333	0.250006904	0.435655904	0.96581102
17311	'Kitl'	9070	9129	3778	4529	20276	14572	7325.666667	13125.66667	0.829105608	0.117770858	0.706468633
17312	'Clec10a'	1	3	2	1	2	10	2	4.333333333	1.026033523	0.482157844	0.972790461
17313	'Mgp'	289	330	70	136	583	589	229.6666667	436	0.924644949	0.157821022	0.768765891
17314	'Mgmt'	127	137	73	32	173	330	112.3333333	178.3333333	0.582259527	0.397565548	0.954563638
17318	'Mid1'	721	795	2056	163	379	230	1190.666667	257.3333333	-2.286967208	2.21E-04	0.024317809
17319	'Mif'	2382	2123	12573	3801	1587	3277	5692.666667	2888.333333	-0.964157896	0.267787172	0.883600995
17329	'Cxcl9'	0	0	20	0	1	0	6.666666667	0.333333333	-4.377047242	0.150327602	0.759693458
17330	'Minpp1'	929	947	635	883	1176	1289	837	1116	0.476476736	0.06451146	0.570648987
17339	'Mip'	7	6	3	0	6	1	5.333333333	2.333333333	-1.231976293	0.40629484	0.957157474
17341	'Bhlha15'	8	10	273	5	6	28	97	13	-3.170958795	0.018009498	0.335354139
17342	'Mitf'	111	120	97	264	160	153	109.3333333	192.3333333	0.991126703	0.061934051	0.564873458
17344	'Pias2'	3156	3033	2053	2270	2234	2892	2747.333333	2465.333333	-0.064853308	0.838150204	0.974723675
17345	'Mki67'	4285	4348	921	226	2839	1302	3184.666667	1455.666667	-1.15537731	0.155014306	0.766453805
17346	'Mknk1'	608	625	906	700	730	837	713	755.6666667	0.118071809	0.777775749	0.972790461
17347	'Mknk2'	2825	2770	2304	2053	1880	2421	2633	2118	-0.226191666	0.488964326	0.972790461

17349	'Mlf1'	300	271	349	261	172	270	306.6666667	234.3333333	-0.301197982	0.519705582	0.972790461
17350	'Mlh1'	580	661	289	137	454	419	510	336.6666667	-0.603986513	0.195250564	0.82024344
17354	'Mllt10'	2023.5	2001.3	1609.2	528.23	1615.6	1378	1878.01	1173.933333	-0.706274906	0.060496445	0.558329328
17355	'Aff1'	1922	1788	1971	1529	2486	2096	1893.666667	2037	0.128027745	0.670305485	0.972790461
17356	'Afdn'	3601	3031	2371	1795	3689	4408	3001	3297.333333	0.132857366	0.674215224	0.972790461
17357	'Marcksl1'	4505	4636	4881	1085	5263	4417	4674	3588.333333	-0.468732959	0.359308614	0.933706718
17364	'Trpm1'	8	6	1	0	2	6	5	2.666666667	-0.953293856	0.536722416	0.972790461
17380	'Mme'	1542	1568	630	832	2552	2272	1246.666667	1885.333333	0.60669477	0.193090733	0.816149625
17381	'Mmp12'	4	2	4	191	9	293	3.333333333	164.3333333	5.712080279	4.63E-07	1.96E-04
17384	'Mmp10'	11	1	82	17	5	11	31.33333333	11	-1.49667305	0.271858076	0.884539076
17385	'Mmp11'	927	1075	557	499	1864	1946	853	1436.333333	0.718614474	0.131046674	0.728882802
17386	'Mmp13'	2	4	43	47	4	12	16.33333333	21	0.522388209	0.710881119	0.972790461
17387	'Mmp14'	3054	3097	3000	3461	5591	5631	3050.333333	4894.333333	0.69914079	0.006145292	0.185170597
17388	'Mmp15'	2193	2469	128	168	2519	1501	1596.666667	1396	-0.206750535	0.837587416	0.974723675
17389	'Mmp16'	168	157	43	52	269	181	122.6666667	167.3333333	0.44847839	0.495207637	0.972790461
17390	'Mmp2'	2889	2711	1155	1566	2367	1147	2251.666667	1693.333333	-0.280201193	0.569209413	0.972790461
17391	'Mmp24'	37.49	36.35	53.25	2.15	37.69	18.17	42.36333333	19.33666667	-1.277653835	0.139306849	0.741642687
17392	'Mmp3'	0	0	1	1829	0	14	0.333333333	614.3333333	11.15936257	0.004045544	0.14678221
17393	'Mmp7'	0	0	0	1	0	0	0	0.333333333	1.020273531	0.802557913	0.972790461
17394	'Mmp8'	2	2	0	19	9	4	1.333333333	10.66666667	3.307738006	0.01783169	0.333677259
17395	'Mmp9'	47	35	88	61	51	50	56.66666667	54	-0.021073465	0.973642819	0.999493374
17420	'Mnat1'	579	610	1037	448	541	691	742	560	-0.416600492	0.361330464	0.933706718
17423	'Ndst2'	808	784	433	724	1165	1033	675	974	0.595438922	0.059780514	0.554643744
17425	'Foxk1'	1744	1673	2103	1630	1524	1841	1840	1665	-0.084201569	0.827179757	0.973067256
17427	'Mns1'	248.44	315.47	28	31	517.62	324.27	197.3033333	290.9633333	0.536755986	0.576758833	0.972790461
17428	'Mnt'	1017	1003	3497	543	837	974	1839	784.6666667	-1.319634178	0.045896412	0.499760929
17433	'Mobp'	1	5	1	0	2	1	2.333333333	1	-1.240564265	0.521166398	0.972790461
17434	'Mocs2'	414	425	723	635	514	544	520.6666667	564.3333333	0.183118044	0.722203117	0.972790461
17436	'Me1'	2188	1842	4862	38923	2538	13657	2964	18372.66667	2.873009373	0.003674364	0.139157376
17441	'Mog'	1.13	5.23	1	0	0	0	2.453333333	0	-3.548222443	0.207294295	0.83479753
17444	'Grap2'	5	13	1	1	15	5	6.333333333	7	0.147352396	0.910364855	0.989561592
17448	'Mdh2'	1707	1832	2340	4096	2178	3335	1959.666667	3203	0.828855485	0.112442183	0.697927127
17449	'Mdh1'	1894	2141	3267	3227	2309	3475	2434	3003.666667	0.360984296	0.467093588	0.972790461
17450	'Morc1'	90	110	24	30	106	100	74.66666667	78.66666667	0.093540002	0.885075309	0.983980573
17451	'Mos'	4	0	3	0	4	14	2.333333333	6	1.167046453	0.533854367	0.972790461
17454	'Mov10'	2557	2603	1177	433	2852	2075	2112.333333	1786.666667	-0.284835212	0.635086743	0.972790461
17463	'Psmd7'	1499	1644	1792	2276	1665	2086	1645	2009	0.389524283	0.366337933	0.936406787
17470	'Cd200'	462	422	315	86	720	657	399.6666667	487.6666667	0.196098411	0.760018508	0.972790461
17472	'Gbp4'	145.91	118.15	374.38	5	23	42.91	212.8133333	23.63666667	-3.372439422	2.07E-05	0.004291
17474	'Clec4d'	24	28	10	114	76	81	20.66666667	90.33333333	2.316759451	1.33E-04	0.017110932

17475	'Mpdz'	2248	2218	843	3004	2130	2227	1769.666667	2453.666667	0.666288513	0.199639274	0.827115347
17476	'Mpeg1'	124	87	23	569	146	604	78	439.6666667	2.712795231	4.71E-04	0.042077778
17480	'Mpl'	3	0	1	0	0	0	1.333333333	0	-2.785807963	0.483270404	0.972790461
17523	'Mpo'	0	0	1	0	7	1	0.333333333	2.666666667	2.785316422	0.323950629	0.919383393
17524	'Mpp1'	589	613	602	467	734	806	601.3333333	669	0.166229214	0.544718329	0.972790461
17527	'Mpv17'	446	508	622	272	512	526	525.3333333	436.6666667	-0.291679789	0.430144062	0.962548446
17528	'Mpz'	94	83	34	13	28	13	70.33333333	18	-1.882009768	0.001738408	0.08981863
17532	'Mras'	694	690	954	1984	697	775	779.3333333	1152	0.777043376	0.253567169	0.872664697
17533	'Mrc1'	72	74	16	7	163	252	54	140.6666667	1.305553175	0.187590412	0.808805329
17534	'Mrc2'	1909	1827	270	1434	1781	1410	1335.333333	1541.666667	0.372064427	0.576170387	0.972790461
17535	'Mre11a'	1209	1178	846	335.96	877	779.09	1077.666667	664.0166667	-0.705956721	0.03868251	0.460965603
17536	'Meis2'	375	312	717	963	343	444	468	583.3333333	0.4803332	0.506115368	0.972790461
17537	'Meis3'	347	344	485	125	445	332	392	300.6666667	-0.459462595	0.347390194	0.928392235
17540	'Mrvi1'	482	407	57	13	291	210	315.3333333	171.3333333	-0.920381717	0.341966214	0.925308103
17681	'Msc'	8	6	29	4	4	1	14.33333333	3	-2.23645683	0.058749228	0.552152463
17684	'Cited2'	1112	1027	6754	8498	1629	2663	2964.333333	4263.333333	0.6759088	0.509063206	0.972790461
17685	'Msh2'	1735	1880	1240	982	1714	2114	1618.333333	1603.333333	0.003578369	0.989849363	0.999493374
17686	'Msh3'	588.52	661.56	530	474	844.37	607.61	593.36	641.9933333	0.153128918	0.615034287	0.972790461
17687	'Msh5'	476	447	176	25	172	62	366.3333333	86.33333333	-2.093354286	0.001863657	0.093735029
17688	'Msh6'	2987	3084	1432	1670	2333	3094	2501	2365.666667	-0.019014258	0.957413427	0.999493374
17690	'Msi1'	525	510	407	409	588	520	480.6666667	505.6666667	0.130591972	0.629020158	0.972790461
17691	'Sik1'	895	903	3566	2177	545	857	1788	1193	-0.436089075	0.624739474	0.972790461
17692	'Msl3'	1322	1331	1677	695	1365	1353	1443.333333	1137.666667	-0.369389333	0.296509267	0.90029104
17695	'Msmb'	0	0	0	1	0	0	0	0.333333333	1.020273531	0.802557913	0.972790461
17698	'Msn'	2055	2066	3154	4071	2643	3774	2425	3496	0.61067268	0.226157576	0.853679947
17700	'Mstn'	0	0	0	0	0	1	0	0.333333333	1.020273531	0.802557913	0.972790461
17701	'Msx1'	134	146	43	16	251	94	107.6666667	120.3333333	0.120399956	0.886524076	0.984398578
17702	'Msx2'	94	88	49	17	68	70	77	51.66666667	-0.602693948	0.267586668	0.883600995
17703	'Msx3'	2	2	0	2	3	0	1.333333333	1.666666667	0.54104676	0.804230483	0.972790461
17713	'Grpel1'	789.82	752.23	1529.8	1357.8	884.58	1439.99	1023.94	1227.46	0.308251219	0.583527755	0.972790461
17714	'Grpel2'	699	716	1026	929	711	995	813.6666667	878.3333333	0.173568774	0.706771837	0.972790461
17748	'Mt1'	682	717	4288	850	707	1605	1895.666667	1054	-0.956340283	0.260285008	0.878537553
17749	'Polr2k'	487.91	510.94	527.87	178.95	251.9	421.97	508.9066667	284.2733333	-0.855741554	0.018652555	0.340229851
17750	'Mt2'	511	536	2233	421	408	910	1093.333333	579.6666667	-1.020856416	0.176136257	0.796060628
17751	'Mt3'	6	13	16	29	11	68	11.66666667	36	1.614047547	0.063031845	0.566852792
17752	'Mt4'	0	0	0	1	0	0	0	0.333333333	1.020273531	0.802557913	0.972790461
17754	'Map1a'	1099	1109	350	1724.7	1105	1419.74	852.6666667	1416.49	0.930937004	0.093981101	0.657941197
17755	'Map1b'	2108	2022	3362	2061	2860	2720	2497.333333	2547	0.028884759	0.945938734	0.996592403
17756	'Map2'	837	792	544	906	618	493	724.3333333	672.3333333	0.082903064	0.87038532	0.979911512
17758	'Map4'	5106	5297	8201	5019	5665	5920	6201.333333	5534.666667	-0.139142174	0.745308672	0.972790461

17760	'Map6'	37	39	55	2	4	9	43.66666667	5	-3.181853852	1.59E-05	0.003595222
17761	'Map7'	272	272	533	314	421	678	359	471	0.337209912	0.509671069	0.972790461
17762	'Mapt'	139	96	669	79	34	37	301.3333333	50	-2.521496917	0.006779063	0.195187218
17763	'Mtcp1'	103.15	102.65	22.79	36.67	114.22	51.2	76.19666667	67.36333333	-0.107574438	0.874909208	0.98053371
17764	'Mtf1'	524.78	494.4	376.76	533.02	481.85	578.47	465.3133333	531.1133333	0.294515424	0.398020205	0.954563638
17765	'Mtf2'	2138	2137	1534	1471	1979	1483	1936.333333	1644.333333	-0.149524512	0.639436474	0.972790461
17766	'Nudt1'	171	166	84	50	150	181	140.3333333	127	-0.158497691	0.742521719	0.972790461
17768	'Mthfd2'	336	327	2226	299	405	569	963	424.3333333	-1.315954236	0.122705493	0.714169238
17769	'Mthfr'	1135	1110	1081	1095	1062	952	1108.666667	1036.333333	0.002383023	0.994938047	0.999562152
17771	'Tesmin'	330	307	98	1	119	78	245	66	-1.970441998	0.061471978	0.562960142
17772	'Mtm1'	356	296	459	278	358	527	370.3333333	387.6666667	0.051790028	0.899468738	0.986106073
17773	'Mtnr1a'	0	0	0	0	1	0	0	0.333333333	1.020273531	0.802557913	0.972790461
17775	'Laptm4a'	6816	6956	7422	6397	9358	10874	7064.666667	8876.333333	0.339949704	0.233381239	0.86022569
17776	'Mast2'	2590	2558	3416	2167	2120	2500	2854.666667	2262.333333	-0.286101894	0.469234229	0.972790461
17777	'Mttp'	202	203	65	167	187	141	156.6666667	165	0.22903095	0.663449304	0.972790461
17826	'Fam89b'	512	520	496	587	694	540	509.3333333	607	0.334050579	0.348351216	0.928444937
17827	'Mtx1'	443	444	280	197	408	345	389	316.6666667	-0.266694638	0.396613283	0.954358954
17828	'Bloc1s5'	538	574	348	164	564	733	486.6666667	487	-0.04498136	0.92541807	0.992381554
17829	'Muc1'	37	36	57	2415	33	454	43.33333333	967.3333333	4.83216215	4.99E-05	0.008322658
17831	'Muc2'	160	118	457	30	50	25	245	35	-2.847751612	9.33E-05	0.013233875
17833	'Muc5ac'	2	0	0	0	0	0	0.666666667	0	-1.711373851	0.67234292	0.972790461
17836	'Mug1'	6	10.3	0	0	11.49	2.44	5.433333333	4.643333333	-0.304697055	0.871283185	0.980167805
17837	'Mug2'	13	19.7	2	0	54.51	12.56	11.56666667	22.35666667	0.917519721	0.525161011	0.972790461
17842	'Mup3'	0	0	0	1	0	2	0	1	2.507928928	0.530239069	0.972790461
17846	'Commd1'	472	535	537.01	400.02	660.08	805	514.67	621.7	0.266063769	0.39359926	0.953178011
17847	'Usp34'	7167.9	7681.3	3065.5	4440.1	6242	6974.59	5971.563333	5885.563333	0.061435113	0.872726	0.980171118
17850	'Mmut'	968	1013	461	714	1206	1188	814	1036	0.409504987	0.246984145	0.868602029
17855	'Mvk'	267	259	112	89	270	188	212.6666667	182.3333333	-0.19857596	0.675369235	0.972790461
17859	'Mxi1'	1128	967	6554	2287	876	1470	2883	1544.333333	-0.855116984	0.350044754	0.92886351
17863	'Myb'	30	34	18	2	23	4	27.33333333	9.666666667	-1.524411833	0.103251574	0.673650969
17864	'Mybl1'	792	857	184	140	470	292	611	300.6666667	-0.976350807	0.112278277	0.697242245
17865	'Mybl2'	436	422	119	33	337	802	325.6666667	390.6666667	0.180626807	0.837950886	0.974723675
17868	'Mybpc3'	41	63	11	55	116	52	38.33333333	74.33333333	1.067946416	0.121111389	0.712428751
17869	'Myc'	393	394	4069	193	552	764	1618.666667	503	-1.920380411	0.051105452	0.524065182
17872	'Ppp1r15a'	482	424	6254	1332	558	985	2386.666667	958.3333333	-1.326887773	0.217590488	0.844165262
17873	'Gadd45b'	365	352	6128	1055	232	520	2281.666667	602.3333333	-1.862980364	0.113387709	0.698948431
17874	'Myd88'	348	375	557	451	445	593	426.6666667	496.3333333	0.243003908	0.58058056	0.972790461
17876	'Myef2'	2987.2	3063.2	1352.6	1122.1	2888.9	2235.76	2467.63	2082.256667	-0.214136495	0.603598527	0.972790461
17877	'Myf5'	5	1	1	2	2	3	2.333333333	2.333333333	0.085762886	0.953759145	0.99917615
17878	'Myf6'	0	1	0	0	4	0	0.333333333	1.333333333	1.792572865	0.652588671	0.972790461

17879	'Myh1'	7.51	5.05	1	0	2	2	4.52	1.333333333	-1.706989762	0.2886525	0.895874398
17880	'Myh11'	2125	1966	56	291	1147	972	1382.333333	803.3333333	-0.735606063	0.451734149	0.970649024
17882	'Myh2'	0.88	5	1	0	2.94	3	2.293333333	1.98	-0.333749581	0.878617379	0.981388029
17883	'Myh3'	5	16	4	3	2	0	8.333333333	1.666666667	-2.080360738	0.141511126	0.746299453
17884	'Myh4'	74.99	42.97	6	0	3	2	41.32	1.666666667	-4.57557427	1.63E-04	0.019471673
17885	'Myh8'	387.6	479.98	14	3	141.06	89	293.86	77.68666667	-1.945696829	0.105384208	0.678099427
17886	'Myh9'	5038	4685	5342	8428	4941	5957	5021.666667	6442	0.502466307	0.309669554	0.910476663
17888	'Myh6'	48.9	56.93	21	32	49.74	41.97	42.27666667	41.23666667	0.051620659	0.919256943	0.990815958
17896	'Myl4'	59.01	83	27	36	58.02	15	56.33666667	36.34	-0.484269259	0.493794819	0.972790461
17898	'Myl7'	28	38	38	1	8	4	34.66666667	4.333333333	-3.044098345	1.12E-04	0.015060594
17901	'Myl1'	1	3	2	3	11	5	2	6.333333333	1.652438339	0.176655947	0.796450095
17904	'Myl6'	2677	3017	4168	2139	2951	3313	3287.333333	2801	-0.234139871	0.546227714	0.972790461
17907	'Mylpf'	20	23	6	37	11	17	16.33333333	21.66666667	0.692811445	0.434589885	0.965354095
17909	'Myo10'	2162	2301	851	1754	2858	2365	1771.333333	2325.666667	0.482649887	0.246456697	0.868334735
17910	'Myo15'	96	66	13	5	39	75	58.33333333	39.66666667	-0.601821932	0.519202325	0.972790461
17912	'Myo1b'	2492	2691	1319	2519	3817	3481	2167.333333	3272.333333	0.671417447	0.041593293	0.476655631
17913	'Myo1c'	1754	1781	2561	2591	1859	2376	2032	2275.333333	0.245880726	0.607046108	0.972790461
17916	'Myo1f'	32	36	6	17	51	102	24.66666667	56.66666667	1.192081994	0.135395531	0.736012522
17918	'Myo5a'	3385	3171.6	1606	928.01	2068.7	1925.28	2720.863333	1640.653333	-0.700032188	0.057079584	0.547413448
17919	'Myo5b'	1706	1790	336	489	2491	1848	1277.333333	1609.333333	0.341018047	0.620142454	0.972790461
17920	'Myo6'	903	920	794	1260	1766.5	1488	872.3333333	1504.826667	0.844662482	0.002652828	0.115677893
17921	'Myo7a'	191	259	1239	860	672	633	563	721.6666667	0.353663151	0.672389302	0.972790461
17922	'Myo7b'	13	16	6	1	25	4	11.66666667	10	-0.26146153	0.82057056	0.972790461
17925	'Myo9b'	2867	2635	1624	1738	2263	2044	2375.333333	2015	-0.148067498	0.636455792	0.972790461
17926	'Myoc'	16	10	20	27	15	21	15.33333333	21	0.554563607	0.451007374	0.970649024
17927	'Myod1'	0	1	0	0	0	0	0.333333333	0	-0.903279821	0.824807108	0.972790461
17928	'Myog'	22	19	3	0	5	15	14.66666667	6.666666667	-1.200371198	0.356288934	0.931977458
17929	'Myom1'	111	117	53	114	128	37	93.66666667	93	0.179737774	0.787114513	0.972790461
17930	'Myom2'	2	0	16	8	0	1	6	3	-0.786053446	0.712709896	0.972790461
17931	'Ppp1r12a'	2361	2242	2764	1678	2592	2239	2455.666667	2169.666667	-0.160485397	0.629687858	0.972790461
17932	'Myt1'	6	3	2	3	7	5	3.666666667	5	0.488554527	0.655655283	0.972790461
17933	'Myt1l'	484	429	53	6	197	190	322	131	-1.348459358	0.193196807	0.816416469
17936	'Nab1'	1194	1274	2728	8926	1606	2752	1732	4428	1.585932301	0.063891255	0.5684378
17937	'Nab2'	1150	1161	2831	727	1256	1267	1714	1083.333333	-0.728673315	0.189264577	0.812029435
17938	'Naca'	6469	6896	15255	8607	6314	8397	9540	7772.666667	-0.257958715	0.658126021	0.972790461
17939	'Naga'	419	408	419	1265	723	878	415.3333333	955.3333333	1.354233643	0.00666751	0.193826765
17940	'Naip1'	18	15	3	0	13	9	12	7.333333333	-0.757910321	0.548682571	0.972790461
17948	'Naip2'	6	8	0	18	8.3	28	4.666666667	18.1	2.110650709	0.062604193	0.566066012
17951	'Naip5'	3	4	0	7	20.69	43.01	2.333333333	23.56666667	3.308795367	0.002865997	0.119683851
17952	'Naip6'	4	0	0	35	4	7.99	1.333333333	15.66333333	3.939163559	0.019921788	0.349283865

17954	'Napl12'	53	69	34	43	77	70	52	63.33333333	0.332900834	0.427745894	0.961947592
17955	'Napl14'	3594	3640	3636	1469	3519	4183	3623.333333	3057	-0.288177036	0.425729256	0.960707966
17957	'Napb'	162	156	315	249	166	139	211	184.6666667	-0.087197126	0.889590134	0.984980969
17960	'Nat1'	9	10	7	3	17	14	8.666666667	11.33333333	0.341833294	0.684448007	0.972790461
17961	'Nat2'	71	89	94	57	86	84	84.66666667	75.66666667	-0.145586202	0.713101497	0.972790461
17962	'Nat3'	0	0	0	0	1	0	0	0.333333333	1.020273531	0.802557913	0.972790461
17965	'Nbl1'	474	478	399	562	639	749	450.3333333	650	0.592247668	0.035569079	0.452939946
17966	'Nbr1'	3548	3645	5018	4034	3798	3880	4070.333333	3904	0.004109667	0.992346814	0.999493374
17967	'Ncam1'	1191	987	1297	837	1005	786	1158.333333	876	-0.341613203	0.383128626	0.945460723
17968	'Ncam2'	12	22	6	5	32	22	13.33333333	19.66666667	0.543280325	0.517198226	0.972790461
17969	'Ncf1'	29	51	13	45	67	42	31	51.33333333	0.848420673	0.16580053	0.780440621
17970	'Ncf2'	99	102	24	24	97	100	75	73.66666667	-0.022583617	0.973218496	0.999493374
17972	'Ncf4'	11	14	13	6	8	23	12.66666667	12.33333333	-0.074854447	0.923358166	0.991932584
17973	'Nck1'	842	905	1041	563	775	1008	929.3333333	782	-0.247446275	0.458404307	0.972790461
17974	'Nck2'	798	824	1264	1906	1045	1432	962	1461	0.715805407	0.195753309	0.821394515
17975	'Ncl'	13623	13252	20483	5712	11848	15465	15786	11008.33333	-0.588688994	0.180198903	0.801341923
17977	'Ncoa1'	1702	1619	911	869	1844	1781	1410.666667	1498	0.112792693	0.735762059	0.972790461
17978	'Ncoa2'	2539	2426	1098	663	2528	2246	2021	1812.333333	-0.170810598	0.729860525	0.972790461
17979	'Ncoa3'	1514	1381	689	1861	1181	1004	1194.666667	1348.666667	0.392869053	0.471914093	0.972790461
17984	'Ndn'	1600	1709	1392	363	1944	1830	1567	1379	-0.265219702	0.619249182	0.972790461
17986	'Ndp'	3	3	1	1	8	4	2.333333333	4.333333333	0.879119068	0.511838866	0.972790461
17988	'Ndrg1'	3345	2105	53389	636	707	2021	19613	1121.333333	-4.347425195	1.25E-04	0.016489852
17991	'Ndufa2'	629	659	756	491	515	687	681.3333333	564.3333333	-0.234336946	0.508359357	0.972790461
17992	'Ndufa4'	1499	1645	1098	762	1205	1608	1414	1191.666667	-0.225394011	0.424477693	0.960285372
17993	'Ndufs4'	610	614	629	420	692	841	617.6666667	651	0.072315328	0.807566854	0.972790461
17995	'Ndufv1'	2305	2538	1534	1600	1999	2832	2125.666667	2143.666667	0.064814695	0.826369762	0.972844676
17996	'Neb'	513	438	40	71	568	1044	330.3333333	561	0.726643942	0.445772483	0.969915971
17997	'Nedd1'	1036	1029	666	823	1120	1092	910.3333333	1011.666667	0.223383997	0.417000083	0.957382938
17999	'Nedd4'	30741	30208	31473	26226	34673	32461	30807.33333	31120	0.059965099	0.838190027	0.974723675
18000	'Septin2'	5827	6113	8978	5112	6411	7132	6972.666667	6218.333333	-0.156846047	0.694621456	0.972790461
18002	'Nedd8'	1015	1035	917	620	733	1012	989	788.3333333	-0.28890654	0.305063562	0.907188578
18003	'Nedd9'	544	586	1103	10587	709	1802	744.3333333	4366	2.873002855	0.00509187	0.16781708
18004	'Nek1'	2795	2684	2379	750	1764	1787	2619.333333	1433.666667	-0.888331724	0.00505833	0.16781708
18005	'Nek2'	536	581	109	80	333	388	408.6666667	267	-0.614228539	0.372238807	0.938594721
18007	'Neo1'	4951	4806	1357	4156	6476	6106	3704.666667	5579.333333	0.690358732	0.161235949	0.771435531
18008	'Nes'	1059	919	488	957	1789	1379	822	1375	0.804730601	0.026523623	0.39926138
18010	'Neu1'	202	169	312	1116	340	460	227.6666667	638.6666667	1.688666786	0.019950966	0.349283865
18011	'Neurl1a'	266	276	1452	220	159	174	664.6666667	184.3333333	-1.855452805	0.025378004	0.39436194
18013	'Neurod2'	0	0	0	2	0	0	0	0.666666667	2.299096387	0.566358544	0.972790461
18015	'Nf1'	4330	4070	2573	3655	3318	3628	3657.666667	3533.666667	0.074838533	0.838146274	0.974723675

18016	'Nf2'	2292	2213	1675	1501	2119	2221	2060	1947	-0.030363903	0.896638038	0.985710789
18018	'Nfatc1'	116	76	259	208	133	151	150.3333333	164	0.18577052	0.788677346	0.972790461
18019	'Nfatc2'	32	27	25	206	35	125	28	122	2.357638208	0.002870599	0.119683851
18020	'Nfatc2ip'	471	497	161	324	410	394	376.3333333	376	0.116942748	0.801182608	0.972790461
18021	'Nfatc3'	2218.3	2363.3	1934.5	2003.3	2134.5	2168.16	2172.043333	2101.986667	0.037831591	0.902615363	0.987331639
18022	'Nfe2'	30	37	6	9	69	29	24.33333333	35.66666667	0.558641709	0.524456419	0.972790461
18023	'Nfe2l1'	3704	3537	6662	5158	4803	5912	4634.333333	5291	0.216990557	0.659166577	0.972790461
18024	'Nfe2l2'	1831	1894	5388	3960	2636	3760	3037.666667	3452	0.211269548	0.748013111	0.972790461
18025	'Nfe2l3'	41	37	46	211	36	40	41.33333333	95.66666667	1.529294864	0.0939916	0.657941197
18027	'Nfia'	1930	2076	2175	1194	1563	1546	2060.333333	1434.333333	-0.483970213	0.112849384	0.698666814
18028	'Nfib'	2514	2449	2550	1949	2014	1891	2504.333333	1951.333333	-0.278489544	0.431831747	0.963677723
18029	'Nfic'	2482	2428	3172	2186	2334	2820	2694	2446.666667	-0.102545926	0.782447943	0.972790461
18030	'Nfil3'	359	318	3017	831	343	567	1231.333333	580.3333333	-1.068740969	0.279278245	0.889355605
18032	'Nfix'	982.61	808.23	768	479.86	999.61	1039	852.9466667	839.49	-0.030005891	0.92130403	0.991066749
18033	'Nfkb1'	1087	1041	2695	780	1171	1304	1607.666667	1085	-0.626676347	0.266456939	0.88347493
18034	'Nfkb2'	649.23	622.27	3144.5	876.98	660.67	611.22	1472.003333	716.29	-1.03122422	0.201874337	0.82996621
18035	'Nfkbia'	772	729	8490	1746	559	897	3330.333333	1067.333333	-1.591454774	0.135366282	0.736012522
18036	'Nfkbib'	253	281	572	342	203	326	368.6666667	290.3333333	-0.288093111	0.634208067	0.972790461
18037	'Nfkbie'	198	216	703	87	151	106	372.3333333	114.6666667	-1.765740895	0.009707763	0.24220996
18038	'Nfkbil1'	237.95	254.73	558.92	276	250.96	372.92	350.5333333	299.96	-0.22979666	0.684871861	0.972790461
18039	'Nefl'	32	32	107	9	71	101	57	60.33333333	-0.138132938	0.877461403	0.981341203
18040	'Nefm'	28	29	220	153	22	24	92.33333333	66.33333333	-0.276288679	0.815467582	0.972790461
18041	'Nfs1'	815	880	484	351	871	1059	726.3333333	760.3333333	0.057976638	0.882739187	0.982992879
18044	'Nfya'	1099	1184	1410	1217	1372	1162	1231	1250.333333	0.085040439	0.827619616	0.973238667
18045	'Nfyb'	602	594	822.97	533.93	639.97	744	672.99	639.3	-0.056658806	0.881761578	0.982528614
18046	'Nfyc'	824	867	823	248	838	845	838	643.6666667	-0.441434385	0.304914917	0.907188578
18049	'Ngf'	14	7	13	97	5	20	11.33333333	40.66666667	2.186694109	0.056957327	0.547413448
18050	'Klk1b3'	0	0	0	0	1	0	0	0.333333333	1.020273531	0.802557913	0.972790461
18053	'Ngfr'	690	683	386	2534	885	827	586.3333333	1415.333333	1.564926766	0.023145251	0.377626024
18054	'Ngp'	0	64	0	0	171	0	21.33333333	57	1.419783047	0.703593454	0.972790461
18071	'Nhlh1'	1	0	0	3	0	1	0.333333333	1.333333333	2.20694279	0.470369097	0.972790461
18072	'Nhlh2'	1	0	0	0	0	1	0.333333333	0.333333333	0.058500858	0.988561293	0.999493374
18073	'Nid1'	3108	2951	1544	5334	2806	3296	2534.333333	3812	0.805099988	0.136657933	0.737898499
18074	'Nid2'	1149.9	1008.9	267.33	822.6	1286.7	1111.82	808.7133333	1073.696667	0.518529722	0.328656122	0.921648675
18080	'Nin'	925	904	503	266	1639	1166.49	777.3333333	1023.83	0.348087364	0.540734009	0.972790461
18081	'Ninj1'	340	373	862	708	367	901	525	658.6666667	0.347181314	0.592851165	0.972790461
18082	'Nipsnap1'	877	930	541	310	1166.8	1142	782.6666667	872.9466667	0.122431744	0.79086742	0.972790461
18087	'Nktr'	4994	5050.4	5846	3313	4276.6	3302	5296.79	3630.526667	-0.490502	0.178021806	0.797422443
18088	'Nkx2-2'	4	0	19	0	0	0	7.666666667	0	-5.489119572	0.040737288	0.473970635
18089	'Nkx2-3'	0	0	0	3	0	0	0	1	2.899270347	0.465759809	0.972790461

18091	'Nkx2-5'	1	1	2	0	0	0	1.333333333	0	-2.856115697	0.367295997	0.937190492
18094	'Nkx2-9'	0	1	5	1	0	2	2	1	-1.091100476	0.639136876	0.972790461
18095	'Nkx3-1'	1	1	0	20	0	1	0.666666667	7	3.909607301	0.079699092	0.617305615
18096	'Nkx6-1'	4	1	2	1	0	0	2.333333333	0.333333333	-2.640090247	0.243443466	0.865085867
18099	'Nlk'	1255	1408	691	833	1295	1114	1118	1080.666667	0.027722964	0.933926785	0.994249017
18100	'Mrpl40'	467.65	473.44	521.16	120.11	402.49	483.25	487.4166667	335.2833333	-0.619231886	0.188234876	0.81021541
18101	'Nmbr'	10	14	1	3	5	10	8.333333333	6	-0.413203586	0.712765193	0.972790461
18102	'Nme1'	706	850	825	353	687	933	793.6666667	657.6666667	-0.30473681	0.399285609	0.955210216
18103	'Nme2'	1694	1818	4737	1262	1529	1960	2749.666667	1583.666667	-0.843547788	0.151406513	0.75995796
18104	'Nqo1'	107	120	118	608	125	1151	115	628	2.505079542	8.10E-04	0.058366224
18105	'Nqo2'	932	1072	1045	92	1082	825	1016.333333	666.3333333	-0.734513853	0.296601866	0.90029104
18106	'Cd244a'	3	5	1	8	3	4	3	5	0.973300316	0.433793872	0.965077633
18107	'Nmt1'	1500	1535	2159	1904	1469	1989	1731.333333	1787.333333	0.112903437	0.803714712	0.972790461
18108	'Nmt2'	3411.8	3352.9	2515.3	2444.9	3189.9	3528.35	3093.333333	3054.383333	0.03909959	0.870918577	0.980126495
18109	'Mycn'	746	678	830	136	431	602	751.3333333	389.6666667	-1.036190992	0.034542385	0.445535842
18111	'Nnat'	724	809	327	99	636	205	620	313.3333333	-0.992949083	0.129620286	0.727578716
18113	'Nnmt'	2	2	14	78	11	25	6	38	2.835425398	0.018662886	0.340229851
18114	'Rrp1'	2131	2160	3371	3151	2593	3397	2554	3047	0.306686368	0.510689861	0.972790461
18115	'Nnt'	1115	1142	975	316	892	1033	1077.333333	747	-0.572990399	0.143814027	0.750739232
18117	'Emc8'	691.91	702	269.44	668.14	701.1	871.57	554.45	746.9366667	0.546910661	0.202900643	0.83083072
18119	'Nodal'	0	0	0	2	1	0	0	1	2.732929573	0.492631579	0.972790461
18120	'Mrpl49'	732	809	510	455	673	724	683.6666667	617.3333333	-0.096587547	0.712509809	0.972790461
18121	'Nog'	11	4	17	2	7	5	10.66666667	4.666666667	-1.276624075	0.215109776	0.842760284
18124	'Nr4a3'	22	30	182	216	16	62	78	98	0.513717699	0.663827346	0.972790461
18125	'Nos1'	436	462	201	50	385	1518	366.3333333	651	0.716402124	0.425927031	0.96081214
18126	'Nos2'	30	30	506	228	112	94	188.6666667	144.6666667	-0.354790361	0.758296767	0.972790461
18127	'Nos3'	318	293	29	7	408	193	213.3333333	202.6666667	-0.112756788	0.919554247	0.990815958
18128	'Notch1'	518	509	137	617	880	599	388	698.6666667	0.984990537	0.066971842	0.57823282
18129	'Notch2'	2032	1861	766	2297	2743	2482	1553	2507.333333	0.81548098	0.055315992	0.539316735
18130	'Ints6'	4779	4847	1563	729	2904	2457	3729.666667	2030	-0.876967336	0.114229999	0.699220241
18131	'Notch3'	761	563	257	1393	649	655	527	899	1.032750696	0.106257302	0.679124905
18132	'Notch4'	306	265	40	16	562	329	203.6666667	302.3333333	0.524349561	0.60548402	0.972790461
18133	'Ccn3'	11	5	63	6	14	16	26.33333333	12	-1.310403159	0.227412567	0.854007746
18139	'Zfp638'	2621	2789	1553	2071	3121	2614	2321	2602	0.241829585	0.435062406	0.965608974
18140	'Uhrf1'	2155	2266	548	223	1442	2176	1656.333333	1280.333333	-0.417561766	0.574129086	0.972790461
18141	'Nup50'	1800.4	1847.2	2550.2	1066	1637	1697	2065.946667	1466.666667	-0.50133868	0.175550893	0.794684462
18142	'Npas1'	5	3	18	8	6	1	8.666666667	5	-0.708285898	0.580976978	0.972790461
18143	'Npas2'	65	51	170	446	59	213	95.33333333	239.3333333	1.502541205	0.104749756	0.67684333
18145	'Npc1'	1627	1618	1049	2454	2274	2324	1431.333333	2350.666667	0.839725299	0.02107491	0.360644632
18146	'Npdc1'	492	532	917	398	771	767	647	645.3333333	-0.056721484	0.902942358	0.987381169

18148	'Npm1'	20509	20054	51980	7869.4	13822	16667.99	30847.57	12786.33	-1.35730401	0.017575611	0.331280729
18150	'Npm3'	671	668	649	145	554	975	662.6666667	558	-0.349959727	0.537130638	0.972790461
18155	'Pnoc'	12	8	7	63	1	10	9	24.66666667	1.866006443	0.156958412	0.768337387
18158	'Nppb'	0	2	4	1	1	2	2	1.333333333	-0.651128451	0.72877596	0.972790461
18159	'Nppc'	12	5	26	23	3	4	14.33333333	10	-0.278668984	0.819156189	0.972790461
18160	'Npr1'	1411	1529	655	5695	2221	3568	1198.333333	3828	1.916290081	0.001542033	0.084487393
18162	'Npr3'	76	74	53	4	57	55	67.66666667	38.66666667	-0.906633772	0.242379244	0.86449675
18163	'Ctnnd2'	40	23	21	1	16	23	28	13.33333333	-1.166554454	0.191073619	0.813837363
18164	'Nptx1'	51	42	23	47	51	40	38.66666667	46	0.375102375	0.468248294	0.972790461
18166	'Npy1r'	27	24	4	33	35	14	18.33333333	27.33333333	0.789579523	0.357709144	0.932505998
18167	'Npy2r'	12	9	1	0	22	8	7.333333333	10	0.409089368	0.780541104	0.972790461
18168	'Npy5r'	1	0	0	0	0	0	0.333333333	0	-0.903279821	0.824807108	0.972790461
18169	'Npy6r'	1	0	0	0	0	0	0.333333333	0	-0.903279821	0.824807108	0.972790461
18171	'Nr1i2'	29	23	5	7	21	19	19	15.66666667	-0.236764751	0.768101642	0.972790461
18173	'Slc11a1'	41	61	15	45	61	108	39	71.33333333	0.929913722	0.115721128	0.702825844
18174	'Slc11a2'	1543	1624	1352	1027	1556	1332	1506.333333	1305	-0.159244469	0.542781407	0.972790461
18175	'Nrap'	30	40	0	1	53	26	23.33333333	26.66666667	0.17406237	0.907062427	0.988823459
18176	'Nras'	1941	1964	3485	768	1897	2003	2463.333333	1556	-0.747339642	0.125882073	0.72022881
18181	'Nrf1'	934	1016	807	502	891	914	919	769	-0.240431399	0.336897884	0.923457695
18183	'Nrg3'	0	0	0	0	1	0	0	0.333333333	1.020273531	0.802557913	0.972790461
18185	'Nrl'	0	0	0	0	1	0	0	0.333333333	1.020273531	0.802557913	0.972790461
18186	'Nrp1'	794	736	368	320	1096	847	632.6666667	754.3333333	0.25248635	0.581073502	0.972790461
18187	'Nrp2'	376	334	363	1464	305	395	357.6666667	721.3333333	1.309799652	0.096899918	0.663693139
18188	'Nrtn'	72	91	42	58	163	159	68.33333333	126.6666667	0.892092071	0.052770273	0.532526438
18189	'Nrxn1'	95	87	64	12	74	60	82	48.66666667	-0.812078495	0.174755599	0.793295795
18190	'Nrxn2'	193	169	116	69	128	146	159.3333333	114.3333333	-0.456775272	0.181719751	0.802050906
18191	'Nrxn3'	85	85	51	10	39	18	73.66666667	22.33333333	-1.711341925	0.00255963	0.113806392
18193	'Nsd1'	7821	7832	3364	3748	6430	5391	6339	5189.666667	-0.212589855	0.576263817	0.972790461
18194	'Nsdhl'	440	525	300	142	381	350	421.6666667	291	-0.536106764	0.15499695	0.766453805
18195	'Nsf'	621	642	493	135	655	812	585.3333333	534	-0.214456637	0.695503496	0.972790461
18196	'Nsg1'	629.78	710.77	470.89	42.31	784.5	831.56	603.8133333	552.79	-0.245598875	0.761400615	0.972790461
18197	'Nsg2'	9	3	8	1	8	15	6.666666667	8	0.131881251	0.906140759	0.988799253
18198	'Musk'	0	0	0	3	10	28	0	13.66666667	6.110589378	7.44E-04	0.054446012
18201	'Nsmaf'	1424	1458	780	905	2004	1595	1220.666667	1501.333333	0.333840426	0.33537253	0.922335099
18203	'Ntan1'	503	503	771	226	493	534	592.3333333	417.6666667	-0.563310767	0.1964114	0.822696823
18205	'Ntf3'	4	7	1	21	2	1	4	8	1.468572917	0.331128896	0.921648675
18207	'Nthl1'	230	184	136	72	186	238	183.3333333	165.3333333	-0.171298039	0.687339653	0.972790461
18208	'Ntn1'	94	57	96	36	66	53	82.33333333	51.66666667	-0.673512013	0.157133294	0.768337387
18209	'Ntn3'	340	331	50	19	432	297	240.3333333	249.3333333	0.010212929	0.991443827	0.999493374
18211	'Ntrk1'	28	15	17	3	8	8	20	6.333333333	-1.660374802	0.025764174	0.39630136

18212	'Ntrk2'	30	24	23	14	81	61	25.66666667	52	0.951213771	0.138450225	0.739741822
18213	'Ntrk3'	60	42	14	2	81	132	38.66666667	71.66666667	0.799984145	0.452828018	0.970649024
18214	'Ddr2'	245	203	421	160	311	529	289.6666667	333.3333333	0.104828904	0.847755553	0.975439569
18216	'Ntsr1'	60	57	18	4	52	21	45	25.66666667	-0.837646256	0.330223364	0.921648675
18218	'Dusp8'	117	120	719	657	127	178	318.6666667	320.6666667	0.174369065	0.866506038	0.978294615
18220	'Nucb1'	2591	2541	2627	5563	3174	5049	2586.333333	4595.333333	0.953928927	0.041231684	0.475995577
18221	'Nudc'	1970	2153	2220	2434	1896	3206	2114.333333	2512	0.313772657	0.426737775	0.961386251
18222	'Numb'	1812.6	1770.8	1435.1	2338.7	1986.2	1920.22	1672.83	2081.71	0.443434303	0.257091898	0.8773831
18223	'Numbl'	546	498	1633	632	678	867	892.3333333	725.6666667	-0.340070696	0.59570798	0.972790461
18226	'Nup62'	1372	1455	1124	1124	1324	1640	1317	1362.666667	0.106355901	0.685604101	0.972790461
18227	'Nr4a2'	71	74	161	745	65	95	102	301.6666667	1.883696008	0.07379052	0.599788529
18230	'Nxn'	727	725	700	475	653	740	717.3333333	622.6666667	-0.174555375	0.512988604	0.972790461
18231	'Nxph1'	0	1	1	1	3	1	0.666666667	1.666666667	1.307704765	0.530467353	0.972790461
18232	'Nxph2'	2	1	0	0	0	0	1	0	-2.286544758	0.567674219	0.972790461
18241	'Gpr143'	9	9	1	0	42	159	6.333333333	67	3.309349722	0.024832895	0.389862092
18242	'Oat'	3344	3598	4780	4194	3572	5299	3907.333333	4355	0.201866516	0.633842648	0.972790461
18245	'Oaz1'	4620	4890	4528	4563	4900	6051	4679.333333	5171.333333	0.202016075	0.495961159	0.972790461
18247	'Oaz2'	3238	3321	2450	2870	2815	3642	3003	3109	0.134233982	0.66152644	0.972790461
18256	'Oc90'	1	0	0	0	0	0	0.333333333	0	-0.903279821	0.824807108	0.972790461
18260	'Ocln'	61	37	35	74	92	190	44.33333333	118.6666667	1.429805765	0.00333248	0.131719543
18261	'Ocm'	7	2	1	0	2	6	3.333333333	2.666666667	-0.389503462	0.815201809	0.972790461
18263	'Odc1'	7602	7406	16098	25112	4265	6579	10368.66667	11985.33333	0.459248208	0.599795937	0.972790461
18285	'Odf1'	1	0	0	0	0	0	0.333333333	0	-0.903279821	0.824807108	0.972790461
18286	'Odf2'	1632	1619	1264	905	1671	1621	1505	1399	-0.08555123	0.729580869	0.972790461
18291	'Nobox'	139	100	12	0	270	1300	83.66666667	523.3333333	2.55495241	0.226023078	0.85360199
18293	'Ogdh'	3556.5	3386	1798	2743.7	3002	3547.82	2913.523333	3097.823333	0.188010955	0.586776651	0.972790461
18294	'Ogg1'	196.42	232.15	124.21	122.11	172.05	246.09	184.26	180.0833333	0.009865218	0.977756934	0.999493374
18295	'Ogn'	54	53	11	27	122	78	39.33333333	75.66666667	0.96364832	0.177497457	0.797090452
18300	'Oit1'	0	0	0	1	0	2	0	1	2.507928928	0.530239069	0.972790461
18301	'Fxyd5'	91	52	143	129	94	134	95.33333333	119	0.361381429	0.552524219	0.972790461
18302	'Oit3'	90	134	30	29	162	61	84.66666667	84	0.007872064	0.991354158	0.999493374
18310	'Olfr13'	1	1	0	0	0	1	0.666666667	0.333333333	-0.744986434	0.839556299	0.974723675
18312	'Olfr15'	1	1	0	0	1	0	0.666666667	0.333333333	-0.74517548	0.838722495	0.974723675
18315	'Olfr18'	0	0	1	0	0	0	0.333333333	0	-0.903279821	0.824807108	0.972790461
18330	'Olfr31'	0	0	0	0	0	3	0	1	2.274429265	0.570697589	0.972790461
18331	'Olfr32'	0	0	0	0	1	0	0	0.333333333	1.020273531	0.802557913	0.972790461
18332	'Olfr33'	0	0	0	0	0	1	0	0.333333333	1.020273531	0.802557913	0.972790461
18348	'Olfr49'	0	0	0	8	0	2	0	3.333333333	4.540848537	0.141665093	0.746904257
18349	'Olfr5'	0	0	0	0	0	2	0	0.666666667	1.749449488	0.66566965	0.972790461
18377	'Omg'	7	8	0	0	27	9	5	12	1.236243892	0.474555807	0.972790461

18378	'Omp'	2	3	1	4	3	3	2	3.333333333	0.878749862	0.508139633	0.972790461
18379	'Omt2a'	0	0	0	0	0	2	0	0.666666667	1.749449488	0.66566965	0.972790461
18383	'Tnfrsf11b'	16	18	133	28	23	37	55.66666667	29.33333333	-1.007036213	0.304662463	0.907164095
18386	'Oprd1'	78	73	10	196	109	123	53.66666667	142.6666667	1.666487424	0.031574178	0.431445911
18387	'Oprk1'	69	74	0	11	212	82	47.66666667	101.6666667	1.09270921	0.42153283	0.959296626
18389	'Oprl1'	73	89	7	8	99	105	56.33333333	70.66666667	0.298416429	0.764643643	0.972790461
18391	'Sigmar1'	391	387	325	574	561	652	367.6666667	595.6666667	0.781858992	0.01677208	0.323948168
18392	'Orc1'	385	375	179	37	205	260	313	167.3333333	-0.960445757	0.120013111	0.710364031
18393	'Orc2'	1255	1280	655	431	1041	1255	1063.333333	909	-0.225387547	0.572944552	0.972790461
18399	'Slc22a6'	0	1	0	0	0	0	0.333333333	0	-0.903279821	0.824807108	0.972790461
18400	'Slc22a18'	8	12	8	1	15	19	9.333333333	11.66666667	0.220164304	0.825141973	0.972790461
18405	'Orm1'	0	0	0	1	0	0	0	0.333333333	1.020273531	0.802557913	0.972790461
18406	'Orm2'	1	0	1	0	0	1	0.666666667	0.333333333	-0.894127833	0.804654786	0.972790461
18407	'Orm3'	0	1	2	0	1	0	1	0.333333333	-1.522939023	0.64403203	0.972790461
18408	'Slc25a15'	342	340	266	147	378	649	316	391.3333333	0.253679805	0.588176075	0.972790461
18412	'Sqstm1'	4070	4067	29285	13697	5509	10614	12474	9940	-0.313080838	0.737300464	0.972790461
18413	'Osm'	3	3	6	7	0	0	4	2.333333333	-0.400466322	0.839657152	0.974723675
18414	'Osmr'	105	68	1301	1200	147	331	491.3333333	559.3333333	0.329247699	0.790142507	0.972790461
18415	'Hspa4l'	1418	1508	629	24488	995	5176	1185	10219.66667	3.532030924	6.98E-04	0.052467355
18416	'Otc'	3	4	1	0	5	8	2.666666667	4.333333333	0.62466115	0.684329142	0.972790461
18417	'Cldn11'	67	73	123	66	75	64	87.66666667	68.33333333	-0.328568944	0.535394684	0.972790461
18419	'Otog'	7	11	3	0	10	6	7	5.333333333	-0.449466208	0.728618399	0.972790461
18420	'Otp'	4	3	2	0	1	0	3	0.333333333	-2.98147375	0.152815197	0.763754606
18422	'Ott'	0	5.06	0	0	1.66	0	1.686666667	0.553333333	-2.046244302	0.605319211	0.972790461
18423	'Otx1'	19	22	5	8	10	3	15.33333333	7	-0.955891657	0.308333986	0.908027422
18424	'Otx2'	0	0	0	2	1	0	0	1	2.732929573	0.492631579	0.972790461
18426	'Ovol1'	43	58	77	14	54	103	59.33333333	57	-0.190622298	0.782847507	0.972790461
18429	'Oxt'	0	1	2	1	1	1	1	1	-0.008673646	0.996763263	0.999616639
18430	'Oxtr'	10	12	11	21	8	12	11	13.66666667	0.487530603	0.555131773	0.972790461
18431	'Oca2'	47	27	1	4	29	19	25	17.33333333	-0.507806636	0.659045346	0.972790461
18432	'Mybbp1a'	3403	3478	4207	1305	3396	3590	3696	2763.666667	-0.478142911	0.231835839	0.858905111
18436	'P2rx1'	14	9	1	1	7	6	8	4.666666667	-0.762126279	0.535564479	0.972790461
18438	'P2rx4'	271	288	388	214	422	559	315.6666667	398.3333333	0.281489451	0.504169067	0.972790461
18439	'P2rx7'	165	125	62	111	259	179	117.3333333	183	0.690222932	0.132222123	0.729657304
18440	'P2rx6'	46	56	9	4	91	70	37	55	0.524555855	0.596324361	0.972790461
18441	'P2ry1'	47	34	38	11	38	47	39.66666667	32	-0.366729288	0.52353956	0.972790461
18442	'P2ry2'	38	47	343	243	18	87	142.6666667	116	-0.144218708	0.90268244	0.987331639
18451	'P4ha1'	3130	2424	6117	6270	1993	4039	3890.333333	4100.666667	0.205554539	0.773864973	0.972790461
18452	'P4ha2'	980	555	2516	1133	375	899	1350.333333	802.3333333	-0.680258425	0.392793023	0.952648938
18453	'P4hb'	9712	8264	48375	9712	7981	13628	22117	10440.33333	-1.152450599	0.152122408	0.762497025

18457	'Bloc1s6'	880	878	879	306	712	931	879	649.6666667	-0.481463961	0.203077815	0.83089164
18458	'Pabpc1'	19794	18238	35881	13463	10933	13169	24637.66667	12521.66667	-0.929618953	0.074455973	0.603391716
18472	'Pafah1b1'	5668	5822	7160	3276	5409	6110	6216.666667	4931.666667	-0.34821792	0.298731136	0.902535634
18475	'Pafah1b2'	3250	3307	3968	2899	2924	3815	3508.333333	3212.666667	-0.084044052	0.81604186	0.972790461
18476	'Pafah1b3'	875	1007	2088	445	918	1042	1323.333333	801.6666667	-0.811137482	0.133632867	0.733301819
18477	'Prdx1'	6749	7591	6075	10468	7834	16025	6805	11442.33333	0.818565785	0.038158736	0.459930654
18478	'Pah'	0	0	1	3	2	0	0.333333333	1.666666667	2.467305623	0.389635274	0.951231321
18479	'Pak1'	1419	1419	835	1989	936	1120	1224.333333	1348.333333	0.362650811	0.517864411	0.972790461
18481	'Pak3'	478	539	1364	1106	1152	614	793.6666667	957.3333333	0.31462598	0.637544764	0.972790461
18483	'Palm'	1234	1131	1396	953	1311	1716	1253.666667	1326.666667	0.082974773	0.801861527	0.972790461
18484	'Pam'	4503	4790	5870	7022	5168	6262	5054.333333	6150.666667	0.378521341	0.398365484	0.954563638
18488	'Cntn3'	71	85	9	5	86	34	55	41.66666667	-0.413431706	0.68121237	0.972790461
18489	'Reg3b'	22	7	24	288	0	1	17.66666667	96.33333333	2.882719042	0.240986863	0.86449675
18491	'Pappa'	242	225	745	366	317	474	404	385.6666667	-0.093590334	0.888117419	0.984534762
18503	'Pax1'	0	1	1	0	0	0	0.666666667	0	-1.851176388	0.646290011	0.972790461
18504	'Pax2'	6	8	1	13	3	4	5	6.666666667	0.745948042	0.549632625	0.972790461
18505	'Pax3'	0	1	1	1	0	0	0.666666667	0.333333333	-0.885963919	0.798167427	0.972790461
18506	'Pax4'	0	0	0	0	0	1	0	0.333333333	1.020273531	0.802557913	0.972790461
18507	'Pax5'	19	18	3	0	12	3	13.33333333	5	-1.437130537	0.28050143	0.889355605
18508	'Pax6'	23.87	24.74	13.82	8.46	4.32	2.18	20.81	4.986666667	-1.889480569	0.036016624	0.456194572
18509	'Pax7'	3	0	0	0	0	0	1	0	-2.292035055	0.566710385	0.972790461
18510	'Pax8'	124	97	171	441	161	220	130.6666667	274	1.246094421	0.066139458	0.575225699
18511	'Pax9'	13	12	8	6	16	9	11	10.33333333	-0.061750855	0.934126201	0.994249017
18514	'Pbx1'	2915	2860	2401	2186	2868	2831	2725.333333	2628.333333	0.005962628	0.981140032	0.999493374
18515	'Pbx2'	1549	1634	1701	593	1647	1456	1628	1232	-0.444737741	0.238594171	0.864053647
18516	'Pbx3'	1981	2075	781	429	1750	1245	1612.333333	1141.333333	-0.498554342	0.34384153	0.925308103
18518	'Igbp1'	707	700	1096	807	829	964	834.3333333	866.6666667	0.085132533	0.845188204	0.975182082
18519	'Kat2b'	371	381	452	358	595	619	401.3333333	524	0.380418652	0.251304211	0.871230154
18521	'Pcbp2'	11990	11782	11265	10423	8078.3	10889.3	11678.88333	9796.77	-0.154522754	0.685281211	0.972790461
18526	'Pcdh10'	16	17	13	81	18	20	15.33333333	39.66666667	1.681140768	0.0627825	0.566066012
18530	'Pcdh8'	49	34	8	17	46	4	30.33333333	22.33333333	-0.311993697	0.754507244	0.972790461
18534	'Pck1'	8	5	4	14	1	8	5.666666667	7.666666667	0.68886616	0.565860855	0.972790461
18536	'Pcm1'	5669	5262	3797	2015	4464	4362	4909.333333	3613.666667	-0.436697346	0.14581653	0.753611197
18537	'Pcmt1'	1218	1285	1133	1211	1183	1376	1212	1256.666667	0.13292324	0.681676907	0.972790461
18538	'Pcna'	1867	2049	1935	1087	1809	2103	1950.333333	1666.333333	-0.22568227	0.414785451	0.95722888
18541	'Pcnt'	1362	1311	619	838	1518	1129	1097.333333	1161.666667	0.15511194	0.681575156	0.972790461
18542	'Pcolce'	1242	1146	793	1936	1498	2036	1060.333333	1823.333333	0.905103452	0.018915298	0.342493002
18545	'Pcp2'	1	0	0	0	0	0	0.333333333	0	-0.903279821	0.824807108	0.972790461
18546	'Pcp4'	4	2	4	0	1	10	3.333333333	3.666666667	-0.037073279	0.982011912	0.999493374
18548	'Pcsk1'	2	3	1	6	2	0	2	2.666666667	0.765010358	0.663545772	0.972790461

18549	'Pcsk2'	2	8	4	28	1	5	4.666666667	11.33333333	1.662319789	0.233560941	0.860393388
18550	'Furin'	1362	1193	1070	3595	2093	2779	1208.333333	2822.333333	1.378795538	0.00339257	0.132988763
18551	'Pcsk4'	67	63	115	26	86	61	81.66666667	57.66666667	-0.581994794	0.311984362	0.911537035
18552	'Pcsk5'	544	493	406	507	522	468	481	499	0.157480051	0.657229425	0.972790461
18553	'Pcsk6'	3189	3297	406	125	4988	3754	2297.333333	2955.666667	0.313415368	0.761658543	0.972790461
18554	'Pcsk7'	1014.1	1016.3	347.98	768.73	725.86	597.67	792.7833333	697.42	-0.011638789	0.981840603	0.999493374
18555	'Cdk16'	4331	4579	3666	2117	4975	5096	4192	4062.666667	-0.060916094	0.846055055	0.975182082
18557	'Cdk18'	125	114	116	175	142	158	118.3333333	158.3333333	0.524821267	0.218293017	0.844387594
18559	'Pctp'	166	151	40	77	130	208	119	138.3333333	0.266656117	0.644056979	0.972790461
18563	'Pcx'	2028	2024	732	1614	1033	1366	1594.666667	1337.666667	-0.062888898	0.905318294	0.988451813
18566	'Pdcd1'	0	1	0	0	2	0	0.333333333	0.666666667	0.839717162	0.835573887	0.974723675
18567	'Pdcd2'	337	371	520	278	385	547	409.3333333	403.3333333	-0.04454899	0.914202545	0.989964366
18569	'Pdcd4'	3582.2	3966.6	2406.5	1555.1	3645.5	3957.38	3318.43	3052.65	-0.122173646	0.718617586	0.972790461
18570	'Pdcd6'	644	691	1459	756	827	979	931.3333333	854	-0.133568687	0.8014821	0.972790461
18571	'Pdcd6ip'	4099	4242	4917	3718	3608	4636	4419.333333	3987.333333	-0.096103261	0.790844446	0.972790461
18572	'Pdcd11'	1887	1970	1070	1169	1700	1839	1642.333333	1569.333333	-0.001928614	0.994835822	0.999562152
18573	'Pde1a'	1219	1248	465	197	1560	1458	977.3333333	1071.666667	0.080126842	0.905387213	0.988470294
18574	'Pde1b'	98	83	304	98	87	97	161.6666667	94	-0.789083231	0.2635785	0.88013169
18575	'Pde1c'	39	42	34	7	18	11	38.33333333	12	-1.659012656	0.003238523	0.129350407
18576	'Pde3b'	533	571	324	74	335	187	476	198.6666667	-1.279092535	0.011684262	0.268550885
18577	'Pde4a'	151	153	332	182	116	174	212	157.3333333	-0.38011901	0.53246045	0.972790461
18578	'Pde4b'	833.26	696.3	751.89	438.91	570.12	414.23	760.4833333	474.42	-0.611569731	0.090229884	0.648589738
18582	'Pde6d'	720	747	791	603	641	851	752.6666667	698.3333333	-0.06542144	0.842952989	0.975182082
18583	'Pde7a'	1635	1603	725	378	1028	909	1320.993333	771.6666667	-0.758034071	0.073641215	0.599721598
18584	'Pde8a'	860	831	840	354	789	844	843.6666667	662.3333333	-0.375271905	0.255381712	0.875162567
18585	'Pde9a'	59	56	150	61	49	33	88.33333333	47.66666667	-0.823546297	0.250888582	0.871167957
18587	'Pde6b'	0	0	1	0	0	1	0.333333333	0.333333333	0.058500858	0.988561293	0.999493374
18588	'Pde6g'	10	3	3	0	6	3	5.333333333	3	-0.890140673	0.532546502	0.972790461
18590	'Pdgfa'	463	460	571	698	441	657	498	598.6666667	0.365296161	0.441108938	0.968551476
18591	'Pdgfb'	314	277	586	154	711	491	392.3333333	452	0.08004479	0.895478683	0.985505807
18595	'Pdgfra'	2998	2597	942	7180	2422	2027	2179	3876.333333	1.164644409	0.123492194	0.715310266
18596	'Pdgfrb'	1150	1044	324	1056	1241	1011	839.3333333	1102.666667	0.541387077	0.289502124	0.896711368
18597	'Pdha1'	1425.5	1431.5	1759.2	1194.5	1437	1475.72	1538.733333	1369.086667	-0.131339019	0.70714388	0.972790461
18598	'Pdha2'	57	65	19	0	45	55	47	33.33333333	-0.587160445	0.617357679	0.972790461
18599	'Padi1'	9	10	4	60	22	32	7.666666667	38	2.551300754	0.001779695	0.09046942
18600	'Padi2'	22	17	4	50	22	56	14.33333333	42.66666667	1.754939467	0.025931192	0.397137149
18601	'Padi3'	3	4	3	114	4	18	3.333333333	45.33333333	4.163864065	0.001139395	0.070791417
18602	'Padi4'	98	147	2	12	12	3	82.33333333	9	-2.92323429	0.01493313	0.305110509
18604	'Pdk2'	715	675	675	327	781	734	688.3333333	614	-0.187892874	0.572976355	0.972790461
18605	'Enpp1'	507	416	219	585	268	266	380.6666667	373	0.225732479	0.717707378	0.972790461

18606	'Enpp2'	1119	970	1403	15386	3032	2989	1164	7135.666667	2.918450879	5.75E-04	0.047144852
18607	'Pdpk1'	3452	3357	2620	2243	1897	2610	3143	2250	-0.383914194	0.26828931	0.883600995
18610	'Pdyn'	48	39	176	737	89	207	87.66666667	344.3333333	2.182512277	0.032501227	0.434844008
18611	'Pea15a'	1000	1017	1763	2200	1261	1274	1260	1578.333333	0.450308088	0.450703102	0.970590732
18612	'Etv4'	94	124	219	244	103	142	145.6666667	163	0.294674416	0.668494695	0.972790461
18613	'Pecam1'	744	735	174	46	1102	1197	551	781.6666667	0.434955063	0.637721008	0.972790461
18616	'Peg3'	38876	38999	5664	16747	26105	11327	27846.33333	18059.66667	-0.453941602	0.520292762	0.972790461
18617	'Rhox5'	41	34	45	32	34	68	40	44.66666667	0.158362695	0.758271684	0.972790461
18618	'Pemt'	114	95	39	16	49	92	82.66666667	52.33333333	-0.685393806	0.286774433	0.894529207
18619	'Penk'	56	43	41	98	83	61	46.66666667	80.66666667	0.930224226	0.077186226	0.611268953
18624	'Pepd'	564	591	818	402	575	700	657.6666667	559	-0.246187081	0.521574001	0.972790461
18626	'Per1'	543	441	2345	1302	425	532	1109.666667	753	-0.443067247	0.623281913	0.972790461
18627	'Per2'	131	131	218	69	122	93	160	94.66666667	-0.772651049	0.106100251	0.679124905
18628	'Per3'	1012	915	171	86	781	749	699.3333333	538.6666667	-0.408410483	0.611091355	0.972790461
18630	'Dcaf8l'	988	916	86	6	649	1005	663.3333333	553.3333333	-0.328152887	0.78723617	0.972790461
18631	'Pex11a'	310	305	273	174	280	307	296	253.6666667	-0.203553692	0.452710903	0.970649024
18632	'Pex11b'	558	602	624	222	533	690	594.6666667	481.6666667	-0.356727705	0.369526971	0.937712122
18633	'Pex16'	294	297	207	165	228	278	266	223.6666667	-0.205204803	0.465833719	0.972790461
18634	'Pex7'	652	679	396	378	511	606	575.6666667	498.3333333	-0.147248764	0.615935413	0.972790461
18636	'Cfp'	81	112	53	20	146	100	82	88.66666667	0.061217322	0.924942718	0.992381554
18637	'Pfdn2'	819.5	916.04	1365.9	1881.9	699.76	1353.3	1033.81	1311.643333	0.48143307	0.440915851	0.968551476
18639	'Pfkfb1'	135	146	38	11	62	62	106.3333333	45	-1.256584498	0.073211341	0.598921693
18640	'Pfkfb2'	801	824	664	962	812	525	763	766.3333333	0.165105192	0.732640625	0.972790461
18641	'Pfkl'	5175	4309	16649	3374	3333	5237	8711	3981.333333	-1.188393274	0.079944038	0.617305615
18642	'Pfkm'	1574	1726	1058	1182	1830	1450	1452.666667	1487.333333	0.104325518	0.726453575	0.972790461
18643	'Pfn1'	3155.5	3183.2	5675.8	2323.5	3439.1	4556.96	4004.846667	3439.87	-0.262966378	0.566720911	0.972790461
18645	'Pfn2'	2077	2198	1193	1010	1934	2329	1822.666667	1757.666667	-0.031335042	0.925064062	0.992381554
18646	'Prf1'	1	0	0	0	0	1	0.333333333	0.333333333	0.058500858	0.988561293	0.999493374
18647	'Cdk14'	1255	1345	852	164	1397	1331	1150.666667	964	-0.343252831	0.595473529	0.972790461
18648	'Pgam1'	7613	7004	37168	11902	5115	10523	17261.66667	9180	-0.894700899	0.289086947	0.896301537
18654	'Pgf'	625	564	716	161	1041	615	635	605.6666667	-0.168270422	0.775254088	0.972790461
18655	'Pgk1'	13964	11035	97341	12554	5531	14326	40780	10803.66667	-1.956797868	0.037804749	0.459930654
18667	'Pgr'	158	179	125	24	170	105	154	99.66666667	-0.693123981	0.251978023	0.871252558
18669	'Abcb1b'	39.6	32.64	270.43	67.43	30.16	42.17	114.2233333	46.58666667	-1.2690093	0.201478472	0.82952222
18670	'Abcb4'	48	40	19	10	31	52	35.66666667	31	-0.226142861	0.729335674	0.972790461
18671	'Abcb1a'	248.4	254.36	154.57	106.57	325.84	254.83	219.11	229.08	0.058355007	0.886894623	0.984522884
18673	'Phb'	1789	2010	2432	2193	2062	3077	2077	2444	0.270767295	0.489693893	0.972790461
18674	'Slc25a3'	4551	4620	7746	10617	4959	7370	5639	7648.666667	0.563732776	0.345382827	0.926100052
18675	'Phex'	22	19	4	7	12	3	15	7.333333333	-0.880780341	0.366124329	0.936406787
18676	'Phf2'	1643	1507	1775	1012	1585	1601	1641.666667	1399.333333	-0.22100883	0.466372083	0.972790461

18679	'Phka1'	1239	1132	248	107	815	655	873	525.6666667	-0.753816405	0.309448635	0.910300259
18682	'Phkg1'	7	2	1	6	5	9.15	3.333333333	6.716666667	1.105376669	0.337929272	0.924020038
18685	'Phtf1'	1302	1283	441	278	894	787	1008.666667	653	-0.612572733	0.231769806	0.858905111
18693	'Pick1'	669	642	369	206	526	611	560	447.6666667	-0.329662667	0.406270055	0.957157474
18700	'Piga'	609	538	2484	406	420	499	1210.333333	441.6666667	-1.508142397	0.040957981	0.474812891
18701	'Pigf'	227.74	235.83	132.19	202	170.08	210.2	198.5866667	194.0933333	0.095865829	0.813569154	0.972790461
18704	'Pik3c2a'	2924	3258	1921	4281	3300	3300	2701	3627	0.583284758	0.174378066	0.793128181
18705	'Pik3c2g'	11	11	9	7	20	21	10.33333333	16	0.611176508	0.375889237	0.940406075
18706	'Pik3ca'	2585.7	2399.6	2015.1	2726	2120.5	2312.33	2333.466667	2386.266667	0.162296296	0.684994263	0.972790461
18707	'Pik3cd'	123	119	53	48	124	215	98.33333333	129	0.371991477	0.497811033	0.972790461
18708	'Pik3r1'	1976	2193	1546	1082	2415	1923	1905	1806.666667	-0.062006472	0.836370075	0.974723675
18709	'Pik3r2'	1537	1566	1265	1505	1907	2135	1456	1849	0.398760449	0.105367167	0.678099427
18710	'Pik3r3'	5825	6227	664	2101	5218	3627	4238.666667	3648.666667	-0.126991626	0.86033818	0.976863639
18711	'Pikfyve'	2356	2402	765	2507	1598	1369	1841	1824.666667	0.223757405	0.708718937	0.972790461
18712	'Pim1'	481	493	2278	2520	426	778	1084	1241.333333	0.372681113	0.705101474	0.972790461
18715	'Pim2'	219	227	1743	147	169	203	729.6666667	173	-2.181776249	0.014853975	0.303986836
18717	'Pip5k1c'	1247	1200	604	765	1243	1090	1017	1032.666667	0.093828272	0.784288314	0.972790461
18718	'Pip4k2a'	390	437	364	575	377	446	397	466	0.37402207	0.410924457	0.95722888
18719	'Pip5k1b'	139	158	420	234	106	204	239	181.3333333	-0.34098755	0.634414262	0.972790461
18720	'Pip5k1a'	1425	1460	3154	2940	1523	2041	2013	2168	0.203306894	0.749373644	0.972790461
18722	'Pira1'	4	0	0	0	0	2.47	1.333333333	0.823333333	-1.013778784	0.797196166	0.972790461
18725	'Pira2'	0	1.18	0	0	3.98	6.27	0.393333333	3.416666667	2.917837912	0.272784627	0.884738928
18726	'Lilra6'	0	3	0	0	0	0	1	0	-2.275512037	0.569613276	0.972790461
18733	'Pirb'	1	3.01	0	13	11.59	39.13	1.336666667	21.24	4.020856856	8.08E-04	0.058366224
18736	'Pou1f1'	1	0	2	1	0	0	1	0.333333333	-1.526634549	0.634193714	0.972790461
18738	'Pitpna'	2028	2093	1316	1548	2223	2354	1812.333333	2041.666667	0.230156542	0.374588173	0.940406075
18739	'Pitpnm1'	159	161	169	705	173	282	163	386.6666667	1.498606422	0.038284726	0.459930654
18740	'Pitx1'	4	3	4	6	4	3	3.666666667	4.333333333	0.368878018	0.742777135	0.972790461
18741	'Pitx2'	2	1	2	11	2	3	1.666666667	5.333333333	1.935770881	0.175842009	0.795298979
18742	'Pitx3'	1	0	4	4	0	0	1.666666667	1.333333333	-0.026322601	0.992505552	0.999493374
18744	'Pja1'	2282	2373	2330	2254	2619	3154	2328.333333	2675.666667	0.245423452	0.398260788	0.954563638
18746	'Pkm'	21816	18714	89572	39670	15504	33015	43367.33333	29396.33333	-0.523944043	0.524422668	0.972790461
18747	'Prkaca'	1820	1874	1909	2639	1864	2473	1867.666667	2325.333333	0.424778562	0.313171123	0.912356687
18749	'Prkacb'	2716	2634	3169	1983	3002	3864	2839.666667	2949.666667	0.044936054	0.891203595	0.985096898
18750	'Prkca'	276	256	309	356	308	381	280.3333333	348.3333333	0.386751312	0.337313857	0.92380462
18751	'Prkcb'	1212	1210	206	1430	1222	1677	876	1443	0.888739872	0.163961853	0.776592614
18752	'Prkcg'	35	26	10	42	18	15	23.66666667	25	0.356778005	0.666404175	0.972790461
18753	'Prkcd'	1810	1811	2025	2149	2156	2472	1882	2259	0.32540598	0.353384656	0.930050349
18754	'Prkce'	666	582	270	293	639	754	506	562	0.173419335	0.681070104	0.972790461
18755	'Prkch'	165	168	79	170	178	159	137.3333333	169	0.430138485	0.320040821	0.917050664

18759	'Prkci'	1331	1411	1635	2528	1790	1988	1459	2102	0.641044108	0.164451805	0.777557251
18760	'Prkd1'	991	1066	715	1599	1031	1255	924	1295	0.647288908	0.150905573	0.759755159
18761	'Prkcq'	12	24	11	0	6	6	15.66666667	4	-2.033424916	0.052480559	0.532138871
18762	'Prkcz'	315	354	482	80	254	342	383.6666667	225.3333333	-0.870074636	0.096935898	0.663693139
18763	'Pkd1'	3252	2987.6	992.77	2244.6	3639.1	3141	2410.78	3008.253333	0.414990174	0.369863772	0.937712122
18764	'Pkd2'	1514	1514	761	6834	3207	3277	1263	4439.333333	2.061593263	5.36E-04	0.045614454
18766	'Pkdrej'	80	81	12	5	98	54	57.66666667	52.33333333	-0.170974972	0.86058817	0.976863639
18767	'Pkia'	205	201	626	462	311	271	344	348	0.08248333	0.908323584	0.988823459
18768	'Pkib'	0	0	4	1	3	18	1.333333333	7.333333333	2.153018206	0.277388364	0.889176796
18769	'Pkig'	1063	1082	733	604	1094	1296	959.3333333	998	0.073023261	0.79600389	0.972790461
18770	'Pklr'	45	57	4	2	8	2	35.33333333	4	-3.045020923	0.005551068	0.175894852
18771	'Pknox1'	787	799	1043	311	731	900	876.3333333	647.3333333	-0.500872843	0.233142751	0.859679981
18772	'Pkp1'	8	9	145	1	2	2	54	1.666666667	-5.195861407	1.75E-04	0.020572308
18777	'Lypla1'	1014	1079	780	250	1020	1148	957.6666667	806	-0.311382347	0.523020094	0.972790461
18778	'Pla2g1b'	28	32	4	15	63	30	21.33333333	36	0.803322908	0.338644953	0.924608313
18779	'Pla2r1'	767	875	42	135	2120	884	561.3333333	1046.333333	0.893939899	0.385002813	0.94674509
18781	'Pla2g2c'	51	41	8	1	29	32	33.33333333	20.66666667	-0.742868856	0.480273537	0.972790461
18782	'Pla2g2d'	0	2	0	0	1	3	0.666666667	1.333333333	0.950087202	0.756799145	0.972790461
18783	'Pla2g4a'	575	670	1433	5420	857	1072	892.6666667	2449.666667	1.724773915	0.062563298	0.566066012
18784	'Pla2g5'	256	294	42	17	325	462	197.3333333	268	0.381966797	0.694179591	0.972790461
18786	'Plaa'	3092	3130	3409	1773	2347	2996	3210.333333	2372	-0.422059137	0.161701654	0.771541907
18787	'Serpine1'	71	38	1132	3351	73	242	413.6666667	1222	1.809085375	0.227833545	0.854324922
18788	'Serpinb2'	3	0	373	4	1	4	125.3333333	3	-5.466163068	0.00300071	0.122531121
18789	'Papola'	6274	6685	5021	5869	7316	6159	5993.333333	6448	0.189262328	0.529067963	0.972790461
18791	'Plat'	1011	1044	1143	69629	1272	5031	1066	25310.66667	4.996868038	3.86E-05	0.007055669
18792	'Plau'	741	741	317	2971	990	960	599.6666667	1640.333333	1.764867648	0.0137647	0.292512191
18793	'Plaur'	57	57	83	730	86	296	65.66666667	370.6666667	2.751295219	0.0014881	0.083456524
18795	'Plcb1'	104	117.01	84	17	156	133	101.67	102	-0.087132189	0.897538857	0.985794067
18796	'Plcb2'	66	56	4	18	149	45	42	70.66666667	0.777730687	0.435642088	0.96581102
18797	'Plcb3'	1774	1880	1073	1378	2365	2219	1575.666667	1987.333333	0.384734137	0.181063212	0.801670656
18798	'Plcb4'	811	755	1575	179	712	868	1047	586.3333333	-0.980457837	0.110041586	0.689784826
18799	'Plcd1'	859	864	1256	937	855	1070	993	954	-0.011152165	0.979438347	0.999493374
18802	'Plcd4'	9	10	4	10	4	1	7.666666667	5	-0.314047355	0.792063469	0.972790461
18803	'Plcg1'	2588	2589	1550	2251	2643	2345	2242.333333	2413	0.210982378	0.526949301	0.972790461
18805	'Pld1'	607	680	1055	1032	565	577	780.6666667	724.6666667	0.029533507	0.960643775	0.999493374
18806	'Pld2'	460	406	1015	334	574	495	627	467.6666667	-0.473666916	0.38919141	0.951190011
18807	'Pld3'	1498	1678	1076	3975	2602	2855	1417.333333	3144	1.3194525	0.004204294	0.149966291
18810	'Plec'	3289	2986	2748	3138	5231	4008	3007.666667	4125.666667	0.496785558	0.080069909	0.617305615
18811	'Prl2c2'	0	0	0	9	1	5	0	5	5.005249665	0.026254052	0.397367216
18812	'Prl2c3'	0	0	1	3	0	0	0.333333333	1	1.937497013	0.625951132	0.972790461

18813	'Pa2g4'	2306	2353	3341	1017	2235	2789	2666.666667	2013.666667	-0.471166049	0.270056111	0.883851074
18816	'Serpinf2'	3	2	5	3	2	2	3.333333333	2.333333333	-0.449780912	0.731232044	0.972790461
18817	'Plk1'	390	403	93	90	337	268	295.3333333	231.6666667	-0.328050206	0.596572239	0.972790461
18821	'Pln'	54	57	2	1	83	42	37.66666667	42	0.126740763	0.922376524	0.991300958
18822	'Plod1'	1446	1193	1414	1013	812	861	1351	895.3333333	-0.48745027	0.24934458	0.869928149
18823	'Plp1'	613	705	119	17	647	421	479	361.6666667	-0.459354638	0.643584954	0.972790461
18824	'Plp2'	1343.6	1209.5	2559.5	1936.9	1352.9	1657.82	1704.2	1649.186667	0.016215613	0.977114921	0.999493374
18826	'Lcp1'	307	343	262	173	628	803	304	534.6666667	0.748471212	0.123375262	0.715310266
18828	'Plscr2'	100	102	172	58	69	119	124.6666667	82	-0.636047839	0.211747998	0.840560909
18829	'Ccl21a'	0	1.31	1.11	0	8.71	10.78	0.806666667	6.496666667	2.997693216	0.134586076	0.734639815
18830	'Pltp'	68.48	93.28	65.73	90.72	90.02	112.14	75.83	97.62666667	0.445420812	0.255275073	0.875097312
18844	'Plxna1'	2924	3075	686	3329	4419	4557	2228.333333	4101.666667	1.001906783	0.06551838	0.574555093
18845	'Plxna2'	1438	1485	235	895	2013	1168	1052.666667	1358.666667	0.471038503	0.471013717	0.972790461
18846	'Plxna3'	980	878	387	532	1122	706	748.3333333	786.6666667	0.141759411	0.744091506	0.972790461
18854	'Pml'	790	860	735	655	1010	974	795	879.6666667	0.179502383	0.472998105	0.972790461
18857	'Pmp2'	0	0	1	0	0	0	0.333333333	0	-0.903279821	0.824807108	0.972790461
18858	'Pmp22'	794	719	471	160	943	1035	661.3333333	712.6666667	0.034801135	0.952491547	0.998773884
18861	'Pms2'	1205	1370	670	129	915	567	1081.666667	537	-1.057790685	0.080132416	0.617305615
18933	'Prrx1'	383	399	733	564	561	340	505	488.3333333	0.023435488	0.967407912	0.999493374
18938	'Ppp1r14b'	886	954	1850	778	1266	1482	1230	1175.333333	-0.120005605	0.808328691	0.972790461
18946	'Pnliprp1'	4	4	0	0	12	18	2.666666667	10	1.8478292	0.280162332	0.889355605
18947	'Pnliprp2'	4	3	0	1	7	9	2.333333333	5.666666667	1.269834299	0.391975675	0.952648938
18948	'Pnmt'	2	3	2	0	15	3	2.333333333	6	1.262932327	0.427051065	0.961636249
18949	'Pnn'	2853	2982	2512	1920	3270	2425	2782.333333	2538.333333	-0.090557179	0.751928285	0.972790461
18950	'Pnp'	426.67	539.41	586.62	504.7	680.64	784.79	517.5666667	656.71	0.357185918	0.302310547	0.905122538
18951	'Septin5'	216.66	197.58	822.68	289.09	137.4	296.66	412.3066667	241.05	-0.763818335	0.330593792	0.921648675
18952	'Septin4'	76	83	22	95	117	97	60.33333333	103	0.907683396	0.101810474	0.671776916
18968	'Pola1'	759	831	339	308	682	749	643	579.6666667	-0.122478632	0.768604414	0.972790461
18969	'Pola2'	456	448	355	313	463	382	419.6666667	386	-0.061961984	0.828180058	0.973238667
18970	'Polb'	405.24	409	410.2	243.98	338	384.91	408.1466667	322.2966667	-0.3182216	0.267618582	0.883600995
18971	'Pold1'	900	951.04	485.03	542.87	866.94	994	778.69	801.27	0.090239485	0.780103181	0.972790461
18972	'Pold2'	553	603	925	282	481	816	693.6666667	526.3333333	-0.468944849	0.327127356	0.920734892
18973	'Pole'	2269	2207	363	53	1320	857	1613	743.3333333	-1.163284279	0.217083422	0.84361198
18974	'Pole2'	126	136	43	75	168	138	101.6666667	127	0.377460499	0.451571292	0.970649024
18975	'Polg'	1428	1426.1	814.75	1139.5	1475.9	1518.35	1222.943333	1377.903333	0.25381121	0.403358184	0.955996639
18976	'Pomc'	36	45	35	8	31	34	38.66666667	24.33333333	-0.715515631	0.217985976	0.84418897
18979	'Pon1'	1	0	0	0	0	0	0.333333333	0	-0.903279821	0.824807108	0.972790461
18983	'Cnot7'	1939.9	2116.3	3937.6	1534.3	2850.7	2051.63	2664.583333	2145.553333	-0.345270974	0.486336587	0.972790461
18984	'Por'	958	986	1161	4404	1202	1683	1035	2429.666667	1.473479821	0.037093248	0.459930654
18985	'Pou2af1'	1	3	1	0	4	0	1.666666667	1.333333333	-0.365461813	0.874415991	0.980175262

18986	'Pou2f1'	12154	12291	2936.1	2998.5	15891	9248.41	9127.043333	9379.233333	0.047386026	0.94226694	0.996294926
18987	'Pou2f2'	29	21	49	36	13	9	33	19.33333333	-0.57360693	0.520912891	0.972790461
18988	'Pou2f3'	12	7	2	18	5	7	7	10	0.811737182	0.456441593	0.97151713
18991	'Pou3f1'	68	69	24	24	14	20	53.66666667	19.33333333	-1.296948442	0.045343527	0.496605985
18992	'Pou3f2'	3	6	2	1	1	0	3.666666667	0.666666667	-2.301694984	0.192060943	0.813837363
18993	'Pou3f3'	3	3	3	117	2	3	3	40.66666667	4.217661332	0.00545982	0.174480089
18994	'Pou3f4'	3	3	13	8	2	0	6.333333333	3.333333333	-0.701564509	0.662527799	0.972790461
18996	'Pou4f1'	266	251	40	4	121	173	185.6666667	99.33333333	-0.967483519	0.347316454	0.928387732
18997	'Pou4f2'	1	3	0	1	1	1	1.333333333	1	-0.259075872	0.901185859	0.98672375
18998	'Pou4f3'	2	0	0	0	0	0	0.666666667	0	-1.711373851	0.67234292	0.972790461
18999	'Pou5f1'	32	32	78	0	16	171	47.33333333	62.33333333	0.131346311	0.925867722	0.992381554
19009	'Pou6f1'	552	447	457	251	547	466	485.3333333	421.3333333	-0.206107832	0.521133451	0.972790461
19011	'Endou'	2	2	1	8	4	2	1.666666667	4.666666667	1.722666962	0.210985084	0.840335617
19012	'Plpp1'	276	250	193	1081	345	339	239.6666667	588.3333333	1.580224481	0.025029211	0.3916505
19013	'Ppara'	399	404	108	206	522	427	303.6666667	385	0.394412132	0.463183793	0.972790461
19014	'Med1'	2819	2873	1692	1045	1993	1625	2461.333333	1554.333333	-0.61722174	0.042421063	0.479588013
19015	'Ppard'	406	373	1178	933	290	475	652.3333333	566	-0.074015635	0.926046304	0.992381554
19016	'Pparg'	95	88	191	12	40	58	124.6666667	36.66666667	-1.897452755	0.003900224	0.14342566
19017	'Ppargc1a'	59	62	98	537	120	160	73	272.3333333	2.149091047	0.008419278	0.222807422
19018	'Scand1'	202.19	244	466.29	159.97	312.35	324.22	304.16	265.5133333	-0.267380452	0.611841088	0.972790461
19023	'Ppef2'	20	33	143	31	31	22	65.33333333	28	-1.243921104	0.169713382	0.785344769
19024	'Ppfibp2'	640	685	264	378	634	873	529.6666667	628.3333333	0.290652963	0.492922961	0.972790461
19025	'Ctsa'	1328.8	1318.6	2520.1	1791.3	1780	2144.46	1722.51	1905.23	0.160747062	0.745732103	0.972790461
19027	'Sypl'	2185	2040	1973	1664	2284	2727	2066	2225	0.134549467	0.599587605	0.972790461
19035	'Ppib'	2750.8	2945.7	5720.6	1854	3253.7	4302.59	3805.666667	3136.74	-0.352612754	0.484643605	0.972790461
19038	'Ppic'	1440	1508	1678	513	1941	2033	1542	1495.666667	-0.129208515	0.787157161	0.972790461
19039	'Lgals3bp'	237	254	144	280	267	1171	211.6666667	572.6666667	1.416726562	0.019084233	0.344064642
19041	'Ppl'	100	85	171	825	141	250	118.6666667	405.3333333	2.021976414	0.018403404	0.338053636
19042	'Ppm1a'	1612	1564	2265	2038	1814	2215	1813.666667	2022.333333	0.211717139	0.623327775	0.972790461
19043	'Ppm1b'	2232	2219	2662	2556	1961	2399	2371	2305.333333	0.051346983	0.904247755	0.987963813
19044	'Ppox'	488	525	391	404	794	490	468	562.6666667	0.308712336	0.366445744	0.936406787
19045	'Ppp1ca'	3194.7	3342.3	4208.7	2692.7	3746.7	5139.81	3581.883333	3859.716667	0.0965457	0.787129573	0.972790461
19046	'Ppp1cb'	5657	5695	10310	5163	5287	6540	7220.666667	5663.333333	-0.33822018	0.47712435	0.972790461
19047	'Ppp1cc'	3949.1	4476.1	6177.7	5504	5068.7	5632.77	4867.636667	5401.843333	0.20650584	0.637891579	0.972790461
19049	'Ppp1r1b'	5	3	18	24	7	8	8.666666667	13	0.725811899	0.524949323	0.972790461
19052	'Ppp2ca'	3240	3277	5641	3649	3979	5160	4052.666667	4262.666667	0.075621744	0.867994522	0.979191352
19053	'Ppp2cb'	1140.6	1185.6	2551.8	1734.9	1523.8	2210.92	1625.983333	1823.19	0.169258092	0.757605095	0.972790461
19054	'Ppp2r3d'	754	731	838	491	807	656	774.3333333	651.3333333	-0.230061376	0.480717119	0.972790461
19055	'Ppp3ca'	1741	1640	3746	3882	1982	2613	2375.666667	2825.666667	0.349317601	0.587342591	0.972790461
19056	'Ppp3cb'	1965	1974	1916	1647	1760	2070	1951.666667	1825.666667	-0.035271767	0.908050057	0.988823459

19057	'Ppp3cc'	939	970	657	190	655	574	855.3333333	473	-0.884037841	0.034502137	0.445535842
19058	'Ppp3r1'	2390	2258	5395	2330	2684	3819	3347.666667	2944.333333	-0.223399467	0.682271491	0.972790461
19059	'Ppp3r2'	1	4	0	0	2	3	1.666666667	1.666666667	-0.025017094	0.990950257	0.999493374
19060	'Ppp5c'	1027	1080	524	648	1027	1089	877	921.3333333	0.131475578	0.692895552	0.972790461
19062	'Inpp5k'	460	521	339	385	607	539	440	510.3333333	0.268068238	0.329914094	0.921648675
19063	'Ppt1'	2392	2508	475	928	5048	3350	1791.666667	3108.666667	0.80277414	0.247600006	0.868994456
19065	'Npy4r'	8	4	4	57	2	18	5.333333333	25.66666667	2.614371683	0.033711265	0.442872044
19069	'Nup88'	1790	1858	1652	1357	1636	2045	1766.666667	1679.333333	-0.029376994	0.91234503	0.989773583
19070	'Mob4'	1037	1142	1613	854	1225	1532	1264	1203.67	-0.088777618	0.822971647	0.972790461
19072	'Prep'	1157	1185	2183	1343	1409	1890	1508.333333	1547.333333	0.035901826	0.940753112	0.996019386
19073	'Srgn'	11	22	20	146	50	56	17.66666667	84	2.476249101	0.001558481	0.084694293
19074	'Prg2'	0	0	1	12	5	2	0.333333333	6.333333333	4.409693271	0.021123905	0.360833505
19075	'Prim1'	603	588	361	78	459	499	517.3333333	345.3333333	-0.652707366	0.263927832	0.880205046
19076	'Prim2'	566	568	378	211	400	474	504	361.6666667	-0.465529235	0.127492977	0.723917691
19079	'Prkab1'	623	619	757	540	637	734	666.3333333	637	-0.033949795	0.922103539	0.991293892
19082	'Prkag1'	816	896	1024	1084	899	1169	912	1050.666667	0.278042653	0.492964312	0.972790461
19084	'Prkar1a'	8035	8177	8065	10818	8213	10097	8092.333333	9709.333333	0.370564473	0.355619734	0.931809687
19085	'Prkar1b'	511	499	1674	699	581	1024	894.6666667	768	-0.260738441	0.699827137	0.972790461
19087	'Prkar2a'	1200	1332	1236	327	953	1122	1256	800.6666667	-0.707250068	0.086955058	0.640026928
19088	'Prkar2b'	1634	1741	1711	9465	1505	2822	1695.333333	4597.333333	1.74294349	0.031297376	0.429713045
19089	'Prkcsh'	1580	1540	1225	1972	2102	2462	1448.333333	2178.666667	0.666403221	0.021009131	0.360113137
19090	'Prkdc'	1738	1768	276	415	1395	1118	1260.666667	976	-0.32685434	0.628144377	0.972790461
19091	'Prkg1'	45	28	97	38	29	36	56.66666667	34.33333333	-0.700745298	0.327767592	0.921056277
19092	'Prkg2'	102	88	153	49	85	92	114.3333333	75.33333333	-0.635458539	0.179189425	0.798783498
19094	'Mapk11'	55	54	26	40	89	66	45	65	0.574832596	0.21787519	0.844165262
19099	'Mapk8ip1'	798	871	342	438	954	705	670.3333333	699	0.11780712	0.779758347	0.972790461
19106	'Eif2ak2'	1021.1	1111	987.09	593.97	1610	1655.46	1039.716667	1286.476667	0.268449271	0.465063191	0.972790461
19108	'Prkx'	537	515	1241	772	516	541	764.3333333	609.6666667	-0.254085482	0.686293774	0.972790461
19116	'Prlr'	192	208	71	351	817	532	157	566.6666667	1.914476356	5.70E-05	0.008884271
19119	'Prm2'	0	0	0	2	0	0	0	0.666666667	2.299096387	0.566358544	0.972790461
19122	'Prnp'	1600.7	1639.8	1550.3	1865.3	2665.9	1929.87	1596.916667	2153.68	0.491303838	0.132347891	0.72996754
19123	'Proc'	0	0	0	0	2	1	0	1	2.320886	0.562538074	0.972790461
19124	'Procr'	209	187	502	335	152	297	299.3333333	261.3333333	-0.131873034	0.847727747	0.975439569
19125	'Prodh'	117	112	101	13	149	153	110	105	-0.184938822	0.800915124	0.972790461
19126	'Prom1'	56	40	154	41	44	67	83.33333333	50.66666667	-0.776290535	0.275220359	0.886562091
19127	'Prop1'	1	0	0	0	0	0	0.333333333	0	-0.903279821	0.824807108	0.972790461
19128	'Pros1'	475	479	243	1509	874	1092	399	1158.333333	1.731623185	6.81E-04	0.052033928
19130	'Prox1'	67	80	95	141	125	89	80.66666667	118.3333333	0.660170246	0.211423695	0.840335617
19132	'Prph'	50	42	110	254	71	509	67.33333333	278	2.022828909	0.011771491	0.268666967
19133	'Prph2'	2	0	0	0	2	0	0.666666667	0.666666667	0.031622541	0.993710128	0.999493374

19134	'Prpf4b'	3664	3716	2091	3622	4333	2546	3157	3500.333333	0.285227761	0.509736473	0.972790461
19139	'Prps1'	1259.1	1217.8	1945.6	422	916	1195.01	1474.176667	844.3366667	-0.878700044	0.055106379	0.539208689
19141	'Lgmn'	816	794	1283	12525	1157	3671	964.3333333	5784.333333	2.870948251	0.001724824	0.089596578
19142	'Prss12'	9	11	26	26	20	18	15.33333333	21.33333333	0.532805063	0.504955255	0.972790461
19143	'St14'	21	21	10	56	13	24	17.33333333	31	1.124011774	0.188697443	0.810921289
19144	'Klk6'	0	0	0	1	0	0	0	0.333333333	1.020273531	0.802557913	0.972790461
19146	'Tmprss15'	1	3	0	0	0	1	1.333333333	0.333333333	-1.735451621	0.587572465	0.972790461
19152	'Prtn3'	20	13	21	9	13	8	18	10	-0.80896073	0.252620519	0.871252558
19153	'Prx'	250	228	138	260	231	279	205.3333333	256.6666667	0.440822355	0.26031924	0.878537553
19155	'Npepps'	2742	2607	6274	6770	2457	4160	3874.333333	4462.333333	0.327326454	0.643502028	0.972790461
19156	'Psap'	8331	8837	4665	17187	14000	18451	7277.666667	16546	1.316601046	8.56E-04	0.060494815
19157	'Cyth1'	860	950	574	688	955	904	794.6666667	849	0.167495467	0.555348334	0.972790461
191578	'Helq'	472	410	208	232	372	317	363.3333333	307	-0.166067432	0.657069764	0.972790461
19158	'Cyth2'	1069	1080	1524	577	1088	1102	1224.333333	922.3333333	-0.440787472	0.253859386	0.873052317
19159	'Cyth3'	1655	1730	1369	1836	1724	2001	1584.666667	1853.666667	0.321426618	0.328756032	0.921648675
19164	'Psen1'	1183	1131	916	1496	1800	2019	1076.666667	1771.666667	0.781523998	0.002714243	0.116485742
19165	'Psen2'	215	225	184	2705	342	970	208	1339	2.995066016	2.94E-04	0.029716625
19166	'Psma2'	1697	1798	1954	1761	1486	2128	1816.333333	1791.666667	0.047254299	0.901400068	0.98672375
19167	'Psma3'	2036	2301.6	2271	1592	1899.6	2550	2202.86	2013.87	-0.101380811	0.740522278	0.972790461
19170	'Psmb1'	1835	1820	2617	2077	1616	2172	2090.666667	1955	-0.031008131	0.945555726	0.996592403
19171	'Psmb10'	214	216	402	31	194	193	277.3333333	139.3333333	-1.145551552	0.087991238	0.642430655
19172	'Psmb4'	1984	2339	2740	2266	2211	3059	2354.333333	2512	0.132416666	0.73024692	0.972790461
19173	'Psmb5'	1053	1137	1278	1580	1223	1774	1156	1525.666667	0.475960428	0.246486777	0.868334735
19175	'Psmb6'	1485	1676	1982	1490	1404	1897	1714.333333	1597	-0.055212515	0.886286316	0.984348372
19177	'Psmb7'	1990	2288	2243	1622	1785	2456	2173.666667	1954.333333	-0.116463946	0.715207561	0.972790461
19179	'Psmc1'	2053	2284	2163	1559	2171	2863	2166.666667	2197.666667	0.033155852	0.907431057	0.988823459
19181	'Psmc2'	1733	1946	2055	1536	1657	2257	1911.333333	1816.666667	-0.037522657	0.910786711	0.989608384
19182	'Psmc3'	2385	2586	2286	1293	2450	3044	2419	2262.333333	-0.110530126	0.713318151	0.972790461
19183	'Psmc3ip'	312.72	307.83	128.65	25.91	179.96	264.52	249.7333333	156.7966667	-0.743621131	0.293668431	0.90029104
19184	'Psmc5'	1091	1201	898	1064	1236	1577	1063.333333	1292.333333	0.338991408	0.20896877	0.836616817
19185	'Psmd4'	2060	2140	2718	3952	2272	3345	2306	3189.666667	0.581204078	0.245939904	0.867497116
19186	'Psme1'	1196	1373	2162	560	1363	1725	1577	1216	-0.465187506	0.343627184	0.925308103
19188	'Psme2'	1145	1290.2	1873	625.41	1152.6	1325	1436.086667	1034.33	-0.519421064	0.214111714	0.842446242
19192	'Psme3'	2328	2259	1957	1082	1788	2292	2181.333333	1720.666667	-0.337283082	0.211464134	0.840335617
19193	'Pipox'	30	44	4	0	35	13	26	16	-0.73669548	0.578606291	0.972790461
19197	'Pspn'	1	4	2	3	3	1	2.333333333	2.333333333	0.136713456	0.925679492	0.992381554
19200	'Pstpip1'	21	23	7	5	17	53	17	25	0.504080345	0.573251515	0.972790461
19201	'Pstpip2'	23	38	23	9	48	28	28	28.33333333	-0.020758362	0.97513962	0.999493374
19202	'Rhox6'	28	25	30.11	0	6	16	27.70333333	7.333333333	-2.061082371	0.042316012	0.479588013
19204	'Ptafr'	11	10	10	11	11	23	10.33333333	15	0.558037495	0.425289189	0.960285372

19205	'Ptbp1'	5271	5420	9854	3850	5598	5084	6848.333333	4844	-0.512188046	0.270770762	0.883910323
19206	'Ptch1'	992	872	669	648	922	1127	844.3333333	899	0.129855475	0.617910797	0.972790461
19207	'Ptch2'	255	282	14	24	275	157	183.6666667	152	-0.274376568	0.784755404	0.972790461
19208	'Ptcra'	1	0	1	0	2	0	0.666666667	0.666666667	-0.105959209	0.974948688	0.999493374
19210	'Ptdss1'	931	931	869	1045	1291	1601	910.3333333	1312.333333	0.566498603	0.036128313	0.456753899
19211	'Pten'	2726	2635	3212	1612	2386	2612	2857.666667	2203.333333	-0.369623828	0.246264308	0.868334735
192113	'Atp12a'	10	15	3	2	5	6	9.333333333	4.333333333	-1.061597221	0.294821697	0.90029104
192119	'Dicer1'	3797	3681	948	1150	2743	3130	2808.666667	2341	-0.223946135	0.682346127	0.972790461
19212	'Pter'	255.66	315.17	243.39	52	285.31	319.83	271.4066667	219.0466667	-0.397719459	0.487570677	0.972790461
192120	'Bspry'	196	193	165	804	131	338	184.6666667	424.3333333	1.481598913	0.056886116	0.547413448
192136	'Sugct'	58	80	81	140	103	207	73	150	1.088261888	0.024042405	0.383174818
192140	'Tmc2'	138	126	6	1	91	171	90	87.66666667	-0.098781165	0.939352527	0.995594283
19215	'Ptgds'	20	16	64	1289	61	504	33.33333333	618	4.419302711	4.82E-05	0.008204297
192156	'Mvd'	272	325	429	365	301	329	342	331.6666667	0.031637134	0.94661827	0.996592403
192157	'Socs7'	1816	1781	1318	1859	1376	1548	1638.333333	1594.333333	0.102707668	0.803522819	0.972790461
192159	'Prpf8'	9136	8341	4938	6088	8068	8297	7471.666667	7484.333333	0.081641472	0.787174248	0.972790461
19216	'Ptger1'	71.78	77.17	37.18	42.46	64.76	16.93	62.04333333	41.38333333	-0.45795696	0.496756973	0.972790461
192160	'Casc3'	1697	1657	1870	1175	1471	1507	1741.333333	1384.333333	-0.291409781	0.355622478	0.931809687
192161	'Pcdha9'	6.39	2.04	0	0	16.02	9.5	2.81	8.506666667	1.600934068	0.372749738	0.938916841
192163	'Pcdha3'	2.12	4.07	0	0	0	0	2.063333333	0	-3.276441223	0.380561172	0.944042914
192164	'Pcdha12'	2.17	2.08	0	0	4.68	0	1.416666667	1.56	0.004485678	0.998745477	0.999900097
192166	'Sardh'	910	968	413	324	1006	959	763.6666667	763	-2.20E-04	0.999618208	0.999900097
192167	'Nlgn1'	43	29	4	2	48	29	25.33333333	26.33333333	0.02435908	0.982211187	0.999493374
192169	'Ufsp2'	577	627	841	297	516	659	681.6666667	490.6666667	-0.512558652	0.196747298	0.822696823
19217	'Ptger2'	18	14	63	164	57	60	31.66666667	93.66666667	1.702185041	0.063180545	0.566852792
192170	'Eif4a3'	1544.3	1582	1549	1245	1530	2052	1558.446667	1609	0.074240202	0.796517451	0.972790461
192173	'Mcrip1'	1071	1139	1264	974	930	1295	1158	1066.333333	-0.069684474	0.847197596	0.975231333
192174	'Rwdd4a'	507	533	922	787	547	580	654	638	0.052857137	0.923812434	0.991932584
192176	'Flna'	15641	14921	11792	6988	17097	13012	14118	12365.66667	-0.187774044	0.55489643	0.972790461
19218	'Ptger3'	49	48	128	98	69	90	75	85.66666667	0.226137997	0.736365662	0.972790461
192185	'Nadk'	1991	2008	2208	1887	2681	2996	2069	2521.333333	0.301170837	0.304879199	0.907188578
192187	'Stab1'	216	209	28	27	273	205	151	168.3333333	0.137412684	0.874008058	0.980171118
192188	'Stab2'	446	346	39	2	224	219	277	148.3333333	-0.959264763	0.418366582	0.958127699
19219	'Ptger4'	64	59	88	91	38	53	70.33333333	60.66666667	-0.058161124	0.92958167	0.993164002
192190	'Pkhd1l1'	203	255	38	5045	537	918	165.3333333	2166.666667	4.161448	4.33E-05	0.007630056
192191	'Med9'	559	605	587	180	597	731	583.6666667	502.6666667	-0.288590827	0.53042868	0.972790461
192192	'Shkbp1'	219	215	74	367	284	269	169.3333333	306.6666667	1.048241131	0.054281513	0.534951117
192193	'Edem1'	1434	1307	3558	1364	1092	2024	2099.666667	1493.333333	-0.512979744	0.400815796	0.955860942
192194	'Btnl10'	3	3	1	0	10	1	2.333333333	3.666666667	0.602650496	0.732663732	0.972790461
192195	'Ash1l'	3850	3837	2821	2409	3456	3038	3502.666667	2967.666667	-0.175269661	0.499749636	0.972790461

192196	'Luc7l2'	8575	8519.9	5826	4954.1	7928.3	4913	7640.3	5931.793333	-0.286499309	0.408429007	0.957157474
192197	'Bcas3'	1114	1121	406	385	793	986	880.3333333	721.3333333	-0.25393783	0.577053413	0.972790461
192198	'Lrrc4'	436	451	50	3	538	488	312.3333333	343	0.074931602	0.950907099	0.998103338
192199	'Rspo1'	1157	1234	83	94	1127	1452	824.6666667	891	0.078220961	0.936764982	0.994413066
19220	'Ptgfr'	32	32	40	20	28	10	34.66666667	19.33333333	-0.768897092	0.245458016	0.866601262
19221	'Ptgfrn'	2388.7	2207.4	932.02	2814.1	2657.4	3181	1842.703333	2884.17	0.780112324	0.07063779	0.589903167
192212	'Prom2'	9	10	7	0	4	9	8.666666667	4.333333333	-1.102325526	0.352045623	0.929748874
192216	'Tmem47'	883	827	999	886	1134	1376	903	1132	0.344577842	0.287826062	0.894952226
19222	'Ptgir'	5	7	9	7	9	47	7	21	1.475199096	0.123961667	0.716557984
19223	'Ptgis'	364	408	588	553	399	460	453.3333333	470.6666667	0.141503821	0.779369701	0.972790461
192231	'Hexim1'	1830	1778	842	1850	2030	1954	1483.333333	1944.666667	0.513660537	0.187203178	0.808483975
192232	'Hps4'	748	680	355	487	670	740	594.3333333	632.3333333	0.164215317	0.629785326	0.972790461
192236	'Hps1'	388.07	341.94	231	258	497.97	466	320.3366667	407.3233333	0.37740769	0.215937694	0.843391934
19224	'Ptgs1'	71	69	126	659	68	202	88.66666667	309.6666667	2.073078504	0.025231651	0.393200117
19225	'Ptgs2'	17	20	3907	39336	19	754	1314.666667	13369.66667	3.605154018	0.172238041	0.790009561
19226	'Pth'	0	0	0	0	1	0	0	0.333333333	1.020273531	0.802557913	0.972790461
19227	'Pthlh'	0	4	6	0	1	4	3.333333333	1.666666667	-1.212668248	0.553250212	0.972790461
19228	'Pth1r'	224	187	84	95	210	158	165	154.3333333	-0.0431094	0.923528546	0.991932584
192285	'Phf21a'	2534	2391	2752	1575	1957	1956	2559	1829.333333	-0.441799636	0.167217657	0.781391371
192287	'Slc25a36'	4685	4490	4553	4561	4470	2867	4576	3966	-0.084376626	0.850721097	0.975734242
192289	'Tmlhe'	375	378	457	150	470	450	403.3333333	356.6666667	-0.244851745	0.579014892	0.972790461
19229	'Ptk2b'	270	246	193	344	477	761	236.3333333	527.3333333	1.17496985	3.34E-04	0.032745436
192292	'Nrbp1'	3030	3002	2180	435	2643	2852	2737.333333	1976.666667	-0.553759267	0.338581003	0.924608313
19230	'Twf1'	1286	1346	951	2487	1294	1664	1194.333333	1815	0.790878568	0.120165557	0.711044992
19231	'Ptma'	23827	24447	50785	19655	19122	28577.98	33019.64	22451.65333	-0.567118033	0.280106365	0.889355605
19240	'Tmsb10'	1744	1820	4880	771	1968	2712	2814.666667	1817	-0.772647932	0.223893634	0.850759465
19241	'Tmsb4x'	5892	6696	11258	10393	7673	8315	7948.666667	8793.666667	0.222528133	0.67760167	0.972790461
19242	'Ptn'	938	909	69	111	1356	610	638.6666667	692.3333333	0.114160492	0.904601567	0.988180016
19243	'Ptp4a1'	2784	2836	12798	18375	2974	5005	6139.333333	8784.666667	0.708524726	0.475175863	0.972790461
19244	'Ptp4a2'	4559	4533	9433	10112	4891	7570	6175	7524.333333	0.381916551	0.542738272	0.972790461
19245	'Ptp4a3'	996	978	632	273	1305	1161	868.6666667	913	0.019866141	0.968813155	0.999493374
19246	'Ptpn1'	1091	1096	544	1338	1183	1040	910.3333333	1187	0.542838294	0.220017358	0.848644756
19247	'Ptpn11'	3236	3283	2442	4144	3120	3932	2987	3732	0.448532835	0.250330431	0.871106156
19248	'Ptpn12'	2519.7	2433	2245	4208	1857	1900	2399.246667	2655	0.364642227	0.539111197	0.972790461
19249	'Ptpn13'	3799	3889	1814	10806	4044	5212	3167.333333	6687.333333	1.344483776	0.033917355	0.443491574
19250	'Ptpn14'	1633	1698	1424	812	1801	1703	1585	1438.666667	-0.146104526	0.619087519	0.972790461
19252	'Dusp1'	163	126	941	3165	130	447	410	1247.333333	1.858721766	0.134089849	0.733405125
19253	'Ptpn18'	16	22	6	14	37	186	14.66666667	79	2.356915427	0.010689168	0.255370433
19255	'Ptpn2'	1587	1587	2781	585	1485	1316	1985	1128.666667	-0.888788952	0.063983636	0.5684378
19256	'Ptpn20'	76	60	5	0	40	28	47	22.66666667	-1.097436606	0.403017143	0.955996639

19258	'Ptpn4'	2715	2935	1277	1403	2819	2562	2309	2261.333333	0.018042955	0.961458931	0.999493374
19259	'Ptpn5'	37	32	42	193	33	43	37	89.66666667	1.582035956	0.078528254	0.615492265
19260	'Ptpn22'	3	7	3	10	6	9	4.333333333	8.333333333	1.077870795	0.270020237	0.883851074
19261	'Sirpa'	854	739	492	297	1070	1176	695	847.6666667	0.246207881	0.596911851	0.972790461
19262	'Ptpra'	2118	1919	1320	1633	2167	2058	1785.666667	1952.666667	0.204920055	0.467133419	0.972790461
19263	'Ptprb'	734	688	55	128	682	537	492.3333333	449	-0.112632899	0.894994752	0.985422597
19264	'Ptprc'	22	26	9	23	34	65	19	40.66666667	1.128191591	0.065646232	0.574593252
19265	'Ptprcap'	1	1	1	8	4	1	1	4.333333333	2.357318557	0.140004149	0.742885537
192650	'Cabp7'	12.14	10.09	4.07	4.05	7.05	1.01	8.766666667	4.036666667	-0.982184878	0.372206497	0.938594721
192651	'Zfp286'	253.15	270.36	144	109	320	200	222.5033333	209.6666667	-0.065950666	0.878208967	0.981341203
192652	'Wdr81'	949	875	849	610	884	951	891	815	-0.099310762	0.698361786	0.972790461
192653	'Ttc36'	6	7	6	0	7	8	6.333333333	5	-0.455431514	0.710022326	0.972790461
192654	'Pla2g15'	337	281	212	470	473	531	276.6666667	491.3333333	0.925578639	0.006730923	0.194707262
192656	'Ripk2'	444	453	1500	535	324	442	799	433.6666667	-0.846651739	0.236725723	0.862272407
192657	'Ell2'	806	757	705	822	499	739	756	686.6666667	-0.007943396	0.98592728	0.999493374
192658	'Rfpl4'	0	1	1	5	0	4	0.666666667	3	2.336033867	0.285870863	0.893820325
19266	'Ptprd'	2711	2524	596	852	3455	3194	1943.666667	2500.333333	0.371057378	0.555724183	0.972790461
192662	'Arhgdia'	4913	4799	14508	3601	4404	6453	8073.333333	4819.333333	-0.813717937	0.190319152	0.813136091
192663	'Abcg4'	92	66	17	39	70	73	58.33333333	60.66666667	0.135188	0.829018654	0.973678583
19267	'Ptpre'	175	208	149	391	425	476	177.3333333	430.6666667	1.358500743	1.89E-05	0.004028556
192678	'Rassf3'	523	456	270	273	531	684	416.3333333	496	0.267138666	0.463954046	0.972790461
19268	'Ptprf'	6608	5943	1740	5039	8232	8427	4763.666667	7232.666667	0.68934827	0.1627874	0.774309245
19270	'Ptprg'	2439	2307	974	1494	3533	3431	1906.666667	2819.333333	0.593966614	0.158614951	0.768890221
19271	'Ptprj'	637	572	245	3020	690	1782	484.6666667	1830.666667	2.193675599	0.002187282	0.104483922
19272	'Ptprk'	1359	1209	1221	1373	1366	1776	1263	1505	0.31651663	0.324833473	0.91982931
19273	'Ptpru'	60	37	53	84	27	36	50	49	0.18957562	0.798180584	0.972790461
192734	'Lrrc75b'	43	36	41	18	55	55	40	42.66666667	0.049104664	0.923763323	0.991932584
19274	'Ptprm'	527	486	141	45	606	491	384.6666667	380.6666667	-0.0693382	0.931117587	0.993601701
19275	'Ptprn'	37	33	405	1140	66	75	158.3333333	427	1.684079062	0.214875495	0.842760284
19276	'Ptprn2'	51	49	14	53	73	24	38	50	0.568575505	0.428280668	0.962191208
19277	'Ptpro'	116	177	119	30	759	814	137.3333333	534.3333333	1.826162277	0.037009506	0.459648634
192775	'Kcnh6'	317.84	323.73	79.58	211.82	441.52	272.68	240.3833333	308.6733333	0.45621347	0.418106769	0.957831774
192786	'Rapgef6'	1618	1583	650	1253	1299	1147	1283.666667	1233	0.087855128	0.843345752	0.975182082
19279	'Ptprr'	6	0	8	43	1	10	4.666666667	18	2.231759275	0.162043253	0.772315447
19280	'Ptprs'	4260	3946	1883	7966	5527	4884	3363	6125.666667	1.068130156	0.038902122	0.461389364
19281	'Ptprt'	59	62	23	0	30	50	48	26.66666667	-0.951517196	0.401598487	0.955860942
19283	'Ptprz1'	57	61	42	39	97	26	53.33333333	54	0.075019151	0.90293981	0.987381169
19285	'Cavin1'	2331	2377	3370	1804	2877	3574	2692.666667	2751.666667	0.002509675	0.994812581	0.999562152
19286	'Pts'	520	553	186	100	544	436	419.6666667	360	-0.242390134	0.690375459	0.972790461
19288	'Ptx3'	10	14	94	31	7	41	39.33333333	26.33333333	-0.620830854	0.576257583	0.972790461

19289	'Igdcc3'	230	250	16	6	205	97	165.3333333	102.6666667	-0.71333153	0.517826123	0.972790461
192897	'Itgb4'	42	32	51	22	40	34	41.66666667	32	-0.387468647	0.451313502	0.970649024
19290	'Pura'	1487	1492.9	1780.2	836.99	1432	1596.8	1586.713333	1288.58	-0.314858239	0.335887869	0.922539021
19291	'Purb'	3472	3853	3063	5198	3689	4041	3462.666667	4309.333333	0.458181819	0.286199959	0.894057448
19293	'Pvalb'	3	0	1	0	1	0	1.333333333	0.333333333	-1.824355968	0.564012095	0.972790461
19294	'Nectin2'	1503	1521	2081	3189	2022	2682	1701.666667	2631	0.731713794	0.139422234	0.741642687
192950	'Nacad'	41	47	137	33	21	35	75	29.66666667	-1.330368804	0.076433281	0.609792246
192970	'Dhrs11'	135	192	242	83	279	231	189.6666667	197.6666667	-0.019843281	0.969165567	0.999493374
192976	'Lrrc75a'	411.5	415	513.58	652	379.29	503.66	446.6933333	511.65	0.320685305	0.527791891	0.972790461
19298	'Pex19'	1704	1757	1245	839	1820	1789	1568.666667	1482.666667	-0.07581333	0.795185097	0.972790461
192986	'Cyb5d2'	201	194	80	56	154	144	158.3333333	118	-0.404777064	0.396367927	0.954259077
19299	'Abcd3'	1793	1931	1267	1829	1987	2030	1663.666667	1948.666667	0.32483111	0.295949963	0.90029104
19300	'Abcd4'	192.57	170.11	89	240	274	243	150.56	252.3333333	0.864332203	0.033030271	0.438221572
193003	'Pirt'	0	1	0	0	0	1	0.333333333	0.333333333	0.058500858	0.988561293	0.999493374
19301	'Pxmp2'	98	87	44	15	60	121	76.33333333	65.33333333	-0.284163818	0.673461673	0.972790461
19302	'Pex2'	709	798	753	263	864	863	753.3333333	663.3333333	-0.243222984	0.56819248	0.972790461
19303	'Pxn'	2140	2026	3652	2339	1920	2315	2606	2191.333333	-0.19886758	0.69128101	0.972790461
193034	'Trpv1'	10	11	4	0	2	0	8.333333333	0.666666667	-3.607548968	0.027275749	0.404239957
193043	'Zfp3'	239	215	72	79	204	196	175.3333333	159.6666667	-0.101490196	0.84488281	0.975182082
19305	'Pex5'	1531	1493	2495	3545	1369	1837	1839.666667	2250.333333	0.457318743	0.483645622	0.972790461
19309	'Pygm'	490	474	147	447	384	317	370.3333333	382.6666667	0.239755344	0.667065182	0.972790461
193116	'Slu7'	1358	1578	1908	972	1287	1414	1614.666667	1224.333333	-0.385080321	0.27897834	0.889355605
19317	'Qk'	6625	6458	6729	3960	4888	5012	6604	4620	-0.467545413	0.11206734	0.69684179
19324	'Rab1a'	2873	3023	9424	4452	2715	4096	5106.666667	3754.333333	-0.41661963	0.547936598	0.972790461
19325	'Rab10'	2902	3084	4278	2205	3122	3879	3421.333333	3068.666667	-0.170325338	0.655279004	0.972790461
19326	'Rab11b'	2580	2568	2794	2368	2853	2962	2647.333333	2727.666667	0.089322292	0.774597175	0.972790461
19328	'Rab12'	1111	1149	1853	2132	1340	1681	1371	1717.666667	0.420754743	0.434024555	0.965086068
193286	'BC049762'	61	66	28	0	45	39	51.66666667	28	-0.982850502	0.378296924	0.9419364
19329	'Rab17'	10	12	20	11	12	24	14	15.66666667	0.133512402	0.855638949	0.975734242
19330	'Rab18'	2836	2814	5234	4047	2900	3807	3628	3584.666667	0.042469088	0.936570717	0.994413066
19331	'Rab19'	17	16	5	1	32	19	12.66666667	17.33333333	0.393799298	0.71166688	0.972790461
19332	'Rab20'	42	51	175	377	67	185	89.33333333	209.6666667	1.374840255	0.139245675	0.741642687
193322	'Oog1'	3	0	1.57	0	1	1	1.523333333	0.666666667	-1.048309543	0.67921816	0.972790461
19334	'Rab22a'	1448	1426	1227	1062	942	1250	1367	1084.666667	-0.246926999	0.465219045	0.972790461
19335	'Rab23'	788	707	762	230	693	695	752.3333333	539.3333333	-0.534237461	0.189316177	0.812029435
19336	'Rab24'	679	725	933	397	717	728	779	614	-0.364705149	0.318254702	0.916136994
19337	'Rab33a'	56	44	385	17	35	23	161.6666667	25	-2.828436915	0.002778739	0.118186559
19338	'Rab33b'	455.19	454.21	897.2	467.01	446.19	604	602.2	505.7333333	-0.244400142	0.634373414	0.972790461
193385	'Ripor2'	19	31	27	5	21	40	25.66666667	22	-0.321030581	0.668810883	0.972790461
19339	'Rab3a'	105	101	230	371	108	154	145.3333333	211	0.71621703	0.360030654	0.933706718

19340	'Rab3d'	240	276	427	682	507	1267	314.3333333	818.6666667	1.375829192	0.0095005	0.240190842
19341	'Rab4a'	218	225	336	254	183	255	259.6666667	230.6666667	-0.102295401	0.836665016	0.974723675
19342	'Rab4b'	350	409	330	391	426	450	363	422.3333333	0.293772918	0.354059553	0.93103461
19344	'Rab5b'	2452.2	2531	2826.1	2514	2644	3206	2603.1	2788	0.147918892	0.662797725	0.972790461
19345	'Rab5c'	2036	2265	2858	2508	2288	3148	2386.333333	2648	0.195582734	0.628855427	0.972790461
193452	'Zfp184'	317	305	308	152	277	181	310	203.3333333	-0.577738857	0.103444744	0.674448379
19346	'Rab6a'	3314	3160	7919	1961	3069	3632	4797.666667	2887.333333	-0.799710627	0.149008287	0.757738857
19347	'Dennd5a'	5568	5527	4680	9957	5016	6546	5258.333333	7173	0.628098202	0.221884255	0.849517424
19348	'Kif20a'	974.51	1078.8	269.82	119.48	756.38	595.97	774.3666667	490.61	-0.677303047	0.316649811	0.915640551
19349	'Rab7'	2972	2975	6586	5865	3461	4790	4177.666667	4705.333333	0.242429208	0.691204178	0.972790461
19352	'Rabggtb'	583	614	3033	1257	522	869	1410	882.6666667	-0.629523536	0.462753346	0.972790461
19353	'Rac1'	3575	3712	7856	3337	4172	4926	5047.666667	4145	-0.310675829	0.546258572	0.972790461
19354	'Rac2'	95	118	30	89	76	87	81	84	0.215933473	0.709318922	0.972790461
19355	'Rad1'	598.59	582.2	208.8	114.01	373.94	370.59	463.1966667	286.18	-0.69020765	0.185542752	0.805706284
19356	'Rad17'	1016	1118	661	521	764	775	931.6666667	686.6666667	-0.379442241	0.170155341	0.785573906
19357	'Rad21'	3214.8	3319.9	5144	3914	3967	4492.68	3892.903333	4124.56	0.117563546	0.788574329	0.972790461
19358	'Rad23a'	564.27	572.65	474.95	851.09	670.57	732.71	537.29	751.4566667	0.610061093	0.127951738	0.72420912
19359	'Rad23b'	3540	3661	7846	4852	2828	4548	5015.666667	4076	-0.2409546	0.688557665	0.972790461
19360	'Rad50'	1889	2065	712	687	1498	1976	1555.333333	1387	-0.14157807	0.76386862	0.972790461
19361	'Rad51'	612	590	194	29	504	801	465.3333333	444.6666667	-0.152242343	0.863636528	0.977452409
19362	'Rad51ap1'	264	254	105	9	186	207	207.6666667	134	-0.720249822	0.403126135	0.955996639
19363	'Rad51b'	89	91.63	50	37	76	72.71	76.87666667	61.90333333	-0.281569722	0.486737434	0.972790461
19364	'Rad51d'	575	558	431	177	502	482	521.3333333	387	-0.453269127	0.221619723	0.849403976
19365	'Rad52'	204	173	590	58	179	137	322.3333333	124.6666667	-1.500900981	0.028186356	0.409664118
19366	'Rad54l'	362.99	384.96	49.67	30.76	293.91	378.76	265.8733333	234.4766667	-0.218469024	0.805473772	0.972790461
19367	'Rad9a'	225.3	275.7	324.35	267.35	276.31	259.19	275.1166667	267.6166667	0.023842281	0.955006958	0.999493374
193670	'Rnf185'	1164	1160	1461	1060	921	1261	1261.666667	1080.666667	-0.166041025	0.679120359	0.972790461
19373	'Rag1'	17	17	1	0	35	11	11.66666667	15.33333333	0.36260481	0.806627086	0.972790461
193736	'Zbtb12'	532	578	631	331	588	587	580.3333333	502	-0.214295209	0.501990462	0.972790461
19374	'Rag2'	2	7	2	0	2	1	3.666666667	1	-1.89121618	0.268437284	0.883600995
193740	'Hspa1a'	109.12	98.07	521.99	3661.8	112.68	1347.59	243.06	1707.346667	3.005283703	0.010247842	0.250748987
193742	'Abhd16a'	420	412	526	101	587	575	452.6666667	421	-0.222619168	0.70584374	0.972790461
19376	'Rab34'	868	799	1921	360	906	986	1196	750.6666667	-0.781563518	0.168774573	0.784384366
19377	'Rai1'	1895	1929	1610	818	1872	1810	1811.333333	1500	-0.282400316	0.350541427	0.92886351
19378	'Aldh1a2'	3207	3341	1116	1994	3208	3952	2554.666667	3051.333333	0.322153103	0.467908998	0.972790461
193796	'Kdm4b'	1993	1761	4321	1221	1745	1704	2691.666667	1556.666667	-0.824695971	0.124262001	0.716961086
193813	'Mcfd2'	1045	1034	2224	2529	1348	2360	1434.333333	2079	0.600426702	0.325693196	0.920269559
19383	'Raly'	4254	4439	5940	3713	4061	5051	4877.666667	4275	-0.164449383	0.667401336	0.972790461
193838	'Eme2'	148	165	49	46	153	161	120.6666667	120	-0.0012159	0.998310931	0.999900097
19384	'Ran'	4463	4589	5444	2532	4226	5642	4832	4133.333333	-0.251403858	0.46922363	0.972790461

19385	'Ranbp1'	1748	1831	1497	264	1445	1762	1692	1157	-0.642222863	0.258786069	0.877564912
19386	'Ranbp2'	5327	5878	4094	5210	5285	5980	5099.666667	5491.666667	0.200326285	0.517269638	0.972790461
19387	'Rangap1'	1170	1193	986	2395	1253	1840	1116.333333	1829.333333	0.875003706	0.074674116	0.603900623
19395	'Rasgrp2'	48	50	28	51	67	56	42	58	0.552978588	0.206219891	0.833294914
19400	'Rapsn'	4	5	11	1	9	8	6.666666667	6	-0.295239624	0.791306197	0.972790461
19401	'Rara'	745	735	1638	1103	837.97	939	1039.333333	959.99	-0.064009546	0.912550175	0.989773583
19411	'Rarg'	296	280	1090	268	220	359	555.3333333	282.3333333	-1.01650475	0.156596898	0.768046725
194126	'Mtmr11'	259	249	517	239	265	329	341.6666667	277.6666667	-0.308049797	0.550980831	0.972790461
19414	'Rasa3'	1400	1503	454	712	1636	1137	1119	1161.666667	0.121537998	0.805208238	0.972790461
19415	'Rasal1'	55	47	154	452	67	336	85.33333333	285	1.852805913	0.038341004	0.459930654
19416	'Rasd1'	211	203	42	47	236	206	152	163	0.100848321	0.885736001	0.984282742
19417	'Rasgrf1'	92	77	40	16	29	34	69.66666667	26.33333333	-1.361385676	0.005100927	0.16781708
19418	'Rasgrf2'	9	8	189	13	7	13	68.66666667	11	-2.718775472	0.030420615	0.4240152
19419	'Rasgrp1'	433	476	150	174	507	194	353	291.6666667	-0.206727991	0.72087472	0.972790461
194219	'Slfnl1'	2	2	0	1	2	0	1.333333333	1	-0.233806777	0.920899701	0.991047523
194231	'Cnksr1'	13	11	3	12	10	11	9	11	0.439997057	0.603557837	0.972790461
194237	'Rimkla'	49	41	11	33	42	34	33.66666667	36.33333333	0.2420829	0.705399548	0.972790461
194268	'9930104L06Rik'	460	506	385	179	427	382	450.3333333	329.3333333	-0.455319582	0.158043387	0.768890221
19428	'Rasl2-9'	9	17	3	14	16	17	9.666666667	15.66666667	0.816932447	0.311288654	0.911336601
194309	'Vps37d'	214	187	397	115	208	206	266	176.3333333	-0.645105457	0.204815455	0.833294914
19434	'Rax'	1	1	15	0	1	0	5.666666667	0.333333333	-4.100396884	0.064159058	0.568781147
194352	'Trpv5'	1	0	1	2	2	0	0.666666667	1.333333333	1.137696115	0.642450943	0.972790461
194388	'Tet3'	2725	2567	2025	2346	2334	2230	2439	2303.333333	0.024844643	0.942140293	0.996253096
194401	'Mical3'	1622	1497	575	1969	1602	1201	1231.333333	1590.666667	0.570973388	0.291051579	0.899022994
194433	'Olfr707'	0	0	1	0	0	0	0.333333333	0	-0.903279821	0.824807108	0.972790461
194588	'Obox7'	1	1	0	0	0	1	0.666666667	0.333333333	-0.744986434	0.839556299	0.974723675
194590	'Reps2'	396	425	214	769	459	388	345	538.6666667	0.865294505	0.12237708	0.71347226
194597	'Tmprss11a'	126	133	62	0	3	0	107	1	-6.695814838	9.76E-11	1.16E-07
194604	'Serpina16'	10	9	0	0	1	0	6.333333333	0.333333333	-3.980841515	0.087720446	0.642214018
194655	'Klf11'	807	835	980	491	658	723	874	624	-0.466962308	0.153935187	0.765411885
194735	'Btg1c'	0	0	0	2	0	1	0	1	2.714766126	0.495614689	0.972790461
194738	'Rhox11'	4	4	1	0	0	0	3	0	-3.905169016	0.121896977	0.712603747
194744	'Slc25a43'	50	64	36	14	58	56	50	42.66666667	-0.262226789	0.639516271	0.972790461
194854	'Gm9'	2	0	1	0	1	3	1	1.333333333	0.279461072	0.907754991	0.988823459
194856	'Rhox4e'	1.04	4.28	1.2	0	1.37	0	2.173333333	0.456666667	-2.375245065	0.325909795	0.920269559
194908	'Pld6'	69	59	20	6	65	139	49.33333333	70	0.422656356	0.64150803	0.972790461
194952	'Jmjd4'	812.63	822.76	368.5	931.63	596.83	550.85	667.9633333	693.1033333	0.2655027	0.622368731	0.972790461
194974	'Sun3'	27.09	28.04	11.85	5.98	26.09	8.52	22.32666667	13.53	-0.738528746	0.363545052	0.935022643
195018	'Zzef1'	1952	1689	1031	983	1381	1440	1557.333333	1268	-0.227151698	0.454027753	0.970649024
195040	'Tmem199'	604	554	405	286	376	483	521	381.6666667	-0.40194122	0.147019984	0.753611197

195046	'Nlrp1a'	2	2	1	1	3	0	1.666666667	1.333333333	-0.239879945	0.899313206	0.986106073
195208	'Dcdc2a'	167	162	92	8	97	168	140.3333333	91	-0.73118016	0.368466223	0.937530612
195209	'Zfp469'	153	117	156	33	94	85	142	70.66666667	-1.052228367	0.019559881	0.348059072
195236	'Pom121l2'	37	36	6	2	9	5	26.33333333	5.333333333	-2.250407291	0.016691116	0.323948168
195333	'Gsc2'	6	5	3	0	0	1	4.666666667	0.333333333	-3.615834271	0.054572999	0.536237452
195359	'Trim40'	0	0	1	0	0	2	0.333333333	0.666666667	0.78767502	0.845746136	0.975182082
195434	'Utp14b'	354.32	377.44	112.04	34.66	250.34	220.46	281.2666667	168.4866667	-0.777518904	0.262000805	0.879908021
195522	'Zfp691'	173	146	259	341	146	158	192.6666667	215	0.327966403	0.623269551	0.972790461
195531	'Zfp982'	333.79	382.5	248.72	569.02	858	599	321.67	675.34	1.150251784	6.12E-04	0.048420886
195555	'Pramel11'	2	1	1	0	0	0	1.333333333	0	-2.782259126	0.384965334	0.94674509
195646	'Hs3st2'	5	5	2	0	7	2	4	3	-0.463004982	0.756804567	0.972790461
195726	'Scml1'	147	138	21	3	88	179	102	90	-0.252962762	0.814964649	0.972790461
195727	'Nhs'	485	469	533	457	576	478	495.6666667	503.6666667	0.07819586	0.825531312	0.972790461
195733	'Grhl1'	43	43	34	30	61	67	40	52.66666667	0.401694244	0.339253281	0.924888762
19645	'Rb1'	843	806	826	590	607	627	825	608	-0.365173251	0.284149992	0.892640391
19646	'Rbbp4'	5691.8	5988.6	5806.7	3314.5	4872.4	6002.27	5829.06	4729.733333	-0.29001427	0.283237304	0.891393415
19647	'Rbbp6'	4470	4285	3012	2508	4674	3212	3922.333333	3464.666667	-0.128367621	0.678781284	0.972790461
19649	'Robo3'	6	5	4	3	5	9	5	5.666666667	0.181604396	0.852497727	0.975734242
19650	'Rbl1'	609	627	237	111	625	448	491	394.6666667	-0.338408613	0.567857654	0.972790461
19651	'Rbl2'	873.74	829.36	535.04	1280.3	1181.7	1312.44	746.0466667	1258.13	0.872553787	0.015316915	0.307843112
19652	'Rbm3'	2738.5	3022.3	5591.4	436.01	3172.4	2601	3784.08	2069.786667	-1.029798762	0.130113985	0.727620313
19653	'Rbm4'	410.99	554.88	319.56	276.29	463.07	434.95	428.4766667	391.4366667	-0.083513293	0.783123458	0.972790461
19654	'Rbm6'	2450	2523	1422	1040	2279	1651	2131.666667	1656.666667	-0.324410496	0.342740813	0.925308103
19655	'Rbmx'	1689	1765	1448	507	2106	924	1634	1179	-0.498242988	0.314099857	0.913921554
19656	'Rbmxl1'	1720	1666	1634	1260	1738	1726	1673.333333	1574.666667	-0.047911879	0.857358726	0.976287979
19659	'Rbp1'	926	1027	811	318	750	420	921.3333333	496	-0.872069608	0.019236859	0.346336332
19660	'Rbp2'	0	1	1	0	0	0	0.666666667	0	-1.851176388	0.646290011	0.972790461
19661	'Rbp3'	1	3	0	0	3	1	1.333333333	1.333333333	-0.002474296	0.999160712	0.999900097
19662	'Rbp4'	43	47	117	9	18	44	69	23.66666667	-1.685348972	0.02696858	0.403699531
19663	'Rbpms'	1075	1122	3534	1568	991	1499	1910.333333	1352.666667	-0.477377379	0.490098536	0.972790461
19664	'Rbpj'	2423	2264	6742	3831	2119	2398	3809.666667	2782.666667	-0.373390582	0.593967878	0.972790461
19668	'Rbpjl'	0	0	0	1	1	0	0	0.666666667	2.050872943	0.610671515	0.972790461
19671	'Rce1'	210	214	202	158	252	351	208.6666667	253.6666667	0.27828352	0.392821616	0.952648938
19672	'Rcn1'	2327	2064	2545	1422	2861	3418	2312	2567	0.114696746	0.745886275	0.972790461
19674	'Rcvrn'	1	1	0	2	0	0	0.666666667	0.666666667	0.506111843	0.877512995	0.981341203
19679	'Pitpnm2'	741	720	231	153	737	614	564	501.3333333	-0.181346811	0.762246871	0.972790461
19682	'Rdh5'	52	39	23	28	46	24	38	32.66666667	-0.124186234	0.824168209	0.972790461
19683	'Rdh16'	0	0	0	4.07	0	1	0	1.69	3.536062214	0.339382682	0.924888762
19684	'Rdx'	4002	4009	3534	7126	4945	8397	3848.333333	6822.666667	0.923734004	0.0208129	0.358419252
19687	'Rfc1'	2564	2548	1255	1226	2415	2877	2122.333333	2172.666667	0.059691263	0.870337723	0.979911512

19691	'Recql'	952.61	874.78	235.68	232.67	688.89	644.58	687.69	522.0466667	-0.36839849	0.514720228	0.972790461
19692	'Reg1'	1	0	0	0	0	0	0.333333333	0	-0.903279821	0.824807108	0.972790461
19693	'Reg2'	21	16	4	0	6	1	13.66666667	2.333333333	-2.5496812	0.054936487	0.538100206
19694	'Reg3a'	5	1	0	2	0	0	2	0.666666667	-1.087686711	0.705976716	0.972790461
19695	'Reg3g'	0	1	8	0	0	1	3	0.333333333	-3.183197252	0.249093289	0.869642097
19696	'Rel'	514	566	1278	569	532	387	786	496	-0.614985719	0.315736817	0.914723408
19697	'Rela'	1127	1139	3102	1106	1207	1368	1789.333333	1227	-0.567084404	0.343422046	0.925308103
19698	'Relb'	392.15	384	1721	285	282.39	353	832.3833333	306.7966667	-1.49402168	0.045709292	0.498244985
19699	'Reln'	651	631	64	614	567	716	448.6666667	632.3333333	0.670953593	0.366426118	0.936406787
19700	'Rem1'	40	39	65	42	55	37	48	44.66666667	-0.070443883	0.899396503	0.986106073
19701	'Ren1'	20	24	6	3792	35	21	16.66666667	1282.666667	6.813247097	7.74E-06	0.002072764
19703	'Renbp'	515	487	1386	743	599	865	796	735.6666667	-0.114892582	0.854466596	0.975734242
19704	'Upf1'	2159	2136	1074	1644	1957	1863	1789.666667	1821.333333	0.133382262	0.710026949	0.972790461
19707	'Reps1'	1127	1149	1642	918	1135	1274	1306	1109	-0.222884529	0.568048944	0.972790461
19708	'Dpf2'	2170	2245	2582	1355	2045	2130	2332.333333	1843.333333	-0.330330438	0.287867864	0.894952226
19711	'Resp18'	0	0	1	0	0	0	0.333333333	0	-0.903279821	0.824807108	0.972790461
19712	'Rest'	2471	2481	2575	1426	2269	2603	2509	2099.333333	-0.253435036	0.36512897	0.935765447
19713	'Ret'	113	115	88	4	180	61	105.3333333	81.66666667	-0.47746642	0.615812711	0.972790461
19714	'Rev3l'	4143	4572	1450	1099	4666	3630	3388.333333	3131.666667	-0.114645424	0.837505393	0.974723675
19716	'Bex1'	344.42	359.27	470.2	303.87	171.89	313.05	391.2966667	262.9366667	-0.487495677	0.339552137	0.924888762
19718	'Rfc2'	827	847	422	333	800	962	698.6666667	698.3333333	0.002376401	0.995317019	0.999562152
19719	'Rfng'	349	313	214	253	394	408	292	351.6666667	0.3157476	0.264904006	0.88128484
19720	'Trim27'	1562	1471	2371.2	1338	1309	1644	1801.383333	1430.333333	-0.302785711	0.490898705	0.972790461
19724	'Rfx1'	615	647	472	501	696	605	578	600.6666667	0.12269883	0.660301333	0.972790461
19725	'Rfx2'	251	285	101	171	229	224	212.3333333	208	0.072408894	0.868373493	0.979229375
19726	'Rfx3'	779	785	425	493	690	387	663	523.3333333	-0.223881123	0.600285415	0.972790461
19727	'Rfxank'	369.38	386.86	368.81	273.53	352.63	312.2	375.0166667	312.7866667	-0.204514787	0.517299149	0.972790461
19729	'Slc50a1'	199	212	192	101	271	279	201	217	0.075399661	0.844413634	0.975182082
19730	'Ralgds'	940	932	1375	973	1069	1157	1082.333333	1066.333333	0.010116458	0.980408741	0.999493374
19731	'Rgl1'	1363	1300	801	1069	1177	1453	1154.666667	1233	0.179998776	0.56437105	0.972790461
19732	'Rgl2'	638	651	343	402	1027	688	544	705.6666667	0.406026112	0.299572024	0.903468167
19733	'Rgn'	31	35	19	20	50	31	28.33333333	33.66666667	0.282674552	0.594979644	0.972790461
19734	'Rgs16'	190	202	1241	556	252	194	544.3333333	334	-0.615991774	0.517848524	0.972790461
19735	'Rgs2'	2437	2710	1131	517	2642	1612	2092.666667	1590.333333	-0.413140718	0.45513964	0.970950306
19736	'Rgs4'	27	9	214	549	15	19	83.33333333	194.3333333	1.500626575	0.317088644	0.916136994
19737	'Rgs5'	90	96	19	229	133	80	68.33333333	147.3333333	1.397392199	0.0694686	0.587499452
19739	'Rgs9'	200	189	297	596	93	154	228.6666667	281	0.579268272	0.503609104	0.972790461
19743	'Rhag'	16	10	2	4	31	1	9.333333333	12	0.405972597	0.758542946	0.972790461
19744	'Rheb'	936	945	1914	1128	1035	1279	1265	1147.333333	-0.121456221	0.817102249	0.972790461
19746	'Rhd'	7	10	1	0	51	4	6	18.33333333	1.578126982	0.327449471	0.920734892

19752	'Rnase1'	23	29	5	5	4	4	19	4.333333333	-1.967373759	0.036404392	0.457448971
19763	'Ring1'	298	331	233	155	613	579	287.3333333	449	0.594372546	0.202265243	0.8304652
19765	'Ralbp1'	1508	1474	1554	1798	1559	2244	1512	1867	0.371898757	0.305335722	0.907188578
19766	'Ripk1'	1098.3	1095.4	1314.4	622.75	1354.7	1328.73	1169.36	1102.07	-0.11874389	0.737853516	0.972790461
19769	'Rit1'	471	569	721	609	639	768	587	672	0.229352726	0.571446077	0.972790461
19771	'Rlbp1'	22	22	5	0	11	3	16.33333333	4.666666667	-1.832367101	0.137736729	0.739191241
19773	'Rln1'	5	2	5	0	0	0	4	0	-4.419155204	0.048923511	0.514354314
19775	'Xpr1'	1672	1643	961	2324	2071	2046	1425.333333	2147	0.730952595	0.064554444	0.570648987
19777	'Uri1'	1838	1949	1592	1322	1687	2147	1793	1718.666667	-0.021193576	0.933126957	0.994031155
19819	'Rnaseh1'	219	242	177	83	258	225	212.6666667	188.6666667	-0.197574453	0.624248319	0.972790461
19820	'Rlim'	3844	3893	5035	5030	3813	5300	4257.333333	4714.333333	0.221555358	0.611114169	0.972790461
19821	'Rnf2'	2475	2491	3613	3718	2606	3285	2859.666667	3203	0.251034971	0.604843445	0.972790461
19822	'Rnf4'	3346	3330	3915	1675	2709	3229	3530.333333	2537.666667	-0.487276794	0.128282677	0.724799196
19823	'Rnf7'	1198	1311	1846	817	1146	1620	1451.666667	1194.333333	-0.306972526	0.449073662	0.97049895
19824	'Trim10'	19	24	2	7	34	1	15	14	-0.005990017	0.99628823	0.999616639
19826	'Rnps1'	3043	3028	4883	1938	2345	3035	3651.333333	2439.333333	-0.587668361	0.166990976	0.781229856
19876	'Robo1'	442	402	205	433	620	457	349.6666667	503.3333333	0.626963243	0.107928499	0.684280429
19877	'Rock1'	2935	2974	2000	1949	2539	3037	2636.333333	2508.333333	-0.014408872	0.955019365	0.999493374
19878	'Rock2'	3148	3118	4298	6725	3143	4214	3521.333333	4694	0.566281825	0.323934325	0.919383393
19879	'Slc22a8'	3	3	0	0	6	5	2	3.666666667	0.838463148	0.657545059	0.972790461
19881	'Rom1'	67	75	27	69	66	54	56.33333333	63	0.319138408	0.559605418	0.972790461
19882	'Mst1r'	273	272	105	265	378	337	216.6666667	326.6666667	0.691880782	0.103907986	0.675846267
19883	'Rora'	493	405	2848	3605	257	821	1248.666667	1561	0.533465388	0.641954373	0.972790461
19885	'Rorc'	697	649	655	298	797	930	667	675	-0.031941496	0.934861035	0.994413066
19886	'Ros1'	7	7	1	1	25	26	5	17.33333333	1.73652682	0.150908512	0.759755159
19888	'Rp1'	0	1	0	0	2	2	0.333333333	1.333333333	1.768901033	0.57287116	0.972790461
19889	'Rp2'	628	690	805	598	702	770	707.6666667	690	-4.29E-04	0.999026418	0.999900097
19891	'Rpa2'	667	653	435	500	676	985	585	720.3333333	0.336958732	0.262794072	0.88013169
19893	'Rpgr'	1189	1207	259	127	788	790	885	568.3333333	-0.660188301	0.355648751	0.931809687
19894	'Rph3a'	0	2	1	1	0	3	1	1.333333333	0.425826827	0.85935107	0.976863639
19895	'Rpia'	607	608	891	302	443	635	702	460	-0.641541216	0.119766233	0.710007992
19896	'Rpl10a'	10021	10224	16178	7539	7699	8981	12141	8073	-0.559928822	0.202616322	0.830701895
19899	'Rpl18'	7234	7489	12450	12654	5899	7972	9057.666667	8841.666667	0.099171888	0.870174946	0.979794316
19921	'Rpl19'	10309	10822	18444	13595	8540.7	11122.23	13191.88333	11086.00333	-0.164078604	0.763913251	0.972790461
19933	'Rpl21'	9382.1	9632.2	17056	12024	8239.4	10112.38	12023.41333	10125.30333	-0.171480958	0.750227276	0.972790461
19934	'Rpl22'	5155	5262	9544	5045	4398	5898	6653.666667	5113.666667	-0.351738822	0.478809362	0.972790461
19935	'Mrpl23'	348	343	405	155	284	410	365.3333333	283	-0.407157882	0.292132293	0.89958198
19941	'Rpl26'	9883	10019	17121	15143	7457	9520	12341	10706.66667	-0.075617732	0.900110581	0.986505007
19942	'Rpl27'	4573	4790	8266	6528	4010	5253	5876.333333	5263.666667	-0.0699904	0.899385317	0.986106073
19943	'Rpl28'	8377	8154	19747	9998	6007	7412	12092.66667	7805.666667	-0.558357874	0.37760197	0.941536807

19944	'Rpl29'	6016.8	6032.9	11960	8768	5584	7173	8003.253333	7175	-0.082361855	0.885941778	0.984348372
19946	'Rpl30'	4380.4	4784.5	8761.9	7721	5080	6419	5975.58	6406.666667	0.175487062	0.754268642	0.972790461
19951	'Rpl32'	8526	8727	16002	11011	5988	8057	11085	8352	-0.306689416	0.602339484	0.972790461
19981	'Rpl37a'	5795	6317	9156	8459	3960	5292	7089.333333	5903.666667	-0.120180689	0.838227359	0.974723675
19982	'Rpl36a'	4115	4303	6706	4235	2661	3701	5041.333333	3532.333333	-0.425553326	0.414795994	0.95722888
19988	'Rpl6'	14842	15450	24831	19963	13132	16159.44	18374.33333	16418.07	-0.072186638	0.891008031	0.985046501
19989	'Rpl7'	15245	16149	27449	21133	14967	16965	19614.33333	17688.33333	-0.068148198	0.898230186	0.985862578
20005	'Rpl9'	10575	10767	22373	18804	10042	12365.79	14571.79333	13737.22	0.018212002	0.976810564	0.999493374
20014	'Rpn2'	2357.1	2166.1	1797	1929.9	2690.4	4008.86	2106.706667	2876.373333	0.467155615	0.105078054	0.677660775
20016	'Polr1c'	1351	1405	2271	1219	1042	1397	1675.666667	1219.333333	-0.418777219	0.375925004	0.940406075
20017	'Polr1b'	776	710	480	307	917	1044	655.3333333	756	0.176949919	0.670790992	0.972790461
20018	'Polr1d'	1190	1122	2101	1375	1123	1589	1471	1362.333333	-0.07749527	0.878191606	0.981341203
20019	'Polr1a'	2315	2339	1461	1696	2467	2111	2038.333333	2091.333333	0.111369583	0.700402188	0.972790461
20020	'Polr2a'	9821	9299	4100	2161	5448	6024	7740	4544.333333	-0.756162383	0.079827507	0.617305615
20021	'Polr2c'	520.35	511.76	916.54	640.3	577.25	713.48	649.55	643.6766667	0.0206357	0.966528977	0.999493374
20022	'Polr2j'	698	763	721	233	469	798	727.3333333	500	-0.589616967	0.154665066	0.766453805
20024	'Sub1'	3124	3331	3791	1401	2722	3746	3415.333333	2623	-0.424602181	0.255386173	0.875162567
20028	'Pdc'	0	0	0	1	0	0	0	0.333333333	1.020273531	0.802557913	0.972790461
20042	'Rps12'	7825	8402	17991	13096	7269	8774	11405.87	9712.96	-0.137322876	0.828246675	0.973238667
20044	'Rps14'	13823	14601	24446	21842	10857	16738	17623.33333	16479	0.011682198	0.98401653	0.999493374
20054	'Rps15'	7140	7340	12193	11706	6180	8884	8891	8923.333333	0.113053983	0.842861625	0.975182082
20055	'Rps16'	8738	8779	20870	21924	7002	9824	12795.66667	12916.66667	0.17517798	0.816204814	0.972790461
20068	'Rps17'	6507	6849	12890	10004	5597	7021	8748.666667	7540.666667	-0.113488805	0.849273292	0.975734242
20084	'Rps18'	10320	11025	20917	13417	7927	10826	14087.33333	10723.33333	-0.311363016	0.592637759	0.972790461
20085	'Rps19'	9994.9	10324	21141	16884	7091	9534	13819.86333	11169.66667	-0.173103904	0.796241196	0.972790461
20088	'Rps24'	11113	11763	20688	17268	9194	11909	14521.44667	12790.33	-0.07192305	0.903247283	0.987531419
20090	'Rps29'	2217	2528	4592	2711	1611	2214	3112.333333	2178.666667	-0.432785638	0.455320555	0.971118957
20091	'Rps3a1'	17699	17689	37413	29553	15997	20996	24267	22182	-0.035533894	0.95428082	0.99943739
20102	'Rps4x'	17415	17493	30088	19176	14848	16374	21665.33333	16799.33333	-0.296583755	0.563179701	0.972790461
20103	'Rps5'	11593	11668	19256	15397	10621	13718	14172.33333	13245.33333	-0.020646381	0.967981871	0.999493374
20104	'Rps6'	17952	17867	32295	26080	14431	17140	22704.66667	19217	-0.127031494	0.829550174	0.973678583
20111	'Rps6ka1'	586	574	626	1435	734	1039	595.3333333	1069.333333	0.995236811	0.05483275	0.537360953
20112	'Rps6ka2'	1092	1046	476	626	1257	1176	871.3333333	1019.666667	0.273239126	0.472213276	0.972790461
20115	'Rps7'	14276	14416	24510	19605	12437	16274	17734	16105.33333	-0.052496054	0.922371828	0.991300958
20116	'Rps8'	11559	12062	24522	18676	10916	13897	16047.88333	14496.22	-0.060448358	0.920085287	0.990973345
20128	'Trim30a'	63	82.59	155.62	44.47	56	270.07	100.4033333	123.5133333	0.152814772	0.843708193	0.975182082
20129	'Rptn'	0	0	0	0	1	1	0	0.666666667	1.775692139	0.660844521	0.972790461
20130	'Rras'	381	407	2184	436	358	669	990.6666667	487.6666667	-1.102942462	0.178019137	0.797422443
20132	'Rrh'	1	5	2	0	0	2	2.666666667	0.666666667	-2.049758732	0.349737529	0.92886351
20133	'Rrm1'	1726	1875	1132	1166	2179	2025	1577.666667	1790	0.217985062	0.437901599	0.966382496

20135	'Rrm2'	421	425	116	77	424	356	320.6666667	285.6666667	-0.181081396	0.781431676	0.972790461
20147	'Rs1'	6	2	4	2	4	2	4	2.666666667	-0.553545547	0.654559211	0.972790461
20148	'Dhrs3'	979	1030	2479	103	819	594	1496	505.3333333	-1.74410459	0.017993688	0.335354139
20163	'Rsu1'	772	789	963	286	983	1031	841.3333333	766.6666667	-0.217564119	0.642901069	0.972790461
20166	'Rtkn'	403	414	820	190	475	416	545.6666667	360.3333333	-0.680860009	0.193975677	0.817889904
20167	'Rtn2'	65	83	58	292	85	120	68.66666667	165.6666667	1.531755512	0.030634049	0.425756906
20168	'Rtn3'	3121	2885	3114	4625	3300	4302	3040	4075.666667	0.531251149	0.206535903	0.834040055
20170	'Hps6'	212	207	268	189	251	289	229	243	0.097274825	0.789917291	0.972790461
20174	'Ruvbl2'	918.4	937.38	2809.2	772.06	935.7	1204.85	1554.976667	970.87	-0.737123462	0.237631274	0.863337624
20181	'Rxra'	1654	1610	650	729	1943	2132	1304.666667	1601.333333	0.306631174	0.504260713	0.972790461
20182	'Rxrb'	1099	1048	1257	798	996	1109	1134.666667	967.6666667	-0.200593659	0.539311511	0.972790461
20183	'Rxrg'	2	0	5	0	3	1	2.333333333	1.333333333	-1.017023576	0.640432237	0.972790461
20184	'Uimc1'	1352	1274	681	712	1066	715	1102.333333	831	-0.308645624	0.419436025	0.958308933
20185	'Ncor1'	4564	4520	4146	8027	5321	5461	4410	6269.666667	0.66042226	0.154815299	0.766453805
20186	'Nr1h4'	10	14	124	6	15	18	49.33333333	13	-2.125370644	0.052151072	0.530211865
20187	'Ryk'	1126	1206	2550	1924	1591	2083	1627.333333	1866	0.221423991	0.69113305	0.972790461
20190	'Ryr1'	505	525	119	565	940	397	383	634	0.87599693	0.160652762	0.771114735
20191	'Ryr2'	4335	4309	421	331	5439	3338	3021.666667	3036	-0.017566604	0.985191348	0.999493374
20192	'Ryr3'	174	158	36	7	36	45	122.6666667	29.33333333	-2.074352112	0.005369922	0.17331813
20193	'S100a1'	220	215	182	217	248	281	205.6666667	248.6666667	0.337746261	0.26399496	0.880205046
20194	'S100a10'	1011	1095	3008	1538	2299	2269	1704.666667	2035.333333	0.197221825	0.742926898	0.972790461
20195	'S100a11'	1241.6	1299.6	2855.9	1709.1	1678.9	1893.7	1799.043333	1760.58	-0.021626734	0.968572625	0.999493374
20196	'S100a13'	41	51	100	94	57	57	64	69.33333333	0.212405215	0.755888768	0.972790461
20197	'S100a3'	1	0	0	6	1	6	0.333333333	4.333333333	3.745977419	0.066519248	0.576509273
20198	'S100a4'	4	3	1	69	10	27	2.666666667	35.33333333	4.051671114	2.80E-04	0.0284244
20200	'S100a6'	76	68	162	604	155	361	102	373.3333333	2.01891508	0.008598418	0.225480165
20201	'S100a8'	0	55	5	6	113	5	20	41.33333333	1.050434154	0.521130118	0.972790461
20202	'S100a9'	0	79	5	4	175	2	28	60.33333333	1.102406928	0.543333628	0.972790461
20203	'S100b'	8	7	3	22	3	11	6	12	1.28249246	0.239709925	0.86449675
20204	'Prrx2'	124	127	46	113	141	126	99	126.6666667	0.473168877	0.315069515	0.914723408
20208	'Saa1'	0	0	1	0	0	0	0.333333333	0	-0.903279821	0.824807108	0.972790461
20209	'Saa2'	0	0	0	1	0	0	0	0.333333333	1.020273531	0.802557913	0.972790461
20210	'Saa3'	77	75	3759	276	34	186	1303.666667	165.3333333	-2.962354779	0.033608217	0.442492678
20215	'Sag'	4	9	7	44	13	5	6.666666667	20.66666667	1.940888199	0.078207124	0.614954936
20216	'Acsm3'	336.8	406.72	7.61	41.74	994.35	1492.57	250.3766667	842.8866667	1.712114333	0.159223466	0.770378392
20218	'Khdrbs1'	2947	3031	3027	1484	3510	4047	3001.666667	3013.666667	-0.034308117	0.922835573	0.991576236
20219	'Apcs'	0	0	0	0	0	1	0	0.333333333	1.020273531	0.802557913	0.972790461
20220	'Sap18'	386.17	387.36	447.19	516.51	422.73	448.74	406.9066667	462.66	0.281028464	0.511961553	0.972790461
20222	'Sf3a2'	698	717	820	384	564	665	745	537.6666667	-0.466789679	0.143176071	0.749054337
20224	'Sar1a'	2873	2996	3803	4102	3318	3988	3224	3802.666667	0.317167991	0.460818667	0.972790461

20226	'Sars'	2696	2647	14569	3368	2431	4741	6637.333333	3513.333333	-0.982589257	0.228946124	0.855540033
20227	'Sart1'	1670	1800	1844	1664	1731	2033	1771.333333	1809.333333	0.089157451	0.78735191	0.972790461
20229	'Sat1'	511	503	2123	5913	564	1072	1045.666667	2516.333333	1.514061455	0.157618566	0.768337387
20230	'Satb1'	900	871	504	543	936	1158	758.3333333	879	0.242749982	0.447216771	0.970198456
20231	'Nkx1-2'	1	4	0	8	0	1	1.666666667	3	1.339494656	0.545306149	0.972790461
20238	'Atxn1'	262	251	645	283	285	295	386	287.6666667	-0.42023564	0.477661015	0.972790461
20239	'Atxn2'	2128.6	2005.8	1725.6	2174.2	1975	2110.71	1953.343333	2086.633333	0.200696071	0.569424123	0.972790461
20249	'Scd1'	397.67	421.2	216.42	376	404	287	345.0966667	355.6666667	0.186898737	0.666866605	0.972790461
20250	'Scd2'	14795	13903	15021	5692	17013	12245	14572.96333	11650	-0.360138862	0.355976063	0.931977458
20254	'Scg2'	13	20	9	0	0	3	14	1	-3.833974091	0.005727626	0.178221963
20255	'Scg3'	1	6	3	0	10	5	3.333333333	5	0.477419957	0.75706501	0.972790461
20256	'Clec11a'	54	57	13	18	63	60	41.33333333	47	0.204378716	0.763225191	0.972790461
20257	'Stmn2'	5	1	2	3	0	4	2.666666667	2.333333333	-0.065879709	0.968791229	0.999493374
20259	'Scin'	7	12	18	17	15	16	12.33333333	16	0.421685926	0.5780799	0.972790461
20262	'Stmn3'	2	0	0	0	2	1	0.666666667	1	0.555285763	0.861918139	0.976863639
20264	'Scn10a'	11	14	1	5	14	8	8.666666667	9	0.138240311	0.894795536	0.985422597
20265	'Scn1a'	8	10	2	101	6	11	6.666666667	39.33333333	3.030399069	0.015948752	0.314541149
20266	'Scn1b'	23	36	38	113	22	47	32.33333333	60.66666667	1.148005073	0.170596419	0.785702308
20269	'Scn3a'	21	26	8.02	24	49	43	18.34	38.66666667	1.131474254	0.053379596	0.533767484
20271	'Scn5a'	19	29	10	65	22	66	19.33333333	51	1.58110263	0.031188995	0.428752843
20272	'Scn7a'	30	33	0	1	31	8	21	13.33333333	-0.652303174	0.665030806	0.972790461
20273	'Scn8a'	377	349	149	6	258	107	291.6666667	123.6666667	-1.314683963	0.162917552	0.774492858
20274	'Scn9a'	0	1	1	3	1	1	0.666666667	1.666666667	1.503511681	0.470533897	0.972790461
20276	'Scnn1a'	61	61	29	22	72	27	50.33333333	40.33333333	-0.279895098	0.644891257	0.972790461
20277	'Scnn1b'	1	4	2	2	2	3	2.333333333	2.333333333	0.0557474	0.968472534	0.999493374
20278	'Scnn1g'	1	0	0	0	0	0	0.333333333	0	-0.903279821	0.824807108	0.972790461
20280	'Scp2'	1612	1872	2035	772	1803	2227	1839.666667	1600.666667	-0.255050808	0.516915605	0.972790461
20284	'Scrg1'	0	0	0	4	1	2	0	2.333333333	3.886484978	0.140733438	0.744590744
20286	'Zc3h7b'	3128	3133	2786	544	3456	3934	3015.666667	2644.666667	-0.29305301	0.62309479	0.972790461
20287	'Sct'	0	0	3	1	3	1	1	1.666666667	0.58019241	0.804130381	0.972790461
20288	'Msr1'	35	30	5	14	56	97	23.33333333	55.66666667	1.238590041	0.139921695	0.742885537
20289	'Scx'	45	41	63	25	45	50	49.66666667	40	-0.339581235	0.495340029	0.972790461
20290	'Ccl1'	1	0	9	61	0	10	3.333333333	23.66666667	3.058874594	0.11682406	0.704874333
20292	'Ccl11'	4	0	46	89	2	20	16.66666667	37	1.33062683	0.436394974	0.965982602
20293	'Ccl12'	1	1	1	0	4	0	1	1.333333333	0.327675531	0.894927201	0.985422597
20295	'Ccl17'	1	3	1	7	0	0	1.666666667	2.333333333	0.967863812	0.669208705	0.972790461
20296	'Ccl2'	88	95	5305	2489	71	551	1829.333333	1037	-0.654486108	0.740103486	0.972790461
20297	'Ccl20'	14	13	244	1	6	2	90.33333333	3	-5.131627002	1.08E-04	0.014617609
20299	'Ccl22'	0	0	2	0	2	3	0.666666667	1.666666667	1.011842144	0.725630941	0.972790461
20300	'Ccl25'	80	74	46	72	81	56	66.66666667	69.66666667	0.184961308	0.691778502	0.972790461

20301	'Ccl27a'	70.71	76.68	60.48	87.39	85.22	74.47	69.29	82.36	0.362007019	0.401476513	0.955860942
20302	'Ccl3'	67	77	108	87	26	20	84	44.33333333	-0.674583786	0.416669322	0.95722888
20303	'Ccl4'	23	22	66	7	10	9	37	8.666666667	-2.140261319	0.00746661	0.207233844
20304	'Ccl5'	4	0	602	7	2	5	202	4.666666667	-5.468999812	0.026194508	0.397137149
20305	'Ccl6'	2	6	1	23	3	16	3	14	2.484741806	0.042055431	0.477918618
20306	'Ccl7'	10	10	514	1130	14	173	178	439	1.50128507	0.465061731	0.972790461
20307	'Ccl8'	0	0	2	1	0	4	0.666666667	1.666666667	1.122043009	0.702327819	0.972790461
20308	'Ccl9'	12	20	10	37	18	21	14	25.33333333	1.053725265	0.155810697	0.766832248
20309	'Cxcl15'	0	0	0	2	0	0	0	0.666666667	2.299096387	0.566358544	0.972790461
20310	'Cxcl2'	47	50	670	1428	20	45	255.6666667	497.6666667	1.24256868	0.417571948	0.957720764
20311	'Cxcl5'	38	32	1846	1597	34	393	638.6666667	674.6666667	0.237587066	0.878396433	0.981341203
20312	'Cx3cl1'	245	235	153	1613	438	631	211	894	2.368608204	7.23E-04	0.053564853
20315	'Cxcl12'	364	311	279	521	476	590	318	529	0.824424332	0.018319835	0.336844005
20316	'Sdf2'	777	779	1304	942	987	1044	953.3333333	991	0.08567686	0.852082952	0.975734242
20317	'Serpinf1'	569	592	396	237	743	863	519	614.3333333	0.205716493	0.631272967	0.972790461
20318	'Sdf4'	2036	1999	1378	2007	2532	3410	1804.333333	2649.666667	0.604937919	0.025661688	0.396135865
20319	'Sfrp2'	160	150	96	94	358	147	135.3333333	199.6666667	0.565487485	0.281178991	0.889731741
20320	'Nptn'	1815	1781	1779	3146	2335	2896	1791.666667	2792.333333	0.750817838	0.065934871	0.574593252
20321	'Frrs1'	136	142	115	161	161	203	131	175	0.491405304	0.151199433	0.759950471
20322	'Sord'	355	340	501	920	347	639	398.6666667	635.3333333	0.820279103	0.182574085	0.803681064
20324	'Cavin2'	184	188	43	185	297	359	138.3333333	280.3333333	1.099915751	0.047276156	0.50504201
20333	'Sec22b'	1193	1188	2631	1038	1089	1492	1670.666667	1206.333333	-0.486962983	0.363144992	0.935009831
20334	'Sec23a'	2938	2881	5138	2630	2444	3619	3652.333333	2897.666667	-0.32232625	0.500906013	0.972790461
20335	'Sec61g'	663	624	2382	433	500	829	1223	587.3333333	-1.147533523	0.098593674	0.668019578
20336	'Exoc4'	1373	1399	1316	825	1485	1453	1362.666667	1254.333333	-0.111585601	0.672650151	0.972790461
20338	'Sel1l'	3068	2648	2822	3009	3137	4640	2846	3595.333333	0.378869771	0.240753797	0.86449675
20339	'Sele'	7	12	121	77	9	21	46.66666667	35.66666667	-0.240124687	0.852096903	0.975734242
20340	'Glg1'	5918	5628	2517	7702	6558	7270	4687.666667	7176.666667	0.767715627	0.081882603	0.621457906
20341	'Selenbp1'	819.77	889.01	6432.3	201	872.74	922.44	2713.696667	665.3933333	-2.264632272	0.014608393	0.300778621
20342	'Selenbp2'	84.23	87.99	528.69	20	70.26	81.56	233.6366667	57.27333333	-2.241011463	0.012244254	0.273764981
20343	'Sell'	0	5	0	0	4	0	1.666666667	1.333333333	-0.311200649	0.931507055	0.993601701
20344	'Selp'	2	1	2	1	1	2	1.666666667	1.333333333	-0.31618725	0.849144076	0.975734242
20345	'Selplg'	3	8	1	22	9	28	4	19.66666667	2.457804546	0.014437066	0.299352014
20346	'Sema3a'	1084	991	194	213	861	553	756.3333333	542.3333333	-0.4471354	0.502762946	0.972790461
20347	'Sema3b'	89	110	217	111	111	192	138.6666667	138	-0.036833868	0.948822032	0.997622192
20348	'Sema3c'	466	570	1509	1028	909	983	848.3333333	973.3333333	0.207075924	0.747062328	0.972790461
20349	'Sema3e'	75	56	430	474	88	248	187	270	0.627961336	0.538495324	0.972790461
20350	'Sema3f'	916	834	230	876	1141	922	660	979.6666667	0.707546107	0.181567189	0.802050906
20351	'Sema4a'	153	181	114	38	223	224	149.3333333	161.6666667	0.043477696	0.94086923	0.996035736
20352	'Sema4b'	1133	1187	599	2871	2166	1774	973	2270.333333	1.411707132	0.004151407	0.149677594

20353	'Sema4c'	969	946	443	1372	1358	1041	786	1257	0.834733674	0.064815642	0.572086808
20354	'Sema4d'	183	213	110	213	257	357	168.6666667	275.6666667	0.774327142	0.02710874	0.403710299
20355	'Sema4f'	155	159	29	12	87	79	114.3333333	59.33333333	-0.96228644	0.224232972	0.850759465
20356	'Sema5a'	1303	1322	533	393	1134	810	1052.666667	779	-0.405460534	0.375207809	0.940406075
20357	'Sema5b'	1506	1546	146	132	2315	1195	1066	1214	0.166461495	0.862251426	0.977060681
20358	'Sema6a'	476	491	205	142	672	614	390.6666667	476	0.25685909	0.644411782	0.972790461
20359	'Sema6b'	72	63	30	80	62	55	55	65.66666667	0.430746689	0.438774044	0.96707305
20360	'Sema6c'	910	960	855	870	1966	1246	908.3333333	1360.666667	0.595015764	0.083620888	0.627883225
20361	'Sema7a'	664	729	71	365	1569	988	488	974	1.039544669	0.183733976	0.804729813
20362	'Septin8'	1824	1702	1449	1686	2089	2347	1658.333333	2040.666667	0.355355168	0.16181534	0.771541907
20363	'Selenop'	1455.5	1301.4	420	784.94	1853.8	2094.71	1058.946667	1577.816667	0.607762368	0.229211623	0.855596006
20364	'Selenow'	1004	1075	1270	1077	838	854	1116.333333	923	-0.169559448	0.707600927	0.972790461
20365	'Serf1'	437	484	703	135	381	509	541.3333333	341.6666667	-0.76179241	0.133169236	0.732373013
20370	'Sez6'	63	39	19	1	44	6	40.33333333	17	-1.29518183	0.242112244	0.86449675
20371	'Foxp3'	7.35	8.23	4.2	0	4.07	1.03	6.593333333	1.7	-1.953974826	0.16298895	0.774492858
20375	'Spi1'	14	39	8	24	34	41	20.33333333	33	0.782613548	0.26217438	0.88013169
20377	'Sfrp1'	1440	1307	59	1954	1635	1200	935.3333333	1596.333333	1.019598538	0.264730643	0.88128484
20378	'Frzb'	34	36	161	517	29	107	77	217.6666667	1.743709316	0.130162123	0.727620313
20379	'Sfrp4'	11	17	16	13	36	13	14.66666667	20.66666667	0.501712787	0.485417453	0.972790461
20382	'Srsf2'	5124	5201	3288	2542.9	4671	3694.98	4537.653333	3636.28	-0.271228636	0.349162015	0.928823768
20383	'Srsf3'	4073	4344	4494	641	4971	3964	4303.666667	3192	-0.543704627	0.378413727	0.9419364
20384	'Srsf5'	7063	7402	4192	2705	6791	5261	6219	4919	-0.319547204	0.3658962	0.936406787
20387	'Sftpa1'	1	1	0	1	2	1	0.666666667	1.333333333	1.104064927	0.607441764	0.972790461
20389	'Sftpc'	1	0	0	6	1	0	0.333333333	2.333333333	3.091793008	0.269436279	0.883851074
20390	'Sftpd'	0	0	0	13	0	2	0	5	5.166946634	0.072485508	0.595707787
20391	'Sgca'	0	3	1	2	2	0	1.333333333	1.333333333	0.203688385	0.929265476	0.993096978
20392	'Sgce'	686	661	344	172	967	1030	563.6666667	723	0.302229836	0.605066621	0.972790461
20393	'Sgk1'	194	205	839	14192	224	1227	412.6666667	5214.333333	3.975128935	0.002325667	0.108371515
20394	'Scg5'	17	28	20	124	14	21	21.66666667	53	1.653929194	0.104314679	0.676639601
20397	'Sgpl1'	3040	3177	1637	4488	3599	3630	2618	3905.666667	0.738054379	0.0900175	0.648437606
20399	'Sh2b1'	1966	2027	1554	1177	1777	1536	1849	1496.666667	-0.2509621	0.325121368	0.920096374
20401	'Sh3bp1'	641	649	1032	596	601	521	774	572.6666667	-0.378666384	0.425808776	0.960773373
20402	'Zfp106'	5290	5014	6652	6155	4533	6017	5652	5568.333333	0.062618306	0.887943945	0.984534762
20403	'Itsn2'	1820.9	1820	1353.9	594.9	2215.8	1952	1664.926667	1587.56	-0.111492191	0.79709109	0.972790461
20404	'Sh3gl2'	119	119	55	8	76	82	97.66666667	55.33333333	-0.887929755	0.222913881	0.85026726
20405	'Sh3gl1'	984	1134	1493	1467	1313	1557	1203.666667	1445.666667	0.322174544	0.456203999	0.971368622
20408	'Sh3gl3'	229	205	374	748	132	259	269.3333333	379.6666667	0.736835873	0.372973828	0.939078058
20409	'Ostf1'	403	451	1139	420	426	557	664.3333333	467.6666667	-0.529253603	0.377324266	0.941504733
20410	'Sorbs3'	1777	1840	2691	589	1846	1865	2102.666667	1433.333333	-0.642109087	0.180304339	0.801341923
20411	'Sorbs1'	536	527	296	339	404	237	453	326.6666667	-0.335738796	0.451411067	0.970649024

20415	'Shbg'	189	208	5	2	285	81	134	122.6666667	-0.15041715	0.911130229	0.989682221
20416	'Shc1'	2551	2400	4691	1972	2316	3167	3214	2485	-0.391503057	0.415671449	0.95722888
20418	'Shc3'	204	165	23	263	85	265	130.6666667	204.3333333	0.878338955	0.287270315	0.894554788
20419	'Shcbp1'	139	194	70	15	185	152	134.3333333	117.3333333	-0.26179397	0.732241677	0.972790461
20420	'Shd'	21	18	6	5	25	5	15	11.66666667	-0.322090682	0.732421082	0.972790461
20422	'Sem1'	1003	1105	1388	1156	905	1142	1165.333333	1067.666667	-0.048296483	0.912755469	0.989773583
20423	'Shh'	11	10	6	0	4	2	9	2	-2.202490035	0.068838361	0.585310788
20425	'Shmt1'	458	468	218	145	442	490	381.3333333	359	-0.096643145	0.834846187	0.974723675
20429	'Shox2'	32	44	6	5	15	7	27.33333333	9	-1.51856927	0.085911225	0.636212038
20430	'Cyfip1'	2199.9	2231.7	1746.4	1932	2637.4	2867.19	2059.326667	2478.86	0.316570848	0.170802795	0.786030911
20431	'Pmel'	102.02	118.04	27.04	8	46	41	82.36666667	31.66666667	-1.384608787	0.05764756	0.549224181
20437	'Siah1a'	559.52	610.21	802.11	357.04	652.52	623.53	657.28	544.3633333	-0.292954173	0.437732388	0.96622803
20438	'Siah1b'	832.08	792.64	409.44	52	482	700	678.0533333	411.3333333	-0.810755551	0.26685999	0.883600995
20439	'Siah2'	197	172	1178	269	228	332	515.6666667	276.3333333	-0.967764937	0.258256008	0.877405867
20440	'St6gal1'	953	980	2045	765	1507	1077	1326	1116.333333	-0.29505102	0.573774546	0.972790461
20441	'St3gal3'	301	297	293	439	317	453	297	403	0.54068814	0.192092159	0.813837363
20442	'St3gal1'	827	786	802	1219	1197	969	805	1128.333333	0.590872608	0.137978664	0.739360306
20443	'St3gal4'	559.67	617	376.98	1130	803	870	517.8833333	934.3366667	1.013836721	0.022591381	0.372676826
20444	'St3gal2'	894	971	841	1843	1232	1507	902	1527.32	0.89517379	0.041071224	0.475545746
20446	'St6galnac2'	44	61	30	11	94	121	45	75.33333333	0.658262519	0.377646561	0.941536807
20447	'St6galnac3'	784.95	771.52	181.99	612.36	662.56	541.56	579.4866667	605.4933333	0.233006631	0.685646144	0.972790461
20448	'St6galnac4'	289	314	238	465	414	393	280.3333333	424	0.718676424	0.066879823	0.578108476
20449	'St8sia1'	656.27	697.52	28.79	1674.2	1064.5	933.29	460.86	1223.973333	1.689258683	0.070711305	0.59015072
20450	'St8sia2'	183	180	63	116	242	122	142	160	0.26339754	0.617086749	0.972790461
20451	'St8sia3'	2	2	1	0	0	1	1.666666667	0.333333333	-2.126777302	0.381454144	0.94429001
20452	'St8sia4'	1185.9	1208.3	271.45	456.36	9780.5	7213.79	888.5266667	5816.87	2.65534778	0.002743106	0.117459312
20454	'St3gal5'	993	1058	224	577	1493	1182	758.3333333	1084	0.575022648	0.3222838	0.918216634
20459	'Ptk6'	6	1	2	0	1	0	3	0.333333333	-2.984382737	0.178359848	0.797877044
20460	'Stil'	282	253	187	242	269	161	240.6666667	224	0.025829582	0.953768608	0.99917615
20462	'Tra2b'	2955	3231	4167	1134	3137	3155	3451	2475.333333	-0.548629329	0.202774396	0.83083072
20463	'Cox7a2l'	2187	2079	2947	5190	2402	3403	2404.333333	3665	0.755051452	0.187231266	0.808483975
20464	'Sim1'	1	4	0	45	0	1	1.666666667	15.33333333	3.766609627	0.072971289	0.59727171
20465	'Sim2'	0	3	1	5	0	1	1.333333333	2	0.959913028	0.673989495	0.972790461
20466	'Sin3a'	2928	2807	2785	2008	2746	3185	2840	2646.333333	-0.074259917	0.775990979	0.972790461
20467	'Sin3b'	2724	2696	1928	1230	2319	2758	2449.333333	2102.333333	-0.212333431	0.453861502	0.970649024
20469	'Sipa1'	990	1015	662	808	1312	1058	889	1059.333333	0.31325258	0.272985522	0.884738928
20471	'Six1'	23	25	16	3	24	34	21.33333333	20.33333333	-0.151834306	0.849583159	0.975734242
20472	'Six2'	5	7	2	1	1	0	4.666666667	0.666666667	-2.643936122	0.118135141	0.706729169
20473	'Six3'	0	2	0	1	0	0	0.666666667	0.333333333	-0.733814424	0.856146772	0.975734242
20474	'Six4'	115	120	116	228	149	150	117	175.6666667	0.733979029	0.146275785	0.753611197

20475	'Six5'	968.3	986.58	578.71	354	1241.2	750.16	844.53	781.79	-0.113110515	0.797714942	0.972790461
20476	'Six6'	6	7	1	0	1	1	4.666666667	0.666666667	-2.767399177	0.118094256	0.706706957
20479	'Vps4b'	2080	2110	2249	2007	2126	2388	2146.333333	2173.666667	0.077226347	0.815156398	0.972790461
20480	'Clpb'	741.02	682.08	745.62	1646	759	1120	722.9066667	1175	0.866275889	0.110017885	0.689784826
20481	'Ski'	3033	2953	2253	1725	2665	2918	2746.333333	2436	-0.138106018	0.543431432	0.972790461
20482	'Skil'	1405	1395	3673	2646	1271	1664	2157.666667	1860.333333	-0.118019277	0.864959316	0.978035808
20491	'Sla'	22	23	11	45	12	9	18.66666667	22	0.569804648	0.545244335	0.972790461
20492	'Slbp'	1931	1969	1769	840	1580	1740	1889.666667	1386.666667	-0.449307477	0.099636835	0.669835754
20493	'Slc10a1'	0	2	0	4	0	0	0.666666667	1.333333333	1.563564742	0.675021662	0.972790461
20495	'Slc12a1'	2	1	2	2	1	0	1.666666667	1	-0.538526994	0.782083186	0.972790461
20496	'Slc12a2'	1550	1559	1278	2622	1710	1429	1462.333333	1920.333333	0.573575651	0.256150121	0.876684814
20497	'Slc12a3'	4	2	1	0	1	0	2.333333333	0.333333333	-2.596385923	0.256256891	0.876684814
20498	'Slc12a4'	638	557	759	1155	787	927	651.3333333	956.3333333	0.663186063	0.167090206	0.781318221
20499	'Slc12a7'	1704	1651	499	4329	3481	2812	1284.666667	3540.666667	1.670080936	0.003366052	0.132437773
20500	'Slc13a2'	2	2	13	0	3	4	5.666666667	2.333333333	-1.52216779	0.335753369	0.922539021
20501	'Slc16a1'	1210	1183	868	669	1270	1827	1087	1255.333333	0.199058205	0.554644221	0.972790461
20502	'Slc16a2'	648	653	633	1134	959	804	644.6666667	965.6666667	0.708968312	0.102059614	0.671776916
20503	'Slc16a7'	61	87	32	328	67	109	60	168	1.821046767	0.026141315	0.397137149
20504	'Slc17a1'	1	2	0	12	0	1	1	4.333333333	2.620837263	0.234835353	0.861576558
20505	'Slc34a1'	0	0	0	7	0	0	0	2.333333333	4.140057783	0.292845669	0.900043079
20508	'Slc18a3'	0	1	0	1	0	0	0.333333333	0.333333333	0.058500858	0.988561293	0.999493374
20509	'Slc19a1'	256	278	221	158	326	331	251.6666667	271.6666667	0.111650237	0.712881332	0.972790461
20510	'Slc1a1'	13	24	7	29	6	12	14.66666667	15.66666667	0.393687873	0.682542105	0.972790461
20511	'Slc1a2'	57	36	125	3	27	27	72.66666667	19	-2.118893173	0.01320506	0.287077572
20512	'Slc1a3'	140	122	85	2012	140	298	115.6666667	816.6666667	3.232391963	0.001329702	0.077785538
20513	'Slc1a6'	83	88	58	59	115	210	76.33333333	128	0.723457195	0.119744342	0.710007992
20514	'Slc1a5'	281	283	206	240	311	261	256.6666667	270.6666667	0.156414263	0.622725496	0.972790461
20515	'Slc20a1'	2265	2011	1501	219	1420	1487	1925.666667	1042	-0.970193176	0.10171501	0.671759705
20516	'Slc20a2'	660	641	543	356	950	1115	614.6666667	807	0.354706593	0.350721928	0.92886351
20517	'Slc22a1'	4	1	0	1	2	4	1.666666667	2.333333333	0.527405157	0.768557672	0.972790461
20518	'Slc22a2'	0	1	0	0	0	0	0.333333333	0	-0.903279821	0.824807108	0.972790461
20519	'Slc22a3'	5	5	2	0	2	2	4	1.333333333	-1.612097579	0.291429527	0.899311502
20520	'Slc22a5'	470.33	478.3	403.56	633.24	506.79	555.23	450.73	565.0866667	0.447230083	0.260635896	0.878537553
20521	'Slc22a12'	0	0	0	0	2	0	0	0.666666667	1.801491674	0.656112935	0.972790461
20522	'Slc23a1'	11	12	4	8	8	7	9	7.666666667	-0.102568413	0.904125588	0.987963813
20523	'Slc25a14'	391	374	119	76	232	203	294.6666667	170.3333333	-0.765925516	0.153617401	0.765411885
20524	'Slc25a17'	789	834	713	428	837	938	778.6666667	734.3333333	-0.090168091	0.753792597	0.972790461
20525	'Slc2a1'	2002	1451	10399	6602	1162	4398	4617.333333	4054	-0.113088875	0.90873931	0.988896137
20526	'Slc2a2'	7	7	3	0	21	17	5.666666667	12.66666667	1.06599337	0.410688556	0.95722888
20527	'Slc2a3'	591	398	2967	887	321	508	1318.666667	572	-1.142184917	0.216481518	0.843391934

20528	'Slc2a4'	78	62	39	238	87	119	59.66666667	148	1.558992724	0.01851388	0.33942708
20529	'Slc31a1'	1313	1420	1282	2394	1791	1998	1338.333333	2061	0.746633455	0.072799733	0.596837647
20530	'Slc31a2'	355	380	261	750	479	686	332	638.3333333	1.086406104	0.013752859	0.292512191
20531	'Slc34a2'	1	2	1	53	4	28	1.333333333	28.33333333	4.68643401	3.54E-04	0.03396508
20532	'Slc3a1'	20.15	16.28	5.52	39.25	11.82	9.77	13.98333333	20.28	0.867680228	0.381814593	0.94429001
20533	'Slc4a1'	56	64	16	6	627	62	45.33333333	231.6666667	2.302600348	0.054467284	0.535883893
20534	'Slc4a1ap'	1622	1471	889	592	1065	1333	1327.333333	996.6666667	-0.390231989	0.23246558	0.859679981
20535	'Slc4a2'	1166	1207	980	1109	1862	1785	1117.666667	1585.333333	0.532595557	0.024018644	0.383174818
20536	'Slc4a3'	1537	1668	554	774	2914	995	1253	1561	0.361041645	0.546532974	0.972790461
20537	'Slc5a1'	0	1	1	1	0	1	0.666666667	0.666666667	0.098639155	0.971302795	0.999493374
20538	'Slc6a2'	9	4	5	5	1	2	6	2.666666667	-0.961244536	0.43766346	0.96622803
20539	'Slc7a5'	791	687	1051	864	1681	2315	843	1620	0.882719163	0.038294216	0.459930654
20540	'Slc7a7'	115.08	113	168	54.26	257	196	132.0266667	169.0866667	0.253491749	0.657260512	0.972790461
20541	'Slc8a1'	286	294	79	83	469	368	219.6666667	306.6666667	0.46828593	0.476565231	0.972790461
20544	'Slc9a1'	790	774	356	1100	1025	1083	640	1069.333333	0.881101485	0.036300907	0.456985794
20555	'Slfn1'	0	3	0	0	1	0	1	0.333333333	-1.313730691	0.742696629	0.972790461
20556	'Slfn2'	5	11	64	27	14	36	26.66666667	25.66666667	-0.118178776	0.912080897	0.989773583
20557	'Slfn3'	3	6.01	1	0	4.76	6	3.336666667	3.586666667	-0.056226559	0.971027322	0.999493374
20558	'Slfn4'	3	1.99	0	1	9.24	3	1.663333333	4.413333333	1.711668037	0.313703003	0.913268497
20562	'Slit1'	21	17	6	67	14	11	14.66666667	30.66666667	1.448465601	0.157510964	0.768337387
20563	'Slit2'	2353	2053	1185	1390	2199	1872	1863.666667	1820.333333	0.038456734	0.906287237	0.988823459
20564	'Slit3'	159	163	189	910	126	171	170.3333333	402.3333333	1.572166601	0.081053916	0.618950211
20568	'Slpi'	1	0	14	14	4	10	5	9.333333333	0.902889188	0.568373852	0.972790461
20583	'Snai2'	772	763	956	346	609	453	830.3333333	469.3333333	-0.814209201	0.026144337	0.397137149
20585	'Hltf'	767.6	794.05	546	595	796.37	745.01	702.55	712.1266667	0.090977793	0.740263654	0.972790461
20586	'Smarca4'	4898	4650	2834	1727	3810	3387	4127.333333	2974.666667	-0.449924164	0.160239268	0.771114735
20587	'Smarcb1'	1330	1354	1391	349	1446	1418	1358.333333	1071	-0.423898796	0.381030906	0.944233523
20588	'Smarcc1'	4634	4582	3198	2044	3879	4950	4138	3624.333333	-0.187790523	0.535854548	0.972790461
20589	'Ighmbp2'	501	505	329	105	459	430	445	331.3333333	-0.473648437	0.33650596	0.923048811
20591	'Kdm5c'	5363	4992	5295	4028	5125	4973	5216.666667	4708.666667	-0.099313077	0.737377498	0.972790461
20595	'Smn1'	321	399	514	249	316	434	411.3333333	333	-0.311215014	0.446438856	0.970198456
20597	'Smpd1'	374	386	388	543	601	776	382.6666667	640	0.785937646	0.012529751	0.277443073
20598	'Smpd2'	358	356	194	234	447	420	302.6666667	367	0.318869177	0.337850297	0.924020038
20599	'Smr3a'	1	0	0	0	0	0	0.333333333	0	-0.903279821	0.824807108	0.972790461
20600	'Smr2'	1	2	0	0	0	0	1	0	-2.28103714	0.568641838	0.972790461
20602	'Ncor2'	4468	4247	2065	3836	4426	4170	3593.333333	4144	0.322386839	0.393841626	0.953178011
20603	'Sms'	1230	1280	1070	302	944	1112	1193.333333	786	-0.656288555	0.120524264	0.711929925
20605	'Sstr1'	4	2	1	0	8	10	2.333333333	6	1.273179466	0.40655363	0.957157474
20606	'Sstr2'	0	2	1	8	14	24	1	15.33333333	3.921262599	0.001541877	0.084487393
20607	'Sstr3'	134	170	3	6	230	245	102.3333333	160.3333333	0.609427018	0.627126213	0.972790461

20608	'Sstr4'	10	10	12	11	11	3	10.66666667	8.333333333	-0.229412309	0.801753769	0.972790461
20609	'Sstr5'	2	0	3	1	1	3	1.666666667	1.666666667	-0.079952202	0.965675792	0.999493374
20610	'Sumo3'	2554	2714	1340	2979	2484	4017	2202.666667	3160	0.631636111	0.114518843	0.700525178
20612	'Siglec1'	5	7	1	1	3	8	4.333333333	4	-0.119803118	0.926598965	0.992535631
20613	'Snai1'	98	87	95	277	106	126	93.33333333	169.6666667	1.076948138	0.097556947	0.663834175
20614	'Snap25'	1	1	4	50	3	2	2	18.33333333	3.538313877	0.023564748	0.380103384
20615	'Snapin'	632.29	656.86	534.97	189.13	952.92	985.05	608.04	709.0333333	0.142610086	0.788382662	0.972790461
20616	'Snap91'	922	747	100	9	87	108	589.6666667	68	-3.146600571	4.05E-04	0.037893507
20617	'Snca'	46	45	125	24	155	76	72	85	0.084420137	0.91331806	0.989773583
20618	'Sncg'	0	3	1	19	1	5	1.333333333	8.333333333	2.999384103	0.076158081	0.60811316
20619	'Snap23'	982	979	1554	1007	1164	1174	1171.666667	1115	-0.047522023	0.912500405	0.989773583
20620	'Plk2'	1636	1774	2925	1285	2295	1139	2111.666667	1573	-0.419168728	0.413085408	0.95722888
20621	'Snn'	1153	1191	593	359	1428	1069	979	952	-0.055368127	0.908096744	0.988823459
20623	'Snrk'	1539	1538	1116	1361	1586	1503	1397.666667	1483.333333	0.173834044	0.559243038	0.972790461
20624	'Eftud2'	2788	2841	1404	1039	2395	2685	2344.333333	2039.666667	-0.18841539	0.623078879	0.972790461
20630	'Snrpc'	730	755	885	675	668	1015	790	786	0.024954179	0.946282555	0.996592403
20637	'Snrnp70'	8458	8662	5128	3599	6538	3617	7416	4584.666667	-0.617092109	0.106218319	0.679124905
20638	'Snrpb'	1657	1845	2149	1451	1798	2482	1883.666667	1910.333333	0.028047052	0.935745493	0.994413066
20639	'Snrpb2'	1190	1289	1588	570	1121	1325	1355.666667	1005.333333	-0.471354618	0.207581221	0.835068595
20641	'Snrpd1'	805	956	1075	382	797	913	945.3333333	697.3333333	-0.478773942	0.199969786	0.82759347
20643	'Snrpe'	1148	1166	1445	379	754	960	1253	697.6666667	-0.889517704	0.019392903	0.347725165
20646	'Snrpn'	191.91	202.57	395.55	251.71	213.69	431.18	263.3433333	298.86	0.166380284	0.763634268	0.972790461
20648	'Snta1'	193	207	150	213	201	292	183.3333333	235.3333333	0.436463652	0.206661646	0.834086989
20649	'Sntb1'	529	600	2257	429	523	635	1128.666667	529	-1.166608877	0.099496256	0.669364052
20650	'Sntb2'	1150	1191	721	3625	981	1054	1020.666667	1886.666667	1.20018768	0.102833517	0.672865573
20652	'Soat1'	7156	8271	4475	2966	9117	7011	6634	6364.666667	-0.061269723	0.878414768	0.981341203
20655	'Sod1'	3854	3780	4795	5770	3185	6940.99	4142.98	5298.646667	0.433066557	0.381615133	0.94429001
20656	'Sod2'	1004	1075	2899	1712	1073	1665	1659.333333	1483.333333	-0.131262862	0.8412083	0.975182082
20657	'Sod3'	31	28	8	825	57	222	22.33333333	368	4.451803182	1.22E-05	0.003055455
20658	'Son'	9497	9559	4660	6070	12023	7524	7905.333333	8539	0.18125452	0.641615348	0.972790461
20660	'Sorl1'	1274	1277	159	2157	1787	1696	903.3333333	1880	1.272732292	0.076814691	0.609792246
20661	'Sort1'	704	713	238	2387	799	1222	551.6666667	1469.333333	1.696691962	0.01331301	0.288185595
20662	'Sos1'	1835	1898	1632	3317	1916	2252	1788.333333	2495	0.645942072	0.184785941	0.804884883
20663	'Sos2'	1364	1222	1653	756	1318	1373	1413	1149	-0.315004996	0.36808339	0.937190492
20664	'Sox1'	1	1	0	0	0	0	0.666666667	0	-1.703500596	0.673820112	0.972790461
20665	'Sox10'	5	0	0	0	0	0	1.666666667	0	-3.025187063	0.445317893	0.969888068
20666	'Sox11'	722	702	1201	661	735	452	875	616	-0.444117578	0.396949251	0.954358954
20667	'Sox12'	1759	1713	1284	407	1829	1807	1585.333333	1347.666667	-0.297079926	0.547770607	0.972790461
20668	'Sox13'	390	397	213	237	493	302	333.3333333	344	0.104102022	0.789112244	0.972790461
20670	'Sox15'	4	4	2	1	15	44	3.333333333	20	2.467182444	0.043234539	0.484697385

20671	'Sox17'	197	167	29	19	201	75	131	98.33333333	-0.417828364	0.630496853	0.972790461
20672	'Sox18'	223	267	21	4	483	322	170.3333333	269.6666667	0.615118259	0.608965446	0.972790461
20674	'Sox2'	8	12	14	0	2	0	11.33333333	0.666666667	-4.123596902	0.006474965	0.190560416
20675	'Sox3'	93	70	12	18	31	101	58.33333333	50	-0.214327351	0.79878128	0.972790461
20677	'Sox4'	4850	4765	10832	5011	5143	5241	6815.666667	5131.666667	-0.397948371	0.468002381	0.972790461
20678	'Sox5'	771.96	747.99	466.11	198.36	722.67	646.57	662.02	522.5333333	-0.369729942	0.405929339	0.957157474
20679	'Sox6'	539.9	474.96	160.95	74	476.01	394	391.9366667	314.67	-0.342444418	0.602612395	0.972790461
20680	'Sox7'	492	503	113	37	932	275	369.3333333	414.6666667	0.128569729	0.890537209	0.9849842
20681	'Sox8'	97	77	123	31	116	92	99	79.66666667	-0.392998274	0.466016815	0.972790461
20682	'Sox9'	154	127	717	1016	143	333	332.6666667	497.3333333	0.752940021	0.461363476	0.972790461
20683	'Sp1'	3757	3540	5246	3407	3053	3513	4181	3324.333333	-0.272366877	0.531622913	0.972790461
20684	'Sp100'	11	13	43	9	15	35	22.33333333	19.66666667	-0.326707496	0.711400862	0.972790461
20686	'Spa17'	253	225	73	38	113	170	183.6666667	107	-0.783866307	0.192102112	0.813837363
20687	'Sp3'	3019	3090	6858	2110	3151	3243	4322.333333	2834.666667	-0.651130985	0.214076768	0.842446242
20688	'Sp4'	675	706	319	414	579	452	566.6666667	481.6666667	-0.130324276	0.73864095	0.972790461
20689	'Sall3'	173	171	21	4	66	43	121.6666667	37.66666667	-1.714814203	0.072458248	0.595707787
20692	'Sparc'	9009	8532	10073	17312	13986	17846	9204.666667	16381.33333	0.914577211	0.022596521	0.372676826
20698	'Sphk1'	105	129	229	573	119	154	154.3333333	282	1.115370895	0.191483041	0.813837363
20700	'Serpina1a'	0	2.23	2.39	3	0	3.37	1.54	2.123333333	0.699700277	0.743014528	0.972790461
20701	'Serpina1b'	7.05	5	1.4	0	7	6	4.483333333	4.333333333	-0.048020061	0.97398812	0.999493374
20702	'Serpina1c'	5.02	1.77	0.22	0	5	5.31	2.336666667	3.436666667	0.694085994	0.731248105	0.972790461
20703	'Serpina1d'	0.94	0	0	1	0	3.33	0.313333333	1.443333333	2.867003166	0.470908566	0.972790461
20704	'Serpina1e'	0	0	0	0	0	1	0	0.333333333	1.020273531	0.802557913	0.972790461
20706	'Serpinb9b'	3	0	106	10	4	11	36.33333333	8.333333333	-2.214579882	0.18290168	0.804092742
20707	'Serpinb9c'	0	0	0	1	0	0	0	0.333333333	1.020273531	0.802557913	0.972790461
20708	'Serpinb6b'	2106	2046	10376	614	1420	2177	4842.666667	1403.666667	-1.970182516	0.01335082	0.288185595
20709	'Serpinb9f'	0	0	0	1	0	0	0	0.333333333	1.020273531	0.802557913	0.972790461
20713	'Serpini1'	899	1050	220	1531	852	815	723	1066	0.822942491	0.224700461	0.850759465
20714	'Serpina3k'	0	0	0	1	0	2	0	1	2.507928928	0.530239069	0.972790461
20715	'Serpina3g'	475.18	422.31	3010.7	414.33	187	332.72	1302.72	311.35	-2.058129897	0.024106857	0.383852227
20716	'Serpina3n'	16	10	267.24	59.67	6	28	97.74666667	31.22333333	-1.577785869	0.235920627	0.861636582
207165	'Bptf'	4987	4569	2303	1821	4130	3798	3953	3249.666667	-0.25333889	0.498273882	0.972790461
20717	'Serpina3m'	0	0	0	0	2	5	0	2.333333333	3.506068397	0.329985307	0.921648675
207175	'Cetn4'	188	183	49	16	113	207	140	112	-0.379519702	0.636382554	0.972790461
207181	'Rbms3'	955.84	832.17	1131.7	697.71	1045.6	1295.67	973.2366667	1012.98	0.046946282	0.893922779	0.985171
207182	'Ggt7'	21	22	21	2	27	21	21.33333333	16.66666667	-0.457823605	0.584372186	0.972790461
20719	'Serpinb6a'	3975.7	4155.6	10412	3695.3	4605.3	6350.27	6180.973333	4883.596667	-0.393796821	0.491178679	0.972790461
20720	'Serpine2'	639	710	948	915	997	903	765.6666667	938.3333333	0.345278052	0.412597498	0.95722888
207209	'Ccdc154'	0	0	1	1	0	1	0.333333333	0.666666667	1.053024025	0.762187968	0.972790461
207212	'Arhgef17'	4549	4497	3130	7153	5798	4756	4058.666667	5902.333333	0.699793718	0.113081822	0.698948431

207214	'Larp4'	2901	3071	2776	2034	2171	2588	2916	2264.333333	-0.301347434	0.310846278	0.910971113
207215	'Fbxo40'	0	2	1	0	3	6	1	3	1.446939012	0.49509141	0.972790461
207227	'Stxbp5l'	221	263	51	39	168	27	178.3333333	78	-1.121492288	0.171851268	0.788996934
20723	'Serpinb9'	451	560	701	155	650	614	570.6666667	473	-0.37270599	0.482514794	0.972790461
20724	'Serpinb5'	0	0	0	0	0	1	0	0.333333333	1.020273531	0.802557913	0.972790461
20725	'Serpinb8'	18	27	118	29	50	49	54.33333333	42.66666667	-0.46351708	0.588592787	0.972790461
207259	'Zbtb7c'	1632	1771	2097	163	1114	855	1833.333333	710.6666667	-1.482420098	0.013655084	0.292512191
20726	'Serpinb9d'	1	0	0	0	2	3	0.333333333	1.666666667	2.081200529	0.486776433	0.972790461
207278	'Fchsd2'	1766	1656	1201	1057	1353	1325	1541	1245	-0.233995673	0.3895649	0.951231321
20728	'Spic'	3	6	5	1	7	3	4.666666667	3.666666667	-0.395895125	0.739145552	0.972790461
20729	'Spin1'	5126	5235.7	3479.8	4816	4745	5475	4613.83	5012	0.221113939	0.492093477	0.972790461
20730	'Spink1'	0	0	1	0	0	0	0.333333333	0	-0.903279821	0.824807108	0.972790461
207304	'Hectd1'	7395.3	7261.8	3304.9	3789.7	6635.7	7130.06	5987.34	5851.816667	0.019264053	0.957595381	0.999493374
20731	'Spink4'	8	16	2	3	23	10	8.666666667	12	0.480727749	0.649363304	0.972790461
20732	'Spint1'	59	70	67	95	29	86	65.33333333	70	0.246020823	0.708307887	0.972790461
20733	'Spint2'	1068	1112	1129	1461	1548	2441	1103	1816.666667	0.749964463	0.02437874	0.386416857
207352	'Sec23ip'	1324	1328	1752	2191	1333	1760	1468	1761.333333	0.376186838	0.451600848	0.970649024
20737	'Spn'	32	19	9	8	44	16	20	22.66666667	0.190212487	0.814277139	0.972790461
207375	'Fam120c'	841	817	454	234	845	1083	704	720.6666667	-0.007664607	0.987764787	0.999493374
20739	'Spta1'	39	48	8	1919	65	269	31.66666667	751	5.05352206	1.56E-05	0.00358374
207393	'Elfn2'	55	40	294	53	32	59	129.6666667	48	-1.473461025	0.104782108	0.67684333
20740	'Sptan1'	9085	8391	6049	4706	8124	7123	7841.666667	6651	-0.194416203	0.449797676	0.970590732
20741	'Sptb'	432	406	257	550	210	309	365	356.3333333	0.197521582	0.742925604	0.972790461
20742	'Sptbn1'	11002	10234	6372	9629	14947	15081	9202.666667	13219	0.578135404	0.034898309	0.447758353
207425	'Wdr11'	1978	1973	825	576	1684	1506	1592	1255.333333	-0.329931375	0.464721307	0.972790461
20743	'Sptbn2'	402	382	48	72	164	147	277.3333333	127.6666667	-1.038847599	0.144702447	0.752114557
20744	'Strbp'	3422.2	3439.3	2459.9	1288.3	2329.2	2453.1	3107.143333	2023.523333	-0.596837428	0.017585622	0.331280729
20745	'Spock1'	33	19	14	17	11	41	22	23	0.110364798	0.878392501	0.981341203
20747	'Spop'	1846	1866	1803	2584	2261	2957	1838.333333	2600.666667	0.58173225	0.098824803	0.668585731
207474	'Kctd12b'	370	423	110	195	436	345	301	325.3333333	0.179408915	0.731698112	0.972790461
207495	'Baiap2l2'	4	0	6	16	3	4	3.333333333	7.666666667	1.41694539	0.345296739	0.926100052
20750	'Spp1'	15	16	1326	80531	170	16875	452.3333333	32525.33333	6.343136531	0.005087558	0.16781708
20751	'Spr'	394	427	445	142	441	524	422	369	-0.264301988	0.555940165	0.972790461
207521	'Dtx4'	435	412	817	204	340	267	554.6666667	270.3333333	-1.06068208	0.032846027	0.436386204
20753	'Sprr1a'	13	11	546	363	32	51	190	148.6666667	-0.176773032	0.905199589	0.988451813
20754	'Sprr1b'	0	0	0	1	0	1	0	0.666666667	2.022653929	0.615796212	0.972790461
20755	'Sprr2a1'	1	0	7.32	0.5	5	1	2.773333333	2.166666667	-0.682130857	0.758452395	0.972790461
20756	'Sprr2b'	0	0	18	0	1	0	6	0.333333333	-4.224638545	0.170549175	0.785702308
207565	'Camkk2'	962	886	265	988	1138	859	704.3333333	995	0.656137703	0.215164487	0.842760284
20758	'Sprr2d'	0	0	6	6	2	0	2	2.666666667	0.550108693	0.832297972	0.974662707

20759	'Sprr2e'	0	0	16	2	0	0	5.333333333	0.666666667	-2.79961582	0.371903721	0.938527219
207592	'Tbc1d16'	2079	1944	1254	936	2038	2098	1759	1690.666667	-0.046266846	0.884362213	0.983763057
207596	'Thsd4'	272	225	693	586	283	418	396.6666667	429	0.190400158	0.787948769	0.972790461
20760	'Sprr2f'	1	0	3	8	0	1	1.333333333	3	1.446132404	0.518683664	0.972790461
207607	'Ccdc40'	27	28	38	16	32	24	31	24	-0.376699406	0.495100723	0.972790461
207615	'Wdr37'	1073	1170	747	960	1090	987	996.6666667	1012.333333	0.123833783	0.704461331	0.972790461
207618	'Zfp804b'	2	7	0	0	10	2	3	4	0.406268639	0.838585279	0.974723675
20765	'Sprr2k'	1	0	33	3	1	0	11.33333333	1.333333333	-2.99708905	0.162928961	0.774492858
207667	'Skor1'	2	1	1	2	4	1	1.333333333	2.333333333	0.883270047	0.580396827	0.972790461
20768	'Sephs2'	730	787	526	824	628	1144	681	865.3333333	0.437692866	0.259431417	0.877955237
207683	'Igsf11'	129	143	84	11	895	680	118.6666667	528.6666667	2.035811687	0.046224758	0.500755043
207686	'Cfap69'	315	334	403	258	215	176	350.6666667	216.3333333	-0.587594929	0.222798758	0.850230829
207704	'Gtpbp10'	493	555	517	317	512	516	521.6666667	448.3333333	-0.201516854	0.469692905	0.972790461
207728	'Pde2a'	507	437	83	73	761	575	342.3333333	469.6666667	0.42736751	0.601548123	0.972790461
20773	'Sptlc2'	2094	2214	1122	8187	2272	3782	1810	4747	1.672903116	0.013928474	0.29304295
207740	'Ubald1'	452	416	1388	364	360	488	752	404	-0.933587457	0.153645414	0.765411885
207742	'Rnf43'	347	346	176	210	534	433	289.6666667	392.3333333	0.460166744	0.242445931	0.86449675
20775	'Sqle'	781	809	499	498	885	730	696.3333333	704.3333333	0.066820327	0.81973171	0.972790461
20776	'Tmie'	229	261	95	98	395	356	195	283	0.526530749	0.333863682	0.921648675
207777	'Tspoap1'	49	58	3	8	62	34	36.66666667	34.66666667	-0.062198386	0.951127241	0.998224185
20778	'Scarb1'	992	892	698	1722	934	1071	860.6666667	1242.333333	0.718610683	0.161814781	0.771541907
207781	'C2cd2'	540	551	208	294	558	614	433	488.6666667	0.223682135	0.599498963	0.972790461
207785	'Csrnp2'	964	937	298	304	566	426	733	432	-0.675315999	0.160827716	0.771356342
20779	'Src'	1598	1709	1731	724	1564	1832	1679.333333	1373.333333	-0.323954268	0.343785831	0.925308103
207792	'BC034090'	1270.5	1313	652	98	1450	860	1078.483333	802.6666667	-0.502248913	0.503416632	0.972790461
207798	'Gramd1c'	104	112	31	33	114	107	82.33333333	84.66666667	0.053996408	0.928571428	0.993096978
207806	'Usf3'	1796	1935	703	1397	1364	1191	1478	1317.333333	-0.005818244	0.9902089	0.999493374
207818	'Smagp'	71	77	193	46	154	249	113.6666667	149.6666667	0.231810317	0.742802115	0.972790461
207819	'Bnip5'	15	16	8	31	10	21	13	20.66666667	0.878530432	0.273809548	0.885016511
207839	'Galnt6'	58	51	9	110	174	138	39.33333333	140.6666667	1.957900949	0.00230366	0.108371515
207854	'Fmr1nb'	411	378	166	2	228	517	318.3333333	249	-0.475454537	0.677205087	0.972790461
20787	'Srebf1'	776	725	603	1091	1120	1004	701.3333333	1071.666667	0.715797163	0.040861749	0.474565411
20788	'Srebf2'	1259	1320	1320	3794	1736	1670	1299.666667	2400	1.09384854	0.067619491	0.580700792
207911	'Mchr1'	2	1	0	0	0	0	1	0	-2.286544758	0.567674219	0.972790461
207920	'Esrp1'	792	677	114	39	875	2458	527.6666667	1124	1.009776212	0.34305777	0.925308103
207921	'Fam228b'	243	235	79	12	178	83	185.6666667	91	-1.074096732	0.183014085	0.804092742
207932	'Urb1'	1111	1162	290	425	780	746	854.3333333	650.3333333	-0.312692681	0.548912622	0.972790461
207952	'Klhl25'	828	910	694	2133	1122	1507	810.6666667	1587.333333	1.143514436	0.022291377	0.370441983
207958	'Alg11'	779	751	741	733	744	877	757	784.6666667	0.120045335	0.710471369	0.972790461
207965	'Vcpkmt'	291	294	529	267	226	238	371.3333333	243.6666667	-0.552250052	0.29302534	0.900145058

208043	'Setd1b'	1014	964	514	268	1018	1181	830.6666667	822.3333333	-0.051165557	0.918691823	0.990815958
20807	'Srf'	914	859	1064	438	854	988	945.6666667	760	-0.347547734	0.326286599	0.920422149
208076	'Pknox2'	716	760	554	1527	781	808	676.6666667	1038.666667	0.825665768	0.131614153	0.72897159
208080	'Ubap1l'	28	22.15	20	89	29	18	23.38333333	45.33333333	1.254421429	0.144940256	0.752280685
208084	'Pif1'	48	61	13	23	76	23	40.66666667	40.66666667	0.0750772	0.920630946	0.991047523
208092	'Chmp6'	520	560	230	293	538	651	436.6666667	494	0.218551746	0.586971786	0.972790461
208098	'Panx3'	0	2	0	0	0	0	0.666666667	0	-1.695595436	0.675304306	0.972790461
20810	'Srm'	544	556	1729	385	517	690	943	530.6666667	-0.906430476	0.155134854	0.766453805
208104	'Mlxip'	1932	1814	1072	1543	1570	1543	1606	1552	0.071123482	0.84440859	0.975182082
20811	'Srms'	2	3	5	0	4	1	3.333333333	1.666666667	-1.12525616	0.493302994	0.972790461
208111	'Zfp976'	312.93	359.58	94.79	243	442.8	183.82	255.7666667	289.8733333	0.312375495	0.599020017	0.972790461
208117	'Aph1b'	475.85	441.82	361.48	346.69	552.35	468.14	426.3833333	455.7266667	0.143794228	0.59763734	0.972790461
20813	'Srp14'	1206	1252	1511	1229	1003	1480	1323	1237.333333	-0.035185318	0.931274635	0.993601701
208144	'Dhx37'	823	834.05	472	454	653	697	709.6833333	601.3333333	-0.174900177	0.556278172	0.972790461
208146	'Yeats2'	2680.4	2770.1	1339.9	2135.7	3186.1	2104.67	2263.46	2475.493333	0.233567601	0.552365296	0.972790461
20815	'Srpk1'	1820	1923	847	1424	1658	1785	1530	1622.333333	0.19116942	0.611673945	0.972790461
208151	'Tmem132b'	17	23	8	13	10	11	16	11.33333333	-0.35248795	0.63241431	0.972790461
208154	'Btla'	5	7	0	1	4	2	4	2.333333333	-0.698983658	0.661304789	0.972790461
208158	'Map6d1'	35	35	41	16	24	14	37	18	-0.995011583	0.076882371	0.609798765
208164	'Fam180a'	30	30	43	267	58	68	34.33333333	131	2.204426593	0.009689816	0.24220996
208166	'Gm609'	1	0	0	3	1	0	0.333333333	1.333333333	2.219991816	0.467295783	0.972790461
208169	'Slc9c1'	0	0	0	0	0	1	0	0.333333333	1.020273531	0.802557913	0.972790461
20817	'Srpk2'	1791.8	2045.8	2104.5	2315.9	2438.3	2175.02	1980.703333	2309.74	0.300290238	0.420571189	0.958511083
208171	'Tmprss7'	0	4	2	1	4	2	2	2.333333333	0.204269004	0.905294068	0.988451813
208177	'Phldb2'	1256	1208	1398	4344	1432	1463	1287.333333	2413	1.151947478	0.096064911	0.662457477
20818	'Srprb'	534	588	718	780	919	1058	613.3333333	919	0.61123858	0.0909746	0.649965349
208188	'Ghsr'	1	0	13	0	0	0	4.666666667	0	-4.80175853	0.117451516	0.705525503
208194	'Exog'	287	308	251	244	280	292	282	272	0.017905596	0.953140027	0.999032683
208198	'Btbd2'	1262	1314	1223	972	1221	1607	1266.333333	1266.666667	0.030460369	0.912283941	0.989773583
20821	'Trim21'	240	233	222	72	408	434	231.6666667	304.6666667	0.298884188	0.603150714	0.972790461
208211	'Alg1'	532.42	598.06	159.78	412.82	459.79	748.23	430.0866667	540.28	0.425447852	0.411644143	0.95722888
208213	'Tmem132c'	461	350	46	57	1000	966	285.6666667	674.3333333	1.196264612	0.212821362	0.841028891
20822	'Ro60'	1102	1155	1303	1925	1180	1063	1186.666667	1389.333333	0.385893803	0.468358257	0.972790461
208228	'Mob3a'	492	536	1592	300	495	560	873.3333333	451.6666667	-1.03952035	0.099932378	0.670873716
20823	'Ssb'	3882.9	4205.3	4385.3	2707.7	4007.2	4511.28	4157.8	3742.06	-0.140568931	0.625374398	0.972790461
208258	'Ankrd33'	1	0	1	1	1	0	0.666666667	0.666666667	0.117746761	0.96570115	0.999493374
20826	'Snu13'	2385	2590	2723	952	1762	2234	2566	1649.333333	-0.663785921	0.046085909	0.50074283
208263	'Tor1aip1'	1609.7	1699.6	1375.6	643.44	1942.5	1665.91	1561.623333	1417.276667	-0.170098777	0.649311744	0.972790461
208266	'Dot1l'	2602	2647	2303	3446	2378	1643	2517.333333	2489	0.165188347	0.753518599	0.972790461
208285	'Cyp4f17'	229.36	235	160	220	346	220	208.12	262	0.409757102	0.260669474	0.878537553

208292	'Zfp871'	1675	1854	2106	3019	2184	2042	1878.333333	2415	0.488952708	0.309548097	0.910446847
20832	'Ssr4'	940	917	1168	643	770	1240	1008.333333	884.3333333	-0.193370896	0.608257022	0.972790461
20833	'Ssrp1'	3789	3982	4066	2304	3541	4255	3945.666667	3366.666667	-0.225045552	0.427635545	0.961947592
208366	'Rpp40'	164	182	130	150	194	198	158.6666667	180.6666667	0.249734591	0.406641459	0.957157474
20840	'Stac'	3	0	1	4	0	1	1.333333333	1.666666667	0.661387342	0.774419813	0.972790461
20841	'Zfp143'	608	616	434	727	494	357	552.6666667	526	0.1231799	0.815375645	0.972790461
20842	'Stag1'	2381.4	2511.5	1993.3	1288.1	2516.1	2114.61	2295.363333	1972.923333	-0.198430571	0.454433996	0.970649024
20843	'Stag2'	3768	3761	5188	4271	4091	4430	4239	4264	0.06589844	0.873962991	0.980171118
208431	'Shroom4'	1347	1394	1452	1618	1358	1385	1397.666667	1453.666667	0.160842987	0.692378022	0.972790461
208439	'Klhl29'	61	65	119	59	26	33	81.66666667	39.33333333	-0.923801591	0.188399979	0.810712105
20844	'Stam'	1071	1056	2689	1230	998	1228	1605.333333	1152	-0.457880236	0.447204979	0.970198456
208440	'Dip2c'	2050	1946	1654	292	1019	960	1883.333333	757	-1.363075313	0.001197689	0.072517381
208449	'Sgms1'	520	559	1139	719	780	886	739.3333333	795	0.104102871	0.842206504	0.975182082
20845	'Star'	379	328	2089	6404	296	1299	932	2666.333333	1.74941495	0.140165466	0.74306325
20846	'Stat1'	939	894	764	824	883	2102	865.6666667	1269.666667	0.556232428	0.178467017	0.798139878
20847	'Stat2'	508	564	394	842	604	1089	488.6666667	845	0.888679498	0.026295933	0.397367216
20848	'Stat3'	2370	2187	5533	6819	2204	4834	3363.333333	4619	0.567733676	0.431911313	0.963677723
20849	'Stat4'	9	6	27	1	12	8	14	7	-1.185087694	0.283701835	0.892170588
20850	'Stat5a'	165.84	168.79	182.82	296.8	204.89	270.43	172.4833333	257.3733333	0.687057445	0.130413291	0.727955808
208501	'Ndufaf8'	163	160	137	35	113	141	153.3333333	96.33333333	-0.72367468	0.121190182	0.712451374
20851	'Stat5b'	1025.2	979.21	1071.2	866.2	1075.1	1325.57	1025.183333	1088.96	0.115202937	0.702549243	0.972790461
208518	'Cep78'	373	361	128	175	367	293	287.3333333	278.3333333	0.017928933	0.969079453	0.999493374
20852	'Stat6'	1091	1040	2168	386	1252	1304	1433	980.6666667	-0.667437575	0.237443141	0.86298394
20853	'Stau1'	2853	2894	2750	1762	2292	2834	2832.333333	2296	-0.27272109	0.303138063	0.906189263
20855	'Stc1'	528	319	1017	10574	281	698	621.3333333	3851	2.994292582	0.015244904	0.307526371
20856	'Stc2'	468	431	1155	558	600	931	684.6666667	696.3333333	-0.020405626	0.972282851	0.999493374
208583	'Nek11'	17	20	11	0	18	21	16	13	-0.410236132	0.714303389	0.972790461
20859	'Sult2a1'	0	1	0	0	0	0	0.333333333	0	-0.903279821	0.824807108	0.972790461
208595	'Mterf1b'	102.44	144.78	101.32	120.24	125.84	75.12	116.18	107.0666667	0.010144642	0.983479296	0.999493374
20860	'Sult1e1'	0	0	9	72	0	40	3	37.33333333	3.701861253	0.27620764	0.88793704
208606	'Rsrc2'	1687	1708	2825	1193	2019	1657	2073.333333	1623	-0.370120982	0.397063924	0.954358954
20861	'Stfa1'	0	10	1	1	41	1	3.666666667	14.33333333	1.946400252	0.295099032	0.90029104
208618	'Etl4'	1178	1177	742	285	1867	1175	1032.333333	1109	0.047142746	0.934151402	0.994249017
20862	'Stfa2'	0	16	0	0	9	0	5.333333333	3	-0.821102044	0.791766334	0.972790461
208624	'Alg3'	173	160	83	111	179	212	138.6666667	167.3333333	0.318927146	0.390357942	0.95208613
208628	'Kntc1'	1451	1452	147	26	759	906	1016.666667	563.6666667	-0.904416952	0.385961081	0.947558378
20863	'Stfa3'	0	14	0	4	39	0	4.666666667	14.33333333	1.684668274	0.46771734	0.972790461
208634	'Tspan10'	25	24	0	0	15	3	16.33333333	6	-1.451063532	0.407885558	0.957157474
208638	'Slc25a38'	399	375	472	168	526	440	415.3333333	378	-0.196122287	0.652339914	0.972790461
208643	'Eif4g1'	9890	9595	6236	6987	9639	11502	8573.666667	9376	0.182592055	0.484825469	0.972790461

208647	'Creb3l2'	950	851	1164	1582	963	1265	988.3333333	1270	0.476972068	0.339420655	0.924888762
208650	'Cblb'	996	993	3057	2736	933	882	1682	1517	0.016293711	0.984325087	0.999493374
208659	'Fam20a'	71	63	18	58	51	55	50.66666667	54.66666667	0.271286443	0.655756724	0.972790461
20866	'Stim1'	2147	2013	482	1215	3765	3634	1547.333333	2871.333333	0.919155802	0.119034864	0.709078602
208665	'Akr1d1'	0	1	0	0	0	0	0.333333333	0	-0.903279821	0.824807108	0.972790461
208666	'Diras1'	11	5	8	2	2	5	8	3	-1.410306781	0.179947791	0.800835064
20867	'Stip1'	2992	3180	3810	5538	2663	4062	3327.333333	4087.666667	0.439280648	0.415412871	0.95722888
208677	'Creb3l3'	4	3	0	14	4	7	2.333333333	8.333333333	2.127550738	0.114003739	0.698948431
20868	'Stk10'	263	236	515	358	275	313	338	315.3333333	-0.047092549	0.934217005	0.994252894
20869	'Stk11'	1730	1759	2933.7	2618	1646	2064.99	2140.873333	2109.656667	0.072238357	0.894062022	0.985171
208691	'Eif5a2'	652	575	366	168	385	535	531	362.6666667	-0.562064572	0.162456856	0.773899871
20871	'Aurkc'	32	35	9	1	21	11	25.33333333	11	-1.234369152	0.205911505	0.833294914
208715	'Hmgcs1'	5178	5969	3618	2573	5666	4103	4921.666667	4114	-0.227561497	0.481562928	0.972790461
208718	'Dis3l2'	412	449	327	154	507	411	396	357.3333333	-0.175242904	0.665418011	0.972790461
20872	'Stk16'	789.02	870.6	353.58	448.56	788.24	793.87	671.0666667	676.89	0.072011958	0.851521392	0.975734242
208727	'Hdac4'	1136.7	1094	727	1089	1042	1225.05	985.91	1118.683333	0.288369945	0.392352327	0.952648938
20873	'Plk4'	921	840	288	176	617	644	683	479	-0.512650835	0.351139586	0.928880731
20874	'Slk'	2396	2544	2623	1558	2530	2594	2521	2227.333333	-0.170113518	0.546223065	0.972790461
208748	'Prrg3'	1778	1589	670	188	1235	1319	1345.666667	914	-0.610438967	0.342436606	0.925308103
208760	'Aqp12'	6	3	0	61	4	30	3	31.66666667	3.757068494	0.004896692	0.165356847
208768	'Sde2'	1136	1137	1837	1159	993	1354	1370	1168.666667	-0.189907269	0.684352812	0.972790461
20877	'Aurkb'	148	166	80	64	199	138	131.3333333	133.6666667	0.03810235	0.93380375	0.994249017
208777	'Sned1'	2354	2337	146	1204	2811	727	1612.333333	1580.666667	0.12296278	0.890723068	0.985046501
20878	'Aurka'	300.03	263	104	76	246	244	222.3433333	188.6666667	-0.232746234	0.6550565	0.972790461
208795	'Tmem63a'	174	186	132	807	175	243	164	408.3333333	1.626999856	0.034883465	0.447758353
208820	'Triqk'	135	155	158	97	114	138	149.3333333	116.3333333	-0.32460782	0.363975884	0.93523525
208836	'Fanci'	486.02	482.9	77.25	15.53	345.11	392.65	348.7233333	251.0966667	-0.532409633	0.581817961	0.972790461
208846	'Daam1'	1379	1198	762	1188	1304	1487	1113	1326.333333	0.34668386	0.286574662	0.894199485
208869	'Dock3'	1476	1267	160	19	691	589	967.6666667	433	-1.210968519	0.242728407	0.864872493
20887	'Sult1a1'	81.14	78.98	10.22	32.83	134.88	82.39	56.78	83.36666667	0.594020676	0.448152895	0.970198456
20888	'Sult1c1'	1	0	0	0	34	70	0.333333333	34.66666667	6.430712425	0.001340197	0.078158993
208884	'Zdhhc9'	390	363	719	336	468	594	490.6666667	466	-0.111127823	0.81860097	0.972790461
208890	'Slc26a7'	455	483	34	99	1782	1301	324	1060.666667	1.68368263	0.092468858	0.653540343
208898	'Unc13c'	63	49	4	5	35	123	38.66666667	54.33333333	0.437941175	0.694407502	0.972790461
20890	'Wnt8a'	3	4	0	0	0	4	2.333333333	1.333333333	-0.858346192	0.745228625	0.972790461
208908	'Ccdc62'	578	609	135	21	560	153	440.6666667	244.6666667	-0.886589738	0.348858893	0.928792223
20892	'Cenpx'	226	253	260	127	268	390	246.3333333	261.6666667	0.03973194	0.922284865	0.991300958
208922	'Cpeb3'	258	311	180	144	271	486	249.6666667	300.3333333	0.252205645	0.557949556	0.972790461
20893	'Bhlhe40'	709	517	13478	1135	301	1248	4901.333333	894.6666667	-2.510268354	0.036225431	0.456985794
208936	'Adamts18'	8	6	1	0	17	16	5	11	1.072595141	0.451644587	0.970649024

208943	'Myo5c'	14	14	6	9	9	13	11.33333333	10.33333333	-0.046586104	0.949968103	0.998065566
208967	'Thnsl1'	641	682	203	331	477	552	508.6666667	453.3333333	-0.07695375	0.871034366	0.980126495
208968	'Zfp280c'	913	956	671	293	1381	647	846.6666667	773.6666667	-0.159777679	0.755918912	0.972790461
20897	'Stra6'	199	220	153	17	228	102	190.6666667	115.6666667	-0.806155889	0.274440331	0.885700147
208982	'Hmgcll1'	75.81	78.41	54.11	251.08	93.63	159.29	69.44333333	168	1.491012308	0.015333864	0.307843112
20899	'Stra8'	121	96	560	0	11	9	259	6.666666667	-5.528520315	2.49E-07	1.28E-04
208990	'Npb'	1	2	1	0	2	0	1.333333333	0.666666667	-1.017906319	0.681666644	0.972790461
208994	'Fam83b'	9	13	21	9	13	26	14.33333333	16	0.097357502	0.898047104	0.985794067
209003	'Rbmx2'	792	837	489	82	346	478	706	302	-1.285474985	0.017803294	0.333677259
209005	'Fsip2l'	111	115	7	1	41	71	77.66666667	37.66666667	-1.097771028	0.359005206	0.933706718
20901	'Strap'	3323	3389	3955	2701	2930	4345	3555.666667	3325.333333	-0.074207292	0.831965637	0.974662707
209011	'Sirt7'	427	389	246	393	475	382	354	416.6666667	0.341285706	0.344222292	0.925308103
209012	'Ulk4'	262	271	58	16	204	213	197	144.3333333	-0.498995461	0.555768866	0.972790461
209018	'Vps8'	1730	1571	867	463	1101	1565	1389.333333	1043	-0.424219935	0.320151553	0.917050664
209027	'Pycr1'	109	102	249	68	115	355	153.3333333	179.3333333	0.077117927	0.913363895	0.989773583
209032	'Zc3hav1l'	1802	1777	985	87	1253	1337	1521.333333	892.3333333	-0.86819289	0.258165666	0.877405867
209039	'Tns2'	1098	1052	947	444	1443	1295	1032.333333	1060.666667	-0.004861848	0.990320374	0.999493374
209047	'Gipc3'	21	24	10	6	39	24	18.33333333	23	0.302859102	0.688711851	0.972790461
20905	'Sts'	21	10	44	177	35	55	25	89	2.038696333	0.034018337	0.443914809
20907	'Stx1a'	383	400	329	367	516	272	370.6666667	385	0.146371506	0.726760729	0.972790461
20908	'Stx3'	462	412	786	443	352	459	553.3333333	418	-0.358626757	0.487681898	0.972790461
209086	'Samd9l'	149	174	151	62	238	306	158	202	0.27568261	0.594282396	0.972790461
20909	'Stx4a'	840	848	1856	524	995	972	1181.333333	830.3333333	-0.575603803	0.273513698	0.884812393
209091	'Ccnb3'	2836	2900	307	10	1272	869	2014.333333	717	-1.542931498	0.189001607	0.811494705
20910	'Stxbp1'	1364	1308	2103	4011	1009	1589	1591.666667	2203	0.689500716	0.351870997	0.929651389
20911	'Stxbp2'	1228	1329	1685	1083	1504	1933	1414	1506.666667	0.085043538	0.813568353	0.972790461
20912	'Stxbp3'	586	614	1264	844	618	966	821.3333333	809.3333333	0.00664168	0.990561715	0.999493374
20913	'Stxbp4'	1130	1203.3	372.64	566.6	1297.6	914.09	901.9666667	926.1066667	0.104019263	0.831142811	0.974324192
209131	'Snx30'	902	861	1017	859	672	906	926.6666667	812.3333333	-0.108346407	0.792072238	0.972790461
20916	'Sucla2'	1335	1443	1145	2012	1453	1864	1307.666667	1776.333333	0.567230016	0.160013569	0.771114735
20917	'Suclg2'	1891	2046	1775	916	1934	2738	1904	1862.666667	-0.065759597	0.855080969	0.975734242
209176	'Ido2'	3	1	13	0	1	0	5.666666667	0.333333333	-4.064743823	0.050573843	0.519735087
20918	'Eif1'	3771.5	3721.8	20659	4620	3732.1	5864.95	9384.09	4739.003333	-1.045291539	0.196969574	0.822696823
209186	'Acnat2'	5.16	5.02	0	2.8	4.36	3.08	3.393333333	3.413333333	-0.042932498	0.976862652	0.999493374
209195	'Clic6'	41	70	22	22	61	51	44.33333333	44.66666667	0.039076483	0.945700315	0.996592403
209200	'Dtx3l'	297.51	330.25	511.67	54.48	275.51	399.14	379.81	243.0433333	-0.793312035	0.223031274	0.85026726
209212	'Osgin2'	531	519	347	88	355	294	465.6666667	245.6666667	-0.958297879	0.038577495	0.460411379
20922	'Supt4a'	748	726	1076	597	574.35	773	850	648.1166667	-0.359972221	0.396310145	0.954259077
209224	'Enox2'	504	456	340	66	501	534	433.3333333	367	-0.331642265	0.601623884	0.972790461
209225	'Zfp710'	485	433	285	226	440	483	401	383	-0.046621463	0.883147373	0.983244267

209232	'Wfdc5'	0	1	0	0	0	0	0.333333333	0	-0.903279821	0.824807108	0.972790461
209239	'Gan'	205	163	47	171	103	102	138.3333333	125.3333333	0.094228905	0.886889173	0.984522884
20924	'Supt5'	4100	4023	4510	6363	3643	4867	4211	4957.666667	0.37156157	0.448065054	0.970198456
20926	'Supt6'	3723	3548	3726	3845	3823	4722	3665.666667	4130	0.235257407	0.47517624	0.972790461
209268	'Igsf1'	31	36	3	67	90	80	23.33333333	79	1.903011215	0.012842077	0.282912603
20927	'Abcc8'	15	9	73	3	9	7	32.33333333	6.333333333	-2.509864694	0.017093682	0.32733359
20928	'Abcc9'	586	567	74	234	847	442	409	507.6666667	0.373333163	0.611798757	0.972790461
209294	'Csta1'	0	0	0	1	0	1	0	0.666666667	2.022653929	0.615796212	0.972790461
20930	'Surf1'	272	257	536	152	303	458	355	304.3333333	-0.319486012	0.558949558	0.972790461
20931	'Surf2'	175	175	370	132	240	263	240	211.6666667	-0.246480061	0.640568396	0.972790461
209318	'Gps1'	1877	1998	1258	1048	1803	2510	1711	1787	0.075207464	0.811701731	0.972790461
20932	'Surf4'	3071	3014	5240	3032	3568	5874	3775	4158	0.109378953	0.813978294	0.972790461
20933	'Med22'	1900	2042	999	1001	1693	1833	1647	1509	-0.076545456	0.816363084	0.972790461
209334	'Gen1'	462	470	97	58	250	203	343	170.3333333	-0.995371283	0.133928918	0.733301819
20935	'Surf6'	822	908	820	523	746	885	850	718	-0.220874744	0.40583653	0.957157474
209351	'Wfdc6a'	62.83	69	3.34	3	96	160.95	45.05666667	86.65	0.898151674	0.457433239	0.972459822
209354	'Eif2b1'	722	761	923	394	720	950	802	688	-0.258691231	0.488677009	0.972790461
209357	'Gtf2h3'	661	672	456	201	588	721	596.3333333	503.3333333	-0.27950898	0.504870423	0.972790461
209361	'Taf3'	488	433	478	463	335	424	466.3333333	407.3333333	-0.087755648	0.838091481	0.974723675
20937	'Suv39h1'	2732	2816	1171	252	1581	1439	2239.666667	1090.666667	-1.083622854	0.075599974	0.605310548
209378	'Itih5'	720	749	16	230	941	749	495	640	0.419712618	0.677652184	0.972790461
209387	'Trim30d'	24	50.41	86.38	131.53	97	230.93	53.59666667	153.1533333	1.494852589	0.026050802	0.397137149
20941	'Svs4'	3	2	0	0	0	0	1.666666667	0	-3.018420173	0.443727506	0.969836424
209416	'Gpkow'	1358	1462	1062	1384	1471	1584	1294	1479.666667	0.281749972	0.3510233	0.92886351
20944	'Svs5'	11	9	0	3	62	37	6.666666667	34	2.328587872	0.067483932	0.580020123
209446	'Tfe3'	908	905	1930	1791	1151	1546	1247.666667	1496	0.325227289	0.580641472	0.972790461
209448	'Hoxc10'	51	59	229	228	66	75	113	123	0.273322012	0.768767259	0.972790461
20945	'Svs6'	3	4	0	0	9	9	2.333333333	6	1.317357471	0.46110342	0.972790461
209456	'Trp53bp2'	1043	1085	860	611	1169	1020	996	933.3333333	-0.073156959	0.780277084	0.972790461
209462	'Hace1'	694	691	466	219	555	686	617	486.6666667	-0.363479133	0.342983219	0.925308103
20947	'Swap70'	1146	1158	1030	518	1266	1316	1111.333333	1033.333333	-0.131326499	0.695261667	0.972790461
209478	'Tbc1d12'	739	695	734	404	764	759	722.6666667	642.3333333	-0.174619196	0.555937552	0.972790461
209488	'Hsh2d'	3	1	2	6	4	3	2	4.333333333	1.257817891	0.331859058	0.921648675
209497	'Tmem164'	1855	1973	950	1503	2020	1985	1592.666667	1836	0.293949454	0.387393654	0.949501169
209540	'Rtl9'	16	18	7	29	21	19	13.66666667	23	0.926218879	0.188590383	0.810884334
20955	'Vamp7'	663	804	1007	443	720	1077	824.6666667	746.6666667	-0.185339672	0.652753978	0.972790461
209550	'Rad51ap2'	623	651	140	2	260	74	471.3333333	112	-2.126823825	0.058041563	0.550916724
209558	'Enpp3'	22	34	10	7	26	33	22	22	-0.009958023	0.989078139	0.999493374
20957	'Sycp1'	7982	8135	1588	40	3805	3875	5901.666667	2573.333333	-1.27185945	0.250451159	0.871106156
209584	'Tyw3'	222.41	229.99	68.15	39.39	202.23	172.47	173.5166667	138.03	-0.343055869	0.590672248	0.972790461

209586	'Nudcd3'	1654	1430	1492	1055	1195	1460	1525.333333	1236.666667	-0.253610926	0.39603663	0.954181778
209588	'Sectm1a'	13	12	5	4	5	14	10	7.666666667	-0.361133555	0.684362028	0.972790461
209590	'Il23r'	6	9	1	2	10	2	5.333333333	4.666666667	-0.129286402	0.91962314	0.990815958
209601	'Erich3'	3	2	2	0	1	3	2.333333333	1.333333333	-0.895640667	0.607252874	0.972790461
20962	'Sycp3'	5979	5888	2635	65	2220	4484	4834	2256.333333	-1.216077178	0.218336283	0.844387594
20963	'Syk'	760	886	113	323	1059	676	586.3333333	686	0.287087879	0.683925525	0.972790461
209630	'Frmd4a'	676.14	639	395	1091	772	752	570.0466667	871.6666667	0.787553328	0.092388952	0.653540343
20964	'Syn1'	194	151	120	126	75	59	155	86.66666667	-0.635790608	0.273385772	0.884738928
209645	'Bend7'	149	139	185	26	117	85	157.6666667	76	-1.134567843	0.035790246	0.455146595
20965	'Syn2'	319	289	96	42	101	68	234.6666667	70.33333333	-1.674041501	0.00151824	0.084153859
209683	'Ttc28'	1294	1142	682	241	1437	1204	1039.333333	960.6666667	-0.168917984	0.765959744	0.972790461
20969	'Sdc1'	1212	1327	1099	1755	1832	2572	1212.666667	2053	0.816504697	0.006259305	0.187109913
209692	'Dhtkd1'	615.64	634.98	213.67	350.71	676.89	475.89	488.0966667	501.1633333	0.121620219	0.794621438	0.972790461
20970	'Sdc3'	970	875	935	1588	1077	1312	926.6666667	1325.666667	0.641055841	0.149131203	0.757738857
209707	'Lcorl'	1093.7	1054.8	1077.5	580.22	1183.5	1023.12	1075.333333	928.9533333	-0.213230536	0.479023371	0.972790461
20971	'Sdc4'	2759	2512	6237	13395	4834	8523	3836	8917.333333	1.336238747	0.05893156	0.552865869
20972	'Syngr1'	45	44	138	349	95	269	75.66666667	237.6666667	1.740024499	0.032668277	0.435852129
20973	'Syngr2'	470	413	323	375	612	835	402	607.3333333	0.607519394	0.045171291	0.495554868
209737	'Kif15'	791	828	180	20	501	442	599.6666667	321	-0.963474657	0.296118764	0.90029104
20974	'Syngr3'	5	4	8	60	3	17	5.666666667	26.66666667	2.526322727	0.035395944	0.451946059
209743	'Minar1'	13	12	141	9	2	6	55.33333333	5.666666667	-3.27705763	0.007246589	0.202308598
20975	'Synj2'	348.85	368.13	228.24	823.85	392.44	287.08	315.0733333	501.1233333	0.926583294	0.146444416	0.753611197
209760	'Tmc7'	39	32	36	104	23	31	35.66666667	52.66666667	0.841674567	0.314498582	0.914257958
20977	'Syp'	52	57	10	3	46	18	39.66666667	22.33333333	-0.843872399	0.379834068	0.943232396
209773	'Dennd2a'	1403	1486	1053	836	1357	1215	1314	1136	-0.163613158	0.511619613	0.972790461
209776	'Gpr139'	0	0	2	0	0	0	0.666666667	0	-1.995285526	0.619817042	0.972790461
20979	'Syt1'	130	122	112	13	157	115	121.3333333	95	-0.461570592	0.521968372	0.972790461
20980	'Syt2'	156	150	49	7	237	119	118.3333333	121	-0.033946941	0.971182824	0.999493374
20981	'Syt3'	15	12	2	75	35	34	9.666666667	48	2.589405403	0.002947436	0.121291461
209815	'Tbc1d25'	306	275	277	225	285	366	286	292	0.060525309	0.839721101	0.974723675
209824	'Vmn1r183'	1	1	0	0	2	0	0.666666667	0.666666667	0.038821536	0.990916081	0.999493374
20983	'Syt4'	3	5	3	0	8	12	3.666666667	6.666666667	0.741590653	0.588540109	0.972790461
209837	'Slc38a5'	12	15	1	2	12	5	9.333333333	6.333333333	-0.514008986	0.662591918	0.972790461
209966	'Pgbd5'	487	512	184	444	409	443	394.3333333	432	0.281807028	0.550835138	0.972790461
20997	'T'	0	4	0	0	2	0	1.333333333	0.666666667	-0.944810252	0.810614536	0.972790461
210004	'B3gntl1'	293.71	322.99	160.77	108.28	372.66	227.32	259.1566667	236.0866667	-0.124895859	0.789017863	0.972790461
210009	'Mtrr'	432	434	435	272	521	503	433.6666667	432	-0.007436565	0.980077685	0.999493374
210027	'Slc35f3'	17	14	15	2	11	14	15.33333333	9	-0.83787592	0.297801463	0.900843503
210029	'Metrnl'	98	111	204	105	142	197	137.6666667	148	0.064446193	0.900816025	0.98672375
210035	'Nemp1'	680	698	224	423	469	471	534	454.3333333	-0.098133715	0.837543049	0.974723675

210044	'Adcy2'	403	365	214	37	349	313	327.3333333	233	-0.568863807	0.401607313	0.955860942
210045	'Nlrp4b'	0	1	0	0	0	7	0.333333333	2.333333333	2.52210884	0.498325011	0.972790461
210094	'Iglon5'	28	12	36	58	9	6	25.33333333	24.33333333	0.25231607	0.820851788	0.972790461
210104	'Zfp658'	106	142	112	96	157	139	120	130.6666667	0.155143113	0.646501076	0.972790461
210105	'Zfp719'	483	475	1051	622	471	472	669.6666667	521.6666667	-0.298150659	0.615341273	0.972790461
210106	'Tent4a'	1029.9	1052	1038	1143	883	1036	1039.966667	1020.666667	0.082080522	0.839600246	0.974723675
210108	'D130043K22Rik'	11	11	0	9	7	7	7.333333333	7.666666667	0.279524931	0.823321247	0.972790461
210126	'Lpp'	2180	2205	2169	3601	1988	2338	2184.666667	2642.333333	0.437860284	0.387976038	0.949979571
210135	'Zfp180'	900	867	827	1356	826	781	864.6666667	987.6666667	0.362935497	0.476946869	0.972790461
210145	'Irgc1'	1	0	0	5	2	6	0.333333333	4.333333333	3.700954432	0.057154202	0.547413448
210146	'Irgq'	787	813	1157	954	808	853	919	871.6666667	-0.001708052	0.997040708	0.9996806
210148	'Slc30a6'	215	220	161	181	253	241	198.6666667	225	0.238266302	0.405097602	0.957157474
210155	'Lypd11'	7	5	5	0	4	2	5.666666667	2	-1.56731712	0.241675861	0.86449675
210162	'Zkscan2'	1771.4	1749.6	269.22	103.58	1166	608.48	1263.43	626.0266667	-1.02941541	0.22090088	0.849076011
210172	'Zfp526'	629.4	668.43	741.11	486.41	545.92	670.8	679.6466667	567.71	-0.223190974	0.507434469	0.972790461
210274	'Shank2'	158	147	30	151	115	72	111.6666667	112.6666667	0.257289693	0.720332761	0.972790461
210293	'Dock10'	126	133	254	98	117	76	171	97	-0.783875614	0.167210378	0.781391371
210297	'Lrch2'	798	799	773	491	953	885	790	776.3333333	-0.023764864	0.93342542	0.994078204
210321	'BC048679'	5	5	10	0	0	0	6.666666667	0	-5.180231286	0.0074644	0.207233844
210356	'Nckap5'	173.13	165.19	106.08	367.05	141.51	251.38	148.1333333	253.3133333	0.982096894	0.0957793	0.662411648
210376	'Mtmr9'	2204	2217	1128	758	1717	2385	1849.666667	1620	-0.193620644	0.639212292	0.972790461
210417	'Thsd7b'	282	276	74	177	199	194	210.6666667	190	-0.00700199	0.989648005	0.999493374
210463	'Slc22a22'	0	1	0	0	3	0	0.333333333	1	1.381884369	0.730152291	0.972790461
210503	'Zfp677'	201	215	222	121	327	217	212.6666667	221.6666667	0.039382679	0.921934646	0.991224424
210510	'Tdrd6'	37	30	8	4	16	7	25	9	-1.422864046	0.091740352	0.651188901
210529	'Mettl14'	643	666	558	305	666	676	622.3333333	549	-0.189253444	0.531208047	0.972790461
210530	'P3h2'	267	234	162	229	224	130	221	194.3333333	-0.029751429	0.951726873	0.998640168
210544	'Tbc1d31'	567	580	954	912	535	715	700.3333333	720.6666667	0.14012991	0.800391897	0.972790461
210554	'Hus1b'	40	36	12	0	18	22	29.33333333	13.33333333	-1.217577396	0.268776264	0.883772801
210573	'Tmem151b'	234	198	111.59	153	295	328	181.1966667	258.6666667	0.545306372	0.142487591	0.748929878
210582	'Coq10a'	178	168	145	105	192	181	163.6666667	159.3333333	-0.019253171	0.948674089	0.997521806
210583	'Gm4767'	41.73	40.49	29.77	23.57	93.44	59.95	37.33	58.98666667	0.64614186	0.234591488	0.861206181
210622	'Pamr1'	159	124	1105	194	244	285	462.6666667	241	-1.0562719	0.239184923	0.86449675
210673	'Prrt3'	9	12	9	8	3	7	10	6	-0.611148713	0.495359834	0.972790461
210710	'Gab3'	13	18	6	10	18	19	12.33333333	15.66666667	0.398009984	0.56463326	0.972790461
210711	'Mcmbp'	1481	1528	1334	1080	1520	1640	1447.666667	1413.333333	0.002173509	0.992780558	0.999493374
210719	'Mkx'	19	19	69	398	64	58	35.66666667	173.3333333	2.532232283	0.016165242	0.316838738
210741	'Kcnk12'	3	6	8	0	4	4	5.666666667	2.666666667	-1.225493799	0.363121749	0.935009831
210757	'Themis'	1	1	2	0	2	0	1.333333333	0.666666667	-1.088957393	0.659967391	0.972790461
210762	'Ppp1r36'	30	17	3	14	28	16	16.66666667	19.33333333	0.325159896	0.706988033	0.972790461

210766	'Brcc3'	394	436	590	213	515	544	473.3333333	424	-0.220505413	0.609295852	0.972790461
210789	'Tbc1d4'	1255.9	1346	891	1493	1304	1150.11	1164.303333	1315.703333	0.318800831	0.433407644	0.964977881
210801	'Unc5d'	80	78	9	6	118	87	55.66666667	70.33333333	0.302592578	0.76319163	0.972790461
210808	'Laccl'	300	244	279	89	224	199	274.3333333	170.6666667	-0.711606359	0.059235022	0.552865869
210853	'Zfp947'	94.99	80.04	99.03	23	113	144.68	91.35333333	93.56	-0.070103644	0.907753183	0.988823459
210876	'Vmn2r111'	0	0	0	0	0	1	0	0.333333333	1.020273531	0.802557913	0.972790461
210925	'Ints9'	534	553	308	504	497	568	465	523	0.28108688	0.433962439	0.96508292
210933	'Adgrb3'	35	45	29	0	39	18	36.33333333	19	-1.047979552	0.34294164	0.925308103
210940	'4931408C20Rik'	0	0	0	0	0	1	0	0.333333333	1.020273531	0.802557913	0.972790461
210973	'Kbtbd2'	1401.8	1475.4	2317.9	825.98	1130.9	1208.89	1731.726667	1055.24	-0.72143858	0.086149348	0.637027553
210982	'Bicral'	1077	1075	696	795	1332	1102	949.3333333	1076.333333	0.236831477	0.402281756	0.955996639
210992	'Lpcat1'	642	652	253	336	768	947	515.6666667	683.6666667	0.426301908	0.34769282	0.928444937
210998	'Fam91a1'	4238	4127	4181	6112	3304	4834	4182	4750	0.327890737	0.501360286	0.972790461
211006	'Sepsecs'	1078	1037	527	588	763	743	880.6666667	698	-0.241974523	0.487026588	0.972790461
211007	'Trim41'	2048	2034	1205	730	1499	1623	1762.333333	1284	-0.439291253	0.169344471	0.785100556
211064	'Alkbh1'	295.75	291.81	527.87	96.9	273	283.94	371.81	217.9466667	-0.866892334	0.09719699	0.663789939
211134	'Lzts1'	1307	1393	81	388	935	461	927	594.6666667	-0.520083492	0.540221593	0.972790461
211135	'D130040H23Rik'	57	55	13	31	65	57	41.66666667	51	0.361905421	0.558005978	0.972790461
211147	'Marchf11'	2	4	5	4	1	3	3.666666667	2.666666667	-0.350295296	0.78990901	0.972790461
211151	'Churc1'	429.92	473	413.99	159.41	323	486	438.97	322.8033333	-0.475766276	0.210122314	0.838372599
211187	'Lrtm2'	5	2	18	2	3	3	8.333333333	2.666666667	-1.733501292	0.173153541	0.791556755
211208	'Gm382'	0	0	0	0	0	1	0	0.333333333	1.020273531	0.802557913	0.972790461
211223	'Vmn2r15'	0	2	0	0	1	0	0.666666667	0.333333333	-0.733814424	0.856146772	0.975734242
211228	'Lrrc25'	13	13	7	36	10	12	11	19.33333333	1.098308946	0.228325432	0.854848979
211232	'Cpne9'	6	1	2	2	3	7	3	4	0.412834223	0.753175611	0.972790461
211253	'Mtrf1'	179	160	120	71.99	210	242	153	174.6633333	0.156140344	0.717307156	0.972790461
211255	'Kbtbd7'	1174.1	1294.1	616.82	434.86	1023.7	1130.69	1028.36	863.0833333	-0.241907251	0.535270668	0.972790461
211286	'Cln5'	368	349	633	652	587	747	450	662	0.588329773	0.230118758	0.856721174
211305	'Fbxw13'	0	0	0	0	1	1	0	0.666666667	1.775692139	0.660844521	0.972790461
211323	'Nrg1'	133	119.77	192	302	196.68	156	148.2566667	218.2266667	0.694087089	0.243217861	0.865044378
211329	'Ncoa7'	541	549	937	544	666	676	675.6666667	628.6666667	-0.095471873	0.833369477	0.974723675
211347	'Pank3'	6608	7039	4408	8508	6794	7225	6018.333333	7509	0.463598891	0.249813071	0.870208199
211378	'6720489N17Rik'	145	132	71	56	152.01	129.93	116	112.6466667	-0.032262713	0.940701919	0.996019386
211383	'Amer3'	2	3	1	0	2	0	2	0.666666667	-1.574167598	0.489121124	0.972790461
211389	'Suox'	187	205	104	151	282	291	165.3333333	241.3333333	0.583743665	0.095901791	0.662457477
211401	'Mtss1'	1122	1053	1549	278	1322	1235	1241.333333	945	-0.50757245	0.359545683	0.933706718
211429	'Pla2g4b'	220.51	227.37	121.83	174.56	534.44	214.12	189.9033333	307.7066667	0.733975727	0.146036675	0.753611197
211446	'Exoc3'	1695	1778	1093	990	1595	1949	1522	1511.333333	0.022753365	0.93536712	0.994413066
211468	'Kcnh8'	2	3	14	0	8	1	6.333333333	3	-1.299626408	0.43189412	0.963677723
211472	'Olfr1373'	0	1	0	0	0	0	0.333333333	0	-0.903279821	0.824807108	0.972790461

211480	'Kcnj14'	16	9	14	3	10	5	13	6	-1.130352604	0.182688407	0.803812079
211482	'Efhb'	21	13	6	20	18	13	13.33333333	17	0.514416594	0.498223208	0.972790461
211484	'Tsga10'	901	860	163	67	450	285	641.3333333	267.3333333	-1.264019128	0.087650905	0.642214018
211488	'Ado'	994.06	1034.4	806.33	880.62	851.14	1015.42	944.9133333	915.7266667	0.043493715	0.891701027	0.985171
211499	'Tmem87a'	838.69	915.94	624.78	454	910	900.83	793.1366667	754.9433333	-0.054964005	0.844503999	0.975182082
211535	'Ccdc114'	132	167	88	63	275	133	129	157	0.273867588	0.61032246	0.972790461
211548	'Nomo1'	1374	1370	1353	2233	1847	2258	1365.666667	2112.666667	0.7246957	0.054614086	0.536322519
211550	'Tifa'	135	137	310	97	253	211	194	187	-0.145483017	0.800925955	0.972790461
211556	'Aplar'	1186	1290	988	999	1324	1414	1154.666667	1245.666667	0.162822165	0.505550231	0.972790461
211577	'Mrgprf'	5	2	0	36	4	11	2.333333333	17	3.25309683	0.020097029	0.351180801
211586	'Tfdp2'	2502	2589	1688	14605	3280	5561	2259.666667	7815.333333	2.086593847	0.004006228	0.145912662
211612	'Ptchd1'	5	7	57	1	18	20	23	13	-1.109686548	0.383877098	0.945936404
211623	'Plac9a'	10	19	6	6	36	20	11.66666667	20.66666667	0.810769713	0.327332887	0.920734892
211651	'Fancd2'	815	749.99	131.83	25	710	1126.97	565.6066667	620.6566667	0.063277163	0.950293508	0.998065566
211652	'Wwc1'	463	409	325	1718	311	593	399	874	1.437723522	0.063656082	0.567649824
211660	'Cspp1'	1044	1135	336	952	994	647	838.34	864.3333333	0.225223627	0.681615841	0.972790461
211666	'Mgst2'	6	13	5	11	15	36	8	20.66666667	1.376145068	0.080756891	0.618593878
211673	'Arfgef1'	4710	4509	3474	3559	3828	4532	4231	3973	-0.012724884	0.964339621	0.999493374
211712	'Pcdh9'	126	140	125	186	205	173	130.3333333	188	0.613814215	0.106326101	0.679124905
211739	'Vstm2a'	6	3	2	1	17	10	3.666666667	9.333333333	1.285388169	0.281424081	0.889731741
211770	'Trib1'	238	253	411	176	280	234	300.6666667	230	-0.396055052	0.382916857	0.945352503
211798	'Mfsd9'	81	72	76	142	126	140	76.33333333	136	0.922815844	0.027116456	0.403710299
211896	'Depdc7'	225	255	91	70	64	91	190.3333333	75	-1.223040673	0.011388377	0.264366075
211914	'Asap2'	648.62	677.58	488.57	335.9	612.36	557.94	604.9233333	502.0666667	-0.239727353	0.360926178	0.933706718
211922	'Dennd6a'	1027	977	876	550	977	866	960	797.6666667	-0.244508048	0.345999805	0.927022024
211924	'Dsg1c'	2	1	0	0	1	0	1	0.333333333	-1.329381507	0.692813154	0.972790461
211936	'Ccdc73'	1270	1313	225	25	708	927	936	553.3333333	-0.825433364	0.404373597	0.956545617
211945	'Plekhh1'	550.02	538.32	615.7	136.3	390.63	507	568.0133333	344.6433333	-0.794279506	0.07678068	0.609792246
211948	'Pde12'	351	411	350	327	426	472	370.6666667	408.3333333	0.182207533	0.516617592	0.972790461
211949	'Spsb4'	145	180	220	17	149	118	181.6666667	94.66666667	-1.070850612	0.116644483	0.704546565
211961	'Asxl3'	447	437	236	123	278	233	373.3333333	211.3333333	-0.78961201	0.03111072	0.42861408
211978	'Zfyve26'	848	841	479	949	890	913	722.6666667	917.3333333	0.473535454	0.21441986	0.84261066
211986	'Tmem18'	705	752	205	474	807	823	554	701.3333333	0.421729229	0.393933316	0.953178011
212032	'Hk3'	34	33	5	17	50	75	24	47.33333333	0.99459369	0.205805748	0.833294914
212073	'Syne3'	28	26	28	255	50	72	27.33333333	125.6666667	2.495167587	0.003051575	0.123439464
212085	'Trim52'	634	604	66	8	243	273	434.6666667	174.6666667	-1.367758876	0.189467337	0.812029435
212090	'Tmem60'	271	257	567	465	236	398	365	366.3333333	0.081456286	0.897225716	0.985794067
212108	'Rln3'	0	0	0	1	0	1	0	0.666666667	2.022653929	0.615796212	0.972790461
212111	'Inpp5a'	1170	1170	3669	2809	1212	1791.99	2002.983333	1937.653333	0.039280971	0.957986886	0.999493374
212114	'Nhlrc3'	175	134	103	189	115	185	137.3333333	163	0.386010191	0.420866563	0.958945978

212123	'Dcaf15'	771	704	368	566	775	584	614.3333333	641.6666667	0.167963758	0.66015351	0.972790461
212124	'Cfap46'	40	23	7	15	31	28	23.33333333	24.66666667	0.148784852	0.836102487	0.974723675
212127	'Proser1'	695	727	667	661	699	547	696.3333333	635.6666667	-0.032373231	0.932185547	0.993860034
212139	'Cc2d1a'	819	807	608	1091	778	1152	744.6666667	1007	0.554395482	0.163932611	0.776592614
212153	'Ccdc191'	582	535	150	51	449	443	422.3333333	314.3333333	-0.470545416	0.528308947	0.972790461
212163	'8030462N17Rik'	1314	1344	1280	1180	824	1196	1312.666667	1066.666667	-0.193147412	0.638891797	0.972790461
212167	'Gsap'	126.65	150.55	153.31	104.73	102.99	93.48	143.5033333	100.4	-0.438846248	0.304012633	0.906771226
212168	'Zswim4'	1327	1214	2013	2452	869	1120	1518	1480.333333	0.150765219	0.823328529	0.972790461
212190	'Ubxn10'	95	99	22	33	83	113	72	76.33333333	0.110535004	0.86311923	0.977286808
212198	'Wdr25'	299	305	159	48	193	212	254.3333333	151	-0.787191845	0.127754122	0.723917691
212276	'Zfp748'	503	516	341	213	448	384	453.3333333	348.3333333	-0.356200235	0.242558811	0.864553452
212281	'Zfp729a'	478.13	514.82	489.94	462.79	471.68	517.63	494.2966667	484.0333333	0.042051432	0.900585688	0.986627584
212285	'Arap2'	384	382	607	201	566	689	457.6666667	485.3333333	-0.010696591	0.982941007	0.999493374
212307	'Mapre2'	564	569	519	546	578	601	550.6666667	575	0.139483644	0.664136469	0.972790461
212326	'Fam149a'	225	225	184	485	273	248	211.3333333	335.3333333	0.861747671	0.116652701	0.704546565
212377	'Mms22l'	709	686	153	62	418	366	516	282	-0.891583068	0.216665486	0.843391934
212390	'Klhl32'	90	107	26	21	38	36	74.33333333	31.66666667	-1.149941731	0.052847781	0.532526438
212391	'Lcor'	1060	1030	774	557	711	1012	954.6666667	760	-0.290469732	0.296503021	0.90029104
212392	'Ccdc110'	2	4	0	1	2	2	2	1.666666667	-0.169087667	0.924054176	0.991932584
212398	'Frat2'	96	117	219	607	132	200	144	313	1.342807863	0.106086434	0.679124905
212427	'A730008H23Rik'	511.28	499.19	337.85	182.28	400.89	353.49	449.44	312.22	-0.509383962	0.104808898	0.67684333
212439	'AA986860'	26	18	43	90	19	28	29	45.66666667	0.882794224	0.330832124	0.921648675
212442	'Lactb2'	241	266	543	294	302	392	350	329.3333333	-0.093622651	0.861589442	0.976863639
212448	'9330159F19Rik'	18	17	4	1	12	2	13	5	-1.362472106	0.240638489	0.86449675
212483	'Fam193b'	1834	1720	905	918	2130	1173	1486.333333	1407	-0.023982865	0.95394923	0.999255249
212503	'Paox'	522.79	552.26	448.21	190.25	524.15	694.55	507.7533333	469.65	-0.160413062	0.700278083	0.972790461
212508	'Mtg1'	305	326	150	39	278	248	260.3333333	188.3333333	-0.522554394	0.410184565	0.957157474
212514	'Spice1'	1108	1054	393	316	772	610	851.6666667	566	-0.543792126	0.23549407	0.861636582
212516	'Efcab12'	75	49	5	37	79	129	43	81.66666667	0.972448629	0.248654744	0.869491261
212517	'Cfap44'	23	22	0	4	25	7	15	12	-0.260723508	0.845707426	0.975182082
212518	'Sprn'	42	31	3	0	68	25	25.33333333	31	0.25055741	0.857942582	0.976475731
212528	'Trmt1'	1281	1258	1409	746	1304	1305	1316	1118.333333	-0.237097607	0.433264888	0.964886031
212531	'Sh3bgrl2'	412	427	369	1483	303	455	402.6666667	747	1.191556955	0.121928255	0.712603747
212539	'Gm266'	77	80	111	89	51	45	89.33333333	61.66666667	-0.387546594	0.534254655	0.972790461
212541	'Rho'	28	26	16	34	29	11	23.33333333	24.66666667	0.27287242	0.710945186	0.972790461
212547	'Nepro'	1190.7	1257.1	1148.1	849.51	1049	919.25	1198.646667	939.25	-0.284874517	0.357465765	0.932505998
212555	'Slc66a1'	220	205	95.81	285	302	269	173.6033333	285.3333333	0.852920813	0.049489016	0.51583617
212569	'Zfp273'	122.9	133.21	67.74	68	204	162	107.95	144.6666667	0.437165266	0.325662045	0.920269559
212627	'Prpsap2'	528	517	540	26	648	720	528.3333333	464.6666667	-0.339002349	0.694973285	0.972790461
212632	'Iffo2'	511	525	624	934	568	568	553.3333333	690	0.465302378	0.376218141	0.940891778

212647	'Aldh4a1'	395	409	174	488	554	600	326	547.3333333	0.858291297	0.031156357	0.42861408
212670	'Catsper2'	256.03	253.08	74.01	5	219.05	109.02	194.3733333	111.0233333	-0.872725897	0.369329784	0.937712122
212679	'Mars2'	210	189	82	201	153	155	160.3333333	169.6666667	0.258790711	0.614921709	0.972790461
212706	'N4bp3'	621	609	348	158	883	576	526	539	-0.005201622	0.992419595	0.999493374
212712	'Satb2'	361	385	104	133	133	98	283.3333333	121.3333333	-1.044186952	0.069160499	0.586644191
212728	'Tarbp1'	320	282	167	281	277	217	256.3333333	258.3333333	0.154109794	0.72311083	0.972790461
212733	'Bicdl2'	4	1	2	12	4	20	2.333333333	12	2.439354805	0.033842914	0.443127738
212772	'Arl14ep'	939	941	1424	546	1028	1122	1101.333333	898.6666667	-0.336364925	0.412658163	0.95722888
212862	'Chpt1'	817	914	434	679	1273	1125	721.6666667	1025.666667	0.559748107	0.104509158	0.67684333
212880	'Ddx46'	2658	2574	1601	1840	2600	2578	2277.666667	2339.333333	0.106870316	0.698704446	0.972790461
212892	'Rsph4a'	9	6	10	20	4	14	8.333333333	12.66666667	0.767232248	0.428581976	0.962386546
212898	'Dse'	458	387	707	272	535	657	517.3333333	488	-0.149097328	0.747994189	0.972790461
212919	'Kctd7'	208	228	383	208	243	268	273	239.6666667	-0.181094126	0.70140625	0.972790461
212933	'Pm20d1'	101	139	9	5	300	123	83	142.6666667	0.749849001	0.519244223	0.972790461
212937	'Tifab'	47	54	3	3	20	45	34.66666667	22.66666667	-0.639225259	0.560536835	0.972790461
212943	'Tent5a'	228	169	880	319	139	160	425.6666667	206	-0.963953078	0.259479827	0.877955237
212952	'Mageb11'	0	0	1	0	0	0	0.333333333	0	-0.903279821	0.824807108	0.972790461
212974	'Pgghg'	790	838	360	367	988	754	662.6666667	703	0.113139585	0.791195663	0.972790461
212980	'Slc45a3'	96	97	68	2475	68	566	87	1036.333333	3.965505198	2.33E-04	0.02516575
212986	'Scfd2'	206	199	201	131	184	298	202	204.3333333	0.012237915	0.972739334	0.999493374
212989	'Best2'	2	2	1	1	1	2	1.666666667	1.333333333	-0.26841044	0.871454791	0.980171118
212996	'Galnt17'	56	42	11	4	25	45	36.33333333	24.66666667	-0.59833065	0.495224411	0.972790461
212998	'BC016579'	2	2	0	7	0	0	1.333333333	2.333333333	1.360651362	0.61120764	0.972790461
212999	'Tnpo2'	2079	2062	2883	1867	2041	2151	2341.333333	2019.666667	-0.174170187	0.660864701	0.972790461
213002	'Ifitm6'	0	3	0	8	3	0	1	3.666666667	2.297006567	0.354725682	0.931809687
213006	'Mfsd4a'	943.31	1064.2	1010.6	560.74	970.22	708.36	1006.063333	746.44	-0.401645417	0.209136176	0.83689686
213011	'Zfp583'	239	275	233	97	192	190	249	159.6666667	-0.638569249	0.040725311	0.473970635
213012	'Abhd10'	505	534	242	82	603	434	427	373	-0.24395854	0.697518001	0.972790461
213019	'Pdlim2'	120	115	109	255	119	160	114.6666667	178	0.813367673	0.149801755	0.759066532
213027	'Evi5l'	982	1033	821	559	887	815	945.3333333	753.6666667	-0.287264258	0.240455245	0.86449675
213043	'Aox2'	14	8	2	4	5	7	8	5.333333333	-0.491667028	0.63095679	0.972790461
213053	'Slc39a14'	468	468	329	1368	576	558	421.6666667	834	1.231801532	0.047445155	0.505054472
213054	'Gabpb2'	3315	3244	1544	862	2312	1921	2701	1698.333333	-0.649898924	0.110443515	0.691390221
213056	'Fam126b'	1045	1119	772	711	831	779	978.6666667	773.6666667	-0.249802466	0.411491758	0.95722888
213068	'Tmem71'	4	1	9	1	4	1	4.666666667	2	-1.313224759	0.374257562	0.940069332
213081	'Wdr19'	841	825	214	269	876	722	626.6666667	622.3333333	0.018611212	0.974123434	0.999493374
213084	'Cdkl3'	683	710	187	127	465	257	526.6666667	283	-0.858549432	0.147311356	0.754291274
213109	'Phf3'	3111	3109	3795	6463	3072	3400	3338.333333	4311.666667	0.54588435	0.345955897	0.927022024
213119	'Itga10'	40	30	29	64	70	31	33	55	0.870446864	0.148188951	0.75654359
213121	'Ankrd35'	109	132	4	185	151	51	81.66666667	129	0.958405785	0.355103158	0.931809687

213171	'Prss27'	1	0	2	0	1	1	1	0.666666667	-0.74235737	0.776278206	0.972790461
213208	'Il20rb'	11	11	23	18	13	8	15	13	-0.106955755	0.899419535	0.986106073
213211	'Rnf26'	527	536	543	484	663	774	535.3333333	640.3333333	0.281909986	0.320305645	0.917254244
213233	'Tapbpl'	125	106	68	27	171	126.16	99.66666667	108.0533333	0.063190734	0.916595594	0.990526893
213234	'Zbbx'	4	7	1	0	0	0	4	0	-4.30818135	0.070116065	0.589465353
213236	'Dnd1'	46	65	39	22	70	108	50	66.66666667	0.364436864	0.518489948	0.972790461
213248	'Wdr49'	32	43	0	0	1	1	25	0.666666667	-5.157754115	0.004848941	0.164621536
213262	'Fstl5'	1	0	0	0	1	0	0.333333333	0.333333333	0.058500858	0.988561293	0.999493374
213272	'Txndc2'	12	21	3	1	13	9	12	7.666666667	-0.659018653	0.546374138	0.972790461
21331	'T2'	3	2	3	7	5	5	2.666666667	5.666666667	1.193440204	0.284422535	0.893053879
213311	'Fbxl21'	99	97	22	60	48	51	72.66666667	53	-0.269457414	0.679450059	0.972790461
213326	'Scyl2'	1681	1655	1787	2173	2030	1939	1707.666667	2047.333333	0.354066558	0.360101314	0.933706718
21333	'Tac1'	0	1	4	6	2	0	1.666666667	2.666666667	0.871432619	0.687189789	0.972790461
213332	'Mfsd4b4'	1039	1069.6	218	137	1049	580	775.5166667	588.6633333	-0.405880558	0.583723945	0.972790461
21334	'Tac2'	29	55	125	12	12	6	69.66666667	10	-2.772524553	8.99E-04	0.062657876
21335	'Tacc3'	905.59	883.57	312.11	101.4	583.82	756.01	700.4233333	480.41	-0.5917742	0.369050027	0.937712122
213350	'Gatd1'	694	732	711	333	682	823	712.3333333	612.6666667	-0.243026116	0.462763824	0.972790461
21336	'Tacr1'	2	0	1	0	2	0	1	0.666666667	-0.645083752	0.838793988	0.974723675
21337	'Tacr2'	5	7	0	1	5	1	4	2.333333333	-0.690478602	0.680502562	0.972790461
21338	'Tacr3'	17	23	16	14	30	24	18.66666667	22.66666667	0.299624321	0.591712113	0.972790461
213389	'Prdm9'	1399	1309	696	140	526	247	1134.666667	304.3333333	-1.886954278	1.58E-04	0.01938235
21339	'Taf1a'	497	478	271	162	443	444	415.3333333	349.6666667	-0.25096599	0.529554032	0.972790461
213391	'Rassf4'	152	143	827	450	88	86	374	208	-0.653314586	0.535368809	0.972790461
213393	'Depp1'	51	29	70	4	31	16	50	17	-1.67291776	0.032310284	0.434162511
21340	'Taf1b'	840	940	431	146	601	622	737	456.3333333	-0.722680374	0.161452871	0.771435531
213402	'Armc2'	49	65	9	41	79	26	41	48.66666667	0.393918859	0.618685089	0.972790461
213409	'Lemd1'	2	4	3	2	1	0	3	1	-1.385571711	0.420972943	0.958945978
21341	'Taf1c'	354.27	357.35	218.17	203.78	428.85	382.06	309.93	338.23	0.151014105	0.639308327	0.972790461
213417	'Klhdc8a'	15	18	20	7	6	7	17.66666667	6.666666667	-1.349913076	0.065210635	0.573626167
21343	'Taf6'	1605.4	1570.1	783.64	867.8	1135	1303.58	1319.71	1102.126667	-0.180678144	0.594126565	0.972790461
213435	'Mylk3'	87	78	6	10	103	100	57	71	0.297911327	0.763375891	0.972790461
213436	'Rtl3'	97	110	2	0	338	188	69.66666667	175.3333333	1.29427709	0.410210346	0.957157474
213438	'P2ry10b'	3	4	0	1	9	11	2.333333333	7	1.566018241	0.28257701	0.890642259
213439	'Gpr174'	6	4	2	0	10	14	4	8	0.903238349	0.51075239	0.972790461
213449	'Tent5d'	41	38	5	1	35	33	28	23	-0.330587313	0.766608431	0.972790461
21345	'Tagln'	558	563.86	1379.4	332.9	715.89	803.89	833.7633333	617.56	-0.534890769	0.351035363	0.92886351
213452	'Dstyk'	1429	1444	892	484	1700	1520	1255	1234.666667	-0.050080613	0.90738041	0.988823459
213454	'Gm378'	0	1	0	0	1	0	0.333333333	0.333333333	0.058500858	0.988561293	0.999493374
21346	'Tagln2'	780	728	3959	1627	1099	1720	1822.333333	1482	-0.326338915	0.687813346	0.972790461
213464	'Rbbp5'	1217	1302	1238.7	943	1231	1296.7	1252.563333	1156.9	-0.072850453	0.791643861	0.972790461

213469	'Lgi3'	129	149	8	66	181	122	95.33333333	123	0.455792311	0.598070449	0.972790461
213484	'Nudt18'	206	192	361	360	178	317	253	285	0.259944793	0.66459433	0.972790461
21349	'Tal1'	100	91	9	1	135	42	66.66666667	59.33333333	-0.203681392	0.869295418	0.979633196
213491	'Szrd1'	1422	1434	2365	1972	1545	1699	1740.333333	1738.666667	0.070644907	0.888341002	0.984577587
213498	'Arhgef11'	1957.2	1940.6	1780.6	1082.3	2057.4	1911.71	1892.773333	1683.8	-0.16112674	0.542471342	0.972790461
213499	'Fbxo42'	1489	1527	1660	1413	1060	1160	1558.666667	1211	-0.251738636	0.562548625	0.972790461
21350	'Tal2'	4	4	6	1562	4	218	4.666666667	594.6666667	7.378938027	2.23E-07	1.21E-04
21351	'Taldo1'	848	908	1268	2891	1067	2322	1008	2093.333333	1.186941388	0.050181167	0.518969204
213522	'Plekhg6'	16	16	19	157	28	37	17	74	2.423768246	0.008036975	0.216529539
213527	'Pth2r'	3	7	4	1	2	3	4.666666667	2	-1.21840505	0.331511719	0.921648675
21353	'Tank'	489	468	537	881	526	529	498	645.3333333	0.52854917	0.312385121	0.911537035
213539	'Bag2'	285	300	765	384	357	400	450	380.3333333	-0.236577218	0.696579781	0.972790461
21354	'Tap1'	174	208	267	57	294	363	216.3333333	238	0.009458233	0.987706447	0.999493374
213541	'Ythdf2'	2040	2110	1289	725	1593	1833	1813	1383.666667	-0.387205694	0.248318991	0.869491261
21355	'Tap2'	220	193	99	225	156	297	170.6666667	226	0.526618943	0.266017804	0.882486564
213550	'Dis3l'	860	795	433	69	599	595	696	421	-0.801322957	0.230678792	0.857634106
213556	'Plekhh2'	887	939	305	1355	916	845	710.3333333	1038.666667	0.767143916	0.179148412	0.798783498
21356	'Tapbp'	1158	1186	739	5933	1812	2714	1027.666667	3486.333333	2.028144604	0.001969071	0.097744074
21357	'Tarbp2'	572	606	396	190	525	439	524.6666667	384.6666667	-0.451811811	0.214620362	0.842635023
213573	'Cracr2b'	85	95	202	86	142	247	127.3333333	158.3333333	0.2199563	0.710213678	0.972790461
213575	'Dync2li1'	759	794	380	71	611	667	644.3333333	449.6666667	-0.59411826	0.383156125	0.945460723
213582	'Map9'	295	310	257	95	288	199	287.3333333	194	-0.583743944	0.139985882	0.742885537
213603	'Slc44a3'	54	67	38	266	81	147	53	164.6666667	1.878423129	0.005506617	0.175069909
213649	'Arhgef19'	653	645	214	283	360	253	504	298.6666667	-0.613319812	0.220118172	0.848689247
21366	'Slc6a6'	844	749	571	8334	923	2232	721.3333333	3829.666667	2.759245395	0.00156255	0.084694293
21367	'Cntn2'	67	51	10	1	45	56	42.66666667	34	-0.392652267	0.719987593	0.972790461
213673	'9530068E07Rik'	2976	3071	1958	3221	4639	5122	2668.333333	4327.333333	0.751186609	0.003013608	0.122594353
213696	'Duoxa1'	1	0	0	2	0	0	0.333333333	0.666666667	1.337350669	0.73871782	0.972790461
21371	'Tbca'	1424	1417	2352	1029	1095	1468	1731	1197.333333	-0.524968247	0.239150847	0.86449675
21372	'Tbl1x'	5248	4945	6284	7038	5557	5783	5492.333333	6126	0.25757377	0.561456682	0.972790461
21374	'Tbp'	591	662	579	348	522	527	610.6666667	465.6666667	-0.360164664	0.170663349	0.785820195
21375	'Tbr1'	22	25	22	1	10	5	23	5.333333333	-2.154924997	0.009193178	0.234872395
213753	'Zfp598'	905	850	1031	1590	923	1032	928.6666667	1181.666667	0.493833692	0.340407517	0.925308103
21376	'Tbrg1'	1413	1388	4563	1293	1471	2273	2454.666667	1679	-0.619044342	0.342079811	0.925308103
213760	'Prepl'	1007.9	1162.7	628.48	837.75	1357.2	1181.23	933.0166667	1125.386667	0.334768451	0.272952278	0.884738928
213765	'Nutm1'	18	9	1	2	9	9	9.333333333	6.666666667	-0.45868397	0.692263773	0.972790461
213773	'Tbl3'	810	875	334	273	707	1089	673	689.6666667	0.027129109	0.957626336	0.999493374
213783	'Plekhg1'	1099	1069	389	349	1289	931	852.3333333	856.3333333	0.017362699	0.973529127	0.999493374
213788	'Chrm5'	15	10	1	0	6	1	8.666666667	2.333333333	-1.887285844	0.230168067	0.856721174
21379	'Tbrg4'	681	663	752	285	599	693	698.6666667	525.6666667	-0.444839352	0.207204939	0.83479753

21380	'Tbx1'	21	20	18	6	19	12	19.66666667	12.33333333	-0.678217416	0.297129988	0.900647119
213819	'Casd1'	992	994	356	231	1498	929	780.6666667	886	0.158788613	0.800638504	0.972790461
213827	'Arcn1'	4898	4872	9754	4952	4779	6945	6508	5558.666667	-0.22981185	0.652310805	0.972790461
21384	'Tbx15'	3	1	10	7	4	6	4.666666667	5.666666667	0.291874258	0.81441497	0.972790461
21385	'Tbx2'	923	882	394	523	1000	541	733	688	-0.002584946	0.995387428	0.999562152
21386	'Tbx3'	216	188	361	260	167	98	255	175	-0.398420782	0.552022902	0.972790461
21387	'Tbx4'	42	28	8	1	11	29	26	13.66666667	-0.990993734	0.342162928	0.925308103
21388	'Tbx5'	0	0	0	0	0	1	0	0.333333333	1.020273531	0.802557913	0.972790461
21389	'Tbx6'	8	10	7	4	5	14	8.333333333	7.666666667	-0.128870834	0.883169013	0.983244267
213895	'Bms1'	3065	3109	1685	1291	2373	2709	2619.666667	2124.333333	-0.271313385	0.399553235	0.955510201
21390	'Tbxa2r'	45	38	4	5	43	18	29	22	-0.382409705	0.700836027	0.972790461
21391	'Tbxas1'	7	5	0	15	14	32	4	20.33333333	2.443779819	0.017870555	0.333747543
21393	'Tcap'	4	2	1	6	7	2	2.333333333	5	1.263261176	0.326076426	0.920269559
213945	'Col28a1'	26	20	6	9	38	21	17.33333333	22.66666667	0.412016268	0.595376144	0.972790461
213948	'Atg9b'	49	47	406	13	38	28	167.3333333	26.33333333	-2.851233274	0.003358412	0.132437773
213956	'Fam83f'	85	94	34	29	43	123	71	65	-0.126326731	0.840951683	0.975182082
213980	'Fbxw10'	17	14	1	1	12	9	10.66666667	7.333333333	-0.542080443	0.656713565	0.972790461
213988	'Tnrc6b'	4024	4114	2885	2254	3124	2921	3674.333333	2766.333333	-0.343269811	0.180195333	0.801341923
213989	'Tmem82'	11	12	11	13	17	15	11.33333333	15	0.457356993	0.480047117	0.972790461
21399	'Tcea1'	2408	2487	4876	1692	2499	3090	3257	2427	-0.470566376	0.336082647	0.922539021
213990	'Agap3'	1307	1323	1453	1072	1302	1395	1361	1256.333333	-0.074509902	0.812737754	0.972790461
213993	'Ccdc186'	1277	1108	2254	1598	1101	1539	1546.333333	1412.666667	-0.072757194	0.894053509	0.985171
21400	'Tcea2'	168	223	67	18	216	157	152.6666667	130.3333333	-0.280814627	0.716082831	0.972790461
21401	'Tcea3'	317	266	209	277	269	394	264	313.3333333	0.321394967	0.347959772	0.928444937
21402	'Skp1'	6751.8	7321.7	4586.9	3229.9	5386.9	8887.58	6220.11	5834.796667	-0.090611524	0.799519004	0.972790461
214048	'Larp1b'	1376	1167	524	533	657	1006	1022.333333	732	-0.410849083	0.324633813	0.91982931
21405	'Hnf1a'	6	5	5	1	4	8	5.333333333	4.333333333	-0.363360477	0.741354095	0.972790461
214058	'Megf11'	67	57	54	35	64.98	77	59.33333333	58.99333333	-0.013590353	0.972605563	0.999493374
21406	'Tcf12'	6286	6870	3781	1301	8083	6130	5645.666667	5171.333333	-0.181513337	0.748659251	0.972790461
214063	'Dnajc16'	905	900	241	581	799	863	682	747.6666667	0.239303475	0.63708153	0.972790461
21407	'Tcf15'	24	19	48	5	9	7	30.33333333	7	-2.154303233	0.005431094	0.174124727
21408	'Zfp354a'	302.55	319.67	227.67	346.59	353.69	263.55	283.2966667	321.2766667	0.306927069	0.449664507	0.97049895
214084	'Slc18a2'	352	425	50	2433	442	231	275.6666667	1035.333333	2.371903145	0.024879097	0.389943433
21410	'Hnf1b'	84	49	3	13	22	16	45.33333333	17	-1.277437437	0.193959523	0.817889904
214105	'Sox30'	468	420	91	7	176	114	326.3333333	99	-1.770598996	0.055368159	0.539548664
214106	'4933430I17Rik'	94	117	14	38	57	36	75	43.66666667	-0.629109356	0.400318861	0.955860942
21411	'Tcf20'	2910	3099	1107	1354	2344	2262	2372	1986.666667	-0.183418191	0.661426331	0.972790461
214111	'Slc24a1'	5	7	1	5	6	2	4.333333333	4.333333333	0.178235075	0.883942961	0.98358443
214112	'Nipal4'	3	3	3	4	2	3	3	3	0.11647403	0.924977727	0.992381554
21412	'Tcf21'	877	936	335	239	891	664	716	598	-0.252460932	0.627801954	0.972790461

21413	'Tcf4'	2644	2618	2179	2592	3074	2837	2480.333333	2834.333333	0.271062305	0.355876929	0.931977458
214133	'Tet2'	1435	1319	1712	503	1235	846	1488.666667	861.3333333	-0.815805536	0.039337681	0.462726104
214137	'Arhgap29'	10446	10692	8355	3965	9908	8187	9831	7353.333333	-0.424435834	0.181682784	0.802050906
21414	'Tcf7'	908.24	860.29	582.14	672.07	791.12	1173.42	783.5566667	878.87	0.218954626	0.471867032	0.972790461
21415	'Tcf7l1'	514.01	453.01	1002	476	375.31	362	656.34	404.4366667	-0.635475877	0.270992032	0.884301025
214150	'Ago3'	1033	1087	582	2130	1096	883	900.6666667	1369.666667	0.852474157	0.156223435	0.767792163
214158	'Trim38'	8	7	5	1	3	2	6.666666667	2	-1.714821803	0.131444431	0.72897159
21416	'Tcf7l2'	481	466	1020	364	525	422	655.6666667	437	-0.600417809	0.257544595	0.877405867
214162	'Kmt2a'	4697	4377	1864	4247	3718	2843	3646	3602.666667	0.166230975	0.739488119	0.972790461
21417	'Zeb1'	2230	2158.1	1936	2905.6	2141.6	1975.42	2108.036667	2340.85	0.298496507	0.505792684	0.972790461
21418	'Tfap2a'	50	49	12	5	28	23	37	18.66666667	-0.985500691	0.205520789	0.833294914
214189	'Scgn'	0	5	0	0	0	1	1.666666667	0.333333333	-2.04622596	0.60573731	0.972790461
21419	'Tfap2b'	0	0	1	4	2	0	0.333333333	2	2.767861624	0.318958133	0.916136994
214191	'Ttc24'	1	0	0	0	0	1	0.333333333	0.333333333	0.058500858	0.988561293	0.999493374
21420	'Tfap2c'	11	7	19	7	3	4	12.33333333	4.666666667	-1.311606136	0.186109056	0.807018167
21422	'Tfcp2'	851	865	510	685	844	833	742	787.3333333	0.174700312	0.574382723	0.972790461
21423	'Tcf3'	2668	2684	2550	1395	3000	3200	2634	2531.666667	-0.076913096	0.802616018	0.972790461
214230	'Pak6'	118	142	82	261	485	433	114	393	1.823610331	1.09E-08	7.94E-06
214239	'Ccdc9b'	41	54	26	90	245	151	40.33333333	162	2.031211294	1.43E-05	0.003393278
214240	'Disp2'	14	15	10	7	29	6	13	14	0.12305782	0.885737937	0.984282742
21425	'Tfeb'	215	195	145	339	237	299.97	185	291.99	0.796554008	0.068285318	0.583566634
214253	'Etnk2'	1021	1171	683	378	1477	1055	958.3333333	970	-0.005945995	0.989546702	0.999493374
214254	'Nudt15'	252	262	141	51	156	127	218.3333333	111.3333333	-0.968614275	0.024635699	0.388406457
21426	'Tfec'	5	4	1	3	10	10	3.333333333	7.666666667	1.215406741	0.262392422	0.88013169
21427	'Vps72'	941	979	1515	558	1020	1189	1145	922.3333333	-0.359844976	0.397389703	0.954563638
21428	'Mlx'	284.28	336.17	480.35	647.09	381.04	510.48	366.9333333	512.87	0.587849549	0.280501885	0.889355605
21429	'Ubtf'	2904.8	2875	1674	722	3396	3378	2484.583333	2498.666667	-0.042159705	0.935257309	0.994413066
214290	'Tut7'	1764	1812	1453	2312	1836	1617	1676.333333	1921.666667	0.342602616	0.427329061	0.961699381
214292	'Syna'	22	13	28	29	10	7	21	15.33333333	-0.237402054	0.797940491	0.972790461
214301	'Crygn'	0	1	0	0	0	0	0.333333333	0	-0.903279821	0.824807108	0.972790461
214305	'Hhipl1'	35	37	14	3	27	25	28.66666667	18.33333333	-0.686859027	0.39047342	0.952120131
21432	'Tcl1'	14	7	2	1	85	328	7.666666667	138	4.062912831	0.002017759	0.099125678
214321	'Gm4787'	11	9	1	20	15	6	7	13.66666667	1.226926231	0.250959862	0.871167957
214345	'Lrrc1'	460	489	498	692	785	919	482.3333333	798.6666667	0.774808492	0.013883444	0.292954545
214359	'Tmem51'	149	182	150	497	272	537	160.3333333	435.3333333	1.557181146	0.001255785	0.07531539
214384	'Myocd'	142	141	22	5	47	27	101.6666667	26.33333333	-1.950625188	0.023346987	0.379052873
214403	'Gm4788'	40.05	44.44	20.15	2	63.03	31.61	34.88	32.21333333	-0.192180262	0.83866287	0.974723675
214424	'Parp16'	212	183	137	139	212	196	177.3333333	182.3333333	0.093534806	0.758115565	0.972790461
214425	'Cilp'	97	124	4	44	96	36	75	58.66666667	-0.205552057	0.834110116	0.974723675
214444	'Cdk5rap2'	1152	1047	524	741	1267	1242	907.6666667	1083.333333	0.313915159	0.36951303	0.937712122

214459	'Fnbp1l'	3232	3350	3361	1944	3088	2907	3314.333333	2646.333333	-0.303813809	0.265435223	0.881878584
214469	'Fam168b'	5380	5244	5962	4164	5303	5922	5528.666667	5129.666667	-0.078621607	0.797449676	0.972790461
214489	'BC003965'	262	292	241	211	309	432	265	317.3333333	0.272622031	0.367874692	0.937190492
214498	'Cdc73'	1529	1583	1911	1075	1497	1787	1674.333333	1453	-0.199871339	0.546900157	0.972790461
214505	'Gnptg'	279.42	259.19	265.78	292.71	349.63	540.03	268.13	394.1233333	0.576062357	0.081064747	0.618950211
21452	'Tcn2'	662	680	256	1255	1038	1397	532.6666667	1230	1.352736479	0.003656767	0.139157376
214523	'Tmprss4'	1	2	0	2	1	0	1	1	0.339814551	0.890299369	0.984980969
21453	'Tcof1'	1282	1249	1237	1228	1222	1433	1256	1294.333333	0.114648261	0.726175444	0.972790461
214531	'Tmprss13'	197	253	9	4	451	308	153	254.3333333	0.693539717	0.584463049	0.972790461
21454	'Tcp1'	10094	9483.5	15279	4241.8	6987.6	7873.21	11618.91333	6367.526667	-0.900209012	0.027583172	0.406520358
214547	'She'	403	402	242	88	401	265	349	251.3333333	-0.502312511	0.315654966	0.914723408
214552	'Cep164'	1119	1068	394	1043	1252	858	860.3333333	1051	0.437680794	0.367634684	0.937190492
214568	'Gm136'	2	1	0	0	0	0	1	0	-2.286544758	0.567674219	0.972790461
214572	'Prmt7'	1129	1189	583	346	893	1304	967	847.6666667	-0.206966854	0.650653153	0.972790461
214575	'Tdrd5'	768	695	71	29	786	1253	511.3333333	689.3333333	0.371074755	0.727787127	0.972790461
214579	'Aldh5a1'	724	830	276	119	808	617	610	514.6666667	-0.278578429	0.665385789	0.972790461
214580	'Pstk'	303.69	312.06	234.48	153	294	369	283.41	272	-0.057855214	0.855718294	0.975734242
214585	'Spg11'	1623.4	1616.4	597.44	572.74	1559.1	1819.17	1279.063333	1317.006667	0.051040289	0.916480574	0.990526893
214593	'Duox2'	9	12.66	2	10	4	2	7.886666667	5.333333333	-0.214449699	0.856696096	0.976055348
214597	'Sidt2'	2430	2413	1095	2550	3766	3046	1979.333333	3120.666667	0.75274809	0.046130165	0.50074283
214601	'Slc10a3'	260.66	236.8	140.65	64.37	320.58	353.47	212.7033333	246.14	0.15644862	0.781327045	0.972790461
21461	'Tcp10a'	0	0	2.9	0	0	0	0.966666667	0	-1.995285526	0.619817042	0.972790461
214616	'Spata5l1'	354	343	263	318	293	270	320	293.6666667	-0.003078081	0.993667828	0.999493374
21462	'Tcp10b'	1	2.92	34.39	9	10	13	12.77	10.66666667	-0.345437594	0.798175306	0.972790461
214627	'Tent4b'	1130	1215	1219	1749	1168	1204	1188	1373.666667	0.350650368	0.459038106	0.972790461
21463	'Tcp11'	6	5	14	57	4	8	8.333333333	23	1.770900908	0.153486612	0.765299624
214639	'4930486L24Rik'	17	25	6	14	23	60	16	32.33333333	1.022964496	0.179908972	0.800835064
214642	'Cped1'	696	671	285	795	635	592	550.6666667	674	0.47149729	0.338680473	0.924608313
214663	'Slc25a29'	60	58	32	253	49	92	50	131.3333333	1.708214684	0.033830689	0.443127738
214669	'L3mbtl2'	626	643	271	328	669	901	513.3333333	632.6666667	0.322089042	0.452854666	0.970649024
214685	'Chadl'	113.23	111.96	39.57	13.38	91.2	134.13	88.25333333	79.57	-0.202160958	0.786366259	0.972790461
214704	'Iqub'	24	15	3	0	11	19	14	10	-0.552368586	0.669058116	0.972790461
214742	'Rcor3'	439	446	553	259	557	575	479.3333333	463.6666667	-0.08518952	0.819604041	0.972790461
214763	'Cgas'	99	124	52	30	57	41	91.66666667	42.66666667	-1.035790525	0.025884774	0.397046689
214764	'Edrf1'	1402	1397	848	782	992	1167	1215.666667	980.3333333	-0.241132008	0.401052027	0.955860942
214766	'Mmp21'	0	0	0	0	1	0	0	0.333333333	1.020273531	0.802557913	0.972790461
214779	'Zfp879'	163	168	32	16	144	104	121	88	-0.480013073	0.550335973	0.972790461
214791	'Sertad4'	256	273	754	219	213	217	427.6666667	216.3333333	-0.982812205	0.121844218	0.712603747
214804	'Syde2'	381	368	724	580	263	484	491	442.3333333	-0.057142897	0.927027895	0.992770481
214812	'Zfp609'	2789	2796	2541	2743	2411	2872	2708.666667	2675.333333	0.076503589	0.827787687	0.973238667

214854	'Neurl3'	152	147	1577	22	62	114	625.3333333	66	-3.493823508	6.16E-04	0.048420886
214855	'Arid5a'	170	154	672	180	128	119	332	142.3333333	-1.193376037	0.127812615	0.723917691
214895	'Lman2l'	1104.1	1074.3	483.26	887	1290.7	1253	887.2166667	1143.563333	0.45082307	0.221111684	0.849076011
214897	'Csnk1g1'	1667	1565	1390	1016	1416	1314	1540.666667	1248.666667	-0.251124999	0.332836646	0.921648675
214899	'Kdm5a'	6412.1	6324.6	3617.9	3071	4117.9	4292.95	5451.513333	3827.276667	-0.434262421	0.142142765	0.74797073
214901	'Chtf18'	250.84	227.61	83.98	31	202.78	209	187.4766667	147.5933333	-0.383288801	0.568267347	0.972790461
214917	'Antkmt'	328	325	207	219	383	563	286.6666667	388.3333333	0.446078016	0.204409357	0.833239858
214922	'Slc39a2'	12	8	2	27	19	8	7.333333333	18	1.550040752	0.11204462	0.69684179
214931	'Fbxl16'	153	142	202	29	156	170	165.6666667	118.3333333	-0.60627618	0.319144504	0.916136994
214932	'Hdhd5'	314	332	134	168	414	318	260	300	0.246575911	0.573384032	0.972790461
214944	'Mob3b'	253	215	121	64	225	183	196.3333333	157.3333333	-0.327861659	0.488283377	0.972790461
214951	'Rhbdl1'	47	49	25	33	63	55	40.33333333	50.33333333	0.36611811	0.414263276	0.95722888
214952	'Rhot2'	927	938	713	567	954	902	859.3333333	807.6666667	-0.055028982	0.819941208	0.972790461
214968	'Sema6d'	4062	3992	656	1929	4839	2875	2903.333333	3214.333333	0.235049594	0.718274787	0.972790461
214987	'Chtf8'	1357.6	1411	726.06	694.61	1140.6	1412.65	1164.9	1082.633333	-0.065686722	0.842882329	0.975182082
215001	'Wfikkn1'	8	8	3	38	25	9	6.333333333	24	2.176161455	0.019851315	0.349283865
215008	'Vezt'	600	598	655	479	772	584	617.6666667	611.6666667	0.015625017	0.962564166	0.999493374
215015	'Fam20b'	1719	1790	994	1186	1777	1649	1501	1537.333333	0.10504429	0.725890553	0.972790461
215031	'Vgll2'	1	4	1	0	2	0	2	0.666666667	-1.57312467	0.503507648	0.972790461
215051	'Bud13'	408	410	481	170	360	392	433	307.3333333	-0.528323583	0.146174733	0.753611197
215061	'Trim50'	79	68	38	30	171	101	61.66666667	100.6666667	0.676534195	0.265030347	0.88128484
215085	'Slc35f1'	961	991	73	54	1052	705	675	603.6666667	-0.187932267	0.851733876	0.975734242
215090	'Maneal'	18	23	4	1	17	4	15	7.333333333	-1.032540912	0.363027775	0.935009831
215095	'Astl'	0	1	0	0	0	23	0.333333333	7.666666667	4.230931362	0.166504387	0.780893009
215113	'Slc43a2'	201	202	89	56	371	262	164	229.6666667	0.446429749	0.472870901	0.972790461
215114	'Hip1'	1439.4	1407.3	943.2	3050.4	1715.2	1937	1263.276667	2234.213333	1.015552976	0.044546919	0.492112739
215160	'Rhbdd2'	729.83	743.87	350.02	246	663	469	607.9066667	459.3333333	-0.374489514	0.370128107	0.937712122
215193	'Utp25'	606	572	557	408	511	516	578.3333333	478.3333333	-0.221172719	0.44427739	0.969888068
215194	'Kri1'	638.53	587.41	984.41	566.86	618.88	624.6	736.7833333	603.4466667	-0.258137964	0.564555646	0.972790461
215201	'Trmt2b'	666	706	459	462	738	755	610.3333333	651.6666667	0.138477887	0.591988347	0.972790461
215210	'Tmem120a'	173	169	349	407	190	268	230.3333333	288.3333333	0.436947889	0.50299364	0.972790461
215243	'Traf3ip3'	3.07	4.04	2.06	0	8.23	1.01	3.056666667	3.08	-0.063520405	0.969426939	0.999493374
215257	'Il1f9'	6.96	15.99	33	4	6.27	5	18.65	5.09	-1.896634543	0.053266457	0.533562632
215274	'Il1f10'	0	0	0	0	1	0	0	0.333333333	1.020273531	0.802557913	0.972790461
215280	'Wipf1'	385	460	453	313	477	381	432.6666667	390.3333333	-0.11326138	0.734922046	0.972790461
215303	'Camk1g'	168	176	75	66	177	118	139.6666667	120.3333333	-0.177545364	0.703160742	0.972790461
215332	'Slc36a3'	0	0	1	0	0	0	0.333333333	0	-0.903279821	0.824807108	0.972790461
215335	'Slc36a1'	837	724	297	904	774	792	619.3333333	823.3333333	0.575979715	0.23447834	0.861206181
215351	'Senp6'	4463	4457	4170	1534	3959	3709	4363.333333	3067.333333	-0.53757102	0.114420235	0.700147253
215378	'Brinp3'	2	0	0	2	2	3	0.666666667	2.333333333	1.917738436	0.368759411	0.937530612

215384	'Fcgbp'	94	92	6	2	84	66	64	50.66666667	-0.375488179	0.745873945	0.972790461
215387	'Ncaph'	467	468	170	764	398	424	368.3333333	528.6666667	0.764568026	0.207921338	0.835552837
215418	'Csrnp1'	138	140	686	2217	127	371	321.3333333	905	1.748134902	0.1310252	0.728882802
215436	'Slc35e3'	546	570	324	589	742	609	480	646.6666667	0.531938322	0.125512954	0.72022881
215445	'Rab11fip3'	984	935	681	553	1150	1043	866.6666667	915.3333333	0.095535245	0.737257126	0.972790461
215446	'Entpd3'	15	11	3	20	8	8	9.666666667	12	0.58496715	0.549797124	0.972790461
215449	'Rap1b'	2653	2868	6096	2344	2985	3950	3872.333333	3093	-0.365153655	0.486712668	0.972790461
215456	'Gpat2'	2440	2160	376	10	986	1382	1658.666667	792.6666667	-1.141647747	0.319330828	0.916507972
215474	'Sec22c'	402	364	175	341	358	362	313.6666667	353.6666667	0.297469163	0.468288077	0.972790461
215476	'Prr14l'	2099	1972	1025	1610	1575	1545	1698.666667	1576.666667	0.026762246	0.946190227	0.996592403
215493	'A3galt2'	8	5	12	139	11	14	8.333333333	54.66666667	3.063312565	0.00958494	0.240725072
215494	'Pomgnt2'	260	266	79	319	462	578	201.6666667	453	1.249245557	0.009701243	0.24220996
215512	'Fam117a'	708	803	277	1291	739	902	596	977.3333333	0.929538642	0.098981704	0.668585731
215615	'Rnpep'	250	261	222	121	416	306	244.3333333	281	0.166899173	0.693095657	0.972790461
215627	'Zbtb8b'	442	395	2517	7841	302	535	1118	2892.666667	1.662700418	0.197002876	0.822696823
215632	'Psd4'	51	57	87	46	61	56	65	54.33333333	-0.246338714	0.622699601	0.972790461
215641	'Mageb18'	7	17	1	0	8	14	8.333333333	7.333333333	-0.236025796	0.871843381	0.980171118
215653	'Rassf2'	403	440	86	274	646	428	309.6666667	449.3333333	0.619609853	0.303008364	0.906189263
215654	'Cdh12'	181	189	6	0	149	114	125.3333333	87.66666667	-0.56005736	0.701120019	0.972790461
215690	'Nav1'	1214	1053	424	2122	1193	1171	897	1495.333333	0.97477185	0.099022642	0.668585731
215693	'Zmat1'	848	857	268	92	978	623	657.6666667	564.3333333	-0.264001993	0.720220217	0.972790461
215705	'Arrdc1'	315	287	353	132	303	357	318.3333333	264	-0.317410365	0.421571813	0.959296626
215707	'Ccdc92'	496	465	143	159	269	373	368	267	-0.407362297	0.420473254	0.958511083
215708	'Miga1'	769	767	397	351	953	831	644.3333333	711.6666667	0.153624167	0.698107537	0.972790461
215723	'Mfsd6l'	3	6	1	0	8	11	3.333333333	6.333333333	0.853660497	0.566782375	0.972790461
215748	'Cnksr3'	300	269	341	310	243	316	303.3333333	289.6666667	0.014336787	0.97348502	0.999493374
215751	'Ginm1'	575	592	518	1280	1056	1536	561.6666667	1290.666667	1.286656321	3.57E-04	0.034093749
215772	'Adgb'	42	24	7	1	27	28	24.33333333	18.66666667	-0.439705299	0.676721328	0.972790461
215789	'Phactr2'	981	889	245	336	949	778	705	687.6666667	0.005626036	0.991863753	0.999493374
215798	'Adgrg6'	66	68	71	45	68	45	68.33333333	52.66666667	-0.328158056	0.45397972	0.970649024
215814	'Ccdc28a'	377	338	216	58	267	189	310.3333333	171.3333333	-0.889112546	0.078483894	0.615492265
215819	'Nhsl1'	594.49	664.01	517.44	603.38	700.75	635.95	591.98	646.6933333	0.209908319	0.504377855	0.972790461
215821	'Arfgef3'	50	42	112	33	34	90	68	52.33333333	-0.460748945	0.502805612	0.972790461
215890	'Clvs2'	1	3	1	0	3	0	1.666666667	1	-0.771606349	0.742010845	0.972790461
215900	'Calhm6'	4	4	2	0	4	7	3.333333333	3.666666667	0.049341954	0.972746661	0.999493374
215928	'Mfsd4b5'	4	6.38	1	0	3	3	3.793333333	2	-0.902248687	0.565979018	0.972790461
215929	'Mfsd4b1'	9	8.08	6	2	8.01	8	7.693333333	6.003333333	-0.376135198	0.679202692	0.972790461
215951	'Afg1l'	112	121	101	173	164	145	111.3333333	160.6666667	0.638205378	0.120589869	0.71200453
215999	'Mcu'	398	376	543	384	384	347	439	371.6666667	-0.176030118	0.691203086	0.972790461
216001	'Micu1'	1397	1550	776	1680	1639	1513	1241	1610.666667	0.513968279	0.1977792	0.823747311

216011	'Lrrc20'	296	319	240	289	313	340	285	314	0.220223255	0.480274464	0.972790461
216019	'Hkdc1'	0	1	9	14	7	7	3.333333333	9.333333333	1.515628718	0.309450377	0.910300259
216021	'Stox1'	44	42	5	3	33	24	30.33333333	20	-0.612104476	0.535413523	0.972790461
216028	'Lrrtm3'	5	1	2	1	4	3	2.666666667	2.666666667	-0.013715959	0.992255779	0.999493374
216033	'Ctnna3'	33	37	8	10	71	143	26	74.66666667	1.463377975	0.09791881	0.665106257
216049	'Zfp365'	36	26	100	232	72	146	54	150	1.578415944	0.057130745	0.547413448
216080	'Ube2d1'	524	483	691	208	517	529	566	418	-0.497316953	0.240688797	0.86449675
216119	'Ybey'	137.25	133	61	38.09	108.15	103	110.4166667	83.08	-0.399787853	0.400061045	0.955860942
216131	'Trappc10'	1609	1488	754	1659	1271	1636	1283.666667	1522	0.394646951	0.364269736	0.935498612
216134	'Pdxk'	386	380	406	481	431	517	390.6666667	476.3333333	0.36436885	0.331038157	0.921648675
216136	'Ilvbl'	644	617	294	400	845	936	518.3333333	727	0.514203596	0.184173572	0.804780995
216148	'Shc2'	546	526	1054	181	633	460	708.6666667	424.6666667	-0.841754701	0.132780385	0.731080419
216150	'Cdc34'	1020	1018	1849	553	760	1331	1295.666667	881.3333333	-0.615582011	0.214069744	0.842446242
216151	'Polrmt'	688	644	319	559	664	686	550.3333333	636.3333333	0.312375355	0.39599876	0.954181778
216152	'Plppr3'	699	698	565	214	957	536	654	569	-0.241757217	0.619111827	0.972790461
216154	'Med16'	793	797.97	448	264	958	799	679.6566667	673.6666667	-0.031213017	0.944495544	0.996592403
216156	'Wdr18'	814	896	672	450	850	952	794	750.6666667	-0.074522126	0.786623242	0.972790461
216157	'Tmem259'	1231	1208	1064	2562	1505	1694	1167.666667	1920.333333	0.884551841	0.070411781	0.589465353
216161	'Sbno2'	809	826	1863	3097	852	1238	1166	1729	0.752730586	0.334021098	0.921648675
216166	'Plk5'	5	5	19	8	1	5	9.666666667	4.666666667	-0.955539988	0.438547684	0.966976432
216169	'Abhd17a'	792	868	980	1006	993	1229	880	1076	0.343343902	0.347942098	0.928444937
216177	'AU041133'	163	161	124	21	189	171	149.3333333	127	-0.328722774	0.622113567	0.972790461
216188	'Aldh1l2'	357	242	3721	802	360	913	1440	691.6666667	-1.119161429	0.286858256	0.894554788
216190	'Appl2'	1233	1139	1112	1184	1641	1042	1161.353333	1289	0.224732129	0.542097835	0.972790461
216197	'Ckap4'	1330	1230	1892	1534	1820	2164	1484	1839.333333	0.321302432	0.422924932	0.959848252
216198	'Tcp11l2'	476	472	1038	1563	391	781	662	911.6666667	0.631445968	0.410794212	0.95722888
216225	'Slc5a8'	4	6	10	2	9	3	6.666666667	4.666666667	-0.577233571	0.59775866	0.972790461
216227	'Slc17a8'	7	10	18	1	3	10	11.66666667	4.666666667	-1.458022343	0.175323149	0.79419674
216233	'Socs2'	375	391	292	123	289	315	352.6666667	242.3333333	-0.552889639	0.103850152	0.675747811
216238	'Eea1'	2739	2613	3164	2463	2362	2928	2838.666667	2584.333333	-0.078429156	0.831359239	0.974324192
216274	'Cep290'	1404.3	1711.4	361.58	429.91	900.33	599.42	1159.07	643.22	-0.757191883	0.181198406	0.801887586
216285	'Alx1'	7	5	41	28	15	10	17.66666667	17.66666667	0.066377282	0.952074481	0.998640168
216292	'Mettl25'	263	265	159	117	239	223	229	193	-0.218349823	0.509498088	0.972790461
216343	'Tph2'	29	15	1	63	27	34	15	41.33333333	1.75297513	0.084782737	0.633355364
216344	'Rab21'	949	942	1517	1845	796	1447	1136	1362.666667	0.381433983	0.521666412	0.972790461
216345	'Zfc3h1'	2689	2781	650	1900	2813	1863	2040	2192	0.241996068	0.665744616	0.972790461
216350	'Tspan8'	36	51	17	87	88	165	34.66666667	113.3333333	1.784827667	6.06E-04	0.048169622
216363	'Rab3ip'	374	362	541	667	323	562	425.6666667	517.3333333	0.39318121	0.483263586	0.972790461
216393	'D930020B18Rik'	44	33	5	13	36	101	27.33333333	50	0.855396826	0.343567129	0.925308103
216395	'Rxylt1'	787	770	827	318	692	785	794.6666667	598.3333333	-0.442181276	0.200286525	0.82759347

216438	'Marchf9'	246	282	457	313	379	555	328.3333333	415.6666667	0.31836241	0.495615296	0.972790461
216439	'Agap2'	61	79	14	109	75	47	51.33333333	77	0.84276678	0.26546353	0.881878584
216440	'Os9'	1324	1324	842	1811	1975	2031	1163.333333	1939	0.836767068	0.008467054	0.222807422
216441	'Slc26a10'	3.42	5	12.46	4.52	3.32	1	6.96	2.946666667	-1.248756053	0.323000434	0.918843465
216443	'Mars1'	1755	1766	1285	2287	1543	2225	1602	2018.333333	0.465991348	0.260694183	0.878537553
216445	'Arhgap9'	11	12	4	24	18	16	9	19.33333333	1.27802284	0.096509012	0.663111432
21645	'Tcte1'	0	0	5.41	0	0	0	1.803333333	0	-3.332616029	0.399165646	0.955184049
216454	'Rdh16f2'	0	1	0	0	1	0	0.333333333	0.333333333	0.058500858	0.988561293	0.999493374
216456	'Gls2'	26	45	263	34	17	16	111.3333333	22.33333333	-2.276330053	0.025701495	0.396299128
216459	'Myl6b'	134	132	119	207	111	198	128.3333333	172	0.551523846	0.266492211	0.88347493
21646	'Tcte2'	34	38	8	5	39	39	26.66666667	27.66666667	0.02686148	0.974898574	0.999493374
21647	'Tcte3'	305.71	252.38	238.64	445	309	197	265.5766667	317	0.444206071	0.424659499	0.960285372
21648	'Dynlt1b'	655.19	761.87	702.62	362.76	387.14	974	706.56	574.6333333	-0.31140161	0.48332636	0.972790461
216505	'Pik3ip1'	618	593	422	96	733	639	544.3333333	489.3333333	-0.236986028	0.700634621	0.972790461
216516	'Ccdc157'	1234	1119	322	196	1020	736	891.6733333	650.67	-0.458744143	0.46826516	0.972790461
21652	'Phf1'	598	528	792	731	706	1065	639.3333333	834	0.406380561	0.331500421	0.921648675
216527	'Ccm2'	587	719	665	675	612	848	657	711.6666667	0.182032649	0.614240743	0.972790461
216543	'Cep68'	1602	1651	598	372	1563	1271	1283.666667	1068.666667	-0.271464943	0.616922873	0.972790461
216549	'Aftph'	1679	1738	1997	1595	1493	1784	1804.666667	1624	-0.087045306	0.816019172	0.972790461
216551	'Lgals1'	771.96	702	1761.1	152	726	998	1078.36	625.3333333	-0.962289673	0.16672996	0.781229856
216558	'Ugp2'	2302	2404	6911	2176	3070	3982	3872.333333	3076	-0.408104027	0.50451615	0.972790461
216560	'Wdpcp'	150	152	121	73	144	159	141	125.3333333	-0.165498732	0.612610448	0.972790461
216565	'Ehbp1'	900.99	866.3	547	966.16	1057	968	771.43	997.0533333	0.481020235	0.162604457	0.774020016
216578	'Papolg'	1044	1172	584	456	971	780	933.3333333	735.6666667	-0.302102596	0.390529258	0.95213417
216613	'Ccdc85a'	41	46	94	9	75	41	60.33333333	41.66666667	-0.682816344	0.381490736	0.94429001
216616	'Efemp1'	167	151	188	56	247	305	168.6666667	202.6666667	0.160012041	0.775601124	0.972790461
216618	'Cfap36'	619.76	667.88	440.42	255.41	782.54	687.82	576.02	575.2566667	-0.020437243	0.958196871	0.999493374
216622	'Fem1al'	1	0	0	2	1	1	0.333333333	1.333333333	2.064943779	0.415102862	0.95722888
216635	'Hbq1a'	0	0	1	0	1	2	0.333333333	1	1.347412201	0.684356062	0.972790461
21664	'Phlda1'	137	145	914	521	203	304	398.6666667	342.6666667	-0.165582476	0.859244749	0.976863639
216643	'Gabrp'	2	2	0	5	1	10	1.333333333	5.333333333	2.12502898	0.185523753	0.805706284
21665	'Tdg'	1187.4	1166.9	2361.5	1882.9	1482.2	1273.01	1571.94	1546.046667	0.053096839	0.926656998	0.992540506
21667	'Tdgf1'	1	2	1	253	0	7	1.333333333	86.66666667	6.513775487	3.91E-04	0.036760231
216705	'Clint1'	2156	2387	3144	3476	2480	3489	2562.333333	3148.333333	0.37508495	0.418629064	0.958226521
21672	'Prdx2'	3157	3587	3563	2201	3924	3558	3435.666667	3227.666667	-0.083620448	0.77563634	0.972790461
216724	'Rufy1'	515	480	264	267	552	686	419.6666667	501.6666667	0.270557911	0.469621115	0.972790461
216725	'Adamts2'	1461.1	1295.8	305.38	1668	1596	1449.07	1020.76	1571.026667	0.803528078	0.173937098	0.792829561
216739	'Acsl6'	74	79	15	1	67	66	56	44.66666667	-0.39292884	0.718758113	0.972790461
216742	'Fnip1'	2476	2436	3476	2059	1814	2062	2796	1978.333333	-0.435585953	0.317938582	0.916136994
216749	'Nmur2'	20	13	0	0	14	29	11	14.33333333	0.325558591	0.847912958	0.975464187

21676	'Tead1'	2373	2498	2478	6970	2562	2660	2449.666667	4064	0.967180606	0.137344511	0.738626463
216760	'Mfap3'	1080	1031	526	1066	1300	1211	879	1192.333333	0.545933555	0.130373069	0.727945018
216766	'Gemin5'	2957	2848	801	1475	1726	1986	2202	1729	-0.226016411	0.653263302	0.972790461
216767	'Mrpl22'	242	270	247	285	326	359	253	323.3333333	0.411016761	0.192272864	0.814004755
21677	'Tead2'	1617	1645	2258	852	1856	1365	1840	1357.666667	-0.464198228	0.246780604	0.868602029
21678	'Tead3'	555	510	375	652	497	473	480	540.6666667	0.325548324	0.459585368	0.972790461
216781	'Trim58'	4	0	0	1	9	1	1.333333333	3.666666667	1.491871249	0.492424775	0.972790461
21679	'Tead4'	63	57	23	92	45	52	47.66666667	63	0.635228883	0.337712239	0.924020038
216792	'Iba57'	234	271	194	120	221	195	233	178.6666667	-0.3540056	0.232782557	0.859679981
216795	'Wnt9a'	358	323	200	70	157	190	293.6666667	139	-1.075618687	0.004928912	0.16556266
216797	'Prss38'	10	13	3	16	8	19	8.666666667	14.33333333	0.879276191	0.31015491	0.910971113
216799	'Nlrp3'	5	9	12	2	3	3	8.666666667	2.666666667	-1.709741215	0.103623516	0.675150888
216805	'Flcn'	634	633	1863	2004	1129	1313	1043.333333	1482	0.579058558	0.409163857	0.957157474
21681	'Alyref'	952	986	1030	262	789	1102	989.3333333	717.6666667	-0.541220678	0.243276735	0.865044378
216810	'Toml12'	1432	1395	1580	2335	1372	1426	1469	1711	0.375185256	0.468657144	0.972790461
21682	'Tec'	101	95	290	134	61	94	162	96.33333333	-0.673983438	0.366844654	0.936796583
216820	'Dhrs7b'	262	254	164	178	310	338.88	226.6666667	275.6266667	0.312218846	0.297291452	0.900711016
216821	'Tmem11'	377	416	1171	740	505	771	654.6666667	672	0.051940737	0.93757827	0.994698142
216825	'Usp22'	3803	3669	9616	2837	4202	4714	5696	3917.666667	-0.599663269	0.290400068	0.898321851
216829	'Mmgt2'	441.5	436	261.42	181	536.71	493.34	379.64	403.6833333	0.079230301	0.843474085	0.975182082
21683	'Tecta'	6	0	9	4	5	3	5	4	-0.323389534	0.822892779	0.972790461
216831	'Arhgap44'	374	353	168	278	235	371	298.3333333	294.6666667	0.096427662	0.822611097	0.972790461
216835	'Usp43'	88	74	567	114	45	102	243	87	-1.483626391	0.118759504	0.70823579
21684	'Tectb'	1	0	2	184	3	8	1	65	6.405264308	1.81E-04	0.02101658
216846	'Cntrob'	641	755	261	250	612	516	552.3333333	459.3333333	-0.225200498	0.621014177	0.972790461
216848	'Chd3'	5471	5224	2124	1799	6311	5068	4273	4392.666667	0.044191086	0.928337693	0.993096978
21685	'Tef'	2555	2594	1096	422	2613	2093	2081.666667	1709.333333	-0.324323945	0.587946602	0.972790461
216850	'Kdm6b'	1682	1493	4020	3732	1741	1929	2398.333333	2467.333333	0.160093122	0.821145857	0.972790461
216853	'Wrap53'	206	205	122	155	202	213	177.6666667	190	0.170256823	0.598202794	0.972790461
216856	'Nlgn2'	2030	1775	2293	1430	1969	1201	2032.666667	1533.333333	-0.348392726	0.404464034	0.956545617
216858	'Kctd11'	221	172	2353	348	218	332	915.3333333	299.3333333	-1.667419488	0.103106127	0.673510422
216859	'Acap1'	12	19	29	3	20	4	20	9	-1.236345004	0.196971431	0.822696823
216860	'Neurl4'	2193	2073	1026	641	1941	2000	1764	1527.333333	-0.214683156	0.626953721	0.972790461
216864	'Mgl2'	18	11	7	0	12	28	12	13.33333333	0.035143948	0.97712888	0.999493374
216867	'Slc16a11'	19	14	6	3	8	5	13	5.333333333	-1.232081146	0.1571872	0.768337387
216869	'Arrb2'	348	354	313	388	318	528	338.3333333	411.3333333	0.354456675	0.340924298	0.925308103
21687	'Tek'	152	165	26	3	125	84	114.3333333	70.66666667	-0.744850439	0.470372008	0.972790461
216871	'Gltpd2'	7	2	8	1	3	2	5.666666667	2	-1.548044355	0.227006979	0.854001521
216873	'Spag7'	723	805	1334	520	757	795	954	690.6666667	-0.485321505	0.279140409	0.889355605
216874	'Camta2'	667	668	433	553	688	513	589.3333333	584.6666667	0.09242582	0.794319865	0.972790461

216877	'Dhx33'	1394	1486	954	847	1484	1144	1278	1158.333333	-0.088228191	0.762441506	0.972790461
216881	'Wscd1'	49	42	7	8	62	104	32.66666667	58	0.78552367	0.394326042	0.953178011
21689	'Tekt1'	11	5	7	1	4	2	7.666666667	2.333333333	-1.719743905	0.126074136	0.72022881
216892	'Spns2'	222	226	87	95	166	166	178.3333333	142.3333333	-0.260623736	0.545809084	0.972790461
216961	'Coro6'	24	26	8	4	16	17	19.33333333	12.33333333	-0.64434281	0.405548908	0.957157474
216963	'Git1'	1902	1791	2011	2184	1677	2212	1901.333333	2024.333333	0.182031437	0.653536089	0.972790461
216964	'Trp53i13'	174	209	179	118	224	224	187.3333333	188.6666667	0.014259602	0.963988548	0.999493374
216965	'Taok1'	4042	4052	3415	2077	3529	3350	3836.333333	2985.333333	-0.336049222	0.150954679	0.759755159
216971	'Fam222b'	591	577	541	417	513	517	569.6666667	482.3333333	-0.183132427	0.525974948	0.972790461
216974	'Proca1'	51	56	21	23	56	67	42.66666667	48.66666667	0.20742204	0.702419742	0.972790461
216976	'Rskr'	17	28	7	0	13	3	17.33333333	5.333333333	-1.736667334	0.156106567	0.767625991
216984	'Evi2b'	10	10	0	2	12	12	6.666666667	8.666666667	0.395648573	0.764895649	0.972790461
216987	'Utp6'	1348	1407	965	738	1507	1516	1240	1253.666667	0.028445661	0.919729577	0.990870897
216991	'Adap2'	27	25	11	39	21	41	21	33.66666667	0.832682205	0.201140052	0.828798149
217011	'Nle1'	189	182	308	151	195	243	226.3333333	196.3333333	-0.217808904	0.629688507	0.972790461
217012	'Unc45b'	151	137	25	89	244	225	104.3333333	186	0.883259864	0.177322097	0.79704932
217026	'Heatr6'	602	671	502	302	654	500	591.6666667	485.3333333	-0.268910207	0.380755368	0.944042914
217030	'Synrg'	1375.7	1381.2	1044.6	603.86	1423.7	1508.81	1267.16	1178.783333	-0.116967921	0.717113658	0.972790461
217031	'Tada2a'	485	499	307	155	430	289	430.3333333	291.3333333	-0.5498572	0.154649001	0.766453805
217038	'Mrm1'	266	239	454	107	317	341	319.6666667	255	-0.427869529	0.422546398	0.959735331
217039	'Ggnbp2'	2024	2028	2296	1335	1805	1946	2116	1695.333333	-0.296296304	0.33255254	0.921648675
217057	'Ptrh2'	457	455	865	171	397	380	592.3333333	316	-0.981767883	0.050294766	0.518969204
217066	'Elob1'	2	2	0	0	0	2	1.333333333	0.666666667	-0.99885442	0.740500622	0.972790461
217069	'Trim25'	1589	1633	1726	2344	1639	2650	1649.333333	2211	0.509695537	0.222492331	0.84974371
217071	'Gm525'	0	1	0	0	0	0	0.333333333	0	-0.903279821	0.824807108	0.972790461
217082	'Hlf'	176	154	55	2240	144	415	128.3333333	933	3.298208638	0.001244102	0.074850865
217109	'Utp18'	955	941	758	431	563	866	884.6666667	620	-0.48939197	0.099019878	0.668585731
217116	'Spata20'	4	4	13	4	4	5	7	4.333333333	-0.717471794	0.50830478	0.972790461
217119	'Xylt2'	720	601	373	421	713	639	564.6666667	591	0.122007005	0.702660463	0.972790461
217122	'Gm11545'	0	1	2	7	0	2	1	3	1.831567444	0.400249702	0.955860942
217124	'Ppp1r9b'	2618	2667	1343	5062	3379	3789	2209.333333	4076.666667	1.066295242	0.025670071	0.396135865
217125	'Samd14'	667	659	1076	172	754	566	800.6666667	497.3333333	-0.79452041	0.150798018	0.759755159
217127	'Kat7'	1874	1686	2131	1126	1686	2107	1897	1639.666667	-0.217258474	0.512530457	0.972790461
217138	'Prr15l'	8	9	0	29	7	25	5.666666667	20.33333333	2.105113638	0.077196337	0.611268953
217140	'Scrn2'	294	366	117	124	428	415	259	322.3333333	0.317771937	0.552947031	0.972790461
217143	'Gpr179'	127	160	9	14	88	83	98.66666667	61.66666667	-0.669662961	0.478831715	0.972790461
217149	'Cisd3'	40	65	176	79	65	55	93.66666667	66.33333333	-0.468330971	0.536907502	0.972790461
217151	'Arl5c'	97	102	440	28	59	122	213	69.66666667	-1.7966354	0.028071113	0.409247928
217154	'Stac2'	190	162	798	382	149	116	383.3333333	215.6666667	-0.69946028	0.442642503	0.96952987
217166	'Nr1d1'	110.49	98.56	422.58	771.59	113.54	346.55	210.5433333	410.56	1.119672255	0.24289067	0.864948315

217169	'Tns4'	74	80	4	78	87	138	52.66666667	101	1.07132035	0.216445612	0.843391934
217194	'Klhl11'	445	496	105	290	374	423	348.6666667	362.3333333	0.173349107	0.756615418	0.972790461
217198	'Plekhh3'	434	529	503	2258	737	832	488.6666667	1275.666667	1.630073708	0.016651812	0.323882143
217201	'Rundc1'	645.03	603.15	651.65	662.58	724.14	714.21	633.2766667	700.31	0.212338261	0.532571361	0.972790461
217203	'Tmem106a'	52	49	31	11	70	77	44	52.66666667	0.191587776	0.772611233	0.972790461
217207	'Dhx8'	1340	1364	1130	1049	1180	1369	1278	1199.333333	-0.025844703	0.925529929	0.992381554
217212	'Pyy'	0	1	9	0	0	1	3.333333333	0.333333333	-3.339393485	0.220690871	0.849076011
217214	'Nags'	15	13	726	36	10	48	251.3333333	31.33333333	-3.104232198	0.024416235	0.386512464
217216	'Hrob'	321	317	140	10	161	238	259.3333333	136.3333333	-1.023186882	0.22471685	0.850759465
217217	'Asb16'	6	3	65	0	5	7	24.66666667	4	-2.931377115	0.049180048	0.515210762
217218	'Atxn7l3'	2233.6	2193.8	1822.5	1732.7	2448.8	2447.98	2083.283333	2209.816667	0.132605943	0.573000779	0.972790461
217219	'Fam171a2'	500	570	180	386	802	554	416.6666667	580.6666667	0.555378274	0.242988447	0.864948315
217232	'Cdc27'	2087	2254	1701	1532	1997	1863	2013.996667	1797.326667	-0.096096924	0.718673173	0.972790461
217258	'Abca8a'	101	87	12	18	87	129	66.66666667	78	0.214748031	0.798773797	0.972790461
217262	'Abca9'	126	180	2	7	259	152	102.6666667	139.3333333	0.418417614	0.743148176	0.972790461
217265	'Abca5'	856	845	458	716	735	657	719.6666667	702.6666667	0.095772487	0.805837808	0.972790461
217302	'Gpr142'	1	0	0	0	0	0	0.333333333	0	-0.903279821	0.824807108	0.972790461
217303	'Cd300a'	41	34	16	50	38	112	30.33333333	66.66666667	1.198684374	0.047823341	0.50765905
217304	'Cd300lb'	3	7	0	46	14	20	3.333333333	26.66666667	3.319547758	0.004197338	0.149966291
217305	'Cd300ld'	0	2	0	10	6	7	0.666666667	7.666666667	3.720102896	0.023538824	0.380103384
217306	'Cd300e'	1	1	0	0	0	0	0.666666667	0	-1.703500596	0.673820112	0.972790461
217310	'Hid1'	131	104	552	580	127	379	262.3333333	362	0.550342202	0.550612184	0.972790461
217316	'Slc16a5'	90	79	4	26	89	132	57.66666667	82.33333333	0.536074841	0.571052347	0.972790461
217325	'Llgl2'	182	184	198	229	168	249	188	215.3333333	0.281708991	0.511568688	0.972790461
217328	'Myo15b'	27	24	7	6	49	53	19.33333333	36	0.854017466	0.311685348	0.911537035
217331	'Unk'	1130	1029	1151.2	829	1044	1014	1103.386667	962.3333333	-0.150864597	0.630955644	0.972790461
217333	'Trim47'	109	125	1150	922	146	418	461.3333333	495.3333333	0.202736457	0.855027373	0.975734242
217335	'Fbf1'	1485	1512	665	767	1620	1179	1220.666667	1188.666667	0.019448913	0.960920782	0.999493374
217337	'Srp68'	1650	1663	4596	1554	1658	2247	2636.333333	1819.666667	-0.571902854	0.342089957	0.925308103
217340	'Rnf157'	367	356	312	405	310	258	345	324.3333333	0.060460851	0.896816913	0.985793429
217341	'Qrich2'	1	0	0	1	1	0	0.333333333	0.666666667	1.081626198	0.75986402	0.972790461
217342	'Ube2o'	1606	1496	1790	2100	1362	1844	1630.666667	1768.666667	0.228386425	0.619233561	0.972790461
217344	'Rhbdf2'	471	421	1130	2731	640	1201	674	1524	1.351389373	0.09557982	0.662411648
217351	'Tnrc6c'	2002	2002	1079	713	1839	1516	1694.333333	1356	-0.304912917	0.413331711	0.95722888
217353	'Tmc6'	460	510	264	488	811	639	411.3333333	646	0.722015078	0.032296425	0.434162511
217356	'Tmc8'	74	69	34	55	67	78	59	66.66666667	0.259453255	0.548603751	0.972790461
217364	'Engase'	135	135	87	123	192	241	119	185.3333333	0.671816056	0.037139696	0.459930654
217365	'Nploc4'	2797	2852	1603	2199	2128	2889	2417.333333	2405.333333	0.09228989	0.789850624	0.972790461
217366	'Lrrc45'	571	560	364	548	845	592	498.3333333	661.6666667	0.487380756	0.144193279	0.750780289
217369	'Uts2r'	0	4	1	0	1	1	1.666666667	0.666666667	-1.342712826	0.587778012	0.972790461

217370	'Cybc1'	492	502	416	513	684	663	470	620	0.456174747	0.085727796	0.63542178
217371	'Rab40b'	53	52	44	84	91	51	49.66666667	75.33333333	0.722640749	0.160665659	0.771114735
217378	'Dnajc27'	377	374	225	118	281	248	325.3333333	215.6666667	-0.577247416	0.102427927	0.671776916
217379	'Ubxn2a'	819	841	886	284	764	950	848.6666667	666	-0.410494944	0.320476561	0.917329173
217410	'Trib2'	2065	2199	1253	1354	3548	2988	1839	2630	0.524366775	0.145342393	0.753611197
21743	'Inmt'	0	2	0	0	1	1	0.666666667	0.666666667	0.021482588	0.994987788	0.999562152
217430	'Pqlc3'	58	54	57	89	89	207	56.33333333	128.3333333	1.190516464	0.01143811	0.264873743
217431	'Nol10'	542	569	369	345	518	522	493.3333333	461.6666667	-0.042487503	0.872630517	0.980171118
21744	'Adad1'	519	556	151	3	219	236	408.6666667	152.6666667	-1.505085174	0.149732726	0.758922578
217449	'Trappc12'	1023	1017	469	353	951	1083	836.3333333	795.6666667	-0.071403226	0.870600663	0.979986751
21745	'Tep1'	1011	1045	456	140	974	1007	837.3333333	707	-0.302833672	0.635933979	0.972790461
217463	'Snx13'	1199	1116	876	2123	1425	1451	1063.666667	1666.333333	0.812114378	0.078603788	0.615492265
217473	'Ankmy2'	652	733	584	116	947	847	656.3333333	636.6666667	-0.144454439	0.819029053	0.972790461
217480	'Dgkb'	23	24	7	0	16	8	18	8	-1.223718099	0.288481609	0.895739401
21749	'Terf1'	543	549	406	221	374	529	499.3333333	374.6666667	-0.40929797	0.192098182	0.813837363
21750	'Terf2'	1037	1082	1069	739	970	899	1062.666667	869.3333333	-0.240851357	0.419314264	0.958308933
217517	'Stxbp6'	712	713	1715	352	863	755	1046.666667	656.6666667	-0.768407365	0.177779618	0.797422443
21752	'Tert'	433	386	155	652	458	322	324.6666667	477.3333333	0.783128779	0.18409474	0.804780995
21753	'Tes'	481	477	3694	979	515	801	1550.666667	765	-1.028516663	0.268874966	0.883851074
21754	'Tesk1'	1041.8	1058.7	758.78	837.78	1160.9	1255.82	953.0766667	1084.833333	0.239684084	0.321726731	0.917918531
21755	'Prss39'	0	0	0	1	0	0	0	0.333333333	1.020273531	0.802557913	0.972790461
217558	'G2e3'	1001	921	2342	375	702	461	1421.333333	512.6666667	-1.517844215	0.008324666	0.22166463
21756	'Prss40'	0	0	0	0	0	1	0	0.333333333	1.020273531	0.802557913	0.972790461
217578	'Baz1a'	1177	1171	734	780	822	769	1027.333333	790.3333333	-0.264962574	0.446966252	0.970198456
217588	'Mbip'	465	538	267	118	453	390	423.3333333	320.3333333	-0.422966337	0.375386982	0.940406075
217593	'Slc25a21'	48	48	27	5	50	46	41	33.66666667	-0.355667039	0.634529811	0.972790461
21761	'Morf4l1'	6746.5	6879.2	11916	11047	7984.1	10963.22	8513.88	9998.266667	0.290737271	0.571984932	0.972790461
21762	'Psmd2'	8165	8495	5006	5249	6271	8989	7222	6836.333333	-0.018243062	0.952470911	0.998773884
21763	'Tex2'	1172	1104	559	1258	842	1322	945	1140.666667	0.420828175	0.356239373	0.931977458
217648	'Gm527'	147.95	150	221.95	78	133.94	177	173.3	129.6466667	-0.45863666	0.299364125	0.903198439
217653	'Mis18bp1'	543	506	157	56	355	216	402	209	-0.956358034	0.146645744	0.753611197
21766	'Tex261'	839	784	633	739	1086	1199	752	1008	0.460746325	0.051927718	0.529072762
217664	'Mgat2'	1150	1034	1326	625	1164	1709	1170	1166	-0.051854953	0.894714441	0.985419473
217666	'L2hgdh'	612	687	452	376	545	527	583.6666667	482.6666667	-0.215911758	0.416305494	0.95722888
21767	'Tex264'	912	842	890	362	818	1380	881.3333333	853.3333333	-0.109235882	0.803348334	0.972790461
217674	'Gphb5'	1	0	0	0	0	0	0.333333333	0	-0.903279821	0.824807108	0.972790461
217682	'Plekhd1'	51	53	3	10	34	28	35.66666667	24	-0.513825434	0.58711842	0.972790461
217684	'Susd6'	1478	1493	1125	4452	1693	2372	1365.333333	2839	1.2819238	0.030751983	0.425931054
21769	'Zfand3'	1761	1720	2720	2759	1713	2805	2067	2425.666667	0.304573827	0.552535933	0.972790461
217692	'Sipa1l1'	2008	1772	1543	1113	1346	1673	1774.333333	1377.333333	-0.314576195	0.234639107	0.861206181

217695	'Zfyve1'	1155.1	1222	1580	1344	1306	1434.01	1319.023333	1361.336667	0.102526584	0.799944862	0.972790461
217698	'Acot5'	0	0	2	1	0	1	0.666666667	0.666666667	-0.028559312	0.993076972	0.999493374
21770	'Ppp2r5d'	1511	1481	1008	825	1483	1544	1333.333333	1284	-0.026932814	0.918398252	0.990727847
217700	'Acot6'	130	143	111.29	16	152.74	132.04	128.0966667	100.26	-0.454631814	0.502890928	0.972790461
217705	'Fam161b'	157	158	48	83	139	103	121	108.3333333	-0.058695236	0.909583793	0.989364653
217707	'Coq6'	359	383	295	261	318	368	345.6666667	315.6666667	-0.073676951	0.789350394	0.972790461
217708	'Lin52'	188	170	205	200	172	181	187.6666667	184.3333333	0.063027643	0.883376166	0.983244267
21771	'Utp4'	1370	1457	1601	1199	1049	1846	1476	1364.666667	-0.073715417	0.848811443	0.975686757
217715	'Eif2b2'	709	715	688	620	721	821	704	720.6666667	0.085678719	0.769196497	0.972790461
217716	'Mlh3'	1280	1298	350	173	1103	1220	976	832	-0.265504943	0.701182563	0.972790461
217718	'Nek9'	4270	4335	2266	3017	3934	3958	3623.666667	3636.333333	0.092789782	0.773930986	0.972790461
217721	'Flvcr2'	69	77	215	200	168	103	120.3333333	157	0.444968596	0.539070367	0.972790461
217732	'Cipc'	1125	1196	541	549	1017	915	954	827	-0.149249855	0.679678149	0.972790461
217733	'Tmem63c'	10	20	3	5	19	9	11	11	0.051714796	0.956278892	0.999493374
217734	'Pomt2'	937.38	853.14	298.79	528.7	984.11	928.65	696.4366667	813.82	0.293415918	0.518610194	0.972790461
217737	'Ahsa1'	1642	1825	1218	1430	2160	2207	1561.666667	1932.333333	0.35561642	0.141804837	0.747136019
217738	'Ism2'	28	16	6	0	30	30	16.66666667	20	0.179822093	0.884705712	0.98386821
217779	'Lysmd1'	339	371	455	513	339	423	388.3333333	425	0.237735622	0.62498305	0.972790461
21778	'Tex9'	652.56	690.53	101	54	804.38	437.73	481.3633333	432.0366667	-0.182327796	0.836828253	0.974723675
21780	'Tfam'	1031	1089	1187	701	1093	1606	1102.333333	1133.333333	0.020363895	0.952878727	0.999032683
21781	'Tfdp1'	1599	1730	1229	878	1569	1588	1519.333333	1345	-0.15133669	0.541297138	0.972790461
217826	'Kcnk13'	3	5	0	1	9	9	2.666666667	6.333333333	1.240866471	0.397612245	0.954563638
217827	'Nrde2'	687	708	388	241	641	627	594.3333333	503	-0.239374562	0.535234154	0.972790461
217830	'Dglucy'	88	89	56	12	145	161	77.66666667	106	0.348887948	0.647556256	0.972790461
217835	'Rin3'	344	345	304	118	359	292	331	256.3333333	-0.398605063	0.305836144	0.907188578
217837	'Itpk1'	338	313	1244	431	247	818	631.6666667	498.6666667	-0.40915151	0.596110796	0.972790461
21784	'Tff1'	0	0	2	0	0	0	0.666666667	0	-1.995285526	0.619817042	0.972790461
217843	'Unc79'	70.72	94.03	15.45	0	144.13	43	60.06666667	62.37666667	0.010635768	0.993645516	0.999493374
217845	'Ifi27l2b'	0	0	3	11	0	29	1	13.33333333	3.596533256	0.093933719	0.657941197
217847	'Serpina10'	1	0	0	2	0	0	0.333333333	0.666666667	1.337350669	0.73871782	0.972790461
217864	'Rcor1'	2577	2570	3809	2625	2153	2320	2985.333333	2366	-0.259471646	0.579123706	0.972790461
217866	'Cdc42bpb'	3739	3594.5	2425	5732	4335	4502	3252.826667	4856.333333	0.731426203	0.083556517	0.627659848
217869	'Eif5'	8797	8421	10071	4403	5669	7642	9096.333333	5904.666667	-0.615374765	0.059494293	0.553378427
21787	'Tfg'	1779	1829	3023	1456	1941	2530	2210.333333	1975.666667	-0.18471896	0.672033787	0.972790461
21788	'Tfpi'	714	803	655	907	1011	948	724	955.3333333	0.483325413	0.130080164	0.727620313
217882	'Cep170b'	3089	2936	3549	2812	2725	3312	3191.333333	2949.666667	-0.056039396	0.878349328	0.981341203
217887	'Clba1'	134	157	256	365	155	273	182.3333333	264.3333333	0.652994174	0.302026295	0.904841461
21789	'Tfpi2'	21	17	45	118	34	19	27.66666667	57	1.281605304	0.184390175	0.804780995
217893	'Pacs2'	2673	2327	2111	2064	1981	2725	2370.333333	2256.666667	0.001399356	0.996499022	0.999616639
217935	'Wdr60'	670	694	212	230	762	508	525.3333333	500	-0.039952352	0.941002815	0.996040262

217944 'Rapgef5'	856	808	217	958	639	517	627	704.6666667	0.409924307	0.51925279	0.972790461
217946 'Cdca7l'	390.18	394.45	221.75	65.79	303.7	420.05	335.46	263.18	-0.413861497	0.472983089	0.972790461
217951 'Tmem196'	71	72	17	9	65	65	53.33333333	46.33333333	-0.229983294	0.769448502	0.972790461
217980 'Larp4b'	2066	2022	1722	1635.2	1742	2229	1936.666667	1868.73	0.012714387	0.964568259	0.999493374
217995 'Heatr1'	1776	1824	828	852	1354	1284	1476	1163.333333	-0.270344867	0.447305704	0.970198456
21802 'Tgfa'	547	633	449	582	834	1074	543	830	0.643113399	0.015932854	0.314541149
21803 'Tgfb1'	321	281	816	775	393	692	472.6666667	620	0.444334921	0.514262571	0.972790461
218030 'Pou6f2'	1	0	0	0	2	2	0.333333333	1.333333333	1.768945179	0.572584055	0.972790461
218035 'Vps41'	2543	2600	1340	647	2302	2652	2161	1867	-0.242463757	0.614103907	0.972790461
218038 'Amph'	296	283	117	317	465	205	232	329	0.643926466	0.225396914	0.852446017
21804 'Tgfb1i1'	1272	1350	1490	37	1883	1358	1370.666667	1092.666667	-0.486558797	0.608342846	0.972790461
21807 'Tsc22d1'	14138	15244	17895	7615	19976	18153	15759	15248	-0.097135341	0.802577109	0.972790461
21808 'Tgfb2'	728	650	535	425	715	364	637.6666667	501.3333333	-0.273300711	0.496568272	0.972790461
21809 'Tgfb3'	809	728	752	390	934	1147	763	823.6666667	0.067770559	0.855597697	0.975734242
21810 'Tgfbi'	2919	2687	2805	760	2334	1763	2803.666667	1619	-0.830996107	0.033633312	0.442516625
218100 'Zfp322a'	1609	1628	980	538	1277	1171	1405.666667	995.3333333	-0.486340763	0.14698088	0.753611197
218103 'Slc17a2'	3	1	0	0	0	0	1.333333333	0	-2.70051453	0.497202575	0.972790461
21812 'Tgfbr1'	3190	3354	3267	2679	3983	4096	3270.333333	3586	0.157465817	0.547794588	0.972790461
218121 'Mboat1'	50	54	100	120	66	96	68	94	0.554817326	0.380059937	0.943549167
21813 'Tgfbr2'	638	559	440	641	1249	1243	545.6666667	1044.333333	0.951511339	6.16E-04	0.048420886
218138 'Gmds'	280	278	320	97	244	509	292.6666667	283.3333333	-0.141313735	0.793877198	0.972790461
21814 'Tgfbr3'	806	731	570	3261	719	1046	702.3333333	1675.333333	1.558808693	0.03767156	0.459930654
21815 'Tgif1'	928	950	2214	918	951	1296	1364	1055	-0.390667379	0.482586579	0.972790461
21816 'Tgm1'	10	15	397	131	8	34	140.6666667	57.66666667	-1.140710252	0.429251585	0.962386546
218165 'Ofcc1'	1	0	0	2	1	0	0.333333333	1	1.757540602	0.586010649	0.972790461
21817 'Tgm2'	1965	1815	5165	3997	1156	2013	2981.666667	2388.666667	-0.175967132	0.822171801	0.972790461
21818 'Tgm3'	7	11	6	5	10	10	8	8.333333333	0.083881474	0.917065425	0.990536123
21819 'Tg'	59	37	13	27	28	19	36.33333333	24.66666667	-0.391744789	0.574676348	0.972790461
218194 'Phactr1'	120	114	248	106	59.42	52	160.6666667	72.47333333	-1.031024508	0.133876348	0.733301819
218203 'Mylip'	697	700	340	847	567	617	579	677	0.41171435	0.403003993	0.955996639
21821 'Ift88'	1245	1179	456	170	803	859	960	610.6666667	-0.682915526	0.24200735	0.86449675
218210 'Nup153'	3375	3354	1931	3178	2545	2581	2886.666667	2768	0.094682509	0.822897479	0.972790461
218214 'Kdm1b'	1038.3	964.37	392.65	619.72	1767.1	5766.8	798.4333333	2717.86	1.704428658	0.015475722	0.309500711
218215 'Rnf144b'	599	583	207	101	882	939	463	640.6666667	0.409998083	0.566725546	0.972790461
21822 'Tgtp1'	28.88	19.03	68.49	35	25.83	88.87	38.8	49.9	0.284345373	0.71798633	0.972790461
21823 'Th'	3	4	0	0	32	127	2.333333333	53	4.41793111	0.007583525	0.209755373
218232 'Ptpdc1'	868	806	385	363	664	453	686.3333333	493.3333333	-0.398956344	0.315785706	0.914723408
218236 'Fam120a'	5645	5383	4908	4210	5120	5707	5312	5012.333333	-0.030002482	0.908742079	0.988896137
21824 'Thbd'	247	224	351	714	286	177	274	392.3333333	0.747077906	0.317896065	0.916136994
21825 'Thbs1'	2835	2684	1162	17742	5378	5400	2227	9506.666667	2.418940852	0.001012146	0.066185633

21826	'Thbs2'	1946	2154	887	2980	3731	2122	1662.333333	2944.333333	0.971852688	0.040950001	0.474812891
218268	'Eif4e1b'	0	1	0	2	0	6	0.333333333	2.666666667	2.905406091	0.286171636	0.894057448
21827	'Thbs3'	434	453	110	80	374	125	332.3333333	193	-0.745471646	0.27963594	0.889355605
218271	'B4galt7'	373	344	295	210	402	424.99	337.3333333	345.6633333	0.040357899	0.887258443	0.984534762
21828	'Thbs4'	31	40	18	129	20	81	29.66666667	76.66666667	1.6330523	0.049368998	0.515210762
218294	'Cdc14b'	1604	1561	1181	1508	2172	1785	1448.676667	1821.676667	0.397587545	0.153113591	0.764400413
218304	'Prss47'	0	1	0	0	0	1	0.333333333	0.333333333	0.058500858	0.988561293	0.999493374
218311	'Zfp455'	53	66.01	45	37	77	93	54.67	69	0.334248062	0.419367469	0.958308933
218314	'Zfp595'	135.61	124.45	74	95	163.48	148	111.3533333	135.4933333	0.337732981	0.333566439	0.921648675
21832	'Thpo'	93	88	27	12	42	19	69.33333333	24.33333333	-1.460245415	0.028043933	0.40918746
21833	'Thra'	2175.5	2165.4	2456.4	1757.4	2554.5	3036.45	2265.79	2449.44	0.118228494	0.697428218	0.972790461
218333	'Ice1'	2927	2865	2155	2752	2976	2440	2649	2722.666667	0.14900305	0.67057386	0.972790461
218335	'Clptm1l'	1694	1624	1500	1365	2141	3112	1606	2206	0.454403953	0.133113365	0.732277571
21834	'Thrb'	37	38	26	204	30	99	33.66666667	111	2.000383275	0.01553982	0.309500711
218341	'Rfesd'	296	344	193	145	257	340	277.6666667	247.3333333	-0.147724092	0.668942398	0.972790461
218343	'Ttc37'	951	899	1016	756	810	965	955.3333333	843.6666667	-0.129534839	0.697930207	0.972790461
21835	'Thrsp'	26	24	22	10	19	10	24	13	-0.841054766	0.169557273	0.785100556
21838	'Thy1'	4	4	3	54	9	10	3.666666667	24.33333333	3.073978793	0.006355458	0.189092275
218397	'Rasa1'	3788	4005	1799	1024	3103.2	2953	3197.333333	2360.056667	-0.437598586	0.32075163	0.917442833
21841	'Tia1'	2419	2659	1023	1062	3667	1344	2033.666667	2024.333333	0.036171367	0.948157621	0.997364872
21843	'Tial1'	2495	2525	2158	1930	2444	1649	2392.666667	2007.666667	-0.163026087	0.651184455	0.972790461
21844	'Tiam1'	766	724	275	1459	805	982	588.3333333	1082	1.100073361	0.053864555	0.534031734
218440	'Ankrd34b'	69	67	43	1	13	11	59.66666667	8.333333333	-2.883323311	3.97E-05	0.007195467
218441	'Zfyve16'	1232	1240	879	816	1262	1137	1117	1071.666667	-0.006067653	0.980912852	0.999493374
218442	'Serinc5'	2073	2018	745	2289	2490	2468	1612	2415.666667	0.718148351	0.108711523	0.687337371
218454	'Lhfpl2'	1737	1811	1251	1878	2068	2892	1599.666667	2279.333333	0.574781984	0.046316478	0.501177506
21846	'Tie1'	249	279	45	0	569	411	191	326.6666667	0.706843765	0.617433834	0.972790461
218460	'Wdr41'	777.58	865.53	393.39	137	922.4	642.95	678.8333333	567.45	-0.303128834	0.615315888	0.972790461
218461	'Pde8b'	397.42	474.47	104.61	6	636.6	617.05	325.5	419.8833333	0.289760568	0.793474793	0.972790461
21847	'Klf10'	1238	1162	3094	674	655	896	1831.333333	741.6666667	-1.322688943	0.024541167	0.387844278
218476	'Gcnt4'	51	59	83	310	130	197	64.33333333	212.3333333	1.871769311	0.003202974	0.12819988
21848	'Trim24'	2348	2425	1686	2249	2776	2316	2153	2447	0.276651589	0.375038168	0.940406075
21849	'Trim28'	6893	6956.4	5565	3987	7061.7	6699.76	6471.446667	5916.146667	-0.105401525	0.653242219	0.972790461
218490	'Btf3'	4381	4663	9827	4212	3916	5138	6290.333333	4422	-0.502139901	0.35144737	0.929144534
218503	'Fcho2'	1938	2038	1702	1290	2244	1766	1892.666667	1766.666667	-0.063975657	0.814040655	0.972790461
218506	'Mrps27'	730	794	583	457	681	737	702.3333333	625	-0.128696037	0.592249478	0.972790461
218518	'Marveld2'	80	68	27	25	190	302	58.33333333	172.3333333	1.491821556	0.051921108	0.529072762
21853	'Timeless'	747	814	207	144	706	679	589.3333333	509.6666667	-0.22164314	0.729326061	0.972790461
21854	'Timm17a'	459	481	849	440	512	747	596.3333333	566.3333333	-0.094916714	0.841724806	0.975182082
218543	'Srek1'	2732	2956	1633	951	3654	1723	2440.333333	2109.333333	-0.208157732	0.668559101	0.972790461

218544	'Sgtb'	264	265	538	735	191	231	355.6666667	385.6666667	0.334075879	0.677378542	0.972790461
21855	'Timm17b'	566	625	659	312	490	581	616.6666667	461	-0.420811394	0.178570338	0.798255175
21856	'Timm44'	1106	1076	1299	598	1071	1071	1160.333333	913.3333333	-0.356079189	0.276428966	0.888034012
21857	'Timp1'	315	367	2553	706	317	442	1078.333333	488.3333333	-1.113377698	0.239841946	0.86449675
21858	'Timp2'	1872	1704	805	5785	3101	3740	1460.333333	4208.666667	1.744387132	0.001458516	0.082561154
218581	'Depdc1b'	118	101	11	6	69	91	76.66666667	55.33333333	-0.506018744	0.606481729	0.972790461
21859	'Timp3'	2826	2707	1629	8545	3922	5580	2387.333333	6015.666667	1.543981218	0.004557906	0.158708616
218613	'Mier3'	1032	1099	1016	4447	1393	1598	1049	2479.333333	1.498210847	0.028029514	0.40918746
218624	'Il31ra'	4	3	1	22	15	6	2.666666667	14.33333333	2.665578106	0.01495419	0.305110509
218629	'Dhx29'	951	930	323	500	966	1014	734.6666667	826.6666667	0.225674417	0.615334864	0.972790461
218630	'Ccno'	51	37	25	5	214	933	37.66666667	384	3.202150801	0.005298715	0.172483979
218639	'Arl15'	313	307.99	435	269.86	337	375	351.9966667	327.2866667	-0.090460719	0.817143922	0.972790461
21869	'Nkx2-1'	0	2	0	0	0	0	0.666666667	0	-1.695595436	0.675304306	0.972790461
218693	'Paip1'	2158	2231	2608	908	2155	2449	2332.333333	1837.333333	-0.396000026	0.301580641	0.90469437
218695	'Gm10044'	23	15	6	2	27	10	14.66666667	13	-0.200409064	0.839732752	0.974723675
218699	'Pxk'	554	451	299	1647	530	523	434.6666667	900	1.346897134	0.056618392	0.547413448
21871	'Atp6v0a2'	839	878	376	1136	1146	1098	697.6666667	1126.666667	0.83076956	0.04978989	0.517270701
21872	'Tjp1'	6214	5888	4204	3330	5588	5551	5435.333333	4823	-0.135134514	0.576680586	0.972790461
21873	'Tjp2'	706	767	703	599	945	925	725.3333333	823	0.206646227	0.430927129	0.963403879
218734	'3830406C13Rik'	753.22	693.48	233.05	63.28	645.64	459.07	559.9166667	389.33	-0.571187068	0.43463109	0.965354095
218756	'Slc4a7'	1158	1091	917	2856	1151	1119	1055.333333	1708.666667	0.939428799	0.13546879	0.736012522
218763	'Lrrc3b'	106	150	84	5	140	90	113.3333333	78.33333333	-0.636524061	0.464684647	0.972790461
21877	'Tk1'	211.78	203.95	47	113	245.93	163	154.2433333	173.9766667	0.264010599	0.652535898	0.972790461
218772	'Rarb'	54	65	314	213	18	48	144.3333333	93	-0.416782682	0.71216578	0.972790461
218793	'Ube2e2'	488	479	1389	196	371	435	785.3333333	334	-1.334991561	0.030494317	0.424731104
21881	'Tkt'	4939	5018	5997	3358	9804	12502	5318	8554.666667	0.605092557	0.180142217	0.801341923
218811	'Sec24c'	2965	2879	3815	2649	2792	3310	3219.666667	2917	-0.103269355	0.7840987	0.972790461
218820	'Zfp503'	1842	1904	1398	546	2584	1748	1714.666667	1626	-0.125294538	0.797364031	0.972790461
218832	'Polr3a'	717	775	334	451	683	716	608.6666667	616.6666667	0.091635564	0.802410763	0.972790461
21885	'Tle1'	1945	1928	2253	1083	2183	2639	2042	1968.333333	-0.090247337	0.798663031	0.972790461
218850	'Tasor'	2286.2	2320.3	757.7	1345.9	2469.9	2026.99	1788.05	1947.6	0.204616985	0.654480239	0.972790461
21886	'Tle2'	1319	1474	229	370	3321	907	1007.333333	1532.666667	0.616047346	0.460615196	0.972790461
218865	'Chdh'	48.73	23.56	7.22	234.89	54.43	238.54	26.50333333	175.9533333	2.97766088	4.50E-04	0.041129735
21887	'Tle3'	1776	1654	2761	1478	1585	1583	2063.666667	1548.666667	-0.379799676	0.396431	0.954259077
218877	'Sema3g'	197	193	107	357	217	143	165.6666667	239	0.762059107	0.208801891	0.836616817
21888	'Tle4'	1402	1448	1017	666	909	1174	1289	916.3333333	-0.451297307	0.080971499	0.618950211
218885	'Oxnad1'	466.74	466.92	550.65	235.04	425.33	553.07	494.77	404.48	-0.319263191	0.375802127	0.940406075
218914	'Wapl'	3958	4132	4043	4591	4222	4564	4044.333333	4459	0.23032742	0.522436671	0.972790461
21892	'Tll1'	7	6	10	2	9	13	7.666666667	8	-0.037651832	0.968295238	0.999493374
218921	'4930474N05Rik'	1	0	0	0	0	0	0.333333333	0	-0.903279821	0.824807108	0.972790461

21894	'Tln1'	5591	5259	6291	5332	6398	5687	5713.666667	5805.666667	0.077356917	0.825810312	0.972817749
218952	'Fermt2'	2315	2375	4335	3654	2405	2837	3008.333333	2965.333333	0.063368195	0.909667253	0.989364653
21897	'Tlr1'	27.5	26.99	13.04	3	6	3	22.51	4	-2.388527267	0.002696685	0.116112669
218973	'Wdhd1'	719	776	206	151	621	547	567	439.6666667	-0.363510783	0.544118216	0.972790461
218975	'Mapk1ip1l'	3126	3199	5233	2636	2859	3289	3852.666667	2928	-0.377694822	0.39442693	0.953202591
218977	'Dlgap5'	377	424	89	40	317	466	296.6666667	274.3333333	-0.161276119	0.839872336	0.974766672
21898	'Tlr4'	64	63	43	116	113	135	56.66666667	121.3333333	1.191437558	0.002332335	0.108416511
218989	'Tmem260'	463	450	124	441	816	632	345.6666667	629.6666667	0.956780525	0.062832864	0.566066012
21899	'Tlr6'	36.5	38.01	21.96	2	18	22	32.15666667	14	-1.239395457	0.108420132	0.685723069
219022	'Ttc5'	844	959	751	666	1158	1226	851.3333333	1016.666667	0.272344742	0.280677686	0.889418676
219024	'Pip4p1'	428	378	664	759	393	551	490	567.6666667	0.326800978	0.573871748	0.972790461
219026	'Eddm3b'	0	0	0	0	3	0	0	1	2.343657704	0.558557988	0.972790461
219033	'Ang4'	0	1	0	0	0	1	0.333333333	0.333333333	0.058500858	0.988561293	0.999493374
219038	'Tppp2'	0	0	0	1	0	0	0	0.333333333	1.020273531	0.802557913	0.972790461
21906	'Otop1'	0	1	4	0	0	0	1.666666667	0	-3.272106993	0.393925493	0.953178011
21907	'Nr2e1'	0	1	1	0	0	4	0.666666667	1.333333333	0.791283198	0.80037212	0.972790461
219072	'Haus4'	357	377	217	154	381	546	317	360.3333333	0.164324993	0.702056499	0.972790461
21908	'Tlx1'	0	1	0	0	0	1	0.333333333	0.333333333	0.058500858	0.988561293	0.999493374
21909	'Tlx2'	0	1	1	0	1	0	0.666666667	0.333333333	-0.886945817	0.805479824	0.972790461
219094	'Khnyn'	2054.5	1857.7	3772.6	888.64	1527.4	1233.35	2561.593333	1216.466667	-1.104925281	0.023136567	0.377626024
219103	'Cenpj'	332	365	146	204	362	292	281	286	0.100281533	0.805893001	0.972790461
219105	'Zmym5'	2530.5	2399.6	1380.9	733.99	2046	1585	2103.66	1454.996667	-0.522247434	0.173686973	0.79261444
219114	'Ska3'	252	237	90	66	168	216	193	150	-0.353373424	0.48779114	0.972790461
21912	'Tspan7'	806	864	964	679	1150	1327	878	1052	0.248746133	0.439329215	0.967427574
219131	'Phf11a'	6.46	8.06	6.49	8	8	11.81	7.003333333	9.27	0.491147893	0.543565511	0.972790461
219132	'Phf11d'	13.32	12.14	37	1	9.09	36.74	20.82	15.61	-0.665935508	0.556051574	0.972790461
219134	'Shisa2'	923	806	240	69	742	685	656.3333333	498.6666667	-0.447826745	0.560442745	0.972790461
219135	'Mtmr6'	1684	1720	1445	1491	1433	1500	1616.333333	1474.666667	-0.034961455	0.917562416	0.990547571
219140	'Spata13'	416	392	213	575	477	457	340.3333333	503	0.721491292	0.103948705	0.675879884
219144	'Arl11'	7	5	1	6	13	14	4.333333333	11	1.391631341	0.149586026	0.758505335
219148	'Fam167a'	64	67	21	7	21	28	50.66666667	18.66666667	-1.428357163	0.029964178	0.421360177
219149	'Xkr6'	252	268	24	268	153	106	181.3333333	175.6666667	0.256940758	0.766650954	0.972790461
21915	'Dtymk'	664	709	426	322	726	934	599.6666667	660.6666667	0.132285371	0.720780132	0.972790461
219150	'Hmbox1'	2987	2958	2640	2857	2691	2522	2861.666667	2690	0.017746774	0.961314331	0.999493374
219151	'Scara3'	219	218	244	252	594	554	227	466.6666667	1.008964301	0.005426516	0.174124727
219158	'Ccar2'	2295	2260	1113	1183	2032	1616	1889.333333	1610.333333	-0.15809514	0.654608315	0.972790461
21916	'Tmod1'	13.87	14.78	548.36	110.42	35.36	64.19	192.3366667	69.99	-1.45533552	0.273775121	0.885016511
21917	'Tmpo'	3586	3735	3205	2283	3784	3205	3508.666667	3090.666667	-0.148195145	0.562517644	0.972790461
219181	'Akap11'	4849	4858	1787	3048	5081	5741	3831.333333	4623.333333	0.335806177	0.422681614	0.95973972
219189	'Vwa8'	938	1023	495	655	1363	1446	818.6666667	1154.666667	0.523053673	0.144857884	0.752280685

21922	'Clec3b'	7	1	2	1	2	2	3.333333333	1.666666667	-0.963195181	0.521358995	0.972790461
219228	'Pcdh17'	200	223	40	34	400	173	154.3333333	202.3333333	0.373453941	0.657015908	0.972790461
21923	'Tnc'	460	374	1124	9144	691	879	652.6666667	3571.333333	2.772635658	0.011832104	0.26929851
21924	'Tnnc1'	6	7	31	36	3	9	14.66666667	16	0.335656432	0.787262569	0.972790461
219249	'Tdrd3'	1128	1057.5	864	153.97	1040	1025	1016.483333	739.6566667	-0.550878921	0.355438948	0.931809687
21925	'Tnnc2'	6	0	5	1	2	0	3.666666667	1	-1.833763221	0.375675501	0.940406075
219257	'Pcdh20'	19	29	10	0	16	3	19.33333333	6.333333333	-1.661632679	0.169841452	0.785454025
21926	'Tnf'	15	15	26	12	9	6	18.66666667	9	-0.961201055	0.2324013	0.859679981
21927	'Tnfaip1'	1549	1514	3222	1765	1607	1868	2095	1746.666667	-0.238853764	0.656568517	0.972790461
21928	'Tnfaip2'	175	157	1221	4295	158	727	517.6666667	1726.666667	1.975091136	0.114693887	0.701370273
21929	'Tnfaip3'	342	285	3191	425	197	251	1272.666667	291	-2.110414945	0.0379281	0.459930654
21930	'Tnfaip6'	12	6	235	505	24	57	84.33333333	195.3333333	1.424686124	0.333230325	0.921648675
21933	'Tnfrsf10b'	230	242.01	383	341	284	489	285.0033333	371.3333333	0.401846259	0.406873568	0.957157474
21934	'Tnfrsf11a'	9	4	11	53	10	19	8	27.33333333	2.005911403	0.04609561	0.50074283
21935	'Tnfrsf17'	0	0	0	0	0	1	0	0.333333333	1.020273531	0.802557913	0.972790461
21936	'Tnfrsf18'	27	32	8	1	32	20	22.33333333	17.66666667	-0.390251678	0.703591572	0.972790461
21937	'Tnfrsf1a'	960	973	1481	2658	1623	2433	1138	2238	1.063387622	0.039590177	0.464335867
21938	'Tnfrsf1b'	134	113	384	619	211	456	210.3333333	428.6666667	1.110806043	0.152290806	0.762591043
21939	'Cd40'	13	14	26	34	9	15	17.66666667	19.33333333	0.308422212	0.730910903	0.972790461
21940	'Cd27'	19	21	0	15	10	3	13.33333333	9.333333333	-0.192505735	0.889162861	0.984896546
21941	'Tnfrsf8'	8	4	18	14	5	19	10	12.66666667	0.350648083	0.725602679	0.972790461
21942	'Tnfrsf9'	6	4	95	12	4	14	35	10	-1.879424732	0.148882359	0.757738857
21943	'Tnfsf11'	0	3	14	57	0	5	5.666666667	20.66666667	2.147847284	0.275091643	0.886386629
21944	'Tnfsf12'	138.03	142.14	146.16	148.57	181.61	218.36	142.11	182.8466667	0.395440712	0.231660003	0.858905111
21945	'Dedd'	941.51	973.79	1161.2	503.75	922.2	1155.53	1025.503333	860.4933333	-0.285180729	0.424159037	0.960285372
21946	'Pglyrp1'	40	56	38	5	77	106	44.66666667	62.66666667	0.364370418	0.668691614	0.972790461
21948	'Cd70'	1	0	1	0	0	0	0.666666667	0	-1.858475606	0.644939423	0.972790461
21949	'Tnfsf8'	0	0	0	84	0	0	0	28	20.08874806	2.76E-07	1.29E-04
21950	'Tnfsf9'	60	35	233	420	29	191	109.3333333	213.3333333	1.121377105	0.306327461	0.907431861
21951	'Tnks'	1542	1439	1175	3297	1573	1645	1385.333333	2171.666667	0.862726282	0.12692298	0.72225672
21952	'Tnni1'	283	345	155	135	405	645	261	395	0.564450179	0.281528267	0.889731741
21953	'Tnni2'	2	0	0	12	1	1	0.666666667	4.666666667	3.259499132	0.136262631	0.736689633
21954	'Tnni3'	48	67	15	41.12	109	48.23	43.33333333	66.11666667	0.686592115	0.296775642	0.90029104
21955	'Tnnt1'	196	213	64	722	116	173	157.6666667	337	1.486442234	0.095026103	0.661529207
21956	'Tnnt2'	3	3	1	1	7	4	2.333333333	4	0.76731649	0.567231039	0.972790461
21957	'Tnnt3'	5	6	5	6	3	5	5.333333333	4.666666667	-0.076989138	0.939533013	0.995607401
21958	'Tnp1'	0	0	1	0	0	0	0.333333333	0	-0.903279821	0.824807108	0.972790461
21960	'Tnr'	5	6	0	5	22	9	3.666666667	12	1.767067269	0.143372534	0.749669589
21961	'Tns1'	2853	2698	2210	9986	2466	3794	2587	5415.333333	1.344739918	0.057456522	0.548760163
21968	'Tom1'	368	401	1071	333	475	534	613.3333333	447.3333333	-0.515816645	0.389462748	0.951231321

21969	'Top1'	3613	3913	2714	2646	4048	3704	3413.333333	3466	0.075603818	0.75839244	0.972790461
21973	'Top2a'	4535	4522	1633	464	2688	1522	3563.333333	1558	-1.206907643	0.046352867	0.501285957
21974	'Top2b'	4032	4205	2508	2256	4313	3402	3581.666667	3323.666667	-0.06142458	0.838982557	0.974723675
21975	'Top3a'	848	757	411	587	648	769	672	668	0.088921744	0.802794126	0.972790461
21976	'Top3b'	1538	1499	710	323	1528	1297	1249	1049.333333	-0.281606571	0.597078292	0.972790461
21981	'Ppp1r13b'	1451	1353	680	616	1053	1272	1161.333333	980.3333333	-0.20260388	0.568231733	0.972790461
21982	'Tmem165'	1039	1044	668	1113	1625	1758	917	1498.666667	0.76246157	0.003324088	0.131661584
21983	'Tpbg'	203	218	260	212	278	241	227	243.6666667	0.140816257	0.708778434	0.972790461
21985	'Tpd52'	699	580	4435	862	823	1403	1904.666667	1029.333333	-0.993959153	0.252000055	0.871252558
21987	'Tpd52l1'	203	220	214	554	250	505	212.3333333	436.3333333	1.175257144	0.026949559	0.403699531
21990	'Tph1'	9	7	192	7	23	28	69.33333333	19.33333333	-2.099042246	0.093773977	0.657893867
21991	'Tpi1'	8870	8417	30924	9111	6108	12524	16070.33333	9247.666667	-0.827583451	0.24499812	0.866091613
22003	'Tpm1'	6351	6909	7033	7982	7125	6085	6764.333333	7064	0.172637556	0.680031256	0.972790461
22004	'Tpm2'	337	316	1038	770	337	459	563.6666667	522	-0.019269171	0.979690964	0.999493374
22017	'Tpmt'	278	346	145	153	475	432	256.3333333	353.3333333	0.464691262	0.312828343	0.911912367
22018	'Tpo'	2	1	1	0	4	2	1.333333333	2	0.504748865	0.786092698	0.972790461
22019	'Tpp2'	2783	2723	4037	2234	2599	3298	3181	2710.333333	-0.221794341	0.577634405	0.972790461
22021	'Tpst1'	618	623	314	418	565	602	518.3333333	528.3333333	0.106858139	0.7505655	0.972790461
22022	'Tpst2'	526	520	268	555	818	741	438	704.6666667	0.765662109	0.023011113	0.377626024
22024	'Crisp2'	1	0	0	0	2	2	0.333333333	1.333333333	1.768945179	0.572584055	0.972790461
22025	'Nr2c1'	386	409	322	187	509	540	372.3333333	412	0.11610841	0.755891768	0.972790461
22026	'Nr2c2'	2451.1	2514.9	875.68	805.88	1823.4	1379.46	1947.2	1336.23	-0.484816041	0.28770517	0.894952226
22027	'Hsp90b1'	15949	13628	38443	11160	13080	39037	22673.33333	21092.33333	-0.225422141	0.735395142	0.972790461
22029	'Traf1'	69	74	268	64	110	180	137	118	-0.3500449	0.633527242	0.972790461
22030	'Traf2'	538	517	1349	759	492	545	801.3333333	598.6666667	-0.355243198	0.58449461	0.972790461
22031	'Traf3'	1810	1697	1287	300	1213	1219	1598	910.6666667	-0.86655623	0.064738594	0.571937799
22032	'Traf4'	833	861	1227	1068	930	1097	973.6666667	1031.666667	0.143493198	0.746398124	0.972790461
22033	'Traf5'	129	117	201	168	120	116	149	134.6666667	-0.048930353	0.930521261	0.993474751
22034	'Traf6'	661	653	1078	900	860	744	797.3333333	834.6666667	0.128550693	0.793514965	0.972790461
22035	'Tnfsf10'	11.92	15.18	6	2	5	6	11.03333333	4.333333333	-1.274802668	0.159758904	0.771114735
22036	'Traip'	229	227	72	36	193	245	176	158	-0.192308456	0.774403575	0.972790461
22037	'Trap1a'	78	78	40	3	76	236	65.33333333	105	0.555557686	0.591686921	0.972790461
22038	'Plscr1'	329	368	1390	506	317	514	695.6666667	445.6666667	-0.643734245	0.392308118	0.952648938
22040	'Trex1'	83	100	127	58	145	126	103.3333333	109.6666667	0.040110557	0.930065231	0.99325468
22041	'Trf'	78	101	80	6265	197	359	86.33333333	2273.666667	5.165231095	1.34E-05	0.003270239
22042	'Tfrc'	7517	6518	7819	5041	5313	6160	7284.666667	5504.666667	-0.350605143	0.304492606	0.907164095
22044	'Trh'	0	1	2	0	0	0	1	0	-2.490290069	0.532417892	0.972790461
22045	'Trhr'	0	0	0	0	0	1	0	0.333333333	1.020273531	0.802557913	0.972790461
22051	'Trip6'	723	785	1714	729	841	1022	1074	864	-0.335655227	0.53375863	0.972790461
22057	'Tob1'	1465	1403	1271	1144	1119	1426	1379.666667	1229.666667	-0.092766516	0.768351446	0.972790461

22059	'Trp53'	1526	1558	2595	670	1444	1344	1893	1152.666667	-0.771847399	0.086016231	0.63631929
22061	'Trp63'	29	21	1	1	104	456	17	187	3.379285683	0.017295032	0.328156826
22062	'Trp73'	14	18	2	3	8	6	11.33333333	5.666666667	-0.924826176	0.366437307	0.936406787
22063	'Trpc1'	225	258	158	62	148	79	213.6666667	96.33333333	-1.109733457	0.008170819	0.218179228
22064	'Trpc2'	78	82	29	39	115.58	49	63	67.86	0.160275493	0.787166408	0.972790461
22065	'Trpc3'	115	120	4	4	100	49	79.66666667	51	-0.658032805	0.57313477	0.972790461
22066	'Trpc4'	22	17	3	4	18	4	14	8.666666667	-0.624603585	0.549211757	0.972790461
22067	'Trpc5'	87	71	23	11	71	64	60.33333333	48.66666667	-0.334894228	0.644995744	0.972790461
22068	'Trpc6'	32.73	45	20	2.04	39	19	32.57666667	20.01333333	-0.757148719	0.391568831	0.952648938
22070	'Tpt1'	33298	31629	87638	74020	33262	40620	50855	49300.66667	0.06635515	0.926356167	0.992391389
22074	'Try4'	0	0	0	0	0	2	0	0.666666667	1.749449488	0.66566965	0.972790461
22083	'Ctr9'	2404	2480	1585	2016	2285	2385	2156.333333	2228.666667	0.139255164	0.643119604	0.972790461
22084	'Tsc2'	1931	1730.4	1188.2	1047.4	2028.9	1982	1616.553333	1686.08	0.085852488	0.762795956	0.972790461
22088	'Tsg101'	1428	1550	1570	1096	1277	1760	1516	1377.666667	-0.109766413	0.724769277	0.972790461
22092	'Rsph1'	47	52	37	10	58	60	45.33333333	42.66666667	-0.156459544	0.803982641	0.972790461
22094	'Tshb'	50	38	4	4	27	2	30.66666667	11	-1.414476533	0.228042788	0.854463832
22095	'Tshr'	63	63	12	15	36	40	46	30.33333333	-0.549839265	0.432651465	0.964346274
22099	'Tsn'	2585	2478	1586	574	2108	2507	2216.333333	1729.666667	-0.404887297	0.394225259	0.953178011
22110	'Tspyl1'	1814	1837	2600	1539	1712	2067	2083.666667	1772.666667	-0.21058649	0.593539087	0.972790461
22113	'Phlda2'	5	5	43	21	5	9	17.66666667	11.66666667	-0.512049107	0.674842387	0.972790461
22114	'Tssk1'	9	11	2	1	10	9	7.333333333	6.666666667	-0.154897909	0.89003507	0.984980969
22115	'Tssk2'	1	1	1	2	1	2	1	1.666666667	0.832791105	0.633382753	0.972790461
22116	'Tsks'	24	16	1	2	11	6	13.66666667	6.333333333	-1.06292517	0.365926973	0.936406787
22117	'Tst'	900	1004	328	680	1141	1453	744	1091.333333	0.610257298	0.175245227	0.794032951
22121	'Rpl13a'	17565	17880	39668	34550	15981	20590	25037.66667	23707	0.037884021	0.954717158	0.999493374
22122	'Gfus'	321	295	384	226	369	464	333.3333333	353	0.062807715	0.862469443	0.977161807
22123	'Psmd3'	1687	1712	2076	2600	1841	2260	1825	2233.666667	0.394171106	0.383483527	0.945630828
22127	'Tsx'	2	4	5	0	3	2	3.666666667	1.666666667	-1.252388935	0.414419756	0.95722888
22129	'Ttc3'	7502.8	7443	5967	2863	7199	6826	6970.903333	5629.33	-0.32404677	0.317601828	0.916136994
22130	'Ttf1'	746.09	785	479	261.21	643	587.98	670.03	497.3966667	-0.424350505	0.217363354	0.843822303
22134	'Tgoln1'	2254	2277	1726	9327	2453	4899	2085.666667	5559.666667	1.660864396	0.011979847	0.271043001
22137	'Ttk'	891	968	240	18	531	687	699.6666667	412	-0.843388147	0.378419431	0.9419364
22138	'Ttn'	57	77	21	38	133	185	51.66666667	118.6666667	1.184784505	0.062639334	0.566066012
22139	'Ttr'	0	0	0	0	0	1	0	0.333333333	1.020273531	0.802557913	0.972790461
22141	'Tub'	360	322	42	15	174	123	241.3333333	104	-1.234263009	0.166295951	0.780893009
22142	'Tuba1a'	2513.1	2735.5	4621.9	2324.4	3986.6	2997.53	3290.146667	3102.813333	-0.101645919	0.825803646	0.972817749
22143	'Tuba1b'	8100.8	8408.6	10840	3503.5	8762.6	9291.55	9116.363333	7185.893333	-0.404233439	0.321002731	0.917442833
22144	'Tuba3a'	315.64	276.69	211.22	0	53	122.01	267.85	58.33666667	-2.347619408	0.039921968	0.467074744
22145	'Tuba4a'	143.98	132.14	225.68	269.44	154.76	349.13	167.2666667	257.7766667	0.672583189	0.227141306	0.854001521
22146	'Tuba1c'	898.08	988.75	551.22	2053.1	1211.8	2621.92	812.6833333	1962.293333	1.395861239	0.002584814	0.114019676

22147	'Tuba3b'	66.36	45.31	53.78	0	6	4.99	55.15	3.663333333	-4.085700452	2.95E-07	1.34E-04
22151	'Tubb2a'	486.43	453.52	1093.2	645.03	779.58	907.49	677.7	777.3666667	0.171558991	0.753222041	0.972790461
22152	'Tubb3'	46	40	173	103	96	143	86.33333333	114	0.348727735	0.642856579	0.972790461
22153	'Tubb4a'	100	92	174	79	82	39	122	66.66666667	-0.792561091	0.208962068	0.836616817
22154	'Tubb5'	13178	13572	38365	12433	13394	14733	21705	13520	-0.704874456	0.249203257	0.869809497
22156	'Tuft1'	380	394	961	284	351	399	578.3333333	344.6666667	-0.779503343	0.170308604	0.785667192
22157	'Tulp1'	57	79	28	5	53	19	54.66666667	25.66666667	-1.11784028	0.163413655	0.775648491
22158	'Tulp3'	1337	1295	991	2233	1767	1680	1207.666667	1893.333333	0.793456064	0.058354241	0.551665011
22160	'Twist1'	101	89	290	264	105	93	160	154	0.091381774	0.911757244	0.989773583
22163	'Tnfrsf4'	12	14	8	6	10	11	11.33333333	9	-0.293465894	0.685005285	0.972790461
22164	'Tnfsf4'	0	1	1	0	3	0	0.666666667	1	0.448174855	0.889343571	0.984980969
22165	'Txk'	10	15	2	5	5	5	9	5	-0.701520393	0.494818112	0.972790461
22166	'Txn1'	698	754	2360	3709	1241	2223	1270.666667	2391	1.016768948	0.196636032	0.822696823
22169	'Cmpk2'	58	47	107	34	74	123	70.66666667	77	0.016040144	0.979259295	0.999493374
22171	'Tyms'	387.78	397.48	164.36	110.01	394.14	302.94	316.54	269.03	-0.230297449	0.64369324	0.972790461
22173	'Tyr'	6.07	2	1	2.88	4.92	4	3.023333333	3.933333333	0.20753919	0.873762448	0.980171118
22174	'Tyro3'	1149	1171	168	444	1555	1561	829.3333333	1186.666667	0.544847135	0.437642346	0.96622803
22177	'Tyrobp'	30	37	6	29	42	100	24.33333333	57	1.26820955	0.087445392	0.642039209
22183	'Zrsr1'	367	373	745.99	654.98	450.92	597	495.33	567.6333333	0.254139892	0.65431762	0.972790461
22184	'Zrsr2'	1498	1445	1440	498	1146	1830	1461	1158	-0.393044089	0.351110519	0.928880731
22185	'U2af2'	4481	4730	4445	2050	4635	4338	4552	3674.333333	-0.326185141	0.292784807	0.900043079
22186	'Uba52'	7058	7450	11773	9049	4552	6483	8760.333333	6694.666667	-0.268217936	0.641387778	0.972790461
22187	'Ubb'	24024	24646	55415	21232	25281	33857.13	34694.86	26789.93667	-0.408563661	0.445063291	0.969888068
22190	'Ubc'	17373	17013	39647	28666	18216	24499.87	24677.80667	23794.06333	0.005818931	0.992363084	0.999493374
22192	'Ube2m'	1045	1099	1214	739	1147	1338	1119.333333	1074.666667	-0.059505271	0.847088323	0.975209338
22193	'Ube2e3'	2136	2327	1753	872	1980	2232	2072	1694.666667	-0.305026507	0.341925081	0.925308103
22194	'Ube2e1'	1111	1148.2	1791.8	371.06	947.43	1584.88	1350.343333	967.79	-0.589556098	0.267721466	0.883600995
22195	'Ube2l3'	2410.9	2433	3198.8	1779.9	2285	2745	2680.883333	2269.96	-0.230771308	0.518076232	0.972790461
22196	'Ube2i'	4621	4624	5376	3744	4202	4832	4873.666667	4259.333333	-0.152264069	0.649160614	0.972790461
22200	'Uba3'	1478	1690	2292	2528	1603	1817	1820	1982.666667	0.237993129	0.648855681	0.972790461
22201	'Uba1'	10134	10956	7909	9580	14012	13565	9666.333333	12385.66667	0.410195694	0.079925323	0.617305615
22209	'Ube2a'	860	925	615	319	633	922	800	624.6666667	-0.366985226	0.305078296	0.907188578
22210	'Ube2b'	1908	1965	3255	2075	1953	2150	2376	2059.333333	-0.164164033	0.727810228	0.972790461
22213	'Ube2g2'	826	839	2101	1540	790	1433	1255.333333	1254.333333	0.052433377	0.936326738	0.994413066
22214	'Ube2h'	4064	4220	5774	6835	4163	5256	4686	5418	0.322928113	0.528132448	0.972790461
22215	'Ube3a'	3129	3143	3533	2200	3114	3532	3268.333333	2948.666667	-0.134972176	0.652353066	0.972790461
22217	'Usp12'	1600	1589	1163	1399	1639	1927	1450.666667	1655	0.258525548	0.332103685	0.921648675
22218	'Sumo1'	2347	2468	2286	1292	1878	2516	2367	1895.333333	-0.311207953	0.262279895	0.88013169
22221	'Ubp1'	2624.1	2508.5	2094.5	2455.9	2524.5	1979.38	2409.026667	2319.95	0.059526622	0.874244592	0.980171118
22222	'Ubr1'	1017	1130	445	476	1386	1006	864	956	0.172170051	0.704925211	0.972790461

22223	'Uchl1'	702	706	777	1004	1382	6163	728.3333333	2849.666667	1.867694739	0.00384618	0.141968654
22224	'Usp10'	1058	1176	887	1299	1377	1130	1040.333333	1268.666667	0.394140566	0.272571392	0.884738928
22225	'Usp5'	2442	2402	1778	1630	2535	2417	2207.333333	2194	0.037868051	0.872211188	0.980171118
22226	'Ucn'	0	0	0	0	1	1	0	0.666666667	1.775692139	0.660844521	0.972790461
22227	'Ucp1'	6	9	0	0	48	41	5	29.66666667	2.521063253	0.121868777	0.712603747
22228	'Ucp2'	1526	1862	496	914	2183	1970	1294.666667	1689	0.433392994	0.392738092	0.952648938
22229	'Ucp3'	1	1	0	1	1	1	0.666666667	1	0.724224998	0.748891473	0.972790461
22230	'Ufd1'	824	921	1339	764	870	1053	1028	895.6666667	-0.185644916	0.657681817	0.972790461
22232	'Slc35a2'	331	332	300	325	371	480	321	392	0.336126196	0.254746968	0.874390569
22234	'Ugcg'	1013	1017	1599	836	1445	1782	1209.666667	1354.333333	0.117288031	0.781763909	0.972790461
22235	'Ugdh'	1372	1373	6365	2336	1274	1642	3036.666667	1750.666667	-0.760030953	0.347903951	0.928444937
22236	'Ugt1a2'	3.87	0	0	0	0	0	1.29	0	-2.292035055	0.566710385	0.972790461
22239	'Ugt8a'	751	788	259	14	373	153	599.3333333	180	-1.795130266	0.042463951	0.479588013
22240	'Dpysl3'	1374	1454	2275	1160	2233	1894	1701	1762.333333	0.018559498	0.965395003	0.999493374
22241	'Ulk1'	1767	1753	1179	1699	1921	1670	1566.333333	1763.333333	0.27589814	0.406565839	0.957157474
22242	'Umod'	0	0	0	5	0	0	0	1.666666667	3.648996947	0.355284993	0.931809687
22245	'Uck1'	626	699	1026	945	710	817	783.6666667	824	0.152449049	0.758555331	0.972790461
22247	'Umps'	926	987	963	544	889	1020	958.6666667	817.6666667	-0.225998237	0.420098756	0.958308933
22248	'Unc119'	750	851	928	647	1104	1288	843	1013	0.250996645	0.443802697	0.969836424
22249	'Unc13b'	1716.3	1716.6	1023	517	1661	1155	1485.3	1111.003333	-0.420197565	0.305615558	0.907188578
22253	'Unc5c'	383.88	389.57	42	93.95	412.9	348.74	271.8166667	285.1966667	0.092382942	0.90610968	0.988799253
22255	'Uncx'	0	0	0	1	0	0	0	0.333333333	1.020273531	0.802557913	0.972790461
22256	'Ung'	320	307	436	237	228	497	354.3333333	320.6666667	-0.156463076	0.736446551	0.972790461
22258	'Usp4'	2660	2815	1630	1545	2535	2893	2368.333333	2324.333333	0.012588933	0.964723763	0.999493374
22259	'Nr1h3'	28	39	12	4	39	29	26.33333333	24	-0.172658247	0.833827704	0.974723675
22260	'Nr1h2'	722	699	1513	1035	876	1131	978	1014	0.075903084	0.889819659	0.984980969
22262	'Uox'	1	2	1	1	0	1	1.333333333	0.666666667	-0.862943462	0.687699568	0.972790461
22264	'Prap1'	1	1	1	4	0	0	1	1.333333333	0.833946791	0.733929642	0.972790461
22268	'Upk1b'	9	16	13	23	357	431	12.66666667	270.3333333	4.272716383	8.56E-07	3.19E-04
22269	'Upk2'	0	0	2	0	0	3	0.666666667	1	0.220651274	0.95546967	0.999493374
22270	'Upk3a'	0	5	2	0	1	0	2.333333333	0.333333333	-2.631321933	0.347114421	0.928307691
22271	'Upp1'	90	92	296	65	112	119	159.3333333	98.66666667	-0.781820681	0.244245334	0.866091613
22272	'Uqcrq'	426	537	704	859	520	831	555.6666667	736.6666667	0.493133531	0.338496223	0.924608313
22273	'Uqcrc1'	2274	2342	1703	1907	2358	3098	2106.333333	2454.333333	0.271072357	0.295637837	0.90029104
22275	'Urod'	831	945	548	553	1014	967	774.6666667	844.6666667	0.16239742	0.575509487	0.972790461
22276	'Uros'	247	282	63.04	190	286	189	197.3466667	221.6666667	0.30210266	0.599646775	0.972790461
22278	'Usf1'	852	936	1449	994	1013	1153	1079	1053.333333	-0.004396635	0.992198795	0.999493374
22282	'Usf2'	963	951	1830	866	1053	1286	1248	1068.333333	-0.239741839	0.618251758	0.972790461
22283	'Ush2a'	19	10	14	0	7	6	14.33333333	4.333333333	-1.824877011	0.085401048	0.634617076
22284	'Usp9x'	15753	16454	6483	8676	17336	21289	12896.66667	15767	0.322610408	0.441552986	0.968551476

22286	'Utf1'	4	10	49	2	2	4	21	2.666666667	-3.098363534	0.01226845	0.273764981
22287	'Scgb1a1'	0	1	0	1	1	0	0.333333333	0.666666667	1.081692101	0.760081965	0.972790461
22288	'Utrn'	1972	1974	1983	2481	2098	2205	1976.333333	2261.333333	0.298768137	0.445589416	0.969915971
22289	'Kdm6a'	2822	2928	2198	1485	2536	2224	2649.333333	2081.666667	-0.309788264	0.20542922	0.833294914
22290	'Uty'	5.02	2	1	4	6.02	2	2.673333333	4.006666667	0.715746843	0.583552569	0.972790461
22293	'Slc45a2'	0	1	1	0	3	0	0.666666667	1	0.448174855	0.889343571	0.984980969
22294	'Uxt'	215	162	139	71	159	186	172	138.6666667	-0.319835255	0.398220221	0.954563638
22295	'Cdh23'	631	746	97	47	1951	520	491.3333333	839.3333333	0.740370948	0.479830078	0.972790461
22297	'Vmn1r45'	3	1.01	0	0	1	2	1.336666667	1	-0.43987197	0.855554365	0.975734242
22301	'Vmn2r89'	0	0	0	1	1	0	0	0.666666667	2.050872943	0.610671515	0.972790461
22306	'Vmn2r30'	3	3	0	1.75	0	0	2	0.583333333	-2.323116309	0.41104239	0.95722888
22307	'Vmn2r10'	1	0	0	1	1	0	0.333333333	0.666666667	1.081626198	0.75986402	0.972790461
22308	'Vmn2r122'	0	1	0	0	0	0	0.333333333	0	-0.903279821	0.824807108	0.972790461
22310	'Vmn2r42'	1	0	0	0	0	0	0.333333333	0	-0.903279821	0.824807108	0.972790461
22317	'Vamp1'	253	266	65	170	254	125	194.6666667	183	0.06232013	0.918027444	0.990721335
22318	'Vamp2'	637	547	712	469	712	567	632	582.6666667	-0.09102528	0.799431094	0.972790461
22319	'Vamp3'	3094	3119	2702	1286	2562	3229	2971.666667	2359	-0.349838082	0.258535825	0.877564912
22320	'Vamp8'	724	904	1526	361	918	1010	1051.333333	763	-0.555946996	0.282071663	0.890082399
22321	'Vars'	1574	1448	1792	1109	1568	1830	1604.666667	1502.333333	-0.087988108	0.784311679	0.972790461
223227	'Sox21'	6	9	13	0	2	0	9.333333333	0.666666667	-3.863823161	0.015495598	0.309500711
22323	'Vasp'	1408	1523	2057	2177	1616	1945	1662.666667	1912.666667	0.287994059	0.53959936	0.972790461
22324	'Vav1'	41	39	13	13	44	38	31	31.66666667	0.047355963	0.941050861	0.996040262
22325	'Vav2'	1584	1676	554	2956	2009	2114	1271.333333	2359.666667	1.098388101	0.045093097	0.494982652
223254	'Farp1'	1686	1518	2102	1206	1934	1847	1768.666667	1662.333333	-0.091064498	0.797008178	0.972790461
223255	'Stk24'	4954	5071	7621	4799	5232	6494	5882	5508.333333	-0.077057795	0.852625814	0.975734242
22326	'Vax1'	3	8	4	0	3	0	5	1	-2.355189567	0.190510267	0.813136091
223262	'Timm8a2'	11	12	1	6	7	27	8	13.33333333	0.770641857	0.472807868	0.972790461
223267	'Ggact'	173	179	74	122	153	189	142	154.6666667	0.208234781	0.615826737	0.972790461
22327	'Vbp1'	1707	1773	2419	856	1225	1952	1966.333333	1344.333333	-0.582656276	0.151811485	0.761340005
223272	'Itgbl1'	4.28	3	0	12	2	3	2.426666667	5.666666667	1.659495396	0.285281759	0.893711786
22329	'Vcam1'	220	155	1248	178	179	203	541	186.6666667	-1.607499054	0.063770756	0.568241586
22330	'Vcl'	3090	2940	2435	6241	3940	4110	2821.666667	4763.666667	0.923515153	0.051750586	0.528400722
22333	'Vdac1'	4459	4546	14809	3785	4229	5272	7938	4428.666667	-0.891108536	0.169531437	0.785100556
223332	'Ranbp3l'	20	23	2	1392	10	675	15	692.3366667	5.915868961	5.97E-06	0.001645292
223337	'Ugt3a2'	0	0	0	1	0	0	0	0.333333333	1.020273531	0.802557913	0.972790461
22334	'Vdac2'	2725	2882	7293	2658	2436	3674	4300	2922.666667	-0.577051364	0.330832898	0.921648675
22335	'Vdac3'	4720	4934	4041	2044	5619	6104	4565	4589	-0.029189739	0.936930426	0.994413066
22337	'Vdr'	311	295	84	335	208	208	230	250.3333333	0.352974846	0.57176931	0.972790461
22339	'Vegfa'	8580	6580	29557	6430	4283	6227	14905.66667	5646.666667	-1.398675549	0.058508317	0.552046072
22340	'Vegfb'	468	437	1184	455	449	597	696.3333333	500.3333333	-0.496044174	0.404317112	0.956545617

22341	'Vegfc'	24.93	14.9	209.72	46.64	33.96	31.89	83.18333333	37.49666667	-1.195345843	0.264033735	0.880205046
22342	'Lin7b'	4	5	6	11	2	3	5	5.333333333	0.336722381	0.780175405	0.972790461
22343	'Lin7c'	1589	1473	2729	1579	1700	1967	1930.333333	1748.666667	-0.130161503	0.781315255	0.972790461
223433	'Otulinl'	57	60	34	149	63	68	50.33333333	93.33333333	1.134194293	0.08558585	0.634617076
223435	'Trio'	2816	2601	3201	2198	2887	2260	2872.666667	2448.333333	-0.181212375	0.618930752	0.972790461
22344	'Vezf1'	1786	1683	2671	2483	1719	1951	2046.666667	2051	0.097711367	0.848505905	0.975686757
223453	'Dap'	696	683	1472	1785	806	1232	950.3333333	1274.333333	0.530061418	0.416028405	0.95722888
223455	'Marchf6'	3755	3670	2256	5977	3783	4336	3227	4698.666667	0.72016502	0.126045232	0.72022881
22346	'Vhl'	1032	1008	2436	648	977	1147	1492	924	-0.750732091	0.170279313	0.785667192
223473	'Nipal2'	194.51	213.77	64.22	82.61	247.41	136.15	157.5	155.39	0.031693562	0.954638036	0.999493374
22348	'Slc32a1'	1	0	4	0	0	1	1.666666667	0.333333333	-2.306425405	0.443152484	0.96975311
22349	'Vil1'	64	69	26	33	93	307	53	144.3333333	1.383752348	0.063973382	0.5684378
223499	'Dcaf13'	1126	1234	1234	453	870	1161	1198	828	-0.563098421	0.102420763	0.671776916
22350	'Ezr'	1170	1261	1730	2248	1683	2459	1387	2130	0.683033703	0.135890307	0.736689633
22351	'Vill'	317	329	334	441	536	588	326.6666667	521.6666667	0.719498072	0.019027042	0.344064642
223513	'Abra'	1	0	4	10	0	0	1.666666667	3.333333333	1.322764429	0.617206746	0.972790461
22352	'Vim'	10765	10158	52671	16261	19064	18726	24531.33333	18017	-0.510671238	0.508042336	0.972790461
223527	'Eny2'	1106	1222	1428	1018	1141	1301	1252	1153.333333	-0.079443345	0.822972502	0.972790461
22353	'Vip'	0	3	0	0	4	1	1	1.666666667	0.726836349	0.801770458	0.972790461
22354	'Vipr1'	21	26	33	96	24	29	26.66666667	49.66666667	1.145781292	0.177513201	0.797090452
22355	'Vipr2'	36.04	33	15	9	25	16	28.01333333	16.66666667	-0.709130759	0.256994676	0.8773831
22359	'Vldlr'	1086	808	1774	386	401	858	1222.666667	548.3333333	-1.203504618	0.024853848	0.389869108
223593	'Washc5'	2074	1898	1868	978	1889	2340	1946.666667	1735.666667	-0.183449749	0.554206755	0.972790461
22360	'Nrsn1'	7	14	1	0	1	1	7.333333333	0.666666667	-3.403679254	0.048197415	0.510205601
223601	'Cyrib'	1552	1575	1023	394	1180	1368	1383.333333	980.6666667	-0.527523728	0.202897302	0.83083072
223604	'Kcnk9'	2	3	0	0	0	0	1.666666667	0	-3.014977875	0.444538231	0.969888068
22361	'Vnn1'	295	344	1687	2005	369	706	775.3333333	1026.666667	0.558842452	0.572246371	0.972790461
22362	'Vpreb1'	0	3	2	0	1	1	1.666666667	0.666666667	-1.400185414	0.55970161	0.972790461
223626	'Them6'	9	8	5	143	12	33	7.333333333	62.66666667	3.469933342	0.00110294	0.070130753
22363	'Vpreb2'	0	2	0	0	1	0	0.666666667	0.333333333	-0.733814424	0.856146772	0.975734242
22364	'Vpreb3'	7	3	2	10	2	10	4	7.333333333	1.051727491	0.367857211	0.937190492
223642	'Zc3h3'	657	616	794	545	522	487	689	518	-0.333926433	0.424531969	0.960285372
223645	'Mroh6'	17	8	4	19	23	10	9.666666667	17.33333333	1.000190066	0.239019536	0.864416858
223646	'Naprt'	145	176	107	69	220	201	142.6666667	163.3333333	0.174995227	0.685366957	0.972790461
223648	'Ccdc166'	92	85	89	3	92	101	88.66666667	65.33333333	-0.589149906	0.5151257	0.972790461
223649	'Nrbp2'	1768	1864	586	136	4921	1831	1406	2296	0.644400277	0.490674094	0.972790461
22365	'Vps45'	656	623	300	138	423	479	526.3333333	346.6666667	-0.613605164	0.188663622	0.810921289
223650	'Eppk1'	52	41	36	216	154	87	43	152.3333333	2.019333209	9.52E-04	0.064169552
223658	'Mroh1'	1221	1144	1911	2870	1298	1750	1425.333333	1972.666667	0.611412055	0.315480952	0.914723408
223664	'Lrrc14'	512	567	299	190	559	495	459.3333333	414.6666667	-0.149342593	0.714067505	0.972790461

223665	'C030006K11Rik'	118	138	114	115	114	150	123.3333333	126.3333333	0.10033768	0.780196964	0.972790461
223666	'Arhgap39'	1125.6	1162.1	649	427.07	1131	1142	978.89	900.0233333	-0.122818578	0.747247995	0.972790461
223669	'Zfp7'	241	251	325	256	262	222	272.3333333	246.6666667	-0.072245938	0.869412155	0.979633196
22367	'Vrk1'	739	757	422	263	612	507	639.3333333	460.6666667	-0.447739545	0.200965871	0.828439535
223672	'Apol9a'	8.03	8.39	102.11	18.8	1	42.16	39.51	20.65333333	-1.088061169	0.426571199	0.961238785
22368	'Trpv2'	16	11	13	23	18	33	13.33333333	24.66666667	0.947009367	0.130995573	0.728882802
223690	'Ankrd54'	408	419	357	491	508	444	394.6666667	481	0.384706646	0.285100845	0.893711786
223691	'Eif3l'	2539	2608	3226	2293	2850	3609	2791	2917.333333	0.077024739	0.8230286	0.972790461
223693	'Tmem184b'	934	921	753	2339	1027	1167	869.3333333	1511	1.015298976	0.079617306	0.617305615
223696	'Tomm22'	774	743	869	628	1039	1384	795.3333333	1017	0.33423814	0.323540449	0.918976057
223697	'Sun2'	768	749	898	1020	1316	1496	805	1277.333333	0.688236238	0.034260762	0.444932876
22370	'Vtn'	0	4	0	0	2	6	1.333333333	2.666666667	0.947772834	0.719292549	0.972790461
223701	'Mrtfa'	1033	953	602	1382	765	1022	862.6666667	1056.333333	0.476230717	0.337802138	0.924020038
22371	'Vwf'	19	20	12	68	21	54	17	47.66666667	1.680756179	0.02059818	0.356011446
223722	'Mcat'	552	562	282	337	567	553	465.3333333	485.6666667	0.119476489	0.721753177	0.972790461
223723	'Ttll12'	1535	1506	1120	1514	1560	2059	1387	1711	0.378534433	0.201701648	0.82952222
223726	'Mpped1'	111	103	12	0	24	28	75.33333333	17.33333333	-2.174372634	0.066065628	0.575109762
223732	'Rtl6'	876	845	352	167	857	856	691	626.6666667	-0.177306045	0.760023387	0.972790461
223739	'5031439G07Rik'	1731	1724	2166	1141	1659	1945	1873.666667	1581.666667	-0.245694431	0.465550694	0.972790461
22375	'Wars'	1084	1143	2281	597	993	1293	1502.666667	961	-0.712262237	0.160926161	0.771435531
223752	'Gramd4'	1309	1193	1191	1781	1684	1931	1231	1798.666667	0.630100653	0.065897843	0.574593252
223753	'Cerk'	592	607	489	1077	999	1167	562.6666667	1081	1.037556009	0.002063257	0.100323878
223754	'Tbc1d22a'	777.02	841.42	1387	678.92	794	932.28	1001.826667	801.7333333	-0.319304616	0.478704056	0.972790461
22376	'Was'	9	13	0	6	10	9	7.333333333	8.333333333	0.310266566	0.799287769	0.972790461
22377	'Wbp1'	511	538	255	349	674	613	434.6666667	545.3333333	0.375613556	0.29344236	0.90029104
223770	'Brd1'	2492	2443	2250	1021	2309	2271	2395	1867	-0.375566713	0.220847821	0.849076011
223773	'Zbed4'	1182	1206	521	535	964	758	969.6666667	752.3333333	-0.292951685	0.450170789	0.970590732
223774	'Alg12'	129	120	204	80	157	188	151	141.6666667	-0.149639767	0.7518152	0.972790461
223775	'Pim3'	800.69	753.85	2866	2607	666.98	1005.9	1473.506667	1426.61	0.106943089	0.902987396	0.987381169
223776	'Selenoo'	347.61	338.15	280.95	754.43	414.06	412.61	322.2366667	527.0333333	0.903866322	0.091921956	0.65161082
22378	'Wbp2'	928	971	3073	1904	883	1345	1657.333333	1377.333333	-0.197374687	0.789414043	0.972790461
223780	'Adm2'	49	27	535	18	15	68	203.6666667	33.66666667	-2.815827945	0.013663998	0.292512191
22379	'Fmnl3'	981	1016	455	699	1092	1056	817.3333333	949	0.289610596	0.420252625	0.958308933
22380	'Wbp4'	958	1001	931	1071	945	1214	963.3333333	1076.666667	0.243560477	0.488406096	0.972790461
22381	'Tceal9'	2773	2759	8622	3004	3102	3166	4718	3090.666667	-0.625818346	0.330509049	0.921648675
223825	'Mroh2b'	3	2	1	0	1	2	2	1	-1.044409837	0.579589091	0.972790461
223827	'Gxylt1'	1704	1677	685	772	1227	1115	1355.333333	1038	-0.30204831	0.444342176	0.969888068
223828	'Pphln1'	1402	1476	982	597	1256	1571	1286.666667	1141.333333	-0.177603706	0.590899018	0.972790461
223838	'Adamts20'	115	119	63	4	76	48	99	42.66666667	-1.293586441	0.108991459	0.688191839
22384	'Eif4h'	5175	5424	7400	4413	4996	5724	5999.666667	5044.333333	-0.223727323	0.562562933	0.972790461

223843	'Dbx2'	2	1	20	0	2	0	7.666666667	0.666666667	-3.726754559	0.071362173	0.591776309
22385	'Baz1b'	6471	6384	2777	4017	5294	3918	5210.666667	4409.666667	-0.119590117	0.77318803	0.972790461
223864	'Rapgef3'	213	181	95	273	321	230	163	274.6666667	0.883526909	0.045304687	0.496605985
223870	'Senp1'	1172	1180	716	516	1117	838	1022.666667	823.6666667	-0.277569519	0.396470253	0.954259077
22388	'Wdr1'	2309	2456	2543	2431	2527	3038	2436	2665.333333	0.18601425	0.571616732	0.972790461
223881	'Rnd1'	45	70	846	380	41	98	320.3333333	173	-0.736405242	0.557755216	0.972790461
22390	'Wee1'	796	737	372	399	586	1021	635	668.6666667	0.103713118	0.800037291	0.972790461
223917	'Krt79'	40	49	47	1	32	69	45.33333333	34	-0.578482777	0.562185673	0.972790461
223918	'Spryd3'	648	678	934	278	543	885	753.3333333	568.6666667	-0.477069484	0.296564782	0.90029104
223920	'Soat2'	12	6	61	4	13.01	9	26.33333333	8.67	-1.771056422	0.101298519	0.671759705
223921	'Aaas'	858	873	422	242	521	776	717.6666667	513	-0.483530856	0.261614414	0.87954257
223922	'Atf7'	2186	2059	3783	1411	1732	1723	2676	1622	-0.715831121	0.121453482	0.712603747
22393	'Wfs1'	478	520	831	237	615	694	609.6666667	515.3333333	-0.329440298	0.500203597	0.972790461
223970	'Rmi2'	62	69	16	13	24	25	49	20.66666667	-1.170427536	0.071465083	0.592255916
223978	'Cpped1'	667	720	789	97	768	731	725.3333333	532	-0.571574428	0.373318616	0.93929675
223989	'Marf1'	4727	4543	2452	2029	3629	4014	3907.333333	3224	-0.23894806	0.459212611	0.972790461
224008	'Spidr'	1130.3	1086.2	3560.6	504.78	735.53	937.9	1925.71	726.07	-1.494126911	0.019719005	0.349283865
22401	'Zmat3'	586	547	707	691	493	750	613.3333333	644.6666667	0.149291247	0.737009183	0.972790461
224014	'Fgd4'	745	805	650	407	940	846	733.3333333	731	-0.010117842	0.973759934	0.999493374
224019	'Tmem191c'	108	117	96	13	147	115	107	91.66666667	-0.327898005	0.645459015	0.972790461
22402	'Ccn4'	357	333	620	442	305	520	436.6666667	422.3333333	-0.007452862	0.988854241	0.999493374
224020	'Pi4ka'	4216	4016	2187	1287	2819	2950	3473	2352	-0.543421929	0.122014995	0.712891547
224022	'Slc7a4'	157	148	34	88	326	228	113	214	0.95102411	0.134211591	0.733437989
224023	'Klhl22'	2317	2270.9	1796	1199	1729	1686	2127.95	1538	-0.41759343	0.076874352	0.609798765
224024	'Scarf2'	1029	924	478	467	1001	799	810.3333333	755.6666667	-0.056503358	0.879190414	0.981796025
22403	'Ccn5'	1	2	5	16	6	15	2.666666667	12.33333333	2.281213715	0.042025078	0.47785932
22404	'Wiz'	2079	2027	1997	1423	1840	1656	2034.333333	1639.666667	-0.255095406	0.389191185	0.951190011
224044	'Cyp2ab1'	1	0	2	1	1	0	1	0.666666667	-0.51759566	0.841477705	0.975182082
224045	'Eif2b5'	1177	1257	1202	709	1271	1691	1212	1223.666667	-0.004248483	0.989333052	0.999493374
224055	'Rtp2'	1	0	0	0	0	0	0.333333333	0	-0.903279821	0.824807108	0.972790461
224079	'Atp13a4'	29	37	8	1	6	10	24.66666667	5.666666667	-2.12733848	0.023022515	0.377626024
22408	'Wnt1'	0	0	1	8	0	0	0.333333333	2.666666667	3.367484634	0.317306503	0.916136994
224088	'Atp13a3'	3221	3432	2945	7208	4543	5130	3199.333333	5627	0.969183717	0.036955529	0.459648634
22409	'Wnt10a'	16	24	22	12	9	11	20.66666667	10.66666667	-0.873085397	0.196934869	0.822696823
224090	'Tmem44'	196	204	33	42	373	181	144.3333333	198.6666667	0.456324727	0.576019082	0.972790461
224092	'Lsg1'	856	941	1020	753	862	940	939	851.6666667	-0.094931828	0.776877106	0.972790461
224093	'Fam43a'	337	339	351	214	575	453	342.3333333	414	0.245519088	0.511728333	0.972790461
224098	'Gm536'	3	5	7	0	1	0	5	0.333333333	-3.789742407	0.043319181	0.484746476
22410	'Wnt10b'	146	116	318	33	36	13	193.3333333	27.33333333	-2.766431529	1.55E-04	0.019123368
224105	'Pak2'	2579	2459	3837	1422	2501	2917	2958.333333	2280	-0.416719745	0.3073645	0.907830798

224109	'Nrros'	830.24	674.91	263.15	211	649.94	443.37	589.4333333	434.77	-0.408030704	0.423513511	0.960285372
22411	'Wnt11'	114	88	229	57	144	103	143.6666667	101.3333333	-0.582414108	0.327602229	0.920812178
224111	'Ubxn7'	2698	2867	3463	2789	2418	2642	3009.333333	2616.333333	-0.121551122	0.769448427	0.972790461
224116	'Muc20'	0	2	2	6	5	5	1.333333333	5.333333333	2.074113209	0.144923798	0.752280685
22412	'Wnt9b'	8	5	0	0	6	0	4.333333333	2	-1.107058146	0.647847961	0.972790461
224129	'Adcy5'	108	104	30	142	91	87	80.66666667	106.6666667	0.627698534	0.327362259	0.920734892
22413	'Wnt2'	10	10	12	0	9	16	10.66666667	8.333333333	-0.511069616	0.660183831	0.972790461
224132	'Slc49a4'	478	493	361	344	752	775	444	623.6666667	0.489937565	0.109066885	0.688210958
224139	'Golgb1'	4513	4163	2702	2580	4382	3581	3792.666667	3514.333333	-0.052713715	0.855848354	0.975734242
22414	'Wnt2b'	1360	1444	55	73	1512	1287	953	957.3333333	-0.021782155	0.984075516	0.999493374
224143	'Poglut1'	683	740	418	318	968	905	613.6666667	730.3333333	0.238746506	0.557407664	0.972790461
22415	'Wnt3'	14	11	3	0	4	5	9.333333333	3	-1.670460757	0.183090547	0.804092742
22416	'Wnt3a'	0	3	0	0	0	0	1	0	-2.275512037	0.569613276	0.972790461
22417	'Wnt4'	2283.9	2346.2	1987	500	2461	2287	2205.68	1749.333333	-0.4104319	0.421711258	0.959384282
224170	'Dzip3'	4604	4443	1044	615	2839	2168	3363.666667	1874	-0.83654209	0.191212255	0.813837363
224171	'Cip2a'	1095	1113	300	95	920	1034	836	683	-0.345202082	0.652739146	0.972790461
22418	'Wnt5a'	895	875	384	3717	797	882	718	1798.666667	1.687508541	0.038386012	0.459930654
22419	'Wnt5b'	236	267.9	99	26	398	293	200.9666667	239	0.183221956	0.816955325	0.972790461
22420	'Wnt6'	1672	1791	318	667	3470	2106	1260.333333	2081	0.739839125	0.290109445	0.898146215
22421	'Wnt7a'	12	4	2	4	9	42	6	18.33333333	1.557868735	0.163669054	0.776172627
22422	'Wnt7b'	28	22	94	54	24	22	48	33.33333333	-0.419371308	0.641356431	0.972790461
224224	'Impg2'	206.35	261.34	99.09	295.57	305.53	166.66	188.9266667	255.92	0.614540519	0.25849684	0.877564912
22423	'Wnt8b'	1	1	0	0	0	0	0.666666667	0	-1.703500596	0.673820112	0.972790461
224247	'Ftdc2'	0	0	0	0	2	4	0	2	3.287939522	0.381618281	0.94429001
224250	'Cldnd1'	901	960	1608	2333	1287	1878	1156.333333	1832.666667	0.761384716	0.179125463	0.798783498
22427	'Wrn'	658	642	298	243	736	596	532.6666667	525	-0.010319178	0.981385413	0.999493374
224273	'Crybg3'	646.35	568.26	205.26	461.05	831.64	1373.56	473.29	888.75	0.937477872	0.061275405	0.562479029
22428	'Dctn6'	899	890	823	564	1015	1020	870.6666667	866.3333333	0.001304703	0.996026912	0.999602178
22431	'Wt1'	4677	4480	2303	780	4209	3674	3820	2887.666667	-0.448553067	0.41854717	0.958226521
224318	'Speer2'	1	2	1	0	0	0	1.333333333	0	-2.778388356	0.385894181	0.947516488
22433	'Xbp1'	1140	1175	7585	1861	1281	2854	3300	1998.666667	-0.806842705	0.353556276	0.930225841
224344	'Rbm11'	58	54	35	12	58	58	49	42.66666667	-0.245566957	0.678102058	0.972790461
22436	'Xdh'	12	13	108	420	92	295	44.33333333	269	2.646896719	0.011807927	0.269175441
22437	'Xirp1'	3	2	0	2	5	4	1.666666667	3.666666667	1.206233685	0.426256196	0.960958977
22439	'Xk'	275	302	70	26	208	227	215.6666667	153.6666667	-0.52791459	0.487636703	0.972790461
224405	'Cyyr1'	95	92	16	0	216	109	67.66666667	108.3333333	0.617865017	0.647109935	0.972790461
22441	'Xlr'	346	333	164	2	129	248	281	126.3333333	-1.275172788	0.223157763	0.850405971
224419	'Map3k7cl'	3	4	2	2	0	3	3	1.666666667	-0.735138627	0.64519457	0.972790461
224432	'Scaf4'	1754	1782	1017	1316	1535	1347.01	1517.686667	1399.353333	-0.007796084	0.982022886	0.999493374
224440	'Setd4'	116	130	76	21	123	105	107.3333333	83	-0.425219889	0.46796737	0.972790461

22445	'Xlr3a'	66.22	54.36	129.49	16.45	66.08	197.08	83.35666667	93.20333333	-0.029307651	0.971779855	0.999493374
224454	'Zdhhc14'	65.8	61.94	37	46.85	60.96	50.96	54.91333333	52.92333333	0.023309906	0.956677369	0.999493374
22446	'Xlr3c'	182.28	157.83	95.37	0	63.62	118.3	145.16	60.64	-1.394563935	0.231487782	0.858905111
224480	'Nox3'	0	1	0	0	0	0	0.333333333	0	-0.903279821	0.824807108	0.972790461
224481	'Tfb1m'	392.83	424.56	241.37	35	279.93	375.25	352.92	230.06	-0.707489982	0.300635799	0.904128654
224530	'Acat3'	7.99	8.96	6.03	1264.9	20.23	345.05	7.66	543.4066667	6.647638763	2.06E-08	1.35E-05
224576	'Vmn2r106'	1	0	0	0	0	0	0.333333333	0	-0.903279821	0.824807108	0.972790461
224585	'Zfp160'	735	803	505	297	1098	510	681	635	-0.102589534	0.829333905	0.973678583
224598	'Zfp758'	253	279	283	149	310	262	271.6666667	240.3333333	-0.182581788	0.58917395	0.972790461
224613	'Flywch1'	1067.7	1149.6	760	487	1143.1	1009.99	992.4266667	880.02	-0.165767189	0.600121247	0.972790461
224617	'Tbc1d24'	1035	992	765	1139	1009	963	930.6666667	1037	0.282079846	0.461652319	0.972790461
224619	'Traf7'	1062	1142	1181	1531	1410	1230	1128.333333	1390.333333	0.403546196	0.324408936	0.919683661
224624	'Rab40c'	671	712	815	632	655	765	732.6666667	684	-0.047963718	0.893878221	0.985171
224630	'Bnip1'	236	268	458	127	266	273	320.6666667	222	-0.594529981	0.223630349	0.850759465
224640	'Lemd2'	723	689	471	1350	1010	1338	627.6666667	1232.666667	1.104139626	0.005600087	0.177152822
224647	'Ilrun'	2842	2868	5494	8160	2573	4440	3734.666667	5057.666667	0.597848364	0.394099518	0.953178011
224648	'Uhrf1bp1'	1392	1325	912	660	1256	1382	1209.666667	1099.333333	-0.12118215	0.669040649	0.972790461
224650	'Anks1'	1211	1227.5	465	1335	1111.2	1081.25	967.8233333	1175.8	0.45586516	0.353293273	0.930050349
224656	'Zfp523'	896	918	409	252	972	767	741	663.6666667	-0.167659513	0.733502219	0.972790461
224661	'Slc26a8'	458	465	69	26	322	214	330.6666667	187.3333333	-0.844834164	0.329337495	0.921648675
224671	'Btbd9'	2227	2241	1705	756	1141	1246	2057.666667	1047.666667	-0.936555861	4.04E-05	0.007241689
224674	'Slc37a1'	79	80	62	74	164	110	73.66666667	116	0.678701763	0.078120141	0.614954936
224691	'Zfp472'	203	172	125	53	199	203	166.6666667	151.6666667	-0.176806543	0.712778004	0.972790461
224694	'Zfp81'	673	671	735	282	738	599	693	539.6666667	-0.393221138	0.289301183	0.896381044
224697	'Adamts10'	1445	1302	418	1019	1987	971	1055	1325.666667	0.438413502	0.414953268	0.95722888
224703	'Marchf2'	554	562	297	590	520	662	471	590.6666667	0.448981403	0.248748338	0.869594312
224705	'Vps52'	964	1078	647	286	1049	947	896.3333333	760.6666667	-0.268090725	0.545507345	0.972790461
224727	'Bag6'	5301	5185	4992	4018	6177	5722	5159.333333	5305.666667	0.072000076	0.779409939	0.972790461
224742	'Abcf1'	2178	2115	1934	2069	2067	2614	2075.666667	2250	0.186293116	0.546383579	0.972790461
224792	'Adgrf5'	1076	977	208	258	1740	1503	753.6666667	1167	0.612595254	0.400767538	0.955860942
224794	'Enpp4'	332	357	138	154	356	433	275.6666667	314.3333333	0.207987652	0.648734519	0.972790461
224796	'Clic5'	31	29	151	211	50	115	70.33333333	125.3333333	0.934061356	0.328827781	0.921648675
224805	'Aars2'	269	293	243	434	452	372	268.3333333	419.3333333	0.747157759	0.047292681	0.50504201
224807	'Tmem63b'	688	650	660	1316	884	1222	666	1140.666667	0.891817094	0.038715088	0.460965603
224813	'Lrrc73'	36	25	29	7	29	20	30	18.66666667	-0.722229007	0.247787941	0.869184025
224814	'Abcc10'	2933.1	2700.6	592.34	327.41	5614.5	11021.84	2075.323333	5654.596667	1.36884424	0.148464357	0.756933322
224823	'Rrp36'	345	319	332	162.15	322	367	332	283.7166667	-0.244137854	0.455666043	0.971239019
224824	'Pex6'	1204	1219	705	1118	1502	1754	1042.666667	1458	0.548438349	0.059198559	0.552865869
224826	'Ubr2'	3043	2949	2351	2051	3299	2495	2781	2615	-0.034234404	0.903474915	0.987564084
224829	'Trerf1'	801	781	978	668	768	765	853.3333333	733.6666667	-0.172092553	0.637147912	0.972790461

224833	'AI661453'	55	64	56	21	79	76	58.33333333	58.66666667	-0.045822377	0.929088329	0.993096978
224836	'Usp49'	889	879	641	469	775	637	803	627	-0.3066655	0.258126097	0.877405867
224840	'Treml4'	3	1	0	0	5	5	1.333333333	3.333333333	1.273509718	0.531497391	0.972790461
224860	'Plcl2'	807	741	644	348	615	871	730.6666667	611.3333333	-0.265861593	0.405898271	0.957157474
224893	'Zfp959'	162.13	215.23	131.98	53.96	174	137	169.78	121.6533333	-0.496640818	0.256170418	0.876684814
224897	'Dpp9'	786	697	669	515	786	732	717.3333333	677.6666667	-0.046135514	0.861785096	0.976863639
224902	'Safb2'	3048.5	2958	3011.3	2583.4	3378.7	2051.7	3005.963333	2671.276667	-0.089512042	0.820828469	0.972790461
224903	'Safb'	3135.5	3129	3146.7	1993.6	3270.3	2455.3	3137.036667	2573.056667	-0.251526453	0.41278705	0.95722888
224904	'Micos13'	666	722	802	463	534	849	730	615.3333333	-0.237223453	0.50736992	0.972790461
224907	'Dus3l'	684	706	518	307	702	670	636	559.6666667	-0.184382791	0.55006398	0.972790461
224912	'Crb3'	116	114	39	42	78	218	89.66666667	112.6666667	0.315272669	0.628699065	0.972790461
224916	'Vmn2r120'	0	0	0	1	0	0	0	0.333333333	1.020273531	0.802557913	0.972790461
224938	'Pja2'	3879	4088	6487	7005	3820	4884	4818	5236.333333	0.237444925	0.675412791	0.972790461
224997	'Dlgap1'	41	54	44	117	132	88	46.33333333	112.3333333	1.378082936	0.0034429	0.134593203
225004	'Pcare'	21	13	1	1	4	7	11.66666667	4	-1.521027127	0.222953806	0.85026726
225010	'Lclat1'	594	599	289	702	736	695	494	711	0.653873676	0.100203619	0.671535889
225020	'Fez2'	432	418	584	1150	496	828	478	824.6666667	0.927908165	0.109815852	0.689773582
225027	'Srsf7'	3328	3247	2838	671	3435	2338	3137.666667	2148	-0.612067131	0.227028195	0.854001521
225028	'Map4k3'	1828	1836	1674	1272	2231	1939	1779.333333	1814	0.050297572	0.846939187	0.975209338
225030	'Kcng3'	787.02	784.08	165	119	1013	635.01	578.7	589.0033333	0.007299972	0.992281719	0.999493374
225049	'Ttc7'	279	303	124	155	304	298	235.3333333	252.3333333	0.148943012	0.712909452	0.972790461
225055	'Fbxo11'	2519	2556	2524	2228	2807	2675	2533	2570	0.074621335	0.79967253	0.972790461
225058	'Gm4832'	0	0	0	0	0	1	0	0.333333333	1.020273531	0.802557913	0.972790461
225115	'Svil'	1750	1679	636	787	2277	1633	1355	1565.666667	0.240880327	0.61245406	0.972790461
225131	'Wac'	2555	2557	3768	1894	2537	2744	2960	2391.666667	-0.304439397	0.434982551	0.965608974
225152	'Gjd4'	3	3	0	1	1	1	2	1	-0.844587602	0.662463344	0.972790461
225160	'Thoc1'	1281.5	1453.9	851.64	363.15	1412.4	830.3	1195.653333	868.6	-0.474147159	0.303589715	0.906520549
225164	'Mib1'	4909	5129	3561	3471	4377	4321	4533	4056.333333	-0.085057018	0.749915723	0.972790461
225182	'Rbbp8'	1236	1190	748	757	940	807	1058	834.6666667	-0.245115653	0.4522866	0.970649024
225187	'Ankrd29'	129	115	51	3	68	28	98.33333333	33	-1.634044359	0.058370741	0.551665011
225192	'Hrh4'	4	1	0	0	1	2	1.666666667	1	-0.758373329	0.748700869	0.972790461
225207	'Zfp521'	1225	1202	483	114	1199	1059	970	790.6666667	-0.359778465	0.616927727	0.972790461
225215	'Rsl24d1'	1125	1218	1846	784	920	1563	1396.333333	1089	-0.383466746	0.390046292	0.951811077
225256	'Dsg1b'	0	0	0	0	39.84	26	0	21.94666667	6.735745716	0.0026702	0.115903767
225266	'Klhl14'	46	49	15	12	75	52	36.66666667	46.33333333	0.321401419	0.654500102	0.972790461
225280	'Ino80c'	334	389	185	258	378	372	302.6666667	336	0.224840489	0.512671707	0.972790461
225283	'Rprd1a'	1873	1863	1601	1934	1604	1938	1779	1825.333333	0.143371381	0.695053576	0.972790461
225288	'Fhod3'	423	387	459	243	400	443	423	362	-0.228146127	0.478669245	0.972790461
225289	'AW554918'	634	650.26	285	71	464	422	523.0866667	319	-0.762512703	0.217309924	0.843822303
225326	'Pik3c3'	959.15	874.66	849.82	763.11	865.08	1126.96	894.5433333	918.3833333	0.084782355	0.770622929	0.972790461

225339	'Ammecr1l'	2831	2884	1964	916	2240	2245	2559.666667	1800.333333	-0.513486485	0.119931578	0.710102513
225341	'Lims2'	17	25	38	9	21	16	26.66666667	15.33333333	-0.841329211	0.214350046	0.84261066
225348	'Wdr36'	2588	2551	1831	650	1877	3906	2323.333333	2144.333333	-0.187538565	0.728265339	0.972790461
225358	'Fam13b'	2459.2	2550	2609.2	1949.1	2588.6	2541.24	2539.493333	2359.65	-0.065726114	0.821061933	0.972790461
225362	'Reep2'	174	210	216	353	498	491	200	447.3333333	1.18854961	3.12E-04	0.030714333
225363	'Etf1'	3683	3691	6120	5884	3211	4998	4498	4697.666667	0.157791819	0.776008425	0.972790461
225372	'Apbb3'	366	385	172	185	352	253	307.6666667	263.3333333	-0.15533305	0.697203885	0.972790461
225392	'Rell2'	117.68	130.66	27.86	30.33	91.9	78.65	92.06666667	66.96	-0.421125641	0.511590549	0.972790461
225432	'Rbm27'	1965	2006	1316	1351	1480	1553	1762.333333	1461.333333	-0.175608037	0.567203072	0.972790461
225467	'Pggt1b'	1142	1155	1263	670	1382	1346	1186.666667	1132.666667	-0.08642009	0.788692901	0.972790461
225471	'Ticam2'	22	20	51	6	12	17	31	11.66666667	-1.499316187	0.04807625	0.509206498
225518	'Prdm6'	38	30	17	0	25	19	28.33333333	14.66666667	-1.045180584	0.332800414	0.921648675
225523	'Cep120'	1201	1194	1912	1320	1212	1443	1435.666667	1325	-0.072812329	0.873562928	0.980171118
225579	'Slc27a6'	119	119	59	71	286	251	99	202.6666667	1.011277357	0.050004695	0.518369283
225583	'Minar2'	92	105	14	2	171	75	70.33333333	82.66666667	0.186865511	0.869934541	0.979794316
225594	'Gm4841'	12	13	10	3	14	11	11.66666667	9.333333333	-0.353737136	0.653682817	0.972790461
225600	'Pde6a'	1	3	1	0	4	9.34	1.666666667	4.446666667	1.272564502	0.450917196	0.970649024
225608	'Sh3tc2'	1171	1232	393	324	1598	1489	932	1137	0.266843461	0.657056384	0.972790461
225609	'Gm9949'	8	2	2	2	7	2	4	3.666666667	-0.07967459	0.950838805	0.998086758
225631	'Onecut2'	29	39	13	103	26	12	27	47	1.186608322	0.230169472	0.856721174
225638	'Alpk2'	17	25	1	3	16	16	14.33333333	11.66666667	-0.275250561	0.804602836	0.972790461
225642	'Grp'	0	0	0	1	0	0	0	0.333333333	1.020273531	0.802557913	0.972790461
225644	'Cplx4'	1	0	0	0	0	0	0.333333333	0	-0.903279821	0.824807108	0.972790461
225651	'Mppe1'	192.12	194.96	37.32	53.99	278.43	201.11	141.4666667	177.8433333	0.337979459	0.635283966	0.972790461
225655	'Prelid3a'	28	21	44	2	21	8	31	10.33333333	-1.701251857	0.051914185	0.529072762
225659	'Cep76'	270	286	418	441	237	318	324.6666667	332	0.152593023	0.786082829	0.972790461
225724	'Mapk4'	24	21	222	260	20	25	89	101.6666667	0.42934026	0.742749858	0.972790461
225742	'St8sia5'	1	3	3	2	0	0	2.333333333	0.666666667	-1.495780127	0.499072322	0.972790461
225743	'Rnf165'	41	21	221	19	12	10	94.33333333	13.66666667	-2.77575737	0.006429006	0.190013707
225745	'Haus1'	393	362	208	89	335	357	321	260.3333333	-0.335319843	0.492963924	0.972790461
225791	'Zadh2'	1294	1329	1052	1489	1421	1522	1225	1477.333333	0.370706803	0.269417895	0.883851074
225825	'Cd226'	0	1	1	0	4	0	0.666666667	1.333333333	0.858918255	0.78279937	0.972790461
225845	'Plaat3'	159	178	129	48	320	283	155.3333333	217	0.400966542	0.502932731	0.972790461
225849	'Ppp2r5b'	343	363	450	1427	565	631	385.3333333	874.3333333	1.383101231	0.030969564	0.427799512
225852	'Gm550'	3	2	1	0	0	5	2	1.666666667	-0.366431386	0.866770978	0.978391607
225861	'Snx32'	155	172	68	90	202	116	131.6666667	136	0.113782149	0.811350682	0.972790461
225870	'Rin1'	80	96	433	70	74	166	203	103.3333333	-1.099654207	0.178214256	0.797673555
225872	'Npas4'	20	23	12	1	13	1	18.33333333	5	-1.889201526	0.089232979	0.645546952
225875	'Lrfn4'	634	606	694	367	935	867	644.6666667	723	0.125324712	0.736154711	0.972790461
225876	'Kdm2a'	5188.7	4697	5891.4	2837.5	3773.1	3465.19	5259.023333	3358.59	-0.612340325	0.0702134	0.589465353

225884	'Gstp3'	1	2	1	4	2	1	1.333333333	2.333333333	1.02080256	0.526958234	0.972790461
225887	'Ndufs8'	412	448	343	419	510	563	401	497.3333333	0.372381862	0.172423862	0.790141656
225888	'Kmt5b'	2474	2506	2380	359	2569	1714	2453.333333	1547.333333	-0.757897041	0.197685469	0.823747311
22589	'Atrx'	4647	4859	2560	5826	7368	4944	4022	6046	0.710213151	0.073339766	0.599174173
225895	'Taf6l'	538	568	204	345	656	661	436.6666667	554	0.400439574	0.352157149	0.929748874
225896	'Ubxn1'	1502	1634	2752	1635	1645	2376	1962.666667	1885.333333	-0.054820004	0.907177134	0.988823459
225898	'Eml3'	1021	1101	444	659	1105	909	855.3333333	891	0.140531881	0.721826766	0.972790461
22590	'Xpa'	122	110	317	123	186	217	183	175.3333333	-0.130213922	0.830607865	0.974303846
225908	'Myrf'	2685	2369	1323	2548	2149	2519	2125.666667	2405.333333	0.317141909	0.435472444	0.96581102
22591	'Xpc'	774	769	630	361	826	1069	724.3333333	752	0.026050945	0.941813849	0.996253096
225912	'Cyb561a3'	436.7	507.26	214.94	325	601.71	662.6	386.3	529.77	0.500458974	0.188603541	0.810884334
225913	'Tkfc'	533	594	290	267	414	510	472.3333333	397	-0.201398544	0.555347381	0.972790461
22592	'Ercc5'	596	620	699	784	610	543	638.3333333	645.6666667	0.137574903	0.768231819	0.972790461
225929	'Patl1'	1765	1642	3350	1231	1398	1678	2252.333333	1435.666667	-0.656540257	0.18350747	0.804729813
22594	'Xrcc1'	971	936	830	310	905	1235	912.3333333	816.6666667	-0.218971173	0.61571531	0.972790461
22596	'Xrcc5'	1427	1373	605	143	1190	1264	1135	865.6666667	-0.457674991	0.501291754	0.972790461
22599	'Slc6a20b'	0	2	0	0	8	46.5	0.666666667	18.16666667	4.651510877	0.029228095	0.417580391
225994	'Nmrk1'	68	84	60	41.23	81.01	50.01	70.66666667	57.41666667	-0.260826553	0.539020181	0.972790461
225995	'D030056L22Rik'	611	567	414	357	499	558	530.6666667	471.3333333	-0.120811769	0.64086672	0.972790461
225997	'Trpm6'	60	67	32	95	58	39	53	64	0.501116153	0.441205675	0.968551476
225998	'Rorb'	10	9	24	33	3	2	14.33333333	12.66666667	0.141396135	0.913372582	0.989773583
22601	'Yap1'	3860	4093	5229	3511	4498	5203	4394	4404	0.016367627	0.963207579	0.999493374
226016	'Abhd17b'	540	527	893	856	686	827	653.3333333	789.6666667	0.332248025	0.503602297	0.972790461
226025	'Trpm3'	8	9	3	6	18	11	6.666666667	11.66666667	0.843220897	0.322122845	0.918054015
226026	'Smc5'	2232	2180	1577	1269	1994	1717	1996.333333	1660	-0.211480636	0.412595921	0.95722888
226040	'Tmem252'	5.51	7.23	7.3	6.79	13.58	2.59	6.68	7.653333333	0.198166132	0.8498469	0.975734242
226041	'Pgm5'	485.49	445.77	780.7	162.21	445.42	336.41	570.6533333	314.68	-0.926670514	0.057315054	0.54812566
226043	'Cbwd1'	660	652	578	213	415	516	630	381.3333333	-0.736777049	0.018198044	0.336230526
226049	'Dmrt2'	3	3	0	1	1	0	2	0.666666667	-1.337785331	0.565631739	0.972790461
226075	'Glis3'	336	300	319	460	166	332	318.3333333	319.3333333	0.177822863	0.76336841	0.972790461
22608	'Ybx1'	6729	7116	15402	5483	7146	8343	9749.003333	6990.666667	-0.513627234	0.326108795	0.920269559
226089	'Ric1'	1589	1575	1817	2189	1348	1453	1660.333333	1663.333333	0.144780019	0.772571505	0.972790461
226090	'Ermp1'	1511	1528	736	2249	1381	1512	1258.333333	1714	0.646466213	0.207460159	0.835068595
226098	'Hectd2'	303	304	470	250	212	105	359	189	-0.801810046	0.187438305	0.808617437
226101	'Myof'	1139	1051	1955	2378	1130	1492	1381.666667	1666.666667	0.398874481	0.517911109	0.972790461
226105	'Cyp2c70'	0	1	0	0	0	0	0.333333333	0	-0.903279821	0.824807108	0.972790461
226115	'Opalin'	0	0	0	0	1	0	0	0.333333333	1.020273531	0.802557913	0.972790461
22612	'Yes1'	1238.1	1280.4	1209.2	1094.1	1376.8	1448.42	1242.543333	1306.456667	0.120439815	0.662677904	0.972790461
226122	'Ubtd1'	185	184	268	190	204	223	212.3333333	205.6666667	-0.011155019	0.979248969	0.999493374
226123	'Morn4'	230	205	287	73	243	243	240.6666667	186.3333333	-0.449751199	0.352946166	0.930050349

226139	'Cox15'	840.51	867.75	568.34	212.1	794.57	913.27	758.8666667	639.98	-0.295247222	0.533134818	0.972790461
226143	'Cyp2c23'	0	2	1	0	4	0	1	1.333333333	0.320847321	0.914223619	0.989964366
226144	'Erlin1'	511	564	332	265	588	520	469	457.6666667	-0.014496157	0.964593797	0.999493374
226151	'Slf2'	3968	3973	3091	2979	4535	4209	3677.333333	3907.666667	0.135206535	0.564391129	0.972790461
226153	'Twnk'	1386	1360.9	818.22	939.49	983.37	1142.42	1188.37	1021.76	-0.119318637	0.715854524	0.972790461
226154	'Lzts2'	1789	1780	2295	1356.9	2186	2091	1954.666667	1877.963333	-0.058769897	0.864392187	0.977779046
226162	'Dpcd'	277	279	166	125	262	303	240.6666667	230	-0.054212866	0.877783423	0.981341203
226169	'Pprc1'	1225	1087	976	965	1009	1326	1096	1100	0.06915145	0.817265947	0.972790461
226178	'Wbp11'	1685	1545	2657	2891	2090	2844	1962.333333	2608.333333	0.475093464	0.344159234	0.925308103
226180	'Ina'	112.9	83.42	45.42	0	45.84	39.6	80.58	28.48	-1.61651137	0.135738426	0.736689633
226182	'Taf5'	1253	1224	458	333	893	1773	978.3333333	999.6666667	0.005856075	0.991948903	0.999493374
22619	'Siae'	651	657	61	796	1381	1433	456.3333333	1203.333333	1.505530678	0.041495359	0.47647028
226243	'Habp2'	1	3	0	0	4	4	1.333333333	2.666666667	0.96379378	0.645251314	0.972790461
226245	'Plekhs1'	5	7	1	26	8	9	4.333333333	14.33333333	2.036106078	0.064875617	0.572150983
22625	'Map3k19'	3	1	2	6	2	2	2	3.333333333	0.945902689	0.507767516	0.972790461
226250	'Afap1l2'	348	361	521	1344	557	456	410	785.6666667	1.14581297	0.096310824	0.662981155
226251	'Ablim1'	1759	1693	381	158	2940	2895	1277.666667	1997.666667	0.584374131	0.508163728	0.972790461
226252	'Fam160b1'	1288	1148	1482	1305	1413	1356	1306	1358	0.115435858	0.759984765	0.972790461
226255	'Atrnl1'	2301	1973	950	1586	1994	2151	1741.333333	1910.333333	0.229439636	0.542688591	0.972790461
22626	'Slc23a3'	14	10	13	32	13	12	12.33333333	19	0.830551511	0.322724269	0.918775094
226265	'Eno4'	167	195	32	13	143	55	131.3333333	70.33333333	-0.907539566	0.291714413	0.89958198
22627	'Ywhae'	8485	8610	13173	4376	8023	9709	10089.33333	7369.333333	-0.503026076	0.227981274	0.85440173
226278	'Prlhr'	0	0	0	2	0	0	0	0.666666667	2.299096387	0.566358544	0.972790461
22628	'Ywhag'	3588	3689	7228	6190	3810	5707	4835	5235.666667	0.179703829	0.753346751	0.972790461
22629	'Ywhah'	2671	2746	4959	3219	3441	3919	3458.666667	3526.333333	0.040841287	0.931675767	0.993601701
22630	'Ywhaq'	4363.4	4692.3	5197.2	2714.4	4889.1	4985.48	4750.956667	4196.303333	-0.189354597	0.54521065	0.972790461
226304	'Npbwr1'	39	27	3	1	30	22	23	17.66666667	-0.413628492	0.721025791	0.972790461
22631	'Ywhaz'	8298	8828	12848	4662	8192	9483	9991.333333	7445.666667	-0.463220255	0.248448773	0.869491261
22632	'Yy1'	2560	2506	4278	4017	2761	3199	3114.666667	3325.666667	0.180911194	0.73291218	0.972790461
22634	'Plagl1'	1777	1551	987	663	1918	898	1438.333333	1159.666667	-0.282000475	0.527769026	0.972790461
22635	'Zan'	47	49	7	6	51.75	8	34.33333333	21.91666667	-0.63298263	0.533241402	0.972790461
226351	'Tmem185b'	269	273	233	365	387	414	258.3333333	388.6666667	0.667843263	0.037213367	0.459930654
226352	'Epb41l5'	2317	2345	850	1041	1765	1874	1837.333333	1560	-0.168140586	0.690111942	0.972790461
226356	'Cfap221'	0	5	1	3	2	2	2	2.333333333	0.396937906	0.819638576	0.972790461
226359	'C1ql2'	0	1	0	0	0	0	0.333333333	0	-0.903279821	0.824807108	0.972790461
22637	'Zap70'	11	6	13	1	5	3	10	3	-1.794514047	0.085968269	0.636212038
22640	'Zfp1'	430	386	532	185	385	408	449.3333333	326	-0.501759234	0.19510228	0.819942118
226407	'Rab3gap1'	1373	1352	1017	1400	1293	1292	1247.333333	1328.333333	0.207318633	0.564225587	0.972790461
226409	'Zranb3'	397	404	114.03	135	470	345	305.01	316.6666667	0.079421391	0.888705987	0.984710124
226412	'R3hdm1'	3049	3017	3425	1481	2545	2448	3163.666667	2158	-0.550716055	0.069834014	0.588251779

226413	'Lct'	45	33	13	2	27	20	30.33333333	16.33333333	-0.935800613	0.285050586	0.893711786
226414	'Dars'	2720	2679	2435	926	2445	3032	2611.333333	2134.333333	-0.338100542	0.381701071	0.94429001
226418	'Yod1'	237	234	105	217	174	134	192	175	0.054552919	0.917433855	0.990547571
226419	'Dyrk3'	64	91	107	211	82	121	87.33333333	138	0.829418691	0.206217845	0.833294914
22642	'Zbtb17'	643	697	512	935	646	691	617.3333333	757.3333333	0.449380132	0.314354707	0.914150085
226421	'Rab7b'	149	143	206	61	187	238	166	162	-0.127915511	0.803536737	0.972790461
226422	'Rab29'	122	107	67	64	103	122	98.66666667	96.33333333	0.004741698	0.989576444	0.999493374
22643	'Zfp101'	374	349	170	127	428	206	297.6666667	253.6666667	-0.2051385	0.68311042	0.972790461
226432	'Ipo9'	3000	3013	2285	2177	3156	2656	2766	2663	0.008566344	0.97441128	0.999493374
226438	'Igfn1'	41	46	0	0	42	20	29	20.66666667	-0.5159409	0.762058453	0.972790461
226439	'Ascl5'	0	0	0	1	0	0	0	0.333333333	1.020273531	0.802557913	0.972790461
22644	'Rnf103'	941	977	546	287	983	942	821.3333333	737.3333333	-0.178113812	0.686304455	0.972790461
226442	'Zfp281'	1275	1364	888	1142	1235	1250	1175.666667	1209	0.138949415	0.660644854	0.972790461
22646	'Zfp105'	603	629	589	101	476	455	607	344	-0.900383975	0.078180947	0.614954936
226470	'Zbtb41'	1236	1217	1004	1642	1460	1352	1152.333333	1484.666667	0.488239884	0.205897393	0.833294914
22648	'Zfp11'	186	188	130	66	204	147	168	139	-0.280186214	0.496618909	0.972790461
226499	'Odr4'	917.91	997.91	1149.2	211	846.81	1032	1021.666667	696.6033333	-0.653644039	0.210867839	0.840289112
226517	'Smg7'	3981	3924	1990	1725	2814	2492	3298.333333	2343.666667	-0.423181334	0.203010637	0.83089164
226518	'Nmnat2'	327	381	24	24	251	211	244	162	-0.603612573	0.530482528	0.972790461
226519	'Lamc1'	4338	3817	2350	9343	5871	5903	3501.666667	7039	1.201696292	0.016119737	0.316599625
22652	'Mkrn3'	145	140	65	62	155	137	116.6666667	118	0.042050079	0.923827475	0.991932584
226525	'Rasal2'	1898	1768	423	682	964	946.01	1363	864.0033333	-0.54163002	0.319749031	0.916646777
226527	'Cryzl2'	400.02	465.69	293.78	137.62	584.2	514.88	386.4966667	412.2333333	0.047476822	0.921631607	0.991066749
226539	'Dars2'	603	599	200	149	480	456	467.3333333	361.6666667	-0.358246925	0.495963059	0.972790461
22654	'Zfp13'	492	468	392	342	389	419	450.6666667	383.3333333	-0.162481898	0.579183121	0.972790461
226541	'Klhl20'	1037	1118	402	370	794	727	852.3333333	630.3333333	-0.385557009	0.375869469	0.940406075
226548	'Aph1a'	1266.2	1430.8	1919.6	1670.3	1460.9	1869.21	1538.89	1666.8	0.168642386	0.697317121	0.972790461
226551	'Suco'	2011	2136	1957	1685	2169	2128	2034.666667	1994	0.023193909	0.932945511	0.994012556
226562	'Prrc2c'	10217	9795	5924	6284	8521	8003	8645.333333	7602.666667	-0.104749623	0.722551577	0.972790461
226564	'Fmo4'	7	8	9	1	2	1	8	1.333333333	-2.567448974	0.032807181	0.436246529
226565	'Fmo6'	1	0	0	1	0	0	0.333333333	0.333333333	0.058500858	0.988561293	0.999493374
22658	'Pcgf2'	663	641	1734	424	745	745	1012.666667	638	-0.741279042	0.203826981	0.832079769
226591	'Tiprl'	845.19	839	1627	1084	953	944	1103.73	993.6666667	-0.101045657	0.849445811	0.975734242
226594	'Rcsd1'	662	601	688	197	680	584	650.3333333	487	-0.479501351	0.273911569	0.885195775
226601	'Gm4846'	0	0	0	0	0	1	0	0.333333333	1.020273531	0.802557913	0.972790461
22661	'Zfp148'	2441	2550	1863	2484	2972	2719	2284.666667	2725	0.339752209	0.248048614	0.869491261
226610	'Fam78b'	241	231	128	90	251	165	200	168.6666667	-0.223722887	0.596896311	0.972790461
226641	'Atf6'	1133	1040	1764	6154	1355	2310	1312.333333	3273	1.548227192	0.044309482	0.490480355
226646	'Ndufs2'	2740	2979	2034	3245	3265	4109	2584.333333	3539.666667	0.540403674	0.076791231	0.609792246
226652	'Arhgap30'	36	41	4	15	37	41	27	31	0.258102449	0.754698583	0.972790461

226654	'Tstd1'	2	7	2	2	2	4	3.666666667	2.666666667	-0.395626058	0.760059391	0.972790461
22666	'Zbtb14'	907	865	745	132	682	757	839	523.6666667	-0.765914598	0.16038434	0.771114735
22668	'Sf1'	3653	3659	4201	3367	3927	3685	3837.666667	3659.666667	-0.013588994	0.96864226	0.999493374
226691	'Ifi207'	7	4	27.39	3	7	14	12.79666667	8	-0.836534032	0.444487046	0.969888068
226695	'Ifi205'	2	2.01	31.14	0	2	1.21	11.71666667	1.07	-3.787108711	0.029758753	0.420649375
22670	'Trim26'	1623	1676	911	754	1644	1461	1403.333333	1286.333333	-0.096911614	0.772664221	0.972790461
22671	'Rnf112'	9	10	4	1	10	2	7.666666667	4.333333333	-0.824138799	0.478859958	0.972790461
226720	'Becn2'	5	0	1	0	1	2	2	1	-1.071035652	0.645713607	0.972790461
22673	'Zfp185'	375	439	337	106	441	390	383.6666667	312.3333333	-0.353686396	0.455053484	0.970934082
226744	'Cnst'	465	467	473	458	615	568	468.3333333	547	0.269443958	0.372474928	0.938816563
226747	'Ahctf1'	2837	3024	1820	1644	2572	2530	2560.333333	2248.666667	-0.132497368	0.619415467	0.972790461
226751	'Cdc42bpa'	2957.3	3041.8	1483.5	1405.8	2499	2617	2494.193333	2173.936667	-0.150783247	0.65498146	0.972790461
226757	'Wdr26'	4721	4720	4993	5192	4556	5364	4811.333333	5037.33	0.15052118	0.685346339	0.972790461
226777	'C130074G19Rik'	1147	1145	249	125	1278	915	847	772.6666667	-0.164499841	0.832691884	0.974723675
226778	'Mark1'	1237	1134	825	830	1083	1040	1065.333333	984.3333333	-0.041120256	0.881510441	0.982528614
22678	'Zfp2'	437	456	236	217	450	288	376.3333333	318.3333333	-0.183465138	0.634031718	0.972790461
226781	'Slc30a10'	28	32	61	1	11	1	40.33333333	4.333333333	-3.315669355	0.001283624	0.075554997
226791	'Lyplal1'	256	241	271	31	282	364	256	225.6666667	-0.31906035	0.652825295	0.972790461
22680	'Zfp207'	3417	3700	4584	1882	3494	3144	3900.333333	2840	-0.47170446	0.180945476	0.801479605
22682	'Zfand5'	2835.2	2859.9	5736.3	12588	2783.1	3988.02	3810.463333	6453.12	0.987244561	0.22441214	0.850759465
226823	'Kctd3'	2491	2393	2042	1069	1834	2281	2308.666667	1728	-0.413623936	0.123557696	0.715310266
226830	'Smyd2'	949	896	1338	253	973	1056	1061	760.6666667	-0.587986275	0.263966899	0.880205046
226841	'Vash2'	483	501	362	148	375	177	448.6666667	233.3333333	-0.912997742	0.030984637	0.427799512
226844	'Flvcr1'	318	306	192	421	371	289	272	360.3333333	0.561270988	0.211825436	0.840560909
226849	'Ppp2r5a'	1334	1302.1	1656.2	1388	1485	2101	1430.766667	1657.973333	0.235783663	0.520148395	0.972790461
22685	'Zfp239'	148	162	36	64	101	123	115.3333333	96	-0.181956171	0.751931276	0.972790461
226856	'Lpgat1'	1884	1956	1101	609	1880	1464	1647	1317.666667	-0.323787587	0.421132079	0.958984679
226861	'Hhat'	117	121	80	50	148	170	106	122.6666667	0.18364397	0.678753184	0.972790461
226866	'Sbspon'	67	69	3	62	75	106	46.33333333	81	0.938369757	0.29260254	0.900043079
22687	'Zpr1'	594	691	999	730	535	738	761.3333333	667.6666667	-0.123517808	0.801203281	0.972790461
22688	'Zfp26'	1646	1708	985	486	1691	1493	1446.333333	1223.333333	-0.262988981	0.543394523	0.972790461
22689	'Zfp27'	298	328	390	172	363	363	338.6666667	299.3333333	-0.209830658	0.575360023	0.972790461
226896	'Tfap2d'	163.07	171.17	69.82	110.63	143.3	79.05	134.6866667	110.9933333	-0.139059702	0.786856558	0.972790461
22690	'Zfp28'	435	446	592	119	442	337	491	299.3333333	-0.791989488	0.104099993	0.676400911
22691	'Zscan2'	468	470.55	495	308	448	396.99	477.85	384.33	-0.280129254	0.366044775	0.936406787
226922	'Kcnq5'	25	12	59	14	6	14	32	11.33333333	-1.474900155	0.104647417	0.67684333
22693	'Zfp30'	415	345	372	83	377	355	377.3333333	271.6666667	-0.553614725	0.277922913	0.889355605
22694	'Zfp35'	507	577	346	338	519	496	476.6666667	451	-0.022660419	0.935927951	0.994413066
22695	'Zfp36'	209	169	1935	1141	217	306	771	554.6666667	-0.334478668	0.767430984	0.972790461
22696	'Zfp37'	294	268	259	112	377	269	273.6666667	252.6666667	-0.150951976	0.721011804	0.972790461

22697	'Zscan21'	811	865	711	436	906	799	795.6666667	713.6666667	-0.150005069	0.595340118	0.972790461
226970	'Arhgef4'	226	219	501	202	425	421	315.3333333	349.3333333	0.066976401	0.902655978	0.987331639
226971	'Plekhb2'	432	391	591	2491	520	1396	471.3333333	1469	1.839302518	0.011996284	0.271043001
226976	'Kansl3'	3637	3533	2417	2017	3000	2639	3195.666667	2552	-0.260004602	0.329426849	0.921648675
226977	'Actr1b'	1422	1495	2301	2702	1648	2044	1739.333333	2131.333333	0.397988737	0.459192355	0.972790461
22698	'Zfp39'	298	267	258	36	234	249	274.3333333	173	-0.765515959	0.205733027	0.833294914
226982	'Eif5b'	4031.1	3823.3	4527.6	1737.4	3194.7	4009.18	4127.346667	2980.443333	-0.499063386	0.143101837	0.749054337
226999	'Slc9a2'	10	11	6	17	2	11	9	10	0.387557921	0.709972576	0.972790461
22700	'Zfp40'	450	499	254	273	694	456	401	474.3333333	0.274617645	0.488893179	0.972790461
22701	'Zfp41'	1167	1133	244	349	1285	928	848	854	0.041355574	0.94740628	0.997071032
22702	'Zfp42'	147	115	22	3	72	141	94.66666667	72	-0.467313527	0.654606422	0.972790461
22704	'Zfp46'	926	957	956	921	1214	828	946.3333333	987.6666667	0.132507333	0.718823122	0.972790461
227058	'Dnah7b'	1026.3	1085.5	115.91	53.62	854.56	520.48	742.5733333	476.22	-0.666345704	0.475899726	0.972790461
227059	'Slc39a10'	1982	1978	1264	1813	2232	2124	1741.333333	2056.333333	0.328219023	0.270296427	0.883851074
22709	'Zfp51'	266	296	325	273	311	292	295.6666667	292	0.039248566	0.91562786	0.990380433
227094	'Nemp2'	282	242	120	441	351	256	214.6666667	349.3333333	0.896184866	0.091017358	0.649965349
227095	'Hibch'	418	453	326	591	548	591	399	576.6666667	0.63966333	0.070536171	0.589465353
227099	'Pms1'	251	231	133	58	262	217	205	179	-0.228650728	0.658816598	0.972790461
22710	'Zfp52'	209	263	586	523	285	310	352.6666667	372.6666667	0.177368567	0.795663836	0.972790461
227102	'Ormdl1'	428	471	220	321	581	643	373	515	0.506886673	0.155469294	0.766717687
22712	'Zfp54'	75	86	80	64	126	90	80.33333333	93.33333333	0.234283997	0.544879232	0.972790461
227120	'Plcl1'	246	197	149	133	236	194	197.3333333	187.6666667	-0.027308892	0.933382081	0.994078204
22715	'Zfp57'	668.58	585.77	2289	634	681	809	1181.116667	708	-0.784848091	0.253024065	0.87178208
227154	'Stradb'	955	967	445	187	760	961	789	636	-0.3503368	0.519913036	0.972790461
227157	'Mpp4'	42.63	30.79	7	4	20.22	9.35	26.80666667	11.19	-1.221264618	0.165483775	0.78006879
22717	'Zfp59'	439.03	472.99	278	98	463	393	396.6733333	318	-0.361874343	0.478901987	0.972790461
22718	'Zfp60'	1042	1055	598	436	1087	922	898.3233333	815	-0.124849468	0.726831603	0.972790461
22719	'Zfp61'	505	571	359	46	588	451	478.3333333	361.6666667	-0.498879426	0.487955979	0.972790461
227195	'Ino80d'	1261	1219	1043	980	1417	1115	1174.333333	1170.666667	0.056653804	0.845750469	0.975182082
227197	'Ndufs1'	2231	2332	1185	958	2131	2238	1916	1775.666667	-0.091149793	0.800293713	0.972790461
22720	'Zfp62'	2009	2004	1500	1398	2013	1520	1837.666667	1643.666667	-0.087683014	0.770801268	0.972790461
227210	'Ccnyl1'	1557	1495	737	577	907	761	1263	748.3333333	-0.674450735	0.057698159	0.549302654
22722	'Zfp64'	270	293	471	191	357	369	344.6666667	305.6666667	-0.218184717	0.627160371	0.972790461
227231	'Cps1'	91	62	7	0	3	2	53.33333333	1.666666667	-4.961960215	3.40E-05	0.006398791
22724	'Zbtb7b'	386	360	2047	2171	413	1046	931	1210	0.495489381	0.610288496	0.972790461
227290	'Aamp'	2482	2737	3713	2363	2843	3623	2977.333333	2943	-0.009826589	0.979775241	0.999493374
227292	'Ctdsp1'	1709	1715	1837	774	1941	2128	1753.666667	1614.333333	-0.166286883	0.654637366	0.972790461
227298	'Retreg2'	1387	1374	1112	1083	1750	2370	1291	1734.333333	0.433237028	0.120839277	0.712100232
227325	'Dner'	29	18	6	2	23	48	17.66666667	24.33333333	0.39035212	0.701828046	0.972790461
227326	'Gpr55'	169	169	99	649	58	184	145.6666667	297	1.391488127	0.128872129	0.72577634

227327	'B3gnt7'	128	148	63	34	217	222	113	157.6666667	0.427311775	0.505156994	0.972790461
227331	'Gigyf2'	3888	3733	1889	6489	3538	3448	3170	4491.666667	0.731839723	0.191254437	0.813837363
227333	'Dgkd'	3608	3313	1759	778	2609	3152	2893.333333	2179.666667	-0.437174985	0.36051707	0.933706718
227334	'Usp40'	1522	1582	547	540	1779	1361	1217	1226.666667	0.030264233	0.952253835	0.998689867
227357	'Espnl'	28	22	7	8	16	18	19	14	-0.387026301	0.592352288	0.972790461
227358	'Erfe'	21.32	6.25	7.71	109.94	8.28	82.62	11.76	66.94666667	2.795830138	0.007120761	0.199970326
227377	'Farp2'	253.85	246.32	148.26	115.89	162.03	161.1	216.1433333	146.34	-0.494945932	0.12452668	0.717642086
227394	'Slco4c1'	1	4	0	2	0	1	1.666666667	1	-0.405872044	0.863649624	0.977452409
227399	'Ppip5k2'	2779	2943	1132	1190	2667	2509	2284.666667	2122	-0.066546913	0.874486261	0.980175262
227446	'Relch'	1852	1877	795	787	1355	1197	1508	1113	-0.366144318	0.337742493	0.924020038
227449	'Zcchc2'	1074	1055	499	1541	767	1247	876	1185	0.632079714	0.237439697	0.86298394
22746	'Zfp85'	171.1	179.79	122.26	159	193	132	157.7166667	161.3333333	0.142450267	0.721189184	0.972790461
227485	'Cdh19'	7	0	0	1	2	2	2.333333333	1.666666667	-0.393141669	0.8591034	0.976863639
22750	'Zfp9'	359	319.01	2570.6	221	305	426.99	1082.863333	317.6633333	-1.912627019	0.030605113	0.425756906
22751	'Zfp90'	401	405	373	222	542	341	393	368.3333333	-0.090357332	0.804488679	0.972790461
227522	'Rpp38'	268.24	325.09	350.67	42.11	194.09	192.65	314.6666667	142.95	-1.234302367	0.021821409	0.365836629
227525	'Dclre1c'	530.68	508.94	208.07	410.68	473.5	326.29	415.8966667	403.49	0.104700657	0.825366991	0.972790461
227526	'Cdnf'	123	116	38	6	52	41	92.33333333	33	-1.515952643	0.040457797	0.472434971
22754	'Zfp92'	13	6	9	3	3	4	9.333333333	3.333333333	-1.440061512	0.136165196	0.736689633
227541	'Camk1d'	57	32	399	74	41	45	162.6666667	53.33333333	-1.601578262	0.115950159	0.70293889
227545	'Proser2'	101	70	543	110	74	100	238	94.66666667	-1.354841962	0.128552266	0.725663802
22755	'Zfp93'	289	320	148	120	277	235	252.3333333	210.6666667	-0.22689313	0.56565527	0.972790461
22756	'Zfp94'	130	110	76	30	92	119	105.3333333	80.33333333	-0.422870742	0.385429653	0.946987408
22757	'Zkscan5'	547	505	455	468	504	565	502.3333333	512.3333333	0.100196485	0.74254609	0.972790461
22758	'Zscan12'	562	592	441	404	472	502	531.6666667	459.3333333	-0.137631044	0.6279991	0.972790461
227580	'C1ql3'	6.34	7.83	4.61	0	10.69	1.17	6.26	3.953333333	-0.694362386	0.643287498	0.972790461
22759	'Zfp97'	206.59	242.12	400.77	101.85	194.96	254.98	283.16	183.93	-0.697559179	0.160566023	0.771114735
227606	'Tbpl2'	0	0	0	0	3	11	0	4.666666667	4.497441735	0.134352776	0.733998556
22761	'Zfpm1'	416	456	368	516	493	571	413.3333333	526.6666667	0.43846169	0.191477996	0.813837363
227612	'Tor4a'	443	531	218	395	709	804	397.3333333	636	0.724385639	0.05909301	0.552865869
227613	'Tubb4b'	3420	3530	1893	1513	2741	4826	2947.666667	3026.666667	0.036617056	0.929719237	0.993164002
227615	'Tmem203'	159	147	70	133	182	254	125.3333333	189.6666667	0.656386398	0.101372568	0.671759705
227619	'Man1b1'	1735	1735	1207	1624	2102	1888	1559	1871.333333	0.343552803	0.227463959	0.854007746
22762	'Zfpm2'	1026	1132	1671	572	1140	942	1276.333333	884.6666667	-0.560338446	0.184218048	0.804780995
227620	'Uap1l1'	287	350	1550	879	465	859	729	734.3333333	0.008622241	0.991594881	0.999493374
227622	'Paxx'	64	65	288	112	79	120	139	103.6666667	-0.444053536	0.576290524	0.972790461
227624	'Rab16'	1783	1833	1712	1468	2091	1909	1776	1822.666667	0.081057594	0.763720483	0.972790461
227627	'Obp2a'	1	0	0	0	0	0	0.333333333	0	-0.903279821	0.824807108	0.972790461
22763	'Zfr'	3832	4055	4886	4888	4224	5269	4257.666667	4793.666667	0.239920798	0.549936276	0.972790461
227631	'Sohlh1'	235	213	19	13	346	1366	155.6666667	575	1.807468897	0.129999839	0.727578716

227632	'Kcnt1'	1826	1904	275	285	4989	1446	1335	2240	0.73006233	0.430910495	0.963403879
227634	'Camsap1'	2759	2737	1776	1135	2151	1900	2424	1728.666667	-0.451997092	0.103123755	0.673510422
227638	'Qsox2'	663	649	265	976	623	598	525.6666667	732.3333333	0.693406348	0.207761508	0.835213465
22764	'Zfx'	2350	2497	1828	1305	2111	1739	2225	1718.333333	-0.322114563	0.218108402	0.844365088
227644	'Snapc4'	778	730	595	517	797	496	701	603.3333333	-0.14386029	0.682821075	0.972790461
227648	'Sec16a'	3512	3352	3962	1956	3083	3232	3608.666667	2757	-0.384646998	0.209608831	0.837080112
227656	'Rexo4'	870	931	707	634	831	749	836	738	-0.110483526	0.691747322	0.972790461
227659	'Slc2a6'	6	3	30	1	1	9	13	3.666666667	-2.049861522	0.138376514	0.739741822
227671	'Gbgt1'	31	15	2	2	42	18	16	20.66666667	0.350064278	0.763845984	0.972790461
227674	'Ddx31'	266	250.79	206	83	264	252.97	240.93	199.99	-0.304835975	0.46012158	0.972790461
227682	'Trub2'	551	598	294	232	520	543.66	481	431.8866667	-0.136476097	0.715154866	0.972790461
227683	'Coq4'	296.94	252.01	158	97	245	242.34	235.65	194.78	-0.269227046	0.489048172	0.972790461
227693	'Zer1'	1155	1155	591	406	1127	1172	967	901.6666667	-0.103337323	0.802521642	0.972790461
227695	'Spout1'	548.26	522.84	507.65	182.27	527.07	610.18	526.25	439.84	-0.310961459	0.447947078	0.970198456
227696	'Phyhd1'	292	307	374	20	344	346	324.3333333	236.6666667	-0.612422203	0.447392461	0.970198456
227697	'Dolk'	351	404	139	276	394	407	298	359	0.359852545	0.402835009	0.955996639
227699	'Nup188'	2704	2592	1462	947	1936	1938	2252.666667	1607	-0.459684942	0.159310622	0.770494417
22770	'Zhx1'	2487	2503	1039	689	1890	1619	2009.666667	1399.333333	-0.50029185	0.253943106	0.873052317
227700	'Sh3glb2'	669	695	336	361	691	864	566.6666667	638.6666667	0.198074019	0.595926398	0.972790461
227707	'BC005624'	668	695	627	490	621	796	663.3333333	635.6666667	-0.027902749	0.918643603	0.990815958
22771	'Zic1'	1	0	1	0	1	0	0.666666667	0.333333333	-0.894008961	0.803817041	0.972790461
227715	'Exosc2'	457	514	534	125	407	441	501.6666667	324.3333333	-0.699185177	0.119068513	0.709078602
227717	'Qrfp'	2.02	1.01	4.76	2	6	1	2.596666667	3	0.331896254	0.82170741	0.972790461
22772	'Zic2'	36	51	33	0	13	9	40	7.333333333	-2.534478591	0.00488282	0.165356847
227720	'Nup214'	1718	1621	782	656	1323	1247	1373.666667	1075.333333	-0.310473861	0.39714164	0.954425086
227721	'Plpp7'	58	82	69	95	105	137	69.66666667	112.3333333	0.735500225	0.059235629	0.552865869
227723	'Prrc2b'	7428	7091	5056	6232	7771	7161	6525	7054.666667	0.196149053	0.493053401	0.972790461
22773	'Zic3'	23	26	9	7	18	7	19.33333333	10.66666667	-0.784591216	0.306501306	0.907713832
227731	'Slc25a25'	488.26	433.81	406.75	796.54	293.59	298.63	442.94	462.92	0.311236849	0.635628759	0.972790461
227733	'Pip5kl1'	2	2	1	6	1	0	1.666666667	2.333333333	0.875419138	0.641457059	0.972790461
227736	'Cfap157'	5	3	3	4	8	3	3.666666667	5	0.507451991	0.643673683	0.972790461
227737	'Niban2'	741	714	2257	3114	880	1354	1237.333333	1782.666667	0.676936187	0.410574623	0.95722888
227738	'Lrsam1'	536	580	399	197	608	583	505	462.6666667	-0.153410753	0.699090272	0.972790461
22774	'Zic4'	18	13	2	2	12	5	11	6.333333333	-0.758700861	0.488901707	0.972790461
227743	'Mapkap1'	1335	1408	611	973	1516	1564	1118	1351	0.343968011	0.343184397	0.925308103
227746	'Rabepk'	357.95	361.09	347.5	228.78	400.32	456.62	355.5133333	361.9066667	0.025018613	0.931754875	0.993601701
22775	'Zik1'	292	308	163	103	270	216	254.3333333	196.3333333	-0.355745642	0.36711086	0.937098773
227753	'Gsn'	1917	1972	1375	5839	2771	3605	1754.666667	4071.666667	1.416952699	0.008151733	0.218179228
22776	'Zim1'	3066	2938	105	1275	2080	460	2036.333333	1271.666667	-0.467895934	0.639877066	0.972790461
22778	'Ikzf1'	219	212	22	5	82	64	151	50.33333333	-1.611826463	0.098026366	0.665361397

22779	'Ikzf2'	128	158	192	70	194	149	159.3333333	137.6666667	-0.258606217	0.570822633	0.972790461
22780	'Ikzf3'	4	4	4	4	1	1	4	2	-0.791073457	0.562618173	0.972790461
227800	'Rabgap1'	2985.9	2887	2132.6	2307.6	2705.9	3172.9	2668.483333	2728.773333	0.101183964	0.702888784	0.972790461
227801	'Dennd1a'	1288	1281	745	669	1032	1126	1104.666667	942.3333333	-0.176962566	0.538841095	0.972790461
22781	'Ikzf4'	182	155	234	62	141	133	190.3333333	112	-0.808043127	0.062915798	0.566357744
22782	'Slc30a1'	591	616	299	189	655	732	502	525.3333333	0.04155003	0.93146757	0.993601701
227835	'Gtdc1'	376.54	393.63	455.01	484.18	310.07	305.67	408.3933333	366.64	-0.014827816	0.977011049	0.999493374
22784	'Slc30a3'	6	5	6	6	14	95	5.666666667	38.33333333	2.614170994	0.012572914	0.277949116
22785	'Slc30a4'	1532	1441	1529	4149	1840	2064	1500.666667	2684.333333	1.038776122	0.075751793	0.605637955
22786	'Zp1'	1	0	0	0	1	96	0.333333333	32.33333333	6.304241559	0.007663869	0.210557046
227867	'Epc2'	1201	1190	1887	509	1205	912	1426	875.3333333	-0.751899394	0.091242646	0.649965349
22787	'Zp2'	1	2	0	1	106	545	1	217.3333333	7.649262916	2.73E-06	9.17E-04
22788	'Zp3'	1	0	0	1	14	163	0.333333333	59.33333333	7.190272982	1.62E-04	0.019471673
22789	'Zp3r'	0	0	1	3	0	0	0.333333333	1	1.937497013	0.625951132	0.972790461
22791	'Dnajc2'	1627	1599	1262	523	1147	1807	1496	1159	-0.403865097	0.307376114	0.907830798
227929	'Cytip'	20	15	13	8	14	24	16	15.33333333	-0.065175378	0.920405408	0.991018506
22793	'Zyx'	1980	1836	3644	3367	2061	2505	2486.666667	2644.333333	0.17869804	0.757828093	0.972790461
227933	'Ccdc148'	55	57	10	28	45	33	40.66666667	35.33333333	-0.081557533	0.906543886	0.988823459
227937	'Pkp4'	1622	1438	731	995	1232	1281	1263.666667	1169.333333	-0.014877025	0.967101623	0.999493374
227960	'Gca'	144	143	204	253	137	151	163.6666667	180.3333333	0.281399019	0.62968539	0.972790461
227998	'4933409G03Rik'	1	1	1	0	0	0	1	0	-2.390783043	0.494152846	0.972790461
228003	'Klhl41'	6	5	3	1	4	8	4.666666667	4.333333333	-0.147043966	0.897786002	0.985794067
228005	'Ppig'	2483	2510	2272	1267	2535	2463	2421.666667	2088.333333	-0.214954239	0.432509689	0.964333788
228012	'Tlk1'	2708	2505	2231	547	2135	2414	2481.333333	1698.666667	-0.617158034	0.194103462	0.818065845
228019	'Mettl8'	429.56	456.92	131.56	192.04	430.55	376.66	339.3466667	333.0833333	0.03206441	0.949252932	0.997777885
228026	'Pdk1'	2816	2288	5959	732	1705	2669	3687.666667	1702	-1.244682607	0.038291536	0.459930654
228033	'Atp5g3'	3211	3503	3188	3021	3224	4497	3300.666667	3580.666667	0.16503224	0.584868372	0.972790461
228061	'Agps'	2200	2286	1469	1590	1935	1741	1985	1755.333333	-0.084396792	0.783755938	0.972790461
228071	'Sestd1'	1594	1668	1050	1480	1549	1244	1437.333333	1424.333333	0.11257602	0.765318046	0.972790461
228094	'Cerkl'	4	4	0	1	5	6	2.666666667	4	0.601539285	0.691370032	0.972790461
228136	'Zdhhc5'	1674.7	1649.3	1674.9	1525	1759.5	2073.01	1666.293333	1785.86	0.147468095	0.617273136	0.972790461
228139	'P2rx3'	297	311	160	3	132	80	256	71.66666667	-1.932197779	0.034330179	0.445167916
228140	'Tnks1bp1'	2886	2837	3238	2228	2960	3021	2987	2736.333333	-0.095952565	0.756427086	0.972790461
228151	'Fads2b'	0	0	0	0	0	2	0	0.666666667	1.749449488	0.66566965	0.972790461
228355	'Madd'	758	717	216	247	742	451	563.6666667	480	-0.183807824	0.736233674	0.972790461
228356	'1110051M20Rik'	559.07	508.58	248.27	67.62	471.82	371.05	438.64	303.4966667	-0.582796754	0.349065428	0.928823768
228357	'Lrp4'	463	488	178	389	570	480	376.3333333	479.6666667	0.45272421	0.294260224	0.90029104
228359	'Arhgap1'	2191	2438	2203	1668	2613	2657	2277.333333	2312.666667	0.044600969	0.860523819	0.976863639
228361	'Ambra1'	2059	1979	1167	1260	1883	1676	1735	1606.333333	-0.038536493	0.897920421	0.985794067
228366	'Large2'	159	204	11	40	242	164	124.6666667	148.6666667	0.27688099	0.763350828	0.972790461

228368	'Slc35c1'	208	235	237	318	299	351	226.6666667	322.6666667	0.581987175	0.12091923	0.712100232
228410	'Cstf3'	1481	1521	789	461	999	691	1263.666667	717	-0.768966329	0.036272875	0.456985794
228413	'Prrg4'	56	52	158	860	110	261	88.66666667	410.3333333	2.43464025	0.010801918	0.256475153
228421	'Kif18a'	519	493	161	162	271	210	391	214.3333333	-0.7693638	0.111330258	0.695338656
228432	'Ano3'	2	6	13	1	9	8	7	6	-0.391617534	0.745240202	0.972790461
228482	'Arhgap11a'	771	730	383	419	575	399	628	464.3333333	-0.326260219	0.407147871	0.957157474
228491	'Zfp770'	881	904	445	314	598	574	743.3333333	495.3333333	-0.540453363	0.119695976	0.710007992
228536	'Bahd1'	1456	1341	1359	1237	1171	1613	1385.333333	1340.333333	0.016205874	0.961293246	0.999493374
228543	'Rhov'	151	116	264	38	81	95	177	71.33333333	-1.391293119	0.011717894	0.268550885
228545	'Vps18'	540	477	850	545	669	838	622.3333333	684	0.128489495	0.772019912	0.972790461
228550	'Itpka'	84	98	64	44	65	69	82	59.33333333	-0.420304894	0.240733284	0.86449675
228564	'Frmd5'	109	96	209	477	225	182	138	294.6666667	1.255606672	0.080185529	0.617305615
228576	'Mall'	7	9	2	7	35	25	6	22.33333333	1.891669067	0.030218373	0.422745916
228592	'F830045P16Rik'	0	1	0	0	1	0	0.333333333	0.333333333	0.058500858	0.988561293	0.999493374
228598	'Ebf4'	491	436	288	306	523	443	405	424	0.119414883	0.693860733	0.972790461
228602	'4930402H24Rik'	1659	1660	2035	1759	1970	1681	1784.666667	1803.333333	0.078420492	0.839434187	0.974723675
228607	'Mavs'	854	1003	500	430	1199	1003	785.6666667	877.3333333	0.167370483	0.672631795	0.972790461
228608	'Smox'	502	525.5	1470	1038	528.94	793.97	832.5	786.97	-0.016458006	0.981171241	0.999493374
228662	'Btbd3'	1254	1220	2198	4956	1245	1444	1557.333333	2548.333333	0.947707463	0.230730598	0.857634106
228677	'Sptlc3'	0	0	0	0	2	1	0	1	2.320886	0.562538074	0.972790461
228684	'Sel1l2'	0	0	0	0	0	1	0	0.333333333	1.020273531	0.802557913	0.972790461
228714	'Kat14'	1227.7	1243.4	603.85	533.95	1031	1009.37	1025.003333	858.1066667	-0.213256148	0.543853624	0.972790461
228715	'Smim26'	51	58	98	66	52	74	69	64	-0.070064538	0.90102311	0.98672375
228730	'Kiz'	1143	1126	986	214	950	1028	1085	730.6666667	-0.647960272	0.198332034	0.824298956
228731	'Nkx2-4'	37	39	8	3	15	25	28	14.33333333	-0.982668931	0.262418078	0.88013169
228756	'Cstl1'	1	0	1	0	0	2	0.666666667	0.666666667	-0.156033058	0.963274716	0.999493374
228765	'Sdcbp2'	56	83	34	17	59	32	57.66666667	36	-0.658615195	0.251864127	0.871252558
228767	'Tmem74bos'	3	1	7	0	2	0	3.666666667	0.666666667	-2.587122962	0.215137865	0.842760284
228769	'Psmf1'	844.84	852.98	869.92	253	745.95	922.81	855.9133333	640.5866667	-0.483336157	0.261266811	0.879306888
228770	'Rspo4'	4	2	9	7	0	3	5	3.333333333	-0.400940642	0.797288495	0.972790461
228775	'Trib3'	237	197	6835	75	173	671	2423	306.3333333	-3.299701652	0.009846129	0.244698834
228777	'Nrsn2'	28	25	7	1	14	4	20	6.333333333	-1.658954342	0.106288281	0.679124905
228778	'6820408C15Rik'	39	52	25	3	42	32	38.66666667	25.66666667	-0.662797341	0.412113373	0.95722888
228785	'Mylk2'	0	1	98	43	3	5	33	17	-0.777040224	0.681311695	0.972790461
228787	'Xkr7'	10	14	2	1	8	1	8.666666667	3.333333333	-1.330828565	0.307758011	0.907963267
228788	'Ccm2l'	30	26	6	8	38	17	20.66666667	21	0.055265822	0.946636977	0.996592403
228790	'Asxl1'	4698	4518	3167	2857	3856	4129	4127.666667	3614	-0.130894023	0.597768979	0.972790461
228796	'Bpifb6'	0	4	0	2	1	0	1.333333333	1	-0.037341393	0.98991328	0.999493374
228801	'Bpifb1'	0	1	0	0	0	0	0.333333333	0	-0.903279821	0.824807108	0.972790461
228802	'Bpifb5'	6	7	1	0	6	1	4.666666667	2.333333333	-1.009371434	0.538354839	0.972790461

228807	'Zfp341'	102	109	168	40	90	85	126.3333333	71.66666667	-0.868932961	0.072176347	0.595063618
228812	'Pigu'	433	424	277	366	644	673	378	561	0.601924406	0.031359206	0.43015961
228829	'Phf20'	2452	2371	1679	2400	1758	2258	2167.333333	2138.666667	0.115238623	0.773933422	0.972790461
228836	'Dlgap4'	2475.5	2322.1	3928.1	1797.4	3139.2	2973.89	2908.536667	2636.83	-0.171928329	0.686139288	0.972790461
228839	'Tgif2'	1814	1957	1755	1038	2328	1927	1842	1764.333333	-0.067182012	0.826298186	0.972844676
228846	'D630003M21Rik'	163	147	76	36	114	68	128.6666667	72.66666667	-0.802438298	0.101528062	0.671759705
228850	'Ralgapb'	3317	3346	1074	3790	2874	2429	2579	3031	0.44293806	0.429183691	0.962386546
228852	'Ppp1r16b'	333	346	171.94	682.88	485.99	468	283.6466667	545.6233333	1.128610003	0.02187509	0.366099649
228858	'Gdap1l1'	4	4	10	3	5	1	6	3	-0.984825666	0.420280869	0.958308933
228859	'Fitm2'	131	129	52	143	118	163	104	141.3333333	0.578383862	0.23115749	0.8583057
228866	'Pcif1'	832	752	548	816	782	905	710.6666667	834.3333333	0.333572067	0.321606805	0.917800747
228869	'Ncoa5'	1695	1580	1208	490	1867	1215	1494.333333	1190.666667	-0.356269778	0.416180478	0.95722888
228875	'Slc35c2'	546	564	708	444	607	870	606	640.3333333	0.068169942	0.852783296	0.975734242
228876	'Zfp334'	1082	1076	950	645	1004	875	1036	841.3333333	-0.25991391	0.318909527	0.916136994
228880	'Zmynd8'	1913.8	1829.2	1307.5	1664	2514	2407.19	1683.483333	2195.08	0.436543197	0.075953843	0.606992211
228889	'Ddx27'	1278	1254	1116	791	1015	1399	1216	1068.333333	-0.158315467	0.560745832	0.972790461
228911	'Tshz2'	1971	1890	2259	1878	1493	1929	2040	1766.666667	-0.125005778	0.760142216	0.972790461
228913	'Zfp217'	2633	2429	1110	2300	1353	2294	2057.333333	1982.333333	0.114605068	0.818771751	0.972790461
228942	'Cbln4'	14	8	4	76	10	13	8.666666667	33	2.327032321	0.034125457	0.444207284
228960	'Stx16'	2703	2763	827	937	2363	1581	2097.666667	1627	-0.306094712	0.547396534	0.972790461
228961	'Npepl1'	929	960	647	164	1124	943	845.3333333	743.6666667	-0.260209762	0.657883038	0.972790461
228966	'Ppp1r3d'	110.83	97.6	138.01	11.62	57.78	61.31	115.48	43.57	-1.526386929	0.011858137	0.269351141
228980	'Taf4'	637	628	495	430	723	568	586.6666667	573.6666667	0.01363696	0.961104873	0.999493374
228983	'Osbpl2'	1219	1146	2309	716	1041	1367	1558	1041.333333	-0.628888061	0.197825256	0.823747311
228993	'Slc17a9'	78	69	45	18	91	107	64	72	0.109896379	0.85558993	0.975734242
228994	'Ythdf1'	2394	2447	2265	1490	2155	2410	2368.666667	2018.333333	-0.204681129	0.407303601	0.957157474
228998	'Arfgap1'	1227	1229	882	874	1255	1279	1112.666667	1136	0.083257147	0.730114388	0.972790461
229003	'Helz2'	558	584	478	796	819	761	540	792	0.650480067	0.058235695	0.551665011
229004	'Gmeb2'	1016	1029.2	935.05	443.84	852.99	709	993.4233333	668.61	-0.558106861	0.04663171	0.503156685
229007	'Zgpat'	783	805	337	446	865	661	641.6666667	657.3333333	0.101975439	0.799634383	0.972790461
229011	'Samd10'	141	144	116	21	185	241	133.6666667	149	0.043788618	0.95041302	0.998065566
229055	'Zbtb10'	1676	1746	2009	884	1521	1795	1810.333333	1400	-0.388348446	0.236484434	0.862272407
229096	'Ythdf3'	3730	3808	3006	3082	3954	3174	3514.666667	3403.333333	0.034534168	0.910076064	0.989498235
229211	'Acad9'	893.52	948.94	549.31	697.96	961.37	996.01	797.2566667	885.1133333	0.220692089	0.442502724	0.969447084
229214	'Qrfpr'	1	2	0	1	0	0	1	0.333333333	-1.32519453	0.684707949	0.972790461
229227	'4932438A13Rik'	4557	4577	811	2494	4472	4856	3315	3940.666667	0.335277264	0.579185917	0.972790461
229228	'Nudt6'	186.42	178.51	127.31	156.81	149.25	142.46	164.08	149.5066667	-0.020398136	0.958284333	0.999493374
229277	'Stoml3'	4	0	1	3	1	0	1.666666667	1.333333333	0.008529207	0.997060173	0.9996806
229279	'Hnrnpa3'	14360	15271	28706	13622	14466	16226	19445.66667	14771.33333	-0.38610138	0.434642912	0.965354095
229285	'Spg20'	1156	1136	1154	457	1042	1222	1148.666667	907	-0.377211779	0.281512331	0.889731741

229302	'Tm4sf4'	0	1	0	0	0	0	0.333333333	0	-0.903279821	0.824807108	0.972790461
229317	'Eif2a'	1157	1104	2465	1000	1267	1282	1575.333333	1183	-0.429312197	0.411764614	0.95722888
229320	'Clrn1'	1	1	0	0	4	5	0.666666667	3	2.108044379	0.335126885	0.922326626
229323	'Gpr171'	0	1	1	3	2	2	0.666666667	2.333333333	1.908215721	0.311605291	0.911537035
229333	'Aadacl2fm1'	1	0	0	0	2	1	0.333333333	1	1.368261242	0.684350922	0.972790461
229357	'Gpr149'	0	1	0	1	0	4	0.333333333	1.666666667	2.193802445	0.469971303	0.972790461
229363	'Gmps'	3358.5	3357.4	2705	980.54	2404.9	2375.48	3140.283333	1920.313333	-0.725935549	0.02415862	0.384033173
229445	'Ctso'	327	334	121	256	513	438	260.6666667	402.3333333	0.688526214	0.118088415	0.706706957
229473	'Tmem131l'	1521	1480	461	781	1645	1620	1154	1348.666667	0.279877284	0.5610974	0.972790461
229474	'Fhdc1'	73	59	123	141	126	188	85	151.6666667	0.849367103	0.112111458	0.69684179
229487	'Gatb'	764	801	214	171	560	504	593	411.6666667	-0.505203034	0.371670669	0.938527219
229488	'Fam160a1'	287	281	225	512	298	590	264.3333333	466.6666667	0.934849384	0.04011596	0.46876745
229499	'Fcrl1'	0	3	0	0	2	7	1	3	1.523622204	0.56144116	0.972790461
229503	'Rrnad1'	486.26	418.88	389.06	254.59	486.18	358.42	431.4	366.3966667	-0.208729009	0.504086455	0.972790461
229504	'Isg20l2'	1018	1021	1160	576	918	1006	1066.333333	833.3333333	-0.355226597	0.248455657	0.869491261
229512	'Smg5'	1967	2069	1143	774	1871	1841	1726.333333	1495.333333	-0.198160415	0.576785918	0.972790461
229517	'Slc25a44'	427	493	633	820	611	757	517.6666667	729.3333333	0.578672631	0.216167307	0.843391934
229521	'Syt11'	704	619.04	145	443	473.84	464.12	489.3466667	460.32	0.067974437	0.906728421	0.988823459
229524	'Msto1'	565.87	601.08	335.54	151.68	581.92	737.26	500.83	490.2866667	-0.07873668	0.877494814	0.981341203
229534	'Pbxip1'	898	1014	287	694	1390	1055	733	1046.333333	0.593602773	0.226912481	0.854001521
229541	'Dennd4b'	491	436	516	203	530	413	481	382	-0.363396943	0.341434838	0.925308103
229542	'Gatad2b'	2838	2788	3494	3237	2224	2491	3040	2650.666667	-0.082885537	0.86111847	0.976863639
229543	'Ints3'	2247	2364	1499	8600	2440	2898	2036.666667	4646	1.485736499	0.035066586	0.449249282
229550	'S100a7l2'	0	0	1	0	0	0	0.333333333	0	-0.903279821	0.824807108	0.972790461
229571	'Tdpoz8'	0	0	1	0	0	0	0.333333333	0	-0.903279821	0.824807108	0.972790461
229574	'Flg2'	1	0	1	0	0	1	0.666666667	0.333333333	-0.894127833	0.804654786	0.972790461
229584	'Pogz'	2588	2680	1661	1916	2525	2291	2309.666667	2244	0.041810359	0.886698973	0.984461626
229588	'Gm128'	11.31	6	22	0	9.67	9.22	13.10333333	6.296666667	-1.314781153	0.279047411	0.889355605
229589	'Prune1'	797.63	774.35	880.45	1079.5	712.06	1089.42	817.4766667	960.3166667	0.330717566	0.455861023	0.971239019
229593	'Golph3l'	983	1087	2602	565	991	1259	1557.333333	938.3333333	-0.81940929	0.152519942	0.763026656
229595	'Adamtsl4'	37	22	9	77	27	23	22.66666667	42.33333333	1.216185013	0.161181713	0.771435531
229599	'Ciart'	136	94	802	16	63	61	344	46.66666667	-3.097915297	8.44E-04	0.060130669
229600	'BC028528'	146	142	41.68	19	150	110	109.8933333	93	-0.266088481	0.714585893	0.972790461
229603	'Otud7b'	2381	2301	1910	3489	1717	2169	2197.333333	2458.333333	0.351179723	0.504928518	0.972790461
229615	'Pias3'	670	692	465	527	757	532	609	605.3333333	0.075605687	0.821605183	0.972790461
229644	'Trim45'	324	312	167	106	308	310	267.6666667	241.3333333	-0.154303874	0.719103132	0.972790461
229658	'Vangl1'	241	246	171	107	274	260	219.3333333	213.6666667	-0.04363759	0.903280632	0.987531419
229663	'Csde1'	9220	9125	15958	4651	7186	10849	11434.33333	7562	-0.654275121	0.159949157	0.771114735
229665	'Ampd1'	2	1	0	1	0	0	1	0.333333333	-1.330404752	0.683404215	0.972790461
229672	'Bcl2l15'	83.09	109.99	63.98	56	52	17	85.68666667	41.66666667	-0.840502048	0.207384719	0.83498481

229675	'Rsbn1'	1100	1037	2845	1274	1015	976	1660.666667	1088.333333	-0.567515013	0.372014576	0.938527219
229681	'St7l'	394	368	220	216	408	404	327.3333333	342.6666667	0.100910244	0.752057863	0.972790461
229687	'Chil5'	4	5	0	0	0	2	3	0.666666667	-2.153973437	0.407395041	0.957157474
229688	'Chil6'	0	0	2	0	1	0	0.666666667	0.333333333	-1.033504517	0.79720072	0.972790461
229697	'Cym'	2	4	0	0	0	3	2	1	-1.042171652	0.705340998	0.972790461
229699	'Slc16a4'	179	199	24	33	456	368	134	285.6666667	1.060163125	0.245219345	0.86624121
229700	'Rbm15'	488	531	352	437	362	381	457	393.3333333	-0.083589295	0.833658961	0.974723675
229706	'Slc6a17'	111	90	58	9	59	43	86.33333333	37	-1.266704621	0.041785269	0.477375478
229707	'Strip1'	996	937	1301	1196	1135	1193	1078	1174.666667	0.187039302	0.654759736	0.972790461
229709	'Ahcyl1'	3630	3582	3061	5217	3222	3719	3424.333333	4052.666667	0.401635927	0.389169174	0.951190011
229714	'Gpr61'	12	15	1	1	15	2	9.333333333	6	-0.613009038	0.65347893	0.972790461
229715	'Amigo1'	454	418	211	759	493	579	361	610.3333333	0.941093635	0.057399619	0.548658397
229722	'Elapor1'	4	10	2	29	10	40	5.333333333	26.33333333	2.451858031	0.008943055	0.231327017
229725	'Clcc1'	473.5	520.51	252	368	645	606	415.3366667	539.6666667	0.434426664	0.200101976	0.82759347
229731	'Slc25a24'	609	679	1526	216	750	829	938	598.3333333	-0.790031613	0.203412307	0.831671997
229759	'Olfm3'	2	1	0	1	1	0	1	0.666666667	-0.338798923	0.89654217	0.985710789
229776	'Cdc14a'	324	322	319	260	512	349	321.6666667	373.6666667	0.235158339	0.488935314	0.972790461
229780	'Trmt13'	282.84	267.39	323.83	301.46	247.85	171.63	291.3533333	240.3133333	-0.149368811	0.770389919	0.972790461
229782	'Slc35a3'	932	909	604	750	1064	1097.72	815	970.5733333	0.311466369	0.233046942	0.859679981
229791	'Plppr4'	28	32	29	46	37	32	29.66666667	38.33333333	0.490855942	0.378342192	0.9419364
229801	'Tram1l1'	155	143	40	22	186	143	112.6666667	117	0.022298658	0.976165653	0.999493374
229841	'Cenpe'	1393	1310	239	174	1013	882	980.6666667	689.6666667	-0.51376493	0.48098052	0.972790461
229862	'Gm4861'	0	0	0	0	0	1	0	0.333333333	1.020273531	0.802557913	0.972790461
229877	'Rap1gds1'	1568	1511	2183	755	1540	1735	1754	1343.333333	-0.434218365	0.279260866	0.889355605
229898	'Gbp5'	37	44	152	2	15	13	77.66666667	10	-3.143714714	5.10E-04	0.044231386
229900	'Gbp7'	310	329	173	24	293	353	270.6666667	223.3333333	-0.372532651	0.623556334	0.972790461
229905	'Kyat3'	473	632	143	140	704	683	416	509	0.278422972	0.671353071	0.972790461
229906	'Gtf2b'	798	826	798	630	776	870	807.3333333	758.6666667	-0.045669951	0.872233746	0.980171118
229927	'Clca3b'	24	41	11	0	32	37	25.33333333	23	-0.229614036	0.846726979	0.975209338
229933	'Clca2'	16	11	12	141	35	59	13	78.33333333	2.846756048	4.36E-04	0.040018506
229937	'Znhit6'	629	687	655	169	508	706	657	461	-0.582835134	0.20390561	0.832079769
229949	'Ak5'	93	80	235	21	66	73	136	53.33333333	-1.488114246	0.02927807	0.417580391
230025	'Prdm13'	16	23	1	0	8	7	13.33333333	5	-1.438156754	0.307746852	0.907963267
230027	'Coq3'	540	570	204	138	421	532	438	363.6666667	-0.273554995	0.602847299	0.972790461
230073	'Ddx58'	457	408	868	336	447	934	577.6666667	572.3333333	-0.093457566	0.866114163	0.978294615
230075	'Ndufb6'	739	854	658	385	499	750	750.3333333	544.6666667	-0.437436473	0.142402038	0.748920481
230082	'Nol6'	1357	1367	1322	1214	1329	1511	1348.666667	1351.333333	0.063473228	0.833946853	0.974723675
230085	'Phf24'	326	276	51	333	295	134	217.6666667	254	0.470329893	0.529835163	0.972790461
230088	'Fam214b'	436	427	740	494	525	460	534.3333333	493	-0.074692826	0.877787267	0.981341203
230098	'Arhgef39'	59	59	34	22	48	51	50.66666667	40.33333333	-0.310093648	0.492042669	0.972790461

230099	'Car9'	37	39	242	27	39	116	106	60.66666667	-1.005577863	0.285278161	0.893711786
230101	'Gba2'	413	409	265	358	462	391	362.3333333	403.6666667	0.244478133	0.448827803	0.97049895
230103	'Npr2'	1312	1411	506	250	2366	1287	1076.333333	1301	0.232681133	0.737971339	0.972790461
230119	'Zbtb5'	911	901	698	553	882	833	836.6666667	756	-0.105627159	0.661170833	0.972790461
230125	'Slc25a51'	2397	2443	3375.6	2705	2384	2523	2738.533333	2537.333333	-0.038647039	0.929922491	0.993240809
230126	'Shb'	291	252	864	1153	220	546	469	639.6666667	0.60461447	0.489668379	0.972790461
230145	'Galnt12'	89	89	78	109	66	181	85.33333333	118.6666667	0.538242293	0.287342536	0.894554788
230157	'Tmeff1'	1354	1562	1905	1428	1735	1705	1607	1622.666667	0.049171578	0.895001372	0.985422597
230161	'Acnat1'	29.71	31	4	16	28	18	21.57	20.66666667	0.076794188	0.924542646	0.992176833
230162	'Zfp189'	355	350	478	233	319	339	394.3333333	297	-0.400041167	0.286888714	0.894554788
230163	'Aldob'	0	2	1	8	1	0	1	3	1.985188685	0.378665661	0.94215965
230233	'Elp1'	2114	2128	1172	857	1822	1769	1804.666667	1482.666667	-0.258165124	0.43639808	0.965982602
230234	'Abitram'	822	855	564	625	719	621	747	655	-0.087650645	0.792547283	0.972790461
230235	'Frrs1l'	43	30	3	8	15	9	25.33333333	10.66666667	-1.12051717	0.239373889	0.86449675
230249	'Ecpas'	5156	5187	3403	1851	4340	4974	4582	3721.666667	-0.307999528	0.367585478	0.937190492
230257	'Ptbp3'	2600	2626	4829	4296	2952	3111	3351.666667	3453	0.130776012	0.815440972	0.972790461
230259	'E130308A19Rik'	2053	2089	768	858	1586	1545	1636.666667	1329.666667	-0.236698595	0.573878214	0.972790461
230279	'Tmem268'	225	242	100	57	253	191	189	167	-0.191588391	0.725420007	0.972790461
230316	'Megf9'	320	309	78	225	322	303	235.6666667	283.3333333	0.379618025	0.479216442	0.972790461
230376	'Haus6'	2279	2213	590	693	1237	1051	1694	993.6666667	-0.677850458	0.187377672	0.808617437
230379	'Acer2'	107	115	95	90	156	104	105.6666667	116.6666667	0.189192878	0.607982775	0.972790461
230393	'Focad'	1634.5	1657.5	551.17	316.09	1225.3	1122.04	1281.073333	887.81	-0.531383114	0.333931461	0.921648675
230398	'Ifna16'	0	0	2	0	0	0	0.666666667	0	-1.995285526	0.619817042	0.972790461
230405	'Ifne'	0	2	0	0	1	0	0.666666667	0.333333333	-0.733814424	0.856146772	0.975734242
230459	'Cyp2j13'	0	0	1	0	0	0	0.333333333	0	-0.903279821	0.824807108	0.972790461
230484	'Usp1'	1697	1748	1496	616	1719	1680	1647	1338.333333	-0.333081876	0.35819936	0.933245301
230500	'Efcab7'	330	314	145	36	187	121	263	114.6666667	-1.216470445	0.032022562	0.433742328
230514	'Leprot'	919	938	585	1561	1229	1486	814	1425.333333	0.943574012	0.017106944	0.32733359
230558	'C8a'	0	0	0	0	21	31	0	17.33333333	6.399804702	0.005356015	0.17331813
230576	'Ttc22'	9	13	0	1	20	14	7.333333333	11.66666667	0.657605748	0.644627053	0.972790461
230577	'Pars2'	172	189	117	61	202	198	159.3333333	153.6666667	-0.077893348	0.860584088	0.976863639
230579	'Fam151a'	5.1	3.84	0	0	1.68	11.53	2.98	4.403333333	0.515654675	0.801633709	0.972790461
230582	'Cyb5rl'	511	501	122	59	265	227	378	183.6666667	-1.037406405	0.106808748	0.680968451
230584	'Yipf1'	283	307	165	240	466	509	251.6666667	405	0.713020915	0.034526631	0.445535842
230587	'Glis1'	20	8	21	2	6	6	16.33333333	4.666666667	-1.860780278	0.037823945	0.459930654
230590	'Zyg11a'	513	466	61	17	166	128	346.6666667	103.6666667	-1.757650041	0.041910063	0.477648737
230594	'Tut4'	3184	3002	3652	2417	2738	2603	3279.333333	2586	-0.28690707	0.422924162	0.959848252
230596	'Prpf38a'	878	977	931	379	824	872	928.6666667	691.6666667	-0.448268164	0.168644441	0.784384366
230597	'Zfyve9'	2555.8	2394.8	1474	367	1982.1	2343.21	2141.52	1564.106667	-0.522243962	0.361677407	0.933706718
230598	'Nrd1'	4390.8	4256.7	2992.6	2343.8	3949.2	3775.66	3880.063333	3356.23	-0.169222027	0.488583131	0.972790461

230603	'Ttc39a'	122	138	87	14	144	134	115.6666667	97.33333333	-0.339748199	0.629976644	0.972790461
230612	'Slc5a9'	1	0	0	0	1	2	0.333333333	1	1.346160497	0.689889638	0.972790461
230613	'Skint10'	0	0	0	0	1	0	0	0.333333333	1.020273531	0.802557913	0.972790461
230622	'Skint6'	1	0	0	0	0	5	0.333333333	1.666666667	2.039628124	0.607445844	0.972790461
230648	'Efcab14'	1497.5	1359.2	717.64	1187.7	1242.5	1624.22	1191.443333	1351.466667	0.280016537	0.440992415	0.968551476
230649	'Atpaf1'	798	772	286	556	605	817	618.6666667	659.3333333	0.197932697	0.654170701	0.972790461
230654	'Lrrc41'	3059	3173	1821.3	999.24	3013.1	3244.24	2684.46	2418.856667	-0.170784532	0.680529515	0.972790461
230657	'Tmem69'	214.11	225.04	129.81	50.15	182	220	189.6533333	150.7166667	-0.368951603	0.460728471	0.972790461
230661	'Tesk2'	270.59	257.57	110.42	128.05	221.98	160.37	212.86	170.1333333	-0.23883772	0.578049261	0.972790461
230673	'Ipo13'	972	969	577	1156	960	954	839.3333333	1023.333333	0.43442981	0.294500086	0.90029104
230674	'Kdm4a'	2144	2182	2063	646	2698	1920	2129.666667	1754.666667	-0.338633835	0.463342422	0.972790461
230676	'Szt2'	2036	1998	581	1640	2507	1431	1538.333333	1859.333333	0.40939468	0.443975823	0.969880311
230678	'Tmem125'	2	7	18	13	5	18	9	12	0.403582401	0.712858676	0.972790461
230696	'AU022252'	646	642	639	800.9	577	635	642.3333333	670.9666667	0.187889255	0.667723421	0.972790461
230700	'Foxj3'	2114.9	1953.7	3117.8	1211	1582	1985.75	2395.47	1592.896667	-0.598645233	0.141977397	0.747514336
230709	'Zmpste24'	960	1006	625	1272	1382	1506	863.6666667	1386.666667	0.778229236	0.012906372	0.283999932
230718	'Nt5c1a'	0	0	1	0	0	2	0.333333333	0.666666667	0.78767502	0.845746136	0.975182082
230721	'Pabpc4'	2315	2297	6966	2000	2220	3528	3859.333333	2582.666667	-0.644108541	0.306897786	0.907830798
230726	'Rhbdl2'	3	4	3	1	5	3	3.333333333	3	-0.175473506	0.888020157	0.984534762
230734	'Yrdc'	542	564	725	359	426	602	610.3333333	462.3333333	-0.394419083	0.295069128	0.90029104
230735	'Epha10'	9	12.22	3	1	15	9	8.073333333	8.333333333	0.027598824	0.979756987	0.999493374
230737	'Gnl2'	2010	2018	1756	817	1806	1740	1928	1454.333333	-0.415551639	0.158779106	0.768918948
230738	'Zc3h12a'	112	119	672	218	66	148	301	144	-1.009681227	0.279504413	0.889355605
230751	'Oscp1'	120	132	38	24	127	130	96.66666667	93.66666667	-0.067887542	0.919093108	0.990815958
230752	'Evalb'	498	462	689	841	689	668	549.6666667	732.6666667	0.502387299	0.295245914	0.90029104
230753	'Thrap3'	6876	7030	5182	6813	6603	7530	6362.666667	6982	0.232095244	0.467525871	0.972790461
230757	'5730409E04Rik'	319	371	145	13	336	415	278.3333333	254.6666667	-0.225387514	0.802715057	0.972790461
230761	'Zfp362'	1360	1390	827	327	1503	1232	1192.333333	1020.666667	-0.265399899	0.593113168	0.972790461
230766	'Fam167b'	8	9	0	1	32	11	5.666666667	14.66666667	1.360114558	0.349294338	0.928823768
230767	'Iqcc'	168	178	102	80	275	183	149.3333333	179.3333333	0.260546137	0.565741208	0.972790461
230770	'Tmem39b'	215.99	201	163	152	300	251	193.33	234.3333333	0.302370927	0.319136034	0.916136994
230775	'Adgrb2'	836	780	139	574	422	527	585	507.6666667	-0.004216115	0.994906671	0.999562152
230777	'Hcrtr1'	1	3	4	49	1	5	2.666666667	18.33333333	3.151005384	0.037178325	0.459930654
230779	'Serinc2'	106	79	119	1890	138	581	101.3333333	869.6666667	3.405683504	3.05E-04	0.030488342
230784	'Sesn2'	320	346	1730	255	471	510	798.6666667	412	-1.090362226	0.164018431	0.776667102
230787	'Themis2'	6	15	2	8	9	14	7.666666667	10.33333333	0.529631744	0.575927363	0.972790461
230789	'Fam76a'	613	555	713	675	572	730	627	659	0.141824561	0.728786396	0.972790461
230793	'Ahdc1'	2325	2295	1403	2102	1901	1986	2007.666667	1996.333333	0.121618779	0.745911675	0.972790461
230796	'Wdtc1'	1304	1165	810	1607	1312	1847	1093	1588.666667	0.651322677	0.079286761	0.617305615
230801	'Pigv'	346	321	197	362	403	396	288	387	0.529308141	0.127602182	0.723917691

230806	'Crybg2'	20	23	8	5	10	15	17	10	-0.735107478	0.322579362	0.918775094
230809	'Pdik1l'	751	734	349	312	575	539	611.3333333	475.3333333	-0.309272803	0.392472813	0.952648938
230810	'Slc30a2'	98	113	84	284	339	425	98.33333333	349.3333333	1.882593537	8.41E-10	7.99E-07
230815	'Man1c1'	370	353	317	532	444	650	346.6666667	542	0.728685901	0.044743048	0.492557243
230822	'Ncmap'	7	5	6	25	4	7	6	12	1.285568377	0.242269573	0.86449675
230824	'Grhl3'	3	0	6	113	7	25	3	48.33333333	4.276557364	0.002011553	0.099125678
230828	'Il22ra1'	6	6	1	484	10	295	4.333333333	263	6.251177235	2.76E-07	1.29E-04
230837	'Asap3'	350	273	107	544	393	268	243.3333333	401.6666667	0.951614537	0.118367852	0.707231175
230848	'Zbtb40'	609	616	289	595	570	465	504.6666667	543.3333333	0.262203818	0.558895436	0.972790461
230857	'Ece1'	2281	2216	566	2905	3479	3505	1687.666667	3296.333333	1.100343281	0.037018475	0.459648634
230861	'Eif4g3'	4126	3926	2240	2838	4625	5030	3430.666667	4164.333333	0.327720806	0.271775015	0.884539076
230863	'Sh2d5'	50	66	39	224	61	96	51.66666667	127	1.567489071	0.031304048	0.429713045
230866	'Emc1'	1080	1054	319	678	1219	1107	817.6666667	1001.333333	0.373076757	0.43673152	0.96622803
230868	'Igsf21'	32	25	1	1	31	29	19.33333333	20.33333333	0.039767488	0.975094281	0.999493374
230872	'Crocc'	760	703	306	179	923	635	589.6666667	579	-0.04702239	0.933634819	0.994245515
230883	'Aadacl3'	1	0	0	0	0	0	0.333333333	0	-0.903279821	0.824807108	0.972790461
230895	'Vps13d'	3701	3397	1869	4013	3422	3230	2989	3555	0.406806519	0.344042751	0.925308103
230899	'Nppa'	3	3	3	0	1	1	3	0.666666667	-2.208195036	0.215571384	0.843391934
230903	'Fbxo44'	34	35	71	683	107	393	46.66666667	394.3333333	3.257038547	1.01E-04	0.013994201
230904	'Fbxo2'	12	14	14	2436	14	476	13.33333333	975.3333333	6.575305141	2.76E-07	1.29E-04
230908	'Tardbp'	7008.1	7272.7	3723	5182.7	6448.7	5116	6001.246667	5582.456667	0.009738857	0.978956939	0.999493374
230909	'Gm572'	1	1	1	1	0	1	1	0.666666667	-0.466310306	0.836180741	0.974723675
230917	'Tmem201'	1333	1274	675	672	1310	1197	1094	1059.666667	-0.002685471	0.993631908	0.999493374
230935	'Dnajc11'	1495	1443	988	752	1460	1426	1308.666667	1212.666667	-0.086862971	0.752265809	0.972790461
230936	'Phf13'	2678	2673	1935	1954	2153	3016	2428.666667	2374.333333	0.02923889	0.918281226	0.990727847
230959	'Ajap1'	48	44	1	55	47	22	31	41.33333333	0.685479638	0.523964484	0.972790461
230967	'Cep104'	968	869	505	692	752	700	780.6666667	714.6666667	-0.010666482	0.976844485	0.999493374
230971	'Megf6'	1187	991	118	1332	1605	1754	765.3333333	1563.666667	1.178906441	0.098955713	0.668585731
230972	'Arhgef16'	37	36	26	49	59	218	33	108.6666667	1.681331154	0.007632658	0.21034504
230979	'Tnfrsf14'	69.64	67.18	30.04	25.87	94.05	57.13	55.62	59.01666667	0.094869452	0.865816954	0.978294615
230991	'Fndc10'	232	206	163	230	294	447	200.3333333	323.6666667	0.723909284	0.023386757	0.379052873
230996	'9430015G10Rik'	700	682	258	285	711	363	546.6666667	453	-0.204255795	0.681340422	0.972790461
231002	'Plekhn1'	62	58	81	31	104	59	67	64.66666667	-0.099489114	0.852750943	0.975734242
231003	'Klhl17'	601.89	559.95	292	284	664.96	559	484.6133333	502.6533333	0.083186104	0.825459812	0.972790461
231004	'Samd11'	60.46	53.24	54.62	26.64	34.09	37	56.10666667	32.57666667	-0.746239511	0.077128533	0.611256471
231014	'Elapor2'	90	90	78	295	131	197	86	207.6666667	1.453018044	0.010433186	0.252950741
231042	'Nupl2'	204	212	97	29	185	169	171	127.6666667	-0.470142504	0.449475694	0.97049895
231044	'Gbx1'	4	5	2	2	6	3	3.666666667	3.666666667	0.040161865	0.972629941	0.999493374
231045	'Iqca1l'	4	2	0	0	1	0	2	0.333333333	-2.325246033	0.421329985	0.959205565
231050	'Galnt11'	605	616	558	607	522	572	593	567	0.039508797	0.916432536	0.990526893

231051	'Kmt2c'	9131	8749	2487	4738	5208	6086	6789	5344	-0.214893727	0.670024108	0.972790461
231070	'Insig1'	1095	1068	1055	524	895	1347	1072.666667	922	-0.24003751	0.48011174	0.972790461
231086	'Hadhb'	2800	3078	1860	4128	4275	5266	2579.333333	4556.333333	0.91301505	0.003636055	0.139091896
231093	'Agbl5'	829	795	814	145	814	802	812.6666667	587	-0.567787589	0.309364643	0.910300259
231098	'Dnajc5g'	31	26	16	0	5	20	24.33333333	8.333333333	-1.660277509	0.135267048	0.736012522
231103	'Gckr'	22	27	8	0	10	4	19	4.666666667	-2.059683479	0.064109643	0.568781147
231123	'Haus3'	542	551	399	414	533	584	497.3333333	510.3333333	0.097717601	0.708439934	0.972790461
231125	'Zfyve28'	56.46	80	13	9.99	38	37	49.82	28.33	-0.811059166	0.290032864	0.898062672
231128	'Fam193a'	2037	1974	1314	806	1856	1833	1775	1498.333333	-0.23982887	0.452612357	0.970649024
231130	'Tnip2'	345	310	520	238	374	304	391.6666667	305.3333333	-0.361366957	0.409074588	0.957157474
231134	'Dok7'	20	25	11	15	53	14	18.66666667	27.33333333	0.591283661	0.429650613	0.962386546
231147	'Sh3tc1'	125	115	93	124	203	239	111	188.6666667	0.786186938	0.010136203	0.249300759
231148	'Ablim2'	42	58	31	319	40	124	43.66666667	161	2.20087347	0.010805628	0.256475153
231151	'Tada2b'	718.18	712.77	824.23	388.19	599.42	682.01	751.7266667	556.54	-0.432883226	0.170574165	0.785702308
231201	'AF366264'	20.24	29.28	6.81	3	11.37	10	18.77666667	8.123333333	-1.165505778	0.173928184	0.792829561
231207	'Cpeb2'	698	683	708	282	579	521	696.3333333	460.6666667	-0.603114446	0.051956026	0.52907765
231214	'Cc2d2a'	863	938	240	49	1524	790	680.3333333	787.6666667	0.151915581	0.869468259	0.979633196
231225	'Tapt1'	775	654	441	538	924	739	623.3333333	733.6666667	0.292326431	0.348612901	0.928444937
231238	'Sel1l3'	26	20	28	112	124	145	24.66666667	127	2.408842268	4.82E-08	2.86E-05
231252	'Chrna9'	0	0	0	1	0	0	0	0.333333333	1.020273531	0.802557913	0.972790461
231279	'Gufl'	932	938	317	129	972	562	729	554.3333333	-0.422139787	0.526304464	0.972790461
231290	'Slc10a4'	9	5	1	0	4	0	5	1.333333333	-1.898632133	0.328006044	0.92113012
231293	'Cwh43'	0	0	0	0	2	3	0	1.666666667	3.030642133	0.444945299	0.969888068
231296	'Lrrc66'	3	2	0	0	10	11	1.666666667	7	2.012220286	0.26297879	0.88013169
231326	'Aasdh'	794	727	217	210	737	456	579.3333333	467.6666667	-0.276084905	0.627686027	0.972790461
231327	'Ppat'	939.66	996.87	739.47	1022.3	928.11	1063.02	892	1004.48	0.277449498	0.421008592	0.958945978
231329	'Polr2b'	4619	4720	1641	2381	3121	3747	3660	3083	-0.154188158	0.721239545	0.972790461
231380	'Uba6'	2620	2590	1305	1384	1871	2131	2171.666667	1795.333333	-0.199477004	0.54612912	0.972790461
231382	'Tmprss11d'	5	0	0	0	0	0	1.666666667	0	-3.025187063	0.445317893	0.969888068
231386	'Ythdc1'	2672	2641	2924	1624	2419	2290	2745.666667	2111	-0.356167718	0.23087373	0.857634106
231396	'Ugt2b36'	4	2	0	18	7	10	2	11.66666667	2.80298834	0.025745932	0.39630136
231413	'Grsf1'	2747	2807	3755	1142	2214	3264	3103	2206.666667	-0.552207663	0.188435556	0.810712105
231430	'Cox18'	217	262.5	176	122	179	254	218.5	185	-0.215761091	0.503779659	0.972790461
231440	'Parm1'	187	166	165	588	201	274	172.6666667	354.3333333	1.265210665	0.050404811	0.519327729
231452	'Sdad1'	2703	2668	2570	954	1840	2630	2647	1808	-0.579954821	0.092877621	0.654455509
231464	'Cnot6l'	1803	1651	1859	1173	1572	1964	1771	1569.666667	-0.158114145	0.596781487	0.972790461
231470	'Fras1'	1662	1605	131	201	1861	1015	1132.666667	1025.666667	-0.14021173	0.876799073	0.981341203
231474	'Paqr3'	514.38	480.42	341.14	495.58	444.26	421.61	445.3133333	453.8166667	0.156634991	0.68910875	0.972790461
231503	'Tmem150c'	118	108	121	19	212	143	115.6666667	124.6666667	-0.010285984	0.988480954	0.999493374
231506	'Lin54'	474	529	542	723	412	458	515	531	0.200255453	0.699946087	0.972790461

231507	'Plac8'	66	69	180	1271	57	82	105	470	2.510423838	0.036674615	0.457518227
231510	'Gpat3'	118	99	182	149	238	311	133	232.6666667	0.763510746	0.102004866	0.671776916
231532	'Arhgap24'	226	212	191	249	235	198	209.6666667	227.3333333	0.230534836	0.57192932	0.972790461
231549	'Lrrc8d'	339	298	382	383	548	761	339.6666667	564	0.722285637	0.041800024	0.477375478
231571	'Rpap2'	563	589	545	189	657	649	565.6666667	498.3333333	-0.242307993	0.577890067	0.972790461
231580	'Gak'	1943	1902	1782	1709	1843	2168	1875.666667	1906.666667	0.086877743	0.76894335	0.972790461
231583	'Slc26a1'	39	27	11	4	46	22.61	25.66666667	24.20333333	-0.129639388	0.881524741	0.982528614
231589	'Vmn2r13'	3.02	3	1	0	2	1	2.34	1	-1.239715923	0.49850004	0.972790461
231591	'Vmn2r14'	2.98	0	0	0	1	1	0.993333333	0.666666667	0.006630366	0.998451383	0.999900097
231600	'Chfr'	681	683	268	449	617	857	544	641	0.305066552	0.463399122	0.972790461
231602	'P2rx2'	16	12	12	3	34	16	13.33333333	17.66666667	0.328388276	0.707366119	0.972790461
231605	'Galnt9'	4	8	1	0	3	1	4.333333333	1.333333333	-1.694594591	0.31177466	0.911537035
231630	'Ficd'	148	138	172	129	185	286	152.6666667	200	0.372386172	0.340530565	0.925308103
231633	'Tmem119'	130	135	40	690	174	152	101.6666667	338.6666667	2.109041424	0.013142316	0.286638321
231637	'Ssh1'	1151	1151	796	996	936	889	1032.666667	940.3333333	-0.011910325	0.974229239	0.999493374
231642	'Alkbh2'	118	122	364	111	86	177	201.3333333	124.6666667	-0.730980739	0.283880707	0.892236734
231646	'Myo1h'	16.89	20.94	62.61	4.18	21.89	15.69	33.48	13.92	-1.450443655	0.104218988	0.676639601
231655	'Oasl1'	21	18	53	12	16	57	30.66666667	28.33333333	-0.254041875	0.76122979	0.972790461
231659	'Gcn1'	3080	3005	1113	2068	3184	3095	2399.333333	2782.333333	0.300624159	0.472780729	0.972790461
231668	'Vsig10'	503	589	271	340	708	867	454.3333333	638.3333333	0.508277457	0.190172457	0.813136091
231670	'Fbxo21'	4656	5073	3464	2002	6198	6603	4397.666667	4934.333333	0.131151017	0.744809163	0.972790461
231672	'Fbxw8'	2472	2464	1961	1038	2265	2456	2299	1919.666667	-0.269250934	0.370434828	0.938038679
231691	'Sds'	8	10	5	11	5	3	7.666666667	6.333333333	-0.038448964	0.970019568	0.999493374
231699	'Oas1e'	0	2	0	2	3	54	0.666666667	19.66666667	4.792202579	0.010855791	0.257024034
231712	'Trafd1'	588	647	587	300	901	815	607.3333333	672	0.104131592	0.788890563	0.972790461
231713	'Naa25'	1313	1370	1038	1376	1106	1272	1240.333333	1251.333333	0.135833366	0.722211288	0.972790461
231717	'Pheta1'	428	445	374	216	470	404	415.6666667	363.3333333	-0.190793315	0.527882839	0.972790461
231724	'Rad9b'	791.63	781.52	195.49	54.83	528.44	269.18	589.5466667	284.15	-1.076851499	0.155104031	0.766453805
231727	'B3gnt4'	12.04	18.02	8.03	18.1	9.01	7.01	12.69666667	11.37333333	0.061389592	0.942995786	0.996301887
231760	'Rimbp2'	31	37	21	0	26	15	29.66666667	13.66666667	-1.217755821	0.252389339	0.871252558
231769	'Sfswap'	1980	2063	1273	818	2242	1497	1772	1519	-0.209294091	0.574443228	0.972790461
231798	'Lrch4'	653	665	360	1330	721	714	559.3333333	921.6666667	0.942828835	0.088848524	0.644974469
231801	'Agfg2'	311	366	527	5038	367	2211	401.3333333	2538.666667	2.911863652	0.001541731	0.084487393
231803	'Mepce'	1208	1229	1083	640	1175	1273	1173.333333	1029.333333	-0.18589055	0.482283195	0.972790461
231805	'Pilra'	1.1	4.41	3.21	0	6.27	7.32	2.906666667	4.53	0.563152623	0.714921936	0.972790461
231807	'Map11'	552	506	189	182	508	456	415.6666667	382	-0.097205172	0.841551326	0.975182082
231821	'Adap1'	29	24	34	18	21	46	29	28.33333333	-0.056053059	0.924041828	0.991932584
231830	'Micall2'	282	351	385	641	359	392	339.3333333	464	0.602973364	0.26948549	0.883851074
231832	'Tmem184a'	195	205	150	245	387	481	183.3333333	371	1.039686796	2.49E-04	0.026447937
231834	'Snx8'	834	828	733	867	673	990	798.3333333	843.3333333	0.173687034	0.64425772	0.972790461

231841	'Bratl'	569	632	544	404	550	656	581.6666667	536.6666667	-0.084572979	0.748882524	0.972790461
231842	'Amz1'	12	9	94	17	22	22	38.33333333	20.33333333	-1.024319307	0.321816406	0.917918531
231855	'Ap5z1'	345	344	251	128	322	281	313.3333333	243.6666667	-0.364337177	0.287096614	0.894554788
231858	'Radil'	155	137	81	60	62	120	124.3333333	80.66666667	-0.569660258	0.189839954	0.813027197
231861	'Tnrc18'	4909	4813	1559	2308	6912	5096	3760.333333	4772	0.377730612	0.458200651	0.972790461
231863	'Fbxl18'	882	906	314	469	791	870	700.6666667	710	0.086486118	0.842886983	0.975182082
231866	'Zfp12'	816.71	846	970	547	909	792	877.57	749.3333333	-0.216913218	0.504672104	0.972790461
231868	'E130309D02Rik'	311	328	287	182	409	406	308.6666667	332.3333333	0.09380121	0.770582149	0.972790461
231871	'Daglb'	149	182	96	198	279	370	142.3333333	282.3333333	1.035252353	0.002307875	0.108371515
231872	'Aimp2'	470	436	1341	296	388	577	749	420.3333333	-0.912245224	0.145415952	0.753611197
231874	'Ccz1'	968	1016	976	366	880	1025	986.6666667	757	-0.421509726	0.242275002	0.86449675
231876	'Lmtk2'	813	793	408	1714	1087	1161	671.3333333	1320.666667	1.170761212	0.019873157	0.349283865
231887	'Pdap1'	1449	1536	1975	1239	1385	1877	1653.333333	1500.333333	-0.12262418	0.743434651	0.972790461
231889	'Bud31'	841.41	895.85	1314.2	1047.6	848.36	1146.47	1017.156667	1014.14	0.050243586	0.913304862	0.989773583
231903	'Urad'	0	1	0	0	0	0	0.333333333	0	-0.903279821	0.824807108	0.972790461
231912	'Katnal1'	965	897	343	597	781	697	735	691.6666667	0.027313385	0.950138396	0.998065566
231915	'Uspl1'	1254	1274	1404	712	1036	1105	1310.666667	951	-0.446603942	0.133684389	0.733301819
231931	'Gimap6'	209	212	29	5	304	205	150	171.3333333	0.139026639	0.898340907	0.98586843
231946	'Fam221a'	86	77	178	202	81	123	113.6666667	135.3333333	0.374929336	0.596682214	0.972790461
231986	'Jazf1'	343	302	300	16	208	533	315	252.3333333	-0.477639453	0.586621669	0.972790461
231991	'Creb5'	130	145	1454	240	187	211	576.3333333	212.6666667	-1.492903037	0.131040462	0.728882802
231997	'Fkbp14'	485	561	286	293	690	666	444	549.6666667	0.323774027	0.376015285	0.94050817
231999	'Plekha8'	2117	2111	768	849	1469	1458	1665.333333	1258.666667	-0.333248892	0.431197319	0.963623551
232016	'Itprid1'	0	0	0	0	0	6	0	2	3.261750676	0.410112517	0.957157474
232023	'Vopp1'	701	625	719	236	571	453	681.6666667	420	-0.720688039	0.044898941	0.493464388
232035	'Ccser1'	97	102	243	33	102	74	147.3333333	69.66666667	-1.185108393	0.061342723	0.562479029
232077	'Foxi3'	4	0	1	0	0	0	1.666666667	0	-3.091469523	0.434947663	0.965608974
232078	'Thnsl2'	180	179	189	13	177	164	182.6666667	118	-0.764000918	0.310216804	0.910971113
232086	'Tmem150a'	268	272	253	363	392	261	264.3333333	338.6666667	0.464805745	0.271622982	0.884539076
232087	'Mat2a'	5080	5327	1845	4284	4467	4738	4084	4496.333333	0.275539703	0.544025755	0.972790461
232089	'Elmod3'	277	285	222	300	363	377	261.3333333	346.6666667	0.475337097	0.098708845	0.668509357
232146	'Eva1a'	43	40	21	29	81	45	34.66666667	51.66666667	0.609023618	0.254272429	0.87355031
232156	'Slc4a5'	20	31	5	10	24	36	18.66666667	23.33333333	0.350972158	0.655991322	0.972790461
232157	'Mob1a'	2218	2258	2344	1656	2201	2172	2273.333333	2009.666667	-0.138535473	0.63455997	0.972790461
232164	'Paip2b'	2325	2248	3179	1540	1478	2001	2584	1673	-0.591845115	0.146185945	0.753611197
232174	'Cyp26b1'	191	143	757	219	48	49	363.6666667	105.3333333	-1.599430179	0.108861416	0.68767651
232187	'Smyd5'	819	893	991	225	740	878	901	614.3333333	-0.634146972	0.174195428	0.793056387
232196	'C87436'	306	334	219	201	302	271	286.3333333	258	-0.090871434	0.752858854	0.972790461
232201	'Arhgap25'	67	67	4	7	113	90	46	70	0.58176383	0.584327983	0.972790461
232210	'Hmces'	597	625	639	402	509	627	620.3333333	512.6666667	-0.247500096	0.408198081	0.957157474

232223	'Txnrd3'	499	535	584	25	379	650	539.3333333	351.3333333	-0.782142819	0.350403524	0.92886351
232227	'Iqsec1'	1565	1491	819	1265	1420	1539	1291.666667	1408	0.225677459	0.507655827	0.972790461
232232	'Hdac11'	834	831	845	590	884	1249	836.6666667	907.6666667	0.113726138	0.714163919	0.972790461
232236	'Ccdc174'	433	421	573	249	419	424	475.6666667	364	-0.398954201	0.279520071	0.889355605
232237	'Fgd5'	285	266	125	276	391	326	225.3333333	331	0.651662239	0.096822586	0.663693139
232286	'Tmf1'	1385	1455	1460	906	1238	1310	1433.333333	1151.333333	-0.28478093	0.308041708	0.907963267
232288	'Frmd4b'	1749	1802	1158	1054	1762	1842	1569.666667	1552.666667	0.022580033	0.930245337	0.99325468
232313	'Gxylt2'	2424	2374	1765	1303	2148	2714	2187.666667	2055	-0.073405324	0.781942053	0.972790461
232314	'Ppp4r2'	3721	3839	5499	2224	3148	3630	4352.983333	3000.666667	-0.545199131	0.15501688	0.766453805
232333	'Slc6a1'	1	0	1	0	0	1	0.666666667	0.333333333	-0.894127833	0.804654786	0.972790461
232334	'Vgll4'	1677.3	1730.6	2372	2201.2	1775.2	1692.24	1926.63	1889.536667	0.066682018	0.88829409	0.984577587
232337	'Zfp637'	377	340	415	107	437	356	377.3333333	300	-0.408220316	0.40377969	0.956473894
232339	'Ankrd26'	1243	1295	278	365	821	552	938.6666667	579.3333333	-0.610891754	0.291392144	0.899311502
232341	'Wnk1'	14034	13451	6133	8123	10018	11130	11206	9757	-0.103268056	0.779882274	0.972790461
232345	'A2m'	381	279	767	63	134	94	475.6666667	97	-2.35612986	5.28E-05	0.008506171
232367	'Vmn2r27'	1	1	1	0	4	2	1	2	0.899856303	0.644285956	0.972790461
232370	'Clstn3'	251	192	213	1620	347	427	218.6666667	798	2.170125781	0.006058614	0.18382242
232371	'C1rl'	96	91	54	113	204	250	80.33333333	189	1.257624595	5.43E-04	0.045840415
232400	'A2ml1'	2	2	0	0	2	0	1.333333333	0.666666667	-0.95060596	0.752126908	0.972790461
232406	'BC035044'	2	4	2	0	2	2	2.666666667	1.333333333	-1.058145464	0.524470595	0.972790461
232408	'Klrb1f'	0	0	0	0	2	0	0	0.666666667	1.801491674	0.656112935	0.972790461
232409	'Clec2e'	0	1	0	8	0	0	0.333333333	2.666666667	3.367678366	0.319599887	0.916646777
232413	'Clec12a'	3	5	3	0	14	8	3.666666667	7.333333333	0.892286582	0.518050267	0.972790461
232414	'Clec9a'	1	1	0	3	4	6	0.666666667	4.333333333	2.765769748	0.095707183	0.662411648
232430	'Crebl2'	791	771	356	1934	456	782	639.3333333	1057.333333	1.035787372	0.158488325	0.768890221
232431	'Gprc5a'	112	123	114	205	95	105	116.3333333	135	0.409395429	0.493994982	0.972790461
232440	'H2aj'	241	231	459	811	258	339	310.3333333	469.3333333	0.781297558	0.289161015	0.896355874
232441	'Rerg'	96	108	422	988	96	603	208.6666667	562.3333333	1.555535907	0.118442662	0.707455825
232449	'Dera'	226	255	203	127	241	402	228	256.6666667	0.142518348	0.721276806	0.972790461
232491	'Pyroxd1'	518.86	551.64	346.03	139.33	540.64	437.97	472.1766667	372.6466667	-0.373336499	0.410859833	0.95722888
232493	'Gys2'	1	1	0	0	0	2	0.666666667	0.666666667	-0.01112152	0.997410093	0.999723781
232533	'Stk38l'	904	916	676	1174	880	1060	832	1038	0.451469762	0.260197319	0.878537553
232536	'Mrps35'	388	375	445	152	367	498	402.6666667	339	-0.310817948	0.46493269	0.972790461
232539	'Klhl42'	557	619	432	119	875	973	536	655.6666667	0.195877721	0.756132741	0.972790461
232560	'Caprin2'	6327	6292	1135	411	3666	3278	4584.666667	2451.666667	-0.931916989	0.237390686	0.86298394
232566	'Amn1'	221	204	143	129	200	230	189.3333333	186.3333333	0.014717663	0.960465877	0.999493374
232585	'Vwde'	0	0	0	0	5	3	0	2.666666667	3.723174712	0.276519643	0.888039096
232664	'Ccdc136'	2082	2005	588	463	2148	1446	1558.333333	1352.333333	-0.199761329	0.73978516	0.972790461
232670	'Tspan33'	217	214	212	115	227	291	214.3333333	211	-0.045388186	0.897286709	0.985794067
232679	'Zc3hc1'	761	800	585	544	707	829	715.3333333	693.3333333	0.007131746	0.977397959	0.999493374

232680	'Cpa2'	603	732	108	9	499	194	481	234	-1.083725464	0.304009776	0.906771226
232714	'Mgam'	29	25	63	10	29	11	39	16.66666667	-1.285410778	0.091345651	0.650235019
232748	'Tcaf2'	181	184	50	343	211	195	138.3333333	249.6666667	1.09108461	0.088439218	0.643723745
232784	'Zfp212'	636	662	655	323	622	791	651	578.6666667	-0.193538107	0.558084198	0.972790461
232790	'Oscar'	3	3	0	7	4	2	2	4.333333333	1.394502291	0.356386658	0.931997682
232791	'Cnot3'	1684.9	1662.1	1496.1	951.65	1425.9	1370.14	1614.35	1249.23	-0.333392165	0.174773289	0.793295795
232798	'Leng8'	5027	5008	1542	2416	6175	2537	3859	3709.333333	0.026626263	0.962198029	0.999493374
232801	'Lilra5'	0	1	0	5	0	2	0.333333333	2.333333333	3.002459894	0.27126604	0.884312405
232807	'Ppp1r12c'	967	923	1081	1354	1039	953	990.3333333	1115.333333	0.292809605	0.526969495	0.972790461
232811	'Kmt5c'	1105	1191	769	486	1058	727	1021.666667	757	-0.39899644	0.224628368	0.850759465
232813	'Shisa7'	82	79	22	129	134	64	61	109	1.037711525	0.11530139	0.702526958
232816	'Zfp628'	279	317	273	339	318	315	289.6666667	324	0.260673434	0.488719027	0.972790461
232821	'Ccdc106'	166	177	138	44	189	245	160.3333333	159.3333333	-0.084580879	0.875838453	0.981170271
232827	'Nlrp2'	2	0	1	0	0	1	1	0.333333333	-1.433748416	0.667550462	0.972790461
232853	'Zfp954'	159	161	354	205	173	178	224.6666667	185.3333333	-0.235888561	0.68789575	0.972790461
232854	'Zfp418'	76	77	59	87	58	120	70.66666667	88.33333333	0.408858506	0.385192535	0.946894294
232855	'Zfp772'	176	147	198	293	230	234	173.6666667	252.3333333	0.640784083	0.173716893	0.79261444
232875	'Zscan18'	334	323	145	106	380	367	267.3333333	284.3333333	0.077069733	0.877455702	0.981341203
232878	'Zscan22'	324	300	481	212	345	394	368.3333333	317	-0.245971733	0.556877098	0.972790461
232879	'Zbtb45'	267	261.01	186.01	103	269	288	238.0066667	220	-0.129339498	0.730649044	0.972790461
232889	'Pla2g4c'	62	67	21	26	76	16	50	39.33333333	-0.25870621	0.729713994	0.972790461
232906	'Arhgap35'	4804	4831	2246	1864	3703	3600	3960.333333	3055.666667	-0.331335258	0.3608955	0.933706718
232910	'Ap2s1'	675	762	844	764	726	958	760.3333333	816	0.155159049	0.677183063	0.972790461
232933	'Ccdc61'	452	491	307	207	425	658	416.6666667	430	0.031225932	0.936709576	0.994413066
232934	'Mypop'	160	165	119	199	171	180	148	183.3333333	0.429483928	0.284666583	0.893377528
232941	'Ppm1n'	1	1	1	2	1	0	1	1	0.22872257	0.914703118	0.989989467
232943	'Klc3'	83.84	99.43	80.81	153.72	34.36	81.34	88.02666667	89.80666667	0.269851148	0.712046006	0.972790461
232944	'Mark4'	1246	1226	1668	2310	1470	1834	1380	1871.333333	0.549431994	0.269156601	0.883851074
232946	'Bloc1s3'	215	266	419	326	273	290	300	296.3333333	0.042742841	0.934650385	0.994413066
232947	'Ppp1r37'	1366	1417	1227	1199	1587	1671	1336.666667	1485.666667	0.200006121	0.424118354	0.960285372
232966	'Zfp114'	69	80	11	24	82	50	53.33333333	52	0.02024209	0.978553427	0.999493374
232969	'Zfp428'	290	289	389	169	318	352	322.6666667	279.6666667	-0.23869396	0.537017492	0.972790461
232970	'Phldb3'	82	73	123	188	33	96	92.66666667	105.6666667	0.39449364	0.625324259	0.972790461
232972	'Lypd10'	70	66	47	0	0	1	61	0.333333333	-7.336112652	1.90E-08	1.29E-05
232973	'Lypd4'	63	57	27	0	33	28	49	20.33333333	-1.364589202	0.202463175	0.830701895
232974	'Erf1'	1	0	0	0	0	1	0.333333333	0.333333333	0.058500858	0.988561293	0.999493374
232975	'Atp1a3'	9	11	21	51	7	19	13.66666667	25.66666667	1.131872901	0.263803658	0.880205046
232976	'Zfp574'	693	638	704	676	698	718	678.3333333	697.3333333	0.110844488	0.752597209	0.972790461
232983	'Cxcl17'	0	0	0	7	1	0	0	2.666666667	4.266713998	0.196968719	0.822696823
232984	'B3gnt8'	43	42	6	12	62	64	30.33333333	46	0.600460492	0.471738172	0.972790461

232987	'B9d2'	129	117	175	153	142	117	140.3333333	137.3333333	0.049495902	0.920547943	0.991047523
232989	'Hnrnpul1'	5373	5437	6386	3412	4187	4420	5732	4006.333333	-0.481274024	0.144172948	0.750780289
233001	'Nlrp9a'	0	0	0	0	0	1	0	0.333333333	1.020273531	0.802557913	0.972790461
233005	'Cyp2a22'	0	0	0	0	1	0	0	0.333333333	1.020273531	0.802557913	0.972790461
233011	'Itpkc'	336.72	352.31	608.59	442.77	385.22	478.17	432.54	435.3866667	0.048617383	0.922318302	0.991300958
233016	'Blvrb'	299	304	405	687	350	647	336	561.3333333	0.844820276	0.112183546	0.697004434
233020	'Hipk4'	15	29	17	51	39	18	20.33333333	36	1.014372985	0.167882637	0.782491958
233033	'Samd4b'	2077	2001	2471	3757	1581	2213	2183	2517	0.379434073	0.516255427	0.972790461
233038	'Nccrp1'	1	0	2	53	0	4	1	19	4.60718091	0.020663805	0.356497521
233040	'Fbxo27'	60	90	39	49	107	88	63	81.33333333	0.40637797	0.364599388	0.935515019
233046	'Rasgrp4'	188.11	169.8	119.02	47.46	196.84	135.02	158.9766667	126.44	-0.360908652	0.459486222	0.972790461
233056	'Zfp790'	493.6	525.94	598.01	419.06	643	611	539.1833333	557.6866667	0.064305065	0.843044399	0.975182082
233057	'Zfp940'	141	161.01	79.2	157	106	141	127.07	134.6666667	0.241313013	0.617848519	0.972790461
233058	'Zfp420'	291.01	246	182	204	314.02	217	239.67	245.0066667	0.106407212	0.763237057	0.972790461
233060	'Zfp382'	467	417	563	345	393	339	482.3333333	359	-0.370093559	0.357464255	0.932505998
233064	'Wdr62'	303	303	122	73	290	274	242.6666667	212.3333333	-0.206163166	0.701298178	0.972790461
233065	'Alkbh6'	161	173	341	103	215	241	225	186.3333333	-0.35123598	0.50841549	0.972790461
233066	'Syne4'	79	78	94	83	107	86	83.66666667	92	0.185119203	0.658259265	0.972790461
233067	'Lrfn3'	164	181	135	263	266	236	160	255	0.779664787	0.039133389	0.462320279
233071	'Arhgap33'	463	373	322	12	247	213	386	157.3333333	-1.415132677	0.080267932	0.617305615
233073	'U2af1l4'	224	240	225	80	256	247	229.6666667	194.3333333	-0.291123852	0.493395933	0.972790461
233079	'Ffar2'	2	1	8	0	2	8	3.666666667	3.333333333	-0.398657762	0.814251971	0.972790461
233080	'Ffar3'	1	0	0	0	0	1	0.333333333	0.333333333	0.058500858	0.988561293	0.999493374
233103	'Garre1'	2679	2500	2282	2917	2158	2177	2487	2417.333333	0.097993911	0.820360126	0.972790461
233107	'Kctd15'	474	474	533	58	437	468	493.6666667	321	-0.746240276	0.246273272	0.868334735
233115	'Dpy19l3'	644	639	265	496	811	929	516	745.3333333	0.590355838	0.132241947	0.729657304
233147	'Zfp939'	180.41	175.11	201.44	49	147.93	118	185.6533333	104.9766667	-0.867768671	0.043971556	0.488595692
233168	'AI987944'	231.78	273.87	258.81	202.7	291.23	241.7	254.82	245.21	-0.010490676	0.974876488	0.999493374
233186	'Siglecf'	28	14	10	20	13	10	17.33333333	14.33333333	-0.082382223	0.91677251	0.990536123
233189	'Ctu1'	278	239	429	202	289	354	315.3333333	281.6666667	-0.18802414	0.673914472	0.972790461
233199	'Mybpc2'	9	17	14	9	4	6	13.33333333	6.333333333	-0.944720932	0.276804543	0.888503794
233204	'Tbc1d17'	619	624	1044	407	779	746	762.3333333	644	-0.28704372	0.516522383	0.972790461
233208	'Scaf1'	2459	2414	2068	2159	2472	2351	2313.666667	2327.333333	0.087918821	0.768873295	0.972790461
233210	'Prr12'	1749	1669	1133	694	2021	1681	1517	1465.333333	-0.059558724	0.872707155	0.980171118
233222	'Mrgpra3'	0	0	0	0	1	1	0	0.666666667	1.775692139	0.660844521	0.972790461
233246	'Ano5'	144	130	6	18	148	68	93.33333333	78	-0.237484819	0.81485489	0.972790461
233271	'Luzp2'	11	14	5	9	38	27	10	24.66666667	1.299192141	0.080310522	0.617305615
233274	'Siglech'	0	0	0	1	2	0	0	1	2.548734582	0.523314857	0.972790461
233276	'Tubgcp5'	1780	1791	497	728	1301	1196	1356	1075	-0.25125023	0.613151742	0.972790461
233280	'Nipa1'	154	171	83	94	204	160	136	152.6666667	0.208697125	0.595378197	0.972790461

233315 'Mtmr10'	805	708	420	551	700	638	644.3333333	629.6666667	0.061610033	0.855828969	0.975734242
233328 'Lrrk1'	837	827	440	359	949	847	701.3333333	718.3333333	0.044741568	0.907831252	0.988823459
233332 'Adamts17'	484	439	20	32	391	164	314.3333333	195.6666667	-0.678464934	0.511604694	0.972790461
233335 'Synm'	256	268	89	36	204	121	204.3333333	120.3333333	-0.77211818	0.220698483	0.849076011
233405 'Vps33b'	440	427	222	316	402	524	363	414	0.258932059	0.453666876	0.970649024
233406 'Prc1'	577	621	252	445	760	1196	483.3333333	800.3333333	0.755977184	0.082168246	0.622135682
233410 'Zfp592'	1435	1359	1371	1322	1408	1428	1388.333333	1386	0.071259316	0.827735187	0.973238667
233424 'Tmc3'	292	288	25	39	216	243	201.6666667	166	-0.280005321	0.746950994	0.972790461
233489 'Picalm'	3572	3620	8124	6459	3582	4663	5105.333333	4901.333333	0.026658254	0.966309575	0.999493374
233490 'Crebzf'	1844	1925	2432	2857	2013	1670	2067	2180	0.210920987	0.684640998	0.972790461
233529 'Kctd14'	11077	12047	13198	355	15945	8570	12107.33333	8290	-0.698991577	0.450775197	0.970590732
233532 'Rsf1'	2393	2541	2532	1614	2732	2665	2488.666667	2337	-0.080963862	0.768428832	0.972790461
233537 'Gdpd4'	24	33	3	0	28	21	20	16.33333333	-0.340611194	0.795931104	0.972790461
233545 'Emsy'	1452	1487	1572	1088	1436	1163	1503.666667	1229	-0.236895117	0.478459776	0.972790461
233549 'Mogat2'	9	5	11	15	9	14	8.333333333	12.66666667	0.681789586	0.412200612	0.95722888
233552 'Gdpd5'	335	329	434	97	378	311	366	262	-0.565640176	0.254736747	0.874390569
233571 'P2ry6'	57	37	563	34	56	56	219	48.66666667	-2.326731517	0.023558674	0.380103384
233575 'Pgap2'	797	929	995	456	1306	1344	907	1035.333333	0.130308282	0.753230291	0.972790461
233649 'Cnga4'	39	42	2	7	29	6	27.66666667	14	-0.888059328	0.419978949	0.958308933
233651 'Dchs1'	1741	1609	461	1293	2142	1741	1270.333333	1725.333333	0.544238341	0.28054975	0.889355605
233724 'Tmem41b'	832	955	1341	1410	1098	1267	1042.666667	1258.333333	0.348163661	0.468065184	0.972790461
233726 'Ipo7'	3893	3954	6299	4646	3707	4323	4715.333333	4225.333333	-0.091973879	0.849595527	0.975734242
233733 'Galnt18'	97	105	255	78	137	142	152.3333333	119	-0.426726706	0.471589676	0.972790461
233744 'Spon1'	66	61	22	1047	111	505	49.66666667	554.3333333	3.810074895	1.56E-05	0.00358374
233752 'Insc'	9	9	15	46	16	14	11	25.33333333	1.414699045	0.116658562	0.704546565
233765 'Plekha7'	263	233	93	372	310	354	196.3333333	345.3333333	0.973048755	0.047112661	0.50504201
233781 'Xylt1'	135	140	29	171	204	159	101.3333333	178	0.970751746	0.10980088	0.689773582
233789 'Smg1'	8390	8680	3174	12437	6199	5659	6748	8098.333333	0.527444981	0.401356305	0.955860942
233799 'Acsm2'	1	0	0	0	0	0	0.333333333	0	-0.903279821	0.824807108	0.972790461
233802 'Thumpd1'	1632	1680	1906	472	1513	1747	1739.333333	1244	-0.561796218	0.214112152	0.842446242
233805 'Dcun1d3'	591	582	695	863	390	625	622.6666667	626	0.15723944	0.77590589	0.972790461
233806 'Tmem159'	175	181	439	298	221	308	265	275.6666667	0.081786542	0.894012193	0.985171
233810 'Abca16'	59	57	4	0	34	65	40	33	-0.343027131	0.80154021	0.972790461
233812 'Mosmo'	657	658	816	990	606	708	710.3333333	768	0.241671949	0.632214543	0.972790461
233813 'Vwa3a'	14	11	20	5	11	4	15	6.666666667	-1.167671179	0.166212553	0.780893009
233824 'Cog7'	715	772	402	340	578	832	629.6666667	583.3333333	-0.087444396	0.809018966	0.972790461
233826 'Palb2'	469	450	96	104	271	294	338.3333333	223	-0.561072047	0.357446821	0.932505998
233833 'Tnrc6a'	3849	3772	1984	2545	3675	2779	3201.666667	2999.666667	0.002043029	0.995424022	0.999562152
233836 'Slc5a11'	1	4	0	2	1	2	1.666666667	1.666666667	0.191541467	0.918569729	0.990815958
233863 'Gtf3c1'	4923	4767	2802	5263	4936	4549	4164	4916	0.377901097	0.334525966	0.921767653

233865	'Katnip'	1157	1170	606	1324	1077	868	977.6666667	1089.666667	0.334100103	0.481169184	0.972790461
233870	'Tufm'	1419	1475	1742	1680	1323	1878	1545.333333	1627	0.146885643	0.721464399	0.972790461
233871	'Atxn2l'	3459	3117	7703	3374	3250	2998	4759.666667	3207.333333	-0.543134679	0.34592534	0.927022024
233875	'Ino80e'	657.21	729.17	857.2	430.12	632.47	725.13	747.86	595.9066667	-0.327017758	0.334623543	0.921773568
233876	'Hirip3'	717	721	450	138	508	404	629.3333333	350	-0.869765838	0.048306806	0.510510842
233877	'Kctd13'	340	415	366	280	311	340	373.6666667	310.3333333	-0.20643235	0.527465547	0.972790461
233878	'Sez6l2'	83	104	23	20	82	73	70	58.33333333	-0.252564791	0.707093302	0.972790461
233879	'Asphd1'	9	13	10	4	7	3	10.66666667	4.666666667	-1.137177893	0.197121971	0.822696823
233887	'Zfp553'	900	825	1031	510	820	1141	918.6666667	823.6666667	-0.181623446	0.610051013	0.972790461
233890	'Zfp768'	615	620	555	305	549	622	596.6666667	492	-0.276438246	0.317294491	0.916136994
233893	'Zfp764'	152.15	143.91	217.54	21	190	166.39	171.2	125.7966667	-0.585900534	0.40414994	0.956543098
233895	'Prr14'	1201.4	1230.1	899.49	447.73	1415.9	974.41	1110.333333	946.0233333	-0.245609935	0.529715306	0.972790461
233899	'Ccdc189'	154.1	142.07	41.41	15.44	148.48	122.64	112.5266667	95.52	-0.283209492	0.71403604	0.972790461
233900	'Rnf40'	1612	1547	1398	1446	1791	2143	1519	1793.333333	0.284892964	0.270405802	0.883851074
233902	'Fbxl19'	1494	1517	1513	621	1658	1473	1508	1250.666667	-0.304199067	0.395544071	0.954014376
233904	'Setd1a'	2986	2902	1620	1993	2455	2000	2502.666667	2149.333333	-0.109631818	0.758413717	0.972790461
233905	'Zfp646'	2073.8	2082.1	794.02	934.99	1540.8	1552.13	1649.95	1342.636667	-0.224608893	0.581389237	0.972790461
233908	'Fus'	7704	8372	3392	2498	6780	4949	6489.333333	4742.333333	-0.42036831	0.334005666	0.921648675
233912	'Armc5'	494.2	501.97	281.28	150.74	557.2	366.83	425.8166667	358.2566667	-0.258271589	0.573478294	0.972790461
233913	'Rusf1'	453.65	507.87	218	212.9	729.6	644.48	393.1733333	528.9933333	0.423044161	0.373456503	0.93929675
233977	'Ppfia1'	1584	1519	1237	1465	1813	1651	1446.666667	1643	0.259490864	0.364987883	0.935765447
233979	'Tpcn2'	190	190	66	79	251	119	148.6666667	149.6666667	0.05683994	0.919214267	0.990815958
233987	'Zfp958'	675	737	450	648	876	780	620.6666667	768	0.387335192	0.195071737	0.819942118
234023	'Arglu1'	1498	1466	570	1299	1718	1087	1178	1368	0.352935305	0.453138109	0.970649024
234069	'Pcid2'	919	835	1232	548	733.91	739	995.3333333	673.6366667	-0.550915467	0.151115469	0.75985382
234072	'Adprhl1'	1	3	0	0	3	5	1.333333333	2.666666667	0.955412916	0.649987779	0.972790461
234076	'Tmco3'	579.53	626.04	349	1008	857	836.28	518.19	900.4266667	0.945559853	0.023133702	0.377626024
234086	'Erich1'	160	135	173	98	137	174	156	136.3333333	-0.190250547	0.604562102	0.972790461
234094	'Arhgef10'	679	658	1227	766	654	809	854.6666667	743	-0.163253889	0.747985763	0.972790461
234129	'Tpte'	0	0	0	0	1	1	0	0.666666667	1.775692139	0.660844521	0.972790461
234130	'Dkk4'	1	0	1	0	0	2	0.666666667	0.666666667	-0.156033058	0.963274716	0.999493374
234135	'Nsd3'	2437	2208	2371	3102	2193	2205	2338.666667	2500	0.23164135	0.612799442	0.972790461
234138	'Tti2'	340	361.23	497.47	257.2	338	425.48	399.5666667	340.2266667	-0.235228854	0.549230446	0.972790461
234155	'Mboat4'	8	5	1	0	2	1	4.666666667	1	-2.208199649	0.18946784	0.812029435
234199	'Fgl1'	4	5	3	3	8	18	4	9.666666667	1.225065752	0.224466256	0.850759465
234214	'Sorbs2'	2843	2942	1579	934	4769	3871	2454.666667	3191.333333	0.336151983	0.524692497	0.972790461
234219	'Helt'	1	1	0	0	2	0	0.666666667	0.666666667	0.038821536	0.990916081	0.999493374
234258	'Neil3'	137	130	25	1	94	55	97.33333333	50	-1.018133023	0.354534329	0.931809687
234267	'Gpm6a'	182	162	42	350	454	684	128.6666667	496	2.033035794	1.91E-04	0.021798792
234290	'BC030500'	0	1	0	0	1	1	0.333333333	0.666666667	0.817382893	0.82412705	0.972790461

234309	'Cbr4'	227	219	131	44	280	228	192.3333333	184	-0.122053202	0.836183504	0.974723675
234311	'Ddx60'	117	117	102	19	147	294	112	153.3333333	0.323091684	0.669318648	0.972790461
234329	'Trim60'	0	0	2	10	0	13	0.666666667	7.666666667	3.511157572	0.119124608	0.709078602
234344	'Naf1'	502	487	921	534	567	704	636.6666667	601.6666667	-0.0769527	0.8741633	0.980171118
234353	'Psd3'	1101	1060	699	1012	1163	1036	953.3333333	1070.333333	0.269430478	0.413904484	0.95722888
234356	'Csgalnact1'	1272	1286	118	149	2238	1794	892	1393.666667	0.615084193	0.517044154	0.972790461
234358	'Zfp930'	451	461	240	268	406	250	384	308	-0.216869619	0.592566173	0.972790461
234362	'Zfp868'	513.53	534.58	565.99	184	525	384	538.0333333	364.3333333	-0.594146901	0.130907264	0.728882802
234365	'Yjefn3'	11	10	11	2	20	23	10.66666667	15	0.383066481	0.670086211	0.972790461
234366	'Gatad2a'	1954	1855	1709	2175	1700	2514	1839.333333	2129.666667	0.303492266	0.415090564	0.95722888
234371	'Tmem161a'	550	564	534	412	693	651	549.3333333	585.3333333	0.110601381	0.681943917	0.972790461
234373	'Sugp2'	1462.7	1471	345.24	286	1159.8	775	1092.976667	740.25	-0.538123033	0.385386284	0.946987408
234374	'Ddx49'	754.66	692.54	880.71	491.63	654.48	982.33	775.97	709.48	-0.137849533	0.703100051	0.972790461
234378	'Klhl26'	541	601	570	260	707	632	570.6666667	533	-0.133291274	0.718221755	0.972790461
234384	'Mpv17l2'	181	153	135	176	136	207	156.3333333	173	0.241016733	0.550716292	0.972790461
234388	'Ccdc124'	498	538	387	641	480	619	474.3333333	580	0.415121734	0.29706595	0.900647119
234395	'Ushbp1'	153	138	22	3	198	105	104.3333333	102	-0.083016678	0.939556505	0.995607401
234396	'Ankle1'	94	118	14	15	135	85	75.33333333	78.33333333	0.049888251	0.954380501	0.999486729
234404	'Nxnl1'	33	34	4	10	26	20	23.66666667	18.66666667	-0.26847228	0.749531572	0.972790461
234407	'Colgalt1'	1697	1685	1017	1901	2186	2255	1466.333333	2114	0.623014726	0.046756117	0.503641528
234413	'Zfp961'	364	358	146	138	348	368	289.3333333	284.6666667	-0.004630589	0.991865285	0.999493374
234421	'Cib3'	0	0	0	0	1	0	0	0.333333333	1.020273531	0.802557913	0.972790461
234463	'Tmem184c'	438	466	444	432	591	573	449.3333333	532	0.285341606	0.318830738	0.916136994
234515	'Inpp4b'	87	70	232	23	69	85	129.6666667	59	-1.283778736	0.070068891	0.589465353
234542	'Rtbdn'	5	1	1	21	2	8	2.333333333	10.33333333	2.471322507	0.073769046	0.599788529
234549	'Heatr3'	1061	1103	716	291	990	1133	960	804.6666667	-0.296386067	0.50718693	0.972790461
234564	'Ces1f'	1	2	0	9	0	2	1	3.666666667	2.314039209	0.279481981	0.889355605
234577	'Cpne2'	122	96	212	323	134	231	143.3333333	229.3333333	0.792488021	0.232573461	0.859679981
234582	'Ccdc102a'	290	287	190	181	434	377	255.6666667	330.6666667	0.380472368	0.263302581	0.88013169
234593	'Ndrg4'	374	309	460	975	415	1314	381	901.3333333	1.309581333	0.023159686	0.377626024
234594	'Cnot1'	12405	12679	4805	4827	12834	12155	9963	9938.543333	0.019259738	0.966106576	0.999493374
234595	'Slc38a7'	405	408	531	459	625	742	448	608.6666667	0.445851056	0.213031726	0.841028891
234663	'Dync1li2'	2781	2694	3184	1481	3295	2750	2886.333333	2508.666667	-0.224715553	0.51739249	0.972790461
234664	'Nae1'	1773	1856	1122	295	1400	1580	1583.666667	1091.666667	-0.597667362	0.261969581	0.879908021
234669	'Ces2b'	39.42	25.93	7.04	34.6	41.79	15.33	24.13	30.57333333	0.532401422	0.509031757	0.972790461
234671	'Ces2c'	0	2	0	1	2	0	0.666666667	1	0.780671449	0.803880134	0.972790461
234673	'Ces2e'	1	0	1	0	1	1	0.666666667	0.666666667	-0.113177234	0.967544413	0.999493374
234677	'Ces4a'	2	3	0	0	0	1	1.666666667	0.333333333	-2.058912803	0.492510925	0.972790461
234678	'D230025D16Rik'	1042.9	1105.4	741.73	1682.8	1426.9	1494.79	963.3266667	1534.81	0.803496596	0.036443535	0.457518227
234683	'Elmo3'	203	220	144	125	240	264	189	209.6666667	0.166302861	0.596847074	0.972790461

234684	'Lrrc29'	30	43	3	6	38	33	25.33333333	25.66666667	0.028220979	0.976886025	0.999493374
234686	'Fhod1'	411	412	224	363	529	378	349	423.3333333	0.373313054	0.31188633	0.911537035
234695	'Carmil2'	18	16	17	20	28	61	17	36.33333333	1.072736597	0.071749754	0.592919704
234699	'Edc4'	2038	1975	1045	1306	1826	1715	1686	1615.666667	0.022381553	0.945481602	0.996592403
234700	'Nrn1l'	3	0	1	1	2	6	1.333333333	3	1.118961142	0.529481948	0.972790461
234723	'Txnl4b'	245	227	210	46	232	241	227.3333333	173	-0.476575713	0.380981206	0.944233436
234724	'Tat'	12	9	0	58	7	2	7	22.33333333	2.185747426	0.161136913	0.771435531
234725	'Zfp612'	1288	1243	515	604	1030	853	1015.333333	829	-0.212487518	0.596541311	0.972790461
234728	'Cmtr2'	218	229	331	163	275	312	259.3333333	250	-0.081939563	0.84205444	0.975182082
234729	'Vac14'	727	798	434	394	770	802	653	655.3333333	0.033771401	0.916253325	0.990526893
234730	'Fcsk'	372	593	173	594	404	425	379.3333333	474.3333333	0.526174573	0.351516991	0.929144534
234733	'Ddx19b'	977.43	922.32	859.7	205.74	814.28	905.86	919.8166667	641.96	-0.593500365	0.217569921	0.844165262
234734	'Aars'	1929.1	2017.6	4918	2207.9	2448.4	3301.84	2954.91	2652.73	-0.18918518	0.738756932	0.972790461
234736	'Rfwd3'	3832	3845	2072	2361	3266	3547	3249.666667	3058	-0.015979471	0.958366357	0.999493374
234740	'Tmem231'	429	419	225	273	350	386	357.6666667	336.3333333	-0.008580153	0.979470741	0.999493374
234776	'Atmin'	1130	1067	772	655	932	1240	989.6666667	942.3333333	-0.036831427	0.891850985	0.985171
234779	'Plcg2'	126	103	72	81	164	574	100.3333333	273	1.372483263	0.036174998	0.456982767
234788	'Slc38a8'	2	3	1	0	2	20	2	7.333333333	1.748405292	0.305586184	0.907188578
234796	'Klhl36'	173	170	139	218	251	250	160.6666667	239.6666667	0.6526129	0.041275893	0.475995577
234797	'6430548M08Rik'	522	487	973	662	279	419	660.6666667	453.3333333	-0.415874158	0.523544367	0.972790461
234814	'Mthfsd'	568	638	238	292	609	456	481.3333333	452.3333333	-0.026053915	0.951733703	0.998640168
234825	'Klhdc4'	269	284	251	274	448	480	268	400.6666667	0.597519576	0.026121266	0.397137149
234836	'Il17c'	2	3	2	3	5	4	2.333333333	4	0.820852639	0.494186149	0.972790461
234839	'Piezo1'	1527.6	1452.4	879.39	556.78	1866.8	1816.25	1286.483333	1413.26	0.111016601	0.796970018	0.972790461
234847	'Spg7'	769	774	307	688	884	703	616.6666667	758.3333333	0.421258549	0.329316831	0.921648675
234852	'Chmp1a'	1791	1864	1530	1160	1638	2142	1728.333333	1646.666667	-0.045363244	0.858218889	0.976475731
234854	'Cdk10'	533	614	325	241	689	578	490.6666667	502.6666667	0.037644654	0.924314331	0.992099817
234857	'Spire2'	130	152	66	269	175	223	116	222.3333333	1.113635354	0.028473019	0.410671038
234865	'Nup133'	2445	2298	668	594	1434	1741	1803.666667	1256.333333	-0.492437965	0.354353362	0.931678345
234875	'Ttc13'	484	521	187	611	698	664	397.3333333	657.6666667	0.856014827	0.054260581	0.534951117
234878	'Map3k21'	79	88	537	970	191	320	234.6666667	493.6666667	1.218061303	0.236969734	0.862272407
234889	'Gucy1a2'	321	326	359	152	433	486	335.3333333	357	0.029117164	0.945111371	0.996592403
234911	'Mmp27'	2	0	2	2	0	2	1.333333333	1.333333333	0.098725447	0.964894288	0.999493374
234912	'Cfap300'	497	505	338	162	276	333	446.6666667	257	-0.773693636	0.007899838	0.214208031
234915	'Cep126'	471	517	169	269	433	249	385.6666667	317	-0.162716081	0.745961581	0.972790461
234959	'Med17'	1400	1504	763	657	1232	1954	1222.333333	1281	0.073220316	0.855651686	0.975734242
234964	'Deup1'	74	46	7	0	14	11	42.33333333	8.333333333	-2.381293785	0.041583761	0.476655631
234967	'Slc36a4'	1140	1148	1063	2278	928	1283	1117	1496.333333	0.622905388	0.281071668	0.889731741
234988	'Mbd3l2'	4	2	3	0	2	0	3	0.666666667	-2.204501624	0.278415378	0.889355605
235028	'Zfp426'	895	918	753	333	939	861	855.3333333	711	-0.293573321	0.416088953	0.95722888

235036 'Ppan'	528	528	975	337	468	650	677	485	-0.522553209	0.273967696	0.885226688
235040 'Atg4d'	221.47	193.59	257.59	221.14	283.12	336.4	224.2166667	280.22	0.340158211	0.347005592	0.928289998
235041 'Kank2'	2028	2008	1723	832	2373	2348	1919.666667	1851	-0.088168261	0.811079314	0.972790461
235043 'Tmem205'	135	156	142	110	216	301	144.3333333	209	0.506681467	0.180965266	0.801479605
235044 'Plppr2'	309	363	265	453	543	367	312.3333333	454.3333333	0.644285293	0.096447781	0.663111432
235047 'Zfp809'	208	243	188	320	243	216	213	259.6666667	0.433675562	0.349448561	0.92886351
235048 'Zfp599'	85	107	83	84	97	81	91.66666667	87.33333333	0.012706963	0.974478441	0.999493374
235050 'Zfp810'	306	355	175	185	342	283	278.6666667	270	0.009920843	0.977498153	0.999493374
235072 'Septin7'	3795	3880	6174	2495	4082	5033	4616.333333	3870	-0.295505084	0.485408913	0.972790461
235086 'Igsf9b'	390	372	80	813	235	264	280.6666667	437.3333333	0.99004361	0.22762398	0.854207358
235106 'Ntm'	14	9	3	5	7	7	8.666666667	6.333333333	-0.359469011	0.696679672	0.972790461
235130 'Adamts15'	98	82	20	15	103	48	66.66666667	55.33333333	-0.264665133	0.732665063	0.972790461
235132 'Zbtb44'	2261	2307.9	1895	946	1659	1872	2154.63	1492.333333	-0.519500063	0.038307548	0.459930654
235134 'Nfrkb'	1211	1125	870	762	1115	945	1068.666667	940.6666667	-0.121764	0.655674605	0.972790461
235135 'Tmem45b'	125.76	93.73	43.19	101.03	73.92	46.63	87.56	73.86	-0.026022797	0.967442152	0.999493374
235169 'Foxred1'	777	769	333	360	707	620	626.3333333	562.3333333	-0.100056334	0.797837292	0.972790461
235180 'Fez1'	134	143	171	44	259	267	149.3333333	190	0.230908915	0.704253207	0.972790461
235184 'Msantd2'	1467	1426	746	871.42	1527	831.16	1213	1076.526667	-0.081371441	0.84459939	0.975182082
235281 'Scn3b'	52	64	18	20	78	42	44.66666667	46.66666667	0.091773931	0.887924774	0.984534762
235283 'Gramd1b'	359.16	370.65	376.85	1910.8	826.32	852.57	368.8866667	1196.546667	1.910190281	0.00170574	0.089092131
235293 'Sc5d'	331	397	353	1334	527	683	360.3333333	848	1.444075723	0.016800594	0.323948168
235300 'Tlcd5'	265	271	176	336	346	233	237.3333333	305	0.499847209	0.251217271	0.871230154
235312 'C1qtnf5'	20.85	17.09	8.7	11.77	37.49	114.75	15.54666667	54.67	1.775880689	0.032140566	0.433766761
235315 'Rnf214'	2501.1	2505	1539.8	1495.6	2215.8	1847.73	2181.936667	1853.033333	-0.16083093	0.588003308	0.972790461
235320 'Zbtb16'	26	27	32	43	98	76	28.33333333	72.33333333	1.338999202	0.004257449	0.151577956
235323 'Usp28'	1345	1391	1119	733	1463	1309	1285	1168.333333	-0.12445866	0.640428265	0.972790461
235330 'Ttc12'	169	183	79	197	205	205	143.6666667	202.3333333	0.620102672	0.147105034	0.753843909
235339 'Dlat'	1774	1890	1519	1019	1475	2007	1727.666667	1500.333333	-0.184853498	0.487320207	0.972790461
235344 'Sik2'	1634	1706.4	1328.1	3007.7	1222.8	1682.84	1556.146667	1971.093333	0.554132789	0.335548119	0.922413343
235345 'Hoatz'	4	7	3	0	3	1	4.666666667	1.333333333	-1.834515712	0.227320388	0.854001521
235379 'Gldn'	201	209	25	52	170	90	145	104	-0.407187637	0.591291753	0.972790461
235380 'Dmxl2'	2245	2365	842	244	1685	1549	1817.333333	1159.333333	-0.692448193	0.285694755	0.893820325
235386 'Hykk'	232	230	101	53	195	168	187.6666667	138.6666667	-0.442084119	0.384900353	0.94674509
235402 'Lingo1'	138	119	206	32	125	108	154.3333333	88.33333333	-0.90398416	0.106437946	0.679124905
235406 'Snx33'	626	644	612	639	956	922	627.3333333	839	0.451029381	0.090486221	0.649178882
235415 'Cplx3'	3	3	1	0	2	1	2.333333333	1	-1.239715923	0.49850004	0.972790461
235416 'Lman1l'	0	1	0	0	0	0	0.333333333	0	-0.903279821	0.824807108	0.972790461
235431 'Coro2b'	161	184	294	57	246	349	213	217.3333333	-0.111830406	0.857921953	0.976475731
235435 'Lctl'	9.13	7.03	1	2	18.06	10.05	5.72	10.03666667	0.816147675	0.474139301	0.972790461
235439 'Herc1'	7434.7	7015.6	1598.5	4676.7	5039.6	4845.29	5349.593333	4853.873333	0.018274992	0.974426969	0.999493374

235441	'Usp3'	2125	2120	3250	4474	1795.9	2247	2498.333333	2838.966667	0.357807515	0.575746626	0.972790461
235442	'Rab8b'	1076	1040	1386	1212	970	1226	1167.333333	1136	0.035259038	0.935161539	0.994413066
235459	'Gtf2a2'	809	845	540	643	726	677	731.3333333	682	-2.84E-04	0.999305051	0.999900097
235461	'Mindy2'	1091	1079	2002	1125	984	1282	1390.666667	1130.333333	-0.269355402	0.591450109	0.972790461
235469	'Zfp280d'	1146	1219	1218	306	1290	779	1194.333333	791.6666667	-0.650739334	0.172893706	0.790917985
235472	'Prtg'	304	357	99	23	179	355	253.3333333	185.6666667	-0.517606121	0.521222302	0.972790461
235493	'Fam214a'	1536	1649	604	1235	2380	1980	1263	1865	0.630489436	0.134193862	0.733437989
235497	'Leo1'	923	873	912	427	897.03	956	902.6666667	760.01	-0.269506163	0.398530675	0.954563638
235504	'Slc17a5'	251	245	168	718	359	350	221.3333333	475.6666667	1.325702156	0.021103793	0.360814128
235505	'Cd109'	104	92	95	28	104	123	97	85	-0.260815036	0.612045757	0.972790461
235527	'Plscr4'	274	275	119	239	284	193	222.6666667	238.6666667	0.239610179	0.605298056	0.972790461
235533	'Gk5'	722	858	793	4749	991	1194	791	2311.333333	1.852698915	0.019478608	0.347725165
235534	'Pxylp1'	189	171	80	277	190	257	146.6666667	241.3333333	0.881529412	0.074090391	0.601711452
235542	'Ppp2r3a'	2059	2031	1754	1326	1752	1801	1948	1626.333333	-0.208769655	0.400774539	0.955860942
235559	'Topbp1'	5536	5616	934	1224	3111	2380	4028.666667	2238.333333	-0.776463298	0.222001289	0.849517424
235567	'Dnajc13'	3850	3864	1665	2319	3339	2911	3126.333333	2856.333333	-0.033955486	0.929254548	0.993096978
235574	'Atp2c1'	2075	2109	824	3351	2403	2890	1669.333333	2881.333333	0.965383266	0.050511039	0.519548742
235582	'Glyctk'	212	228	24	19	112	80	154.6666667	70.33333333	-1.121003605	0.179194605	0.798783498
235584	'Dusp7'	384	440	754	289	489	799	526	525.6666667	-0.078626685	0.877259114	0.981341203
235587	'Parp3'	165	159	294	259	150	250	206	219.6666667	0.162493781	0.777565723	0.972790461
235599	'6430571L13Rik'	41	43	10	6	27	19	31.33333333	17.33333333	-0.832518783	0.277272036	0.88901132
235604	'Camkv'	53	41	9	26	31	58	34.33333333	38.33333333	0.240614082	0.73242985	0.972790461
235606	'Apeh'	909	936	1021	710	1058	1553	955.3333333	1107	0.199418047	0.553623233	0.972790461
235610	'Atrip'	410	377	339	112	437	380	375.3333333	309.6666667	-0.331950202	0.465864231	0.972790461
235611	'Plxnb1'	2421	2580	414	170	3222	1781	1805	1724.333333	-0.102073735	0.909802628	0.989364653
235623	'Scap'	1954	2000	1703	1778	2861	3080	1885.666667	2573	0.470028908	0.043235296	0.484697385
235626	'Setd2'	4523	4463	2904	4358	4296	4309	3963.333333	4321	0.241035687	0.486799945	0.972790461
235627	'Nbeal2'	256	260	60	325	394	299	192	339.3333333	0.977777576	0.090044572	0.648437606
235628	'Prss42'	44	31	6	3	21	14	27	12.66666667	-1.082791525	0.240390403	0.86449675
235631	'Prss50'	43	24	19	9	17	94	28.66666667	40	0.396149957	0.650714266	0.972790461
235633	'Als2cl'	314	284	302	1024	521	453	300	666	1.3511953	0.022038837	0.36727039
235634	'Fam240a'	0	0	0	1	0	0	0	0.333333333	1.020273531	0.802557913	0.972790461
235636	'Rtp3'	3	2	0	2	0	0	1.666666667	0.666666667	-0.82444629	0.773940896	0.972790461
235661	'Dync1li1'	1031	987	1015	667	983	1175	1011	941.6666667	-0.08963582	0.746005836	0.972790461
235674	'Acaa1b'	35.19	14.09	6.43	5.12	5.11	5.87	18.57	5.366666667	-1.741667913	0.061261648	0.562479029
235682	'Zfp445'	5718.9	5953.2	2829	3571	6036.5	3228	4833.69	4278.503333	-0.074739766	0.861781376	0.976863639
235779	'Nlrp4g'	1	1	0	1	19	91	0.666666667	37	5.677227799	7.12E-04	0.053299707
235907	'Zfp65'	455.71	523.56	532.07	242.6	551.52	423.74	503.78	405.9533333	-0.325068807	0.358934128	0.933693976
235956	'Zfp825'	373.08	485.42	223.26	109.46	429.96	304.61	360.5866667	281.3433333	-0.371484042	0.448121966	0.970198456
235973	'Pate14'	0	0	9	0	0	0	3	0	-4.185556921	0.287145672	0.894554788

236082	'Dhrsx'	137	157	126	90	229	169	140	162.6666667	0.213784425	0.566108576	0.972790461
236193	'Zfp709'	293	316	144	56	352	246	251	218	-0.242169441	0.685970072	0.972790461
236266	'Alms1'	2476	2587	744	476	1278	1118	1935.666667	957.3333333	-0.976764224	0.058480802	0.552046072
236285	'Lancl3'	116	141	302	132	99	133	186.3333333	121.3333333	-0.58897941	0.338083383	0.924308496
236293	'Slc22a29'	3	2	0	1	0	0	1.666666667	0.333333333	-2.06277773	0.482179682	0.972790461
236312	'Ifi209'	0	1	5	1	1	0	2	0.666666667	-1.575882381	0.514534792	0.972790461
236366	'5730507C01Rik'	113.82	163.15	110.12	25.34	96.01	78.95	129.03	66.76666667	-0.991928828	0.042521036	0.479768513
236451	'Phf11b'	2.54	5.94	8.51	1	9	2.19	5.663333333	4.063333333	-0.425948226	0.74458337	0.972790461
236511	'Ago1'	2793	2744	1525	673	2616	2377	2354	1888.666667	-0.346382076	0.460158924	0.972790461
236537	'Zfp352'	0	0	0	1	0	0	0	0.333333333	1.020273531	0.802557913	0.972790461
236539	'Phgdh'	1394	1528	2182	1247	2414	3430	1701.333333	2363.666667	0.409008536	0.352129358	0.929748874
236573	'Gbp9'	579.32	570.19	1226.8	8	339	385	792.0966667	244	-1.917258128	0.052528953	0.532345658
236576	'Spry3'	36	55	47	49	55	40	46	48	0.145085472	0.773952374	0.972790461
236643	'Sytl5'	21	22	78	48	16	20	40.33333333	28	-0.400951594	0.665351638	0.972790461
236690	'Nyx'	142	128	21	12	60	33	97	35	-1.438377075	0.066560396	0.576513094
236727	'Slc9a7'	428	484	119	390	849	648	343.6666667	629	0.946888872	0.075046755	0.603900623
236732	'Rbm10'	1515.4	1488.5	1467.1	247.99	1641.1	1455.17	1490.33	1114.746667	-0.521034045	0.373331953	0.93929675
236733	'Usp11'	1699	1628	1055	252	2188	1528	1460.666667	1322.666667	-0.218795814	0.730051516	0.972790461
236749	'Tesl1'	0	0	1	0	0	1	0.333333333	0.333333333	0.058500858	0.988561293	0.999493374
236781	'Gpr119'	0	0	0	1	0	0	0	0.333333333	1.020273531	0.802557913	0.972790461
236790	'Ints6l'	1101	1109	305	141	1406	374	838.3333333	640.3333333	-0.399942071	0.611144437	0.972790461
236792	'Mmgt1'	1015	1083	677	684	812	1140	925	878.6666667	-0.015323851	0.958842295	0.999493374
236794	'Slc9a6'	875	868	262	316	921	1244	668.3333333	827	0.309624237	0.581077413	0.972790461
236798	'Adgrg4'	24	35	3	0	24	16	20.66666667	13.33333333	-0.674999121	0.606540695	0.972790461
236848	'Tmem185a'	826	880	680	401	810	932	795.3333333	714.3333333	-0.159162079	0.591031515	0.972790461
236852	'Magea10'	12	9	3	0	13	17	8	10	0.240308924	0.851016045	0.975734242
236899	'Pcyt1b'	114	108	65	10	90	55	95.66666667	51.66666667	-0.947596441	0.16112056	0.771435531
236900	'Pdk3'	2061	1972	2604	1052	1642	2404	2212.333333	1699.333333	-0.412061849	0.275905903	0.887417193
236904	'Klhl15'	1190	1283	478	420	785	1110	983.6666667	771.6666667	-0.318645301	0.474415703	0.972790461
236915	'Arhgef9'	246	250	68	159	301	212	188	224	0.346841171	0.51295794	0.972790461
236920	'Stard8'	77	106	57	182	150	140	80	157.3333333	1.124149902	0.017234219	0.327921282
236930	'Ercc6l'	619	625	104	72	362	364	449.3333333	266	-0.757510182	0.299874602	0.903723114
237010	'Klhl4'	81	73	8	0	125	67	54	64	0.194346852	0.886358624	0.984371176
237038	'Nox1'	40.1	44.43	201.9	6.23	21.88	6.2	95.47666667	11.43666667	-3.234355838	5.45E-04	0.045845564
237052	'Tceal1'	172	150	138	87	186	148	153.3333333	140.3333333	-0.11665928	0.731430559	0.972790461
237073	'Rbm41'	391	398	280.4	177	384	249	356.4666667	270	-0.366583013	0.288858083	0.896176545
237082	'Nxt2'	287	343	393	390	307	310	341	335.6666667	0.078814951	0.865535641	0.978261888
237091	'Lhfpl1'	10	9	1	1	6	7	6.666666667	4.666666667	-0.506335029	0.678395837	0.972790461
237107	'Gnl3l'	1264	1338	1863	1791	1253	1704	1488.333333	1582.666667	0.170166109	0.72179003	0.972790461
237175	'Adgrg2'	371	358	79	130	675	838	269.3333333	547.6666667	1.001853348	0.157663727	0.768337387

237178	'Ppef1'	15	20	3	2	9	8	12.66666667	6.333333333	-0.972054673	0.328241969	0.921384366
237211	'Fancb'	112	125	28	70	83	77	88.33333333	76.66666667	-0.064482104	0.913060087	0.989773583
237213	'Glra2'	0	0	3	0	0	0	1	0	-2.588571306	0.51580235	0.972790461
237221	'Gemin8'	350	387	240	134	282	307	325.6666667	241	-0.424110618	0.196895676	0.822696823
237222	'Ofd1'	2036.9	1990.9	555.58	131.41	1295.8	1848	1527.796667	1091.733333	-0.54842759	0.489556563	0.972790461
237253	'Lrp11'	190	187	109	47	181	177	162	135	-0.293875773	0.549514727	0.972790461
237256	'Zc3h12d'	5	9	2	6	3	8	5.333333333	5.666666667	0.219283575	0.83780604	0.974723675
237310	'Il22ra2'	0	0	1	0	0	0	0.333333333	0	-0.903279821	0.824807108	0.972790461
237313	'Il20ra'	4	6	0	3	10	15	3.333333333	9.333333333	1.505234399	0.23269197	0.859679981
237320	'Aldh8a1'	0	2	5	2	0	2	2.333333333	1.333333333	-0.771273566	0.71878851	0.972790461
237336	'Tbpl1'	727	722	1808	553	630	712	1085.666667	631.6666667	-0.802712726	0.158512598	0.768890221
237339	'L3mbtl3'	1007	1001	717	490	1442	1027	908.3333333	986.3333333	0.112543223	0.76330931	0.972790461
237353	'Sh3rf3'	97	86	35	10	102	123	72.66666667	78.33333333	0.039177397	0.960066448	0.999493374
237360	'Adamts14'	148	130	94	64	167	116	124	115.6666667	-0.087075516	0.826655817	0.972859382
237362	'Npffr1'	31	18	1	1	28	32	16.66666667	20.33333333	0.247403035	0.846089617	0.975182082
237387	'Lrrc3'	165	178	179	259	207	425	174	297	0.814191813	0.054093981	0.534315058
237397	'C2cd4c'	20	25	16	62	17	24	20.33333333	34.33333333	1.021164704	0.208290136	0.836151197
237400	'Mex3d'	748	684	2974	1174	765	1009	1468.666667	982.6666667	-0.568816629	0.454584692	0.970649024
237403	'Lingo3'	4	1	5	0	2	0	3.333333333	0.666666667	-2.40914927	0.247596428	0.868994456
237411	'Zfp938'	141	178	169	120	171	165	162.6666667	152	-0.066435572	0.845971959	0.975182082
237412	'Gm4924'	200	216	136	55	223	151	184	143	-0.387592043	0.417646903	0.957720764
237422	'Ric8b'	629	706	898	974	761	642	744.3333333	792.3333333	0.202875059	0.684852301	0.972790461
237433	'Gm4925'	12	19	3	2	17	10	11.33333333	9.666666667	-0.229404843	0.822153328	0.972790461
237436	'Gas2l3'	120.98	135.41	208.79	459.97	150.23	134	155.06	248.0666667	0.908852718	0.234391276	0.861206181
237459	'Cdk17'	999	974	1157	1192	1045	794	1043.333333	1010.333333	0.067215728	0.884767241	0.98386821
237465	'Ccdc38'	35	41	8	3	23	12.01	28	12.67	-1.135958609	0.199687536	0.827115347
237500	'Tmtc3'	1149	1154	567	2694	1419	1681	956.6666667	1931.333333	1.230527877	0.023857638	0.382446383
237504	'Rassf9'	30	31	20	21	66	65	27	50.66666667	0.889208983	0.089625043	0.646660839
237523	'Ptprq'	32	28	7	3	47	39	22.33333333	29.66666667	0.362107506	0.702404099	0.972790461
237542	'Osbpl8'	1989	2036	1883	1660	1459	1717	1969.333333	1612	-0.195547888	0.583376358	0.972790461
237553	'Trhde'	118	92	35	15	86	44	81.66666667	48.33333333	-0.757498181	0.265089831	0.88128484
237558	'Myrf1'	0	2	1	1	2	1	1	1.333333333	0.45498643	0.823181338	0.972790461
237560	'Lrrc10'	0	0	1	1	0	1	0.333333333	0.666666667	1.053024025	0.762187968	0.972790461
237611	'Stac3'	16	18	10	8	19	5	14.66666667	10.66666667	-0.388848746	0.628054867	0.972790461
237615	'Ankrd52'	2081	2089	704	1065	2150	1715	1624.666667	1643.333333	0.087078753	0.848505564	0.975686757
237625	'Pla2g3'	20	12	7	14	25	5	13	14.66666667	0.300347069	0.733223375	0.972790461
237636	'Npc1l1'	39	27	16	10	26	7	27.33333333	14.33333333	-0.860104584	0.247461954	0.868994456
237711	'Eml6'	177.68	158.63	109.47	55.08	198.67	255.91	148.5933333	169.8866667	0.144647498	0.774966123	0.972790461
237716	'Gpr75'	44	42	5	8	41	20	30.33333333	23	-0.359907101	0.688375156	0.972790461
237730	'Fbll1'	2	3	0	2	0	0	1.666666667	0.666666667	-0.821185222	0.774842092	0.972790461

237754	'Btnl9'	18	8	3	2	29	40	9.666666667	23.66666667	1.225880596	0.252394913	0.871252558
237758	'Zfp454'	116	116	41	70	124	95	91	96.33333333	0.166386148	0.73131427	0.972790461
237759	'Col23a1'	245	315	62	273	392	214	207.3333333	293	0.655600084	0.288551863	0.895811238
237761	'Sowaha'	22	25	56	39	42	49	34.33333333	43.33333333	0.328691898	0.609632968	0.972790461
237775	'Zfp867'	377.37	430.24	400.5	141.37	415.17	298.15	402.7033333	284.8966667	-0.52748376	0.181681971	0.802050906
237781	'Mief2'	242	231	165	181	325	357	212.6666667	287.6666667	0.45761274	0.116596648	0.704546565
237782	'Smcr8'	1359	1378	1194	2031	1323	1406	1310.333333	1586.666667	0.431551594	0.352672472	0.930050349
237806	'Dnah9'	54	55	8	11	45	75	39	43.66666667	0.153904795	0.854937773	0.975734242
237823	'Pfas'	1342	1309	458	436	1076	697	1036.333333	736.3333333	-0.434195274	0.367340873	0.937190492
237831	'Slc13a5'	24	28	8	38	28	30	20	32	0.854324972	0.20434563	0.833158723
237847	'Rtn4rl1'	64	48	9	427	61	107	40.33333333	198.3333333	2.707794267	0.006026153	0.183570701
237858	'Trarg1'	2	4	1	0	4	9	2.333333333	4.333333333	0.805744404	0.613703442	0.972790461
237859	'Nsrp1'	872	815	861	541	777	767	849.3333333	695	-0.25890402	0.360966726	0.933706718
237860	'Ssh2'	1116	1100	519	321	1101	895	911.6666667	772.3333333	-0.241666115	0.59997739	0.972790461
237868	'Sarm1'	56	72	33	9	34	40	53.66666667	27.66666667	-0.976605832	0.097252551	0.663789939
237877	'Atad5'	770	795	282	206	608	402	615.6666667	405.3333333	-0.566146588	0.251695355	0.871252558
237880	'1700071K01Rik'	0	1	0	0	0	0	0.333333333	0	-0.903279821	0.824807108	0.972790461
237886	'Slfn9'	371.5	379	134.01	1040.7	238	307.79	294.8366667	528.8133333	1.193708939	0.136073396	0.736689633
23789	'Coro1b'	2151	2220	2545	2181	2270	3109	2305.333333	2520	0.165362809	0.634128498	0.972790461
237890	'Slfn14'	26	31	4	0	51	16	20.33333333	22.33333333	0.091363251	0.946013472	0.996592403
237891	'Gas2l2'	0	1	0	1	0	0	0.333333333	0.333333333	0.058500858	0.988561293	0.999493374
237898	'Usp32'	5042	4856	2036	3762	2849	3447	3978	3352.666667	-0.080333521	0.864789125	0.978012661
23790	'Coro1c'	2027	1934	1289	2026	1646	2575	1750	2082.333333	0.353538829	0.337077554	0.92380462
237911	'Brip1'	403.93	449.26	108	47	251.21	244	320.3966667	180.7366667	-0.83967511	0.211189207	0.840335617
23792	'Adam23'	177	173	53	104	90	137	134.3333333	110.3333333	-0.151335296	0.778336173	0.972790461
237926	'Rsad1'	247	262	164	107	196	176	224.3333333	159.6666667	-0.451204596	0.146564125	0.753611197
237928	'Phospho1'	18.48	18.26	203.39	805.51	13.92	128.64	80.04333333	316.0233333	2.21496982	0.113721344	0.698948431
23793	'Adam25'	0	0	0	0	4	0	0	1.333333333	2.75434637	0.489126355	0.972790461
237930	'Ttll6'	8	4	6	10	0	0	6	3.333333333	-0.409060793	0.828247708	0.973238667
237934	'Krt39'	0	1	0	0	0	0	0.333333333	0	-0.903279821	0.824807108	0.972790461
23794	'Adamts5'	99	76	176	32	71	86	117	63	-0.979749389	0.08769687	0.642214018
237940	'Aoc2'	161	156	108	345	173	81	141.6666667	199.6666667	0.765045844	0.283812735	0.892170588
237943	'Gpatch8'	2468	2449	1312	1859	2309	1577	2076.333333	1915	0.006983843	0.98603309	0.999493374
23795	'Agr2'	0	0	0	0	2	0	0	0.666666667	1.801491674	0.656112935	0.972790461
237954	'Lrrc37a'	0	2.06	0	0	7.04	8.18	0.686666667	5.073333333	2.843943737	0.231045903	0.858106017
23796	'Aplnr'	96	143	10	15	151	16	83	60.66666667	-0.416759202	0.699321165	0.972790461
23797	'Akt3'	2894.7	2713.7	2009	1400.5	1945.4	2652.59	2539.156667	1999.49	-0.311318904	0.245839868	0.867305172
237979	'Sdk2'	548	542	69	154	636	525	386.3333333	438.3333333	0.210379224	0.777366573	0.972790461
237987	'Otop2'	0	1	0	2	0	1	0.333333333	1	1.739900272	0.590612721	0.972790461
237988	'Cdr2l'	574	569	1213	1796	627	827	785.3333333	1083.333333	0.627163237	0.385540507	0.947137372

23801	'Aloxe3'	22	24	29	33	20	11	25	21.33333333	-0.05667245	0.940521564	0.996003484
23802	'Amfr'	1899	1919	1359	6800	2942	5190	1725.666667	4977.333333	1.713142282	0.001260081	0.075335398
238021	'Fscn2'	3	9	5	9	1	4	5.666666667	4.666666667	-0.034056372	0.978265873	0.999493374
238023	'Hexdc'	1062.7	1151.8	488.58	600.51	1221.7	1384.79	901.0233333	1068.993333	0.278895226	0.479945941	0.972790461
238024	'Fn3krp'	259	275	140	144	322	317	224.6666667	261	0.238768731	0.522520624	0.972790461
238037	'Wdcp'	249.33	248.28	307.7	277.72	174	199	268.4366667	216.9066667	-0.181368588	0.72219164	0.972790461
23805	'Apc2'	163	185	26	84	248	158	124.6666667	163.3333333	0.458043671	0.511114091	0.972790461
238055	'Apob'	15	25	2	1	4	6	14	3.666666667	-1.899363357	0.100856143	0.671759705
238057	'Gdf7'	1	0	1	1	1	0	0.666666667	0.666666667	0.117746761	0.96570115	0.999493374
23806	'Arih1'	3725	3766	3829	4057	3474	3877	3773.333333	3802.666667	0.106972756	0.778473202	0.972790461
23807	'Arih2'	2219.7	2191.5	2652.3	2033.7	1592.1	2372.06	2354.49	1999.26	-0.167359795	0.683819499	0.972790461
238076	'Kcns3'	29	38	40	16	39	28	35.66666667	27.66666667	-0.380037925	0.474880844	0.972790461
23808	'Ash2l'	2096	2163.1	1501	878	1946	2244	1920.036667	1689.333333	-0.191400912	0.552457518	0.972790461
238123	'Cog5'	1150.9	1152.3	600.9	731.88	1369.4	1519.08	968.0066667	1206.783333	0.352149585	0.295141247	0.90029104
238130	'Dock4'	1656	1660	445	905	1822	1537	1253.666667	1421.333333	0.257661867	0.614947668	0.972790461
238161	'Akap6'	14	14	5	7	12	12	11	10.33333333	-0.026507185	0.972143151	0.999493374
238205	'Lrfn5'	78	105	12	4	143	58	65	68.33333333	0.039099922	0.970699529	0.999493374
23821	'Bace1'	894.87	786.86	281.82	1319.8	1162.5	1053.71	654.5166667	1178.656667	1.027335572	0.047180953	0.50504201
238217	'Rpl10l'	247	246	47	0	218	580	180	266	0.468816248	0.738887132	0.972790461
238247	'Arid4a'	951	960	763	690	1105	873	891.3333333	889.3333333	0.047482225	0.863402775	0.977375026
23825	'Banf1'	1022	1052	1257	592	1035	1531	1110.333333	1052.666667	-0.11686205	0.758762049	0.972790461
238257	'Tmem30b'	58	46	6	62	21	53	36.66666667	45.33333333	0.552554587	0.531957102	0.972790461
238266	'Syt16'	18	21	3	1	53	161	14	71.66666667	2.267211661	0.068497581	0.583886121
23827	'Bpnt1'	894	818.96	477	714	688	954	729.9866667	785.3333333	0.204181478	0.568559298	0.972790461
238271	'Kcnh5'	0	0	0	0	3	0	0	1	2.343657704	0.558557988	0.972790461
238276	'Akap5'	344	421	73	152	341	352	279.3333333	281.6666667	0.074164584	0.903896953	0.987837693
23828	'Bves'	43	73	82	33	195	41	66	89.66666667	0.371728248	0.62475044	0.972790461
23829	'C1ql1'	36	27	6	14	15	26	23	18.33333333	-0.227864767	0.765532749	0.972790461
23830	'Capn10'	529	494	303	230	475	622	442	442.3333333	0.004196037	0.990814945	0.999493374
23831	'Car14'	107.8	76.17	39.36	35.68	23.13	52.79	74.44333333	37.2	-0.902780102	0.116294299	0.703687844
238317	'Mideas'	866.35	829.78	5697.9	1675.1	793.85	1288.2	2464.66	1252.366667	-0.966147768	0.286507476	0.894199485
23832	'Xcr1'	4	5	1	0	1	2	3.333333333	1	-1.749626571	0.314971944	0.914723408
238323	'Rps6kl1'	11	8	6	29	7	4	8.333333333	13.33333333	1.014575178	0.353322009	0.930050349
238328	'Vash1'	1147.4	1150.3	317	325.39	1534.3	909.56	871.5766667	923.0933333	0.093570369	0.879237278	0.981796025
23833	'Cd52'	9	17	10	24	10	16	12	16.66666667	0.649445089	0.414908207	0.95722888
238330	'Irf2bpl'	850	819	2161	2872	1063	1684	1276.666667	1873	0.67235673	0.356578837	0.932066196
238331	'Zdhhc22'	0	1	0	1	2	0	0.333333333	1	1.583008287	0.630177186	0.972790461
238333	'Samd15'	49	57	3	0	44	35	36.33333333	26.33333333	-0.509703473	0.709823244	0.972790461
23834	'Cdc6'	184	194	105	55	148	130	161	111	-0.526695507	0.199201367	0.826361855
23836	'Cdh20'	14	35	8	1	26	22	19	16.33333333	-0.278506136	0.78910664	0.972790461

23837	'Cfdp1'	1486	1585	2392	1145	1681	1973	1821	1599.666667	-0.207278655	0.611720247	0.972790461
238377	'Gpr68'	10	13	76	3	13	16	33	10.66666667	-1.844443562	0.082220462	0.62228321
238384	'Slc24a4'	15	11	12	0	6	4	12.66666667	3.333333333	-2.005008537	0.060197826	0.556925094
238386	'Btbd7'	3990	4012	3088	2928	3079	3056	3696.666667	3021	-0.195770407	0.533735064	0.972790461
238393	'Serpina3f'	98.92	89	530.45	65.59	25.94	31.56	239.4566667	41.03	-2.492160804	0.007909428	0.214208031
238395	'Serpina3j'	0	0	0	0	0	1	0	0.333333333	1.020273531	0.802557913	0.972790461
238405	'Adam6b'	0	0	0	0	1	0	0	0.333333333	1.020273531	0.802557913	0.972790461
23844	'Clca1'	1	2	0	4	7	0	1	3.666666667	2.076395996	0.313746508	0.913268497
23845	'Clec5a'	1	2	0	0	5	8	1	4.333333333	2.052681009	0.305480131	0.907188578
238455	'Macc1'	2	0	2	136	1	2	1.333333333	46.33333333	5.544935435	0.002570101	0.113899205
238463	'Tubal3'	1	0	2	1	0	0	1	0.333333333	-1.526634549	0.634193714	0.972790461
23849	'Klf6'	898	787	7303	2828	1232	1483	2996	1847.666667	-0.655851131	0.506459512	0.972790461
23850	'Pappa2'	709	553	817	461	133	105	693	233	-1.303250268	0.09649671	0.663111432
238505	'Mtr'	2524	2808	345	1882	5569	3252	1892.333333	3567.666667	0.991625278	0.161144444	0.771435531
23853	'Def6'	44	54	53	6	66	44	50.33333333	38.66666667	-0.489363682	0.510257773	0.972790461
23854	'Def8'	703	714	350	518	941	826	589	761.6666667	0.428287322	0.21680461	0.843391934
238555	'Btn2a2'	16	16	65	117	8	9	32.33333333	44.66666667	0.764041518	0.543643677	0.972790461
23856	'Dido1'	3328	3337	1020	1486	3049	2351	2561.666667	2295.333333	-0.083183344	0.863196665	0.977316281
238564	'Mylk4'	22	19	5	10	7	2	15.33333333	6.333333333	-1.023795987	0.315813405	0.914723408
23857	'Dmtf1'	1423	1451.6	1744	1210	1568	1296	1539.54	1358	-0.1355326	0.708819459	0.972790461
23859	'Dlg2'	82	68	155	137	76	179	101.6666667	130.6666667	0.389010807	0.537091377	0.972790461
23863	'Dand5'	50	56	47	47	53	62	51	54	0.138952418	0.729166483	0.972790461
238662	'Spata31d1b'	1.13	0	0	0	0	0	0.376666667	0	-0.903279821	0.824807108	0.972790461
238663	'Spata31d1d'	0	5	3	0	1	1	2.666666667	0.666666667	-2.06805611	0.356706987	0.932066196
238673	'Zfp367'	426	444	522	234	391	378	464	334.3333333	-0.472236363	0.158784646	0.768918948
238680	'Cntnap3'	1	4	1	1	8	2	2	3.666666667	0.87233266	0.564830991	0.972790461
238683	'Spata31d1c'	6	6	0	0	0	0	4	0	-4.278004955	0.160476402	0.771114735
238690	'Zfp458'	107.08	106.98	79.14	71	115	78	97.73333333	88	-0.083552754	0.824920813	0.972790461
238692	'Zfp874a'	170.48	183.96	246.84	196	383.67	424	200.4266667	334.5566667	0.704662542	0.080867267	0.618940615
238693	'Zfp58'	156	165	126	146	235	265	149	215.3333333	0.556850445	0.050721602	0.520971962
23871	'Ets1'	2510	2432	1463	1232	3273	2525	2135	2343.333333	0.145741503	0.688417142	0.972790461
23872	'Ets2'	2107	2015	4025	4175	2485	2509	2715.666667	3056.333333	0.280551513	0.645259777	0.972790461
238722	'Zfp72'	178	173	126	66	245	296	159	202.3333333	0.292879038	0.552725407	0.972790461
238725	'Gpr150'	2	0	0	0	1	1	0.666666667	0.666666667	0.006630366	0.998451383	0.999900097
238726	'Fam81b'	0	0	0	1	0	0	0	0.333333333	1.020273531	0.802557913	0.972790461
23873	'Faim'	429	443	472	162	353	422	448	312.3333333	-0.557222152	0.125803635	0.72022881
23874	'Farsb'	1529	1585	1427	884.88	1155	1707.98	1513.666667	1249.286667	-0.260519051	0.371084926	0.938513534
23876	'Fbln5'	199	161	31	52	368	273	130.3333333	231	0.815333976	0.29285012	0.900043079
23877	'Fiz1'	592	620	1268	378	610	700	826.6666667	562.6666667	-0.609922981	0.230821793	0.857634106
23879	'Fxr2'	3019	2901	2652	2879	2686	2821	2857.333333	2795.333333	0.066139749	0.849785678	0.975734242

238799	'Tnpo1'	2833	2928	2789	3027	2531	3035	2850	2864.333333	0.104166262	0.779752934	0.972790461
23880	'Fyb'	55	67	49	30	70	114	57	71.33333333	0.286004336	0.561935264	0.972790461
238803	'Zfp366'	19	32	1	0	25	21	17.33333333	15.33333333	-0.216818367	0.881108549	0.982528614
23881	'G3bp2'	5067	5233	3011	6097	5368	9361	4436.993333	6942	0.732658633	0.048244223	0.510416894
23882	'Gadd45g'	204	237	305	213	283	296	248.6666667	264	0.099782663	0.799085856	0.972790461
238831	'Ppwd1'	733.86	806.79	502.91	232	642.91	580.95	681.1866667	485.2866667	-0.496336832	0.17804851	0.797422443
23885	'Gmcl1'	1162	1225	513	676	1028	1087	966.6666667	930.3333333	0.018635062	0.960319697	0.999493374
23886	'Gdf15'	12	10	55	148	6	75	25.66666667	76.33333333	1.723124779	0.146881992	0.753611197
23887	'Ggt5'	439	514	108	247	861	829	353.6666667	645.6666667	0.886947443	0.150444239	0.759693458
238871	'Pde4d'	257	277	342	279	160	184	292	207.6666667	-0.355009069	0.510942466	0.972790461
238875	'Gapt'	0	0	0	0	0	1	0	0.333333333	1.020273531	0.802557913	0.972790461
23888	'Gpc6'	311	214	452	218	204	253	325.6666667	225	-0.504778754	0.316639999	0.915640551
238896	'Cdc20b'	5	3	0	0	5	6	2.666666667	3.666666667	0.419990894	0.821757774	0.972790461
23890	'Gpr34'	1	1	0	1	2	3	0.666666667	2	1.628082727	0.411739824	0.95722888
23892	'Grem1'	3	2	24	197	1	18	9.666666667	72	3.173761407	0.044075348	0.489462917
23893	'Grem2'	405	363	125	1125	250	238	297.6666667	537.6666667	1.233964805	0.148902288	0.757738857
238939	'Gm281'	1	0	1	0	0	0	0.666666667	0	-1.858475606	0.644939423	0.972790461
23894	'Gtf2h2'	790	792	420	181	636	640	667.3333333	485.6666667	-0.481369169	0.298104773	0.901330779
23897	'Hax1'	732	854	2108	1363	757	1291	1231.333333	1137	-0.071340056	0.913916947	0.989964366
238988	'Erc2'	85	76	151	25	88	146	104	86.33333333	-0.409221048	0.533450971	0.972790461
23900	'Hcst'	1	1	0	0	0	4	0.666666667	1.333333333	0.925404586	0.768461929	0.972790461
239017	'Ogdhl'	995	987	448	179	884	1425	810	829.3333333	-0.028827949	0.963560957	0.999493374
239027	'Arhgap22'	171	150	151	204	160	152	157.3333333	172	0.254898843	0.574348322	0.972790461
23908	'Hs2st1'	1042	1094	1025	1644	1368	1353	1053.666667	1454.996667	0.581193293	0.15023015	0.759693458
239083	'Ccnb1ip1'	531	515	143	1	200	306	396.3333333	169	-1.324413115	0.256694416	0.877127174
239096	'Cdh24'	249	263	169	219	297	161	227	225.6666667	0.10205345	0.813297424	0.972790461
239099	'Homez'	308.58	384.08	123.5	154.31	287.99	337.9	272.0533333	260.0666667	-0.016566925	0.971302316	0.999493374
239102	'Zfhx2'	369	399	101	372	347	238	289.6666667	319	0.339027952	0.57143423	0.972790461
239114	'Il17d'	46	41	72	11	42	52	53	35	-0.708831223	0.268693102	0.883652181
23912	'Rhof'	1025	980	227	265	1499	545	744	769.6666667	0.071547622	0.919463982	0.990815958
239122	'Setdb2'	423.7	468.94	161.9	160.17	281.53	190.09	351.5133333	210.5966667	-0.641982627	0.168686826	0.784384366
239126	'C1qtnf9'	10	9	3	3	7	4	7.333333333	4.666666667	-0.582926222	0.556688328	0.972790461
239133	'Dleu7'	6	1	6	2	2	0	4.333333333	1.333333333	-1.593087869	0.335498456	0.922413343
239134	'Gucy1b2'	1	2	0	0	0	1	1	0.333333333	-1.323989397	0.695217833	0.972790461
239157	'Pnma2'	83	80	48	0	70.16	83	70.33333333	51.05333333	-0.590204077	0.611260149	0.972790461
239167	'Synb'	1	6	0	8	7	16	2.333333333	10.33333333	2.256559329	0.085427104	0.634617076
23917	'Impdh1'	579	579	2017	509	534	771	1058.333333	604.6666667	-0.865755272	0.199026262	0.825995916
239170	'Fam160b2'	664	699	451	336	854	688	604.6666667	626	0.057239354	0.867548472	0.978978606
23918	'Impdh2'	2307	2213	3501	2502	2436	3209	2673.666667	2715.666667	0.049465857	0.909500154	0.989364653
239188	'Enox1'	212	191	364	22	270	251	255.6666667	181	-0.674725086	0.388314427	0.950023663

23919	'Insl5'	4	5	1	3	5	0	3.333333333	2.666666667	-0.141396175	0.929593876	0.993164002
23920	'Insrr'	3	6	37	0	1	0	15.33333333	0.333333333	-5.516917211	0.002816353	0.118987799
23921	'Sh2b2'	170	166	508	198	110	108	281.3333333	138.6666667	-0.934463373	0.207134749	0.83479753
239217	'Kctd12'	1974	1727	2264	194	1657	1230	1988.333333	1027	-1.078515413	0.09582638	0.662411648
23922	'Jtb'	262	250	321	330	402	486	277.6666667	406	0.56872721	0.113273053	0.698948431
23923	'Aadat'	0	0	0	1	2	0	0	1	2.548734582	0.523314857	0.972790461
23924	'Katna1'	608	644	1077	659	627	885	776.3333333	723.6666667	-0.086083736	0.855437311	0.975734242
23925	'Kel'	11	9	1	1	30	1	7	10.66666667	0.605361618	0.689073894	0.972790461
239250	'Slitrk6'	60	56	15	1	129	53	43.66666667	61	0.417341688	0.71480836	0.972790461
239273	'Abcc4'	92	91	71	243	67	148	84.66666667	152.6666667	1.072923857	0.109708946	0.689773582
23928	'Lamc3'	58	49	34	11	42	35	47	29.33333333	-0.697860491	0.201947847	0.829968109
239283	'Oxgr1'	17	8	1	0	2	5	8.666666667	2.333333333	-1.91842223	0.202650715	0.830701895
239318	'Plcxd3'	35	23	3	0	1	0	20.33333333	0.333333333	-5.681490906	6.81E-04	0.052033928
239319	'Card6'	258	250	115	89	198	132	207.6666667	139.6666667	-0.516693557	0.230654554	0.857634106
239336	'Rxfp3'	0	1	0	0	0	0	0.333333333	0	-0.903279821	0.824807108	0.972790461
239337	'Adamts12'	398	374	90	335	365	365	287.3333333	355	0.455141996	0.419762565	0.958308933
23934	'Ly6h'	60	57	21	28	120	79	46	75.66666667	0.721459825	0.236800768	0.862272407
23936	'Lynx1'	169	178	45	403	154	248	130.6666667	268.3333333	1.303148246	0.062639423	0.566066012
239364	'Tspyl5'	427	469	168	156	317	341	354.6666667	271.3333333	-0.341895595	0.439725262	0.967599153
239368	'Erich5'	19	11	6	0	17	30	12	15.66666667	0.278252806	0.821733853	0.972790461
23937	'Mab21l2'	1	0	1	1	0	0	0.666666667	0.333333333	-0.892941112	0.796499214	0.972790461
23938	'Map2k5'	627	592	489	172	632	647	569.3333333	483.6666667	-0.289537702	0.518927972	0.972790461
23939	'Mapk7'	1004	1050	1035	457	949	872	1029.666667	759.3333333	-0.448155999	0.140756319	0.744590744
239393	'Lrp12'	369	364	198	832	465	600	310.3333333	632.3333333	1.219369995	0.017472812	0.330869625
239405	'Rspo2'	43	44	7	0	52	65	31.33333333	39	0.242272697	0.85063089	0.975734242
239408	'Tmem74'	44	50	32	64	99	108	42	90.33333333	1.145203352	0.001994557	0.098751347
23942	'Mta2'	2698	2929	2376	2103	3138	2930	2667.666667	2723.666667	0.074167632	0.759363208	0.972790461
239420	'Csmd3'	20	14	6	1	23	8	13.33333333	10.66666667	-0.363291073	0.732427973	0.972790461
23943	'Esyt1'	618	558	414	871	980	1106	530	985.6666667	0.97461084	9.00E-04	0.062657876
239435	'Aard'	214	239	133	151	696	381	195.3333333	409.3333333	1.046536912	0.043597449	0.487000407
239447	'Colec10'	9	4	0	2	8	4	4.333333333	4.666666667	0.174214121	0.903707019	0.987713587
23945	'Mgll'	109	90	86	863	76	336	95	425	2.467068497	0.005481662	0.174861338
239463	'Fam83a'	5	6	1.01	4.78	0	0	4.003333333	1.593333333	-1.108969513	0.586441496	0.972790461
23947	'Mid2'	411	386	581	202	609	581	459.3333333	464	-0.06486589	0.890194852	0.984980969
23948	'Mmp17'	67	72	22	90	57	54	53.66666667	67	0.539513377	0.403313793	0.955996639
23950	'Dnajb6'	2409	2526	4120	3813	2044	3886	3018.333333	3247.666667	0.181017551	0.743114161	0.972790461
239510	'Phf20l1'	1689	1735	1122	1364	2160	1283	1515.333333	1602.333333	0.165748885	0.650872008	0.972790461
239528	'Ago2'	2205	1860	1892	3564	1425	1755	1985.666667	2248	0.393791757	0.51186765	0.972790461
239530	'Gpr20'	24	16	16	82	13	32	18.66666667	42.33333333	1.465672649	0.101368418	0.671759705
23954	'Nek3'	434	425	257	55	243	170	372	156	-1.280830228	0.009358621	0.23755153

239546	'Zfp647'	211	253	257	93	204	148	240.3333333	148.3333333	-0.700995852	0.064171868	0.568781147
23955	'Nek4'	1420	1531	398	176	935	950	1116.333333	687	-0.720939615	0.269673786	0.883851074
239552	'Apol8'	3	1	6	7	10	2	3.333333333	6.333333333	0.966654651	0.446237209	0.970172328
239554	'Foxred2'	248.07	239	174	80	172	246	220.3566667	166	-0.425392016	0.273444453	0.884738928
239555	'Mief1'	1470	1347	1511	607	969	1071	1442.666667	882.3333333	-0.705470098	0.016000687	0.315238408
239556	'Cacna1i'	43	28	32	1	337	290	34.33333333	209.3333333	2.455136633	0.039282979	0.46244458
239559	'A4galt'	89	113	160	232	152	160	120.6666667	181.3333333	0.695402549	0.220463003	0.849076011
23956	'Neu2'	5	4	1	0	7	5	3.333333333	4	0.210600146	0.889017485	0.984792893
23957	'Nr0b2'	35	48	22	22	31	47	35	33.33333333	-0.028613342	0.955186248	0.999493374
239570	'Ttc38'	699	733	139	163	966	718	523.6666667	615.6666667	0.231067237	0.745798017	0.972790461
23958	'Nr2e3'	19	29	0	0	14	15	16	9.666666667	-0.76201248	0.649390548	0.972790461
23959	'Nt5e'	20	14	40	145	6	58	24.66666667	69.66666667	1.731749014	0.117569151	0.705814751
239591	'Ttll8'	19	26	7	7	18	12	17.33333333	12.33333333	-0.433590754	0.56166169	0.972790461
23960	'Oas1g'	2.42	2.37	11.49	1.23	5.79	19.41	5.426666667	8.81	0.492243333	0.71787274	0.972790461
239606	'Slc2a13'	70	54	35	177	93	124	53	131.3333333	1.499748375	0.008449787	0.222807422
239618	'Pdzrn4'	15	27	11	3	58	65	17.66666667	42	1.153621577	0.220089401	0.848689247
23962	'Oasl2'	294	278	778	221	170	979	450	456.6666667	-0.120229188	0.878109017	0.981341203
23963	'Tenm1'	18	22	9	2	20	7	16.33333333	9.666666667	-0.776229605	0.397037854	0.954358954
23964	'Tenm2'	28	25	2	6	18	14	18.33333333	12.66666667	-0.46585528	0.630280407	0.972790461
239647	'Pced1b'	738	850.21	552	427	1085	689	713.4033333	733.6666667	0.058654857	0.872002401	0.980171118
23965	'Tenm3'	1096.8	1020.9	438.1	439.73	1104.2	926.62	851.9233333	823.5033333	-0.016556572	0.969249516	0.999493374
239650	'Ccdc184'	3	9	27	11	2	1	13	4.666666667	-1.26741658	0.356940408	0.932419764
239652	'Zfp641'	339	525	119	56	521	171	327.6666667	249.3333333	-0.404487552	0.60219457	0.972790461
239659	'C1ql4'	2	3	3	24	0	0	2.666666667	8	2.041037242	0.318750692	0.916136994
23966	'Tenm4'	3499	3310	655	559	3721	2386	2488	2222	-0.16684104	0.818171385	0.972790461
239667	'Dip2b'	1286	1238	807	787	1006	862	1110.333333	885	-0.236614549	0.450736128	0.970590732
23967	'Osr1'	103	106	141	2	97	60	116.6666667	53	-1.300858071	0.168585318	0.784384366
239673	'Krt90'	1	2	4	0	0	1	2.333333333	0.333333333	-2.718793528	0.233902032	0.860643592
23968	'Nlrp5'	68	56	12	1	305	1510	45.33333333	605.3333333	3.634113336	0.008473057	0.222807422
23969	'Pacsin1'	18	25	58	14	5	11	33.66666667	10	-1.686755086	0.053631445	0.533767484
239691	'AU021092'	11	11	28	1	9	16	16.66666667	8.666666667	-1.124661299	0.269341112	0.883851074
23970	'Pacsin2'	3197	3323	3005	4219	3353	4568	3175	4046.666667	0.446159278	0.229867719	0.856721174
239706	'Mettl22'	136	146	143	34	124	149	141.6666667	102.3333333	-0.543182621	0.279880561	0.889355605
23971	'Papss1'	1497	1503	4130	1453	1601	2203	2376.666667	1752.333333	-0.482131507	0.420552167	0.958511083
239719	'Mrtfb'	1744	1778.1	1237	824.88	1670	1442	1586.353333	1312.293333	-0.250046153	0.367765718	0.937190492
23972	'Papss2'	209	202	1306	48	302	225	572.3333333	191.6666667	-1.811332741	0.049272933	0.515210762
239731	'Rimbp3'	20	15	4	12	16	60	13	29.33333333	1.176409445	0.173806522	0.79261444
239739	'Lamp3'	2	3	0	0	4	3	1.666666667	2.333333333	0.460068293	0.82127675	0.972790461
239743	'Klhl6'	24	19	7	9	29	21	16.66666667	19.66666667	0.266761076	0.707754556	0.972790461
239759	'Liph'	21	18	39	60	11	7	26	26	0.289555185	0.785393548	0.972790461

239766	'Rtp1'	1	5	1	1	3	1	2.333333333	1.666666667	-0.417275397	0.79792031	0.972790461
239789	'Gmnc'	4	1	0	0	2	1	1.666666667	1	-0.739244753	0.754811607	0.972790461
239790	'Ostn'	0	0	5	0	0	2	1.666666667	0.666666667	-1.638005055	0.658136019	0.972790461
239796	'Mb21d2'	330	349	458	157	326	415	379	299.3333333	-0.39449432	0.344053346	0.925308103
23980	'Pebp1'	4313	4463	4368	4189	4955	7551	4381.333333	5565	0.366144553	0.235614388	0.861636582
239827	'Pigz'	6	6	7	5	8	2	6.333333333	5	-0.270582591	0.792140428	0.972790461
23983	'Pcbp1'	3782	3875	9578	4038	4453	6034	5745	4841.666667	-0.280258675	0.621731388	0.972790461
239833	'Lmln'	343	269	161	168	284	231	257.6666667	227.6666667	-0.111887088	0.760654337	0.972790461
239839	'Ccdc14'	338	356	100	73	284	241	264.6666667	199.3333333	-0.400395229	0.496484682	0.972790461
23984	'Pde10a'	863.95	867.45	509.18	1384.5	1190.5	765.96	746.86	1113.626667	0.755077054	0.125255559	0.720137383
239845	'Gpr156'	25	18	7	4	30	19	16.66666667	17.66666667	0.064502167	0.93918531	0.995472549
239849	'Cd200r4'	0	0	1	10	3	14	0.333333333	9	4.747282202	0.006528465	0.190952592
23985	'Slc26a4'	2	3	0	0	4	2	1.666666667	2	0.247581103	0.905944602	0.988794924
239852	'Zpld1'	2	2	0	1	12	23	1.333333333	12	3.106791255	0.036340431	0.456985794
239853	'Adgrg7'	34	20	0	42	43	11	18	32	1.101617793	0.37988273	0.943232396
239857	'Cadm2'	17	2	10	21	18	14	9.666666667	17.66666667	0.98326744	0.291882482	0.89958198
23986	'Eci2'	1394	1485	1488.9	961	1942	1822	1455.953333	1575	0.104571688	0.729375359	0.972790461
23988	'Pin1'	681	769	963	416	677	882	804.3333333	658.3333333	-0.317043373	0.40140498	0.955860942
23989	'Med24'	1029	1060	483	616	1104	1045	857.3333333	921.6666667	0.161897225	0.652448845	0.972790461
23991	'Cib1'	420	444	307	444	340	644	390.3333333	476	0.37122641	0.345712927	0.926775829
23992	'Prkra'	576	657	793	353	568	606	675.3333333	509	-0.415837414	0.239405198	0.86449675
23993	'Klk7'	1	2	2	0	3	0	1.666666667	1	-0.828725313	0.717340079	0.972790461
23994	'Dazap2'	2856	2724	4279	1803	2300	2987	3286.333333	2363.333333	-0.485064317	0.235177107	0.861636582
23996	'Psmc4'	1328	1344	1079	1313	1356	1621	1250.333333	1430	0.271800885	0.360562756	0.933706718
23997	'Psmd13'	1195	1249	829	1209	1192	1448	1091	1283	0.329381684	0.303191137	0.906189263
239985	'Arid1b'	2726	2641	1423	996	2828	2671	2263.333333	2165	-0.06510252	0.870999171	0.980126495
23999	'Twf2'	375	381	335	269	474	508	363.6666667	417	0.207275813	0.442277668	0.969177389
24000	'Ptpn21'	829	736	980	715	636	671	848.3333333	674	-0.253806011	0.54597232	0.972790461
24001	'Tiam2'	204.17	189.44	139.63	86	207.07	183.75	177.7466667	158.94	-0.155654545	0.65686099	0.972790461
240023	'Pnldc1'	113	104	29	3	96	202	82	100.3333333	0.19894056	0.848771657	0.975686757
240025	'Dact2'	164	136	421	396	258	290	240.3333333	314.6666667	0.444660278	0.513768364	0.972790461
240028	'Lnpep'	465	476	241	2787	693	1444	394	1641.333333	2.334077218	7.45E-04	0.054446012
240034	'Zfp760'	577	568	259	346	629	336	468	437	-0.004786541	0.991464356	0.999493374
240038	'Zfp994'	195.91	214.21	192.23	73.05	243	217.99	200.7833333	178.0133333	-0.219612983	0.609241501	0.972790461
24004	'Rai2'	103	121	90	26	88	92	104.6666667	68.66666667	-0.648042462	0.166103217	0.780893009
240041	'Zfp945'	873	872	435	346	933	612	726.6666667	630.3333333	-0.175363008	0.671143605	0.972790461
240047	'Mmp25'	4	9	4	2	19	14	5.666666667	11.66666667	0.981788944	0.33316093	0.921648675
240055	'Neurl1b'	89	103	51	1076	106	203	81	461.6666667	2.910960837	0.002169403	0.10389089
240057	'Syngap1'	3330	3153	1179	1195	2519	1147	2554	1620.333333	-0.561511154	0.271090851	0.884312405
240058	'Cpne5'	204	198	365	16	160	225	255.6666667	133.6666667	-1.11925777	0.155009354	0.766453805

240063	'Zfp811'	109	135	29	52	126	53	91	77	-0.143586519	0.826119233	0.972844676
240064	'Zfp799'	800	825	598	209	893	658	741	586.6666667	-0.380039766	0.41479349	0.95722888
240066	'Zfp870'	202	250	264	204	299	288	238.6666667	263.6666667	0.164277617	0.635575214	0.972790461
240067	'Zfp952'	205	236	198	249	343	311	213	301	0.551327679	0.066614311	0.576717337
240068	'Zfp563'	315.07	326.31	163.65	123.04	383.69	308.17	268.3433333	271.6333333	0.021322698	0.961087737	0.999493374
240069	'Morc2b'	0	1	0	0	0	1	0.333333333	0.333333333	0.058500858	0.988561293	0.999493374
240084	'Cchcr1'	294	296	240	251	345	300	276.6666667	298.6666667	0.172380059	0.556627466	0.972790461
240087	'Mdc1'	4032	3933	1214	766	2408	2338	3059.666667	1837.34	-0.720197292	0.177226276	0.79704932
240095	'H2-M5'	7	11	4	1	8	3	7.333333333	4	-0.875260905	0.430675761	0.963068403
24010	'Ik'	3323	3397	4125	2535	2814	3757	3615	3035.333333	-0.226347485	0.519115296	0.972790461
240119	'St6gal2'	26	45	1	0	13	1	24	4.666666667	-2.355974906	0.137460168	0.738626463
24012	'Rgs7'	46	47	35	8	35	20	42.66666667	21	-1.04128037	0.076628624	0.609792246
240120	'Zfp119b'	106.87	153.13	91.02	33	112	87	117.0066667	77.33333333	-0.609766974	0.191477599	0.813837363
240121	'Fsd1'	67	69	26	72	46	69	54	62.33333333	0.371794521	0.515337296	0.972790461
24013	'Grk1'	47.53	44.99	5.15	0	22.14	15.09	32.55666667	12.41	-1.417829057	0.25240101	0.871252558
240131	'Lrrc30'	5	11	0	1	6	5	5.333333333	4	-0.379465105	0.800397694	0.972790461
24014	'Rnasel'	934	968	227	58	968	507	709.6666667	511	-0.515915382	0.540769906	0.972790461
24015	'Abce1'	3026	3261	3927	2187	2873	3242	3404.666667	2767.333333	-0.283058948	0.401888812	0.955996639
240168	'Rasgrp3'	336	369	56	36	625	266	253.6666667	309	0.258914827	0.776108574	0.972790461
24017	'Rnf13'	773	792	1858	830	1067	1234	1141	1043.666667	-0.164889225	0.763090545	0.972790461
240174	'Thada'	1112	1070	252	411	807	825	811.3333333	681	-0.181484849	0.741585842	0.972790461
24018	'Rngtt'	770.85	827.52	538.3	624.05	675.57	730.03	712.2233333	676.55	0.018695636	0.95198016	0.998640168
240185	'Jcad'	2676	2681	786	904	4184	3368	2047.666667	2818.666667	0.456776477	0.445090189	0.969888068
240186	'Zfp438'	397	391	264	155	248	242	350.6666667	215	-0.658978756	0.018082789	0.335677246
240215	'Slc4a9'	0	1	0	0	0	0	0.333333333	0	-0.903279821	0.824807108	0.972790461
240216	'E230025N22Rik'	1	1	2	1	0	1	1.333333333	0.666666667	-0.9279376	0.66533809	0.972790461
240239	'Gpr151'	4	2	2	4	4	0	2.666666667	2.666666667	0.195270043	0.902571285	0.987331639
240255	'Ythdc2'	1462	1506	328	167	967	950	1098.666667	694.6666667	-0.67862171	0.330544775	0.921648675
240261	'Ccdc112'	278	318	495	343	376	460	363.6666667	393	0.123373994	0.785931197	0.972790461
240263	'Fem1c'	846	859	714	756	699	825	806.3333333	760	0.007440919	0.982358905	0.999493374
240283	'Dmxl1'	1649	1866	1046	1510	1708	1574	1520.333333	1597.333333	0.179459776	0.598354213	0.972790461
24030	'Mrps12'	257	282	417	294	295	424	318.6666667	337.6666667	0.098463431	0.824697639	0.972790461
240322	'Adamts19'	2858.8	3199	92	58	6275	4542	2049.923333	3625	0.782884822	0.53866285	0.972790461
240327	'Gm4951'	5	2	11	0	5	16	6	7	-0.019752272	0.989224717	0.999493374
240328	'F830016B08Rik'	5507.7	5529.7	771.27	672.6	6612.7	3831.23	3936.226667	3705.516667	-0.098743867	0.905272528	0.988451813
240332	'Slc6a7'	74	71	4	53	151	62	49.66666667	88.66666667	0.943037917	0.296811399	0.90029104
240334	'Pcyox1l'	102	87	53	65	133	102	80.66666667	100	0.353342958	0.375181669	0.940406075
240354	'Malt1'	859	777.34	485	279	954	719	707.1133333	650.6666667	-0.131635102	0.758475919	0.972790461
240396	'Mex3c'	4304	4546	4281	3332	4939	5815	4377	4695.333333	0.117562644	0.648566528	0.972790461
240427	'Setbp1'	697	640	966	557	504	477	767.6666667	512.6666667	-0.509181316	0.270479313	0.883851074

24044	'Scamp2'	568	585	387	1132	961	1062	513.3333333	1051.666667	1.162325851	0.002236036	0.106013738
240442	'Adnp2'	1335	1332	857	1110	1021	1347	1174.666667	1159.333333	0.082545123	0.808647148	0.972790461
240444	'Kcng2'	24	16	30	3	15	9	23.33333333	9	-1.445324094	0.062676696	0.566066012
24045	'Scamp3'	430.2	465.49	444.59	766.82	589.08	697.45	446.76	684.45	0.724857524	0.0772707	0.611392561
24046	'Scn11a'	12	3	3	16	6	20	6	14	1.362604374	0.174195125	0.793056387
24047	'Ccl19'	3.4	1	1.53	0	3	0	1.976666667	1	-0.777524957	0.74008545	0.972790461
240476	'Zfp407'	1221	1206	509	605	910	762	978.6666667	759	-0.272350615	0.491468249	0.972790461
240479	'Dipk1c'	2	2	2	1	3	2	2	2	-0.004602464	0.997485469	0.999746625
24050	'Septin3'	108	100	155	193	55	42	121	96.66666667	-0.064246961	0.937916185	0.994698142
240505	'Cdc42bpg'	678	644	522	1790	804	949	614.6666667	1181	1.154494671	0.041947143	0.477648737
24051	'Sgcb'	493	465	253	579	688	600	403.6666667	622.3333333	0.73547977	0.045730986	0.498244985
240514	'Ccdc85b'	354.27	331.28	396.64	393.71	475.3	543.85	360.73	470.9533333	0.41691601	0.213905537	0.842446242
240518	'Peli3'	147	147	147	95	70	143	147	102.6666667	-0.461720471	0.295556171	0.90029104
24052	'Sgcd'	295	253	458	7	194	201	335.3333333	134	-1.513802149	0.093777213	0.657893867
24053	'Sgcg'	7	4	0	0	2	4	3.666666667	2	-0.900967647	0.640736743	0.972790461
240549	'Gm4952'	0	0	0	0	2	1	0	1	2.320886	0.562538074	0.972790461
24055	'Sh3bp2'	248	252	363	360	251	312	287.6666667	307.6666667	0.185891391	0.70967402	0.972790461
24056	'Sh3bp5'	1453	1616	1441	1959	1925	2275	1503.333333	2053	0.525596317	0.106388474	0.679124905
24057	'Sh3yl1'	164	148	352	66	115	136	221.3333333	105.6666667	-1.137682763	0.042362758	0.479588013
24058	'Sigirr'	34	33	15	5	33	27	27.33333333	21.66666667	-0.367811075	0.612847239	0.972790461
24059	'Slco2a1'	30	25	40	129	12	50	31.66666667	63.66666667	1.268651357	0.183502446	0.804729813
240590	'Dmrt3'	5	6	6	0	1	0	5.666666667	0.333333333	-3.936798675	0.027361155	0.404817338
240595	'Kcnv2'	16	11	2	0	4	3	9.666666667	2.333333333	-2.058570287	0.125361397	0.72022881
24060	'Slc35a1'	695.05	659.51	752	454	885	771	702.1866667	703.3333333	-0.002663007	0.993402413	0.999493374
24061	'Smc1a'	2308	2406	2113	1242	2461	2394	2275.666667	2032.333333	-0.16218443	0.548111121	0.972790461
240613	'9930021J03Rik'	3159.3	3261.9	1500.2	1279.2	2125	1724.66	2640.5	1709.606667	-0.549263445	0.129905595	0.727578716
240614	'Ranbp6'	719	693	392	390	765	567	601.3333333	574	-0.014521842	0.966174763	0.999493374
24063	'Spry1'	127	128.9	555.86	262	136.74	206	270.5866667	201.58	-0.393489947	0.626038996	0.972790461
240633	'Lipk'	0	2	2	0	0	0	1.333333333	0	-2.855525825	0.472040045	0.972790461
240638	'Slc16a12'	13	11	6	21	11	7	10	13	0.612413473	0.489635206	0.972790461
24064	'Spry2'	345	311.54	1979.5	1044.3	330.85	489.97	878.67	621.69	-0.409407471	0.664602001	0.972790461
240641	'Kif20b'	934	1043	265	185	706	517	747.3333333	469.3333333	-0.650966999	0.268295297	0.883600995
24066	'Spry4'	318	268	291	1360	294	402	292.3333333	685.3333333	1.519781652	0.049238763	0.515210762
240660	'Slc35g1'	1185	1326	238	1591	1715	1575	916.3333333	1627	0.995737227	0.10226296	0.671776916
240665	'Ccnj'	326	347	194	87	325	272	289	228	-0.363312055	0.431986072	0.963731425
240667	'Sec31b'	82	120	20	44	90	43	74	59	-0.209211142	0.76143432	0.972790461
24067	'Srp54a'	856.82	854.66	2083.9	737.93	1370.5	1964.43	1265.133333	1357.626667	-3.21E-04	0.999555616	0.999900097
240672	'Dusp5'	163	169	573	247	111	194	301.6666667	184	-0.659245397	0.389257299	0.951228762
240675	'Vwa2'	8	9	0	1	5	2	5.666666667	2.666666667	-1.014129489	0.513604637	0.972790461
24068	'Sra1'	596	649	1442	693	583	815	895.6666667	697	-0.351750363	0.537933589	0.972790461

24069	'Sufu'	1082	1105	890	714	1123	953	1025.666667	930	-0.09460081	0.714996917	0.972790461
240690	'St18'	4	5	2	2	2	4	3.666666667	2.666666667	-0.399176818	0.744987757	0.972790461
240697	'Mcmdc2'	315.38	348.7	83.07	6.94	117.22	47.18	249.05	57.11333333	-2.169777802	0.013212333	0.287077572
24070	'Mpdu1'	441	503	371	145	705	646	438.3333333	498.6666667	0.118790878	0.816991309	0.972790461
24071	'Synj2bp'	1423.5	1439.7	973.88	707.18	1324.8	1274.66	1279.01	1102.213333	-0.186695131	0.481990514	0.972790461
240725	'Sulf1'	5857	5783	786	548	8574	6483	4142	5201.666667	0.293353346	0.745030822	0.972790461
240726	'Slco5a1'	61	74	49	94	46	32	61.33333333	57.33333333	0.141211612	0.836740268	0.974723675
24074	'Taf7'	856	971	1151	314	868	1406	992.6666667	862.6666667	-0.296699532	0.553688291	0.972790461
24075	'Taf10'	549	625	714	529	563	1078	629.3333333	723.3333333	0.198959832	0.6239903	0.972790461
240752	'Pik3c2b'	250	234	339	74	186	241	274.3333333	167	-0.792344353	0.092139347	0.652602851
240753	'Plekha6'	383	368	223	473	297	176	324.6666667	315.3333333	0.189406372	0.753621095	0.972790461
240754	'Lax1'	16	11	12	19	12	9	13	13.33333333	0.197572297	0.799796181	0.972790461
240755	'Mgat4f'	0	1	1	3	0	0	0.666666667	1	0.973636407	0.754425532	0.972790461
240756	'Klhl12'	955	887	676	763	1085	1432	839.3333333	1093.333333	0.413046562	0.119875307	0.710102513
240776	'Kcnt2'	42	30	43	8	52	57	38.33333333	39	-0.086935091	0.901053148	0.98672375
240816	'Rgsl1'	5	3	0	0	8	4	2.666666667	4	0.557786609	0.765872996	0.972790461
240817	'Teddm2'	8	7	0	0	4	4	5	2.666666667	-0.924909281	0.608360672	0.972790461
24083	'Natd1'	1543	1622	1365	1731	1492	1838	1510	1687	0.258023409	0.463979189	0.972790461
240832	'Tor1aip2'	2821.3	3138.4	4535.4	8156.6	3229.5	4190.09	3498.376667	5192.056667	0.74273005	0.244368286	0.866091613
24084	'Tekt2'	82	79	54	19	55	23	71.66666667	32.33333333	-1.118642019	0.036014366	0.456194572
240843	'Brinp2'	31	24	15	2	40	22	23.33333333	21.33333333	-0.203283102	0.821996049	0.972790461
24086	'Tlk2'	2754	2850	2704	1114	2028	2091	2769.343333	1744.333333	-0.665577017	0.013354992	0.288185595
240869	'Zbtb37'	1537	1613	671	930	1291	1101	1273.67	1107.333333	-0.098246217	0.803235038	0.972790461
24087	'Tll2'	4	5.33	1	0	5	1	3.443333333	2	-0.755072844	0.655656105	0.972790461
240873	'Tnfsf18'	2	0	1	38	4	4	1	15.33333333	4.315765444	0.007590558	0.209755373
240879	'Mettl11b'	1	1	1	0	0	0	1	0	-2.390783043	0.494152846	0.972790461
24088	'Tlr2'	105	97	338	197	67	95	180	119.6666667	-0.467175576	0.573324385	0.972790461
240880	'Scyl3'	899.44	928.42	475.99	466.78	875.05	697.71	767.95	679.8466667	-0.118389446	0.730437491	0.972790461
240888	'Gpr161'	543.81	574	190	158	533	354	435.9366667	348.3333333	-0.296488279	0.573631691	0.972790461
240892	'Dusp27'	11	19	8	1	11	2	12.66666667	4.666666667	-1.45113005	0.180399602	0.801341923
240899	'Lrrc52'	1	1	0	0	0	0	0.666666667	0	-1.703500596	0.673820112	0.972790461
240913	'Adamts4'	212	223	334	2128	228	198	256.3333333	851.3333333	2.088546045	0.041263862	0.475995577
240916	'Vsig8'	3	5	20	24	1	4	9.333333333	9.666666667	0.298232501	0.834212893	0.974723675
240960	'Dnah14'	129	127	21	0	75	91	92.33333333	55.33333333	-0.812339626	0.518949169	0.972790461
24099	'Tnfsf13b'	16	13	54	19	8	3	27.66666667	10	-1.31650314	0.221106708	0.849076011
24100	'Tpra1'	674	671	309	270	857	595	551.3333333	574	0.072634616	0.872615295	0.980171118
241035	'Pkhd1'	60	66	14	2	73	71	46.66666667	48.66666667	-0.005099053	0.996025142	0.999602178
24105	'Rbck1'	1037	1114	781	391	1305	1236	977.3333333	977.3333333	-0.035312162	0.931716307	0.993601701
241062	'Pgap1'	374	387	156	541	474	416	305.6666667	477	0.812838472	0.094644713	0.660077618
241066	'Carf'	616	634	235	213	579	377	495	389.6666667	-0.300982688	0.527775044	0.972790461

241070	'Gpr1'	0	1	0	0	1	0	0.333333333	0.333333333	0.058500858	0.988561293	0.999493374
241075	'Plekhm3'	896	962	283	1277	552	648	713.6666667	825.6666667	0.483258585	0.467128761	0.972790461
24108	'Ubd'	0	0	1	0	0	0	0.333333333	0	-0.903279821	0.824807108	0.972790461
24109	'Ubl3'	1046	1018	1619	1892	1071	1397	1227.666667	1453.333333	0.355486514	0.518701278	0.972790461
24110	'Usp18'	42	21	99	32	16	151	54	66.33333333	0.143850702	0.880025524	0.98198411
241112	'Catip'	12	9	1	7	5	4	7.333333333	5.333333333	-0.24481352	0.829730542	0.973695269
241113	'Prkag3'	4	0	9	9	7	2	4.333333333	6	0.549336882	0.713815424	0.972790461
241116	'Cfap65'	8	6	0	1	8	1	4.666666667	3.333333333	-0.424146191	0.798953128	0.972790461
241118	'Asic4'	32	51	5	4	281	116	29.33333333	133.6666667	2.145901075	0.062945188	0.566357744
241128	'Fam124b'	84	76	4	0	103	30	54.66666667	44.33333333	-0.333615233	0.816913545	0.972790461
24113	'Vax2'	1	2	1	4	0	0	1.333333333	1.333333333	0.43999165	0.852228221	0.975734242
241134	'Nyap2'	4	4	0	2	1	2	2.666666667	1.666666667	-0.488623449	0.775302487	0.972790461
24115	'Best1'	18	40	22	34	50	38	26.66666667	40.66666667	0.675556473	0.216453123	0.843391934
241158	'Ankmy1'	25.95	23.74	3.21	48.57	25.66	24.73	17.63333333	32.98666667	1.196747751	0.176784105	0.796450095
241159	'Neu4'	1	1	0	0	0	1	0.666666667	0.333333333	-0.744986434	0.839556299	0.974723675
24116	'Nelfa'	563	560	890	425	552	665	671	547.3333333	-0.301402277	0.47450711	0.972790461
24117	'Wif1'	20	21	2	3	13	5	14.33333333	7	-0.966688731	0.364595678	0.935515019
241175	'Cntnap5b'	15	28	30	0	28	11	24.33333333	13	-1.057421467	0.360330653	0.933706718
241197	'Serpinb10'	0	1	0	0	1	1	0.333333333	0.666666667	0.817382893	0.82412705	0.972790461
241201	'Cdh7'	12	12	11	2	24	7	11.66666667	11	-0.152888179	0.873876597	0.980171118
241226	'Itga8'	297	267	8	2188	301	237	190.6666667	908.6666667	2.74113277	0.024154975	0.384033173
241230	'St8sia6'	38	44	13	5	30	23	31.66666667	19.33333333	-0.72064382	0.33856632	0.924608313
241263	'Gpr158'	0	0	0	0	2	1	0	1	2.320886	0.562538074	0.972790461
24127	'Xrn1'	1823	1930	1034	1209	2015	1756	1595.666667	1660	0.119997016	0.699388139	0.972790461
241274	'Pnpla7'	372	356	139	89	465	507	289	353.6666667	0.253385791	0.678573473	0.972790461
241275	'Noxa1'	7	7	20	4	3	3	11.33333333	3.333333333	-1.748441235	0.096273784	0.662981155
24128	'Xrn2'	2818	2851	3046	2356	3204	3453	2905	3004.333333	0.075431949	0.793226933	0.972790461
241289	'Ppp1r26'	1095	1035	323	100	966	918	817.6666667	661.3333333	-0.358145204	0.628223815	0.972790461
241296	'Lrrc8a'	656	594	576	769	664	892	608.6666667	775	0.43231529	0.226061752	0.85360199
241303	'Fam78a'	69	66	85	95	113	151	73.33333333	119.6666667	0.72321772	0.073662071	0.599721598
241308	'Ralgps1'	1052	924	210	294	764	803	728.6666667	620.3333333	-0.190833227	0.749173328	0.972790461
24131	'Ldb3'	46	66	32	8	43	17	48	22.66666667	-1.089174336	0.106202234	0.679124905
241311	'Zbtb34'	1039	1042	825	1158	766	865	968.6666667	929.6666667	0.094548565	0.83341125	0.974723675
24132	'Zfp53'	311.38	361.03	425.74	247	374.94	359.01	366.05	326.9833333	-0.152919699	0.668776461	0.972790461
241322	'Zbtb6'	588	616	530	356	519	500	578	458.3333333	-0.293250635	0.256143507	0.876684814
241324	'Crb2'	700	642	36	177	554	584	459.3333333	438.3333333	-0.017845373	0.983658585	0.999493374
241327	'Olfml2a'	58	33	2	405	57	111	31	191	3.044289079	0.006454595	0.190255454
24135	'Zfp68'	1011	1080	1212	787	1239	976	1101	1000.666667	-0.112329247	0.739916382	0.972790461
24136	'Zeb2'	832	827	2858	1273	1153	1421	1505.666667	1282.333333	-0.252045733	0.710054955	0.972790461
241391	'Galnt5'	15	6	3	0	16	7	8	7.666666667	-0.123137984	0.927276036	0.992812524

241431	'Xirp2'	0	4	8	0	1	1	4	0.666666667	-2.741390069	0.200205026	0.82759347
241447	'Cers6'	594	549	474	568	1039	1037	539	881.3333333	0.723780542	0.005180379	0.169516979
241452	'Dhrs9'	2	6	30	51	6	13	12.66666667	23.33333333	1.061551296	0.411745644	0.95722888
241489	'Pde11a'	8	4	3	0	2	0	5	0.666666667	-2.894489466	0.113835833	0.698948431
241490	'Rbm45'	641	660	443	527	665	753	581.3333333	648.3333333	0.222727316	0.408689732	0.957157474
241494	'Zfp385b'	279	295	321	67	306	378	298.3333333	250.3333333	-0.356367659	0.52081531	0.972790461
241514	'Zfp804a'	8	7	50	5	23	18	21.66666667	15.33333333	-0.699198019	0.508007015	0.972790461
241516	'Fsip2'	0	0	1	1	2	0	0.333333333	1	1.582610237	0.62491275	0.972790461
241520	'Fam171b'	123	119	189	25	112	131	143.6666667	89.33333333	-0.807570375	0.181709499	0.802050906
241525	'Ypel4'	111	130	51	217	154	73	97.33333333	148	0.847973476	0.202269355	0.8304652
241528	'Lrrc55'	8	11	18	18	14	4	12.33333333	12	0.09663422	0.919335944	0.990815958
241547	'Harbi1'	562	557	438.98	218	375	423	519.3266667	338.6666667	-0.598262152	0.023110763	0.377626024
241556	'Tspan18'	1536	1592	1639	317	3344	2784	1589	2148.333333	0.309329825	0.651386485	0.972790461
241568	'Lrrc4c'	135.01	96	105	83	164	110	112.0033333	119	0.112757754	0.774893432	0.972790461
241576	'Ldlrad3'	296	349	246	609	684	602	297	631.6666667	1.184522668	4.78E-04	0.042360559
241589	'D430041D05Rik'	96	93	10	0	11	11	66.33333333	7.333333333	-3.206901605	0.003853136	0.141968654
241593	'Pin1rt1'	0	1	0	0	2	1	0.333333333	1	1.368205316	0.684632717	0.972790461
241624	'Exd1'	187	176	22	134	161	381	128.3333333	225.3333333	0.885975914	0.234224678	0.861206181
241627	'Wdr76'	345	402	149	114	313	640	298.6666667	355.6666667	0.220565145	0.70886809	0.972790461
241633	'Atp8b4'	41	72	25	119	81	48	46	82.66666667	1.077069644	0.112117507	0.69684179
241634	'Spdye4c'	0	1	0	0	0	0	0.333333333	0	-0.903279821	0.824807108	0.972790461
241636	'Tgm6'	1	0	1	0	0	0	0.666666667	0	-1.858475606	0.644939423	0.972790461
241638	'Lzts3'	403	439	145	682	382	393	329	485.6666667	0.799254629	0.184706951	0.804884883
241639	'Fermt1'	56	56	5	21	27	29	39	25.66666667	-0.466036084	0.576841946	0.972790461
241656	'Pak5'	14	23	3	2	11	9	13.33333333	7.333333333	-0.842521929	0.403347418	0.955996639
241688	'Dzank1'	424	398	134	105	293	257	318.6666667	218.3333333	-0.514792079	0.313773923	0.913268497
241694	'Ralgapa2'	1418	1343	981	1059	815	939	1247.333333	937.6666667	-0.276566933	0.489071086	0.972790461
241727	'Snph'	117	117	111	96	99	83	115	92.66666667	-0.220054312	0.592810691	0.972790461
241732	'Tspyl3'	282	362	176	33	308	296	273.3333333	212.3333333	-0.442382253	0.519950859	0.972790461
241764	'L3mbtl1'	16	24	3	4	19	15	14.33333333	12.66666667	-0.155021031	0.868401475	0.979229375
241770	'Rims4'	68	78	22	15	59	79	56	51	-0.149673431	0.82406511	0.972790461
241794	'Kcng1'	73	72	4	4	58	26	49.66666667	29.33333333	-0.759821405	0.484345351	0.972790461
241846	'Lsm14b'	2085	1889	1306	954	1975	3999	1760	2309.333333	0.354158711	0.446742658	0.970198456
241850	'Abhd16b'	11	14	2	6	7	5	9	6	-0.434750195	0.663209162	0.972790461
241877	'Slc10a5'	0	3	0	84	3	0	1	29	5.41566724	0.015035104	0.305758542
241915	'Phc3'	1923	1854	1761	2204	1923	1733	1846	1953.333333	0.200004613	0.619410258	0.972790461
241919	'Slc7a14'	1	1	2.14	2.22	2.04	2	1.38	2.086666667	0.620045628	0.695394035	0.972790461
241943	'Ccdc144b'	1	1	1	1	2	0	1	1	0.084466755	0.968507372	0.999493374
241944	'Zfp267'	667	686	421	456	751	621	591.3333333	609.3333333	0.103530134	0.726608598	0.972790461
241950	'Bbs12'	388	396	142	108	409	234	308.6666667	250.3333333	-0.282002922	0.604597956	0.972790461

241989	'Pabpc4l'	253	286	146	74	362	290	228.3333333	242	0.047655976	0.928934799	0.993096978
242022	'Frem2'	1124.1	1052.8	132.35	575.69	887.15	251	769.7433333	571.28	-0.234903716	0.768572445	0.972790461
242037	'Ankub1'	1	0	0	0	0	1	0.333333333	0.333333333	0.058500858	0.988561293	0.999493374
242050	'Igsf10'	677.72	616.93	156.06	261.52	956.64	661.05	483.57	626.4033333	0.403389388	0.50436294	0.972790461
242083	'Ppm1l'	1001	991	526	1335	1679	1520	839.3333333	1511.333333	0.949256917	0.00585657	0.180170079
242093	'Rxfp4'	0	0	0	1	1	0	0	0.666666667	2.050872943	0.610671515	0.972790461
242100	'Pglyrp3'	0	0	51	3	0	5	17	2.666666667	-2.799714398	0.235146337	0.861636582
242109	'Zfp697'	355.93	366.19	181.48	346.44	723.1	489.99	301.2	519.8433333	0.844799797	0.028167872	0.409664118
242122	'Vtcn1'	2	2	3	1	3	1	2.333333333	1.666666667	-0.492930672	0.743541676	0.972790461
242125	'Mab21l3'	73	50	999	173	34	69	374	92	-1.938097669	0.107487251	0.683111836
242126	'Slc22a15'	112	101	92	98	122	115	101.6666667	111.6666667	0.198429012	0.564818073	0.972790461
242202	'Pde5a'	741	766	599	341	607	661	702	536.3333333	-0.373510006	0.146983488	0.753611197
242235	'Lrit3'	2	2	1	0	3	3	1.666666667	2	0.192074924	0.912394261	0.989773583
242248	'Bank1'	2	5	1	38	1	19	2.666666667	19.33333333	3.188551412	0.020440897	0.355230659
242253	'Wdr63'	3	0	3	0	0	2	2	0.666666667	-1.737392423	0.530390076	0.972790461
242259	'Slc44a5'	19	16	4	1	13	9	13	7.666666667	-0.780807171	0.450570514	0.970590732
242274	'Lrrc7'	226.78	220.3	94.27	145.22	169.4	101.77	180.45	138.7966667	-0.228664602	0.650779244	0.972790461
242291	'Bpnt2'	2024	1930	1400	3075	2482	2777	1784.666667	2778	0.769641042	0.046540847	0.502746922
242297	'Fam110b'	266	283	267	102	296	271	272	223	-0.325251621	0.408026917	0.957157474
242316	'Gdf6'	65	50	27	40	41	22	47.33333333	34.33333333	-0.302309742	0.618374022	0.972790461
242341	'Atp6v0d2'	0	3	39	3	5	30	14	12.66666667	-0.448774716	0.783826811	0.972790461
242362	'Manea'	803	843	540	683	907	997	728.6666667	862.3333333	0.306590672	0.250491129	0.871106156
242377	'Pm20d2'	408	464	649	4	457	420	507	293.6666667	-0.97831627	0.361112255	0.933706718
242384	'Lingo2'	0	2	2	4	9	13	1.333333333	8.666666667	2.642278289	0.0441622	0.489855158
242406	'Rgp1'	1672.4	1612.7	1394	654	1495.8	1340.81	1559.72	1163.533333	-0.428799111	0.153711648	0.765411885
242408	'Fam221b'	17	13	7	0	11	8	12.33333333	6.333333333	-1.03436977	0.358950624	0.933693976
242409	'Tmem8b'	795	730	276	123	575	565	600.3333333	421	-0.535274883	0.3573944	0.932505998
242418	'Dcaf10'	1340.2	1352	1445	824	1096	1121	1379.05	1013.666667	-0.409768164	0.167697236	0.782203104
242425	'Gabbr2'	8	5	2	1	3	2	5	2	-1.272009136	0.318883712	0.916136994
242443	'Grin3a'	54	47	9	34	76	22	36.66666667	44	0.390421376	0.622036108	0.972790461
242466	'Zfp462'	1521	1596	475	1220	1688	1087	1197.333333	1331.666667	0.293669116	0.567694754	0.972790461
242474	'Tmem245'	2382.2	2377.6	706.34	6257.2	2516.3	2280.18	1822.06	3684.553333	1.323970571	0.063900228	0.5684378
242484	'D630039A03Rik'	1168	1185	191	713	748	751	848	737.3333333	-0.034066016	0.957670067	0.999493374
242505	'Rasef'	64	58	148	4	35	44	90	27.66666667	-1.888450765	0.021920692	0.366505149
242506	'Frmd3'	14	8	75	4	14	3	32.33333333	7	-2.342976405	0.039693449	0.464973418
242509	'Bnc2'	2947	2899	964	397	2309	1994	2270	1566.666667	-0.562447703	0.367985248	0.937190492
242521	'Klh19'	2338	2454	3433	2187	2967	3147	2741.666667	2767	0.01877651	0.960642018	0.999493374
242523	'Dmrta1'	413	419	458	38	566	730	430	444.6666667	-0.104771503	0.895427746	0.985505807
242553	'Kank4'	721	734	312	135	594	402	589	377	-0.652274432	0.217861466	0.844165262
242557	'Atg4c'	409	459	287	344	488	468	385	433.3333333	0.23539505	0.403960478	0.956543098

242570	'Raver2'	463.33	466.56	99.55	81.06	471.47	481.26	343.1466667	344.5966667	-0.010954576	0.98775191	0.999493374
242574	'C130073F10Rik'	0	1	0	2	1	0	0.333333333	1	1.757594831	0.586247018	0.972790461
242584	'Wdr78'	169	176	88	123	191	119	144.3333333	144.3333333	0.097012441	0.819456418	0.972790461
242585	'Slc35d1'	597	599	317	275	730	624	504.3333333	543	0.119036839	0.759142285	0.972790461
242594	'Fyb2'	2	4	1	14	21	23	2.333333333	19.33333333	3.107720454	5.51E-04	0.04617786
242602	'Lexm'	0	1	5	0	0	0	2	0	-3.545962815	0.332224827	0.921648675
242603	'Cdcp2'	1	1	0	1	2	1	0.666666667	1.333333333	1.104064927	0.607441764	0.972790461
242607	'Slc1a7'	0	1	0	0	0	0	0.333333333	0	-0.903279821	0.824807108	0.972790461
242608	'Podn'	32	37	18	8	30	28	29	22	-0.408119966	0.506263115	0.972790461
242620	'Dmrta2'	76	76	38	4	61	156	63.33333333	73.66666667	0.102544549	0.913196979	0.989773583
242627	'Skint5'	1	0	0	0	5	6	0.333333333	3.666666667	3.214379048	0.205452097	0.833294914
242642	'Hpdl'	7	6	9	4	4	23	7.333333333	10.33333333	0.407427224	0.687151271	0.972790461
242646	'Tctex1d4'	1	0	0	0	1	2	0.333333333	1	1.346160497	0.689889638	0.972790461
242653	'Cldn19'	1	1	3	2	0	1	1.666666667	1	-0.601505002	0.76300497	0.972790461
242662	'Rims3'	467	402	67	24	155	503	312	227.3333333	-0.518903315	0.581536589	0.972790461
242667	'Dlgap3'	201	202	95	44	241	263	166	182.6666667	0.088038517	0.884056284	0.983608145
242669	'Azin2'	339	351	127	700	324	400	272.3333333	474.6666667	1.049221045	0.090572538	0.649553032
242681	'Rab42'	49	51	4	35	68	69	34.66666667	57.33333333	0.818289059	0.313404997	0.91289349
242687	'Wasf2'	3171	3177	3615	2259	2723	3111	3321	2697.666667	-0.265813215	0.403121023	0.955996639
242691	'Gpatch3'	222	211	112	58	203	250	181.6666667	170.3333333	-0.124055417	0.80729431	0.972790461
242700	'Ifnlr1'	29	12	25	75	64	47	22	62	1.608928125	0.012724688	0.280651706
242702	'Myom3'	367	380	45	39	135	169	264	114.3333333	-1.185438704	0.122285224	0.71347226
242705	'E2f2'	112	109	38	98	181	109	86.33333333	129.3333333	0.678488081	0.184927515	0.804884883
242707	'Lactbl1'	2	0	0	0	0	1	0.666666667	0.333333333	-0.74959284	0.853034393	0.975734242
242711	'Cela3a'	1	2	0	3	3	1	1	2.333333333	1.43002818	0.435908556	0.96581102
242721	'Klhdc7a'	293	322	38	158	393	347	217.6666667	299.3333333	0.531064981	0.452472472	0.970649024
242726	'Padi6'	17	20	1	0	49	1035	12.66666667	361.3333333	4.742417161	0.039248592	0.46244458
242735	'Lrrc38'	2	5	2	57	14	50	3	40.33333333	3.938904021	4.74E-05	0.008204297
242736	'Pramel12'	867	781	587	364	689	775	745	609.3333333	-0.277926401	0.331598207	0.921648675
242747	'Zfp933'	361.07	394.99	347.35	512	570	463	367.8033333	515	0.577441264	0.109931304	0.689773582
242748	'Disp3'	7	8	5	17	24	17	6.666666667	19.33333333	1.612873039	0.021366876	0.362154416
242773	'Slc45a1'	1	0	1	5	0	0	0.666666667	1.666666667	1.722085122	0.55755902	0.972790461
242785	'Klhl21'	591	502	1250	1099	399	1042	781	846.6666667	0.19060818	0.782080647	0.972790461
242800	'Ttc34'	36	24	12	1	7	29	24	12.33333333	-1.046182121	0.308126492	0.907963267
242805	'Ankrd65'	0	1	0	0	1	0	0.333333333	0.333333333	0.058500858	0.988561293	0.999493374
242819	'Rundc3b'	668	652	808	44	464	471	709.3333333	326.3333333	-1.2637038	0.075422594	0.605290989
242838	'Lrrd1'	0	1	0	1	0	0	0.333333333	0.333333333	0.058500858	0.988561293	0.999493374
242851	'Gnat3'	1	0	0	0	0	0	0.333333333	0	-0.903279821	0.824807108	0.972790461
242860	'Rsbn1l'	909	895	701	866	816	754	835	812	0.076672798	0.835613944	0.974723675
242864	'Napepld'	324	299	104	391	414	339	242.3333333	381.3333333	0.810225044	0.10829334	0.685149076

242891	'Cct8l1'	2	2	0	2	3	0	1.333333333	1.666666667	0.54104676	0.804230483	0.972790461
242894	'Actr3b'	107	94	211	127	151	290	137.3333333	189.3333333	0.403212399	0.476226539	0.972790461
242915	'Garem2'	20	23	25	16	8	7	22.66666667	10.33333333	-0.976595143	0.205492079	0.833294914
242939	'Cpz'	44	51	9	7	75	56	34.66666667	46	0.380618717	0.665804552	0.972790461
242960	'Fbxl5'	3836	3958	1955	2511	2738	2961	3249.666667	2736.666667	-0.137650943	0.701699486	0.972790461
243025	'Tmem156'	2	14	0	5	9	11	5.333333333	8.333333333	0.743691433	0.590579947	0.972790461
243043	'Kctd8'	12	9	51	17	26	22	24	21.66666667	-0.228369303	0.798719403	0.972790461
243078	'Tecrl'	14	18	3	2	4	7	11.66666667	4.333333333	-1.379756577	0.17702426	0.79682499
243083	'Tmprss11f'	8	7	1	0	17	22	5.333333333	13	1.214880301	0.392022484	0.952648938
243084	'Tmprss11e'	0	1	0	1	0	1	0.333333333	0.666666667	1.054274597	0.766617507	0.972790461
243085	'Ugt2b35'	0	0	0	1	0	0	0	0.333333333	1.020273531	0.802557913	0.972790461
243168	'Hsd17b13'	0	4	14	0	6	1	6	2.333333333	-1.604365456	0.421422891	0.959296626
243197	'Mfsd7a'	70	60	21	4	74	40	50.33333333	39.33333333	-0.40734461	0.644654634	0.972790461
243219	'2900026A02Rik'	726	614	409	410	1033	1054	583	832.3333333	0.51086866	0.16453383	0.777691433
243270	'Hcar1'	9	7	2	2	7	7	6	5.333333333	-0.148831919	0.88770719	0.984534762
243272	'Sbno1'	6769	6577	3347	4397	4810	4552	5564.333333	4586.333333	-0.157712741	0.673354109	0.972790461
243274	'Tmem132d'	15	8	60	12	14	34	27.66666667	20	-0.609280682	0.527536819	0.972790461
243277	'Adgrd1'	17	9	2	2	2	15	9.333333333	6.333333333	-0.562254413	0.645455446	0.972790461
243300	'Nyap1'	206	189	800	3340	183	847	398.3333333	1456.666667	2.107094072	0.055713367	0.542079085
243302	'Gm4963'	74.75	65.9	152.31	1.15	1.05	0	97.65333333	0.733333333	-7.193664165	5.54E-09	4.39E-06
243308	'A430033K04Rik'	208	246	128	37	313	258	194	202.6666667	-0.007294487	0.991139323	0.999493374
243312	'Elfn1'	414	412	395	94	347	268	407	236.3333333	-0.834677134	0.058269906	0.551665011
243328	'Slc29a4'	20	16	17	12	21	11	17.66666667	14.66666667	-0.220009331	0.732688737	0.972790461
243339	'Tmem130'	71	55	88	13	54	26	71.33333333	31	-1.262269845	0.038720917	0.460965603
243362	'Stard13'	193	223	139	548	289	208	185	348.3333333	1.151440338	0.061726654	0.563910613
243369	'Sspo'	89	71	45	55	119	55	68.33333333	76.33333333	0.224188515	0.654566432	0.972790461
243371	'Lrrc61'	192	203	175	42	193	180	190	138.3333333	-0.527075233	0.303607684	0.906520549
243372	'Zfp775'	159	152	137	94	180	223	149.3333333	165.6666667	0.141602834	0.674699541	0.972790461
243374	'Gimap8'	81	96.83	14	7	135.98	81	63.94333333	74.66	0.188555031	0.843609926	0.975182082
243376	'Doxl2'	0	0	0	0	0	1	0	0.333333333	1.020273531	0.802557913	0.972790461
243382	'Ppm1k'	995	1070	551	1373	1171	750	872	1098	0.518726077	0.30462598	0.907164095
243385	'Gprin3'	112	126	19	91	161	144	85.66666667	132	0.726024829	0.265919249	0.882313572
243407	'Qrfprl'	5	6	0	0	2	3	3.666666667	1.666666667	-1.149851828	0.551749128	0.972790461
243499	'Lrrtm4'	141.07	151.6	35.24	56.79	184.28	103.87	109.3033333	114.98	0.119642528	0.848831159	0.975686757
243510	'Ccdc142'	318	344	67	106	381	191	243	226	-0.051604014	0.937750136	0.994698142
243529	'H1f10'	737	735	537	60	506	496	669.6666667	354	-1.014541613	0.11571186	0.702825844
243537	'Uroc1'	0	0	0	4	4	0	0	2.666666667	4.064929245	0.210572237	0.839637916
243538	'Cfap100'	10	14	3	0	15	6	9	7	-0.410778555	0.754264836	0.972790461
243547	'Grip2'	9	15	3	4	7	5	9	5.333333333	-0.659805597	0.493822677	0.972790461
243548	'Prickle2'	277	291	156	298	449	519	241.3333333	422	0.859372931	0.00700879	0.197779062

243574	'Kbtbd8'	208	241	55	48	196	368	168	204	0.248426004	0.730748917	0.972790461
243612	'Ssu2'	0	1	0	1	6	7	0.333333333	4.666666667	3.619450755	0.071130578	0.591632421
243616	'Slc6a11'	6	8	1	0	2	6	5	2.666666667	-0.951175456	0.537508717	0.972790461
243621	'Iqsec3'	32	25	16	23	9	2	24.33333333	11.33333333	-0.787514987	0.437479619	0.96622803
243628	'Vmn2r24'	1	0	0	0	3	0	0.333333333	1	1.381884369	0.730152291	0.972790461
243634	'Ano2'	5	5	4	2	21	10	4.666666667	11	1.170721509	0.258427384	0.877564912
243653	'Clec1a'	6	14	7	1	31	24	9	18.66666667	0.948838753	0.37581996	0.940406075
243655	'Klre1'	0	0	1	0	0	0	0.333333333	0	-0.903279821	0.824807108	0.972790461
243659	'Styk1'	12	18	16	27	37	25	15.33333333	29.66666667	1.01347304	0.084630834	0.633124552
243725	'Ppp1r9a'	2235.5	2230.8	834.43	702.26	3217.3	1710.25	1766.913333	1876.586667	0.091993025	0.870029286	0.979794316
243743	'Plxna4'	3499	3171	662	2177	4208	4022	2444	3469	0.587895953	0.312005262	0.911537035
243753	'Slc23a4'	16	10	14	1	8	7	13.33333333	5.333333333	-1.3910725	0.125848569	0.72022881
243755	'Slc13a4'	4	6	1	4	6	4	3.666666667	4.666666667	0.460375479	0.689584625	0.972790461
243764	'Chrm2'	1	3	2	0	14	5	2	6.333333333	1.544958517	0.333261287	0.921648675
243771	'Parp12'	955	968	475	186	573	793	799.3333333	517.3333333	-0.654880335	0.180958729	0.801479605
243780	'Dennd1l'	747.88	770.99	706.68	387.88	709.56	835.15	741.85	644.1966667	-0.209203793	0.469206763	0.972790461
243813	'Leng9'	41	51	45	31	79	77	45.66666667	62.33333333	0.425393184	0.34413239	0.925308103
243816	'Gp6'	1	0	2	2	1	2	1	1.666666667	0.762579973	0.694270469	0.972790461
243819	'Ppp6r1'	3215	3426	3150	1922	2729	3602	3263.666667	2751	-0.232819582	0.392048174	0.952648938
243822	'Fam71e2'	1	1	0	1	1	0	0.666666667	0.666666667	0.24523346	0.929078356	0.993096978
243833	'Zfp128'	208	204	159	81	217	201	190.3333333	166.3333333	-0.20874897	0.572482822	0.972790461
243834	'Zfp324'	295	287	286	205	293	323	289.3333333	273.6666667	-0.054644962	0.848601075	0.975686757
243842	'Bicra'	1064	1089	793	991	857	958	982	935.3333333	0.047143403	0.897068542	0.985794067
243846	'Ccdc9'	1202.4	1150.5	1293.6	571.07	1268.7	1043.79	1215.49	961.17	-0.355208307	0.292640382	0.900043079
243853	'Fkrp'	493	491	413	176	613	549	465.6666667	446	-0.107480927	0.799163963	0.972790461
243862	'Psg22'	0	4.85	0	6	5	4	1.616666667	5	2.116944427	0.229057845	0.855540033
243864	'Mill2'	90.49	66.12	6	10	156	92	54.20333333	86	0.652308018	0.522695652	0.972790461
243866	'Meiosin'	1978.7	1845.4	951.29	8	1255.8	1702.84	1591.803333	988.8766667	-0.808890411	0.472633221	0.972790461
243867	'Fbxo46'	312	348	327	224	321	346	329	297	-0.120865583	0.674735909	0.972790461
243874	'Nlrp9b'	5	5	1	2	0	2	3.666666667	1.333333333	-1.264250452	0.446252736	0.970172328
243880	'Nlrp4a'	1	2	0	1	0	1	1	0.666666667	-0.359181764	0.890454647	0.984980969
243881	'Cyp2b23'	0	0	0	1	0	1	0	0.666666667	2.022653929	0.615796212	0.972790461
243897	'Ggn'	18	22	44	31	19	18	28	22.66666667	-0.212159796	0.780060237	0.972790461
243905	'Zfp568'	1517	1467	1398	465	1179	869	1460.666667	837.6666667	-0.814141368	0.017363607	0.329129504
243906	'Zfp14'	394	392.1	90	138	267	178	292.0333333	194.3333333	-0.485494694	0.399690925	0.955719264
243910	'Nfkbid'	29	41	25	32	62	47	31.66666667	47	0.607212156	0.189202924	0.811992322
243911	'Kirrel2'	2	1	1	0	3	0	1.333333333	1	-0.470877137	0.84516253	0.975182082
243912	'Hspb6'	125	124	272	62	208	174	173.6666667	148	-0.34343614	0.56649173	0.972790461
243914	'Lgi4'	9	11	10	28	20	27	10	25	1.421467675	0.030759696	0.425931054
243923	'Rgs9bp'	40	25	77	35	42	61	47.33333333	46	-0.083787424	0.897149156	0.985794067

243931	'Tshz3'	244	234	535	391	226	176	337.6666667	264.3333333	-0.229371546	0.738809242	0.972790461
243937	'Zfp536'	41	30	9	7	16	29	26.66666667	17.33333333	-0.604689659	0.428296896	0.962191208
243958	'Siglecg'	0	4	18	0	5	1	7.333333333	2	-2.126754443	0.282568218	0.890642259
243961	'Shank1'	375	322	74	87	226	76	257	129.6666667	-0.884291239	0.195072958	0.819942118
243963	'Zfp473'	373	332	87	36	138	211	264	128.3333333	-1.056798718	0.117787064	0.706468633
243967	'Ntn5'	18.19	13.61	2	2.48	7.12	4.46	11.26666667	4.686666667	-1.277830494	0.241215465	0.86449675
243978	'Mrgprx2'	0	0	0	2	0	1	0	1	2.714766126	0.495614689	0.972790461
243979	'Mrgprb2'	0	1	0	0	5	0	0.333333333	1.666666667	2.111857871	0.592799815	0.972790461
243983	'Zdhhc13'	423	465	394	205	391	423	427.3333333	339.6666667	-0.330943892	0.252143545	0.871252558
244049	'Mctp2'	19	23	15	95	58	159	19	104	2.526812597	8.59E-06	0.002236954
244058	'Rgma'	176	150	254	350	151	201	193.3333333	234	0.428209686	0.499336177	0.972790461
244059	'Chd2'	4051	4016	3870	1474	3028	2420	3979	2307.333333	-0.781265723	0.008163396	0.218179228
244071	'Agbl1'	0	1	0	0	0	0	0.333333333	0	-0.903279821	0.824807108	0.972790461
244091	'Fsd2'	6	7	1	0	1	2	4.666666667	1	-2.219949336	0.18419959	0.804780995
244141	'Nars2'	616.36	642.25	544.78	630.22	555.55	482.47	601.13	556.08	0.012453746	0.97564439	0.999493374
244144	'Usp35'	482	456	279	480	469	370	405.6666667	439.6666667	0.25706519	0.538316507	0.972790461
244152	'Tsku'	224	218	596	182	297	334	346	271	-0.42735483	0.472259614	0.972790461
244183	'Trim30b'	0	0	0	1	4	3	0	2.666666667	3.814054989	0.140272144	0.74306325
244187	'Olfr684'	1	1	0	0	1.51	0	0.666666667	0.503333333	-0.74517548	0.838722495	0.974723675
244198	'Olfml1'	155	143	22	103	189	166	106.6666667	152.6666667	0.618133228	0.35404694	0.93103461
244199	'Ovch2'	0	0	0	1	0	0	0	0.333333333	1.020273531	0.802557913	0.972790461
244202	'Nlrp10'	68	83	91	61	49	55	80.66666667	55	-0.467233852	0.339957667	0.925145808
244209	'Cyp2r1'	42	66	12	3	42	40	40	28.33333333	-0.539492613	0.558494835	0.972790461
244216	'Zfp771'	262.86	280.23	377	263	389	444	306.6966667	365.3333333	0.245716599	0.52009256	0.972790461
244218	'Ctf2'	3	2	1	1	2	0	2	1	-0.877697373	0.642229564	0.972790461
244219	'Zfp668'	455	464	193	242	398	378	370.6666667	339.3333333	-0.05483628	0.888735589	0.984710124
244234	'5830411N06Rik'	14	18	0	1	16	11	10.66666667	9.333333333	-0.194038185	0.892293316	0.985171
244237	'Tnfrsf26'	1	1	1	16	3	7	1	8.666666667	3.359307408	0.018682935	0.340229851
244238	'Mrgpre'	33	31	23	26	54	72	29	50.66666667	0.799444164	0.093000602	0.654455509
244281	'Myo16'	6	8	12	9	3	2	8.666666667	4.666666667	-0.701238206	0.529257345	0.972790461
244310	'Dlgap2'	34	24	8	1	24	6	22	10.33333333	-1.115516549	0.295682108	0.90029104
244329	'Mcph1'	989	906	449	218	754	902	781.3333333	624.6666667	-0.348736625	0.48345146	0.972790461
244332	'Defb14'	0	1	0	0	0	0	0.333333333	0	-0.903279821	0.824807108	0.972790461
244349	'Kat6a'	3141	3050	1895	1621	2684	2220	2695.333333	2175	-0.248512099	0.392953588	0.952669422
244373	'Erlin2'	1947	1948	969	1174	2590	2788	1621.333333	2184	0.449755639	0.225085912	0.851475917
244416	'Ppp1r3b'	349	246	2810	284	100	202	1135	195.3333333	-2.522339156	0.013181605	0.287066074
244418	'Prag1'	809	749	339	210	725	791	632.3333333	575.3333333	-0.152057572	0.760578255	0.972790461
244421	'Lonrf1'	623	543	1336	228	464	769	834	487	-0.893988245	0.130301573	0.727759547
244431	'Sgcz'	4	6	5	0	4	1	5	1.666666667	-1.653065888	0.25940829	0.877955237
244448	'Triml1'	1	3	0	2	0	0	1.333333333	0.666666667	-0.49090606	0.870687698	0.980019804

244484	'Wdr17'	25	25	2	8	25	11	17.33333333	14.66666667	-0.152816203	0.875174503	0.980678781
244495	'Sgo2b'	1	1	1	0	1	1	1	0.666666667	-0.639776922	0.778463284	0.972790461
244548	'Elmod2'	930	874	357	328	1006	1117	720.3333333	817	0.179393605	0.715292672	0.972790461
244550	'Podnl1'	1	0	0	0	0	1	0.333333333	0.333333333	0.058500858	0.988561293	0.999493374
244551	'Nanos3'	14	10	6	0	9	2	10	3.666666667	-1.498539827	0.238416958	0.864053647
244556	'Zfp791'	132.96	157	257	67	171	163	182.32	133.6666667	-0.521688041	0.310489616	0.910971113
244562	'Abcc12'	5	1	2	1	5	2	2.666666667	2.666666667	-0.007796716	0.995699451	0.999562152
244579	'Tox3'	107.94	102.55	259.36	163.13	85.88	89.67	156.6166667	112.8933333	-0.367768616	0.604867819	0.972790461
244585	'Rpgrip1l'	1156	1306	223	424	1096	832	895	784	-0.122772264	0.84408541	0.975182082
244595	'Ces1a'	0	0	1	3	0	0	0.333333333	1	1.937497013	0.625951132	0.972790461
244608	'Ccdc113'	13	16	8	2	8	8	12.33333333	6	-1.044671517	0.213899136	0.842446242
244631	'Pskh1'	1697	1646	989	731	1586	1634	1444	1317	-0.118118708	0.717690253	0.972790461
244646	'Pkd1l3'	167.2	158.99	94.76	407.98	92.33	79.26	140.3166667	193.19	0.817555791	0.329687854	0.921648675
244650	'Phlpp2'	1211	1173	375	165	1095	894	919.6666667	718	-0.387480705	0.555047847	0.972790461
244653	'Hydin'	20	26	5	0	16	20	17	12	-0.571062859	0.63761027	0.972790461
244654	'Mtss2'	2688	2735.9	1828.7	1064	2956	2456.95	2417.536667	2158.973333	-0.168916974	0.634975832	0.972790461
244666	'Sprtn'	1288	1263	813	925	842	943	1121.333333	903.3333333	-0.19310482	0.591467943	0.972790461
244667	'Disc1'	153	153	128	25	270	178	144.6666667	157.6666667	0.025436969	0.970681077	0.999493374
244668	'Sipa1l2'	2259	2220	1037	3572	2117	2942	1838.666667	2877	0.831484464	0.096977674	0.663693139
244672	'Cwf19l2'	951.04	945.56	986.94	1005.3	953.53	861.5	961.18	940.1233333	0.064813222	0.868225257	0.979229375
244682	'Cntn5'	1679.1	1593.9	617.82	791.7	2198.5	1087.32	1296.943333	1359.16	0.123447858	0.805957485	0.972790461
244694	'Kdm4d'	153	150	18	9	81	65	107	51.66666667	-1.061329988	0.23211811	0.859234717
244698	'Hephl1'	0	0	1	50	0	17	0.333333333	22.33333333	6.300585238	0.00300164	0.122531121
244713	'Zfp317'	1343	1314	1148	599	1351	1002	1268.333333	984	-0.359442255	0.248643569	0.869491261
244721	'Zfp846'	565	546	324	288	561	438	478.3333333	429	-0.109987304	0.73680585	0.972790461
244723	'Olfm2'	19	15	40	93	19	43	24.66666667	51.66666667	1.245931387	0.164773993	0.778361468
244745	'Dpy19l1'	1178	1213	1019	1569	1420	1498	1136.666667	1495.666667	0.501677331	0.160187573	0.771114735
244757	'Glb1l2'	328	338	20	33	550	453	228.6666667	345.3333333	0.568370001	0.579388889	0.972790461
244810	'AW551984'	406	389	393	57	622	311	396	330	-0.368047445	0.593312764	0.972790461
244813	'Bsx'	0	0	0	0	1	0	0	0.333333333	1.020273531	0.802557913	0.972790461
244853	'Nxpe4'	93.77	120	29	36	145	77	80.92333333	86	0.123343761	0.848749592	0.975686757
244859	'Ankk1'	2	2	0	3	2	2	1.333333333	2.333333333	1.005976989	0.554446256	0.972790461
244864	'Layn'	341.05	344.2	209.58	880	590.58	548	298.2766667	672.86	1.360944586	0.00593025	0.181288429
244867	'Arhgap20'	395	376	337	753	505	344	369.3333333	534	0.71806537	0.188000159	0.809755101
244871	'Zc3h12c'	525	544	810	359	659	592	626.3333333	536.6666667	-0.248105093	0.544748552	0.972790461
244879	'Npat'	1866	1955	1223	1040	1490	1425	1681.333333	1318.333333	-0.284361372	0.291518746	0.899440832
244882	'Tnfaip8l3'	19	26	8	1	19	16	17.66666667	12	-0.610547025	0.527849866	0.972790461
244885	'Sh2d7'	2	2.04	0	0	0	1	1.346666667	0.333333333	-1.740010796	0.579911417	0.972790461
244886	'Tmem266'	5	12	17	18	3	13	11.33333333	11.33333333	0.135348492	0.896364983	0.985710789
244891	'Scaper'	2078	2054	1022	166	1350	1628	1718	1048	-0.791339057	0.242739303	0.864872493

244895	'Peak1'	4513	4295	3327	7146	5184	4884	4045	5738	0.663877108	0.137752712	0.739191241
244911	'C2cd4a'	4	6	0	1	1	3	3.333333333	1.666666667	-0.909028496	0.597089339	0.972790461
244923	'Klhl31'	2	3	2	6	1	1	2.333333333	2.666666667	0.486983085	0.749086163	0.972790461
244954	'Prss35'	899.29	797.63	415	41835	2042.7	11360.59	703.9733333	18412.76	5.101227968	2.79E-07	1.29E-04
244958	'Mrap2'	32	30	1	1	25	18	21	14.66666667	-0.536331982	0.671532626	0.972790461
244962	'Snx14'	890	964	419	735	1180	1069	757.6666667	994.6666667	0.468733687	0.200062286	0.82759347
245000	'Atr'	9025	8992	1839	1040	3818	3328	6618.666667	2728.666667	-1.255610011	0.047291081	0.50504201
245007	'Zbtb38'	718	703	394	1029	715	892	605	878.6666667	0.697991362	0.117960475	0.706573583
245020	'Slc35g2'	64.46	77.95	23.04	71.26	73.89	46.86	55.15	64.00333333	0.386626182	0.522113837	0.972790461
245026	'Col6a6'	379.56	335.79	6	28	582.02	426.92	240.45	345.6466667	0.503927961	0.670767709	0.972790461
245038	'Dclk3'	2	7	7	12	6	1	5.333333333	6.333333333	0.472383701	0.712998649	0.972790461
245049	'Myrip'	58	71	30	0	55	94	53	49.66666667	-0.212745776	0.858693471	0.976637967
245050	'Gask1a'	344	372	12	10	752	489	242.6666667	417	0.744824377	0.547656506	0.972790461
245109	'Zscan4c'	5	2	0	0	0	0	2.333333333	0	-3.505057958	0.3270664	0.920734892
245126	'Tarm1'	2	0	0	0	1	0	0.666666667	0.333333333	-0.74959284	0.853034393	0.975734242
245128	'AU018091'	10	9	4	0	3	3	7.666666667	2	-1.968468279	0.117257476	0.70525123
245174	'Zfp937'	544.04	564.37	424.6	288.43	656.83	626.21	511.0033333	523.8233333	0.034137632	0.91185679	0.989773583
245195	'Retnlg'	0	0	0	1	4	1	0	2	3.438591787	0.232083152	0.859234717
245240	'9930111J21Rik2	0.23	0	1.32	0	0	7.1	0.516666667	2.366666667	2.522409821	0.494096088	0.972790461
245263	'Gm4981'	1	3	0	0	2.31	0	1.333333333	0.77	-0.946807057	0.757411937	0.972790461
245269	'Nim1k'	67	58	47	18	83	50	57.33333333	50.33333333	-0.221391161	0.69039589	0.972790461
245282	'Apol10a'	7	8	0	0	0	0	5	0	-4.598892744	0.110934519	0.693550499
245305	'B230307C23Rik'	402.59	486	215	544	595.71	445.63	367.8633333	528.4466667	0.663224294	0.128360977	0.724799196
245347	'Gm4984'	0	2	0	3	3	1	0.666666667	2.333333333	2.025488186	0.354954921	0.931809687
245350	'AA414768'	401	473	67	347	758	1112	313.6666667	739	1.281461509	0.05711667	0.547413448
245368	'Zfp300'	125.06	109.73	31.32	38.74	158.07	92.08	88.70333333	96.29666667	0.143856178	0.823095025	0.972790461
245381	'Sowahd'	12	8	2	3	1	4	7.333333333	2.666666667	-1.314961367	0.279823128	0.889355605
245386	'Tmem255a'	432.33	350	127	186	486.41	185	303.11	285.8033333	-0.004220999	0.994213617	0.999562152
245403	'Dcaf12l2'	122	101	95	24	91	96	106	70.33333333	-0.646678981	0.194930347	0.819942118
245404	'Dcaf12l1'	3333	3531	772	208	3444	2764	2545.333333	2138.666667	-0.303195735	0.721782813	0.972790461
245423	'Gm364'	656	621	97	10	494	1187	458	563.6666667	0.217182985	0.848387573	0.975686757
245424	'Gpr101'	0	2	0	0	1	0	0.666666667	0.333333333	-0.733814424	0.856146772	0.975734242
245440	'Gm4988'	0	3	0	0	1	3	1	1.333333333	0.372790504	0.900312242	0.986604124
245446	'Slitrk4'	16	37	83	7	16	8	45.33333333	10.33333333	-2.202353011	0.010304458	0.250957475
245450	'Slitrk2'	55	64	8	3	57	21	42.33333333	27	-0.663612602	0.516602121	0.972790461
245468	'Pnma3'	4	2	0	3	2	1	2	2	0.248545889	0.887086325	0.984534762
245469	'Pdzd4'	608	592	137	75	471	313	445.6666667	286.3333333	-0.644287028	0.355711228	0.931809687
245474	'Dkc1'	1323	1340	883	334	1104	1336	1182	924.6666667	-0.397295641	0.374954379	0.940406075
245492	'4930595M18Rik'	1	1	11	0	0	0	4.333333333	0	-4.665396866	0.064074431	0.568781147
245511	'4930415L06Rik'	0	0	0	0	1	0	0	0.333333333	1.020273531	0.802557913	0.972790461

245522	'Zc4h2'	248	265	175	75	273	258	229.3333333	202	-0.218790727	0.628711593	0.972790461
245525	'Hsf3'	5	12	0	7	0	7	5.666666667	4.666666667	-0.002056478	0.999102068	0.999900097
245526	'Pgr15l'	11	5	1	0	12	13	5.666666667	8.333333333	0.495264798	0.733240565	0.972790461
245527	'Eda2r'	160	190	151	698	294	450	167	480.6666667	1.71558009	0.002512776	0.112406815
245532	'Awat2'	11	11	8	7	13	16	10	12	0.276927926	0.692863142	0.972790461
245536	'Gm614'	0	1	0	0	1	0	0.333333333	0.333333333	0.058500858	0.988561293	0.999493374
245537	'Nlgn3'	24	18	6	13	28	4	16	15	0.038584398	0.967302353	0.999493374
245545	'Pabpc1l2a'	290.26	206.92	70.39	109.51	182.48	257.77	189.19	183.2533333	0.009537199	0.9859268	0.999493374
245555	'Nexmif'	1276	1317	400	118	1662	1687	997.6666667	1155.666667	0.145200737	0.857134863	0.976149994
245572	'Tbx22'	0	0	0	0	1	0	0	0.333333333	1.020273531	0.802557913	0.972790461
245578	'Pcdh11x'	96	94	75	14	122	125	88.33333333	87	-0.120520151	0.859994159	0.976863639
245595	'Zfp711'	1296	1304	467	174	942	553	1022.333333	556.3333333	-0.887357347	0.136641182	0.737898499
245596	'Hdx'	32	29	20	88	38	45	27	57	1.297770019	0.053586254	0.533767484
245607	'Gprasp2'	1510.9	1620.3	337.96	1162.4	2270.3	1773.52	1156.413333	1735.4	0.679677148	0.229606751	0.856609803
245610	'Nxf3'	82	50	64	722	34	43	65.33333333	266.3333333	2.45672962	0.035094361	0.449302356
245622	'Fam199x'	737	817	594	978	833	1220	716	1010.333333	0.588227274	0.092622537	0.654099957
245631	'Pwwp3b'	1512	1554	830	402	979	754	1298.666667	711.6666667	-0.840809445	0.021371014	0.362154416
245638	'Tbc1d8b'	917	932	1204	1092	712	772	1017.666667	858.6666667	-0.122995223	0.806834989	0.972790461
245643	'Frmpd3'	43	36	63	2	32	16	47.33333333	16.66666667	-1.641098513	0.052360305	0.531770361
245650	'Gucy2f'	215	268	28	2	123	99	170.3333333	74.66666667	-1.239918414	0.261898652	0.879908021
245666	'Iqsec2'	393	394	445	413	348	397	410.6666667	386	-0.002197154	0.995704767	0.999562152
245670	'Rragb'	388	414	97	37	306	255	299.6666667	199.3333333	-0.618595329	0.405418838	0.957157474
245671	'Klf8'	700	752	406	122	771	568	619.3333333	487	-0.398825224	0.489130672	0.972790461
245683	'Klhl34'	35	30	17	5	26	25	27.33333333	18.66666667	-0.5809638	0.395991137	0.954181778
245684	'Cnksr2'	323	292	98	18	220	226	237.6666667	154.6666667	-0.682719219	0.388244463	0.950023663
245688	'Rbbp7'	6445	6126	10108	4380	6122	7464	7559.666667	5988.666667	-0.356261151	0.394366623	0.953178011
245695	'Tceanc'	494	500	235	150	365	374	409.6666667	296.3333333	-0.450332658	0.269418529	0.883851074
245827	'Fat2'	10	6	0	3	12	2	5.333333333	5.666666667	0.188064359	0.898061921	0.985794067
245828	'Trappc1'	381	405	307	261	330	312	364.3333333	301	-0.205727613	0.478497298	0.972790461
245841	'Polr2h'	314	387	660	261	302	404	453.6666667	322.3333333	-0.507845512	0.303165342	0.906189263
245847	'Amdhd2'	162	190	275	149	252	394	209	265	0.283403074	0.546181604	0.972790461
245857	'Ssh3'	500	521	545	442	753	736	522	643.6666667	0.308679844	0.296126713	0.90029104
245860	'Atg9a'	640	694	500	651	908	772	611.3333333	777	0.414465049	0.14311271	0.749054337
245865	'Spag4'	31	30	22	34	35	24	27.66666667	31	0.281556765	0.609638081	0.972790461
245866	'Ift52'	1211	1300	759	329	1016	1133	1090	826	-0.426256023	0.316003758	0.914806078
245867	'Pcmtd2'	1717	1806	1711	1395	2028	1734	1744.666667	1719	0.022612851	0.936902605	0.994413066
245877	'Map7d1'	1374	1363	2136	2569	1458	1665	1624.333333	1897.333333	0.349203179	0.53491128	0.972790461
245880	'Wasf3'	475.72	484.05	114.02	15	243	139.01	357.93	132.3366667	-1.475916531	0.080231051	0.617305615
245884	'Fam71f2'	37	13	132	43	8	15	60.66666667	22	-1.320299492	0.230174533	0.856721174
245886	'Ankrd27'	955	909	888	623	914	1077	917.3333333	871.3333333	-0.055481423	0.832988611	0.974723675

245902	'Ccdc15'	923.37	940.61	240.09	310	688.08	728.68	701.3566667	575.5866667	-0.233714687	0.66255824	0.972790461
245944	'Vps54'	1687	1698	1198	665	1652	1785	1527.666667	1367.333333	-0.174312499	0.611995899	0.972790461
245945	'Rbm47'	163	200	406	565	257	443	256.3333333	421.6666667	0.807270782	0.223272526	0.850672799
246048	'Chodl'	8	6	1	1	5	2	5	2.666666667	-0.855840795	0.520655382	0.972790461
246049	'Slc36a2'	5	7	55	60	1	5	22.33333333	22	0.245348966	0.872190861	0.980171118
246086	'Onecut3'	2	0	1	7	2	0	1	3	1.918996195	0.376980684	0.941504733
246102	'Rttn'	454	404	152	180	395	355	336.6666667	310	-0.069062157	0.880672927	0.982302944
246103	'Atxn7'	945	903	707	555	932	642	851.6666667	709.6666667	-0.206556131	0.508260926	0.972790461
246104	'Rhbdl3'	385	387	89	63	299	235	287	199	-0.523012035	0.427491704	0.961831039
246133	'Kcne2'	15	18	5	2	11	7	12.66666667	6.666666667	-0.912367157	0.322178434	0.918054015
246154	'Vasn'	621	688	499	2641	932	1443	602.6666667	1672	1.699305757	0.005080558	0.16781708
246177	'Myo1g'	5	6	3	6	17	20	4.666666667	14.33333333	1.608258392	0.056430962	0.545987502
246179	'Fktn'	777	754	433	513	757	533	654.6666667	601	-0.031165045	0.9307253	0.99348433
246190	'Otoa'	6	1	1	0	6	7	2.666666667	4.333333333	0.621189968	0.706843183	0.972790461
246196	'Zfp277'	775	780.84	826.98	586.06	892.08	1052.34	794.2733333	843.4933333	0.093471146	0.74852682	0.972790461
246198	'Mllt6'	2552	2451	1649	1443	2577	2203	2217.333333	2074.333333	-0.052180147	0.847788946	0.975439569
246221	'Mpst'	478	442	515	1008	492	643	478.3333333	714.3333333	0.741261421	0.175528403	0.794684462
246228	'Vwa1'	52	70	72	68	48	63	64.66666667	59.66666667	-0.022375751	0.965285154	0.999493374
246229	'Bivm'	703	693	726	216	671	681	707.3333333	522.6666667	-0.495274432	0.234659746	0.861206181
246256	'Fcgr4'	3	9	1	3	7	24	4.333333333	11.33333333	1.36612489	0.249639051	0.869928149
246257	'Ovca2'	469.41	486.83	319.89	246.03	593.91	440.55	425.3766667	426.83	0.023177343	0.945719181	0.996592403
246277	'Csad'	944.5	982.11	323.07	302.42	1131.7	766.61	749.8933333	733.5666667	-0.01642956	0.975739069	0.999493374
246293	'Klhl8'	758	689	543	174	775	843	663.3333333	597.3333333	-0.218815713	0.666543431	0.972790461
246313	'Prokr2'	39	44	100	164	57	102	61	107.6666667	0.941695679	0.214511277	0.842621568
246316	'Lgi2'	80	65	157	1162	117	261	100.6666667	513.3333333	2.631468352	0.006859797	0.196117976
246317	'Neto1'	94.45	116.14	50.67	35.16	85.32	40.22	87.08666667	53.56666667	-0.638719707	0.221643839	0.849403976
246691	'Prok1'	8	8	2.24	2	5	4	6.08	3.666666667	-0.652569049	0.551198355	0.972790461
246694	'Hps5'	1112	1166	756	756	949	724	1011.333333	809.6666667	-0.220923709	0.516916116	0.972790461
246696	'Slc25a28'	494	450	585	524	515	556	509.6666667	531.6666667	0.122675085	0.7553599	0.972790461
246700	'Defb19'	12	18	7	2	43	36	12.33333333	27	1.048702153	0.28250503	0.890642259
246703	'Naxe'	1017	1034	576	452	997	1391	875.6666667	946.6666667	0.107032288	0.78618824	0.972790461
246707	'Emilin2'	247	259	88	331	194	195	198	240	0.507450414	0.397879358	0.954563638
246709	'Rgs13'	1	1	0	0	3	0	0.666666667	1	0.583740724	0.857105885	0.976149994
246710	'Rhobtb2'	391	420	290	860.6	401	463	367	574.8666667	0.861927136	0.127105445	0.722729007
246727	'Oas3'	6.87	8.69	7.48	6.79	1	7	7.68	4.93	-0.477755095	0.66100033	0.972790461
246728	'Oas2'	64	90	125	133	70	226	93	143	0.638822097	0.300074402	0.903836268
246729	'Oas1h'	0	0	0	0	2	61	0	21	6.645923194	0.008999664	0.231771405
246730	'Oas1a'	46.58	55.63	68.51	13.77	23.21	114.59	56.90666667	50.52333333	-0.304709239	0.70090187	0.972790461
246735	'AY074887'	4	6	3	2	1	1	4.333333333	1.333333333	-1.556862302	0.258096385	0.877405867
246738	'Dnajc28'	312	344	86	280	441	359	247.3333333	360	0.651983354	0.214919686	0.842760284

246746	'Cd300lf'	3	2	3.35	1	5	15	2.783333333	7	1.270762387	0.31780815	0.916136994
246747	'Adig'	0	0	1	0	0	0	0.333333333	0	-0.903279821	0.824807108	0.972790461
246779	'Il27'	0	1	0	5	0	0	0.333333333	1.666666667	2.683819621	0.465346482	0.972790461
246782	'Atpaf2'	396	359	304	209	422.02	377	353	336.0066667	-0.057908475	0.84180812	0.975182082
246787	'Slc5a2'	2.35	1.13	0	1.1	4.4	4.52	1.16	3.34	1.602492218	0.360770436	0.933706718
246788	'Trpv3'	5	6	3	19	15	7	4.666666667	13.66666667	1.739343947	0.06238427	0.566066012
246791	'Obox3'	3	0	0	0	2	1	1	1	-0.023018389	0.99402566	0.999562152
246792	'Obox2'	1	0	0	0	0	0	0.333333333	0	-0.903279821	0.824807108	0.972790461
252829	'Obox5'	0	0	0	0	2	0	0	0.666666667	1.801491674	0.656112935	0.972790461
252830	'Obox6'	0	3	0	1	0	0	1	0.333333333	-1.313730691	0.742696629	0.972790461
252837	'Ackr4'	135	148	8	0	86	39	97	41.66666667	-1.256037011	0.358449157	0.933467324
252838	'Tox'	16	7	20	5	26	24	14.33333333	18.33333333	0.241621531	0.772141279	0.972790461
252864	'Dusp15'	17	21	19	77	12	19	19	36	1.233362683	0.193768688	0.817561096
252870	'Usp7'	3500	3601	3095	2922	3924	4824	3398.666667	3890	0.228163148	0.349310432	0.928823768
252875	'Mios'	1239	1268	761	344	894	1140	1089.333333	792.6666667	-0.478211282	0.23581856	0.861636582
252876	'Gin1'	3189.5	3200.4	577.5	294.9	6010.8	12850.1	2322.443333	6385.253333	1.382106953	0.165423446	0.78006879
252903	'Ap1s3'	75.01	91.49	34.53	75.2	43.03	59.73	67.01	59.32	0.011922429	0.983857623	0.999493374
252966	'Cables2'	811	938	481	451	806	792	743.3333333	683	-0.076473722	0.809777624	0.972790461
252967	'Ropn1l'	70	66	30	1	54	108	55.33333333	54.33333333	-0.140533493	0.89451694	0.985259027
252972	'Tpcn1'	742	750	257	657	904	889	583	816.6666667	0.589803855	0.182340062	0.80302276
252973	'Grhl2'	121	102	32	23	55	123	85	67	-0.353650083	0.601325952	0.972790461
252974	'Tspear'	15	14	6	8	8	5	11.66666667	7	-0.596447535	0.475919504	0.972790461
257630	'Il17f'	12	10	1	1	7	3	7.666666667	3.666666667	-1.024501418	0.419935828	0.958308933
257632	'Nod2'	9	6	15	10	10	10	10	10	0.027005173	0.973979375	0.999493374
257633	'Acsf3'	408	410	177	120	342	370	331.6666667	277.3333333	-0.255450234	0.582220346	0.972790461
257635	'Sdsl'	189	227	203	137	176	235	206.3333333	182.6666667	-0.151076806	0.640317343	0.972790461
257875	'Olfr1116'	0	0	0	0	0	1	0	0.333333333	1.020273531	0.802557913	0.972790461
257882	'Olfr1344'	2	3	0	0	1	2	1.666666667	1	-0.75018494	0.743459437	0.972790461
257883	'Olfr1357'	1	0	0	0	0	0	0.333333333	0	-0.903279821	0.824807108	0.972790461
257891	'Olfr479'	0	0	0	0	0	1	0	0.333333333	1.020273531	0.802557913	0.972790461
257900	'Olfr1024'	2	0	0	0	0	0	0.666666667	0	-1.711373851	0.67234292	0.972790461
257905	'Olfr298'	1	0	0	0	8	0	0.333333333	2.666666667	2.787166248	0.438478281	0.966976432
257919	'Olfr467'	3	3	0	0	2	0	2	0.666666667	-1.529866446	0.584024522	0.972790461
257926	'Olfr544'	0	0	1	2	2	0	0.333333333	1.333333333	2.089747025	0.486504263	0.972790461
257938	'Olfr1419'	1	0	0	0	1	0	0.333333333	0.333333333	0.058500858	0.988561293	0.999493374
257947	'Olfr543'	1	1	0	6	4	0	0.666666667	3.333333333	2.640875247	0.222352356	0.849721203
257951	'Olfr987'	0	1	0	0	0	0	0.333333333	0	-0.903279821	0.824807108	0.972790461
257961	'Olfr1432'	0	0	0	1	0	0	0	0.333333333	1.020273531	0.802557913	0.972790461
258019	'Olfr212'	1	0	0	2	0	0	0.333333333	0.666666667	1.337350669	0.73871782	0.972790461
258064	'Olfr316'	14	12	0	0	31	28	8.666666667	19.66666667	1.138258892	0.493555747	0.972790461

258098	'Olfr1490'	0	1	0	0	0	0	0.333333333	0	-0.903279821	0.824807108	0.972790461
258135	'Olfr597'	0	0	1	1	0	0	0.333333333	0.333333333	0.058500858	0.988561293	0.999493374
258154	'Olfr1029'	0	0	0	6	0	0	0	2	3.915308806	0.320433418	0.917329173
258155	'Olfr1425'	1	0	0	0	0	0	0.333333333	0	-0.903279821	0.824807108	0.972790461
258160	'Olfr685'	0	0	0	0	0	1	0	0.333333333	1.020273531	0.802557913	0.972790461
258168	'Olfr566'	0	1	0	0	0	0	0.333333333	0	-0.903279821	0.824807108	0.972790461
258178	'Olfr180'	0	0	0	1	0	0	0	0.333333333	1.020273531	0.802557913	0.972790461
258181	'Olfr406'	0	0	0	0	1	0	0	0.333333333	1.020273531	0.802557913	0.972790461
258198	'Olfr224'	0	0	0	3	0	0	0	1	2.899270347	0.465759809	0.972790461
258207	'Olfr452'	0	1	0	0	0	0	0.333333333	0	-0.903279821	0.824807108	0.972790461
258216	'Olfr1034'	0	0	0	19	12	1	0	10.66666667	6.11817815	0.002837111	0.119599022
258222	'Olfr310'	0	0	0	0	1	0	0	0.333333333	1.020273531	0.802557913	0.972790461
258246	'Olfr594'	0	0	0	0	1	0	0	0.333333333	1.020273531	0.802557913	0.972790461
258267	'Olfr370'	0	1	1	0	0	0	0.666666667	0	-1.851176388	0.646290011	0.972790461
258280	'Olfr1366'	0	0	0	0	1	0	0	0.333333333	1.020273531	0.802557913	0.972790461
258309	'Olfr657'	0	0	0	0	0	1	0	0.333333333	1.020273531	0.802557913	0.972790461
258318	'Olfr186'	0	0	0	2	0	0	0	0.666666667	2.299096387	0.566358544	0.972790461
258319	'Olfr187'	0	0	0	1	0	0	0	0.333333333	1.020273531	0.802557913	0.972790461
258322	'Olfr554'	4	4	0	0	0	0	2.666666667	0	-3.693860587	0.278291699	0.889355605
258330	'Olfr1274'	0	0	0	0	1	0	0	0.333333333	1.020273531	0.802557913	0.972790461
258335	'Olfr374'	1	0	0	0	0	0	0.333333333	0	-0.903279821	0.824807108	0.972790461
258339	'Olfr1269'	0	1.02	0	0	2	1	0.34	1	1.368205316	0.684632717	0.972790461
258340	'Olfr1265'	0	0.98	0	0	0	1	0.326666667	0.333333333	1.020273531	0.802557913	0.972790461
258342	'Olfr1491'	0	0	1	0	1	0	0.333333333	0.333333333	0.058500858	0.988561293	0.999493374
258352	'Olfr692'	1	2	0	0	1	0	1	0.333333333	-1.324111362	0.694133627	0.972790461
258355	'Olfr677'	1	1	0	0	0	0	0.666666667	0	-1.703500596	0.673820112	0.972790461
258356	'Olfr564'	0	0	0	0	0	1	0	0.333333333	1.020273531	0.802557913	0.972790461
258358	'Olfr557'	0	1	0	0	1	0	0.333333333	0.333333333	0.058500858	0.988561293	0.999493374
258364	'Olfr976'	5	6	0	1	10	35	3.666666667	15.33333333	2.008221311	0.166964154	0.781229856
258373	'Olfr323'	0	1	0	0	0	1	0.333333333	0.333333333	0.058500858	0.988561293	0.999493374
258374	'Olfr127'	0	0	0	0	2	0	0	0.666666667	1.801491674	0.656112935	0.972790461
258380	'Olfr461'	1	5	2	2	4	4	2.666666667	3.333333333	0.348351036	0.790501482	0.972790461
258381	'Olfr460'	4	1	0	0	6	3	1.666666667	3	0.816143291	0.693639193	0.972790461
258392	'Olfr190'	0	0	0	1	0	0	0	0.333333333	1.020273531	0.802557913	0.972790461
258404	'Olfr1080'	1	0	0	0	0	0	0.333333333	0	-0.903279821	0.824807108	0.972790461
258405	'Olfr1420'	16	18	5	1	17	2	13	6.666666667	-0.969361727	0.413629098	0.95722888
258407	'Olfr464'	0	0	0	0	0	1	0	0.333333333	1.020273531	0.802557913	0.972790461
258408	'Olfr463'	0	0	0	0	0	2	0	0.666666667	1.749449488	0.66566965	0.972790461
258416	'Olfr888'	1	0	0	0	0	0	0.333333333	0	-0.903279821	0.824807108	0.972790461
258417	'Olfr470'	0	0	0	0	1	0	0	0.333333333	1.020273531	0.802557913	0.972790461

258420	'Olfr221'	4	1	0	0	2	0	1.666666667	0.666666667	-1.274695528	0.667822984	0.972790461
258426	'Olfr995'	0	0	0	0	0	1	0	0.333333333	1.020273531	0.802557913	0.972790461
258428	'Olfr998'	0	0	1	0	0	0	0.333333333	0	-0.903279821	0.824807108	0.972790461
258433	'Olfr933'	0	0	0	1	0	0	0	0.333333333	1.020273531	0.802557913	0.972790461
258434	'Olfr934'	0	0	0	2	0	0	0	0.666666667	2.299096387	0.566358544	0.972790461
258439	'Olfr1309'	0	0	0	0	0	1	0	0.333333333	1.020273531	0.802557913	0.972790461
258440	'Olfr1317'	1	0	0	0	0	0	0.333333333	0	-0.903279821	0.824807108	0.972790461
258449	'Olfr282'	1	0	0	0	1	1	0.333333333	0.666666667	0.817432597	0.823901819	0.972790461
258456	'Olfr1196'	0	0	0	0	1	0	0	0.333333333	1.020273531	0.802557913	0.972790461
258462	'Olfr1392'	0	2	0	2	0	0	0.666666667	0.666666667	0.545035566	0.890974668	0.985046501
258463	'Olfr1393'	2	3	2	9	3	9.01	2.333333333	7.003333333	1.718539493	0.138893944	0.740857359
258464	'Olfr1384'	1.98	0.75	0	0	0	0	0.91	0	-0.903279821	0.824807108	0.972790461
258469	'Olfr90'	2	0	1.24	3	0	0	1.08	1	0.421591567	0.887744239	0.984534762
258470	'Olfr91'	2	3	2.76	1	0	0	2.586666667	0.333333333	-2.635861275	0.227870907	0.854324922
258471	'Olfr891'	1	0	0	0	0	0	0.333333333	0	-0.903279821	0.824807108	0.972790461
258498	'Olfr148'	0	1	0	0	0	0	0.333333333	0	-0.903279821	0.824807108	0.972790461
258504	'Olfr107'	0	0	0	0	0	1	0	0.333333333	1.020273531	0.802557913	0.972790461
258508	'Olfr99'	0	0	1	0	0	0	0.333333333	0	-0.903279821	0.824807108	0.972790461
258509	'Olfr1375'	0	0	0	0	1	0	0	0.333333333	1.020273531	0.802557913	0.972790461
258531	'Olfr315'	0	2	0	0	2	1	0.666666667	1	0.569767569	0.858446798	0.976475731
258534	'Olfr1361'	0	0	0	0	0	1	0	0.333333333	1.020273531	0.802557913	0.972790461
258545	'Olfr811'	0	0	0	1	0	0	0	0.333333333	1.020273531	0.802557913	0.972790461
258553	'Olfr872'	0	1	0	1	1	0	0.333333333	0.666666667	1.081692101	0.760081965	0.972790461
258554	'Olfr873'	1	1	0	3	2	0	0.666666667	1.666666667	1.631983848	0.492174815	0.972790461
258571	'Olfr1033'	151	163	1389	4558	316	264	567.6666667	1712.666667	1.856252791	0.155323758	0.766453805
258573	'Olfr1020'	2	1	0	0	0	0	1	0	-2.286544758	0.567674219	0.972790461
258579	'Olfr1018'	3	0	0	0	0	0	1	0	-2.292035055	0.566710385	0.972790461
258581	'Olfr1030'	0	0	0	3	0	0	0	1	2.899270347	0.465759809	0.972790461
258583	'Olfr1085'	0	2	0	0	0	0	0.666666667	0	-1.695595436	0.675304306	0.972790461
258597	'Olfr716'	1	0	0	0	0	0	0.333333333	0	-0.903279821	0.824807108	0.972790461
258605	'Olfr968'	1	1	0	0	0	0	0.666666667	0	-1.703500596	0.673820112	0.972790461
258608	'Olfr986'	0	1	0	0	0	1	0.333333333	0.333333333	0.058500858	0.988561293	0.999493374
258624	'Olfr120'	0	0	0	0	0	1	0	0.333333333	1.020273531	0.802557913	0.972790461
258627	'Olfr1504'	0	1	0	0	0	1	0.333333333	0.333333333	0.058500858	0.988561293	0.999493374
258652	'Olfr1131'	1	0	0	0	0	0	0.333333333	0	-0.903279821	0.824807108	0.972790461
258665	'Olfr815'	0	1	0	0	0	0	0.333333333	0	-0.903279821	0.824807108	0.972790461
258669	'Olfr824'	3	0	0	2	0	1	1	1	0.341669693	0.909858876	0.989364653
258693	'Olfr1443'	0	0	0	0	1	0	0	0.333333333	1.020273531	0.802557913	0.972790461
258694	'Olfr1445'	0	1	0	0	0	0	0.333333333	0	-0.903279821	0.824807108	0.972790461
258696	'Olfr1448'	0	2	0	0	0	0	0.666666667	0	-1.695595436	0.675304306	0.972790461

258697	'Olfr1444'	1	0	0	0	0	0	0.333333333	0	-0.903279821	0.824807108	0.972790461
258699	'Olfr1446'	0	2	0	0	1	0	0.666666667	0.333333333	-0.733814424	0.856146772	0.975734242
258729	'Olfr478'	2	0	0	0	0	0	0.666666667	0	-1.711373851	0.67234292	0.972790461
258734	'Olfr502'	1	1	0	0	0	0	0.666666667	0	-1.703500596	0.673820112	0.972790461
258745	'Olfr689'	0	0	0	0	0	1	0	0.333333333	1.020273531	0.802557913	0.972790461
258749	'Olfr556'	0	2	0	0	0	0	0.666666667	0	-1.695595436	0.675304306	0.972790461
258753	'Olfr678'	4	6	0	0	0	1	3.333333333	0.333333333	-3.055335295	0.240558797	0.86449675
258767	'Olfr1176'	0	1	0	0	0	0	0.333333333	0	-0.903279821	0.824807108	0.972790461
258773	'Olfr818'	1	0	0	0	2	1	0.333333333	1	1.368261242	0.684350922	0.972790461
258778	'Olfr921'	0	0	0	5	1	0	0	2	3.828059121	0.277975466	0.889355605
258783	'Olfr920'	15	11	43	73	13	7	23	31	0.694053751	0.545973116	0.972790461
258787	'Olfr1248'	0	1	0	3	0	0	0.333333333	1	1.937497013	0.625951132	0.972790461
258793	'Olfr1502'	0	0	0	0	1	0	0	0.333333333	1.020273531	0.802557913	0.972790461
258802	'Olfr143'	2	1	0	0	1	0	1	0.333333333	-1.329381507	0.692813154	0.972790461
258810	'Olfr665'	0	1	0	0	0	0	0.333333333	0	-0.903279821	0.824807108	0.972790461
258812	'Olfr923'	0	0	0	0	1	0	0	0.333333333	1.020273531	0.802557913	0.972790461
258819	'Olfr640'	0	0	0	0	1	0	0	0.333333333	1.020273531	0.802557913	0.972790461
258822	'Olfr39'	0	1	0	1	0	0	0.333333333	0.333333333	0.058500858	0.988561293	0.999493374
258825	'Olfr975'	0	0	0	0	1	0	0	0.333333333	1.020273531	0.802557913	0.972790461
258833	'Olfr1132'	0	2	0	0	0	0	0.666666667	0	-1.695595436	0.675304306	0.972790461
258837	'Olfr545'	1	0	0	2	3	1	0.333333333	2	2.571442575	0.267540412	0.883600995
258854	'Olfr985'	5	2	0	0	0	0	2.333333333	0	-3.505057958	0.3270664	0.920734892
258858	'Olfr371'	0	1	0	0	0	0	0.333333333	0	-0.903279821	0.824807108	0.972790461
258873	'Olfr911-ps1'	0	0	0	0	2	0	0	0.666666667	1.801491674	0.656112935	0.972790461
258875	'Olfr895'	0	0	0	0	1	0	0	0.333333333	1.020273531	0.802557913	0.972790461
258879	'Olfr330'	0	5	0	1	4	0	1.666666667	1.666666667	0.133529833	0.961917388	0.999493374
258882	'Olfr874'	1	0	0	0	0	0	0.333333333	0	-0.903279821	0.824807108	0.972790461
258896	'Olfr1206'	1	0	0	0	0	0	0.333333333	0	-0.903279821	0.824807108	0.972790461
258899	'Olfr1214'	0	0	0	1	0	0	0	0.333333333	1.020273531	0.802557913	0.972790461
258900	'Olfr1213'	0	0	0	1	0	0	0	0.333333333	1.020273531	0.802557913	0.972790461
258902	'Olfr1220'	0	0	0	0	0	1	0	0.333333333	1.020273531	0.802557913	0.972790461
258915	'Olfr1348'	0	2	0	0	0	0	0.666666667	0	-1.695595436	0.675304306	0.972790461
258922	'Olfr267'	0	0	0	0	1	0	0	0.333333333	1.020273531	0.802557913	0.972790461
258924	'Olfr376'	0	1	0	0	0	0	0.333333333	0	-0.903279821	0.824807108	0.972790461
258925	'Olfr20'	13	8	2	0	4	0	7.666666667	1.333333333	-2.511961904	0.148058409	0.756249141
258938	'Olfr1417'	1	1	1	5	1	0	1	2	1.342004911	0.509812181	0.972790461
258949	'Olfr338'	0	1	0	0	0	0	0.333333333	0	-0.903279821	0.824807108	0.972790461
258963	'Olfr539'	1	0	0	0	0	0	0.333333333	0	-0.903279821	0.824807108	0.972790461
258967	'Olfr1250'	5	7	4	16	0	0	5.333333333	5.333333333	0.485163663	0.801643898	0.972790461
258984	'Olfr1257'	1	0	0	0	0	0	0.333333333	0	-0.903279821	0.824807108	0.972790461

258987	'Olfr1270'	0	0	0	0	1	0	0	0.333333333	1.020273531	0.802557913	0.972790461
258988	'Olfr38'	0	1	0	0	1	0	0.333333333	0.333333333	0.058500858	0.988561293	0.999493374
258991	'Olfr1496'	2	0	0	0	2	0	0.666666667	0.666666667	0.031622541	0.993710128	0.999493374
258992	'Olfr1494'	2	0	0	0	1	0	0.666666667	0.333333333	-0.74959284	0.853034393	0.975734242
258993	'Olfr206'	0	0	0	0	1	10	0	3.666666667	4.14034111	0.212941334	0.841028891
258999	'Olfr178'	1	0	0	0	0	0	0.333333333	0	-0.903279821	0.824807108	0.972790461
259038	'Olfr283'	0	1	0	0	0	0	0.333333333	0	-0.903279821	0.824807108	0.972790461
259041	'Olfr1414'	0	2	0	0	0	0	0.666666667	0	-1.695595436	0.675304306	0.972790461
259044	'Olfr1353'	1	0	0	1	0	0	0.333333333	0.333333333	0.058500858	0.988561293	0.999493374
259047	'Olfr683'	1	1	0	0	1.49	0	0.666666667	0.496666667	-0.74517548	0.838722495	0.974723675
259049	'Olfr618'	0	0	0	0	1	0	0	0.333333333	1.020273531	0.802557913	0.972790461
259051	'Olfr658'	1	0	0	0	2	0	0.333333333	0.666666667	0.839717162	0.835573887	0.974723675
259063	'Olfr691'	1	0	0	0	0	1	0.333333333	0.333333333	0.058500858	0.988561293	0.999493374
259066	'Olfr520'	0	1	0	0	0	0	0.333333333	0	-0.903279821	0.824807108	0.972790461
259071	'Olfr166'	0	1	0	0	0	0	0.333333333	0	-0.903279821	0.824807108	0.972790461
259085	'Olfr610'	0	0	0	0	1	0	0	0.333333333	1.020273531	0.802557913	0.972790461
259087	'Olfr622'	0	0	0	0	1	0	0	0.333333333	1.020273531	0.802557913	0.972790461
259088	'Olfr639'	0	1	0	0	1	0	0.333333333	0.333333333	0.058500858	0.988561293	0.999493374
259097	'Olfr558'	0	3	0	0	2	2	1	1.333333333	0.389931636	0.893753461	0.985171
259103	'Olfr616'	0	0	0	0	1	0	0	0.333333333	1.020273531	0.802557913	0.972790461
259104	'Olfr613'	35.39	47.08	12.43	5.16	33.45	16.78	31.63333333	18.46333333	-0.794850845	0.320528354	0.917339314
259115	'Olfr586'	0	0	0	0	1	0	0	0.333333333	1.020273531	0.802557913	0.972790461
259116	'Olfr559'	1	0	0	0	0	0	0.333333333	0	-0.903279821	0.824807108	0.972790461
259122	'Olfr635'	1	0.5	0	0	0	0	0.5	0	-0.903279821	0.824807108	0.972790461
259144	'Olfr456'	0	0	0	1	0	0	0	0.333333333	1.020273531	0.802557913	0.972790461
259145	'Olfr1251'	6	15	13	30	2	0	11.33333333	10.66666667	0.331161875	0.833302754	0.974723675
259148	'Olfr329'	0	0	1	0	0	0	0.333333333	0	-0.903279821	0.824807108	0.972790461
259161	'Olfr688'	0	1	0	0	0	0	0.333333333	0	-0.903279821	0.824807108	0.972790461
259163	'Olfr1354'	1	0	0	0	0	0	0.333333333	0	-0.903279821	0.824807108	0.972790461
259172	'Mfrp'	3.15	1.91	1.3	3.23	13.51	64.25	2.12	26.99666667	3.882403096	0.002677192	0.115942558
259277	'Klk8'	84	89	77	43	56	56	83.33333333	51.66666667	-0.640469086	0.081695602	0.621278714
259279	'Tubgcp3'	1154	1155	603	499	1041	868	970.6666667	802.6666667	-0.232884186	0.501249161	0.972790461
259300	'Ehd2'	1640	1651	1233	761	2089	1986	1508	1612	0.076219969	0.829338328	0.973678583
259302	'Srgap3'	2307	2314	1073	2009	4024	2804	1898	2945.666667	0.698704547	0.070152772	0.589465353
260296	'Trim61'	1	5	0	4	7	37	2	16	2.976356516	0.034903107	0.447758353
260297	'Prrt1'	12	12	1	10	11	8	8.333333333	9.666666667	0.386080462	0.702711939	0.972790461
260298	'Fev'	0	0	2	0	0	1	0.666666667	0.333333333	-1.033504517	0.79720072	0.972790461
260299	'Cadm4'	503	486	420	5018	671	1614	469.6666667	2434.333333	2.690040334	0.001126862	0.070791417
260301	'Otos'	1	0	0	0	0	0	0.333333333	0	-0.903279821	0.824807108	0.972790461
260302	'Gga3'	903	835	499	609	715	676	745.6666667	666.6666667	-0.06016968	0.858664669	0.976637967

260305	'Nphp4'	97	86	32	117	107	79	71.66666667	101	0.674151049	0.235683931	0.861636582
260315	'Nav3'	69.95	53.97	90.66	29.85	89.17	40.72	71.52666667	53.24666667	-0.461868315	0.426345938	0.960958977
260408	'Prss45'	0	4	1	0	4	4	1.666666667	2.666666667	0.590919887	0.776556739	0.972790461
260409	'Cdc42ep3'	160	181	798	248	168	274	379.6666667	230	-0.748493523	0.34515944	0.926100052
260423	'H3c7'	1	1	8.15	0	5	1	3.383333333	2	-0.968312917	0.603422823	0.972790461
263406	'Plekhg3'	450	441	558	634	355	422	483	470.3333333	0.102184623	0.847776	0.975439569
26356	'Ing1'	887	897	1823	858	907	1042	1202.333333	935.6666667	-0.357203669	0.486585848	0.972790461
26357	'Abcg2'	497	545	428	584	722	574	490	626.6666667	0.439254682	0.182645458	0.803809129
26358	'Aldh1a7'	173	170	29	250	437	747	124	478	1.998618553	0.002163482	0.10389089
26359	'Anxa10'	0	1	50	0	0	1	17	0.333333333	-5.723238001	0.018255707	0.33668007
26360	'Angptl2'	948	975	293	4715	1103	1008	738.6666667	2275.333333	2.004540944	0.017851882	0.333726637
26361	'Avpr1b'	0	0	0	1	0	0	0	0.333333333	1.020273531	0.802557913	0.972790461
26362	'Axl'	7313	7384	6259	12002	11049	12371	6985.333333	11807.33333	0.855416352	0.011585867	0.267046959
26363	'Btd'	388	372	187	207	527	344	315.6666667	359.3333333	0.22375362	0.59049274	0.972790461
26364	'Adgre5'	659	827	401	1054	956	1214	629	1074.666667	0.891529964	0.020726603	0.35725674
26365	'Ceacam1'	9.78	16.67	4	7	38	24.95	10.15	23.31666667	1.244527379	0.136267638	0.736689633
26366	'Ceacam10'	0	0	1	0	1	1	0.333333333	0.666666667	0.818407084	0.819542986	0.972790461
26367	'Ceacam2'	5.22	10.33	0	3	0	1.05	5.183333333	1.35	-1.524667919	0.448960613	0.97049895
26369	'Cetn1'	1	6	2	0	1	10	3	3.666666667	0.167640762	0.924401515	0.992137383
26370	'Cetn2'	760	816	253	311	645	829	609.6666667	595	8.23E-04	0.998665088	0.999900097
26371	'Ciao1'	623	632	908	788	757	1109	721	884.6666667	0.319669633	0.448998976	0.97049895
26372	'Clcn6'	825	840	822	488	638	566	829	564	-0.501548256	0.100375786	0.671759705
26373	'Clcn7'	785	815	375	849	1051	992	658.3333333	964	0.656578596	0.07817557	0.614954936
26374	'Cop1'	2227	2162	2536	2976	2222	2781	2308.333333	2659.666667	0.301065608	0.48004316	0.972790461
263764	'Creg2'	54	36	23	22	59	49	37.66666667	43.33333333	0.21889301	0.677817433	0.972790461
26377	'Dapp1'	142	166	281	171	246	283	196.3333333	233.3333333	0.221892652	0.6430212	0.972790461
26378	'Decr2'	433	443	501	188	643	519	459	450	-0.089887581	0.838632658	0.974723675
26379	'Esrra'	277	305	454	462	249	365	345.3333333	358.6666667	0.162673673	0.771555883	0.972790461
26380	'Esrrb'	10	14	15	80	13	42	13	45	2.015279757	0.023722322	0.380598139
263803	'Pkn3'	84	104	35	105	157	114	74.33333333	125.3333333	0.860978844	0.075532263	0.605310548
26381	'Esrrg'	11	11	20	3	12	13	14	9.333333333	-0.679324273	0.413755879	0.95722888
26382	'Fgd2'	11	11	1	3	9	13	7.666666667	8.333333333	0.149415429	0.889591064	0.984980969
26383	'Fto'	1715	1808	2005	1562	2035	2223	1842.666667	1940	0.101566368	0.743823386	0.972790461
26384	'Gnpda1'	1252	1322	1182	860	1410	1632	1252	1300.666667	0.064148887	0.803970036	0.972790461
26385	'Grk6'	1141	1066	644	587	1133	957	950.3333333	892.3333333	-0.048053507	0.877459385	0.981341203
26386	'Hsf4'	2	2	3	1	1	1	2.333333333	1	-1.18958642	0.464174007	0.972790461
263876	'Spata2'	725	773	567	732	613	896	688.3333333	747	0.212509996	0.548556765	0.972790461
26388	'Ifi202b'	11	6	357	0	0	0	124.6666667	0	-9.555129201	7.83E-04	0.05683522
26390	'Mapkbp1'	1439	1389	400	537	1776	886	1076	1066.333333	0.036343098	0.949594908	0.997836337
26394	'Lypla2'	686	744	868	566	725	942	766	744.3333333	-0.031628781	0.926197423	0.992381554

26395	'Map2k1'	1524	1439	5768	1638	1201	2090	2910.333333	1643	-0.857018207	0.237776518	0.863437626
26396	'Map2k2'	1236	1147	1654	647	1235	1397	1345.666667	1093	-0.340352275	0.380848189	0.944149925
26397	'Map2k3'	786	880	1388	2860	856	1134	1018	1616.666667	0.873809017	0.227116974	0.854001521
26398	'Map2k4'	1711	1776	2081	1868	1861	2126	1856	1951.666667	0.130793421	0.722143424	0.972790461
26399	'Map2k6'	385	468	308	151	524	429	387	368	-0.101278497	0.811863894	0.972790461
26400	'Map2k7'	1638	1611	1311	768	1815	1250	1520	1277.666667	-0.239854694	0.46274057	0.972790461
26401	'Map3k1'	2322	1986	7544	3591	1930	3240	3950.666667	2920.333333	-0.40709791	0.578069533	0.972790461
26403	'Map3k11'	1428	1370.1	891	735.91	1507.7	2398.35	1229.696667	1547.326667	0.314757538	0.424807043	0.960285372
26404	'Map3k12'	921.87	923.43	595.05	1063.3	1080.7	782.7	813.45	975.5633333	0.399785283	0.331125037	0.921648675
26405	'Map3k2'	905	928	1664	2272	1086	1334	1165.666667	1564	0.557368004	0.373100148	0.939271718
26406	'Map3k3'	1908.7	1815.8	2049	1737	2068.8	1816.69	1924.513333	1874.183333	0.022217572	0.948638802	0.997521806
264064	'Cdk8'	2256	2233	1799	548	1707	1692	2096	1315.666667	-0.711028962	0.070390077	0.589465353
26407	'Map3k4'	1627	1574	1227	1381	1421	1654	1476	1485.333333	0.093707854	0.755683546	0.972790461
26408	'Map3k5'	277	249	268	288	469	617	264.6666667	458	0.783753351	0.014639296	0.300888969
26409	'Map3k7'	1839	1787	1277	1491	2236	1832	1634.333333	1853	0.246818491	0.373789517	0.939638542
26410	'Map3k8'	78	84	260	147	72	66	140.6666667	95	-0.459232205	0.564463143	0.972790461
26411	'Map4k1'	506	454	106	51	388	317	355.3333333	252	-0.519524013	0.48314093	0.972790461
26412	'Map4k2'	419	519	218	288	760	592	385.3333333	546.6666667	0.53106584	0.208385704	0.836358246
26413	'Mapk1'	4451	4450	5152	3955	4557	5696	4684.333333	4736	0.047040836	0.88581305	0.984282742
264134	'Ttc26'	433	452	119	124	295	219	334.6666667	212.6666667	-0.58862486	0.275289291	0.886633914
26414	'Mapk10'	43	56	19	0	69	115	39.33333333	61.33333333	0.53074935	0.66981022	0.972790461
26415	'Mapk13'	316	286	563	849	396	784	388.3333333	676.3333333	0.8840068	0.146532457	0.753611197
26416	'Mapk14'	2309	2159	1084	1380	1923	2459	1850.666667	1920.666667	0.116318654	0.737131833	0.972790461
26417	'Mapk3'	1700	1724	4132	1838	1825	2561	2518.666667	2074.666667	-0.298452121	0.597290912	0.972790461
26419	'Mapk8'	1228	1131	1183	727	1120	1227	1180.666667	1024.666667	-0.190123277	0.484041593	0.972790461
26420	'Mapk9'	2484	2507	2155	2203	2479	2596	2382	2426	0.099043642	0.730471692	0.972790461
26422	'Nbea'	2135.9	1988.4	979.43	1284.9	1876.6	1720.42	1701.226667	1627.32	0.020676777	0.953263828	0.999032683
26423	'Nr5a1'	2857	3043	1933	2183	3092	3047	2611	2774	0.151803779	0.55907048	0.972790461
26424	'Nr5a2'	204	238	324	1	395	285	255.3333333	227	-0.35665275	0.763223284	0.972790461
26425	'Nubp1'	430	426	173	189	441	482	343	370.6666667	0.136198261	0.757524339	0.972790461
26426	'Nubp2'	360	349	318	304	373	478	342.3333333	385	0.208945107	0.460760682	0.972790461
26427	'Creb3l1'	487	462	1997	849	570	1072	982	830.3333333	-0.274454588	0.716885953	0.972790461
26428	'Orc4'	1145.4	1223.8	932.53	749.32	1112	1072.81	1100.6	978.03	-0.123378036	0.606624687	0.972790461
26429	'Orc5'	842	911	566	300	703	916	773	639.6666667	-0.287765339	0.44541624	0.969906489
26430	'Parg'	2218	2145	1021	1008	1804	2247	1794.666667	1686.333333	-0.053781755	0.884642757	0.98386821
26431	'Git2'	1045	1109	998	1041	1157	1085	1050.666667	1094.333333	0.137218289	0.670189751	0.972790461
26432	'Plod2'	1994	1579	3293	9812	1432	1849	2288.666667	4364.333333	1.224036269	0.18189425	0.802193703
26433	'Plod3'	1033.5	1021.1	1710.1	2509.3	1252.6	1405.01	1254.863333	1722.296667	0.59883097	0.325971293	0.920269559
26434	'Prnd'	504	483	42	2	911	508	343	473.6666667	0.4173603	0.750786889	0.972790461
26436	'Psg16'	721.86	755.12	364.85	478.52	776.61	473.41	613.9433333	576.18	0.005549567	0.989129786	0.999493374

26437	'Psg17'	0	2	0	1	1	10	0.666666667	4	2.558228337	0.245131442	0.866091613
26439	'Psg19'	0	0	1	0	3	0	0.333333333	1	1.381884369	0.730152291	0.972790461
26440	'Psma1'	1467	1582	1810	1383	1326	1696	1619.666667	1468.333333	-0.087545984	0.812699886	0.972790461
26441	'Psma4'	1435	1545	1631	1013	1559	2004	1537	1525.333333	-0.016083804	0.958586866	0.999493374
26442	'Psma5'	2175	2292	3026	2613	1958	2473	2497.666667	2348	-0.006582703	0.988444137	0.999493374
26443	'Psma6'	2034	2253	2703	1397	1797	2265	2330	1819.666667	-0.348067974	0.315436667	0.914723408
26444	'Psma7'	1365	1499	2690	1517	1651	2252	1851.333333	1806.666667	-0.043531808	0.928240457	0.993096978
26445	'Psmb2'	1092	1117	1096	830	1042	1412	1101.666667	1094.666667	0.01501732	0.958716099	0.999493374
26446	'Psmb3'	807	873	684	1361	877	1138	788	1125.333333	0.657135205	0.135824256	0.736689633
26447	'Poli'	816	809	270	50	439	522	631.6666667	337	-0.966143599	0.187526614	0.808805329
26448	'Mok'	147	138	83	141	121	98	122.6666667	120	0.123991906	0.79437423	0.972790461
26450	'Rbbp9'	1025	1146	782	687	1323	1340	984.3333333	1116.666667	0.199282998	0.461223791	0.972790461
26451	'Rpl27a'	9006.9	9443.2	18047	12696	7941	9566.83	12165.73333	10067.88	-0.187450187	0.746399438	0.972790461
26456	'Sema4g'	1267	1187	1019	1380	1556	870	1157.666667	1268.666667	0.254515597	0.56363292	0.972790461
26457	'Slc27a1'	1569.3	1489	422	513	2559	1634	1160.083333	1568.666667	0.438636995	0.480388663	0.972790461
26458	'Slc27a2'	53	38	17	3	42	72	36	39	0.03001977	0.973918372	0.999493374
26459	'Slc27a5'	2	0	2	1	2	2	1.333333333	1.666666667	0.282499983	0.878417031	0.981341203
26462	'Txnrd2'	203	230	120	22	239	163	184.3333333	141.3333333	-0.455515331	0.516463964	0.972790461
26464	'Vnn3'	0	0	89	14	1	1	29.66666667	5.333333333	-2.290607731	0.299533885	0.903468167
26465	'Zfp146'	1925	1975	3391	1049	2329	2038	2430.333333	1805.333333	-0.487105824	0.291088675	0.899022994
26466	'Zfp260'	1807	1911	2273	1333	2007	1674	1997	1671.333333	-0.233756467	0.497735832	0.972790461
264895	'Acsf2'	955	1019	471	2895	944	1499	815	1779.333333	1.392906435	0.03204819	0.433742328
26549	'Itgb1bp2'	46.14	63.03	20.93	87	49.19	20	43.36666667	52.06333333	0.562385539	0.484292103	0.972790461
26554	'Cul3'	5669	5586	5399	2635	3692	4584	5551.333333	3637	-0.590505571	0.023939537	0.382913609
26556	'Homer1'	990	978	1239	622	726	753	1069	700.3333333	-0.571378597	0.115019464	0.702296207
26557	'Homer2'	183	167	144	15	126	232	164.6666667	124.3333333	-0.530224111	0.478785148	0.972790461
26558	'Homer3'	636.34	624.46	748.29	355.37	688.52	622.67	669.6966667	555.52	-0.282583264	0.404147688	0.956543098
26559	'Hunk'	1181	1219	462	413	1330	1313	954	1018.666667	0.096789414	0.844541671	0.975182082
26561	'Mmp23'	301	287	166	47	443	396	251.3333333	295.3333333	0.153240514	0.821444968	0.972790461
26562	'Ncdn'	580	634.62	605	530.64	723	688	606.54	647.2133333	0.136180429	0.639026475	0.972790461
26563	'Ror1'	145	118	22	25	150	129	95	101.3333333	0.088017949	0.909694475	0.989364653
26564	'Ror2'	224	215	157	294	319	292	198.6666667	301.6666667	0.702595574	0.043875677	0.488386633
26565	'Pla2g10'	4	5	3	1	1	0	4	0.666666667	-2.455071837	0.146264243	0.753611197
26568	'Slc27a3'	386	391	407	786	509	567	394.6666667	620.6666667	0.790912112	0.097313543	0.663789939
26569	'Slc27a4'	1066	1074	604	564	1230	1188	914.6666667	994	0.141616069	0.671708923	0.972790461
26570	'Slc7a11'	579.09	493.08	902.26	755	792	1139	658.1433333	895.3333333	0.445167207	0.33346512	0.921648675
26572	'Cops3'	1686	1732	1193	593	1308	2000	1537	1300.333333	-0.267318232	0.495388804	0.972790461
26611	'Rcn2'	2072	1990	1010	954	2663	2515	1690.666667	2044	0.278092673	0.496857933	0.972790461
266614	'Ly6g5b'	34.96	30.57	13.88	38.11	21.03	14.01	26.47	24.38333333	0.167497615	0.826764058	0.972859382
266620	'Defb36'	0	0	7	1	0	0	2.333333333	0.333333333	-2.857163414	0.417938345	0.957720764

266632	'Irak4'	69	88	38	66	95	89	65	83.33333333	0.437976526	0.299117592	0.903100471
266645	'Acmsd'	2	1	1	2	2	0	1.333333333	1.333333333	0.181105622	0.925827196	0.992381554
266690	'Cyb5r4'	646	797	739	465	817	895	727.3333333	725.6666667	-0.005851525	0.984577307	0.999493374
266692	'Cpne1'	876	785.27	1146	2577	1083	1537	935.7566667	1732.333333	1.050783581	0.080416094	0.617305615
266744	'Lgsn'	2	0	0	2	1	0	0.666666667	1	0.939232173	0.762233008	0.972790461
266781	'Snx17'	1284	1402	1388	985	1464	1916	1358	1455	0.101864756	0.730989693	0.972790461
266815	'Mill1'	0	1	1	0	2	2	0.666666667	1.333333333	0.850169464	0.73146968	0.972790461
267019	'Rps15a'	6909.7	7060.6	11746	10090	5267	6767.97	8572.223333	7375.103333	-0.095820419	0.869440758	0.979633196
26754	'Cops5'	1318	1472	1680	535	1294	1626	1489.993333	1151.666667	-0.432336917	0.291274387	0.899151786
26757	'Dpysl4'	175	191	97	13	163	53	154.3333333	76.33333333	-1.068945557	0.162586649	0.774020016
268281	'Shprh'	1923	1873	984	1251	1862	1664	1593.333333	1592.333333	0.078529983	0.811220931	0.972790461
268291	'Rnf217'	680	585	4701	481	524	857	1988.666667	620.6666667	-1.804175141	0.040769422	0.473970635
268294	'Zbtb24'	783	800	403	517	575	638	662	576.6666667	-0.096035385	0.787755071	0.972790461
268297	'Scml4'	16	9	18	21	13	7	14.33333333	13.66666667	0.087447802	0.919180725	0.990815958
268301	'Sowahc'	537	536	1397	664	504	628	823.3333333	598.6666667	-0.43205154	0.486925449	0.972790461
268345	'Kcnc2'	40	28	36	0	37	25	34.66666667	20.66666667	-0.894881388	0.417086321	0.957382938
268354	'Tafa2'	35	36	485	4	49	26	185.3333333	26.33333333	-3.096439578	0.008372228	0.22261929
268373	'Ppia'	11116	12499	15844	6125	11381	16080	13152.87333	11195.33333	-0.28422892	0.481063036	0.972790461
268379	'Abca13'	3	9	2	21	11	26	4.666666667	19.33333333	2.189837468	0.017168246	0.327713554
268390	'Ahsa2'	670.12	698.66	735.53	358.86	1072	630.41	701.4366667	687.1033333	-0.057079747	0.891432517	0.985171
268395	'Mpg'	323.98	376.83	330.2	156.37	344.69	339.04	343.67	280.0333333	-0.307907568	0.347251665	0.928387732
268396	'Sh3pxd2b'	1778	1625	2099	2543	1530	1376	1834	1816.333333	0.142023078	0.794579847	0.972790461
268417	'Zkscan17'	1293	1241	2034	1588	1159	1418	1522.666667	1388.333333	-0.057690558	0.90851113	0.988823459
268420	'Alkbh5'	5249.7	5027.7	12166	5143.7	2825.5	5003.61	7481.213333	4324.256667	-0.740619322	0.23856087	0.864053647
268445	'Ankrd13b'	519	460	264	200	474	306	414.3333333	326.6666667	-0.302717728	0.441250911	0.968551476
268448	'Phf12'	2081	2074	1192	1561	1780	1464	1782.333333	1601.666667	-0.036155546	0.920988199	0.991047523
268449	'Rpl23a'	17216	18158	33797	27069	12874	15926	23057.07333	18623.16	-0.178746608	0.77779483	0.972790461
268451	'Rab11fip4'	1644	1451	179	354	1241	2415	1091.333333	1336.666667	0.286025925	0.723076977	0.972790461
268465	'Eme1'	255.9	267.42	62.37	10.01	178.13	260.13	195.23	149.4233333	-0.453583613	0.621201774	0.972790461
268469	'Zfp652'	567	515	461	406	391	465	514.3333333	420.6666667	-0.20637546	0.534014737	0.972790461
268470	'Ube2z'	3157	3197	4602	2780	2749	3990	3652	3173	-0.18186927	0.66081944	0.972790461
268480	'Rapgefl1'	401	362	145	98	386	391	302.6666667	291.6666667	-0.068167016	0.901143836	0.98672375
268481	'Krt222'	36	22	2	0	19	12	20	10.33333333	-0.989787215	0.465846561	0.972790461
268482	'Krt12'	1	1	0	0	3	6	0.666666667	3	2.100546935	0.341146929	0.925308103
268490	'Lsm12'	923	909	1558	541	742	981	1130	754.6666667	-0.611335022	0.169458657	0.785100556
268491	'Meioc'	2849	2926	643	14	1538	483	2139.333333	678.3333333	-1.713539208	0.121566196	0.712603747
268510	'Mgat5b'	20	24	2	2	20	11	15.33333333	11	-0.471150514	0.666308468	0.972790461
268512	'Slc26a11'	413	343	125	183	469	491	293.6666667	381	0.401699749	0.425076381	0.960285372
268515	'Bahcc1'	1745	1675	365	1098	1858	1565	1261.666667	1507	0.362691957	0.52004487	0.972790461
268527	'Greb1'	4261	4428	555	1165	5832	3122	3081.333333	3373	0.163741391	0.829402058	0.973678583

268534	'Sntg2'	42	39	21	9	52	31	34	30.66666667	-0.173138552	0.793942678	0.972790461
268564	'Zbtb1'	785	821	861	375	907	724	822.3333333	668.6666667	-0.319938194	0.361673068	0.933706718
268566	'Gphn'	900	849	2262	329	908	1040	1337	759	-0.948148211	0.122909833	0.714169238
268567	'Tmem229b'	274	306	304	142	248	235	294.6666667	208.3333333	-0.492098505	0.116474538	0.704330126
268591	'Serpina5'	0	0	1	0	0	2	0.333333333	0.666666667	0.78767502	0.845746136	0.975182082
268595	'D430019H16Rik'	460	494	420	280	384	323	458	329	-0.41770495	0.163476936	0.775648491
268656	'Sptlc1'	984	971	383	352	1015	1007	779.3333333	791.3333333	0.033048644	0.944014409	0.996592403
268663	'Cdhr2'	5	4	1	0	0	1	3.333333333	0.333333333	-3.095149928	0.146290583	0.753611197
268670	'Zfp759'	152.03	195	105	36	225	194	150.6766667	151.6666667	-0.050348777	0.932787355	0.993970337
268686	'S100z'	0	0	0	0	0	1	0	0.333333333	1.020273531	0.802557913	0.972790461
268697	'Ccnb1'	363.49	416.67	141.2	69.93	404.56	317.49	307.12	263.9933333	-0.245915737	0.691601873	0.972790461
268706	'Slc38a9'	640	654	261	386	644	426	518.3333333	485.3333333	0.003635451	0.993344076	0.999493374
268709	'Fam107a'	7	8	2	1	2	3	5.666666667	2	-1.452633958	0.244704799	0.866091613
268721	'Zswim8'	3510	3378	2167	3298	4058	2894	3018.333333	3416.666667	0.290678984	0.426123052	0.960958977
268729	'Frmpd2'	20	16	1	1	45	19	12.33333333	21.66666667	0.786044606	0.540074797	0.972790461
268739	'Arhgef40'	966	958	865	1071	1327	1043	929.6666667	1147	0.381636892	0.248244977	0.869491261
26874	'Abcd2'	83	82	6	101	91	65	57	85.66666667	0.81587954	0.340734106	0.925308103
268741	'Tox4'	1398.2	1504.8	2220.9	1856	1420	1960.85	1707.943333	1745.616667	0.092243218	0.845305768	0.975182082
268747	'Carmil3'	554	502	192	99	794	360	416	417.6666667	-0.019979692	0.976629443	0.999493374
268749	'Rnf31'	425	438	784	576	486	622	549	561.3333333	0.070368674	0.889743492	0.984980969
26875	'Pclo'	352	348	50	90	254	48	250	130.6666667	-0.813037697	0.325402408	0.920269559
268752	'Wdfy2'	1329	1421.9	881	1579	1595	1467.01	1210.64	1547.003333	0.474528813	0.1868737	0.808483975
268756	'Gulo'	49	49	13	41	116	94	37	83.66666667	1.215463043	0.041722191	0.477375478
268759	'9930012K11Rik'	80	86	13	49	88	87	59.66666667	74.66666667	0.41623065	0.536858945	0.972790461
26876	'Adh4'	21	9	1	12	7	5	10.33333333	8	-0.101632159	0.930196825	0.99325468
26877	'B3galt1'	84	83	33	23	78	48	66.66666667	49.66666667	-0.399522258	0.476485846	0.972790461
26878	'B3galt2'	1	0	0	0	3	0	0.333333333	1	1.381884369	0.730152291	0.972790461
268780	'Egflam'	596.76	520.7	132.63	166.04	628.32	485	416.6966667	426.4533333	0.056874918	0.926360719	0.992391389
268782	'Agxt2'	2	5	0	0	3	0	2.333333333	1	-1.199353354	0.657277539	0.972790461
268783	'Mtmr12'	789	817	482	326	669	618	696	537.6666667	-0.344125688	0.270016833	0.883851074
26879	'B3galnt1'	491	444	476	127	560	525	470.3333333	404	-0.300243256	0.548420478	0.972790461
268807	'Klhl38'	0	3	2	1	0	2	1.666666667	1	-0.700822293	0.759423083	0.972790461
268816	'Mroh5'	1	1	0	0	0	0	0.666666667	0	-1.703500596	0.673820112	0.972790461
268822	'Adck5'	576	532	342	305	805	714	483.3333333	608	0.332338517	0.361574781	0.933706718
26885	'Casp8ap2'	1496	1731	578	873	1137	1077	1268.333333	1029	-0.188345298	0.66774201	0.972790461
268857	'Nlrc3'	34	32	32	36	36	23	32.66666667	31.66666667	0.063592797	0.909901166	0.989364653
268859	'Rbfox1'	184	191	96	17	165	78	157	86.66666667	-0.907224912	0.192022398	0.813837363
26886	'Cenph'	246	242	98	22	165	294	195.3333333	160.3333333	-0.362176521	0.631086016	0.972790461
268860	'Abat'	2548	2912	1249	2606	3367	2844	2236.333333	2939	0.505614698	0.190221742	0.813136091
26887	'Chst4'	21	17	0	71	33	11	12.66666667	38.33333333	1.975149381	0.119551823	0.710007992

268878	'Atp13a5'	11	2	0	0	3	5	4.333333333	2.666666667	-0.736550925	0.706421212	0.972790461
26888	'Clec4a2'	10	7	0	11	5	4	5.666666667	6.666666667	0.541072836	0.692230158	0.972790461
268880	'Xxylt1'	431	401	184	354	692	709	338.6666667	585	0.831469454	0.031589139	0.431445911
268882	'Fbxo45'	1043	1038	979	773	826	786	1020	795	-0.278015646	0.403630391	0.956239376
268885	'Stfa2l1'	0	5	0	0	21	0	1.666666667	7	2.066004107	0.498255	0.972790461
26889	'Cln8'	354	340	1227	1755	490	769	640.3333333	1004.666667	0.789912308	0.353177457	0.930050349
268890	'Lsamp'	16	20	19	3	40	14	18.33333333	19	-0.039963301	0.964199222	0.999493374
268902	'Robo2'	280.99	276	563	129	504	559	373.33	397.3333333	-0.045863395	0.939752114	0.995607401
268903	'Nrip1'	1860	1965	1499	952	2061	2089	1774.666667	1700.666667	-0.06371822	0.826199549	0.972844676
26891	'Cops4'	1864	1772	2098	842	1679	1912	1911.333333	1477.666667	-0.402176034	0.239800376	0.86449675
26893	'Cops6'	1587	1869	1801	1016	1739	2496	1752.333333	1750.333333	-0.024638996	0.942429866	0.996301887
268930	'Pkmyt1'	205.27	239.25	111.58	84	226	330.75	185.3666667	213.5833333	0.18703365	0.700522819	0.972790461
268932	'Caskin1'	576	573	370	326	1045	896	506.3333333	755.6666667	0.559499451	0.166950387	0.781229856
268933	'Wdr24'	688	702	377	365	622	701	589	562.6666667	-0.024527395	0.938977483	0.995379339
268934	'Grm4'	83	58	17	230	58	102	52.66666667	130	1.622523698	0.049143212	0.515210762
268935	'Scube3'	616	511	420	622	629	780	515.6666667	677	0.475001413	0.138459144	0.739741822
268936	'Brpf3'	1652	1812	1002	429	2499	1792	1488.666667	1573.333333	0.031067815	0.955080202	0.999493374
26894	'Cops7a'	1504	1388	1347	556	1218	1661	1413	1145	-0.34111005	0.349221079	0.928823768
268949	'Mucl3'	42	39	10	20	22	28	30.33333333	23.33333333	-0.26373599	0.690059968	0.972790461
26895	'Cops7b'	531	555	277	273	533	484	454.3333333	430	-0.034744363	0.920403802	0.991018506
268958	'Capn11'	6	4	3	1	3	5	4.333333333	3	-0.547517559	0.645677293	0.972790461
26896	'Med14'	1738	1911	824	2076	1804	1595	1491	1825	0.459865718	0.319671049	0.916646777
26897	'Acot1'	661.81	690.82	661.16	581.48	864.96	1013.45	671.2633333	819.9633333	0.304664334	0.258738793	0.877564912
268970	'Arhgap28'	881	815	1060	2012	1116	1119	918.6666667	1415.666667	0.782461089	0.158313763	0.768890221
268973	'Nlrc4'	5	4	1	3	4	5	3.333333333	4	0.345479024	0.770691336	0.972790461
268977	'Ltbp1'	2466	2399	731	1014	2670	1053	1865.333333	1579	-0.157639082	0.783923617	0.972790461
268980	'Strn'	1890	1726	1480	1802	1560	2039	1698.666667	1800.333333	0.178978864	0.604973727	0.972790461
268996	'Ss18'	1534	1562	1413	1183	1756	1649	1503	1529.333333	0.063614287	0.801458059	0.972790461
269003	'Sap130'	1844	1685	1824	767	1474	1335	1784.333333	1192	-0.58333341	0.049884971	0.517975463
26901	'Ss18l2'	77	80	214	67	89	127	123.6666667	94.33333333	-0.458240711	0.465914062	0.972790461
269016	'Sh3rf2'	9	12	11	7	36	25	10.66666667	22.66666667	1.026486031	0.167398904	0.781391371
269019	'Stk32a'	12	13	21	2	10	11	15.33333333	7.666666667	-1.097236373	0.197128781	0.822696823
269023	'Zfp608'	209	184	205	228	150	211	199.3333333	196.3333333	0.088310287	0.84767242	0.975439569
26903	'Dysf'	239	214	79	49	290	349	177.3333333	229.3333333	0.325873448	0.625390207	0.972790461
269033	'4930503L19Rik'	355	385	317	68	484	330	352.3333333	294	-0.344436289	0.561028273	0.972790461
269037	'Ctif'	445	381	648	741	473	495	491.3333333	569.6666667	0.326468581	0.554496565	0.972790461
26904	'Sh2d1b1'	2	2	0	2	2	2	1.333333333	2	0.733808845	0.674045659	0.972790461
26905	'Eif2s3x'	4839	4890	9200	3554	4618	6779	6309.666667	4983.666667	-0.380663123	0.429351289	0.962386546
269053	'Gpr152'	9	5	0	6	3	1	4.666666667	3.333333333	-0.157444128	0.921402968	0.991066749
269060	'Dagla'	220	177	60	178	195	202	152.3333333	191.6666667	0.465398626	0.366346473	0.936406787

269061	'Cpsf7'	2742	2798	2279	969	2469	1782	2606.333333	1740	-0.584841248	0.079609966	0.617305615
26908	'Eif2s3y'	30.8	24.66	14.14	23.18	26.38	32.59	23.2	27.38333333	0.331273344	0.539546677	0.972790461
26909	'Exo1'	578	568	82	26	349	413	409.3333333	262.6666667	-0.685444697	0.450498584	0.970590732
26910	'Figla'	249	211	62	2	255	978	174	411.6666667	1.131547142	0.365697272	0.936382025
269109	'Dpp10'	85	77	13	0	118	61	58.33333333	59.66666667	-0.026103879	0.984087724	0.999493374
269113	'Nup54'	1026	1078	1642	858	848	1202	1248.666667	969.3333333	-0.348070405	0.427034782	0.961636249
269116	'Nfasc'	51	54	33	29	20	27	46	25.33333333	-0.735092815	0.16873975	0.784384366
26912	'Gcat'	169.96	178	320	163	154	189	222.6533333	168.6666667	-0.37500573	0.463227365	0.972790461
269120	'Optc'	5	16	12	1	4	5	11	3.333333333	-1.77209228	0.087844696	0.642347449
26913	'Gprin1'	108	100	115	2	113	62	107.6666667	59	-1.014573715	0.290606432	0.898595513
269132	'Colgalt2'	256	229	73	16	190	56	186	87.33333333	-1.113446571	0.17119848	0.787010498
26914	'Macroh2a1'	3535	3494	2534	1926	3211	3292	3187.666667	2809.666667	-0.148751458	0.525944203	0.972790461
269152	'Kif26b'	620	548	228	63	380	296	465.3333333	246.3333333	-0.949398471	0.12079827	0.712100232
26918	'Ern2'	10	6	1	0	4	1	5.666666667	1.666666667	-1.761050851	0.27552243	0.887234492
269180	'Inpp4a'	901	936	544	740	774	538	793.6666667	684	-0.073076558	0.862834012	0.977220339
269181	'Mgat4a'	339	282	70	137	240	251	230.3333333	209.3333333	-0.055258978	0.922298197	0.991300958
26919	'Zfp346'	1672	1652	751	1214	1438	1453	1358.333333	1368.333333	0.119917535	0.752102093	0.972790461
269198	'Nbeal1'	1339	1460	1051	3476	1497	1633	1283.333333	2202	1.009394984	0.087558367	0.642214018
26920	'Cntrl'	1313	1196	298	282	1259	710	935.6666667	750.3333333	-0.295685284	0.643039997	0.972790461
269209	'Stk36'	399	371	148	100	439	536	306	358.3333333	0.194653223	0.740916135	0.972790461
26921	'Map4k4'	7878	7692	9017	9124	8254	8786	8195.666667	8721.333333	0.171479723	0.661051474	0.972790461
26922	'Mecr'	368	359	289	218	339	366	338.6666667	307.6666667	-0.106817177	0.675644237	0.972790461
269224	'Pask'	1019	955	162	50	505	464	712	339.6666667	-1.099441529	0.184152098	0.804780995
269233	'Fam171a1'	757	784	408	272	1204	1036	649.6666667	837.3333333	0.333136278	0.509442635	0.972790461
269252	'Gtf3c4'	2661	2670.2	1468	1135	2052	1837.03	2266.403333	1674.676667	-0.385894699	0.213722682	0.842446242
269254	'Setx'	6276	6285	1500	1879	4355	4333	4687	3522.333333	-0.358605629	0.514261744	0.972790461
26926	'Aifm1'	1532	1641	1918	2712	1493	2478	1697	2227.666667	0.505389337	0.307295549	0.907830798
269261	'Rpl12'	12671	13023	27643	17880	10421	14866	17778.97333	14389.05667	-0.233044976	0.699270474	0.972790461
26927	'Foxl2'	1687	1797	264	39	2579	2133	1249.333333	1583.666667	0.281425006	0.794320667	0.972790461
269275	'Acvr1c'	49	51	39	58	88	63	46.33333333	69.66666667	0.651475997	0.112746472	0.698666814
269295	'Rtn4rl2'	10	8	195	100	8	40	71	49.33333333	-0.420847388	0.757312508	0.972790461
26931	'Ppp2r5c'	2174	2157	3775	1604	2176	2696	2702	2158.666667	-0.347998897	0.435430923	0.96581102
26932	'Ppp2r5e'	1743	1676	1428	1387	1722	1835	1615.666667	1648	0.087718829	0.733619027	0.972790461
269336	'Ccdc32'	205	217	197	293	288	272	206.3333333	284.3333333	0.556925776	0.137848289	0.739360306
269338	'Vps39'	1212	1164	884	708	1226	1299	1086.666667	1077.666667	0.012048894	0.960953457	0.999493374
26934	'Racgap1'	954	960	220	390	769	842	711.3333333	667	-0.026912456	0.961253709	0.999493374
269344	'Ell3'	30	45	15	32	63	60	30	51.66666667	0.834432224	0.11393526	0.698948431
269346	'Slc28a2'	1.23	1.67	2	1	4	1	1.633333333	2	0.549577997	0.743500542	0.972790461
269356	'Slc4a11'	87	91	270	354	100	281	149.3333333	245	0.798397181	0.317853029	0.916136994
26936	'Mprip'	8091.4	8007	4985	10770	9302	9268	7027.796667	9780	0.617212431	0.116063102	0.702960083

269378	'Ahcy'	2134.1	1314.3	924.49	7028	3043.7	5118.76	1457.633333	5063.483333	2.003198101	4.99E-04	0.043523633
26938	'St6galnac5'	7	7	2	5	5	2	5.333333333	4	-0.241781778	0.831002721	0.974324192
269389	'Tox2'	21	24	68	68	19	43	37.66666667	43.33333333	0.312162976	0.719354293	0.972790461
26939	'Polr3e'	821	899	492	375	929	1018	737.3333333	774	0.069141623	0.854026225	0.975734242
269397	'Ss18l1'	391	393	545	397	317	346	443	353.3333333	-0.243080195	0.602934864	0.972790461
26940	'Ecsit'	511	523	521	287	333	463	518.3333333	361	-0.490661909	0.116353447	0.703821742
269400	'Rtel1'	594	592	267	190	638	419	484.3333333	415.6666667	-0.207182853	0.660354264	0.972790461
269401	'Zfp512b'	2944	2770	885	546	2638	1619	2199.666667	1601	-0.455568377	0.445087694	0.969888068
26941	'Slc9a3r1'	531	505	220	930	750	925	418.6666667	868.3333333	1.204274947	0.008147486	0.218179228
26942	'Spag1'	210.09	215.06	89.13	142.05	236.1	190.03	171.4266667	189.3933333	0.226272027	0.586851265	0.972790461
269423	'Abhd18'	393	358	777	308	258	165	509.3333333	243.6666667	-0.978053156	0.115472688	0.702825844
269424	'Jade1'	1173	1184	1903	2424	1080	1368	1420	1624	0.344941519	0.577568809	0.972790461
26943	'Serinc3'	11832	12628	7234	24749	19834	22316	10564.66667	22299.66667	1.222068811	0.002414861	0.110099123
269437	'Plch1'	103	113	18	192	136	73	78	133.6666667	1.048480714	0.178269581	0.797673555
26945	'Tpsg1'	0	2	0	0	2	0	0.666666667	0.666666667	0.047401214	0.990575369	0.999493374
26946	'Trpc7'	98	98	113	0	35	24	103	19.66666667	-2.54012142	0.010377765	0.251982208
269470	'Wdr3'	1418	1401	1013	556	1000	1248	1277.333333	934.6666667	-0.441153004	0.122798025	0.714169238
269473	'Lrig2'	1424	1491	588	788	1521	1143	1167.666667	1150.666667	0.050586816	0.902576647	0.987331639
26949	'Vat1'	2027	1985	6856	2679	3058	4813	3622.666667	3516.666667	-0.119848785	0.857036685	0.976149994
26950	'Vsnl1'	52	78	16	11	62	31	48.66666667	34.66666667	-0.474309284	0.532558472	0.972790461
26951	'Zw10'	681	684	338	494	743	645	567.6666667	627.3333333	0.226526545	0.51663273	0.972790461
269513	'Nkain3'	126	153	71	16	117	46	116.6666667	59.66666667	-0.994499135	0.138379239	0.739741822
269514	'Fbxl4'	355	363	456	400	379	492	391.3333333	423.6666667	0.163125888	0.680001354	0.972790461
269523	'Vcp'	13817	14060	17417	8839	13823	14811	15098	12491	-0.276781394	0.401854429	0.955996639
269529	'Fbxo10'	1058	1112	911	430	1034	1143	1027	869	-0.264986448	0.431853314	0.963677723
269536	'Tex10'	2357.1	2367.6	1948.4	638	1429.9	1827.09	2224.333333	1298.32	-0.803008645	0.019469667	0.347725165
269582	'Clspn'	600	592	161	59	340	323	451	240.6666667	-0.927715936	0.16501476	0.778863111
269585	'Zscan20'	467	436	254	373	386	370	385.6666667	376.3333333	0.083877046	0.823149968	0.972790461
269587	'Epb41'	2299.1	2297.1	2009.7	1654	1893.4	2104.59	2201.963333	1883.973333	-0.161416497	0.559183773	0.972790461
269589	'Sytl1'	22	22	21	13	18	36	21.66666667	22.33333333	0.034203482	0.95353626	0.999097899
269593	'Luzp1'	2841.1	2826	985.04	1506.1	1592	1277	2217.386667	1458.37	-0.446041277	0.360689227	0.933706718
269604	'Gpr157'	139	122	120	81	99	124	127	101.3333333	-0.28662015	0.392842283	0.952648938
269608	'Plekhg5'	619	632	294	552	979	680	515	737	0.596328276	0.123933001	0.716557984
26961	'Rpl8'	9704	10150	17083	18188	9198	11832	12312.33333	13072.66667	0.210793909	0.723044701	0.972790461
269610	'Chd5'	156	118	56	66	46	30	110	47.33333333	-0.998093844	0.115987218	0.70293889
269614	'Pank4'	615	574	387	911	703	530	525.3333333	714.6666667	0.620803033	0.200449424	0.827926233
269615	'Plch2'	16	18	10	12	17	14	14.66666667	14.33333333	0.039586863	0.950085261	0.998065566
269623	'Rbm48'	197.14	211.57	206.35	68.17	229.86	204.34	205.02	167.4566667	-0.346023005	0.430059376	0.962548446
269629	'Lhfpl3'	8	2	5	0	5	10	5	5	-0.141247318	0.919943706	0.990933129
269633	'Wdr86'	274	313	37	61	451	269	208	260.3333333	0.328309356	0.68964205	0.972790461

269637	'Cnpy1'	431	421	57	3	570	1196	303	589.6666667	0.879852435	0.486603752	0.972790461
269639	'Zfp512'	1644	1654	1050	463	1437	1273	1449.333333	1057.666667	-0.470909608	0.232692902	0.859679981
269642	'Nat8l'	78	80	63	34	39	74	73.66666667	49	-0.565407925	0.190128505	0.813136091
269643	'Ppp2r2c'	98	101	14	32	78	148	71	86	0.295268697	0.700400073	0.972790461
26965	'Cul1'	3998	4050	3801	871	3667	3896	3949.666667	2811.333333	-0.570316827	0.243218231	0.865044378
26968	'Islr'	1004	1122	175	1111	1442	931	767	1161.333333	0.768745072	0.23967262	0.86449675
269682	'Golga3'	2624	2437	2525	1803	1765	2106	2528.666667	1891.333333	-0.348436854	0.295757758	0.90029104
269693	'Ccdc60'	24	21	1	6	26	51	15.33333333	27.66666667	0.843702304	0.431675197	0.963677723
269695	'Rnft2'	838	838	101	91	663.82	368.02	592.3333333	374.28	-0.654857467	0.424465995	0.960285372
26970	'Pla2g2e'	0	1	5	1	0	1	2	0.666666667	-1.600457595	0.507971673	0.972790461
269700	'Hectd4'	3572	3273	1260	3158	2857	2185	2701.666667	2733.333333	0.200366904	0.696323853	0.972790461
269701	'Wdr66'	8	14	5	3	13	12	9	9.333333333	0.043283869	0.960633874	0.999493374
269702	'Mphosph9'	411	402	154	102	428	248	322.3333333	259.3333333	-0.305224996	0.580373243	0.972790461
269704	'Zfp664'	4636	4731	2332	1677	3591	3550	3899.666667	2939.333333	-0.378515087	0.289245631	0.896355874
26971	'Pla2g2f'	6	3	1	15	3	7	3.333333333	8.333333333	1.608200595	0.190696602	0.813136091
269713	'Clip2'	779	793	840	1485	817	799	804	1033.666667	0.537054763	0.324344639	0.919683661
269717	'Orai2'	208	205	232	588	372	493	215	484.3333333	1.290863354	0.006599786	0.192446523
26972	'Spo11'	547	550	114	0	312	151	403.6666667	154.3333333	-1.45266589	0.274214924	0.885423597
269774	'Aak1'	2855	2965	1328	3138	2376	2114	2382.666667	2542.666667	0.282739742	0.566490799	0.972790461
269784	'Cntn4'	1557	1477	102	30	2469	1676	1045.333333	1391.666667	0.368569664	0.753761534	0.972790461
269788	'Lhfpl4'	9	12	1	2	2	4	7.333333333	2.666666667	-1.351989129	0.279445062	0.889355605
269799	'Clec4a1'	8	17	6	20	21	24	10.33333333	21.66666667	1.166828525	0.090121247	0.648437606
269800	'Zfp384'	1522	1546	1428	673	1318	1218	1498.666667	1069.666667	-0.483132874	0.078916269	0.616000706
269823	'Pon3'	388.21	455	263	300	634	756	368.7366667	563.3333333	0.616335466	0.075054064	0.603900623
269831	'Tspan12'	424	473	321	157	423	372	406	317.3333333	-0.36358107	0.310570062	0.910971113
269854	'Nat14'	49	64	59	38	73	76	57.33333333	62.33333333	0.116374099	0.777562978	0.972790461
269855	'Ssc5d'	147	117	34	43	119	51	99.33333333	71	-0.407010109	0.527045343	0.972790461
269862	'Olfr1349'	0	1	0	0	0	0	0.333333333	0	-0.903279821	0.824807108	0.972790461
26987	'Eif4e2'	2099	2097	2140	1562	1673	1976	2112	1737	-0.225360231	0.472111075	0.972790461
269870	'Zfp446'	359	392	233	129	320	260	328	236.3333333	-0.458262299	0.200006773	0.82759347
269878	'Megf8'	2729	2625	654	1965	3580	2648	2002.666667	2731	0.548901195	0.307736181	0.907963267
269881	'Map3k10'	833	833	756	1292	822	1049	807.3333333	1054.333333	0.523699085	0.24385219	0.865593324
269902	'Vmn2r57'	6	12	4	0	18	11	7.333333333	9.666666667	0.315330365	0.806004196	0.972790461
26992	'Brd7'	1983	2082	889	964	1857	1918	1651.333333	1579.666667	-0.01843937	0.961339637	0.999493374
269941	'Chsy1'	1015	951	868	762	1006	1071	944.6666667	946.3333333	0.049008572	0.848065546	0.975580696
269951	'Idh2'	2811	2865	4218	2706	2937	3434	3298	3025.666667	-0.098733131	0.809885236	0.972790461
269952	'Gdpgp1'	115	112	54	178	99	154	93.66666667	143.6666667	0.793899948	0.139607608	0.741816612
269954	'Ttll13'	91	116	27	18	82	97	78	65.66666667	-0.261357281	0.706679816	0.972790461
269955	'Rccd1'	126	148	74	112	191	143	116	148.6666667	0.427563329	0.262575037	0.88013169
269959	'Adamtsl3'	35	35	35	19	105	119	35	81	1.114494537	0.079372877	0.617305615

269966	'Nup98'	2200	2281	2417	4415	2223	2355	2299.346667	2997.666667	0.563986729	0.311611649	0.911537035
269994	'Gsg1l'	179	230	691	14	311	234	366.6666667	186.3333333	-1.212380393	0.203905935	0.832079769
269997	'Zfp747'	168.68	184.23	341.18	171.17	184.26	248.64	231.3633333	201.3566667	-0.20814487	0.683482313	0.972790461
269999	'Orai3'	321	367	258	256	510	592	315.3333333	452.6666667	0.523061271	0.090999942	0.649965349
270004	'Foxi2'	0	1	0	0	0	0	0.333333333	0	-0.903279821	0.824807108	0.972790461
270028	'Fam155a'	6	11	6	1	3	5	7.666666667	3	-1.369605097	0.198101148	0.823955156
270035	'Letm2'	526	534	169	84	348	225	409.6666667	219	-0.890672505	0.121273943	0.712503156
270049	'Galntl6'	2	0	0	6	3	4	0.666666667	4.333333333	2.909663477	0.119114512	0.709078602
270058	'Map1s'	848	839	766	470	905	950	817.6666667	775	-0.078977028	0.775904809	0.972790461
270066	'Slc35e1'	977	893	759	1265	973	1427	876.3333333	1221.666667	0.581605287	0.122853377	0.714169238
270076	'Gcdh'	497	560.06	312	493	686	667	456.3533333	615.3333333	0.50509619	0.094678712	0.660077618
27008	'Micall1'	1294.3	1363.2	1239.1	764.51	1382.1	1285.69	1298.866667	1144.106667	-0.170866088	0.516685693	0.972790461
270084	'Lpcat2'	17	26	33	160	53	45	25.33333333	86	1.99703152	0.01484199	0.303986836
270086	'Ogfod1'	658	658	590	1069	615	831	635.3333333	838.3333333	0.549859072	0.247171867	0.868940743
270091	'Lrrc36'	6	7	3	0	9	12	5.333333333	7	0.298830301	0.817706666	0.972790461
270096	'Mon1b'	649	635	405	565	550	562	563	559	0.106405906	0.766692381	0.972790461
270097	'Vat1l'	156	136	470	48	136	249	254	144.3333333	-0.9943346	0.1814435	0.802050906
270106	'Rpl13'	12359	11967	25025	19436	11719	14938.8	16450.39	15364.55	-0.016795133	0.977444542	0.999493374
270109	'Pcnx2'	235	182	13	78	127	169	143.3333333	124.6666667	-0.102977007	0.902395515	0.987331639
270110	'Irf2bp2'	2229	2285	10020	2199	2567	2902	4844.666667	2556	-0.990929131	0.17737827	0.79704932
270118	'Maml2'	227	217	348	121	160	159	264	146.6666667	-0.841130614	0.054577081	0.536237452
270120	'Fat3'	107	88	26	6	47	39	73.66666667	30.66666667	-1.287391286	0.092437879	0.653540343
27015	'Polk'	307	274	407	442	354	385	329.3333333	393.6666667	0.339991753	0.471084587	0.972790461
270150	'Ccdc153'	0	0	0	1	0	1	0	0.666666667	2.022653929	0.615796212	0.972790461
270151	'Nlrx1'	243	231	140	26	366	283	204.6666667	225	0.045810828	0.95128477	0.998334403
270152	'Jam1'	47	41	4	19	52	52	30.66666667	41	0.475159562	0.568847584	0.972790461
270156	'Nkapd1'	1104	1188	744	431	838	846	1012	705	-0.498110732	0.08257724	0.623598561
270160	'Rab39'	47	47	101	2	16	14	65	10.66666667	-2.746681614	4.59E-04	0.041791682
270162	'Elmod1'	8	3	4	4	8	2	5	4.666666667	-0.02602278	0.98154491	0.999493374
270163	'Myo9a'	6514	6556	1982	1415	4053	3522	5017.333333	2996.666667	-0.712262659	0.166995484	0.781229856
270166	'Clpx'	1580	1557	1415	2229	1496	1931	1517.333333	1885.333333	0.44286596	0.301626255	0.90469437
270190	'Ephb1'	190	168	194	118	192	221	184	177	-0.056165709	0.866434047	0.978294615
270192	'Rab6b'	181	182	298	426	403	1050	220.3333333	626.3333333	1.465312491	0.005921974	0.181288429
270198	'Pfkfb4'	268	287	130	227	338	214	228.3333333	259.6666667	0.29357683	0.494124398	0.972790461
270201	'Klhl18'	548	615	425	367	595	531	529.3333333	497.6666667	-0.042441427	0.872506209	0.980171118
270210	'Zfp651'	918	915	532	443	1148	1247	788.3333333	946	0.25692592	0.500733706	0.972790461
27027	'Tspan32'	63	60	2	3	70	51	41.66666667	41.33333333	-0.034806744	0.976627407	0.999493374
27028	'Ermap'	53	42	10	10	90	8	35	36	0.061892487	0.952062854	0.998640168
27029	'Sgsh'	227.47	237.6	24.56	252.78	414.61	456	163.21	374.4633333	1.307389413	0.070522072	0.589465353
270328	'Gsdmc3'	1	0	0	0	0	0	0.333333333	0	-0.903279821	0.824807108	0.972790461

27041	'G3bp1'	3483	3410	3971	3284	3306	3736	3621.333333	3442	-0.012862172	0.971255756	0.999493374
27045	'Nit1'	564.49	607.21	470.79	632.25	687.8	881.47	547.4966667	733.84	0.487387539	0.091351659	0.650235019
27047	'Omd'	2	2	1	0	5	14	1.666666667	6.333333333	1.806638795	0.261908854	0.879908021
27049	'Etv3'	829	837	1295	1022	861	957	986.9966667	946.6666667	0.005115934	0.991373396	0.999493374
27050	'Rps3'	15321	15874	33258	31956	12901	18613	21484.33333	21156.66667	0.109069317	0.872084682	0.980171118
27052	'Aoah'	37	44	11	8	18	17	30.66666667	14.33333333	-1.038629533	0.132888759	0.731465282
27053	'Asns'	823	876	4609	660	1107	1508	2102.666667	1091.666667	-1.090839374	0.169923975	0.785454025
27054	'Sec23b'	1030	1067	2184	1070	1118	1400	1427	1196	-0.257891758	0.618418836	0.972790461
27055	'Fkbp9'	5782	5597	2073	1664	3650	3942	4484	3085.333333	-0.502905147	0.258968764	0.877743829
27056	'Irf5'	218.38	234.07	173.22	150.4	221.04	230.37	208.5566667	200.6033333	-0.012979435	0.962303001	0.999493374
27057	'Ncoa4'	3701	4054	6561	3953	3517	4438	4772	3969.333333	-0.228046211	0.630913078	0.972790461
27058	'Srp9'	1047	1000	2262	997	1053	1778	1436.333333	1276	-0.209180238	0.701085949	0.972790461
27059	'Sh3d19'	985	1049	980	3529	1044	1621	1004.666667	2064.666667	1.276451078	0.053062049	0.532859388
270599	'Gm648'	0	1	2	0	2	0	1	0.666666667	-0.733221529	0.816021492	0.972790461
27060	'Tcirg1'	407	457	244	803	673	710	369.3333333	728.6666667	1.124971631	0.006704676	0.194609615
27061	'Bcap31'	1623	1783	1214	1359	1856	2843	1540	2019.333333	0.417159653	0.163939397	0.776592614
27062	'Cadps'	23	25	10	12	14	16	19.33333333	14	-0.375680814	0.557671456	0.972790461
270624	'Spin4'	176	170	186	36	191	185	177.3333333	137.3333333	-0.464314835	0.410028299	0.957157474
270627	'Taf1'	3137	3130	1896	1415	2463	2335	2721	2071	-0.348457325	0.211255494	0.840335617
270669	'Mbtps2'	596	625	542	246	623	686	587.6666667	518.3333333	-0.213453943	0.55110884	0.972790461
270672	'Map3k15'	1013.6	874.47	229.78	19.47	540.99	786.28	705.9333333	448.9133333	-0.734388541	0.449183343	0.97049895
270685	'Mthfd1l'	718	631	3662	99	489	700	1670.333333	429.3333333	-2.200768671	0.013057761	0.285349599
270711	'Calhm4'	6	18	7	9	5	5	10.33333333	6.333333333	-0.530087018	0.578050209	0.972790461
270757	'Bpifc'	3	6	1	4	1	4	3.333333333	3	0.030130379	0.982473997	0.999493374
27078	'B9d1'	184	189	61	6	176	132	144.6666667	104.6666667	-0.53996224	0.555020257	0.972790461
270802	'BC048403'	227	222	192	80	145	204	213.6666667	143	-0.588758991	0.090267644	0.648589738
27081	'Zfp275'	1333	1298	1379	1292	1281	1305	1336.666667	1292.666667	0.030804567	0.931251243	0.993601701
27083	'Xlr4b'	648.51	632.77	318.61	3.59	212.45	388.51	533.2966667	201.5166667	-1.529775467	0.146236914	0.753611197
27084	'Xlr5c'	128.3	122.81	39.52	3	34.54	133.63	96.87666667	57.05666667	-0.863113274	0.387886814	0.949955443
270893	'Tmem132e'	16	16	27	21	8	11	19.66666667	13.33333333	-0.417854927	0.612667103	0.972790461
270906	'Prr11'	233	237	270	75.07	183	126	246.6666667	128.0233333	-0.962905652	0.015908616	0.314401882
27096	'Trappc3'	790	883	851	732	815	988	841.3333333	845	0.055351271	0.858448105	0.976475731
271005	'Klhdc1'	239	234	200	37	221	194	224.3333333	150.6666667	-0.655064208	0.247892421	0.869384008
27103	'Eif2ak4'	925	970	714	1321	905	805	869.6666667	1010.333333	0.389614082	0.416909523	0.957382938
271036	'Catsperb'	1	0	0	0	1	0	0.333333333	0.333333333	0.058500858	0.988561293	0.999493374
271047	'Serpina3b'	2	1	0	1	2	4	1	2.333333333	1.254494417	0.496418213	0.972790461
271127	'Adamts16'	1079	1245	32	105	3442	1754	785.3333333	1767	1.148527886	0.325622094	0.920269559
271144	'Ankdd1b'	1.5	4.53	3.4	0	2	3.98	3.143333333	1.993333333	-0.788065387	0.63893535	0.972790461
271209	'Rpl1l'	0	0	0	0	1	0	0	0.333333333	1.020273531	0.802557913	0.972790461
271221	'Rubcnl'	10	9	79	8	8	3	32.66666667	6.333333333	-2.386833388	0.038116275	0.459930654

271278	'BC024139'	3	7	0	0	7	2	3.333333333	3	-0.158214835	0.935453876	0.994413066
271305	'Phf21b'	42	38	71	11	22	5	50.33333333	12.66666667	-1.97474579	0.009357774	0.23755153
271375	'Cd200r2'	3	1	0	0	0	0	1.333333333	0	-2.70051453	0.497202575	0.972790461
271377	'Zbtb11'	1566	1659	978	1192	1098	1198	1401	1162.666667	-0.146007662	0.689523002	0.972790461
27140	'Tlx3'	10	10	2	1	6	2	7.333333333	3	-1.246442354	0.305476033	0.907188578
271424	'Ip6k3'	2	0	13	21	7	14	5	14	1.513457089	0.285105703	0.893711786
271457	'Rab5a'	1457.9	1473.9	4485.9	2834	1868.9	2647.99	2472.58	2450.31	0.011677922	0.986185824	0.999493374
271508	'Brd8dc'	48	47	24	29	58	14	39.66666667	33.66666667	-0.130961347	0.848876218	0.975686757
271564	'Vps13a'	1760	1867	743	2016	2014	3114	1456.666667	2381.333333	0.80957722	0.054726748	0.536875608
271639	'Adcy10'	155	152	22	6	89	73	109.6666667	56	-1.002517317	0.278991278	0.889355605
271697	'Cdk15'	2	1	22	11	7	7	8.333333333	8.333333333	-0.02003946	0.988081656	0.999493374
271711	'Tmem169'	16	11	13	34	9	10	13.33333333	17.66666667	0.667013374	0.46909685	0.972790461
27176	'Rpl7a'	15053	15496	22063	18634	12141	15250	17537.06667	15341.50333	-0.092735477	0.853092025	0.975734242
271786	'Galnt13'	5	2	5	2	4	9	4	5	0.25518982	0.823386145	0.972790461
271813	'Agbl2'	93	100	20	2	71	72	71	48.33333333	-0.618164858	0.541519294	0.972790461
271842	'Rpusd2'	197	243	130	66	171	244	190	160.3333333	-0.266869797	0.558607002	0.972790461
271844	'Pla2g4f'	2	1	0	3	0	0	1	1	0.522981972	0.861338487	0.976863639
271849	'Shc4'	203	218	292	34	135	57	237.6666667	75.33333333	-1.713907546	0.002324811	0.108371515
271944	'C2cd4d'	23	21	6	4	24	21	16.66666667	16.33333333	-0.041014172	0.960931867	0.999493374
271970	'Arsj'	26	22	71	1	24	10	39.66666667	11.66666667	-1.955191345	0.05417935	0.534539595
271981	'Tbck'	973	938	560	226	640	680	823.6666667	515.3333333	-0.689754105	0.085558416	0.634617076
272009	'Srsf12'	426	422	616	217	437	380	488	344.6666667	-0.53198178	0.191613876	0.813837363
272027	'Tstd2'	1233.3	1305.9	1084.6	522.59	1139.1	1051.81	1207.953333	904.4833333	-0.419862135	0.148960334	0.757738857
272031	'Plppr1'	24	28	43	26	35	18	31.66666667	26.33333333	-0.221725902	0.729669114	0.972790461
27204	'Syn3'	94.86	73.23	22.03	45.75	105.02	49.08	63.37333333	66.61666667	0.164809945	0.794741413	0.972790461
27205	'Podxl'	8840	8331	1572	1643	9914	8259	6247.666667	6605.333333	0.072930663	0.919626467	0.990815958
27206	'Nrk'	1095	1046	268	156	1330	443	803	643	-0.325448987	0.667075156	0.972790461
27207	'Rps11'	6760	6784	15123	11276	5569	7015	9555.666667	7953.333333	-0.156341275	0.811346114	0.972790461
27214	'Dbf4'	618	578	376	329	471	569	524	456.3333333	-0.151305596	0.594479806	0.972790461
27215	'Azi2'	1352.5	1325.8	1311.9	1974.7	1393.5	1545.62	1330.04	1637.943333	0.430603404	0.326390822	0.920537354
272158	'Poln'	687	718	95	8	272	132	500	137.3333333	-1.902114042	0.058742643	0.552152463
27217	'Mixl1'	4	10	4	0	4	0	6	1.333333333	-2.198741218	0.205180487	0.833294914
27218	'Slamf1'	0	0	1	0	0	1	0.333333333	0.333333333	0.058500858	0.988561293	0.999493374
27219	'Sgk2'	4	5	1	2	2	1	3.333333333	1.666666667	-0.841655136	0.56023132	0.972790461
27220	'Cartpt'	0	0	1	0	0	1	0.333333333	0.333333333	0.058500858	0.988561293	0.999493374
27221	'Chaf1a'	1087.2	1128.6	343.35	208.49	802.2	1011.24	853.0266667	673.9766667	-0.356163435	0.549639998	0.972790461
27222	'Atp1a4'	4	0	0	0	0	0	1.333333333	0	-2.70473077	0.496508955	0.972790461
27223	'Trp53bp1'	2864	2803.6	1353	1630.7	3344.9	2232.41	2340.213333	2402.68	0.100478838	0.79508549	0.972790461
27224	'Eloa'	2247	2229	3266	1899	1738	2109	2580.666667	1915.333333	-0.38110818	0.377184845	0.941504733
27225	'Ddx24'	2765	2934	1531	2286	1989	3068	2410	2447.666667	0.128534477	0.73555555	0.972790461

27226	'Pla2g7'	210	212	150	494	207	427	190.6666667	376	1.150148335	0.034718961	0.447068151
272322	'Arntl2'	48	44	30	21	144	90	40.66666667	85	1.00695366	0.117153191	0.705163127
272347	'Zfp398'	1159.7	1046.8	428.03	749.36	875.9	827.62	878.1633333	817.6266667	0.020822025	0.961471643	0.999493374
272359	'Irf2bp1'	802	831	930	1164	1063	1176	854.3333333	1134.333333	0.487010072	0.205517233	0.833294914
272381	'Lrrc4b'	41	62	32	20	292	228	45	180	1.905808872	0.015790929	0.312400768
272382	'Spib'	13	12	116	0	5	1	47	2	-4.788635781	5.82E-04	0.047144852
272396	'Tarsl2'	482	506	613	101	413	452	533.6666667	322	-0.827302028	0.111106973	0.694172122
272411	'B3gnt6'	1	0	1	4	1	1	0.666666667	2	1.80201535	0.37965156	0.943151111
272428	'Acsm5'	0	0	0	0	6	1	0	2.333333333	3.546986629	0.333234524	0.921648675
272465	'Tmem255b'	98	97	6	100	182	111	67	131	1.107485815	0.188883549	0.811354278
272538	'Tango6'	415	398	200	385	349	438	337.6666667	390.6666667	0.333461312	0.408661914	0.957157474
272551	'Gins2'	278	315	256	32	285	301	283	206	-0.568401537	0.396975003	0.954358954
272589	'Tbcel'	1529	1496	1647	1529	1241	1620	1557.333333	1463.333333	-0.005862462	0.988039546	0.999493374
27260	'Plek2'	23	21	97	48	8	28	47	28	-0.640137837	0.52853107	0.972790461
27261	'Dok3'	25	38	14	48	39	11	25.66666667	32.66666667	0.583791526	0.473357738	0.972790461
27263	'Smok2a'	0	1	1	0	0	0	0.666666667	0	-1.851176388	0.646290011	0.972790461
272636	'Esyt3'	149.16	146.25	37.33	118.96	144.82	128.17	110.9133333	130.65	0.370265768	0.509586495	0.972790461
272643	'Prss43'	0	0	0	0	1	0	0	0.333333333	1.020273531	0.802557913	0.972790461
27267	'Cars'	922	864	2337	1178	938	1245	1374.333333	1120.333333	-0.281250988	0.645911125	0.972790461
27273	'Pdk4'	183	190	1924	2468	178	747	765.6666667	1131	0.724701875	0.547971443	0.972790461
27274	'Zfp354b'	62.45	64.33	32.33	56.41	99.31	68.45	53.03666667	74.72333333	0.568640951	0.199651476	0.827115347
27275	'Nufip1'	498	409	1291	387	397	495	732.6666667	426.3333333	-0.806989937	0.193552997	0.81737663
27276	'Plekhb1'	170	219	65	56	274	159	151.3333333	163	0.110276645	0.857713468	0.976399524
27277	'Golga5'	995	979	1043	912	941	973	1005.666667	942	-0.023309436	0.946401588	0.996592403
27278	'Clnk'	7	3	1	0	1	0	3.666666667	0.333333333	-3.231114078	0.128898787	0.72577634
27279	'Tnfrsf12a'	94	97	1051	1416	150	617	414	727.6666667	0.92530049	0.427826435	0.962014923
272790	'Magee2'	87	76	23	37	91	83	62	70.33333333	0.22730313	0.690552475	0.972790461
27280	'Phlda3'	286	295	587	363	457	538	389.3333333	452.6666667	0.196434289	0.695983277	0.972790461
27281	'Plaat1'	7	4	1	0	3	2	4	1.666666667	-1.278709687	0.424690526	0.960285372
27354	'Nbn'	842	798	1256	222	757	658	965.3333333	545.6666667	-0.912816727	0.065828283	0.574593252
27355	'Pald1'	1227	1262	448	1561	2113	1541	979	1738.333333	0.955543575	0.037422619	0.459930654
27356	'Insl6'	22	16	15	38	15	38	17.66666667	30.33333333	0.916585887	0.187428921	0.808617437
27357	'Gyg'	787	752	2210	608	673	814	1249.666667	698.3333333	-0.87415494	0.153802508	0.765411885
27359	'Sytl4'	1232.9	1295	509	790	2085	1845	1012.313333	1573.333333	0.662091705	0.133211671	0.732394533
27360	'Add3'	2352	2471	3094	2065	3054	3647	2639	2922	0.140123459	0.68412825	0.972790461
27361	'Msrb1'	116	161	292	208	154	216	189.6666667	192.6666667	0.060311085	0.918114307	0.990727847
27362	'Dnajb9'	1184	918	3811	1062	746	1959	1971	1255.666667	-0.717519545	0.326861173	0.920734892
27364	'Srr'	853.24	867.87	568.8	477.01	939.01	788.57	763.3033333	734.8633333	-0.0205734	0.943326083	0.996436989
27366	'Txnl4a'	1190.5	1165.6	2169.2	1772.7	1294.3	1489.14	1508.42	1518.713333	0.079866549	0.882377465	0.982819066
27367	'Rpl3'	22434	22174	43344	30185	19342	24987	29317.21667	24838	-0.163808724	0.772702723	0.972790461

27368	'Tbl2'	1304	1174	2656	510	931	1175	1711.333333	872	-1.052633025	0.045691021	0.498244985
27369	'Dguok'	301	291	278	75	281	213	290	189.6666667	-0.661353531	0.139545817	0.741695575
27370	'Rps26'	8614	9090	16759	8647	6248	7352	11487.66667	7415.666667	-0.563907906	0.300499872	0.904081584
27371	'Sh2d2a'	2	1	0	0	0	2	1	0.666666667	-0.591584204	0.853544845	0.975734242
27373	'Csnk1e'	1143	1132	2082	2103	1191	1555	1452.333333	1616.333333	0.253470053	0.662863266	0.972790461
27374	'Prmt5'	1185	1207	1119	678	1378	1376	1170.333333	1144	-0.039622442	0.890090791	0.984980969
27375	'Tjp3'	15	23	13	66	10	47	17	41	1.496400599	0.085518626	0.634617076
27376	'Slc25a10'	397	419	197	854	506	682	337.6666667	680.6666667	1.196784052	0.017741349	0.333162012
27377	'Yme1l1'	3057	3133	3564	2440	3337	3752	3251.333333	3176.333333	-0.016479648	0.957314947	0.999493374
27378	'Tcl1b3'	0	0	0	0	0	1	0	0.333333333	1.020273531	0.802557913	0.972790461
27379	'Tcl1b1'	0	0	0	0	1	2	0	1	2.297812331	0.566584047	0.972790461
27380	'Tcl1b4'	0	0	0	0	1	0	0	0.333333333	1.020273531	0.802557913	0.972790461
27381	'Tcl1b2'	0	0	0	0	10	14	0	8	5.288386187	0.041911204	0.477648737
27384	'Akr1c13'	77	98	26	19	78	89	67	62	-0.121016304	0.853767631	0.975734242
27385	'Magel2'	64	66	12	12	113	45	47.33333333	56.66666667	0.256407822	0.766046193	0.972790461
27386	'Npas3'	257.95	274.81	137.68	19.7	142.32	168.73	223.48	110.25	-1.093379698	0.10041766	0.671759705
27387	'Sh2d3c'	278	328	97	310	519	293	234.3333333	374	0.793202989	0.127120019	0.722729007
27388	'Ptdss2'	767	758	417	557	885	982	647.3333333	808	0.370428827	0.230382242	0.857148178
27389	'Dusp13'	1	0	0	1	0	0	0.333333333	0.333333333	0.058500858	0.988561293	0.999493374
27390	'Mme11'	26	23	21	46	12	21	23.33333333	26.33333333	0.409959093	0.608546877	0.972790461
27392	'Pign'	1436	1507	961	1049	1663	1609	1301.333333	1440.333333	0.19730774	0.443677385	0.969836424
27393	'Mrpl39'	1078.2	1178.4	982.17	712.33	1017.6	1056	1079.57	928.65	-0.178053239	0.465472856	0.972790461
27395	'Mrpl15'	765	865	821	347.59	652.02	894	817	631.2033333	-0.399255215	0.245660178	0.866832091
27397	'Mrpl17'	1000	1132	1101	580	699	1092	1077.666667	790.3333333	-0.433488329	0.187694567	0.808805329
27398	'Mrpl2'	615	624	423	307	502	705	554	504.6666667	-0.119689032	0.699533063	0.972790461
27399	'Ip6k1'	3165	3097	3338	4706	3032	3168	3200	3635.333333	0.327798388	0.49697797	0.972790461
27400	'Hsd17b6'	0	1	0	0	0	0	0.333333333	0	-0.903279821	0.824807108	0.972790461
27401	'Skp2'	531	583	287	584	490	294	467	456	0.160112796	0.763348647	0.972790461
27402	'Pdhx'	588	596	437	383	574	646	540.3333333	534.3333333	0.021869615	0.929217946	0.993096978
27403	'Abca7'	174	162	103	1538	215	332	146.3333333	695	2.616842082	0.002808218	0.118908317
27404	'Abca8b'	127	137	121	218	188	265	128.3333333	223.6666667	0.880308456	0.019951729	0.349283865
27405	'Abcg3'	4	3	2	0	7	19	3	8.666666667	1.403754207	0.33072978	0.921648675
27406	'Abcf3'	1380	1402	1480	1066	1271	1762	1420.666667	1366.333333	-0.032545142	0.916701066	0.990526893
27407	'Abcf2'	1661	1732	843	1107	1555	1946	1412	1536	0.183961745	0.585552609	0.972790461
27409	'Abcg5'	3	8	2	2	5	2	4.333333333	3	-0.456944549	0.713322726	0.972790461
27410	'Abca3'	1025	920	539	556	1224	1654	828	1144.666667	0.464542102	0.235515698	0.861636582
27411	'Slc14a2'	2	0	3	0	1	2	1.666666667	1	-0.926043395	0.685622207	0.972790461
27412	'Peg12'	164	176	109	24	148	133	149.6666667	101.6666667	-0.620838473	0.291984293	0.89958198
27413	'Abcb11'	9	15	2	1	20	12	8.666666667	11	0.315479695	0.785118703	0.972790461
27414	'Sergef'	186.95	166.97	188.15	230.02	143.64	185.48	180.69	186.38	0.173045255	0.722655898	0.972790461

27416	'Abcc5'	2964	3069	1377	4948	4712	2505	2470	4055	0.905818801	0.089681347	0.64682161
27418	'Mkln1'	2134	2197	1596	2062	2275	2307	1975.666667	2214.666667	0.254316238	0.394324666	0.953178011
27419	'Naglu'	362	367	226	561	594	699	318.3333333	618	1.049641632	0.001238592	0.074755901
27421	'Abcc6'	16	11	0	0	8	3	9	3.666666667	-1.303393761	0.45886605	0.972790461
27425	'Atp5l'	1188	1261	1346	995	915	1282	1265	1064	-0.192287197	0.593928439	0.972790461
27426	'Nagpa'	195	194	73	128	243	245	154	205.3333333	0.470407737	0.285290176	0.893711786
27428	'Shroom3'	961	859	2165	2703	942	1415	1328.333333	1686.666667	0.485680804	0.506226002	0.972790461
27494	'Amot'	1550	1563	617	1000	1787	1582	1243.333333	1456.333333	0.298917241	0.457897768	0.972790461
27528	'Nrep'	3853	4030	868	6346	4065	2642	2917	4351	0.848352639	0.223708679	0.850759465
27632	'Nelfe'	1017	1006	684	506	808	1207	902.3333333	840.3333333	-0.089935107	0.778586132	0.972790461
27643	'Ubl4a'	703.87	740.37	956.24	281.47	717.95	854.88	800.16	618.1	-0.443521682	0.306841744	0.907830798
27660	'1700088E04Rik'	108.69	136.85	137.86	40.49	87.88	68.31	127.8	65.56	-0.973092884	0.01708245	0.32733359
276742	'Taar7e'	1	0	0	1	1	0	0.333333333	0.666666667	1.081626198	0.75986402	0.972790461
276770	'Eif5a'	5728.6	5830.3	10487	5567.8	4906.4	7954.43	7348.526667	6142.896667	-0.25045002	0.612151325	0.972790461
27681	'Snf8'	784	961	449	577	635	954	731.3333333	722	0.058686472	0.873166941	0.980171118
276829	'Smtnl2'	645	514	4116	357	557	615	1758.333333	509.6666667	-1.921473603	0.02613381	0.397137149
276846	'Pigs'	1096	1085	404	821	1340	1641	861.6666667	1267.333333	0.616874966	0.145667129	0.753611197
276852	'D11Wsu47e'	872.24	942.19	456.52	346.19	816.92	767.28	756.9833333	643.4633333	-0.212752928	0.572057013	0.972790461
276891	'Timd4'	2	2	0	0	2	18	1.333333333	6.666666667	2.235181112	0.263944427	0.880205046
276905	'Armc7'	207	209	190	104	193.68	224	202	173.8933333	-0.220504947	0.483555808	0.972790461
276919	'Gemin4'	746	734	247	91	672	780	575.6666667	514.3333333	-0.215040999	0.758150858	0.972790461
276920	'Ccdc42'	66	43	6	2	33	26	38.33333333	20.33333333	-0.940273207	0.365437169	0.936220381
276950	'Slfn8'	11.34	8	21.3	52.35	6	15	13.54666667	24.45	1.115247721	0.297691684	0.900843503
276952	'Rasl10b'	225	238	41	18	168	122	168	102.6666667	-0.730518254	0.369943064	0.937712122
277010	'Marveld1'	694	708	425	493	959	1065	609	839	0.482443588	0.120435362	0.711929925
277154	'Nynrin'	3736	3738	2707	981	4319	3207	3393.666667	2835.666667	-0.304935443	0.516336821	0.972790461
277203	'Tm4sf19'	1	2	1	0	3	4	1.333333333	2.333333333	0.712524114	0.693243544	0.972790461
277250	'Kdm3b'	3210	3209	1504	1835	3472	3114	2641	2807	0.142029219	0.690833205	0.972790461
277328	'Trpa1'	5	7	16	109	11	51	9.333333333	57	2.809447164	0.006919239	0.196928988
277343	'Wfdc8'	4	3	0	6	3	2	2.333333333	3.666666667	0.933497818	0.543722792	0.972790461
277353	'Tcf15'	408	370.82	74	18	191	378	284.2733333	195.6666667	-0.601024141	0.506045974	0.972790461
277360	'Prex1'	463	502	154	1621	441	665	373	909	1.603817857	0.031932458	0.433742328
277396	'Klhl23'	3758	4579	509	710	7350	4089	2948.666667	4049.666667	0.448891069	0.6007847	0.972790461
277414	'Trp53i11'	1034	1108	529	292	1929	1935	890.3333333	1385.333333	0.576752043	0.34802496	0.928444937
277432	'Vstm2l'	2	3	0	1	0	2	1.666666667	1	-0.587707834	0.797487794	0.972790461
277463	'Gpr107'	1237	1192	537	837	1377	1422	988.6666667	1212	0.357683684	0.329719656	0.921648675
277468	'Slc39a12'	2	1	1	0	6	4	1.333333333	3.333333333	1.224900629	0.481711658	0.972790461
277496	'Lkaaear1'	1	1	0	0	1	0	0.666666667	0.333333333	-0.74517548	0.838722495	0.974723675
27756	'Lsm2'	465	496	296	94	367	608	419	356.3333333	-0.301136993	0.593571133	0.972790461
27762	'Vwa7'	16.28	7.53	2.17	0	9.69	11.25	8.66	6.98	-0.382290672	0.777895339	0.972790461

277666	'Pramel24'	0	2	3	0	0	0	1.666666667	0	-3.209945109	0.399129577	0.955184049
277743	'Fam131c'	4	3	48	139	4	17	18.33333333	53.33333333	1.77558596	0.227526469	0.854044074
277744	'Srarp'	1	0	0	0	0	0	0.333333333	0	-0.903279821	0.824807108	0.972790461
277773	'Fam205c'	6	2	0	10	1	6	2.666666667	5.666666667	1.398706858	0.380933515	0.944233436
27784	'Commd8'	961	957	1070	878	1005	1051	996	978	0.024349502	0.941573509	0.996210609
277854	'Depdc5'	1286	1332	564	1043	842	827	1060.666667	904	-0.057882905	0.902829756	0.987381169
277898	'Slc15a5'	1	6	0	1	0	2	2.333333333	1	-1.063003344	0.644901954	0.972790461
277939	'C2cd3'	1212	1189	580	479	1275	1131	993.6666667	961.6666667	-0.032369284	0.936125289	0.994413066
277973	'Slc9a5'	152	174	75	176	144	60	133.6666667	126.6666667	0.145980014	0.820863519	0.972790461
277978	'Exoc3l'	216	289	69	66	490	302	191.3333333	286	0.561455322	0.428035886	0.962191208
27801	'Zdhhc8'	755	721	907	1019	918	968	794.3333333	968.3333333	0.363931342	0.375146153	0.940406075
278087	'Gm5071'	0	0	0	0	0.5	2.35	0	0.95	1.749449488	0.66566965	0.972790461
278097	'Armcx6'	935	1097	1183	505	1378	1170	1071.666667	1017.666667	-0.119314282	0.761410579	0.972790461
278167	'Mageb6b1'	2.68	0	1	0	0	1.21	1.226666667	0.403333333	-1.433748416	0.667550462	0.972790461
278240	'Spin2c'	81	56	31	14	87	65	56	55.33333333	-0.052907023	0.936222671	0.994413066
278279	'Tmtc2'	338	339	673	928	361	484	450	591	0.543013998	0.429356502	0.962386546
278304	'Zfp385c'	11	15	8	52	8	6	11.33333333	22	1.341197603	0.224292206	0.850759465
278507	'Wfikkn2'	75	53	9	20	73	33	45.66666667	42	-0.057523284	0.942994152	0.996301887
278672	'Duxbl1'	128	125.39	48.48	10.58	196	87	100.6233333	97.86	-0.098184532	0.908300311	0.988823459
278679	'Apol7b'	7.31	2.32	0	0	0	0	3.21	0	-3.86813749	0.248830143	0.869594312
27878	'Tada1'	635.08	611.49	542.5	177.12	720.85	691.91	596.3566667	529.96	-0.235751386	0.615704641	0.972790461
278795	'Lrrc10b'	9	15	2	1	9	4	8.666666667	4.666666667	-0.874627116	0.456406545	0.97151713
27883	'Tango2'	125	128	80	185	138	128	111	150.3333333	0.600412925	0.213024101	0.841028891
27886	'Ess2'	486	421	519	334	460	575	475.3333333	456.3333333	-0.050912081	0.877136628	0.981341203
279028	'Adamts13'	106.77	91.45	251.11	2	35.13	17.52	149.7766667	18.21666667	-3.231942862	2.13E-04	0.023582255
279029	'Stkld1'	33	45	13	0	8	12	30.33333333	6.666666667	-2.245204972	0.026427503	0.398761661
279185	'Pramel21'	1	2	1	0	0	0	1.333333333	0	-2.778388356	0.385894181	0.947516488
279499	'Kctd19'	5	7	0	0	2	1	4	1	-1.976964683	0.330451367	0.921648675
279561	'Wnk3'	741	828	232	171	500	516	600.3333333	395.6666667	-0.580783757	0.2823399	0.890463
279572	'Tlr13'	13	19	0	20	38	44	10.66666667	34	1.765863065	0.087881332	0.642368277
279618	'Gm715'	56	57	12	2	33	47	41.66666667	27.33333333	-0.665726168	0.491563495	0.972790461
27965	'Spg21'	1146	1117	4095	640	1126	1623	2119.333333	1129.666667	-1.035441461	0.131823079	0.72897159
279653	'Pcdh19'	142	138	19	140	294	150	99.66666667	194.6666667	1.085902386	0.127417113	0.723768792
27966	'Rrp9'	216	225	298	207	223	235	246.3333333	221.6666667	-0.108998047	0.789946574	0.972790461
27967	'Cherp'	1851	1762	1487	1042	1500	1639	1700	1393.666667	-0.248760904	0.281698835	0.889731741
279706	'Nup62cl'	517	484	74	69	274	482	358.3333333	275	-0.394755575	0.611982448	0.972790461
27973	'Vkorc1'	440	469	464	463	553	1079	457.6666667	698.3333333	0.604704422	0.113450726	0.698948431
279766	'Rhbdd3'	332.76	330	187	65	338	288	283.2533333	230.3333333	-0.3428805	0.528524904	0.972790461
27979	'Eif3b'	3734	3706	4347	4153	3794	4872	3929	4273	0.183157574	0.623926511	0.972790461
27981	'Rsrp1'	2567	2563	3338	3359	3157	2295	2822.666667	2937	0.155368472	0.740421018	0.972790461

27984	'Efhd2'	711	749	1014	930	849	923	824.6666667	900.6666667	0.190906098	0.658147895	0.972790461
27993	'Imp4'	626	650	611	201	603	753	629	519	-0.339396481	0.432297793	0.963974389
27998	'Exosc5'	284	305	286	115	204	386	291.6666667	235	-0.353532989	0.409672963	0.957157474
27999	'Fam3c'	427	439	280	282	666	616	382	521.3333333	0.456410968	0.170399408	0.785667192
28000	'Prpf19'	2828	3112	2968	2053	2817	3214	2969.333333	2694.666667	-0.113250198	0.673841638	0.972790461
28006	'Washc2'	2233	2276	1893	679	2363	2007	2134	1683	-0.386051871	0.348630781	0.928444937
28010	'Miip'	118	106	124	143	299	231	116	224.3333333	0.948363099	0.009969253	0.246470009
28015	'Polr2m'	4109	3866	5378	2120	3741	4265	4451	3375.333333	-0.428758243	0.244145365	0.866091613
28018	'Ubfd1'	3171	3325	3299	1492	2738	3443	3265	2557.666667	-0.371165281	0.233103071	0.859679981
28019	'Ing4'	1340	1472	747	575	1675	1352	1186.333333	1200.666667	0.023736134	0.953053417	0.999032683
28028	'Mrpl50'	711	774	1031	478	703	737	838.6666667	639.3333333	-0.393023576	0.292594241	0.900043079
280287	'Kiss1'	1	2	1	1	2	1	1.333333333	1.333333333	0.055174554	0.974746012	0.999493374
28030	'Gfm1'	555	580	426	795	698	922	520.3333333	805	0.727387626	0.035180166	0.449795097
28035	'Usp39'	645	607	664	1111	648	834	638.6666667	864.3333333	0.576246903	0.238493773	0.864053647
28036	'Larp7'	1954	1914	1022	244	1176	1187	1630	869	-0.95424136	0.075014969	0.603900623
28040	'D6Wsu163e'	409	411	322	106	300	425	380.6666667	277	-0.508182528	0.249886511	0.870208199
280408	'Rilp'	60	39	22	5	55	24	40.33333333	28	-0.567863535	0.482430896	0.972790461
280411	'Lix1l'	1343	1293	2044	1232	1490	1696	1560	1472.666667	-0.07430138	0.857703691	0.976399524
28042	'Selenoi'	749	801	828	1154	950	926	792.6666667	1010	0.460086786	0.277038827	0.888870971
280621	'Selenov'	4	11	5	0	16	10	6.666666667	8.666666667	0.276026541	0.831851492	0.974620113
280635	'Emilin3'	803	756	55	30	872	532	538	478	-0.202379795	0.84853069	0.975686757
28064	'Yipf3'	736	769	1027	867	909	1111	844	962.3333333	0.223050456	0.570102808	0.972790461
280645	'B3gat2'	76.49	72.92	42.15	61.6	61.78	30.04	63.85333333	51.14	-0.158425412	0.783924295	0.972790461
280662	'Afm'	4	1	1	0	34	14	2	16	2.904612792	0.06281814	0.566066012
280667	'Adam1b'	4.53	8.67	0	1.32	9.4	0	4.4	3.573333333	-0.193326698	0.923730271	0.991932584
280668	'Adam1a'	107.47	132.33	31	66.68	188.6	65	90.26666667	106.76	0.324655836	0.624707547	0.972790461
28071	'Twistnb'	755	802	903	424	630	768	820	607.3333333	-0.433641239	0.177739754	0.797422443
28075	'Desi1'	735	720	1476	1459	647	861	977	989	0.145634128	0.824739317	0.972790461
28077	'Med10'	1391	1573	1640	1315	1518	1674	1534.666667	1502.333333	0.015205418	0.962665376	0.999493374
28080	'Atp5o'	1992	2322	1920	2350	1949	2681	2078	2326.666667	0.254484663	0.480689883	0.972790461
28081	'Fam104a'	696.76	676.45	1475.5	1036.7	719.66	915.79	949.5633333	890.7166667	-0.037138455	0.949146795	0.997742943
28084	'Vps25'	796	836	414	303	702	854	682	619.6666667	-0.132538992	0.739422529	0.972790461
28088	'Rtcb'	3203	3565	3475	2246	2992	3668	3414.333333	2968.666667	-0.179236619	0.525825457	0.972790461
28105	'Trim36'	50	59	47	36	35	29	52	33.33333333	-0.541195619	0.266592006	0.883600995
28106	'Mydgf'	339	388.98	594.99	515.96	485.99	612.99	440.99	538.3133333	0.31937581	0.491986399	0.972790461
28109	'D10Wsu102e'	2433	2708	1215	1100	2893	3078	2118.666667	2357	0.15964159	0.702118324	0.972790461
28113	'Tinf2'	977	976	442	389	695	928	798.3333333	670.6666667	-0.21859255	0.577995221	0.972790461
28114	'Nsun2'	2039	1928	5287	1312	1783	2395	3084.666667	1830	-0.819800909	0.160405614	0.771114735
28126	'Nop16'	886	859	1129	484	669	1052	958	735	-0.404797456	0.293771662	0.90029104
28135	'Cep63'	1207	1309	512	714	896	646	1009.333333	752	-0.291605541	0.511998004	0.972790461

28146	'Serp1'	2003	2023	11693	5721	2110	4587	5239.666667	4139.333333	-0.313384211	0.725331191	0.972790461
28169	'Agpat3'	1045	1132	735	388	1299	1585	970.6666667	1090.666667	0.122430984	0.786796329	0.972790461
28185	'Tomm70a'	3301	3490	3221	2318	2723	3526	3337.333333	2855.666667	-0.184018712	0.51023333	0.972790461
28193	'Reep3'	1415	1516	1125	1845	2024	2413	1352	2094	0.704572097	0.01205459	0.271542485
28194	'Apon'	2	5	0	2	3	4	2.333333333	3	0.46269857	0.766343128	0.972790461
28199	'Dcaf11'	1109	1077	1064	868	1194	1239	1083.333333	1100.333333	0.0584255	0.826766728	0.972859382
28200	'Dhrs4'	452	528	294	34	605	406	424.6666667	348.3333333	-0.379976207	0.626910425	0.972790461
28240	'Trpm2'	35	15	120	3	5	6	56.66666667	4.666666667	-3.718968183	1.78E-04	0.020741164
28248	'Slco1a1'	0	0	0	0	3	0	0	1	2.343657704	0.558557988	0.972790461
28250	'Slco1a4'	9	10	0	0	136	38	6.333333333	58	3.164763955	0.057830969	0.549741188
28253	'Slco1b2'	1	1	0	0	5	4	0.666666667	3	2.115481286	0.333426888	0.921648675
28254	'Slco1a6'	0	0	0	0	1	0	0	0.333333333	1.020273531	0.802557913	0.972790461
282619	'Sbsn'	449	372	761	1599	451	607	527.3333333	885.6666667	0.952280779	0.207275615	0.83479753
282663	'Serpinb1b'	5	6	6	8	7	3	5.666666667	6	0.212135876	0.832282874	0.974662707
28295	'Gatd3a'	725	712	876	302	713	861	771	625.3333333	-0.358892941	0.371474783	0.938527219
286940	'Flnb'	1843	1555	2395	3723	2437	2088	1931	2749.333333	0.645359377	0.241097082	0.86449675
286942	'Kif19a'	47	72	171	117	76	44	96.66666667	79	-0.185552213	0.814913394	0.972790461
29805	'Znhit2'	192	156	103	118	167	265	150.3333333	183.3333333	0.316140505	0.405417988	0.957157474
29806	'Limd1'	1169	1166	1323	786	1444	1329	1219.333333	1186.333333	-0.043452182	0.889977297	0.984980969
29807	'Tpk1'	326	341	365	140	350	382	344	290.6666667	-0.288202189	0.45564387	0.971239019
29808	'Mga'	5143	5352.2	3323	3770	4551	4413	4606.05	4244.666667	-0.027315757	0.927146675	0.992785839
29809	'Rabgap1l'	1753	1726	773	1111	1295	1162	1417.333333	1189.333333	-0.130652894	0.744032976	0.972790461
29810	'Bag3'	1180	1224	1660	1243	1432	1619	1354.666667	1431.333333	0.105506741	0.783345838	0.972790461
29811	'Ndrg2'	799	696	3308	4069	742	1503	1601	2104.666667	0.555476921	0.559710258	0.972790461
29812	'Ndrg3'	1041	1091	1450	3346	1409	1830	1194	2195	1.050644264	0.083480292	0.627571099
29813	'Zfp385a'	362	326	313	547	287	403	333.6666667	412.3333333	0.464046396	0.360010612	0.933706718
29815	'Bcar3'	378	392	32	179	645	612	267.3333333	478.6666667	0.879213853	0.275792346	0.887352188
29816	'Hip1r'	470	475	324	319	477	1064	423	620	0.536523106	0.221221084	0.849153089
29817	'Igfbp7'	376	375	1280	12463	543	1912	677	4972.666667	3.167600039	0.005460533	0.174480089
29818	'Hspb7'	16	15	37	9	7	3	22.66666667	6.333333333	-1.760342324	0.054791447	0.537233105
29819	'Stau2'	1044.8	1143.7	1275.7	197.42	912	1220.57	1154.74	776.6633333	-0.685561168	0.226540393	0.854001521
29820	'Tnfrsf19'	4403	4501	336	269	6952	6488	3080	4569.666667	0.527794406	0.612981891	0.972790461
29846	'Olfr156'	0	0	1	0	0	0	0.333333333	0	-0.903279821	0.824807108	0.972790461
29856	'Smtn'	721	643	310	1557	882	586	558	1008.333333	1.1103827	0.076759142	0.609792246
29857	'Mapk12'	102	116	64	123	128	113	94	121.3333333	0.486995092	0.24176851	0.86449675
29858	'Pmm1'	451	422	1119	1025	433	880	664	779.3333333	0.301811632	0.662493515	0.972790461
29859	'Sult4a1'	32	28	40	42	16	18	33.33333333	25.33333333	-0.209609034	0.781133366	0.972790461
29861	'Dpf1'	236.97	253	807	71	245	232.97	432.3233333	182.99	-1.398812932	0.04741742	0.50504201
29862	'Ninj2'	1	1	0	0	1	11	0.666666667	4	2.491657223	0.278032051	0.889355605
29863	'Pde7b'	671	665	301	144	369	251	545.6666667	254.6666667	-1.060013686	0.013409134	0.288713988

29864	'Rnf11'	1202	1190	2581	1657	1156	1695	1657.666667	1502.666667	-0.101567789	0.85950891	0.976863639
29865	'Cabp5'	5	8	0	1	4	2	4.333333333	2.333333333	-0.81281976	0.609674888	0.972790461
29866	'Cabp2'	0	1	0	2	0	0	0.333333333	0.666666667	1.337350669	0.73871782	0.972790461
29867	'Cabp1'	11	16	4	13	5	14	10.33333333	10.66666667	0.214682984	0.812749079	0.972790461
29869	'Ulk2'	2293	2312	1048	767	1862	1765	1884.333333	1464.666667	-0.340008022	0.395378573	0.954014376
29870	'Gtse1'	325	338	105	41	327	333	256	233.6666667	-0.178996278	0.802051793	0.972790461
29871	'Scmh1'	1740	1730	964	530	1524	1264	1478	1106	-0.415097393	0.289040512	0.896301537
29873	'Cspg5'	78	72	39	18	162	317	63	165.6666667	1.293297693	0.107125321	0.682129732
29875	'Iqgap1'	2621	2565	4115	7252	3275	4178	3100.333333	4901.666667	0.810827263	0.184353287	0.804780995
29876	'Clic4'	2439	2410	4224	1863	1937	2534	3024.333333	2111.333333	-0.510247795	0.26956102	0.883851074
29877	'Hdgfl3'	1410.2	1402	1455.9	880.91	1349	1623.02	1422.666667	1284.296667	-0.14253444	0.615621981	0.972790461
30044	'Opn4'	0	0	1	2	0	2	0.333333333	1.333333333	2.061542391	0.4933139	0.972790461
30045	'Dnajc12'	109	110	102	103	148	132	107	127.6666667	0.299384981	0.364070768	0.935240298
30046	'Zfp292'	3328	3242	3025	2841	3450	3205	3198.333333	3165.333333	0.051845268	0.860307407	0.976863639
30049	'Scd3'	11	17.8	7.03	8	8	9	11.94333333	8.333333333	-0.387482547	0.614154843	0.972790461
30050	'Fbxw2'	1663	1557	1241	969	1318	1664	1487	1317	-0.137297526	0.581357446	0.972790461
30051	'Spdef'	2	7	17	10	10	3	8.666666667	7.666666667	-0.126339165	0.913344629	0.989773583
30052	'Pcsk1n'	10	6	19	6	14	35	11.66666667	18.33333333	0.513627884	0.567918403	0.972790461
30054	'Rnf17'	2190	2240	650	7	1096	1382	1693.333333	828.3333333	-1.125755495	0.325430177	0.920269559
30055	'Timm13'	584	634	631	416	516	734	616.3333333	555.3333333	-0.131886903	0.674760963	0.972790461
30056	'Timm9'	205	240	258	140	225	323	234.3333333	229.3333333	-0.052704725	0.888163039	0.984534762
30057	'Timm8b'	548	568	908	405	476	599	674.6666667	493.3333333	-0.451386892	0.301157339	0.904375823
30058	'Timm8a1'	531	667	849	272	624	547	682.3333333	481	-0.548005409	0.190532959	0.813136091
30059	'Timm10'	173	183	211	83	169	244	189	165.3333333	-0.241802371	0.565051459	0.972790461
30060	'Meltf'	116	121	78	0	244	171	105	138.3333333	0.270810907	0.831033536	0.974324192
30785	'Cttnbp2'	504	492	240	185	846	681	412	570.6666667	0.443266909	0.397347214	0.954563638
30791	'Slc39a1'	1708	1604	1949	3067	2392	3242	1753.666667	2900.333333	0.8062912	0.050528383	0.519548742
30794	'Pdlim4'	502	522	1032	534	667	709	685.3333333	636.6666667	-0.117597981	0.814232095	0.972790461
30795	'Fkbp3'	1433	1451.8	1453.1	702.29	1362.8	1562.84	1445.94	1209.296667	-0.275340795	0.36857778	0.937530612
30800	'Mmp20'	0	0	1	0	0	0	0.333333333	0	-0.903279821	0.824807108	0.972790461
30805	'Slc22a4'	19	25	17	16	16	12	20.33333333	14.66666667	-0.366820673	0.559189587	0.972790461
30806	'Adamts8'	55	69	8	24	85	102	44	70.33333333	0.696184675	0.376663708	0.941504733
30838	'Fbxw4'	195	200	220	30	259	211	205	166.6666667	-0.416726746	0.526740356	0.972790461
30839	'Fbxw5'	805	744	424	533	835	907	657.6666667	758.3333333	0.259249719	0.402501201	0.955996639
30840	'Fbxl6'	469	437	258	362	478	559	388	466.3333333	0.332490909	0.284127826	0.892640391
30841	'Kdm2b'	1696	1682	1089	350	1205	1335	1489	963.3333333	-0.667629344	0.13387828	0.733301819
30843	'Fbxl12'	362.01	354.73	866.43	264.17	381.15	357.89	527.7233333	334.4033333	-0.695052697	0.214191027	0.842553243
30853	'Mlf2'	2161	2261	2904	2713	2351	3412	2442	2825.333333	0.25911395	0.526737295	0.972790461
30877	'Gnl3'	959	948	3096	519	798	1243	1667.666667	853.3333333	-1.076854784	0.100925699	0.671759705
30878	'Apln'	551	421	1028	69	660	1464	666.6666667	731	-0.08190695	0.92452467	0.992176833

30923	'Foxe3'	1	0	0	0	1	1	0.333333333	0.666666667	0.817432597	0.823901819	0.972790461
30924	'Angptl3'	0	0	0	0	1	2	0	1	2.297812331	0.566584047	0.972790461
30925	'Slamf6'	0	0	0	0	2	1	0	1	2.320886	0.562538074	0.972790461
30926	'Glrx3'	2367	2274	3550	909	2077	2505	2730.333333	1830.333333	-0.650008835	0.145567081	0.753611197
30927	'Snai3'	17	15	1	5	22	39	11	22	0.992429769	0.344888229	0.926100052
30928	'Zbtb18'	1469	1474	1710	727	1205	1691	1551	1207.666667	-0.387228598	0.268613514	0.883600995
30930	'Vps26a'	3110	2806	3315	1878	2315	3468	3077	2553.666667	-0.261351431	0.434895791	0.965608974
30931	'Tor1a'	250	275	252	439.93	381	435	259	418.6433333	0.787147702	0.03898698	0.461532041
30932	'Zfp330'	805	851	2556	473	735	989	1404	732.3333333	-1.03395476	0.102409652	0.671776916
30933	'Tor2a'	315	319	187	378	442.98	438	273.6666667	419.66	0.71124919	0.032743193	0.436246529
30934	'Tor1b'	488	516	332	552.07	719	579	445.3333333	616.69	0.559902724	0.084752308	0.633355364
30935	'Tor3a'	117	122	139	288	192	329	126	269.6666667	1.179051055	0.010742721	0.255940608
30936	'Slc46a2'	2	3	0	2	1	1	1.666666667	1.333333333	-0.085227903	0.964181496	0.999493374
30937	'Lmcd1'	176	171	80	325	287	110	142.3333333	240.6666667	0.975066591	0.125515017	0.72022881
30938	'Fgd3'	16	22	83	180	37	109	40.33333333	108.6666667	1.534829264	0.10750424	0.683111836
30939	'Pttg1'	206	211	91	431	329	413	169.3333333	391	1.363165917	0.003669325	0.139157376
30940	'Usp25'	957	876	1738	1351	986	1436	1190.333333	1257.666667	0.123604369	0.817203759	0.972790461
30941	'Usp21'	599	690	518	530	861	780	602.3333333	723.6666667	0.303680338	0.237207164	0.862621002
30942	'Hnf4g'	0	0	0	0	1	0	0	0.333333333	1.020273531	0.802557913	0.972790461
30943	'Prss30'	0	0	2	0	0	0	0.666666667	0	-1.995285526	0.619817042	0.972790461
30944	'Zfp354c'	1101	1051	710	803	1100	941	954	948	0.069775251	0.813342169	0.972790461
30945	'Rnf19a'	2867	2702	4768	1361	2107	2249	3445.666667	1905.666667	-0.882249086	0.043555374	0.486816449
30946	'Abt1'	414	418	543	274	362	514	458.3333333	383.3333333	-0.26558207	0.480259562	0.972790461
30947	'Adat1'	408.98	348	137	170	395.98	305	297.9933333	290.3266667	0.014474892	0.975243754	0.999493374
30948	'Bin1'	889	904	1888	1229	1003	1082	1227	1104.666667	-0.104163168	0.852442492	0.975734242
30949	'Lcmt1'	564	590	488	500	609	835	547.3333333	648	0.282600209	0.308343416	0.908027422
30951	'Cbx8'	165	203	278	92	182	204	215.3333333	159.3333333	-0.481580901	0.278147427	0.889355605
30952	'Cngb3'	1	0	0	2	0	1	0.333333333	1	1.739847053	0.590374857	0.972790461
30953	'Schip1'	206	215	497	238	248	269	306	251.6666667	-0.281830701	0.621733351	0.972790461
30954	'Siva1'	477	503	781	290	395	467	587	384	-0.624809078	0.140689177	0.744590744
30955	'Pik3cg'	72	52	11	6	146	50	45	67.33333333	0.548294913	0.585136447	0.972790461
30956	'Aass'	123	142	14	78	184	276	93	179.3333333	0.990356372	0.196833384	0.822696823
30957	'Mapk8ip3'	2916	2844	3685	4399	2853	2274	3148.333333	3175.333333	0.163490534	0.76523087	0.972790461
30959	'Ddx25'	536	520	127	28	330	491	394.3333333	283	-0.54298388	0.52267067	0.972790461
30960	'Vapa'	2639	2727	7627	3139	2438	4153	4331	3243.333333	-0.431983813	0.495492726	0.972790461
30962	'Slc7a9'	14	14	0	0	18	9	9.333333333	9	-0.077926593	0.963579075	0.999493374
30963	'Hacd1'	306	352	621	105	327	433	426.3333333	288.3333333	-0.687346665	0.23161292	0.858905111
317652	'Klk15'	0	3	0	0	2	2	1	1.333333333	0.389931636	0.893753461	0.985171
317653	'Klk14'	0	0	1	1	0	0	0.333333333	0.333333333	0.058500858	0.988561293	0.999493374
317677	'C1s2'	65.2	57	244.36	4.01	3	4	122.1866667	3.67	-5.097404183	2.38E-08	1.51E-05

317717	'Sec22a'	630	653	347	209	634	759	543.3333333	534	-0.047056863	0.916498255	0.990526893
317750	'Slc24a5'	36.85	25.83	21.43	9.92	48.07	35.24	28.03666667	31.07666667	0.122464403	0.851016873	0.975734242
317755	'Zar1'	2	1	0	0	2	3	1	1.666666667	0.695336772	0.761274171	0.972790461
317757	'Gimap5'	35.15	40.65	7.61	37	32	27	27.80333333	32	0.420669109	0.570549749	0.972790461
317758	'Gimap9'	95	90	84	17	90	61	89.66666667	56	-0.740104856	0.185424338	0.805706284
319146	'Ifnz'	0	0	0	1.1	1	3.79	0	1.963333333	3.168569389	0.291679716	0.89958198
319148	'H3c3'	2	3.17	5.44	0	1	1	3.536666667	0.666666667	-2.407152801	0.178502565	0.798139878
319149	'H3c4'	0	5.75	2.55	5	1.31	1.2	2.766666667	2.503333333	0.295319941	0.871090924	0.980126495
319150	'H3c2'	0	1.25	2.67	0	0	0	1.306666667	0	-2.490290069	0.532417892	0.972790461
319151	'H3c6'	2	3.03	11.2	1	6	9	5.41	5.333333333	-0.189286873	0.881665314	0.982528614
319152	'H3c10'	1	4.25	14.88	0	2.69	5.22	6.71	2.636666667	-1.686253405	0.296534006	0.90029104
319153	'H3c11'	0	1.42	5.81	5	0	4.88	2.41	3.293333333	0.642056104	0.758776348	0.972790461
319154	'H3c13'	2.7	0	3.71	0	0.99	0	2.136666667	0.33	-3.215996145	0.397756436	0.954563638
319155	'H4c3'	2	1.03	14.64	0	3.15	4	5.89	2.383333333	-1.546666667	0.356178982	0.931977458
319156	'H4c4'	4	6	24	0	3	6	11.33333333	3	-2.151812952	0.117329389	0.705393896
319157	'H4c6'	1	1	1	1	1	1	1	1	0.063933044	0.973473023	0.999493374
319158	'H4c9'	25	29	47.36	43	18	16	33.78666667	25.66666667	-0.218534677	0.783010139	0.972790461
319159	'H4c11'	1.06	3.08	2.05	9	2.07	1	2.063333333	4.023333333	1.30644802	0.391327857	0.952371379
319160	'H4c12'	14.99	16.46	14.34	58	16.6	11	15.26333333	28.53333333	1.248549479	0.18111032	0.801692526
319161	'H4c18'	8.91	16.22	47.65	2.48	4.5	9.99	24.26	5.656666667	-2.391377153	0.021280068	0.361918385
319162	'H2aw'	109.67	105.77	340.77	48.42	143.6	116.29	185.4033333	102.77	-0.981897878	0.155794424	0.766832248
319163	'H2ac1'	1	2	1	0	0	1	1.333333333	0.333333333	-1.817629741	0.480898329	0.972790461
319164	'H2ac6'	1.07	0	6.89	3	5.15	3.16	2.653333333	3.77	0.560866205	0.73118419	0.972790461
319165	'H2ac7'	7.74	7.08	9.3	0.14	3.02	6.72	8.04	3.293333333	-1.476006565	0.227258159	0.854001521
319166	'H2ac8'	12.6	8.48	53.62	0	11.25	13.85	24.9	8.366666667	-1.872200106	0.143579185	0.750131209
319167	'H2ac11'	11.03	7.91	13.39	1	7.02	4.94	10.77666667	4.32	-1.44019338	0.151567367	0.760516968
319168	'H2ac12'	2.85	4.27	0	2.86	2.69	1.41	2.373333333	2.32	-0.063194309	0.971741457	0.999493374
319169	'H2ac15'	1	0	6.1	0	0	4	2.366666667	1.333333333	-1.129336693	0.679440011	0.972790461
319170	'H2ac22'	3.76	13.56	8.28	2	2.4	2.18	8.533333333	2.193333333	-1.939868924	0.100938829	0.671759705
319171	'H2ac24'	1.56	0.69	9.97	0	0	0	4.073333333	0	-4.306687891	0.186293854	0.807528217
319172	'H2ac4'	1.13	1.23	2.66	0	2.76	1.08	1.673333333	1.28	-0.534757164	0.793409317	0.972790461
319173	'H2ac10'	1.15	0	0	0	0	0	0.383333333	0	-0.903279821	0.824807108	0.972790461
319176	'H2ac20'	0	0	0	0	1	0	0	0.333333333	1.020273531	0.802557913	0.972790461
319177	'H2bc1'	4	3	4	0	0	0	3.666666667	0	-4.275757269	0.057561231	0.548823528
319178	'H2bc3'	0	0	4	2	2	0	1.333333333	1.333333333	-0.002914312	0.99917275	0.999900097
319179	'H2bc6'	3.28	6.09	5.5	4	3	10.07	4.956666667	5.69	0.284563604	0.787562618	0.972790461
319180	'H2bc7'	1.88	1.03	1.23	0	3	3	1.38	2	0.891086014	0.643851115	0.972790461
319181	'H2bc8'	3.6	2.43	3.87	2.38	4.68	0	3.3	2.353333333	-0.340386592	0.833642659	0.974723675
319182	'H2bc9'	1.01	0	0	2	0	1	0.336666667	1	1.739847053	0.590374857	0.972790461
319183	'H2bc11'	5.11	4.54	6.62	0	1	2	5.423333333	1	-2.404177712	0.111646347	0.695712994

319184	'H2bc12'	13.48	7.13	10.07	1.24	0	4	10.22666667	1.746666667	-2.598963456	0.043263805	0.484697385
319185	'H2bc13'	1.03	1.53	0.89	0	1	0	1.15	0.333333333	-0.74517548	0.838722495	0.974723675
319186	'H2bc14'	1.01	1	1.01	1	1	2	1.006666667	1.333333333	0.438882115	0.810470176	0.972790461
319187	'H2bc15'	0	3.02	3.38	4	0	1	2.133333333	1.666666667	0.008939685	0.996726882	0.999616639
319188	'H2bc22'	1.35	0.9	2.89	2	0	0	1.713333333	0.666666667	-0.277686711	0.928336246	0.993096978
319189	'H2bc18'	7	6	7	1	0	0	6.666666667	0.333333333	-4.171291356	0.015389874	0.308641642
319190	'H2bc21'	306	294	94	59	483	435	231.3333333	325.6666667	0.449202625	0.527281593	0.972790461
319191	'H2ac13'	9.09	8.74	22.99	0	3.46	10.27	13.60666667	4.576666667	-1.790695033	0.152028897	0.762229272
319192	'H2ac19'	22.13	20.3	15.14	0	5.9	16.87	19.19	7.59	-1.559424621	0.152620077	0.763180669
319195	'Rpl17'	12744	12781	23870	15885	8962.8	12542.53	16464.74	12463.26667	-0.310694509	0.591601028	0.972790461
319196	'Ankef1'	48	53	4	2	14	16	35	10.66666667	-1.708288825	0.096560843	0.663197379
319197	'Gpr4'	13	18	8	80	23	20	13	41	1.97504794	0.026988329	0.403699531
319200	'Gpr82'	0	2	1	1	0	7	1	2.666666667	1.340499287	0.55615398	0.972790461
319207	'Pgbd1'	273	279	378	18	182	114	310	104.6666667	-1.697421578	0.014506742	0.299476615
319211	'Nol4'	44	59	18	10	36	61	40.33333333	35.66666667	-0.202489801	0.773054622	0.972790461
319217	'Vmn2r7'	0	0	0	0	1	1	0	0.666666667	1.775692139	0.660844521	0.972790461
319229	'Sctr'	1	0	2	5	0	0	1	1.666666667	1.0865872	0.702593194	0.972790461
319236	'Trim12c'	323	310.15	117.6	218.97	538.86	532.03	250.25	429.9533333	0.809003493	0.081096412	0.618950211
319239	'Npsr1'	10	11	3	0	6	7	8	4.333333333	-0.937686396	0.454656291	0.970682247
319259	'Bricd5'	0	1.09	0	3.33	0	1.07	0.363333333	1.466666667	2.206981601	0.470577023	0.972790461
319262	'Fchsd1'	397.32	383.34	323.14	454.67	460.1	407.35	367.9333333	440.7066667	0.364282615	0.316228312	0.914949423
319263	'Pcmtd1'	2380	2540	2573	2503	3564	2634	2497.666667	2900.333333	0.26771556	0.42564538	0.960632713
319266	'A130010J15Rik'	338.93	342.96	197.94	98	248.77	279.99	293.2766667	208.92	-0.492486712	0.211503217	0.840335617
319277	'Washc4'	1457	1468.7	1396.3	1651.6	1488.6	1540.72	1440.686667	1560.303333	0.214488142	0.561488124	0.972790461
319278	'Shf1'	196.55	179.76	163.93	631.79	205.13	280.51	180.08	372.4766667	1.293873382	0.049401916	0.515210762
319293	'Gpr141b'	0	0	0	0	2	1	0	1	2.320886	0.562538074	0.972790461
319322	'Sf3b2'	4853	4804	4981	3526	4622	4856	4879.333333	4334.666667	-0.133550053	0.635267011	0.972790461
319352	'Pianp'	434.01	389	122	48	78	69	315.0033333	65	-2.182200894	2.11E-05	0.004291
319370	'Ubald2'	386	374	2209	612	472	604	989.6666667	562.6666667	-0.8493802	0.308434711	0.908155602
319387	'Adgrl3'	1967	1825	345	2288	1877	1468	1379	1877.666667	0.66882038	0.315158116	0.914723408
319415	'Hs3st5'	13	5	131	10	8	19	49.66666667	12.33333333	-2.155837525	0.072360484	0.595548709
319430	'C5ar2'	1	0	0	3	1	6	0.333333333	3.333333333	3.266198883	0.125938527	0.72022881
319433	'Serpine3'	1	0	1	0	1	0	0.666666667	0.333333333	-0.894008961	0.803817041	0.972790461
319446	'Dpep2'	3	6	5	3	6	4	4.666666667	4.333333333	-0.088460198	0.932811994	0.993970337
319448	'Fndc3a'	2707	2623	8082	1120	2516	2710	4470.666667	2115.333333	-1.199121351	0.058381549	0.551665011
319455	'Pld5'	496.45	509.52	484.31	253.8	510.88	434.37	496.76	399.6833333	-0.310974824	0.301134347	0.904375823
319468	'Ppm1h'	161.49	163.22	214.81	416.27	170.69	464.1	179.84	350.3533333	1.059429751	0.068334396	0.583635907
319475	'Zfp672'	1380	1444	389	914	1397	787	1071	1032.666667	0.086062219	0.8751826	0.980678781
319476	'Lrtm1'	7	3	1	5	11	3	3.666666667	6.333333333	0.897089392	0.452703041	0.970649024
319477	'Insyn1'	485	576	654	141	355	502	571.6666667	332.6666667	-0.854578029	0.059732439	0.554643744

319478	'Cxxc4'	345	306	96	263	396	328	249	329	0.511660171	0.305348436	0.907188578
319480	'Itgal1'	217	186	135	121	172	147	179.3333333	146.6666667	-0.220280611	0.505028823	0.972790461
319481	'Wdr59'	848	798	909	1125	760	917	851.6666667	934	0.252402606	0.580003425	0.972790461
319482	'9530053A07Rik'	0	1	1	0	1	0	0.666666667	0.333333333	-0.886945817	0.805479824	0.972790461
319486	'A430057M04Rik'	21	32	33	24	29	13	28.66666667	22	-0.296147738	0.654299835	0.972790461
319504	'Nrcam'	81	74	39	1	48	61	64.66666667	36.66666667	-0.925360273	0.34175364	0.925308103
319508	'Syt15'	0	1	0	0	0	1	0.333333333	0.333333333	0.058500858	0.988561293	0.999493374
319513	'Pced1a'	763.86	728.2	394.56	137.7	620.32	491.6	628.8733333	416.54	-0.624828356	0.218781108	0.845396589
319518	'Pdpr'	2560	2488	1213	1260	1958	2308	2087	1842	-0.12392058	0.718711153	0.972790461
319520	'Dusp4'	84	91	1843	1030	83	493	672.6666667	535.3333333	-0.244092854	0.850734079	0.975734242
319530	'Zfp750'	14	19	3	1	12	10	12	7.666666667	-0.662037521	0.537298967	0.972790461
319535	'Zfp182'	384	413	199	131	649	306	332	362	0.114042236	0.837706507	0.974723675
319552	'Spx'	0	3	7	4	10	6	3.333333333	6.666666667	0.908049952	0.508209936	0.972790461
319554	'Idi1'	798	862	776	535	755	549	812	613	-0.343055737	0.299070857	0.903100471
319555	'Nwd1'	25	14	11	111	22	31	16.66666667	54.66666667	2.04138361	0.024532724	0.387844278
319565	'Syne2'	5098	4572	3328	11261	7409	6945	4332.666667	8538.333333	1.160502093	0.016476314	0.321279678
319579	'Defb20'	1	0	2	0	1	0	1	0.333333333	-1.527647908	0.642855063	0.972790461
319581	'Xkr5'	43	33	6	6	38	20	27.33333333	21.33333333	-0.340892325	0.700074108	0.972790461
319582	'Trmt9b'	79	93	40	45	105	72	70.66666667	74	0.113397114	0.807747702	0.972790461
319583	'Lig4'	288	309	114	256	260	234	237	250	0.225362495	0.633946145	0.972790461
319586	'Celf5'	18	16	4	6	17	6	12.66666667	9.666666667	-0.308719623	0.731430266	0.972790461
319594	'Hif1an'	2223	2235	1154	1373	2021	2629	1870.666667	2007.666667	0.15273988	0.644102514	0.972790461
319601	'Zfp653'	426	449	299	50	399	239	391.3333333	229.3333333	-0.839520233	0.174541646	0.793295795
319604	'Fam168a'	2210	2142	2185	2033	1744	1668	2179	1815	-0.153289062	0.703922896	0.972790461
319613	'Sybu'	368	457	184	195	666	607	336.3333333	489.3333333	0.535952432	0.267988353	0.883600995
319615	'Zfp944'	532	553	327	414	613	398	470.6666667	475	0.106527185	0.773193267	0.972790461
319618	'Dcp1b'	239	230	188	124	253	207	219	194.6666667	-0.151010907	0.625473911	0.972790461
319622	'Itprip12'	678	619	1120	3217	861	1313	805.6666667	1797	1.364046513	0.065658152	0.574593252
319625	'Galm'	189	157	264	534	201	333	203.3333333	356	0.962648943	0.133476268	0.733301819
319634	'Efcab5'	112	105	22	5	55	21	79.66666667	27	-1.566325715	0.071059287	0.591632421
319636	'Fsd1l'	978	990	504	346	744	605	824	565	-0.503702031	0.159551951	0.771073131
319638	'Nt5dc1'	152	168	143	79	157	165	154.3333333	133.6666667	-0.20777651	0.524622524	0.972790461
319642	'Rab9b'	24	23	34	2	12	11	27	8.333333333	-1.782123578	0.019092583	0.344064642
319651	'Usp37'	2226	2310	1443	1282	1597	1794	1993	1557.666667	-0.282570762	0.307099353	0.907830798
319653	'Slc25a40'	1120.1	1198.3	478	1536.5	1771.9	959.17	932.1033333	1422.54	0.774354089	0.128344673	0.724799196
319655	'Podxl2'	712	750	266	114	734	581	576	476.3333333	-0.307364733	0.626322416	0.972790461
319660	'Agmo'	1	1	0	8	1	4	0.666666667	4.333333333	2.993186344	0.101500434	0.671759705
319670	'Eml5'	1099.8	1073	223.82	351.46	627.53	472.31	798.8733333	483.7666667	-0.61564446	0.290885197	0.898944955
319675	'Cep295'	1472	1582	515	450	1192	882	1189.666667	841.3333333	-0.455059833	0.348752957	0.928640228
319695	'Ankar'	14	16	1	1	10	2	10.33333333	4.333333333	-1.211538418	0.360230051	0.933706718

319701	'Fbxo48'	258	284	35	45	172	102	192.3333333	106.3333333	-0.80306798	0.285953946	0.893820325
319710	'Frmd6'	1981	1803	1505	2157	1568	1634	1763	1786.333333	0.165031611	0.701742729	0.972790461
319713	'Ablim3'	313	307	155	116	669	352	258.3333333	379	0.526320384	0.363288139	0.935012144
319719	'Simc1'	821	817	436	270	641	514	691.3333333	475	-0.513210298	0.161168479	0.771435531
319734	'Cacna2d4'	1	0	2	1	0	0	1	0.333333333	-1.526634549	0.634193714	0.972790461
319740	'Zfyve27'	1227	1237	393	700	1076	893	952.3333333	889.6666667	0.007005199	0.988051147	0.999493374
319742	'Mpzl3'	110	115	58	112	105	114	94.33333333	110.3333333	0.349860321	0.417105138	0.957382938
319748	'Zfp865'	693	656	483	500	655	575	610.6666667	576.6666667	-0.005437245	0.985217626	0.999493374
319757	'Smo'	2738	2870	1280	430	3686	3165	2296	2427	0.017070326	0.979436779	0.999493374
319758	'Rfx7'	2240	2372	1246	912	2141	1630	1952.666667	1561	-0.291260317	0.416511674	0.95722888
319764	'A730046J19Rik'	17	26	2	0	39	40	15	26.33333333	0.754026462	0.584312307	0.972790461
319765	'Igf2bp2'	2754	2606	6265	2101	2375	3273	3875	2583	-0.618292045	0.258810565	0.877564912
319767	'Atp10b'	4	8	0	10	26	58	4	31.33333333	2.973624889	0.005610443	0.177185604
319772	'C130050018Rik'	5	8	0	16	5	11	4.333333333	10.66666667	1.571800192	0.21467428	0.842635023
319776	'Tmem72'	13	20	12	2	15	17	15	11.33333333	-0.47022527	0.573518582	0.972790461
319800	'Slc22a30'	0	0	1	0	1	0	0.333333333	0.333333333	0.058500858	0.988561293	0.999493374
319801	'Tigar'	223.97	225.61	116.04	494.38	299.91	373.01	188.54	389.1	1.232225161	0.014607903	0.300778621
319804	'Glt1d1'	41	51	268	5	83	78	120	55.33333333	-1.385601878	0.18613374	0.807018167
319807	'Nwd2'	47.49	63.82	16.11	44.39	57.92	34.82	42.47333333	45.71	0.246931235	0.693590404	0.972790461
319817	'Rc3h2'	1848	1870	1636	1278	1670	1344	1784.666667	1430.666667	-0.248245027	0.418780881	0.958308933
319822	'Smyd4'	561.88	555.99	115	116	342	352.92	410.9566667	270.3066667	-0.571326444	0.357674313	0.932505998
319832	'Tmem229a'	35	24	197	0	16	12	85.33333333	9.333333333	-3.470813111	0.004485732	0.157418103
319845	'Bbs9'	416	367	106	222	411	385	296.3333333	339.3333333	0.273352343	0.595898025	0.972790461
319848	'Slc17a4'	2	4	1	1	1	2	2.333333333	1.333333333	-0.735527764	0.644457864	0.972790461
319875	'Tmprss11b'	0	0	0	0	2	1	0	1	2.320886	0.562538074	0.972790461
319876	'Cobll1'	548	525	376	3580	356	1224	483	1720	2.164762876	0.012439655	0.276611367
319880	'Tmcc3'	333	291	90	76	304	649	238	343	0.482242037	0.498476797	0.972790461
319885	'Zcchc7'	949	945	1178	559	961	691	1024	737	-0.460179755	0.212294668	0.84083615
319887	'E030030I06Rik'	52.88	47.67	53.66	174.84	110.12	78	51.40333333	120.9866667	1.42666852	0.018652247	0.340229851
319888	'Oacyl'	6	15	5	11	11	12	8.666666667	11.33333333	0.49443304	0.544209338	0.972790461
319899	'Dock6'	803	713	237	549	903	761	584.3333333	737.6666667	0.431352165	0.362716876	0.934677306
319901	'Dsel'	715	759	555	263	770	715	676.3333333	582.6666667	-0.239180593	0.523185982	0.972790461
319909	'Ism1'	51	77	25	22	51	45	51	39.33333333	-0.33134109	0.542415417	0.972790461
319922	'Vwc2'	7	7	6	1	5	4	6.666666667	3.333333333	-1.027218327	0.327107994	0.920734892
319924	'Apba1'	191	174	148	66	112	141	171	106.3333333	-0.67496502	0.037283162	0.459930654
319930	'Ceacam19'	0	1	2	1	1	1	1	1	-0.008673646	0.996763263	0.999616639
319934	'Sbf2'	2424	2346	2463	2863	2541	2680	2411	2694.666667	0.254895957	0.502267469	0.972790461
319939	'Tns3'	7788	8154	7551	24628	10229	12925	7831	15927.33333	1.23012462	0.034491804	0.445535842
319942	'A530016L24Rik'	46	73	16	39	32	98	45	56.33333333	0.396792482	0.56545525	0.972790461
319944	'Taf2'	1925	1918	759	1570	1803	1645	1534	1672.666667	0.254352971	0.552671092	0.972790461

319945	'Flad1'	401	408	218	116	462	383	342.3333333	320.3333333	-0.118797777	0.805288833	0.972790461
319953	'Ttll1'	218	224	174	447	372	393	205.3333333	404	1.099171373	0.005701975	0.178006489
319955	'Ercc6'	1411	1368	477	519	1183	956	1085.333333	886	-0.23816263	0.606451586	0.972790461
319965	'Cc2d1b'	807	905	425	302	1009	862	712.3333333	724.3333333	0.018515105	0.967115685	0.999493374
319974	'Auts2'	1074	975	1487	534	947	1147	1178.666667	876	-0.468757071	0.243372186	0.86506088
319984	'Jph4'	47	38	26	73	28	17	37	39.33333333	0.378263121	0.640480565	0.972790461
319991	'Kif6'	33	40	9	23	34	38	27.33333333	31.66666667	0.301913216	0.63533326	0.972790461
319996	'Golm2'	1129.5	1127.9	1133	1665	2047	2597	1130.126667	2103	0.928567028	0.00114931	0.070943768
319997	'A630001G21Rik'	30	25	4	4	19	18	19.66666667	13.66666667	-0.509145586	0.575591174	0.972790461
319998	'Tmem198'	66	68	41	55	64	21	58.33333333	46.66666667	-0.163463264	0.7970051	0.972790461
320007	'Sidt1'	1	4	1	0	1	1	2	0.666666667	-1.586079421	0.441578869	0.968551476
320011	'Uggt1'	1912	1851	1178	3487	2276	2580	1647	2781	0.927864636	0.044252081	0.490480355
320022	'Terb1'	1009	1055	246	3	444	361	770	269.3333333	-1.591953677	0.156657853	0.768046725
320024	'Nceh1'	122	116	89	718	179	396	109	431	2.226380366	0.001129174	0.070791417
320027	'Fstl4'	25	20	4	6	28	51	16.33333333	28.33333333	0.767415514	0.395930259	0.954181778
320040	'Rnf222'	9	12	5	5	17	10	8.666666667	10.66666667	0.319861162	0.69650541	0.972790461
320051	'Exph5'	205	243	363	398	281	221	270.3333333	300	0.26889969	0.642990031	0.972790461
320078	'Olfml2b'	391	340	459	2740	359	856	396.6666667	1318.333333	2.021831873	0.017113906	0.32733359
320080	'Zbtb39'	703	692	573	680	677	781	656	712.6666667	0.204501503	0.51867199	0.972790461
320082	'Fbxw21'	2	0	1	0	2	8	1	3.333333333	1.577467615	0.4632797	0.972790461
320083	'Fbxw16'	0	0	0	0	0	2	0	0.666666667	1.749449488	0.66566965	0.972790461
320091	'Ano4'	49	58	36	23	113	96	47.66666667	77.33333333	0.649688254	0.255114279	0.874703817
320095	'6430550D23Rik'	88	71	6	35	76	35	55	48.66666667	-0.047111283	0.956702619	0.999493374
320100	'Relt'	122	149	66	217	84	67	112.3333333	122.6666667	0.421244034	0.560064112	0.972790461
320106	'Slc38a11'	0	0	0.93	36	0	0	0.31	12	6.512778158	0.044295982	0.490480355
320111	'Prr18'	27	40.91	54.87	97.12	31.76	12	40.92666667	46.96	0.481092037	0.611611256	0.972790461
320116	'Fndc9'	18.76	7.51	14.77	16.01	10.5	1.58	13.68	9.363333333	-0.305435197	0.786729115	0.972790461
320118	'Fbxl13'	14	26	2	1	13	5	14	6.333333333	-1.128911499	0.343402094	0.925308103
320119	'Rps6kc1'	572	591	654	266	630	799	605.6666667	565	-0.154006016	0.697610158	0.972790461
320127	'Dgki'	27	25	10	6	19	6	20.66666667	10.33333333	-0.940125957	0.236019863	0.861764862
320129	'Grk3'	437	398	235	83	414	209	356.6666667	235.3333333	-0.620490717	0.252102236	0.871252558
320135	'BC049715'	12.34	31.77	7	0	28.86	9.26	17.03666667	12.70666667	-0.495082756	0.698375466	0.972790461
320139	'Ptpn7'	16	22	3	9	28	12	13.66666667	16.33333333	0.333182816	0.706573606	0.972790461
320145	'Sp8'	6	4	4	2	3	2	4.666666667	2.333333333	-0.944072293	0.419660509	0.958308933
320148	'B430306N03Rik'	81.49	61	10	2	42	75	50.83	39.66666667	-0.415246619	0.69611688	0.972790461
320150	'Zdhhc17'	883	902	615	652	1019	791	800	820.6666667	0.102052681	0.727802073	0.972790461
320158	'Zmat4'	19	25	1	6	14	11	15	10.33333333	-0.441749936	0.673835283	0.972790461
320159	'Togaram2'	13	11	15	19	17	15	13	17	0.470512166	0.48883612	0.972790461
320162	'Cep95'	566.42	536.23	557.25	327.19	474	390.23	553.3	397.14	-0.434631711	0.165892106	0.780651734
320165	'Tacc1'	1523	1394	1844	3659	1500	1680	1587	2279.666667	0.718395803	0.25102754	0.871167957

320181	'Fndc7'	15	12	3	7	7	8	10	7.333333333	-0.320193321	0.721537612	0.972790461
320183	'Msrb3'	459	408	253	536	488	661	373.3333333	561.6666667	0.694562512	0.059119766	0.552865869
320184	'Lrrc58'	3102.1	3239	5484.1	3039	3535	3760	3941.713333	3444.666667	-0.183016372	0.686180974	0.972790461
320191	'Hook3'	2024.4	2152	1952.5	1040	2256.1	2205.33	2042.966667	1833.82	-0.168542379	0.573388975	0.972790461
320202	'Lefty2'	3	1	57	0	1	0	20.33333333	0.333333333	-5.967179489	0.003665972	0.139157376
320204	'Etfbkmt'	820	937	115	112	907	618	624	545.6666667	-0.199370956	0.807755082	0.972790461
320207	'Pik3r5'	21	15	111	68	19	44	49	43.66666667	-0.107374223	0.915590493	0.990380433
320208	'Tmem91'	27	24	1	21	13	4	17.33333333	12.66666667	-0.128720528	0.913843315	0.989964366
320209	'Ddx11'	442	445	99	361	538	285	328.6666667	394.6666667	0.416564631	0.493964367	0.972790461
320213	'Senp5'	1421	1451	1365	1006	1397	1559	1412.333333	1320.666667	-0.066164668	0.79566778	0.972790461
320214	'Maats1'	15	15	16	6	7	4	15.33333333	5.666666667	-1.366093	0.080041658	0.617305615
320225	'Catsperg1'	73.99	84.21	6.01	28	58	26.11	54.73666667	37.37	-0.403649817	0.645185819	0.972790461
320226	'Ccdc171'	340	381	165	385	647	490	295.3333333	507.3333333	0.862231654	0.026312336	0.397367216
320234	'Ccdc66'	524.85	579.7	426.3	158.12	621.07	538.01	510.2833333	439.0666667	-0.262657626	0.561682477	0.972790461
320237	'Smim10l2a'	77	81	35	4	107	71	64.33333333	60.66666667	-0.165511271	0.853219824	0.975734242
320244	'Ttll5'	906.39	861.28	304	283	581	713.03	690.5566667	525.6766667	-0.356024754	0.447969215	0.970198456
320253	'Marchf3'	101	107	167	32	162	109	125	101	-0.414579357	0.492806459	0.972790461
320256	'Dlec1'	54	51	15	26	61.99	46	40	44.66333333	0.208906728	0.725257859	0.972790461
320265	'Tafa1'	10	5	1	0	8	13	5.333333333	7	0.326366372	0.823478577	0.972790461
320267	'Fubp3'	1611	1632	2111	1097	1424	1511	1784.666667	1344	-0.389796814	0.27122094	0.884312405
320271	'Scai'	1194	1268	515	1604	1732	1128	992.3333333	1488	0.746272709	0.12157074	0.712603747
320277	'Spef2'	26	28	7	7	46	61	20.33333333	38	0.86263671	0.299332349	0.903198439
320292	'Rasgef1b'	461	517	786	2177	650	780	588	1202.333333	1.250520355	0.088042693	0.642559569
320299	'Iqcb1'	3741	3563	2284	345	1540	1173	3196	1019.333333	-1.686668748	4.67E-04	0.042077778
320302	'Glt28d2'	50	46	36	198	61	67	44	108.6666667	1.579303909	0.033005379	0.438197118
320309	'1520401A03Rik'	34	49	17	11	37	23	33.33333333	23.66666667	-0.467002368	0.460545135	0.972790461
320311	'Rnf152'	265	318	261	114	323	206	281.3333333	214.3333333	-0.402330973	0.311116898	0.911205466
320332	'H4f16'	1	1	1	0	1	0	1	0.333333333	-1.427484097	0.601914152	0.972790461
320333	'D830030K20Rik'	60.15	56.83	17.63	6	63.52	24	44.87	31.17333333	-0.534134504	0.523972807	0.972790461
320343	'Lypd6'	896	1056	419	229	872	865	790.3333333	655.3333333	-0.286955767	0.57586409	0.972790461
320351	'Tmem251'	113	115	297	213	176	225	175	204.6666667	0.238435897	0.702195754	0.972790461
320352	'Lrrc31'	3	8	1	0	18	44	4	20.66666667	2.277122968	0.122616508	0.714169238
320355	'Lipi'	0	2	0	0	0	1	0.666666667	0.333333333	-0.733814424	0.856146772	0.975734242
320360	'Ric3'	222	207	43	27	217	170	157.3333333	138	-0.210177862	0.786092586	0.972790461
320365	'Fry'	171	145	49	637	181	275	121.6666667	364.3333333	1.89240956	0.011768325	0.268666967
320376	'Bcorl1'	1430	1247	841	999	1026	1084	1172.666667	1036.333333	-0.071952965	0.832104973	0.974662707
320394	'Cenpt'	282	298	272	61	353	305	284	239.6666667	-0.332075804	0.552183879	0.972790461
320398	'Lrig3'	180	183	278	808	203	273	213.6666667	428	1.23916675	0.105812589	0.679124905
320404	'Itpkb'	2119	2370	2712	2436	2588	2010	2400.333333	2344.666667	0.046446862	0.910801994	0.989608384
320405	'Cadps2'	280	232	117	8	229	475	209.6666667	237.3333333	0.061948993	0.949426484	0.997836337

320415	'Gchfr'	26	38	12	26	2	4	25.33333333	10.66666667	-0.838342447	0.465023198	0.972790461
320429	'Trank1'	4157	4408	1489	2311	4696	3089	3351.333333	3365.333333	0.087268204	0.850737275	0.975734242
320435	'Rin1'	88	65	19	37	65	110	57.33333333	70.66666667	0.341702168	0.585785347	0.972790461
320438	'Alg6'	226	240	119	76	261	309	195	215.3333333	0.116096741	0.815267927	0.972790461
320452	'P4ha3'	63	81	35	11	115	104	59.66666667	76.66666667	0.291010241	0.697228799	0.972790461
320460	'Vwc2l'	0	0	0	0	0	2	0	0.666666667	1.749449488	0.66566965	0.972790461
320472	'Ppm1e'	710	719	321	216	788	406	583.3333333	470	-0.29428497	0.559629322	0.972790461
320473	'Heatr5b'	2299	2215	770	1384	1299	1092	1761.333333	1258.333333	-0.31144326	0.539570771	0.972790461
320484	'Rasal3'	6	11	3	2	5	5	6.666666667	4	-0.697401791	0.504482516	0.972790461
320487	'Heatr5a'	1721	1694	706	2054	2080	2164	1373.666667	2099.333333	0.746743725	0.078529794	0.615492265
320492	'A830018L16Rik'	15	22	6	2	21	5	14.33333333	9.333333333	-0.621457236	0.542545317	0.972790461
320495	'Ipcef1'	28	30	4	1	8	4	20.66666667	4.333333333	-2.219781055	0.036343495	0.456985794
320500	'Tmem215'	5	4	8	1	9	13	5.666666667	7.666666667	0.296336109	0.787600247	0.972790461
320502	'Lmod3'	1	1	0	0	0	3	0.666666667	1	0.518269698	0.873573771	0.980171118
320506	'Lmbrd2'	687	585	409	2329	826	1145	560.3333333	1433.333333	1.603027339	0.010309162	0.250957475
320508	'Cachd1'	1167	1066	307	1146	1909	1837	846.6666667	1630.666667	1.034789857	0.037806872	0.459930654
320522	'Bhlha9'	2	3	0	0	0	0	1.666666667	0	-3.014977875	0.444538231	0.969888068
320528	'Vps13c'	2268	2168	932	2029	1786	1380	1789.333333	1731.666667	0.133214835	0.78726279	0.972790461
320534	'Tmem104'	270	276	89	215	355	395	211.6666667	321.6666667	0.675175915	0.145533714	0.753611197
320538	'Ubn2'	2327.3	2343.2	1115.1	1128	2310.2	1492.01	1928.526667	1643.423333	-0.165637345	0.672958675	0.972790461
320541	'Slc35e2'	1129	1178	682.01	853	1377	863	996.3366667	1031	0.134883792	0.711946248	0.972790461
320554	'Tcp11l1'	432.68	409.7	191.43	474.76	390.33	375.9	344.6033333	413.6633333	0.430788191	0.359527846	0.933706718
320557	'Fam169a'	546.03	485.91	1450.7	170.62	342.44	475.95	827.5466667	329.67	-1.449393738	0.020866467	0.358627562
320558	'Sycp2'	3588	3889	673	8	1649	914	2716.666667	857	-1.726183696	0.14314548	0.749054337
320560	'Dennd5b'	909.63	857.07	459	177.28	701.16	593	741.9	490.48	-0.619194941	0.198007848	0.823747311
320563	'Islr2'	12	15	32	80	22	22	19.66666667	41.33333333	1.281626163	0.167757041	0.782225778
320571	'Atp8b5'	46	58	15	1	88	58	39.66666667	49	0.231366317	0.831990526	0.974662707
320581	'Idi2'	3	7	8	0	0	0	6	0	-5.009442282	0.01253543	0.277443073
320587	'Tmem88b'	9	12	7	4	11	15	9.333333333	10	0.083696581	0.915970598	0.990491125
320590	'Svopl'	7	10	0	1	7	8	5.666666667	5.333333333	-0.075438139	0.95847533	0.999493374
320595	'Phf8'	3338	3169	1617	919	2110	2064	2708	1697.666667	-0.651375335	0.079381898	0.617305615
320604	'Ccdc169'	3	1	2	0	2	3	2	1.666666667	-0.368918333	0.833231217	0.974723675
320609	'Strip2'	260	258	68	156	158	100	195.3333333	138	-0.310701997	0.609216491	0.972790461
320615	'Dop1a'	2668.5	2592.2	443.81	516.88	1416	1165.21	1901.5	1032.7	-0.822269996	0.19990003	0.82759347
320631	'Abca15'	1	0	0	0	0	0	0.333333333	0	-0.903279821	0.824807108	0.972790461
320632	'Snrnp200'	7443	6997	3236	2433	5237	5857	5892	4509	-0.360468991	0.361111802	0.933706718
320633	'Zbtb26'	911	978	410	238	784	814	766.3333333	612	-0.332744464	0.490327648	0.972790461
320634	'Ocrl'	1629	1565	648	614	1674	1261	1280.666667	1183	-0.083052136	0.852213411	0.975734242
320635	'Cyb5r2'	6	8	19	9	3	12	11	8	-0.443852209	0.658586645	0.972790461
320655	'Pgap3'	193	215	120	67	236	211	176	171.3333333	-0.059109967	0.897450998	0.985794067

320661	'D5Ertd579e'	2387	2386	947	1252	1778	2237	1906.666667	1755.666667	-0.046459333	0.907662404	0.988823459
320662	'Casc1'	60	62	10	48	85	81	44	71.33333333	0.791196038	0.234689103	0.861206181
320664	'Cass4'	11	14	4	4	7	7	9.666666667	6	-0.619693368	0.482199527	0.972790461
320678	'Iffo1'	295	368	105	215	637	397	256	416.3333333	0.746971312	0.161359509	0.771435531
320679	'Samd12'	193	192	84	37	149	254	156.3333333	146.6666667	-0.141766489	0.820060536	0.972790461
320683	'Zfp629'	1216	1157	710	890	1190	1170	1027.666667	1083.333333	0.153717024	0.601542742	0.972790461
320685	'Dctd'	418	497	299	30	297	242	404.6666667	189.6666667	-1.178632281	0.079538848	0.617305615
320696	'Ccdc158'	308	325	143	124	132	198	258.6666667	151.3333333	-0.686324201	0.095992645	0.662457477
320700	'A930033H14Rik'	146	161	84	39	143	145	130.3333333	109	-0.282531624	0.569158922	0.972790461
320701	'Tafa4'	70.96	78.55	21.11	63.81	92.69	62.27	56.87333333	72.92333333	0.486223226	0.395111211	0.954014376
320705	'Bend6'	30	26	49	43	14	23	35	26.66666667	-0.236420929	0.762556107	0.972790461
320706	'Soga1'	2742	2579	2284	2186	2859	2542	2535	2529	0.061243954	0.826714707	0.972859382
320707	'Atp2b3'	357	384	31	23	949	694	257.3333333	555.3333333	1.067025889	0.326014636	0.920269559
320709	'Tmem117'	23	24	20	0	22	27	22.33333333	16.33333333	-0.589516396	0.590032689	0.972790461
320712	'Abi3bp'	334	278	43	245	557	367	218.3333333	389.6666667	0.931346788	0.172433081	0.790141656
320713	'Mysm1'	1787.1	1723.3	820.45	1553.7	2480.7	2464	1443.6	2166.14	0.653191589	0.060937697	0.560497095
320714	'Trappc11'	2724	2604	836	1098	1958	2048	2054.666667	1701.333333	-0.202862749	0.6625865	0.972790461
320717	'Pptc7'	519.37	598.48	582.51	1104.2	536.56	749.82	566.7866667	796.85	0.654736983	0.220807568	0.849076011
320718	'Slc26a9'	4	2	1	1	2	4	2.333333333	2.333333333	0.0134879	0.992604069	0.999493374
320720	'Fastkd1'	369	401	208	332	452	450	326	411.3333333	0.41509594	0.205276786	0.833294914
320722	'Akain1'	1	1	2	0	1	0	1.333333333	0.333333333	-1.889143717	0.460859139	0.972790461
320727	'Ipo8'	1014	992	819	595	1205	1538	941.6666667	1112.666667	0.22613871	0.477724283	0.972790461
320736	'Vstm4'	392	453	95	72	538	462	313.3333333	357.3333333	0.164552624	0.823261879	0.972790461
320747	'Lingo4'	7	3	1	1	7	7	3.666666667	5	0.431495365	0.736071292	0.972790461
320752	'Dpy19l2'	9	7	1	0	5	9	5.666666667	4.666666667	-0.333593499	0.819015118	0.972790461
320769	'Prdx6b'	1	2	0	0	0	0	1	0	-2.28103714	0.568641838	0.972790461
320772	'Mdga2'	49	36	56	5	28	21	47	18	-1.468732525	0.02693852	0.403699531
320782	'Tmem154'	15	12	16	2	23	28	14.33333333	17.66666667	0.174265286	0.845562134	0.975182082
320790	'Chd7'	871	863	439	275	701	923	724.3333333	633	-0.203574335	0.638638497	0.972790461
320795	'Pkn1'	1336.2	1290.8	1230.8	1612.5	1583.2	1617.07	1285.956667	1604.283333	0.408463795	0.234509508	0.861206181
320799	'Zhx3'	753	721	296	745	666	692	590	701	0.403338718	0.380727417	0.944042914
320802	'Ifitm10'	3	1	11	6	1	12	5	6.333333333	0.274995227	0.843516151	0.975182082
320806	'Gfm2'	876	804	466.02	335	888.08	740.04	715.34	654.3733333	-0.117410786	0.756774732	0.972790461
320808	'Dcaf5'	1788	1880	1679	1553	1824	2379	1782.333333	1918.666667	0.149189204	0.588208845	0.972790461
320816	'Ankrd16'	319	321	179	146	343	199	273	229.3333333	-0.205998718	0.612289757	0.972790461
320817	'Atad2b'	2048	2052	1394.7	997	2158	1821	1831.58	1658.666667	-0.123105233	0.676808956	0.972790461
320825	'Samd5'	95	109	68	9	105	56	90.66666667	56.66666667	-0.752481102	0.29937975	0.903198439
320827	'Cracd'	276	284	320	12	206	128	293.3333333	115.3333333	-1.481540282	0.056271694	0.545946937
320832	'Sirpb1a'	0	0	0	0	0	1	0	0.333333333	1.020273531	0.802557913	0.972790461
320840	'Negr1'	82.54	81.74	55.86	18.81	66.25	71.91	73.38	52.32333333	-0.527271111	0.301409176	0.90469437

320844	'Amigo3'	72.14	60.66	54	79	87.74	52	62.26666667	72.91333333	0.344624613	0.485151916	0.972790461
320858	'L3mbtl4'	2	5	4	4	382	42	3.666666667	142.6666667	5.153418116	5.36E-05	0.008568148
320864	'Krt26'	1	0	0	0	1	0	0.333333333	0.333333333	0.058500858	0.988561293	0.999493374
320865	'Cdh18'	17	17	2	2	5	5	12	4	-1.513946217	0.15907734	0.769953766
320869	'Spata33'	159	188	53	32	165	157	133.3333333	118	-0.194362126	0.76458323	0.972790461
320873	'Cdh10'	10	10	19	1	15	4	13	6.666666667	-1.084336691	0.310228239	0.910971113
320878	'Mical2'	2355	2098	3506	6450	2233	3612	2653	4098.333333	0.796221801	0.224252965	0.850759465
320910	'Itgb8'	345	306	232	272	443	567	294.3333333	427.3333333	0.557475388	0.058686261	0.552152463
320916	'Wscd2'	60	55	15	7	52	46	43.33333333	35	-0.33326106	0.670593314	0.972790461
320923	'Map7d3'	61	57	49	8	79	55	55.66666667	47.33333333	-0.323203799	0.647201644	0.972790461
320924	'Ccbe1'	606	539	86	361	625	571	410.3333333	519	0.445417302	0.491464364	0.972790461
320938	'Tnpo3'	2470.6	2700.9	2464.8	1255.6	2296	2395.63	2545.443333	1982.396667	-0.35986011	0.182966372	0.804092742
320940	'Atp11c'	1387	1467	461	370	1295	1264	1105	976.3333333	-0.17474101	0.747432203	0.972790461
320951	'Pisd'	1166	1134	1137	813	1449	1359	1145.666667	1207	0.084318721	0.758532895	0.972790461
320974	'Lrrn4'	154	158	10	71	242	183	107.3333333	165.3333333	0.682225137	0.423925381	0.960285372
320981	'Enpp6'	20	14	4	2	34	18	12.66666667	18	0.469969056	0.646677303	0.972790461
320982	'Arl4c'	527	514	1137	717	664	534	726	638.3333333	-0.136179539	0.814565465	0.972790461
320995	'Rfx6'	1	0	0	0	0	0	0.333333333	0	-0.903279821	0.824807108	0.972790461
320997	'Cyp4f39'	1	1	19	3	2	3	7	2.666666667	-1.473649646	0.340776843	0.925308103
321000	'Lrif1'	611	598	615	312	605	571	608	496	-0.29680603	0.321260347	0.917500633
321003	'Xpnpep3'	697	684	570	408	538	541	650.3333333	495.6666667	-0.336081804	0.196833204	0.822696823
321006	'Dcaf1'	2150	2125	1409	874	1533	1900	1894.666667	1435.666667	-0.38178538	0.184909267	0.804884883
321007	'Serac1'	509.15	489.87	421.76	319.15	429.56	361.92	473.5933333	370.21	-0.29056085	0.331020107	0.921648675
321008	'Zswim9'	315.55	327.84	143.8	127	295	215.94	262.3966667	212.6466667	-0.254947203	0.545147687	0.972790461
321019	'Gpr183'	8	11	0	1	16	8	6.333333333	8.333333333	0.399487868	0.782711628	0.972790461
321022	'Cdv3'	4803.2	4688.4	8905.7	7242	4012.4	6193.41	6132.453333	5815.94	0.006786903	0.990627623	0.999493374
326618	'Tpm4'	3809	3750	8792	7083	5155	5532	5450.333333	5923.333333	0.177919358	0.768027486	0.972790461
326619	'H4c1'	1	4.09	6.52	2	1.03	1	3.87	1.343333333	-1.388562928	0.364453332	0.935515019
326620	'H4c2'	9.03	3.08	42.61	1	4.11	10	18.24	5.036666667	-2.084342783	0.101379334	0.671759705
326622	'Upf2'	2467	2499	2099	2653	2137	2073	2355	2287.666667	0.090326669	0.824896509	0.972790461
326623	'Tnfsf15'	2	4	48	11	4	9	18	8	-1.196190139	0.36866734	0.937530612
327655	'Ppip5k1'	587	630	328	871.27	729	642.26	515	747.51	0.700675969	0.118303303	0.707231175
327743	'Ccn6'	26	24.16	1	0	27.3	10	17.05333333	12.43333333	-0.489923044	0.738859216	0.972790461
327747	'Mettl24'	263	321	29	30	459	323	204.3333333	270.6666667	0.380239902	0.68968121	0.972790461
327762	'Dna2'	235	224	42	38	171	153	167	120.6666667	-0.458710525	0.517084906	0.972790461
327766	'Tmem26'	43	49	11	523	55	54	34.33333333	210.6666667	3.082199719	0.004164709	0.149677594
327799	'Usp44'	40.26	33.39	16.8	0	8.55	7.11	30.15	5.22	-2.615077591	0.005033931	0.16781708
327814	'Ppfia2'	11	12	16	2	17	8	13	9	-0.617013124	0.492931102	0.972790461
327826	'Frs2'	2777	2440	1866	2832	2020	2376	2361	2409.333333	0.174416028	0.679183271	0.972790461
327900	'Ubtd2'	1729	1737	3901	1487	1532	1424	2455.666667	1481	-0.710705063	0.201594373	0.82952222

327942	'Pigl'	376	412	199	733	383	468	329	528	0.895350425	0.101965209	0.671776916
327951	'Cyb5d1'	1402	1404	681	487.05	1059	926.09	1162.333333	824.0466667	-0.458772016	0.21198666	0.84083615
327954	'Dnah2'	290	238	15	7	147	99	181	84.33333333	-1.126089577	0.292036574	0.89958198
327956	'Vmo1'	2	3	7	0	0	1	4	0.333333333	-3.502536729	0.082981844	0.625803578
327957	'Scimp'	0	2	0	0	0	0	0.666666667	0	-1.695595436	0.675304306	0.972790461
327958	'Pitpnm3'	396	450	42	97	294	210	296	200.3333333	-0.494558355	0.518871346	0.972790461
327959	'Xaf1'	103	124	208	31	95	268	145	131.3333333	-0.305809432	0.675554686	0.972790461
327963	'Zfp616'	0	0	0	0	0	1	0	0.333333333	1.020273531	0.802557913	0.972790461
327978	'Slfn5'	34	38.03	21	19.01	34	68.62	31.01	40.54333333	0.371381641	0.509900772	0.972790461
327987	'Med13'	3623	3549	3622	3106	3204	3004	3598	3104.666667	-0.129428534	0.71272541	0.972790461
327992	'Hsf5'	189	217	39	4	78	101	148.3333333	61	-1.340899328	0.155640063	0.766786441
328019	'Spata32'	1	0	0	1	0	0	0.333333333	0.333333333	0.058500858	0.988561293	0.999493374
328035	'Fads6'	296	319	300	1430	578	846	305	951.3333333	1.834124865	0.001580707	0.085308652
328059	'Slc7a15'	2	1	0	15	0	1	1	5.333333333	2.927675567	0.180410185	0.801341923
328092	'Dtd2'	366	338	175	167	346	530	293	347.6666667	0.247990426	0.568279474	0.972790461
328099	'Prpsl13'	1082.5	1224.2	1024.4	594.63	1132	1223.99	1110.366667	983.54	-0.176092926	0.526303173	0.972790461
328108	'Togaram1'	1431	1438	1315	1194	1359	1429	1394.666667	1327.333333	-0.005379646	0.985313664	0.999493374
328110	'Prpf39'	1713	1809.2	1239.9	3385.7	2017.2	1109.16	1587.393333	2170.703333	0.687296998	0.264201187	0.880608864
328133	'Slc39a9'	1203	1176	632	765	1292	1356.01	1003.666667	1137.67	0.229909505	0.466596919	0.972790461
328162	'Trmt61a'	230	234	136	44	146	222	200	137.3333333	-0.58786605	0.260274435	0.878537553
328232	'Gfod1'	297	269	707	3841	338	721	424.3333333	1633.333333	2.231003627	0.027407826	0.404916403
328234	'Rnf182'	235	279	53	927	333	462	189	574	1.902539533	0.011526828	0.266279538
328258	'Slc25a48'	1	2	0	18	0	9	1	9	3.527777092	0.064134043	0.568781147
328274	'Zfp459'	65	66	36	54	105	248	55.66666667	135.6666667	1.254641517	0.025389176	0.39436194
328329	'Mast4'	1907	1756	1194	2142	1725	1594	1619	1820.333333	0.323077491	0.449097397	0.97049895
328330	'D130037M23Rik'	118.18	116.08	60.04	137.12	198.28	179.13	98.1	171.51	0.885105107	0.016785452	0.323948168
328365	'Zmiz1'	2659	2397	5187	3598	2324	2707	3414.333333	2876.333333	-0.16794487	0.775539767	0.972790461
328370	'Rft1'	555	566	134	195	607	501	418.3333333	434.3333333	0.089588591	0.878310225	0.981341203
328381	'Sh2d4b'	43	42	27	185	60	28	37.33333333	91	1.617060509	0.061989367	0.564935721
328401	'Rnase9'	0	0	0	0	1	0	0	0.333333333	1.020273531	0.802557913	0.972790461
328417	'Parp4'	659	643	652	365	823	867	651.3333333	685	0.045813416	0.89060649	0.985003525
328424	'Kcnrg'	8.21	5.26	1	6.15	4.43	0	4.823333333	3.526666667	-0.175573279	0.911044249	0.98964537
328440	'Npm2'	22	18	9	5	12	17	16.33333333	11.33333333	-0.51704474	0.473894638	0.972790461
328505	'Skint7'	0	1	0	0	0	0	0.333333333	0	-0.903279821	0.824807108	0.972790461
328561	'Apol10b'	90	56	4	3	36	30	50	23	-1.13145152	0.295803957	0.90029104
328563	'Apol11b'	1	1	0	0	0	1	0.666666667	0.333333333	-0.744986434	0.839556299	0.974723675
328572	'Ep300'	3719.4	3609.4	2406.8	3317	3413.3	3312.22	3245.2	3347.496667	0.156499291	0.643599628	0.972790461
328573	'4930407I10Rik'	2	3	1	0	0	0	2	0	-3.337472277	0.23673828	0.862272407
328577	'7530416G11Rik'	0	0	0	1	0	0	0	0.333333333	1.020273531	0.802557913	0.972790461
328580	'Tubgcp6'	1252.4	1203.9	315.05	894.57	1170.9	854.39	923.7633333	973.3	0.220941664	0.68459179	0.972790461

328643	'Vwa5b2'	13	10	1	15	8	2	8	8.333333333	0.389106774	0.754782863	0.972790461
328660	'Bex6'	0	0	0	0	1	0	0	0.333333333	1.020273531	0.802557913	0.972790461
328778	'Rab26'	14	16	25	19	7	24	18.33333333	16.66666667	-0.079831766	0.919879338	0.990919937
328779	'Hs3st6'	10	6	43	1	0	8	19.66666667	3	-2.917452499	0.041178514	0.475995577
328780	'Prss34'	2	0	0	0	1	0	0.666666667	0.333333333	-0.74959284	0.853034393	0.975734242
328783	'Mslnl'	4	3	3	7	3	2	3.333333333	4	0.471063201	0.702005623	0.972790461
328789	'Lhfpl5'	0	0	1	0	0	0	0.333333333	0	-0.903279821	0.824807108	0.972790461
328795	'Ubash3a'	28	40	4	0	60	27	24	29	0.226711185	0.866488515	0.978294615
328801	'Zfp414'	309	344	354	83	338	316	335.6666667	245.6666667	-0.528232049	0.280554779	0.889355605
328829	'9830107B12Rik'	7	6	0	0	0	0	4.333333333	0	-4.393988266	0.141358594	0.746115932
328830	'A530064D06Rik'	21.51	31	1	1	8	15	17.83666667	8	-1.153653427	0.355228043	0.931809687
328833	'Treml2'	5	2	1	0	6	3	2.666666667	3	0.117328624	0.942673163	0.996301887
328918	'Zscan30'	192.19	236.29	52.45	83.08	233.15	131.45	160.31	149.2266667	-0.03597375	0.952575847	0.998807192
328949	'Mcc'	358	342	2181	362	287	520	960.3333333	389.6666667	-1.380322555	0.10457865	0.67684333
328967	'Arhgef37'	23.27	16.27	38.09	43.95	4.99	20.42	25.87666667	23.12	0.007651533	0.993978522	0.999562152
328971	'Spink10'	0	1	2	0	4	0	1	1.333333333	0.223774474	0.939940945	0.995607401
328977	'Zfp532'	1169	1180	1184	663	1390	1235	1177.666667	1096	-0.11164425	0.713814216	0.972790461
329002	'Zfp236'	1651	1631	1006	1099	1485	1092	1429.333333	1225.333333	-0.124925627	0.71591523	0.972790461
329003	'Zfp516'	2452	2212	3534	2848	2185.2	2618	2732.666667	2550.413333	-0.025232074	0.958031997	0.999493374
329015	'Atg2a'	915	913	585	1384	1158	883	804.3333333	1141.666667	0.670057992	0.141826946	0.747136019
329055	'Lipo1'	1	2	0	0	0	1	1	0.333333333	-1.323989397	0.695217833	0.972790461
329064	'Pkd2l1'	12	17	4	4	8	4	11	5.333333333	-0.948289791	0.3106976	0.910971113
329065	'Scd4'	4	6	2	9	8	5	4	7.333333333	1.034329918	0.310800845	0.910971113
329093	'Cpa6'	2	1	0	0	0	0	1	0	-2.286544758	0.567674219	0.972790461
329152	'Hecw2'	191	215	171	74	135	102	192.3333333	103.6666667	-0.857907294	0.009539913	0.240209487
329154	'Ankrd44'	370	343	216	171	273	220	309.6666667	221.3333333	-0.417874987	0.198312428	0.824298956
329165	'Abi2'	1928	2085	1778	782	1700	1962	1930.333333	1481.333333	-0.402639363	0.205078594	0.833294914
329178	'Unc80'	28	29	13	10	57	37	23.33333333	34.66666667	0.548134174	0.426675876	0.961360721
329207	'Rbm44'	907	866	99	2	429	661	624	364	-0.8488102	0.499406796	0.972790461
329244	'Il19'	0	0	2	7	0	0	0.666666667	2.333333333	2.089229059	0.540825864	0.972790461
329251	'Ppp1r12b'	1649	1644	969	440	1224	1091	1420.666667	918.3333333	-0.630692163	0.094355432	0.658790111
329252	'Lgr6'	239.09	242	14	1171	141	194	165.03	502	2.072650528	0.065366822	0.574020335
329260	'Dennd1b'	999	1062	1702	2027	1130	1125	1254.333333	1427.333333	0.322261123	0.588523115	0.972790461
329274	'Fam163a'	20	27	14	88	7	5	20.33333333	33.33333333	1.157473521	0.335094611	0.922326626
329278	'Tnn'	3	5	16	1164	8	9	8	393.6666667	5.991320315	1.67E-04	0.019862371
329324	'Syt14'	1042	1085	786	1084	765	843	971	897.3333333	0.0368814	0.932152783	0.993860034
329360	'Rnf224'	3	0	2	0	2	3	1.666666667	1.666666667	-0.145867288	0.9454794	0.996592403
329366	'Ccdc187'	175	135	0	2	299	119	103.3333333	140	0.412444196	0.796332432	0.972790461
329375	'Cfap77'	14	8	3	2	11	13	8.333333333	8.666666667	0.038824601	0.968974957	0.999493374
329384	'Ptrh1'	57	53	279	103	41	103	129.6666667	82.33333333	-0.651308844	0.461526751	0.972790461

329416	'Nostrin'	19	19	6	1	29	24	14.66666667	18	0.230097353	0.824056573	0.972790461
329421	'Myo3b'	7	4	1	8	13	7	4	9.333333333	1.337046624	0.191912216	0.813837363
329470	'Accs'	235	216	124	85	328	198	191.6666667	203.6666667	0.08183897	0.866642103	0.97830422
329482	'Dcdc5'	15	20	3	0	6	0	12.66666667	2	-2.654648605	0.100715334	0.671759705
329502	'Pla2g4e'	119	82	9	1	1	0	70	0.666666667	-6.537340723	3.13E-06	9.75E-04
329504	'Lcmt2'	214.51	238.29	162.3	166.99	298.82	332.88	205.0333333	266.23	0.39420574	0.180417219	0.801341923
329506	'Ctdspl2'	1656.6	1729.5	2351.6	1261	1574	1619	1912.573333	1484.666667	-0.342723848	0.367572527	0.937190492
329509	'1810024B03Rik'	6	5	3	0	1	2	4.666666667	1	-2.253592448	0.14285821	0.749054337
329540	'Nol41'	1742	1751	1081	1546	1463	1280	1524.666667	1429.666667	0.043904736	0.910799962	0.989608384
329547	'Bpi'	7	0	0	0	0	1	2.333333333	0.333333333	-2.547775496	0.491341656	0.972790461
329554	'Gm826'	21	27	5	0	60	40	17.66666667	33.33333333	0.84726664	0.512945153	0.972790461
329559	'Zfp335'	693	707	633	853	759	597	677.6666667	736.3333333	0.248105255	0.56117185	0.972790461
329575	'Gm14325'	814.36	810.1	461.54	518.36	688	521.12	695.3333333	575.8266667	-0.170068273	0.633958097	0.972790461
329581	'Birc7'	0	0	0	2	0	0	0	0.666666667	2.299096387	0.566358544	0.972790461
329628	'Fat4'	3069	2896	190	783	3961	1973	2051.666667	2239	0.171847423	0.844598372	0.975182082
329641	'Sertm1'	25	23	261	13	13	5	103	10.33333333	-3.346842931	0.00268903	0.116112669
329650	'Med12l'	598.49	608.3	143.96	368.12	803.36	662.84	450.25	611.44	0.515135322	0.349940541	0.92886351
329659	'E130311K13Rik'	198	206	178	33	201	246	194	160	-0.377830759	0.536581205	0.972790461
329679	'Fnip2'	707.08	699.34	864.35	535.2	584.26	773.53	756.9233333	630.9966667	-0.233996855	0.515028697	0.972790461
329693	'Fcrl5'	0	2	0	0	1	1	0.666666667	0.666666667	0.021482588	0.994987788	0.999562152
329702	'Dcst2'	10	12	4	11	6	7	8.666666667	8	0.071740734	0.936831425	0.994413066
329716	'BC107364'	10	8	3	46	9	6	7	20.33333333	1.92880455	0.088692761	0.64452174
329727	'Dennd2c'	400	398	247	92	201	186	348.3333333	159.6666667	-1.104470745	9.45E-04	0.064169552
329731	'Tafa3'	10	4	8	3	10	3	7.333333333	5.333333333	-0.463337616	0.653131149	0.972790461
329738	'Aknad1'	0	0	2	1	0	2	0.666666667	1	0.467054641	0.880969927	0.982461301
329739	'Fam102b'	980	976	1688	1233	1075	1015	1214.666667	1107.666667	-0.067754758	0.894147541	0.985194613
329777	'Pigk'	1044	1118	338	850	1435	1469	833.3333333	1251.333333	0.664645099	0.152301609	0.762591043
329795	'Tmem67'	305	290	130	154	367	281	241.6666667	267.3333333	0.186611244	0.659920655	0.972790461
329828	'Myorg'	106	105	46	98	91	84	85.66666667	91	0.234777233	0.631467802	0.972790461
329831	'Fam166b'	18	21	1	3	10	2	13.33333333	5	-1.300490721	0.290757057	0.898719095
329839	'Gm829'	0	0	0	0	1	0	0	0.333333333	1.020273531	0.802557913	0.972790461
329872	'Frem1'	1181	1004	71	353	990	509	752	617.3333333	-0.185852625	0.824793504	0.972790461
329877	'Dennd4c'	2626	2831	1592	3187	3063	2829	2349.666667	3026.333333	0.499193658	0.193598691	0.817388034
329908	'Usp24'	2612	2622	1395	1234	2515	2601	2209.666667	2116.666667	-0.0342829	0.918637436	0.990815958
329909	'Tmem61'	0	0	0	1	0	1	0	0.666666667	2.022653929	0.615796212	0.972790461
329910	'Acot11'	88.9	85.16	152	118	139.32	99.47	108.6866667	118.93	0.171373924	0.746815819	0.972790461
329919	'Skint2'	0	0	1	0	0	0	0.333333333	0	-0.903279821	0.824807108	0.972790461
329934	'Foxo6'	206	256	274	415	267	446	245.3333333	376	0.704378143	0.133856946	0.733301819
329941	'Col8a2'	380	351	12	408	526	320	247.6666667	418	0.951398369	0.310698052	0.910971113
329942	'Csmd2'	5	8	2	14	13	16	5	14.33333333	1.637112521	0.052802898	0.532526438

329954	'Catsper4'	1	1	0	0	0	0	0.666666667	0	-1.703500596	0.673820112	0.972790461
329972	'Spata21'	21	22	2	68	80	55	15	67.66666667	2.348868078	0.002593497	0.11413786
329977	'Fhad1'	24	39	39	7	19	65	34	30.33333333	-0.287715869	0.718812524	0.972790461
329984	'Pramel16'	1	0	0	0	0	0	0.333333333	0	-0.903279821	0.824807108	0.972790461
329993	'Aadacl4fm5'	1	1	0	0	0	0	0.666666667	0	-1.703500596	0.673820112	0.972790461
330010	'Ttll10'	109	82	5	0	88	25	65.33333333	37.66666667	-0.825813916	0.560591521	0.972790461
330050	'Fam185a'	258	259	175	177	256	254	230.6666667	229	0.046546147	0.870050392	0.979794316
330064	'Slc5a6'	292	294	190	108	297	298	258.6666667	234.3333333	-0.154157521	0.689975593	0.972790461
330096	'Shisa3'	19	30	27	13	25	26	25.33333333	21.33333333	-0.252097221	0.65158494	0.972790461
330119	'Adamts3'	192	155	85	289	127	124	144	180	0.58086202	0.371268688	0.938513534
330122	'Cxcl3'	1	2	103	1153	2	23	35.33333333	392.6666667	3.738904811	0.126537423	0.721358164
330149	'Hfm1'	2643	2449	667	9	1723	936	1919.666667	889.3333333	-1.184042784	0.301492165	0.90469437
330171	'Kctd10'	1615.1	1692.1	2961.4	1682.8	1951.1	2178.31	2089.52	1937.413333	-0.104555297	0.821378032	0.972790461
330173	'2610524H06Rik'	270.71	267	68	201	276.01	314	201.9033333	263.67	0.489937421	0.35535208	0.931809687
330177	'Taok3'	317	351	577	922	376	495	415	597.6666667	0.682192373	0.297215444	0.900647119
330188	'Ccdc63'	2	3	0	1	2	1	1.666666667	1.333333333	-0.198936331	0.916258794	0.990526893
330189	'Tmem120b'	105	91	103	109	159	159	99.66666667	142.3333333	0.540518912	0.113537851	0.698948431
330192	'Vps37b'	567	584	639	1585	568	879	596.6666667	1010.666667	0.95662521	0.119734372	0.710007992
330216	'Mblac1'	64	82	41	19	59	78	62.33333333	52	-0.285508479	0.59364272	0.972790461
330217	'Gal3st4'	66	62	14	13	42	34	47.33333333	29.66666667	-0.634948742	0.36033262	0.933706718
330222	'Sdk1'	941	830	296	77	842	735	689	551.3333333	-0.379428326	0.611272979	0.972790461
330228	'Spdye4b'	135	133	21	0	57	27	96.33333333	28	-1.833619381	0.128801546	0.72577634
330230	'Zfp853'	172	130	46	2	165	168	116	111.6666667	-0.143251386	0.893349489	0.985171
330260	'Pon2'	203	162	109	44	286	278	158	202.6666667	0.290301091	0.636315356	0.972790461
330267	'Thsd7a'	45	37	39	9	46	20	40.33333333	25	-0.730468784	0.258441187	0.877564912
330277	'Fam71f1'	0	2	5	2	1	1	2.333333333	1.333333333	-0.762923361	0.683986162	0.972790461
330286	'D630045J12Rik'	703.99	683	115	147	770	730	500.6633333	549	0.132320096	0.856331522	0.975878133
330301	'Zfp786'	90.01	87.01	47	23.01	93.02	97	74.67333333	71.01	-0.103214479	0.849796586	0.975734242
330305	'Gm5111'	0	0	1	0	0	0	0.333333333	0	-0.903279821	0.824807108	0.972790461
330319	'Wipf3'	135	139	75	37.39	150	168	116.3333333	118.4633333	-0.01385223	0.979243127	0.999493374
330323	'Mindy4'	182	194	161	70	220	154	179	148	-0.29524302	0.474682698	0.972790461
330355	'Dnah6'	86	29	4	2	95	133	39.66666667	76.66666667	0.890192158	0.470413546	0.972790461
330361	'Gcfc2'	311.85	321.56	215.2	306.03	482.2	334.72	282.87	374.3166667	0.477842621	0.154611266	0.766453805
330369	'Fbxo41'	77	87	99	35	112	106	87.66666667	84.33333333	-0.117329697	0.808171636	0.972790461
330390	'Gm765'	5	5	0	1	7	3	3.333333333	3.666666667	0.178059647	0.90836437	0.988823459
330401	'Tmcc1'	793	814	690	547	774	872	765.6666667	731	-0.031984515	0.895028266	0.985422597
330406	'B4galnt3'	77	79	18	39	49	41	58	43	-0.294326934	0.637861699	0.972790461
330409	'Cecr2'	1851	1741	333	99	847	1319	1308.333333	755	-0.84228805	0.311192306	0.911205466
330428	'Tmem52b'	1	0	0	0	0	0	0.333333333	0	-0.903279821	0.824807108	0.972790461
330440	'Sult6b2'	1	0	11	5	2	0	4	2.333333333	-0.656346985	0.760078263	0.972790461

330450	'Far2'	110.53	76.34	40.13	50.66	103.07	26.61	75.66666667	60.11333333	-0.226080909	0.734390243	0.972790461
330460	'Tmem150b'	2.06	2.01	2.04	1.01	4.08	0	2.036666667	1.696666667	-0.239168828	0.893445745	0.985171
330463	'Zfp78'	56.1	73.41	111	47	69	51.72	80.17	55.90666667	-0.514744097	0.333016622	0.921648675
330474	'Zc3h4'	2131	2181	923	1213	1998	1474	1745	1561.666667	-0.069600427	0.861534302	0.976863639
330483	'Ceacam16'	0	0	0	19.6	6	3	0	9.533333333	5.964553841	0.002405519	0.110099123
330485	'Tmem145'	23	31	5	6	22	7	19.66666667	11.66666667	-0.680792423	0.453543529	0.970649024
330502	'Zfp82'	167	172	313	66	174	159	217.3333333	133	-0.789250222	0.13101184	0.728882802
330513	'Gm5114'	0	1	0	0	0	2	0.333333333	0.666666667	0.78767502	0.845746136	0.975182082
330554	'Fan1'	595	594	186	243	597	528	458.3333333	456	0.037228103	0.940233087	0.995805886
330577	'Saxo2'	84	77	34	27	73	26	65	42	-0.563304524	0.35342496	0.930050349
330578	'A530021J07Rik'	1	0	0	0	1	2	0.333333333	1	1.346160497	0.689889638	0.972790461
330627	'Trim66'	566	587	403	23	356	199	518.6666667	192.6666667	-1.523107877	0.041133329	0.475974951
330657	'Prss53'	44.25	48.92	34.98	27.01	79.21	181.87	42.71666667	96.03	1.111832799	0.07821171	0.614954936
330660	'Btbd16'	5	3	1	1	5	0	3	2	-0.516125385	0.766161042	0.972790461
330662	'Dock1'	1727	1722	914	375	1793	1602	1454.333333	1256.666667	-0.251400446	0.630694228	0.972790461
330671	'B4galnt4'	206	210	90	135	196	139	168.6666667	156.6666667	3.53E-04	0.999355217	0.999900097
330695	'Ctxn1'	663	704	560	342	783	570	642.3333333	565	-0.174471753	0.585182529	0.972790461
330721	'Nek5'	17	4	7	14	9	3	9.333333333	8.666666667	0.113503202	0.915353084	0.990364946
330723	'Htra4'	0	1	0	9	3	2	0.333333333	4.666666667	3.968991128	0.046821891	0.503906594
330788	'Zfp866'	1173	1260	430	454	1058	934	954.3333333	815.3333333	-0.18168803	0.690790716	0.972790461
330790	'Hapln4'	14	17	8	23	25	10	13	19.33333333	0.731523101	0.333690415	0.921648675
330812	'Rnf150'	216	152	293	491	166	296	220.3333333	317.6666667	0.68763293	0.308253313	0.908027422
330814	'Adgrl1'	5151	5135	1005	5135	7679	6294	3763.666667	6369.333333	0.887055021	0.130277735	0.727759547
330817	'Dhps'	249	251	398	283	337	562	299.3333333	394	0.371477684	0.414156805	0.95722888
330820	'4933402J07Rik'	0	1	1	0	0	0	0.666666667	0	-1.851176388	0.646290011	0.972790461
330830	'Drc7'	1	1	1	0	0	7	1	2.333333333	1.055751621	0.658718432	0.972790461
330836	'Slc7a6'	807.15	750.33	491.77	795.15	678	664	683.0833333	712.3833333	0.202573467	0.616970556	0.972790461
330863	'Trim67'	5	3	4	4	3	2	4	3	-0.297132264	0.801520617	0.972790461
330890	'Piwil4'	21	20	6	0	18	41	15.66666667	19.66666667	0.229639494	0.854231249	0.975734242
330908	'Opcml'	21	26	6	7	16	11	17.66666667	11.33333333	-0.56724275	0.463627009	0.972790461
330914	'Arhgap32'	2307	2173	1942	2080	1960	1878	2140.666667	1972.666667	-0.011367566	0.974920628	0.999493374
330921	'Pate2'	2	1	0	2	2	1	1	1.666666667	0.920964719	0.634264028	0.972790461
330938	'Dixdc1'	810	803	1370	3790	551	1223	994.3333333	1854.666667	1.157222646	0.173783014	0.79261444
330941	'AI593442'	7	7	3	2	7	1	5.666666667	3.333333333	-0.703138649	0.561044898	0.972790461
330953	'Hcn4'	111	83	37	14	52	35	77	33.66666667	-1.176285992	0.04705607	0.50504201
330959	'Snapc5'	191	205	114	26	210	196	170	144	-0.312873413	0.632822953	0.972790461
330962	'Slc51b'	0	1	0	0	0	0	0.333333333	0	-0.903279821	0.824807108	0.972790461
330963	'Ankdd1a'	7	13	1	2	8	1	7	3.666666667	-0.835052457	0.535976608	0.972790461
331004	'Slc9a9'	14	27	8	18	33	28	16.33333333	26.33333333	0.754428951	0.239506226	0.86449675
331026	'Gmppb'	140.3	161	407.76	105	177.66	285.04	236.3533333	189.2333333	-0.427424801	0.496761268	0.972790461

331046	'Tgm4'	10.05	3.85	9.08	0	12.48	2.43	7.66	4.97	-0.783418819	0.586682058	0.972790461
331063	'Gsdmc2'	0	0	0	2	1	1	0	1.333333333	3.039006733	0.327321392	0.920734892
331188	'Zfp781'	245.91	297	168.2	51.3	277.47	172.92	237.0366667	167.23	-0.545029978	0.320152094	0.917050664
331374	'Dgkk'	100	120	415	64	21	41	211.6666667	42	-2.253013001	0.007924052	0.214298825
331401	'Thoc2'	4441	4621	2394	5649	4455	4066	3818.666667	4723.333333	0.478923881	0.293184089	0.90029104
331416	'Gm773'	109	78	34	0	81	221	73.66666667	100.6666667	0.331268805	0.798358589	0.972790461
331461	'Il1rapl1'	2	11	2	0	12	2	5	4.666666667	-0.142845825	0.929438986	0.993114933
331474	'Rtl5'	201.19	184.11	141.17	64.43	173.96	121.41	175.49	119.9333333	-0.550347984	0.154973128	0.766453805
331487	'Uprt'	467	472	581	629	517	505	506.6666667	550.3333333	0.217736973	0.627800721	0.972790461
331491	'Fnd3c2'	0	1	0	1	1	1	0.333333333	1	1.558404368	0.565964169	0.972790461
331493	'Gm5127'	0	1	0	0	0	0	0.333333333	0	-0.903279821	0.824807108	0.972790461
331524	'Xkrx'	44	37	16	16	29	24	32.33333333	23	-0.423069866	0.456995225	0.971964785
331529	'Gm5128'	1049.1	985.1	83.14	3	464.29	756.63	705.7633333	407.9733333	-0.858363119	0.495434429	0.972790461
331531	'AV320801'	825.65	893.03	78.85	3	322.37	603.7	599.1766667	309.69	-1.02195177	0.405202098	0.957157474
331532	'Tceal5'	17.75	15.54	3.14	10.58	10.15	17.37	12.14333333	12.7	0.184796531	0.826182459	0.972844676
331535	'Serpina7'	2	6	0	0	3	0	2.666666667	1	-1.389581454	0.601501341	0.972790461
331537	'Dnaaf6b'	47	58	7	0	12	15	37.33333333	9	-2.098266228	0.069397358	0.58740482
331623	'Bend3'	662	663	461	144	535	639	595.3333333	439.3333333	-0.492162457	0.302155558	0.904943519
332110	'Mapk15'	8	5	2	11	9	4	5	8	0.880244881	0.400372575	0.955860942
332131	'Krt78'	10	15	2	1	10	21	9	10.66666667	0.19874379	0.863967544	0.977632919
332175	'Zdhhc23'	439	378	58	17	201	273	291.6666667	163.6666667	-0.879786451	0.333485452	0.921648675
332221	'Zscan10'	46	62	12	0	8	12	40	6.666666667	-2.63088762	0.011373726	0.26434876
332359	'Tigd3'	55	54	23	41	25	24	44	30	-0.351224017	0.584853309	0.972790461
332396	'Kcnk18'	2	1	0	2	38	88	1	42.66666667	5.31632911	2.53E-04	0.026705556
332397	'Nanos1'	145	126	162	11	115	320	144.3333333	148.6666667	-0.131232726	0.881440368	0.982528614
332427	'Lyg2'	0	1	1	0	0	0	0.666666667	0	-1.851176388	0.646290011	0.972790461
332579	'Card9'	6	7	0	3	7	15	4.333333333	8.333333333	0.973098837	0.450478948	0.970590732
332713	'Fndc11'	16	15	1	0	7	11	10.66666667	6	-0.873190937	0.538086533	0.972790461
332923	'Pramel19'	0	1	1	0	0	0	0.666666667	0	-1.851176388	0.646290011	0.972790461
332934	'Zmynd12'	36	43	6	2	20	31	28.33333333	17.66666667	-0.716937454	0.471104265	0.972790461
332937	'Tfap2e'	34	26	13	1	6	6	24.33333333	4.333333333	-2.487190653	0.002922108	0.120771992
332942	'Ldc1'	7	8	39	2	1	0	18	1	-4.124759644	0.004579438	0.159166866
333048	'Tmem211'	3	1	1	0	4	2	1.666666667	2	0.195756217	0.914336473	0.989964366
333050	'Ksr2'	23	23	2	14	12	33	16	19.66666667	0.39727156	0.670089502	0.972790461
333088	'Kcp'	651	727	135	891	873	541	504.3333333	768.3333333	0.818070452	0.212331914	0.84083615
333182	'Cox6b2'	161	171	66	30	126	185	132.6666667	113.6666667	-0.261549735	0.673273478	0.972790461
333193	'Proser3'	265	290	129	94	313	208	228	205	-0.140600158	0.76717809	0.972790461
333307	'Trim75'	1	3	0	5	12	41	1.333333333	19.33333333	3.822520871	0.003713883	0.140118993
333315	'Frem3'	2	5	0	1	2	6	2.333333333	3	0.390638651	0.815362675	0.972790461
333329	'Cngb1'	8	11	3	5	9	12	7.333333333	8.666666667	0.286794945	0.743214721	0.972790461

333424	'A4gnt'	0	0	0	1	0	0	0	0.333333333	1.020273531	0.802557913	0.972790461
333433	'Gpd1l'	382	430	541	1361	516	610	451	829	1.077385065	0.097738753	0.664560086
333467	'B020031M17Rik'	1	1	1	0	2	0	1	0.666666667	-0.629878411	0.810068044	0.972790461
333473	'Zfp3613'	9	5	1	0	3	1	5	1.333333333	-1.899848142	0.248188463	0.869491261
333564	'Fndc3c1'	56	63	31	93	33	54	50	60	0.497430315	0.462675636	0.972790461
333588	'Gm15104'	20	13	6	1	10	4	13	5	-1.382349106	0.174117633	0.793056387
333605	'Frmpd4'	27	35	14	1	19	8	25.33333333	9.333333333	-1.480018021	0.107561141	0.683205958
333639	'Mamld1'	438	450	682	213	651	486	523.3333333	450	-0.288535648	0.546428139	0.972790461
333654	'Ppp1r13l'	282	222	1287	578	237	339	597	384.6666667	-0.57090196	0.514405495	0.972790461
333669	'Gm5134'	8	5	1	0	3	2	4.666666667	1.666666667	-1.494551913	0.341669221	0.925308103
333670	'Gm867'	6	10	35	21	8	25	17	18	0.079348724	0.936156523	0.994413066
333789	'N4bp2'	2519.6	2836.4	1292.9	190.08	2968.5	1770.37	2216.3	1642.976667	-0.507211892	0.505911631	0.972790461
333883	'Cd59b'	4.17	7.19	0	10.05	11.12	10.09	3.786666667	10.42	1.655925287	0.150102254	0.759580531
338320	'Mia2'	999	948	1083	756	1359	2045	1010	1386.666667	0.419380114	0.258676179	0.877564912
338337	'Cog3'	1314	1216	2013	1180	1115	1097	1514.333333	1130.666667	-0.365750451	0.435234341	0.965765089
338346	'Gpr21'	7	11	2	3	4	3	6.666666667	3.333333333	-0.88058544	0.4274686	0.961831039
338348	'Ttc16'	138	171	27	16	123.02	63	112	67.34	-0.730969326	0.364697225	0.935515019
338349	'Cntln'	1086	1118	354	117	1209	889	852.6666667	738.3333333	-0.255506886	0.726680484	0.972790461
338350	'Acad12'	248.13	242.64	66.02	30.12	251	244.75	185.5966667	175.29	-0.123765899	0.868109793	0.979220701
338351	'Akap17b'	988	952	273	268	880	1433	737.6666667	860.3333333	0.205726631	0.740922668	0.972790461
338352	'Nell1'	270	277	42	4	573	405	196.3333333	327.3333333	0.676125329	0.562096238	0.972790461
338354	'Zfp780b'	533.27	534.34	445.32	412.79	571.98	492.36	504.31	492.3766667	0.024639757	0.930652833	0.993474751
338355	'Fkbp15'	1220	1201	742	1037	990	964	1054.333333	997	0.045860391	0.901853822	0.987163617
338359	'Supv3l1'	596	639	488	257	568	699	574.3333333	508	-0.194829119	0.564903339	0.972790461
338362	'Ust'	143	134	452	342	219	291	243	284	0.255802474	0.718817442	0.972790461
338363	'Tmem241'	197.44	187.09	43	212	252	262.39	142.51	242.13	0.899879659	0.1139469	0.698948431
338364	'Trim65'	168	167	75	45	180	136	136.6666667	120.3333333	-0.190359661	0.717108172	0.972790461
338365	'Slc41a2'	399.63	379	445	105	436	352	407.8766667	297.6666667	-0.530816683	0.277865663	0.889355605
338366	'Mia3'	1955	1839	1080	1411	2354	2594	1624.666667	2119.666667	0.426343647	0.151103997	0.75985382
338367	'Myo1d'	1030	944	1198	2688	1237	1543	1057.333333	1822.666667	0.956397185	0.096055207	0.662457477
338368	'Pheta2'	206	247	199	41	221	214	217.3333333	158.6666667	-0.535303944	0.334789877	0.922066514
338369	'Tmem220'	93.03	79.03	48	21.99	112	76	73.35333333	69.99666667	-0.107235862	0.854339693	0.975734242
338370	'Nalcn'	101	80	10	1	61	101	63.66666667	54.33333333	-0.295353143	0.801768703	0.972790461
338371	'Endov'	673	626	430	298	675	579	576.3333333	517.3333333	-0.140246354	0.659665317	0.972790461
338372	'Map3k9'	662	610	452	377	343	799	574.6666667	506.3333333	-0.144373317	0.725491311	0.972790461
338375	'Atp6v1g3'	0	0	0	1	0	0	0	0.333333333	1.020273531	0.802557913	0.972790461
338403	'Cndp1'	0	0	0	1	0	1	0	0.666666667	2.022653929	0.615796212	0.972790461
338417	'Scgb1c1'	0	1	0	0	0	0	0.333333333	0	-0.903279821	0.824807108	0.972790461
338467	'Morc3'	1510	1533	1368	1687	1541	1278	1470.333333	1502	0.151628097	0.7070969	0.972790461
338521	'Fa2h'	19	13	9	1337	11	177	13.66666667	508.3333333	5.666018515	9.89E-06	0.002540023

338523	'Kdm7a'	1371	1398	1850	6117	1385	1423	1539.666667	2975	1.2338289	0.125532982	0.72022881
347722	'Agap1'	2497	2359	4249	5528	1619	3048	3035	3398.333333	0.333488615	0.632678896	0.972790461
353025	'Caps2'	304	306	43	1	184	85	217.6666667	90	-1.324371115	0.262150634	0.88013169
353047	'Plekhm1'	633	612	378	1523	547	822	541	964	1.075207829	0.079457402	0.617305615
353130	'Prss33'	0	2	0	0	1	0	0.666666667	0.333333333	-0.733814424	0.856146772	0.975734242
353155	'Gjd3'	6	5	3	1	5	7	4.666666667	4.333333333	-0.14167626	0.89977874	0.986311889
353156	'Egfl7'	469	532	54	141	687	396	351.6666667	408	0.250245919	0.752128712	0.972790461
353169	'Slc2a12'	18	18	4	8	4	1	13.33333333	4.333333333	-1.32793121	0.244955091	0.866091613
353170	'Txlng'	1682	1792	759	679	1284	860	1411	941	-0.506607605	0.213836933	0.842446242
353172	'Gars'	2454	2386	5566	1628	2071	3039	3468.666667	2246	-0.680740441	0.209874012	0.837733513
353187	'Nr1d2'	1764	1883	1784	1588	1328	1664	1810.333333	1526.666667	-0.154094113	0.677745272	0.972790461
353188	'Adam32'	10	6	7	5	5	2	7.666666667	4	-0.822758453	0.414832426	0.95722888
353190	'Edc3'	1486.1	1430	1278.9	788.63	1199.3	1452.23	1398.316667	1146.71	-0.268069618	0.284657573	0.893377528
353208	'Zfp931'	273.46	287.38	185.66	114.8	266.68	247.92	248.8333333	209.8	-0.239738559	0.480860661	0.972790461
353211	'Prune2'	235	229	1108	647	212	322	524	393.6666667	-0.3153558	0.723023568	0.972790461
353234	'Pcdha2'	12.7	0	0	0	2.27	2.36	4.233333333	1.543333333	-1.608752259	0.537319658	0.972790461
353236	'Pcdhac1'	2.15	2.06	0	0	0	2.4	1.403333333	0.8	-0.99885442	0.740500622	0.972790461
353237	'Pcdhac2'	29.6	17.14	25.2	3.91	20.98	17.33	23.98	14.07333333	-0.908281982	0.231412342	0.858795911
353242	'Mrpl21'	412	455	511	153	368	486	459.3333333	335.6666667	-0.51329925	0.22273628	0.850163052
353258	'Ltv1'	889	901	545	435	707	816	778.3333333	652.6666667	-0.215611932	0.45125731	0.970649024
353282	'Sfmbt2'	278	211	411	190	272	280	300	247.3333333	-0.290959784	0.525327079	0.972790461
353287	'Clec18a'	3	4	0	1	3	0	2.333333333	1.333333333	-0.657680141	0.759343726	0.972790461
353310	'Zfp703'	1311	1298	1078	1055	1153	1274	1229	1160.666667	-0.007517571	0.979272796	0.999493374
353326	'Rtl1'	765	700	301	1059	588	345	588.6666667	664	0.446938625	0.501681557	0.972790461
353328	'Muc6'	21	9.1	3.06	8.06	7.04	6.03	11.05333333	7.043333333	-0.483160335	0.620814467	0.972790461
353344	'Opn5'	0	0	1	0	0	0	0.333333333	0	-0.903279821	0.824807108	0.972790461
353346	'Gpr141'	6	2	2	1	4	2	3.333333333	2.333333333	-0.497769016	0.715624233	0.972790461
353371	'Oxct2b'	2	3	1	4	0	1	2	1.666666667	0.080176663	0.965501972	0.999493374
353499	'Tmc4'	135	151	128	129	183	138	138	150	0.18130738	0.605865625	0.972790461
353502	'Hcfc1r1'	376.68	389.39	1084.8	1393.1	423.48	810.5	616.97	875.69	0.629465976	0.417534626	0.957720764
360013	'Myo18a'	970	1007	552	2687	1151	1517	843	1785	1.314964875	0.023384751	0.379052873
360198	'H3c1'	0	4.13	7.29	2	2	5.7	3.806666667	3.233333333	-0.371313551	0.812001514	0.972790461
360213	'Trim46'	119	104	482.98	602	106	230	235.3266667	312.6666667	0.572223581	0.551526614	0.972790461
360216	'Zranb1'	2701	2583	3003	1603	2012	1895	2762.333333	1836.666667	-0.544205418	0.098916743	0.668585731
360220	'Speer4d'	0	0	0	8.96	0	0	0	2.986666667	4.334487999	0.270322338	0.883851074
368202	'Prss48'	41	35	8	0	22	43	28	21.66666667	-0.45435738	0.711605551	0.972790461
368203	'Gm5136'	13	12	5	0	5	6	10	3.666666667	-1.500353758	0.194948078	0.819942118
368204	'Khdc1a'	1	2	0	0	0	4	1	1.333333333	0.350554987	0.906900408	0.988823459
373864	'Col27a1'	678	658	206	837	950	418	514	735	0.708302511	0.240897354	0.86449675
378425	'Nlrp12'	0	2	1	0	0	1	1	0.333333333	-1.42378721	0.670046855	0.972790461

378430	'Nanos2'	1	0	0	5	0	0	0.333333333	1.666666667	2.683799063	0.465173521	0.972790461
378431	'Txlnb'	51	41	11	15	76	68	34.33333333	53	0.619318126	0.398395761	0.954563638
378435	'Mafa'	76	80	13	34	27	7	56.33333333	22.66666667	-1.034865174	0.256337622	0.876684814
378460	'Pram1'	58	50	42	17	49	24	50	30	-0.722242491	0.171487894	0.787518803
378462	'Morn2'	266	313	73	16	161	171	217.3333333	116	-0.952622927	0.229095009	0.855540033
378466	'Gm10033'	435	485	116	248	509	362	345.3333333	373	0.198908345	0.715013865	0.972790461
378702	'Serf2'	3121.9	3513.2	4450.8	2246.7	2975.2	3734.53	3695.29	2985.466667	-0.307768196	0.39829907	0.954563638
378937	'Lrrc24'	49	49	35	28	36	23	44.33333333	29	-0.520843526	0.294577742	0.90029104
379043	'Raet1e'	49.39	42.98	134.94	108	118	162	75.77	129.3333333	0.743143567	0.254996022	0.874595535
380601	'Fastkd5'	290.43	257.82	200	157.7	261	278.85	249.4166667	232.5166667	-0.071971385	0.797428632	0.972790461
380608	'Tagap1'	645.51	676.77	593.74	737.42	739.96	577.22	638.6733333	684.8666667	0.211949156	0.589823827	0.972790461
380614	'Intu'	361.73	384.42	206.17	783.72	376.89	309.54	317.44	490.05	0.881810595	0.157655555	0.768337387
380629	'Heca'	1524	1459	1975	1837	1356	2015	1652.666667	1736	0.14109491	0.750956237	0.972790461
380654	'Cfap54'	688	642	114	98	766	660	481.3333333	508	0.057509275	0.941289182	0.996040262
380660	'Acss3'	85	76	2	33	46	44	54.33333333	41	-0.256060847	0.797230204	0.972790461
380664	'Lemd3'	799	766	511	641	888	802	692	777	0.240739816	0.400155577	0.955860942
380669	'Lin28b'	655	658	78	4	423	168	463.6666667	198.3333333	-1.268260669	0.270515331	0.883851074
380683	'Sec14l3'	0	0	1	0	1	1	0.333333333	0.666666667	0.818407084	0.819542986	0.972790461
380684	'Nefh'	161	169	62	1	71	167.96	130.6666667	79.98666667	-0.825561013	0.450784566	0.970590732
380686	'Cnrip1'	213	196	545	35	230	221	318	162	-1.157012492	0.120914191	0.712100232
380694	'Ccnjl'	776	864	286	237	595	411	642	414.3333333	-0.578450977	0.228636189	0.855540033
380698	'Obscn'	100	73	28	14	63	22	67	33	-0.989641609	0.161331322	0.771435531
380701	'Slc47a2'	9	6	3	1	14	2	6	5.666666667	-0.100172943	0.936666064	0.994413066
380702	'Shisa6'	680	689	193	95	1421	1322	520.6666667	946	0.799272377	0.330777266	0.921648675
380705	'Tmem102'	75	95	109	180	85	130	93	131.6666667	0.641121969	0.274383652	0.885667571
380709	'Spata22'	1307	1364	392	3	593	545	1021	380.3333333	-1.512488245	0.186840907	0.808483975
380711	'Rap1gap2'	299	342	151	577	212	156	264	315	0.567200307	0.439029184	0.967306223
380712	'Tlcd2'	136	138	38	58	146	115	104	106.3333333	0.085633558	0.877507008	0.981341203
380713	'Scarf1'	95	117	12	2	166	83	74.66666667	83.66666667	0.120740197	0.916924201	0.990536123
380714	'Rph3al'	437	493	628	266	628	694	519.3333333	529.3333333	-0.030798914	0.941210256	0.996040262
380718	'Mks1'	258	232	149	35	349	231	213	205	-0.130572179	0.847127194	0.975209338
380728	'Kcnh4'	5	2	2	0	1	0	3	0.333333333	-2.982999026	0.159874685	0.771114735
380730	'Gm884'	209	177.94	6	1	92.96	145.82	130.98	79.92666667	-0.774563756	0.555330449	0.972790461
380732	'Milr1'	8	9	4	14	11	13	7	12.66666667	0.983256869	0.213542746	0.842297652
380752	'Eipr1'	339	422	198	117	339	451	319.6666667	302.3333333	-0.101681178	0.829941871	0.973823048
380753	'Atxn7l1'	355	394	288	600	464	372	345.6666667	478.6666667	0.629957072	0.179167404	0.798783498
380755	'Lsmem1'	0	1	0	4	0	1	0.333333333	1.666666667	2.561069105	0.38436112	0.946686568
380768	'Ccdc177'	117	109	15	1	41	33	80.33333333	25	-1.727446683	0.101617564	0.671759705
380773	'Slirp'	276	256	259	101	183	240	263.6666667	174.6666667	-0.608348845	0.072826661	0.596837647
380780	'Serpina11'	0	1	2	0	0	0	1	0	-2.490290069	0.532417892	0.972790461

380785	'Begain'	205	203	204	41	170	182	204	131	-0.716825896	0.157621028	0.768337387
380787	'Lbhd2'	49	58	18	41	48	33	41.66666667	40.66666667	0.10912078	0.853412252	0.975734242
380836	'Mrs2'	1377.7	1249.7	1109.6	1662.7	1254.9	1040.33	1245.67	1319.283333	0.238271346	0.607048661	0.972790461
380839	'Serpinb1c'	0	3	0	2	1	2	1	1.666666667	0.936934731	0.681104955	0.972790461
380840	'Lyrm4'	215	232	329	78	210	300	258.6666667	196	-0.493694283	0.333089116	0.921648675
380842	'Stmnd1'	2	1	0	0	0	0	1	0	-2.286544758	0.567674219	0.972790461
380845	'Gm904'	0	0	0	0	0	5	0	1.666666667	3.001401729	0.449669959	0.97049895
380850	'Gm5141'	145	147.01	51.62	45.76	170	90.97	114.5433333	102.2433333	-0.143233206	0.80465554	0.972790461
380855	'Rsl1'	115.07	104.96	138.89	95	145	167.72	119.64	135.9066667	0.185639121	0.626023086	0.972790461
380863	'Tmem171'	30	32	19	2	126	109	27	79	1.431313255	0.164095465	0.77683839
380878	'AF067063'	0	0	0	0	1	0	0	0.333333333	1.020273531	0.802557913	0.972790461
380882	'Gm906'	0	1.57	0	0	1.47	4.07	0.523333333	1.846666667	2.065845676	0.501717296	0.972790461
380912	'Zfp395'	2611	2396	6686	546	2297	2470	3897.666667	1771	-1.303287151	0.052664573	0.532526438
380916	'Lrch1'	281	284	818	547	288	405	461	413.3333333	-0.091750857	0.895997587	0.98557661
380918	'Siah3'	3	3	0	7	1	1	2	3	0.995565269	0.578907267	0.972790461
380921	'Dgkh'	761	770	367	389	657	644	632.6666667	563.3333333	-0.107295474	0.758364166	0.972790461
380924	'Olfm4'	12	39	10	21	55	21	20.33333333	32.33333333	0.741182478	0.336711152	0.923078936
380928	'Lmo7'	309	360	158	320	586	1018	275.6666667	641.3333333	1.224986089	0.006426875	0.190013707
380959	'Alg10b'	855	777	453	620	895	948	695	821	0.305852212	0.318074533	0.916136994
380967	'Tmem106c'	954.31	996.65	537.86	358.46	973.83	1312.36	829.6066667	881.55	0.066211965	0.880186128	0.982087554
380969	'Nckap5l'	991	965	652	923	1411	948	869.3333333	1094	0.412169331	0.222017016	0.849517424
380993	'Zfat'	296	285	216	378	298	262	265.6666667	312.6666667	0.385603652	0.389658612	0.951231321
381022	'Kmt2d'	4517	4386	1765	5768	5380	3773	3556	4973.666667	0.666116692	0.188189178	0.81021541
381038	'Parl'	609	639	490	247	782	1429	579.3333333	819.3333333	0.423691255	0.422648069	0.95973972
381045	'Ccdc58'	309	348	846	60	254	286	501	200	-1.487275683	0.028464786	0.410671038
381058	'Unc93a'	1.1	6	5.12	0	1	2.15	4.073333333	1.05	-2.101554984	0.220586514	0.849076011
381059	'Gm1604b'	3	5	2	0	2	2	3.333333333	1.333333333	-1.36155604	0.39089659	0.952178913
381062	'Ermard'	896.96	913.38	275.59	124.59	636.34	342.97	695.31	367.9666667	-0.913658918	0.140270741	0.74306325
381066	'Zfp948'	322	345	722	423	316	198	463	312.3333333	-0.465702284	0.477357416	0.972790461
381067	'Zfp229'	580	610	302	131	574	545	497.3333333	416.6666667	-0.289274645	0.576139536	0.972790461
381072	'Abca17'	16	14	3	1	13	8	11	7.333333333	-0.598726035	0.579124466	0.972790461
381073	'Npw'	3	1	5	1	2	0	3	1	-1.573319788	0.381895845	0.94429001
381077	'Ccdc78'	1	0	4	0	1	1	1.666666667	0.666666667	-1.520376382	0.537060838	0.972790461
381085	'Tbc1d22b'	657	600	476	722	628	713	577.6666667	687.6666667	0.363923115	0.318436179	0.916136994
381091	'H2-Eb2'	2	0	0	0	0	0	0.666666667	0	-1.711373851	0.67234292	0.972790461
381101	'Dnph1'	83	76	58	13	77	69	72.33333333	53	-0.513234914	0.391577003	0.952648938
381104	'Prickle4'	55	52	23	44	60	31	43.33333333	45	0.183895952	0.746520993	0.972790461
381107	'Tmem232'	1	3	0	1	2	10	1.333333333	4.333333333	1.683355582	0.335298332	0.922335099
381110	'Rmdn2'	943	1149	493	326	1210	871	861.6666667	802.3333333	-0.105470108	0.826127537	0.972844676
381112	'Arhgef33'	201	199	56	66	149	104	152	106.3333333	-0.442602671	0.417679896	0.957720764

381113	'Cdkl4'	25	18	10	5	21	9	17.66666667	11.66666667	-0.579950491	0.454431805	0.970649024
381122	'Capn13'	0	0	1	0	0	0	0.333333333	0	-0.903279821	0.824807108	0.972790461
381126	'Garem1'	327	357	424	278	239	229	369.3333333	248.6666667	-0.478871964	0.283790178	0.892170588
381142	'Arl14epl'	122	124	28	23	73	238	91.33333333	111.3333333	0.241283923	0.768525992	0.972790461
381148	'Prob1'	83	82	45	134	62	37	70	77.66666667	0.427083922	0.557268519	0.972790461
381157	'Greb1l'	2061	2021	353	664	2242	1378	1478.333333	1428	0.003450966	0.995829304	0.999562152
381175	'Ccdc68'	24	17	28	26	32	20	23	26	0.236067372	0.701963165	0.972790461
381196	'Gm960'	125	126	48	6	36	12	99.66666667	18	-2.45520378	4.90E-04	0.04295735
381199	'Tmem151a'	216	239	157	137	225	344	204	235.3333333	0.213815085	0.544390207	0.972790461
381201	'Ap5b1'	180	174	95	57	174	318	149.6666667	183	0.24275998	0.663849051	0.972790461
381203	'Slc22a20'	0	0	2	0	0	0	0.666666667	0	-1.995285526	0.619817042	0.972790461
381204	'Naaladl1'	8	6	1	1	5	11	5	5.666666667	0.158334162	0.899382634	0.986106073
381213	'Ms4a12'	1	2	0	0	0	0	1	0	-2.28103714	0.568641838	0.972790461
381217	'Fam189a2'	107	138	47	68	161	158	97.33333333	129	0.439435074	0.361029092	0.933706718
381218	'4430402I18Rik'	30.75	44.24	23.71	13.86	26.51	65.95	32.9	35.44	0.06471997	0.920741805	0.991047523
381229	'Cfap58'	3	0	0	0	0	0	1	0	-2.292035055	0.566710385	0.972790461
381236	'Lipo3'	275.23	211.36	151.98	9	325.13	179.4	212.8566667	171.1766667	-0.417596858	0.643406539	0.972790461
381246	'Xkr9'	3	2	0	2	0	0	1.666666667	0.666666667	-0.82444629	0.773940896	0.972790461
381259	'Tmem237'	281	302	346	61	425	407	309.6666667	297.6666667	-0.177785952	0.775834706	0.972790461
381260	'Gm973'	31	30	6	1	22	79	22.33333333	34	0.518934205	0.656577002	0.972790461
381269	'Mreg'	116	105	172	54	64	130	131	82.66666667	-0.703219054	0.169029087	0.784741705
381270	'Marchf4'	5	5	1	2	1	1	3.666666667	1.333333333	-1.266970692	0.398863316	0.954941362
381280	'Hjurp'	2997.7	2796.8	1948.2	1123.7	2195.1	2819.51	2580.883333	2046.11	-0.333314922	0.29559736	0.90029104
381284	'Crocc2'	40	47	4	6	49	12	30.33333333	22.33333333	-0.408055373	0.692342003	0.972790461
381287	'A530032D15Rik'	1	0	0	0	0	4	0.333333333	1.333333333	1.721579076	0.665789268	0.972790461
381290	'Atp2b4'	1926	1633	726	3695	1869	2424	1428.333333	2662.666667	1.122922474	0.049285068	0.515210762
381293	'Kif14'	408	360	44	40	367	755	270.6666667	387.3333333	0.466707338	0.62267912	0.972790461
381305	'Rc3h1'	2128	2331	2105	2563	2115	2394	2188	2357.333333	0.213989184	0.576203184	0.972790461
381306	'BC055324'	1122.6	1114.6	442.01	131.22	628.95	654.29	893.05	471.4866667	-0.954210466	0.09741885	0.663789939
381308	'Ifi211'	4.46	4.99	238.93	13.41	5	43.49	82.79333333	20.63333333	-2.236242781	0.127824095	0.723917691
381310	'Stum'	668	797	95	100	740	365	520	401.6666667	-0.361654759	0.657275556	0.972790461
381314	'Iars2'	2378.4	2364.3	1371.3	1945.5	2456.4	2697.37	2038.006667	2366.416667	0.294584603	0.322119613	0.918054015
381318	'Nsl1'	289	290.21	77	36.88	224	206	218.7366667	155.6266667	-0.516686738	0.454377866	0.970649024
381319	'Batf3'	6	2	6	28	2	2	4.666666667	10.66666667	1.557468527	0.271446107	0.884495016
381334	'Gal3st2'	0.89	1	0	0	2.29	3	0.63	1.763333333	2.081165771	0.487041593	0.972790461
381337	'Fam178b'	238	213	35	2	115	196	162	104.3333333	-0.709668598	0.525479974	0.972790461
381338	'Lonrf2'	1149	1086	286	5	762	954	840.3333333	573.6666667	-0.639630474	0.57822122	0.972790461
381339	'Tmem182'	9	6	16	0	4	1	10.33333333	1.666666667	-2.750442446	0.038414496	0.459930654
381350	'Spag6'	47	50	9	3	32	8	35.33333333	14.33333333	-1.29038988	0.184614521	0.80485988
381352	'Mamdc4'	79	73	38	13	97	33	63.33333333	47.66666667	-0.433970161	0.543145216	0.972790461

381353 'Ajm1'	39	52	15	34	56	62	35.33333333	50.66666667	0.588023598	0.280162765	0.889355605
381356 'Cacfd1'	1121	1166	2629	789	1141	1290	1638.666667	1073.333333	-0.658099911	0.217887181	0.844165262
381359 'Prdm12'	1	2	4	0	0	1	2.333333333	0.333333333	-2.718793528	0.233902032	0.860643592
381373 'Sp9'	34	35	41	2	20	38	36.66666667	20	-1.014385675	0.229290009	0.855596006
381375 'Pjvk'	2	13	1	0	11	4	5.333333333	5	-0.122493888	0.939875237	0.995607401
381379 'Med19'	821.28	822.75	709.09	292.96	684.47	820.99	784.3733333	599.4733333	-0.417719179	0.230049552	0.856721174
381390 'Gm14147'	0	0	0	0	0	2.86	0	0.953333333	1.749449488	0.66566965	0.972790461
381393 '4921509C19Rik'	3	2.08	0	0	1	0	1.693333333	0.333333333	-2.062237612	0.490675181	0.972790461
381399 'Bpifb4'	7	8	0	2	74	59	5	45	3.128093207	0.016854382	0.324656048
381404 'Pabpc1l'	2	4	3	12	9	4	3	8.333333333	1.641561111	0.134971943	0.735841445
381405 'Zfp663'	1	1	0	1	0	0	0.666666667	0.333333333	-0.746871675	0.831393929	0.974324192
381406 'Trp53rka'	458.52	505.15	731.7	300.05	389.13	435.34	565.1233333	374.84	-0.5889539	0.152208469	0.762582916
381409 'Cdh26'	0	2	0	0	52	56	0.666666667	36	5.668106898	0.003594252	0.13804834
381410 'Zfp408'	702	676	711	419	802	607	696.3333333	609.3333333	-0.180529071	0.571444494	0.972790461
381411 'Accsl'	1	0	1	61	2	11	0.666666667	24.66666667	5.564304187	0.001135916	0.070791417
381413 'Gpr176'	6	4	105	21	20	16	38.33333333	19	-1.09126711	0.379808965	0.943232396
381417 'Slc28a2b'	5.77	3.33	0	0	0	1	3.033333333	0.333333333	-2.738196695	0.314413392	0.914150085
381418 'Ctxn2'	1	1	0	0	0	0	0.666666667	0	-1.703500596	0.673820112	0.972790461
381438 'Gm5148'	0	1.31	0	1.27	0	3.71	0.436666667	1.66	1.90582342	0.544620118	0.972790461
381463 'Nr1h5'	3	5	3	1	1	0	3.666666667	0.666666667	-2.33227114	0.176697518	0.796450095
381476 'Stpg2'	23	25	5	0	16	28	17.66666667	14.66666667	-0.346090994	0.779736637	0.972790461
381484 'Gm5150'	1	1	0	0	2	3	0.666666667	1.666666667	1.273050587	0.596162899	0.972790461
381485 'Trim55'	2	2	1	0	1	0	1.666666667	0.333333333	-2.12680111	0.38057625	0.944042914
381489 'Rxfp1'	1	2	4	10	1	2	2.333333333	4.333333333	1.147109673	0.45280128	0.970649024
381493 'S100a7a'	2	2	5	26	3	6	3	11.66666667	2.223004875	0.086212492	0.637027553
381510 'Dpy19l4'	1289	1311	682	1145	1680	1712	1094	1512.333333	0.537584044	0.088476115	0.643745845
381511 'Pdp1'	974	1006	736	985	861	1053	905.3333333	966.3333333	0.200796923	0.56602602	0.972790461
381522 'Ccdc180'	5	10	0	2	6	1	5	3	-0.60333134	0.705555074	0.972790461
381530 'Mup20'	0	1	0	0	2	2	0.333333333	1.333333333	1.768901033	0.57287116	0.972790461
381531 'Mup21'	0	2	1	0	0	0	1	0	-2.386119369	0.550310572	0.972790461
381534 'Ube2u'	0	7	2	0	5	3	3	2.666666667	-0.251531327	0.898676458	0.986013206
381538 'Mroh7'	17	22	8	3	53	18	15.66666667	24.66666667	0.60585462	0.529145783	0.972790461
381544 'Armh1'	11	15	3	3	16	8	9.666666667	9	-0.079986839	0.934161066	0.994249017
381546 'Ccdc24'	85.15	48.85	59.54	4.73	74.22	52.85	64.51333333	43.93333333	-0.679034013	0.41451884	0.95722888
381549 'Zfp69'	167	176	93	43	131	95	145.3333333	89.66666667	-0.686169359	0.123613803	0.715417238
381560 'Xkr8'	161	174	86	594	317	335	140.3333333	415.3333333	1.786651925	0.001606066	0.085771147
381569 'Pramel26'	0	0	0	0	0	1	0	0.333333333	1.020273531	0.802557913	0.972790461
381570 'Oog2'	0	0	0	0	1	0	0	0.333333333	1.020273531	0.802557913	0.972790461
381580 'Ccdc27'	2	2	0	0	4	2	1.333333333	2	0.563683986	0.792433478	0.972790461
381582 'Tmem240'	28	38	33	12	46	38	33	32	-0.091250588	0.873716536	0.980171118

381591	'L1td1'	41	19	27	95	72	114	29	93.66666667	1.777171754	9.80E-04	0.065141401
381605	'Tbc1d2'	42	33	52	65	29	48	42.33333333	47.33333333	0.289683235	0.655401387	0.972790461
381622	'5031410I06Rik'	95.67	114.11	11.78	220.48	101.11	199.61	73.85333333	173.7333333	1.476883046	0.068557201	0.583886121
381626	'Rbm33'	2396	2336	2088	2404	2214	1320	2273.333333	1979.333333	-0.054268112	0.909259351	0.98928916
381628	'Adgrf3'	5	2	3	4	2	0	3.333333333	2	-0.489493961	0.760553266	0.972790461
381629	'Atraid'	555	576	353	366	656	678	494.6666667	566.6666667	0.2294876	0.423205528	0.96014123
381633	'Gm1673'	141	137	98	45	125	159	125.3333333	109.6666667	-0.222871064	0.612589978	0.972790461
381634	'Gm1043'	28	25	2	0	20	4	18.33333333	8	-1.214913031	0.393459085	0.953178011
381644	'Cep135'	385	354	228	80	409	227	322.3333333	238.6666667	-0.465365816	0.382924449	0.945352503
381651	'Odaph'	1	1	2	0	2	0	1.333333333	0.666666667	-1.088957393	0.659967391	0.972790461
381654	'Pramel34'	1	0	0	0	1	4.29	0.333333333	1.763333333	2.065879757	0.50146354	0.972790461
381667	'Lrcol1'	3	1	1	0	2	0	1.666666667	0.666666667	-1.328351342	0.580458721	0.972790461
381668	'Fbrsl1'	952	965	989	1162	1237	1151	968.6666667	1183.333333	0.366213385	0.296408006	0.90029104
381677	'Vgf'	2	9	16	22	6	3	9	10.33333333	0.424233042	0.733663947	0.972790461
381678	'Zcwpw1'	5067.9	4743.6	1635.8	204	2208.7	2557.68	3815.76	1656.803333	-1.271078346	0.095774649	0.662411648
381680	'Nxpe5'	1	1	0	0	0	0	0.666666667	0	-1.703500596	0.673820112	0.972790461
381686	'Kpna7'	6	8	9	1	2	12	7.666666667	5	-0.728908869	0.529033632	0.972790461
381693	'Wdr95'	1	1	1	1	1	1	1	1	0.063933044	0.973473023	0.999493374
381694	'B3glct'	694	629	168	632	711	701	497	681.3333333	0.599232064	0.265028109	0.88128484
381695	'N4bp2l2'	2074.2	2179.8	1556.3	1739.3	2179.3	2053.4	1936.746667	1990.676667	0.117350879	0.671767659	0.972790461
381714	'Gm9758'	0	0	0	111.42	0	36.88	0	49.43333333	8.406114847	0.031488045	0.430994029
381716	'Pttg1ip2'	0	0	0	0	0	1	0	0.333333333	1.020273531	0.802557913	0.972790461
381738	'Drc1'	13	8	18	5	2	27	13	11.33333333	-0.301604831	0.778278814	0.972790461
381741	'Lrrc43'	9	9	1	0	1	1	6.333333333	0.666666667	-3.198114653	0.063086148	0.566852792
381759	'Wee2'	4	3	0	1	4	31	2.333333333	12	2.297562078	0.144170422	0.750780289
381760	'Ssbp1'	867	921	708	327	910	990	832	742.3333333	-0.197493938	0.600297242	0.972790461
381785	'Gm1070'	38	21	3	8	11	57	20.66666667	25.33333333	0.291118459	0.77965316	0.972790461
381798	'4930590J08Rik'	39	51	12	22	22	19	34	21	-0.539595642	0.426202308	0.960958977
381801	'Tatdn2'	903	920	1060	804	663	1139	961	868.6666667	-0.097317891	0.807840233	0.972790461
381802	'Tsen2'	323	297	285	102	271	327	301.6666667	233.3333333	-0.414707854	0.299985834	0.903723114
381809	'Clec4b2'	1	0	0	0	0	0	0.333333333	0	-0.903279821	0.824807108	0.972790461
381810	'Lpar5'	15	19	6	4	10	7	13.33333333	7	-0.875004589	0.285911701	0.893820325
381812	'Cracr2a'	77	95.53	23	30.21	145	99	65.17666667	91.40333333	0.496366376	0.457223168	0.972232063
381813	'Prmt8'	1	1	7	1	9	27	3	12.33333333	1.77861181	0.21214852	0.84083615
381820	'Smim10l1'	868	904	1251	304	1143	1046	1007.666667	831	-0.372415948	0.456918708	0.971964785
381823	'Apold1'	250	271	88	10	381	233	203	208	-0.039660465	0.966425822	0.999493374
381835	'Sbk3'	22	15	5	57	19	22	14	32.66666667	1.520416607	0.085223012	0.634259952
381836	'Sbk2'	48	46	19	33	28	48	37.66666667	36.33333333	0.05145503	0.926091276	0.992381554
381838	'Vmn2r43'	1	1	0	0	2	0	0.666666667	0.666666667	0.038821536	0.990916081	0.999493374
381845	'Rnf225'	25	12	39	55	16	11	25.33333333	27.33333333	0.338094797	0.729037189	0.972790461

381853	'Gipr'	88	44	256	15	10	9	129.3333333	11.33333333	-3.485645213	5.54E-05	0.008769529
381867	'Ovol3'	3	0	0	3	3	0	1	2	1.281062966	0.6344806	0.972790461
381884	'Slc6a16'	93	97	10	1	26	39	66.66666667	22	-1.646834529	0.128303259	0.724799196
381903	'Alg8'	391	391	132	57	353	288	304.6666667	232.6666667	-0.417215948	0.51562549	0.972790461
381917	'Dnah3'	3	7	1	10	2	4	3.666666667	5.333333333	0.842943336	0.521065205	0.972790461
381921	'Taok2'	2734	2771	989	1414	2862	2090	2164.666667	2122	0.04463039	0.920194052	0.990979973
381922	'Cdiptos'	56	60	25	22	40	77	47	46.33333333	-0.012573421	0.982261158	0.999493374
381924	'Itgad'	2	0	1.01	0	3	0	1.003333333	1	-0.098404526	0.974318167	0.999493374
381925	'Plpp4'	1	1	11	0	2	1	4.333333333	1	-2.345166946	0.217391208	0.843822303
381933	'Spef1l'	32	34	6	1	14	23	24	12.66666667	-0.96688781	0.348910199	0.928798754
381944	'Usp17lb'	1	2	0	0	0	1.03	1	0.343333333	-1.323989397	0.695217833	0.972790461
381959	'Gm1096'	2	2	0	0	2	0	1.333333333	0.666666667	-0.95060596	0.752126908	0.972790461
381979	'Brsk1'	180.94	173.99	482.96	99.99	160.92	153	279.2966667	137.97	-1.081052438	0.075188613	0.604547452
381983	'Lmtk3'	211	203	90	455	327	123	168	301.6666667	1.098549745	0.110041941	0.689784826
381990	'Zbtb2'	1596	1663	565	1482	1602	1053	1274.666667	1379	0.283602093	0.582628033	0.972790461
382010	'Cep44'	283.16	255.21	210.04	174.07	276.05	272.09	249.47	240.7366667	-0.014098382	0.958646043	0.999493374
382014	'Ano8'	371	397	163	205	493	314	310.3333333	337.3333333	0.173165558	0.696077779	0.972790461
382018	'Unc13a'	202	166	119	21	87	19	162.3333333	42.33333333	-1.922618972	0.003318515	0.131661584
382019	'Zfp882'	199	214	87	53	259	203	166.6666667	171.6666667	0.021476512	0.969939816	0.999493374
382030	'Cnep1r1'	737	777	689	497	822	794	734.3333333	704.3333333	-0.037316597	0.883258646	0.983244267
382034	'Gse1'	743	768	1200	848	814	932	903.6666667	864.6666667	-0.023364329	0.958838246	0.999493374
382038	'Urb2'	1349	1371	559	450	836	856	1093	714	-0.56061182	0.157071298	0.768337387
382044	'Ces1b'	0	0	0	0	2	0	0	0.666666667	1.801491674	0.656112935	0.972790461
382045	'Adgrg5'	4	1	0	2	0	1	1.666666667	1	-0.415026058	0.860598533	0.976863639
382051	'Pdp2'	435	480	212	323	494	415	375.6666667	410.6666667	0.214642785	0.567809113	0.972790461
382053	'Ces3a'	0	0	0	0	1	0	0	0.333333333	1.020273531	0.802557913	0.972790461
382056	'Crtc1'	1621	1700	729	396	1958	1434	1350	1262.666667	-0.119423902	0.826656211	0.972859382
382062	'AB124611'	21	26	6	6	19	20	17.66666667	15	-0.212324058	0.784168797	0.972790461
382064	'Gm1110'	1	3	1	0	3	2	1.666666667	1.666666667	-0.059382788	0.974315259	0.999493374
382066	'Prdm10'	556	592	373	601	533	374	507	502.6666667	0.147830394	0.748033378	0.972790461
382073	'Ccdc84'	96	99	121.01	147	135.01	95	105.3366667	125.67	0.359808069	0.477401824	0.972790461
382074	'Foxr1'	1	0	1	0	0	10	0.666666667	3.333333333	2.09346034	0.457573244	0.972516785
382075	'Odf3l1'	1	2	0	0	1	0	1	0.333333333	-1.324111362	0.694133627	0.972790461
382077	'Ccdc33'	6	2	3	1	1	1	3.666666667	1	-1.807148683	0.236598813	0.862272407
382083	'Snx22'	37.24	45.32	32.44	35.02	64.35	62.41	38.33333333	53.92666667	0.522000799	0.204322887	0.833158723
382088	'Omt2b'	0	0	0	0	0	2	0	0.666666667	1.749449488	0.66566965	0.972790461
382090	'Cep162'	1173	1170	457	256	834	579	933.3333333	556.3333333	-0.725845474	0.136279057	0.736689633
382097	'Gm1123'	1	0	1	1	0	1	0.666666667	0.666666667	0.092317552	0.973134126	0.999493374
382099	'Gm5161'	0	2	0	0	4	1	0.666666667	1.666666667	1.304106706	0.660129395	0.972790461
382105	'Fbxw15'	1	1	0	0	1	4	0.666666667	1.666666667	1.259479367	0.611247353	0.972790461

382106	'Fbxw24'	0	1	0	0	4	7	0.333333333	3.666666667	3.207671723	0.208905218	0.836616817
382109	'Fbxw26'	0	0	1	0	0	19	0.333333333	6.333333333	3.95630884	0.20615936	0.833294914
382111	'Susd5'	12	3	6	0	4	5	7	3	-1.318770184	0.319470304	0.9165187
382113	'Slc22a14'	3	4	2	0	1	3	3	1.333333333	-1.23350605	0.456500041	0.97151713
382117	'Tcaim'	229	224	179.95	137.44	243	200	210.9833333	193.48	-0.088408442	0.765763661	0.972790461
382118	'Zkscan7'	632	683	289	160	730	494	534.6666667	461.3333333	-0.225579151	0.67233808	0.972790461
382137	'Fdxacb1'	205	226	115	88	183	180	182	150.3333333	-0.245441624	0.506366848	0.972790461
382156	'Fbxw22'	0	0	0	0	0	2	0	0.666666667	1.749449488	0.66566965	0.972790461
382207	'Jade3'	3843	3787	1587	443	2167	2265	3072.333333	1625	-0.955568405	0.094123924	0.658197981
382217	'Gm1140'	259.22	132.34	218	0	27.25	144.84	203.1866667	57.36333333	-2.029793902	0.101229338	0.671759705
382231	'8030474K03Rik'	1523.2	1394.5	263.26	9	844	751.37	1060.34	534.79	-1.056314035	0.339481166	0.924888762
382236	'Brwd3'	784	815	934	998	934	937	844.3333333	956.3333333	0.260613479	0.511036162	0.972790461
382243	'Gm10439'	5.75	0	2.22	0	0	0	2.656666667	0	-3.604535805	0.305926729	0.907188578
382244	'Gm15091'	1.26	3.94	0.78	0	0	1	1.993333333	0.333333333	-1.735451621	0.587572465	0.972790461
382245	'Tmem29'	162	210	93	34	121	114	155	89.66666667	-0.801301042	0.109478416	0.689773582
382252	'Bclaf3'	1252	1284	530	123	852	947	1022	640.6666667	-0.732146735	0.26157448	0.87954257
382253	'Cdkl5'	101	90	71	115	92	121	87.33333333	109.3333333	0.431581196	0.303972745	0.906771226
382265	'Cldn34c3'	0	0	0	0	2.14	0	0	0.713333333	1.801491674	0.656112935	0.972790461
382282	'Rhox12'	0	1	0	37	0	6	0.333333333	14.33333333	5.725277386	0.010639683	0.255084633
382384	'Odf3l2'	0	0	1	1	1	0	0.333333333	0.666666667	1.080329848	0.755640467	0.972790461
382395	'Gm5174'	14.51	9.14	5.94	6.49	9.99	3.61	9.863333333	6.696666667	-0.516797988	0.582527331	0.972790461
382406	'Poc1b'	310	284	309	214	286	274	301	258	-0.181710525	0.566563399	0.972790461
382423	'Atxn7l3b'	6126	6110	5479	3570	5300	5153	5905	4674.333333	-0.299987174	0.210648026	0.839637916
382427	'Best3'	1	1	0	2	5	6	0.666666667	4.333333333	2.724275037	0.106342667	0.679124905
382523	'H3f4'	14	8	4	6	7	5	8.666666667	6	-0.411190128	0.653934739	0.972790461
382543	'Ankfn1'	23	20	2	0	16	4	15	6.666666667	-1.190351931	0.394122901	0.953178011
382551	'Cd300ld3'	0	2	1.65	0	2	2	1.216666667	1.333333333	0.305984104	0.896718385	0.985742119
382562	'Pfn4'	91	83	18	1	88	57	64	48.66666667	-0.457312848	0.677466975	0.972790461
382571	'Kcnf1'	175	200	82	3	189	356	152.3333333	182.6666667	0.145607133	0.891210818	0.985096898
382620	'Tmed8'	1500	1552	656	641	1372	1002	1236	1005	-0.240760433	0.553063824	0.972790461
382639	'Zbtb42'	76	59	95	44	69	81	76.66666667	64.66666667	-0.261462422	0.565779166	0.972790461
382793	'Mtx3'	1060	1038	292	210	1130	539	796.6666667	626.3333333	-0.33741445	0.601770825	0.972790461
382864	'Colq'	57	51	15	36	66	31	41	44.33333333	0.22847808	0.722511463	0.972790461
382867	'Zfp488'	670	746.53	241.53	314.27	1116.9	545.11	552.6866667	658.7733333	0.289185967	0.601444059	0.972790461
382913	'Neil2'	129	114	136	32	107	213	126.3333333	117.3333333	-0.213840617	0.722779185	0.972790461
382985	'Rrm2b'	443	432	617	335	474	476	497.3333333	428.3333333	-0.209705772	0.585205757	0.972790461
383075	'Enthd1'	89	88	9	0	30	50	62	26.66666667	-1.282603938	0.310876319	0.910971113
383103	'Tvp23a'	238	254	60	143	224	152	184	173	0.036768688	0.948388036	0.997386511
383258	'Vmn2r118'	2	1	0	0	0	0	1	0	-2.286544758	0.567674219	0.972790461
383295	'Ypel5'	1194	1196	1911	2557	1147	1966	1433.666667	1890	0.518236184	0.378477953	0.941958743

383348	'Kctd16'	11	9	442	7	0	9	154	5.333333333	-4.929578484	0.001760642	0.090468437
383435	'Ms4a14'	2	3	0	3	5	1	1.666666667	3	1.020154437	0.5413987	0.972790461
383491	'Prdm14'	1	2	3	0	1	0	2	0.333333333	-2.474827987	0.289245912	0.896355874
383563	'Gpr25'	6	6	8	2	0	2	6.666666667	1.333333333	-2.237320445	0.102117596	0.671776916
383592	'Kif28'	0	1	0	1	1	0	0.333333333	0.666666667	1.081692101	0.760081965	0.972790461
383619	'Aim2'	114	119	75	115	40	17	102.6666667	57.33333333	-0.51716104	0.539979777	0.972790461
383678	'Obp2b'	2	3	1	0	2	0	2	0.666666667	-1.574167598	0.489121124	0.972790461
383709	'Gm1322'	0	0	0	1	0	0	0	0.333333333	1.020273531	0.802557913	0.972790461
383766	'Tldc2'	59	80	17	14	31	18	52	21	-1.217688071	0.072934479	0.59727171
383787	'Ankrd63'	14	10	1	54	12	12	8.333333333	26	2.032583588	0.074699841	0.603900623
384009	'Glipr2'	404	367	1665	279	253	250	812	260.6666667	-1.656577033	0.028244157	0.409827099
384059	'Tlr12'	4	8	1	0	24	5	4.333333333	9.666666667	1.114714898	0.473126268	0.972790461
384061	'Fndc5'	1456	1674	140	162	2335	1583	1090	1360	0.297826246	0.75245503	0.972790461
384071	'Slc25a34'	4	2	3	6	11	12	3	9.666666667	1.693372034	0.077334893	0.611392561
384185	'Arl9'	24	19	13	17	7	4	18.66666667	9.333333333	-0.744910875	0.408891981	0.957157474
384198	'Fam47e'	0	0	0	0	0	6	0	2	3.261750676	0.410112517	0.957157474
384214	'Ephx4'	18	8	25	2	8	15	17	8.333333333	-1.158002441	0.213172595	0.841304405
384219	'Vmn2r11'	1	0.33	0	0	1	0	0.443333333	0.333333333	0.058500858	0.988561293	0.999493374
384220	'Vmn2r16'	1	0	0	0	0	0	0.333333333	0	-0.903279821	0.824807108	0.972790461
384244	'Foxl3'	0	1	0	0	0	0	0.333333333	0	-0.903279821	0.824807108	0.972790461
384281	'Gatc'	270	318	249	302	409	322	279	344.3333333	0.373014768	0.252736131	0.871252558
384309	'Trim56'	341.84	388.24	183.05	697.51	267.71	347.98	304.3766667	437.7333333	0.780531176	0.216486793	0.843391934
384452	'Noto'	0	3	3	0	4	63	2	22.33333333	3.248075195	0.081680767	0.621278714
384557	'Ceacam3'	0	1	0	0	0	0	0.333333333	0	-0.903279821	0.824807108	0.972790461
384569	'Nova2'	87	101	28	129	138	113	72	126.6666667	0.970921496	0.078565605	0.615492265
384605	'Wdr88'	1	0	0	0	2	0	0.333333333	0.666666667	0.839717162	0.835573887	0.974723675
384619	'Kash5'	142	137	39	3	64	77	106	48	-1.212917069	0.183870519	0.804729813
384701	'Usp17ld'	5.04	2.33	2	0	0	0	3.123333333	0	-3.94385198	0.109928821	0.689773582
384724	'Cyp2t4'	1	0	1	0	0	2	0.666666667	0.666666667	-0.156033058	0.963274716	0.999493374
384763	'Zfp667'	512	478	449	329	477	401	479.6666667	402.3333333	-0.203798441	0.486571304	0.972790461
384783	'Irs2'	906	825	1427	2265	477	697	1052.666667	1146.333333	0.371180235	0.643352275	0.972790461
384806	'Adam20'	0	0	0	0	1	1	0	0.666666667	1.775692139	0.660844521	0.972790461
384997	'Pglyrp4'	0	0	0	0	0	3	0	1	2.274429265	0.570697589	0.972790461
385024	'Gm5373'	11.82	18.55	2	1	11	10	10.79	7.333333333	-0.505668941	0.655724186	0.972790461
385263	'Gm1527'	7	2	1	0	16	14	3.333333333	10	1.505554203	0.317218775	0.916136994
385343	'Rhox1'	166	143	75	0	73	254	128	109	-0.368542911	0.774393565	0.972790461
385354	'Frmd7'	1.04	0	6.97	1.28	4.59	1.03	2.67	2.3	-0.37320812	0.843919727	0.975182082
385377	'Pnma5'	4	5	4	0	5	30	4.333333333	11.66666667	1.264202875	0.380625653	0.944042914
385380	'Tex28'	5	10	1	0	2	1	5.333333333	1	-2.392345228	0.15252476	0.763026656
385454	'Gm5396'	1	0	0	0	0	0	0.333333333	0	-0.903279821	0.824807108	0.972790461

385643	'Kng2'	1	1	14	1	1	1	5.333333333	1	-2.516255165	0.14958648	0.758505335
385658	'Nxpe3'	62	61	55	715	61	95	59.33333333	290.3333333	2.681281156	0.007725904	0.211955117
385668	'Lca5l'	248	244	32	28	187	86	174.6666667	100.3333333	-0.77993446	0.343415268	0.925308103
385674	'Zfp174'	346.91	359.77	75.08	102.21	245.45	224.26	260.5866667	190.64	-0.390667106	0.510824567	0.972790461
386454	'Rnf39'	9	4	24	7	2	8	12.33333333	5.666666667	-1.113641671	0.321137185	0.917442833
386463	'Cdsn'	16	11	19	42	5	44	15.33333333	30.33333333	1.108557322	0.240266514	0.86449675
386611	'Rnf133'	0	0	0	0	1	0	0	0.333333333	1.020273531	0.802557913	0.972790461
386612	'Thoc6'	206.32	241.61	324.16	307.91	273.52	293.5	257.3633333	291.6433333	0.244507383	0.596642898	0.972790461
386649	'Nsfl1c'	1960	2106	2635	811	1745	2315	2233.666667	1623.666667	-0.519835074	0.203873258	0.832079769
386655	'Eid2'	324	408	614	250	488	520	448.6666667	419.3333333	-0.150164595	0.742589971	0.972790461
386750	'Slitrk3'	8	3	1	0	2	3	4	1.666666667	-1.292141959	0.428376179	0.962191208
386753	'Dbpht2'	5	1	1	0	3	0	2.333333333	1	-1.243917654	0.583088178	0.972790461
387285	'Hcrtr2'	1	2	0	1	0	1	1	0.666666667	-0.359181764	0.890454647	0.984980969
387314	'Tmtc1'	448	450	217	5776	371	577	371.6666667	2241.333333	3.0454568	0.004015872	0.145984247
387353	'Tas2r126'	0	0	0	1	0	0	0	0.333333333	1.020273531	0.802557913	0.972790461
387512	'Tas2r135'	1	0	0	1	1	1	0.333333333	1	1.558384074	0.565724181	0.972790461
387514	'Tas2r143'	0	1	0	5	0	0	0.333333333	1.666666667	2.683819621	0.465346482	0.972790461
387524	'Znrf2'	653	670	571	999	748	1105	631.3333333	950.6666667	0.692079839	0.072831019	0.596837647
387565	'Cd300c'	0	0	0	1	0	0	0	0.333333333	1.020273531	0.802557913	0.972790461
387586	'Ssxb5'	2	7	2	0	4	0	3.666666667	1.333333333	-1.485426519	0.454542748	0.970649024
387609	'Zhx2'	416	407	1115	324	485	575	646	461.3333333	-0.554910367	0.347793224	0.928444937
393082	'Mettl7a2'	0	0	0	0	0	1	0	0.333333333	1.020273531	0.802557913	0.972790461
394252	'Serpinb3d'	1	0	0	0	1	0	0.333333333	0.333333333	0.058500858	0.988561293	0.999493374
394430	'Ugt1a10'	0	2.28	0	0	0	0	0.76	0	-1.695595436	0.675304306	0.972790461
394432	'Ugt1a7c'	30.12	24.49	2.27	184.93	58.72	225.54	18.96	156.3966667	3.268617865	2.69E-04	0.027996512
394435	'Ugt1a6b'	12.94	30.01	18.84	29.57	6.85	38.23	20.59666667	24.88333333	0.402052015	0.643968607	0.972790461
394436	'Ugt1a1'	1.92	9.15	0	31.67	0	2.42	3.69	11.36333333	2.274950269	0.272448077	0.884738928
396184	'Flrt1'	928	885	369	1216	661	1018	727.3333333	965	0.602800626	0.262412779	0.88013169
399510	'Map4k5'	1534	1436	1704	861	1706	1481	1558	1349.333333	-0.217656677	0.507985918	0.972790461
399548	'Scn4b'	16	16	9	1	7	7	13.66666667	5	-1.475729763	0.097454076	0.663789939
399549	'H2-M10.6'	0	1	0	0	0	0	0.333333333	0	-0.903279821	0.824807108	0.972790461
399558	'Flrt2'	1457	1458	951	2847	2036	2517	1288.666667	2466.666667	1.083147726	0.009547051	0.240209487
399566	'Btbd6'	389	372	196	137	475	408	319	340	0.08085863	0.859912531	0.976863639
399568	'Cdin1'	316	300	267	92	258	344	294.3333333	231.3333333	-0.398811294	0.354720641	0.931809687
399591	'Tmsb15l'	36.71	55.61	53.99	92.41	81.22	85.4	48.77	86.34333333	0.926765348	0.059593107	0.554026475
399599	'Ccdc87'	25	20	2	1	16	15	15.66666667	10.66666667	-0.57615688	0.616789774	0.972790461
399603	'Lratd2'	1304	1402	1598	1996	1432	1279	1434.666667	1569	0.261369964	0.593818945	0.972790461
399673	'Tdpoz2'	0	0	0	0	0	1	0	0.333333333	1.020273531	0.802557913	0.972790461
399674	'Tdpoz3'	0	1	0	0	0	0	0.333333333	0	-0.903279821	0.824807108	0.972790461
403088	'Tcaf3'	0	0	0	3	0	0	0	1	2.899270347	0.465759809	0.972790461

403171	'Banf2'	0	0	1	0	0	2	0.333333333	0.666666667	0.78767502	0.845746136	0.975182082
403174	'Msantd1'	87	80	37	142	84	17	68	81	0.553704297	0.521956996	0.972790461
403175	'Tigd4'	1	2	0	4	3	1	1	2.666666667	1.661639795	0.358291291	0.933255791
403178	'Plcxd1'	59.17	42.21	506.19	607.17	54.16	183.36	202.5233333	281.5633333	0.633977367	0.59646103	0.972790461
403180	'Ccdc121'	0	0	1	1	4	0	0.333333333	1.666666667	2.244776272	0.453178666	0.970649024
403185	'Cfap97d2'	1	7	0	0	2	0	2.666666667	0.666666667	-1.936037306	0.488958155	0.972790461
403187	'Opa3'	349	365	344	255	355	361	352.6666667	323.6666667	-0.087002461	0.757465677	0.972790461
403200	'Lypd9'	2	2	4	0	3	1	2.666666667	1.333333333	-1.120139632	0.515700299	0.972790461
403205	'Agr3'	0	0	1	0	3	19	0.333333333	7.333333333	4.183314467	0.079651066	0.617305615
403395	'Clec3a'	1	0	0	0	0	0	0.333333333	0	-0.903279821	0.824807108	0.972790461
404194	'Gfral'	0	1	1	1	0	0	0.666666667	0.333333333	-0.885963919	0.798167427	0.972790461
404318	'Olfr681'	9	8	0	0	10	11	5.666666667	7	0.265187363	0.877110876	0.981341203
404322	'Olfr924'	0	2	0	0	0	0	0.666666667	0	-1.695595436	0.675304306	0.972790461
404335	'Olfr1535'	0	0	0	0	0	1	0	0.333333333	1.020273531	0.802557913	0.972790461
404337	'Olfr1383'	11.02	21.25	11	18	4	7	14.42333333	9.666666667	-0.311539805	0.744683526	0.972790461
404473	'Olfr1082'	0	0	0	0	0	1	0	0.333333333	1.020273531	0.802557913	0.972790461
404545	'Ano7'	3	7	3	1	7	4	4.333333333	4	-0.138431468	0.907684362	0.988823459
404634	'Macroh2a2'	1158.9	1360.7	624.3	93.65	1447.2	1071.37	1047.966667	870.7433333	-0.349772584	0.648118307	0.972790461
404710	'Iqgap3'	248	257	71	12	428	748	192	396	0.952105566	0.348224014	0.928444937
406217	'Bex4'	482.35	444.02	548.23	388.13	444.11	656.95	491.5333333	496.3966667	0.028711929	0.936014096	0.994413066
406218	'Panx2'	18	15	46	4	12	23	26.33333333	13	-1.170072742	0.177491177	0.797090452
406219	'Krt87'	5	4	0	3	2	1	3	2	-0.330908292	0.843134362	0.975182082
406220	'Krt77'	1	2	0	0	2	3	1	1.666666667	0.699834448	0.759774167	0.972790461
406221	'Krt40'	0	0	0	0	0	1	0	0.333333333	1.020273531	0.802557913	0.972790461
407243	'Tmem189'	241	256	369	1072	359	461	288.6666667	630.6666667	1.329110352	0.053524995	0.533767484
407785	'Ndufs6'	399.67	445	334	320	335	483	392.89	379.3333333	0.00970958	0.975100526	0.999493374
407786	'Taf9b'	1013	997	159	22	611	1111	723	581.3333333	-0.387419105	0.707198149	0.972790461
407788	'BC051142'	453	469	131	2	288	73	351	121	-1.598472202	0.155303804	0.766453805
407790	'Ndufa4l2'	472	371	3032	297	172	664	1291.666667	377.6666667	-1.902537009	0.041788712	0.477375478
407800	'Ecm2'	10	15	7	16	18	10	10.66666667	14.66666667	0.588077898	0.43121035	0.963623551
407812	'Zfp941'	77	80	52	26	90	97	69.66666667	71	-0.005218506	0.991589789	0.999493374
407819	'BC031181'	578	677	762	554	622	938	672.3333333	704.6666667	0.081983609	0.823387297	0.972790461
407821	'Znrf3'	596	620	202	297	572	478	472.6666667	449	-4.58E-04	0.99920901	0.999900097
407823	'Baz2b'	1574	1536.1	994	1090	2077	1366	1368.046667	1511	0.198814873	0.549803745	0.972790461
407831	'Tmem204'	65	70	7	209	96	90	47.33333333	131.6666667	1.783928225	0.039083258	0.462096334
408022	'Primpol'	1076.1	1095.9	494.5	292.55	732.52	334.23	888.81	453.1	-0.910398254	0.056283189	0.545946937
408058	'BC048507'	1	1	0	1	0	0	0.666666667	0.333333333	-0.746871675	0.831393929	0.974324192
408059	'BC049352'	3	0	0	0	0	5	1	1.666666667	0.651554123	0.861014274	0.976863639
408062	'Zfp873'	338	361.54	124	65	349	299	274.5133333	237.6666667	-0.230812175	0.705251628	0.972790461
408065	'Zfp456'	85.08	90.55	135.55	66	162	128	103.7266667	118.6666667	0.152135677	0.749606548	0.972790461

408066	'Cspg4b'	1145	1096	178	157	1365	1012	806.3333333	844.6666667	0.04937582	0.950754287	0.998065566
408067	'Zfp874b'	270.52	242.04	276.16	180	273.33	304	262.9066667	252.4433333	-0.047393552	0.880376509	0.982087554
408068	'Zfp738'	227.77	238.72	243.56	142.21	499.32	393.01	236.6833333	344.8466667	0.491894498	0.265548499	0.881878584
408190	'Wfdc13'	4	3	0	5	3	2	2.333333333	3.333333333	0.768952789	0.617438033	0.972790461
408191	'Gm5415'	1	2.93	1	0	4.5	0	1.643333333	1.5	-0.060739301	0.979561661	0.999493374
408192	'Gm9839'	2	2.18	0	0	1.5	0.9	1.393333333	0.8	-1.740097262	0.578803281	0.972790461
408193	'Otud6a'	36	34	7	3	16	22	25.66666667	13.66666667	-0.919378164	0.295630805	0.90029104
414069	'BC024978'	644.28	687.69	332.41	174.23	398.78	324.83	554.7933333	299.28	-0.85654095	0.023621513	0.380264354
414077	'Wdr83os'	227	262	212	160	299	402	233.6666667	287	0.284900693	0.398605129	0.954563638
414084	'Tnip3'	4	5	367	2	3	9	125.3333333	4.666666667	-5.009112784	9.45E-04	0.064169552
414089	'Gja6'	5	5	13	6	11	1	7.666666667	6	-0.331303888	0.778804276	0.972790461
414758	'Zfp950'	845.84	897.89	179.5	2454.6	2088	724.36	641.0766667	1755.636667	1.719676909	0.021991132	0.367072342
414801	'Itprip'	213	204	308	857	282	347	241.6666667	495.3333333	1.24356504	0.075597642	0.605310548
414872	'Zyg11b'	2605	2537	2673	1089	2376	2882	2605	2115.666667	-0.337478966	0.3323878	0.921648675
415115	'Neurl2'	90.67	100.18	213.12	6	129.03	73.4	134.6566667	69.47666667	-1.145452191	0.200944503	0.828439535
431706	'Zfp457'	18.92	25.98	10	7	7	7	18.3	7	-1.229059775	0.097768492	0.664560086
432442	'Akap7'	644	721	281	70	1009	747	548.6666667	608.6666667	0.079147248	0.917944475	0.990688031
432450	'Nkain2'	61	79	33	82	63	34	57.66666667	59.66666667	0.260975525	0.684483432	0.972790461
432467	'Hnrnph3'	1636.5	1619.9	1171.9	2274.3	1374.6	1649.14	1476.08	1765.98	0.428952727	0.361705764	0.933706718
432478	'Tmprss9'	11	9	8	4	19	13	9.333333333	12	0.328741286	0.681390658	0.972790461
432486	'Gnptab'	1043	986	433	1081	1305	1367	820.6666667	1251	0.714441722	0.067708295	0.580700792
432502	'Rpl61'	0	0	0	0	3.23	4.56	0	2.596666667	3.516815001	0.323448415	0.918976057
432508	'Cpsf6'	3047	3088	1782	2800	3249	2567	2639	2872	0.240146173	0.511438027	0.972790461
432516	'Myo1a'	165	185	38	31	186	96	129.3333333	104.3333333	-0.300165849	0.67936276	0.972790461
432530	'Adcy1'	132	140	59	26	89	73	110.3333333	62.66666667	-0.80930771	0.11702617	0.705163127
432552	'Fam71b'	6	2	0	1	5	0	2.666666667	2	-0.313074206	0.883071546	0.983244267
432555	'Gm5431'	0	0	0	1	1	4	0	2	3.406172247	0.238166183	0.864053647
432572	'Specc1'	451	452	549	1523	312	416	484	750.3333333	0.916892747	0.249555018	0.869928149
432582	'Ccdc92b'	33	29	11	3	33	27	24.33333333	21	-0.258412833	0.760731032	0.972790461
432589	'Gm11541'	5	1	0	0	2	1	2	1	-0.999781903	0.668097122	0.972790461
432600	'Gm11568'	0	0	0	0	1.32	0	0	0.44	1.020273531	0.802557913	0.972790461
432611	'Dnaic2'	22	22	151	10	34	44	65	29.33333333	-1.362985792	0.160961028	0.771435531
432613	'Trim80'	3	6	0	1	4	7	3	4	0.430944673	0.779957481	0.972790461
432628	'Mfsd2b'	25	29	17	7	26	13	23.66666667	15.33333333	-0.617336477	0.34839534	0.928444937
432677	'Vrtn'	6	2	4	0	15	57	4	24	2.406292718	0.093027689	0.654455509
432720	'Akr1c19'	33	35	3	1	77	38	23.66666667	38.66666667	0.669235767	0.584709925	0.972790461
432731	'Zscan26'	1909	1968	2218	1144	2018	1402	2031.666667	1521.333333	-0.397447046	0.256674433	0.877127174
432763	'Prr7'	28	27	52	30	21	41	35.66666667	30.66666667	-0.198484927	0.757862776	0.972790461
432769	'Zfp708'	112	113	121	87	184.55	119	115.3333333	130.1833333	0.181301654	0.647359009	0.972790461
432770	'Rslcan18'	48	61	91	18	43.45	45	66.66666667	35.48333333	-0.978486005	0.07734058	0.611392561

432779	'Lrrc14b'	50.08	40.97	48.56	90.39	41.98	57.64	46.53666667	63.33666667	0.610204183	0.325926157	0.920269559
432800	'Gm5454'	0	0	0	1.26	0	0	0	0.42	1.020273531	0.802557913	0.972790461
432825	'Gm5458'	25.11	11.55	10.87	0	7.27	4	15.84333333	3.756666667	-2.120207791	0.054100383	0.534315058
432839	'Gprin2'	173	135	79	253	148	211	129	204	0.835636574	0.107738078	0.683733849
432860	'B020004C17Rik'	0	0	0	0	1	0	0	0.333333333	1.020273531	0.802557913	0.972790461
432867	'Defb48'	0	0	0	0	1	0	0	0.333333333	1.020273531	0.802557913	0.972790461
432879	'Kbtbd6'	416.87	426.87	59.18	36.14	181.3	175.31	300.9733333	130.9166667	-1.19457227	0.118924953	0.709000063
432940	'Otulin'	467	439	406	393	466	574	437.3333333	477.6666667	0.174777991	0.536423789	0.972790461
432964	'Iqank1'	22	24	4	3	9	9	16.66666667	7	-1.203567304	0.187678473	0.808805329
432995	'Smim22'	17	8	7	1	9	11	10.66666667	7	-0.665847844	0.500041	0.972790461
432999	'A930007A09Rik'	215	218	40	33	171	128	157.6666667	110.6666667	-0.501746925	0.487539932	0.972790461
433016	'Cstdc4'	0	23	1	0	37	0	8	12.33333333	0.611518615	0.806292002	0.972790461
433022	'Plcxd2'	105	103	358	283	54	114	188.6666667	150.3333333	-0.160712679	0.860422475	0.976863639
433064	'Gm17748'	7.25	4.81	6.47	0	0	0.56	6.176666667	0.186666667	-4.901326503	0.013795685	0.292512191
433091	'Pnpla1'	13	13	2	180	12	23	9.333333333	71.66666667	3.41345802	0.004477591	0.157418103
433099	'Ly6g6f'	0	1	0	0	0	0	0.333333333	0	-0.903279821	0.824807108	0.972790461
433102	'Sfta2'	1	0	0	0	0	0	0.333333333	0	-0.903279821	0.824807108	0.972790461
433107	'Esp6'	1	1	0	0	1	0	0.666666667	0.333333333	-0.74517548	0.838722495	0.974723675
433182	'Eno1b'	3229.7	3219.2	15639	0	731.19	0	7362.57	243.73	-5.168406533	0.096449074	0.663111432
433215	'Tmem262'	7	9	2	3	8	2	6	4.333333333	-0.37618742	0.741759167	0.972790461
433256	'Acsl5'	359	400	696	2150	784	1501	485	1478.333333	1.737845776	0.008052093	0.216529539
433287	'Gm15455'	0	0	0	1.01	1	1	0	1.003333333	2.526085132	0.463240869	0.972790461
433294	'Mettl21c'	2	1	1	0	12	5	1.333333333	5.666666667	1.985416755	0.238641725	0.864053647
433323	'Sgpp2'	40	40	8	101.12	25	25.02	29.33333333	50.38	1.150406773	0.223449043	0.850759465
433365	'Teddm1b'	9	11	0	1	19	6	6.666666667	8.666666667	0.386596781	0.794246813	0.972790461
433375	'Cregl'	1159	1314	879	3478	2593	5370	1117.333333	3813.666667	1.85146789	3.91E-06	0.001161794
433415	'Gm13420'	15	17	8	12	9	8	13.33333333	9.666666667	-0.314148917	0.681016635	0.972790461
433416	'Gm13547'	47	37	4	50	40	29	29.33333333	39.66666667	0.669949912	0.439220523	0.967427574
433466	'Jmjd7'	153	182	147	100	271	220	160.6666667	197	0.277479007	0.464231183	0.972790461
433470	'AA467197'	4	4	37	28	13	26	15	22.33333333	0.554133336	0.619803123	0.972790461
433485	'Syndig1'	6	6	1	2	12	2	4.333333333	5.333333333	0.344589054	0.790325776	0.972790461
433486	'Gm14151'	0	0.92	0	0	0	1.14	0.306666667	0.38	1.020273531	0.802557913	0.972790461
433502	'Wfdc6b'	2.17	0	1.66	1	0	1.05	1.276666667	0.683333333	-0.457107805	0.860312146	0.976863639
433586	'Maml3'	605	633	306	200	591	660	514.6666667	483.6666667	-0.099781759	0.823169403	0.972790461
433597	'Aadacl2fm2'	1	0	0	0	1	1	0.333333333	0.666666667	0.817432597	0.823901819	0.972790461
433619	'Kprp'	0	0	0	0	1	0	0	0.333333333	1.020273531	0.802557913	0.972790461
433632	'Gm5544'	1	0	23	2	0	0	8	0.666666667	-3.409982867	0.174847671	0.793366093
433638	'I830077J02Rik'	2	2	0	11	6	5	1.333333333	7.333333333	2.70097297	0.050273441	0.518969204
433653	'Gimd1'	0	2	0	0	1	0	0.666666667	0.333333333	-0.733814424	0.856146772	0.975734242
433667	'Ankrd13c'	864	963	730	820.01	1001	976	852.3333333	932.3366667	0.201669979	0.473824152	0.972790461

433693	'Akirin2'	1129	1085	2310	1767	1194	1822	1508	1594.333333	0.124014494	0.827515865	0.973238667
433698	'Fam205a1'	6.13	4.54	1.72	39.47	29.53	16.53	4.13	28.51	3.154694145	7.24E-04	0.053564853
433700	'Spag8'	18	16	3	6	23	17	12.33333333	15.33333333	0.345516361	0.690212396	0.972790461
433702	'Ncbp1'	1739.9	1673.9	1186	1274	1438	1692	1533.273333	1467.996667	0.014138578	0.959983358	0.999493374
433745	'Gm12816'	175.84	148.2	229.31	0	0	0	184.45	0	-9.943802547	2.53E-14	4.82E-11
433748	'Llph-ps1'	1.01	0	1.03	0	1	0	0.68	0.333333333	-0.894008961	0.803817041	0.972790461
433752	'Frg2f1'	75	80	74	24	51	32	76.33333333	35.66666667	-1.077887151	0.014271662	0.297188218
433759	'Hdac1'	1638	1730	1093	1323	1931	2043	1487	1765.666667	0.302959044	0.236221592	0.862272407
433766	'Trim63'	8	7	30	27	9	8	15	14.66666667	0.111711671	0.916313003	0.990526893
433771	'Micos10'	1178	1276	1003	994	922	1221	1152.333333	1045.666667	-0.056466774	0.862185455	0.977044159
433779	'Pramel31'	2	0	0	0	0	0	0.666666667	0	-1.711373851	0.67234292	0.972790461
433791	'Zfp992'	229.96	281.35	350.85	149.73	740.87	614.05	287.3866667	501.55	0.698893235	0.212233537	0.84083615
433801	'Gm13212'	566.78	524.4	1163.9	263.83	538.14	453.91	751.68	418.6266667	-0.908046128	0.084092494	0.630179937
433804	'Zfp985'	96.63	124.48	191.77	18.05	37.84	12	137.6266667	22.63	-2.612980581	4.81E-05	0.008204297
433809	'Rnf207'	6	10	1	10	7	3	5.666666667	6.666666667	0.490095898	0.676367179	0.972790461
433813	'Pus11'	155.11	136.34	197.65	68.3	141.67	191.4	163.0333333	133.79	-0.343372261	0.443397693	0.969836424
433864	'Nom1'	1115	1178	863	727	986	1036	1052	916.3333333	-0.143785707	0.555406893	0.972790461
433874	'Gm5553'	0	0	0	0	1	0	0	0.333333333	1.020273531	0.802557913	0.972790461
433899	'Grxcr1'	0	0	0	0	1	0	0	0.333333333	1.020273531	0.802557913	0.972790461
433904	'Ociad2'	109	124	105	87	167	200	112.6666667	151.3333333	0.418969527	0.224525354	0.850759465
433926	'Lrrc8b'	271	269	420	118	340	405	320	287.6666667	-0.247109151	0.622575214	0.972790461
433931	'Pigg'	307	302	117	115	329	366	242	270	0.163962841	0.740161549	0.972790461
433938	'Mn1'	186	212	52	124	190	186	150	166.6666667	0.251579218	0.635690602	0.972790461
433940	'Fam222a'	221	220	46	178	333	324	162.3333333	278.3333333	0.860448018	0.137523832	0.738626463
433956	'Dnaaf5'	457	448	332	330	407	508	412.3333333	415	0.064063207	0.814317887	0.972790461
434008	'Tmem178b'	64	49	22	1	94	129	45	74.66666667	0.624978723	0.570669372	0.972790461
434110	'Vmn2r38'	1	0	0	0	0	0	0.333333333	0	-0.903279821	0.824807108	0.972790461
434128	'Pnmal2'	798	798	345	1459	998	943	647	1133.333333	1.011004058	0.052898923	0.532526438
434130	'Ccdc8'	2258	2433	3569	2598	2845	3113	2753.333333	2852	0.079216461	0.85047527	0.975734242
434156	'Eid2b'	342	378	311	279	356	413	343.6666667	349.3333333	0.069170248	0.799938572	0.972790461
434171	'Gm5591'	0	0	0	0	0	1	0	0.333333333	1.020273531	0.802557913	0.972790461
434175	'Ccnb1-ps'	26.51	25.33	7.8	1.07	15.44	5.51	19.88	7.34	-1.473228834	0.146004285	0.753611197
434178	'Zfp141'	727	855.04	848	397	784	715	810.0133333	632	-0.364760984	0.251716529	0.871252558
434179	'Zfp975'	395.26	426.98	288.57	145	414.09	220.1	370.27	259.73	-0.494919402	0.235050118	0.861636582
434197	'Fam169b'	13	21	3	2	79	41	12.33333333	40.66666667	1.6720769	0.135207579	0.736012522
434203	'Slc28a1'	7	6	2	6	6	2	5	4.666666667	0.081616514	0.942499455	0.996301887
434204	'Whamm'	234	238	787	350	222	305	419.6666667	292.3333333	-0.494607737	0.487566913	0.972790461
434215	'Lrrc32'	354	290	103	372	339	130	249	280.3333333	0.401302948	0.550279547	0.972790461
434218	'Trim34b'	155.63	138.97	44.25	25.03	85.73	46.04	112.95	52.26666667	-1.068532845	0.078576974	0.615492265
434219	'Trim30c'	0	1	3	1	0	0	1.333333333	0.333333333	-1.964084339	0.521028702	0.972790461

434223	'Gvin3'	24.33	30.57	8.7	9	36	24	21.2	23	0.174438314	0.810322844	0.972790461
434232	'Iqck'	63.11	60.06	20	59	58.06	58	47.72333333	58.35333333	0.434652484	0.434433686	0.965227678
434233	'Ppp1ccb'	323.9	380.92	442.27	164.96	375.28	303.23	382.3633333	281.1566667	-0.470786692	0.228236876	0.854685736
434234	'Rexo5'	858.2	804.22	250.6	124.47	574.23	388	637.6733333	362.2333333	-0.812489797	0.174697312	0.793295795
434246	'Trim72'	52	63	19	9	36	10	44.66666667	18.33333333	-1.233443664	0.102469654	0.671776916
434264	'Sult2a5'	0	0	0	0	0	2	0	0.666666667	1.749449488	0.66566965	0.972790461
434325	'Tmem221'	31	31	5	19	65	77	22.33333333	53.66666667	1.278939096	0.092707335	0.654099957
434341	'Nlrc5'	22	23	29	24	51	46	24.66666667	40.33333333	0.691766199	0.176237071	0.796060628
434377	'Zfp560'	372	361	281	97	358	232	338	229	-0.587541146	0.182301778	0.80302276
434396	'Pate9'	0	0	5	0	0	0	1.666666667	0	-3.332616029	0.399165646	0.955184049
434402	'Gm5617'	188	232	135	72	152	227	185	150.3333333	-0.3101973	0.454226596	0.970649024
434423	'Dppa5a'	75	76	41	10	27.19	81	64	39.39666667	-0.764144787	0.278093066	0.889355605
434436	'Lsmem2'	10	7.15	1	8.23	3.01	3	6.05	4.746666667	-0.081967242	0.947102103	0.996914484
434437	'Amt'	128	142	115	120	198	114	128.3333333	144	0.228200384	0.567636823	0.972790461
434438	'Ihol'	303	303	159	11	93	37	255	47	-2.47491504	2.05E-04	0.022950907
434459	'Gm5622'	1	0	0	0	0	0	0.333333333	0	-0.903279821	0.824807108	0.972790461
434484	'Sp140'	18.62	15.46	93.21	55.01	22	146.12	42.43	74.37666667	0.69850858	0.480632047	0.972790461
434540	'Psg20'	0	1	0	0	0	1	0.333333333	0.333333333	0.058500858	0.988561293	0.999493374
434624	'Ftl1-ps2'	26	36	99.01	47	27	74	53.67	49.33333333	-0.150903196	0.846430897	0.975201539
434674	'Slc22a28'	0	0	0	0	1	0	0	0.333333333	1.020273531	0.802557913	0.972790461
434689	'Gm10220'	59.02	89.31	32.62	233.5	132.81	148.19	60.31666667	171.5	1.724211993	0.003818606	0.141968654
434693	'Mrto4-ps2'	91.03	111.04	27.01	28	96	48.01	76.36	57.33666667	-0.363509717	0.573599228	0.972790461
434726	'Fthl17b'	3.25	0	0	0	0	1.25	1.083333333	0.416666667	-1.33025371	0.739510174	0.972790461
434727	'Fthl17c'	5.48	5.45	0	0	2.56	3.75	3.643333333	2.103333333	-1.014660972	0.603057606	0.972790461
434728	'Fthl17-ps1'	0	3.55	0	0	2.44	0	1.183333333	0.813333333	-0.532516362	0.893197773	0.985171
434729	'Fthl17f'	1.01	0	0	0	2	0	0.336666667	0.666666667	0.839717162	0.835573887	0.974723675
434756	'Akap14'	0	1	0	0	2	0	0.333333333	0.666666667	0.839717162	0.835573887	0.974723675
434758	'Rhox3h'	1.11	3.14	0	0	1.08	6	1.416666667	2.36	0.74960341	0.740009508	0.972790461
434760	'Rhox2d'	9	11	2	0	3	9	7.333333333	4	-0.933376991	0.499802287	0.972790461
434764	'Rhox2f'	2.99	3.11	0	1	0	4	2.033333333	1.666666667	0.072443188	0.973811886	0.999493374
434766	'Rhox2g'	7.18	5.08	2	0	3	6	4.753333333	3	-0.701315426	0.620749457	0.972790461
434768	'Rhox8'	33	46	1	129	41	41	26.66666667	70.33333333	1.770138157	0.109030445	0.688209437
434769	'Rhox10'	1	1	0	0	0	2	0.666666667	0.666666667	-0.01112152	0.997410093	0.999723781
434778	'Ccdc160'	96	87	49	36	75	84	77.33333333	65	-0.228613928	0.588629802	0.972790461
434782	'Gm5637'	3.3	0	0	0	1.01	0	1.1	0.336666667	-1.33025371	0.739510174	0.972790461
434784	'Ldoc1'	2	1	3	17	2	5	2	8	2.254612374	0.10277568	0.672865573
434794	'Xlr4a'	515.09	488.9	209.09	3.41	295.13	519.12	404.36	272.5533333	-0.684273921	0.534078441	0.972790461
434797	'Gm5640'	3.07	3.49	0	0	7	4.05	2.186666667	3.683333333	0.844480054	0.657718166	0.972790461
434800	'Smim9'	2	0	0	0	0	0	0.666666667	0	-1.711373851	0.67234292	0.972790461
434863	'Gm15128'	8	4.79	4.67	0	5.35	10.3	5.82	5.216666667	-0.204564484	0.87743269	0.981341203

434864	'Gm15107'	8.67	0	0	0	0	2.9	2.89	0.966666667	-2.006994016	0.570042027	0.972790461
434865	'Luzp4'	1.67	6.85	3.37	0	0	1.66	3.963333333	0.553333333	-3.152046568	0.142919819	0.749054337
434866	'Gm15127'	3.04	0	0	0	0	0	1.013333333	0	-2.292035055	0.566710385	0.972790461
434869	'Gm15097'	5.72	8.31	11.96	0	3.34	10.84	8.663333333	4.726666667	-1.056701874	0.415682682	0.95722888
434903	'Mageb4'	888	872	147	7	775	2896	635.6666667	1226	0.853021048	0.499044287	0.972790461
435145	'Shisa8'	10	7	18	4	8	7	11.66666667	6.333333333	-0.925054852	0.292750978	0.900043079
435206	'Taar7d'	2	1	0	1	2	3	1	2	1.051048878	0.575705195	0.972790461
435207	'Taar7f'	0	1	0	1	1	0	0.333333333	0.666666667	1.081692101	0.760081965	0.972790461
435273	'Krtap1-3'	0	0	1	0	0	0	0.333333333	0	-0.903279821	0.824807108	0.972790461
435285	'Krtap4-16'	0	0	0	0	1	0	0	0.333333333	1.020273531	0.802557913	0.972790461
435337	'Gm5662'	0	0	1	0	0	0	0.333333333	0	-0.903279821	0.824807108	0.972790461
435350	'Serpinb6e'	1	1	1	0	1	0	1	0.333333333	-1.427484097	0.601914152	0.972790461
435376	'Atp6ap1l'	30	37	0	4	38	13	22.33333333	18.33333333	-0.251883472	0.851573234	0.975734242
435391	'Dupd1'	1	1	3	0	1	1	1.666666667	0.666666667	-1.443450795	0.491384963	0.972790461
435528	'Glyatl3'	1	2	2	0	5	2	1.666666667	2.333333333	0.370928131	0.833177242	0.974723675
435529	'Adgrf2'	6	12	4	46	8	12	7.333333333	22	1.929905963	0.067254453	0.579357341
435626	'Rufy4'	63	72	124	82	54	52	86.33333333	62.66666667	-0.363743179	0.561864499	0.972790461
435653	'Fcrlb'	1	2	1	2	4	4	1.333333333	3.333333333	1.344349137	0.347839992	0.928444937
435684	'Shf'	188	181	151	121	376	297	173.3333333	264.6666667	0.585567338	0.144044215	0.750780289
435732	'Gm5709'	7	4	0	0	23	10	3.666666667	11	1.548946412	0.371208282	0.938513534
435772	'Cnbd1'	0	0	0	0	1	0	0	0.333333333	1.020273531	0.802557913	0.972790461
435802	'Cyp4a30b'	0	0	0	0	2	2	0	1.333333333	2.719229454	0.494880768	0.972790461
435811	'Ldlrad2'	7	8	1	0	9	6	5.333333333	5	-0.13275645	0.927136421	0.992785839
435818	'Slc2a7'	1	2	0	3	1	3	1	2.333333333	1.414194557	0.44061958	0.968551476
435927	'Gm5724'	0	0	0	0	0	1	0	0.333333333	1.020273531	0.802557913	0.972790461
435965	'Lrp3'	817	769	506.98	53	574	502.99	697.66	376.6633333	-0.981716298	0.156133479	0.767625991
435975	'Vmn2r63'	0	0	0	0	0	1	0	0.333333333	1.020273531	0.802557913	0.972790461
436008	'Gm5737'	0	0	1	0	0	1	0.333333333	0.333333333	0.058500858	0.988561293	0.999493374
436022	'Dnaaf3'	64	63	30	194.88	93	55.77	52.33333333	114.55	1.405511296	0.053875072	0.534031734
436062	'Cibar2'	5	9	0	1	8	3	4.666666667	4	-0.179021769	0.907450772	0.988823459
436090	'Gpr62'	25	26	18	22	14	10	23	15.33333333	-0.407763379	0.563642791	0.972790461
436100	'Gm21814'	37	30	3	12	30	10	23.33333333	17.33333333	-0.307811629	0.746135238	0.972790461
436240	'Foxr2'	7	7	1	5	6	5	5	5.333333333	0.226668976	0.835734743	0.974723675
436336	'Gm5767'	1	0	4	1	1	0	1.666666667	0.666666667	-1.301927292	0.59597382	0.972790461
436440	'Gpr31b'	0	4	0	0	2	1	1.333333333	1	-0.417880349	0.888933438	0.984792893
442801	'Arhgef15'	101	119	26	0	148	63	82	70.33333333	-0.287389068	0.823055787	0.972790461
442827	'Rab44'	41	47	6	5	52	64	31.33333333	40.33333333	0.329099371	0.731245568	0.972790461
442829	'Ccin'	0	1	0	5	2	0	0.333333333	2.333333333	3.017731931	0.268453885	0.883600995
442834	'D830031N03Rik'	1067	950	380	1569	730	1134	799	1144.333333	0.741259122	0.207144603	0.83479753
445007	'Nup85'	1101	1255	525	322	865	914	960.3333333	700.3333333	-0.447558061	0.305864295	0.907188578

446099	'Nlrp4e'	0	0	0	1	0	27	0	9.333333333	5.506183195	0.052357918	0.531770361
446101	'Xrra1'	68	59	7	4	33	33	44.66666667	23.33333333	-0.947326639	0.31712447	0.916136994
448850	'Znhit3'	193.93	227.93	148	84	162.82	219	189.9533333	155.2733333	-0.288683985	0.422122581	0.959445304
448987	'Fbxl7'	359	401	412	109	340	312	390.6666667	253.6666667	-0.677459981	0.108178801	0.684880244
449000	'Zfp960'	181.32	186.07	259.39	81.09	132.21	194.68	208.9266667	135.9933333	-0.661632298	0.127327545	0.723692461
449521	'Zfp213'	432	503	154	86	423	457	363	322	-0.199001848	0.748419909	0.972790461
450219	'Gsdma3'	0	0	0	0	1	0	0	0.333333333	1.020273531	0.802557913	0.972790461
474156	'Zbtb9'	508	516	393	219	387	439	472.3333333	348.3333333	-0.423914838	0.116754893	0.704694883
474160	'Smim40'	5	5	6	16	6	4	5.333333333	8.666666667	0.925679485	0.384737768	0.94674509
494448	'Cbx6'	2606.6	2579.1	1967.3	1586.7	2883.7	2336.88	2384.296667	2269.096667	-0.035600675	0.89242463	0.985171
494468	'Armcx5'	548.17	539.86	213.97	154.99	686.13	524.07	434	455.0633333	0.058063284	0.915541293	0.990380433
494504	'Apcdd1'	312	283	82	93	230	199	225.6666667	174	-0.324866615	0.547685553	0.972790461
497071	'Rnase13'	1	0	0	0	2	0	0.333333333	0.666666667	0.839717162	0.835573887	0.974723675
497097	'Xkr4'	61	47	89	39	60	76	65.66666667	58.33333333	-0.202686068	0.690944361	0.972790461
497106	'Rnase12'	0	0	0	0	8	2	0	3.333333333	4.056449706	0.214736308	0.842635023
497113	'Rnase11'	0	1	0	0	1	3	0.333333333	1.333333333	1.750813674	0.584138459	0.972790461
497114	'Defa23'	0	0	0	0	1	0	0	0.333333333	1.020273531	0.802557913	0.972790461
497652	'Acd'	431	419	666	551	469	532	505.3333333	517.3333333	0.094254983	0.84284419	0.975182082
503610	'Zdhhc18'	455	461	357	205	459	398	424.3333333	354	-0.255295659	0.403427364	0.955996639
503692	'Aym1'	0	1	0	0	0	0	0.333333333	0	-0.903279821	0.824807108	0.972790461
504186	'Chrna10'	0	1	0	0	0	0	0.333333333	0	-0.903279821	0.824807108	0.972790461
504193	'Npcd'	218.72	159.16	115.55	98.99	164.17	337.51	164.4766667	200.2233333	0.271555851	0.565467041	0.972790461
50490	'Nox4'	64	90	15	34	142	118	56.33333333	98	0.815739868	0.24891291	0.869594312
50492	'Thop1'	803	797	503	238	696	798	701	577.3333333	-0.305428557	0.459389024	0.972790461
50493	'Txnrd1'	1701	1922	4958	6928	2218	5679	2860.333333	4941.666667	0.872295832	0.24026451	0.86449675
50496	'E2f6'	859	965	1112	863	1014	1146	978.6666667	1007.666667	0.07570156	0.827844267	0.973238667
50497	'Hspa14'	729	752	795	572	706	775	758.6666667	684.3333333	-0.109960864	0.719957961	0.972790461
50498	'Ebi3'	4	8	2	2	5	12	4.666666667	6.333333333	0.427040201	0.702833259	0.972790461
50500	'Ttpa'	66	41	24	12	37	45	43.66666667	31.33333333	-0.485924553	0.422750386	0.95973972
50501	'Prok2'	0	1	14	1	1	0	5	0.666666667	-2.943999865	0.195832823	0.821529929
50505	'Ercc4'	509	501	308	96	606	415	439.3333333	372.3333333	-0.292991093	0.607822476	0.972790461
50518	'a'	4773	3971	2256	22644	7741	5277	3666.666667	11887.33333	2.020141877	0.0068427	0.195923816
50523	'Lats2'	1257	1190	1521	1065	1129	1546	1322.656667	1246.663333	-0.05615323	0.877638256	0.981341203
50524	'Sall2'	977	975	408	210	1191	854	786.6666667	751.6666667	-0.094378302	0.871221001	0.980155848
50525	'Spag61'	10	2	1	9	4	15	4.333333333	9.333333333	1.232628817	0.301150769	0.904375823
50527	'Ero1a'	5054	2944	19652	5026	1591	5242	9216.666667	3953	-1.21228544	0.168861111	0.784384366
50528	'Tmprss2'	6	3	7	112	1	13	5.333333333	42	3.360579249	0.017535378	0.331280729
50529	'Mrps7'	669	657	647	458	718	865	657.6666667	680.3333333	0.055983264	0.840141966	0.974780034
50530	'Mfap5'	4	2	2	0	4	9	2.666666667	4.333333333	0.583767518	0.700423332	0.972790461
50540	'Igbp1b'	7	9	1	0	2	9	5.666666667	3.666666667	-0.686065469	0.657540126	0.972790461

50701	'Elane'	0	0	0	0	1	0	0	0.333333333	1.020273531	0.802557913	0.972790461
50702	'Cfhr1'	0	0.93	1	0	3	0	0.643333333	1	1.381884369	0.730152291	0.972790461
50706	'Postn'	518	416	182	136	739	380	372	418.3333333	0.160086649	0.790020477	0.972790461
50708	'H1f2'	65	52	362	334	59	119	159.6666667	170.6666667	0.239914294	0.817773759	0.972790461
50709	'H1f4'	7	7	20	3	3	8	11.33333333	4.666666667	-1.356820034	0.184224659	0.804780995
50720	'Sacs'	1806	2055	653	329	1871	1109	1504.666667	1103	-0.458494102	0.454567359	0.970649024
50721	'Sirt6'	265	304	373	165	344	403	314	304	-0.092375807	0.818408224	0.972790461
50722	'Dkkl1'	10	14	2	55	7	4	8.666666667	22	1.808520232	0.155237932	0.766453805
50723	'Icosl'	131	138	452	217	136	167	240.3333333	173.3333333	-0.432162188	0.553531973	0.972790461
50724	'Sap30l'	506	495	387	320	382	490	462.6666667	397.3333333	-0.166378234	0.546055321	0.972790461
50753	'Fbxo8'	417.84	402.79	540.96	421.93	357.95	483.91	453.8633333	421.2633333	-0.049985956	0.906273288	0.988823459
50754	'Fbxw7'	1937	1689	524	598	1249	1408	1383.333333	1085	-0.302018452	0.549911656	0.972790461
50755	'Fbh1'	2232.8	2100.4	1541.2	1392.2	2353.9	2365.82	1958.14	2037.296667	0.090951457	0.707530749	0.972790461
50757	'Fbxw14'	2	2	0	0	2	3.24	1.333333333	1.746666667	0.289356044	0.894433345	0.985259027
50758	'Fbxl17'	1727	1798	685	484.02	1627	1417	1403.32	1176.006667	-0.24909504	0.612313762	0.972790461
50759	'Fbxo16'	129	159	52	38	180	134	113.3333333	117.3333333	0.041802166	0.944431751	0.996592403
50760	'Fbxo17'	122	155	161	68	201	190	146	153	0.013269233	0.976239111	0.999493374
50762	'Fbxo6'	290	277	693	339	233	402	420	324.6666667	-0.356740614	0.561885789	0.972790461
50764	'Fbxo15'	300	305	77	11	118	155	227.3333333	94.66666667	-1.320309347	0.10701449	0.68182288
50765	'Tfr2'	13	11	37	30	4	4	20.33333333	12.66666667	-0.425936475	0.717846508	0.972790461
50766	'Crim1'	898	773	595	5084	990	1688	755.3333333	2587.333333	2.086597965	0.006411423	0.190013707
50767	'Pnpla6'	937	883	288	649	1344	1240	702.6666667	1077.666667	0.676961834	0.152359102	0.762678053
50768	'Dlc1'	1828	1547	4382	1807	1535	1691	2585.666667	1677.666667	-0.602374814	0.324187805	0.919644666
50769	'Atp8a2'	29	34	20	4	71	114	27.66666667	63	1.068813833	0.241409701	0.86449675
50770	'Atp11a'	3914	3714	1396	3500.7	4022	3079	3008.01	3533.893333	0.379418512	0.418515351	0.958226521
50771	'Atp9b'	1573	1592	745	697	1809	1329	1303.333333	1278.333333	0.002533038	0.99502583	0.999562152
50772	'Mapk6'	2141	2031	2683	2632	1645	1933	2285	2070	-0.020021599	0.968094056	0.999493374
50773	'Nt5c'	225	208	161	182	290	318	198	263.3333333	0.443136613	0.107745982	0.683733849
50774	'Krtap5-1'	0	0	0	1	2	0	0	1	2.548734582	0.523314857	0.972790461
50776	'Polg2'	129	119	227	110	133	141	158.3333333	128	-0.305020975	0.539362183	0.972790461
50778	'Rgs1'	6	2	16	49	4	39	8	30.66666667	2.037497873	0.084994298	0.633939424
50779	'Rgs6'	64	51	62	12	84	82	59	59.33333333	-0.098644784	0.881847492	0.982528614
50780	'Rgs3'	1241	1341	580	491	1670	1058	1054	1073	0.039759911	0.932758514	0.993970337
50781	'Dkk3'	316	276	214	1160	279	378	268.6666667	605.6666667	1.47231962	0.047102992	0.50504201
50782	'Rgs11'	1178	1106	606	177	1352	722	963.3333333	750.3333333	-0.408973538	0.510351145	0.972790461
50783	'Lsm4'	731	776	659	247	556	781	722	528	-0.488101724	0.195322265	0.820253997
50784	'Plpp2'	265	221	715	236	282	460	400.3333333	326	-0.370828402	0.559928058	0.972790461
50785	'Hs6st1'	2227	2157	4581	5205	2213	2457	2988.333333	3291.666667	0.289045662	0.675061786	0.972790461
50786	'Hs6st2'	1123	1061	481	879	1445	1872	888.3333333	1398.666667	0.70087058	0.068175993	0.583566634
50787	'Hs6st3'	0	7	1	1	3	1	2.666666667	1.666666667	-0.59896458	0.751647217	0.972790461

50788	'Fbxl8'	18	25	81	28	35	38	41.33333333	33.66666667	-0.354024529	0.650591117	0.972790461
50789	'Fbxl3'	1461	1561	2363	562	1868	1452	1795	1294	-0.555611185	0.257797693	0.877405867
50790	'Acsl4'	857	894	6301	6466	1074	2572	2684	3370.666667	0.456341169	0.662679604	0.972790461
50791	'Magi2'	1386	1385	671	110	1960	1507	1147.333333	1192.333333	-0.031138789	0.968644577	0.999493374
50793	'Orc3'	1820	1846	1451	903	1575	1593	1705.666667	1357	-0.307400992	0.194537924	0.819426224
50794	'Klf13'	906	802	3023	1542	588	990	1577	1040	-0.517081282	0.511692679	0.972790461
50795	'Sh3bgr'	19	28	36	44	91	61	27.66666667	65.33333333	1.227795219	0.023028591	0.377626024
50796	'Dmrt1'	27	45	67	0	9	32	46.33333333	13.66666667	-1.974471596	0.081294745	0.619717602
50797	'Copb2'	8108	8003	6624	3264	4700	6906	7578.333333	4956.666667	-0.59965314	0.034018663	0.443914809
50798	'Gne'	411	398	333	179	545	461	380.6666667	395	0.025106317	0.947995264	0.997249264
50799	'Slc25a13'	324	439	596	463	433	548	453	481.3333333	0.12441316	0.788342702	0.972790461
50817	'Capn15'	834	788	487	338	805	557	703	566.6666667	-0.281695325	0.430582462	0.962994098
50849	'Rnf10'	4312	4373	4244	2866	3936	5223	4309.666667	4008.333333	-0.089430363	0.74624079	0.972790461
50850	'Spast'	2199	2187	1067	780	2146	2537	1817.666667	1821	-0.004651689	0.991465451	0.999493374
50868	'Keap1'	1233	1190	1099	761	1226	1571	1174	1186	0.018424403	0.946628621	0.996592403
50873	'Prkn'	70.54	95.37	41.28	23	87	55	69.06333333	55	-0.315795858	0.5635485	0.972790461
50874	'Tmod4'	44	59	42	12	11	7	48.33333333	10	-2.163557059	3.44E-04	0.03332022
50875	'Tmod3'	1216	1191	2227	1792	1281	1462	1544.666667	1511.666667	0.042648191	0.937730324	0.994698142
50876	'Tmod2'	160	150	30	13	130	143	113.3333333	95.33333333	-0.289943441	0.730492434	0.972790461
50877	'Neu3'	550	554.97	344.13	461	538.01	412	483.0333333	470.3366667	0.076268413	0.836735566	0.974723675
50878	'Stag3'	5301	4694	931	30	1717	1395	3642	1047.333333	-1.863315068	0.073492835	0.599577391
50880	'Scly'	84	107	61	109	101	84	84	98	0.357961194	0.43701616	0.96622803
50883	'Chek2'	147	168	151	77	210	269	155.3333333	185.3333333	0.202623998	0.640588526	0.972790461
50884	'Nckap1'	4112	3992	4602	4562	5377	5960	4235.333333	5299.666667	0.363556914	0.258786566	0.877564912
50887	'Hmgn5'	141	185	146	338	293	234	157.3333333	288.3333333	1.005342078	0.027573955	0.406520358
50905	'Il17rb'	25	15	0	16	17	29	13.33333333	20.66666667	0.76488369	0.505647449	0.972790461
50907	'Preb'	1147	1228	2058	1909	1432	1584	1477.666667	1641.666667	0.226586022	0.665011732	0.972790461
50908	'C1s1'	1031.8	832	4755.6	1602	698	1535	2206.48	1278.33	-0.781999671	0.355521144	0.931809687
50909	'C1ra'	529.41	507.11	1192.5	344.63	570.98	705.09	743.0133333	540.2333333	-0.532811787	0.327191952	0.920734892
50911	'Exosc9'	804.05	822.85	389	402.13	850.09	1055.23	671.9666667	769.15	0.211977961	0.591392563	0.972790461
50912	'Exosc10'	1412	1421	1172	361	1312	1057	1335	910	-0.595892252	0.158406785	0.768890221
50913	'Olig2'	0	0	1	0	0	0	0.333333333	0	-0.903279821	0.824807108	0.972790461
50914	'Olig1'	3	1	0	0	0	0	1.333333333	0	-2.70051453	0.497202575	0.972790461
50915	'Grb14'	186	156	344	116	298	268	228.6666667	227.3333333	-0.094372074	0.861291947	0.976863639
50916	'Irx4'	1	4	0	0	0	0	1.666666667	0	-3.011457993	0.447447746	0.970198456
50917	'Galns'	163	168	84	93	253	253	138.3333333	199.6666667	0.532876903	0.214386667	0.84261066
50918	'Myadm'	1536	1480	3046	2513	2096	2387	2020.666667	2332	0.249117948	0.644428933	0.972790461
50926	'Hnrnpdl'	4138.1	4200.2	7008.9	5038	4332.8	3457.5	5115.73	4276.103333	-0.17488824	0.739379309	0.972790461
50927	'Nasp'	4974	5226	2289.3	608.97	3705.3	3072	4163.1	2462.09	-0.80129044	0.174312636	0.793128181
50931	'Il27ra'	128.61	122	221.58	25	107.64	61.94	157.3966667	64.86	-1.381045332	0.021826021	0.365836629

50932	'Mink1'	1769	1750	960	3106	1882	2470	1493	2486	0.913983427	0.056388406	0.545987502
50933	'Uchl3'	554	631	515	586	538	854	566.6666667	659.3333333	0.281472185	0.410332755	0.95722888
50934	'Slc7a8'	180	182	874	301	330	272	412	301	-0.490984308	0.530149764	0.972790461
50935	'St6galnac6'	620	658	236	2035	788	1082	504.6666667	1301.666667	1.633853331	0.011724029	0.268550885
50995	'Uba2'	3972	4035	2598	1444	3210	4412	3535	3022	-0.240749763	0.514828534	0.972790461
50996	'Pdcd7'	995	1026	781	301	735	1159	934	731.6666667	-0.396640471	0.350261318	0.92886351
50997	'Mpp2'	405	353	2153	701	309	660	970.3333333	556.6666667	-0.801084246	0.358581358	0.933467324
51786	'Cpsf2'	1294.1	1262	972	918	1393	1333	1176.033333	1214.666667	0.093673101	0.693709535	0.972790461
51788	'H2az1'	3039	3291	4210	4177	3323	3244	3513.333333	3581.333333	0.126358819	0.785929763	0.972790461
51789	'Tnk2'	1866	1850	777	1136	1968	1823	1497.666667	1642.333333	0.201705492	0.599883267	0.972790461
51791	'Rgs14'	2	1	1	0	7	4	1.333333333	3.666666667	1.362228343	0.43140468	0.963623551
51792	'Ppp2r1a'	3508	3461	5038	2555	4078	4119	4002.333333	3584	-0.172483786	0.649895973	0.972790461
51793	'Ddah2'	1105	1151	1598	1399	1371	1225	1284.666667	1331.666667	0.12011891	0.786492265	0.972790461
51795	'Srpx'	56	50	9	8	86	52	38.33333333	48.66666667	0.325083539	0.714814866	0.972790461
51796	'Srrm1'	5939	5489	4005	3955	4766	3742	5144.333333	4154.333333	-0.207797367	0.535291326	0.972790461
51797	'Ctps'	1576	1630	2707	1550	1180	1620	1971	1450	-0.3894309	0.434245614	0.965118278
51798	'Ech1'	1146	1239	828	616	1640	1772	1071	1342.666667	0.306404985	0.403371328	0.955996639
51799	'Rundc3a'	36	34	44	18	47	36	38	33.66666667	-0.203001524	0.696310241	0.972790461
51800	'Bok'	317	384	307	858	343	380	336	527	0.878399171	0.157385464	0.768337387
51801	'Ramp1'	50	36	9	21	142	121	31.66666667	94.66666667	1.558804931	0.046197292	0.50074283
51810	'Hnrnpu'	16719	17679	10003	13639	17988	17436.2	14800.43	16354.15	0.227517498	0.44597684	0.969915971
51812	'Mcrs1'	1175	1358	661	403	1150	1336	1064.666667	963	-0.15752814	0.71622318	0.972790461
51813	'Ccnc'	520	587	268	110	473	497	458.3333333	360	-0.383160527	0.470591829	0.972790461
51869	'Rif1'	3601	3779	1353	1929	3415	3826	2911	3056.666667	0.128798422	0.761225391	0.972790461
51875	'Tmem141'	135	148	110	71	166	143	131	126.6666667	-0.044058083	0.900099597	0.986505007
51885	'Tubgcp4'	774.99	725.39	339.98	354.27	900.1	760.59	613.4533333	671.6533333	0.158045254	0.701531308	0.972790461
51886	'Fubp1'	5041	5134	3685	2333	5224	3725	4620	3760.666667	-0.274586385	0.378745805	0.94215965
51897	'Atg13'	1385	1341	3639	1453	1043	1567	2121.666667	1354.333333	-0.632018555	0.31281368	0.911912367
51902	'Rnf24'	459.6	499.64	471.8	2195.8	498.51	639.83	477.0133333	1111.383333	1.512380635	0.047348607	0.50504201
51938	'Ccdc39'	111.19	116.77	17.97	15.26	135.01	50.62	81.97666667	66.96333333	-0.280076482	0.747912512	0.972790461
51944	'Knstrn'	295	306	79	169	268	229	226.6666667	222	0.07544376	0.886146023	0.984348372
51960	'Kctd18'	458	454	369	125	444	389	427	319.3333333	-0.461593089	0.279664608	0.889355605
52004	'Cdk2ap2'	349	382	1227	1237	343	676	652.6666667	752	0.325794703	0.693374456	0.972790461
52009	'Jpt2'	1256	1413	1943	220	1094	1690	1537.333333	1001.333333	-0.761858939	0.228963061	0.855540033
52013	'R3hcc1l'	674	710	902	477	644	666	762	595.6666667	-0.339148011	0.34271092	0.925308103
52014	'Nus1'	1817	1834	3381	3065	2115	2703	2344	2627.666667	0.233358806	0.66738954	0.972790461
52020	'Umodl1'	3	4	0	0	6	4	2.333333333	3.333333333	0.487967822	0.797131008	0.972790461
52023	'Pibf1'	337.92	351.56	315.35	133.18	335.64	302.17	334.9433333	256.9966667	-0.400946292	0.247634537	0.868994456
52024	'Ankrd22'	1	0	0	0	3	3	0.333333333	2	2.349183361	0.41255442	0.95722888
52028	'Bbs1'	574	611	149	160	390	272	444.6666667	274	-0.629404447	0.257911792	0.877405867

52033	'Pbk'	308	342	83	28	281	218	244.3333333	175.6666667	-0.51363173	0.503734983	0.972790461
52036	'Ppp6r3'	3499	3507	2240	3613	3792	3884	3082	3763	0.393775279	0.227302128	0.854001521
52040	'Ppp1r10'	2447	2417	1131	2499	1882	2284	1998.333333	2221.666667	0.313253138	0.486818494	0.972790461
52055	'Rab11fip5'	2018	2125	2265	1997	2258	2275	2136	2176.666667	0.084727298	0.798517935	0.972790461
52064	'Coq5'	514	481	378	361	418	562	457.6666667	447	0.020393287	0.943111675	0.996301887
52065	'Mfhas1'	384	360	599	426	342	403	447.6666667	390.3333333	-0.134350909	0.785257428	0.972790461
52076	'Tmem38b'	273	300	525	3705	289	726	366	1573.333333	2.416528653	0.014740459	0.302641036
52118	'Pvr'	256	289	1016	1283	364	618	520.3333333	755	0.663081877	0.437537469	0.96622803
52120	'Hgsnat'	667	644	240	528	830	1130	517	829.3333333	0.738232121	0.09147233	0.650717463
52123	'Agpat5'	1038	1050	701	532	1083	1435	929.6666667	1016.666667	0.124563302	0.712988054	0.972790461
52132	'Ccdc97'	820	780	842	581	764	808	814	717.6666667	-0.147218154	0.617803691	0.972790461
52150	'Kcnk6'	50	52	42	20	50	57	48	42.33333333	-0.198594276	0.665014466	0.972790461
52163	'Camk1'	480.58	559.85	650.79	219.89	606.95	636.91	563.74	487.9166667	-0.273508444	0.522692419	0.972790461
52174	'Tmem222'	641	597	663	309	572	702	633.6666667	527.6666667	-0.285358887	0.386618058	0.948558849
52184	'Odf2l'	1019	1014	409	669	911	512	814	697.3333333	-0.085055604	0.859907295	0.976863639
52187	'Rragd'	562	534	2126	868	705	1101	1074	891.3333333	-0.305017252	0.671278755	0.972790461
52202	'Rbm34'	705	726	532	409	516	598	654.3333333	507.6666667	-0.308714691	0.233698267	0.860393388
52206	'Anapc4'	2305	2192	2250	482	1841	2120	2249	1481	-0.68413001	0.154355029	0.766453805
52231	'Ankzf1'	723.82	687.49	879.66	329.85	663.19	472.73	763.6566667	488.59	-0.650137611	0.084276882	0.631312878
52245	'Commd2'	341	397	326	349	401	432	354.6666667	394	0.216552039	0.474278988	0.972790461
52250	'Reep1'	4323	4345	3330	4045	5218	4790	3999.333333	4684.333333	0.300403822	0.265059915	0.88128484
52276	'Cdca8'	413	467	210	86	321	427	363.3333333	278	-0.422569976	0.431544786	0.963677723
52323	'Klhl7'	1549	1523	1985	844	1445	1515	1685.67	1267.996667	-0.426692604	0.22089228	0.849076011
52331	'Stbd1'	297	275	1153	444	282	428	575	384.6666667	-0.577393085	0.445110829	0.969888068
52335	'Atxn1l'	1635	1568	971	1242	1348	1511	1391.333333	1367	0.070209487	0.825331202	0.972790461
52348	'Vps37a'	1440.4	1314.2	1086.3	1244.7	1474.8	1200.29	1280.286667	1306.593333	0.120670609	0.713912781	0.972790461
52357	'Wwc2'	1434	1485	1597	3345	1552	2785	1505.333333	2560.666667	0.906310038	0.083847613	0.628858794
52372	'D6Ertd527e'	9	8	124	34.53	6	25	47	21.84333333	-1.099658012	0.378047864	0.94187472
52377	'Rcn3'	1954	1888	1596	1471	2110	2503	1812.666667	2028	0.192941028	0.412562819	0.95722888
52389	'Adgra1'	1	0	0	0	3	1	0.333333333	1.333333333	1.784737841	0.575297357	0.972790461
52392	'Macir'	397	393	718	150	416	548	502.6666667	371.3333333	-0.547366776	0.312477776	0.911537035
52397	'Zfp644'	2180	2221	1054	1170	2136	1999	1818.333333	1768.333333	0.014014873	0.96788186	0.999493374
52398	'Septin11'	2785	2733	4150	2986	2366	2315	3222.666667	2555.666667	-0.245822496	0.616644074	0.972790461
52428	'Rhpn2'	195	166	237	253	129	506	199.3333333	296	0.584623926	0.321190229	0.917442833
52430	'Echdc2'	369	410	415	280	510	390	398	393.3333333	-0.001276277	0.996916073	0.9996806
52432	'Ppp2r2d'	810	805	1224	890	802	1021	946.3333333	904.3333333	-0.02180489	0.960902632	0.999493374
52440	'Tax1bp1'	3104	3203	2822	3870	3622	4270	3043	3920.666667	0.45273723	0.172072596	0.789631715
52443	'Mrpl48'	889	999	705	294	717	666	864.3333333	559	-0.633653852	0.051359215	0.525533587
52463	'Tet1'	686	712	639	279	637	340	679	418.6666667	-0.67700868	0.07974287	0.617305615
52466	'Slc46a1'	175	194	135	118	259	388	168	255	0.576982891	0.15533066	0.766453805

52468	'Ctdsp2'	8530	8236	10453	4656	7710	7673	9073	6679.666667	-0.446631538	0.178136686	0.797629457
52469	'Coa3'	331	371	349	293	425	440	350.3333333	386	0.168033979	0.556011608	0.972790461
52477	'Angel2'	1185	1292	630	177	1038	730	1035.666667	648.3333333	-0.71591337	0.206052129	0.833294914
52502	'Carhsp1'	2416	2480	5649	4524	2204	3084	3515	3270.666667	-0.006508437	0.992094078	0.999493374
52504	'Cenpo'	210.51	192.54	163.32	94	197.53	178.4	188.79	156.6433333	-0.259304182	0.414293109	0.95722888
52513	'Ddx56'	758	797	774	341	724	824	776.3333333	629.6666667	-0.327231306	0.318515803	0.916136994
52521	'Zfp622'	1147	1167	1586	902	1019	1326	1300	1082.333333	-0.247613181	0.518221413	0.972790461
52530	'Nhp2'	441	475	409	257	519	595	441.6666667	457	0.037873395	0.902208733	0.987328425
52535	'Mettl17'	196	176	163	85	186	124	178.3333333	131.6666667	-0.420521016	0.246983965	0.868602029
52538	'Acaa2'	2884	3413	957	1410	5079	4706	2418	3731.666667	0.633157063	0.255036695	0.874595535
52551	'Sgta'	1499	1477	2002	1507	1560	1937	1659.333333	1668	0.04265899	0.912415803	0.989773583
52552	'Parp8'	797	754	1099	400	1104	745	883.3333333	749.6666667	-0.285509113	0.520881771	0.972790461
52563	'Cdc23'	1732.6	1691.8	1329.3	991.52	2065.7	1780.03	1584.546667	1612.43	0.038961615	0.887773093	0.984534762
52570	'Ccdc69'	19	22	32	20	49	33	24.33333333	34	0.451791151	0.442227819	0.969177389
52575	'Trmt10c'	439	397	777	345	435	545	537.6666667	441.6666667	-0.304014574	0.524577208	0.972790461
52585	'Dhrs1'	553	573	287	256	639	750	471	548.3333333	0.219766964	0.591953124	0.972790461
52588	'Tspan14'	895	902	362	1512	1175	1129	719.6666667	1272	1.005835699	0.043653045	0.487049111
52589	'Ncald'	194	196	59	25	207	121	149.6666667	117.6666667	-0.372441806	0.604173306	0.972790461
52592	'Brms1l'	893	1010	295	874	1179	947	732.6666667	1000	0.576167895	0.236961881	0.862272407
52609	'Cbx7'	175	182	149	427	222	310	168.6666667	319.6666667	1.089288804	0.034019771	0.443914809
52614	'Adgre4'	1	2	0	0	1	0	1	0.333333333	-1.324111362	0.694133627	0.972790461
52615	'Suz12'	2372	2412	2583	2732	2236	2203	2455.666667	2390.333333	0.070929586	0.866187663	0.978294615
52626	'Cdkn2aipnl'	777	849	552	535	796	1127	726	819.3333333	0.200234649	0.500123113	0.972790461
52633	'Nit2'	539	566	429	129	618	666	511.3333333	471	-0.192113565	0.714404117	0.972790461
52635	'Esyt2'	1830	1834	1116	2337	1950	1682	1593.333333	1989.666667	0.47932389	0.269085927	0.883851074
52637	'Cisd1'	361	330	222	139	309	493	304.3333333	313.6666667	0.020085804	0.962381203	0.999493374
52639	'Wipi1'	537	490	1015	1352	448	566	680.6666667	788.6666667	0.393083902	0.590197331	0.972790461
52653	'Nudcd2'	507	548	620	329	637	661	558.3333333	542.3333333	-0.062773385	0.853531109	0.975734242
52662	'Ldlrad4'	455.11	451.03	251.29	1320.9	422.51	476.69	385.81	740.02	1.230746462	0.074916245	0.603900623
52665	'Echdc1'	639.47	662.62	341.25	214.17	516.67	466.19	547.78	399.01	-0.437054918	0.241642087	0.86449675
52666	'Arhgef25'	1479	1360	1063	619	1292	1128	1300.666667	1013	-0.345484111	0.220635898	0.849076011
52668	'Ifi27'	1056	1242	1394	5479	1133	2807	1230.666667	3139.666667	1.575668769	0.029572063	0.419705596
52670	'Cpsf4l'	10	15	3	4	10	60	9.333333333	24.66666667	1.338770269	0.216792281	0.843391934
52679	'E2f7'	125	119	47	135	94	78	97	102.3333333	0.286510902	0.626489866	0.972790461
52683	'Ncaph2'	2379.8	2394.9	2664	1644.8	2204	2977.82	2479.546667	2275.536667	-0.117386832	0.708401442	0.972790461
52685	'Cd300lg'	0	0	0	2	1	1	0	1.333333333	3.039006733	0.327321392	0.920734892
52686	'Mettl2'	758.03	764	862	308	637	600	794.6766667	515	-0.644714473	0.053447686	0.533767484
52690	'Setd3'	3390	3220	3194	2427	2633	3737	3268	2932.333333	-0.114524432	0.705786878	0.972790461
52696	'Zwint'	2647	2972	5152	4916	3317	3921	3590.333333	4051.333333	0.255122848	0.643967161	0.972790461
52700	'Txndc17'	635	698	1455	448	697	986	929.3333333	710.3333333	-0.458846095	0.389537757	0.951231321

52705	'Krr1'	924.93	953.9	1184.4	677	901.85	987.69	1021.063333	855.5133333	-0.239140806	0.486410062	0.972790461
52708	'Zfp410'	1083	1116	1197	563	959	974	1132	832	-0.442648596	0.13511885	0.736012522
52710	'Slc52a2'	268	306	138	239	356	388	237.3333333	327.6666667	0.532473444	0.136834845	0.738025359
52712	'Zkscan6'	364	368	497	286	342	388	409.6666667	338.6666667	-0.252788598	0.510284955	0.972790461
52713	'Ccdc59'	326	362	754	294	288	458	480.6666667	346.6666667	-0.492131453	0.370942575	0.9384981
52715	'Ccdc43'	497.2	461.01	641.02	242.01	543	445	533.0766667	410.0033333	-0.410906486	0.299931297	0.903723114
52717	'Anapc16'	901	922	1364	769	901	1027	1062.333333	899	-0.224551202	0.581689566	0.972790461
52793	'Fam3b'	4	6	1	1	2	10	3.666666667	4.333333333	0.217645741	0.8740478	0.980171118
52808	'Tspyl2'	1418	1366	4572	875	2212	1899	2452	1662	-0.684099734	0.300705803	0.904162378
52815	'Ldhd'	72.55	81.03	58.07	210.63	169.53	251.45	70.55	210.5366667	1.674823788	3.63E-05	0.006774907
52822	'Rufy3'	836	805	399	438	894	887	680	739.6666667	0.157011337	0.664628147	0.972790461
52829	'Lurap1l'	63	54	69	120	91	91	62	100.6666667	0.809103333	0.110781242	0.692820059
52830	'Pnrc2'	1984	2191	3113	1337	2725	2516	2429.333333	2192.666667	-0.186891211	0.645916072	0.972790461
52837	'Tmx4'	2555	2472	1247	1102	2186	2575	2091.333333	1954.333333	-0.072465891	0.841920106	0.975182082
52838	'Dnlz'	358	405	293	122	397	352	352	290.3333333	-0.308999665	0.449411715	0.97049895
52840	'Dbndd2'	119	100	164	141	119	183	127.6666667	147.6666667	0.24559337	0.608932204	0.972790461
52846	'Cnot11'	712	737	789	618	758	821	746	732.3333333	0.013100337	0.96651492	0.999493374
52850	'Sgsm1'	67.97	45	63	64	64	84	58.65666667	70.66666667	0.323951254	0.47273566	0.972790461
52855	'Lair1'	20	34	1	0	40	39	18.33333333	26.33333333	0.475777623	0.745830769	0.972790461
52856	'Mtg2'	310	297	242	83	234	239	283	185.3333333	-0.638485852	0.097099315	0.663789939
52857	'Gramd1a'	874	744	1497	1650	1112	853	1038.333333	1205	0.331910199	0.586804904	0.972790461
52858	'Cdipt'	899	918	764	936	1283	1490	860.3333333	1236.333333	0.56115866	0.019447644	0.347725165
52864	'Slx4'	809	758	370	501	791	557	645.6666667	616.3333333	0.026262596	0.946422262	0.996592403
52874	'Pum3'	1467	1499	1675	548	1231	1361	1547	1046.666667	-0.603425504	0.091522117	0.650717463
52882	'Rgs7bp'	22	24	23	52	42	30	23	41.33333333	0.980525571	0.103176618	0.673624266
52892	'Sco1'	428	437	392	223	402	456	419	360.3333333	-0.216157707	0.446578176	0.970198456
52897	'Rbfox3'	41	37	43	5	32	29	40.33333333	22	-0.961574694	0.150231169	0.759693458
52898	'Rnasek'	262	280	522	410	253	462	354.6666667	375	0.125731125	0.825203654	0.972790461
52906	'Ahi1'	691	647	439	439	511	432	592.3333333	460.6666667	-0.25995713	0.446968073	0.970198456
52915	'Zmiz2'	1865	1755	1368	989	1537	1827	1662.666667	1451	-0.169269923	0.487489564	0.972790461
53310	'Dlg3'	931	986	859	708	785	1028	925.3333333	840.3333333	-0.08709065	0.762237788	0.972790461
53311	'Mybph'	3.18	0	0.68	0	1	1.56	1.286666667	0.853333333	-0.57225899	0.860284643	0.976863639
53312	'Nub1'	2041	2148	3776	1848	2115	2869	2655	2277.333333	-0.232973789	0.617278136	0.972790461
53313	'Atp2a3'	83	114	18	131	210	122	71.66666667	154.3333333	1.247383291	0.057661028	0.549224181
53314	'Batf'	9	9	65	20	7	13	27.66666667	13.33333333	-1.020252049	0.351933416	0.929687107
53315	'Sult1d1'	0	0	0	17	0	1	0	6	5.475545928	0.058381184	0.551665011
53317	'Plrg1'	1223	1252	1286	971	1145	1543	1253.666667	1219.666667	-0.010004848	0.973768596	0.999493374
53318	'Pdlim3'	41	38	64	5	49	61	47.66666667	38.33333333	-0.473432349	0.554876019	0.972790461
53319	'Nxf1'	4905	5195	2824	2040	4966	3043	4308	3349.666667	-0.324575646	0.395270477	0.954014376
53320	'Folh1'	2	8	1	1	15	5	3.666666667	7	0.918689095	0.500446832	0.972790461

53321	'Cntnap1'	348	337	253	433	386	255	312.6666667	358	0.349693542	0.457612832	0.972516785
53322	'Nucb2'	238	260	1020	285	410	866	506	520.3333333	-0.10538533	0.888471999	0.984632659
53323	'Ube2k'	1733	1918	1961	989	1444	1584	1870.666667	1339	-0.466157534	0.10606993	0.679124905
53324	'Nptx2'	444	402	124	60	281	295	323.3333333	212	-0.62587406	0.320428522	0.917329173
53325	'Banp'	684	624	629	996	660	726	645.6666667	794	0.439898152	0.342058088	0.925308103
53328	'Pgrmc1'	2666	2480	4276	2666	3820	5902	3140.666667	4129.333333	0.34811444	0.441216025	0.968551476
53330	'Vamp4'	1171	1181	986	1147	991	1012	1112.666667	1050	0.033078024	0.930248939	0.99325468
53331	'Stx7'	1091	1137	2153	1402	1040	1392	1460.333333	1278	-0.143526712	0.790063119	0.972790461
53332	'Mtmr1'	867	893	729	569	919	635	829.6666667	707.6666667	-0.173220962	0.581464539	0.972790461
53333	'Tomm40'	693	669	1142	624	728	944	834.6666667	765.3333333	-0.129209534	0.771446457	0.972790461
53334	'Gosr1'	1038	989	1184	715	1131	1276	1070.333333	1040.666667	-0.043725685	0.890398521	0.984980969
53356	'Eif3g'	2093	2047	2585	1279	1676	2954	2241.666667	1969.666667	-0.210338473	0.592440506	0.972790461
53357	'Pla2g6'	699	672	778	492	734	899	716.3333333	708.3333333	-0.016096193	0.959331965	0.999493374
53374	'Chst3'	223	206	238	145	144	205	222.3333333	164.6666667	-0.390766014	0.294364008	0.90029104
53375	'Mtx2'	1134	1096	850	397	1036	1242	1026.666667	891.6666667	-0.234462414	0.532283316	0.972790461
53376	'Usp2'	114	97	57	218	99	301	89.33333333	206	1.330482848	0.019275044	0.346695486
53378	'Sdcbp'	2809	2893	8399	7730	3656	5190	4700.333333	5525.333333	0.314452111	0.660623877	0.972790461
53379	'Hnrnpa2b1'	16244	17867	9657	12529	18491	12988	14589.33333	14669.33333	0.10168069	0.772450867	0.972790461
53380	'Psmd10'	657	612	657	514	554	767	642	611.6666667	-0.030194611	0.925782202	0.992381554
53381	'Prdx4'	1087	1164	682	247	1167	1653	977.6666667	1022.333333	-0.006385617	0.991067833	0.999493374
53382	'Txnl1'	2162	2159	5390	1320	1935	2621	3237	1958.666667	-0.796396504	0.158402644	0.768890221
53404	'Atoh7'	1	0	0	0	0	0	0.333333333	0	-0.903279821	0.824807108	0.972790461
53412	'Ppp1r3c'	392	302	1106	550	252	332	600	378	-0.579908709	0.44326116	0.969836424
53413	'Exoc7'	1116	1099	838	585	1211	1238	1017.666667	1011.333333	-0.004324711	0.987779284	0.999493374
53414	'Bysl'	374.1	415.49	370.79	600.99	458.1	571.06	386.7933333	543.3833333	0.600219952	0.137123848	0.738570697
53415	'Htatip2'	66	72	22	347	84	266	53.33333333	232.3333333	2.372542482	0.001439433	0.082373028
53416	'Stk39'	1350	1440	1326	439	1956	1965	1372	1453.333333	0.002130004	0.996592518	0.999616639
53417	'Hif3a'	1134	1011	1809	626	860	787	1318	757.6666667	-0.796931277	0.073544647	0.599584406
53418	'B4galt2'	685	683	493	55	785	837	620.3333333	559	-0.265415399	0.726901425	0.972790461
53419	'Corin'	27	14	3	9	10	8	14.66666667	9	-0.548812063	0.556181603	0.972790461
53420	'Syt5'	33	14	43	78	17	38	30	44.33333333	0.750682879	0.394272582	0.953178011
53421	'Sec61a1'	2562	2747	5024	2590	3427	4593	3444.333333	3536.666667	0.003755298	0.993710486	0.999493374
53422	'Ybx2'	1484	1444	306	441	1228	2639	1078	1436	0.402566291	0.56229317	0.972790461
53424	'Tsnax'	2557	2485	2306	1325	2225	2496	2449.333333	2015.333333	-0.272853702	0.281665212	0.889731741
53598	'Dctn3'	463	438	810	472	425	622	570.3333333	506.3333333	-0.155757349	0.75265352	0.972790461
53599	'Cd164'	4728	4648	3497	5741	5309	6343	4291	5797.666667	0.534851549	0.104798389	0.67684333
53600	'Timm23'	1237	1326	1780	876	1166	1742	1447.666667	1261.333333	-0.217722365	0.57986783	0.972790461
53601	'Pcdh12'	247	256	14	5	393	186	172.3333333	194.6666667	0.141817319	0.906096405	0.988799253
53602	'Hpcal1'	1055	1055	1811	1224	1374	1486	1307	1361.346667	0.074427151	0.870178152	0.979794316
53603	'Tslp'	2	3	5	0	1	4	3.333333333	1.666666667	-1.156886446	0.480024941	0.972790461

53604	'Zpbp'	391	367	97	22	233	303	285	186	-0.674039247	0.407193106	0.957157474
53605	'Nap1l1'	10452	9689	12475	4546	6964	8215	10872	6575	-0.733540734	0.027855278	0.408314996
53607	'Snrpa'	1182	1260	1279	554	1047	1409	1240.333333	1003.333333	-0.335317759	0.327225374	0.920734892
53608	'Map3k6'	59	63	151	81	96	166	91	114.3333333	0.267619686	0.662386905	0.972790461
53609	'Clasrp'	867	871	537	799	922	592	758.3333333	771	0.153351236	0.710474075	0.972790461
53610	'Nono'	8054	8005	11101	4771	8061	9060	9053.333333	7297.333333	-0.336292101	0.3628044	0.934765857
53611	'Vti1a'	477.2	491.14	387.87	803	554.52	635.02	452.07	664.18	0.700706604	0.109265021	0.689232441
53612	'Vti1b'	1084	1005	1138.8	392.61	688	913	1075.936667	664.5366667	-0.717885867	0.032812568	0.436246529
53614	'Reck'	721	701	214	617	735	708	545.3333333	686.6666667	0.463797305	0.344780259	0.926100052
53617	'Krt35'	0	0	0	1	0	0	0	0.333333333	1.020273531	0.802557913	0.972790461
53618	'Fut8'	2183.7	2399	605.78	612.23	2523.7	1867.91	1729.493333	1667.933333	-0.03880712	0.948260975	0.997385684
53619	'Blcap'	862	887	614	716	946	1007	787.6666667	889.6666667	0.238133686	0.352623211	0.930050349
53620	'Vamp5'	62	70	67	103	60	77	66.33333333	80	0.408235118	0.445183809	0.969888068
53621	'Cnot4'	1196	1153	1035	940	960	971	1128	957	-0.149184412	0.649970843	0.972790461
53622	'Krt85'	0	1	0	0	1	1	0.333333333	0.666666667	0.817382893	0.82412705	0.972790461
53623	'Gria3'	348	386	58	133	455	324	264	304	0.250767674	0.714532392	0.972790461
53624	'Cldn7'	9	10	20	18	7	41	13	22	0.731679162	0.410128843	0.957157474
53625	'B3gnt2'	340	318	367	290	539	730	341.6666667	519.6666667	0.572770353	0.113596368	0.698948431
53626	'Insm1'	23	31	4	0	14	3	19.33333333	5.666666667	-1.788756369	0.171211625	0.787010498
53627	'Porcn'	51	34	103	9	35	43	62.66666667	29	-1.256046579	0.088578373	0.644243319
53761	'Prrc2a'	11420	10562	5924	6183	10585	9515	9302	8761	-0.028158951	0.928297977	0.993096978
53791	'Tlr5'	175	131	61	18	155	133	122.3333333	102	-0.315765389	0.656054886	0.972790461
53814	'Oaz3'	18.27	28.33	9	6	7	6.32	18.53333333	6.44	-1.42476863	0.067461071	0.580020123
53817	'Ddx39b'	2905	2948	3639	764	3325	2886	3164	2325	-0.53779159	0.28760341	0.894952226
53856	'Prg3'	0	3	0	0	0	0	1	0	-2.275512037	0.569613276	0.972790461
53857	'Tuba8'	4	4	11	88	3	29	6.333333333	40	2.911914428	0.018078632	0.335677246
53858	'Rwdd2b'	164	219	113	33	175	92	165.3333333	100	-0.75130347	0.186096679	0.807018167
53859	'Map3k14'	110	84	192	156	102	116	128.6666667	124.6666667	0.035254844	0.954176795	0.99943739
53860	'Septin9'	3878	4125	3179	5488	5024	4990	3727.333333	5167.333333	0.586117707	0.1035915	0.675150888
53861	'Zranb2'	3307	3364	2134	1783	3084	2705	2935	2524	-0.169286421	0.532080587	0.972790461
53867	'Col5a3'	51	37	69	172	69	89	52.33333333	110	1.237914849	0.073368266	0.599174173
53868	'Rab25'	0	1	4	4	2	22	1.666666667	9.333333333	2.309338114	0.14622226	0.753611197
53869	'Rab11a'	2291	2367	3229	2578	2418	3416	2629	2804	0.128057955	0.754436874	0.972790461
53870	'Cntn6'	4	6	0	1	3	1	3.333333333	1.666666667	-0.887374874	0.606395658	0.972790461
53871	'Pkd2l2'	69.8	56.96	19.76	58.9	177.39	196.76	48.84	144.35	1.589044253	0.006227369	0.186739406
53872	'Caprin1'	9995	10145	12102	6577	10531	10284	10747.33333	9130.666667	-0.231111084	0.468228331	0.972790461
53878	'Svs2'	2	0	0	0	0	0	0.666666667	0	-1.711373851	0.67234292	0.972790461
53881	'Slc5a3'	759	697	187	11059	640	1359	547.6666667	4352.666667	3.465355494	0.001378708	0.079475261
53883	'Celsr2'	420	401	38	340	424	369	286.3333333	377.6666667	0.563946426	0.454522127	0.970649024
53885	'Nphp1'	817	804	286	55	729	907	635.6666667	563.6666667	-0.250946303	0.754989341	0.972790461

53886	'Cdkl2'	1462.6	1437	214	60	766	561.08	1037.86	462.36	-1.195614208	0.166879526	0.781229856
53890	'Sart3'	1674	1615	1252	1212	1539	1534	1513.666667	1428.333333	-0.015710123	0.95212521	0.998640168
53892	'Ppm1d'	419	392	467	7224	428	1766	426	3139.333333	3.220466897	9.52E-04	0.064169552
53893	'Nudt5'	682	733	555	367	634	819	656.6666667	606.6666667	-0.108247875	0.706524773	0.972790461
53895	'Clpp'	513	527	916	287	451	733	652	490.3333333	-0.477080125	0.328942132	0.921648675
53896	'Slc7a10'	6	7	3.02	1	8	3.01	5.34	4.003333333	-0.421543349	0.717014139	0.972790461
53897	'Gal3st1'	4	5	2	50	1	50	3.666666667	33.66666667	3.415246227	0.007006769	0.197779062
53901	'Rcan2'	204	190	1546	120	137	225	646.6666667	160.6666667	-2.142487127	0.017661411	0.332125367
53902	'Rcan3'	940	897	559	710	888	1085	798.6666667	894.3333333	0.230584102	0.429411634	0.962386546
53945	'Slc40a1'	1292	1297	1310	6101	2346	5672	1299.666667	4706.333333	1.997951595	3.12E-04	0.030714333
53951	'Gpatch11'	509	537	240	618	452	517	428.6666667	529	0.473685579	0.317093162	0.916136994
53970	'Rfx5'	1030	1048	530	355	1104	822	869.3333333	760.3333333	-0.186354676	0.661484316	0.972790461
53972	'Ngef'	170	194	39	48	99	108	134.3333333	85	-0.596277285	0.32473168	0.91982931
53975	'Ddx20'	1097	1100	524	585	811	900	907	765.3333333	-0.168692114	0.626908751	0.972790461
53978	'Lpar2'	335	376	211	441	423	477	307.3333333	447	0.654334327	0.073472803	0.599577391
54003	'Nell2'	394	390	237	9	235	689	340.3333333	311	-0.26972558	0.787589545	0.972790461
54004	'Diaph2'	502	459	434	913	645	807	465	788.3333333	0.885242821	0.036277716	0.456985794
54006	'Deaf1'	1098	1042	644	939	1228	824	928	997	0.212863223	0.572962983	0.972790461
54120	'Gipc2'	2	3	13	21	0	7	6	9.333333333	0.842188312	0.598765551	0.972790461
54122	'Uevld'	899	952.63	763	865	743.88	812	871.5433333	806.96	0.001342764	0.997104103	0.9996806
54123	'Irf7'	65	56	295	78	68	319	138.6666667	155	-0.015349113	0.98633389	0.999493374
54124	'Cks1b'	398	420	1194	462	446	476	670.6666667	461.3333333	-0.543883606	0.391267929	0.952371379
54125	'Polm'	212.99	230.99	272	106	299	198	238.66	201	-0.279718559	0.519067103	0.972790461
54126	'Arhgef7'	1755	1686	1014	1180	1778	1637	1485	1531.666667	0.112319396	0.699036786	0.972790461
54127	'Rps28'	4671	4763	8062	7099	2511	4073	5832	4561	-0.196078317	0.76644945	0.972790461
54128	'Pmm2'	422	485	436	129	431	379	447.6666667	313	-0.565696363	0.179521155	0.799685147
54130	'Actr1a'	1770	1813	1597	1493	1763	1753	1726.666667	1669.666667	0.019188618	0.946590383	0.996592403
54131	'Irf3'	748	754	527	819	867	757	676.3333333	814.3333333	0.378755968	0.279713242	0.889355605
54132	'Pdlim1'	156	177	876	94	122	189	403	135	-1.699469713	0.034159007	0.444207284
54135	'Lsr'	945	999	771	1098	1344	1682	905	1374.666667	0.654210766	0.011059652	0.259994952
54137	'Acrbp'	92	101	88	66	118	38	93.66666667	74	-0.26493458	0.634542211	0.972790461
54138	'Atxn10'	2572	2630	4644	1438	2641	2522	3282	2200.333333	-0.621012082	0.172185841	0.789960718
54139	'Irf6'	72	64	206	181	44	77	114	100.6666667	-0.020048015	0.981593596	0.999493374
54140	'Avpr1a'	1	0	3	3	2	2	1.333333333	2.333333333	0.834402263	0.637331998	0.972790461
54141	'Spag5'	451	421	151	1836	341	646	341	941	1.812124181	0.02543251	0.394712562
541463	'Tex24'	0	0	2	4	6	0	0.666666667	3.333333333	2.306056326	0.362993388	0.935009831
54151	'Cyhr1'	1698.1	1820.1	1051.7	2088	2478	2479.04	1523.3	2348.346667	0.718687859	0.02127005	0.361918385
54152	'Dnal4'	354	377	392	165	356	349	374.3333333	290	-0.389058748	0.251496039	0.871252558
54153	'Rasa4'	623	615	591	419	650	581	609.6666667	550	-0.115566821	0.675041359	0.972790461
54156	'Egfl6'	488	502	18	132	863	856	336	617	0.882208773	0.371857682	0.938527219

54160	'Copg2'	2325.8	2362.2	1215.2	859.41	2154	2706.02	1967.726667	1906.48	-0.051590935	0.900506156	0.986605212
54161	'Copg1'	2576	2655	4329	2288	2633	3032	3186.666667	2651	-0.255950802	0.558491046	0.972790461
541610	'Trcg1'	0	0	0	4	0	3	0	2.333333333	3.887466434	0.247644223	0.868994456
54167	'Icos'	4	1	2	10	11	3	2.333333333	8	1.923433702	0.112660851	0.698600165
54169	'Kat6b'	2294	2181	2332	1040	2564	1934	2269	1846	-0.316521283	0.36367704	0.935022643
54170	'Rragc'	1650	1621	3314	1540	1423	2028	2195	1663.666667	-0.395057911	0.447268985	0.970198456
54188	'Cpsf4'	530	560	563	433	423	464	551	440	-0.249278815	0.482661081	0.972790461
54189	'Rabep1'	2242	2275	2282	2913	1950	2638	2266.333333	2500.333333	0.258655832	0.549807187	0.972790461
54194	'Akap8l'	1450	1448	1395	1011	1826	1125	1431	1320.666667	-0.077984315	0.822132619	0.972790461
54195	'Gucy1b1'	488	522	420	564	521	444	476.6666667	509.6666667	0.218271305	0.583230623	0.972790461
54196	'Pabpn1'	2952	3084.2	1318.1	1817	3962.3	2326.38	2451.426667	2701.88	0.208231551	0.627418571	0.972790461
54197	'Rnf5'	444	518	781	603	488	674	581	588.3333333	0.064911678	0.893290833	0.985171
54198	'Snx3'	1855	2057	4991	3180	1861	3020	2967.666667	2687	-0.099428032	0.876621154	0.981341203
54199	'Ccrl2'	13	12	367	328	21	70	130.6666667	139.6666667	0.26051134	0.854775148	0.975734242
54200	'Sult2b1'	3	5	6	5	16	8	4.666666667	9.666666667	1.022357051	0.279913141	0.889355605
54201	'Zfp316'	763	836	658	337	814	888.84	752.3333333	679.9466667	-0.168248251	0.619359749	0.972790461
54204	'Septin1'	66	75	30	11	107	87	57	68.33333333	0.204355086	0.782610621	0.972790461
54208	'Arl6ip1'	3441	3708	1155	2006	4025	4013	2768	3348	0.332471007	0.472719896	0.972790461
54214	'Golga4'	3959	3814	4040	5627	3391	4475	3937.666667	4497.666667	0.325945314	0.487238574	0.972790461
54215	'Cd160'	5	6	2	8	8	10	4.333333333	8.666666667	1.099494593	0.234583879	0.861206181
54216	'Pcdh7'	262	192	717	167	315	337	390.3333333	273	-0.617443513	0.344848661	0.926100052
54217	'Rpl36'	2929	2991	7766	3262	1907	2729	4562	2632.666667	-0.738100925	0.257502787	0.877405867
54218	'B3galt4'	69	62	42	5	79	92	57.66666667	58.66666667	-0.081578846	0.921247341	0.991066749
54219	'Cd320'	817	901	165	674	980	1085	627.6666667	913	0.649297423	0.268423887	0.883600995
54324	'Arhgef5'	1289	1294	849	1808	1242	1295	1144	1448.333333	0.508589846	0.263912026	0.880205046
54325	'Elovl1'	510	531	532	786	720	792	524.3333333	766	0.633138758	0.086210727	0.637027553
54326	'Elovl2'	153	156	37	7	751	457	115.3333333	405	1.741371573	0.113954127	0.698948431
54338	'Slc23a2'	1888	2126	854	9697	2890	3485	1622.666667	5357.333333	2.035870118	0.004465332	0.157418103
54342	'Gnpnat1'	425.55	496.34	1045.7	343.44	485.68	591.98	655.8733333	473.7	-0.519727687	0.334157096	0.921648675
54343	'Atf7ip'	3131	3286	503	3007	3719	3086	2306.666667	3270.666667	0.664120827	0.302768826	0.905839913
54351	'Elp5'	521.15	579.43	945.98	481	592.33	915	682.1866667	662.7766667	-0.06987188	0.880280853	0.982087554
54352	'Irx5'	450	549	296	57	226	257	431.6666667	180	-1.304581201	0.009527193	0.240209487
54353	'Skap2'	495	487	416	552	390	632	466	524.6666667	0.272679671	0.497705032	0.972790461
54354	'Rassf5'	17.33	25.42	176.85	528.81	13.3	78.89	73.2	207	1.750309342	0.19167932	0.813837363
54357	'Epb41l4b'	663	583	189	106	471	968	478.3333333	515	0.055807875	0.937349927	0.994594529
54364	'Rpp30'	422	380	376	132	294	369	392.6666667	265	-0.598790594	0.0983901	0.666877924
54366	'Ctnnal1'	1315	1379	1111	2519	1980	1837	1268.333333	2112	0.877476403	0.039150834	0.462320279
54367	'Zfp326'	1124	1188	785	278	1318	1061	1032.333333	885.6666667	-0.27118075	0.584800683	0.972790461
54368	'Gp9'	1	2	1	14	0	1	1.333333333	5	2.349721448	0.221864746	0.849517424
54369	'Nme6'	302	313	524	51	250	303	379.6666667	201.3333333	-1.056214643	0.091136522	0.649965349

54371	'Chst2'	372	367	278	1248	509	397	339	718	1.347279044	0.043248313	0.484697385
54373	'Prss16'	36	50	30	22	11	5	38.66666667	12.66666667	-1.371253017	0.089197839	0.645538378
54375	'Azin1'	2594	2622	4401	2660	2404	2453	3205.666667	2505.666667	-0.30053452	0.536368543	0.972790461
54376	'Cacng3'	12	13	30	0	2	2	18.33333333	1.333333333	-3.895480447	0.001136647	0.070791417
54377	'Cacng4'	105	96	106	2	99	66	102.3333333	55.66666667	-1.023731377	0.276461306	0.888034012
54378	'Cacng6'	8	3	0	1	1	4	3.666666667	2	-0.809311829	0.639154439	0.972790461
54380	'Smarcal1'	949	1005	693	507	1030	972	882.3333333	836.3333333	-0.060117736	0.829592003	0.973678583
54381	'Cpq'	687	764	261	260	1013	830	570.6666667	701	0.296689245	0.57999343	0.972790461
54383	'Phc2'	1543	1518	1602	2508	1653	1725	1554.333333	1962	0.477564864	0.314850525	0.914723408
54384	'Mtmr7'	494.61	438.81	141.72	332.27	339.24	516.71	358.38	396.0733333	0.256853687	0.609924358	0.972790461
54387	'Mcm3ap'	2337.8	2216	709	624.91	2185.9	2217	1754.25	1675.92	-0.063577664	0.908235814	0.988823459
54388	'H1f9'	1	0	0	0	0	0	0.333333333	0	-0.903279821	0.824807108	0.972790461
54390	'Sit1'	1	0	0	1	0	1	0.333333333	0.666666667	1.054214044	0.766400104	0.972790461
54391	'Rfk'	912	842	1119	857	811	1386	957.6666667	1018	0.109399088	0.785634912	0.972790461
54392	'Ncapg'	1997.3	2052.2	432.55	52.78	943.48	1254.88	1494	750.38	-1.061409355	0.242287431	0.86449675
54393	'Gabbr1'	1046	889	402	263	1177	468	779	636	-0.279817035	0.627128617	0.972790461
54394	'Crlf3'	600	602	541	380	580	557	581	505.6666667	-0.166237613	0.517231107	0.972790461
54396	'Irgm2'	310.49	286.98	343	339	260	673	313.49	424	0.453136448	0.334978032	0.922183948
54397	'Ppt2'	233	257	209	519	379	306	233	401.3333333	0.945796679	0.053443562	0.533767484
54399	'Bet1l'	389	389	876	360	353	488	551.3333333	400.3333333	-0.471322584	0.393248737	0.953141889
54401	'Ywhab'	3748	3820	3199	3248	4544	5052	3589	4281.333333	0.293079133	0.20021846	0.82759347
54402	'Stk19'	173	161	183	180	182	181	172.3333333	181	0.1435957	0.712087856	0.972790461
54403	'Slc4a4'	768	687	1784	698	631	871	1079.666667	733.3333333	-0.562686743	0.333132182	0.921648675
54405	'Ndufa1'	1175	1216	568	404	517	711	986.3333333	544	-0.789519211	0.029910002	0.421360177
54409	'Ramp2'	228	258	68	5	193	148	184.6666667	115.3333333	-0.749870872	0.436213397	0.965982602
54411	'Atp6ap1'	1364	1322	1249	1419	2161	2540	1311.666667	2040	0.653036194	0.010040306	0.247582744
54418	'Fmn2'	64	76	56	63	74	56	65.33333333	64.33333333	0.068605922	0.872720251	0.980171118
54419	'Cldn6'	7	6	18	183	3	25	10.33333333	70.33333333	3.094428613	0.018774195	0.341238046
54420	'Cldn8'	6	2	2	7	1	8	3.333333333	5.333333333	0.832528743	0.52095009	0.972790461
54422	'Barhl1'	3	3	1	2	4	3	2.333333333	3	0.424497813	0.747187318	0.972790461
54426	'Hgfac'	7	3	8	1	6	4	6	3.666666667	-0.7896634	0.489708511	0.972790461
54427	'Dnmt3l'	1	3	19	10	1	4	7.666666667	5	-0.504551822	0.735929416	0.972790461
54445	'Unc93b1'	157	146	52	204	257	266	118.3333333	242.3333333	1.142688739	0.013326239	0.288185595
54446	'Nfat5'	7771	7653	2726	10097	10389	6875	6050	9120.333333	0.768774779	0.137402818	0.738626463
54447	'Asah2'	227	267	129	186	362	392	207.6666667	313.3333333	0.624606642	0.083385896	0.627571099
54448	'Il1f6'	1	0	11	0	0	2	4	0.666666667	-2.861113237	0.278026186	0.889355605
54450	'Il1f5'	0	0	1	0	0	1	0.333333333	0.333333333	0.058500858	0.988561293	0.999493374
54451	'Cpsf3'	1791	1745	1339	519	1662	1814	1625	1331.666667	-0.33072741	0.42557999	0.960599166
544678	'Cfap74'	58.42	58.95	45.01	6	24	6	54.12666667	12	-2.148946556	0.001698567	0.088961867
544696	'Tbc1d32'	638	557	151	113	595	499	448.6666667	402.3333333	-0.166538164	0.799848715	0.972790461

544717	'1190007I07Rik'	110	116	81	14	92	148	102.3333333	84.66666667	-0.371594433	0.588677667	0.972790461
54473	'Tollip'	2005	2063	3166	1332	1655	2051	2411.333333	1679.333333	-0.524606495	0.203861081	0.832079769
544736	'Glipr1l3'	2	3	0	0	0	1	1.666666667	0.333333333	-2.058912803	0.492510925	0.972790461
544763	'Hbq1b'	2	0	2	0	11	3	1.333333333	4.666666667	1.64132146	0.410042327	0.957157474
544791	'Myh13'	25.03	23	0	3	15	16	16.01	11.33333333	-0.469321441	0.718525386	0.972790461
544806	'Tmem92'	0	0	0	1	0	0	0	0.333333333	1.020273531	0.802557913	0.972790461
544817	'Arhgap27'	154	173	101	24	222	134	142.6666667	126.6666667	-0.239420281	0.72016969	0.972790461
54483	'Mefv'	0	0	0	2	2	2	0	2	3.527067868	0.192081385	0.813837363
54484	'Mkrn1'	1479	1449	2537	1471	1694	2111	1821.666667	1758.666667	-0.052282806	0.907951895	0.988823459
544848	'Gm5784'	382.11	343.59	274.29	127.32	339.03	275.96	333.33	247.4366667	-0.437143432	0.221786291	0.849517424
54485	'Dll4'	435	358	46	8	749	382	279.6666667	379.6666667	0.393002829	0.731160886	0.972790461
54486	'Hpgds'	20	14	2	34	24	39	12	32.33333333	1.597895919	0.053112168	0.532859388
544864	'Gm5785'	120.66	109.22	31.1	86.26	90.35	91.94	86.99333333	89.51666667	0.183201205	0.744891927	0.972790461
544881	'BB287469'	0	1.79	3.63	0	0	1	1.806666667	0.333333333	-1.964920319	0.529831773	0.972790461
544922	'Zkscan4'	117.12	86.19	40.18	27	144	113	81.16333333	94.66666667	0.200829828	0.748429094	0.972790461
544923	'Serpinb9h'	1	1	0	1	0	0	0.666666667	0.333333333	-0.746871675	0.831393929	0.974324192
544963	'Iqgap2'	177	180	106	606	160	260	154.3333333	342	1.429663313	0.04247552	0.479588013
544971	'Bdp1'	1578	1640	1138	1523	1381	1397	1452	1433.666667	0.103892675	0.776211122	0.972790461
544990	'Gm5795'	0	0	1.4	0	0	1	0.466666667	0.333333333	0.058500858	0.988561293	0.999493374
544998	'Gm16440'	1.87	1.89	2	0	0	0	1.92	0	-2.856115697	0.367295997	0.937190492
545007	'Gm5796'	16.07	12.5	13.7	0	7.24	8.48	14.09	5.24	-1.55534993	0.137229077	0.738626463
545030	'Wdfy4'	290	263	10	9	126	153	187.6666667	96	-0.995500626	0.364885019	0.935688425
545085	'Wdr70'	781	812	783	766	686	1004	792	818.6666667	0.113220085	0.746218152	0.972790461
545091	'Hnrnpa1l2-ps2'	0	0	2.01	0	0	0	0.67	0	-1.995285526	0.619817042	0.972790461
545123	'Cyp2d11'	0	0	0	2	0	0	0	0.666666667	2.299096387	0.566358544	0.972790461
545124	'Tdg-ps'	244.65	288.92	567.4	30.06	97.08	29.51	366.99	52.21666667	-2.877068051	1.88E-05	0.004028556
545126	'CN725425'	415	385	107	0	204	132	302.3333333	112	-1.511676275	0.232628276	0.859679981
545136	'Fam186b'	599	594	97	234	613	224	430	357	-0.161690074	0.819158842	0.972790461
545156	'Kalrn'	1656.5	1653.3	903.47	451.2	1278.9	1144.58	1404.416667	958.2366667	-0.550732257	0.161840371	0.771541907
54519	'Apbb1ip'	40	34	121	50	50	41	65	47	-0.464456997	0.528551819	0.972790461
545192	'Baiap3'	130	100	26	72	61	98	85.33333333	77	-0.008913251	0.988628924	0.999493374
545205	'Olfr111'	0	1	0	0	8	2	0.333333333	3.333333333	3.100823138	0.24376666	0.865593324
54524	'Syt6'	42	46	20	13	27	8	36	16	-1.073320703	0.12077388	0.712100232
54525	'Syt7'	898	865	822	212	541	340	861.6666667	364.3333333	-1.244405277	6.97E-04	0.052467355
545253	'Gm5820'	8	6	7	8	15	15	7	12.66666667	0.863455603	0.246842314	0.868602029
54526	'Syt10'	3	4	0	0	0	0	2.333333333	0	-3.500233719	0.323881481	0.919383393
545260	'Arsi'	127	141	49	92	166	141	105.6666667	133	0.406136262	0.377152201	0.941504733
545276	'Gal3st3'	58	49	19	9.12	61	60	42	43.37333333	0.006561598	0.992812998	0.999493374
545291	'Hpse2'	113	103	23	254	55	55	79.66666667	121.3333333	1.000050983	0.278991286	0.889355605
545366	'Cfhr2'	5.36	18.77	4.69	0	17.97	1	9.606666667	6.323333333	-0.629944883	0.687304926	0.972790461

545370	'Hmcn1'	2698	2525	445	245	2891	1478	1889.333333	1538	-0.317192557	0.702662798	0.972790461
545384	'Ifi214'	0	1	0	0	0	0	0.333333333	0	-0.903279821	0.824807108	0.972790461
545389	'Cep170'	2408.8	2217.7	4539.4	3846.3	1975.2	2351	3055.326667	2724.19	-0.04720819	0.939920768	0.995607401
545391	'Catspere2'	389.95	340	111	279	240	241	280.3166667	253.3333333	0.030145358	0.955573043	0.999493374
545428	'Ccdc141'	513	483	394	170	623	326	463.3333333	373	-0.331478171	0.466511967	0.972790461
545471	'Zfp345'	54.42	56.33	16.97	12	59.41	70	42.57333333	47.13666667	0.140284453	0.844123184	0.975182082
545474	'Scrt2'	81	96	8	0	56	96	61.66666667	50.66666667	-0.351187465	0.793497776	0.972790461
545477	'Bpifa6'	0	0	1	0	1	0	0.333333333	0.333333333	0.058500858	0.988561293	0.999493374
545481	'Arhgap40'	2	3	1	3	0	3	2	2	0.188319264	0.913400879	0.989773583
545486	'Tubb1'	1	1	0	0	1	0	0.666666667	0.333333333	-0.74517548	0.838722495	0.974723675
545490	'Zfp973'	91.26	26.42	18.15	65.31	29.06	98.91	45.27666667	64.42666667	0.645055457	0.413603479	0.95722888
545527	'Erich6'	9	7	1	0	11	1	5.666666667	4	-0.52020039	0.753565575	0.972790461
545531	'Gm5848'	0	1	0	0	0	1	0.333333333	0.333333333	0.058500858	0.988561293	0.999493374
545554	'Ankrd34a'	44	49	62	121	36	53	51.66666667	70	0.64860281	0.381489947	0.94429001
54561	'Nap1l3'	124	124	93	38	186	148	113.6666667	124	0.071179848	0.892714465	0.985171
545611	'Fam205a2'	140.56	98.78	31.87	26.07	117.55	45.08	90.40333333	62.9	-0.482237036	0.495161027	0.972790461
54562	'Lrrc6'	40	41	15	0	31	24	32	18.33333333	-0.88784058	0.427691184	0.961947592
545622	'Ptpn3'	347	340	358	597	392	403	348.3333333	464	0.556455208	0.252510432	0.871252558
54563	'Nup210'	1062	988	253	21	862	718	767.6666667	533.6666667	-0.596021756	0.541985074	0.972790461
545645	'Gm13283'	0	0	0	0	2	1	0	1	2.320886	0.562538074	0.972790461
545646	'Gm13290'	1.31	0	0	1.46	0	0	0.436666667	0.486666667	0.058500858	0.988561293	0.999493374
545647	'Gm13289'	1.31	0	0	1.46	0	0	0.436666667	0.486666667	0.058500858	0.988561293	0.999493374
545649	'Gm13276'	3.76	6.17	7.54	0	4.93	0	5.823333333	1.643333333	-2.098691411	0.235121373	0.861636582
545650	'Gm13277'	0	0	0	1.43	0	3.64	0	1.69	2.867003166	0.470908566	0.972790461
545651	'Gm13278'	11.09	11.5	18.56	5.16	2.16	9.04	13.71666667	5.453333333	-1.31166799	0.15723695	0.768337387
545652	'Gm13275'	13.51	20.1	13.93	3.37	12.62	14.67	15.84666667	10.22	-0.702051874	0.35532774	0.931809687
545667	'Shisal2a'	3	7	52	4	4	4	20.66666667	4	-2.457040364	0.050308239	0.518969204
545693	'Gm13043'	0	0	0	0	1	0	0	0.333333333	1.020273531	0.802557913	0.972790461
545725	'Mterf1a'	63.56	79.22	100.68	52.76	90.16	51.88	81.15333333	64.93333333	-0.306669134	0.542817756	0.972790461
545732	'4933402N22Rik'	1	0	0	0	0	0	0.333333333	0	-0.903279821	0.824807108	0.972790461
545739	'Gm5862'	0.68	1.44	0	29.94	2.28	9.09	0.706666667	13.77	5.538249591	0.002843411	0.119599414
545798	'Tmem233'	0	0	0	0	1	0	0	0.333333333	1.020273531	0.802557913	0.972790461
545812	'Pilrb2'	0	1	0	1	1	1	0.333333333	1	1.558404368	0.565964169	0.972790461
545824	'Zar1l'	0	0	0	0	1	0	0	0.333333333	1.020273531	0.802557913	0.972790461
545884	'Gm5885'	0	0	0	0	0	1	0	0.333333333	1.020273531	0.802557913	0.972790461
545893	'Mansc4'	23	23	0	1	18	19	15.33333333	12.66666667	-0.293727885	0.837609716	0.974723675
545902	'Ptprh'	4	3	1	1	1	0	2.666666667	0.666666667	-1.819705149	0.338457574	0.924608313
545913	'Zscan4d'	0	1	0	0	0	0	0.333333333	0	-0.903279821	0.824807108	0.972790461
545938	'Zfp607a'	167.36	171.12	124.34	22	196.4	154.35	154.2733333	124.25	-0.398931664	0.543397767	0.972790461
545975	'Cers3'	159	162	41	1	54	80	120.6666667	45	-1.504619991	0.145831123	0.753611197

54598	'Calcrl'	566	558	913	707	804	652	679	721	0.128655597	0.783333255	0.972790461
54601	'Foxo4'	828	842	579	1142	861	798	749.6666667	933.6666667	0.476609228	0.283746733	0.892170588
546024	'Crxos'	2	1	1	9	1	4	1.333333333	4.666666667	2.06843182	0.175935103	0.795452599
54604	'Pcnx'	2114	1876	1069	1991	2630	2612	1686.333333	2411	0.601099216	0.063510075	0.567415027
546049	'Rps23rg1'	16.38	13.71	6.3	0	7.14	2.02	12.13	3.053333333	-1.991070429	0.100633088	0.671759705
54607	'Socs6'	1654	1736	1435	790	1313	1507	1608.333333	1203.333333	-0.404449459	0.101556463	0.671759705
546071	'Mast3'	734	694	700	611	789	683	709.3333333	694.3333333	0.027515101	0.929906977	0.993240809
54608	'Abhd2'	818	889	984	5360	1090	1374	897	2608	1.834332414	0.021663539	0.364161986
54609	'Ubqln2'	1231	1211	1789	818	1178	1213	1410.333333	1069.666667	-0.40096697	0.301554639	0.90469437
54610	'Tbc1d8'	339.14	323.16	161.07	546.13	456.17	476.14	274.4566667	492.8133333	0.998366112	0.024285632	0.385407711
54611	'Pde3a'	306	297	167	135	305	287	256.6666667	242.3333333	-0.061577153	0.864594363	0.977908741
546118	'Ubtfl1'	2	1	0	8	0	0	1	2.666666667	1.965661896	0.469841944	0.972790461
54612	'Sfrp5'	2	2	0	0	1	2	1.333333333	1	-0.434829477	0.853935196	0.975734242
54613	'St3gal6'	132	127	183	1506	346	648	147.3333333	833.3333333	2.725910798	2.42E-04	0.025854303
546134	'Gramd2'	85	62	14	5	39	46	53.66666667	30	-0.866996777	0.312453051	0.911537035
54614	'Prpf40b'	1284	1239	724	1093	1380	975	1082.333333	1149.333333	0.200662212	0.595502137	0.972790461
546143	'Ccpg1os'	107	96	76	39	110	112	93	87	-0.116701233	0.782067681	0.972790461
546144	'Wdr72'	91	79	15	31	57	55	61.66666667	47.66666667	-0.283095616	0.672267963	0.972790461
54615	'Npff'	7	3	2	2	4	1	4	2.333333333	-0.680545384	0.609041817	0.972790461
546157	'7420426K07Rik'	0	5	1	0	1	1	2	0.666666667	-1.59377775	0.512613801	0.972790461
54616	'Extl3'	1435	1416	461	1389	2299	2462	1104	2050	0.968346103	0.032787617	0.436246529
546160	'Fbxw25'	0	0	0	0	2	0.76	0	0.92	1.801491674	0.656112935	0.972790461
546161	'Fbxw18'	0	0	2	0	6	17	0.666666667	7.666666667	3.208776256	0.161406973	0.771435531
54624	'Paf1'	1074.9	1079	1153.8	1786.9	1217.5	1235.29	1102.596667	1413.25	0.495159598	0.294680905	0.90029104
546250	'Gm5930'	0	0	0	0	0	4.99	0	1.663333333	2.683352581	0.500796233	0.972790461
546268	'Gm5932'	0	0	0	0	1	0	0	0.333333333	1.020273531	0.802557913	0.972790461
546294	'Rhox3g'	1.27	0	0	0	0	0	0.423333333	0	-0.903279821	0.824807108	0.972790461
54630	'Prickle3'	150.39	192.51	130.5	180.11	257.15	163.18	157.8	200.1466667	0.430254692	0.271933127	0.884539076
54631	'Nphs1'	6	1	3	0	4	0	3.333333333	1.333333333	-1.394822517	0.49767479	0.972790461
54632	'Ftsj1'	255	295	315	235	329	448	288.3333333	337.3333333	0.226226567	0.511206792	0.972790461
546325	'Gm5936'	3.95	5.78	0	0	0	0	3.243333333	0	-3.691647008	0.280270411	0.889355605
54633	'Pqbp1'	430	480	886	398	503	671	598.6666667	524	-0.22180218	0.652810887	0.972790461
546336	'Prrg1'	313	323	492	317	438	403	376	386	0.04644308	0.9123976	0.989773583
54634	'Magix'	12	16	0	4	11	4	9.333333333	6.333333333	-0.436909025	0.744011524	0.972790461
54635	'Pdgfc'	603	550	600	253	506	742	584.3333333	500.3333333	-0.265177898	0.486307723	0.972790461
54636	'Wdr45'	450	454	1004	355	417	503	636	425	-0.603370505	0.259001835	0.877743829
54637	'Praf2'	297	305	431	342	380	480	344.3333333	400.6666667	0.237282325	0.558993347	0.972790461
54638	'Ccdc22'	280.65	282.77	271.8	411	352.93	308.97	278.4066667	357.6333333	0.482529659	0.260551693	0.878537553
54644	'Otud5'	3180	2964	3065	1401	2542	2976	3069.666667	2306.333333	-0.423414941	0.148800963	0.757633614
54645	'Gripap1'	453	507	458	982	566	477	472.6666667	675	0.70227608	0.203656073	0.832079769

54646	'Ppp1r3f'	391	366	82	554	300	160	279.6666667	338	0.581797843	0.451794517	0.970649024
54648	'Ccdc120'	235	265	255	168	223	210	251.6666667	200.3333333	-0.28446578	0.377574526	0.941536807
54650	'Sfmbt1'	1544	1550	799	233	1061	1159	1297.666667	817.6666667	-0.711912732	0.183089463	0.804092742
54651	'Usp27x'	408	434	341	81	450	383	394.3333333	304.6666667	-0.446110721	0.409428424	0.957157474
546519	'Tmem235'	2	2	3	0	1	2	2.333333333	1	-1.329814424	0.457099224	0.972077231
54652	'Cacna1f'	2	2	1	1	3	0	1.666666667	1.333333333	-0.239879945	0.899313206	0.986106073
546611	'Klhl33'	18	18	6	0	14	30	14	14.66666667	-0.031026344	0.979779089	0.999493374
546643	'Ly6a2'	0	1	0	0	6	0	0.333333333	2	2.373750729	0.532788375	0.972790461
546644	'Ly6g'	0	0	0	0	5	0	0	1.666666667	3.073512668	0.438501572	0.966976432
546648	'Klhdc7b'	6	5	0	1	5	1	3.666666667	2.333333333	-0.568148206	0.735782303	0.972790461
54667	'Atp8b2'	1858	1759	2061	3427	2376	3065	1892.666667	2956	0.749784407	0.090144151	0.648437606
54670	'Atp8b1'	49	31	52	147	51	96	44	98	1.319601306	0.05387205	0.534031734
54672	'Adgrg3'	15	14	17	5	39	7	15.33333333	17	0.086004995	0.925797818	0.992381554
546726	'Cyp26c1'	0	0	1	1	1	1	0.333333333	1	1.557984566	0.561051704	0.972790461
54673	'Sh3glb1'	2444	2333	5741	3126	2284	2970	3506	2793.333333	-0.291789507	0.626258597	0.972790461
546752	'Gm5977'	283.96	230.53	140.42	89.17	230.82	138.5	218.3033333	152.83	-0.480750039	0.263538389	0.88013169
54678	'Zfp108'	158	135.99	45	79	160	143	112.9966667	127.3333333	0.240942074	0.636744578	0.972790461
546801	'Etv3l'	1	1	2.01	0	2	0	1.336666667	0.666666667	-1.088957393	0.659967391	0.972790461
54683	'Prdx5'	1166.4	1190.6	2966.1	1254.5	979.55	1507.74	1774.363333	1247.25	-0.504125763	0.400988478	0.955860942
546840	'Ldlrad1'	2	3.98	1	5	4	1	2.326666667	3.333333333	0.945148533	0.509425379	0.972790461
546849	'AAdacl4fm3'	9	11	12	0	0	0	10.66666667	0	-5.819030998	5.37E-04	0.045614454
546886	'Cfap73'	0	0	0	0	1	1	0	0.666666667	1.775692139	0.660844521	0.972790461
546896	'Olfr455'	0	0	0	3	1	0	0	1.333333333	3.197118853	0.416786276	0.95722888
546901	'Vmn1r34'	1	2	0	0	3	1	1	1.333333333	0.405502065	0.866582138	0.978294615
546912	'Vmn2r21'	1	0	0	0	0	0	0.333333333	0	-0.903279821	0.824807108	0.972790461
546913	'Vmn2r22'	1	0	0	0	0	0	0.333333333	0	-0.903279821	0.824807108	0.972790461
546964	'Vmn2r62'	1	0	0	0	0	0	0.333333333	0	-0.903279821	0.824807108	0.972790461
54698	'Crtam'	4	2	0	1	0	1	2	0.666666667	-1.363247368	0.561742048	0.972790461
546983	'Vmn2r77'	0	0	0	0	0	1	0	0.333333333	1.020273531	0.802557913	0.972790461
54709	'Eif3i'	1543	1594	2328	1697	1398	1957	1821.666667	1684	-0.063382551	0.888012277	0.984534762
547097	'B020011L13Rik'	6	5	18	11	6	16	9.666666667	11	0.171695258	0.858974093	0.976840259
54710	'Hs3st3b1'	732.84	747.27	1035.9	456.75	545.04	747.45	838.6833333	583.08	-0.521726394	0.18194084	0.802193703
547109	'Trim43a'	10	9	6	25	1	5	8.333333333	10.33333333	0.688003462	0.58702954	0.972790461
54711	'Plagl2'	1408	1470	1394	1017	1264	1333	1424	1204.666667	-0.192177942	0.492767472	0.972790461
54712	'Plxnc1'	1551	1504	188	200	2639	2168	1081	1669	0.595575322	0.505911247	0.972790461
54713	'Fezf2'	1	4	0	1	2	3	1.666666667	2	0.332015859	0.855239527	0.975734242
547154	'H2al1k'	0	0	0	0	0	1	0	0.333333333	1.020273531	0.802557913	0.972790461
547160	'H2al1e'	0	0	1	0	0	0	0.333333333	0	-0.903279821	0.824807108	0.972790461
547168	'Rhox7a'	33	31	8	1	9	20	24	10	-1.31006539	0.183822697	0.804729813
547176	'Zc3h12b'	438.36	441	175.66	98	281	320.7	351.6733333	233.2333333	-0.589088709	0.227756033	0.854324922

54720	'Rcan1'	541	526	1343	6679	454	1080	803.3333333	2737.666667	2.071352909	0.049379187	0.515210762
54721	'Tyk2'	569	611	448	448	717	763	542.6666667	642.6666667	0.276440437	0.256300977	0.876684814
54722	'Gsdme'	212	237	351	82	194	175	266.6666667	150.3333333	-0.881814661	0.055009911	0.538541927
54723	'Tfip11'	1631	1605.7	1014.1	987.49	1250.8	1695.89	1416.906667	1311.403333	-0.056876757	0.844433996	0.975182082
54725	'Cadm1'	752	630	202	755	547	779	528	693.6666667	0.565010439	0.303770039	0.906636731
547253	'Parp14'	162	158	120	33	237	309	146.6666667	193	0.296009795	0.653935207	0.972790461
547347	'Gm6034'	4	6.25	3	2	0	2	4.416666667	1.333333333	-1.555315042	0.302788378	0.905839913
547431	'Btnl2'	1	1	0	0	0	0	0.666666667	0	-1.703500596	0.673820112	0.972790461
550619	'Arid3c'	3	3	0	3	2	2	2	2.333333333	0.428811465	0.792540691	0.972790461
552899	'Ugt2a2'	1	1	0	0	0	0	0.666666667	0	-1.703500596	0.673820112	0.972790461
553127	'Rtl8b'	333.46	320.51	311.3	158.25	426.1	379.54	321.7566667	321.2966667	-0.030908062	0.932853604	0.993970337
554292	'Methig1'	1	0	0	0	0	0	0.333333333	0	-0.903279821	0.824807108	0.972790461
554327	'2610042L04Rik'	51.49	63.61	39.9	4	33.05	18.64	51.66666667	18.56333333	-1.522552289	0.027356711	0.404817338
55925	'Syt8'	1	1	0	1	1	0	0.666666667	0.666666667	0.24523346	0.929078356	0.993096978
55927	'Hes6'	302	292	425	94	226	334	339.6666667	218	-0.723729951	0.136091569	0.736689633
55932	'Gbp3'	98	95	448	28	47	193	213.6666667	89.33333333	-1.47810499	0.104093585	0.676400911
55934	'Rp9'	297	326	861	265	521	693	494.6666667	493	-0.116893615	0.849575024	0.975734242
55935	'Fnbp4'	1501	1497	1194	1989	1704	1093	1397.333333	1595.333333	0.350463384	0.463634051	0.972790461
55936	'Ctps2'	1686	1743	1055	754	1832	1734	1494.666667	1440	-0.047816666	0.887883361	0.984534762
55938	'Apom'	1	2	2	3	2	1	1.666666667	2	0.400346233	0.797281772	0.972790461
55942	'Sertad1'	226	191	813	288	256	262	410	268.6666667	-0.619641782	0.397197148	0.954437838
55943	'Stx8'	284	334	476	189	306	441	364.6666667	312	-0.272048202	0.539723433	0.972790461
55944	'Eif3d'	2627	2584	4525	2619	2458	3238	3245.333333	2771.666667	-0.204618008	0.665111497	0.972790461
55946	'Ap3m1'	1725	1751	1388	826	1599	1738	1621.333333	1387.666667	-0.220443966	0.411631322	0.95722888
55947	'Dclre1a'	513	514	243	132	430	393	423.3333333	318.3333333	-0.415284049	0.365486677	0.936221059
55948	'Sfn'	13	19	79	57	25	73	37	51.66666667	0.456841349	0.617373818	0.972790461
55949	'Eef1b2'	6394	6674	11419	7583	5760	7155	8162.333333	6832.666667	-0.195516875	0.701960779	0.972790461
55950	'Bri3'	498	513	1296	508	448	790	769	582	-0.430991082	0.473658219	0.972790461
55951	'Mpc1'	438	481	1195	672	525	763	704.6666667	653.3333333	-0.100900647	0.869332509	0.979633196
55960	'Ebag9'	557	526	638	775	489	642	573.6666667	635.3333333	0.263895395	0.580350419	0.972790461
55961	'Slc13a1'	0	1	0	0	0	0	0.333333333	0	-0.903279821	0.824807108	0.972790461
55963	'Slc1a4'	616	468	492	417	511	797	525.3333333	575	0.150928861	0.650181085	0.972790461
55978	'Ift20'	405	419	197	295	405	409	340.3333333	369.6666667	0.203591046	0.569629059	0.972790461
55979	'Agpat1'	1069.8	1001.8	1025.8	836.74	1013.9	1400.97	1032.473333	1083.883333	0.097174861	0.743510816	0.972790461
55980	'Impa1'	710	839	750	1609	1045	1036	766.3333333	1230	0.837421504	0.085219552	0.634259952
55981	'Pigb'	312.95	325.96	126.35	349.75	375.36	315.12	255.0866667	346.7433333	0.589563733	0.200854017	0.828371216
55982	'Paxip1'	999	993	517.33	730	1062	957	836.4433333	916.3333333	0.213360541	0.520244299	0.972790461
55983	'Pdzrn3'	409	360	466	2931	332	469	411.6666667	1244	1.943152936	0.038576156	0.460411379
55984	'Camkk1'	104	85	38	54	43	24	75.66666667	40.33333333	-0.700117206	0.277607512	0.889355605
55985	'Cxcl13'	1	2	0	2	4	4	1	3.333333333	1.805560637	0.274355242	0.885667571

55987	'Cpxm2'	17	14	5	2	14	8	12	8	-0.587260049	0.528743587	0.972790461
55988	'Snx12'	1172	1336	1225	647.01	945	1094	1244.333333	895.3366667	-0.455591412	0.091791351	0.651188901
55989	'Nop58'	2316	2264	3166	1150	1859	2436	2582	1815	-0.540513269	0.158148097	0.768890221
55990	'Fmo2'	12	7	10	15	4	2	9.666666667	7	-0.184738041	0.869590447	0.979712805
55991	'Panx1'	269	218	208	64	304	329	231.6666667	232.3333333	-0.075075028	0.888868058	0.984792893
55992	'Trim3'	533	533	867	750	534	676	644.3333333	653.3333333	0.094994208	0.852477547	0.975734242
55993	'Msh4'	578	544	84	0	237	181	402	139.3333333	-1.593479587	0.230810925	0.857634106
55994	'Smad9'	105	111	50	10	102	106	88.66666667	72.66666667	-0.358794475	0.628792462	0.972790461
56009	'Alyref2'	122	123	133	44	111	163	126	106	-0.31092378	0.504763612	0.972790461
56012	'Pgam2'	5	8	11	25	12	9	8	15.33333333	1.109193688	0.230086649	0.856721174
56013	'Srcin1'	99	115	11	49	80	52	75	60.33333333	-0.170313762	0.826835814	0.972880461
56014	'Olfr70'	1	0	0	0	0	0	0.333333333	0	-0.903279821	0.824807108	0.972790461
56016	'Hebp2'	129	169	201	117	228	271	166.3333333	205.3333333	0.263826908	0.530168769	0.972790461
56017	'Slc2a8'	182	144	106	592	214	329	144	378.3333333	1.625692919	0.00892338	0.231288612
56018	'Stard10'	572	518	546	1394	455	951	545.3333333	933.3333333	0.961146045	0.11398929	0.698948431
56030	'Tmem131'	2579	2352	1304	3619	3480	3271	2078.333333	3456.666667	0.871868403	0.028512822	0.410671038
56031	'Ppie'	503	552	630	367	479	600	561.6666667	482	-0.208780672	0.535915289	0.972790461
56032	'Nprl2'	237	229	226	370	220	363	230.6666667	317.6666667	0.578184235	0.212596428	0.841028891
56036	'Ccnl2'	3355	3200	2475	3792	3839	1983	3010	3204.666667	0.241647076	0.618054589	0.972790461
56040	'Rplp1'	5521	5498	9744	4926	4489	5669	6921	5028	-0.430979929	0.371562629	0.938527219
56041	'Uso1'	2093	2018	5727	1951	1797	2937	3279.333333	2228.333333	-0.591947618	0.33601016	0.922539021
56043	'Akr1e1'	236.03	301.48	217	184	498	468	251.5033333	383.3333333	0.588000288	0.115597788	0.702825844
56044	'Rala'	1488	1564	3071	1759	1452	1623	2041	1611.333333	-0.294884935	0.586474386	0.972790461
56045	'Samhd1'	1565	1645	643	751	1528	1687	1284.333333	1322	0.08057478	0.846146614	0.975182082
56046	'Uqcc1'	508	457	485	306	591	627	483.3333333	508	0.061618263	0.841012059	0.975182082
56047	'Msln'	34	27	73	310	127	256	44.66666667	231	2.456357192	5.21E-04	0.044654124
56048	'Lgals8'	662	689	1893	815	915	1065	1081.333333	931.6666667	-0.245933699	0.684163733	0.972790461
56050	'Cyp39a1'	193	187	101	247	194	310	160.3333333	250.3333333	0.758074682	0.074588777	0.603900623
56055	'Gtpbp2'	1209	1176	3996	1691	1278	1207	2127	1392	-0.577415288	0.412249417	0.95722888
56057	'Btg4'	4	4	1	2	6	22	3	10	1.685239211	0.166421708	0.780893009
56068	'Ammecr1'	763	809	749	211	604	650	773.6666667	488.3333333	-0.711721024	0.070022236	0.589465353
56070	'Tcerg1'	2070	2001	2329	1841	1951	2177	2133.333333	1989.666667	-0.046234419	0.893718126	0.985171
56072	'Lgals12'	20	19	4	8	35	43	14.33333333	28.66666667	0.990911438	0.227973277	0.85440173
56075	'Pdss1'	178	183	97	54	148	128	152.6666667	110	-0.462278008	0.271167398	0.884312405
56077	'Dgke'	441	421.11	141	601	338	370	334.37	436.3333333	0.616905442	0.305946936	0.907188578
56078	'Car5b'	32	46	28	118	39	71	35.33333333	76	1.322694227	0.053265411	0.533562632
56079	'Astn2'	45	37	12	20	42	17	31.33333333	26.33333333	-0.148132247	0.831098038	0.974324192
56085	'Ubqln1'	2450	2457	3039	1932	2471	3181	2648.666667	2528	-0.058579907	0.863299775	0.97731662
56086	'Set'	5693	5727	8449	2867	5993	6973	6623	5277.666667	-0.385902672	0.355603578	0.931809687
56087	'Dnah10'	77	81	12	7	64	32	56.66666667	34.33333333	-0.722635005	0.413082172	0.95722888

56088	'Psmg1'	712	742	544	303	530	820	666	551	-0.280216768	0.414312436	0.95722888
56089	'Ramp3'	45	52	9	112	44	42	35.33333333	66	1.214803542	0.148366261	0.756674202
56093	'Pfpl'	1	0	0	0	0	0	0.333333333	0	-0.903279821	0.824807108	0.972790461
56095	'Ftsj3'	1273	1313	1133	1072	1335	1449	1239.666667	1285.333333	0.106023494	0.683519763	0.972790461
56096	'Plac1'	12	14	1	12	13	4	9	9.666666667	0.329115211	0.767082267	0.972790461
56149	'Grasp'	255	259	276	407	280	237	263.3333333	308	0.375235508	0.46376075	0.972790461
56150	'Mad2l1'	896	1002	473	177	564	749	790.3333333	496.6666667	-0.697630521	0.154032499	0.765411885
56174	'Nagk'	468	582	458	154	513	640	502.6666667	435.6666667	-0.269394217	0.559579023	0.972790461
56175	'Bace2'	594	583	264	316	737	854	480.3333333	635.6666667	0.418935493	0.317939872	0.916136994
56176	'Pigp'	944.22	959.04	635.03	92	624.83	473	846.0966667	396.61	-1.162134025	0.045345465	0.496605985
56177	'Olfm1'	844	864	303	3376	1227	2020	670.3333333	2207.666667	1.979966568	0.002311154	0.108371515
56183	'Nmu'	0	0	0	0	0	1	0	0.333333333	1.020273531	0.802557913	0.972790461
56185	'Hao2'	52	71	14	25	190	215	45.66666667	143.3333333	1.612213954	0.041501935	0.47647028
56187	'Rabggta'	274	279	411	345	294	391	321.3333333	343.3333333	0.145002377	0.750537437	0.972790461
56188	'Fxyd1'	160	186	137	82	155	245	161	160.6666667	-0.020838117	0.957824102	0.999493374
56189	'Prodh2'	1	0	0	0	0	0	0.333333333	0	-0.903279821	0.824807108	0.972790461
56190	'Rbm38'	500	454	360	397	379	575	438	450.3333333	0.110671096	0.738677002	0.972790461
56191	'Tro'	1190	1140	1024	1046	1177	779	1118	1000.666667	-0.052883061	0.894510001	0.985259027
56193	'Plek'	154	157	148	53	184	274	153	170.3333333	0.073755937	0.887840597	0.984534762
56194	'Prpf40a'	2741	2855	1918	1996	2822	2870	2504.666667	2562.666667	0.09210151	0.708686645	0.972790461
56195	'Ptbp2'	3182	3043	1423	371	2163	2786	2549.333333	1773.333333	-0.585543908	0.348104981	0.928444937
56196	'Tdp2'	1343	1335	1374	822	1026	1265	1350.666667	1037.666667	-0.349692239	0.226287587	0.853679947
56198	'Hey1'	238	250	48	92	351	327	178.6666667	256.6666667	0.539828936	0.415741457	0.95722888
56199	'Abcb10'	706	676	154	130	666	498	512	431.3333333	-0.244934284	0.714167958	0.972790461
56200	'Ddx21'	2298	2236	1909	2536	2223	2573	2147.666667	2444	0.289536272	0.41081583	0.95722888
56205	'Ensa'	1971	1964	2052	2289	1846	2023	1995.666667	2052.666667	0.145784435	0.718674238	0.972790461
56207	'Uchl5'	537	589	701	453	429	607	609	496.3333333	-0.253051726	0.514707308	0.972790461
56208	'Becn1'	1924.6	2019.2	1457.5	1456.2	1778.9	2005.3	1800.436667	1746.81	0.021825969	0.93233188	0.993860034
56209	'Gde1'	601	558	935	353	691	858	698	634	-0.200993131	0.654071488	0.972790461
56210	'Rev1'	1462.9	1412.7	890.4	472.57	1241.3	1161.82	1255.32	958.5566667	-0.39110358	0.275873057	0.887417193
56212	'Rhog'	477	463	1250	591	515	661	730	589	-0.307940636	0.613705505	0.972790461
56213	'Htra1'	730	732	531	6200	997	1915	664.3333333	3037.333333	2.517090214	0.001682894	0.088782789
56214	'Scamp4'	736	765	720	534	788	848	740.3333333	723.3333333	-0.008400931	0.974253294	0.999493374
56215	'Acin1'	4386.8	4352.3	2700	2514.2	4928.9	3595.34	3813.03	3679.493333	-0.004058912	0.989683943	0.999493374
56216	'Stx1b'	302	243	40	50	235	83	195	122.6666667	-0.618223984	0.429831846	0.962548446
56217	'Mpp5'	1633	1641	2398	2663	1441	1661	1890.666667	1921.666667	0.160603313	0.775071948	0.972790461
56218	'Patz1'	956	875	892	430	1031	1017	907.6666667	826	-0.163365494	0.628481206	0.972790461
56219	'Extl1'	53	26	44	188	69	163	41	140	1.913269144	0.003762946	0.140640662
56220	'Zfp386'	566	622	794	224	603	444	660.6666667	423.6666667	-0.686394789	0.106448149	0.679124905
56221	'Ccl24'	0	1	1	2	0	0	0.666666667	0.666666667	0.369435326	0.909857284	0.989364653

56222	'Cited4'	232	289	439	285	432	468	320	395	0.280727766	0.530809204	0.972790461
56223	'Fscn3'	1	3	0	0	0	0	1.333333333	0	-2.692051488	0.498596374	0.972790461
56224	'Tspan5'	1161	1089	1635	1645	1411	1818	1295	1624.666667	0.380713692	0.388381341	0.950065114
56226	'Espn'	1099	1094	1259	473	2209	1863	1150.666667	1515	0.306453725	0.549529901	0.972790461
56228	'Ube2j1'	1019	1091	2821	857	1280	1432	1643.666667	1189.666667	-0.52965657	0.362285603	0.934315503
56229	'Thsd1'	270	303	55	36	463	163	209.3333333	220.6666667	0.060172916	0.944115702	0.996592403
56233	'Hdac7'	974	951	711	908	1131	891	878.6666667	976.6666667	0.243908333	0.453740449	0.970649024
56248	'Ak3'	1170.1	1145.2	1221.1	714.04	1587.2	1614.21	1178.79	1305.14	0.119347368	0.71885112	0.972790461
56249	'Actr8'	910	871	580	460	663	829	787	650.6666667	-0.231982321	0.398663347	0.954582816
56258	'Hnrnph2'	1841	1950	1560	1331	1548	1756	1783.666667	1545	-0.142897092	0.593255303	0.972790461
56264	'Cpxm1'	1055	1077	894	2163	1838	1811	1008.666667	1937.333333	1.064533258	0.006237088	0.186739406
56273	'Pex14'	723	800	707	673	862	1079	743.3333333	871.3333333	0.262981841	0.333273763	0.921648675
56274	'Stk3'	815.57	843.48	1130.2	453.6	1204	1248.09	929.7633333	968.5633333	-0.008420814	0.984391081	0.999493374
56275	'Rbm14'	1029.8	1083.2	1061	565.86	901.67	799.55	1057.993333	755.6933333	-0.459532449	0.103438726	0.674448379
56277	'Tmem45a'	355	330	1456	1230	286	531	713.6666667	682.3333333	0.072426832	0.936520333	0.994413066
56278	'Gkap1'	1562	1441	916	231	1174	1860	1306.333333	1088.333333	-0.344806112	0.571215535	0.972790461
56279	'Dipk1b'	236	226	156	79	321	310	206	236.6666667	0.154366955	0.749955288	0.972790461
56280	'Mrpl37'	677	719	533	337	624	772	643	577.6666667	-0.15128383	0.60536762	0.972790461
56282	'Mrpl12'	602	644	534	604	654	954	593.3333333	737.3333333	0.36200683	0.228097731	0.854501292
56284	'Mrpl19'	518.15	590.44	441.8	315.97	567.8	563.28	516.7966667	482.35	-0.07977719	0.761051409	0.972790461
56289	'Rassf1'	563	616	3002	554	531	623	1393.666667	569.3333333	-1.343237366	0.08717487	0.640887434
56291	'Styx'	996.45	958.66	1176.3	455.56	927.32	779.02	1043.793333	720.6333333	-0.549240446	0.115243651	0.702526958
56292	'Naa10'	360	399	322	158	409	395	360.3333333	320.6666667	-0.189814676	0.590340507	0.972790461
56293	'Slc35g3'	17	18	1	4	13	1	12	6	-0.869798704	0.504884052	0.972790461
56294	'Ptpn9'	1393	1427	1939	1141	1461	1954	1586.333333	1518.666667	-0.066124283	0.860693674	0.976863639
56295	'Higd1a'	1082	981	8033	673	588	1074	3365.333333	778.3333333	-2.218790675	0.013627866	0.292430015
56296	'Dmrtb1'	53	46	10	0	35	143	36.33333333	59.33333333	0.6063775	0.652815084	0.972790461
56297	'Arl6'	317	297	158	100	274	278	257.3333333	217.3333333	-0.242370076	0.565652806	0.972790461
56298	'Atl2'	913	947	998	1418	1074	1429	952.6666667	1307	0.550413879	0.176903597	0.796610891
56299	'Fkbpl'	153	183	162	113	141	222	166	158.6666667	-0.049929573	0.888019091	0.984534762
56305	'Pitpnb'	1570	1691	1755	1468	1690	1893	1672	1683.666667	0.05703521	0.856153935	0.975734242
56306	'Sinhcaf'	756	793	1750	487	668	803	1099.666667	652.6666667	-0.796906991	0.134158144	0.733437989
56307	'Metap2'	3973.7	4010.6	4616.2	1958	3431.5	4485.89	4200.183333	3291.78	-0.378601059	0.265660988	0.88190787
56309	'Mycbp'	462	511	282	280	365	687	418.3333333	444	0.112738374	0.772448256	0.972790461
56310	'Gps2'	477.4	459.69	358.33	237.16	463.58	564.57	431.8066667	421.77	-0.03539313	0.90833875	0.988823459
56312	'Nupr1'	24	27	957	3429	21	1108	336	1519.333333	2.317278767	0.250846558	0.871167957
56314	'Zfp113'	681	707.35	220	169	582	385	536.1166667	378.6666667	-0.473741878	0.382769985	0.945348183
56315	'Rhcg'	7	4	3	2	7	2	4.666666667	3.666666667	-0.311926435	0.789576627	0.972790461
56316	'Ggcx'	548	516	301	703	655	566	455	641.3333333	0.639834465	0.124836818	0.718643987
56317	'Anapc7'	1147	1149	1448	1371	1218	1509	1248	1366	0.193865843	0.62921061	0.972790461

56318	'Acpp'	27	30	30	15	48	78	29	47	0.61800268	0.311867455	0.911537035
56320	'Dbn1'	1727	1770	1760	1842	2482	1981	1752.333333	2101.666667	0.31935099	0.318702735	0.916136994
56321	'Aatf'	775	766	948	124	608	803	829.6666667	511.6666667	-0.817874604	0.16144676	0.771435531
56322	'Timm22'	660	752	419	259	544	706	610.3333333	503	-0.276549771	0.448541864	0.97049895
56323	'Dnajb5'	104	123	148	126	148	124	125	132.6666667	0.13813395	0.749459238	0.972790461
56324	'Stam2'	1219	1246	1488	1370	1194	1510	1317.666667	1358	0.111528222	0.777580507	0.972790461
56325	'Abcb9'	56	67	16	34	76	120	46.33333333	76.66666667	0.747548659	0.234092776	0.861178766
56327	'Arl2'	382	368	386	198	394	612	378.6666667	401.3333333	0.040147679	0.919156625	0.990815958
56330	'Pdcd5'	872	905	1034	582	718	1248	937	849.3333333	-0.150371912	0.68767349	0.972790461
56332	'Amotl2'	809	802	2438	1114	781	1027	1349.666667	974	-0.447515688	0.502974981	0.972790461
56334	'Tmed2'	4534	4462	10827	4996	4212	7756	6607.666667	5654.666667	-0.246814373	0.672125278	0.972790461
56335	'Mettl3'	443.85	440.21	412.11	135	523	528.15	432.0566667	395.3833333	-0.194987147	0.676703746	0.972790461
56336	'B4galt5'	1665	1600	2218	4401	1775	2239	1827.666667	2805	0.79978331	0.198418853	0.824374834
56338	'Txnip'	8101	7378	23217	5359	7183	1884	12898.66667	4808.666667	-1.392028951	0.06829559	0.583566634
56347	'Eif3c'	6729	6618	9002	4046	5833	8927	7449.666667	6268.666667	-0.278348681	0.474382114	0.972790461
56348	'Hsd17b12'	1015	1058	1039	840	1263	1431	1037.333333	1178	0.198614987	0.460941721	0.972790461
56349	'Net1'	943	919	1869	2825	884	1227	1243.666667	1645.333333	0.583459861	0.426067712	0.960958977
56350	'Arl3'	452	468	704	823	509	789	541.3333333	707	0.467134589	0.365210373	0.935765447
56351	'Ptges3'	3463.3	3360	6786.7	2936.6	2604.5	3918.72	4536.66	3153.243333	-0.517851381	0.317661053	0.916136994
56353	'Rybp'	2683.1	2797	3740.3	2582.1	2431.4	3087.54	3073.466667	2700.366667	-0.140600466	0.729220829	0.972790461
56354	'Dnajc7'	3216.8	3380.9	2274	1569	3372.9	2909	2957.23	2616.97	-0.157984614	0.585695112	0.972790461
56356	'Gltp'	295	298	250	156	282	415	281	284.3333333	0.003419137	0.99212751	0.999493374
56357	'Ivd'	1048	1173	280	488	1755	1249	833.6666667	1164	0.510173447	0.38653883	0.94848753
56358	'Copz2'	249	268	801	172	246	372	439.3333333	263.3333333	-0.832683537	0.198234982	0.824298956
56360	'Acot9'	365	415	547	517	420	586	442.3333333	507.6666667	0.255928672	0.56731209	0.972790461
56361	'Pus1'	435	403	644	480	367	534	494	460.3333333	-0.047638968	0.920745232	0.991047523
56362	'Sult1b1'	12	13	4	0	6	5	9.666666667	3.666666667	-1.439738115	0.229061706	0.855540033
56363	'Tmeff2'	12	11	2	15	19	8	8.333333333	14	0.919553419	0.317393998	0.916136994
56364	'Zmym3'	3657	3824	1160	2049	5000	3221	2880.333333	3423.333333	0.314627102	0.531119667	0.972790461
56365	'Clcnkb'	0	0	0	3	2	1	0	2	3.63077071	0.184855137	0.804884883
56367	'Scoc'	632	610	776.06	467	791	605	672.6866667	621	-0.103517974	0.775637911	0.972790461
56368	'Cyb561d2'	99	104	75	109	133	184	92.66666667	142	0.661925121	0.046833734	0.503906594
56369	'Apip'	251	267	339	86	301	404	285.6666667	263.6666667	-0.217689201	0.680528975	0.972790461
56370	'Tagln3'	2	1	1	0	1	2	1.333333333	1	-0.490619081	0.810152226	0.972790461
56371	'Fzr1'	640	656	902	351	694	950	732.6666667	665	-0.19808128	0.638282262	0.972790461
56372	'1110004F10Rik'	1597	1685	2505	1075	1770	1826	1929	1557	-0.332138301	0.406965701	0.957157474
56373	'Cpb2'	5	4	1	0	3	3	3.333333333	2	-0.770761376	0.626408035	0.972790461
56374	'Tmem59'	2427	2519	1880	3532	3182	4552	2275.333333	3755.333333	0.808878037	0.013780905	0.292512191
56375	'B4galt4'	322	373	179	829	1232	1635	291.3333333	1232	2.127919664	6.48E-10	6.85E-07
56376	'Pdlim5'	1004	1006	1481	1295	945	1239	1163.666667	1159.666667	0.07095671	0.881908836	0.982528614

56378	'Arpc3'	806	883	1421	1361	986	1315	1036.666667	1220.666667	0.300385766	0.555811365	0.972790461
56379	'Kcnj1'	1	0	0	2	0	0	0.333333333	0.666666667	1.337350669	0.73871782	0.972790461
56380	'Arid3b'	333	344	111	55	254	435	262.6666667	248	-0.128363629	0.849744257	0.975734242
56381	'Spen'	2817	2670	996	6291	2427	2672	2161	3796.666667	1.102027044	0.102375305	0.671776916
56382	'Rab9'	547	560	795	886	633	846	634	788.3333333	0.393800692	0.409788753	0.957157474
56384	'Letm1'	1052	1000	340	565	1121	1311	797.3333333	999	0.371180713	0.427044165	0.961636249
56386	'B4galt6'	570	527	243.54	101	195	201	446.8466667	165.6666667	-1.385580084	4.65E-04	0.042068288
56389	'Stx5a'	1242	1251	3219	1099	1318	1423	1904	1280	-0.600416546	0.297212843	0.900647119
56390	'Znrd2'	203	270	216	269	269	446	229.6666667	328	0.557429829	0.125287852	0.720137383
56392	'Shoc2'	1822	1758	1996	1181	1562	2215	1858.666667	1652.666667	-0.165514108	0.607986023	0.972790461
56395	'Tmem115'	514	498	415	605	671	802	475.6666667	692.6666667	0.60827892	0.03346694	0.441243737
56397	'Morf4l2'	5100	5385	10437	4375	5959	6798	6974	5710.666667	-0.316942471	0.514784506	0.972790461
56398	'Chp1'	1512.3	1551.8	1627.2	4429	1717	1882	1563.796667	2676	0.996079076	0.113955981	0.698948431
56399	'Akap8'	2631	2736	1828	1237	2795	1809	2398.333333	1947	-0.269377912	0.424630388	0.960285372
56401	'P3h1'	973	935	784	1593	1519	1898	897.3333333	1670	0.980728549	0.002166871	0.10389089
56403	'Syncrip'	4658	4636	9237	2886	4469	4486	6177	3947	-0.681451187	0.159246112	0.770378392
56404	'Trip4'	1755.9	1833	1182.1	585	1816	1401.51	1590.316667	1267.503333	-0.337620814	0.383996757	0.945936404
56405	'Dusp14'	41	53	75	46	24	67	56.33333333	45.66666667	-0.266613216	0.673372625	0.972790461
56406	'Ncoa6'	2395	2079	1933	1255	1901	2143	2135.666667	1766.333333	-0.248950631	0.312387626	0.911537035
56407	'Trpc4ap'	3283	3263	3479	2546	3143	3650	3341.666667	3113	-0.067535149	0.818772497	0.972790461
56409	'Nudt3'	1389	1501	1455	1603	1416	1736	1448.333333	1585	0.213393524	0.554769677	0.972790461
56410	'Cbln3'	115	92	96	13	65	28	101	35.33333333	-1.554740901	0.008088593	0.217203844
56412	'Noa1'	739.28	720	350	402	427	415	603.0933333	414.6666667	-0.416093943	0.29241736	0.900043079
56417	'Adar'	1235	1198	823	654	1371	1619	1085.333333	1214.666667	0.162600217	0.609596376	0.972790461
56418	'Ykt6'	1866	2006	1212	1709	2064	2255	1694.666667	2009.333333	0.326671815	0.257830262	0.877405867
56419	'Diaph3'	450	444	131	204	425	582	341.6666667	403.6666667	0.27467994	0.600595541	0.972790461
56420	'Ppp4c'	1469	1599	1445	1015	1501	2002	1504.333333	1506	0.009998275	0.971475194	0.999493374
56421	'Pfkp'	1530.1	1133	9519	1673	597	1583	4060.703333	1284.333333	-1.665195236	0.077797531	0.613825367
56422	'Hbs1l'	2327	2215	2208	1527	1662	1963	2250	1717.333333	-0.331947126	0.270749656	0.883910323
56424	'Stub1'	1211.1	1308.7	1509.7	1464.4	1348.4	1948.43	1343.16	1587.056667	0.288432128	0.446184066	0.970172328
56426	'Pdcd10'	818	880	1227	1012	868	1255	975	1045	0.142900159	0.744216359	0.972790461
56427	'Tubd1'	216	237	84	196	263	211	179	223.3333333	0.436835429	0.340186744	0.92526901
56428	'Mtch2'	1142	1243	1430	778	1303	1397	1271.666667	1159.333333	-0.141572506	0.664131345	0.972790461
56429	'Dpt'	11	12	0	892	19	16	7.666666667	309	5.909413263	0.005742217	0.178384046
56430	'Clip1'	1535	1504	1295	1002	1465	2091	1444.666667	1519.333333	0.08379728	0.766455332	0.972790461
56431	'Dstn'	3013	3174	3909	5014	4376	4849	3365.333333	4746.333333	0.572948652	0.166441707	0.780893009
56433	'Vps29'	993.82	986.44	1212.5	779.38	839.9	1023.69	1064.253333	880.99	-0.234412079	0.512746834	0.972790461
56434	'Tspan3'	1912	1985	822	2366	3451	3480	1573	3099	1.062637393	0.004928051	0.16556266
56436	'Adrm1'	1363	1489	1212	2528	1347	1986	1354.666667	1953.666667	0.686205644	0.157535231	0.768337387
56437	'Rrad'	197	208	695	58	327	197	366.6666667	194	-1.094011752	0.15494826	0.766453805

56438	'Rbx1'	1073	1243	1457	786	1230	1395	1257.666667	1137	-0.153313045	0.655438074	0.972790461
56440	'Snx1'	1571	1744	2949	1209	1703	2145	2088	1685.666667	-0.340725969	0.454156921	0.970649024
56441	'Naa80'	108.04	128.67	185.42	97	213.76	162	140.71	157.5866667	0.129620421	0.780524611	0.972790461
56442	'Serinc1'	3001	2992	2921	4421	4256	5561	2971.333333	4746	0.742287403	0.022844994	0.376368311
56443	'Arpc1a'	3118	2913	3969	1606	1949	2643	3333.333333	2066	-0.684102919	0.061947318	0.564873458
56444	'Actr10'	1851	1975.2	1905	1461	1611	1943	1910.396667	1671.666667	-0.139982689	0.642968718	0.972790461
56445	'Dnaja2'	3476	3414	2994	4267	3183	4741	3294.666667	4063.666667	0.403837371	0.290154326	0.898146215
56447	'Copz1'	1446	1490	2444	1240	1499	1792	1793.333333	1510.333333	-0.248977218	0.568008616	0.972790461
56448	'Cyp2d22'	208	231	58	107	229	204	165.6666667	180	0.185482877	0.730512762	0.972790461
56449	'Ybx3'	2388	2272	9069	3349	2491	4533	4576.333333	3457.666667	-0.443580352	0.540445689	0.972790461
56451	'Suclg1'	848	886	876	970	826	1229	870	1008.333333	0.28230457	0.438992279	0.967306223
56452	'Orc6'	517	548	641	289	516	465	568.6666667	423.3333333	-0.42871533	0.20613407	0.833294914
56453	'Mbtps1'	4242	4100	1348	1138	5546	5307	3230	3997	0.286915754	0.632529119	0.972790461
56454	'Aldh18a1'	751	723	2152	815	913	1401	1208.666667	1043	-0.272403998	0.660874194	0.972790461
56455	'Dynll1'	2536	2669	2444	674	2191	2172	2549.666667	1679	-0.657302268	0.110130225	0.690110694
56456	'Actl6a'	1029	1106	664	507	1059	1115	933	893.6666667	-0.047394334	0.879860543	0.981857642
56457	'Clptm1'	1749	1789	1411	3232	2445	3537	1649.666667	3071.333333	1.004951815	0.007782325	0.213195328
56458	'Foxo1'	1340	1273	995	768	978	1364	1202.666667	1036.666667	-0.175597083	0.517038574	0.972790461
56459	'Sae1'	1935	1994	1519	886	1552	2087	1816	1508.333333	-0.26635992	0.364019611	0.93523525
56460	'Pkp3'	5	3	5	8	3	4	4.333333333	5	0.365306737	0.74376121	0.972790461
56461	'Kcnip3'	121	138	50	14	130	78	103	74	-0.517088025	0.47227887	0.972790461
56462	'Mtch1'	2387	2537	1668	2685	3017	3305	2197.333333	3002.333333	0.536398852	0.064784821	0.572080365
56463	'Snd1'	3004	2710	6536	2445	2537	3957	4083.333333	2979.666667	-0.485807309	0.377110908	0.941504733
56464	'Ctsf'	643	628	287	1195	815	972	519.3333333	994	1.115353121	0.021937874	0.366505149
56468	'Socs5'	910	863	678	1626	993	1020	817	1213	0.748219827	0.127875632	0.723993903
56469	'Pias1'	1340	1422	1018	1097	1135	1166	1260	1132.666667	-0.055968934	0.860763735	0.976863639
56470	'Rgs19'	260	289	409	93	288	296	319.3333333	225.6666667	-0.587171242	0.233029919	0.859679981
56471	'Stmn4'	3	3	129	8	5	5	45	6	-2.962465612	0.038782457	0.461389364
56473	'Fads2'	1528	1663	643	676	2190	1367	1278	1411	0.167860919	0.72836071	0.972790461
56480	'Tbk1'	1571	1628	861	955	1042	1193	1353.333333	1063.333333	-0.247443277	0.467894536	0.972790461
56484	'Foxo3'	3572	3236	7390	3576	3287	4696	4732.666667	3853	-0.297206239	0.582293585	0.972790461
56485	'Slc2a5'	64	57	20	20	59	34	47	37.66666667	-0.271579498	0.654789309	0.972790461
56486	'Gabarap'	1990	1920	7247	2124	2499	3421	3719	2681.333333	-0.546075342	0.424495024	0.960285372
56488	'Nxt1'	404	477	342	383	398	450	407.6666667	410.3333333	0.093582686	0.769115576	0.972790461
56489	'Ikbke'	206	227	256	51	219	170	229.6666667	146.6666667	-0.724688321	0.154450015	0.766453805
56490	'Zbtb20'	689	722	274	2004	738	1188	561.6666667	1310	1.47798459	0.020652457	0.356497521
56491	'Vapb'	2272	2279	1942	3962	1997	2655	2164.333333	2871.333333	0.585004047	0.254035631	0.873052317
56492	'Cldn18'	1	0	0	0	1	0	0.333333333	0.333333333	0.058500858	0.988561293	0.999493374
56494	'Gosr2'	2412	2449	2919.1	1698	2105	2488	2593.35	2097	-0.283479272	0.390627988	0.952178913
56495	'Get3'	612	692	1099	782	696	1047	801	841.6666667	0.092188557	0.846504891	0.975201539

56496	'Tspan6'	915	1003	330	774	1539	1230	749.3333333	1181	0.727877544	0.107422102	0.683046488
56501	'Elf4'	1422	1298	2752	1307	1168	1397	1824	1290.666667	-0.472269628	0.371728386	0.938527219
56503	'Ankrd49'	792	734	488	96	450	417	671.3333333	321	-1.115689984	0.028292495	0.409950544
56504	'Srpk3'	2	4	1	0	2	0	2.333333333	0.666666667	-1.786757392	0.422419257	0.959731705
56505	'Ruvbl1'	1223	1263	1104	385	1187	1372	1196.666667	981.3333333	-0.340977947	0.413587611	0.95722888
56506	'Cib2'	561	620.96	238	204	585	478	473.32	422.3333333	-0.140881096	0.759890354	0.972790461
56508	'Rapgef4'	98	115.98	17	205	168	124	76.99333333	165.6666667	1.339440823	0.062180979	0.565368139
56513	'Pard6a'	125	91	198	124	92	158	138	124.6666667	-0.120197615	0.829700406	0.973695269
56515	'Rnf138'	1670	1556	1699	294	1046	1174	1641.666667	838	-1.048213399	0.023907053	0.382913609
56516	'Rbms2'	2726	2835	5962	2127	2651	3091	3841	2623	-0.574688714	0.263318705	0.88013169
56517	'Slc22a21'	469.67	430.7	389.44	107.76	305.21	170.77	429.9366667	194.58	-1.148034978	0.004838291	0.164614637
56520	'Nme4'	331	374	352	27	276	265	352.3333333	189.3333333	-1.018695652	0.140862874	0.744698431
56522	'Papolb'	60	49	9	0	37	12	39.33333333	16.33333333	-1.311703044	0.291035733	0.899022994
56523	'Pmfbp1'	5	5	1	0	6	19	3.666666667	8.333333333	1.094702287	0.465244547	0.972790461
56524	'Mpp6'	1164	1114	2279	501	787	1148	1519	812	-0.97167417	0.056004017	0.544349879
56525	'Zfp235'	327	299.01	128	116	304	241	251.3366667	220.3333333	-0.157556207	0.728186322	0.972790461
56526	'Septin6'	463	454	403	570	671	495	440	578.6666667	0.487242997	0.180873515	0.801479605
56527	'Mast1'	22	12	17	53	20	12	17	28.33333333	0.994859453	0.260807172	0.878537553
56529	'Sec11a'	1125	1187	2207	1125	1420	1823	1506.333333	1456	-0.073871104	0.877557237	0.981341203
56530	'Cnpy2'	1125	1142	976	221	1404	1607	1081	1077.333333	-0.105930613	0.859421376	0.976863639
56531	'Ylpm1'	2654	2565	926	1315	2487	2039	2048.333333	1947	0.001192726	0.997827983	0.999879071
56532	'Ripk3'	180	204	673	160	212	165	352.3333333	179	-1.016049489	0.141309944	0.746115932
56533	'Rgs17'	118	86	202	264	116	89	135.3333333	156.3333333	0.384460029	0.607880534	0.972790461
56535	'Pex3'	829.83	806.34	424.58	169	597.21	664.57	686.9166667	476.9266667	-0.55375097	0.248062676	0.869491261
56541	'Habp4'	537	499	439	433	507	512	491.6666667	484	0.04732789	0.872429337	0.980171118
56542	'Cilk1'	703	752	893	205	838	656	782.6666667	566.3333333	-0.547678933	0.26322701	0.88013169
56543	'Kcnd3'	191	177	88	52	136	185	152	124.3333333	-0.297939973	0.534945723	0.972790461
56544	'Vmn2r1'	48.74	49.61	40.52	14.92	62.09	49	46.29	42.00333333	-0.177618667	0.748592546	0.972790461
56546	'Sec1'	216.86	225.34	26	11.52	96.88	148.54	156.0666667	85.64666667	-0.908404988	0.32271848	0.918775094
56550	'Ube2d2a'	3232	3339	4947	2917	3068	3557	3839.333333	3180.666667	-0.241588133	0.564134981	0.972790461
56551	'Txn2'	1029	1093	1901	1043	1091	1334	1341	1156	-0.202663839	0.668061796	0.972790461
56554	'Raet1d'	38.61	33.02	37.06	48	62	52	36.23	54	0.639373831	0.153145443	0.764400413
56612	'Pfdn5'	2005	2131	2274	1783	1755	2436	2136.666667	1991.333333	-0.053298624	0.877204689	0.981341203
56613	'Rps6ka4'	389	403	639	244	415	527	477	395.3333333	-0.318007569	0.469642253	0.972790461
56615	'Mgst1'	1031	1138	1678	2046	1669	3181	1282.333333	2298.666667	0.858183135	0.068955258	0.585534444
56619	'Clec4e'	2	0	17	5	2	9	6.333333333	5.333333333	-0.365707702	0.821282212	0.972790461
56620	'Clec4n'	22	11	4	144	22	88	12.33333333	84.66666667	3.072114788	0.001028626	0.066973385
56622	'Adam21'	7	15	1	4	9	2	7.666666667	5	-0.475401014	0.699718628	0.972790461
56626	'Poll'	299	285	190	51	290	337	258	226	-0.265504825	0.65165393	0.972790461
56629	'Dnase2b'	0	1	0	0	0	0	0.333333333	0	-0.903279821	0.824807108	0.972790461

56631	'Trim17'	138.33	151.23	307.23	91.58	143.4	128.71	198.93	121.23	-0.751557408	0.161616238	0.771541907
56632	'Sphk2'	905.41	977.58	584.53	550.46	1257.6	1097.36	822.5066667	968.47	0.253472975	0.434885883	0.965608974
56635	'Prl2a1'	0	0	0	1	0	0	0	0.333333333	1.020273531	0.802557913	0.972790461
56636	'Fgf21'	2	0	19	74	1	31	7	35.33333333	2.464628198	0.141942175	0.747514336
56637	'Gsk3b'	3455	3474	5188	2439	2939	3257	4039	2878.333333	-0.474191289	0.238560752	0.864053647
56642	'Ankrd2'	0	0	0	0	1	1	0	0.666666667	1.775692139	0.660844521	0.972790461
56643	'Slc15a1'	4	3	0	7	7	19	2.333333333	11	2.304303101	0.053780214	0.533926597
56644	'Clec7a'	3	5	6	8	18	98	4.666666667	41.33333333	2.99787543	0.003371549	0.132437773
56690	'Mlycd'	442	493	255	333	463	574	396.6666667	456.6666667	0.262029689	0.417828213	0.957720764
56692	'Lamtor3'	582	594	1139	1165	628	955	771.6666667	916	0.334004972	0.574629833	0.972790461
56693	'Crtap'	848	920	982	359	1213	1352	916.6666667	974.6666667	0.011203828	0.980464878	0.999493374
56695	'Pnkd'	829	806	350	803	980	938	661.6666667	907	0.566924722	0.153329025	0.765115861
56696	'Gpr132'	3	1	23	9	0	0	9	3	-1.304857581	0.5307208	0.972790461
56697	'Akap10'	1078	1133	735	739	1076	891	982	902	-0.049917709	0.863676784	0.977452409
56698	'Phax'	781	824	1208	320	805	999	937.6666667	708	-0.487697634	0.295600513	0.90029104
56699	'Cdc42ep4'	1146	1153	1055	846	1207	1197	1118	1083.333333	-0.006313741	0.980110294	0.999493374
56700	'Glmp'	654	649	810	869	969	1340	704.3333333	1059.333333	0.609886427	0.090446367	0.649137912
56702	'H1f5'	1	1	3	32	2	1	1.666666667	11.66666667	3.165950243	0.053767179	0.533926597
56703	'Pigo'	461	462	214	349	649	411	379	469.6666667	0.388887759	0.342359692	0.925308103
56705	'Ranbp9'	1817	1770	1535	1598	1535	1923	1707.333333	1685.333333	0.061634345	0.845863632	0.975182082
56706	'Ccnl1'	2110	2138	2645	2862	2867	1514	2297.666667	2414.333333	0.185197063	0.718695043	0.972790461
56707	'Zfp111'	566.51	575.83	339.46	249.54	457.57	390.04	493.9333333	365.7166667	-0.38598997	0.211768399	0.840560909
56708	'Clcf1'	218	199	254	388	192	202	223.6666667	260.6666667	0.393759759	0.504690534	0.972790461
56709	'Dnajb12'	592	516	878	723	684	784	662	730.3333333	0.183952928	0.688320723	0.972790461
56710	'Brinp1'	126	84	56	12	96	50	88.66666667	52.66666667	-0.795980614	0.24431773	0.866091613
56711	'Plag1'	1402	1250	1174	806	1374	950	1275.333333	1043.333333	-0.245788495	0.43587459	0.96581102
56715	'Rabgef1'	552	576	828	798	550	677	652	675	0.141439912	0.77641989	0.972790461
56716	'Mlst8'	507	510.91	292	1133.7	700	589.93	436.6366667	807.8666667	1.09960232	0.041436553	0.476408328
56717	'Mtor'	3620	3491	770	1696	2293	2292	2627	2093.666667	-0.204249712	0.714505598	0.972790461
56720	'Tdo2'	1	1	0	1	2	1	0.666666667	1.333333333	1.104064927	0.607441764	0.972790461
56722	'Litaf'	1603	1618	3332	2231	1391	1639	2184.333333	1753.666667	-0.23332974	0.69577092	0.972790461
56724	'Cript'	1004	990	1090	619	725	805	1028	716.3333333	-0.477568267	0.133680195	0.733301819
56726	'Sh3bgrl'	2265	2325	2800	1274	2681	2282	2463.333333	2079	-0.265597307	0.449279104	0.97049895
56727	'Miox'	1	1	1	3	1	0	1	1.333333333	0.696694547	0.738061988	0.972790461
56734	'Tulp2'	223	257	19	32	305	216	166.3333333	184.3333333	0.142534454	0.877896398	0.981341203
56735	'Krt71'	0	0	0	0	0	1	0	0.333333333	1.020273531	0.802557913	0.972790461
56736	'Rnf14'	1492	1561	1436	1589	1564	1389	1496.333333	1514	0.117329938	0.750790155	0.972790461
56737	'Alg2'	1072	1058	987	870	1297	1400	1039	1189	0.220449291	0.373500207	0.93929675
56738	'Mocs1'	328	366	353	384	329	565	349	426	0.340895953	0.370926211	0.9384981
56739	'Rec8'	176	166	52	31	458	196	131.3333333	228.3333333	0.755290853	0.356707008	0.932066196

56741	'Igdcc4'	3074	2891	1126	930	4050	3118	2363.666667	2699.333333	0.182694665	0.736302044	0.972790461
56742	'Psrc1'	92	100	129	232	109	141	107	160.6666667	0.74032086	0.218819515	0.845396589
56743	'Lat2'	24	18	27	47	30	52	23	43	0.979079382	0.099028899	0.668585731
56744	'Pf4'	11	5	1	74	11	25	5.666666667	36.66666667	3.060261952	0.006234845	0.186739406
56745	'C1qtnf1'	456	439	192	277	704	732	362.3333333	571	0.671630334	0.122898427	0.714169238
56746	'Tex101'	953	929	564	16	529	564	815.3333333	369.6666667	-1.258960289	0.162689206	0.774103809
56747	'Sez6l'	48	58	10	55	121	77	38.66666667	84.33333333	1.215602374	0.063870023	0.5684378
56748	'Nfu1'	472	486	762	207	506	663	573.3333333	458.6666667	-0.40935342	0.399299787	0.955210216
56749	'Dhodh'	312	357	214	92	322	343	294.3333333	252.3333333	-0.25768295	0.573679514	0.972790461
56752	'Aldh9a1'	1544	1469	1938	503	1714	3448	1650.333333	1888.333333	0.068666608	0.908136321	0.988823459
56753	'Tacstd2'	4	3	5	27	5	11	4	14.33333333	2.071667559	0.056981844	0.547413448
56758	'Mbnl1'	1115	1058	1818	1482	1215	1481	1330.333333	1392.666667	0.120259799	0.805860564	0.972790461
56760	'Clec1b'	2	3	2	0	0	0	2.333333333	0	-3.597622416	0.169448056	0.785100556
56771	'Med20'	873.17	877.49	792.09	874.61	1063.8	917.35	847.5833333	951.9266667	0.240506863	0.442997627	0.96975311
56772	'Mllt11'	146.2	162	487.23	217.41	135	166	265.1433333	172.8033333	-0.57310693	0.422572294	0.959735331
56773	'Chst5'	1	4	1	11	8	6	2	8.333333333	2.238375393	0.060034267	0.55595299
56774	'Slc6a14'	80	78	51	38	199	102	69.66666667	113	0.666021227	0.245599551	0.866779035
56784	'Ralgapa1'	1792	1975	1580	1906	2435	2385	1782.333333	2242	0.393343395	0.13738515	0.738626463
56786	'Tmem9b'	385	397	354	897	469	718	378.6666667	694.6666667	1.027025559	0.037989214	0.459930654
56787	'Ascl3'	2	1	1	4	2	1	1.333333333	2.333333333	1.017996177	0.528157082	0.972790461
56788	'Scube2'	162	149	71	481	179	355	127.3333333	338.3333333	1.630271503	0.008049046	0.216529539
56790	'Supt20'	1345	1328	1196	602	1715	1143	1289.666667	1153.333333	-0.176540066	0.634830702	0.972790461
56791	'Ube2l6'	551	632.79	677	364	715	909	620.2633333	662.6666667	0.06043968	0.86774749	0.979066656
56792	'Stap1'	7	6	1	9	2	3	4.666666667	4.666666667	0.313283943	0.809108216	0.972790461
56794	'Hacl1'	225	253	128	72	219	286	202	192.3333333	-0.095917705	0.841803897	0.975182082
56795	'Arl10'	431	460	330	457	659	567	407	561	0.526472273	0.059734836	0.554643744
56805	'Zbtb33'	1247.7	1298	1013	962	1137.6	1342	1186.223333	1147.196667	0.012510932	0.961948507	0.999493374
56807	'Scamp5'	682	689	346	207	1042	827.89	572.3333333	692.2966667	0.238500638	0.656993248	0.972790461
56808	'Cacna2d2'	65	67	11	10	95	102	47.66666667	69	0.499672807	0.568911371	0.972790461
56809	'Gmeb1'	961.89	916.65	818.12	1620	829	686	898.8866667	1045	0.43325619	0.459116714	0.972790461
56811	'Dkk2'	17	6	2	13	13	7	8.333333333	11	0.589615271	0.558240084	0.972790461
56812	'Dnajb2'	709	772	1051	406	845	902	844	717.6666667	-0.280928431	0.486283179	0.972790461
56838	'Ccl28'	27	41	17	29	31	16	28.33333333	25.33333333	-0.008688758	0.989333395	0.999493374
56839	'Lgi1'	34	24	5	3	51	20	21	24.66666667	0.209645565	0.837523252	0.974723675
56843	'Trpm5'	465	399	84	3	522	308	316	277.6666667	-0.25176171	0.828216112	0.973238667
56844	'Tssc4'	367.69	432.55	446.81	810.22	393.5	621.81	415.6833333	608.51	0.695696918	0.19056192	0.813136091
56846	'Necab3'	148	162	37	28	211	185	115.6666667	141.3333333	0.261531033	0.726125482	0.972790461
56847	'Aldh1a3'	117	158	26	68	174	162	100.3333333	134.6666667	0.479572875	0.455846018	0.971239019
56856	'Insm2'	14	7	1	1	1	0	7.333333333	0.666666667	-3.260048256	0.058946586	0.552865869
56857	'Slc37a2'	517	525	51	545	783	791	364.3333333	706.3333333	1.087469113	0.136697042	0.737900102

56860	'Olfr690'	0	1	0	0	0	0	0.333333333	0	-0.903279821	0.824807108	0.972790461
56863	'Cldn9'	0	0	0	5	0	0	0	1.666666667	3.648996947	0.355284993	0.931809687
56868	'Psg23'	0	1	1	0	2	0	0.666666667	0.666666667	-0.09902169	0.976599173	0.999493374
56869	'Zfp109'	284	334	206	96	297	234	274.6666667	209	-0.403834032	0.319433671	0.9165187
56873	'Lmbr1'	610	589	268	212	609	586	489	469	-0.053459607	0.904213914	0.987963813
56874	'Rnf32'	362	369	73	19	193	191	268	134.3333333	-1.034525119	0.204610726	0.833294914
56876	'Nsmf'	933	1050	672	570	1377	838	885	928.3333333	0.09673483	0.790209199	0.972790461
56878	'Rbms1'	1960	1863	4077	2697	1839	2265	2633.333333	2267	-0.153893626	0.791869585	0.972790461
57014	'Htr3b'	9	7	5	0	2	2	7	1.333333333	-2.42307789	0.066372622	0.575690169
57028	'Pdxp'	285	266	881	114	214	275	477.3333333	201	-1.363294848	0.038494229	0.460284458
57080	'Gtf2ird1'	1043	1021	1290	1400	1272	1395	1118	1355.666667	0.350205082	0.387640262	0.949501169
57138	'Slc12a5'	183	207	53	52	326	93	147.6666667	157	0.105279143	0.886909501	0.984522884
57170	'Dolpp1'	249	214	92	126	283	295	185	234.6666667	0.374718555	0.402764485	0.955996639
57230	'Sap30bp'	799	807	882	432	850	967	829.3333333	749.6666667	-0.170332729	0.605893414	0.972790461
57246	'Tbx20'	6	3	1	2	14	9	3.333333333	8.333333333	1.30852813	0.263390675	0.88013169
57247	'Zfp276'	531.57	564.31	379.97	330.03	610.11	505.72	491.95	481.9533333	0.010324721	0.970984943	0.999493374
57249	'Gabrq'	28	23	3	5	8	9	18	7.333333333	-1.195691198	0.201686312	0.82952222
57253	'Tas2r108'	1	1	0	1	0	1	0.666666667	0.666666667	0.219813875	0.93648942	0.994413066
57255	'Cldn13'	11	12	0	0	11	2	7.666666667	4.333333333	-0.826889623	0.652003669	0.972790461
57257	'Vav3'	244	258	108	364	314	290	203.3333333	322.6666667	0.831678925	0.082263244	0.622359253
57258	'Xpo4'	1240.1	1274	958.02	460	1276	1057	1157.34	931	-0.326894265	0.351373023	0.929144534
57259	'Tob2'	726	699	579	716	569	751	668	678.6666667	0.130209906	0.727830059	0.972790461
57260	'Ltb4r2'	4	7	5	3	4	2	5.333333333	3	-0.754956448	0.493722271	0.972790461
57261	'Brd4'	3032	2701	4500	2587	2322	2751	3411	2553.333333	-0.369786139	0.419155734	0.958308933
57264	'Retn'	1	0	3	8	2	1	1.333333333	3.666666667	1.660854426	0.360287019	0.933706718
57265	'Fzd2'	705	642	278	987	858	787	541.6666667	877.3333333	0.864187539	0.070113839	0.589465353
57266	'Cxcl14'	14	9	7	28	8	10	10	15.33333333	0.882392985	0.343125831	0.925308103
57267	'Apba3'	602	587	1424	661	617	759	871	679	-0.355539678	0.534007732	0.972790461
57269	'Olfr1507'	0	0	0	0	0	1	0	0.333333333	1.020273531	0.802557913	0.972790461
57274	'Slc16a8'	16	6	6	30	11	8	9.333333333	16.33333333	1.086147501	0.268631062	0.883600995
57275	'Lenep'	9	15	5	3	9	14	9.666666667	8.666666667	-0.163669937	0.852774446	0.975734242
57276	'Vsig2'	8	8	4	0	11	3	6.666666667	4.666666667	-0.578503495	0.664851152	0.972790461
57278	'Bcam'	1385	1294	340	1480	2064	2535	1006.333333	2026.333333	1.105592411	0.033133385	0.438703227
57279	'Slc25a20'	685	815	320	733	914	1044	606.6666667	897	0.659039554	0.091024469	0.649965349
57294	'Rps27'	3779	3972.8	7127.6	6768	2766	4172.34	4959.796667	4568.78	0.018606147	0.976947297	0.999493374
57295	'Icmt'	1348	1322	531	2268	1288	1289	1067	1615	0.829639407	0.148548825	0.75695799
57296	'Psmd8'	1402	1526	3773	2728	1648	2214	2233.666667	2196.666667	0.033724756	0.958392915	0.999493374
57312	'Mrps31'	461	403	447	232	385	458	437	358.3333333	-0.289359784	0.350345948	0.92886351
57314	'Nelfcd'	1179	1311	244	549	1030	1042	911.3333333	873.6666667	0.02223626	0.969738199	0.999493374
57315	'Wdr46'	717	729	663	388	594	766	703	582.6666667	-0.261605896	0.343874889	0.925308103

57316	'C1d'	834	865	874	307	1009	1098	857.6666667	804.6666667	-0.159517591	0.715565108	0.972790461
57317	'Srsf4'	1391	1461	1958	1009	1500	1539	1603.333333	1349.333333	-0.249386877	0.49479632	0.972790461
57319	'Smpdl3a'	339	283	123	462	551	730	248.3333333	581	1.315858436	0.001801265	0.091321728
57320	'Park7'	1545	1733	1270	904	1539	2181	1516	1541.333333	0.024488487	0.936294118	0.994413066
57321	'Terf2ip'	850	935	998	774	896	970	927.6666667	880	-0.029971607	0.927534195	0.992951516
57329	'Otor'	2	2	1	0	1	1	1.666666667	0.666666667	-1.335893275	0.514852727	0.972790461
57330	'Gigyf1'	1987	1815	978	1553	2055	1455	1593.333333	1687.666667	0.196557737	0.613015118	0.972790461
57339	'Jph1'	118	102	389	149	104	187	203	146.6666667	-0.489923235	0.499680784	0.972790461
57340	'Jph3'	6	9	2	6	6	4	5.666666667	5.333333333	0.066397208	0.949102032	0.997742943
57342	'Parva'	3162	3166	3266	1110	2972	2871	3198	2317.666667	-0.506796984	0.166553816	0.780893009
57344	'As3mt'	379	495	416	140	611	430	430	393.6666667	-0.18725562	0.701249132	0.972790461
57349	'Ppbp'	0	0	0	19	0	2	0	7	5.676321196	0.039068236	0.462096334
57354	'Crampl1'	2048	2059	914	1325	1518	1453	1673.666667	1432	-0.105283541	0.789221243	0.972790461
57355	'BC051019'	16	12	2	9	2	1	10	4	-0.944058942	0.474580743	0.972790461
57357	'Srd5a3'	282.11	288.16	133.68	120.91	436.44	403.45	234.65	320.2666667	0.434396397	0.375763255	0.940406075
57370	'B4galt3'	378	445	381	822	684	732	401.3333333	746	1.005807504	0.01127567	0.263357544
57373	'Akip1'	177	194	286	289	189	245	219	241	0.228196502	0.665436723	0.972790461
57376	'Smarce1'	2455	2602	2633	929	2745	2218	2563.333333	1964	-0.425843985	0.268997093	0.883851074
57377	'Mogs'	887	936	608	852	1022	1235	810.3333333	1036.333333	0.423465679	0.127012574	0.722550284
57385	'P2ry4'	7	11	1	1	13	5	6.333333333	6.333333333	0.005120778	0.996777417	0.999616639
57390	'Psors1c2'	4	3	1	4	2	3	2.666666667	3	0.340525542	0.796944142	0.972790461
57394	'Cltrn'	2	2	1	5	9	3	1.666666667	5.666666667	1.85714632	0.142962415	0.749054337
57423	'Atp5j2'	1321	1525	1263	733	1010	1459	1369.666667	1067.333333	-0.344735945	0.235500473	0.861636582
57429	'Sult5a1'	85	84	39	99	74	52	69.33333333	75	0.312640059	0.594059175	0.972790461
57431	'Dnajc4'	114	104	137	55	134	140	118.3333333	109.6666667	-0.156866897	0.716751128	0.972790461
57432	'Zc3h8'	264	288	152	59	191	159	234.6666667	136.3333333	-0.787691947	0.073990641	0.601158152
57434	'Xrcc2'	283	321	106	80	231	263	236.6666667	191.3333333	-0.297684773	0.568269612	0.972790461
57435	'Plin4'	8	7	6	12	16	14	7	14	1.060875502	0.141501701	0.746299453
57436	'Gabarapl1'	1379	1433	7426	3717	1585	3328	3412.666667	2876.666667	-0.233610814	0.78451335	0.972790461
57437	'Golga7'	2740	2819	2902	2258	2626	3233.98	2820.303333	2705.986667	-0.020292855	0.946049733	0.996592403
57438	'Marchf7'	2308	2189	3025	2127	2446	2842	2507.333333	2471.666667	0.005141964	0.98903722	0.999493374
57439	'Tmem183a'	1498	1426	1317	828	1183	1582	1413.666667	1197.666667	-0.223692197	0.408363777	0.957157474
57440	'Ehd3'	334	361	126	124	590	444	273.6666667	386	0.484677027	0.400328812	0.955860942
574402	'Gpr17'	3	2	1	1	3	0	2	1.333333333	-0.494456858	0.789156945	0.972790461
574403	'Insyn2b'	635	641	39	16	549	421	438.3333333	328.6666667	-0.452867446	0.680486553	0.972790461
574404	'Gm14685'	53.68	52.74	4.73	0	31.76	43.35	37.05	25.03666667	-0.61753125	0.6420421	0.972790461
574405	'DXBay18'	46.3	27.99	9.27	0	10.24	36.6	27.85333333	15.61333333	-0.929416671	0.453807373	0.970649024
57441	'Gmnn'	1002.6	1066.9	477	519	851	781.75	848.8533333	717.25	-0.171404584	0.633916638	0.972790461
574417	'Tas2r137'	0	0	1	1	2	0	0.333333333	1	1.582610237	0.62491275	0.972790461
574418	'Serinc4'	16.14	4.78	1.21	13.3	10.83	10.47	7.376666667	11.53333333	0.846653966	0.439796617	0.967612218

57442	'Kcne3'	81	66	19	0	7	11	55.33333333	6	-3.247586646	7.24E-04	0.053564853
574428	'Zmynd15'	45.49	58.83	369.74	183.01	64.41	68.96	158.02	105.46	-0.488401576	0.629653691	0.972790461
574429	'Psg26'	0	0	0	0	5	2	0	2.333333333	3.537325462	0.323229498	0.918976057
57443	'Fbxo3'	1929	1994	1788	1525	2147	2401	1903.666667	2024.333333	0.120467448	0.620568497	0.972790461
574437	'Xlr3b'	121.5	107.81	92.14	62.55	168.3	132.62	107.15	121.1566667	0.169613828	0.665662082	0.972790461
574438	'Xlr5a'	244.3	200.77	64.1	3	87.16	265.82	169.7233333	118.66	-0.619551199	0.560208363	0.972790461
57444	'Isg20'	35	31	273	92	118	96	113	102	-0.237876759	0.799839077	0.972790461
57737	'Rhox4b'	0	0	1.11	0	0	2	0.37	0.666666667	0.78767502	0.845746136	0.975182082
57738	'Slc15a2'	53	126	47	142	173	50	75.33333333	121.6666667	0.86633701	0.21264917	0.841028891
57740	'Stk32c'	46	45	155	36	35	24	82	31.66666667	-1.358133893	0.077681485	0.613577234
57741	'Noc2l'	1631.6	1630.8	2230.4	1306.4	1765	2357	1830.943333	1809.436667	-0.026804262	0.942847011	0.996301887
57743	'Sec61a2'	2586	2577	958	466	2569	1255	2040.333333	1430	-0.518359692	0.388559367	0.95037832
57745	'Zfp112'	100	115	149	70	130	83	121.3333333	94.33333333	-0.358067031	0.432670894	0.964346274
57746	'Piwil2'	2331	1898	194	68	1625	4206	1474.333333	1966.333333	0.345677284	0.755366782	0.972790461
57748	'Jmy'	2286	2241	3391	3066	2244	2524	2639.333333	2611.333333	0.073638005	0.88114325	0.982528614
57749	'Piwil1'	78	58	4	1	41	39	46.66666667	27	-0.828965415	0.487337623	0.972790461
57750	'Wdr12'	869	827.54	763	473	608.42	724.55	819.8466667	601.99	-0.406831943	0.121699441	0.712603747
57751	'Rnf25'	427	428	446	366	495	539	433.6666667	466.6666667	0.135114601	0.645104625	0.972790461
57752	'Tacc2'	938	913	989	833	973	1164	946.6666667	990	0.103959902	0.736969682	0.972790461
57753	'Noc3l'	891	809	602	362	856	927	767.3333333	715	-0.111227802	0.741096664	0.972790461
57754	'Cend1'	6	2	2	6	6	6	3.333333333	6	0.950310809	0.381752508	0.94429001
57755	'Dnajb7'	6	6	1	1	3	2	4.333333333	2	-1.049942648	0.440271394	0.968240572
57756	'Fhl5'	2	0	0	0	0	0	0.666666667	0	-1.711373851	0.67234292	0.972790461
57757	'Pglyrp2'	8	4	14	1	2	2	8.666666667	1.666666667	-2.432225375	0.04572174	0.498244985
57764	'Ntn4'	108	71	10	11	193	226	63	143.3333333	1.137316025	0.25799807	0.877405867
57765	'Tbx21'	3	1	1	1	0	0	1.666666667	0.333333333	-2.129640878	0.383586569	0.945636392
57773	'Wdr4'	1096	1065	920	320	1111	640	1027	690.3333333	-0.596787601	0.163156662	0.77490244
57775	'Usp29'	174	174	57	252	110	88	135	150	0.448109049	0.53546919	0.972790461
57776	'Ttyh1'	73	65	37	123	107	186	58.33333333	138.6666667	1.337456358	0.002475742	0.111665047
57778	'Fmnl1'	58	75	16	8	59	63	49.66666667	43.33333333	-0.226538181	0.776830422	0.972790461
57780	'Fxyd7'	4	3	2	15	4	1	3	6.666666667	1.498932114	0.276006763	0.887591438
57781	'Cd200r1'	3	3	2	7	9	18	2.666666667	11.33333333	2.101408955	0.030050429	0.421372044
57782	'Rbak'	583	567	347	226	684	380	499	430	-0.199501164	0.63968438	0.972790461
57783	'Tnip1'	1761	1887	7931	1612	1458	2057	3859.666667	1709	-1.225416422	0.097970918	0.66522253
57784	'Bin3'	453	441	499	329	440	481	464.3333333	416.6666667	-0.131149359	0.675984698	0.972790461
57785	'Rangrf'	138.49	176.76	147.83	125.55	123.78	187	154.36	145.4433333	-0.032819193	0.929371223	0.993098341
57808	'Rpl35a'	4329.5	4431.1	8542.3	8835	4160	6101	5767.626667	6365.333333	0.254396951	0.683680777	0.972790461
57810	'Cdon'	2583	2414	1144	2230	3301	3557	2047	3029.333333	0.637849289	0.071528804	0.592293391
57811	'Rgr'	0	1	0	0	0	0	0.333333333	0	-0.903279821	0.824807108	0.972790461
57813	'Tk2'	569	555	565	429	640	917	563	662	0.230204769	0.465054113	0.972790461

57814	'Kcne4'	187	169	26	548	72	33	127.3333333	217.6666667	1.261757137	0.267666425	0.883600995
57815	'Spata5'	3735	3659.7	1426.3	398	1624.2	2285	2940.306667	1435.73	-1.070516358	0.066896626	0.578108476
57816	'Tesc'	5	7	2	100	12	103	4.666666667	71.66666667	4.149444179	3.02E-05	0.005803392
57837	'Eral1'	330	363	292	234	335	351	328.3333333	306.6666667	-0.058029626	0.824843637	0.972790461
57869	'Adck2'	548	553	255	247	625	646	452	506	0.175571057	0.673607926	0.972790461
57874	'Hacd3'	2525	2764	952	1730	3578	3813	2080.333333	3040.333333	0.592283582	0.179461231	0.799605559
57875	'Angptl4'	854	937	303	2595	1550	1917	698	2020.666667	1.745314952	0.00188744	0.094183748
57890	'Il17re'	51	52	0	5	73	83	34.33333333	53.66666667	0.622687498	0.644500015	0.972790461
57895	'Ccdc126'	213	157	319	693	190	314	229.6666667	399	0.991201302	0.180764087	0.801479605
57896	'Krcc1'	1008	1009	505	588	870	756	840.6666667	738	-0.103818407	0.763763434	0.972790461
57905	'Isy1'	507	552	576	432	528	596	545	518.6666667	-0.034519578	0.912738201	0.989773583
57908	'Zfp318'	3877	3819	1411	600	2465	1975	3035.666667	1680	-0.858165475	0.109852853	0.689773582
57911	'Gsdma'	1	0	2	3	0	0	1	1	0.329428216	0.91197767	0.989773583
57912	'Cdc42se1'	717.8	684	1524.8	1321.6	1019	1363	975.5233333	1234.53	0.373200246	0.508815009	0.972790461
57913	'Pidd1'	209	237	91	47	189	75	179	103.6666667	-0.762074502	0.194188471	0.818242734
57914	'Crlf2'	89	105	174	46	160	179	122.6666667	128.3333333	-0.048861909	0.933100579	0.994031155
57915	'Tbc1d1'	1039	1025	1382	2021	776	891	1148.666667	1229.333333	0.297055292	0.646439826	0.972790461
57916	'Tnfrsf13b'	14	21	10	1	10	12	15	7.666666667	-1.02338783	0.257433861	0.877405867
58170	'Asic5'	7	4	0	0	12	15	3.666666667	9	1.24345471	0.4704856	0.972790461
58172	'Sertad2'	974	835	2101	1082	712	917	1303.333333	903.6666667	-0.472207962	0.440199117	0.968193615
58175	'Rgs20'	10	6	30	17	4	1	15.33333333	7.333333333	-0.836265254	0.517959319	0.972790461
58176	'Rhbg'	4	2	0	6	6	3	2	5	1.520563413	0.287347259	0.894554788
58178	'Sorcs1'	44	53	50	3	180	187	49	123.3333333	1.173797182	0.246796733	0.868602029
58180	'Hic2'	784	777	485	523	470	644	682	545.6666667	-0.219009382	0.536591179	0.972790461
58182	'Prokr1'	26	23	11	35	44	31	20	36.66666667	0.988215205	0.083225992	0.627147271
58184	'Cnot9'	1434	1484	1031	322	994	1173	1316.333333	829.6666667	-0.705755303	0.093609396	0.657687298
58185	'Rsad2'	77	74	177	139	81	161	109.3333333	127	0.244832218	0.704801011	0.972790461
58186	'Rad18'	492	485	201	293	296	307	392.6666667	298.6666667	-0.261032273	0.550047011	0.972790461
58187	'Cldn10'	12	19	11	24	18	33	14	25	0.919850079	0.143886386	0.750780289
58188	'Vstm2b'	108	116	106	147	134	74	110	118.3333333	0.248190394	0.641214238	0.972790461
58193	'Extl2'	369	369	292	241	530	463	343.3333333	411.3333333	0.26705857	0.377287102	0.941504733
58194	'Sh3kbp1'	492	490	752	891	518	436	578	615	0.23851221	0.69078499	0.972790461
58198	'Sall1'	318	385	54	105	94	98	252.3333333	99	-1.162040219	0.090953436	0.649965349
58200	'Ppp1r1a'	8	13	65	20	11	18	28.66666667	16.33333333	-0.823733291	0.41624574	0.95722888
58202	'Nelfb'	1510	1503	1901	718	1420	1602	1638	1246.666667	-0.430146677	0.236440986	0.862272407
58203	'Zbp1'	0	1	19	1	0	3	6.666666667	1.333333333	-2.508956497	0.245097952	0.866091613
58205	'Pdcd1lg2'	6	2	0	3	1	1	2.666666667	1.666666667	-0.380980164	0.834471959	0.974723675
58206	'Zbtb32'	156	145	34	6	132	239	111.6666667	125.6666667	0.091353083	0.926247143	0.992381554
58207	'Slc43a3'	485	464	1493	1136	635	851	814	874	0.157242315	0.825028044	0.972790461
58208	'Bcl11b'	198	224	150	36	324	228	190.6666667	196	-0.042348645	0.948282328	0.997385684

58210	'Sectm1b'	2	4	3	5	0	3	3	2.666666667	0.045984843	0.9767291	0.999493374
58212	'Srrm3'	10	17	4	6	6	3	10.33333333	5	-0.880237196	0.369380038	0.937712122
58217	'Trem1'	0	0	0	2	3	0	0	1.666666667	3.327663315	0.375729461	0.940406075
58218	'Trem3'	0	1	0	0	1	0	0.333333333	0.333333333	0.058500858	0.988561293	0.999493374
58220	'Pard6b'	413	420	1036	1374	372	766	623	837.3333333	0.570339273	0.459078157	0.972790461
58222	'Rab37'	5	5	0	10	7	9	3.333333333	8.666666667	1.570206312	0.189883359	0.813027197
58223	'Mmp19'	104.7	97.78	151.36	569.16	142.97	265.02	117.9466667	325.7166667	1.679539725	0.022559505	0.372676826
58226	'Cacna1h'	2222	1956	1259	1378	3101	2085	1812.333333	2188	0.308683771	0.379123555	0.942578399
58227	'Fam184b'	74	65	187	4	83	68	108.6666667	51.66666667	-1.286042767	0.166004896	0.780822631
58229	'Efcc1'	53	43	29	2	55	35	41.66666667	30.66666667	-0.535140717	0.550318989	0.972790461
58230	'Rnf8'	698	656	506	179	887	1369	620	811.6666667	0.296659952	0.617053769	0.972790461
58231	'Stk4'	1988	2004	1597	1844	1954	2132	1863	1976.666667	0.168758305	0.570572717	0.972790461
58233	'Dnaja4'	842	909	335	304	643	685	695.3333333	544	-0.314470939	0.468550132	0.972790461
58234	'Shank3'	617	624	266	365	628	383	502.3333333	458.6666667	-0.034769683	0.936243892	0.994413066
58235	'Nectin1'	557	610	165	639	1005	827	444	823.6666667	0.999120506	0.045676492	0.498244985
58237	'Nkain4'	85	88	16	70	140	166	63	125.3333333	1.054711128	0.095419382	0.662411648
58238	'Fam181b'	155	173	78	32	41	16	135.3333333	29.66666667	-2.042904769	4.35E-04	0.040018506
58239	'Dexi'	197.72	199.74	209.4	228.13	261.56	217.3	202.2866667	235.6633333	0.295364364	0.433527691	0.964992995
58240	'Hs1bp3'	418	469	963	422	453	590	616.6666667	488.3333333	-0.350466535	0.511495393	0.972790461
58242	'Nudt11'	258.09	257.8	351.62	49.72	217.66	243.46	289.17	170.28	-0.877761452	0.11712067	0.705163127
58243	'Nap1l5'	4	10	15	22	8	4	9.666666667	11.33333333	0.440049661	0.688260771	0.972790461
58244	'Stx6'	1257	1310	1001	1354	1667	1394	1189.333333	1471.666667	0.392463199	0.204227664	0.833035048
58245	'Gpr180'	607	608	402	334	576	562	539	490.6666667	-0.096922728	0.718672333	0.972790461
58246	'Slc35b4'	828	908	421	654	1266	1072	719	997.3333333	0.525572701	0.141818638	0.747136019
58248	'1700123O20Rik'	460.19	513.75	505.97	608.79	565.07	642.66	493.3033333	605.5066667	0.376252835	0.304465599	0.907164095
58249	'Fibp'	542	564	346	194	636	583	484	471	-0.060734475	0.884764464	0.98386821
58250	'Chst11'	902	803	1440	5054	1260	1991	1048.333333	2768.333333	1.616558136	0.03153134	0.431276139
58251	'Cep295nl'	2	3	0	15	4	7	1.666666667	8.666666667	2.673546905	0.053151056	0.532968285
58518	'Cts6'	0	0	0	0	1	0	0	0.333333333	1.020273531	0.802557913	0.972790461
58520	'Erg28'	394.61	452.72	327	115	475	512.97	391.4433333	367.6566667	-0.152459432	0.756201045	0.972790461
58521	'Eid1'	3228	2986	7481	2119	3491	3386	4565	2998.666667	-0.662955056	0.226007795	0.85360199
58523	'Elp2'	2434	2401	1460	818	2318	2520	2098.333333	1885.333333	-0.171890879	0.664406161	0.972790461
58799	'Crbn'	1695.9	1720.8	2222.6	1204.9	1386.2	1597.63	1879.746667	1396.236667	-0.399795644	0.27306913	0.884738928
58800	'Trpm7'	4230	4312	1826	2597	4183	3712	3456	3497.333333	0.097562004	0.796397438	0.972790461
58801	'Pmaip1'	291	252	751	540	108	118	431.3333333	255.3333333	-0.523149693	0.570034059	0.972790461
58802	'Kcnmb4'	35	40	38	13	39	24	37.66666667	25.33333333	-0.585484752	0.278854478	0.889355605
58803	'Pga5'	1	3	1	0	2	0	1.666666667	0.666666667	-1.322446951	0.582200099	0.972790461
58804	'Cdc42ep5'	462	553	632	178	823	740	549	580.3333333	-0.017924817	0.973051881	0.999493374
58805	'Mlxipl'	239	268	399	1330	303	263	302	632	1.345420425	0.112112129	0.69684179
58807	'Slco1c1'	0	1	1	0	1	0	0.666666667	0.333333333	-0.886945817	0.805479824	0.972790461

58809	'Rnase4'	197	181	231	516.89	308	423	203	415.9633333	1.152004921	0.021661358	0.364161986
58810	'Akr1a1'	5028	5754	8254	4694	6283	8612	6345.333333	6529.666667	0.021168101	0.959048718	0.999493374
58859	'Efemp2'	762	804	590	369	1218	1538	718.6666667	1041.666667	0.481361016	0.286654006	0.89430029
58860	'Adamdec1'	2	0	0	2	2	0	0.666666667	1.333333333	1.271241174	0.665607953	0.972790461
58861	'Cysltr1'	41	33	56	6	29	27	43.33333333	20.66666667	-1.165538894	0.080422852	0.617305615
58864	'Tssk3'	15	13	5	2	3	4	11	3	-1.809903582	0.063782219	0.568241586
58865	'Tdh'	84	88	19	37	42	24	63.66666667	34.33333333	-0.712008651	0.302586132	0.905804997
58866	'Treh'	2	0	0	0	1	12	0.666666667	4.333333333	2.600504292	0.325572091	0.920269559
58867	'Syngr4'	186	211	69	7	65	43	155.3333333	38.33333333	-2.051008663	0.004283585	0.152223415
58869	'Pex5l'	70	52	29	1	73	44	50.33333333	39.33333333	-0.448831635	0.660019478	0.972790461
58875	'Hibadh'	1492	1462	1481	554	1486	1765	1478.333333	1268.333333	-0.274454484	0.485451132	0.972790461
58887	'Repin1'	408	421	458	349	501	586	429	478.6666667	0.169509167	0.581660886	0.972790461
58909	'Fam13a'	151	168	51	295	263	251	123.3333333	269.6666667	1.299587843	0.014466566	0.299476615
58911	'Sumf1'	520	528	257	591	763	767	435	707	0.79287341	0.024044159	0.383174818
58916	'Myot'	1	0	2	0	2	18	1	6.666666667	2.479495531	0.232834788	0.859679981
58988	'Rps6kb2'	310	316	247	271	342	304	291	305.6666667	0.145100134	0.63450684	0.972790461
58991	'Ghrl'	0	1	0	2	0	0	0.333333333	0.666666667	1.337350669	0.73871782	0.972790461
58992	'F12'	1	3	0	0	0	0	1.333333333	0	-2.692051488	0.498596374	0.972790461
58994	'Smpd3'	170	167	41	53	252	239	126	181.3333333	0.518684063	0.436151657	0.965982602
58996	'Arhgap23'	1381	1258	2116	2244	1493	1625	1585	1787.33	0.274503539	0.607979228	0.972790461
58998	'Nectin3'	947	962	661	686	1215	1266	856.6666667	1055.666667	0.328840758	0.20190373	0.82996621
59001	'Pole3'	150	184	137	109	161	191	157	153.6666667	-0.001476034	0.99613504	0.999616639
59002	'Wrap73'	510	538	155	218	382	446	401	348.6666667	-0.136230873	0.782008621	0.972790461
59003	'Maea'	2443	2627	3410	4052	2531	3646	2826.666667	3409.666667	0.369547293	0.448827003	0.97049895
59004	'Pias4'	469	543	678	449	396	540	563.3333333	461.6666667	-0.236687683	0.574525079	0.972790461
59005	'Trappc2l'	246	278	230	161	228	298	251.3333333	229	-0.112409726	0.701947326	0.972790461
59007	'Ngly1'	865	892	881	404	840	830	879.3333333	691.3333333	-0.36051726	0.239784107	0.86449675
59008	'Anapc5'	7096	6895	3429	2715	7046	6697	5806.666667	5486	-0.069996296	0.859360239	0.976863639
59009	'Sh3rf1'	414	375	732	379	522	457	507	452.6666667	-0.166228382	0.730308168	0.972790461
59010	'Sqor'	118	129	215	222	216	236	154	224.6666667	0.580493602	0.242180922	0.86449675
59011	'Myoz1'	9	3	10	9	3	4	7.333333333	5.333333333	-0.305808849	0.782302956	0.972790461
59012	'Moxd1'	20	7	8	35	9	10	11.66666667	18	0.921257893	0.351797538	0.929586489
59013	'Hnrnph1'	13418	14524	6834	10306	13865	9280	11592	11150.33333	0.062976164	0.876209601	0.981341203
59014	'Rrs1'	977	919	986	1463	1249	1413	960.6666667	1375	0.610580285	0.113524408	0.698948431
59015	'Nup160'	1489	1619	1258	1108	1357	1296	1455.333333	1253.666667	-0.142521454	0.6111938	0.972790461
59016	'Thap11'	769	785	1154	787	763	1125	902.6666667	891.6666667	0.002654399	0.99505263	0.999562152
59020	'Pdzk1'	14.41	12.33	10.67	3.66	155.01	564.46	12.47	241.0433333	4.162021176	3.52E-04	0.03396508
59021	'Rab2a'	3088	3149.5	4800	3610	3263	4060	3679.166667	3644.333333	0.030650759	0.9452111	0.996592403
59022	'Edf1'	600	718	1661	578	720	1033	993	777	-0.409569806	0.476674601	0.972790461
59024	'Med12'	2843	2486	957	650	2771	1455	2095.333333	1625.333333	-0.353645398	0.527827944	0.972790461

59025	'Usp14'	2281.5	2329.1	2156.4	826.85	2247.7	2826.7	2255.68	1967.066667	-0.251216503	0.530389918	0.972790461
59026	'Huwe1'	22035	20402	5048	7255	17564	16436	15828.33333	13751.66667	-0.150123308	0.786013454	0.972790461
59027	'Nampt'	1901	1680	11723	1430	1222	2056	5101.333333	1569.333333	-1.786082228	0.036511985	0.457518227
59028	'Rcl1'	423	467	833	228	402	405	574.3333333	345	-0.781316884	0.10231765	0.671776916
59029	'Psmd14'	1601	1624	1630	674	1638	1802	1618.333333	1371.333333	-0.278051319	0.43334817	0.964958468
59030	'Mkks'	584.81	650	716.98	298	460	506	650.5966667	421.3333333	-0.622250309	0.052848418	0.532526438
59031	'Chst12'	165	141	61	338	162	219	122.3333333	239.6666667	1.196731109	0.0474851	0.50519682
59032	'Ppp2r3c'	686.04	765.09	865.08	199.3	604.89	490.75	772.07	431.6466667	-0.894631713	0.033418931	0.440916524
59033	'Slc4a8'	159	146	96	159	164	120	133.6666667	147.6666667	0.274992955	0.528357366	0.972790461
59035	'Carm1'	2313.2	2256.3	2480.4	915.68	2040.8	2139.64	2349.953333	1698.706667	-0.501133057	0.140052471	0.742934591
59036	'Dact1'	262	240	292	70	314	247	264.6666667	210.3333333	-0.4136894	0.421040459	0.958945978
59038	'Pxmp4'	309	325	259	164	267	276	297.6666667	235.6666667	-0.308994856	0.241834756	0.86449675
59040	'Rhot1'	1323	1297	1820	1810	1473	1889	1480	1724	0.286049732	0.516187034	0.972790461
59041	'Stk25'	3561.2	3384.7	2618.7	1979.1	2786	3727.9	3188.19	2830.993333	-0.14154376	0.583479552	0.972790461
59042	'Cope'	835	827	2136	878	744	1064	1266	895.3333333	-0.501754663	0.40222088	0.955996639
59043	'Wsb2'	1577.9	1570.6	3294	2600	1816	2858	2147.496667	2424.666667	0.210483099	0.708610121	0.972790461
59044	'Rnf130'	1694	1776	1055	1737	2687	3260	1508.333333	2561.333333	0.804571717	0.004309638	0.152863505
59045	'Stard3'	463	466	375	496	698	693	434.6666667	629	0.583013526	0.020293707	0.353948215
59046	'Arpp19'	2351	2383	1775	1330	1937	2245	2169.666667	1837.333333	-0.201585242	0.384511346	0.94674509
59047	'Pnkp'	604.39	664.97	542.72	368.48	742.32	613.11	604.0266667	574.6366667	-0.056608498	0.843524508	0.975182082
59048	'C1galt1c1'	160	148	96	608	268	315	134.6666667	397	1.789746831	0.002629719	0.114933824
59049	'Slc22a17'	822.05	908.82	133.49	339.81	1508.5	823.59	621.4533333	890.6233333	0.55700542	0.437765703	0.96622803
59050	'Nsa2'	3796	3511.1	6693.3	4083.4	3025.6	3390.05	4666.79	3499.663333	-0.344625172	0.520981214	0.972790461
59052	'Mettl9'	1299	1256	3973	633	1212	1586	2176	1143.666667	-1.046155619	0.103060676	0.673510422
59053	'Hgh1'	224	261	245	126	235	295	243.3333333	218.6666667	-0.171030196	0.617981435	0.972790461
59054	'Mrps30'	556	512	1009	450	528	702	692.3333333	560	-0.323198132	0.505205837	0.972790461
59056	'Evc'	502	475	68	128	580	441	348.3333333	383	0.159883938	0.828500798	0.973333571
59057	'Zfp24'	1149	1242	899	970	1351	1152	1096.666667	1157.666667	0.146634358	0.5974846	0.972790461
59058	'Bhlhe22'	23	20	48	37	18	15	30.33333333	23.33333333	-0.242782071	0.766557557	0.972790461
59069	'Tpm3'	3466	3665	4061	3900	5397	5360	3730.666667	4885.666667	0.415908949	0.177587835	0.797237291
59079	'Erbin'	3259.8	3368.9	3908	1805.9	3954.7	3611.87	3512.233333	3124.156667	-0.196571897	0.57012732	0.972790461
59083	'Fetub'	8	6	2	12	8	72	5.333333333	30.66666667	2.500864035	0.016160168	0.316838738
59090	'Midn'	1899	1873	4999	1581	2030	2110	2923.666667	1907	-0.652633105	0.261036283	0.878842185
59091	'Jph2'	65	60	22	6	63	31	49	33.33333333	-0.587611457	0.458693153	0.972790461
59092	'Pcbp4'	922	924	1163	988	649	781	1003	806	-0.2018636	0.675845249	0.972790461
59093	'Pcbp3'	410	367	462	275	274	269	413	272.6666667	-0.528890924	0.1845176	0.804842425
59095	'Fxyd6'	747	835	608	1016	1464	1823	730	1434.333333	1.00664934	6.35E-05	0.009744897
59125	'Nek7'	1792	1823	1803	1980	1691	1953	1806	1874.666667	0.148582744	0.691806332	0.972790461
59126	'Nek6'	555	576	1380	636	574	617	837	609	-0.438922053	0.455830354	0.971239019
59287	'Ncstn'	1727	1797	842	978	2684	2661	1455.333333	2107.666667	0.539660962	0.195356096	0.820253997

59288	'Dctn5'	990	1040	1111	540	912	926	1047	792.6666667	-0.399162501	0.180441025	0.801341923
59289	'Ackr2'	20	24	7	3	5	3	17	3.666666667	-2.114669858	0.018149116	0.336230526
59290	'Gpa33'	2	2	0	0	3	1	1.333333333	1.333333333	-0.005352462	0.998141948	0.999900097
59308	'Emcn'	132	146	44	58	236	323	107.3333333	205.6666667	0.913136067	0.149473432	0.758505335
594844	'Tceal3'	130.53	119.85	96.41	53.28	71.37	93.85	115.5966667	72.83333333	-0.632460572	0.06249353	0.566066012
595136	'Ndufs5'	693	767	649	561	480	773	703	604.6666667	-0.147341572	0.679187537	0.972790461
60315	'Myg1'	375	401	450	332	445	538	408.6666667	438.3333333	0.115011274	0.725020924	0.972790461
60321	'Wbp11'	2420	2464	2409	1046	1953	2669	2431	1889.333333	-0.388803664	0.238733553	0.864053647
60322	'Chst7'	29	32	4	7	54	26	21.66666667	29	0.430365824	0.643028924	0.972790461
60344	'Fign'	39	26	21	5	23	16	28.66666667	14.66666667	-0.985482668	0.137934601	0.739360306
60345	'Nrip2'	154.66	170.85	306.8	54.36	141.09	249.4	210.77	148.2833333	-0.630205151	0.295833979	0.90029104
60363	'Cldn15'	165	130	9	479	156	174	101.3333333	269.6666667	1.767729893	0.068210859	0.583566634
60364	'Donson'	303	326	135	209	318	260	254.6666667	262.3333333	0.134968028	0.738775114	0.972790461
60365	'Rbm8a'	1772	1938	2077	712	1453	1807	1929	1324	-0.580191245	0.101644066	0.671759705
60367	'Il1rapl2'	0	2	0	1	0	0	0.666666667	0.333333333	-0.733814424	0.856146772	0.975734242
60406	'Sap30'	251	260	2700	581	194	374	1070.333333	383	-1.456368915	0.162703494	0.774103809
60409	'Trappc4'	583	609	954	249	492	636	715.3333333	459	-0.705305668	0.119177493	0.70917136
60411	'Cenpk'	198.14	260.21	61.09	4	150.09	157.05	173.1466667	103.7133333	-0.813896788	0.406456587	0.957157474
60425	'Doc2g'	42	53	49	28	74	23	48	41.66666667	-0.183437056	0.763143955	0.972790461
60440	'Iigp1'	245	203	119	26	73	181	189	93.33333333	-1.075382265	0.085074179	0.634037748
60441	'Mrpl38'	883	864	1036	1224	789	1051	927.6666667	1021.333333	0.250344879	0.59257954	0.972790461
60455	'Pgap6'	352	322	103	70	280	445	259	265	0.002222401	0.997269409	0.999723781
60504	'Il21r'	7	3	0	0	3	5	3.333333333	2.666666667	-0.357157354	0.851910786	0.975734242
60505	'Il21'	0	0	0	1	2	1	0	1.333333333	2.898671682	0.359820271	0.933706718
60507	'Qtrt1'	178	164	344	71	166	197	228.6666667	144.6666667	-0.754170869	0.167522445	0.781678718
60510	'Syt9'	460	382	595	34	175	382	479	197	-1.439175687	0.042917711	0.483384062
60525	'Acss2'	1127	1059	431	681	680	585	872.3333333	648.6666667	-0.272735978	0.557471332	0.972790461
60527	'Fads3'	216	203	506	575	275	323	308.3333333	391	0.456084614	0.511973548	0.972790461
60530	'Fignl1'	1012	1072	343	79	649	574	809	434	-0.943723718	0.174606643	0.793295795
60532	'Wtap'	2201	2241	2167	1673	1954	2014	2203	1880.333333	-0.169020903	0.568774526	0.972790461
60533	'Cd274'	81	91	119	98	71	68	97	79	-0.189207028	0.727169475	0.972790461
60534	'Fancg'	365	292	108	153	292	292	255	245.6666667	0.007492211	0.987472845	0.999493374
60594	'Capn12'	8.11	8.1	3.1	5.05	8.09	1.01	6.436666667	4.716666667	-0.292793961	0.799364995	0.972790461
60595	'Actn4'	4266.9	4107.9	4089.9	12372	4478.9	6848.99	4154.896667	7899.95	1.133338436	0.059397299	0.553094905
60596	'Gucy1a1'	3453	3433	2371	308	3389	2622	3085.666667	2106.333333	-0.645231271	0.344956253	0.926100052
60597	'Mapk8ip2'	49	35	18	6	29	31	34	22	-0.652803765	0.344113886	0.925308103
60599	'Trp53inp1'	1890	2094	3180	1576	2456	2349	2388	2127	-0.180702772	0.664287628	0.972790461
60611	'Foxj2'	1173	1093	606	612	665	957	957.3333333	744.6666667	-0.282513011	0.418016695	0.95774083
60613	'Kcnq4'	98	70	93	36	104	87	87	75.66666667	-0.240779689	0.603565249	0.972790461
606496	'Gsk3a'	1445.6	1530.6	1570.9	1447.6	1431.1	1432.2	1515.686667	1436.956667	0.004190458	0.990749867	0.999493374

613262	'BC029722'	277.51	307.65	341.75	166.85	347.31	436.83	308.97	316.9966667	-0.004797103	0.990036778	0.999493374
613264	'1810020O05Rik'	6	8	4	7	4	2	6	4.333333333	-0.264612084	0.808462304	0.972790461
619287	'Rtl4'	81	107	69	24	122	116	85.66666667	87.33333333	-0.033425751	0.952939496	0.999032683
619288	'Fam71a'	3	0	2	1	1	0	1.666666667	0.666666667	-1.195280337	0.617303295	0.972790461
619289	'Rfx8'	19	26	2	1	13	17	15.66666667	10.33333333	-0.623996787	0.588405225	0.972790461
619297	'Ccdc194'	14	14	41	5	11	7	23	7.666666667	-1.65218481	0.055261469	0.539316735
619301	'Tmem253'	114.03	102.07	11	21.33	77.49	28	75.7	42.27333333	-0.769429088	0.369075253	0.937712122
619309	'Muc3a'	2	2	5	2	2	1	3	1.666666667	-0.809717996	0.577244909	0.972790461
619310	'Zfp872'	24	23	12	47	68	68	19.66666667	61	1.698226109	2.74E-04	0.028291035
619326	'9130409I23Rik'	0	2	1	1	0	0	1	0.333333333	-1.423662873	0.659954786	0.972790461
619331	'Zfp551'	341	287	156	93	354	321	261.3333333	256	-0.049555583	0.920244776	0.990979973
619332	'Taf7l2'	516	455	83	8	221	285	351.3333333	171.3333333	-1.101353688	0.267127961	0.883600995
619441	'Tnfsfm13'	82.89	114.94	87.38	42.27	113.49	145.04	95.07	100.2666667	0.04187647	0.927245692	0.992812524
619547	'Rpl34-ps1'	4327.6	4369.1	6487.9	2.59	0.25	2.16	5061.55	1.666666667	-11.87616402	7.70E-42	1.46E-37
619548	'Defb42'	1	5	3	66	6	8	3	26.66666667	3.534693135	0.006893166	0.196776081
619597	'Gal3st2c'	35.87	35	5	4	19.71	26	25.29	16.57	-0.614128708	0.499415664	0.972790461
619605	'Zcchc17'	556	635	440	485	537	597	543.6666667	539.6666667	0.069417597	0.816013826	0.972790461
619665	'Klf14'	37	42	7	10	18	5	28.66666667	11	-1.230034084	0.168654788	0.784384366
619883	'Gm6109'	1	2	0	2	2	1	1	1.666666667	0.924874406	0.632808025	0.972790461
619941	'Gm13770'	7	7	4	1	7	1	6	3	-0.991898939	0.425298174	0.960285372
620078	'C130026I21Rik'	0	0	2.41	7.59	13	52.22	0.803333333	24.27	4.994153766	0.001013045	0.066185633
620235	'Siglec15'	6	3	0	0	1	4	3	1.666666667	-0.882239156	0.670347634	0.972790461
620246	'Gpr52'	10	13	2	10	11	11	8.333333333	10.66666667	0.490119042	0.582083964	0.972790461
620253	'Dcpp3'	0	1	0	0	0	2	0.333333333	0.666666667	0.78767502	0.845746136	0.975182082
620292	'Cntnap5c'	6	0	1	219	9	5	2.333333333	77.66666667	5.554001358	6.70E-04	0.051978805
620393	'Alkal1'	0	0	1	1	0	0	0.333333333	0.333333333	0.058500858	0.988561293	0.999493374
620419	'Zfp963'	209	190	105	46	217	131	168	131.3333333	-0.373648521	0.487854566	0.972790461
620499	'Gm6158'	0	0	0	0	1.37	0	0	0.456666667	1.020273531	0.802557913	0.972790461
620592	'Tmem28'	274	266	47	0	82	34	195.6666667	38.66666667	-2.392742237	0.047687005	0.506757794
620631	'Ttc30a2'	39.28	38.21	8.81	14.37	23.46	24.94	28.76666667	20.92333333	-0.389977428	0.578879364	0.972790461
620672	'Vmn2r67'	0	0	0	0	0	1	0	0.333333333	1.020273531	0.802557913	0.972790461
620695	'Gm13889'	29	27	482	1645	46	97	179.3333333	596	1.987985553	0.176213251	0.796060628
620913	'Gm12185'	3.05	5.01	6	4	4	12	4.686666667	6.666666667	0.481554362	0.640630108	0.972790461
621080	'AI429214'	158	137	213	112	112	117	169.3333333	113.6666667	-0.527170725	0.248423465	0.869491261
621239	'Nhlrc4'	4	3	0	0	3	2	2.333333333	1.666666667	-0.495686415	0.807967173	0.972790461
621580	'Gm21953'	7.12	0	0	0	2	0	2.373333333	0.666666667	-1.764519343	0.624772804	0.972790461
621603	'Aldh3b2'	2.87	3.99	0	0	2	0	2.286666667	0.666666667	-1.267787067	0.66163929	0.972790461
621628	'Cldn20'	12	7	1	0	5	1	6.666666667	2	-1.733643492	0.278136827	0.889355605
621697	'Rpl32l'	92	103	237	161	84	117	144	120.6666667	-0.173997115	0.798732109	0.972790461
621823	'Psme2b'	2.98	4.58	0	5.59	1.92	0	2.52	2.503333333	0.453719495	0.834785825	0.974723675

621852	'Rhox3f'	6.45	8.67	0	0	0	2.08	5.04	0.693333333	-2.783825176	0.24898261	0.86963876
621893	'H2ac21'	0	1	0	0	0	0	0.333333333	0	-0.903279821	0.824807108	0.972790461
621976	'Tmem170b'	602	615	1006	752	469	460	741	560.3333333	-0.284715564	0.61901795	0.972790461
622032	'Vmn1r167'	0	0	0	0	0	1	0	0.333333333	1.020273531	0.802557913	0.972790461
622098	'Gm6285'	0	0	0	0	0	1	0	0.333333333	1.020273531	0.802557913	0.972790461
622117	'Triml2'	0	2	1	1	0	0	1	0.333333333	-1.423662873	0.659954786	0.972790461
622127	'Cyp3a57'	0	0	1	0	0	0	0.333333333	0	-0.903279821	0.824807108	0.972790461
622301	'Rhox2h'	2	1	0	0	1	2	1	1	-0.029662059	0.990499881	0.999493374
622307	'Albfm1'	2	1	0	0	3	3	1	2	0.959652711	0.665449559	0.972790461
622320	'Kctd21'	147	177	150	134	161	170	158	155	0.028161504	0.93168313	0.993601701
622335	'Gm6314'	8.97	6.23	19.21	0	0	0	11.47	0	-5.927914522	9.27E-04	0.063607704
622402	'Akr1c12'	0	0	1	0	4	0	0.333333333	1.333333333	1.792572865	0.652588671	0.972790461
622404	'Ccdc107'	181	185	171	199	247	316	179	254	0.54044832	0.07351225	0.599577391
622408	'Mcidas'	2	1	0	1	0	0	1	0.333333333	-1.330404752	0.683404215	0.972790461
622434	'Arhgef26'	277	302	98	81	332	415	225.6666667	276	0.267282665	0.657046751	0.972790461
622473	'Ripply1'	1	0	0	0	0	0	0.333333333	0	-0.903279821	0.824807108	0.972790461
622480	'Spocd1'	3	0	2	3	2	3	1.666666667	2.666666667	0.75120619	0.644869831	0.972790461
622554	'Majin'	255	256	128	1	125	53	213	59.66666667	-1.929450371	0.062175701	0.565368139
622645	'Tmem200c'	3	2	0	7	16	9	1.666666667	10.66666667	2.756579477	0.021282598	0.361918385
622665	'Ccdc17'	15	24	7	2	25	9	15.33333333	12	-0.37690596	0.697864121	0.972790461
622675	'Zfp827'	1328	1311	1067	213	1439	1008	1235.333333	886.6666667	-0.557452657	0.329141311	0.921648675
622699	'Gm12569'	31.24	33	5	0	15	32	23.08	15.66666667	-0.627778214	0.618480167	0.972790461
622976	'Gm6377'	6	9	9	10	2	4	8	5.333333333	-0.377550031	0.727419104	0.972790461
623046	'Fscb'	5	6	0	0	4	0	3.666666667	1.333333333	-1.44024112	0.560651212	0.972790461
623078	'Gm8108'	48.34	36.41	2.04	3	25.4	12.73	28.93	13.71	-1.079278702	0.330030524	0.921648675
623121	'Ifi213'	0	5.02	1.34	1	3	1.97	2.12	1.99	-0.198889278	0.91691525	0.990536123
623131	'Prr19'	132	133	93	7	52	146	119.3333333	68.33333333	-0.926802916	0.257845318	0.877405867
623166	'Prr23a1'	9	5	3	1	6	5.65	5.666666667	4.216666667	-0.515417928	0.646373809	0.972790461
623186	'Prr23a2'	1	3	2	0	0	1	2	0.333333333	-2.425913442	0.300301308	0.904081584
623230	'Tmem200b'	99.88	82.88	76.35	57.05	87.62	110.41	86.37	85.02666667	0.002211288	0.995003917	0.999562152
623279	'Dok6'	139	139	406	31	226	179	228	145.3333333	-0.837873305	0.280306844	0.889355605
623286	'Ndufs6b'	0.33	0	0	0	0	2	0.11	0.666666667	1.749449488	0.66566965	0.972790461
623459	'Faiml'	0	1	0	0	0	0	0.333333333	0	-0.903279821	0.824807108	0.972790461
623474	'Rad54b'	393.49	365.91	101.91	76	308.73	292.96	287.1033333	225.8966667	-0.343699942	0.576031361	0.972790461
623661	'Lipt1'	147	127	216	67	126	179	163.3333333	124	-0.460168896	0.339696649	0.925008117
623734	'Vmn2r85'	0	2	0	4	2	0	0.666666667	2	1.960767827	0.474708066	0.972790461
623781	'Gm14137'	30	34	7	18	24	20	23.66666667	20.66666667	-0.070031408	0.92120278	0.991066749
623898	'Gm6460'	0	0	0	1	0	0	0	0.333333333	1.020273531	0.802557913	0.972790461
624049	'Gm6468'	0	0	0	0	0	1	0	0.333333333	1.020273531	0.802557913	0.972790461
624219	'Angptl8'	2	0	0	2	0	2	0.666666667	1.333333333	1.243078519	0.673077728	0.972790461

624224	'Clrn2'	0	0	1	0	0	0	0.333333333	0	-0.903279821	0.824807108	0.972790461
624245	'Speer4e'	0	0	0	57.51	0	5.17	0	20.89333333	7.251981333	0.002944353	0.121291461
624681	'Btnl6'	1	0	1	1	0	0	0.666666667	0.333333333	-0.892941112	0.796499214	0.972790461
624855	'Cks1brt'	0	0	0	0	1	0	0	0.333333333	1.020273531	0.802557913	0.972790461
624866	'Lekr1'	230	274	121	73	192	150	208.3333333	138.3333333	-0.56431341	0.189751558	0.812878913
624931	'LOC624931'	1	0	0	0	0	0	0.333333333	0	-0.903279821	0.824807108	0.972790461
625029	'Vmn2r83'	1	0	0	0	0	1	0.333333333	0.333333333	0.058500858	0.988561293	0.999493374
625068	'Vmn2r84'	2.11	2	0	20	1	0	1.37	7	2.927214439	0.165254636	0.779801723
625098	'Slc38a6'	1404.1	1248.4	811.93	854.9	1937.4	1013.33	1154.82	1268.526667	0.18497046	0.643723524	0.972790461
625109	'Vmn2r86'	0	0	0	0	1.25	1	0	0.75	1.775692139	0.660844521	0.972790461
625131	'Vmn2r87'	3.89	1	0	3	8.75	2	1.63	4.583333333	1.803838929	0.263370953	0.88013169
625249	'Gpx4'	1810	1815	4786	3644	1962	3185	2803.666667	2930.333333	0.118196622	0.85816174	0.976475731
625286	'Tmem236'	7	6	11	3	5	3	8	3.666666667	-1.11924438	0.259413568	0.877955237
625328	'H3f3c'	26.59	18.06	25	12.16	23.65	23.79	23.21666667	19.86666667	-0.255430109	0.654607516	0.972790461
625360	'BC147527'	8	14	0	0	3	2	7.333333333	1.666666667	-2.126879604	0.241390576	0.86449675
625424	'Gm6583'	4	1	0	5	1	0	1.666666667	2	0.710945127	0.753109109	0.972790461
625464	'Gm6588'	0	1	1	4	2	0	0.666666667	2	1.823542756	0.433074256	0.964631919
625530	'Usp17le'	11.96	8.81	2	0	3	20	7.59	7.666666667	0.038227631	0.979298811	0.999493374
625591	'Cldn34c2'	0	1.76	0	1	1.23	2.6	0.586666667	1.61	1.921402722	0.452416357	0.970649024
625599	'Gml'	13	17	5	0	10	18.58	11.66666667	9.526666667	-0.407546043	0.736347748	0.972790461
625638	'Fam43b'	1	4	19	40	3	13	8	18.66666667	1.394566019	0.310522089	0.910971113
625662	'Ankrd31'	1152	1122	233	1	599	393	835.6666667	331	-1.406422741	0.263129447	0.88013169
625716	'Gm6614'	0	1	0	0	0	0	0.333333333	0	-0.903279821	0.824807108	0.972790461
625801	'Gm6625'	0	0	1	0	0	0	0.333333333	0	-0.903279821	0.824807108	0.972790461
626053	'Gm6647'	0	1	0	0	0	0	0.333333333	0	-0.903279821	0.824807108	0.972790461
626215	'Gm6657'	0	0	0	0	0	1	0	0.333333333	1.020273531	0.802557913	0.972790461
626316	'Zfp987'	79.86	78.98	96.63	19.94	74.36	37.31	85.15666667	43.87	-1.007364942	0.069599623	0.587499452
626359	'Wdr93'	161	157	25	3	78	122	114.3333333	67.66666667	-0.821448703	0.424268312	0.960285372
626391	'Zfp951'	194.81	188.38	125.14	51	242.42	172.99	169.4433333	155.47	-0.163910748	0.750035563	0.972790461
626415	'4930467E23Rik'	304.03	356.32	157.44	150.49	239.08	84.97	272.5966667	158.18	-0.657683901	0.234434319	0.861206181
626578	'Gbp10'	142.68	128.4	383.59	0	2.41	10.86	218.2233333	4.423333333	-5.962656383	1.62E-09	1.36E-06
626596	'Rgs22'	114	115	23	7	111	47	84	55	-0.636891977	0.48020744	0.972790461
626615	'Apol11a'	10	0	0	0	0	0	3.333333333	0	-4.022069678	0.306764117	0.907830798
626802	'Gm14322'	356.88	352.79	72.53	59.74	219.99	107.56	260.7333333	129.0966667	-0.967157695	0.148726501	0.757633614
626832	'Gm6710'	210.25	216.22	142.43	57.27	201.03	127.63	189.6333333	128.6433333	-0.570590603	0.204505274	0.833273525
626834	'Klk13'	0	1	4	10	0	0	1.666666667	3.333333333	1.325583992	0.616433482	0.972790461
626848	'Zfp971'	663.5	616.2	243.7	245.59	634.42	533.58	507.8	471.1966667	-0.074808639	0.870085501	0.979794316
626854	'Gm38396'	216.04	228.46	250.24	162.06	366.83	176.01	231.58	234.9666667	0.032592721	0.941663205	0.996210609
626858	'Gm6713'	15.9	10.39	2.45	4.45	11.86	2.38	9.58	6.23	-0.555966088	0.624447016	0.972790461
626870	'Gm11992'	17	16	8	47	9	11	13.66666667	22.33333333	1.05126709	0.285074074	0.893711786

626940	'Gm6721'	8.3	9.84	2.61	1.06	2.02	3	6.916666667	2.026666667	-1.608404564	0.193945305	0.817889904
626942	'Vmn2r90'	1	0	0	0	0	0	0.333333333	0	-0.903279821	0.824807108	0.972790461
627009	'Pramel15'	0	0	0	0	0	3	0	1	2.274429265	0.570697589	0.972790461
627035	'Gm6729'	4.3	3.63	0	0	4.14	3.01	2.643333333	2.383333333	-0.021707428	0.991179608	0.999493374
627049	'Zfp800'	794	768	643	608	698	625	735	643.6666667	-0.106160611	0.737726992	0.972790461
627081	'Xlr5b'	130.4	139.42	43.38	0	73.3	224.55	104.4	99.28333333	-0.184610267	0.886678705	0.984461626
627191	'Syndig1l'	2	1	51	0	1	0	18	0.333333333	-5.793895261	0.005620618	0.177212597
627214	'Insyn2a'	7	9	4	1	8	20	6.666666667	9.666666667	0.453018619	0.67850537	0.972790461
627280	'Vmn1r90'	14	19	8	2	21	7	13.66666667	10	-0.476504977	0.614054339	0.972790461
627302	'Gm14092'	2	0	0	0	1	0	0.666666667	0.333333333	-0.74959284	0.853034393	0.975734242
627352	'Morf4l1b'	1387.5	1441.9	3073	700.56	761.86	1528.78	1967.453333	997.0666667	-1.039311059	0.062014562	0.564935721
627375	'Gm6750'	1144.4	1324.9	946.36	21.8	400.62	205.87	1138.533333	209.43	-2.551410578	0.001047781	0.067756508
627569	'Vmn2r12'	1	3	1	1	4	4	1.666666667	3	0.836061907	0.572082483	0.972790461
627626	'Ptchd4'	27	32	22	3	29	13	27	15	-0.90221211	0.249649063	0.869928149
627821	'Epp13'	1	4	1	1	8	12	2	7	1.745039924	0.200722984	0.828336309
627872	'Dnah7a'	46.14	37.5	5.99	0	8.25	8.24	29.87666667	5.496666667	-2.486173275	0.032338954	0.434162511
627927	'Gm6812'	0	0	0	0	0	1	0	0.333333333	1.020273531	0.802557913	0.972790461
628040	'Gm6829'	1.05	1.04	0	0	1.02	1.02	0.696666667	0.68	0.015019958	0.995726078	0.999562152
628100	'Fbxo39'	0	0	0	0	0	2	0	0.666666667	1.749449488	0.66566965	0.972790461
628147	'Gm2004'	270.52	263.62	87.96	109.93	292.27	248.31	207.3666667	216.8366667	0.102207727	0.840294023	0.974780034
628308	'Zfp970'	1425.2	1476.1	771.81	675.11	1393.2	829.13	1224.343333	965.8233333	-0.279054282	0.471298386	0.972790461
628422	'Vmn2r58'	0	0	0	0	1	1	0	0.666666667	1.775692139	0.660844521	0.972790461
628456	'Ctag2l1'	175.12	158	63	1	83.01	319	132.04	134.3366667	-0.097742623	0.934280407	0.994264713
628518	'Ctag2l2'	39.88	47	14	0	19.99	103	33.62666667	40.99666667	0.168511208	0.897767613	0.985794067
628705	'Phf11c'	33.26	36.95	0	12.61	50.62	5.58	23.40333333	22.93666667	0.072566192	0.957959743	0.999493374
628709	'Gm10324'	38.08	36.31	6.12	0	20.38	70.77	26.83666667	30.38333333	0.080208503	0.951812207	0.998640168
628746	'Rybp-ps'	6.86	14.04	16.7	37.88	131.56	86.46	12.53333333	85.3	2.754557582	2.82E-05	0.005524122
628779	'Hs3st4'	13	13	2	0	7	5	9.333333333	4	-1.250638969	0.342482426	0.925308103
628813	'Gm11437'	3	0	0	0	0	0	1	0	-2.292035055	0.566710385	0.972790461
628870	'Otogl'	3	0	1	1	0	1	1.333333333	0.666666667	-0.851775982	0.735237413	0.972790461
628893	'Cldn34-ps'	1	0	0	0	0	0	0.333333333	0	-0.903279821	0.824807108	0.972790461
628900	'Serpina3i'	74.9	95.69	596.63	69.41	11.06	17.72	255.74	32.73	-2.814117072	0.011103469	0.260294893
628946	'Vmn1r77'	0	0	0	0	0	1	0	0.333333333	1.020273531	0.802557913	0.972790461
629016	'Zfp953'	126.36	122.55	96	81	175.52	224	114.97	160.1733333	0.466081259	0.213202431	0.841304405
629059	'Fam124a'	218	219	79	27	391	342	172	253.3333333	0.489394284	0.539893314	0.972790461
629114	'Defb23'	9	2	1	0	3	2	4	1.666666667	-1.285349617	0.445971206	0.969915971
629147	'Ctxn3'	71	74	4	2	14	10	49.66666667	8.666666667	-2.488566068	0.018187181	0.336230526
629203	'Sult2a3'	0	1	0	0	1	1	0.333333333	0.666666667	0.817382893	0.82412705	0.972790461
629242	'Gm6958'	421.99	552	422	705.93	629.17	376	465.33	570.3666667	0.445398852	0.370863355	0.9384981
629303	'Heatr9'	0	0	7	0	0	0	2.333333333	0	-3.821200639	0.332073658	0.921648675

629378	'Dact3'	391	379	291	790	387	354	353.6666667	510.3333333	0.752390932	0.197793163	0.823747311
629499	'Mroh8'	20.94	13.88	4	1	8	9.14	12.94	6.046666667	-1.054228816	0.304958808	0.907188578
629595	'Gm6988'	1056.5	1252.8	2318.5	1592.4	1171.3	1377.14	1542.576667	1380.27	-0.100172027	0.858239849	0.976475731
629754	'Wfdc9'	2.22	0	0	0	1.03	0	0.74	0.343333333	-0.74959284	0.853034393	0.975734242
629756	'Wfdc10'	26.78	33	0	1	80.97	41	19.92666667	40.99	1.022805728	0.492712212	0.972790461
630077	'LOC630077'	102.96	138.9	64.84	6.03	51.05	31	102.2333333	29.36	-1.831148153	0.006524205	0.190952592
630146	'Cd101'	0	3	1	4	1	2	1.333333333	2.333333333	1.030883641	0.573664865	0.972790461
630294	'Gm7030'	0	0.44	0	1	0.99	0	0.146666667	0.663333333	1.020273531	0.802557913	0.972790461
630537	'Dcpp2'	1	0	0	0	1	0	0.333333333	0.333333333	0.058500858	0.988561293	0.999493374
630579	'Zfp808'	534.73	531	133.71	60.93	483.28	272.92	399.8133333	272.3766667	-0.572584271	0.430150071	0.962548446
630836	'2010315B03Rik'	252	258	103	205	312	194	204.3333333	237	0.32766336	0.478959986	0.972790461
630994	'Lce3d'	0	0	0	0	1	0	0	0.333333333	1.020273531	0.802557913	0.972790461
631145	'Fam90a1b'	231	212	75	3	88	118	172.6666667	69.66666667	-1.396967734	0.135951301	0.736689633
631304	'Cyp4f40'	4	1	0	0	0	0	1.666666667	0	-3.021765648	0.445848131	0.969915971
631323	'Gm12250'	14	29	7	2	6	6	16.66666667	4.666666667	-1.798339703	0.04758736	0.506001612
631415	'Gm20760'	69.76	98.58	12.3	11.01	140.53	72.46	60.21333333	74.66666667	0.301152136	0.74311758	0.972790461
631584	'Catspere1'	5.05	9	6	1	2	5	6.683333333	2.666666667	-1.34677525	0.233031884	0.859679981
631624	'Gm7072'	610.09	639.79	553.78	315.95	772	513	601.22	533.65	-0.168872071	0.628007473	0.972790461
631784	'Gm7073'	0	1	0	0	0	0	0.333333333	0	-0.903279821	0.824807108	0.972790461
631797	'Fer1l6'	38	35	13	4	24	6	28.66666667	11.33333333	-1.316110917	0.121657624	0.712603747
632126	'Btnl4'	1	1	0	0	0	0	0.666666667	0	-1.703500596	0.673820112	0.972790461
632534	'Vmn1r191'	0	0	0	0	1	0	0	0.333333333	1.020273531	0.802557913	0.972790461
632671	'Vmn2r18'	1	1	3	5	0	5	1.666666667	3.333333333	1.091096719	0.532849397	0.972790461
632687	'Marchf10'	3	2	1	0	4	8	2	4	0.906068277	0.574229791	0.972790461
632708	'Dmrtc1b'	1	0	0	0	0	0	0.333333333	0	-0.903279821	0.824807108	0.972790461
632778	'Erich4'	0	1	0	1	0	0	0.333333333	0.333333333	0.058500858	0.988561293	0.999493374
632971	'Rergl'	0	0	0	0	0	1	0	0.333333333	1.020273531	0.802557913	0.972790461
633057	'Gm7102'	21.3	22.24	4.1	10.31	29.15	14.77	15.88	18.07666667	0.246597685	0.764428242	0.972790461
633285	'Rbm46'	717	726	287	118	348	395	576.6666667	287	-1.00562506	0.03982976	0.466282885
633640	'Tmem267'	315	278	162	211	267	254	251.6666667	244	0.046556417	0.894515668	0.985259027
633979	'Ak9'	84	60	8	1	64	84	50.66666667	49.66666667	-0.091197348	0.938053174	0.994698142
634104	'Olfr287'	0	0	0	0	0	2	0	0.666666667	1.749449488	0.66566965	0.972790461
634346	'Gm7135'	1	0	0	0	0	0	0.333333333	0	-0.903279821	0.824807108	0.972790461
634731	'Susd1'	209	194	44	97	248	187	149	177.3333333	0.313772508	0.600540715	0.972790461
634882	'Itih5l-ps'	8	14	2	0	9	7	8	5.333333333	-0.62761999	0.639182241	0.972790461
635169	'CK137956'	0	0	0	1	0	0	0	0.333333333	1.020273531	0.802557913	0.972790461
635253	'Usp51'	83	80	131	60	76	127	98	87.66666667	-0.191861949	0.697683507	0.972790461
635702	'Naaladl2'	89	79	57	72	89	75	75	78.66666667	0.155015169	0.692073903	0.972790461
635895	'Gm7168'	2	4	1	2	6	1	2.333333333	3	0.441201104	0.761524155	0.972790461
636104	'Cfap47'	86	94	21	21	96	121	67	79.33333333	0.228955564	0.749319294	0.972790461

636741	'Zfp964'	419	412	114	54	272	252	315	192.6666667	-0.721771655	0.262706304	0.88013169
636808	'Cntnap5a'	156	155	8	0	385	260	106.3333333	215	0.968800538	0.515420914	0.972790461
636931	'Trim71'	145	151	8	15	118	343	101.3333333	158.6666667	0.600119074	0.579655489	0.972790461
637004	'Vmn2r3'	0	0	3	0	0	0	1	0	-2.588571306	0.51580235	0.972790461
637053	'Vmn2r4'	1.11	0	0	1	0	1	0.37	0.666666667	1.054214044	0.766400104	0.972790461
637079	'Iqcn'	17	16	1	3	11	1	11.33333333	5	-1.064944448	0.416353413	0.95722888
637277	'Sycp2l'	3	2	0	1	36	100	1.666666667	45.66666667	4.684262875	0.001267087	0.07539735
637515	'Nlrp1b'	5.01	2.99	1	2	3.77	4.06	3	3.276666667	0.229901458	0.862915404	0.977227788
637776	'Zfp977'	64	68	8	20	88	53	46.66666667	53.66666667	0.240976199	0.766961877	0.972790461
637898	'Vmn2r60'	1	0	0	0	0	0	0.333333333	0	-0.903279821	0.824807108	0.972790461
63828	'Fn3k'	28	39	34	40	55	46	33.66666667	47	0.530738816	0.250902187	0.871167957
638532	'Gm7241'	1	0	0	0	0	1	0.333333333	0.333333333	0.058500858	0.988561293	0.999493374
63856	'Taf8'	551	644	604	530	707	519	599.6666667	585.3333333	0.029388285	0.933957612	0.994249017
63857	'Bco1'	2	4	0	24	1	10	2	11.66666667	2.922078511	0.060754284	0.559627259
638580	'Gm7244'	0	1	0	0	1	0	0.333333333	0.333333333	0.058500858	0.988561293	0.999493374
63859	'Impg1'	2	9	0	0	19.52	4	3.666666667	7.84	1.048821586	0.58132816	0.972790461
63872	'Zfp296'	23	22	14	12	12	23	19.66666667	15.66666667	-0.2780758	0.650444298	0.972790461
63873	'Trpv4'	19	20	20	23	18	12	19.66666667	17.66666667	-0.022683141	0.973416801	0.999493374
63913	'Niban1'	1590	1644	3400	1363	1793	3515	2211.333333	2223.666667	-0.069110621	0.899843437	0.98632594
639281	'Gm10382'	14	25	63	64	25	18	34	35.66666667	0.226300005	0.810060763	0.972790461
63953	'Dusp10'	55	48	431	132	46	96	178	91.33333333	-0.942963555	0.350251236	0.92886351
63954	'Rbp7'	3	3	0	0	4	0	2	1.333333333	-0.574576642	0.831309445	0.974324192
63955	'Cables1'	298	234	658	1726	258	469	396.6666667	817.6666667	1.284036323	0.160364216	0.771114735
63958	'Ube4b'	3091	2953	4268	4073	2862	3549	3437.333333	3494.666667	0.114864748	0.810884463	0.972790461
63959	'Slc29a1'	431	382	322	365	625	730	378.3333333	573.3333333	0.613442734	0.025061213	0.391828762
639634	'Aadacl2'	5	2	1	0	3	2	2.666666667	1.666666667	-0.714549553	0.675598345	0.972790461
63985	'Gmfb'	2724.7	2698	2400.9	1683.3	2351	2832.95	2607.87	2289.066667	-0.159443796	0.511994289	0.972790461
63986	'Gmfg'	10	10	2	3	9	9	7.333333333	7	-0.031336544	0.974624585	0.999493374
63993	'Slc5a7'	2	5	6	2	3	6	4.333333333	3.666666667	-0.285963522	0.806954017	0.972790461
64008	'Aqp9'	8	4	1	0	12	1	4.333333333	4.333333333	-0.029202018	0.986343141	0.999493374
64009	'Syne1'	9498.6	9581.8	1967.4	2019.1	13058	7538.94	7015.943333	7538.583333	0.103837086	0.883703224	0.983490353
64010	'Sav1'	1358	1369	1770	707	1077	1155	1499	979.6666667	-0.616425214	0.075600009	0.605310548
64011	'Nrgn'	45	45	4	111	67	48	31.33333333	75.33333333	1.550552841	0.079446788	0.617305615
64050	'Yeats4'	824	857	1099	234	805	655	926.6666667	564.6666667	-0.788251131	0.088208423	0.643048174
64051	'Sv2a'	209	193	12	231	237	190	138	219.3333333	0.873217868	0.304532366	0.907164095
640524	'Sptbn5'	279.49	256.63	17.17	12.44	215.56	113.88	184.43	113.96	-0.714724543	0.489304467	0.972790461
640543	'Tgm7'	0	1	0	0	1	0	0.333333333	0.333333333	0.058500858	0.988561293	0.999493374
64058	'Perp'	32	29	104	85	38	73	55	65.33333333	0.2940903	0.713375867	0.972790461
64059	'Oxct2a'	0	1	1	2	0	0	0.666666667	0.666666667	0.369435326	0.909857284	0.989364653
64074	'Smoc2'	56	43	70	654	73	82	56.33333333	269.6666667	2.607415595	0.009185106	0.234872395

64075	'Smoc1'	1997	2142	141	365	3255	2818	1426.666667	2146	0.579943406	0.531603609	0.972790461
64082	'Popdc2'	15	18	7	89	14	17	13.33333333	40	1.966098809	0.051282985	0.525036139
64085	'Clstn2'	999	833	501	141	1585	820	777.6666667	848.6666667	0.057884976	0.934013941	0.994249017
64095	'Gpr35'	99.84	111.05	226.1	48	127	78.99	145.6633333	84.66333333	-0.855221231	0.14726465	0.754255261
64099	'Parvg'	30	37	19	60	36	43	28.66666667	46.33333333	0.865521484	0.146339804	0.753611197
64103	'Tnmd'	0	1	1	0	0	0	0.666666667	0	-1.851176388	0.646290011	0.972790461
64113	'Moap1'	103.08	134.95	94.64	47.16	105.01	130.32	110.89	94.16333333	-0.245324903	0.53834992	0.972790461
641340	'Nrbf2'	455	461	569	671	400	535	495	535.3333333	0.232716136	0.641336281	0.972790461
64136	'Sdf2l1'	183	159	3790	129	129	1046	1377.333333	434.6666667	-1.965480057	0.116947464	0.705163127
641361	'Pinlyp'	15	4	2	0	4	34	7	12.66666667	0.747711967	0.629843934	0.972790461
641368	'Spint5'	1	0	0	0	0	0	0.333333333	0	-0.903279821	0.824807108	0.972790461
641376	'Tomm40l'	379.72	381.88	187	376.56	478.62	504.58	316.2	453.2533333	0.60964694	0.084651936	0.633124552
64138	'Ctsz'	999	1026	1123	2017	1398	2058	1049.333333	1824.333333	0.893345011	0.037397486	0.459930654
64139	'Ctsm'	1	0	0	0	0	0	0.333333333	0	-0.903279821	0.824807108	0.972790461
64143	'Ralb'	1094	1070	2077	1470	1232	1566	1413.666667	1422.666667	0.044543912	0.931698379	0.993601701
64144	'Mllt1'	1404	1406	630	688	1157	1117	1146.666667	987.3333333	-0.150164993	0.680034088	0.972790461
64176	'Sv2b'	54	56	2	95	108	121	37.33333333	108	1.694773274	0.058797472	0.552301156
64177	'Trpv6'	6	4	2	14	2	10	4	8.666666667	1.346324496	0.248028965	0.869491261
64209	'Herpud1'	472	391	6564	539	644	1620	2475.666667	934.3333333	-1.613470994	0.131779058	0.72897159
64213	'St7'	215	263	248	79	330	332	242	247	-0.050465159	0.920439974	0.991018506
64214	'Rgs18'	1	1	0	0	1	4	0.666666667	1.666666667	1.259479367	0.611247353	0.972790461
64242	'Ngb'	9	6	5	17	8	4	6.666666667	9.666666667	0.780697477	0.441584483	0.968551476
64291	'Osbpl1a'	676.52	706.39	471.73	280.82	778.01	645.45	618.2133333	568.0933333	-0.127335632	0.724744569	0.972790461
64292	'Ptges'	158	159	406	2923	217	866	241	1335.333333	2.721612934	0.006287316	0.187652211
64293	'Stk32b'	7	8	0	0	6	15	5	7	0.431699392	0.805581105	0.972790461
64294	'Itm2c'	3204	3503	1483	9532	5982	6696	2730	7403.333333	1.639977317	0.001461746	0.082561154
64295	'Tmub1'	101	96	97	94	102	125	98	107	0.178774356	0.62017571	0.972790461
64296	'Abhd8'	445	431	305	598	554	627	393.6666667	593	0.699656034	0.046147401	0.50074283
64297	'Gprc5b'	1778	1892	931	6503	3454	4379	1533.666667	4778.666667	1.847471411	4.79E-04	0.042360559
64337	'Gng13'	585	842	29	31	572	596	485.3333333	399.6666667	-0.310541844	0.776121477	0.972790461
64339	'Fndc4'	409	368	223	146	383	377	333.3333333	302	-0.141842018	0.7143067	0.972790461
64340	'Dhx38'	1207	1226	963	960	1330	1298	1132	1196	0.134322444	0.583862714	0.972790461
64378	'Gpr88'	30	21	6	0	47	40	19	29	0.532228614	0.673842572	0.972790461
64379	'Irx6'	1	0	0	0	0	0	0.333333333	0	-0.903279821	0.824807108	0.972790461
64383	'Sirt2'	1119	1159	1081	1424	1212	1462	1119.666667	1366	0.381765192	0.297791945	0.900843503
64384	'Sirt3'	343	371	366	252	375	397	360	341.3333333	-0.056033132	0.846977077	0.975209338
64385	'Cyp4f14'	20	24	4	5	43	23	16	23.66666667	0.559033534	0.545079293	0.972790461
64406	'Sp5'	234	237	162	12	321	272	211	201.6666667	-0.182247853	0.832111422	0.974662707
64424	'Polr1e'	266	312	213	271	283	382	263.6666667	312	0.313433544	0.326640522	0.920734892
64435	'Fcamr'	0	1	0	0	0	0	0.333333333	0	-0.903279821	0.824807108	0.972790461

64436	'Inpp5e'	618.5	642.25	402.52	348.47	752.2	725.16	554.4233333	608.61	0.150527103	0.630311941	0.972790461
64450	'Gpr85'	14	17	18	8	9	11	16.33333333	9.333333333	-0.774570742	0.253239934	0.872051735
64451	'Dip2a'	1420	1443	904	907	1566	1360	1255.666667	1277.666667	0.075087111	0.787434912	0.972790461
64452	'Slc5a4a'	27	51	1	4	144	22	26.33333333	56.66666667	1.103952927	0.401597829	0.955860942
64453	'Zfp280b'	1838	1896	695	894	1591	1841	1476.333333	1442	0.020442701	0.961519316	0.999493374
64454	'Slc5a4b'	0	3	0	2	2	1	1	1.666666667	0.94827528	0.677451022	0.972790461
64540	'Tspan4'	473.85	419.36	692.45	1460.5	511.97	931.93	528.5533333	968.1166667	1.02871886	0.110286806	0.690864172
64580	'Ndst4'	118	114	20	4	169	113	84	95.33333333	0.12858973	0.901211483	0.98672375
64602	'Ireb2'	3556	3646	3640	3178	3717	3419	3614	3438	-0.005810454	0.985552838	0.999493374
64652	'Nisch'	11244	10308	8428	9290	13405	8583	9993.333333	10426	0.142537301	0.681501382	0.972790461
64654	'Fgf23'	0	1	4	3	3	1	1.666666667	2.333333333	0.502926853	0.781228739	0.972790461
64655	'Mrps22'	502	476	386	267	468	684	454.6666667	473	0.050901722	0.877252673	0.981341203
64656	'Mrps23'	644	708	683	737	687	910	678.3333333	778	0.265048631	0.445198941	0.969888068
64657	'Mrps10'	405	441	425	246	429	451	423.6666667	375.3333333	-0.171380157	0.556278714	0.972790461
64658	'Mrps25'	1071.9	1157.2	679.32	416.12	1104.7	1016.54	969.4666667	845.77	-0.197360947	0.587634468	0.972790461
64659	'Mrps14'	499	502	493	272	450	510	498	410.6666667	-0.273029065	0.334250348	0.921648675
64660	'Mrps24'	372	423	344	502	430	605	379.6666667	512.3333333	0.51764158	0.148091498	0.756249141
64661	'Krtdap'	0	2	10	2	1	1	4	1.333333333	-1.584160293	0.392611705	0.952648938
64685	'Nmi'	102	107	122	13	121	79	110.3333333	71	-0.748894043	0.273291111	0.884738928
64697	'Kegl'	0	0	0	1	0	0	0	0.333333333	1.020273531	0.802557913	0.972790461
64704	'Htra2'	497.13	487.8	359.06	458.92	451.39	636.45	447.9966667	515.5866667	0.277882206	0.383534098	0.945630828
64705	'Dpys'	0	1	1	0	0	0	0.666666667	0	-1.851176388	0.646290011	0.972790461
64706	'Scube1'	744	748	228	408	874	255	573.3333333	512.3333333	-0.04193136	0.946631999	0.996592403
64707	'Suv39h2'	379.33	387.42	154.86	86.32	278.5	324.71	307.2033333	229.8433333	-0.4280175	0.405160158	0.957157474
64817	'Svep1'	187	185	29	122	312	266	133.6666667	233.3333333	0.86819559	0.19143621	0.813837363
64833	'Acot10'	1	0	0	0	1	0	0.333333333	0.333333333	0.058500858	0.988561293	0.999493374
64898	'Lpin2'	1675	1531	1553	8656	1301	1993	1586.333333	3983.333333	1.658306468	0.050256392	0.518969204
64899	'Lpin3'	355	359	213	102	277	352	309	243.6666667	-0.363305297	0.394740984	0.953476762
64918	'Bhmt2'	4	9	2	1	11	6	5	6	0.24284327	0.838593259	0.974723675
64929	'Scel'	52	43	15	28	43	25	36.66666667	32	-0.077297197	0.900729516	0.98672375
64930	'Tsc1'	2246	2177	1237	1104	1769	1449	1886.666667	1440.666667	-0.317612452	0.316026302	0.914806078
64931	'Izumo1r'	1	0	0	0	4	20	0.333333333	8	4.310717816	0.065231421	0.573626167
64933	'Ap3m2'	191	208	95	58	182	171	164.6666667	137	-0.266222688	0.57002369	0.972790461
64934	'Pes1'	2056	1964	2636	962	1653	2243	2218.666667	1619.333333	-0.492104794	0.195267295	0.82024344
64945	'Cldn12'	636	686	505	645	688	655	609	662.6666667	0.218549987	0.504419717	0.972790461
65019	'Rpl23'	12397	12605	24435	16344	8668	12210	16479	12407.33333	-0.312698946	0.603124615	0.972790461
65020	'Zfp110'	672	688	462	406	745	951	607.3333333	700.6666667	0.215545916	0.482031939	0.972790461
65079	'Rtn4r'	12	11	8	3	17	13	10.33333333	11	0.051754887	0.949603032	0.997836337
65086	'Lpar3'	2	3	8	1	1	0	4.333333333	0.666666667	-2.668799845	0.130883023	0.728882802
65098	'Zfand6'	1565	1581	2733	1404	1417	1587	1959.666667	1469.333333	-0.388220199	0.408728773	0.957157474

65099	'Irak1bp1'	108	139	261	59	160	208	169.3333333	142.3333333	-0.367593161	0.537800581	0.972790461
65100	'Zic5'	9	9	3	0	2	3	7	1.666666667	-2.091865592	0.118613055	0.707806469
65102	'Nif3l1'	659.32	655.45	395.32	150.59	577.5	494.68	570.03	407.59	-0.513787012	0.262610662	0.88013169
65103	'Arl6ip6'	505	551	721	883	549	693	592.3333333	708.3333333	0.369565691	0.46734262	0.972790461
65105	'Arl6ip4'	491	530	346	348	505	628	455.6666667	493.6666667	0.155292927	0.569858064	0.972790461
65106	'Arl6ip5'	583	580	1051	1606	886	1087	738	1193	0.803514188	0.174746201	0.793295795
65107	'Lrp10'	1123	1110	699	2308	1594	2124	977.3333333	2008.666667	1.186433323	0.005252726	0.171346471
65111	'Dap3'	1459	1531	1753	1321	1257	1518	1581	1365.333333	-0.150186098	0.685484069	0.972790461
65112	'Pmepa1'	481	496	460	976	420	410	479	602	0.552523382	0.369502311	0.937712122
65113	'Ndfip1'	5112	5425	4705	5362	6634	8347	5080.666667	6781	0.457281784	0.076746362	0.609792246
65114	'Vps35'	5271	5430	6185	3706	4221	5569	5628.666667	4498.666667	-0.295213335	0.371522387	0.938527219
65115	'Bean1'	117	83	58	3	148	98	86	83	-0.158200647	0.869463949	0.979633196
65116	'Prrg2'	154	186	125	122	275	278	155	225	0.537975252	0.117140373	0.705163127
65221	'Slc15a3'	60	43	42	26	56	75	48.33333333	52.33333333	0.097583562	0.834299062	0.974723675
65246	'Xpo7'	2606	2637	2077	1448	2517	2743	2440	2236	-0.108902277	0.650690736	0.972790461
65247	'Asb1'	851	913	509	970	546	450	757.6666667	655.3333333	0.018860668	0.973546471	0.999493374
65254	'Dpysl5'	115	74	103	66	40	20	97.33333333	42	-1.019103446	0.142989056	0.749054337
65255	'Asb4'	222	230	119	66	191	138	190.3333333	131.6666667	-0.515651478	0.230873547	0.857634106
65256	'Asb2'	12	6	2	1	2	8	6.666666667	3.666666667	-0.868918316	0.488069156	0.972790461
65257	'Asb3'	465	528	411	300	559	650	468	503	0.106114612	0.712800826	0.972790461
652925	'Tmem243'	74	76	77	39	72	148	75.66666667	86.33333333	0.137725923	0.784640229	0.972790461
653016	'Mymx'	6	2	3	0	2	4	3.666666667	2	-0.96566523	0.528484868	0.972790461
654309	'Nrp'	15.25	17.19	10.13	6.1	12	3.06	14.19	7.053333333	-0.916075135	0.279988217	0.889355605
654462	'Kncn'	1	1	0	0	1	0	0.666666667	0.333333333	-0.74517548	0.838722495	0.974723675
654470	'Tctn1'	637.69	634.11	185.04	254.05	757.27	466.42	485.6133333	492.58	0.069144827	0.899063648	0.986106073
654498	'Hhla1'	0	0	0	0	0	1	0	0.333333333	1.020273531	0.802557913	0.972790461
654795	'Sdr39u1'	592.47	619.34	288.41	28.36	535.59	707.65	500.0733333	423.8666667	-0.340966836	0.688372066	0.972790461
654801	'Zfp784'	152	160	79	151	161	119	130.3333333	143.6666667	0.279452954	0.537984425	0.972790461
654812	'Angptl7'	85	74	0	0	122	88	53	70	0.362207137	0.887895856	0.984534762
654818	'Smco3'	18.66	26.23	5	2	9.14	8.74	16.63	6.626666667	-1.342018039	0.146811697	0.753611197
654821	'Gcnt7'	73	69.1	8	37	45	22.01	50.03333333	34.67	-0.331628992	0.689372826	0.972790461
654824	'Ankrd37'	291	210	2662	34	87	138	1054.333333	86.33333333	-3.843727093	1.28E-04	0.01664332
65945	'Clstn1'	2253	2072	754	183	2796	2750	1693	1909.666667	0.099839363	0.901334612	0.98672375
65960	'Twsg1'	2721	2648	1315	6285	3412	3386	2228	4361	1.197791817	0.032404618	0.434162511
65961	'Utp3'	940	920	1422	672	855	937	1094	821.3333333	-0.407396881	0.318576052	0.916136994
65962	'Slc9a3r2'	641	762	519	1091	644	718	640.6666667	817.6666667	0.530610537	0.282746055	0.890879682
65963	'Tmem176b'	2386	2445	5083	2178	2909	3127	3304.666667	2738	-0.296912597	0.557002629	0.972790461
65964	'Map3k20'	428	399	502	124	328	330	443	260.6666667	-0.81914917	0.045570018	0.498244985
65967	'Eefsec'	675	701	335	349	574	713	570.3333333	545.3333333	-0.020352503	0.954493497	0.999493374
65969	'Cubn'	10	12	15	54	11	12	12.33333333	25.66666667	1.338289073	0.173182127	0.791556755

65970	'Lima1'	1819	2077	1330	1315	2363	1995	1742	1891	0.160724983	0.556371361	0.972790461
65971	'Tbata'	1	1	1	8	2	4	1	4.666666667	2.428684902	0.108124534	0.684880244
65972	'Ifi30'	355	340	785	374	462	678	493.3333333	504.6666667	-0.014912289	0.978143933	0.999493374
65973	'Asph'	1563	1275.6	1078	1360	1486.5	1885.5	1305.516667	1577.34	0.34093927	0.250099072	0.870698327
66011	'Ranbp17'	283	272	92	48	283	346	215.6666667	225.6666667	0.022452857	0.973372055	0.999493374
66039	'Tmem254a'	420	515	401	121	917	828	445.3333333	622	0.383594904	0.535969334	0.972790461
66042	'Sostdc1'	6	6	1	0	17	1	4.333333333	6	0.438163083	0.79755441	0.972790461
66043	'Atp5d'	1785	1821	2742	2703	1648	2280	2116	2210.333333	0.15972804	0.758892855	0.972790461
66044	'Dtd1'	324	278	198	39	278	343	266.6666667	220	-0.366914497	0.571115962	0.972790461
66046	'Ndufb5'	998	1033	1281	701	1036	1213	1104	983.3333333	-0.170483204	0.617113727	0.972790461
66047	'Mrpl54'	372	373	530	190	268	350	425	269.3333333	-0.673914172	0.090012325	0.648437606
66048	'Emc6'	693	663	1046	583	495	675	800.6666667	584.3333333	-0.410088141	0.372137596	0.938594721
66049	'Rogdi'	351	360	110	153	410	384	273.6666667	315.6666667	0.238488574	0.643976014	0.972790461
66050	'0610009B22Rik'	244	284	172	155	302	332	233.3333333	263	0.190241087	0.552834482	0.972790461
66052	'Sdhc'	1519	1594	1464	1281	1596	2075	1525.666667	1650.666667	0.146619221	0.591090589	0.972790461
66053	'Ppil2'	1402.8	1516.9	1086	593.73	1512.5	1612.68	1335.226667	1239.64	-0.126523592	0.716010457	0.972790461
66054	'Cndp2'	607	681	575	499	718	667	621	628	0.061465187	0.818825068	0.972790461
66055	'Sf3b6'	470	519	587	288	505	542	525.3333333	445	-0.251635654	0.452832346	0.970649024
66056	'Zfp524'	109	95	273	98	95	141	159	111.3333333	-0.54257543	0.387319451	0.949501169
66058	'Tmem176a'	851	904	1604	532	1064	1214	1119.666667	936.6666667	-0.326342665	0.49461367	0.972790461
66059	'Krtcap2'	566	578	1604	1028	640	986	916.0066667	884.6666667	-0.018391115	0.977744726	0.999493374
66060	'Cystm1'	76	75	285	601	40	158	145.3333333	266.3333333	1.10826851	0.30519211	0.907188578
66061	'Tctex1d2'	202	200	160	43	104	160	187.3333333	102.3333333	-0.910381971	0.037680775	0.459930654
66065	'Hsd17b14'	15	18	1	6	26	15	11.33333333	15.66666667	0.516824894	0.614714903	0.972790461
66066	'Gng11'	183	189	127	15	272	193	166.3333333	160	-0.159347401	0.84025356	0.974780034
66067	'Gtpbp8'	192.3	204.88	127.88	54.49	202.01	195.75	175.02	150.75	-0.247883604	0.598942347	0.972790461
66069	'Snupn'	476	472	632	307	379	454	526.6666667	380	-0.456826657	0.220461118	0.849076011
66070	'Cwc15'	1078	1101	1696	713	937	1246	1291.666667	965.3333333	-0.436529023	0.294338617	0.90029104
66071	'Ethe1'	113	118	83	183	80	149	104.6666667	137.3333333	0.563564226	0.310422052	0.910971113
66072	'Sdhaf2'	658	719	811	499	583	723	729.3333333	601.6666667	-0.248846762	0.46365236	0.972790461
66073	'Txndc12'	523	550	517	494	757	897	530	716	0.44644475	0.100242944	0.671535889
66074	'Tmem167'	1068	1181	1152	1407	1216	1125	1133.666667	1249.333333	0.250957409	0.542559094	0.972790461
66075	'Chchd3'	17941	18073	4642	1919	14088	6391.65	13551.96333	7466.166667	-0.866640526	0.21792358	0.844165262
66077	'Aurkaip1'	457	543	601	279	432	728	533.6666667	479.6666667	-0.189105084	0.638115163	0.972790461
66078	'Tsen34'	1193	1243	1211	695	1469	1611	1215.666667	1258.333333	0.027201321	0.931655824	0.993601701
66079	'Tmem42'	137	137	118	14	131	102	130.6666667	82.33333333	-0.763471266	0.253526407	0.872664697
66082	'Abhd6'	393.61	420.82	213.65	884.99	562.32	544.52	342.6933333	663.9433333	1.15653805	0.025514666	0.395185754
66083	'Setd6'	147	152	138	519	161	301	145.6666667	327	1.378783914	0.031151553	0.42861408
66084	'Rmnd1'	526	603	255	137	624	555	461.3333333	438.6666667	-0.099579114	0.853228083	0.975734242
66085	'Eif3f'	3742	3474	6149	6336	3334	4217	4455	4629	0.175359835	0.764783234	0.972790461

66086	'Cep20'	648	805	451	608	820	772	634.6666667	733.3333333	0.286611445	0.349676444	0.92886351
66087	'Emc3'	990	1026	835	815	1332	1520	950.3333333	1222.333333	0.381280131	0.117355401	0.705393896
66089	'Rmnd5b'	439	530	439	431	562	811	469.3333333	601.3333333	0.378651225	0.211420259	0.840335617
66090	'Ypel3'	570	529	1156	582	612	585	751.6666667	593	-0.32323244	0.546439747	0.972790461
66091	'Ndufa3'	304	321	298	272	287	393	307.6666667	317.3333333	0.094431795	0.76889365	0.972790461
66092	'Ghitm'	3509	3588	13720	11977	3357	6376	6939	7236.666667	0.173977731	0.837829601	0.974723675
66094	'Lsm7'	432	511	343	140	380	526	428.6666667	348.6666667	-0.337231735	0.432105589	0.963771875
66096	'Lamtor4'	200	237	242	218	218	319	226.3333333	251.6666667	0.193491245	0.601855283	0.972790461
66098	'Chchd6'	547	513	320	58	306	524	460	296	-0.720860199	0.258236621	0.877405867
66101	'Ppih'	471.75	562.19	315.53	97	336	421.44	449.8233333	284.8133333	-0.699817651	0.146519018	0.753611197
66102	'Cxcl16'	111.51	127.17	1129.3	682.99	151.59	361.04	455.98	398.54	-0.123532713	0.907803898	0.988823459
66104	'Tceal6'	110.71	105.61	37.45	60.13	125.48	137.77	84.59	107.7933333	0.39627242	0.423526517	0.960285372
66105	'Ube2d3'	7419.4	7251.6	9998.7	4373.5	6287.3	8206.99	8223.263333	6289.286667	-0.402831725	0.275701193	0.887252716
66106	'Smpx'	186	222	63	12	500	437	157	316.3333333	0.93019706	0.339560465	0.924888762
66107	'Wfdc21'	0	1	1	0	1	0	0.666666667	0.333333333	-0.886945817	0.805479824	0.972790461
66108	'Ndufa9'	1858	1916	1260	776	1483	1774	1678	1344.333333	-0.30928452	0.298578628	0.902476451
66109	'Tspan13'	227	197	39	72	460	539	154.3333333	357	1.183858209	0.123439947	0.715310266
66111	'Tmed3'	604	572	1259	414	659	1058	811.6666667	710.3333333	-0.275590019	0.611287355	0.972790461
66112	'Mtarc1'	0	2	0	3	2	2	0.666666667	2.333333333	2.014143728	0.345412133	0.926100052
66113	'Apoa5'	2	2	0	0	1	2	1.333333333	1	-0.434829477	0.853935196	0.975734242
66114	'Dnajc30'	228.28	244.52	245.58	123.82	261.49	273.11	239.46	219.4733333	-0.146001421	0.669840881	0.972790461
66116	'Nat8f1'	73	68	26	13	57	57	55.66666667	42.33333333	-0.408925991	0.517242412	0.972790461
66117	'Fmc1'	98	128	180	107	110	156	135.3333333	124.3333333	-0.111227618	0.816132351	0.972790461
66118	'Sarnp'	1130	1228	2165	954	1079	1310	1507.666667	1114.333333	-0.43734521	0.35551025	0.931809687
66119	'Tomm6'	448	475	818	425	412	827	580.3333333	554.6666667	-0.090418536	0.858265013	0.976475731
66120	'Fkbp11'	74	74	361	98	64	145	169.6666667	102.3333333	-0.788348752	0.339728748	0.925008117
66121	'Chchd1'	325	360	465	168	350	424	383.3333333	314	-0.33909628	0.411249115	0.95722888
66124	'Josd2'	300	311	234	156	236	333	281.6666667	241.6666667	-0.204156213	0.50351343	0.972790461
66125	'Sf3b5'	352	373	381	437	475	650	368.6666667	520.6666667	0.537008769	0.101712196	0.671759705
66126	'Elof1'	652	676	527	553	745	766	618.3333333	688	0.208220713	0.405065114	0.957157474
66128	'Mrps36'	218	249	371	178	230	350	279.3333333	252.6666667	-0.171225424	0.703882112	0.972790461
66129	'Prxl2c'	603.86	573	1391	1029	823.97	1300.51	855.9533333	1051.16	0.29970733	0.606533842	0.972790461
66131	'Tipin'	952	1039	711	243	679	656	900.6666667	526	-0.794123978	0.030324657	0.423915109
66132	'Prorp'	347.46	348	277.95	144	302.33	355	324.47	267.11	-0.2875239	0.373377667	0.93929675
66136	'Znrd1'	194	232	309	89	226	231	245	182	-0.492848454	0.28029577	0.889355605
66138	'Bud23'	518.72	533.48	364.42	416.18	585.51	588.89	472.2066667	530.1933333	0.226478734	0.386931791	0.94901869
66139	'Mymk'	0	1	1	0	0	0	0.666666667	0	-1.851176388	0.646290011	0.972790461
66140	'Ska2'	349	344	313	125	372	294	335.3333333	263.6666667	-0.374772373	0.330460683	0.921648675
66141	'Ifitm3'	502	439	1840	2042	437	1305	927	1261.333333	0.546438195	0.534357474	0.972790461
66142	'Cox7b'	773	784	1205	634	690	880	920.6666667	734.6666667	-0.311663513	0.463570286	0.972790461

66143	'Eef1e1'	265	261	580	205	286	390	368.6666667	293.6666667	-0.38280489	0.476101031	0.972790461
66144	'Atp6v1f'	768	778	1989	837	690	1186	1178.333333	904.3333333	-0.398576706	0.509300611	0.972790461
66146	'Maco1'	1545	1520	1508	832	1417	1557	1524.333333	1268.666667	-0.261763217	0.333790006	0.921648675
66147	'Necap2'	609	598	1108	897	811	1164	771.6666667	957.3333333	0.326782529	0.510078873	0.972790461
66148	'Dnajc15'	600	646	764	291	656	756	670	567.6666667	-0.287922587	0.457746727	0.972692609
66151	'Prr13'	439	482	1531	1204	445	773	817.3333333	807.3333333	0.072446535	0.925911954	0.992381554
66152	'Uqcr10'	576	672	649	605	495	613	632.3333333	571	-0.055944717	0.887215991	0.984534762
66153	'Fbxo36'	108	81	92	85	87	99	93.66666667	90.33333333	0.013591056	0.972733521	0.999493374
66154	'Tmem14c'	582	696	278	601	858	1543	518.6666667	1000.666667	0.984322461	0.024638028	0.388406457
66155	'Ufc1'	528	684	1179	122	657	723	797	500.6666667	-0.825372346	0.207760489	0.835213465
66156	'Anapc11'	650	770	869	272	586	889	763	582.3333333	-0.453802529	0.294305463	0.90029104
66158	'Rtl8a'	302.25	369.24	329.16	159.59	346.4	416.96	333.55	307.65	-0.147600493	0.681238924	0.972790461
66161	'Pop4'	701	719	675	260	707	871	698.3333333	612.6666667	-0.240947328	0.548895935	0.972790461
66162	'Bola2'	89	113	214	110	66	119	138.6666667	98.33333333	-0.453545775	0.466070589	0.972790461
66163	'Mrpl4'	490	470.57	495	532.05	483	645	485.19	553.35	0.258474221	0.47111484	0.972790461
66164	'Nip7'	485	536	826	406.85	475	662	615.6666667	514.6166667	-0.267150253	0.543690256	0.972790461
66165	'Bccip'	602.98	685	883.2	519.73	871	978.46	723.7266667	789.73	0.103404294	0.783041237	0.972790461
66166	'S100a14'	0	1	1	3	0	2	0.666666667	1.666666667	1.500150502	0.52766338	0.972790461
66167	'Tma7'	794.5	1146.1	1357.4	608.52	1169.1	1271.39	1099.326667	1016.32	-0.15105529	0.712581815	0.972790461
66168	'Grina'	1238	1205	2644	2999	1642	2299	1695.666667	2313.333333	0.531734034	0.390906206	0.952178913
66169	'Tomm7'	566	594	747	315	401	484	635.6666667	400	-0.656170705	0.067697133	0.580700792
66170	'Chchd5'	230	193	421	81	198	239	281.3333333	172.6666667	-0.80345646	0.143509197	0.749971646
66171	'Pgls'	526	591	660	462	702	749	592.3333333	637.6666667	0.112627002	0.728103762	0.972790461
66172	'Med11'	138	169	266	220	184	191	191	198.3333333	0.11730913	0.824423232	0.972790461
66174	'Nudt14'	193	254	142	111	278	283	196.3333333	224	0.188617059	0.624527259	0.972790461
66175	'Mustn1'	0	3	1	0	3	0	1.333333333	1	-0.476628609	0.872045563	0.980171118
66176	'Nat9'	380	415	306	117	247	363	367	242.3333333	-0.621576865	0.10142407	0.671759705
66177	'Ubl5'	665.99	719.27	786.57	583.83	560.85	762.11	723.9433333	635.5966667	-0.136847001	0.705551523	0.972790461
66179	'Ogfod3'	175.12	178	101	47	217	250	151.3733333	171.3333333	0.126303185	0.819919048	0.972790461
66180	'P3h4'	728	697	413	1412	1140	1508	612.6666667	1353.333333	1.27094437	0.001191297	0.072517381
66181	'Nop10'	581	665	731	609	501	646	659	585.3333333	-0.093859263	0.818225215	0.972790461
66183	'Sptssb'	1	1	0	0	5	4	0.666666667	3	2.115481286	0.333426888	0.921648675
66185	'Virma'	2309	2241	1615	1610	2306	2371	2055	2095.666667	0.082096533	0.731404349	0.972790461
66190	'Acer3'	488	529	509	128	393	340	508.6666667	287	-0.869455091	0.029938506	0.421360177
66191	'Ier3ip1'	559	522	786	504	523	619	622.3333333	548.6666667	-0.149160099	0.720749952	0.972790461
66192	'Lage3'	389	412	228	102	401	552	343	351.6666667	-0.016731615	0.97504458	0.999493374
66193	'Pithd1'	573	567	663	367	468	610	601	481.6666667	-0.306017707	0.356227639	0.931977458
66194	'Pycrl'	292	356	213	204	413	348	287	321.6666667	0.195309906	0.539714873	0.972790461
66195	'Lce1g'	0	0	1	0	0	0	0.333333333	0	-0.903279821	0.824807108	0.972790461
66196	'Myo19'	435.07	469.07	192	202	556.18	344	365.38	367.3933333	0.047548513	0.917557899	0.990547571

66197	'Cks2'	536	530	449	652	476	825	505	651	0.456425853	0.245566795	0.866779035
66198	'Them5'	0	0	0	1	1	0	0	0.666666667	2.050872943	0.610671515	0.972790461
66199	'Commd4'	195	246	213	108	287	347	218	247.3333333	0.134801595	0.745615411	0.972790461
66200	'Commd6'	286	338	530	424	313	395	384.6666667	377.3333333	0.03835909	0.941292625	0.996040262
66201	'Vta1'	567	579	714	499	567	791	620	619	0.015112454	0.966730456	0.999493374
66202	'1110059G10Rik'	258	309	288	84	233	225	285	180.6666667	-0.697599366	0.078727839	0.615899891
66204	'Acyp1'	146	152	107	87	171	146	135	134.6666667	0.024428936	0.939782067	0.995607401
66205	'Cd302'	108	106	60	131	160	159	91.33333333	150	0.804554341	0.028205887	0.409664118
66206	'1110059E24Rik'	323.95	335.51	492.8	304	468	517	384.0866667	429.6666667	0.147422447	0.713753655	0.972790461
66208	'Nenf'	713	714	474	125	832	774	633.6666667	577	-0.211087087	0.720910367	0.972790461
66209	'Inip'	531	548	273	380	471	434	450.6666667	428.3333333	0.029618388	0.934824601	0.994413066
66211	'Rpl31'	13	12	7	0	5	14	10.66666667	6.333333333	-0.857551755	0.471013012	0.972790461
66212	'Sec61b'	440.27	483.95	1503.5	740	491	809	809.23	680	-0.247101787	0.720542667	0.972790461
66213	'Med7'	736	723	884	478	629	800	781	635.6666667	-0.28913456	0.392233328	0.952648938
66214	'Rgcc'	86	73	827	350	164	156	328.6666667	223.3333333	-0.515029011	0.621864698	0.972790461
66218	'Ndufb9'	1053	1141	1044	1234	909	1372	1079.333333	1171.666667	0.213354821	0.58884997	0.972790461
66220	'Zdhhc12'	100	151	171	85	196	186	140.6666667	155.6666667	0.103548469	0.818213764	0.972790461
66222	'Serpinb1a'	183	187	157	312	234	282	175.6666667	276	0.772721181	0.064383095	0.570121756
66223	'Mrpl35'	496	482	508	264	397	445	495.3333333	368.6666667	-0.41256022	0.155538273	0.766717687
66225	'L1ph'	592.91	668.44	1234.2	552.4	646.33	858.99	831.84	685.9066667	-0.297964735	0.547661183	0.972790461
66226	'Trappc2'	726	810	306	188	536	774	614	499.3333333	-0.310187401	0.549105345	0.972790461
66229	'Rpl7l1'	2143	2295	2342	2039	1943	2503	2260	2161.666667	-6.61E-04	0.998469528	0.999900097
66230	'Mrps28'	203	217	226	125	211	241	215.3333333	192.3333333	-0.166028907	0.611412719	0.972790461
66231	'Thoc7'	630.99	677	1165	166.93	695.99	729.99	824.3133333	530.97	-0.764898923	0.188531462	0.810884334
66233	'Dmap1'	912	873	718	233	769	995	834.3333333	665.6666667	-0.385858605	0.397667524	0.954563638
66234	'Msmo1'	545	685	710	1894	845	831	646.6666667	1190	1.081760513	0.079628138	0.617305615
66235	'Eif1ax'	1982	2148	3021	1829	2376	3407	2383.666667	2537.333333	0.073661413	0.854573641	0.975734242
66237	'Atp6v1g2'	53.05	68.27	17.08	4	59.04	41.08	46.13333333	34.70666667	-0.452210122	0.603926759	0.972790461
66240	'Kcne1l'	160	173	53	20	141	74	128.6666667	78.33333333	-0.729273756	0.293352355	0.90029104
66241	'Tmem9'	972	1029	513	1280	1416	1724	838	1473.333333	0.909932992	0.008929405	0.231288612
66242	'Mrps16'	381	372	437	171	303	374	396.6666667	282.6666667	-0.510779833	0.143489677	0.749971646
66244	'Nemf'	1420	1507	1286	1266	1249	1178	1404.333333	1231	-0.090115051	0.797670754	0.972790461
66245	'Hspbp1'	325	364	314	215	432	456	334.3333333	367.6666667	0.131308749	0.665348821	0.972790461
66246	'Osgep'	584	618	538	308	640	846	580	598	0.018328234	0.957423795	0.999493374
66248	'Alg5'	503	473	251	241	492	532	409	421.6666667	0.07047796	0.845014141	0.975182082
66249	'Pno1'	753	795	621	375	691	988	723	684.6666667	-0.089747091	0.784823326	0.972790461
66251	'Arfgap3'	706	643	2424	875	681	1008	1257.666667	854.6666667	-0.575817903	0.411676959	0.95722888
66253	'Aig1'	65.26	59.39	230	167	139.03	194	118.2166667	166.6766667	0.474416583	0.513532187	0.972790461
66254	'Dimt1'	363.69	391.01	354.34	355.35	409.73	410.93	369.68	392.0033333	0.149350708	0.633586958	0.972790461
66255	'Hsbp1l1'	44	46	21	193	45	43	37	93.66666667	1.691692471	0.049744043	0.517076953

66256	'Ssr2'	1660	1687	2225	1549	2168	3599	1857.333333	2438.666667	0.361438212	0.366167337	0.936406787
66257	'Nicn1'	616	619	626	512	704	654	620.3333333	623.3333333	0.048746732	0.86858689	0.979336612
66258	'Mrps17'	824	824	686	368	846	909	778	707.6666667	-0.153316762	0.6324476	0.972790461
66259	'Camk2n1'	227	204	1010	638	149	242	480.3333333	343	-0.343217727	0.715418176	0.972790461
66260	'Tmem54'	1	0	0	13	1	1	0.333333333	5	4.196839772	0.058564817	0.552046072
66261	'Tm4sf20'	0	0	0	3	2	0	0	1.666666667	3.440499981	0.350686093	0.92886351
66262	'Ing5'	791	706	992	552	829	772	829.6666667	717.6666667	-0.200886087	0.58148241	0.972790461
66264	'Ccdc28b'	134	168	134	114	249	220	145.3333333	194.3333333	0.419535316	0.218599837	0.844891257
66266	'Eapp'	633.01	737.29	1032.8	524.78	703.2	808.32	801.0433333	678.7666667	-0.242576572	0.549240005	0.972790461
66268	'Pigy1'	205	177	218	276	289	354	200	306.3333333	0.662704379	0.070345759	0.589465353
66269	'Tmed6'	0	0	0	0	1	0	0	0.333333333	1.020273531	0.802557913	0.972790461
66270	'Retreg1'	158	141	348	369	209	375	215.6666667	317.6666667	0.604105341	0.340104779	0.92526901
66271	'Tmem126a'	220	228	454	121	234	273	300.6666667	209.3333333	-0.596019571	0.25255376	0.871252558
66272	'Cox16'	296.09	309.36	282.42	77.47	273.64	328.37	295.9566667	226.4933333	-0.457009027	0.33522779	0.922335099
66273	'Aamdc'	164	175	160	72	208	231	166.3333333	170.3333333	-0.012468982	0.976290545	0.999493374
66274	'Lyrm9'	337	386	56	228	299	321	259.6666667	282.6666667	0.252860099	0.695338359	0.972790461
66276	'1810009A15Rik'	235	265	174	136	216	261	224.6666667	204.3333333	-0.106985259	0.717813035	0.972790461
66277	'Klf15'	96	101	151	94	60	125	116	93	-0.276048649	0.609634928	0.972790461
66278	'Smim20'	274	256	128	132	261	300	219.3333333	231	0.103965614	0.788446188	0.972790461
66279	'Tmem218'	221	228	127	241	276	278	192	265	0.563651063	0.109434582	0.689773582
66282	'Tma16'	219	234	616	107	215	254	356.3333333	192	-0.995467704	0.104272622	0.676639601
66286	'Sec11c'	146	127	309	184	137	331	194	217.3333333	0.136023469	0.824735831	0.972790461
66290	'Atp6v1g1'	1687	1713	4124	1767	1658	2573	2508	1999.333333	-0.347738905	0.543535413	0.972790461
66291	'Smim8'	268	282	135	32	200	198	228.3333333	143.3333333	-0.725596351	0.237005932	0.862272407
66292	'Mrps21'	334	377	387	261	287	393	366	313.6666667	-0.188211215	0.58076097	0.972790461
66294	'Fam3a'	374	368	305	66	477	457	349	333.3333333	-0.16144602	0.79222861	0.972790461
66296	'Haus2'	551	570	408	201	685	783	509.6666667	556.3333333	0.07861724	0.859993524	0.976863639
66300	'Inafm1'	199.61	240.53	332.39	143.93	340.35	300.21	257.51	261.4966667	-0.02969486	0.947152657	0.996914484
66302	'Rmdn1'	239	203	157	103	219	204	199.6666667	175.3333333	-0.173711381	0.596247853	0.972790461
66306	'Fam53c'	1785	1712	1857	1513	1421	1848	1784.666667	1594	-0.099078502	0.776692998	0.972790461
66307	'Isoc1'	1893	1971	1301	657	2219	2482	1721.666667	1786	0.011466269	0.979123367	0.999493374
66308	'Mplkip'	404.38	392.99	376.01	458	439	514	391.1266667	470.3333333	0.346597035	0.31155881	0.911537035
66309	'Tmem128'	315	297	298	187	392	418	303.3333333	332.3333333	0.115324735	0.721803413	0.972790461
66310	'Dpy30'	328	374	443	198	385	533	381.6666667	372	-0.085049866	0.833582341	0.974723675
66311	'Cenpw'	169	162	121	30	89	132	150.6666667	83.66666667	-0.894855837	0.064883101	0.572150983
66313	'Smurf2'	2231	2178	2347	1486	2106	2151	2252	1914.333333	-0.208025129	0.466144832	0.972790461
66314	'Tpd52l2'	1560	1523	2119	2031	1723	2084	1734	1946	0.232350418	0.591338544	0.972790461
66315	'Senp7'	896	902	622	773	1149	1001	806.6666667	974.3333333	0.335712002	0.214497766	0.842621568
66317	'Wdr61'	817	927	961	571	867	1061	901.6666667	833	-0.113713598	0.713468184	0.972790461
66320	'Tmem208'	201	221	160	171	278	352	194	267	0.478512092	0.106946098	0.681615561

66322	'Krt88'	2	0	1	4	0	0	1	1.333333333	0.848190343	0.770433489	0.972790461
66323	'1700001K19Rik'	5	2	0	33	3	8	2.333333333	14.66666667	3.067275404	0.036573564	0.457518227
66326	'Dnajc5b'	0	0	0	1	0	0	0	0.333333333	1.020273531	0.802557913	0.972790461
66329	'Susd3'	72	65	16	190	200	206	51	198.6666667	2.108777795	2.29E-04	0.024910793
66330	'1700020L24Rik'	8.18	14.29	3.99	0	7.34	7.98	8.82	5.106666667	-0.887532793	0.484543325	0.972790461
66333	'Aqp11'	49	52	29	68	53	53	43.33333333	58	0.56886311	0.270272222	0.883851074
66335	'Atp6v1c1'	1349	1462	1379	720	968	1765	1396.666667	1151	-0.289631066	0.42334778	0.960234789
66336	'Cenpp'	250.02	251.69	72.02	8	151.48	239.39	191.2433333	132.9566667	-0.60695472	0.50762863	0.972790461
66337	'Fam229b'	125	108	32	5	95	119	88.33333333	73	-0.346911484	0.704117664	0.972790461
66338	'Cdrt4'	1	1	1	0	0	0	1	0	-2.390783043	0.494152846	0.972790461
66340	'Psenen'	505	491	782	496	498	888	592.6666667	627.3333333	0.073931587	0.87164879	0.980171118
66341	'Eid3'	1	5	1	31	0	9	2.333333333	13.33333333	2.915701685	0.080570732	0.617641878
66343	'Tmem177'	269	277	105	178	228	254	217	220	0.116141066	0.785556597	0.972790461
66346	'1700029P11Rik'	12	4	6	2	15	10	7.333333333	9	0.238174716	0.812026119	0.972790461
66349	'Dmac2'	415	424	385	168	394	427	408	329.6666667	-0.332556108	0.33049914	0.921648675
66350	'Pla2g12a'	275	277	1262	174	420	718	604.6666667	437.3333333	-0.655950458	0.408282694	0.957157474
66352	'Blzf1'	729	728	980	329	627	696	812.3333333	550.6666667	-0.596311419	0.11602263	0.70293889
66353	'Riiad1'	19	23	4	42	32	15	15.33333333	29.66666667	1.20187056	0.162569486	0.774020016
66354	'Snw1'	2162	2170	2364	1737	1962	2077	2232	1925.333333	-0.15983521	0.623192429	0.972790461
66355	'Gmpr'	248	320	116	630	429	620	228	559.6666667	1.460559132	0.003537501	0.137037276
66356	'Knop1'	4025.9	4110.9	1675	2584	3751.9	3631	3270.61	3322.313333	0.112351495	0.771278119	0.972790461
66357	'Ostc'	1322	1390	3393	1493	1457	2498	2035	1816	-0.203153687	0.727971749	0.972790461
66358	'Adprm'	196	183	188	112	234	187	189	177.6666667	-0.086415111	0.798900102	0.972790461
66359	'Cox20'	168.86	177.33	346.52	62.44	172.31	157.8	230.9033333	130.85	-0.914315976	0.097480618	0.663789939
66361	'Zfand1'	505	489	222	265	502	379	405.3333333	382	-0.017789441	0.964031941	0.999493374
66362	'Exosc3'	234	243	449	183	220	274	308.6666667	225.6666667	-0.465568913	0.341970962	0.925308103
66364	'Pigbos1'	56	67	55	39	64	64	59.33333333	55.66666667	-0.067014374	0.85998623	0.976863639
66365	'Ccdc90b'	581	618	539	232	667	715	579.3333333	538	-0.150577378	0.699722379	0.972790461
66366	'Ergic3'	1965	2039	1331	1478	2142	2741	1778.333333	2120.333333	0.292533682	0.272076365	0.884539076
66367	'2310022A10Rik'	472	508	560	276	379	502	513.3333333	385.6666667	-0.408480644	0.216777628	0.843391934
66368	'Rtca'	810	799	512	393	592	790	707	591.6666667	-0.223750183	0.447866852	0.970198456
66369	'Dus2'	277	277	142	61	271	275	232	202.3333333	-0.235135158	0.65995875	0.972790461
66371	'Chmp4c'	6	5	8	14	2	7	6.333333333	7.666666667	0.474303191	0.669824361	0.972790461
66373	'Lsm5'	354	351	412	93	296	536	372.3333333	308.3333333	-0.376935006	0.495514465	0.972790461
66374	'2310011J03Rik'	408	396	413	247	428	519	405.6666667	398	-0.036298544	0.906967364	0.988823459
66375	'Dhrs7'	553	673	310	220	897	792	512	636.3333333	0.288932957	0.562019552	0.972790461
66377	'Ndufc1'	416	466	407	324	378	575	429.6666667	425.6666667	0.015783065	0.959973758	0.999493374
66379	'Cox14'	429	532	591	822	474	804	517.3333333	700	0.53786273	0.276327377	0.888021651
66381	'Rnf113a2'	198	235	244	163	224	306	225.6666667	231	0.037235111	0.915106845	0.990211231
66383	'Iscu'	755	792	555	1551	678	888	700.6666667	1039	0.781982759	0.165544039	0.78006879

66384	'Srp19'	462	505	1383	542	525	601	783.3333333	556	-0.503799173	0.41989101	0.958308933
66385	'Ppp1r7'	977	1015	671	299	887	1086	887.6666667	757.3333333	-0.26454062	0.530049675	0.972790461
66387	'Nudt8'	120	140	71	33	135	148	110.3333333	105.3333333	-0.103789782	0.846601154	0.975209338
66388	'Cutc'	357	389	141	34	292	321	295.6666667	215.6666667	-0.515941807	0.469047498	0.972790461
66390	'Prelid3b'	663	699	608	738	667	712	656.6666667	705.6666667	0.204776544	0.571794239	0.972790461
66394	'Nosip'	771	815	1128	335	775	844	904.6666667	651.3333333	-0.534094129	0.205265821	0.833294914
66395	'Ahnak'	10718	9932	5852	5420	11157	9129	8834	8568.666667	-0.005033033	0.987551267	0.999493374
66396	'Ccdc82'	1201	1096.3	603.29	1116.1	1107.6	772.77	966.87	998.8266667	0.203191727	0.652658688	0.972790461
66397	'Sar1b'	816	822	942	1002	998	1323	860	1107.666667	0.412448578	0.244947591	0.866091613
66398	'Commd5'	242	243	236	109	254	298	240.3333333	220.3333333	-0.158393121	0.667492884	0.972790461
66399	'Tsfm'	336	355	372	366	324	554	354.3333333	414.6666667	0.271355274	0.478144539	0.972790461
66400	'Alkbh7'	198	208	239	114	196	262	215	190.6666667	-0.197795984	0.595474734	0.972790461
66401	'Nudt2'	128	140	288	71	140	158	185.3333333	123	-0.668338264	0.224384217	0.850759465
66403	'Asf1a'	658.14	593.54	797.4	334.64	662.33	807.97	683.0266667	601.6466667	-0.22647237	0.554545012	0.972790461
66404	'Rtf2'	1222	1334	1321	724	1159	1449	1292.333333	1110.666667	-0.221575298	0.454434501	0.970649024
66405	'Mcts2'	128	136	142	110	137	139	135.3333333	128.6666667	-0.02964589	0.932714118	0.993970337
66406	'Sac3d1'	200	185	160	102	188	236	181.6666667	175.3333333	-0.052501168	0.871779664	0.980171118
66407	'Mrps15'	359	355	388	143	448	497	367.3333333	362.6666667	-0.0859861	0.845450455	0.975182082
66408	'Aptx'	624	610	441	201	594	713	558.3333333	502.6666667	-0.188991646	0.649638488	0.972790461
66409	'Rsl1d1'	1768	1911	3898	1125	1722	2053	2525.666667	1633.333333	-0.681583495	0.180654275	0.801479605
66410	'Mterf3'	466	477.12	335	406	826	565	426.04	599	0.529511802	0.10057939	0.671759705
66411	'Tbcb'	587	550	939	504	673	1124	692	767	0.104678667	0.821004716	0.972790461
66412	'Arrdc4'	839	792	1923	256	919	218	1184.666667	464.3333333	-1.426494091	0.045675611	0.498244985
66413	'Psmd6'	1674	1835	1199	759	1633	2002	1569.333333	1464.666667	-0.105285973	0.751126083	0.972790461
66414	'Ndufa12'	865	974	781	707	662	918	873.3333333	762.3333333	-0.121125758	0.711007684	0.972790461
66416	'Ndufa7'	893	1007	1041	1541	808	1327	980.3333333	1225.333333	0.451481507	0.366007377	0.936406787
66419	'Mrpl11'	691	683	409	175	654	690	594.3333333	506.3333333	-0.268701244	0.568281771	0.972790461
66420	'Polr2e'	718	816	1459	940	678	939	997.6666667	852.3333333	-0.175843232	0.745146421	0.972790461
66421	'2410004B18Rik'	238	225	431	409	245	364	298	339.3333333	0.262258003	0.647664009	0.972790461
66422	'Dctpp1'	411	448	475	194	274	521	444.6666667	329.6666667	-0.460040452	0.257360783	0.877405867
66423	'Coprs'	358	404	178	105	257	401	313.3333333	254.3333333	-0.312192588	0.51842502	0.972790461
66425	'Pcp4l1'	679	754	70	724	626	413	501	587.6666667	0.472012331	0.551192097	0.972790461
66427	'Cyb5b'	2071	2118	914	3991	2486	3264	1701	3247	1.119394829	0.025762169	0.39630136
66431	'Oxld1'	53	54	31	21	63	60	46	48	0.056748259	0.908560563	0.988823459
66432	'Slc7a6os'	396	369	583	395	385	401	449.3333333	393.6666667	-0.141466764	0.754504162	0.972790461
66433	'Chchd7'	395	373	465	352	361	375	411	362.6666667	-0.120224179	0.754949651	0.972790461
66435	'Uggt2'	1235	1148	280	307	824	1083	887.6666667	738	-0.245949726	0.679706097	0.972790461
66437	'Fis1'	1201	1300	1259	689	1129	1223	1253.333333	1013.666667	-0.298599968	0.275714761	0.887252716
66438	'Hamp2'	0	2	0	2	5	2	0.666666667	3	2.265947021	0.270381432	0.883851074
66439	'Borcs7'	195	234	210	21	200	112	213	111	-1.038099824	0.119634136	0.710007992

66440	'Cdc26'	704	806	805	608	913	886	771.6666667	802.3333333	0.079378567	0.788676361	0.972790461
66441	'Magohb'	130	112	188	63	148	181	143.3333333	130.6666667	-0.206648802	0.672866791	0.972790461
66442	'Spc25'	659	630	160	76	417	469	483	320.6666667	-0.615055099	0.370126196	0.937712122
66443	'Tnfaip8l1'	109	127.02	79.01	58.04	172.01	129.01	105.01	119.6866667	0.186501629	0.657775589	0.972790461
66445	'Cyc1'	1193	1248	784	1586	1249	1729	1075	1521.333333	0.62193956	0.102859854	0.672865573
66446	'Exosc7'	664	713	455	168	635	507	610.6666667	436.6666667	-0.51327597	0.246894759	0.868602029
66447	'Mgst3'	229	305	167	452	462	485	233.6666667	466.3333333	1.104860794	0.002528083	0.11282608
66448	'Mrpl20'	610	595	413	323	461	569	539.3333333	451	-0.216590649	0.419182903	0.958308933
66449	'Pam16'	167	162	330	128	164	241	219.6666667	177.6666667	-0.34787361	0.504367921	0.972790461
66451	'2610528J11Rik'	0	1	2	5	2	5	1	4	2.057550326	0.20946856	0.837080112
66454	'Nmnat1'	146.65	144	114.21	18.12	134	93	134.9533333	81.70666667	-0.797038773	0.196035997	0.822019491
66455	'Cnpy4'	354	418	202	409	628	547	324.6666667	528	0.777777662	0.023093654	0.377626024
66459	'Pyurf'	377	412	185	127	346	303	324.6666667	258.6666667	-0.311657177	0.46458451	0.972790461
66460	'Sys1'	300	301	409	431	363	484	336.6666667	426	0.397807988	0.360447376	0.933706718
664608	'Rhox4g'	1	0	0.3	0	0	1	0.433333333	0.333333333	0.058500858	0.988561293	0.999493374
664609	'Rhox4a'	0	1	0	0	0	0	0.333333333	0	-0.903279821	0.824807108	0.972790461
66461	'Ptpmt1'	187	219	293	21	246	236	233	167.6666667	-0.631143412	0.400678123	0.955860942
664610	'Rhox4d'	0	1.42	1.11	0	1.63	0	0.843333333	0.543333333	-0.886945817	0.805479824	0.972790461
66462	'Rex1bd'	164	183	204	127	214	256	183.6666667	199	0.102556535	0.773759179	0.972790461
66464	'Taf12'	445	446	293	157	332	442	394.6666667	310.3333333	-0.352958986	0.328215507	0.921384366
66467	'Gtf2h5'	608	687	603	736	616	720	632.6666667	690.6666667	0.228168241	0.547613039	0.972790461
66468	'Ska1'	79	64	21	8	70	107	54.66666667	61.66666667	0.112600473	0.892026766	0.985171
66469	'Prxl2b'	154	181	227	82	162	195	187.3333333	146.3333333	-0.398881894	0.34370523	0.925308103
66471	'Anp32e'	1908	1966	2742	1627	1855	2199	2205.333333	1893.666667	-0.19854299	0.610710383	0.972790461
66473	'Ctrb1'	3	3	2	14	6	2	2.666666667	7.333333333	1.734534125	0.167249123	0.781391371
66475	'Rps23'	12943	13495	19506	10396	7748.9	10753.28	15314.47667	9632.623333	-0.601870695	0.197363015	0.822865273
66477	'Atp5md'	459	539	732	505	414	612	576.6666667	510.3333333	-0.130021764	0.775345994	0.972790461
664799	'Ctcfl'	19.94	13.49	1.76	0	8	46	11.73	18	0.629261855	0.680629607	0.972790461
66480	'Rpl15'	11304	11766	22234	13972	9480	12359	15101.55	11937	-0.273161138	0.621631167	0.972790461
664804	'Gm7347'	193.83	268.24	74.71	807.9	480.14	322.23	178.9266667	536.7566667	1.854365531	0.005519563	0.175188521
66481	'Rps21'	3253	3238	5053	5944	2745	3861	3848	4183.333333	0.258753372	0.661809014	0.972790461
66482	'Exoc2'	1239	1237	935	510	1075	1146	1137	910.3333333	-0.32083128	0.272539542	0.884738928
66483	'Rpl36al'	1210	1297	2678	1735	987	1446	1728.333333	1389.333333	-0.241169488	0.691203467	0.972790461
664831	'Gm7358'	1.01	0	1	1	1	0	0.67	0.666666667	0.117746761	0.96570115	0.999493374
664837	'Gm7361'	170.28	174.5	168.19	651.26	308.96	210.91	170.99	390.3766667	1.423277876	0.030346542	0.423915109
66487	'Smim4'	52	61	77	56	54	45	63.33333333	51.66666667	-0.219215339	0.672335894	0.972790461
66488	'Fam136a'	1317	1398	1422.9	430	1360	1514	1379.286667	1101.333333	-0.390974788	0.365158058	0.935765447
664883	'Nova1'	343	376	191	270	268	110	303.3333333	216	-0.301915385	0.600789604	0.972790461
66489	'Rpl35'	6877	7102	14535	9382	4759	7264	9504.666667	7135	-0.323316016	0.601400037	0.972790461
66491	'Polr2l'	410	426	405	691	365	493	413.6666667	516.3333333	0.47741908	0.348227151	0.928444937

66492	'Zmat2'	980	973	819	375	888	1107	924	790	-0.256825353	0.471997785	0.972790461
66493	'Mrpl51'	387	403	581	312	460	568	457	446.6666667	-0.052561027	0.895476093	0.985505807
66494	'Prelid1'	2411	2349	8145	1788	2138	3148	4301.666667	2358	-0.944505316	0.153934947	0.765411885
66495	'Ndufb3'	426	454	465	341	393	465	448.3333333	399.6666667	-0.122745151	0.699699274	0.972790461
66496	'Ppdpf'	582	615	541	272	655	764	579.3333333	563.6666667	-0.072185142	0.839150378	0.974723675
664968	'Tmem238'	13	19	23	5	39	47	18.33333333	30.33333333	0.592706209	0.466609146	0.972790461
664969	'Gm7429'	1437.6	1482.6	3213.1	0	0	0	2044.42	0	-13.45452697	1.58E-23	1.00E-19
66497	'Cmss1'	189	177	291	91	178	290	219	186.3333333	-0.312384336	0.533059089	0.972790461
66498	'Dda1'	487	544	943	895	543	716	658	718	0.21301658	0.70831137	0.972790461
664987	'Gm14393'	364.76	337.6	47.55	179.45	296.66	170.54	249.97	215.55	-0.067462798	0.923750079	0.991932584
664994	'Isoc2a'	420	473	450	172	506	558	447.6666667	412	-0.175445478	0.672354687	0.972790461
66500	'Slc30a7'	979	920	681	783.99	994.41	1089.58	860	955.9933333	0.215577159	0.407765751	0.957157474
665001	'Gm14391'	415.6	449	219.54	163.85	439.48	249.65	361.38	284.3266667	-0.310438467	0.476693997	0.972790461
66501	'1700029H14Rik'	0	1	0	1	0	0	0.333333333	0.333333333	0.058500858	0.988561293	0.999493374
665033	'Col6a5'	83.44	102.21	5	1	109.98	53.08	63.55	54.68666667	-0.254658963	0.840264514	0.974780034
66505	'Zmynd11'	2727	2627	2269	1075	3398	2441	2541	2304.666667	-0.167203189	0.664842789	0.972790461
66506	'Psmg3'	204	229	197	126	180	249	210	185	-0.16898587	0.591551518	0.972790461
66508	'Lamtor1'	891	975	1872	452	804	1181	1246	812.3333333	-0.700168511	0.178272404	0.797673555
665095	'Cyp2j8'	0	0	1	0	0	0	0.333333333	0	-0.903279821	0.824807108	0.972790461
66510	'Rnf181'	1041	1097	1986	1915	942	1344	1374.666667	1400.333333	0.138452611	0.820216866	0.972790461
66511	'Chtop'	3219	3432	3043	2047	3010	2988	3231.333333	2681.666667	-0.233580758	0.340537475	0.925308103
665113	'Tnik'	644	671	665	478	961	810	660	749.6666667	0.184485053	0.545154341	0.972790461
665119	'Sec14l5'	11	7	11	23	14	8	9.666666667	15	0.804373492	0.352616587	0.930050349
66513	'Tab1'	491	489	434	415	574	472	471.3333333	487	0.107129986	0.721024789	0.972790461
66514	'Asrgl1'	320	322	298	170	219	307	313.3333333	232	-0.410401907	0.176456538	0.796450095
66515	'Cul7'	3152	3047	2363	854	3812	2911	2854	2525.666667	-0.22812954	0.629791055	0.972790461
665155	'Srp54b'	1863.8	1743.9	6139.8	1443.4	2009.9	3904.67	3249.17	2452.646667	-0.527583298	0.440772962	0.968551476
665180	'Clec2l'	340	392	35	24	460	466	255.6666667	316.6666667	0.267861516	0.788151779	0.972790461
665181	'Gm11964'	0	0	0	0	0	1	0	0.333333333	1.020273531	0.802557913	0.972790461
665186	'Zpld2'	1	0	0	0	0	0	0.333333333	0	-0.903279821	0.824807108	0.972790461
66520	'2610001J05Rik'	331	395	448	404	429	439	391.3333333	424	0.171488387	0.658280709	0.972790461
66521	'Rwdd1'	907.42	847.61	1294	563.55	794.77	1123.67	1016.343333	827.33	-0.324152725	0.427138421	0.961670256
665211	'Gm14326'	1011.8	1083.1	399.83	432.59	1160.4	625.1	831.5633333	739.3666667	-0.114138625	0.81514332	0.972790461
66522	'Pgpep1'	717	786	1222	1416	890	1287	908.3333333	1197.666667	0.480841211	0.361610448	0.933706718
66523	'2810004N23Rik'	894	935	688	239	703	1073	839	671.6666667	-0.377112492	0.416321391	0.95722888
66525	'Timm50'	771	770	547	378	631	835	696	614.6666667	-0.164709265	0.571468605	0.972790461
66526	'Tceanc2'	531	554	486	195	508	506	523.6666667	403	-0.406660832	0.251397466	0.871230154
665270	'Plb1'	4	4	4	6	1	2	4	3	-0.187115555	0.886235664	0.984348372
66528	'Smim5'	21	25	2	6	27	6	16	13	-0.222229167	0.834984054	0.974723675
66530	'Ubxn6'	703.84	701.43	1287.7	502.51	817.8	927.76	897.64	749.3566667	-0.304963831	0.512036284	0.972790461

66531	'Cmc2'	174	182	106	193	160	216	154	189.6666667	0.416921985	0.300647031	0.904128654
66532	'Rep15'	4	5	3	24	6	11	4	13.66666667	2.018107012	0.050452257	0.519327729
66536	'Nipsnap3b'	324	385	478	428	467	436	395.6666667	443.6666667	0.217717823	0.599597648	0.972790461
66537	'Pomp'	943	1025	926	878	1077	1297	964.6666667	1084	0.210192306	0.44344168	0.969836424
66538	'Rps19bp1'	242	245	420	262	262	422	302.3333333	315.3333333	0.056005719	0.907414213	0.988823459
66540	'Fam107b'	589	563	571	352	456	443	574.3333333	417	-0.41423215	0.163436057	0.775648491
66541	'Immp1l'	490	522	605	135	472	621	539	409.3333333	-0.491636484	0.331103529	0.921648675
665433	'H2ac23'	0	4.03	2.8	0	1.23	3.38	2.276666667	1.536666667	-0.707035215	0.747941092	0.972790461
66548	'Adamtsl5'	30	23	202	74	25	53	85	50.66666667	-0.719675779	0.47962522	0.972790461
66549	'Aggf1'	1235	1430	1084	1324	1518	1782	1249.666667	1541.333333	0.367125128	0.183788363	0.804729813
66552	'Sppl2a'	1215	1242	616	1106	1469	1475	1024.333333	1350	0.486063874	0.149960756	0.759066532
66556	'Drap1'	995	1114	1205	720	885	1565	1104.666667	1056.666667	-0.072336602	0.846969406	0.975209338
665562	'Rpl31-ps12'	0	3.75	9.84	0	0	0	4.53	0	-4.522247132	0.133975414	0.733301819
665563	'Mthfd2l'	172	179	408	163	235	180	253	192.6666667	-0.41022917	0.470095592	0.972790461
66557	'Bpifb2'	1	1	0	0	0	0	0.666666667	0	-1.703500596	0.673820112	0.972790461
665574	'Gm7694'	427	404	984	1214	455	648	605	772.3333333	0.487619969	0.498745895	0.972790461
665578	'Gm7697'	200.6	196.71	17.32	19.05	124.54	71.9	138.21	71.83	-0.927202419	0.295670793	0.90029104
66559	'Metap1d'	432	526	314	247	1143	473	424	621	0.533673568	0.319724866	0.916646777
665596	'H2bc23'	10.55	11.23	24.93	15.87	5.61	1.5	15.57	7.66	-0.870539408	0.459670704	0.972790461
665622	'H2bc24'	10.55	11.23	14.88	15.87	5.61	1.5	12.22	7.66	-0.467072529	0.686135661	0.972790461
66566	'Ntpcr'	377	360	230	159	309	423	322.3333333	297	-0.114431489	0.749405062	0.972790461
66567	'2510022D24Rik'	49.86	45.96	39.88	9.63	45.94	29.17	45.23333333	28.24666667	-0.722237797	0.22366864	0.850759465
66568	'Rwdd3'	80	73	34	13	119	107	62.33333333	79.66666667	0.291961398	0.68739933	0.972790461
66569	'Gdpd1'	343	295	502	106	568	1634	380	769.3333333	0.835806589	0.288666451	0.895874398
66570	'Cenpm'	161	165	53	9	107	143	126.3333333	86.33333333	-0.618719029	0.451042882	0.970649024
665700	'Hmcn2'	218	159	12	30	187	51	129.6666667	89.33333333	-0.48818817	0.613140921	0.972790461
66573	'Dzip1'	1049	1048.4	449.92	411.41	1219	829.92	849.11	820.1066667	-0.024724749	0.956497269	0.999493374
665738	'Gm7762'	1	3	1	0	4	11	1.666666667	5	1.472418774	0.38164007	0.94429001
665755	'LOC665755'	0	1	0	0	0	0	0.333333333	0	-0.903279821	0.824807108	0.972790461
66576	'Uqcrh'	1527	1580	1600	2599	1266	1794	1569	1886.333333	0.427815003	0.415902536	0.95722888
665775	'Bod1l'	2794	2884	1485	1400	2494	2085	2387.666667	1993	-0.200168024	0.543012815	0.972790461
66578	'Mis18a'	188	201	104	150	215	228	164.3333333	197.6666667	0.3336312	0.326783988	0.920734892
665780	'Zscan4b'	0	1	0	0	0	0	0.333333333	0	-0.903279821	0.824807108	0.972790461
66580	'Esf1'	1577	1579	1214	786	1148	1113	1456.666667	1015.666667	-0.469962321	0.048536068	0.511851691
665828	'Gm10654'	3	2	9	9	9	4	4.666666667	7.333333333	0.696631946	0.538415531	0.972790461
66583	'Exosc1'	290	281	232	179	322	445	267.6666667	315.3333333	0.230638436	0.480868338	0.972790461
66585	'Snrnp40'	881	954	679	291	801	780	838	624	-0.445305074	0.217812912	0.844165262
665858	'Gm7827'	1.18	6.45	0	2	4	6	2.543333333	4	0.845148332	0.59398132	0.972790461
66586	'Crls1'	426	494	726	308	605	781	548.6666667	564.6666667	-0.021554215	0.961814883	0.999493374
66587	'Fastk'	1009	1030	1005	554	995	1039	1014.666667	862.6666667	-0.233383659	0.399960497	0.955860942

66588	'Cmpk1'	1188	1275	2122	1211	1285	1573	1528.333333	1356.333333	-0.159668348	0.727907985	0.972790461
66589	'Ube2v1'	2791	2761	3710	2733	2437	3130	3087.333333	2766.666667	-0.103356649	0.801287007	0.972790461
66590	'Farsa'	762	687	938	438	732	817	795.6666667	662.3333333	-0.281993952	0.429662161	0.962386546
66591	'Mad2l1bp'	202	218	512	278	225	283	310.6666667	262	-0.223646871	0.709496554	0.972790461
66592	'Stoml2'	820	862	1296	478	777	1220	992.6666667	825	-0.323038675	0.468768621	0.972790461
66593	'Diablo'	1123	1142	982.97	678.9	1264	1719.99	1082.636667	1220.96	0.15895124	0.615242567	0.972790461
66594	'Uqcr11'	570	636	527	430	395	657	577.6666667	494	-0.16817531	0.627520257	0.972790461
665943	'E330014E10Rik'	0	0	0	0	0	4.76	0	1.586666667	2.683352581	0.500796233	0.972790461
66595	'Aste1'	210	179	161	162	224	192	183.3333333	192.6666667	0.131742068	0.679129972	0.972790461
66596	'Gtf3a'	468.65	485.09	458.74	170.43	492.89	705.12	470.8266667	456.1466667	-0.113622229	0.802645492	0.972790461
66597	'Trim13'	173.79	209.74	135	86.85	257.57	204	172.8433333	182.8066667	0.071117407	0.860020714	0.976863639
66599	'Rdm1'	329	352	150	62	268	254	277	194.6666667	-0.532665192	0.324443254	0.919683661
666002	'Vmn2r114'	0	0	0	0	0	1	0	0.333333333	1.020273531	0.802557913	0.972790461
66601	'Tmigd1'	1	3	0	0	1	1	1.333333333	0.666666667	-0.967023082	0.703869976	0.972790461
66603	'Gemin2'	389	441	393	155	335	365	407.6666667	285	-0.534908521	0.1050005	0.677620334
666040	'Gm7903'	1049.1	985.1	83.14	3	464.29	756.63	705.7633333	407.9733333	-0.858363119	0.495434429	0.972790461
666048	'Trabd2b'	233	193	229	106	345	270	218.3333333	240.3333333	0.089256602	0.838005865	0.974723675
66606	'Lrrc57'	175	194	216	117	219	254	195	196.6666667	-0.01022788	0.977585627	0.999493374
666060	'Frmpd1'	257	329	39	95	343	324	208.3333333	254	0.317676698	0.664815396	0.972790461
66607	'Ms4a4d'	10	11	4	101	21	17	8.333333333	46.33333333	2.852244402	0.004909426	0.165492906
66609	'Cryzl1'	550	605	543	300	673	624	566	532.3333333	-0.100688837	0.749582619	0.972790461
66610	'Abi3'	44.52	37.74	36.61	257.49	52.08	58.36	39.62333333	122.6433333	1.969054864	0.021445217	0.362574735
666105	'Gm7932'	16	11	9	4	8	8	12	6.666666667	-0.822256862	0.28821864	0.895361566
66611	'Ribc1'	326	298	67	9	145	215	230.3333333	123	-0.971300684	0.287898581	0.894952226
66612	'Ormdl3'	1124	1191	962	3125	1454	1921	1092.333333	2166.666667	1.180852871	0.028648601	0.412002421
66614	'Gpatch4'	238	220	280	59	284	253	246	198.6666667	-0.405346448	0.455710282	0.971239019
666145	'Armh2'	1	0	0	0	1	0	0.333333333	0.333333333	0.058500858	0.988561293	0.999493374
66615	'Atg4b'	1395	1521	1260	1536	1583	2121	1392	1746.666667	0.390724355	0.19117927	0.813837363
66616	'Snx9'	1127	1135	1079	2803	1366	1893	1113.666667	2020.666667	1.029626591	0.049305899	0.515210762
66617	'Ntmt1'	304	327	830	541	333	456	487	443.3333333	-0.084563903	0.897077706	0.985794067
666173	'Vps13b'	2972	2969	945	699	2377	2104	2295.333333	1726.666667	-0.398485597	0.45495713	0.970934082
66618	'Snrnp27'	363	342	492	336	356	426	399	372.6666667	-0.066563301	0.869767586	0.979794316
666184	'Gm15080'	6.9	0	0	0	0	0	2.3	0	-3.287206351	0.405794607	0.957157474
666190	'Gm7972'	681	564	315	24	616	567	520	402.3333333	-0.471234185	0.585653361	0.972790461
666207	'Gm7980'	1.98	0	0	0	0	0	0.66	0	-0.903279821	0.824807108	0.972790461
666214	'1700049E17Rik1'	2	3.39	0	0	4.98	2.83	1.796666667	2.603333333	0.247581103	0.905944602	0.988794924
66622	'Ubr7'	1980	1950	2007	1413	1528	2262	1979	1734.333333	-0.156251262	0.624020675	0.972790461
666233	'Gm7995'	1	0	0	0	1	0	0.333333333	0.333333333	0.058500858	0.988561293	0.999493374
66624	'Spcs2'	1905	2039	3918	1834	2433	4396	2620.666667	2887.666667	0.074086378	0.887066679	0.984534762
666244	'Tmsb15b1'	15	22.12	14.41	25.46	38.6	29.52	17.17666667	31.19333333	0.911098412	0.092964411	0.654455509

66625	'Pnisr'	3951	3982	1416	2177	5091	2206	3116.333333	3158	0.104586139	0.841262831	0.975182082
666257	'Zfp660'	340	365	120	81	359	174	275	204.6666667	-0.409180722	0.491297294	0.972790461
66626	'Cdip1'	2389.2	2603.9	2295.2	1593.8	2002.3	1870	2429.426667	1822.033333	-0.353404203	0.218018506	0.84418897
66627	'Ogfod2'	232	241	440	371	219	332	304.3333333	307.3333333	0.089336104	0.876692019	0.981341203
666279	'Dspp'	0	0	2	0	2	1	0.666666667	1	0.294408378	0.92562677	0.992381554
66628	'Thg1l'	315	324	396	116	321	267	345	234.6666667	-0.603936341	0.146920242	0.753611197
66629	'Golph3'	2595	2782	5845	5168	2797	3768	3740.666667	3911	0.153498084	0.80602989	0.972790461
66631	'Mfsd14b'	924	968	614	1086	1369	1579	835.3333333	1344.666667	0.754394668	0.005696215	0.178006489
666311	'Zscan25'	389	399	659	171	365	418	482.3333333	318	-0.668768901	0.150953958	0.759755159
66632	'Dph6'	474	451	314	301	364	379	413	348	-0.169612466	0.564574758	0.972790461
666329	'Gm3317'	27.92	31.91	18.1	1.23	7.12	6.35	25.97666667	4.9	-2.453945794	0.001722502	0.089596578
666339	'Muc3'	0	1	1	0	0	1	0.666666667	0.333333333	-0.887055159	0.80631676	0.972790461
66634	'Mcm8'	1022	1025	287	56	672	922	778	550	-0.568596666	0.488578523	0.972790461
666348	'Apol7e'	12.69	11.68	11	9	28	11	11.79	16	0.502153379	0.49810283	0.972790461
66637	'Tsen15'	420	482	261	104	450	354	387.6666667	302.6666667	-0.38755971	0.431727118	0.963677723
66641	'Sike1'	554	644	866	772	718	763	688	751	0.18625981	0.67442367	0.972790461
66642	'Ctnnbl1'	814	825	698	321	649	790	779	586.6666667	-0.422184143	0.174076035	0.793056387
666420	'Gm8094'	1	1	0	0	1	0	0.666666667	0.333333333	-0.74517548	0.838722495	0.974723675
66643	'Lix1'	155.28	158.99	42.79	18.53	177.46	175.95	119.02	123.98	0.012591797	0.987179465	0.999493374
66645	'Pspc1'	1404	1549	682	722	1527	1305	1211.666667	1184.666667	0.012699528	0.97315072	0.999493374
66646	'Rpe'	654.89	634.3	641.55	583.41	738.07	1032	643.58	784.4933333	0.30672726	0.305598322	0.907188578
666468	'Atg4a'	233	219	208	226	244	310	220	260	0.29641855	0.351532539	0.929144534
66647	'Nsmce3'	448	453	758	517	390	575	553	494	-0.115062809	0.81857274	0.972790461
66648	'Tpgs2'	784	790	404	99	556	595	659.3333333	416.6666667	-0.718607893	0.218256066	0.844387594
66650	'Nepn'	0	4	1	0	0	0	1.666666667	0	-3.078285289	0.437135169	0.96622803
666501	'Gm8137'	0	0	0	0.5	1.7	1	0	1.066666667	1.775692139	0.660844521	0.972790461
666504	'Tmem249'	2	2	0	2	0	0	1.333333333	0.666666667	-0.498389332	0.866434724	0.978294615
666528	'Zfp541'	1980	1858	213	5	951	706	1350.333333	554	-1.341852577	0.268539932	0.883600995
66653	'Brf2'	211	231	261	87	207	295	234.3333333	196.3333333	-0.318849637	0.472468542	0.972790461
666532	'Zfp991'	640.74	741.46	921.81	507.27	1764.9	1313.9	768.0033333	1195.366667	0.566997673	0.22692835	0.854001521
66654	'Tex12'	336	323	257	1	122	151	305.3333333	91.33333333	-1.885635483	0.070431109	0.589465353
66656	'Eef1d'	2450	2457	3608	2498	2183	2889	2838.333333	2523.333333	-0.12250276	0.779560643	0.972790461
666561	'Gm8165'	1	2.43	0	0	0	0	1.143333333	0	-2.28103714	0.568641838	0.972790461
666574	'Gm13144'	0	0	0.77	0	1.19	3	0.256666667	1.396666667	2.701386663	0.497818139	0.972790461
66658	'Ccdc51'	105	139	90	106	187	200	111.3333333	164.3333333	0.58521694	0.074767928	0.603900623
666584	'BC024063'	219.58	248	147.69	84.55	221.77	214.18	205.09	173.5	-0.24470247	0.524062536	0.972790461
66659	'Acp6'	385	326	461	387	268	374	390.6666667	343	-0.100650035	0.830772354	0.974324192
66660	'Sltm'	1707	1593	1298	1264	1955	1370	1532.666667	1529.666667	0.061995302	0.842681907	0.975182082
66661	'Srp72'	3504	3466	7721	1576	2972	4851	4897	3133	-0.751101587	0.182939541	0.804092742
66663	'Uba5'	1005	994	1800	619	943	928	1266.333333	830	-0.632590917	0.165927501	0.780651734

66664	'Tmem41a'	231	243	123	125	225	225	199	191.6666667	-0.007974385	0.981915919	0.999493374
66665	'Msantd3'	477	468	489	189	411	543	478	381	-0.368145096	0.325190858	0.920156064
66667	'Hspbap1'	184	175	213	181	239	211	190.6666667	210.3333333	0.180698329	0.622573607	0.972790461
666675	'Gm8229'	1.26	1	0	0	2	0	0.753333333	0.666666667	0.038821536	0.990916081	0.999493374
666678	'Gm8232'	1.99	1.91	0	0	0	0	1.3	0	-1.703500596	0.673820112	0.972790461
666704	'Samd1'	696	722	863	231	741	670	760.3333333	547.3333333	-0.541516285	0.216951974	0.843391934
666706	'Gm8246'	0	0	1	0	0	0	0.333333333	0	-0.903279821	0.824807108	0.972790461
66671	'Ccnh'	972	1003	498	441	979	1224	824.3333333	881.3333333	0.104669403	0.790234004	0.972790461
66673	'Sorcs3'	118	82	29	6	152	154	76.33333333	104	0.370563946	0.693480085	0.972790461
666731	'Trim43c'	159.22	161.04	26	95	96	55.01	115.42	82.00333333	-0.280748272	0.699023262	0.972790461
666738	'Gm8264'	0	0	0	0.5	1.7	1	0	1.066666667	1.775692139	0.660844521	0.972790461
66674	'Spryd7'	478	491	669	280	357	463	546	366.6666667	-0.574351248	0.135420224	0.736012522
666747	'Trim43b'	13.78	10.96	5	0	3	0.99	9.913333333	1.33	-3.216088686	0.036982178	0.459648634
666750	'Gm8271'	2.81	0	0	0	0	0	0.936666667	0	-1.711373851	0.67234292	0.972790461
66676	'Tmed7'	1968	1775	2063	3639	2516	3400	1935.333333	3185	0.823556103	0.058596096	0.552046072
666765	'Gm8279'	12.46	18.96	3.42	5	3	4	11.61333333	4	-1.282120787	0.215280851	0.842858223
66679	'Rae1'	1334	1254	1356	862	1022	1299	1314.666667	1061	-0.276010871	0.363141271	0.935009831
666790	'Gm8290'	62.02	62	15	21	89.14	24	46.34	44.71333333	0.001193727	0.998769802	0.999900097
666794	'Rbm24'	93	77	39	51	128	121	69.66666667	100	0.541056414	0.241575349	0.86449675
66680	'Oser1'	962	949	2029	864	716	1117	1313.333333	899	-0.538681922	0.324826041	0.91982931
666808	'Gm10410'	0	8.38	0	0	0	0	2.793333333	0	-3.683367796	0.350209853	0.92886351
66681	'Pgm2'	455	447	872	440	453	420	591.3333333	437.6666667	-0.402590811	0.437289975	0.96622803
66682	'Trappc5'	578.99	603.25	1320.6	394.56	588.36	758.34	834.27	580.42	-0.583548154	0.273438128	0.884738928
66684	'Tceal8'	983	1007	2465	703	1232	1158	1485	1031	-0.590528555	0.291876334	0.89958198
666842	'Gm14692'	719.78	841.66	153	8	461.75	738.16	571.48	402.6366667	-0.585575826	0.589720511	0.972790461
66686	'Dcbld1'	428	454	66	106	825	741	316	557.3333333	0.800560779	0.318964155	0.916136994
66687	'Tbc1d15'	1372	1427	4247	3310	1455	2281	2348.666667	2348.666667	0.080272342	0.912524083	0.989773583
66689	'Klhl28'	542	557	777	1030	516	537	625.3333333	694.3333333	0.312424895	0.600129295	0.972790461
66690	'Tmem186'	539	577	335	212	434	439	483.6666667	361.6666667	-0.396702297	0.215906426	0.843391934
666907	'Ms4a4a'	1	0	0	0	2	12	0.333333333	4.666666667	3.535536324	0.166545564	0.780893009
66691	'Gapvd1'	3386.4	3384	1715	1695	3027	2592	2828.46	2438	-0.153820985	0.646231452	0.972790461
666926	'Gm8369'	0	0	0	0	1	0	0	0.333333333	1.020273531	0.802557913	0.972790461
666938	'Bend4'	925	878	305	96	273	479	702.6666667	282.6666667	-1.330226845	0.022345544	0.370441983
66694	'Uqcrfs1'	1128	1143	1867	1426	1112	1473	1379.333333	1337	0.010503738	0.982861615	0.999493374
66695	'Aspn'	64.43	61.81	5	32	143.96	57	43.74666667	77.65333333	0.89516298	0.312339632	0.911537035
66696	'Snx31'	14	13	5	3	13	6	10.66666667	7.333333333	-0.514550495	0.567409331	0.972790461
66700	'Chmp3'	1490	1517	1288	934	1609	1847	1431.666667	1463.333333	0.040261951	0.87406852	0.980171118
66701	'Spryd4'	181	207	180	205	252	288	189.3333333	248.3333333	0.438230355	0.14852827	0.75695799
667034	'Pnp2'	6.33	4.59	14.38	3.3	5.36	6.21	8.433333333	4.956666667	-0.841244472	0.415525359	0.95722888
66704	'Rbm4b'	1165	1153.1	649.31	611.72	1174.8	589.31	989.1433333	791.9433333	-0.242652466	0.561629132	0.972790461

66705	'Dnase1l2'	25	29	29	24	31	23	27.66666667	26	-0.031684178	0.952345237	0.998730633
667055	'Unc93a2'	3.9	0	11.88	0	0	4.85	5.26	1.616666667	-2.105434151	0.38742576	0.949501169
66706	'Ndufaf3'	94	83	92	86	133	130	89.66666667	116.3333333	0.39756938	0.251673223	0.871252558
667060	'Vmn2r5'	5.89	4	0	3.08	12	1	3.296666667	5.36	0.934924642	0.55369444	0.972790461
667069	'Vmn2r6'	1	0	0	1	0	0	0.333333333	0.333333333	0.058500858	0.988561293	0.999493374
66707	'Nkapl'	29	30	12	13	24	23	23.66666667	20	-0.186636335	0.75172	0.972790461
667103	'Gm13570'	0	0	1	0	0	0	0.333333333	0	-0.903279821	0.824807108	0.972790461
66711	'Sbds'	589	574	2151	757	506	853	1104.666667	705.3333333	-0.657740646	0.362663583	0.934677306
667118	'Zbed6'	1586	1523	441	3066	1649	1248	1183.333333	1987.666667	1.026460973	0.129088335	0.726020415
66712	'Spesp1'	41	47	3	1	19	29	30.33333333	16.33333333	-0.929825418	0.424021718	0.960285372
66713	'Actr2'	2528	2656	3077	3803	2598	3023	2753.666667	3141.333333	0.306014301	0.509178988	0.972790461
66714	'4921524J17Rik'	407	418	1125	974	560	838	650	790.6666667	0.332646127	0.617541296	0.972790461
66715	'Henmt1'	139	132	6	2	97	175	92.33333333	91.33333333	-0.072599065	0.953458463	0.999071445
66716	'Ccer1'	13	15	2	0	2	1	10	1	-3.286297437	0.026147347	0.397137149
66717	'Ccdc96'	112	103	16	7	57	24	77	29.33333333	-1.379492745	0.113406097	0.698948431
66720	'Klhl10'	23	28	16	0	34	18	22.33333333	17.33333333	-0.471079507	0.678969386	0.972790461
667214	'9930111J21Rik1	4.77	4.18	4.68	18	8.51	10.9	4.543333333	12.47	1.757287352	0.065950848	0.574593252
66722	'Spag16'	55	39	11	1	38	21	35	20	-0.856538623	0.406804515	0.957157474
66724	'Tab3'	1662	1664	1277	524	1160	1343	1534.333333	1009	-0.617206269	0.053102023	0.532859388
66725	'Lrrk2'	201	204	284	1437	298	366	229.6666667	700.3333333	1.886232025	0.020871712	0.358627562
667250	'Gm12657'	264.33	284.71	365.9	162.34	288.11	262.98	304.98	237.81	-0.36923792	0.329477731	0.921648675
66727	'Plaat5'	3	1	1	0	0	1	1.666666667	0.333333333	-2.129460607	0.391245715	0.952371379
667277	'C1rb'	4.59	4.89	15.48	1.37	4.02	1.91	8.32	2.433333333	-2.031741066	0.125036031	0.719486994
667281	'H60b'	19	27	9	8	27	35	18.33333333	23.33333333	0.33770902	0.632561303	0.972790461
66729	'Ankrd61'	22	29	9	8	18	13	20	13	-0.560611114	0.419873077	0.958308933
66733	'Kcng4'	466	546	23	8	711	575	345	431.3333333	0.278816725	0.820552231	0.972790461
66734	'Map1lc3a'	398	401	655	1026	584	843	484.6666667	817.6666667	0.851750685	0.123337181	0.715310266
66736	'Emc2'	847	926	703	343	892	872	825.3333333	702.3333333	-0.252212047	0.463961098	0.972790461
667370	'Ifit3b'	26.42	26.14	67.35	0	36.57	103.09	39.97	46.55333333	-0.030147802	0.98135676	0.999493374
667373	'Ifit1bl1'	7	1	6	5	8	4	4.666666667	5.666666667	0.311145683	0.785868164	0.972790461
667414	'Gm8618'	2.87	2	0	0	0	0	1.623333333	0	-2.696289772	0.497898105	0.972790461
66743	'Rnf220'	1279	1327	681	1233	1230	1643	1095.666667	1368.666667	0.42218896	0.24072591	0.86449675
66745	'Trpd52l3'	19	13	0	0	8	19	10.66666667	9	-0.298379872	0.861718744	0.976863639
66748	'Erich2'	38	37	8	5	15	15	27.66666667	11.66666667	-1.205103514	0.124124746	0.716775888
66753	'Erlec1'	1511	1376.1	683.04	958	1420	1323	1190.033333	1233.666667	0.133643254	0.70550635	0.972790461
66755	'Ppp1r14bl'	10	3	2	0	4	2	5	2	-1.353777808	0.372763985	0.938916841
667552	'Gm8700'	1	1.45	0	0	5.42	13.07	0.816666667	6.163333333	3.081014347	0.1302001	0.727620313
66756	'Cfap97'	1316	1341	739	421	1157	999	1132	859	-0.392709052	0.304912716	0.907188578
66757	'Adat2'	161.17	162.66	238.42	20	86.79	114.43	187.4166667	73.74	-1.47031516	0.013983992	0.293124205
66758	'Zfp474'	0	0	0	0	1	0	0	0.333333333	1.020273531	0.802557913	0.972790461

66761	'4933417A18Rik'	0	1	0	0	1	0	0.333333333	0.333333333	0.058500858	0.988561293	0.999493374
66763	'Nt5el'	63	55	7	0	27	43	41.66666667	23.33333333	-0.903740453	0.474173567	0.972790461
66765	'4933411K16Rik'	6	7	3	4	8	5	5.333333333	5.666666667	0.149682313	0.876858962	0.981341203
66766	'Tmem239'	1	2	3	5	4	0	2	3	0.752660317	0.657433868	0.972790461
667663	'Myo3a'	4	7	3	0	2	1	4.666666667	1	-2.239648281	0.149573271	0.758505335
667666	'Zfp600'	79.99	62.51	28.7	1.89	12.96	0	57.06666667	4.95	-3.685693257	0.001071275	0.068807711
66768	'Pacrgl'	211	190	128	36	155	198	176.3333333	129.6666667	-0.502715213	0.357360124	0.932505998
667693	'Gm8765'	0	0.98	0	0	4.1	12.86	0.326666667	5.653333333	4.692330816	0.104428516	0.67684333
667705	'Gm8773'	1	4	4	2	3	0	3	1.666666667	-0.761896935	0.650910582	0.972790461
66771	'Gid4'	2876	2605	2718	1205	1589	2890	2733	1894.66	-0.538222463	0.135242479	0.736012522
66772	'Asb17'	2	4	0	0	0	1	2	0.333333333	-2.319703775	0.423543302	0.960285372
66773	'Gm17019'	0	1	0	81.79	2	24.96	0.333333333	36.25	6.983357067	1.32E-04	0.017014102
667742	'Piezo2'	507	462	383	17	538	262	450.6666667	272.3333333	-0.843695854	0.332667772	0.921648675
66775	'Hacd4'	194.49	201.46	30.83	44.91	211.7	277.96	142.26	178.19	0.314725714	0.684381098	0.972790461
667772	'Myh15'	7	6	2	0	3	1	5	1.333333333	-1.91276668	0.21243453	0.840892212
667803	'H2-T-ps'	26.19	22.32	150.58	47.97	10.16	27.11	66.36333333	28.41333333	-1.158393158	0.26844779	0.883600995
667823	'Trim5'	96.97	134.55	81.38	23	46.73	19.02	104.3	29.58333333	-1.755749924	6.97E-04	0.052467355
66784	'Fam187a'	0	0	1	1	2	1	0.333333333	1.333333333	1.935874595	0.443898409	0.969880311
66786	'Olfr701'	0	0	0	1	0	0	0	0.333333333	1.020273531	0.802557913	0.972790461
66787	'Gskip'	382	434	440	436	398	496	418.6666667	443.3333333	0.153508734	0.68029378	0.972790461
66789	'Alg14'	126	105	65	279	158	206	98.66666667	214.3333333	1.30267886	0.013492025	0.290170111
66790	'Grtp1'	163	180	149	118	190	164	164	157.3333333	-0.022065551	0.942364641	0.996301887
667929	'Gm8882'	0	0	0	0	0	1	0	0.333333333	1.020273531	0.802557913	0.972790461
66793	'Efcab1'	61	50	0	4	35	15	37	18	-1.008779055	0.456951071	0.971964785
66795	'Atg10'	108	131	100	37	190	188	113	138.3333333	0.216549958	0.697652232	0.972790461
667962	'Zfp966'	0	8.09	5.42	0	0	0	4.503333333	0	-4.519585931	0.126449687	0.721271206
66797	'Cntnap2'	32.76	30.52	30.68	11	15.94	8	31.32	11.64666667	-1.368409108	0.025546144	0.395185754
667977	'Gm8909'	45.52	42.55	86.68	27.16	11.83	9.04	58.25	16.01	-1.71930882	0.03426161	0.444932876
667988	'Mageb6b2'	4.32	6	0	0	0.5	10.44	3.44	3.646666667	-0.069857874	0.977413285	0.999493374
66799	'Ube2w'	959	952	1211	539	1046	1355	1040.666667	980	-0.131398386	0.731232109	0.972790461
66801	'Prkrip1'	425	404	289	173	355	458	372.6666667	328.6666667	-0.185955151	0.586325977	0.972790461
668039	'Gm14434'	68.67	123.72	0	47.11	105.55	15.45	64.13	56.03666667	-0.007567105	0.995555424	0.999562152
66805	'Tspan1'	11	27	1	49	50	46	13	48.33333333	2.079725071	0.02204962	0.36727039
668050	'Gm8947'	0.09	1.97	2	1	0	0	1.353333333	0.333333333	-1.521952424	0.635355647	0.972790461
66808	'9030624G23Rik'	296.79	310.16	135.2	71.42	253.53	212.7	247.3833333	179.2166667	-0.469963366	0.339876054	0.92508569
66809	'Krt20'	81	73	128	80	124	59	94	87.66666667	-0.070463189	0.900391815	0.986604124
66810	'Rbm22'	871	843	1196	532	845	860	970	745.6666667	-0.389370193	0.296955297	0.900576504
668101	'Sirpb1b'	0	1.77	0	0	0	2.49	0.59	0.83	0.78767502	0.845746136	0.975182082
668106	'Gm8978'	2	0	0	0	0	0	0.666666667	0	-1.711373851	0.67234292	0.972790461
66811	'Duoxa2'	0	0	0	3	0	0	0	1	2.899270347	0.465759809	0.972790461

668110	'Syce1l'	0	3	0	0	2	5	1	2.333333333	1.170421399	0.665042279	0.972790461
66812	'Ppcdc'	3348.7	3368.2	507.31	364	2580	1354.99	2408.083333	1432.996667	-0.742108017	0.339120414	0.9248827
66813	'Bcl2l14'	1	1	0	3	4	2	0.666666667	3	2.308629451	0.199359586	0.826477202
66815	'Mcub'	5	8	247	103	16	96	86.66666667	71.66666667	-0.304361919	0.825331402	0.972790461
668158	'Ccdc85c'	941.56	880.45	405.95	519.56	615.3	936.77	742.6533333	690.5433333	-0.031694922	0.935958622	0.994413066
66816	'Thap2'	347	369	443	321	349	318	386.3333333	329.3333333	-0.16916227	0.669104516	0.972790461
668166	'Zxdb'	815	759	639	237	673	908	737.6666667	606	-0.334401558	0.433077691	0.964631919
66817	'Tmem170'	398	358	292	515	291	385	349.3333333	397	0.348755625	0.474714789	0.972790461
668173	'Pex10'	138	167	134	51	113	135	146.3333333	99.66666667	-0.572066659	0.129869057	0.727578716
668178	'Mettl7a3'	1	0	0	1	1	2	0.333333333	1.333333333	1.92140273	0.452182668	0.970649024
66818	'Smim7'	950	1105	1302	841	1265	1403	1119	1169.666667	0.062448125	0.856069965	0.975734242
668205	'Gm9045'	0	0	0	0	0	1.69	0	0.563333333	1.020273531	0.802557913	0.972790461
668208	'Gm13288'	2.01	6.52	0	5.69	1.3	5.85	2.843333333	4.28	0.691825808	0.669587133	0.972790461
66821	'Bcs1l'	316	314	243	178	250	355	291	261	-0.132623176	0.656114477	0.972790461
668212	'Efr3b'	360	313	161	56	309	219	278	194.6666667	-0.546276984	0.33904412	0.9248827
668216	'Gm9049'	0	0	0	0	5.17	1.7	0	2.29	3.323592684	0.383986269	0.945936404
668218	'Bin2'	163	170	53	7	59	166	128.6666667	77.33333333	-0.818609279	0.356850824	0.932313848
66822	'Fbxo25'	741	782	615	442	864	907	712.6666667	737.6666667	0.055018085	0.840274214	0.974780034
668225	'Fignl2'	503	459	321	361	400	279	427.6666667	346.6666667	-0.179766222	0.655348802	0.972790461
66824	'Pycard'	114	125	53	40	164	166	97.33333333	123.3333333	0.318337665	0.565479884	0.972790461
66825	'Rnf186'	1	2	0	1	1	0	1	0.666666667	-0.334057484	0.89800003	0.985794067
66826	'Taz'	793	778	312	448	898	730	627.6666667	692	0.202966486	0.625659323	0.972790461
66827	'Ttc1'	859	828	1104	776	741	1040	930.3333333	852.3333333	-0.088831473	0.822174714	0.972790461
66830	'Nacc1'	2491	2423	1662	1348	2765	3492	2192	2535	0.207055652	0.523827389	0.972790461
668303	'Kif26a'	712	638	502	328	653	598	617.3333333	526.3333333	-0.211915147	0.450118745	0.970590732
668310	'Cc2d2b'	56	72	4	1	49	44	44	31.33333333	-0.529259076	0.659421646	0.972790461
66832	'Rsph3a'	1003.6	1029.9	1631.3	314.79	822.73	1083.01	1221.636667	740.1766667	-0.818886593	0.099261974	0.668798985
668339	'Gm9112'	0	1	0	0	0	0	0.333333333	0	-0.903279821	0.824807108	0.972790461
66834	'Acot13'	198	212	547	357	234	291	319	294	-0.068470686	0.916983763	0.990536123
668357	'Dmrtc1c2'	357.21	205.21	60.16	0	60.29	154.63	207.5266667	71.64	-1.636258088	0.196824105	0.822696823
66836	'Tmem223'	206	236	89	70	323	283	177	225.3333333	0.327614671	0.563865495	0.972790461
668382	'Pabpc1l2b'	395.74	458.08	263.61	65.49	165.52	231.23	372.4766667	154.08	-1.296286654	0.002562021	0.113806392
66839	'Dele1'	576	591	648	208	699	605	605	504	-0.325013772	0.453259647	0.970649024
66840	'Wdr45b'	1694	1702	1561	1160	1503	1912	1652.333333	1525	-0.084492873	0.747308528	0.972790461
66841	'Etfdh'	1488	1751	1507	1180	1968	2266	1582	1804.666667	0.196599176	0.466243597	0.972790461
668433	'4930544D05Rik'	0	0	0	1	0	0	0	0.333333333	1.020273531	0.802557913	0.972790461
66844	'Ormdl2'	158.27	160.37	407.35	151.37	174.22	314.84	241.9966667	213.4766667	-0.249422542	0.680928249	0.972790461
66845	'Mrpl33'	294	340	272	182	297	420	302	299.6666667	-0.011112237	0.972334173	0.999493374
668455	'Mrto4-ps1'	0	0	0	0	1	0	0	0.333333333	1.020273531	0.802557913	0.972790461
66847	'Hint3'	914	853.91	344	285	869.21	742.44	703.97	632.2166667	-0.139982246	0.768881543	0.972790461

66848	'Fuca2'	325	357	208	480	577	657	296.6666667	571.3333333	1.023912797	8.32E-04	0.059718399
66849	'Ppp1r2'	1411	1454	2099	2399	1613	1969	1654.666667	1993.666667	0.365573496	0.465226304	0.972790461
668501	'Zfp507'	2379	2229	1205	696	1683	1668	1937.666667	1349	-0.509469399	0.171441692	0.787518803
668525	'Gm9222'	841.69	760.36	751.99	245.87	715.48	556.69	784.68	506.0133333	-0.663102874	0.078847949	0.616000706
66853	'Pnpla2'	284	321	793	649	417	587	466	551	0.283556674	0.660022992	0.972790461
66854	'Trim35'	4051	4249	4745	3361	4984	4826	4348.333333	4390.333333	0.032908579	0.914378813	0.989964366
668548	'Gm9234'	2.6	6.11	3.26	0.62	2	4.74	3.99	2.453333333	-0.960322284	0.530457488	0.972790461
66855	'Tcf25'	5098	4752	8344	1778	4843	5255	6064.666667	3958.666667	-0.707570878	0.149379771	0.758505335
668553	'Gm9237'	44.55	34.68	100.31	34.06	49.38	52.41	59.84666667	45.28333333	-0.445823547	0.488893293	0.972790461
668555	'Gm9239'	0	0	0	0	0	1	0	0.333333333	1.020273531	0.802557913	0.972790461
66857	'Plbd1'	95	118	29	34	277	122	80.66666667	144.3333333	0.827057292	0.270566655	0.883851074
668588	'Gm9257'	24.41	22.19	22.45	17.27	27.51	38.12	23.01666667	27.63333333	0.272713132	0.599563798	0.972790461
66859	'Slc16a9'	155	170	48	26	244	275	124.3333333	181.6666667	0.492434085	0.523041729	0.972790461
66860	'Tanc1'	1908	1867	1629	2097	2159	2320	1801.333333	2192	0.366676644	0.239926462	0.86449675
66861	'Dnajc10'	3269	3219	1091	1869	2723	3243	2526.333333	2611.666667	0.129613589	0.769828188	0.972790461
668620	'Zfp936'	46.16	64.2	14.96	3	52	69.02	41.77333333	41.34	-0.065080804	0.945306512	0.996592403
66863	'Lztr1'	1205	1265	1238	1129	1590	1450	1236	1389.666667	0.210105003	0.462356738	0.972790461
66864	'Clec14a'	198	232	33	5	201	85	154.3333333	97	-0.709618776	0.492410427	0.972790461
66865	'Pmpca'	2048.1	2303.4	1855.3	1482	2306.5	2434.46	2068.923333	2074.32	0.031905252	0.891964684	0.985171
66866	'Nhlrc2'	1147	1206	930	1287	1216	1006	1094.333333	1169.666667	0.22110675	0.571808641	0.972790461
668661	'2410002F23Rik'	1607	1569.9	2124	359.94	1476	1305	1766.936667	1046.973333	-0.850644809	0.093046141	0.654455509
66867	'Hmg20a'	1755	1687	1126	1306	1622	1339	1522.666667	1422.333333	-0.001790081	0.995586503	0.999562152
66868	'Mfsd1'	1356	1263	813	1187	1551	2105	1144	1614.333333	0.548004119	0.061545567	0.563362693
66869	'Zfp869'	895.47	872.42	1463	503	918	970	1076.966667	797	-0.478229151	0.271849325	0.884539076
66870	'Serbp1'	7084	7387.4	13482	6955.9	7501.1	10133.62	9317.836667	8196.866667	-0.19060707	0.690521127	0.972790461
66871	'Cpne8'	364.02	384	580	403	223.06	278	442.6733333	301.3533333	-0.434818495	0.442565142	0.969472114
66873	'Tril'	692	631	32	41	977	701	451.6666667	573	0.31453343	0.771287192	0.972790461
66874	'Ncbp3'	1098	1040	719	718	1045	1215	952.3333333	992.6666667	0.10492755	0.682603721	0.972790461
66875	'Swt1'	1142	1147	678	860	880	908	989	882.6666667	-0.051101401	0.88343039	0.983244267
66877	'Crnkl1'	895	835	570	922	819	940	766.6666667	893.6666667	0.339038027	0.350289789	0.92886351
66878	'Riok3'	1498	1535	4451	1609	1575	1536.06	2494.666667	1573.353333	-0.66378931	0.295268356	0.90029104
66880	'Rsrc1'	3412.5	3424.3	901.12	607.66	2543.4	1755.33	2579.283333	1635.45	-0.642316437	0.284494511	0.893132373
66881	'Pcyox1'	2359	2235	962	3722	3358	3424	1852	3501.333333	1.072544905	0.016377587	0.320010986
668814	'Gm9376'	0	0	1	1	0	0	0.333333333	0.333333333	0.058500858	0.988561293	0.999493374
66882	'Bzw1'	7693	8087	8735	6076	6647	8808	8171.666667	7177	-0.150346981	0.643329434	0.972790461
66884	'Appbp2'	1596	1615	1614	1701	1457	1796	1608.333333	1651.333333	0.125538302	0.733198248	0.972790461
66885	'Acadsb'	1416	1537	2466	1115	1654	1960	1806.333333	1576.333333	-0.224725581	0.602915167	0.972790461
66887	'Lonp2'	2182.4	2144.2	1416.5	1320	1668.5	2234.47	1914.333333	1740.97	-0.083682603	0.763514182	0.972790461
668880	'Stard9'	1941	1750	473	885	2051	1266	1388	1400.666667	0.093965124	0.864234985	0.977779046
66889	'Rnf128'	576	581	435	1554	932	1083	530.6666667	1189.666667	1.335536193	0.005319939	0.172598444

668894	'Vinac1'	69	85	9	25	86	25	54.33333333	45.33333333	-0.169710484	0.843490388	0.975182082
66890	'Lman2'	2015	1905	2104	1858	2375	3373	2008	2535.333333	0.347634173	0.266866177	0.883600995
66892	'Eif4e3'	300.7	295.02	214.48	26	222.47	195.21	270.0666667	147.8933333	-0.956759457	0.137920849	0.739360306
668923	'Zfp442'	415.95	376.6	199.62	392.66	570.6	511.4	330.7233333	491.5533333	0.655919192	0.061389858	0.562479029
668929	'Rad21l'	863	977	258	5	307	321	699.3333333	211	-1.81179443	0.076716674	0.609792246
66894	'Wwp2'	1383	1216	840	654	1144	1501	1146.333333	1099.666667	-0.044802509	0.885792827	0.984282742
668940	'Myh7b'	73	58	123	4	35	18	84.66666667	19	-2.284621654	0.00257869	0.114014082
66895	'Pxdc1'	24	20	176	68	37	61	73.33333333	55.33333333	-0.429248984	0.663353833	0.972790461
66897	'Naa16'	516	465	963	449	518	591	648	519.3333333	-0.323646718	0.516059844	0.972790461
66898	'Baiap2l1'	606	579	1980	941	609	1216	1055	922	-0.211043092	0.762922093	0.972790461
66899	'Fip1l1'	2286.8	2237.9	3754	1527.9	2057.7	2333.55	2759.56	1973.043333	-0.495587139	0.24753312	0.868994456
66901	'Proz'	5	11	3	2	14.09	17	6.333333333	11.03	0.75372774	0.460758523	0.972790461
66902	'Mtap'	749	787	1504	648	744	1013	1013.333333	801.6666667	-0.357189221	0.470512048	0.972790461
66904	'Pccb'	692	805	621	177	892	965	706	678	-0.14026542	0.79443108	0.972790461
66905	'Plin3'	1441	1554	1766	802	1905	1948	1587	1551.666667	-0.076299958	0.836961394	0.974723675
66910	'Tmem107'	129	75	114	73	84	130	106	95.66666667	-0.133754	0.763899715	0.972790461
66911	'Nudt16l1'	615	718	737	286	752	1003	690	680.3333333	-0.086220438	0.841299112	0.975182082
66912	'Bzw2'	2895.3	3091.9	1252.2	548.78	5133.7	10079.53	2413.12	5253.996667	1.030316736	0.192812101	0.815697301
66913	'Kdelr2'	1178	1122	3420	1181	1377	2067	1906.666667	1541.666667	-0.371251902	0.551547168	0.972790461
66914	'Vps28'	480	466	563	384	505	733	503	540.6666667	0.102250929	0.770967325	0.972790461
669149	'Vmn2r88'	0	0	1	0	0	0	0.333333333	0	-0.903279821	0.824807108	0.972790461
66915	'Cops9'	486	519	587	341	288	425	530.6666667	351.3333333	-0.535462756	0.178031981	0.797422443
66916	'Ndufb7'	491	554	377	394	519	710	474	541	0.230784345	0.413717273	0.95722888
66917	'Chordc1'	1489	1528	1014	1052	1449	1483	1343.666667	1328	0.046224509	0.856941806	0.976149994
66921	'Prpf38b'	2266	2298	2867	2691	2580	1955	2477	2408.666667	0.054303563	0.903924487	0.987837693
66922	'Rras2'	1056	1124	3205	2369	1423	2559	1795	2117	0.255068462	0.703944195	0.972790461
66923	'Pbrm1'	3012	2934	2457	1035	3020	3093	2801	2382.666667	-0.271091968	0.473412315	0.972790461
66925	'Sdhd'	1226	1317	1827	1283	1394	1832	1456.666667	1503	0.062864993	0.875517319	0.980897882
66926	'Trmt6'	728	700	593	526	740	754	673.6666667	673.3333333	0.045058438	0.854757778	0.975734242
66928	'Dmac1'	388	354	400	133	356	398	380.6666667	295.6666667	-0.41731472	0.301982791	0.904841461
66929	'Asf1b'	576	589	371	111	252	309	512	224	-1.197021821	9.74E-04	0.064990235
66930	'Fank1'	48	45	5	14	24	27	32.66666667	21.66666667	-0.496761255	0.544779119	0.972790461
66931	'1700010I14Rik'	61	57	19	0	53	43	45.66666667	32	-0.598754225	0.609349583	0.972790461
66932	'Rexo1'	1417	1338	885	854	1602	1130	1213.333333	1195.333333	0.030863879	0.922527238	0.991300958
66934	'Dsn1'	695	752	295	159	540	615	580.6666667	438	-0.4195637	0.412919235	0.95722888
66935	'Cir1'	951	875	1143	592	773	842	989.6666667	735.6666667	-0.408619973	0.237020597	0.862272407
66938	'Sh3d21'	268	262	506	149	319	282	345.3333333	250	-0.527040659	0.291744133	0.89958198
66939	'Aagab'	744	776	479	587	846	1129	666.3333333	854	0.397261603	0.17280351	0.790885972
66940	'Shisa5'	811	954	1055	518	1065	1361	940	981.3333333	0.017241099	0.963911809	0.999493374
66942	'Ddx18'	1272	1229	1407	456	1041	1276	1302.666667	924.3333333	-0.543479529	0.150417953	0.759693458

66943	'Slc66a2'	592	602	810	1767	768	977	668	1170.666667	0.978573575	0.104213306	0.676639601
66945	'Sdha'	4545	4683	2945	3816	5542	6511	4057.666667	5289.666667	0.43089823	0.097327898	0.663789939
66948	'Acad8'	639	667	494	281	955	852	600	696	0.178058981	0.666395291	0.972790461
66949	'Trim59'	642	655	687	306	630	585	661.3333333	507	-0.395392886	0.212838581	0.841028891
66950	'Pacc1'	1640	1698	408	525	3468	2538	1248.666667	2177	0.785970535	0.253176361	0.871990757
66952	'2310030G06Rik'	69	89	84	296	109	160	80.66666667	188.3333333	1.422693952	0.025947883	0.397137149
66953	'Cdca7'	792	838	649	176	695	602	759.6666667	491	-0.679765351	0.131229541	0.72897159
66957	'Serpinb11'	16	12	2	0	23	27	10	16.66666667	0.668439884	0.619328159	0.972790461
66958	'Tmx2'	1457	1596	538	1375	2367	2196	1197	1979.333333	0.803600091	0.063153076	0.566852792
66959	'Dusp26'	17	1	16	103	13	17	11.33333333	44.33333333	2.273836706	0.066112216	0.575225699
66960	'Mindy3'	588	579	407	740	546	550	524.6666667	612	0.377193009	0.390184208	0.951958703
66962	'Swsap1'	138	127	83	55	157	135	116	115.6666667	-0.006578314	0.98732378	0.999493374
66964	'Golt1b'	737	773	1009	577	634	885	839.6666667	698.6666667	-0.248864943	0.51676957	0.972790461
66965	'Ctu2'	436.36	463.58	223.61	273.22	374.25	535.75	374.5166667	394.4066667	0.129050817	0.723375994	0.972790461
66966	'Trit1'	414	398	451	416	400	394	421	403.3333333	0.019498319	0.960037288	0.999493374
66967	'Edem3'	1857	1822	1020	6450	2688	3099	1566.333333	4079	1.631045279	0.007001623	0.197779062
66968	'Plin5'	14	17	12	23	21	38	14.33333333	27.33333333	0.985817407	0.091696385	0.651188901
66970	'Ssbp2'	1070.9	1107	965.76	581	907.79	946	1047.896667	811.5966667	-0.34226419	0.155904193	0.76709382
66971	'Cdk5rap1'	343	368	198	63	247	295	303	201.6666667	-0.629185868	0.22109396	0.849076011
66972	'Slc25a23'	1317	1365	807	1646	1476	1746	1163	1622.666667	0.600785311	0.099223382	0.668798985
66973	'Mrps18b'	312	284	799	341	255	432	465	342.6666667	-0.444306044	0.481048989	0.972790461
66975	'Trappc13'	867.85	931.83	588.23	755.95	854.92	920.2	795.97	843.69	0.172129757	0.568569209	0.972790461
66977	'Nuf2'	470	493	110	39	431	308	357.6666667	259.3333333	-0.499349205	0.527930516	0.972790461
66978	'Luc7l'	2470	2313	2602	1518	2434	2180	2461.666667	2044	-0.249972275	0.402466716	0.955996639
66979	'Pole4'	701	797.07	660	193	601	652	719.3566667	482	-0.627586207	0.129484629	0.727256061
66980	'Zdhhc6'	1071	1016	660	962	1084	1154	915.6666667	1066.666667	0.311945135	0.312401916	0.911537035
66983	'Zfp830'	471	461	562	417	478	521	498	472	-0.036753434	0.917408973	0.990547571
66985	'Rassf7'	186	167.07	517	256	108	192	290.0233333	185.3333333	-0.571334978	0.438773836	0.96707305
66988	'Lap3'	1037	1031	1503	578	1024	1482	1190.333333	1028	-0.26616496	0.530607814	0.972790461
66989	'Kctd20'	2626	2752	1817	1283	2505	2527	2398.333333	2105	-0.168320413	0.539241859	0.972790461
66990	'Tmem134'	377	441	472	351	420	589	430	453.3333333	0.092756191	0.789335444	0.972790461
66991	'Khdc3'	5	5	2	4	5	49	4	19.33333333	2.194555948	0.062783099	0.566066012
66993	'Smarcd3'	521.83	576.17	334.16	77.46	530.96	449.2	477.3866667	352.54	-0.505626422	0.397966029	0.954563638
66994	'Cep19'	458	426	300	228	406	464	394.6666667	366	-0.08728037	0.762661165	0.972790461
66995	'Zcchc18'	63	54	20	177	35	23	45.66666667	78.33333333	1.184690998	0.223965516	0.850759465
66997	'Psmd12'	1670	1813	1787	1248	1561	1911	1756.666667	1573.333333	-0.12776591	0.65760337	0.972790461
66998	'Psmd5'	1029	1105	731	2909	1081	1976	955	1988.666667	1.266514803	0.027693401	0.407338487
66999	'Med28'	1048	1123	1066	599	875	1395	1079	956.3333333	-0.182642421	0.583533792	0.972790461
67003	'Uqcrc2'	3070.8	3188.5	3611.9	1808	2844.9	3804.97	3290.433333	2819.296667	-0.238800611	0.469708568	0.972790461
67005	'Polr3k'	692	816	592	667	806	1002	700	825	0.291380964	0.284094752	0.892640391

67006	'Cisd2'	400	454	298	416	534	491	384	480.3333333	0.401318829	0.178756569	0.798524409
67008	'Yae1d1'	604	606	630	464	580	705	613.3333333	583	-0.042511115	0.886448386	0.984398578
67009	'Ttc23'	763	713	392	273	832	730	622.6666667	611.6666667	-0.028747299	0.945292262	0.996592403
67010	'Rbm7'	1121	1193	2310	1000	1137	1411	1541.333333	1182.666667	-0.393086343	0.430356772	0.962808066
67011	'Mettl6'	697	711	744	386	618	618	717.3333333	540.6666667	-0.39487522	0.171434723	0.787518803
67013	'Oma1'	639	708	148	175	591	459	498.3333333	408.3333333	-0.252738566	0.680373223	0.972790461
67014	'Riox2'	549.65	617.74	176.74	78.95	528.36	840.44	448.0433333	482.5833333	0.050478779	0.945542933	0.996592403
67015	'Ccdc91'	579	606	417	166	795	895	534	618.6666667	0.142910481	0.788338258	0.972790461
67016	'Tbc1d2b'	1012	1004	652	3485	1098	1252	889.3333333	1945	1.413031806	0.0383076	0.459930654
67017	'Fam210b'	230	203	129	464	300	394	187.3333333	386	1.202806671	0.010030018	0.247582744
67019	'Actr6'	688	774	453	335	704	662	638.3333333	567	-0.148404126	0.636070822	0.972790461
67020	'Tmem88'	108	96	32	20	115	85	78.66666667	73.33333333	-0.115955911	0.862655258	0.977168837
670211	'Gm12508'	40	33.11	91.36	0	0	0	54.82333333	0	-8.237646653	1.85E-08	1.29E-05
67023	'Use1'	1007	1041	2022	1257	1100	1282	1356.666667	1213	-0.12677801	0.809435772	0.972790461
67025	'Rpl11'	7280	7434	13579	8652	6309	8222	9431	7727.666667	-0.229222625	0.664914404	0.972790461
67026	'Thap4'	877	968	1363	1192	848	1441	1069.333333	1160.333333	0.170873007	0.719830061	0.972790461
67027	'Mkrn2'	1001	988.82	943.74	525.22	877.44	987.94	977.8433333	796.8666667	-0.288310764	0.278615032	0.889355605
67028	'2610002M06Rik'	661	711	1010	232	789	968	794	663	-0.366268284	0.481357718	0.972790461
67030	'Fancl'	305.13	327.22	161.83	30.85	300.22	240.65	264.7266667	190.5733333	-0.546821838	0.428750002	0.962386546
67031	'Upf3a'	819	855	644	235	843	702	772.6666667	593.3333333	-0.418932178	0.321433623	0.917581989
67035	'Dnajb4'	986	960	1453	1406	992	1086	1133	1161.333333	0.133121922	0.792386148	0.972790461
67036	'Mrpl45'	820	836	2687	1134	690	917	1447.666667	913.6666667	-0.624616735	0.375856981	0.940406075
67037	'Pmf1'	399	411	263	50	374	441	357.6666667	288.3333333	-0.399699455	0.537040593	0.972790461
67038	'Ly6m'	0	1	0	13	1	1	0.333333333	5	4.196845014	0.058584301	0.552046072
67039	'Rbm25'	3888.9	3937	3109	3156	3707	2597.99	3644.976667	3153.663333	-0.104493722	0.77534582	0.972790461
67040	'Ddx17'	16942	16520	10020	11475	19131	13430	14494	14678.66667	0.091346577	0.78035792	0.972790461
67041	'Oxct1'	1635	1599	1142	634	1877	1946	1458.666667	1485.666667	-0.002188889	0.995501619	0.999562152
67042	'Ift27'	226	230	117	66	234	491	191	263.6666667	0.401479407	0.518053504	0.972790461
67043	'Syap1'	2259	2398	2054	2839	2598	2875	2237	2770.666667	0.406148945	0.241229377	0.86449675
67044	'Higd2a'	429	497	652	808	483	631	526	640.6666667	0.396576034	0.448076214	0.970198456
67045	'Riok2'	793	772	850	532	891	880	805	767.6666667	-0.062562032	0.8328255	0.974723675
67046	'Tbc1d7'	378	423	107	75	347	351	302.6666667	257.6666667	-0.242599363	0.704565434	0.972790461
67048	'Vma21'	1217	1223	815	735	1022	1383	1085	1046.666667	-0.013015184	0.962695967	0.999493374
67049	'Pus3'	468	478	335	125	347	311	427	261	-0.721449932	0.048695895	0.512912109
670496	'Gm11564'	0	1	0	0	0	0	0.333333333	0	-0.903279821	0.824807108	0.972790461
67050	'Nkap'	951	926	601	390	700	675	826	588.3333333	-0.452778634	0.101725026	0.671759705
67052	'Ndc80'	413	401	114	23	349	372	309.3333333	248	-0.38431194	0.647359286	0.972790461
67053	'Rpp14'	453.48	436	379.65	177.69	578.12	667.44	423.0433333	474.4166667	0.109535633	0.802131367	0.972790461
670533	'Gm11567'	0	0	0	0	0	2	0	0.666666667	1.749449488	0.66566965	0.972790461
67054	'Paics'	4301.3	4217.1	6310.5	6110.7	4121.9	5216.98	4943	5149.853333	0.150692806	0.761903186	0.972790461

670558	'H60c'	3	2	1	1	2	2	2	1.666666667	-0.218097582	0.887129989	0.984534762
67057	'Yaf2'	598	642	831	948	569	826	690.3333333	781	0.282568525	0.572713029	0.972790461
67059	'Ola1'	1300	1404	2169	623	1225	1592	1624.333333	1146.666667	-0.568378442	0.205694231	0.833294914
670593	'Gm9495'	35.31	39.56	3.01	1	23.94	5.21	25.96	10.05	-1.40285947	0.244920339	0.866091613
67062	'Slc25a53'	35	56	9	55	37	49	33.33333333	47	0.690930322	0.340439881	0.925308103
67063	'Pgap4'	98	80	96	27	131	115	91.33333333	91	-0.088463956	0.873917318	0.980171118
67064	'Chmp1b'	641	665	1012	1060	652	766	772.6666667	826	0.206120475	0.702967397	0.972790461
67065	'Polr3d'	637	706	732	383	558	762	691.6666667	567.6666667	-0.287466168	0.372646275	0.938916841
67067	'Romo1'	287	348	287	163	253	312	307.3333333	242.6666667	-0.327271014	0.269576278	0.883851074
67068	'Dynlrb1'	761	795	1016	871	907	1211	857.3333333	996.3333333	0.246896233	0.516578883	0.972790461
67070	'Lsm14a'	2802	2739	6559	2263	2726	3420	4033.333333	2803	-0.559428534	0.306084861	0.907188578
67071	'Rps6ka6'	1377	1369	476	193	952	963	1074	702.6666667	-0.638519401	0.286123649	0.894057448
67072	'Cdc37l1'	1340.9	1298.8	642.9	1299	1504.8	1328.79	1094.21	1377.526667	0.451904166	0.237493779	0.863003008
67073	'Pi4k2b'	355	362	785	608	441	613	500.6666667	554	0.184317114	0.74961306	0.972790461
67074	'Mon2'	2933	2883	2368	3600	2597	2731	2728	2976	0.270748215	0.528545888	0.972790461
67075	'Magt1'	2310	2254	2890	2061	2036	3291	2484.666667	2462.666667	0.008554831	0.982334024	0.999493374
67077	'Catsperz'	10	5	17	3	2	2	10.66666667	2.333333333	-2.156951991	0.052596429	0.532526438
67078	'Pgp'	521	565	846	690	469	879	644	679.3333333	0.1219931	0.807248049	0.972790461
67080	'1700019D03Rik'	13	15	14	15	5	18	14	12.66666667	-0.045169129	0.955089418	0.999493374
67082	'ccdc198'	1	0	0	0	1	0	0.333333333	0.333333333	0.058500858	0.988561293	0.999493374
670832	'Rps12-ps10'	1.01	0	0	0	0	2	0.336666667	0.666666667	0.78767502	0.845746136	0.975182082
67087	'Ctnnbip1'	735	715	401	344	682	686	617	570.6666667	-0.081774291	0.802992396	0.972790461
67088	'Cand2'	1373	1243	390	1269	1441	1338	1002	1349.333333	0.571386423	0.252784231	0.871252558
67089	'Psmc6'	2312	2395	4108	1514	2491	3117	2938.333333	2374	-0.357358745	0.428947698	0.962386546
67091	'Trappc6a'	215	235	459	188	222	342	303	250.6666667	-0.310556207	0.553820051	0.972790461
67092	'Gatm'	1710	2074	629	399	3418	3711	1471	2509.333333	0.714719829	0.31898243	0.916136994
67095	'Trak1'	846	786	901	1348	860	829	844.3333333	1012.333333	0.411035858	0.416653307	0.95722888
67096	'Mmachc'	232	268	162	53	230	237	220.6666667	173.3333333	-0.398069557	0.436906867	0.96622803
67097	'Rps10'	8511	8990	14938	16777	7099	10371	10813	11415.66667	0.219213957	0.725763133	0.972790461
67099	'Mettl21a'	346	338	250	116	315	335	311.3333333	255.3333333	-0.308049858	0.415917595	0.95722888
67101	'2310039H08Rik'	108	141	204	97	123	162	151	127.3333333	-0.258656174	0.582442668	0.972790461
67102	'D16Ertd472e'	280	306	444	188	310	355	343.3333333	284.3333333	-0.301337948	0.468035544	0.972790461
67103	'Ptgr1'	497	541	255	628	818	963	431	803	0.975609171	0.004726674	0.162796256
67105	'Timm21'	596	628	475	212	710	508	566.3333333	476.6666667	-0.272620908	0.50204749	0.972790461
67106	'Zbtb8os'	389.79	492.3	264.1	172.86	388.23	427.02	382.0633333	329.37	-0.207284445	0.569032468	0.972790461
67109	'Zfp787'	465	494	614	513	435	560	524.3333333	502.6666667	0.00462596	0.991185377	0.999493374
67111	'Naaa'	784	764	428	406	791	680	658.6666667	625.6666667	-0.030333532	0.925763424	0.992381554
67112	'Fgf22'	3	1	1	3	3	10	1.666666667	5.333333333	1.68506217	0.209190847	0.836906017
67115	'Rpl14'	9349	9810	13140	8384	7095	8688	10766.33333	8055.666667	-0.352873211	0.411886779	0.95722888
67116	'Cuedc2'	608.58	622.14	745.63	303.6	775.15	913.98	658.7833333	664.2433333	-0.050764034	0.902454959	0.987331639

67117	'Dynlt3'	1294	1337	1746	695	1199	1567	1459	1153.666667	-0.374598616	0.322490963	0.918669192
67118	'Bfar'	856	810	820	582	1008	1074	828.6666667	888	0.103330327	0.707626214	0.972790461
67119	'Ccdc159'	121	141	37	61	169	114	99.66666667	114.6666667	0.254466319	0.653980586	0.972790461
67120	'Ttc14'	2697.8	2724.2	935.03	1804.7	3139	1531.38	2119.023333	2158.37	0.145932212	0.776181571	0.972790461
67121	'Mastl'	259	270	110	69	193	221	213	161	-0.399938017	0.408349131	0.957157474
67122	'Nrarp'	157	148	21	90	193	179	108.6666667	154	0.583639612	0.395277255	0.954014376
67123	'Ubap1'	1229	1329	1305	1885	1225	1369	1287.673333	1493	0.353041178	0.450453837	0.970590732
671232	'Topaz1'	746	662	108	4	309	437	505.3333333	250	-1.088674289	0.333730558	0.921648675
67125	'Tspan31'	1171	1191	1285	1469	1412	1612	1215.666667	1497.666667	0.371918052	0.298096168	0.901330779
67126	'Atp5e'	882	992	1010	666	835	1164	961.3333333	888.3333333	-0.09816435	0.757405716	0.972790461
67127	'Lce1a1'	2	0	0	0	0	1	0.666666667	0.333333333	-0.74959284	0.853034393	0.975734242
67128	'Ube2g1'	2015	1970	3406	1062	1910	2696	2463.666667	1889.333333	-0.451158162	0.333375581	0.921648675
67130	'Ndufa6'	1097	1108	1788	1676	703	1116	1331	1165	-0.054678404	0.928986297	0.993096978
67134	'Nop56'	1858	1905	1347	851	1685	1793	1703.333333	1443	-0.228713494	0.408370925	0.957157474
67136	'Kbtbd4'	716	710	381	205	635	615	602.3333333	485	-0.322801207	0.450887978	0.970649024
67138	'Herc6'	186	206	213	121	169	178	201.6666667	156	-0.345465262	0.293800451	0.90029104
67139	'Mis12'	896.97	924.91	670.97	487	686.93	784.98	830.95	652.97	-0.302602094	0.213418203	0.841981092
67141	'Fbxo5'	268	280	194	55	273.61	205	247.3333333	177.87	-0.527319107	0.306180325	0.907278732
67143	'Ikzf5'	713.31	745.94	1009.5	493	679	588	822.9233333	586.6666667	-0.46474323	0.232285187	0.859679981
67144	'Lrrc40'	1014	1103	917	314	880	1039	1011.333333	744.3333333	-0.487557653	0.21691152	0.843391934
67145	'Tomm34'	860	943	527	320	804	851	776.6666667	658.3333333	-0.239570324	0.518946745	0.972790461
67148	'Ramac'	950.79	985.94	1883.2	969	979	1235.98	1273.316667	1061.326667	-0.255657782	0.609629431	0.972790461
67149	'Nkain1'	325	295	759	173	306	505	459.6666667	328	-0.594628972	0.320148492	0.917050664
67150	'Rnf141'	1451	1406	769.04	979.18	832	1215.02	1208.68	1008.733333	-0.146572054	0.704611805	0.972790461
67151	'Psmd9'	367	397	404	381	565	581	389.3333333	509	0.407798849	0.165619885	0.780109182
67153	'Rnaseh2b'	361	373	173	138	294	274	302.3333333	235.3333333	-0.323890422	0.401799505	0.955996639
671535	'Parp10'	270	308	375	231	301	434	317.6666667	322	0.013310707	0.972270307	0.999493374
67154	'Mtdh'	2781.7	2775.5	2403.4	3822.2	2979.4	3819.73	2653.516667	3540.453333	0.525942554	0.164785657	0.778361468
67155	'Smarca2'	2195	2117	1749	874	2625	2455	2020.333333	1984.666667	-0.059171495	0.875277711	0.980715455
671564	'Rnf212'	218	213	34	1	88	128	155	72.33333333	-1.171914893	0.301644099	0.90469437
67157	'2610301B20Rik'	618	616	500	342	632	599	578	524.3333333	-0.121554347	0.638577902	0.972790461
67158	'Sft2d3'	148	143	84	198	240	220	125	219.3333333	0.907190513	0.011646734	0.268072286
67160	'Eef1g'	13082	12410	22940	16559	11427	16252	16144	14746	-0.070653942	0.893707566	0.985171
67161	'Sclt1'	495	505	213	219	385	222	404.3333333	275.3333333	-0.457357521	0.308953173	0.909541303
67163	'Ccdc47'	1510	1520	1560	2539	1959	2354	1530	2284	0.682569966	0.091794701	0.651188901
67164	'Lipt2'	122	140	83	20	116	183	115	106.3333333	-0.197817503	0.764603851	0.972790461
671641	'Rps12-ps24'	0	0	0	1.12	0	0	0	0.373333333	1.020273531	0.802557913	0.972790461
671650	'Gm20783'	7	6	2	3	4	3	5	3.333333333	-0.480123683	0.667448763	0.972790461
67166	'Arl8b'	1804	1804	2970	1633	1952	2838	2192.666667	2141	-0.050966357	0.907773617	0.988823459
67168	'Lpar6'	248	268	203	60	439	426	239.6666667	308.3333333	0.270681382	0.662660088	0.972790461

67169	'Nradd'	199	187	348	41	253	299	244.6666667	197.6666667	-0.465221754	0.494025292	0.972790461
67171	'Dram2'	604	563	959	292	654	734	708.6666667	560	-0.410648303	0.373615518	0.939449575
67177	'Cdt1'	392	381	397	324	271	335	390	310	-0.243659567	0.530699304	0.972790461
67178	'Zmat5'	158.86	185.91	105.93	145.95	236.95	272.99	150.2333333	218.63	0.581437742	0.070323687	0.589465353
67179	'Ccdc25'	604	606	541	886	703	706	583.6666667	765	0.517003736	0.212145233	0.84083615
67180	'Yipf5'	1184	1243	1164	1080	1159	1439	1197	1226	0.091095959	0.762791685	0.972790461
67181	'Ctdnep1'	882.85	794.57	1824	721	941.67	1113	1167.146667	925.2233333	-0.368240311	0.479643447	0.972790461
67182	'Pdzk1ip1'	1	3	5	13	0	5	3	6	1.237691559	0.449368623	0.97049895
67184	'Ndufa13'	639.97	749.74	707	666.42	686.89	853.12	698.9033333	735.4766667	0.131812645	0.690305688	0.972790461
67186	'Rplp2'	5924.6	6095.9	12451	8334.3	3932.7	5282.01	8157.073333	5849.67	-0.363949299	0.571733194	0.972790461
67187	'Zmynd19'	1597	1499	1084	574	907	1576	1393.333333	1019	-0.452776339	0.209363218	0.837020772
671917	'LOC671917'	1	0	0	0	0	1	0.333333333	0.333333333	0.058500858	0.988561293	0.999493374
67196	'Ube2t'	342	373	304	74	185	211	339.6666667	156.6666667	-1.143111433	0.001930058	0.096058291
67197	'Zcrb1'	730	822	693	348	542	741	748.3333333	543.6666667	-0.456386535	0.119558967	0.710007992
67198	'Spats21'	539	553	902	126	630	649	664.6666667	468.3333333	-0.63861204	0.287620644	0.894952226
67199	'Pfdn1'	730	750	782	329	501	679	754	503	-0.588956624	0.061666779	0.56365808
67200	'Ccdc77'	355.93	365.43	230.1	85.02	331.1	185.05	317.1533333	200.39	-0.671973147	0.15577447	0.766832248
67201	'Glod4'	1578	1749	1166	582	1812	1830.05	1497.666667	1408.016667	-0.119760349	0.765026822	0.972790461
67203	'Nde1'	472	515	296	352	553	498	427.6666667	467.6666667	0.19140022	0.521858739	0.972790461
67204	'Eif2s2'	3843	3716	9771	4222	3182	4269	5776.666667	3891	-0.547954477	0.371204064	0.938513534
67205	'Utp11'	584.1	611.09	1207.3	670.86	545	659.02	800.8133333	624.96	-0.317552581	0.559255018	0.972790461
67207	'Lsm1'	408	383	388	443	407	568	393	472.6666667	0.332174429	0.343233654	0.925308103
67210	'Gatad1'	1173	1258	1686	1012	1196	1601	1372.333333	1269.666667	-0.105140452	0.784468551	0.972790461
67211	'Armc10'	958	974	1349	373	1048	964	1093.666667	795	-0.527741512	0.228815176	0.855540033
67212	'Mrpl55'	287	278	145	120	281	443	236.6666667	281.3333333	0.23543208	0.611107492	0.972790461
672125	'Gm14496'	1	0	0	0	0	0	0.333333333	0	-0.903279821	0.824807108	0.972790461
67213	'Cmtm6'	954	968	673	1596	1281	1591	865	1489.333333	0.906723213	0.016317396	0.31949158
67216	'Mboat2'	1073	1032	986	602	1753	1534	1030.333333	1296.333333	0.294524545	0.430182032	0.962548446
67217	'L3hypdh'	277	279	220	309	234	330	258.6666667	291	0.27877411	0.479431732	0.972790461
67219	'Med18'	120	111	140	92	115	144	123.6666667	117	-0.062809573	0.868655942	0.979356388
67220	'Plekho1'	309	321	672	231	345	397	434	324.3333333	-0.466654627	0.368468768	0.937530612
67222	'Srfbp1'	395	388	806	355	393	460	529.6666667	402.6666667	-0.399899675	0.438584258	0.966976432
67223	'Rrp15'	576	600	665	286	465	506	613.6666667	419	-0.550935403	0.078753032	0.615899891
67224	'Med29'	307.08	318.96	353.17	340.06	266.48	395.71	326.4033333	334.0833333	0.106698321	0.795361762	0.972790461
67225	'Rnpc3'	1313	1316	558	196	1283	730	1062.333333	736.3333333	-0.557982633	0.360227406	0.933706718
67226	'Tmem19'	560	582.96	209	637	787.24	753.71	450.6533333	725.9833333	0.80627462	0.065400807	0.574053623
67228	'Dph7'	209	250	303	87	303	309	254	233	-0.210107268	0.671525325	0.972790461
672284	'Nkx1-1'	0	0	1	0	0	0	0.333333333	0	-0.903279821	0.824807108	0.972790461
67229	'Prpf18'	976	946	753	1167	841	984	891.6666667	997.3333333	0.29907585	0.473113448	0.972790461
67230	'Zfp329'	954	1053	291	542	984	634	766	720	0.015271523	0.976285858	0.999493374

67231	'Tbc1d20'	2314	2101	3067	2994	2190	3492	2494	2892	0.273793549	0.547980373	0.972790461
67235	'Zkscan14'	278	279	225	209	181	286	260.6666667	225.3333333	-0.134833594	0.708586439	0.972790461
67236	'Cinp'	629	665	731	475	410	578	675	487.6666667	-0.405358025	0.291099598	0.899022994
67238	'Fam220a'	419	403	847	1041	569	728	556.3333333	779.3333333	0.583229068	0.346782104	0.928289998
67239	'Rpf2'	297	315	380	75	341	317	330.6666667	244.3333333	-0.533292528	0.315573731	0.914723408
67241	'Smc6'	3189	3200	2904	1459	2402	2448	3097.666667	2103	-0.539755324	0.02513729	0.392372872
67242	'Gemin6'	209.41	219	135	137	201	292	187.8033333	210	0.192308981	0.567985339	0.972790461
67245	'Peli1'	1684	1648	1366	921	1186	1222	1566	1109.666667	-0.438940417	0.082348565	0.622509308
67246	'Resf1'	2517	2713	1109	394	3157	1827	2113	1792.666667	-0.278490306	0.667056733	0.972790461
67247	'Mtarc2'	1224	1214	1246	1185	1521	1791	1228	1499	0.318906344	0.258666835	0.877564912
67248	'Rpl39'	3434	3653	7548	3896	2242	2982	4878.333333	3040	-0.600089467	0.324443421	0.919683661
67249	'Tbc1d19'	794	812	384	119	945	719	663.3333333	594.3333333	-0.215379199	0.736351649	0.972790461
672511	'Rnf213'	15261	15032	2486	2091	16546	16197	10926.33333	11611.33333	0.062845091	0.936481325	0.994413066
67252	'Cap2'	193	212	35	41	307	211	146.6666667	186.3333333	0.33665022	0.666393743	0.972790461
67254	'Bmerb1'	573.76	541.32	33	15	252.1	217.99	382.6933333	161.6966667	-1.271675154	0.217159532	0.84361198
67255	'Zfp422'	1279.9	1364	1110	387	1400.9	1126.86	1251.286667	971.5866667	-0.408204929	0.333495619	0.921648675
67260	'Cers4'	1265	1278	229	955	2135	1329	924	1473	0.769174448	0.217780959	0.844165262
67263	'Zswim6'	453	477	760	769	306	350	563.3333333	475	-0.068997011	0.917710126	0.990547571
67264	'Ndufb8'	1041	1171	893	708	989	1142	1035	946.3333333	-0.090061588	0.710427487	0.972790461
67266	'Dipk1a'	622.88	556.75	257.1	410.93	650.02	591.48	478.91	550.81	0.280622337	0.462574539	0.972790461
67267	'Uqcc2'	468	568	415	958	392	801	483.6666667	717	0.736662461	0.176168854	0.796060628
67268	'Myl12a'	2959.6	3078.3	6944.2	5534	2859	5050	4327.35	4481	0.113543566	0.857660083	0.976399524
672682	'Muc21'	0	0	0	1	0	0	0	0.333333333	1.020273531	0.802557913	0.972790461
67269	'Agtpbp1'	1000	996	485	358	686	857	827	633.6666667	-0.357899766	0.340537135	0.925308103
67270	'Mrpl42'	630	571	535	245	460	623	578.6666667	442.6666667	-0.402859582	0.220265404	0.848971277
67273	'Ndufa10'	1628	1574	1306	760	1358	1596	1502.666667	1238	-0.273588956	0.301873662	0.904841461
67276	'Eri1'	709	700	755	908	874	1167	721.3333333	983	0.503638185	0.147235498	0.754255261
67278	'Pagr1a'	663	623	553	551	674	635	613	620	0.085114226	0.771506488	0.972790461
67279	'Med31'	296.86	334.99	349.78	149.77	241.84	307.8	327.21	233.1366667	-0.499547625	0.139194525	0.741642687
67281	'Rpl37'	4750.9	5231.7	7958.1	6469	3102.5	4211.64	5980.22	4594.36	-0.246477999	0.676054565	0.972790461
67282	'Washc3'	439	465	389	145	351	468	431	321.3333333	-0.461450976	0.232957835	0.859679981
67283	'Slc25a19'	234	241	317	526	319	379	264	408	0.750927026	0.151375103	0.75995796
67285	'Cwc27'	644	586	904	254	547	499	711.3333333	433.3333333	-0.757895568	0.068254816	0.583566634
67286	'Ift22'	447	433	254	165	449	562	378	392	0.033465877	0.937241397	0.994594529
67287	'Parp6'	477	461	954	432	417	410	630.6666667	419.6666667	-0.555577615	0.300530102	0.904081584
67288	'Srek1ip1'	405	418	444	70	436	415	422.3333333	307	-0.567388989	0.334535915	0.921767653
67290	'3110040N11Rik'	196	253	217	56	188	236	222	160	-0.541035063	0.262973787	0.88013169
67291	'Ccdc137'	619	682	933	218	631	597	744.6666667	482	-0.70121406	0.123856741	0.71638709
67292	'Pigc'	506	492	481	306	538	449	493	431	-0.172530848	0.552410289	0.972790461
67295	'Rab3c'	1	0	8	0	0	0	3	0	-4.151035762	0.212424285	0.840892212

67296	'Socs4'	820	827	921	867	874	850	856	863.6666667	0.08836567	0.812927871	0.972790461
67298	'Gprasp1'	5431.9	5928.8	1596.1	2981.6	8056.6	5863.41	4318.92	5633.87	0.434720433	0.409552427	0.957157474
67299	'Dock7'	3592.2	3588	2121	1339	3331	2830.14	3100.383333	2500.046667	-0.297778085	0.389890883	0.951553974
67300	'Cltc'	9641	9602	13630	11849	8429	12051	10957.66667	10776.33333	0.050575571	0.913633139	0.98991242
67302	'Zc3h13'	2165	2015	1318	1263	2023	1864	1832.666667	1716.666667	-0.039118159	0.885548211	0.984282742
67305	'Gpx7'	260	273	240	58	371	222	257.6666667	217	-0.32288777	0.573959649	0.972790461
67306	'Zc2hc1a'	701	625	828	302	691	713	718	568.6666667	-0.381093758	0.321885083	0.917918531
67307	'Pbld2'	677	648	121	1212	712	225	482	716.3333333	0.899948654	0.288986535	0.896282545
67308	'Mrpl46'	282	314	218	174	303	334	271.3333333	270.3333333	0.017443481	0.950554658	0.998065566
673094	'Cd99'	2	3	2	1	2	2	2.333333333	1.666666667	-0.465946654	0.749427566	0.972790461
67310	'Prl8a9'	0	1	0	0	0	0	0.333333333	0	-0.903279821	0.824807108	0.972790461
67311	'Nanp'	323	366	231	121	335	345	306.6666667	267	-0.215761155	0.577776764	0.972790461
67313	'Inava'	22	23	56	114	24	18	33.66666667	52	0.883288801	0.377901623	0.941757196
67317	'1700022I11Rik'	4	6	3	2	2	0	4.333333333	1.333333333	-1.532168166	0.311438931	0.911537035
67326	'1700037H04Rik'	368	365	855	900	334	500	529.3333333	578	0.2599063	0.716905731	0.972790461
67331	'Atp8b3'	19	23	19	12	16	14	20.33333333	14	-0.486227568	0.40729135	0.957157474
67332	'Snrpd3'	637	666	696	387	489	744	666.3333333	540	-0.295766755	0.37188397	0.938527219
67333	'Stk35'	1198	1135	449	382	922	1249	927.3333333	851	-0.115847614	0.808928653	0.972790461
67337	'Cstf1'	867.97	870	745	385	795	889	827.6566667	689.6666667	-0.272473782	0.361402586	0.933706718
67338	'Rffl'	442.35	369.51	310.16	298.27	369.05	556.85	374.0066667	408.0566667	0.160380226	0.607169543	0.972790461
67341	'Ascl4'	2	2	0	1	5	1	1.333333333	2.333333333	0.874155324	0.63518692	0.972790461
67343	'Tex43'	0	0	0	0	2	1	0	1	2.320886	0.562538074	0.972790461
67344	'Tctex1d1'	1	2	0	0	2	0	1	0.666666667	-0.537241284	0.8664971	0.978294615
67345	'Herc4'	1861	1859	888	1695	1716	1832	1536	1747.666667	0.311157577	0.423271719	0.96017682
67358	'1700093K21Rik'	1	0	4	3	3	1	1.666666667	2.333333333	0.500377219	0.782342917	0.972790461
67366	'Tmed11'	0	1	0	0	0	0	0.333333333	0	-0.903279821	0.824807108	0.972790461
67367	'Paxbp1'	1735	1824	1396	1090	2112	1135	1651.666667	1445.666667	-0.140383536	0.704424974	0.972790461
67369	'Qpctl'	194	204	66	181	261	272	154.6666667	238	0.714254898	0.122360035	0.71347226
67370	'Zfp606'	1051	1118	581	361	1152	887	916.6666667	800	-0.196502971	0.642045538	0.972790461
67371	'Gtf3c6'	591	782	571	113	832	544	648	496.3333333	-0.466449224	0.437514562	0.96622803
67373	'2210010C04Rik'	1	0	0	0	1	0	0.333333333	0.333333333	0.058500858	0.988561293	0.999493374
67374	'Jam2'	217	225	491	722	569	737	311	676	1.151829097	0.044366345	0.490688158
67375	'Qprt'	43	40	42	84	38	41	41.66666667	54.33333333	0.574990033	0.380742717	0.944042914
67378	'Bbs2'	554	588	295	289	703	604	479	532	0.174312943	0.643722728	0.972790461
67379	'Dedd2'	328	261	439	634	392	407	342.6666667	477.6666667	0.602091486	0.279803264	0.889355605
67381	'Med4'	1029	1010	947	1023	680	918	995.3333333	873.6666667	-0.064131429	0.881626431	0.982528614
67382	'Brd3'	3076	3187	1864	2389	2872	2761	2709	2674	0.076904711	0.806653888	0.972790461
67383	'Carnmt1'	1349	1331	1195	690	1273	1605	1291.666667	1189.333333	-0.128729123	0.663250247	0.972790461
67384	'Bag4'	1004	1085	721	550	728	1139	936.6666667	805.6666667	-0.186849607	0.546433531	0.972790461
67387	'Unc50'	737	792	508	625	762	910	679	765.6666667	0.240756676	0.386971726	0.94901869

67388	'Rab5if'	815	885	966	2221	1005	1354	888.6666667	1526.666667	0.95220896	0.091245398	0.649965349
67389	'C1qtnf12'	390	376	269	82	499	518	345	366.3333333	0.007475186	0.989775546	0.999493374
67390	'Mrm3'	231	248	135	84	182	236	204.6666667	167.3333333	-0.286652451	0.469213131	0.972790461
67391	'Fundc2'	885	961	1457	596	912	1039	1101	849	-0.399077381	0.335964823	0.922539021
67392	'4833420G17Rik'	1231	1205	1151	658	1014	613	1195.666667	761.6666667	-0.588865292	0.107170288	0.682129732
67393	'Cxxc5'	756	755	1841	1389	1102	1436	1117.333333	1309	0.250318364	0.672905651	0.972790461
67397	'Erp29'	1149	1164	2213	757	1389	1987	1508.666667	1377.666667	-0.211796844	0.674220542	0.972790461
67398	'Srpr'	2307	2341	3191	2259	2507	3603	2613	2789.666667	0.104219943	0.788042986	0.972790461
67399	'Pdlim7'	658	635	1632	1574	635	787	975	998.6666667	0.166647083	0.819104927	0.972790461
67405	'Nts'	62	43	17	1	14	6	40.66666667	7	-2.547186454	0.003974614	0.145317997
67407	'Cylc1'	0	0	0	0	1	0	0	0.333333333	1.020273531	0.802557913	0.972790461
67410	'4930449I24Rik'	0	0	0	0	0	1	0	0.333333333	1.020273531	0.802557913	0.972790461
67412	'Soga3'	11	13	5	1	7	1	9.666666667	3	-1.660810329	0.153922923	0.765411885
67414	'Mfn1'	2410.5	2338.5	1255.1	1039.7	1683.2	1988.69	2001.35	1570.536667	-0.304536398	0.341904148	0.925308103
67416	'Armcx2'	2655	2777	3862	3951	3376	3361	3098	3562.666667	0.28178082	0.538762224	0.972790461
67417	'Ears2'	533	555	328	175	460	501.92	472	378.9733333	-0.326676849	0.396788835	0.954358954
67418	'Ppil4'	1680	1718	1121	878	1331	1226	1506.333333	1145	-0.335930031	0.201708459	0.82952222
67419	'Armh4'	179	142	200	48	91	60	173.6666667	66.33333333	-1.381054231	0.001474339	0.08292939
67420	'Far1'	2165	2413	3946	2261	2394	2422	2841.333333	2359	-0.238463975	0.610520997	0.972790461
67422	'Dhdds'	765	759	1174	704	965	1287	899.3333333	985.3333333	0.110501019	0.789400658	0.972790461
67425	'Eps8l1'	34	32	5	35	214	101	23.66666667	116.6666667	2.315573289	0.003911534	0.143563888
67426	'Coq8a'	1487.7	1445.2	268.53	1776.2	1728	2652	1067.14	2052.063333	1.080061134	0.078326153	0.615492265
67427	'Rps20'	7977	8337	10412	8562	6217	8084	8908.666667	7621	-0.135601346	0.761437763	0.972790461
67429	'Nudcd1'	559	529	1086	1787	650	951	724.6666667	1129.333333	0.791631833	0.252824551	0.871252558
67430	'4921536K21Rik'	31	31	14	16	13	8	25.33333333	12.33333333	-0.865589003	0.225727124	0.852846598
67432	'Hoga1'	46	46	47	29	28	38	46.33333333	31.66666667	-0.496676157	0.28503185	0.893711786
67433	'Ccdc127'	1093.9	1014	1202.4	1190.6	1149	1362.36	1103.463333	1233.996667	0.224314311	0.538729971	0.972790461
67434	'Ankrd33b'	31	22	163	19	10	15	72	14.66666667	-2.298691102	0.018040246	0.335598003
67437	'Ssr3'	6089	5974	14534	8679	6928	9595	8865.666667	8400.666667	-0.062020809	0.915560831	0.990380433
67439	'Xab2'	1324	1302	1093	725	1224	1404	1239.666667	1117.666667	-0.13617692	0.585228752	0.972790461
67440	'Mtpap'	788	814	1199	397	835	816	933.6666667	682.6666667	-0.498235461	0.230015633	0.856721174
67441	'Isoc2b'	33	24	6	22	78	91	21	63.66666667	1.603082964	0.026306804	0.397367216
67442	'Retsat'	255	260	49	240	441	553	188	411.3333333	1.198255939	0.047198285	0.50504201
67443	'Map1lc3b'	1972	2045	4371	5360	2312	2873	2796	3515	0.463822423	0.49620376	0.972790461
67444	'Ilkap'	1073.7	1150.8	1026.3	625.06	968.72	1018.38	1083.573333	870.72	-0.291819806	0.242184642	0.86449675
67445	'C1qtnf4'	132	127	134	24	145	116	131	95	-0.553652376	0.34328033	0.925308103
67446	'Dusp28'	102	113	93	97	116	145	102.6666667	119.3333333	0.264038079	0.42445759	0.960285372
67448	'Plxdc2'	956.72	931.74	1020.5	3008.8	1341.8	1865.96	969.6533333	2072.193333	1.270477533	0.023140728	0.377626024
67451	'Pkp2'	313	295	2063	424	462	621	890.3333333	502.3333333	-0.926124084	0.279648811	0.889355605
67452	'Pnpla8'	1519	1441	2012	1799	1639	1789	1657.333333	1742.333333	0.137442537	0.745892319	0.972790461

67453	'Slc25a46'	714	706	768	720	840	821	729.3333333	793.6666667	0.177903333	0.591154443	0.972790461
67454	'Ikbip'	493	547	531	958	592	900	523.6666667	816.6666667	0.757740705	0.094951639	0.661495261
67455	'Klhl13'	1753	1965	702	238	1751	1550	1473.333333	1179.666667	-0.367630541	0.574855167	0.972790461
67456	'Ergic2'	1204.5	1181.7	809.87	910.34	1318.9	1207.39	1065.333333	1145.553333	0.169241087	0.523831069	0.972790461
67457	'Frmd8'	552	594	880	2181	673	995	675.3333333	1283	1.124095921	0.104925823	0.677368335
67458	'Ergic1'	1238	1145	1180	2509	1522	2164	1187.666667	2065	0.926903608	0.042917752	0.483384062
67459	'Nvl'	2281.6	2273	1240.2	1358.7	2218.9	2118	1931.61	1898.543333	0.034193553	0.91077405	0.989608384
67460	'Decr1'	831	940	763	216	991	1073	844.6666667	760	-0.231200065	0.652975406	0.972790461
67463	'Poc5'	608.5	553.47	279.6	265	459	452.02	480.5233333	392.0066667	-0.236053636	0.507034274	0.972790461
67464	'Entpd4'	53.32	106.77	53.37	52.72	0	104.93	71.15333333	52.55	-0.350623293	0.781617766	0.972790461
67465	'Sf3a1'	2916	2850	2219	780	2157	2046	2661.66	1660.996667	-0.70292341	0.046974341	0.504562804
67466	'Pdcl'	937	1047	1160	724	1094	1206	1048	1008	-0.051278903	0.871501656	0.980171118
67467	'Gpalpp1'	1077	1080.1	1112	854	1080	1092	1089.706667	1008.666667	-0.066075746	0.823668365	0.972790461
67468	'Mmd'	953	950	1090	14061	1261	2538	997.6666667	5953.333333	2.929979105	0.002014382	0.099125678
67469	'Abhd5'	361	354	619	357	355	469	444.6666667	393.6666667	-0.161281973	0.733404814	0.972790461
67470	'Abcg8'	3	1	0	1	3	1	1.333333333	1.666666667	0.415542484	0.830284287	0.974104589
67471	'Gpatch1'	467	530	455	339	538	511	484	462.6666667	-0.035640763	0.893877662	0.985171
67472	'Mtfr1'	960	998	1234	1111	845	1140	1064	1032	0.035246113	0.935180347	0.994413066
67473	'Slc47a1'	9	5	8	1	9	0	7.333333333	3.333333333	-1.180925778	0.415322417	0.95722888
67474	'Snap29'	707	736	756	500	658	707	733	621.6666667	-0.204023458	0.486400669	0.972790461
67475	'Ero1b'	658	644	898	881	844	1173	733.3333333	966	0.431470912	0.290764841	0.898719095
67477	'Abhd15'	28	24	3	23	17	22	18.33333333	20.66666667	0.35991296	0.674614462	0.972790461
67480	'Cwc25'	797	765	895	576	690	758	819	674.6666667	-0.242047638	0.462232521	0.972790461
67483	'1700028P14Rik'	0	0	0	0	1	2	0	1	2.297812331	0.566584047	0.972790461
67484	'Eepd1'	347.8	338.29	273.86	343.11	399.44	468.7	319.9833333	403.75	0.399306464	0.158009028	0.768890221
674842	'Gm9631'	4	4	0	0	1	4	2.666666667	1.666666667	-0.711072337	0.732322324	0.972790461
67486	'Polr3g'	477	436	499	144	392	746	470.6666667	427.3333333	-0.231256811	0.658885773	0.972790461
67487	'Dhx40'	2275	2384	2622	1274	1882	2437	2427	1864.333333	-0.383462329	0.221531355	0.849403976
67488	'Calcoco1'	2100	1987	3438	3721	2413	2682	2508.333333	2938.666667	0.328294924	0.548691881	0.972790461
67489	'Ap4b1'	817.44	821.18	398	724.78	645.24	628.28	678.8733333	666.1	0.12294675	0.775245186	0.972790461
674895	'Nek10'	17	15	4	17	33	31	12	27	1.232921401	0.071622071	0.592807492
67490	'Ufl1'	1283	1330	2230	825	1221	1108	1614.333333	1051.333333	-0.627155981	0.156350061	0.767860225
67492	'Zfand4'	143	132	39	17	104	70	104.6666667	63.66666667	-0.726501613	0.292621751	0.900043079
67493	'Mettl16'	1383	1366	1258	473	1052	1179	1335.666667	901.3333333	-0.590736159	0.066886556	0.578108476
67495	'Tmem167b'	1184	1115	1554	989	989	1410	1284.333333	1129.333333	-0.158981223	0.688600022	0.972790461
67498	'Kcnv1'	2	4	2	0	0	0	2.666666667	0	-3.778209068	0.135692355	0.736689633
67500	'Ccar1'	1966	1997	2207	1889	2257	1828	2056.666667	1991.333333	0.018984236	0.958131776	0.999493374
67501	'Ccdc50'	2490	2583	3423	3253	2747	2734	2832	2911.333333	0.126129571	0.77927643	0.972790461
67504	'Rnf151'	7	8	0	2	7	0	5	3	-0.594742465	0.752623995	0.972790461
67507	'1700019N19Rik'	1	1	0	0	0	0	0.666666667	0	-1.703500596	0.673820112	0.972790461

67509	'Saysd1'	139	168	82	344	160	160	129.6666667	221.3333333	1.01626841	0.100737169	0.671759705
67510	'Tvp23b'	817	880	1159	691	813	1215	952	906.3333333	-0.072067626	0.853649592	0.975734242
67511	'Tmed9'	1577	1565	2268	2350	1865	3067	1803.333333	2427.333333	0.470132224	0.295175977	0.90029104
67512	'Agpat2'	302	296	404	881	299	605	334	595	0.992747602	0.113942546	0.698948431
67513	'Faap20'	228	234	371	135	306	279	277.6666667	240	-0.264613492	0.561055664	0.972790461
67515	'Ttc33'	584	537	395	246	430	556	505.3333333	410.6666667	-0.287593874	0.342110644	0.925308103
67516	'Kctd4'	3	2	0	2	2	1	1.666666667	1.666666667	0.192540122	0.915047864	0.990203767
67525	'Trim62'	787	870	320	211	1133	520	659	621.3333333	-0.087545737	0.884067746	0.983608145
67526	'Atg12'	1344	1384	2010	1028	1061	1526	1579.333333	1205	-0.376003833	0.366632594	0.936632475
67528	'Nudt7'	120	147	64	96	158	157	110.3333333	137	0.37331032	0.337348583	0.92380462
67529	'Fgfr1op2'	1737	1731	1482	1182	1435	1325	1650	1314	-0.255924918	0.378002736	0.94187472
67530	'Uqcrb'	825	922	1441	617	748	1053	1062.666667	806	-0.414109847	0.349168613	0.928823768
67532	'Mfap1a'	959.03	1014.7	1377	918.6	1033.8	1266.57	1116.926667	1072.983333	-0.037019025	0.924223009	0.992057805
67533	'Ppfibp1'	2687	2763	3033	4104	2292	3107	2827.666667	3167.666667	0.302289737	0.541535105	0.972790461
67534	'Ttll4'	2432	2258	704	1972	2151	1706	1798	1943	0.272655156	0.598664393	0.972790461
675363	'Gm9640'	0	3	2	0	0	0	1.666666667	0	-3.145571549	0.413903919	0.95722888
67537	'Glipr1l2'	50	42	9	0	21	49	33.66666667	23.33333333	-0.614627672	0.619809023	0.972790461
67538	'Zswim3'	403	433	168	74	280	373	334.6666667	242.3333333	-0.494225922	0.381710217	0.94429001
67542	'Cog6'	1470	1524	1128	529	1136	1469	1374	1044.666667	-0.411317127	0.222203331	0.849517424
67543	'Pabpc6'	158	145	16	0	71	54	106.3333333	41.66666667	-1.40604929	0.269698605	0.883851074
67544	'Fam120b'	777	724	615	294	870	750	705.3333333	638	-0.171874823	0.645206781	0.972790461
67547	'Slc39a8'	51	65	98	185	58	116	71.33333333	119.6666667	0.900953007	0.205348572	0.833294914
67549	'Gpr89'	503.59	520.67	336.33	258.34	546.99	568.54	453.53	457.9566667	0.024781837	0.93682356	0.994413066
67553	'Gstcd'	469	477	463	226	503	570	469.6666667	433	-0.145305676	0.66960288	0.972790461
67554	'Slc25a30'	337	333	480	1217	314	381	383.3333333	637.3333333	0.980835251	0.197243606	0.822728266
67556	'Pigm'	745	683	420	1265	756	794	616	938.3333333	0.803673006	0.114873075	0.70178885
67557	'Larp6'	39	42	134	22	43	36	71.66666667	33.66666667	-1.176836718	0.104752188	0.67684333
67561	'Wdr48'	2127	2135.9	1396	1269.8	1744.5	1835.27	1886.286667	1616.506667	-0.159434304	0.537204475	0.972790461
67563	'Ciao3'	327.95	314.11	299.45	276.33	394.51	428.46	313.8366667	366.4333333	0.254869514	0.346819746	0.928289998
67564	'Tmem35a'	601	750	169	455	1057	1015	506.6666667	842.3333333	0.787902232	0.14914442	0.757738857
67568	'Mrfap1'	5869	5888	7793	5293	5167	6770	6516.666667	5743.333333	-0.142223774	0.715437664	0.972790461
67569	'Mgat4c'	0	0	1	2	0	2	0.333333333	1.333333333	2.061542391	0.4933139	0.972790461
67573	'Loxl4'	14	15	13	290	5	89	14	128	3.540811949	0.002361184	0.109223443
67574	'Alg13'	521	451	186	135	291	768	386	398	0.014962643	0.980607651	0.999493374
67575	'A26c3'	2	1	0	0	11	13	1	8	2.926460315	0.115893069	0.702825844
67578	'Patl2'	17	30	7	1	46	56	18	34.33333333	0.849516358	0.438359608	0.966976432
67579	'Cpeb4'	924	985	1070	3071	1069	1048	993	1729.333333	1.042280128	0.126764128	0.721784845
67580	'Lrrc18'	12	17	6	5	22	15	11.66666667	14	0.264932362	0.73805871	0.972790461
67581	'Tbc1d23'	2158.6	2401.4	2172.9	3965	2336.3	2086.14	2244.306667	2795.806667	0.49792922	0.347039536	0.928289998
675812	'Zfp605'	319	374	283	105	273	252	325.3333333	210	-0.646737154	0.069346555	0.587268017

67582	'Slc25a26'	227	245	146	34	236	209	206	159.6666667	-0.436187943	0.475114049	0.972790461
67586	'Ubxn11'	157	148	27	36	165	105	110.6666667	102	-0.092399815	0.89797897	0.985794067
67588	'Rnf41'	1334	1312	1325	779	931	1431	1323.666667	1047	-0.319838234	0.310569648	0.910971113
67590	'Tctn3'	160	164	58	81	241	192	127.3333333	171.3333333	0.453730135	0.372811026	0.938916841
67592	'4930524B15Rik'	263	263	112	1	154	88	212.6666667	81	-1.485005156	0.164447817	0.777557251
67593	'4930519G04Rik'	28	27	5	0	18	15	20	11	-0.917672616	0.446904281	0.970198456
675947	'Gm9195'	1	1	1	0	0	0	1	0	-2.390783043	0.494152846	0.972790461
67596	'Tespa1'	1	1	0	0	1	0	0.666666667	0.333333333	-0.74517548	0.838722495	0.974723675
67602	'Necap1'	920	932	1745	1292	868	1303	1199	1154.333333	8.06E-05	0.999882164	0.999935533
67603	'Dusp6'	387	409	991	1259	466	673	595.6666667	799.3333333	0.556367699	0.447196592	0.970198456
67604	'Get4'	1335	1357	1293	1251	1335	1865	1328.333333	1483.666667	0.205826583	0.499892884	0.972790461
67605	'Akt1s1'	1194	1226	2431	957	1281	1394	1617	1210.666667	-0.44044068	0.370897053	0.9384981
67606	'Fibin'	22	29	19	20	28	43	23.33333333	30.33333333	0.401660815	0.43627246	0.965982602
67607	'Zfp788'	535	601	459	141	630	342	531.6666667	371	-0.55763577	0.256847732	0.8773831
67608	'Narf'	1473	1384	3057	1614	1147	1259	1971.333333	1340	-0.49075563	0.402748568	0.955996639
67609	'4930453N24Rik'	765	826	593	399	637	775	728	603.6666667	-0.246555011	0.344319663	0.925308103
67610	'Rspry1'	1240.3	1263.4	830	537	892.6	943.36	1111.236667	790.9866667	-0.453513591	0.078960326	0.616000706
67615	'Ube2r2'	1862	1705	3680	1043	1784	2035	2415.666667	1620.666667	-0.637019647	0.205465183	0.833294914
67618	'Aasdhppt'	741	792	792	453	769	834	775	685.3333333	-0.175992117	0.540583163	0.972790461
67619	'Nob1'	565	528	1313	825	470	734	802	676.3333333	-0.189064647	0.765795669	0.972790461
67620	'Lrp2bp'	43	60	12	8	29	10	38.33333333	15.66666667	-1.224313498	0.124762717	0.718566731
67621	'Bend5'	243	228	712	19	205	304	394.3333333	176	-1.394675592	0.11331521	0.698948431
67622	'Mxra7'	238	226	343	541	387	432	269	453.3333333	0.847131427	0.094199259	0.658197981
67623	'Tm7sf3'	867	912	614	455	1364	2048	797.6666667	1289	0.641008882	0.169969175	0.785454025
67628	'Anp32b'	4105	3886	9970	1279	4128	5986	5987	3797.666667	-0.814023388	0.208587146	0.836616817
67629	'Spc24'	158.9	180.89	65.27	8	221.72	481.57	135.02	237.0966667	0.70914289	0.475100238	0.972790461
67630	'Samd8'	899	886	1299	2164	797	1302	1028	1421	0.631511716	0.314869144	0.914723408
67636	'Etfrf1'	431.94	493	477.51	372.74	553.75	592.64	467.4833333	506.3766667	0.134271192	0.642933071	0.972790461
67645	'Armc12'	1	1	2	0	1	1	1.333333333	0.666666667	-1.094532947	0.611807345	0.972790461
67647	'4930523C07Rik'	337	353	199	149	336	299	296.3333333	261.3333333	-0.158286976	0.650313004	0.972790461
67652	'Spaca1'	92	118	17	5	67	94	75.66666667	55.33333333	-0.500643051	0.597028733	0.972790461
676527	'Gm12248'	6	1	1	1	4	1	2.666666667	2	-0.363756563	0.821057738	0.972790461
67653	'4930544G11Rik'	0	0	0	0	1	0	0	0.333333333	1.020273531	0.802557913	0.972790461
67654	'4930558C23Rik'	0	0	0	0	1	2	0	1	2.297812331	0.566584047	0.972790461
67655	'Ctdp1'	611	600	992	1231	629	765	734.3333333	875	0.384600144	0.517073596	0.972790461
67656	'4930548H24Rik'	3	1	2	0	3	1	2	1.333333333	-0.667121555	0.717720688	0.972790461
67657	'Rab13'	410	390	369	100	333	422	389.6666667	285	-0.519082138	0.263687677	0.88013169
67661	'Ift172'	1703	1626	921	482	1573	1470	1416.666667	1175	-0.285494247	0.505583118	0.972790461
67664	'Rnf125'	270	260.88	158	5	343	190	229.6266667	179.3333333	-0.463704058	0.63856174	0.972790461
67665	'Dctn4'	1767	1787	2413	1685	1857	2145	1989	1895.666667	-0.037460562	0.922051116	0.991293589

67666	'Hapln3'	20	15	4	22	17	5	13	14.66666667	0.43023158	0.657294027	0.972790461
67667	'Alkbh8'	943	970.99	867.03	544	977.09	741.88	927.0066667	754.3233333	-0.267827863	0.357705901	0.932505998
67669	'Hikeshi'	594	545	1208	249	407	719	782.3333333	458.3333333	-0.864749779	0.120539787	0.711929925
67671	'Rpl38'	1681	1819	4481	2639	1156	1691	2660.333333	1828.666667	-0.439211896	0.529411613	0.972790461
67673	'Elob'	984	1028	1207	1116	829	1288	1073	1077.666667	0.078796652	0.852541338	0.975734242
67674	'Trmt112'	670.57	694.44	1123.9	273.54	565.45	866.26	829.6366667	568.4166667	-0.631135159	0.198964289	0.825995916
67675	'Cuta'	270	269	294	300	211	340	277.6666667	283.6666667	0.114340379	0.791322405	0.972790461
67676	'Rpp21'	131	119	175	47	111	197	141.6666667	118.3333333	-0.350596842	0.511667213	0.972790461
67678	'Lsm3'	645	647	613	105	396	679	635	393.3333333	-0.788466442	0.157100489	0.768337387
67680	'Sdhb'	1139	1163	684	885	1028	1384	995.3333333	1099	0.21371197	0.489168199	0.972790461
67681	'Mrpl18'	612.16	631.1	786.08	763.35	579.07	610.05	676.4466667	650.8233333	0.046916515	0.919138657	0.990815958
67683	'Pbdc1'	879	955	1083	391	689	824	972.3333333	634.6666667	-0.636715446	0.060754821	0.559627259
67684	'Luc7l3'	4188	4225	2017	3724	4517	2750	3476.666667	3663.666667	0.217199295	0.626780994	0.972790461
67685	'Dnaaf4'	89	96	43	25	82	76	76	61	-0.317158582	0.536397671	0.972790461
67689	'Aldh3b1'	409.26	516.02	85	134	545	565	336.76	414.6666667	0.310033603	0.652201839	0.972790461
67693	'Hypk'	689	823	1140	195	666	795	884	552	-0.78583201	0.134032391	0.733301819
67694	'Ift74'	1061	1070	638	202	828	715	923	581.6666667	-0.700493191	0.138343263	0.739741822
67695	'Ost4'	594	672	1174	924	492	647	813.3333333	687.6666667	-0.134900538	0.822422556	0.972790461
67698	'Fam174a'	410	469	356	178	425	803	411.6666667	468.6666667	0.129370016	0.786629727	0.972790461
67701	'Wfdc2'	8	9	12	162	10	29	9.666666667	67	3.139102893	0.004836197	0.164614637
67702	'Rnf149'	1488	1714	806	1366	1704	2042	1336	1704	0.43285227	0.203188452	0.831114208
67703	'Kirrel3'	168.78	200.36	75.47	229.3	341.72	300.47	148.2033333	290.4966667	1.065485548	0.010585053	0.254415968
67704	'1810037I17Rik'	388	400	777	493	371	502	521.6666667	455.3333333	-0.152541894	0.780204408	0.972790461
67705	'1810058I24Rik'	655	693	953	860	630	701	767	730.3333333	0.023768288	0.960943794	0.999493374
67706	'Tmem179b'	181	197	129	94	187	209	169	163.3333333	-0.037140745	0.910394582	0.989561592
67707	'Mrpl24'	780.74	692.12	1212.9	696.41	734.82	1065.58	895.2666667	832.27	-0.104405467	0.817254649	0.972790461
67708	'Pcnx4'	389.97	444.55	215.88	542.4	426.02	493.94	350.1333333	487.4533333	0.63042008	0.149180336	0.757738857
67709	'Reg4'	1	0	0	0	1	0	0.333333333	0.333333333	0.058500858	0.988561293	0.999493374
67710	'Polr2g'	685	838	764	405	846	983	762.3333333	744.6666667	-0.059120541	0.860955088	0.976863639
67711	'Nsmce1'	649	632	650	253	622	680	643.6666667	518.3333333	-0.351613519	0.332859305	0.921648675
67712	'Slc25a37'	766.27	817.15	695.37	2331.2	1267.9	694.36	759.5966667	1431.153333	1.14810071	0.074371793	0.603245712
67713	'Dnajc19'	223	265	187	64	270	399	225	244.3333333	0.039605437	0.943467719	0.996436989
677156	'Cyp4f37'	2.97	0	0	0	0	0	0.99	0	-1.711373851	0.67234292	0.972790461
67719	'2310057J18Rik'	1	0	0	0	0	0	0.333333333	0	-0.903279821	0.824807108	0.972790461
67724	'Pop1'	282	289	105	51	429	338	225.3333333	272.6666667	0.228155572	0.740733556	0.972790461
67725	'Nudt13'	248	293	250	447	369	271	263.6666667	362.3333333	0.602050131	0.206723011	0.834086989
67726	'Fam114a2'	1477	1409	1918	891	1091	1184	1601.333333	1055.333333	-0.577845512	0.115710426	0.702825844
67727	'Stx17'	2089	2209	573	386	1965	1681	1623.666667	1344	-0.282382668	0.656496976	0.972790461
67728	'Dph2'	235	260	161	119	216	219	218.6666667	184.6666667	-0.212186087	0.487977895	0.972790461
67729	'Mansc1'	17	26	11	40	111	110	18	87	2.271899321	2.12E-05	0.004291

677296	'Fcrl6'	1	0	0	0	0	0	0.333333333	0	-0.903279821	0.824807108	0.972790461
67731	'Fbxo32'	206	172	579	770	158	242	319	390	0.486691496	0.587450611	0.972790461
67732	'Iah1'	383	380	432	244	463	584	398.3333333	430.3333333	0.081566042	0.817781336	0.972790461
67733	'Itgb3bp'	244	255	102	9	133	129	200.3333333	90.33333333	-1.224234513	0.12270441	0.714169238
67736	'Ccdc130'	262	297	271	139	202	202	276.6666667	181	-0.580072559	0.051147392	0.524212515
67737	'Ttc39d'	62	48	8	0	28	21	39.33333333	16.33333333	-1.321386386	0.273180862	0.884738928
67738	'Ppid'	1654	1660	2250	547	1488	1653	1854.666667	1229.333333	-0.667833589	0.12890865	0.72577634
67739	'Slc48a1'	615	702	626	1899	892	1580	647.6666667	1457	1.319969955	0.01036444	0.251980489
67742	'Samsn1'	18	24	14	4	38	15	18.66666667	19	-0.023739463	0.977124243	0.999493374
67747	'Ribc2'	57	55	5	0	31	28	39	19.66666667	-1.038105697	0.418237525	0.958015883
67749	'Mgarp'	164	129	2151	594	132	603	814.6666667	443	-0.908490803	0.425336772	0.960285372
67752	'Ppp1r32'	5	3	5	2	2	4	4.333333333	2.666666667	-0.693287766	0.556787192	0.972790461
677525	'LOC677525'	0	2.84	0	18.38	6	23.69	0.946666667	16.02333333	4.708580547	0.002038892	0.099648902
67753	'Eqtn'	3	6	2	13	5	1	3.666666667	6.333333333	1.113119192	0.403036806	0.955996639
67755	'Ddx47'	1210	1303	1713	1060	1179	1344	1408.666667	1194.333333	-0.20661961	0.591782976	0.972790461
67758	'Aadac'	3	2	1	0	4	0	2	1.333333333	-0.61945045	0.778430231	0.972790461
67759	'Plgrkt'	172	213	241	168	237	230	208.6666667	211.6666667	0.040160123	0.913982171	0.989964366
67760	'Slc38a2'	2828	2717	5031	18086	3878	3666	3525.333333	8543.333333	1.547387374	0.070941717	0.591553852
67763	'Prpsap1'	952	1014	870	1843	1304	1147	945.3333333	1431.333333	0.756341153	0.109659335	0.689773582
67767	'Jagn1'	332	377	234	336	411	524	314.3333333	423.6666667	0.491958644	0.095962107	0.662457477
67768	'N6amt1'	261	272	181	104	369	243	238	238.6666667	-0.011017169	0.979992758	0.999493374
67769	'Gpatch2'	832	848	435	407	808	663	705	626	-0.122717641	0.723016562	0.972790461
67770	'Caap1'	431	383	244	213	308	404	352.6666667	308.3333333	-0.1509109	0.633510492	0.972790461
67771	'Arpc5'	2123	2224	3028	1579	2492	2829	2458.333333	2300	-0.113807518	0.758769005	0.972790461
67772	'Chd8'	4498	4543	1971	2463	4953	3934	3670.666667	3783.333333	0.102614907	0.791095322	0.972790461
67773	'Kat8'	555	570	710	307	533	661	611.6666667	500.3333333	-0.317384721	0.381947727	0.94429001
67774	'Borcs5'	122	142	99	264	130	183	121	192.3333333	0.849771551	0.115865937	0.702825844
67775	'Rtp4'	54	45	147	9	33	208	82	83.33333333	-0.223043887	0.826970622	0.972978864
67776	'Vwa5a'	1392	1332	3901	2150	1641	1868	2208.333333	1886.333333	-0.199433094	0.758243912	0.972790461
67778	'Zfp639'	976	915	943	393	858	891	944.6666667	714	-0.426204906	0.186030314	0.807018167
67781	'Ilf2'	1901.7	2040.1	1954	5974.9	1707.1	3888.95	1965.293333	3856.966667	1.171727594	0.0639404	0.5684378
67784	'Plxnd1'	2170	1975	1165	1146	2866	2312	1770	2108	0.270247316	0.453719871	0.970649024
67785	'Zmym4'	3127	3163	1397	1479	2724	2982	2562.333333	2395	-0.051825287	0.889602784	0.984980969
67788	'Sfr1'	2618	2515	5514	1800	2483	3616	3549	2633	-0.488714909	0.349292857	0.928823768
677884	'Pakap'	1029	929	4154	6874	1051	1637	2037.333333	3187.333333	0.855029983	0.392223222	0.952648938
67789	'Dalrd3'	458.28	479.93	367.12	399.89	541.72	740.03	435.11	560.5466667	0.396158512	0.149046932	0.757738857
67790	'Rab39b'	20	18	71	8	12	20	36.33333333	13.33333333	-1.557937086	0.066374545	0.575690169
67792	'Rgs8'	6	5	25	3	5	2	12	3.333333333	-1.899890047	0.101390188	0.671759705
67797	'Snrnp48'	580	532	1578	540	568	462	896.6666667	523.3333333	-0.769261941	0.225666741	0.852787933
67800	'Dgat2'	294	306	292	138	164	290	297.3333333	197.3333333	-0.582144161	0.107590924	0.683205958

67801 'Pllp'	3	2	4	8	2	4	3	4.666666667	0.805816108	0.519604239	0.972790461
67803 'Limd2'	852.31	951.12	701.99	372.98	902.99	812.31	835.14	696.0933333	-0.268840904	0.401262666	0.955860942
67804 'Snx2'	1453	1367	2059	1209	1491	2101	1626.333333	1600.333333	-0.031295833	0.937843467	0.994698142
67808 'Tprgl'	1077	1149	1156	1395	1500	1667	1127.346667	1520.666667	0.492180003	0.12773709	0.723917691
67809 'Rmdn3'	868	800	360	609	626	926	676	720.3333333	0.1886247	0.644442237	0.972790461
67811 'Poldip2'	1643	1651	1211	679	1450	1909	1501.666667	1346	-0.173198839	0.610766947	0.972790461
67812 'Ubxn4'	2427	2339	4074	1734	2566	3166	2946.666667	2488.666667	-0.277331996	0.52851183	0.972790461
67815 'Sec14l2'	34	21	23	53	70	45	26	56	1.189994073	0.022316291	0.370441983
67819 'Derl1'	1217	1289	3408	2458	1684	2773	1971.333333	2305	0.237581037	0.708399744	0.972790461
67821 'Atp1b4'	0	1	0	0	0	2	0.333333333	0.666666667	0.78767502	0.845746136	0.975182082
67824 'Nmral1'	178	242	115	25	177	193	178.3333333	131.6666667	-0.507168763	0.436207636	0.965982602
67826 'Snap47'	1101	1177	1383	1760	1152	1673	1220.333333	1528.333333	0.423490695	0.356642259	0.932066196
67830 'Rer1'	1119	1243	1686.9	1585	1432	1817	1349.616667	1611.333333	0.304928988	0.476384057	0.972790461
67832 'Brix1'	1648	1554	1842	529	1316	1226	1681.333333	1023.666667	-0.756537527	0.040479516	0.472434971
67834 'Idh3a'	1254	1330	993	1464	1283	1748	1192.333333	1498.333333	0.42357105	0.211863344	0.840560909
67836 'Wdr83'	294	273	203	75	268	237	256.6666667	193.3333333	-0.442705285	0.31369595	0.913268497
67838 'Dnajb11'	1423	1435	2006	1835	1489	3095	1621.333333	2139.666667	0.415501758	0.363362515	0.935022643
67839 'Gpsm1'	1033	1039	582	366	1159	600	884.6666667	708.3333333	-0.302473214	0.498855127	0.972790461
67840 'Mrpl57'	265	304	264	214	251	285	277.6666667	250	-0.098016219	0.748746394	0.972790461
67841 'Atg3'	681	690	775	298	609	823	715.3333333	576.6666667	-0.354633471	0.346903016	0.928289998
67842 'Nop9'	893.51	831.51	697.93	485	774.71	1096.74	807.65	785.4833333	-0.034827502	0.906617194	0.988823459
67843 'Slc35a4'	1127	1192	1293	857	1114	1401	1204	1124	-0.082001253	0.79135002	0.972790461
67844 'Rab32'	258	271	1000	61	175	219	509.6666667	151.6666667	-1.916668646	0.009401435	0.2383201
67845 'Rnf115'	924	905	1160	743	848	1133	996.3333333	908	-0.114789349	0.750910655	0.972790461
67846 'Tmem39a'	829	791	1515	740	768	1046	1045	851.3333333	-0.296929387	0.54233284	0.972790461
67847 'Sncaip'	467	498	97	121	448	294	354	287.6666667	-0.261494393	0.686327593	0.972790461
67848 'Ddx55'	401	423	225	112	343	304	349.6666667	253	-0.470869377	0.265099306	0.88128484
67849 'Cdca5'	179	191	74	13	156	280	148	149.6666667	-0.074642001	0.929189069	0.993096978
67851 'Mtres1'	454	425	502	627	381	476	460.3333333	494.6666667	0.233510021	0.634638186	0.972790461
67855 'Asprv1'	12	5	8	4	11	3	8.333333333	6	-0.447560485	0.649591473	0.972790461
67856 'Echdc3'	134	125	146	17	157	197	135	123.6666667	-0.263484122	0.714397192	0.972790461
67857 'Ppp6c'	1173	1267	1950	1054	1375	1514	1463.333333	1314.333333	-0.156081948	0.710107847	0.972790461
67859 'Cysrt1'	3	1	0	2	1	1	1.333333333	1.333333333	0.232475138	0.906999904	0.988823459
67860 'S100a16'	260	288	172	9	458	331	240	266	0.033006037	0.972325536	0.999493374
67861 'Akr1b10'	299	312	390	966	431	524	333.6666667	640.3333333	1.114478606	0.062084071	0.565027461
67862 '2310033P09Rik'	306	354	230	139	302	311	296.6666667	250.6666667	-0.236674946	0.466547206	0.972790461
67863 'Slc25a11'	1083.9	1122.1	1308.5	812	1085.3	1294.65	1171.496667	1063.966667	-0.125574264	0.698068877	0.972790461
67864 'Yipf4'	1072	926	1575	1471	1020	1391	1191	1294	0.193879038	0.700472459	0.972790461
67865 'Rgs10'	90	91	71	178	126	124	84	142.6666667	0.916026571	0.059426151	0.553094905
67866 'Wfdc1'	206	275	20	57	434	466	167	319	0.921389207	0.309031412	0.909630779

67867	'Lrrc28'	298	299	128	112	239	326	241.6666667	225.6666667	-0.083431169	0.852699229	0.975734242
67868	'Cela3b'	35	42	43	307	34	76	40	139	2.120346577	0.022601324	0.372676826
67869	'Paip2'	2708	2724	5629	2272	2669	3001	3687	2647.333333	-0.488007518	0.335557379	0.922413343
67870	'Enoph1'	475.86	481.85	425.1	635	519.19	591.5	460.9366667	581.8966667	0.44817981	0.24658843	0.868334735
67871	'Mrrf'	449	481	627.37	243	442.37	642	519.1233333	442.4566667	-0.281137647	0.498151166	0.972790461
67872	'Nsmce4a'	781	776	849	1063	1063	1106	802	1077.333333	0.498763833	0.166318362	0.780893009
67873	'Mri1'	191.31	171.85	211.67	222.7	375.49	364.28	191.61	320.8233333	0.747984874	0.026692107	0.401162323
67874	'Rprm'	37	59	95	3	25	21	63.66666667	16.33333333	-2.104942836	0.007221798	0.202210332
67876	'Coq10b'	175	209	592	2790	255	676	325.3333333	1240.333333	2.180642607	0.031138772	0.42861408
67877	'Naa20'	756	777	920	400	685	987	817.6666667	690.6666667	-0.277803802	0.454553162	0.970649024
67878	'Tmem33'	1256	1305	839	1131	1543	1697	1133.333333	1457	0.422675663	0.102989986	0.673330676
67880	'Dcxr'	63	86	80	50	58	116	76.33333333	74.66666667	-0.035496543	0.938992659	0.995379339
67881	'Mdp1'	442	500	394	205	560	479	445.3333333	414.6666667	-0.121926657	0.733175213	0.972790461
67883	'Uxs1'	525.41	521.34	210.92	571.4	752.99	751.03	419.2233333	691.8066667	0.824178248	0.041446422	0.476408328
67884	'Cfap410'	485	457	309	179	384	395	417	319.3333333	-0.374302692	0.241473818	0.86449675
67885	'Mtln'	80	91	155	57	124	171	108.6666667	117.3333333	0.024921871	0.963092583	0.999493374
67886	'Camsap2'	1862	1889	1993	2003	2042	2143	1914.666667	2062.666667	0.178920982	0.605052165	0.972790461
67887	'Saraf'	1019	862	1709	5085	1586	2844	1196.666667	3171.666667	1.568635784	0.023947206	0.382913609
67888	'Tmem100'	26	18	1	140	15	30	15	61.66666667	2.484339959	0.038403786	0.459930654
67889	'Rbm18'	849	771	1183.6	919	854.63	829	934.5433333	867.5433333	-0.040862552	0.928265753	0.993096978
67890	'Ufm1'	744	821	1829	663	812	1009	1131.333333	828	-0.484756494	0.374382831	0.940259759
67891	'Rpl4'	25390	24349	51220	26210	21378	27072	33653	24886.66667	-0.401255805	0.458142493	0.972790461
67892	'Coa6'	183	212	229	154	181	310	208	215	0.048543377	0.901331781	0.98672375
67893	'Tmem86a'	1603	1854	650	1699	3347	2668	1369	2571.333333	0.978475438	0.022348883	0.370441983
67894	'Dennd10'	345	329	485	160	373	441	386.3333333	324.6666667	-0.317457275	0.472841391	0.972790461
67895	'Ppa1'	825	805	633	303	627	876	754.3333333	602	-0.345064531	0.325027663	0.919968131
67896	'Ccdc80'	1699	1505	383	56	1773	1354	1195.666667	1061	-0.238022588	0.800284654	0.972790461
67897	'Rnmt'	936	935	1112	1031	934	1153	994.3333333	1039.333333	0.129388846	0.736841012	0.972790461
67898	'Pef1'	667	700	1118	807	638	907	828.3333333	784	-0.032662891	0.946275981	0.996592403
67899	'Cmc1'	122.21	132.93	321.35	263.05	185.76	205.43	192.1633333	218.08	0.237033698	0.713401556	0.972790461
67900	'Mtfp1'	93	104	45	81	94	107	80.66666667	94	0.316377681	0.456495852	0.97151713
67902	'Sumf2'	452	479	188	244.02	590	592.81	373	475.61	0.376193315	0.390948275	0.952178913
67903	'Gipc1'	1010	1103	1108	497	1134	1507	1073.666667	1046	-0.08610375	0.822225304	0.972790461
67905	'Ppm1m'	323	364	149	654	610	557	278.6666667	607	1.27837041	0.005019685	0.16781708
67909	'Galntl5'	0	0	0	0	0	1	0	0.333333333	1.020273531	0.802557913	0.972790461
67911	'Zfp169'	357	391	274	423	321	259	340.6666667	334.3333333	0.138310721	0.77119886	0.972790461
67912	'1600012H06Rik'	905.69	967.09	665	1071.5	1096	1124	845.9266667	1097.153333	0.477416281	0.144121701	0.750780289
67914	'Coq9'	808	835	771	445	806	923	804.6666667	724.6666667	-0.15437091	0.584069781	0.972790461
67916	'Plpp3'	855	952	505	704	1549	1229	770.6666667	1160.666667	0.625278556	0.068127471	0.583441208
67917	'Zcchc3'	1087	1150	1009	146	882	1090	1082	706	-0.720838736	0.228543823	0.855498163

67920	'Mak16'	597	600.77	941.53	662.8	694	823.52	713.1	726.7733333	0.052195966	0.904716208	0.988191689
67921	'Ube2f'	584	579	564	735	557	741	575.6666667	677.6666667	0.336604905	0.398164027	0.954563638
67922	'Fam32a'	1449	1440	2763.9	5638.9	1728	2103	1884.313333	3156.626667	0.943327237	0.205031257	0.833294914
67923	'Eloc'	877	1083	1308	913	872	1207	1089.333333	997.3333333	-0.089439297	0.825645741	0.972817749
67926	'Cby2'	0	0	0	0	0	1	0	0.333333333	1.020273531	0.802557913	0.972790461
67928	'Abca14'	2	5	0	1	1	6	2.333333333	2.666666667	0.225640343	0.898440797	0.98586843
67931	'Serpini2'	2	0	0	0	0	0	0.666666667	0	-1.711373851	0.67234292	0.972790461
67933	'Hcfc2'	1373	1365	884.03	1444.1	998.03	1185	1207.343333	1209.033333	0.158131741	0.716517209	0.972790461
67935	'Ces5a'	3	2	0	0	5	3	1.666666667	2.666666667	0.649800454	0.74684645	0.972790461
67936	'Wdr55'	256	232	603	140	287	416	363.6666667	281	-0.485640593	0.4190173	0.958308933
67937	'Tmem59l'	6	19	2	15	5	7	9	9	0.286636222	0.797758121	0.972790461
67938	'Myl12b'	994.45	955.74	748.76	1904	955	1412	899.65	1423.666667	0.837456774	0.096617482	0.663197379
67939	'Prorsd1'	139	161	262	94	188	271	187.3333333	184.3333333	-0.102763581	0.840602771	0.975078699
67941	'Rps27l'	1112	1116	1318	757	816	1229	1182	934	-0.318254061	0.371992572	0.938527219
67942	'Atp5g2'	1802	1846	2626	893	1242	1778	2091.333333	1304.333333	-0.705042983	0.076778479	0.609792246
67943	'Mesd'	1414	1281	752.07	1314	1804	1777.02	1149.036667	1631.683333	0.584084033	0.059021145	0.552865869
67945	'Rpl41'	4982	5726.8	11776	6800	4556	6462	7494.896667	5939.333333	-0.286737948	0.624659151	0.972790461
67946	'Spata6'	611	580	581	481	495	635	590.6666667	537	-0.079773625	0.803358544	0.972790461
67948	'Fbxo28'	1046	1071	1232	1473	929	1018	1116.333333	1140	0.165938226	0.737058091	0.972790461
67949	'Nifk'	871	951	1354	375	888	1033	1058.666667	765.3333333	-0.538949877	0.224816227	0.850926956
67951	'Tubb6'	435	441	1115	685	604	489	663.6666667	592.6666667	-0.119001999	0.849693987	0.975734242
67952	'Tomm20'	3044	2970	5444	6211	2952	4897	3819.333333	4686.666667	0.39866321	0.505694115	0.972790461
67955	'Sugt1'	1056	1171	1891	1391	1259	1434	1372.666667	1361.333333	0.03241274	0.946252018	0.996592403
67956	'Kmt5a'	1497	1586	1704	1981	1498	1738	1595.666667	1739	0.229682841	0.589040046	0.972790461
67958	'U2surp'	3454	3432	3055	2333	3254	3096	3313.666667	2894.333333	-0.147158897	0.559225622	0.972790461
67959	'Puf60'	2481	2494	2726	1516	2366	2723	2567	2201.666667	-0.219655949	0.454935119	0.970934082
67963	'Npc2'	2318	2169	5420	4315	2616	3331	3302.333333	3420.666667	0.118423167	0.851518273	0.975734242
67966	'Zcchc10'	149	152	217	113	142	193	172.6666667	149.3333333	-0.213875419	0.611119958	0.972790461
67967	'Pold3'	1241	1286	699	413	1089	1206	1075.333333	902.6666667	-0.258460186	0.51323769	0.972790461
67968	'Ooep'	13	24	11	36	42	182	16	86.66666667	2.40004098	0.001170455	0.07155208
67971	'Tppp3'	18	41	12	25	57	74	23.66666667	52	1.150978916	0.065033023	0.572836659
67972	'Atp2b1'	3376.8	3077.8	1667	2973.9	2878.6	3185.6	2707.18	3012.69	0.278291304	0.462649678	0.972790461
67973	'Mphosph10'	814	797	702	533	725	793	771	683.6666667	-0.13223832	0.594926103	0.972790461
67974	'Ccny'	3240	3246	3201	2027	2927	3085	3229	2679.666667	-0.241870191	0.350964316	0.92886351
67976	'Trabd'	721	720	1392	553	572	880	944.3333333	668.3333333	-0.513791667	0.306102184	0.907188578
67978	'Tctn2'	269	301	61	191	405	389	210.3333333	328.3333333	0.711476598	0.219238779	0.846500339
67979	'Atad1'	1465	1487	1645	1105	1854	1912	1532.333333	1623.666667	0.083715977	0.779671403	0.972790461
67980	'Gnpda2'	691	704	641	185	729	849	678.6666667	587.6666667	-0.284508247	0.559887695	0.972790461
67981	'Hormad1'	2484	2635	1084	6	1073	922	2067.666667	667	-1.735644075	0.12030187	0.711408757
67983	'Pdzd9'	11.18	12.46	9.06	2.02	4.06	3.03	10.9	3.036666667	-1.797344306	0.051463611	0.526318542

67988	'Tmx3'	1380	1476	829	1622	2343	2479	1228.333333	2148	0.86989822	0.002344131	0.108699072
67991	'Nacc2'	1031	1100	478	239	970	1178	869.6666667	795.6666667	-0.163645937	0.76263838	0.972790461
67993	'Nudt12'	243	252	116	160	308	261	203.6666667	243	0.309865005	0.417497802	0.957720764
67994	'Mrps11'	557	702	235	407	555	599	498	520.3333333	0.157337985	0.713223562	0.972790461
67996	'Srsf6'	5100	5549	3251	1236	5793	3516	4633.333333	3515	-0.428301176	0.389414886	0.951231321
67997	'Ddx59'	401	402	193	158	318	254	332	243.3333333	-0.395279271	0.297331282	0.900711016
67998	'Retreg3'	1310	1327	633	2321	1433	1644	1090	1799.333333	0.918972075	0.069578915	0.587499452
68001	'Cfap298'	519	508	282	103	419	605	436.3333333	375.6666667	-0.271808605	0.623144608	0.972790461
68002	'Sdhaf4'	245	230	97	115	268	373	190.6666667	252	0.409315384	0.393589177	0.953178011
68010	'Bambi'	442	506	141	303	880	714	363	632.3333333	0.835693665	0.113971861	0.698948431
68011	'Snrpg'	1018	1060	1616	609	591	703	1231.333333	634.3333333	-0.920317861	0.037812417	0.459930654
68014	'Zwilch'	1163.9	1082	269	31	772.94	597.95	838.28	467.2966667	-0.905682639	0.317995184	0.916136994
68015	'Trap1'	2354	2481	1359	898	1964	2344	2064.666667	1735.333333	-0.244009448	0.495273799	0.972790461
68016	'Cavin4'	23	22	15	34	25	20	20	26.33333333	0.556048024	0.383223523	0.945476981
68017	'Mrm2'	167	185	101	132	205	210	151	182.3333333	0.327793734	0.316659827	0.915640551
68018	'Cert1'	2044	2087	1365	1163	1854	1787	1832	1601.333333	-0.144472443	0.568524323	0.972790461
68020	'Coa8'	294	282	280	319	298	327	285.3333333	314.6666667	0.227036713	0.535348617	0.972790461
68021	'Bphl'	236	321	236	83	364	370	264.3333333	272.3333333	-0.025963841	0.959579489	0.999493374
68023	'Pdf'	356	337	148	120	406	385	280.3333333	303.6666667	0.110817077	0.821564752	0.972790461
68024	'H2bc4'	31.16	26.87	146.74	269.63	70.09	84.93	68.25666667	141.55	1.208182245	0.221494707	0.849403976
68026	'Pclaf'	300	339	125	26	464	293	254.6666667	261	-0.03309179	0.967506675	0.999493374
68027	'Tmem178'	185	218	52	143	379	372	151.6666667	298	1.011791581	0.071760374	0.592919704
68028	'Rpl22l1'	1973	2079	3803	2344	1653	2149	2618.333333	2048.666667	-0.291673958	0.589764828	0.972790461
68031	'Rnf146'	1678.5	1595.4	2737.7	605.83	1367.3	1465.81	2003.846667	1146.31	-0.877016502	0.056383941	0.545987502
68032	'Emc4'	364	469	482	259	488	661	438.3333333	469.3333333	0.062569641	0.869913812	0.979794316
68033	'Cox19'	266	269	315	296	252	312	283.3333333	286.6666667	0.091439263	0.823338604	0.972790461
68034	'Fam122a'	280	274	228	196	216	272	260.6666667	228	-0.132460421	0.662170874	0.972790461
68035	'Rbm42'	830	840	1141	826	1047	1254	937	1042.333333	0.162208502	0.662491364	0.972790461
68036	'Zfp706'	2182.1	2091.2	2837.8	1358	2085.8	2495.92	2370.336667	1979.893333	-0.275027894	0.442133528	0.969177389
68038	'Chid1'	673.15	672.64	348.55	415.55	687.03	806.07	564.78	636.2166667	0.216878178	0.515382458	0.972790461
68039	'Nmb'	48	41	50	40	30	29	46.33333333	33	-0.380998564	0.490590595	0.972790461
68040	'Zfp593'	200	226	395	221	222	352	273.6666667	265	-0.056002247	0.911670156	0.989773583
68041	'Mid1ip1'	1025	1197	545	565	1271	1277	922.3333333	1037.666667	0.19408226	0.607747888	0.972790461
68043	'Eef1akmt1'	211	234	110	21	195	211	185	142.3333333	-0.454100216	0.520458382	0.972790461
68044	'Chac2'	86	92	164	123	86	88	114	99	-0.118082155	0.842149972	0.975182082
68045	'Rtraf'	3052.1	3059.1	3012.7	2155.4	2395.3	3018.18	3041.286667	2522.97	-0.221496814	0.453506671	0.970649024
68046	'2700062C07Rik'	234	223	196	63	209	237	217.6666667	169.6666667	-0.412989789	0.357581248	0.932505998
68047	'Mpnd'	732	769	641	756	919	1027	714	900.6666667	0.391575089	0.139852159	0.742885537
68048	'Aen'	572	579	675	673	1763	1885	608.6666667	1440.333333	1.186449844	0.00286134	0.119683851
68050	'Akirin1'	1387	1422	1334	2093	1611	2006	1381	1903.333333	0.570424224	0.147016541	0.753611197

68051	'Nutf2'	1318	1531	1605	703	1227	1459	1484.666667	1129.666667	-0.410388141	0.206882824	0.83455469
68052	'Rps13'	4552	4564.8	9961.1	4936.1	3890.8	4888.87	6359.3	4571.92	-0.440790393	0.433639194	0.964992995
68053	'Ubxn2b'	638	766	245	1202	721	680	549.6666667	867.6666667	0.893152351	0.132003043	0.729547052
68054	'Serpina12'	3	0	1	2	0	0	1.333333333	0.666666667	-0.579339665	0.847016467	0.975209338
68055	'Dmac2l'	181	247	101	94	249	226	176.3333333	189.6666667	0.122288463	0.782600901	0.972790461
68058	'Chd1l'	743	737	245	69	687	881	575	545.6666667	-0.142931559	0.851781266	0.975734242
68059	'Tm9sf2'	3150	3303	1447	2864	4625	5364	2633.333333	4284.333333	0.758923979	0.036658093	0.457518227
68066	'Slc25a39'	1351	1445	2128	1596	1493	2135	1641.333333	1741.333333	0.111996412	0.797633361	0.972790461
68067	'Mrnip'	281	294.84	99.15	88.03	211	395	224.9966667	231.3433333	0.034430843	0.952119776	0.998640168
68070	'Pdzd2'	1794	1653	251	602	1598	1803	1232.666667	1334.333333	0.159411447	0.814171487	0.972790461
68073	'Atpsckmt'	557.69	534.46	156.92	297.55	574.68	364.35	416.3566667	412.1933333	0.083202679	0.873428443	0.980171118
68075	'Lurap1'	86	78	91	84	107	130	85	107	0.356582073	0.333975101	0.921648675
68077	'Nop53'	2452	2480	5519	3919	2455	2937	3483.666667	3103.666667	-0.091782582	0.880850161	0.982442994
68079	'Pdcd2l'	324	320	521	252	304	460	388.3333333	338.6666667	-0.216730902	0.628996951	0.972790461
68080	'Gpn3'	445	452	674	329	396	704	523.6666667	476.3333333	-0.164795096	0.711086628	0.972790461
68082	'Dusp19'	128	139	95	46	120	158	120.6666667	108	-0.187098888	0.665829128	0.972790461
68083	'Pak1ip1'	1104	1151	1575	856	985	1750	1276.666667	1197	-0.109581951	0.793878872	0.972790461
68087	'Dcakd'	1306	1242	1099	599	1252	1691	1215.666667	1180.666667	-0.068482604	0.841951079	0.975182082
68089	'Arpc4'	1241.5	1338.7	1504.8	1031.8	1159.9	1645.92	1361.673333	1279.2	-0.067809652	0.843355126	0.975182082
68090	'Yif1a'	287	316	565	221	348	446	389.3333333	338.3333333	-0.253358055	0.601626811	0.972790461
68092	'Ncbp2'	1911	2008	809	888	1820	1742	1576	1483.333333	-0.040621307	0.91922397	0.990815958
68094	'Smarcc2'	3546	3726	1126	916	4854	3257	2799.333333	3009	0.096673075	0.874104718	0.980171118
68095	'Ociad1'	2345.4	2279	2796.2	1783.6	2332.4	2675.48	2473.533333	2263.833333	-0.110465139	0.736021407	0.972790461
68097	'Dynll2'	5212	5629	2852	2878	4057	6290	4564.333333	4408.333333	-0.009831186	0.977871024	0.999493374
68098	'Rchy1'	807	821	1912	492	798	989	1180	759.6666667	-0.706910061	0.196228621	0.822481393
68099	'Cibar1'	711.96	735.92	722.95	124.36	775.83	776.97	723.61	559.0533333	-0.476919154	0.415219187	0.95722888
68106	'Nt5c3b'	709.02	838.09	674	428	1094	697	740.37	739.6766667	2.95E-04	0.999352668	0.999900097
68107	'Cntd1'	259.39	220.78	45.52	18.8	138.07	176.7	175.23	111.19	-0.69403636	0.392650117	0.952648938
68112	'Entr1'	908.41	890.34	551.21	781.58	989.26	1066.38	783.32	945.74	0.347814952	0.228991876	0.855540033
68114	'Pwwp3a'	1208	1177	483	82	1070	1163	956	771.6666667	-0.388593435	0.615576734	0.972790461
68115	'Maip1'	334	334	478	184	328	400	382	304	-0.368488003	0.364726621	0.935515019
68117	'Apool'	493	570	673	124	420	460	578.6666667	334.6666667	-0.875351573	0.068669553	0.584398186
68118	'Atg101'	381	402	502	815	415	567	428.3333333	599	0.624643457	0.25260718	0.871252558
68119	'Cmtm3'	1324	1389	1584	622	2231	1786	1432.333333	1546.333333	0.042457209	0.924710701	0.992301171
68121	'Cep70'	697.84	763.75	224.67	293.04	670.18	492.83	562.0866667	485.35	-0.145368091	0.770547457	0.972790461
68126	'Fahd2a'	207	236	60	39	245	341	167.6666667	208.3333333	0.270122878	0.712376384	0.972790461
68133	'Gcsh'	668	638	646	1270	713	907	650.6666667	963.3333333	0.718635441	0.147001379	0.753611197
68134	'Upf3b'	773	833	389	326	1235	592	665	717.6666667	0.122850956	0.808277313	0.972790461
68135	'Eif3h'	4079	3989	6957	5936	3537	4518	5008.333333	4663.666667	-0.004757725	0.993190905	0.999493374
68137	'Kdelr1'	1279	1201	2897	1288	1503	1826	1792.333333	1539	-0.245806523	0.65217792	0.972790461

68140	'Tigd2'	320	358	506	955	305	484	394.6666667	581.3333333	0.746640697	0.270371935	0.883851074
68142	'Ino80'	888	827.96	869	724	1017	1052	861.6533333	931	0.142500881	0.608960409	0.972790461
68145	'Etaa1'	713	781	470	266	736	767	654.6666667	589.6666667	-0.164859743	0.668710506	0.972790461
68146	'Arl13b'	557	521	1571	667	489	466	883	540.6666667	-0.658901503	0.329288258	0.921648675
68147	'Gar1'	415	440	553	70	217	324	469.3333333	203.6666667	-1.302175296	0.011746284	0.268666967
68149	'Otub2'	86	88	137	803	129	305	103.6666667	412.3333333	2.236509243	0.006840474	0.195923816
68151	'Wls'	2451	2516	2404	4740	3810	4814	2457	4454.666667	0.956581494	0.011588141	0.267046959
68152	'Fam133b'	680	702	623	733	596	595	668.3333333	641.3333333	0.063094789	0.877047097	0.981341203
68153	'Gtf2e2'	1742	1570	2352	1150	1739	1903	1888	1597.333333	-0.252365641	0.504299127	0.972790461
68159	'Stx19'	0	0	0	0	0	4	0	1.333333333	2.683352581	0.500796233	0.972790461
68165	'Fdx2'	193	211	313	98	235	289	239	207.3333333	-0.281816386	0.555851218	0.972790461
68166	'Spire1'	1525	1389	1855	517	1071	1416	1589.666667	1001.333333	-0.720907548	0.071275844	0.591632421
68169	'Ndnf'	59	49	103	40	66	79	70.33333333	61.66666667	-0.2376105	0.661547708	0.972790461
68170	'Iftap'	556	643	379	59	492	535	526	362	-0.627566681	0.342005502	0.925308103
68172	'Rpl39l'	144	141	47	2	79	332	110.6666667	137.6666667	0.199383309	0.86161875	0.976863639
68175	'4930591A17Rik'	0	0	0	0	1	0	0	0.333333333	1.020273531	0.802557913	0.972790461
68176	'Inka1'	109	117	126	101	141	181	117.3333333	141	0.273669455	0.439443748	0.967427574
68177	'Ebpl'	231	269	93	50	223	275	197.6666667	182.6666667	-0.144983672	0.811143763	0.972790461
68178	'Cgnl1'	3569	3794	1398	4839	4614	4565	2920.333333	4672.666667	0.831360313	0.070415853	0.589465353
68183	'Bcas2'	1207	1293.4	1261.6	561	908	1411	1253.98	960	-0.405589663	0.238828209	0.864053647
68184	'Denr'	1652	1691	1761	670	1269	1733	1701.333333	1224	-0.506574482	0.142522002	0.748929878
68185	'Coa4'	147	172	172	34	119	158	163.6666667	103.6666667	-0.740258426	0.150821498	0.759755159
68187	'Fam135a'	2618	2681	1478	1032	2607	1649	2259	1762.666667	-0.325476257	0.395374056	0.954014376
68188	'Sympk'	4206	4212	2735	3981	3041	3635	3717.666667	3552.333333	0.074049388	0.853167434	0.975734242
68192	'Leprotl1'	331	342	601	482	511	613	424.6666667	535.3333333	0.345956384	0.466812754	0.972790461
68193	'Rpl24'	5824	6345	14385	10986	5092	6905	8851.333333	7661	-0.101510863	0.880367792	0.982087554
68194	'Ndufb4'	302.48	352.26	215.73	307.01	457.94	539.74	290.1566667	434.8966667	0.627162366	0.027410447	0.404916403
68195	'Rnaset2b'	226.28	172.74	49.44	105.52	885.13	2302.84	149.4866667	1097.83	2.802727955	0.001528082	0.084453179
68196	'Hsbp1'	1178	1246	1197	971	1163	1441	1207	1191.666667	0.020571464	0.942695009	0.996301887
68197	'Ndufc2'	1110	1243	1448	765	1053	1375	1267	1064.333333	-0.252837154	0.461672625	0.972790461
68198	'Ndufb2'	282	335	278	157	274	307	298.3333333	246	-0.270799693	0.357551087	0.932505998
68201	'Ccdc34'	382	383	326	141	309	297	363.6666667	249	-0.550122391	0.075002833	0.603900623
68202	'Ndufa5'	543	536	483	326	397	532	520.6666667	418.3333333	-0.279007291	0.324153365	0.919644666
68203	'Diras2'	32	34	181	4	40	25	82.33333333	23	-2.069489451	0.037531499	0.459930654
68205	'Urm1'	625	665	526	255	500	608	605.3333333	454.3333333	-0.420003792	0.167399908	0.781391371
68209	'Rnaseh2c'	188	214	84	283	234	316	162	277.6666667	0.915676524	0.047307273	0.50504201
68212	'Tmbim4'	227	281	248	336	267	407	252	336.6666667	0.497666543	0.20962226	0.837080112
68214	'Gsto2'	138	110	17	5	61	102	88.33333333	56	-0.708061887	0.465714485	0.972790461
68215	'Fam98b'	962	1029	787	326	743	937	926	668.6666667	-0.49284063	0.157982981	0.768890221
68219	'Nudt21'	1348	1410	2126	1130	1079	1562	1628	1257	-0.35186073	0.421988499	0.959445304

68221	'Wfdc15a'	12	20	2	0	15	40	11.33333333	18.33333333	0.616574535	0.657875661	0.972790461
68222	'Fam166a'	5	8	2	1	1	1	5	1	-2.212518421	0.128848898	0.72577634
68226	'Efcab2'	939	976	155	35	536	485	690	352	-1.01435678	0.254028906	0.873052317
68229	'Spindoc'	913	896	958	486	695	650	922.3333333	610.3333333	-0.563289111	0.0587526	0.552152463
68233	'Fam229a'	0	2	2	2	1	1	1.333333333	1.333333333	0.111693678	0.954260064	0.99943739
68235	'Mturn'	741	665	289	645	708	577	565	643.3333333	0.329435006	0.462574728	0.972790461
68236	'Gtsf1l'	0	0	0	0	0	1	0	0.333333333	1.020273531	0.802557913	0.972790461
68239	'Krt42'	0	0	0	1	0	0	0	0.333333333	1.020273531	0.802557913	0.972790461
68240	'Rpa3'	272	293	164	169	222	262	243	217.6666667	-0.093242069	0.770373412	0.972790461
68241	'Mcrip2'	97	98	153	276	100	206	116	194	0.875982485	0.175660989	0.794780279
68243	'A930018P22Rik'	2	3	4	0	6	16	3	7.333333333	1.106984848	0.449965042	0.970590732
68250	'Ciao2a'	920	941	1163	538	937	1210	1008	895	-0.202429567	0.576401787	0.972790461
68251	'Babam1'	802	902	620	405	956	1146	774.6666667	835.6666667	0.091852276	0.794196905	0.972790461
68255	'Tmem86b'	48	41	47	46	37	30	45.33333333	37.66666667	-0.149155478	0.787133631	0.972790461
68259	'Ift80'	1708	1669	427	1289	1355	1404	1268	1349.333333	0.240680218	0.654957908	0.972790461
68260	'Trmt12'	256	253	108	92	236	285	205.6666667	204.3333333	-0.002932701	0.995001473	0.999562152
68262	'Agpat4'	134	115	384	283	211	218	211	237.3333333	0.204410133	0.768140461	0.972790461
68263	'Pdhb'	1606	1595	1593	1501	1628	1992	1598	1707	0.14870177	0.625478058	0.972790461
68265	'Iqcf3'	0	0	0	0	0	1	0	0.333333333	1.020273531	0.802557913	0.972790461
68267	'Slc25a22'	365	379	624	637	485	707	456	609.6666667	0.467024819	0.349696058	0.92886351
68268	'Zdhhc21'	864.81	976.08	373.46	585.78	1140.9	790.89	738.1166667	839.1866667	0.260263235	0.539926904	0.972790461
68270	'Dnaaf1'	176.27	180.3	7.67	1.08	64.37	61.07	121.4133333	42.17333333	-1.566737593	0.203682224	0.832079769
68272	'Rbm28'	1243.1	1259.5	829.05	1696.9	1717	1447	1110.556667	1620.296667	0.669725964	0.072773683	0.596837647
68273	'Pomgnt1'	979	1049	542	488	1365	1304	856.6666667	1052.333333	0.296547197	0.45835132	0.972790461
68275	'Rpa1'	1270.1	1431	603	884	1341	1554.08	1101.376667	1259.693333	0.258886125	0.4802587	0.972790461
68276	'Toe1'	459.41	418.43	618.58	145.95	451.02	457.63	498.8066667	351.5333333	-0.588469498	0.214958721	0.842760284
68277	'2310057M21Rik'	395	383	267	221	310	386	348.3333333	305.6666667	-0.145833991	0.600223719	0.972790461
68278	'Ddx39a'	559	684	524	340	454	482	589	425.3333333	-0.420830298	0.120896375	0.712100232
68279	'Mcoln2'	3	5	4	8	5	13	4	8.666666667	1.169626688	0.226910706	0.854001521
68281	'4930430F08Rik'	187.29	177.94	460.42	460.48	197.67	290.58	275.2166667	316.2433333	0.305757565	0.666867811	0.972790461
68283	'9530077C05Rik'	116	96.1	22	84	100	68	78.03333333	84	0.272881343	0.673547263	0.972790461
68291	'Mto1'	413	401	325	586	356	430	379.6666667	457.3333333	0.428151206	0.366129435	0.936406787
68292	'Stt3b'	3887	3699	4544	4419	4367	5793	4043.333333	4859.666667	0.310404726	0.392072319	0.952648938
68294	'Mfsd10'	460	439	540	260	631	565	479.6666667	485.3333333	-0.020927088	0.955966279	0.999493374
68295	'Aar2'	886	950	589	732	870	1040	808.3333333	880.6666667	0.196956081	0.493281708	0.972790461
68298	'Ncapd2'	1083	1116	630	615	1419	757	943	930.3333333	0.031336059	0.938056281	0.994698142
68299	'Vps53'	1115	1015	609	450	804	1101	913	785	-0.20071911	0.562668881	0.972790461
68303	'Fam114a1'	933	929	1590	608	930	1251	1150.666667	929.6666667	-0.352303165	0.43360551	0.964992995
68304	'Poglut3'	1228	1266	588	118.39	1562	1540	1027.333333	1073.463333	-0.021601807	0.97719956	0.999493374
68307	'Lrriq4'	3	3	0	0	3	5	2	2.666666667	0.375361183	0.848752214	0.975686757

68310	'Zmym1'	811.9	761.88	302.13	308.47	518.53	517.01	625.3033333	448.0033333	-0.410029237	0.321381466	0.917570871
68312	'Gstm7'	313	396	64	117	666	380	257.6666667	387.6666667	0.60310134	0.407407051	0.957157474
68316	'Apoo'	449	444	271	202	385	677	388	421.3333333	0.106410119	0.800363945	0.972790461
68318	'Aph1c'	105.15	97.18	72.52	136.31	117.65	135.86	91.61666667	129.94	0.613874868	0.132131285	0.729631651
68323	'Nudt22'	78	77	85	126	117	133	80	125.3333333	0.721645871	0.081486496	0.620681593
68327	'Tsr3'	268	314	876	151	318	450	486	306.3333333	-0.796234811	0.224367918	0.850759465
68328	'Rab13'	468	495	611	247	493	485	524.6666667	408.3333333	-0.388212013	0.287314573	0.894554788
68332	'Sdhaf1'	158	158	186	406	175	207	167.3333333	262.6666667	0.837790088	0.169916467	0.785454025
68337	'Crip2'	349	388	530	340	590	637	422.3333333	522.3333333	0.279553319	0.477354703	0.972790461
68338	'Golt1a'	1	1	0	1	0	1	0.666666667	0.666666667	0.219813875	0.93648942	0.994413066
68339	'Ccdc88c'	873	739	119	715	968	1568	577	1083.666667	1.000072877	0.13069298	0.728882802
68342	'Ndufb10'	776	750	854	996	618	1061	793.3333333	891.6666667	0.263979448	0.562085886	0.972790461
68344	'Tmem174'	10	15	0	0	47	27	8.333333333	24.66666667	1.530654841	0.359298196	0.933706718
68346	'Sirt5'	123	136	127	38	165	163	128.6666667	122	-0.151508151	0.770025375	0.972790461
68347	'Mettl26'	171	219	97	322	320	388	162.3333333	343.3333333	1.192597356	0.002867161	0.119683851
68348	'Serpina1f'	4	5	0	0	1	1	3	0.666666667	-2.123926921	0.334185578	0.921648675
68349	'Ndufs3'	1069	1139	1132	372	1032	1191	1113.333333	865	-0.419954319	0.295491317	0.90029104
68350	'Mul1'	741	727	503	651	673	906	657	743.3333333	0.255764555	0.406172395	0.957157474
68352	'Aspdh'	1	0	1	0	1	0	0.666666667	0.333333333	-0.894008961	0.803817041	0.972790461
68364	'0610030E20Rik'	615	594	622	309	582	518	610.3333333	469.6666667	-0.374425213	0.21276992	0.841028891
68365	'Rab14'	3854	4069	4823	2610	3496	4242	4248.666667	3449.333333	-0.291597604	0.375564189	0.940406075
68366	'Tmem129'	373.41	391.43	244.89	489.6	337.18	379.99	336.5766667	402.2566667	0.418616592	0.355726631	0.931809687
68371	'Pbld1'	16	14	4	10	23.58	10	11.33333333	14.52666667	0.425894912	0.601667143	0.972790461
68375	'Ndufa8'	891	888	936	722	818	1053	905	864.3333333	-0.028013091	0.928395976	0.993096978
68379	'Ciz1'	894	811	1280	653	1013	819	995	828.3333333	-0.260210064	0.528615661	0.972790461
68385	'Tlcd1'	223	209	141	65	276	222	191	187.6666667	-0.062524755	0.897999317	0.985794067
68393	'Mogat1'	3	3	3	13	0	7	3	6.666666667	1.407596801	0.350237777	0.92886351
68394	'Ccdc163'	220	223	164	258	212	184	202.3333333	218	0.25200511	0.569408002	0.972790461
68395	'LOC68395'	13.24	16.93	1.72	6.17	6.51	10.48	10.63	7.72	-0.320421727	0.762738651	0.972790461
68396	'Nat8'	1	0	0	0	2	2	0.333333333	1.333333333	1.768945179	0.572584055	0.972790461
68401	'G6pc3'	340	309	205	152	443	471	284.6666667	355.3333333	0.299463498	0.465097663	0.972790461
68404	'Nrn1'	951	858	9190	1980	1093	2433	3666.333333	1835.333333	-1.068879039	0.278140472	0.889355605
68416	'Sycn'	0	1	0	2	0	0	0.333333333	0.666666667	1.337350669	0.73871782	0.972790461
68420	'Ankrd13a'	1295	1395	1254	2106	1787	1795	1314.666667	1896	0.642053081	0.101540316	0.671759705
68421	'Lmbrd1'	750	709	659	2361	1100	1717	706	1726	1.458528418	0.005370294	0.17331813
68423	'Ankrd13d'	153	146	74	99	163	148	124.3333333	136.6666667	0.20144346	0.59235909	0.972790461
68427	'Slc39a13'	414	350	364	307	506	458	376	423.6666667	0.196493779	0.497890515	0.972790461
68428	'Steap3'	351	381	107	629	358	369	279.6666667	452	0.934141152	0.135495678	0.736012522
68431	'Fbxl15'	73.42	83.86	129.37	58.4	87.85	91.02	95.55	79.09	-0.287387637	0.547241654	0.972790461
68436	'Rpl34'	575.37	622.9	2509.1	6071.4	3608.8	4645.84	1235.783333	4775.333333	1.978514713	0.010492831	0.253803685

68440	'Dusp23'	61	60	164	33	61	70	95	54.66666667	-0.884961389	0.169509721	0.785100556
68441	'Rraga'	954	984	1526	2484	1220	1719	1154.666667	1807.666667	0.773774868	0.181739707	0.802050906
68449	'Tbc1d10b'	1392	1383	1356	1333	1259	1721	1377	1437.666667	0.12694011	0.703086502	0.972790461
68453	'Gpihbp1'	148	153	10	0	284	215	103.6666667	166.3333333	0.630837998	0.662646387	0.972790461
68458	'Ppp1r14a'	66	69	59	54	47	54	64.66666667	51.66666667	-0.231566551	0.597214869	0.972790461
68460	'Dhrs7c'	0	0	0	0	1	0	0	0.333333333	1.020273531	0.802557913	0.972790461
68463	'Mrpl14'	399	422	579	666	367	476	466.6666667	503	0.233509191	0.668071281	0.972790461
68465	'Adipor2'	1764	1855	1444	3994	1888	2633	1687.666667	2838.333333	0.93775854	0.075047908	0.603900623
68468	'Ly6g6c'	5	0	0	0	1	0	1.666666667	0.333333333	-2.063449554	0.601470058	0.972790461
68472	'Tmem126b'	221	253	225	98	221	252	233	190.3333333	-0.316841617	0.376585576	0.941504733
68473	'Mob1b'	830	859	495	900	553	762	728	738.3333333	0.186441042	0.688849598	0.972790461
68475	'Ssna1'	461	522	426	439	411	463	469.6666667	437.6666667	-0.009430467	0.978375056	0.999493374
68477	'Rmnd5a'	3431	3436	3351	2524	3393	3095	3406	3004	-0.131766715	0.64281205	0.972790461
68479	'Phf5a'	593	618	462	410	609	706	557.6666667	575	0.078789514	0.748496614	0.972790461
68480	'Card19'	185	181	522	539	155	319	296	337.6666667	0.312953512	0.692161734	0.972790461
68481	'Mpzl1'	1899	1873	1699	5432	2297	2456	1823.666667	3395	1.118390879	0.062233459	0.565378029
68487	'Tmem140'	28.95	30.75	43.62	33.14	27.26	32.39	34.44	30.93	-0.072764751	0.899487246	0.986106073
68490	'Zfp579'	729	674	530	516	955	449	644.3333333	640	0.057608408	0.889592371	0.984980969
68493	'Ndufaf4'	336	325	252	341	409	432	304.3333333	394	0.442431956	0.121649685	0.712603747
68497	'Arel1'	1477	1440	654	936	1602	1498	1190.333333	1345.333333	0.242324761	0.506621558	0.972790461
68498	'Tspan11'	14	23	92	289	47	133	43	156.3333333	1.99942282	0.048387255	0.51079317
68499	'Mrpl53'	187	184	241	156	215	227	204	199.3333333	-0.020737549	0.955601899	0.999493374
68501	'Nsmce2'	507	528	829	399	461	561	621.3333333	473.6666667	-0.384554185	0.374729634	0.940406075
68505	'Vps51'	802	804	611	213	806	805	739	608	-0.333191389	0.460912972	0.972790461
68507	'Ppfia4'	268	305	64	96	213	114	212.3333333	141	-0.49166149	0.421486777	0.959296626
68509	'Ptx4'	18	18	2	0	7	0	12.66666667	2.333333333	-2.43229876	0.151281804	0.75995796
68510	'Ints1'	2345	2208	520	1188	2039	2233	1691	1820	0.188578387	0.729564013	0.972790461
68511	'Dcdc2c'	57.23	77.92	16.57	21.28	25.46	15.43	50.57333333	20.72333333	-1.13530715	0.10614085	0.679124905
68512	'Tomm5'	282	315	594	437	298	396	397	377	-0.014181741	0.980145494	0.999493374
68514	'Micu2'	1676	1735	1411	538	1678	1812	1607.333333	1342.666667	-0.306216128	0.450463626	0.970590732
68515	'Myadml2'	4	1	2	0	1	2	2.333333333	1	-1.299657215	0.483822897	0.972790461
68519	'Eml1'	495	493	334	197	431	320	440.6666667	316	-0.451994905	0.163933843	0.776592614
68520	'Zfyve21'	447	453	1311	307	546	811	737	554.6666667	-0.524266207	0.406520941	0.957157474
68521	'Fam189b'	382	398	250	222	502	347	343.3333333	357	0.08788068	0.800438537	0.972790461
68523	'Ciao2b'	370	359	685	312	244	378	471.3333333	311.3333333	-0.570735901	0.279215585	0.889355605
68524	'Wipf2'	813	810	795	526	582	665	806	591	-0.390200785	0.202168596	0.8304652
68525	'Evc2'	693	646	265	757	980	910	534.6666667	882.3333333	0.832378052	0.044748292	0.492557243
68526	'Gpr155'	153	143	14	117	240	173	103.3333333	176.6666667	0.883199559	0.244670525	0.866091613
68527	'Ucma'	0	0	1	0	2	8	0.333333333	3.333333333	3.058480901	0.248611762	0.869491261
68528	'Smim6'	7	3	0	2	2	5	3.333333333	3	-0.057079488	0.970649335	0.999493374

68533	'Mphosph6'	232	244	627	79	280	535	367.6666667	298	-0.485627534	0.503110438	0.972790461
68537	'Mrpl13'	610	659	714	344	525	663	661	510.6666667	-0.3764026	0.241198692	0.86449675
68539	'Tmem109'	424	433	293	507	705	699	383.3333333	637	0.795266761	0.002898856	0.120071993
68544	'Trir'	1104	1086	1527	846	1064	1163	1239	1024.333333	-0.258888843	0.494792866	0.972790461
68545	'Ecscr'	135	131	146	40	236	178	137.3333333	151.3333333	0.046998976	0.93528646	0.994413066
68549	'Sgo2a'	513	545	163	102	367	234	407	234.3333333	-0.76936198	0.1697994	0.785454025
68550	'Tefm'	272	286	177	63	249	308	245	206.6666667	-0.297961496	0.561351014	0.972790461
68552	'Smim14'	1123	1162	2897	1125	1305	1981	1727.333333	1470.333333	-0.28458232	0.621527176	0.972790461
68553	'Col6a4'	479	436	48	18	503	163	321	228	-0.516776576	0.61562023	0.972790461
68554	'Cebpzos'	248	250	143	139	209	312	213.6666667	220	0.072641484	0.839312949	0.974723675
68556	'Uckl1'	296	316	261	259	377	328	291	321.3333333	0.194461283	0.49579481	0.972790461
68558	'Ankra2'	451	468	492	454	603	541	470.3333333	532.6666667	0.224797535	0.483946501	0.972790461
68559	'Pdrg1'	925	992	467	1195	908	1089	794.6666667	1064	0.578374478	0.192875178	0.815782621
68563	'Dpm3'	102	128	132	231	130	151	120.6666667	170.6666667	0.647953327	0.236366829	0.862272407
68564	'Nufip2'	2649	2726	2294	2307	1928	2216	2556.333333	2150.333333	-0.139242853	0.705624373	0.972790461
68565	'Mrps18a'	482	533	647	319	408	628	554	451.6666667	-0.303497035	0.424851181	0.960285372
68566	'Caly'	4	4	3	2	4	4	3.666666667	3.333333333	-0.117444506	0.917498146	0.990547571
68567	'Cgref1'	7	4	32	4	9	6	14.33333333	6.333333333	-1.2964126	0.240132585	0.86449675
68572	'Mrpl58'	518	526	377	241	454	516	473.6666667	403.6666667	-0.218938887	0.445117345	0.969888068
68576	'Lamtor5'	783	769	619	417	574	838	723.6666667	609.6666667	-0.22562667	0.431401559	0.963623551
68581	'Tmed10'	2913.1	2806.2	4196.4	3440.7	3533.8	4999.92	3305.246667	3991.486667	0.288757389	0.484735022	0.972790461
68585	'Rtn4'	4999.3	5101.4	5451.5	6361.9	6212.3	7583.09	5184.073333	6719.113333	0.437386537	0.202595997	0.830701895
68588	'Cthrc1'	55	45	30	877	85	473	43.33333333	478.3333333	3.752785503	1.39E-05	0.003351794
68591	'Mocos'	20	13	321	94	30	94	118	72.66666667	-0.745832807	0.53476224	0.972790461
68592	'Syf2'	728	657	1458	1056	746	1036	947.6666667	946	0.042970063	0.93979527	0.995607401
68597	'Ccdc167'	466.27	464.18	197.63	200.43	455.58	314.01	376.0266667	323.34	-0.163435192	0.703321265	0.972790461
68598	'Dnajc8'	1412	1532	1959	1790	1562	1965	1634.333333	1772.333333	0.17766028	0.670144875	0.972790461
68603	'Pmvk'	257	264	359	315	269	232	293.3333333	272	-0.015255228	0.97495053	0.999493374
68606	'Ppm1f'	1350	1302	603	543	1295	1252	1085	1030	-0.051091857	0.898404453	0.98586843
68607	'Serhl'	188.12	196.06	126.36	34.29	240.74	261.41	170.18	178.8133333	-0.013256783	0.983305841	0.999493374
68611	'Mrpl28'	513	602	591	944	618	858	568.6666667	806.6666667	0.61847604	0.169105607	0.784741705
68612	'Ube2c'	273	323	226	61	335	677	274	357.6666667	0.275310786	0.685351587	0.972790461
68614	'Letmd1'	770	782	809	296.04	732	654	787	560.68	-0.517878601	0.13842519	0.739741822
68616	'Gdpd3'	9	6	13	19	5	6	9.333333333	10	0.30586755	0.765952288	0.972790461
68617	'Mtcl1'	1892	1944	880	2013	2454	2539	1572	2335.333333	0.673682803	0.067387633	0.579853218
68618	'Eola1'	180	216	306	184	226	278	234	229.3333333	-0.027557027	0.949284999	0.997777885
68625	'Cfap57'	95	69	12	6	50	30	58.66666667	28.66666667	-1.033297247	0.241188761	0.86449675
68626	'Elac2'	831	783	467	546	670	929	693.6666667	715	0.105510289	0.740022246	0.972790461
68628	'Fbxw9'	484	517	701	84	577	589	567.3333333	416.6666667	-0.582294809	0.363293431	0.935012144
68631	'Cryl1'	812	946	188	173	1071	1185	648.6666667	809.6666667	0.294926475	0.684291572	0.972790461

68632	'Myct1'	46	41	18	0	105	87	35	64	0.768318967	0.536291683	0.972790461
68634	'Tm2d3'	359	422	158	273	450	650	313	457.6666667	0.589789395	0.172289844	0.790015071
68635	'1110025M09Rik'	195.56	230.61	68.23	190.99	187.74	151.78	164.8	176.8366667	0.268429235	0.618820267	0.972790461
68636	'Fahd1'	244	288	257	102	233	300	263	211.6666667	-0.349941343	0.363670998	0.935022643
68642	'Tmem216'	154	174	109	88	164	175	145.6666667	142.3333333	-0.009307737	0.977157021	0.999493374
68644	'Abhd14a'	170	184	144	125	321	323	166	256.3333333	0.606891816	0.096065394	0.662457477
68646	'Nadk2'	1236	1219	3157	4234	1130	2109	1870.666667	2491	0.567266268	0.468889962	0.972790461
68652	'Tab2'	2954	2993	4786	4136	2767	3083	3577.666667	3328.666667	-0.005626061	0.991526779	0.999493374
68653	'Samm50'	2132	2231	1788	1357	1905	2193	2050.333333	1818.333333	-0.135727059	0.557468649	0.972790461
68655	'Fndc1'	569	484	115	65	309	117	389.3333333	163.6666667	-1.213496658	0.083375391	0.627571099
68659	'Gask1b'	1430	1502	1315	1371	2420	2354	1415.666667	2048.333333	0.547944948	0.028001918	0.40918746
68666	'Svop'	0	1	0	0	2	2	0.333333333	1.333333333	1.768901033	0.57287116	0.972790461
68667	'Trpm4'	343	369	364	385	494	401	358.6666667	426.6666667	0.311679003	0.361840484	0.93373993
68671	'Pcyt2'	478	436	457	674	546	485	457	568.3333333	0.439714795	0.32014773	0.917050664
68675	'Fam172a'	840.57	902.96	860	281.96	847	757	867.8433333	628.6533333	-0.509458002	0.192405041	0.814156386
68678	'Smtnl1'	9	20	2	1	36	11	10.33333333	16	0.605417286	0.625512126	0.972790461
68680	'Fitm1'	3	4	2	0	0	1	3	0.333333333	-2.979796787	0.153453955	0.765299624
68682	'Slc44a2'	1139	1083	1035	2465	1520	1993	1085.666667	1992.666667	1.015322806	0.026126368	0.397137149
68691	'Kansl1l'	1531.1	1524.7	627.45	747.59	1767.9	1210	1227.753333	1241.84	0.068542209	0.873668378	0.980171118
68693	'Hnrnpul2'	6857	7072	6663	4219	5425	5983	6864	5209	-0.356367461	0.174321466	0.793128181
68695	'Hddc3'	442.65	434.3	272.84	247.06	435.04	528.73	383.2633333	403.61	0.099627224	0.74744249	0.972790461
68701	'Ppp1r27'	0	0	1	0	1	0	0.333333333	0.333333333	0.058500858	0.988561293	0.999493374
68703	'Rere'	4958	4597	4516	9695	4349	5605	4690.333333	6549.666667	0.673163682	0.226341465	0.853679947
68705	'Gtf2f2'	493	470	434	382	435	512	465.6666667	443	-0.015442814	0.957711127	0.999493374
68708	'Rabl2'	290	234	242	154	399	292	255.3333333	281.6666667	0.129755032	0.727932211	0.972790461
68709	'Cilp2'	67	55	46	209	40	96	56	115	1.30819939	0.091828184	0.651188901
68713	'Ifitm1'	989	1061	315	1101	1195	757	788.3333333	1017.666667	0.545632304	0.318668308	0.916136994
68718	'Rnf166'	586	567	370	835	744	678	507.6666667	752.3333333	0.707038326	0.079610259	0.617305615
68721	'1110032A03Rik'	333	374	205	108	304	348	304	253.3333333	-0.278233669	0.510327503	0.972790461
68723	'Hrnr'	6	2	1	0	6	1	3	2.333333333	-0.395055781	0.822686813	0.972790461
68724	'Arl8a'	521	482	1674	490	598	777	892.3333333	621.6666667	-0.587864342	0.370125799	0.937712122
68725	'1110032F04Rik'	12	20	11	8	61	65	14.33333333	44.66666667	1.558262835	0.04476851	0.492557243
68728	'Trp53inp2'	664	773	971	2481	1166	1270	802.6666667	1639	1.201949881	0.04538554	0.496758712
68729	'Trim37'	3464	3339	1349	550	2314	2922	2717.333333	1928.666667	-0.528876919	0.353271669	0.930050349
68730	'Dus1l'	679	665	270	380	645	936	538	653.6666667	0.318789791	0.459089169	0.972790461
68731	'Rbfa'	343	364	297	542	441	561	334.6666667	514.6666667	0.725812849	0.053620456	0.533767484
68732	'Carmil1'	537	488	857	424	607	649	627.3333333	560	-0.178973886	0.68192682	0.972790461
68734	'Ppp4r3a'	2123	2134	1722	1458	2167	2062	1993	1895.666667	-0.026184006	0.911405053	0.989773583
68735	'Mrps18c'	507	465	411	191	407	501	461	366.3333333	-0.350212627	0.295829703	0.90029104
68736	'Tyw5'	190	172	258	76	195	207	206.6666667	159.3333333	-0.441287188	0.335241157	0.922335099

68737	'Angel1'	463.59	470.68	78	689.61	473.67	649.44	337.4233333	604.24	1.043720553	0.119441848	0.710007992
68738	'Acss1'	344	339	166	286	480	454	283	406.6666667	0.585091678	0.09618002	0.662767142
68742	'Tmem219'	145	128	157	114	145	207	143.3333333	155.3333333	0.123746755	0.738608699	0.972790461
68743	'Anln'	1014	1102.9	290	274	724	546	802.3	514.6666667	-0.587377029	0.272405983	0.884738928
68744	'Zfp740'	3175.5	3220.9	2945.9	2451.6	3161.3	2981.39	3114.106667	2864.766667	-0.063245408	0.818754877	0.972790461
68750	'Rreb1'	1862	1853	1476	1779	2044	2363	1730.333333	2062	0.319827016	0.231860381	0.858905111
68753	'Mybphl'	4	2	1	1	11	4	2.333333333	5.333333333	1.167475074	0.392793132	0.952648938
68755	'Cgrrf1'	200	211	127	295	212	262	179.3333333	256.3333333	0.661482355	0.132999069	0.731860581
68758	'Abhd11'	231	262	421	250	241	333	304.6666667	274.6666667	-0.133302628	0.780829289	0.972790461
68760	'Synpo2l'	26	50	6	3	18	6	27.33333333	9	-1.557194799	0.111555207	0.695601047
68764	'Cdhr3'	0	0	0	0	2	0	0	0.666666667	1.801491674	0.656112935	0.972790461
68767	'Washc1'	719	752.83	852.96	790	839.99	960	774.93	863.33	0.207328049	0.554018397	0.972790461
68768	'Krtap4-6'	0	0	0	0	0	1	0	0.333333333	1.020273531	0.802557913	0.972790461
68770	'Phtf2'	712	748	322	311	630	541	594	494	-0.21373311	0.5824777	0.972790461
68774	'Ms4a6d'	16	10	8	46	36	92	11.33333333	58	2.407369459	1.15E-04	0.015350347
68775	'Atp6v1c2'	18	14	8	9	7	4	13.33333333	6.666666667	-0.833505667	0.327851259	0.921103612
68776	'Taf1l'	903	913.53	683	305	784.85	745.75	833.1766667	611.8666667	-0.459130832	0.17456125	0.793295795
68777	'Tmem53'	66	98	59	31	105	104	74.33333333	80	0.07462118	0.879606525	0.981807495
68778	'Gucd1'	745	797	631	318	668	617	724.3333333	534.3333333	-0.433497872	0.12770213	0.723917691
68792	'Srpx2'	99	97	5	580	111	63	67	251.3333333	2.372245251	0.038393325	0.459930654
68794	'Flnc'	3004	2656	1121	3077	2949	2770	2260.333333	2932	0.529434384	0.247789681	0.869184025
68795	'Ubr3'	3843	3763	1980	2794	2971	3697	3195.333333	3154	0.080151632	0.817592739	0.972790461
68796	'Tmem214'	1018	959	1288	699	1339	1508	1088.333333	1182	0.083995669	0.820144094	0.972790461
68797	'Pdgfrl'	61	58	44	109	112	142	54.33333333	121	1.230212165	0.001041279	0.067565838
68799	'Rgmb'	458	430	1261	2686	577	890	716.3333333	1384.333333	1.14696101	0.185489522	0.805706284
68800	'Prr32'	6	8	10	1	3	1	8	1.666666667	-2.270178915	0.055525222	0.540802007
68801	'Elovl5'	1496	1470	853	5904	2270	2732	1273	3635.333333	1.770498508	0.004324869	0.153118082
68802	'Mypn'	12	9	4	3	6	7	8.333333333	5.333333333	-0.601968393	0.511783226	0.972790461
68810	'Nexn'	122	111	222	21	226	295	151.6666667	180.6666667	0.064383812	0.936233146	0.994413066
68813	'Dock5'	427	492	292	207	337	400	403.6666667	314.6666667	-0.325363114	0.274068092	0.88525018
68815	'Btbd10'	1139	1168	659	821	908	977	988.6666667	902	-0.032231119	0.922427749	0.991300958
68816	'Ppil1'	507	561	311	191	525	668	459.6666667	461.3333333	-0.016065686	0.970092472	0.999493374
68817	'Ddi2'	954.13	889.57	540.78	627.17	687.96	773.55	794.8266667	696.2266667	-0.095932342	0.769353893	0.972790461
68818	'Zfand2b'	146	166	266	139	192	282	192.6666667	204.3333333	0.047832811	0.91960745	0.990815958
68828	'Sync'	49.17	37.36	53.29	26.46	61.61	89.73	46.60666667	59.26666667	0.279752186	0.594183648	0.972790461
68832	'Ldah'	515	549	438	310	602	581	500.6666667	497.6666667	0.002420874	0.992885664	0.999493374
68833	'Pdcl3'	616	647	1292	1729	890	1132	851.6666667	1250.333333	0.662027889	0.293157753	0.90029104
68836	'Mrpl52'	342	310	353	389	263	401	335	351	0.163369751	0.711542591	0.972790461
68837	'Foxk2'	1225	1196	1143	1383	1337	1342	1188	1354	0.280904259	0.425485969	0.960500978
68839	'Ankrd46'	1382	1451	1204	1031	1596	1428	1345.666667	1351.666667	0.048332302	0.846272913	0.975201539

68842	'Tulp4'	4560	4339.2	1549.4	4581.6	3605.7	4516.52	3482.863333	4234.596667	0.446999336	0.366947739	0.936808167
68845	'Pih1d1'	446	448	585	218	411	444	493	357.6666667	-0.492195851	0.186416226	0.807696881
68846	'Rnf208'	54	44	339	22	44	95	145.6666667	53.66666667	-1.650951023	0.079512707	0.617305615
68852	'Lrrn4cl'	86	79	157	183	115	298	107.3333333	198.6666667	0.892570899	0.133762956	0.733301819
68854	'Asb11'	0	1	3	1	0	0	1.333333333	0.333333333	-1.964084339	0.521028702	0.972790461
68857	'Dtwd2'	246	239	157	44	154	231	214	143	-0.636561666	0.223474295	0.850759465
68859	'Smim1'	988.42	1039.6	572.42	880.93	1351.7	1328.68	866.82	1187.1	0.516417417	0.081799332	0.621457906
68861	'Dipk2a'	2242	1968	2112	2413	4327	4299	2107.333333	3679.666667	0.80651346	0.004097628	0.148106649
68865	'Arv1'	640.64	661.2	286.03	282	694.18	439.09	529.29	471.7566667	-0.116634589	0.787896236	0.972790461
68867	'Rnf122'	310	305	696	512	458	478	437	482.6666667	0.173533755	0.762098491	0.972790461
68870	'Ak8'	13	17	13	0	19	34	14.33333333	17.66666667	0.153064649	0.896507999	0.985710789
68874	'Klhdc9'	19.5	24.96	5.11	2.13	57.24	45.7	16.52333333	35.02333333	1.055609671	0.310269944	0.910971113
68875	'Tmcc2'	328	327	414	216	1015	480	356.3333333	570.3333333	0.606574582	0.267659752	0.883600995
68876	'Atp23'	215	224	108	29	186	228	182.3333333	147.6666667	-0.36922498	0.568186584	0.972790461
68877	'Maf1'	1700	1863	2257	1038	1643	1943	1940	1541.333333	-0.345348143	0.316774352	0.915832418
68879	'Prpf6'	1996	1928	2604	2403	1820	2447	2176	2223.333333	0.107194121	0.808872276	0.972790461
68880	'Fam240b'	12	6	5	0	0	0	7.666666667	0	-5.295128064	0.004540522	0.158393409
68889	'Ubac2'	715	760	528	568	811	1015	667.6666667	798	0.294919895	0.253677895	0.87276948
68891	'Cd177'	6	10	2	2	18	42	6	20.66666667	1.712480728	0.120846427	0.712100232
68895	'Rasl11a'	129	140	341	171	151	215	203.3333333	179	-0.189964542	0.754750651	0.972790461
68897	'Disp1'	693	701	289	358	915	1137	561	803.3333333	0.52295488	0.25728548	0.877405867
68904	'Abhd13'	827	839	707	1079	955	1189	791	1074.333333	0.535534822	0.120219019	0.711140009
68910	'Zfp467'	923	955	760	120	1434	1136	879.3333333	896.6666667	-0.08062386	0.908534066	0.988823459
68911	'Pygo2'	1151	1203	1406	1149	1114	1266	1253.333333	1176.333333	-0.028803657	0.938970531	0.995379339
68915	'Vars2'	670	674	227	497	665	794	523.6666667	652	0.408244574	0.36168701	0.933706718
68916	'Cdkal1'	321	298	462	223	295	314	360.3333333	277.3333333	-0.372255315	0.364189779	0.935419628
68917	'Hint2'	121	124	158	81	158	203	134.3333333	147.3333333	0.091713681	0.825707209	0.972817749
68918	'1190005I06Rik'	8.48	6.41	14.11	0	21.57	17.57	9.666666667	13.04666667	0.247163206	0.843842248	0.975182082
68920	'1110065P20Rik'	424.22	477.6	332.24	266.98	527.15	522.53	411.3533333	438.8866667	0.107616134	0.705919984	0.972790461
68922	'Dnaic1'	91	91	10	0	38	20	64	19.33333333	-1.76938857	0.150696206	0.759755159
68925	'Rpap1'	1187	1164	465	299	1174	924	938.6666667	799	-0.235946109	0.648162027	0.972790461
68926	'Ubap2'	3289	2975	2147	1592	2523	3339	2803.666667	2484.666667	-0.15194337	0.590338517	0.972790461
68927	'Ptcd2'	609	627	532	466	588	717	589.3333333	590.3333333	0.045596902	0.861743869	0.976863639
68929	'Mospd3'	237	243	200	283	255	328	226.6666667	288.6666667	0.436479571	0.21516718	0.842760284
68936	'Smim11'	140	166	200	244	141	189	168.6666667	191.3333333	0.301382032	0.57131195	0.972790461
68938	'Aspscr1'	836	861	461	307	785	843	719.3333333	645	-0.15701561	0.686483675	0.972790461
68939	'Rasl11b'	271	271	1533	416	226	270	691.6666667	304	-1.156561334	0.183176786	0.804128465
68942	'Chmp2b'	1469	1418	3207	1535	1357	1891	2031.333333	1594.333333	-0.343444656	0.533453436	0.972790461
68943	'Pink1'	1426	1411	1925	737	1665	1867	1587.333333	1423	-0.214173689	0.596710612	0.972790461
68944	'Tmco1'	937	983	686	605	759	1003	868.6666667	789	-0.088802285	0.741258149	0.972790461

68947	'Chst8'	44	52	9	50	52	26	35	42.66666667	0.491277165	0.517056141	0.972790461
68948	'Fam216a'	968.18	1014.6	544.5	299.62	763.1	958.31	842.4133333	673.6766667	-0.332980582	0.417856312	0.957720764
68952	'Tlcd3b'	24	16	10	45	47	31	16.66666667	41	1.438277368	0.019654692	0.349228972
68953	'Chmp2a'	1021	1042	1262	663	934	1339	1108.333333	978.6666667	-0.191671602	0.587571154	0.972790461
68955	'Srrm4'	42	34	103	352	28	69	59.66666667	149.6666667	1.605687866	0.13000131	0.727578716
68957	'Paqr6'	4	4	0	1	3	3	2.666666667	2.333333333	-0.13011404	0.935802773	0.994413066
68961	'Phkg2'	433.9	408.93	916.59	201.56	459.52	494.36	586.4733333	385.1466667	-0.699857187	0.199352958	0.826477202
68964	'Ctc1'	1421	1430	691	330	1591	1318	1180.666667	1079.666667	-0.161387203	0.761215262	0.972790461
68966	'Ngdn'	444	463	1158	478	470	629	688.3333333	525.6666667	-0.406025199	0.489340911	0.972790461
68968	'Cdan1'	690	683	303	312	927	543	558.6666667	594	0.120634324	0.794294166	0.972790461
68969	'Eif1b'	1118	1034	2131	233	756	1380	1427.666667	789.6666667	-1.006418602	0.113723493	0.698948431
68970	'Dcaf12'	1697	1697	3300	1737	1560	2063	2231.333333	1786.666667	-0.300762339	0.556541856	0.972790461
68971	'Tamm41'	244	245	160	172	222	266	216.3333333	220	0.083107952	0.780222938	0.972790461
68972	'Tatdn3'	153	139.79	74	18	161	93	122.2633333	90.66666667	-0.480747943	0.479693881	0.972790461
68975	'Med27'	161	182	193	115	204	246	178.6666667	188.3333333	0.0600231	0.865731313	0.97826352
68977	'Haghl'	428.05	506.89	90.55	93.67	556.49	418.54	341.83	356.2333333	0.05684066	0.937172992	0.994594529
68979	'Nol11'	1039	1098	802	378	1093	1182	979.6666667	884.3333333	-0.180343621	0.641011289	0.972790461
68980	'Wdr53'	216	235	183	37	198	219	211.3333333	151.3333333	-0.56338563	0.318775838	0.916136994
68981	'Snrpa1'	530	468	676	435	488	627	558	516.6666667	-0.09138738	0.814097544	0.972790461
68988	'Prpf31'	1000	1084	642	201	969	1038	908.6666667	736	-0.362738231	0.495290911	0.972790461
68991	'Ssu72'	605	659	770	635	615	851	678	700.3333333	0.089611731	0.812132622	0.972790461
68992	'Zfp580'	168	165	138	86	143	197	157	142	-0.141167839	0.671729774	0.972790461
68995	'Mcts1'	306	340	408	265	339	363	351.3333333	322.3333333	-0.100775384	0.779929636	0.972790461
68999	'Anapc10'	344	356	403	176	316	379	367.6666667	290.3333333	-0.36111237	0.295126494	0.90029104
69008	'Cab39l'	867	882	560	860	665	1001	769.6666667	842	0.244534321	0.525702823	0.972790461
69009	'Thap7'	251	240	320	147	226	274	270.3333333	215.6666667	-0.338620601	0.36476819	0.935515019
69010	'Anapc13'	574	586	383	106	375	345	514.3333333	275.3333333	-0.932092148	0.032069568	0.433742328
69017	'Prrt2'	327	293	53	209	424	194	224.3333333	275.6666667	0.422973382	0.530616942	0.972790461
69019	'Spcs1'	856.35	1011.2	1070	723	985.49	1233.44	979.1933333	980.6433333	0.012064946	0.970182647	0.999493374
69020	'Zfp707'	206	196	112	154	247	247	171.3333333	216	0.389266652	0.235638861	0.861636582
69024	'Snx15'	233	254	252	183	318	244	246.3333333	248.3333333	0.035506312	0.914818958	0.99001241
69028	'Mitd1'	201	223	149	93	183	289	191	188.3333333	-0.034931088	0.930202925	0.99325468
69029	'Smdt1'	497	563	454	674	578	856	504.6666667	702.6666667	0.558579368	0.113846917	0.698948431
69032	'Lyz14'	0	0	2	1	0	1	0.666666667	0.666666667	-0.028559312	0.993076972	0.999493374
69034	'Nupr1l'	6	9	4	6	10	8	6.333333333	8	0.398785093	0.641015125	0.972790461
69035	'Zdhhc3'	1318	1284.2	1143.9	649	1298.5	1334.57	1248.673333	1094.03	-0.192906215	0.486691904	0.972790461
69036	'Zg16'	12	9	9	10	35	42	10	29	1.484910568	0.030635149	0.425756906
69038	'Tmem258'	563	661	956	328	404	582	726.6666667	438	-0.745079956	0.084881806	0.633597527
69046	'Isca1'	1269	1291	2430	2720	1355	1665	1663.333333	1913.333333	0.324889059	0.602451996	0.972790461
69047	'Atp2c2'	4	3	0	10	14	58	2.333333333	27.33333333	3.544098561	0.001872001	0.093866381

69048	'Slc30a5'	1067	1024	1031	1231	1340	1481	1040.666667	1350.666667	0.438847823	0.158571135	0.768890221
69049	'Nat8f5'	0	0	0	0	1	3	0	1.333333333	2.701386663	0.497818139	0.972790461
69051	'Pycr2'	257	283	469	333	377	397	336.3333333	369	0.149972817	0.749196003	0.972790461
69053	'1810013L24Rik'	1615	1667	2276	1801	1326	1523	1852.666667	1550	-0.164895224	0.727572498	0.972790461
69060	'Pnlip'	20	18	3	3	9	20	13.66666667	10.66666667	-0.357360171	0.716219507	0.972790461
69064	'Fuom'	118	141.94	164	28	114	91	141.3133333	77.66666667	-0.936986387	0.073777508	0.599788529
69065	'Chac1'	105	90	637	44	112	127	277.3333333	94.33333333	-1.741568507	0.04727507	0.50504201
69066	'1810010H24Rik'	73	71	42	4	36	48	62	29.33333333	-1.159418879	0.115804093	0.702825844
69068	'Tcim'	230	217	248	326	275	195	231.6666667	265.3333333	0.323507419	0.512586416	0.972790461
69069	'Tmem273'	3	1	0	5	2	2	1.333333333	3	1.441847508	0.400784738	0.955860942
69071	'Tmem97'	414	448	373	170	495	625	411.6666667	430	0.01068168	0.979910988	0.999493374
69072	'Ebna1bp2'	927	980	1394	436	872	1042	1100.333333	783.3333333	-0.543756842	0.190852991	0.813536636
69073	'Kdf1'	361	402	98	131	269	374	287	258	-0.11344434	0.839407031	0.974723675
69076	'Triap1'	222	235	396	220	245	402	284.3333333	289	0.001987882	0.996703749	0.999616639
69077	'Psmd11'	1613	1619	1920	1971	1430	1857	1717.333333	1752.666667	0.124738615	0.77469814	0.972790461
69080	'Gmppa'	405	411	764	342	403	510	526.6666667	418.3333333	-0.34447438	0.474910474	0.972790461
69082	'Zc3h15'	2406	2386	3601	1638	2057	2525	2797.666667	2073.333333	-0.432750656	0.279783689	0.889355605
69083	'Sult1c2'	10	24	2	0	10	4	12	4.666666667	-1.380353768	0.31834321	0.916136994
69085	'Zcchc9'	287	307	573	397	358	375	389	376.6666667	-0.004763879	0.992822689	0.999493374
69089	'Oxa1l'	1543.9	1410	2204	767.74	1335	1395	1719.306667	1165.913333	-0.589830378	0.138436796	0.739741822
69090	'Ascc1'	941	954.01	692	429	728	865	862.3366667	674	-0.336169687	0.202868619	0.83083072
69091	'Vps26b'	1885.8	1895.8	2285.4	1278.8	1815.8	1771.68	2022.363333	1622.093333	-0.298611252	0.362084679	0.934050734
69094	'Tmem160'	119	124	162	251	139	245	135	211.6666667	0.745074554	0.157343264	0.768337387
69097	'Trim15'	9	8	1	24	5	8	6	12.33333333	1.384686519	0.235833396	0.861636582
69101	'Ydjc'	59.88	62.92	268.98	12	60.87	112.62	130.5933333	61.83	-1.314911741	0.146966245	0.753611197
69104	'Marchf5'	2107	2039	1901	1094	1941	2077	2015.673333	1704	-0.23681095	0.359883231	0.933706718
69106	'Stoml1'	248	274	230	181	439	389	250.6666667	336.3333333	0.412017681	0.221643863	0.849403976
69109	'Ccnq'	225	241	151	184	227	299	205.6666667	236.6666667	0.260294741	0.401660691	0.955860942
69113	'Alkbh3'	434	401	383	172	362	279	406	271	-0.574228989	0.070516141	0.589465353
69116	'Ubr4'	10483	9369	3928	8998	8090	8389	7926.666667	8492.333333	0.255098212	0.577813024	0.972790461
69121	'Chrdl2'	5	4	8	1	5	8	5.666666667	4.666666667	-0.390849812	0.72591023	0.972790461
69123	'Eci3'	12	16.01	3.13	4	20	17	10.38	13.66666667	0.40823885	0.652156165	0.972790461
69125	'Cnot8'	1197	1277	1321	956	1209	1095	1265	1086.666667	-0.167471202	0.602406549	0.972790461
69126	'Rbis'	407	392	639	384	378	373	479.3333333	378.3333333	-0.292793673	0.531719272	0.972790461
69129	'Pex11g'	154	183	91	35	174	199	142.6666667	136	-0.121391887	0.83441467	0.974723675
69131	'Cdk12'	4445	4457	1708	1022	2750	2334	3536.666667	2035.333333	-0.76864785	0.089590598	0.646660839
69134	'Fam25c'	0	0	1	0	0	0	0.333333333	0	-0.903279821	0.824807108	0.972790461
69136	'Tusc1'	90	78	138	27	145	103	102	91.66666667	-0.269174651	0.667475301	0.972790461
69137	'Vstm5'	74	74	131	5	45	35	93	28.33333333	-1.857343722	0.011698378	0.268550885
69146	'Gsdmd'	495	539	246	270	580	499	426.6666667	449.6666667	0.116965705	0.757299001	0.972790461

69147	'2200002J24Rik'	19	19	5	50	27	33	14.33333333	36.66666667	1.573216703	0.036041425	0.456204771
69149	'Kbtbd3'	218	223	58	52	200	103	166.3333333	118.3333333	-0.45394907	0.469325219	0.972790461
69150	'Snx4'	2104	2283	2455	1514	2055	2811	2280.666667	2126.666667	-0.097115961	0.760218871	0.972790461
69151	'Lzic'	632	576	549	394	541	617	585.6666667	517.3333333	-0.145646713	0.579798096	0.972790461
69155	'1810030007Rik'	915	976	708	450	1058	1031	866.3333333	846.3333333	-0.038657833	0.902363143	0.987331639
69156	'Comtd1'	72	77	114	89	128	165	87.66666667	127.3333333	0.518858009	0.245306783	0.866389105
69159	'Rhebl1'	89	77	54	59	54	107	73.33333333	73.33333333	0.057391938	0.897099263	0.985794067
69161	'Manbal'	243	246	328	258	390	396	272.3333333	348	0.35477631	0.337781098	0.924020038
69162	'Sec31a'	4785	4661	5483	3918	4379	5243	4976.333333	4513.333333	-0.101718761	0.760454627	0.972790461
69163	'Mrpl44'	599	606	626	182	485	755	610.3333333	474	-0.437274028	0.341331114	0.925308103
69168	'Bola1'	71	106	96	90	89	123	91	100.6666667	0.194481685	0.644807826	0.972790461
69169	'Fcmr'	1	3	3	0	1	0	2.333333333	0.333333333	-2.676501216	0.236515708	0.862272407
69171	'Cnppd1'	1069	1090	2443	384	975	1246	1534	868.3333333	-0.940742611	0.102779198	0.672865573
69178	'Snx5'	3081	3083	7174	1377	2966	3660	4446	2667.666667	-0.840435684	0.133497067	0.733301819
69179	'Stimate'	329	303	134	249	384	345	255.3333333	326	0.436150795	0.271591038	0.884539076
69181	'Dyrk2'	897	889	1617	1236	1019	1177	1134.333333	1144	0.064183118	0.900408531	0.986604124
69183	'C1qtnf2'	12	10	3	1	7	3	8.333333333	3.666666667	-1.163903143	0.298665764	0.902535634
69185	'Dtwd1'	236	237	226	219	245	245	233	236.3333333	0.088575815	0.789952027	0.972790461
69186	'Tmem256'	241	260	412	157	169	256	304.3333333	194	-0.654824933	0.158586149	0.768890221
69187	'Erp27'	0	1	0	0	0	0	0.333333333	0	-0.903279821	0.824807108	0.972790461
69188	'Kmt2e'	2989	2899.2	4087.5	2112.3	2999.7	2841.53	3325.216667	2651.176667	-0.316090242	0.396926685	0.954358954
69189	'Mcemp1'	1	6	2	2	7	0	3	3	0.070299154	0.966981519	0.999493374
69190	'Dym'	972	985	1176	500	755	991	1044.333333	748.6666667	-0.491983069	0.147670173	0.754705732
69191	'Pdia2'	23	15	33	1	5	8	23.66666667	4.666666667	-2.445167538	0.005817362	0.179836874
69192	'Dhx16'	1045	1012	896	910	960	1147	984.3333333	1005.666667	0.100213234	0.73602568	0.972790461
69195	'Tmem121'	49	62	36	68	44	48	49	53.33333333	0.285194673	0.602620783	0.972790461
69202	'Ptms'	1632	1672	1735	1709	2561	2283	1679.666667	2184.333333	0.409734203	0.160188478	0.771114735
69207	'Srsf11'	5976	6129	4519	4814	6448	3355	5541.333333	4872.333333	-0.07652787	0.855362636	0.975734242
692132	'Trappc3l'	0	0	1	0	4	11	0.333333333	5	3.644919591	0.134890541	0.735841445
69215	'Sat2'	374	507	56	130	430	364	312.3333333	308	0.026622916	0.970840326	0.999493374
69216	'Svbp'	438	448	408	280	398	492	431.3333333	390	-0.123447676	0.650132854	0.972790461
69217	'Plekha4'	180	177	129	55	168	129	162	117.3333333	-0.476167451	0.242913268	0.864948315
69219	'Ddah1'	491	446	832	71	472	544	589.6666667	362.3333333	-0.863137975	0.205044694	0.833294914
69225	'Naxd'	418	446	241	345	573	665	368.3333333	527.6666667	0.559077567	0.077809759	0.613825367
69226	'Snx24'	131	150	182	99.1	159	190	154.3333333	149.3666667	-0.062150253	0.872743158	0.980171118
69227	'Selenot'	2088	2295	1863	2396	2711	3044	2082	2717	0.451229485	0.105072398	0.677660775
69228	'Zfp746'	1381	1436	792	355	1185	1138	1203	892.6666667	-0.450162441	0.300536272	0.904081584
69232	'Qrich1'	3558	3624	3107	1372	2811	3004	3429.666667	2395.666667	-0.524842334	0.063458936	0.567415027
69234	'Zfp688'	107	116	154	95	147	128	125.6666667	123.3333333	-0.018875162	0.963535339	0.999493374
69237	'Gtpbp4'	1306	1331	2313	924	1335	1812	1650	1357	-0.322617902	0.478894844	0.972790461

69239	'Pdzph1'	2	0	1	0	2	0	1	0.666666667	-0.645083752	0.838793988	0.974723675
69241	'Polr2d'	545	571	1552	306	594	760	889.3333333	553.3333333	-0.793172795	0.194741824	0.819935527
69253	'Hspb2'	8	4	5	202	2	36	5.666666667	80	4.220677359	0.001448236	0.082436738
69256	'Zfp397'	2396.8	2411.7	902.55	865.92	1944.9	1675.55	1903.69	1495.44	-0.300425776	0.489651765	0.972790461
69257	'Elf2'	1441	1506	1720	819	1560	1740	1555.666667	1373	-0.204903354	0.540217764	0.972790461
69259	'Kctd5'	1078	1148	1525	773	1017	1159	1250.333333	983	-0.339580562	0.358712157	0.933467324
69260	'Ing2'	424	387	962	228	379	379	591	328.6666667	-0.904806431	0.101104686	0.671759705
69263	'Rfc3'	523	517	328	133	544	613	456	430	-0.136003826	0.785574711	0.972790461
69269	'Scnm1'	232	271	397	180	326	376	300	294	-0.073318187	0.866973955	0.978504531
69270	'Gins1'	238	187	105	35	160	152	176.6666667	115.6666667	-0.644333202	0.243691151	0.865506476
69274	'Ctdspl'	1083	1109	1286	779	1124	1311	1159.333333	1071.333333	-0.108182095	0.732983134	0.972790461
69276	'Sec62'	2314	2360	1987	2869	2905	3245	2220.333333	3006.333333	0.521061061	0.092754759	0.654099957
69277	'3300002I08Rik'	1092.9	1137.5	490.73	818.36	1321.6	1110.85	907.04	1083.596667	0.339239295	0.370162101	0.937712122
69281	'Spata4'	15	21	3	2	7	6	13	5	-1.329895229	0.186417732	0.807696881
69282	'1700001J03Rik'	10	11	15	2	14	5	12	7	-0.847223076	0.365024262	0.935765447
69287	'Odf3'	0	0	0	0	1	0	0	0.333333333	1.020273531	0.802557913	0.972790461
69288	'Rhobtb1'	815	807	871	154	771	683	831	536	-0.724029703	0.169213645	0.784876338
69294	'Cst13'	8	3	1	2	8	3	4	4.333333333	0.166214602	0.89644291	0.985710789
69297	'Lrrc46'	95	77	23	6	46	53	65	35	-0.929680049	0.238076417	0.864053647
69299	'Asb9'	0	0	0	1	0	2	0	1	2.507928928	0.530239069	0.972790461
69301	'Tesc1'	3	15	2	3	2	1	6.666666667	2	-1.518969447	0.270400499	0.883851074
69305	'Dcps'	435	496	829	222	580	526	586.6666667	442.6666667	-0.48517519	0.328133876	0.921353012
69306	'Efcab9'	8	10	3	8	3	3	7	4.666666667	-0.33971413	0.756811993	0.972790461
69307	'Pxt1'	1	0	0	0	1	9	0.333333333	3.333333333	3.0493125	0.270405844	0.883851074
69309	'Slc16a13'	163	148	33	26	194	114	114.6666667	111.3333333	-0.050992001	0.946466816	0.996592403
69310	'Pacrg'	133	116	62	9	56	66	103.6666667	43.66666667	-1.301945076	0.044761544	0.492557243
69312	'Ppp1r42'	7	4	1	5	6	2	4	4.333333333	0.288770016	0.815406772	0.972790461
69315	'1700001L19Rik'	7	11	5	2	6	3	7.666666667	3.666666667	-1.02776521	0.307417141	0.907830798
69318	'Fam24b'	0	0	1	0	0	1	0.333333333	0.333333333	0.058500858	0.988561293	0.999493374
69324	'1700012B07Rik'	0	1	0	0	0	0	0.333333333	0	-0.903279821	0.824807108	0.972790461
69325	'1700012B09Rik'	5	1	0	0	0	0	2	0	-3.284779471	0.389056114	0.951190011
69327	'1700007K13Rik'	14	16	5	3	20	17	11.66666667	13.33333333	0.171086137	0.844965798	0.975182082
69329	'Cfap206'	7	5	9	18	6	14	7	12.66666667	0.982256073	0.283755085	0.892170588
69339	'Ccdc54'	0	0	1	0	0	0	0.333333333	0	-0.903279821	0.824807108	0.972790461
69349	'1700008O03Rik'	8	11	6	7	9	8	8.333333333	8	0.015007843	0.984945208	0.999493374
69352	'Necab1'	44	40.54	8.13	1.01	46.97	20.34	30.89	22.77333333	-0.499789	0.646488383	0.972790461
69354	'Slc38a4'	735	759	472	2492	766	676	655.3333333	1311.333333	1.309593492	0.07233945	0.595548709
69358	'Lrrc51'	140	141	92	8	83	143	124.3333333	78	-0.783354957	0.315694432	0.914723408
69361	'Cypt3'	2	2	1	0	2	1	1.666666667	1	-0.776082708	0.688945319	0.972790461
69362	'Cst12'	783	747	115	47	352	281	548.3333333	226.6666667	-1.285355922	0.105274175	0.678099427

69363	'Spaca4'	0	0	0	3	0	1	0	1.333333333	3.183883522	0.419737082	0.958308933
69367	'Glrx2'	413	455.04	349	371.05	478	393	405.68	414.0166667	0.106114436	0.73590957	0.972790461
69368	'Wdfy1'	1438	1464	2473	679	590	459	1791.666667	576	-1.55488761	0.003539096	0.137037276
69369	'Ms4a20'	0	0	0	0	1	0	0	0.333333333	1.020273531	0.802557913	0.972790461
69371	'Smco2'	2	2	1	0	3	3	1.666666667	2	0.192074924	0.912394261	0.989773583
69372	'Mocs3'	111	128	86	115	155	216	108.3333333	162	0.616033965	0.062044792	0.564940415
69376	'Zpbp2'	21	18	3	3	7	10	14	6.666666667	-1.022327052	0.286541401	0.894199485
69379	'C8g'	66	73	17	6	57	85	52	49.33333333	-0.129894644	0.880542061	0.982214601
69380	'1700013G24Rik'	0	0	1	0	0	0	0.333333333	0	-0.903279821	0.824807108	0.972790461
69386	'H4c8'	4	2.05	11.22	3	1.03	4	5.756666667	2.676666667	-1.100513217	0.403916552	0.956543098
69387	'Dnajb13'	1	1	0	1	2	5	0.666666667	2.666666667	2.00875633	0.296097602	0.90029104
69389	'H2bw2'	44	36	1	0	28	17	27	15	-0.881163013	0.546003079	0.972790461
69397	'1700019A02Rik'	3	5	0	0	5	12	2.666666667	5.666666667	1.031560832	0.572286123	0.972790461
69398	'Cdhr4'	78	89	18	9	97	49	61.66666667	51.66666667	-0.273673942	0.745557799	0.972790461
69399	'1700025G04Rik'	818	713	1436	1126	678	921	989	908.3333333	-0.040012402	0.944409506	0.996592403
69408	'Dnajc17'	168	124	103	57	165	178	131.6666667	133.3333333	-0.007548361	0.986195429	0.999493374
69428	'1700016C15Rik'	0	0	1	13	0	4	0.333333333	5.666666667	4.322662978	0.070466947	0.589465353
69440	'Dennd6b'	204	223	82	90	350	207	169.6666667	215.6666667	0.359600603	0.508309053	0.972790461
69441	'Efcab15'	4	7	6	4	2	5	5.666666667	3.666666667	-0.559389417	0.600817746	0.972790461
69453	'Prss56'	2	2	22	3	1	3	8.666666667	2.333333333	-1.940720664	0.185545419	0.805706284
69454	'Clic3'	4	15	3	43	23	9	7.333333333	25	2.065856974	0.044928827	0.493464388
69456	'Commd10'	509	502	644	419	677	676	551.6666667	590.6666667	0.095766985	0.78243177	0.972790461
69457	'Tmem45a2'	3	6	2	0	3	2	3.666666667	1.666666667	-1.17522955	0.445603164	0.969915971
69459	'Ubl7'	792	760	687	637	706	920	746.3333333	754.3333333	0.068379497	0.812440178	0.972790461
69464	'Krtap4-13'	0	0	0	0	2	0	0	0.666666667	1.801491674	0.656112935	0.972790461
69469	'Tmco2'	0	0	1	0	0	1	0.333333333	0.333333333	0.058500858	0.988561293	0.999493374
69470	'Tmem127'	1645	1698	1069	1702	2115	2194	1470.666667	2003.666667	0.5259454	0.062745733	0.566066012
69478	'2300009A05Rik'	41	53	37	62	57	42	43.66666667	53.66666667	0.426717137	0.409828443	0.957157474
69479	'1700029J07Rik'	674	616	151	29	341	219	480.3333333	196.3333333	-1.325895865	0.095123583	0.661723219
69480	'Ttc9'	105	93	50	143	70	120	82.66666667	111	0.607482194	0.28235847	0.890463
69481	'Actl9'	0	1	0	0	1	2	0.333333333	1	1.346096926	0.690173371	0.972790461
69482	'Nup35'	362	418	376	163	324	378	385.3333333	288.3333333	-0.433986631	0.182979287	0.804092742
69487	'Ndufaf5'	165	193	125	56	120	169	161	115	-0.497463224	0.212682865	0.841028891
69496	'Dydc1'	0	0	0	0	0	1	0	0.333333333	1.020273531	0.802557913	0.972790461
69499	'Tsr2'	1151.8	1174.8	955.89	185.13	812.74	1070.53	1094.156667	689.4666667	-0.749058187	0.158599263	0.768890221
69504	'Zfp932'	703.56	743.87	197.96	324.71	1815.2	757.45	548.4633333	965.7966667	0.830044358	0.212273251	0.84083615
69519	'Rwdd2a'	45	36	27	214	36	42	36	97.33333333	1.791855469	0.048541374	0.511851691
69524	'Esam'	329	369	45	15	563	431	247.6666667	336.3333333	0.391798321	0.704756173	0.972790461
69527	'Mrps9'	950	989	765	360	765	792	901.3333333	639	-0.497689248	0.088260922	0.643164679
69528	'1700030J22Rik'	278	230	35	44	113	147	181	101.3333333	-0.796271353	0.27055549	0.883851074

69534	'Avpi1'	96	89	311	156	95	157	165.3333333	136	-0.26838945	0.712553517	0.972790461
69535	'Ten1'	162	194	179	152	220	211	178.3333333	194.3333333	0.157666886	0.6206173	0.972790461
69536	'Hemk1'	417	403	148	9	182	123	322.6666667	104.6666667	-1.691707779	0.042300939	0.479588013
69537	'Dnase1l1'	78	95	95	53	102	147	89.33333333	100.6666667	0.138429397	0.745234113	0.972790461
69538	'Antxr1'	1226	1192	406	209	1905	1831	941.3333333	1315	0.427492553	0.550710737	0.972790461
69539	'Trnp1'	50	34	77	90	45	92	53.66666667	75.66666667	0.564431347	0.385176345	0.946894294
69540	'Klk10'	0	0	4	0	0	1	1.333333333	0.333333333	-2.046205198	0.605741042	0.972790461
69541	'Lyg1'	0	0	0	0	1	1	0	0.666666667	1.775692139	0.660844521	0.972790461
69542	'2300002M23Rik'	0	1	1	0	1	0	0.666666667	0.333333333	-0.886945817	0.805479824	0.972790461
69543	'Capns2'	5	5	1	8	1	0	3.666666667	3	0.16164676	0.926599775	0.992535631
69544	'Wdr5b'	81	103	19	13	125	98	67.66666667	78.66666667	0.18940877	0.818047423	0.972790461
69546	'Mapk1ip1'	310	306	392	173	363	202	336	246	-0.446591904	0.30086292	0.904264146
69547	'Nkpd1'	4	10	18	6	12	7	10.66666667	8.333333333	-0.393768947	0.675699544	0.972790461
69549	'2310009B15Rik'	74	106	102	198	81	102	94	127	0.625674908	0.329250026	0.921648675
69550	'Bst2'	618	612	1542	605	727	1653	924	995	0.012386005	0.983978643	0.999493374
69551	'2310022B05Rik'	1286	1228	2933	1814	1102	1212	1815.666667	1376	-0.315881436	0.620026725	0.972790461
69553	'Ripor3'	113	109	19	137	143	100	80.33333333	126.6666667	0.848066815	0.21432602	0.84261066
69554	'Klhdc2'	1555	1616	2024	1185	1669	1975	1731.666667	1609.666667	-0.104604727	0.760574349	0.972790461
69556	'Bod1'	843	803	950	766	891	1140	865.3333333	932.3333333	0.13552628	0.683308307	0.972790461
69562	'Cdk13'	3538	3493	2545	2612	2935	2662	3192	2736.333333	-0.124792139	0.692839867	0.972790461
69563	'Mrln'	7	3	0	0	8	3	3.333333333	3.666666667	0.117574592	0.950677779	0.998065566
69564	'Nmrk2'	0	1	0	0	0	0	0.333333333	0	-0.903279821	0.824807108	0.972790461
69568	'Vkorc1l1'	1611.3	1574.7	1386	670.04	1379.3	1407.66	1524.003333	1152.316667	-0.407595533	0.148373734	0.756674202
69572	'Mfsd3'	89	93.78	19.26	38	110.54	153.63	67.34666667	100.7233333	0.596762798	0.374177487	0.940062838
69573	'Hilpda'	237	191	2923	301	155	474	1117	310	-1.959226326	0.066339461	0.575690169
69574	'Cmbl'	133	168	91	633	146	307	130.6666667	362	1.745298842	0.015028848	0.305758542
69576	'Smco1'	3	1	1	0	3	1	1.666666667	1.333333333	-0.3730735	0.847533968	0.975439569
69577	'Fastkd3'	307	287	292	174	224	261	295.3333333	219.6666667	-0.392886341	0.189431729	0.812029435
69581	'Rhou'	1536	1451	1612	1556	1693	1500	1533	1583	0.120901336	0.738106603	0.972790461
69582	'Plekhm2'	588.93	562	553	605.99	776	670.95	567.9766667	684.3133333	0.329273867	0.292057668	0.89958198
69583	'Tnfsf13'	69.08	72.92	60.47	20.16	83.9	111.6	67.49	71.88666667	0.016199478	0.977236811	0.999493374
69585	'Hjv'	1	2	1	1	1	0	1.333333333	0.666666667	-0.840561797	0.695283857	0.972790461
69587	'Pcgf3'	2330	2106	2470	1453	1935	1929	2302	1772.333333	-0.344142988	0.268435047	0.883600995
69590	'Gpx8'	610	731	474	265	837	763	605	621.6666667	0.013607752	0.97300206	0.999493374
69596	'Ap5s1'	256	278	140	189	273	258	224.6666667	240	0.172068338	0.617253518	0.972790461
69597	'Afg3l2'	2769	2704	2355	1798	2615	2876	2609.333333	2429.666667	-0.070019124	0.761264026	0.972790461
69601	'Dab2ip'	2066	2011	986	1282	2125	2066	1687.666667	1824.333333	0.175145952	0.606223424	0.972790461
69602	'Otop3'	0	0	0	0	0	2	0	0.666666667	1.749449488	0.66566965	0.972790461
69605	'Lnpk'	738	724	269	412	694	462	577	522.6666667	-0.041383666	0.927650205	0.992989454
69606	'Mtfmt'	340	386	199	193	300	282	308.3333333	258.3333333	-0.189474104	0.563813249	0.972790461

69608	'Sec24d'	901	848	4948	1377	747	1486	2232.333333	1203.333333	-0.915918461	0.280273342	0.889355605
69612	'Kansl2'	1289	1380	1766	7587	1556	2315	1478.333333	3819.333333	1.6384129	0.036616217	0.457518227
69617	'Pitrm1'	1386.9	1324	1844	1001	1647	1589	1518.296667	1412.333333	-0.112226786	0.757783934	0.972790461
69627	'Fam89a'	0.36	5.8	11.97	0	19.82	10.91	6.043333333	10.24333333	0.611121247	0.731308328	0.972790461
69632	'Arhgef12'	4261.9	4151.5	3226	7195	4175	4796.76	3879.806667	5388.916667	0.650391505	0.180977906	0.801479605
69634	'Clybl'	212	216	312	351	350	562	246.6666667	421	0.781487195	0.071260263	0.591632421
69635	'Dapk1'	3196	3146	1420	6129	4085	3312	2587.333333	4508.666667	1.019216326	0.061600399	0.563593259
69638	'Enho'	25	27	31	0	54	57	27.66666667	37	0.247721586	0.834103254	0.974723675
69639	'Exosc8'	513	551	446	161	495	621	503.3333333	425.6666667	-0.29597025	0.496189334	0.972790461
69640	'Fam83g'	584.91	629	264	300	498	818.97	492.6366667	538.99	0.156316853	0.719021093	0.972790461
69641	'Wdr20'	559.62	606.46	517.06	741.19	633	563.6	561.0466667	645.93	0.329759283	0.426338666	0.960958977
69642	'Mlip'	6	1	1	1	6	2	2.666666667	3	0.185443781	0.902581941	0.987331639
69654	'Dctn2'	1627	1802	1634	1900	2070	2166	1687.666667	2045.333333	0.347724303	0.264979896	0.88128484
69655	'Cd164l2'	29	32	9	39	48	258	23.33333333	115	2.273236286	0.005073253	0.16781708
69656	'Pir'	67	56	45	35	64	178	56	92.33333333	0.667923054	0.261610281	0.87954257
69660	'Tmbim1'	366	406	773	1594	555	973	515	1040.666667	1.154568309	0.093599467	0.657687298
69662	'2310061I04Rik'	785	717	556	93	441	573	686	369	-0.973685049	0.077130458	0.611256471
69663	'Ddx51'	608	672	377	280	649	625	552.3333333	518	-0.079323636	0.818164468	0.972790461
69664	'Krtap1-5'	0	0	0	0	0	1	0	0.333333333	1.020273531	0.802557913	0.972790461
69665	'Upk3bl'	1	1	3	0	0	3	1.666666667	1	-0.945597867	0.686135269	0.972790461
69666	'Psmg4'	197	198	371	28	99	166	255.3333333	97.66666667	-1.529486843	0.016984552	0.326501819
69668	'Ccdc115'	622	626	1378	179	507	830	875.3333333	505.3333333	-0.936047208	0.131837316	0.72897159
69671	'Tmem52'	2	4	6	11	4	4	4	6.333333333	0.831642267	0.476353587	0.972790461
69672	'Txndc15'	443	473	235	210	814	790	383.6666667	604.6666667	0.6291673	0.198983856	0.825995916
69674	'Mif4gd'	292	285	202	285	334	430	259.6666667	349.6666667	0.491044775	0.095104146	0.661723219
69675	'Pxdn'	4022	3724	1312	3496	5153	3758	3019.333333	4135.666667	0.571708149	0.219630342	0.847495851
69683	'Emc10'	729	750	597	371	1258	1314	692	981	0.453605293	0.288049408	0.895128365
69684	'Aarsd1'	479	491	225	450	483	593	398.3333333	508.6666667	0.45682574	0.233695093	0.860393388
69692	'Hddc2'	316	336.08	229.01	147	320	236	293.6966667	234.3333333	-0.298960233	0.363690955	0.935022643
69693	'Pof1b'	4	5	4	9	5	2	4.333333333	5.333333333	0.505538228	0.657936526	0.972790461
69694	'Tatdn1'	394.06	366.2	461.46	295.96	324.77	387.59	407.24	336.1066667	-0.241524769	0.506219979	0.972790461
69697	'Camsap3'	788	710	765	596	993	1207	754.3333333	932	0.30013886	0.314764841	0.914723408
69698	'Slc52a3'	12	15	5	23	33	21	10.66666667	25.66666667	1.375097988	0.04134611	0.476407426
69700	'Col22a1'	253	291	121	75	75	46	221.6666667	65.33333333	-1.596084641	0.002377267	0.109249332
69702	'Ndufaf1'	554	664	325	143	477	488	514.3333333	369.3333333	-0.496642745	0.277289981	0.88901132
69706	'Lrr1'	253	251	77	3	127	161	193.6666667	97	-1.084437866	0.273425654	0.884738928
69707	'Iqcg'	197.13	227.22	36.04	21.52	139.81	108.59	153.4633333	89.97333333	-0.783458499	0.313062783	0.912316006
69709	'Ptrhd1'	113.49	109.6	155.71	44	116.47	83.63	126.2666667	81.36666667	-0.674274422	0.151015924	0.759755159
69710	'Arap1'	1355	1489	925	551	1772	1229	1256.333333	1184	-0.092777061	0.81776005	0.972790461
69713	'Pin4'	233	257	202	161	171	281	230.6666667	204.3333333	-0.132614893	0.696649894	0.972790461

69714	'Tfpt'	227	242	404	96	264	282	291	214	-0.533427285	0.29634591	0.90029104
69716	'Trip13'	358	347	137	65	290	347	280.6666667	234	-0.295014287	0.616912563	0.972790461
69717	'Gm10499'	41.58	32.22	82.22	3.23	5.86	1	52.00666667	3.363333333	-4.10726005	5.61E-06	0.001569535
69718	'Ipmk'	813	782	454	422	617	789	683	609.3333333	-0.117507285	0.70511946	0.972790461
69719	'Cad'	1542	1495	839	269	1572	1834	1292	1225	-0.144937565	0.807473699	0.972790461
69721	'Nkiras1'	280.01	291.79	172.17	264.94	261.57	355.12	247.99	293.8766667	0.334255282	0.339116662	0.9248827
69723	'Rpain'	209	194	307	286	207	258	236.6666667	250.3333333	0.159488355	0.751923894	0.972790461
69724	'Rnaseh2a'	321	275	312	637	371	486	302.6666667	498	0.85694504	0.08279609	0.624650504
69726	'Smyd3'	384	359	482.97	542	370	508	408.6566667	473.3333333	0.304765183	0.517357515	0.972790461
69727	'Usp46'	873	972	1097	597	1113	1141	980.6666667	950.3333333	-0.06227255	0.852197936	0.975734242
69731	'Gemin7'	387	452	495	371	462	592	444.6666667	475	0.112648242	0.742243168	0.972790461
69736	'Nup37'	349	343	234	81	301	382	308.6666667	254.6666667	-0.331762162	0.502817633	0.972790461
69737	'Ttl'	958	954	887	354	901	991	933	748.6666667	-0.354285525	0.324346006	0.919683661
69740	'Dph5'	189	197	183	39	170	182	189.6666667	130.3333333	-0.618491098	0.231803188	0.858905111
69742	'Tm2d2'	562	571	592	799	689	830	575	772.6666667	0.511659648	0.176590167	0.796450095
69743	'Casz1'	218	195	159	254	195	174	190.6666667	207.6666667	0.27296939	0.554078508	0.972790461
69745	'Pold4'	186	181	475	686	170	315	280.6666667	390.3333333	0.641082624	0.428797976	0.962386546
69747	'Zswim7'	249	229	190	27	197	328	222.6666667	184	-0.390078557	0.576685011	0.972790461
69748	'Aldh16a1'	518	534	362	436	522	597	471.3333333	518.3333333	0.207962811	0.45905352	0.972790461
69752	'Zfp511'	190.31	183	331.95	249	196.44	195	235.0866667	213.48	-0.06399044	0.90649026	0.988823459
69754	'Fbxo7'	980.14	991.19	549.03	250.55	669.97	786.92	840.12	569.1466667	-0.574961318	0.159389821	0.770681403
69757	'Leng1'	276.14	275.93	480.88	282.35	297.1	331.86	344.3166667	303.77	-0.15898163	0.738345043	0.972790461
69769	'Tnfaip8l2'	4	4	4	28	12	18	4	19.33333333	2.440966305	0.006152109	0.185170597
69770	'Fam174c'	58	54	60	34	42	27	57.33333333	34.33333333	-0.668391235	0.176742679	0.796450095
69772	'Bdh2'	68	84	119	60	114	78	90.33333333	84	-0.11552449	0.813744817	0.972790461
69773	'Timm29'	1606	1534	1187	1176	1284	1570	1442.333333	1343.333333	-0.030262312	0.914611991	0.989964366
69774	'Ms4a6b'	1	1	0	2	12	8	0.666666667	7.333333333	3.444363832	0.031395933	0.430284336
69780	'Smap2'	993	965	1138	1013	870	969	1032	950.6666667	-0.033478545	0.933986434	0.994249017
69784	'1500009L16Rik'	50	42	91	173	93	79	61	115	1.053421723	0.131823277	0.72897159
69786	'Tprkb'	696	707	845	159	541	645	749.3333333	448.3333333	-0.828625963	0.085415475	0.634617076
69787	'Anxa13'	2	1	2	0	0	0	1.666666667	0	-3.149826602	0.284337584	0.893053879
69790	'Med30'	282	269	322	198	255	361	291	271.3333333	-0.096058861	0.785276236	0.972790461
69792	'Med6'	620	661	547	196	573	637	609.3333333	468.6666667	-0.422308642	0.288749667	0.8959864
69802	'Cox11'	301.38	366.72	356.94	322.92	299.82	344.38	341.68	322.3733333	-0.007940944	0.983352458	0.999493374
69804	'Tmem147'	824	819	784	503	868	1131	809	834	0.034661153	0.90842398	0.988823459
69806	'Slc39a11'	228	253	99	175	419	521	193.3333333	371.6666667	0.954625345	0.038416257	0.459930654
69807	'Trim32'	828	866	1418	1282	973	1222	1037.333333	1159	0.223561983	0.65655556	0.972790461
69809	'1810046K07Rik'	0	0	1	0	0	0	0.333333333	0	-0.903279821	0.824807108	0.972790461
69814	'Prss32'	1	1	2	1	0	0	1.333333333	0.333333333	-1.888603506	0.453455542	0.970649024
69815	'Krtcap3'	28	23	16	34	31	83	22.33333333	49.33333333	1.173245528	0.043449705	0.485921052

69816	'Mzb1'	1	1	0	0	1	2	0.666666667	1	0.547742134	0.835241607	0.974723675
69821	'Mterf4'	326	337	297	192	335	365	320	297.3333333	-0.096535671	0.727765261	0.972790461
69823	'Fyttd1'	3071.5	3101.3	4063.9	2457.9	2894.8	3193.88	3412.203333	2848.863333	-0.231713181	0.525993678	0.972790461
69824	'Glod5'	3	3	7	3	0	6	4.333333333	3	-0.536523851	0.718266397	0.972790461
69826	'Ms4a10'	0	0	0	0	0	1	0	0.333333333	1.020273531	0.802557913	0.972790461
69833	'Polr2f'	530	543	522	380	438	594	531.6666667	470.6666667	-0.138716942	0.645940609	0.972790461
69834	'Rab43'	861	915	1280	1501	945	1067	1018.666667	1171	0.31707549	0.545468152	0.972790461
69836	'Pla2g12b'	0	1	0	0	1	0	0.333333333	0.333333333	0.058500858	0.988561293	0.999493374
69837	'Pcgf1'	181	159	164	103	206	178	168	162.3333333	-0.044537745	0.893037358	0.985171
69847	'Wnk4'	537	522	225	1228	705	428	428	787	1.143128452	0.077295926	0.611392561
69852	'Tcf23'	104	89	12	26	114	64	68.33333333	68	0.034690697	0.96535328	0.999493374
69860	'Eif1ad'	877	887	737	385	690	943	833.6666667	672.6666667	-0.317009703	0.304956499	0.907188578
69861	'2010003K11Rik'	7	3	0	0	0	0	3.333333333	0	-4.018705131	0.213044137	0.841028891
69863	'Ttc39b'	430	424	239	369	497	615	364.3333333	493.6666667	0.499323146	0.112534691	0.698045528
69864	'1810065E05Rik'	0	2	0	5	1	0	0.666666667	2	2.048519886	0.466102452	0.972790461
69865	'A1cf'	29	40	1	0	15	15	23.33333333	10	-1.257489664	0.378382763	0.9419364
69870	'Polr3gl'	716	710	226	456	921	537	550.6666667	638	0.305062452	0.546087346	0.972790461
69871	'Ppp1r35'	177	186	171	335	220	332	178	295.6666667	0.844509025	0.056067812	0.544691483
69875	'Ndufa11'	399	401	314	387	459	661	371.3333333	502.3333333	0.479091827	0.099344084	0.668813644
69876	'Thap3'	138	140	176	99	165	168	151.3333333	144	-0.077041523	0.836787449	0.974723675
69878	'Snrpf'	777	804	1035	354	589	773.99	872	572.33	-0.638609833	0.088433521	0.643723745
69882	'Ints14'	950	1023	634	268	997	940	869	735	-0.277467902	0.536537397	0.972790461
69885	'Aunip'	123	129	56	15	85	207	102.6666667	102.3333333	-0.088093899	0.909066846	0.989136318
69890	'Zfp219'	870.97	900.93	921	1462.7	1343.5	1062	897.6333333	1289.393333	0.635878122	0.136043871	0.736689633
69893	'Coa7'	355	369	360	174	272	358	361.3333333	268	-0.431133326	0.164407947	0.777557251
69894	'Fam241b'	41	57	107	25	32	76	68.33333333	44.33333333	-0.71096059	0.285017003	0.893711786
69900	'Mfsd11'	638.09	606.02	548.27	874.13	621.04	591.02	597.46	695.3966667	0.369038546	0.421990872	0.959445304
69902	'Mrto4'	563.97	566.96	875.99	222	598	811.99	668.9733333	543.9966667	-0.394075204	0.431430868	0.963623551
69903	'Rasip1'	372	382	64	23	637	245	272.6666667	301.6666667	0.109243288	0.91013126	0.989501659
69906	'Slc25a32'	821	913	387	397	771	725	707	631	-0.113597296	0.764682865	0.972790461
69908	'Rab3b'	35	27	41	128	25	31	34.33333333	61.33333333	1.120641313	0.208738714	0.836616817
69912	'Nup43'	427	432	396	176	313	479	418.3333333	322.6666667	-0.39636763	0.264537027	0.88128484
69917	'Nabp2'	289	304	538	349	352	390	377	363.6666667	-0.02709002	0.956107214	0.999493374
69920	'Polr2i'	183	222	206	179	163	257	203.6666667	199.6666667	0.023949995	0.949877761	0.998065566
69922	'Vrk2'	207.87	210.78	144.17	31.15	242.78	206.35	187.6066667	160.0933333	-0.308173097	0.624960466	0.972790461
69923	'Agk'	489.12	545.01	326.32	249.12	588.44	536.85	453.4833333	458.1366667	0.024521487	0.94210405	0.996253096
69926	'Dnah17'	52	57	33	92	61	109	47.33333333	87.33333333	1.001327047	0.042479813	0.479588013
69928	'Cenps'	146	181	58	10	107	192	128.3333333	103	-0.397770028	0.634701742	0.972790461
69930	'Zfp715'	1100	1145	869	887	975	1004	1038	955.3333333	-0.034829951	0.907131236	0.988823459
69934	'Trmt10b'	178	201	196	143	238	287	191.6666667	222.6666667	0.213823046	0.509950002	0.972790461

69938	'Scrn1'	75	86	46	39.61	58	56	69	51.20333333	-0.37250255	0.353196199	0.930050349
69940	'Exoc1'	1073	1064	611	524	1183	1112	916	939.6666667	0.055639809	0.869094759	0.979633196
69942	'Rnf113a1'	75	58	136	34	58	64	89.66666667	52	-0.838597571	0.137942879	0.739360306
69944	'2810021J22Rik'	355	395	302	99	346	342	350.6666667	262.3333333	-0.465280169	0.286499531	0.894199485
69955	'Fars2'	583	622	498	217	495	641	567.6666667	451	-0.359592738	0.317508154	0.916136994
69956	'Ptcd3'	1493	1566	954	717	1394	1418	1337.666667	1176.333333	-0.160345913	0.582470532	0.972790461
69957	'Cdc16'	2388	2458	2476	1662	2445	2830	2440.666667	2312.333333	-0.063434665	0.815420168	0.972790461
69961	'Rpp25l'	199	199	301	67	260	295	233	207.3333333	-0.279905283	0.613136924	0.972790461
69962	'Mettl18'	109	110	111	25	118	143	110	95.33333333	-0.298735455	0.600590144	0.972790461
69976	'Galk2'	473.11	491	664	520	584	664	542.7033333	589.3333333	0.148542505	0.704122304	0.972790461
69981	'Tmem30a'	2355	2292	2187	4353	2534	3483	2278	3456.666667	0.744766818	0.111512902	0.695565384
69982	'Spink2'	1	2	2	1	1	0	1.666666667	0.666666667	-1.207086734	0.554675331	0.972790461
69983	'Sis'	64	50	9	0	0	0	41	0	-7.661393552	8.45E-07	3.19E-04
69987	'Spaca9'	12	20	27	1	27	17	19.66666667	15	-0.546423122	0.583544674	0.972790461
69993	'Chn2'	248	274	42	29	396	251	188	225.3333333	0.232295848	0.790499688	0.972790461
69994	'Rsc1a1'	172.87	136.43	171.22	127.83	138.04	76.45	160.1733333	114.1066667	-0.387089482	0.443765472	0.969836424
70008	'Ace2'	86	93	13	4	107	59	64	56.66666667	-0.212431086	0.833635023	0.974723675
70012	'Cep85'	1132	1060	425	491	665	788	872.3333333	648	-0.344317085	0.400547479	0.955860942
70020	'Ino80b'	309	295	182	206	317	389	262	304	0.254867246	0.408593899	0.957157474
70021	'Nt5dc2'	890	838	1768	1649	941	854	1165.333333	1148	0.1003232	0.876166101	0.981341203
70024	'Mcm10'	280	301	88	83	203	286	223	190.6666667	-0.209954884	0.703768307	0.972790461
70025	'Acot7'	727	813	1451	459	913	1353	997	908.3333333	-0.224883523	0.662870894	0.972790461
70026	'Tspo2'	2	5	2	1	5	4	3	3.333333333	0.136221242	0.916014035	0.990491125
70028	'Dop1b'	1262	1188	501	707	1134	1185	983.6666667	1008.666667	0.106342181	0.785053903	0.972790461
70031	'Cmtm8'	29	29	171	56	42	43	76.33333333	47	-0.71060953	0.430074767	0.962548446
70044	'Tut1'	669	705	919	404	637	758	764.3333333	599.6666667	-0.367557976	0.315804589	0.914723408
70045	'2610528A11Rik'	36	21	603	0	6	0	220	2	-7.048932354	1.13E-05	0.002873412
70047	'Trnt1'	1194.1	1204.2	1235.4	747.08	1060.8	1086.37	1211.253333	964.7633333	-0.300015613	0.281684112	0.889731741
70052	'Prpf4'	898	915	724	1215	858	922	845.6666667	998.3333333	0.386988295	0.374905563	0.940406075
70054	'Ccdc89'	48	41	18	6	22	19	35.66666667	15.66666667	-1.175964171	0.069759928	0.588104126
70059	'Degs2'	175.03	188.86	42.99	92.45	194.53	91.79	135.6266667	126.2566667	0.006675594	0.991466938	0.999493374
70060	'Spata3'	0	1	0	3	0	0	0.333333333	1	1.937497013	0.625951132	0.972790461
70061	'Sdr9c7'	0	1	0	1	2	1	0.333333333	1.333333333	1.935936502	0.448644764	0.97049895
70062	'Ctag2'	0	0	0	0	0	2	0	0.666666667	1.749449488	0.66566965	0.972790461
70069	'H1f7'	1	0	0	0	0	0	0.333333333	0	-0.903279821	0.824807108	0.972790461
70078	'Nol7'	658	629	797	299	746	1063	694.6666667	702.6666667	-0.05867869	0.895829912	0.985506207
70080	'Igsf23'	13	17	2	3	20	14	10.66666667	12.33333333	0.217777186	0.82945014	0.973678583
70081	'Zfp995'	164.01	207.96	477.97	61	213	150.33	283.3133333	141.4433333	-1.120999802	0.082720591	0.624328654
70082	'Lysmd2'	219	230	153	14	152	137	200.6666667	101	-1.083435872	0.12160379	0.712603747
70083	'Metrn'	188	238	69	75	210	281	165	188.6666667	0.198270413	0.723323886	0.972790461

70086	'Cysltr2'	1	0	0	0	1	0	0.333333333	0.333333333	0.058500858	0.988561293	0.999493374
70088	'Meaf6'	536	549	356	49	499	389	480.3333333	312.3333333	-0.707836618	0.293628834	0.90029104
70093	'Ube2q1'	1763	1806	1472	1127	1631	2004	1680.333333	1587.333333	-0.054530121	0.820839631	0.972790461
70097	'Sash1'	843	752	514	1028	867	861	703	918.6666667	0.526492752	0.193350494	0.816702866
70099	'Smc4'	5158	5069	1875	860	3238	3821	4034	2639.666667	-0.630739147	0.250979376	0.871167957
70101	'Cyp4f16'	154.67	173	83	169	300	202	136.89	223.6666667	0.786084005	0.048341638	0.510595119
70103	'Znhit1'	415.54	377.92	587.95	167.69	381.43	354.99	460.47	301.37	-0.662968945	0.122897952	0.714169238
70110	'Ifi35'	110	135	146	159	187	306	130.3333333	217.3333333	0.741329049	0.062716288	0.566066012
70113	'Odf3b'	3	10	3	0	7	7	5.333333333	4.666666667	-0.269663821	0.844500807	0.975182082
70118	'Srrd'	126.02	140.33	103.93	68.51	128.17	193.11	123.4266667	129.93	0.064531977	0.867780992	0.979066656
70120	'Yars2'	361	325	297	98	347	355	327.6666667	266.6666667	-0.352670292	0.430084891	0.962548446
70122	'Mllt3'	971	969	1264	504	968	1120	1068	864	-0.342979871	0.357393187	0.932505998
70123	'Nbdy'	305	329	367	92	282	307	333.6666667	227	-0.623765897	0.161572873	0.771541907
70127	'Dpf3'	235	226	206	185	264	171	222.3333333	206.6666667	-0.034659474	0.925966284	0.992381554
70129	'Slc44a4'	1	1	0	8	0	1	0.666666667	3	2.636836454	0.257126508	0.8773831
70134	'Misp3'	0	3	9	12	2	6	4	6.666666667	0.851699548	0.580187419	0.972790461
70144	'Lrch3'	2474.9	2303.8	1661	1346.5	2376.2	1332.41	2146.536667	1685.026667	-0.279268663	0.447649479	0.970198456
70152	'Mettl7a1'	210	229	62	147	229	225	167	200.3333333	0.35673147	0.481590807	0.972790461
70153	'2210016F16Rik'	584	673	378	230	761	1092	545	694.3333333	0.302702091	0.540628224	0.972790461
70155	'Ogfrl1'	289	299	226	100	284	304	271.3333333	229.3333333	-0.27212641	0.487974724	0.972790461
70160	'Vps36'	880	1024	720	357	1166	977	874.6666667	833.3333333	-0.098628529	0.805299762	0.972790461
70166	'Lipn'	0	3	0	0	1	2	1	1	-0.029125161	0.992453898	0.999493374
70178	'Abhd17c'	751	759	1112	821	709	783	874	771	-0.115053577	0.800878813	0.972790461
70186	'Fam162a'	1469	1135	9610	1110	616	1446	4071.333333	1057.333333	-2.007915297	0.028100285	0.409247928
70207	'Taco1'	411	399	202	201	360	374	337.3333333	311.6666667	-0.067974927	0.846457916	0.975201539
70208	'Med23'	2162	2186	868	681	1967	1607	1738.666667	1418.333333	-0.272077575	0.551040299	0.972790461
70209	'Tmem143'	271	303	357	83	287	262	310.3333333	210.6666667	-0.63267166	0.178665395	0.798304697
70218	'Kif18b'	440	428	104	70	257	236	324	187.6666667	-0.772085373	0.211525189	0.840335617
70223	'Nars'	2692	2857	11211	3304	2785	4593	5586.666667	3560.666667	-0.700067847	0.334237122	0.921648675
70225	'Ppil3'	400.68	433.55	324.68	57.41	366.5	415.32	386.3033333	279.7433333	-0.557227829	0.356207221	0.931977458
70227	'Zfp619'	95	103	57	72	240	158	85	156.6666667	0.882999204	0.056975781	0.547413448
70231	'Gorasp2'	1291	1274	2671	1352	1407	2077	1745.333333	1612	-0.132898138	0.798082716	0.972790461
70233	'Cd2bp2'	1629	1647	1483	1060	1603	1719	1586.333333	1460.666667	-0.092787143	0.6988594	0.972790461
70235	'Poc1a'	165	196	55	37	124	231	138.6666667	130.6666667	-0.111763721	0.865939643	0.978294615
70237	'Bhlhb9'	2427	2465	878	466	1812	1976	1923.333333	1418	-0.455386901	0.408776579	0.957157474
[truncated: 347,684 more chars]
