# Supplementary material for: EZH1/2 plays critical roles in oocyte meiosis prophase I in mice
Source: Biol Res. 2024 Nov 8;57:83. doi: 10.1186/s40659-024-00564-4 (PMC11545252; doi:10.1186/s40659-024-00564-4)
Supplement: Supplementary file 3 — Supplementary Material 3 [file 40659_2024_564_MOESM3_ESM.pdf]

Gene ID	Gene Symbol	WT1	WT2	WT3	dKO1	dKO2	dKO3	WT Average	dKO Average	log2 (dKO/ WT)	Pvalue(dKO/WT	Qvalue(dKO/WT)
100039532	'Gm10029'	669.62	519.21	974.56	0	0	0	721.13	0	-11.92641496	7.70E-20	2.93E-16
100040500	'Gm2808'	173.02	226.83	30.56	10.38	21.35	19.48	143.47	17.07	-3.011873717	3.84E-05	0.007055669
100041379	'Zfp980'	86.77	98.01	38.39	1.19	19.43	6.88	74.39	9.166666667	-3.116222355	2.09E-04	0.023202252
100041874	'Gm3558'	71.6	64.74	40.3	2.13	15.14	1.37	58.88	6.213333333	-3.272399366	3.61E-04	0.034287299
100042074	'Gm3650'	83.65	88.6	71.56	4.26	1.89	3.83	81.27	3.326666667	-4.834371984	2.33E-11	2.95E-08
100042555	'Gm13305'	59.41	54.24	21.49	0	0.78	0	45.04666667	0.26	-7.814564568	2.90E-08	1.78E-05
100042807	'Eif3j2'	1582.02	1529.48	1709.68	51.33	19.46	30.19	1607.06	33.66	-5.422327747	2.89E-20	1.38E-16
100043002	'Llph-ps2'	269.08	331.56	679.8	69.6	80.67	158.01	426.8133333	102.76	-2.125136572	3.00E-04	0.030177047
100043034	'Rex2'	161.55	135.04	60.93	4.06	18.23	4.68	119.1733333	8.99	-3.728888669	2.37E-07	1.25E-04
100048658	'Ddx43'	218	209	126	35	74	27	184.3333333	45.33333333	-1.947780968	1.61E-04	0.019471673
100169868	'Gm3173'	138.29	188.99	176.84	0	7.39	2.7	168.04	3.363333333	-5.842829962	2.44E-14	4.82E-11
100861615	'Gm3411'	232.95	233.43	143.29	0	23.04	7.82	203.2233333	10.28666667	-4.41526861	7.44E-07	2.95E-04
100862349	'Gm21663'	2	8.67	13.78	796.2	16.62	229.1	8.15	347.3066667	5.78087972	3.18E-06	9.75E-04
101055909	'Gm10320'	30.73	43.05	305.53	0	0	0	126.4366667	0	-9.524053958	1.03E-08	7.83E-06
101056073	'Zfp990'	606.98	662.16	351.33	1.96	33.88	29.01	540.1566667	21.61666667	-4.76816373	7.25E-10	7.26E-07
101434	'Ceacam15'	74	37	59	0	4	0	56.66666667	1.333333333	-5.438280198	5.75E-07	2.38E-04
102633424	'Gm31255'	29.04	26.73	25.53	156.51	317.77	171.22	27.1	215.1666667	3.045548973	1.45E-11	1.97E-08
102639702	'Gm35953'	55	44	55	0	7	1	51.33333333	2.666666667	-4.325827273	6.28E-06	0.001706877
104069	'Sncb'	23	13	347	0	5	3	127.6666667	2.666666667	-5.868417501	3.21E-05	0.006112081
104384	'Rhox9'	39	37	58.89	4	12	13	44.96333333	9.666666667	-2.268339086	4.71E-04	0.042077778
105244006	'Gm39701'	182.64	167.12	224.13	366.16	610.83	574.55	191.2966667	517.18	1.433987656	6.36E-05	0.009744897
105246572	'Gm41844'	91.68	74.69	42.07	3.33	4.09	4.3	69.48	3.906666667	-4.164188781	6.43E-10	6.85E-07
105247050	'Gm42226'	67.89	130.23	82.77	827.65	595	459.39	93.63	627.3466667	2.924771371	1.04E-07	5.99E-05
105247207	'Gm42346'	74.34	70.08	44.76	1	20.13	9.82	63.06	10.31666667	-2.699529636	6.04E-04	0.048169622
107769	'Tm6sf1'	90.84	93.04	67.12	2199.09	135.04	750.98	83.66666667	1028.37	3.964679779	2.65E-05	0.005255191
108167871	'Gm11703'	674.91	838.2	1750.17	4.5	3.01	3.62	1087.76	3.71	-8.349921202	6.07E-28	5.77E-24
108168770	'Gm13653'	55.23	52.4	147.61	0	0	0	85.08	0	-8.87713825	8.98E-10	8.13E-07
108168809	'Gm14279'	382.88	274.68	611.15	1	0	0	422.9033333	0.333333333	-10.20418765	1.52E-14	3.62E-11
108169053	'Gm15682'	261.27	444.96	514.1	0	1.62	0	406.7766667	0.54	-10.12622939	8.53E-15	2.32E-11
109801	'Glo1'	3875	4123	3461	1049	2006	2272	3819.666667	1775.666667	-1.108466685	5.63E-05	0.008853638
109820	'Pgc'	2	4	0	182	5	33	2	73.33333333	5.666602615	8.96E-05	0.012810696
110173	'Manba'	411	439	268	1154	950	1303	372.6666667	1135.666667	1.723372111	4.63E-06	0.001354787
11307	'Abcg1'	164	185	76	1079	401	475	141.6666667	651.6666667	2.479764746	2.00E-04	0.02246214
114606	'Tle6'	731	784	469	1115	1236	2035	661.3333333	1462	1.200381509	3.41E-04	0.033218136
115487184	'Tma7-ps'	245.5	185.87	219.64	5.48	11.95	25.61	217.0033333	14.34666667	-4.03359071	2.35E-13	4.07E-10
115488157	'Gm52042'	1024.58	1247.72	406.64	15.98	166.39	50.37	892.98	77.58	-3.559569404	2.84E-06	9.17E-04
11615	'Gm4737'	1308.88	2123.71	1283.51	193.99	687.32	359.24	1572.033333	413.5166667	-1.938223555	4.24E-05	0.00752867
11634	'Aire'	55	52	249	8	7	13	118.6666667	9.333333333	-3.749846127	1.63E-05	0.003636443

11668	'Aldh1a1'	2959	3644	1483	5890	12464	16358	2695.333333	11570.66667	2.118232539	4.14E-07	1.79E-04
11677	'Akr1b3'	1270	1342	1388	136794	2150	29365	1333.333333	56103	5.765923303	4.12E-07	1.79E-04
11686	'Alox12b'	0	1	0	46	4	10	0.333333333	20	6.151903241	5.81E-04	0.047144852
11801	'Cd5l'	0	0	0	6	4	102	0	37.33333333	7.522284413	7.68E-05	0.011410709
11827	'Aqp2'	7	4	1	269	1	31	4	100.3333333	5.143693795	5.84E-04	0.047144852
11830	'Aqp5'	46	62	5	806	42	620	37.66666667	489.3333333	4.013592845	2.27E-04	0.024847753
11838	'Arc'	17	11	36	2684	6	213	21.33333333	967.6666667	5.86709853	5.22E-05	0.00848495
118568607	'LOC118568607'	85.05	140.49	121.2	0	0	0	115.58	0	-9.246153146	1.63E-12	2.38E-09
118568683	'LOC118568683'	1654.49	1659.64	990.45	2159.58	3625.37	3117.43	1434.86	2967.46	1.106436376	1.35E-04	0.017243116
118568705	'LOC118568705'	692.79	731.86	1500.44	2866.08	4644.54	4553.97	975.03	4021.53	2.001263867	7.30E-05	0.01092959
11931	'Atp1b1'	2596.45	2494.64	2689	34730.9	3977.86	13789.6	2593.363333	17499.43333	3.037686024	2.64E-04	0.027583361
11936	'Fxyd2'	13	18	18	689	16	118	16.33333333	274.3333333	4.449926482	1.28E-04	0.01664332
11997	'Akr1b7'	7	10	81	10186	22	665	32.66666667	3624.333333	7.08022401	4.27E-04	0.039568533
12140	'Fabp7'	38	51	35	1182	45	1616	41.33333333	947.6666667	4.659128624	2.96E-06	9.39E-04
12183	'Bpgm'	197	207	167	1501	480	1040	190.3333333	1007	2.603234401	1.88E-05	0.004028556
12309	'S100g'	0	0	0	2830	3	327	0	1053.333333	12.91478734	5.22E-05	0.00848495
12390	'Cav2'	253	230	173	444	470	542	218.6666667	485.3333333	1.234662384	1.02E-04	0.013994201
12424	'Cck'	5	1	31	12752	2	944	12.33333333	4566	8.812516118	1.40E-04	0.017654732
12508	'Cd53'	33	28	11	83	58	123	24	88	1.989265205	6.42E-04	0.049996188
12527	'Cd9'	341	358	298	4108	698	1486	332.3333333	2097.333333	2.953824799	1.45E-04	0.018115704
12751	'Tpp1'	1037	1017	890	2224	1971	2208	981.3333333	2134.333333	1.223295316	5.84E-04	0.047144852
12876	'Cpe'	5058	5471	4441	6426	9836	11140	4990	9134	0.897757903	9.40E-05	0.013244807
12955	'Cryab'	91	81	105	4066	125	895	92.33333333	1695.333333	4.551084218	2.21E-05	0.0044251
13036	'Ctsh'	556	588	606	4468	840	3003	583.3333333	2770.333333	2.452825042	6.05E-04	0.048169622
13040	'Ctss'	31	40	5	412	76	243	25.33333333	243.6666667	3.58175299	6.75E-05	0.01026066
13136	'Cd55'	797.95	807.96	292.88	6669	1263.98	2236	632.93	3389.66	2.772882586	5.62E-04	0.046680135
13417	'Dnah8'	1989	1827	175	76	183	103	1330.333333	120.6666667	-3.35497599	1.46E-05	0.003424174
13476	'Reep5'	1077	1131	1013	1658	2046	2687	1073.666667	2130.333333	1.02388113	1.59E-04	0.01938235
13830	'Stom'	515	676	189	2353	1623	1800	460	1925.333333	2.267726293	4.99E-05	0.008322658
14055	'Ezh1'	1485	1439	761	272	128	215	1228.333333	205	-2.389915898	8.07E-06	0.002131999
14056	'Ezh2'	1218	1229	1117	300	185	108	1188	197.666667	-2.554066681	1.05406E-11	0.026431132
14063	'F2rl1'	14	27	54	594	70	173	31.66666667	279	3.381595522	5.74E-04	0.047144852
140795	'P2ry14'	5	3	5	226	3	92	4.333333333	107	4.921692859	8.82E-05	0.012798273
14411	'Slc6a12'	2	5	5	3188	5	827	4	1340	8.731637333	1.64E-09	1.36E-06
14468	'Gbp2b'	37	51	38	0	10	2	42	4	-3.455463727	3.90E-04	0.036760231
14726	'Pdpn'	523	561	494	5230	1163	1964	526	2785.666667	2.680638716	2.40E-04	0.025741813
14858	'Gsta2'	2	1	3	127	7	103.67	2	79.22333333	5.476533448	5.35E-06	0.001517766
15114	'Hap1'	77	75	33	808	285	336	61.66666667	476.3333333	3.234271009	2.82E-06	9.17E-04
15135	'Hbb-y'	102	165	384	3	36	3	217	14	-4.105317174	4.83E-05	0.008204297

15186	'Hdc'	160	187	58	7145	165	1574	135	2961.333333	4.893423833	1.28E-05	0.003158437
15220	'Foxq1'	21	35	14	1467	18	925	23.33333333	803.3333333	5.402579974	2.52E-06	8.70E-04
15429	'Hoxd1'	59	39	32	6	12	10	43.33333333	9.333333333	-2.177861783	1.82E-04	0.021021471
16158	'Il11ra2'	89.08	96.51	45.61	1.03	12.24	8.26	77.06666667	7.176666667	-3.467891614	8.54E-07	3.19E-04
16174	'Il18rap'	1	1	2	69	10	6	1.333333333	28.33333333	4.755043163	6.34E-04	0.049568551
16432	'Itm2b'	3227	3221	2555	7282	5484	7953	3001	6906.333333	1.311719711	5.14E-04	0.044250111
170756	'Slc8b1'	103	103	70	1470	158	405	92	677.6666667	3.233277251	2.63E-04	0.027583361
17105	'Lyz2'	186	263	8	6540	443	1505	152.3333333	2829.333333	4.682195429	8.95E-05	0.012810696
17110	'Lyz1'	6	9	0	310	25	69	5	134.6666667	5.202465421	2.96E-05	0.005737356
17123	'Madcam1'	181	184	1259	3	65	29	541.3333333	32.33333333	-4.329170115	5.18E-05	0.00848495
17174	'Masp1'	84	89	59	522	193	316	77.33333333	343.6666667	2.371135216	1.01E-04	0.013994201
17175	'Masp2'	19.93	28.35	57.98	1.32	4.31	0	35.42	1.876666667	-4.425254615	1.06E-04	0.014511205
17286	'Meox2'	31	33	17	2243	56	92	27	797	5.373619304	1.92E-05	0.004057658
17295	'Met'	151	160	103	491	708	826	138	675	2.336198973	6.13E-17	1.94E-13
17318	'Mid1'	721	795	2056	163	379	230	1190.666667	257.3333333	-2.286967208	2.21E-04	0.024317809
17381	'Mmp12'	4	2	4	191	9	293	3.333333333	164.3333333	5.712080279	4.63E-07	1.96E-04
17472	'Gbp4'	145.91	118.15	374.38	5	23	42.91	212.8133333	23.63666667	-3.372439422	2.07E-05	0.004291
17474	'Clec4d'	24	28	10	114	76	81	20.66666667	90.33333333	2.316759451	1.33E-04	0.017110932
17476	'Mpeg1'	124	87	23	569	146	604	78	439.6666667	2.712795231	4.71E-04	0.042077778
17760	'Map6'	37	39	55	2	4	9	43.66666667	5	-3.181853852	1.59E-05	0.003595222
17829	'Muc1'	37	36	57	2415	33	454	43.33333333	967.3333333	4.83216215	4.99E-05	0.008322658
17831	'Muc2'	160	118	457	30	50	25	245	35	-2.847751612	9.33E-05	0.013233875
17884	'Myh4'	74.99	42.97	6	0	3	2	41.32	1.666666667	-4.57557427	1.63E-04	0.019471673
17898	'Myl7'	28	38	38	1	8	4	34.66666667	4.333333333	-3.044098345	1.12E-04	0.015060594
17988	'Ndrg1'	3345	2105	53389	636	707	2021	19613	1121.333333	-4.347425195	1.25E-04	0.016489852
18606	'Enpp2'	1119	970	1403	15386	3032	2989	1164	7135.666667	2.918450879	5.75E-04	0.047144852
18764	'Pkd2'	1514	1514	761	6834	3207	3277	1263	4439.333333	2.061593263	5.36E-04	0.045614454
18772	'Pkp1'	8	9	145	1	2	2	54	1.666666667	-5.195861407	1.75E-04	0.020572308
18791	'Plat'	1011	1044	1143	69629	1272	5031	1066	25310.66667	4.996868038	3.86E-05	0.007055669
19116	'Prlr'	192	208	71	351	817	532	157	566.6666667	1.914476356	5.70E-05	0.008884271
19165	'Psen2'	215	225	184	2705	342	970	208	1339	2.995066016	2.94E-04	0.029716625
19215	'Ptgds'	20	16	64	1289	61	504	33.33333333	618	4.419302711	4.82E-05	0.008204297
192190	'Pkhd1l1'	203	255	38	5045	537	918	165.3333333	2166.666667	4.161448	4.33E-05	0.007630056
19229	'Ptk2b'	270	246	193	344	477	761	236.3333333	527.3333333	1.17496985	3.34E-04	0.032745436
19267	'Ptpre'	175	208	149	391	425	476	177.3333333	430.6666667	1.358500743	1.89E-05	0.004028556
194597	'Tmprss11a'	126	133	62	0	3	0	107	1	-6.695814838	9.76E-11	1.16E-07
195531	'Zfp982'	333.79	382.5	248.72	569.02	858	599	321.67	675.34	1.150251784	6.12E-04	0.048420886
19701	'Ren1'	20	24	6	3792	35	21	16.66666667	1282.666667	6.813247097	7.74E-06	0.002072764
20198	'S100a4'	4	3	1	69	10	27	2.666666667	35.33333333	4.051671114	2.80E-04	0.0284244

20297	'Ccl20'	14	13	244	1	6	2	90.33333333	3	-5.131627002	1.08E-04	0.014617609
20531	'Slc34a2'	1	2	1	53	4	28	1.333333333	28.33333333	4.68643401	3.54E-04	0.03396508
20616	'Snap91'	922	747	100	9	87	108	589.6666667	68	-3.146600571	4.05E-04	0.037893507
20657	'Sod3'	31	28	8	825	57	222	22.33333333	368	4.451803182	1.22E-05	0.003055455
20739	'Spta1'	39	48	8	1919	65	269	31.66666667	751	5.05352206	1.56E-05	0.00358374
20899	'Stra8'	121	96	560	0	11	9	259	6.666666667	-5.528520315	2.49E-07	1.28E-04
212980	'Slc45a3'	96	97	68	2475	68	566	87	1036.333333	3.965505198	2.33E-04	0.02516575
213389	'Prdm9'	1399	1309	696	140	526	247	1134.666667	304.3333333	-1.886954278	1.58E-04	0.01938235
21350	'Tal2'	4	4	6	1562	4	218	4.666666667	594.6666667	7.378938027	2.23E-07	1.21E-04
214230	'Pak6'	118	142	82	261	485	433	114	393	1.823610331	1.09E-08	7.94E-06
214239	'Ccdc9b'	41	54	26	90	245	151	40.33333333	162	2.031211294	1.43E-05	0.003393278
214854	'Neurl3'	152	147	1577	22	62	114	625.3333333	66	-3.493823508	6.16E-04	0.048420886
215751	'Ginm1'	575	592	518	1280	1056	1536	561.6666667	1290.666667	1.286656321	3.57E-04	0.034093749
216350	'Tspan8'	36	51	17	87	88	165	34.66666667	113.3333333	1.784827667	6.06E-04	0.048169622
21667	'Tdgf1'	1	2	1	253	0	7	1.333333333	86.66666667	6.513775487	3.91E-04	0.036760231
21684	'Tectb'	1	0	2	184	3	8	1	65	6.405264308	1.81E-04	0.02101658
21813	'Tgfbr2'	638	559	440	641	1249	1243	545.6666667	1044.333333	0.951511339	6.16E-04	0.048420886
218440	'Ankrd34b'	69	67	43	1	13	11	59.66666667	8.333333333	-2.883323311	3.97E-05	0.007195467
218865	'Chdh'	48.73	23.56	7.22	234.89	54.43	238.54	26.50333333	175.9533333	2.97766088	4.50E-04	0.041129735
21949	'Tnfsf8'	0	0	0	84	0	0	0	28	20.08874806	2.76E-07	1.29E-04
22041	'Trf'	78	101	80	6265	197	359	86.33333333	2273.666667	5.165231095	1.34E-05	0.003270239
22147	'Tuba3b'	66.36	45.31	53.78	0	6	4.99	55.15	3.663333333	-4.085700452	2.95E-07	1.34E-04
22268	'Upk1b'	9	16	13	23	357	431	12.66666667	270.3333333	4.272716383	8.56E-07	3.19E-04
223332	'Ranbp3l'	20	23	2	1392.01	10	675	15	692.3366667	5.915868961	5.97E-06	0.001645292
22410	'Wnt10b'	146	116	318	33	36	13	193.3333333	27.33333333	-2.766431529	1.55E-04	0.019123368
224530	'Acat3'	7.99	8.96	6.03	1264.94	20.23	345.05	7.66	543.4066667	6.647638763	2.06E-08	1.35E-05
224671	'Btbd9'	2227	2241	1705	756	1141	1246	2057.666667	1047.666667	-0.936555861	4.04E-05	0.007241689
225362	'Reep2'	174	210	216	353	498	491	200	447.3333333	1.18854961	3.12E-04	0.030714333
227231	'Cps1'	91	62	7	0	3	2	53.33333333	1.666666667	-4.961960215	3.40E-05	0.006398791
22787	'Zp2'	1	2	0	1	106	545	1	217.3333333	7.649262916	2.73E-06	9.17E-04
22788	'Zp3'	1	0	0	1	14	163	0.333333333	59.33333333	7.190272982	1.62E-04	0.019471673
229898	'Gbp5'	37	44	152	2	15	13	77.66666667	10	-3.143714714	5.10E-04	0.044231386
229933	'Clca2'	16	11	12	141	35	59	13	78.33333333	2.846756048	4.36E-04	0.040018506
230779	'Serinc2'	106	79	119	1890	138	581	101.3333333	869.6666667	3.405683504	3.05E-04	0.030488342
230810	'Slc30a2'	98	113	84	284	339	425	98.33333333	349.3333333	1.882593537	8.41E-10	7.99E-07
230828	'Il22ra1'	6	6	1	484	10	295	4.333333333	263	6.251177235	2.76E-07	1.29E-04
230903	'Fbxo44'	34	35	71	683	107	393	46.66666667	394.3333333	3.257038547	1.01E-04	0.013994201
230904	'Fbxo2'	12	14	14	2436	14	476	13.33333333	975.3333333	6.575305141	2.76E-07	1.29E-04
231238	'Sel1l3'	26	20	28	112	124	145	24.66666667	127	2.408842268	4.82E-08	2.86E-05

231832	'Tmem184a'	195	205	150	245	387	481	183.3333333	371	1.039686796	2.49E-04	0.026447937
232345	'A2m'	381	279	767	63	134	94	475.6666667	97	-2.35612986	5.28E-05	0.008506171
232371	'C1rl'	96	91	54	113	204	250	80.33333333	189	1.257624595	5.43E-04	0.045840415
232972	'Lypd10'	70	66	47	0	0	1	61	0.333333333	-7.336112652	1.90E-08	1.29E-05
233744	'Spon1'	66	61	22	1047	111	505	49.66666667	554.3333333	3.810074895	1.56E-05	0.00358374
234267	'Gpm6a'	182	162	42	350	454	684	128.6666667	496	2.033035794	1.91E-04	0.021798792
237038	'Nox1'	40.1	44.43	201.9	6.23	21.88	6.2	95.47666667	11.43666667	-3.234355838	5.45E-04	0.045845564
241576	'Ldlrad3'	296	349	246	609	684	602	297	631.6666667	1.184522668	4.78E-04	0.042360559
242594	'Fyb2'	2	4	1	14	21	23	2.333333333	19.33333333	3.107720454	5.51E-04	0.04617786
242735	'Lrrc38'	2	5	2	57	14	50	3	40.33333333	3.938904021	4.74E-05	0.008204297
243302	'Gm4963'	74.75	65.9	152.31	1.15	1.05	0	97.65333333	0.733333333	-7.193664165	5.54E-09	4.39E-06
244049	'Mctp2'	19	23	15	95	58	159	19	104	2.526812597	8.59E-06	0.002236954
244954	'Prss35'	899.29	797.63	415	41835	2042.69	11360.6	703.9733333	18412.76	5.101227968	2.79E-07	1.29E-04
269378	'Ahcy'	2134.12	1314.29	924.49	7028.01	3043.68	5118.76	1457.633333	5063.483333	2.003198101	4.99E-04	0.043523633
270160	'Rab39'	47	47	101	2	16	14	65	10.66666667	-2.746681614	4.59E-04	0.041791682
272382	'Spib'	13	12	116	0	5	1	47	2	-4.788635781	5.82E-04	0.047144852
279028	'Adamts13'	106.77	91.45	251.11	2	35.13	17.52	149.7766667	18.21666667	-3.231942862	2.13E-04	0.023582255
28240	'Trpm2'	35	15	120	3	5	6	56.66666667	4.666666667	-3.718968183	1.78E-04	0.020741164
317677	'C1s2'	65.2	57	244.36	4.01	3	4	122.1866667	3.67	-5.097404183	2.38E-08	1.51E-05
319352	'Pianp'	434.01	389	122	48	78	69	315.0033333	65	-2.182200894	2.11E-05	0.004291
320299	'Iqcb1'	3741	3563	2284	345	1540	1173	3196	1019.333333	-1.686668748	4.67E-04	0.042077778
320858	'L3mbtl4'	2	5	4	4	382	42	3.666666667	142.6666667	5.153418116	5.36E-05	0.008568148
329278	'Tnn'	3	5	16	1164	8	9	8	393.6666667	5.991320315	1.67E-04	0.019862371
329502	'Pla2g4e'	119	82	9	1	1	0	70	0.666666667	-6.537340723	3.13E-06	9.75E-04
332396	'Kcnk18'	2	1	0	2	38	88	1	42.66666667	5.31632911	2.53E-04	0.026705556
338521	'Fa2h'	19	13	9	1337	11	177	13.66666667	508.3333333	5.666018515	9.89E-06	0.002540023
381196	'Gm960'	125	126	48	6	36	12	99.66666667	18	-2.45520378	4.90E-04	0.04295735
381853	'Gipr'	88	44	256	15	10	9	129.3333333	11.33333333	-3.485645213	5.54E-05	0.008769529
394432	'Ugt1a7c'	30.12	24.49	2.27	184.93	58.72	225.54	18.96	156.3966667	3.268617865	2.69E-04	0.027996512
433375	'Creg1'	1159	1314	879	3478	2593	5370	1117.333333	3813.666667	1.85146789	3.91E-06	0.001161794
433745	'Gm12816'	175.84	148.2	229.31	0	0	0	184.45	0	-9.943802547	2.53E-14	4.82E-11
433804	'Zfp985'	96.63	124.48	191.77	18.05	37.84	12	137.6266667	22.63	-2.612980581	4.81E-05	0.008204297
434438	'Iho1'	303	303	159	11	93	37	255	47	-2.47491504	2.05E-04	0.022950907
50874	'Tmod4'	44	59	42	12	11	7	48.33333333	10	-2.163557059	3.44E-04	0.03332022
52815	'Ldhd'	72.55	81.03	58.07	210.63	169.53	251.45	70.55	210.5366667	1.674823788	3.63E-05	0.006774907
53945	'Slc40a1'	1292	1297	1310	6101	2346	5672	1299.666667	4706.333333	1.997951595	3.12E-04	0.030714333
545124	'Tdg-ps'	244.65	288.92	567.4	30.06	97.08	29.51	366.99	52.21666667	-2.877068051	1.88E-05	0.004028556
54613	'St3gal6'	132	127	183	1506	346	648	147.3333333	833.3333333	2.725910798	2.42E-04	0.025854303
546849	'AAdacl4fm3'	9	11	12	0	0	0	10.66666667	0	-5.819030998	5.37E-04	0.045614454

56047	'Msln'	34	27	73	310	127	256	44.66666667	231	2.456357192	5.21E-04	0.044654124
56375	'B4galt4'	322	373	179	829	1232	1635	291.3333333	1232	2.127919664	6.48E-10	6.85E-07
56386	'B4galt6'	570	527	243.54	101	195	201	446.8466667	165.6666667	-1.385580084	4.65E-04	0.042068288
57816	'Tesc'	5	7	2	100	12	103	4.666666667	71.66666667	4.149444179	3.02E-05	0.005803392
58238	'Fam181b'	155	173	78	32	41	16	135.3333333	29.66666667	-2.042904769	4.35E-04	0.040018506
59020	'Pdzk1'	14.41	12.33	10.67	3.66	155.01	564.46	12.47	241.0433333	4.162021176	3.52E-04	0.03396508
59095	'Fxyd6'	747	835	608	1016	1464	1823	730	1434.333333	1.00664934	6.35E-05	0.009744897
619310	'Zfp872'	24	23	12	47	68	68	19.66666667	61	1.698226109	2.74E-04	0.028291035
619547	'Rpl34-ps1'	4327.63	4369.1	6487.92	2.59	0.25	2.16	5061.55	1.666666667	-11.87616402	7.70E-42	1.46E-37
626578	'Gbp10'	142.68	128.4	383.59	0	2.41	10.86	218.2233333	4.423333333	-5.962656383	1.62E-09	1.36E-06
628746	'Rybp-ps'	6.86	14.04	16.7	37.88	131.56	86.46	12.53333333	85.3	2.754557582	2.82E-05	0.005524122
64297	'Gprc5b'	1778	1892	931	6503	3454	4379	1533.666667	4778.666667	1.847471411	4.79E-04	0.042360559
654824	'Ankrd37'	291	210	2662	34	87	138	1054.333333	86.33333333	-3.843727093	1.28E-04	0.01664332
66329	'Susd3'	72	65	16	190	200	206	51	198.6666667	2.108777795	2.29E-04	0.024910793
664969	'Gm7429'	1437.59	1482.55	3213.12	0	0	0	2044.42	0	-13.45452697	1.58E-23	1.00E-19
66773	'Gm17019'	0	1	0	81.79	2	24.96	0.333333333	36.25	6.983357067	1.32E-04	0.017014102
670211	'Gm12508'	40	33.11	91.36	0	0	0	54.82333333	0	-8.237646653	1.85E-08	1.29E-05
67729	'Mansc1'	17	26	11	40	111	110	18	87	2.271899321	2.12E-05	0.004291
68588	'Cthrc1'	55	45	30	877	85	473	43.33333333	478.3333333	3.752785503	1.39E-05	0.003351794
68774	'Ms4a6d'	16	10	8	46	36	92	11.33333333	58	2.407369459	1.15E-04	0.015350347
69717	'Gm10499'	41.58	32.22	82.22	3.23	5.86	1	52.00666667	3.363333333	-4.10726005	5.61E-06	0.001569535
69983	'Sis'	64	50	9	0	0	0	41	0	-7.661393552	8.45E-07	3.19E-04
70045	'2610528A11Rik	36	21	603	0	6	0	220	2	-7.048932354	1.13E-05	0.002873412
70564	'Prxl2a'	241	236	192	4658	403	864	223	1975	3.525370442	1.98E-04	0.022382587
70911	'Phyhipl'	174.62	169.62	101.64	4.96	45.87	18.1	148.6266667	22.97666667	-2.774691364	8.72E-05	0.012752569
71325	'Tchhl1'	5	5	39	0	0	0	16.33333333	0	-6.568881674	5.85E-04	0.047144852
71721	'Fam13c'	88.38	81.38	48.36	199.04	175.13	246.9	72.70666667	207.0233333	1.619722571	7.30E-05	0.01092959
72003	'Synpr'	20	13	17	44	67	63	16.66666667	58	1.829238837	1.71E-04	0.02019921
73707	'Gucy2g'	64	38	61	7008	54	845	54.33333333	2635.666667	6.002921932	2.34E-06	8.22E-04
74090	'Paqr5'	15	24	9	668	33	347	16	349.3333333	4.767070745	2.03E-06	7.29E-04
74241	'Chpf'	670	681	429	1005	1215	1290	593.3333333	1170	1.061247719	2.75E-04	0.028291035
74525	'Fam234b'	296	360	126	405	1110	945	260.6666667	820	1.679667914	3.10E-04	0.030714333
74558	'Gvin1'	9.71	0	10.73	109.79	116.5	259.48	6.813333333	161.9233333	4.620524028	1.93E-07	1.08E-04
75146	'Mfsd13a'	53	57	35	92	150	125	48.33333333	122.3333333	1.396059753	1.51E-04	0.018745682
75623	'Tex30'	1274	1320	933	264	593	723	1175.666667	526.6666667	-1.170953049	5.55E-04	0.046302974
75659	'Wdr54'	70	84	92	0	16	10	82	8.666666667	-3.364629477	8.69E-05	0.012752569
76072	'Rnf183'	4	5	1	145	3	39	3.333333333	62.33333333	4.642115157	4.07E-04	0.03792354
76487	'Ppp1r3g'	240	188	416	15	65	42	281.3333333	40.66666667	-2.896622183	2.78E-06	9.17E-04
76681	'Trim12a'	6	15.65	23.62	179	408	356	15.09	314.3333333	4.362313211	3.28E-13	5.20E-10

76854	'Gper1'	8	12	6	1380	24	103	8.666666667	502.3333333	6.326516589	6.67E-07	2.70E-04
76933	'Ifi27l2a'	29	44	15	572	21	436	29.33333333	343	3.820530353	1.91E-04	0.021798792
77125	'Il33'	274	272	198	18	101	18	248	45.66666667	-2.453108612	4.83E-04	0.042530043
79235	'Lrat'	87	95	43	10	15	16	75	13.66666667	-2.389323184	3.41E-06	0.001028785
80744	'Cwc22'	2088.08	3472.75	3311.87	402.77	969.36	874.76	2957.566667	748.9633333	-2.01898198	1.03E-06	3.76E-04
80976	'Syt13'	56	83	27	252	156	675	55.33333333	361	2.761179584	1.78E-05	0.003930314
83965	'Enpp5'	573	574	346	998	1004	1234	497.6666667	1078.666667	1.214522713	2.78E-04	0.0284244
85031	'Pla1a'	6	4	9	412	9	37	6.333333333	152.6666667	4.974965313	1.14E-04	0.015255476
93695	'Gpnmb'	35	33	117	758	233	2591	61.66666667	1194	4.152914684	4.78E-06	0.001375516
94180	'Acsbg1'	1002	1163	693	2497	2983	2236	952.6666667	2572	1.544713027	2.08E-05	0.004291
99035	'Olah'	1	0	0	0	32	125	0.333333333	52.33333333	7.013902398	5.12E-04	0.044250111
